# Supplementary material for: A multi-marker association method for genome-wide association studies without the need for population structure correction
Source: Nat Commun. 2016 Nov 10;7:13299. doi: 10.1038/ncomms13299 (PMC5109549; doi:10.1038/ncomms13299)
Supplement: Supplementary Information — Supplementary Figures 1-405, Supplementary Note 1 and Supplementary References [file ncomms13299-s1.pdf]

Supplementary Figures

GWA analysis for three simulation scenarios of structured populations

Simulation with heritability of 0.7 and 50 effects randomly drawn from a Gamma distribution

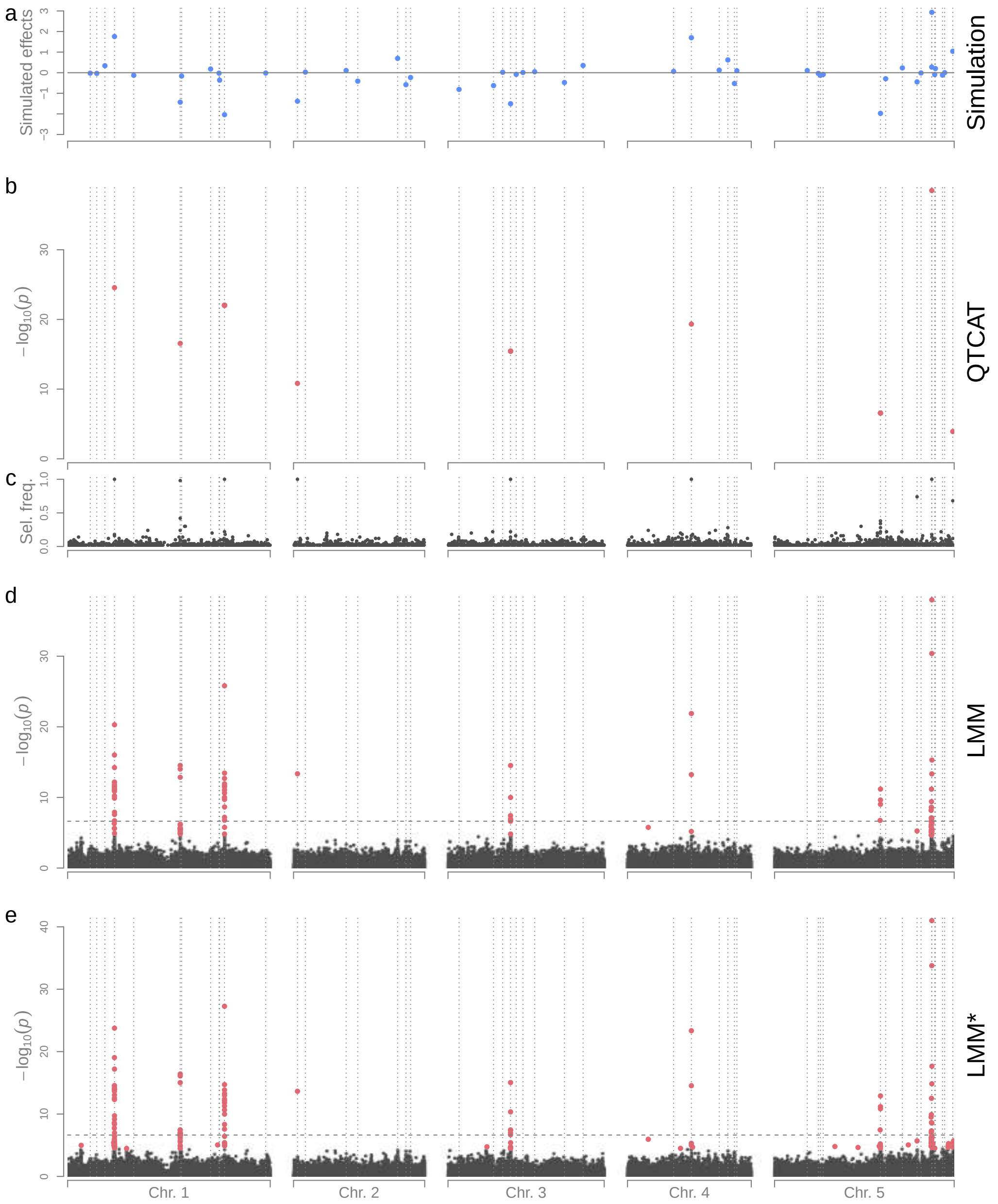

Supplementary Figure 1 Simulation of a GWA analysis based on a structured population with a heritability of 0.7 (run 1). (**a**) Simulation of 50 effects randomly drawn from a Gamma distribution and assigned to random markers. Markers with effect are highlighted with dashed lines. (**b**) Significant QTCs found by QTCAT. (**c**) LASSO selection frequency for each marker during the 50 iterations of QTCAT. (**d**) Manhattan plot of the LMM analysis. The horizontal dashed line depicts the significance threshold when controlling the multiple testing with FWER, whereas the red markers are significantly associated when controlling with FDR. (**e**) The Manhattan plot of the LMM* analysis. GRM was estimated without markers on the chromosome of the actual testing position. The results are shown as in (d).

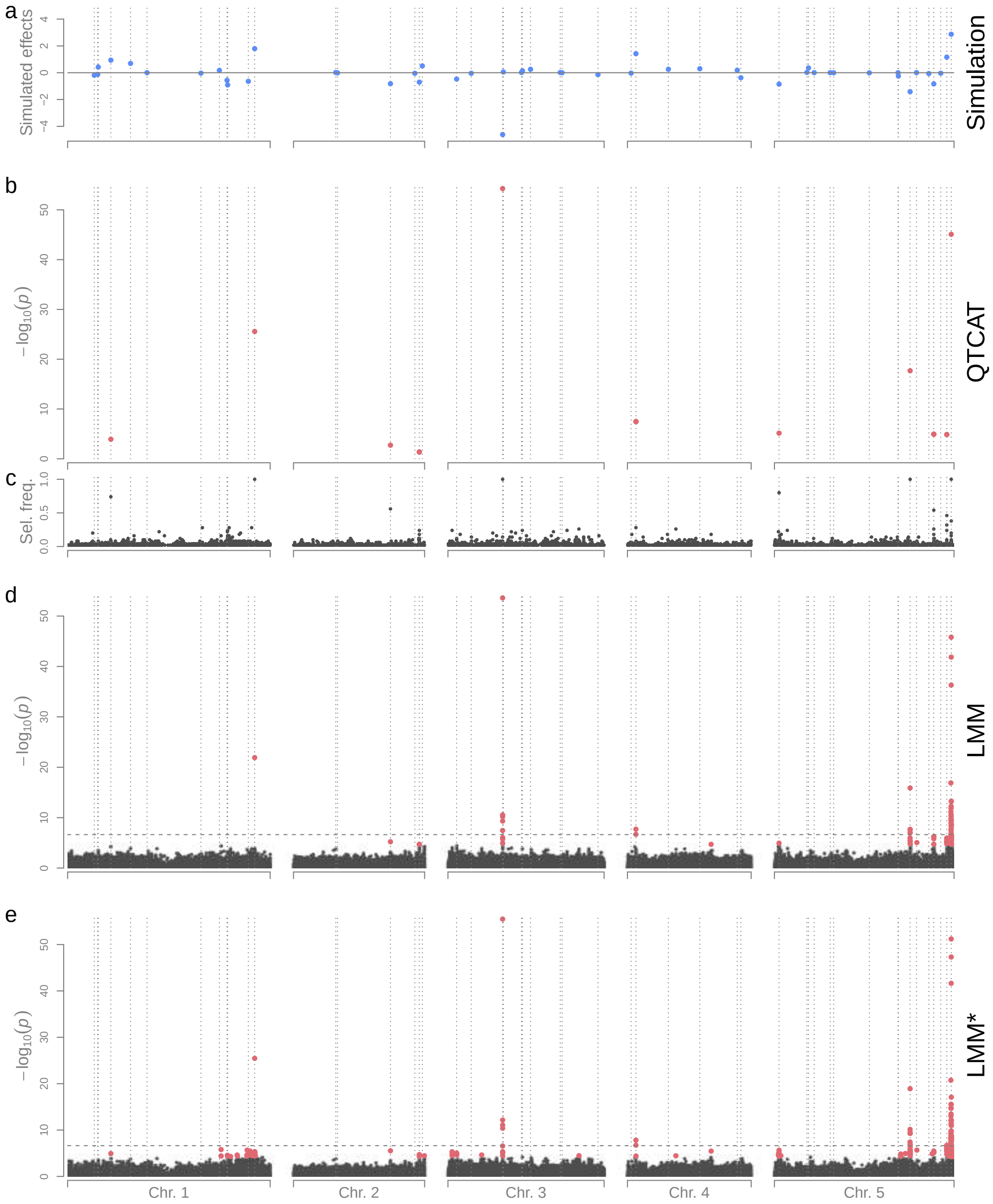

Supplementary Figure 2 Simulation of a GWA analysis based on a structured population with a heritability of 0.7 (run 2). (**a**) Simulation of 50 effects randomly drawn from a Gamma distribution and assigned to random markers. Markers with effect are highlighted with dashed lines. (**b**) Significant QTCs found by QTCAT. (**c**) LASSO selection frequency for each marker during the 50 iterations of QTCAT. (**d**) Manhattan plot of the LMM analysis. The horizontal dashed line depicts the significance threshold when controlling the multiple testing with FWER, whereas the red markers are significantly associated when controlling with FDR. (**e**) The Manhattan plot of the LMM* analysis. GRM was estimated without markers on the chromosome of the actual testing position. The results are shown as in (d).

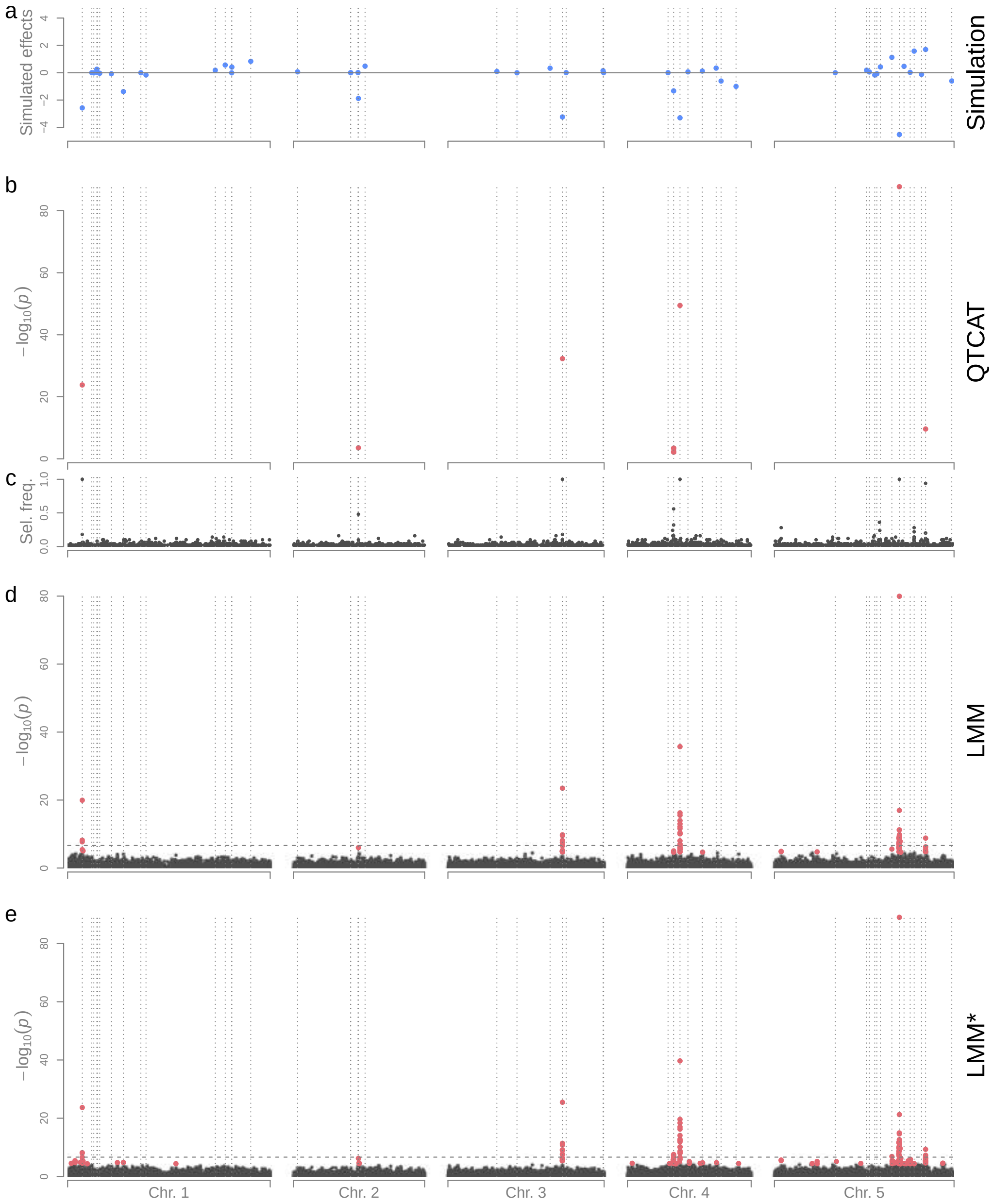

Supplementary Figure 3 Simulation of a GWA analysis based on a structured population with a heritability of 0.7 (run 3). (**a**) Simulation of 50 effects randomly drawn from a Gamma distribution and assigned to random markers. Markers with effect are highlighted with dashed lines. (**b**) Significant QTCs found by QTCAT. (**c**) LASSO selection frequency for each marker during the 50 iterations of QTCAT. (**d**) Manhattan plot of the LMM analysis. The horizontal dashed line depicts the significance threshold when controlling the multiple testing with FWER, whereas the red markers are significantly associated when controlling with FDR. (**e**) The Manhattan plot of the LMM* analysis. GRM was estimated without markers on the chromosome of the actual testing position. The results are shown as in (d).

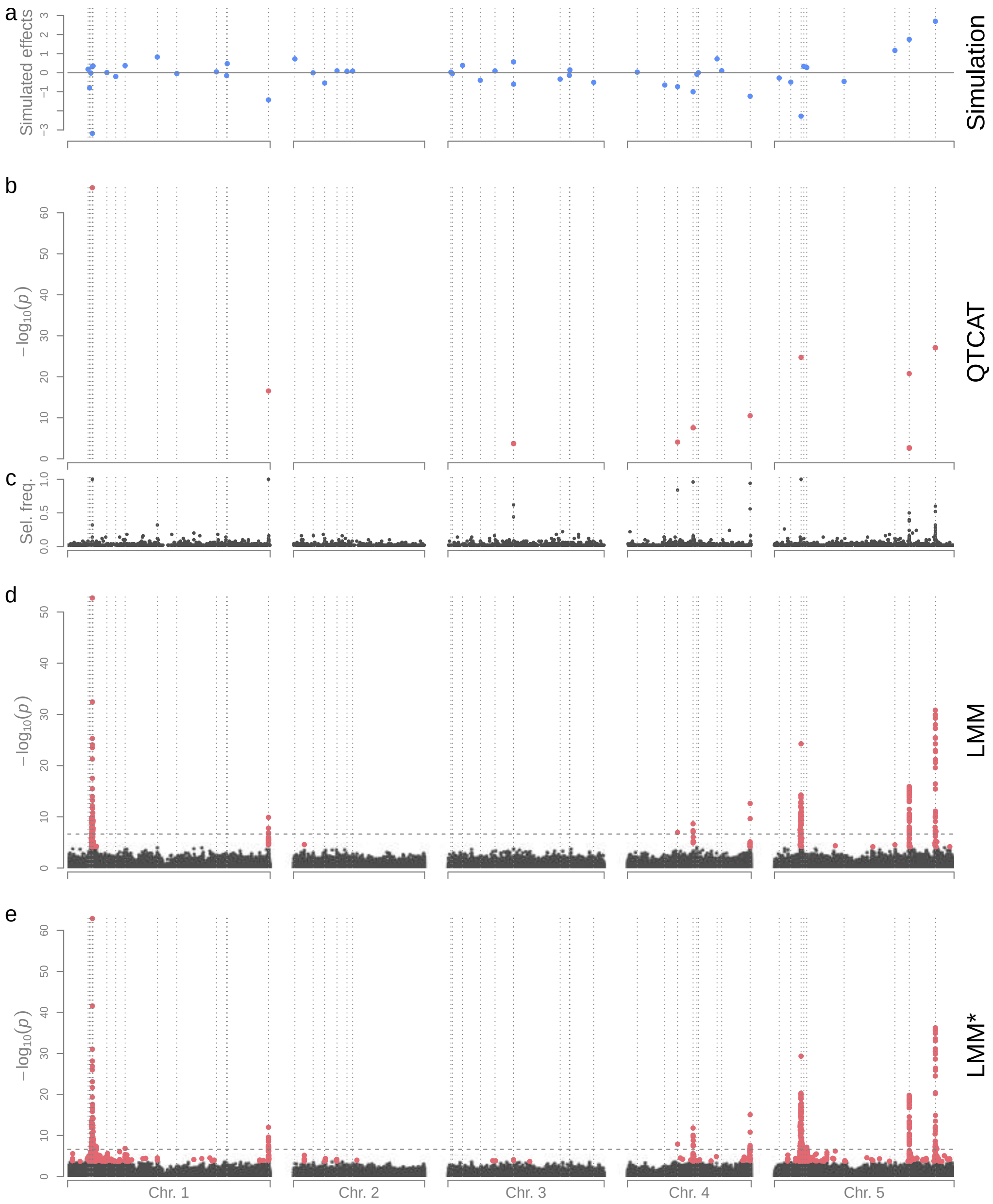

Supplementary Figure 4 Simulation of a GWA analysis based on a structured population with a heritability of 0.7 (run 4). (**a**) Simulation of 50 effects randomly drawn from a Gamma distribution and assigned to random markers. Markers with effect are highlighted with dashed lines. (**b**) Significant QTCs found by QTCAT. (**c**) LASSO selection frequency for each marker during the 50 iterations of QTCAT. (**d**) Manhattan plot of the LMM analysis. The horizontal dashed line depicts the significance threshold when controlling the multiple testing with FWER, whereas the red markers are significantly associated when controlling with FDR. (**e**) The Manhattan plot of the LMM* analysis. GRM was estimated without markers on the chromosome of the actual testing position. The results are shown as in (d).

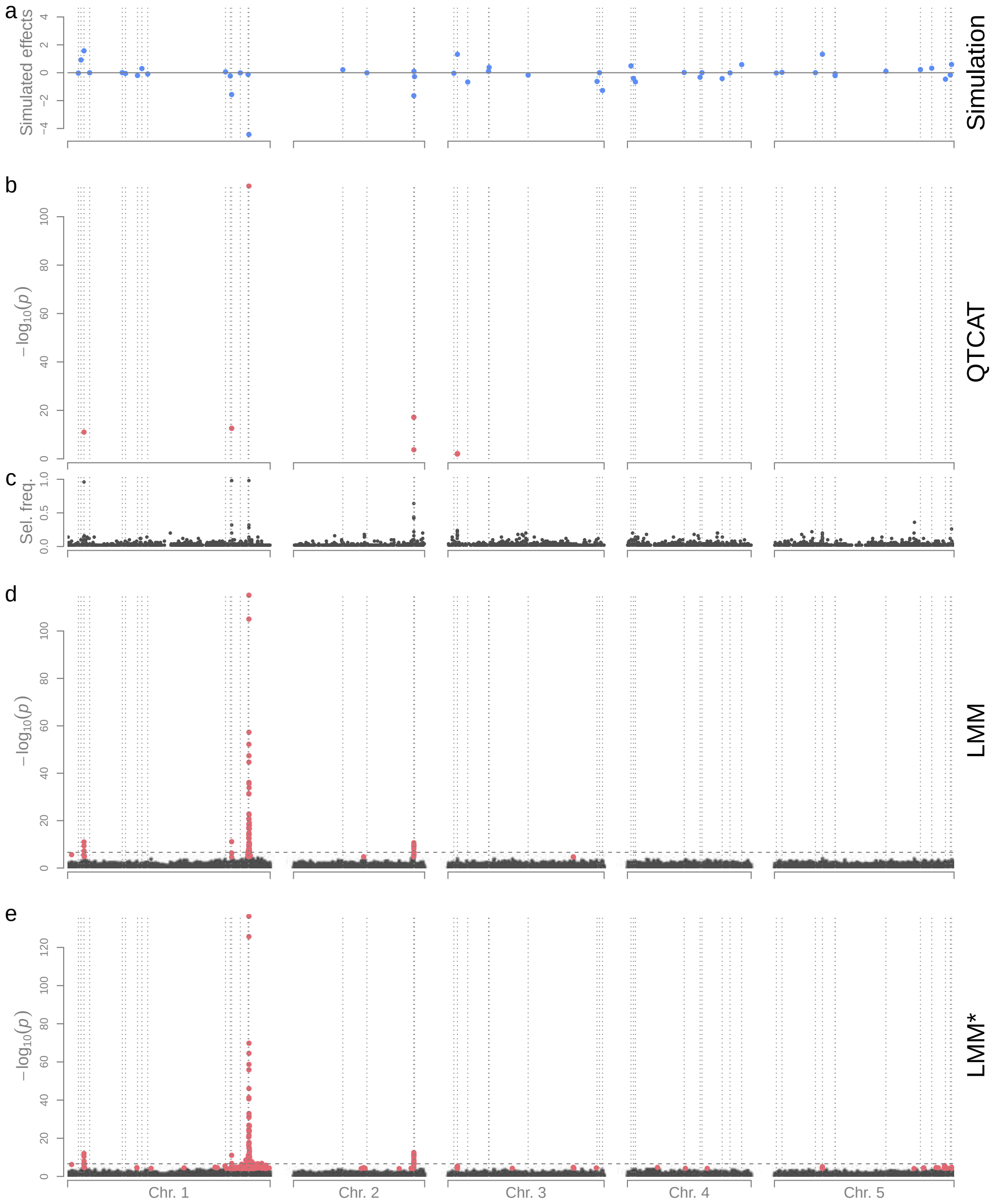

Supplementary Figure 5 Simulation of a GWA analysis based on a structured population with a heritability of 0.7 (run 5). (**a**) Simulation of 50 effects randomly drawn from a Gamma distribution and assigned to random markers. Markers with effect are highlighted with dashed lines. (**b**) Significant QTCs found by QTCAT. (**c**) LASSO selection frequency for each marker during the 50 iterations of QTCAT. (**d**) Manhattan plot of the LMM analysis. The horizontal dashed line depicts the significance threshold when controlling the multiple testing with FWER, whereas the red markers are significantly associated when controlling with FDR. (**e**) The Manhattan plot of the LMM* analysis. GRM was estimated without markers on the chromosome of the actual testing position. The results are shown as in (d).

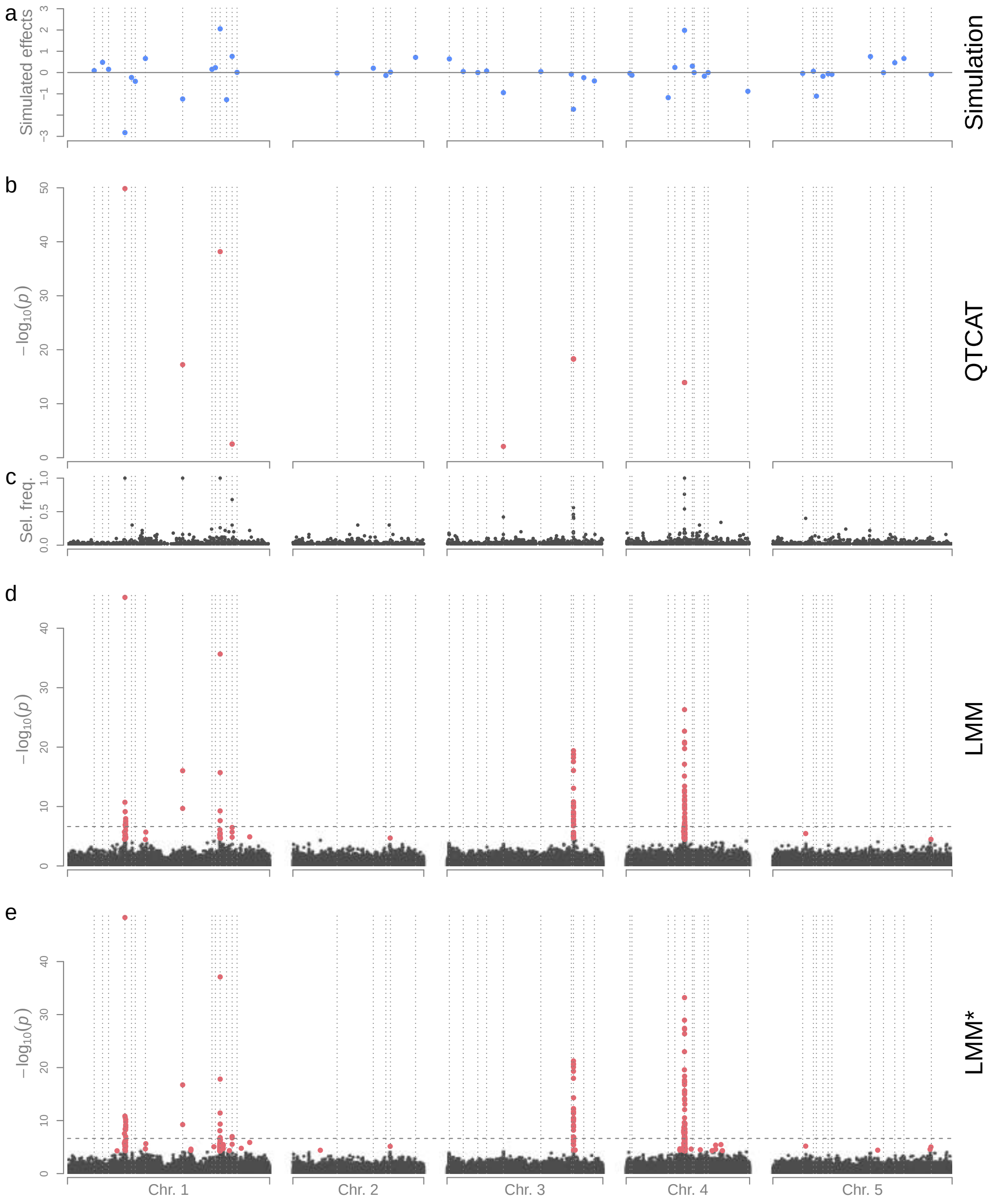

Supplementary Figure 6 Simulation of a GWA analysis based on a structured population with a heritability of 0.7 (run 6). (**a**) Simulation of 50 effects randomly drawn from a Gamma distribution and assigned to random markers. Markers with effect are highlighted with dashed lines. (**b**) Significant QTCs found by QTCAT. (**c**) LASSO selection frequency for each marker during the 50 iterations of QTCAT. (**d**) Manhattan plot of the LMM analysis. The horizontal dashed line depicts the significance threshold when controlling the multiple testing with FWER, whereas the red markers are significantly associated when controlling with FDR. (**e**) The Manhattan plot of the LMM* analysis. GRM was estimated without markers on the chromosome of the actual testing position. The results are shown as in (d).

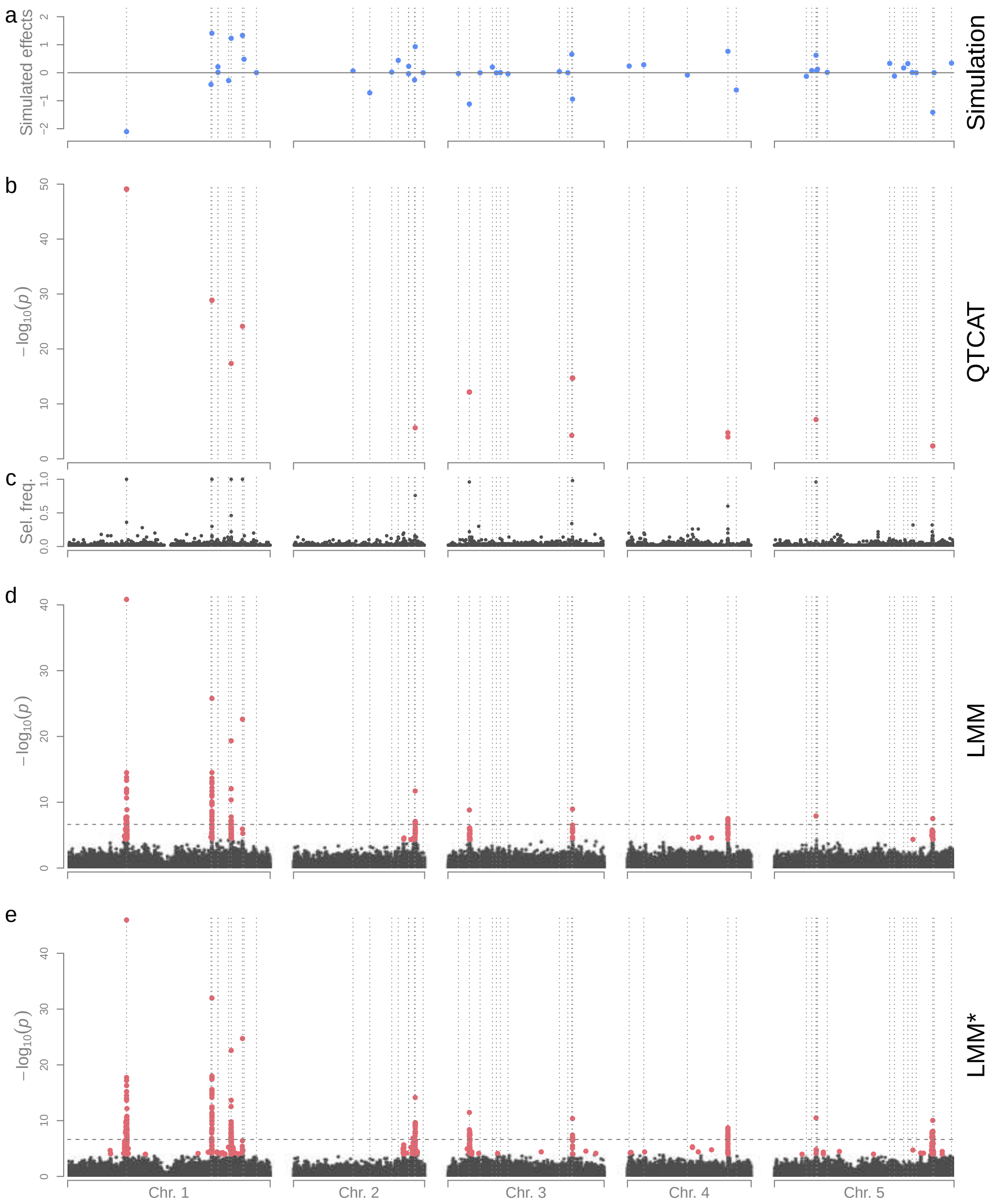

Supplementary Figure 7 Simulation of a GWA analysis based on a structured population with a heritability of 0.7 (run 7). (**a**) Simulation of 50 effects randomly drawn from a Gamma distribution and assigned to random markers. Markers with effect are highlighted with dashed lines. (**b**) Significant QTCs found by QTCAT. (**c**) LASSO selection frequency for each marker during the 50 iterations of QTCAT. (**d**) Manhattan plot of the LMM analysis. The horizontal dashed line depicts the significance threshold when controlling the multiple testing with FWER, whereas the red markers are significantly associated when controlling with FDR. (**e**) The Manhattan plot of the LMM* analysis. GRM was estimated without markers on the chromosome of the actual testing position. The results are shown as in (d).

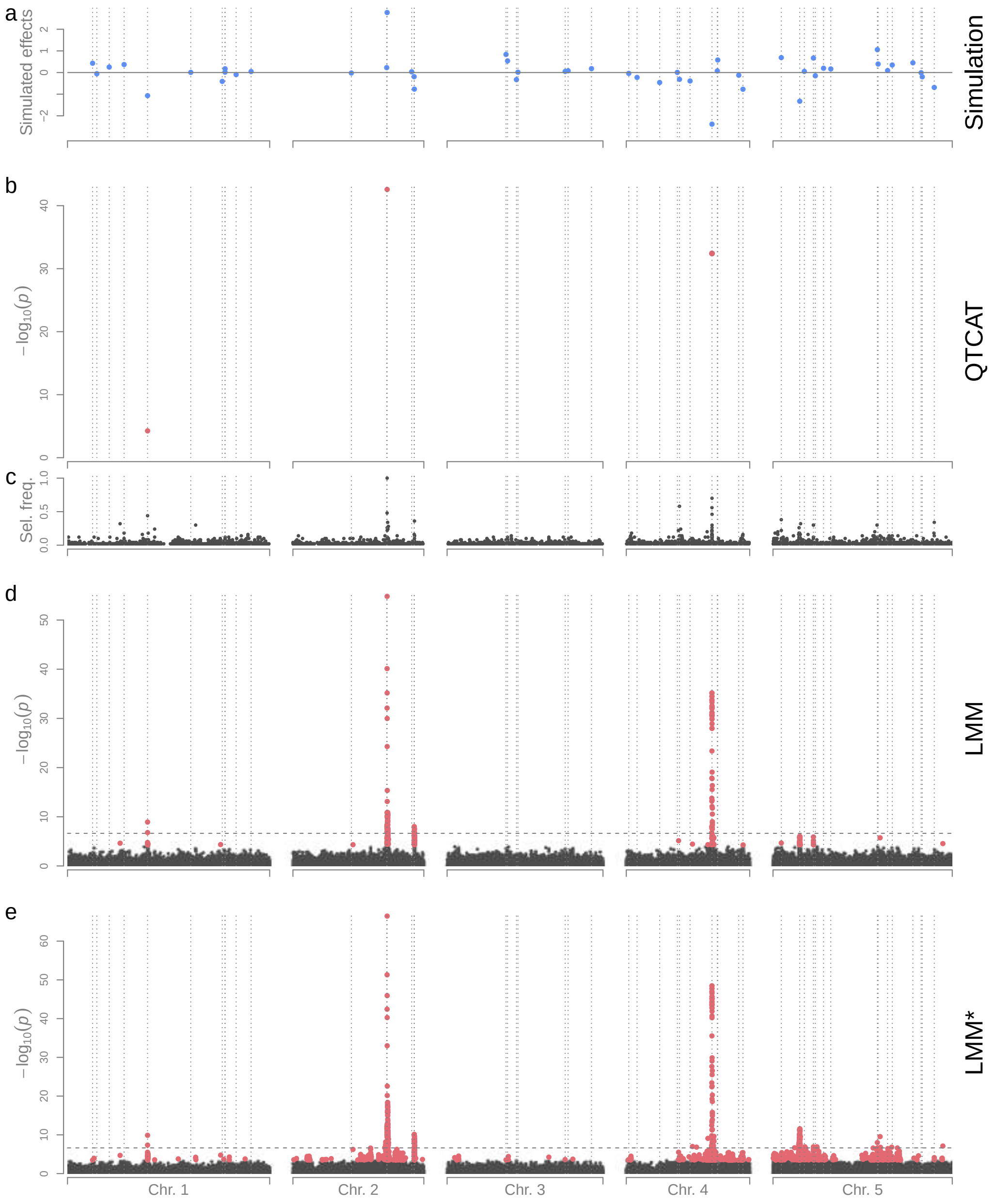

Supplementary Figure 8 Simulation of a GWA analysis based on a structured population with a heritability of 0.7 (run 8). (**a**) Simulation of 50 effects randomly drawn from a Gamma distribution and assigned to random markers. Markers with effect are highlighted with dashed lines. (**b**) Significant QTCs found by QTCAT. (**c**) LASSO selection frequency for each marker during the 50 iterations of QTCAT. (**d**) Manhattan plot of the LMM analysis. The horizontal dashed line depicts the significance threshold when controlling the multiple testing with FWER, whereas the red markers are significantly associated when controlling with FDR. (**e**) The Manhattan plot of the LMM* analysis. GRM was estimated without markers on the chromosome of the actual testing position. The results are shown as in (d).

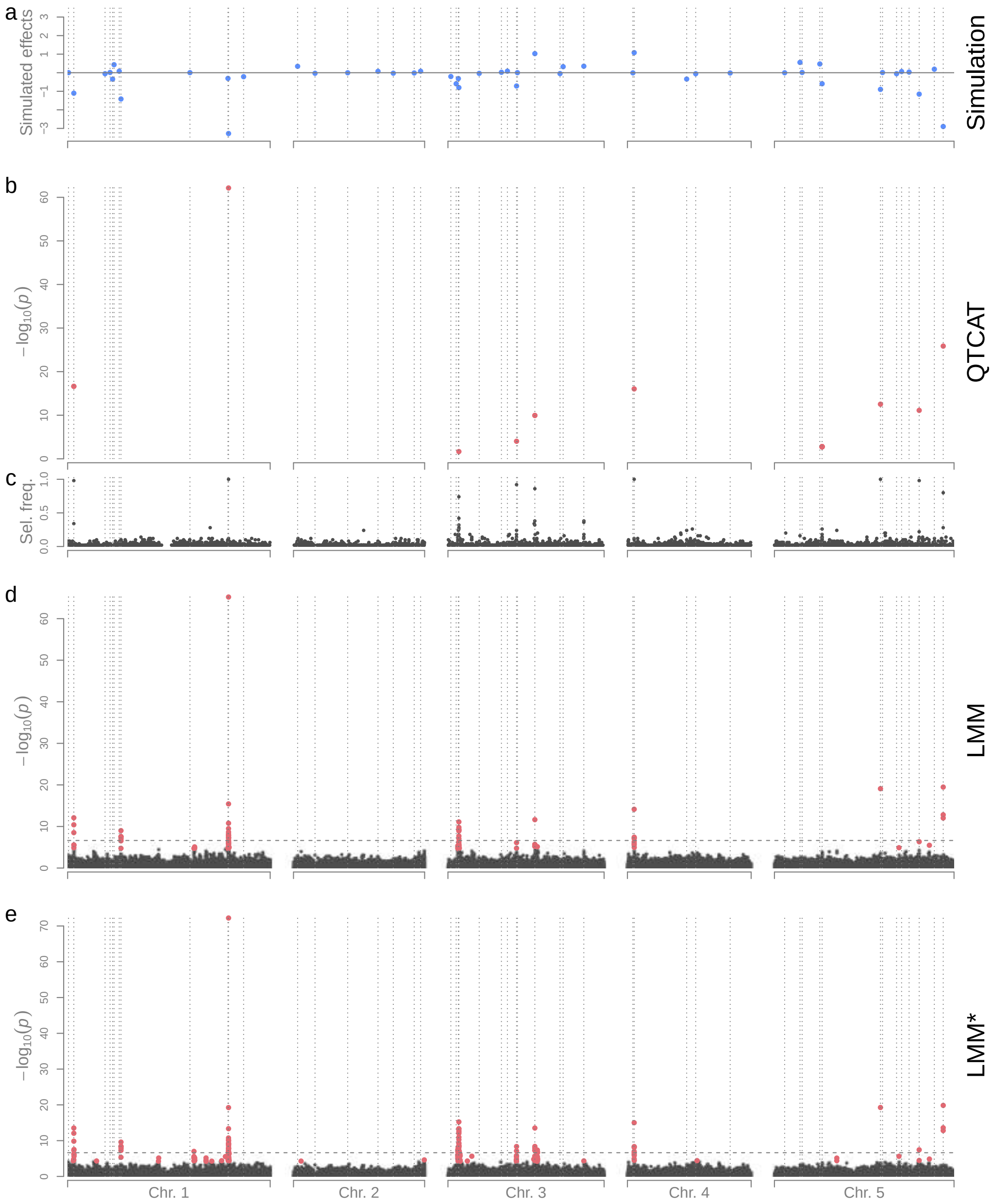

Supplementary Figure 9 Simulation of a GWA analysis based on a structured population with a heritability of 0.7 (run 9). (**a**) Simulation of 50 effects randomly drawn from a Gamma distribution and assigned to random markers. Markers with effect are highlighted with dashed lines. (**b**) Significant QTCs found by QTCAT. (**c**) LASSO selection frequency for each marker during the 50 iterations of QTCAT. (**d**) Manhattan plot of the LMM analysis. The horizontal dashed line depicts the significance threshold when controlling the multiple testing with FWER, whereas the red markers are significantly associated when controlling with FDR. (**e**) The Manhattan plot of the LMM* analysis. GRM was estimated without markers on the chromosome of the actual testing position. The results are shown as in (d).

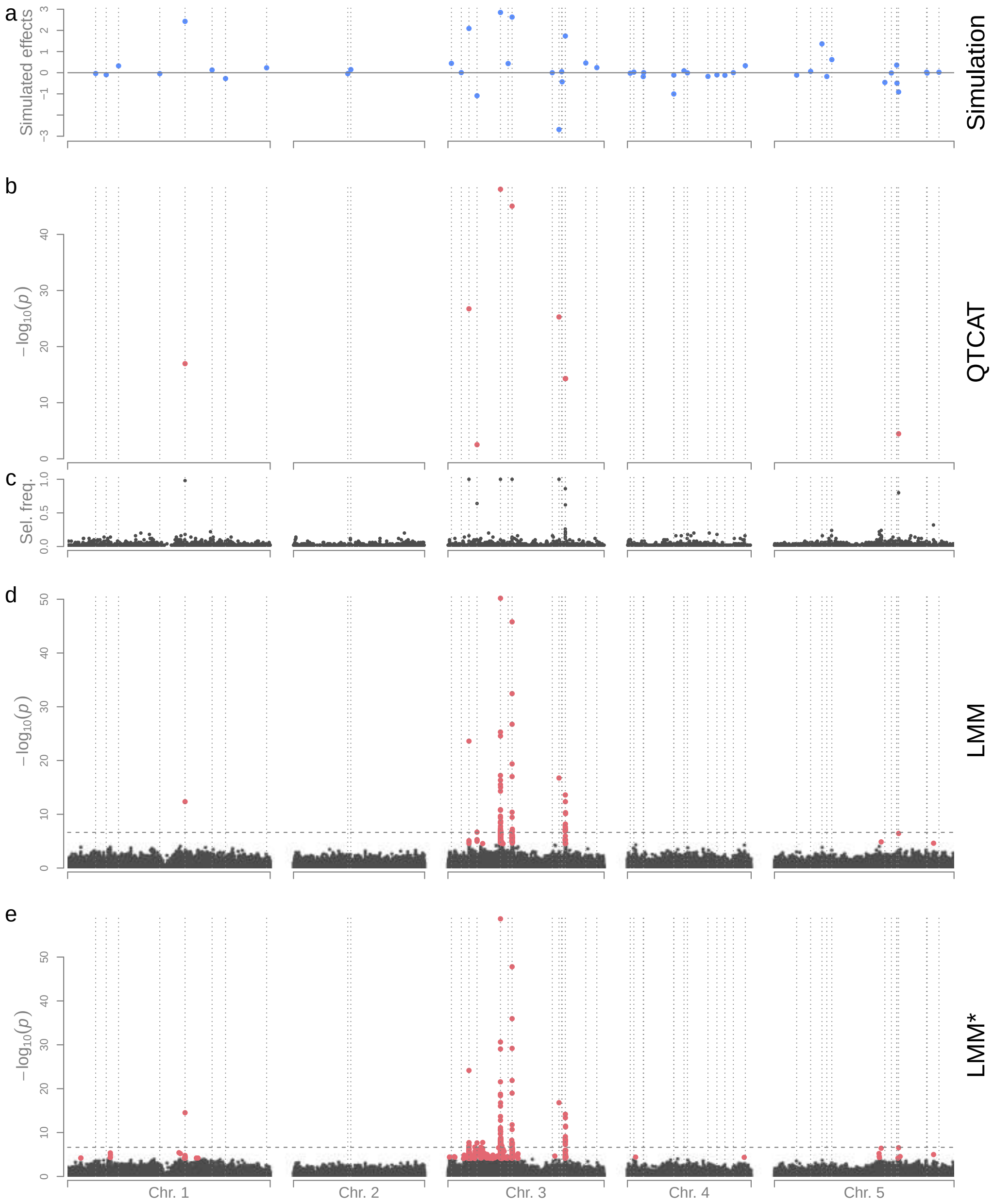

Supplementary Figure 10 Simulation of a GWA analysis based on a structured population with a heritability of 0.7 (run 10). (**a**) Simulation of 50 effects randomly drawn from a Gamma distribution and assigned to random markers. Markers with effect are highlighted with dashed lines. (**b**) Significant QTCs found by QTCAT. (**c**) LASSO selection frequency for each marker during the 50 iterations of QTCAT. (**d**) Manhattan plot of the LMM analysis. The horizontal dashed line depicts the significance threshold when controlling the multiple testing with FWER, whereas the red markers are significantly associated when controlling with FDR. (**e**) The Manhattan plot of the LMM* analysis. GRM was estimated without markers on the chromosome of the actual testing position. The results are shown as in (d).

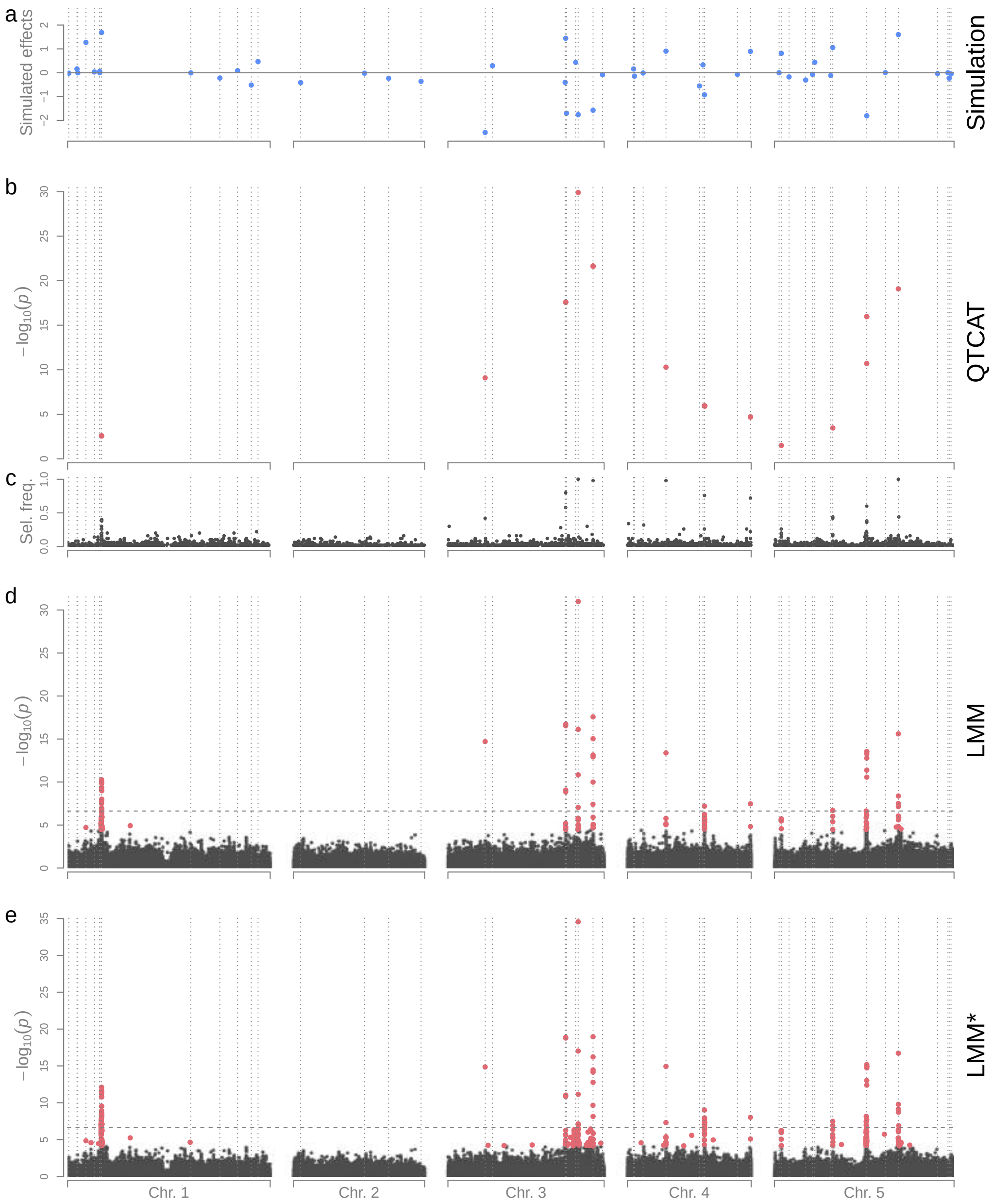

Supplementary Figure 11 Simulation of a GWA analysis based on a structured population with a heritability of 0.7 (run 11). (**a**) Simulation of 50 effects randomly drawn from a Gamma distribution and assigned to random markers. Markers with effect are highlighted with dashed lines. (**b**) Significant QTCs found by QTCAT. (**c**) LASSO selection frequency for each marker during the 50 iterations of QTCAT. (**d**) Manhattan plot of the LMM analysis. The horizontal dashed line depicts the significance threshold when controlling the multiple testing with FWER, whereas the red markers are significantly associated when controlling with FDR. (**e**) The Manhattan plot of the LMM* analysis. GRM was estimated without markers on the chromosome of the actual testing position. The results are shown as in (d).

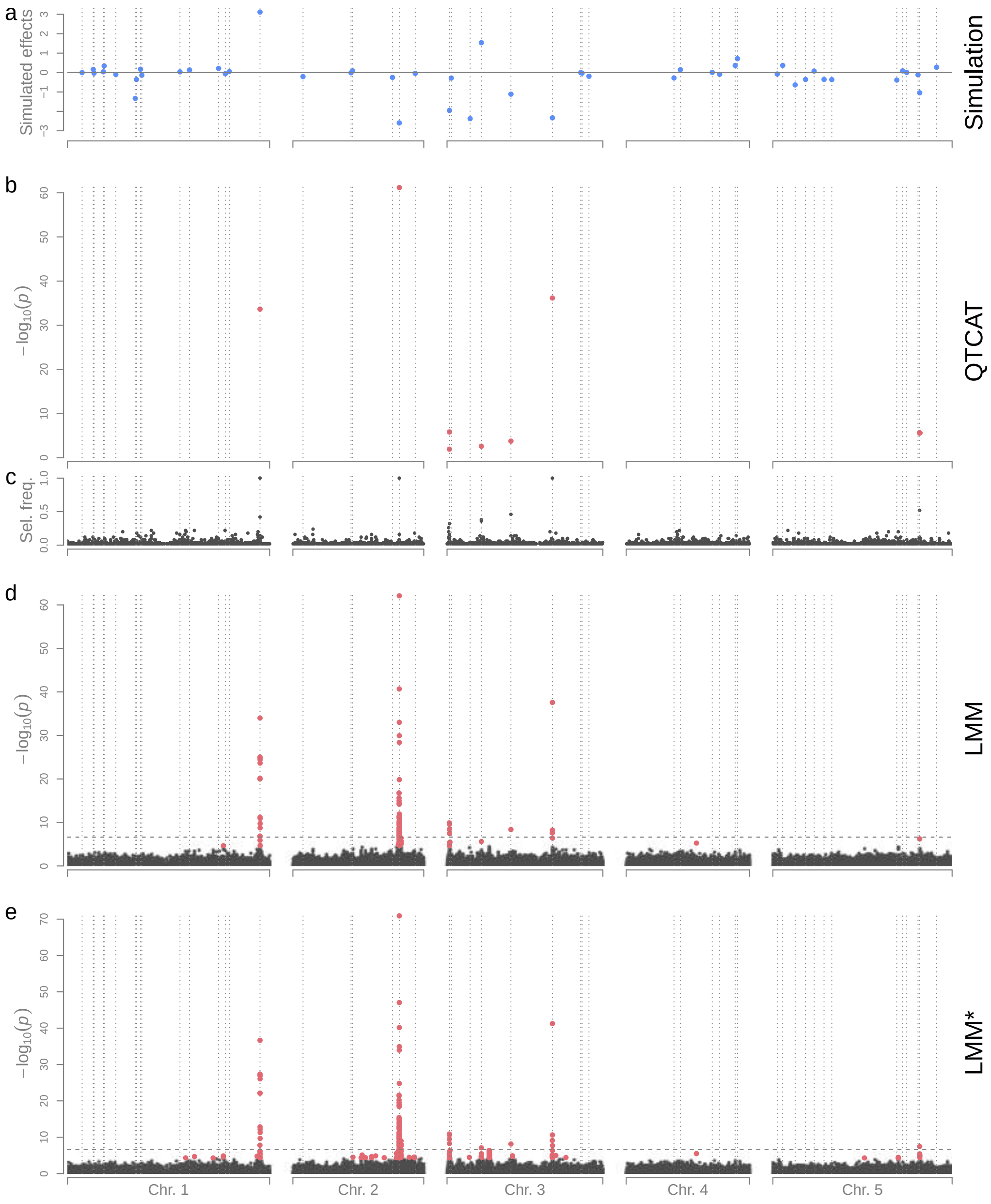

Supplementary Figure 12 Simulation of a GWA analysis based on a structured population with a heritability of 0.7 (run 12). (**a**) Simulation of 50 effects randomly drawn from a Gamma distribution and assigned to random markers. Markers with effect are highlighted with dashed lines. (**b**) Significant QTCs found by QTCAT. (**c**) LASSO selection frequency for each marker during the 50 iterations of QTCAT. (**d**) Manhattan plot of the LMM analysis. The horizontal dashed line depicts the significance threshold when controlling the multiple testing with FWER, whereas the red markers are significantly associated when controlling with FDR. (**e**) The Manhattan plot of the LMM* analysis. GRM was estimated without markers on the chromosome of the actual testing position. The results are shown as in (d).

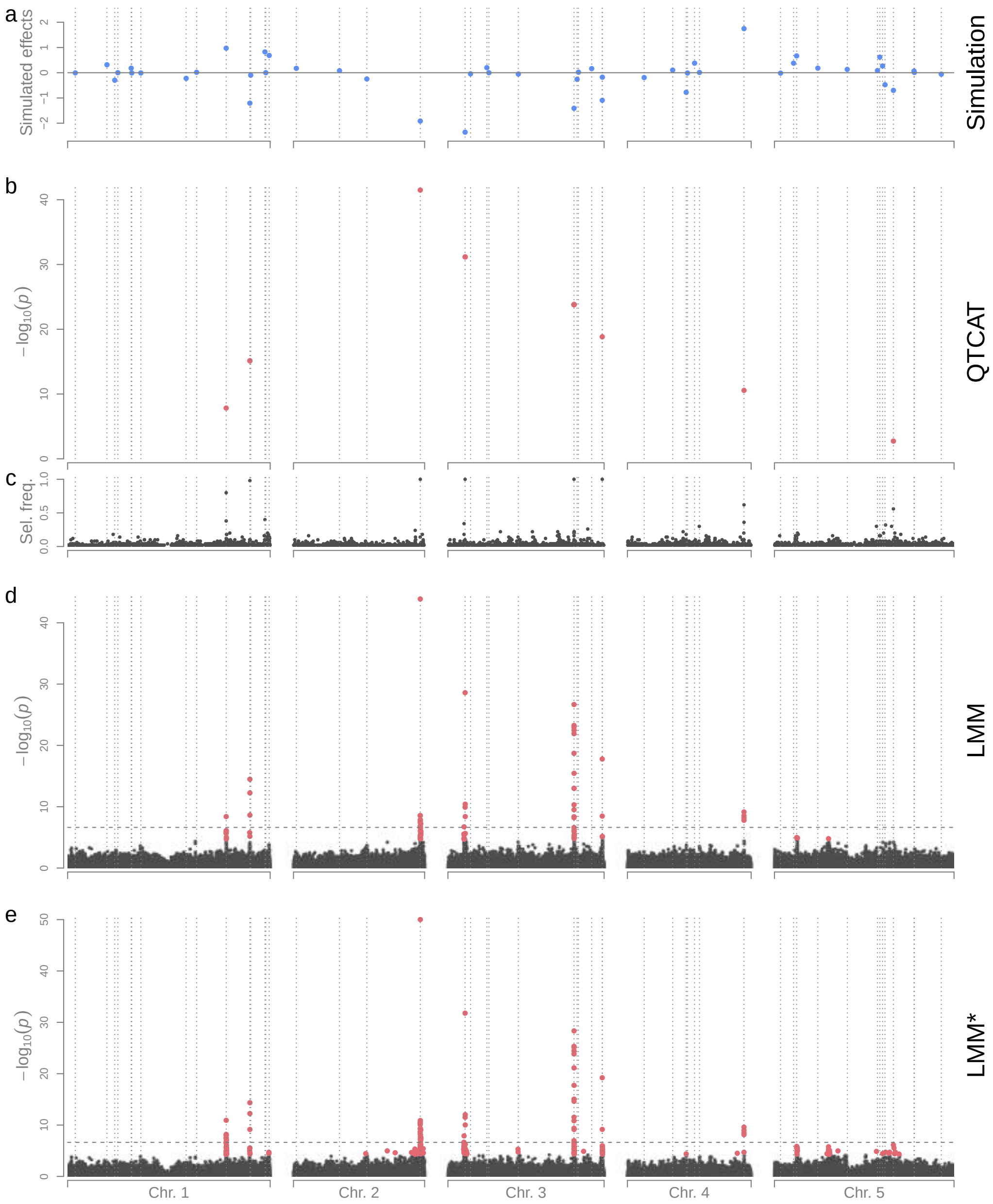

Supplementary Figure 13 Simulation of a GWA analysis based on a structured population with a heritability of 0.7 (run 13). (**a**) Simulation of 50 effects randomly drawn from a Gamma distribution and assigned to random markers. Markers with effect are highlighted with dashed lines. (**b**) Significant QTCs found by QTCAT. (**c**) LASSO selection frequency for each marker during the 50 iterations of QTCAT. (**d**) Manhattan plot of the LMM analysis. The horizontal dashed line depicts the significance threshold when controlling the multiple testing with FWER, whereas the red markers are significantly associated when controlling with FDR. (**e**) The Manhattan plot of the LMM* analysis. GRM was estimated without markers on the chromosome of the actual testing position. The results are shown as in (d).

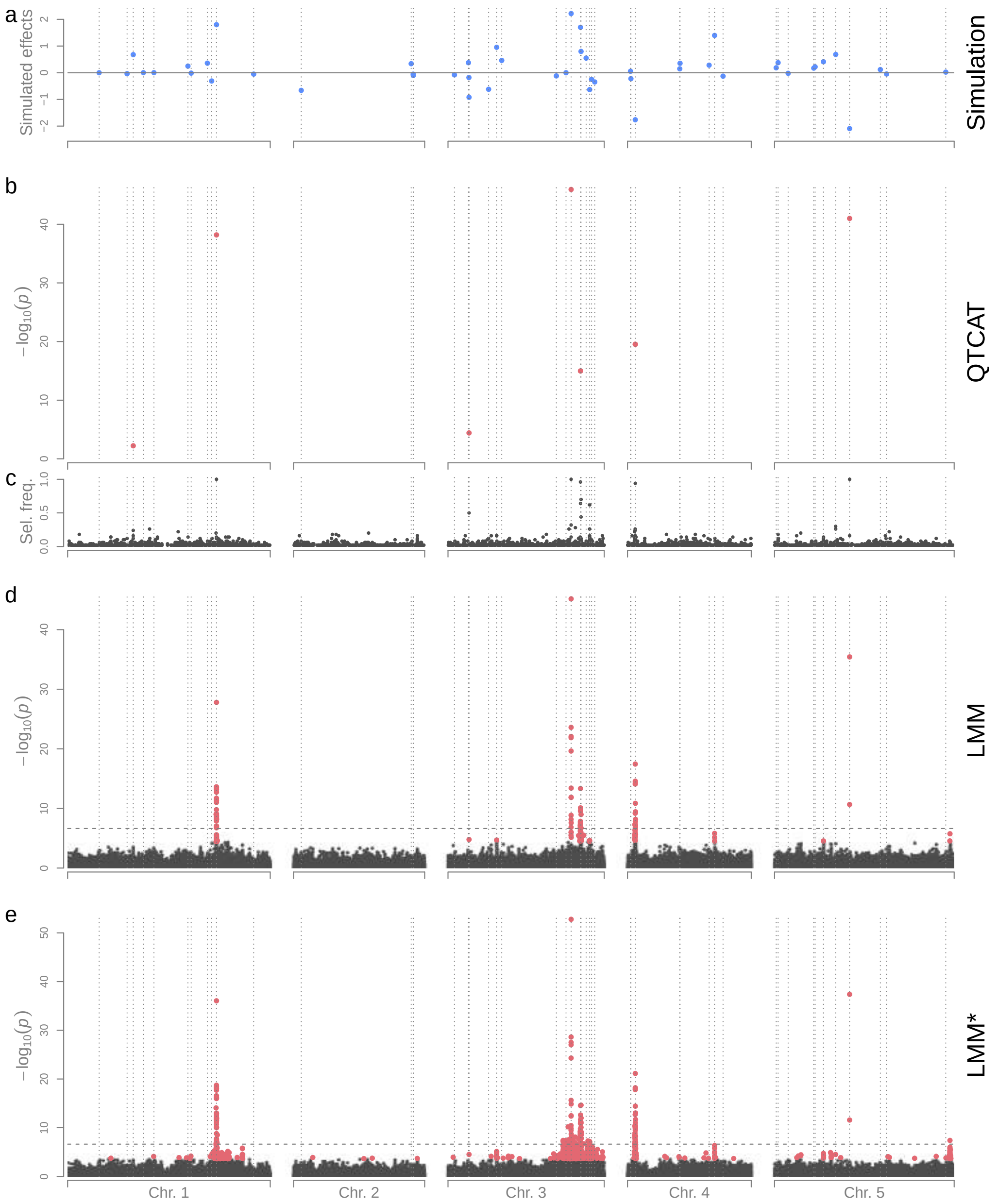

Supplementary Figure 14 Simulation of a GWA analysis based on a structured population with a heritability of 0.7 (run 14). (**a**) Simulation of 50 effects randomly drawn from a Gamma distribution and assigned to random markers. Markers with effect are highlighted with dashed lines. (**b**) Significant QTCs found by QTCAT. (**c**) LASSO selection frequency for each marker during the 50 iterations of QTCAT. (**d**) Manhattan plot of the LMM analysis. The horizontal dashed line depicts the significance threshold when controlling the multiple testing with FWER, whereas the red markers are significantly associated when controlling with FDR. (**e**) The Manhattan plot of the LMM* analysis. GRM was estimated without markers on the chromosome of the actual testing position. The results are shown as in (d).

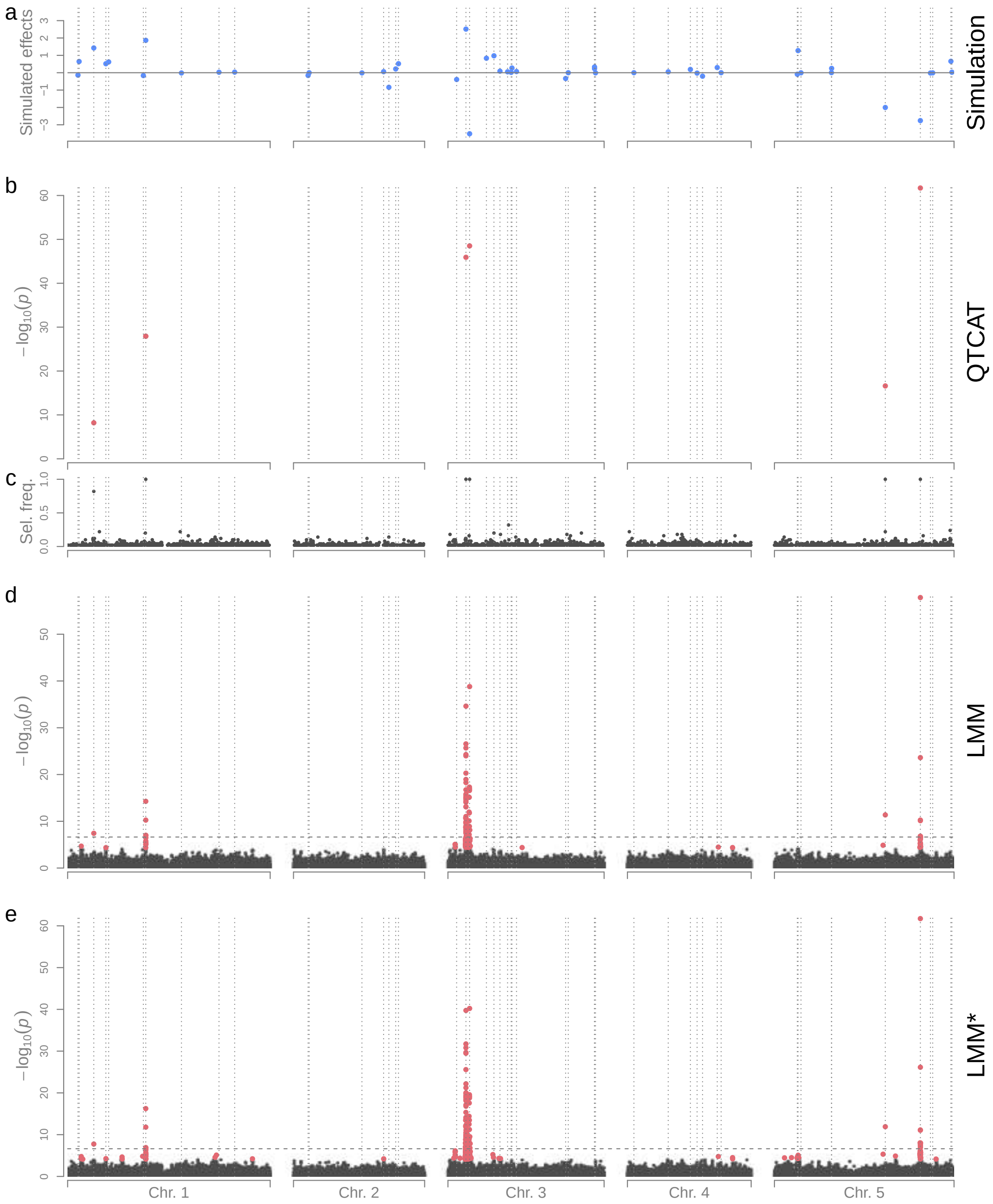

Supplementary Figure 15 Simulation of a GWA analysis based on a structured population with a heritability of 0.7 (run 15). (**a**) Simulation of 50 effects randomly drawn from a Gamma distribution and assigned to random markers. Markers with effect are highlighted with dashed lines. (**b**) Significant QTCs found by QTCAT. (**c**) LASSO selection frequency for each marker during the 50 iterations of QTCAT. (**d**) Manhattan plot of the LMM analysis. The horizontal dashed line depicts the significance threshold when controlling the multiple testing with FWER, whereas the red markers are significantly associated when controlling with FDR. (**e**) The Manhattan plot of the LMM* analysis. GRM was estimated without markers on the chromosome of the actual testing position. The results are shown as in (d).

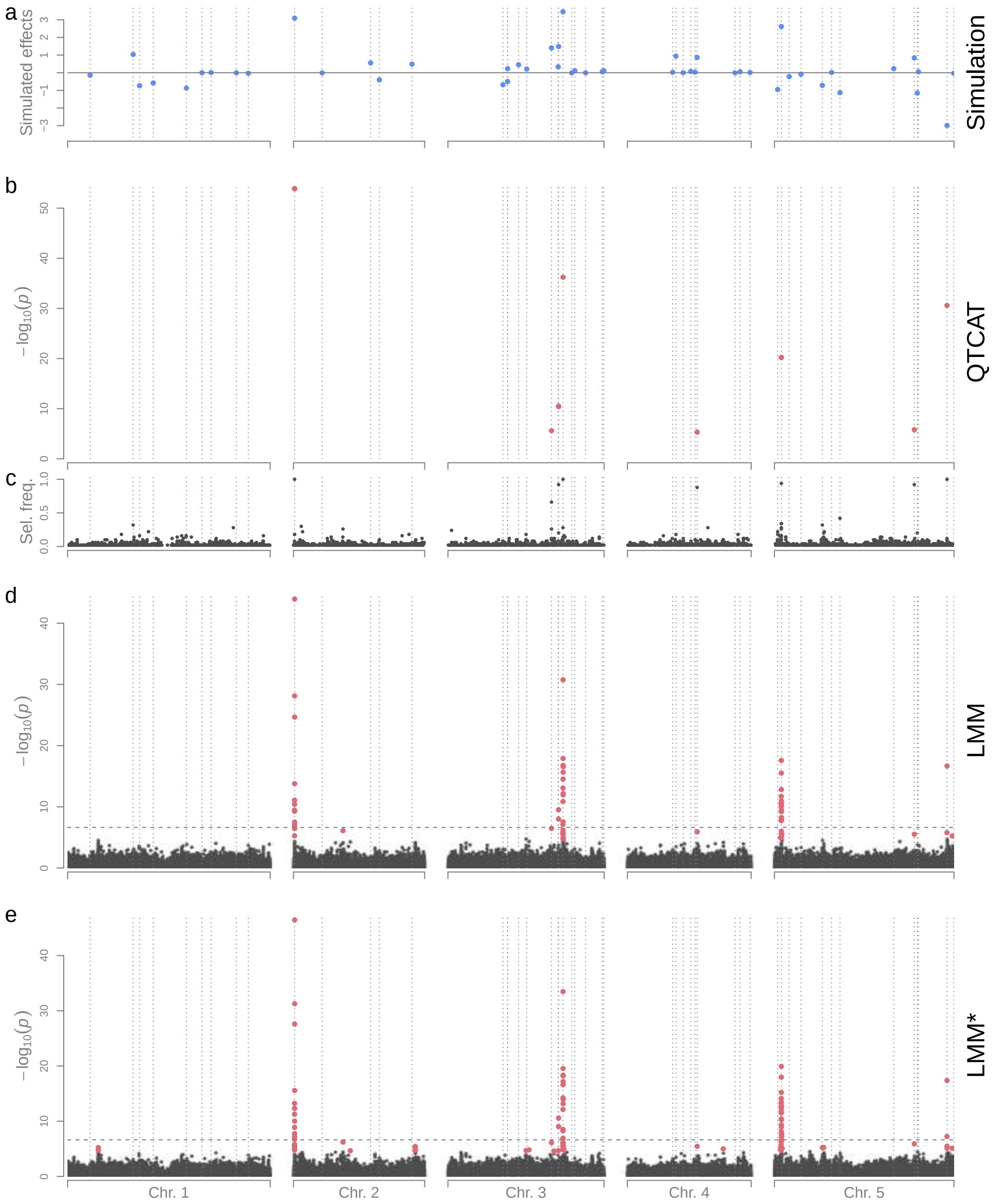

Supplementary Figure 16 Simulation of a GWA analysis based on a structured population with a heritability of 0.7 (run 16). (**a**) Simulation of 50 effects randomly drawn from a Gamma distribution and assigned to random markers. Markers with effect are highlighted with dashed lines. (**b**) Significant QTCs found by QTCAT. (**c**) LASSO selection frequency for each marker during the 50 iterations of QTCAT. (**d**) Manhattan plot of the LMM analysis. The horizontal dashed line depicts the significance threshold when controlling the multiple testing with FWER, whereas the red markers are significantly associated when controlling with FDR. (**e**) The Manhattan plot of the LMM* analysis. GRM was estimated without markers on the chromosome of the actual testing position. The results are shown as in (d).

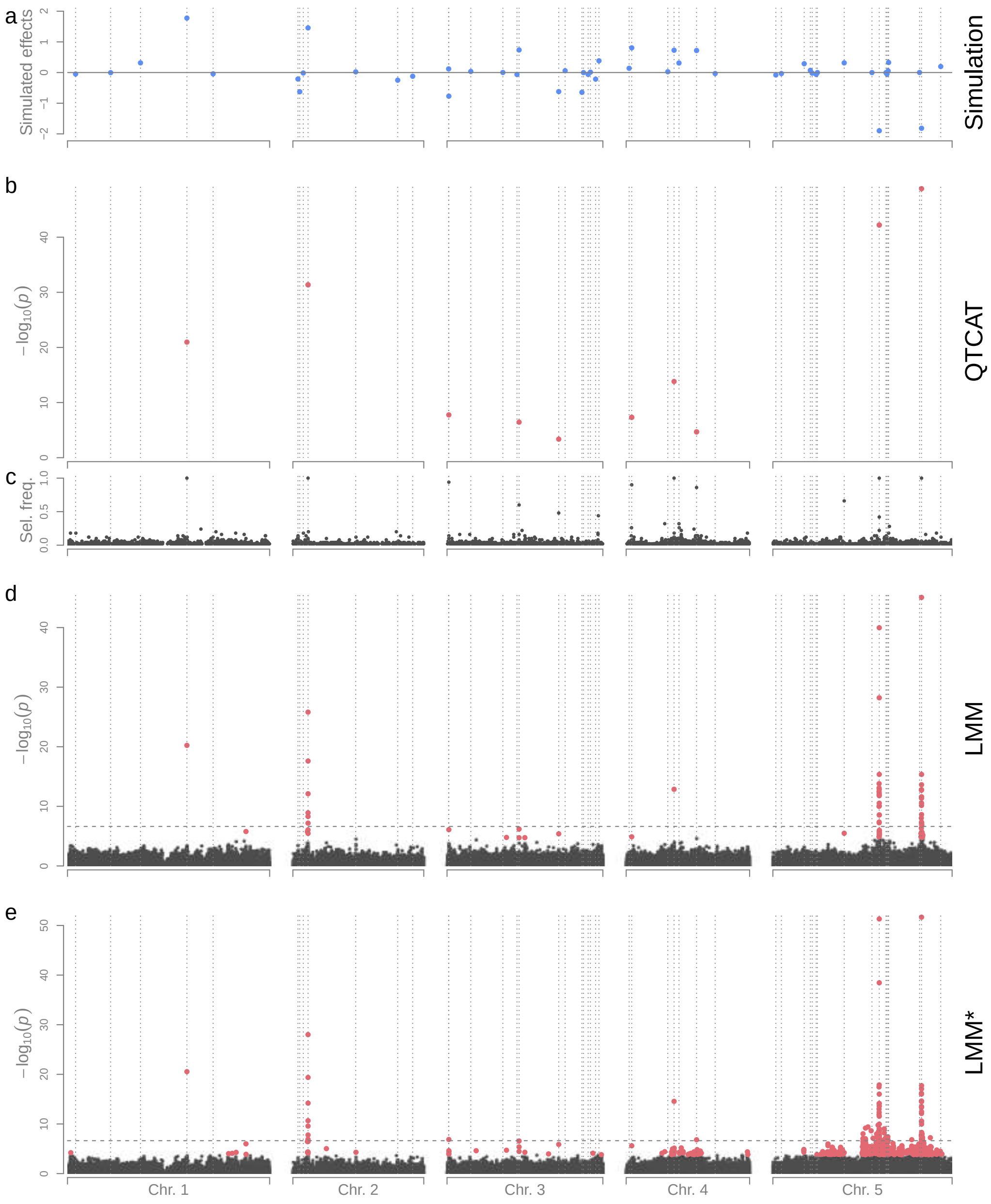

Supplementary Figure 17 Simulation of a GWA analysis based on a structured population with a heritability of 0.7 (run 17). (**a**) Simulation of 50 effects randomly drawn from a Gamma distribution and assigned to random markers. Markers with effect are highlighted with dashed lines. (**b**) Significant QTCs found by QTCAT. (**c**) LASSO selection frequency for each marker during the 50 iterations of QTCAT. (**d**) Manhattan plot of the LMM analysis. The horizontal dashed line depicts the significance threshold when controlling the multiple testing with FWER, whereas the red markers are significantly associated when controlling with FDR. (**e**) The Manhattan plot of the LMM* analysis. GRM was estimated without markers on the chromosome of the actual testing position. The results are shown as in (d).

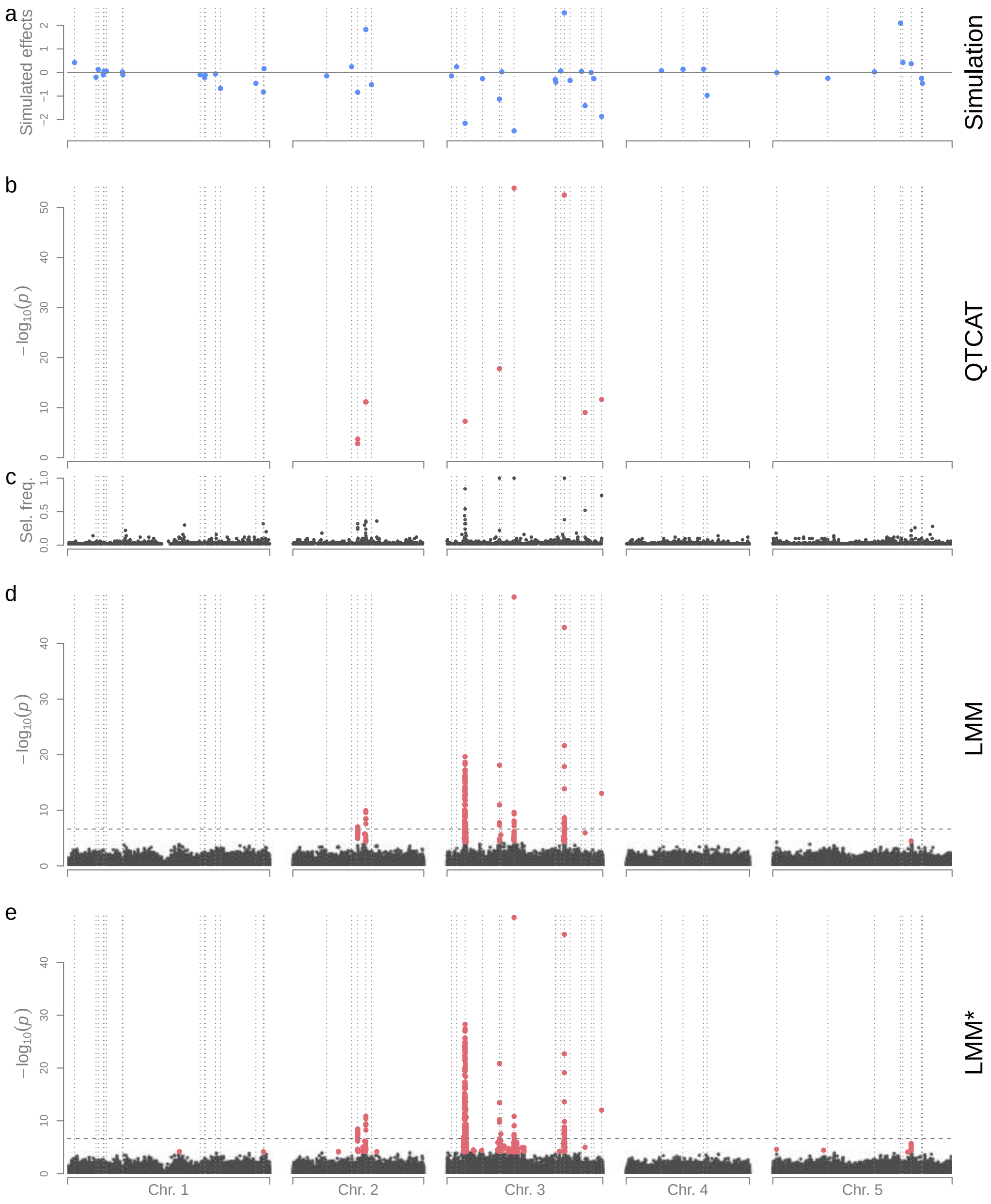

Supplementary Figure 18 Simulation of a GWA analysis based on a structured population with a heritability of 0.7 (run 18). (**a**) Simulation of 50 effects randomly drawn from a Gamma distribution and assigned to random markers. Markers with effect are highlighted with dashed lines. (**b**) Significant QTCs found by QTCAT. (**c**) LASSO selection frequency for each marker during the 50 iterations of QTCAT. (**d**) Manhattan plot of the LMM analysis. The horizontal dashed line depicts the significance threshold when controlling the multiple testing with FWER, whereas the red markers are significantly associated when controlling with FDR. (**e**) The Manhattan plot of the LMM* analysis. GRM was estimated without markers on the chromosome of the actual testing position. The results are shown as in (d).

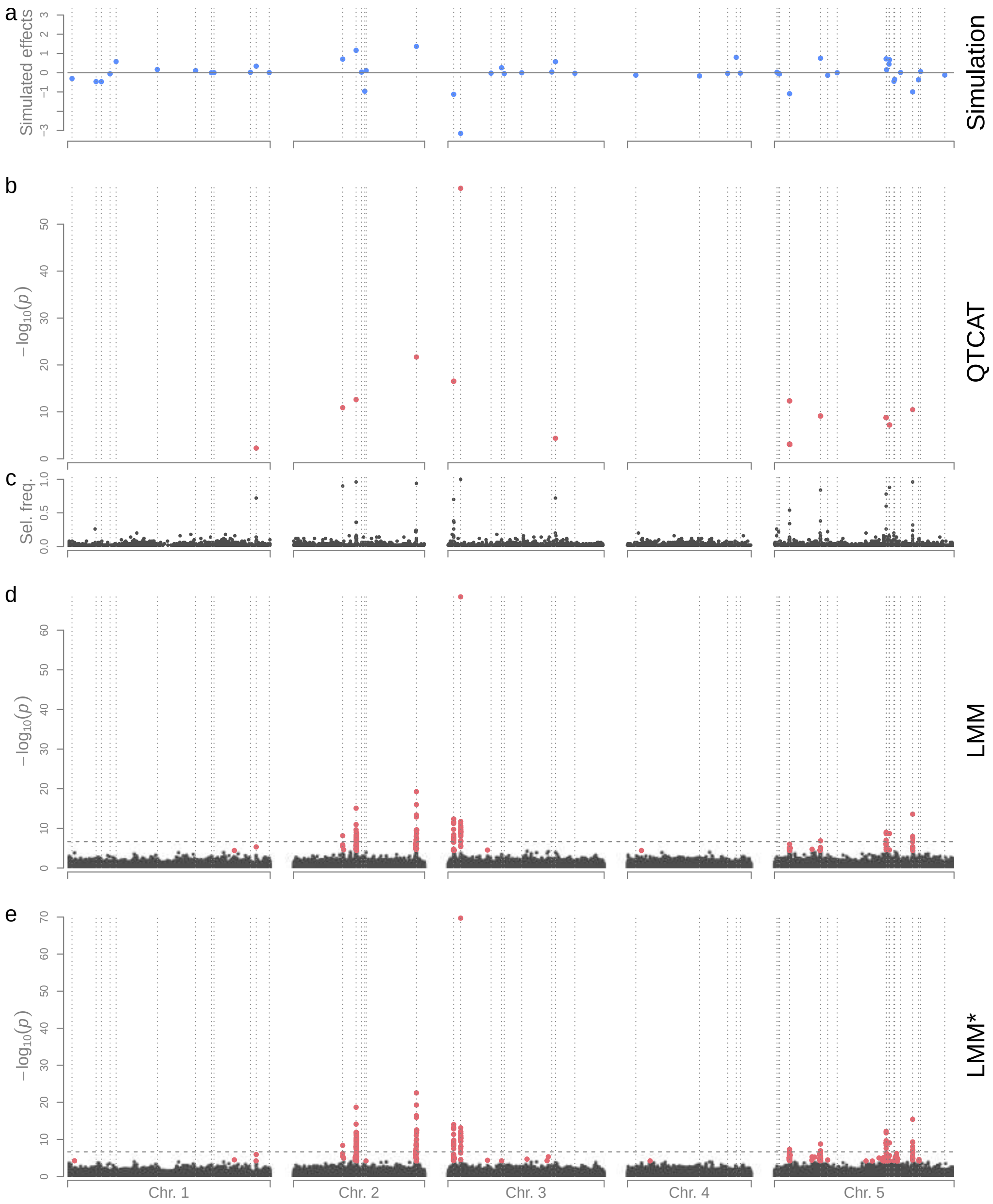

Supplementary Figure 19 Simulation of a GWA analysis based on a structured population with a heritability of 0.7 (run 19). (**a**) Simulation of 50 effects randomly drawn from a Gamma distribution and assigned to random markers. Markers with effect are highlighted with dashed lines. (**b**) Significant QTCs found by QTCAT. (**c**) LASSO selection frequency for each marker during the 50 iterations of QTCAT. (**d**) Manhattan plot of the LMM analysis. The horizontal dashed line depicts the significance threshold when controlling the multiple testing with FWER, whereas the red markers are significantly associated when controlling with FDR. (**e**) The Manhattan plot of the LMM* analysis. GRM was estimated without markers on the chromosome of the actual testing position. The results are shown as in (d).

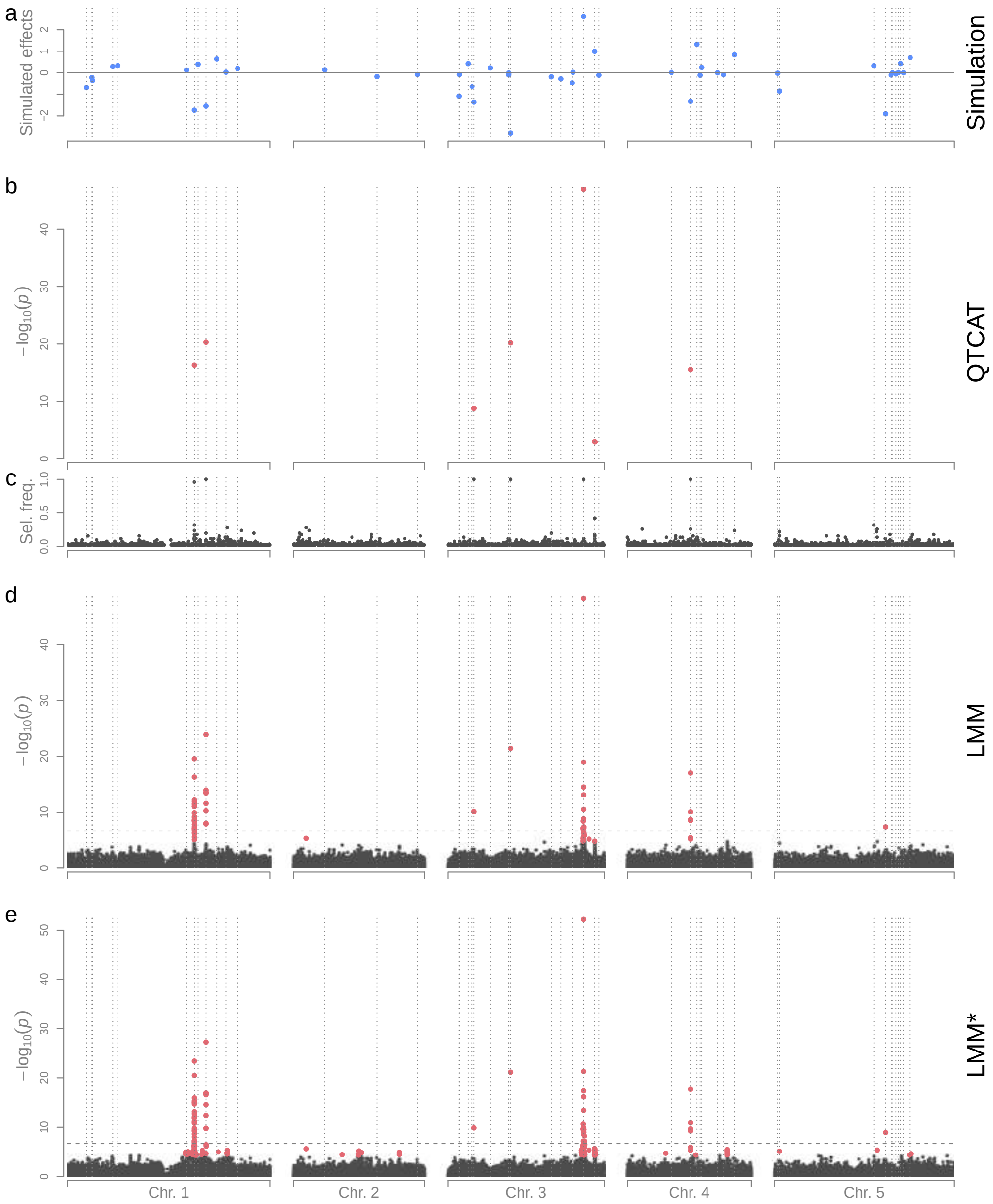

Supplementary Figure 20 Simulation of a GWA analysis based on a structured population with a heritability of 0.7 (run 20). (**a**) Simulation of 50 effects randomly drawn from a Gamma distribution and assigned to random markers. Markers with effect are highlighted with dashed lines. (**b**) Significant QTCs found by QTCAT. (**c**) LASSO selection frequency for each marker during the 50 iterations of QTCAT. (**d**) Manhattan plot of the LMM analysis. The horizontal dashed line depicts the significance threshold when controlling the multiple testing with FWER, whereas the red markers are significantly associated when controlling with FDR. (**e**) The Manhattan plot of the LMM* analysis. GRM was estimated without markers on the chromosome of the actual testing position. The results are shown as in (d).

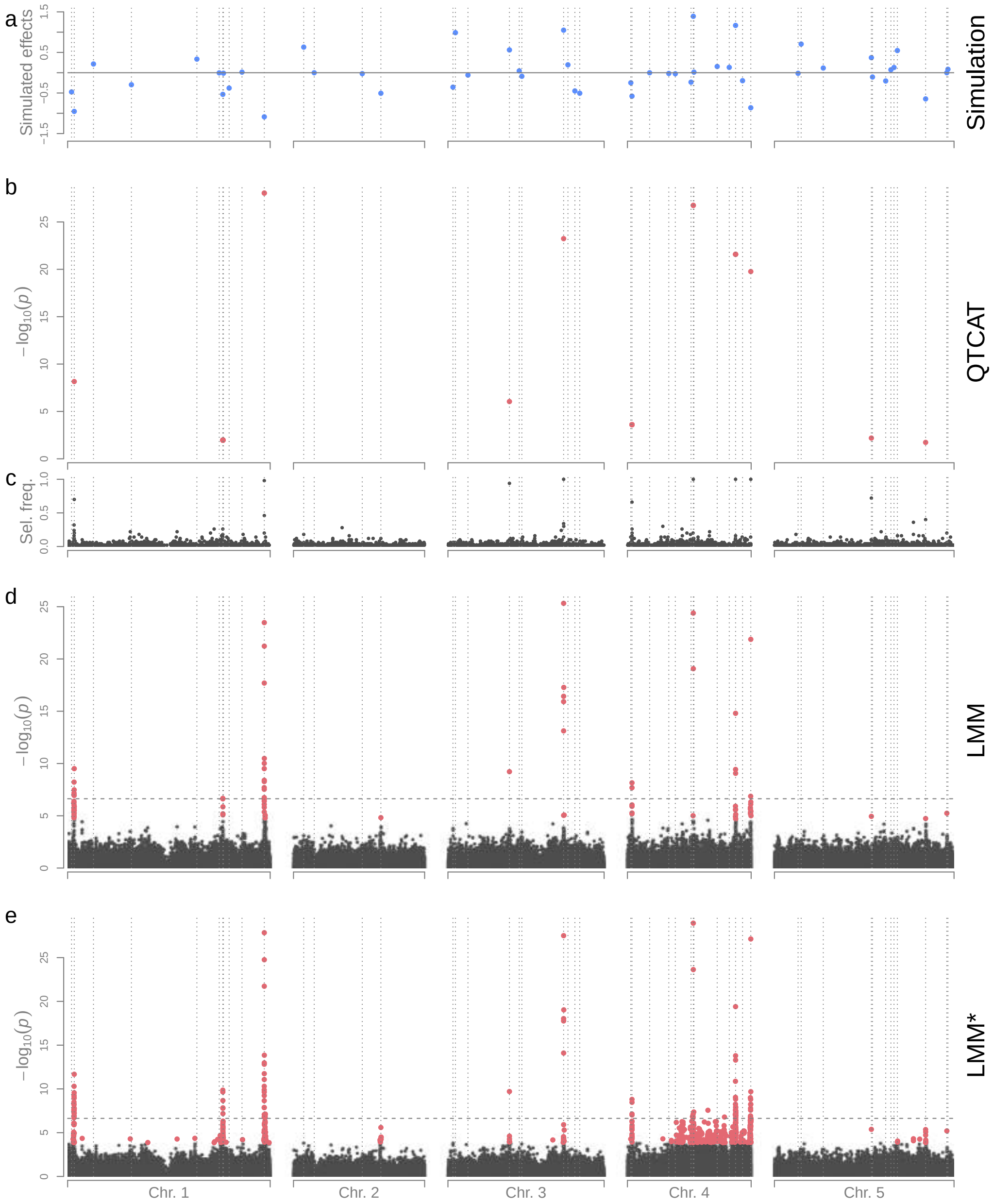

Supplementary Figure 21 Simulation of a GWA analysis based on a structured population with a heritability of 0.7 (run 21). (**a**) Simulation of 50 effects randomly drawn from a Gamma distribution and assigned to random markers. Markers with effect are highlighted with dashed lines. (**b**) Significant QTCs found by QTCAT. (**c**) LASSO selection frequency for each marker during the 50 iterations of QTCAT. (**d**) Manhattan plot of the LMM analysis. The horizontal dashed line depicts the significance threshold when controlling the multiple testing with FWER, whereas the red markers are significantly associated when controlling with FDR. (**e**) The Manhattan plot of the LMM* analysis. GRM was estimated without markers on the chromosome of the actual testing position. The results are shown as in (d).

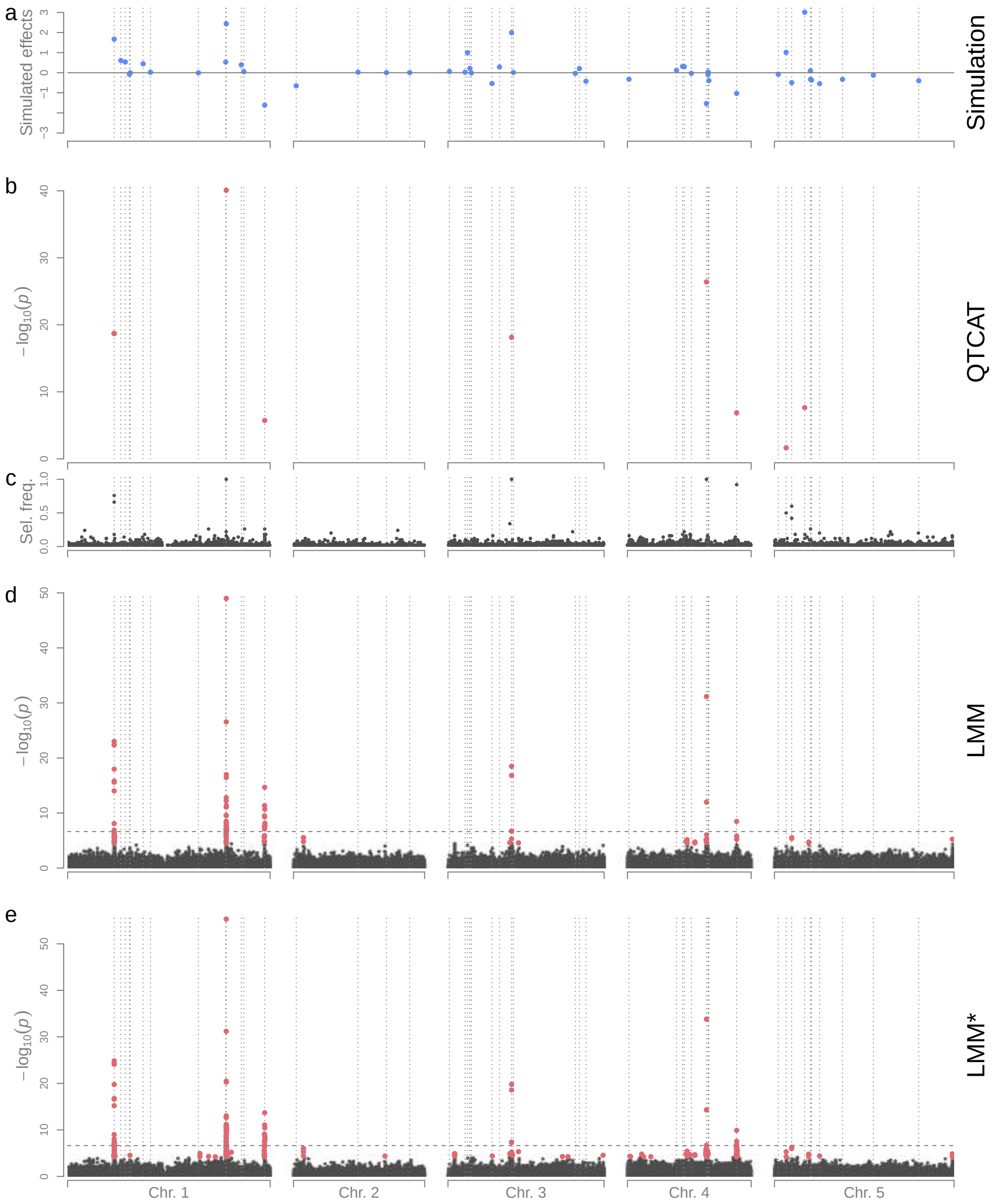

Supplementary Figure 22 Simulation of a GWA analysis based on a structured population with a heritability of 0.7 (run 22). (**a**) Simulation of 50 effects randomly drawn from a Gamma distribution and assigned to random markers. Markers with effect are highlighted with dashed lines. (**b**) Significant QTCs found by QTCAT. (**c**) LASSO selection frequency for each marker during the 50 iterations of QTCAT. (**d**) Manhattan plot of the LMM analysis. The horizontal dashed line depicts the significance threshold when controlling the multiple testing with FWER, whereas the red markers are significantly associated when controlling with FDR. (**e**) The Manhattan plot of the LMM* analysis. GRM was estimated without markers on the chromosome of the actual testing position. The results are shown as in (d).

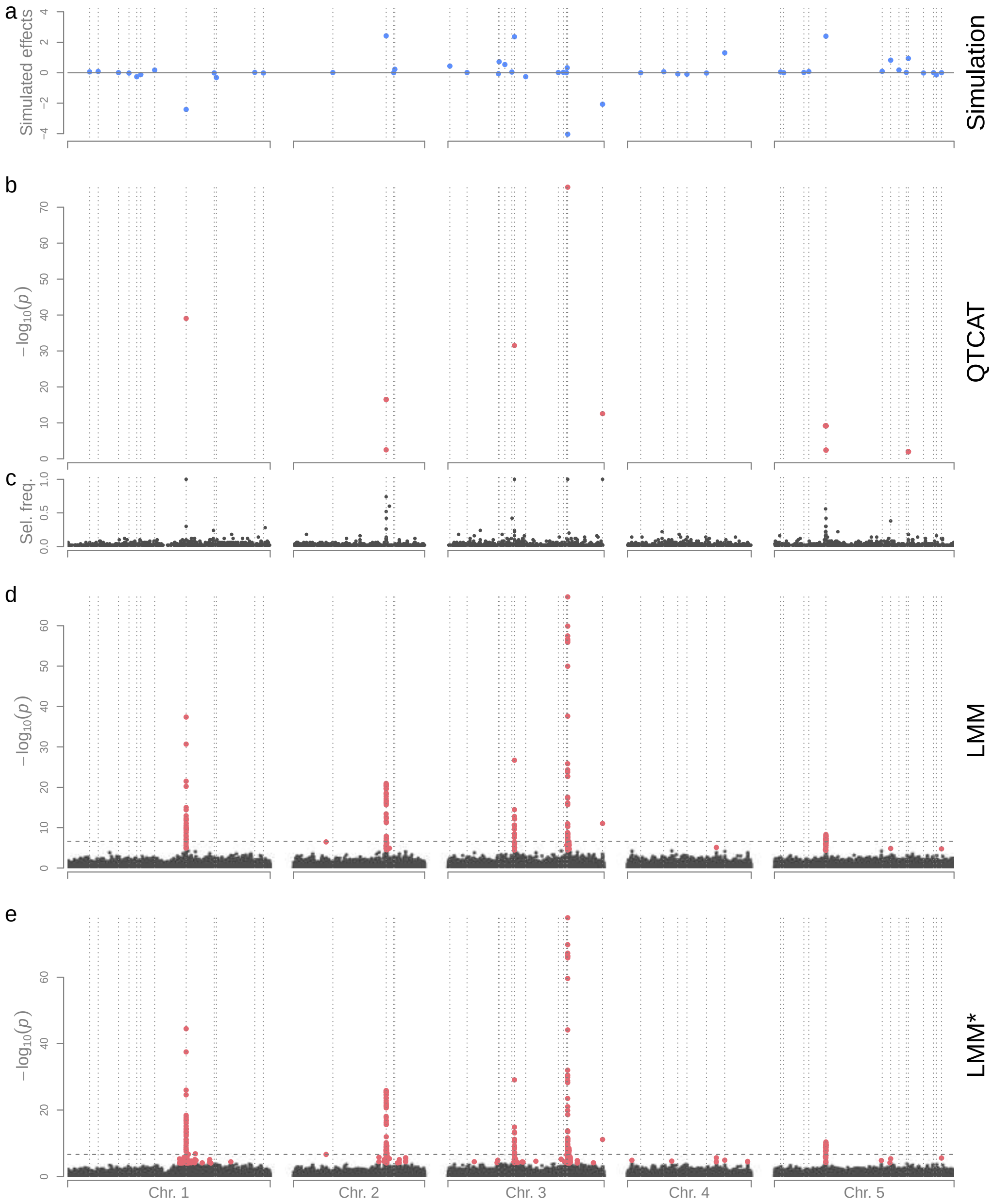

Supplementary Figure 23 Simulation of a GWA analysis based on a structured population with a heritability of 0.7 (run 23). (**a**) Simulation of 50 effects randomly drawn from a Gamma distribution and assigned to random markers. Markers with effect are highlighted with dashed lines. (**b**) Significant QTCs found by QTCAT. (**c**) LASSO selection frequency for each marker during the 50 iterations of QTCAT. (**d**) Manhattan plot of the LMM analysis. The horizontal dashed line depicts the significance threshold when controlling the multiple testing with FWER, whereas the red markers are significantly associated when controlling with FDR. (**e**) The Manhattan plot of the LMM* analysis. GRM was estimated without markers on the chromosome of the actual testing position. The results are shown as in (d).

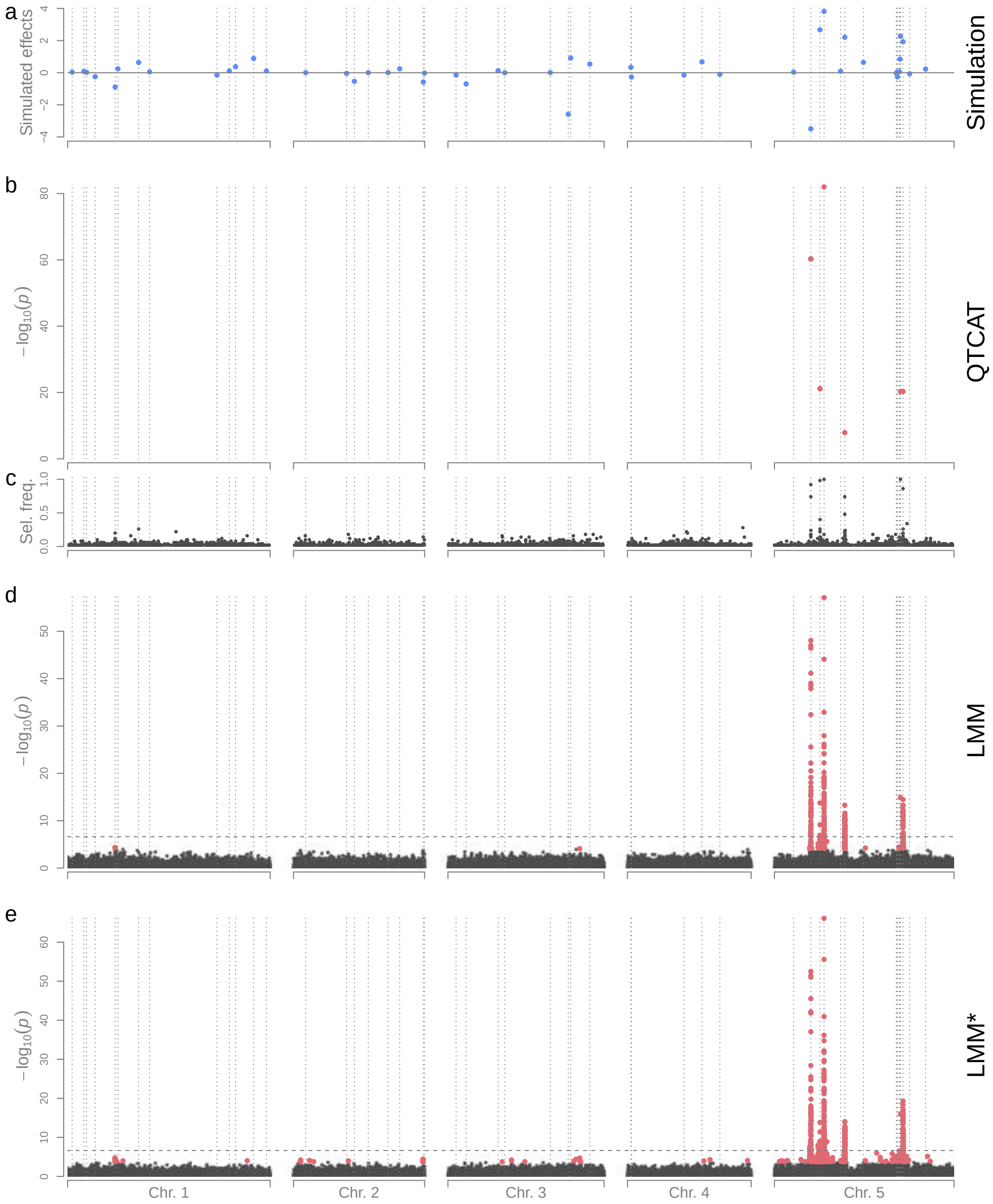

Supplementary Figure 24 Simulation of a GWA analysis based on a structured population with a heritability of 0.7 (run 24). (**a**) Simulation of 50 effects randomly drawn from a Gamma distribution and assigned to random markers. Markers with effect are highlighted with dashed lines. (**b**) Significant QTCs found by QTCAT. (**c**) LASSO selection frequency for each marker during the 50 iterations of QTCAT. (**d**) Manhattan plot of the LMM analysis. The horizontal dashed line depicts the significance threshold when controlling the multiple testing with FWER, whereas the red markers are significantly associated when controlling with FDR. (**e**) The Manhattan plot of the LMM* analysis. GRM was estimated without markers on the chromosome of the actual testing position. The results are shown as in (d).

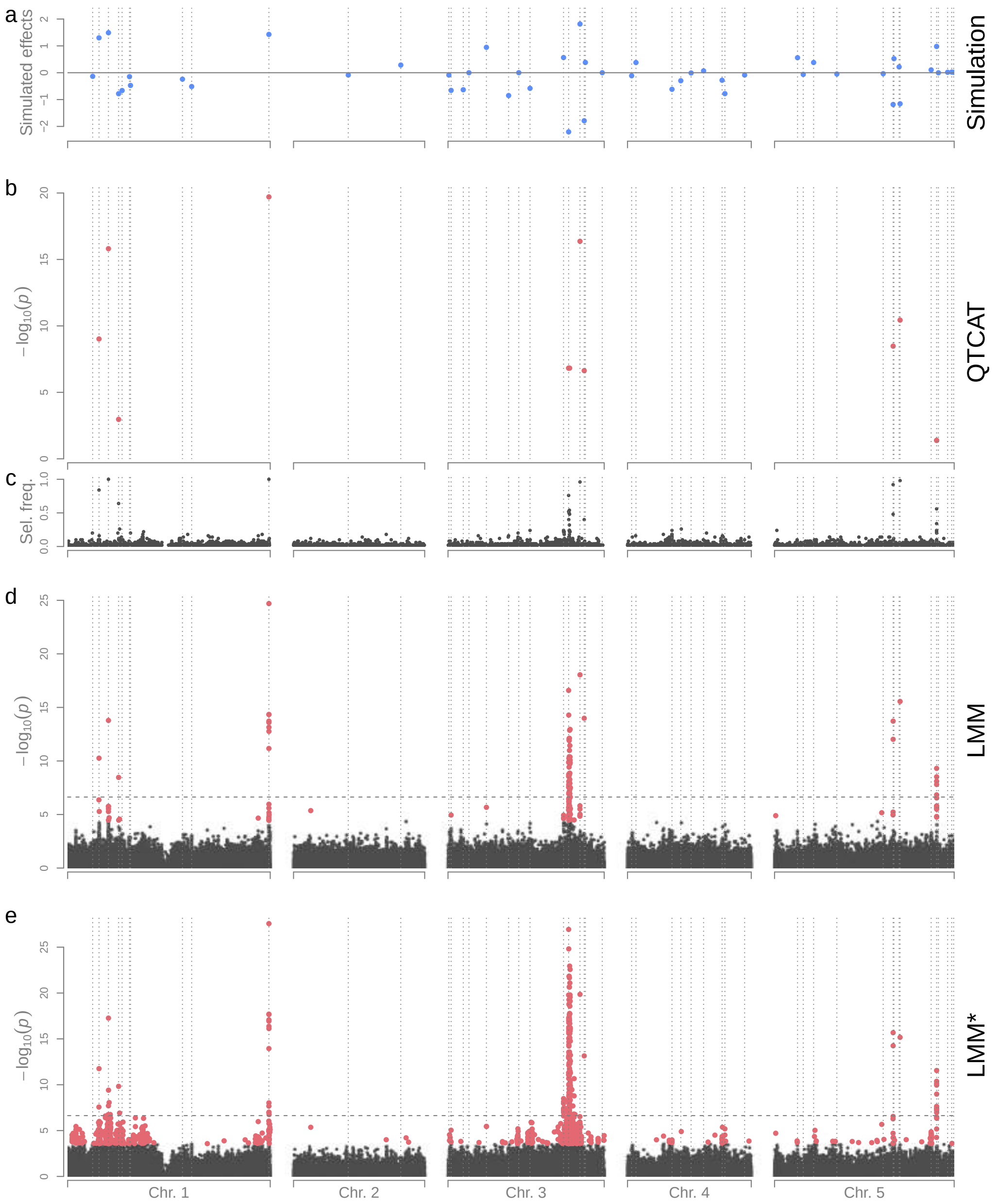

Supplementary Figure 25 Simulation of a GWA analysis based on a structured population with a heritability of 0.7 (run 25). (**a**) Simulation of 50 effects randomly drawn from a Gamma distribution and assigned to random markers. Markers with effect are highlighted with dashed lines. (**b**) Significant QTCs found by QTCAT. (**c**) LASSO selection frequency for each marker during the 50 iterations of QTCAT. (**d**) Manhattan plot of the LMM analysis. The horizontal dashed line depicts the significance threshold when controlling the multiple testing with FWER, whereas the red markers are significantly associated when controlling with FDR. (**e**) The Manhattan plot of the LMM* analysis. GRM was estimated without markers on the chromosome of the actual testing position. The results are shown as in (d).

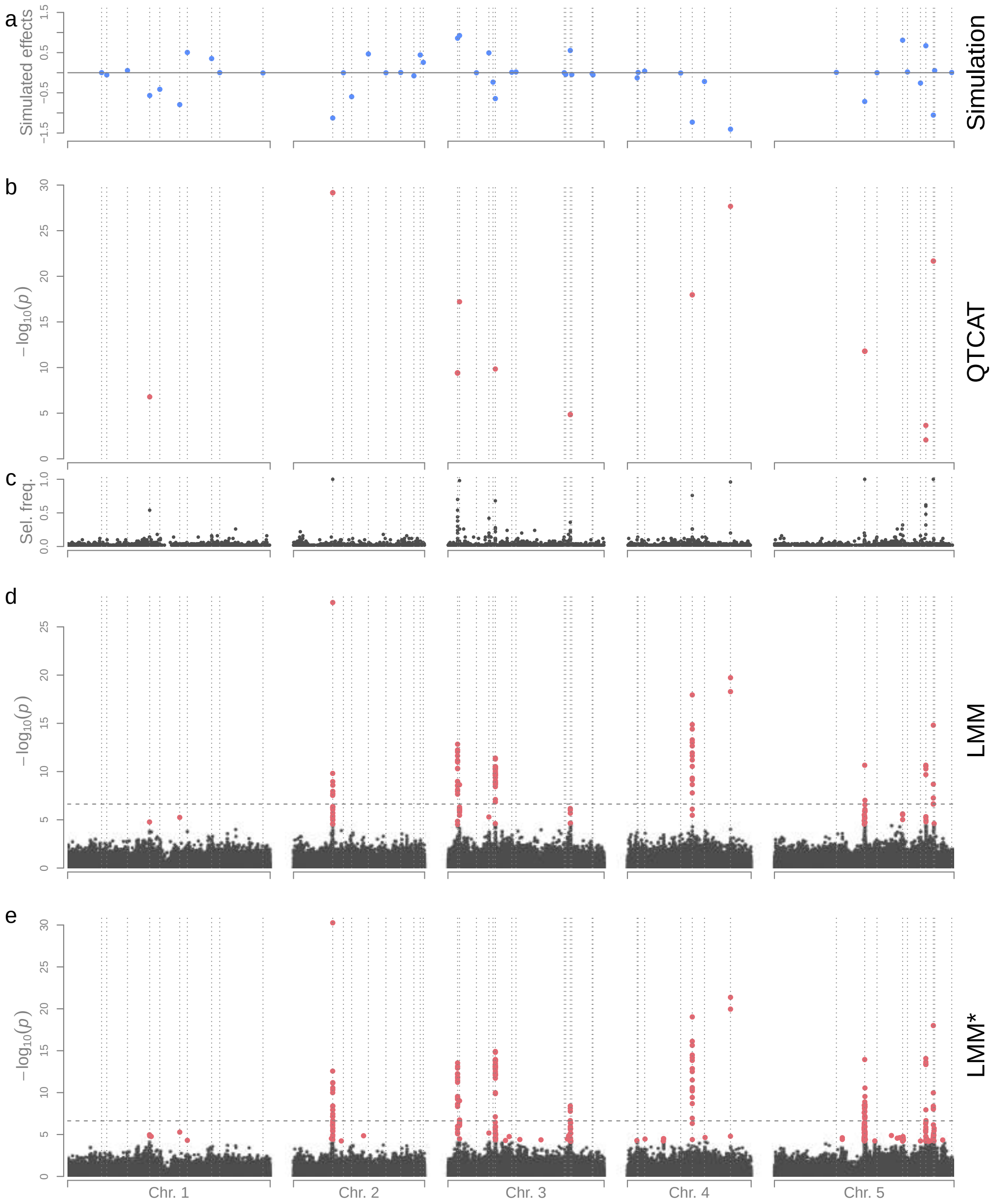

Supplementary Figure 26 Simulation of a GWA analysis based on a structured population with a heritability of 0.7 (run 26). (**a**) Simulation of 50 effects randomly drawn from a Gamma distribution and assigned to random markers. Markers with effect are highlighted with dashed lines. (**b**) Significant QTCs found by QTCAT. (**c**) LASSO selection frequency for each marker during the 50 iterations of QTCAT. (**d**) Manhattan plot of the LMM analysis. The horizontal dashed line depicts the significance threshold when controlling the multiple testing with FWER, whereas the red markers are significantly associated when controlling with FDR. (**e**) The Manhattan plot of the LMM* analysis. GRM was estimated without markers on the chromosome of the actual testing position. The results are shown as in (d).

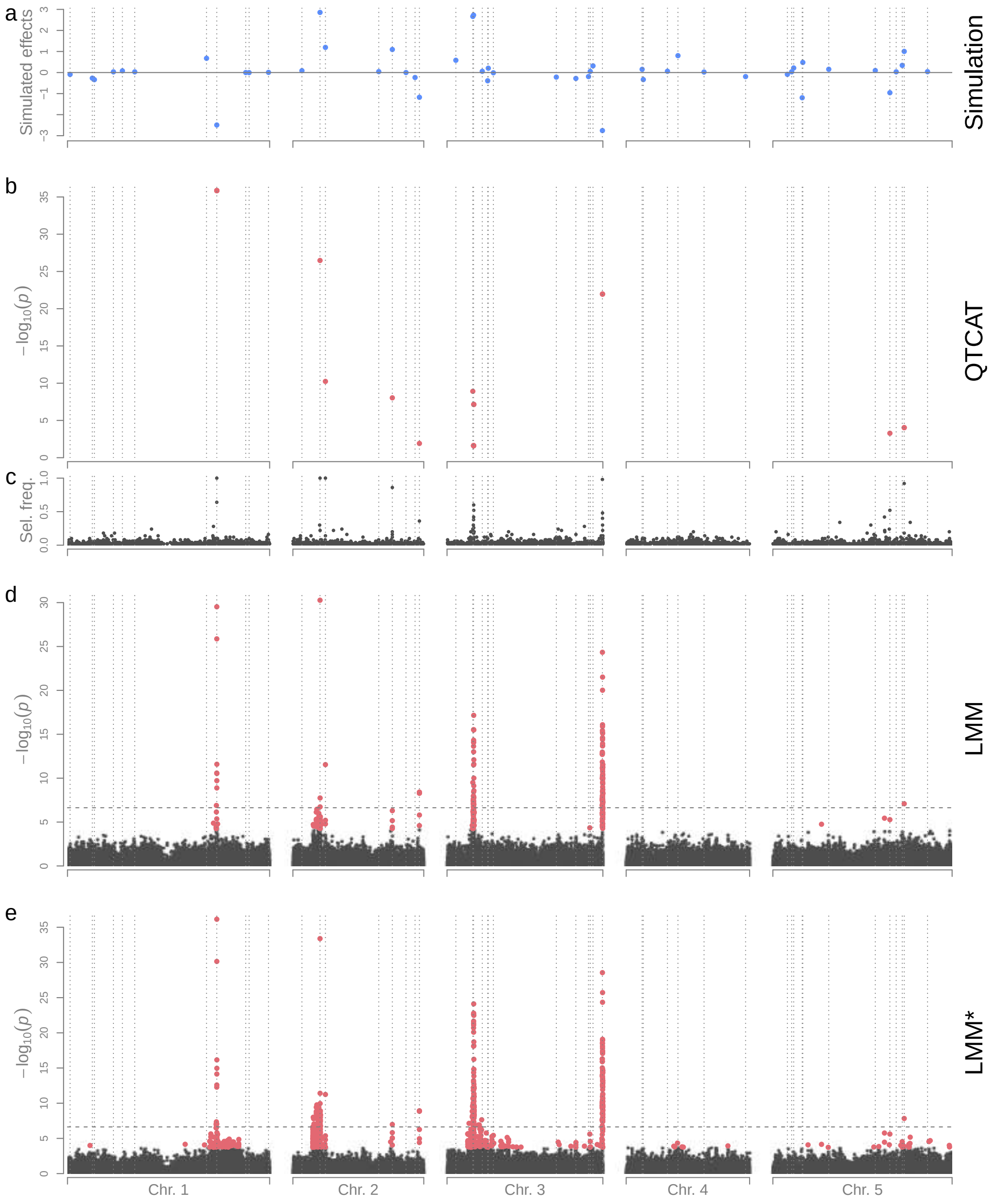

Supplementary Figure 27 Simulation of a GWA analysis based on a structured population with a heritability of 0.7 (run 27). (**a**) Simulation of 50 effects randomly drawn from a Gamma distribution and assigned to random markers. Markers with effect are highlighted with dashed lines. (**b**) Significant QTCs found by QTCAT. (**c**) LASSO selection frequency for each marker during the 50 iterations of QTCAT. (**d**) Manhattan plot of the LMM analysis. The horizontal dashed line depicts the significance threshold when controlling the multiple testing with FWER, whereas the red markers are significantly associated when controlling with FDR. (**e**) The Manhattan plot of the LMM* analysis. GRM was estimated without markers on the chromosome of the actual testing position. The results are shown as in (d).

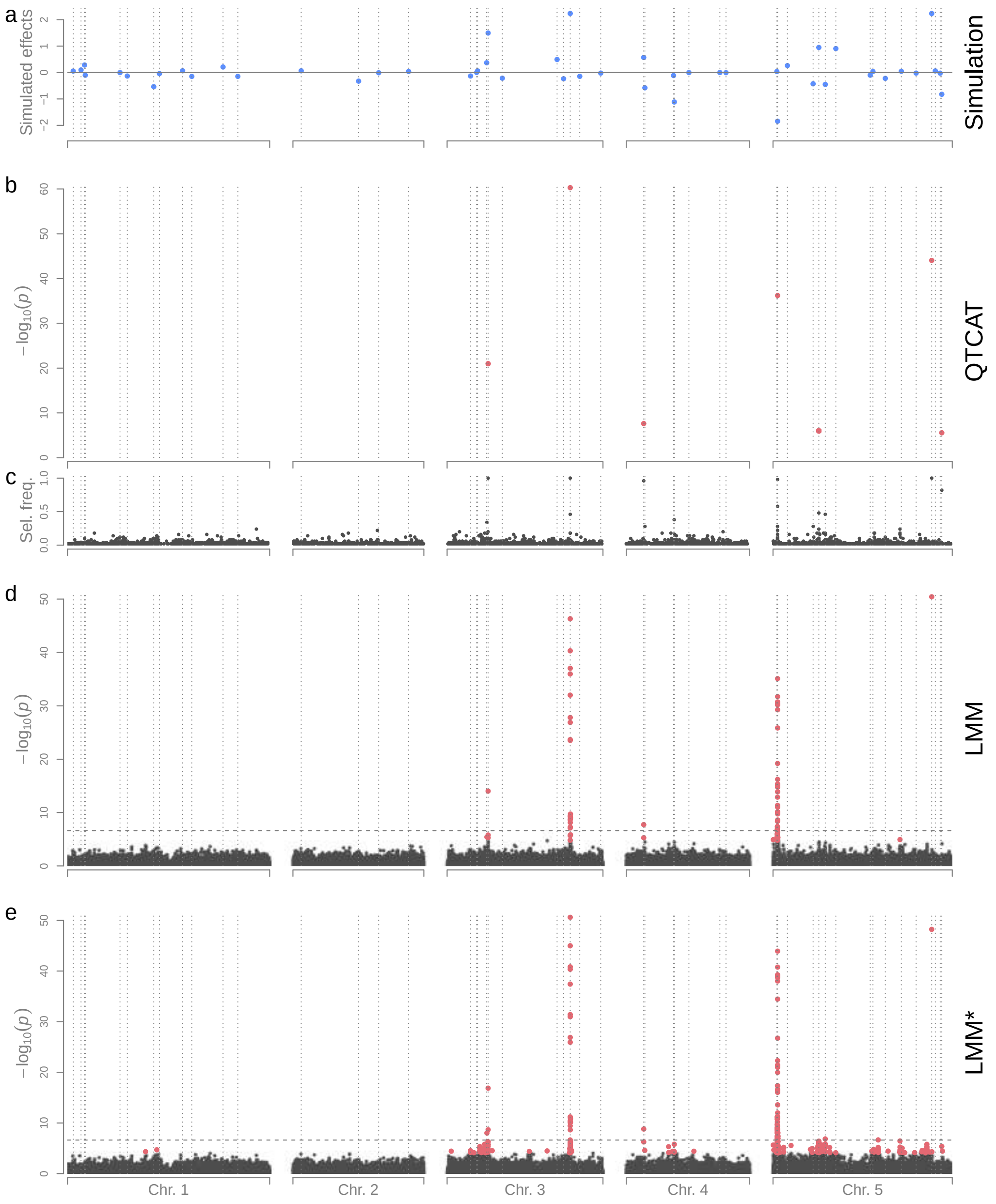

Supplementary Figure 28 Simulation of a GWA analysis based on a structured population with a heritability of 0.7 (run 28). (**a**) Simulation of 50 effects randomly drawn from a Gamma distribution and assigned to random markers. Markers with effect are highlighted with dashed lines. (**b**) Significant QTCs found by QTCAT. (**c**) LASSO selection frequency for each marker during the 50 iterations of QTCAT. (**d**) Manhattan plot of the LMM analysis. The horizontal dashed line depicts the significance threshold when controlling the multiple testing with FWER, whereas the red markers are significantly associated when controlling with FDR. (**e**) The Manhattan plot of the LMM* analysis. GRM was estimated without markers on the chromosome of the actual testing position. The results are shown as in (d).

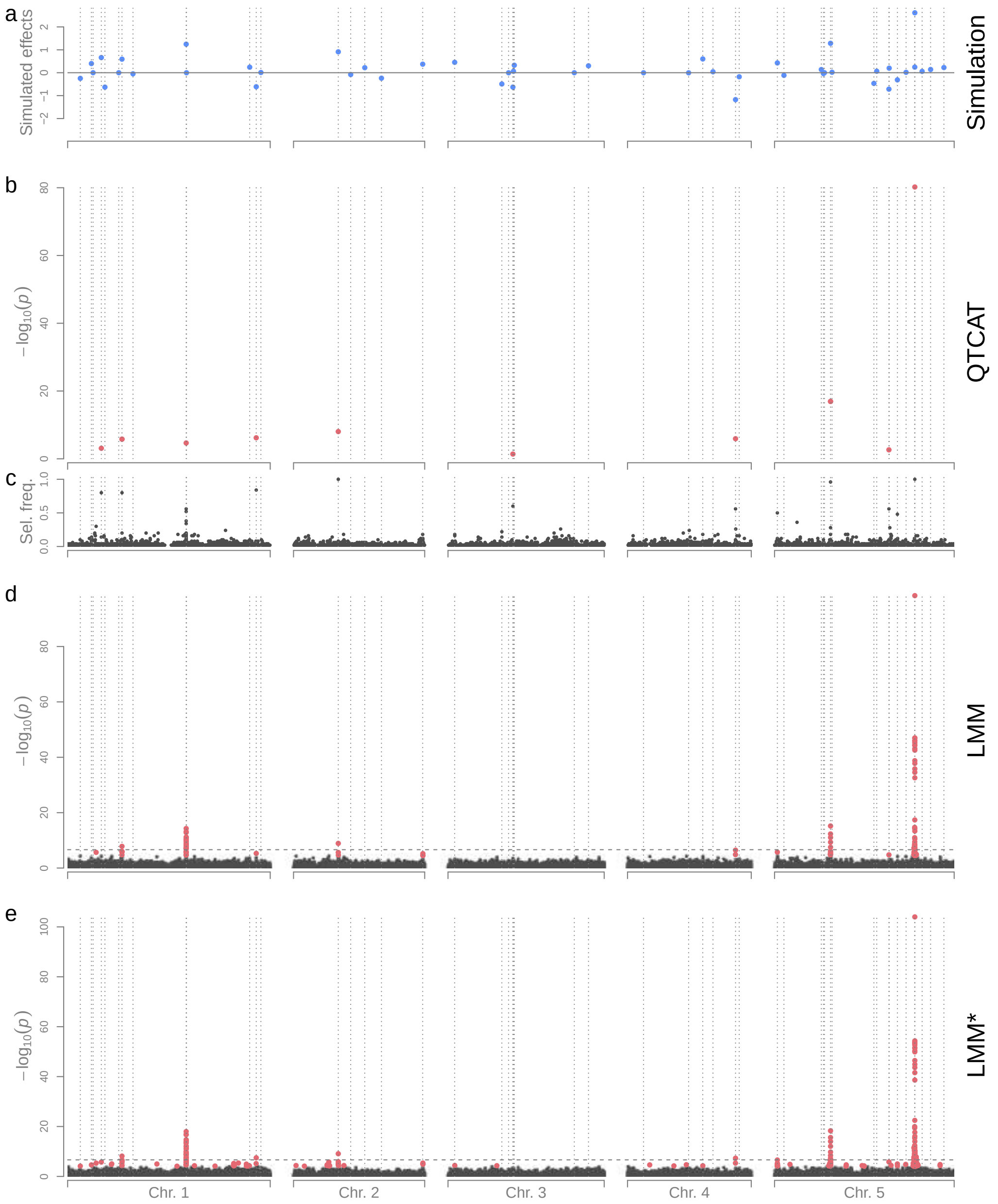

Supplementary Figure 29 Simulation of a GWA analysis based on a structured population with a heritability of 0.7 (run 29). (**a**) Simulation of 50 effects randomly drawn from a Gamma distribution and assigned to random markers. Markers with effect are highlighted with dashed lines. (**b**) Significant QTCs found by QTCAT. (**c**) LASSO selection frequency for each marker during the 50 iterations of QTCAT. (**d**) Manhattan plot of the LMM analysis. The horizontal dashed line depicts the significance threshold when controlling the multiple testing with FWER, whereas the red markers are significantly associated when controlling with FDR. (**e**) The Manhattan plot of the LMM* analysis. GRM was estimated without markers on the chromosome of the actual testing position. The results are shown as in (d).

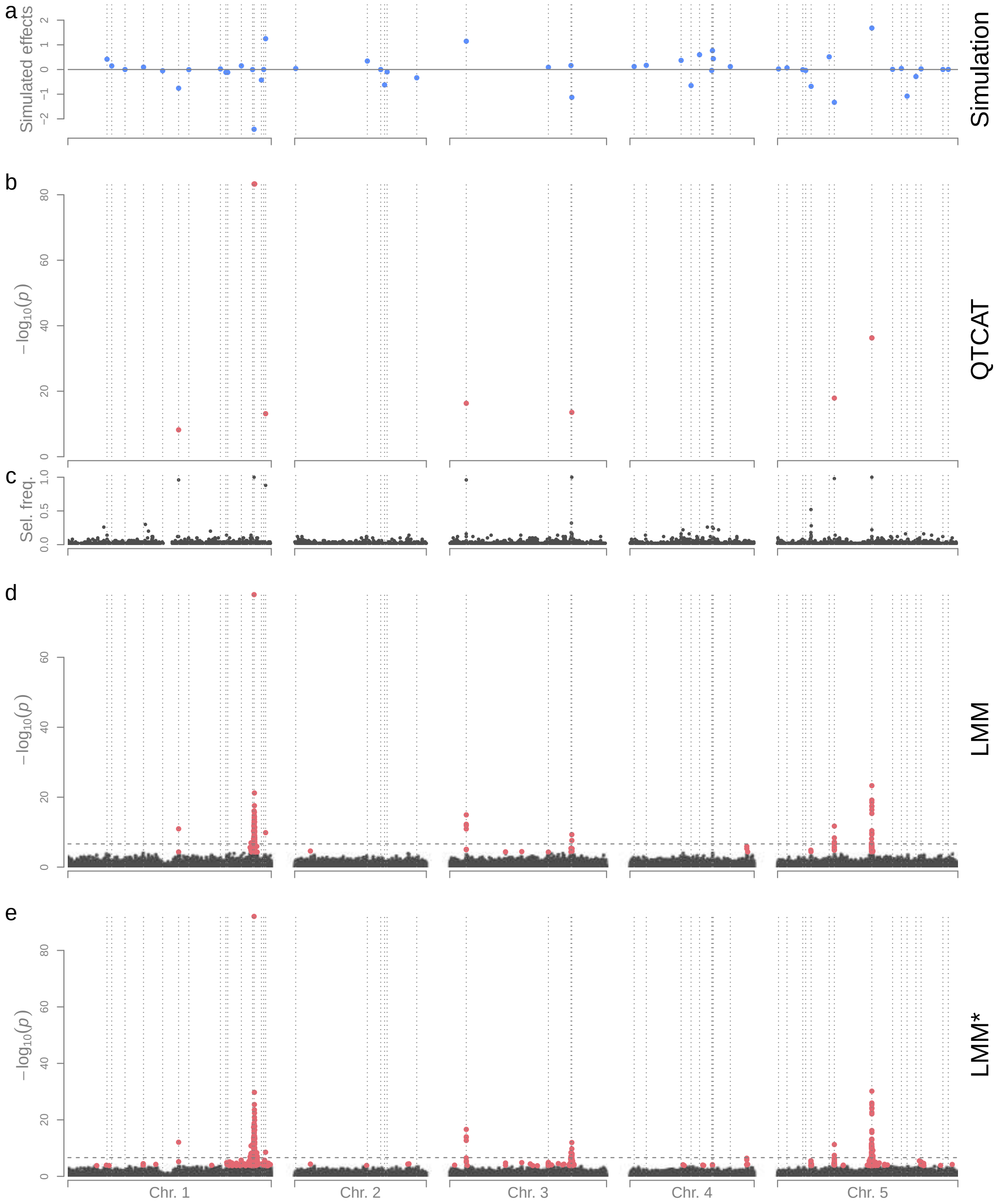

Supplementary Figure 30 Simulation of a GWA analysis based on a structured population with a heritability of 0.7 (run 30). (**a**) Simulation of 50 effects randomly drawn from a Gamma distribution and assigned to random markers. Markers with effect are highlighted with dashed lines. (**b**) Significant QTCs found by QTCAT. (**c**) LASSO selection frequency for each marker during the 50 iterations of QTCAT. (**d**) Manhattan plot of the LMM analysis. The horizontal dashed line depicts the significance threshold when controlling the multiple testing with FWER, whereas the red markers are significantly associated when controlling with FDR. (**e**) The Manhattan plot of the LMM* analysis. GRM was estimated without markers on the chromosome of the actual testing position. The results are shown as in (d).

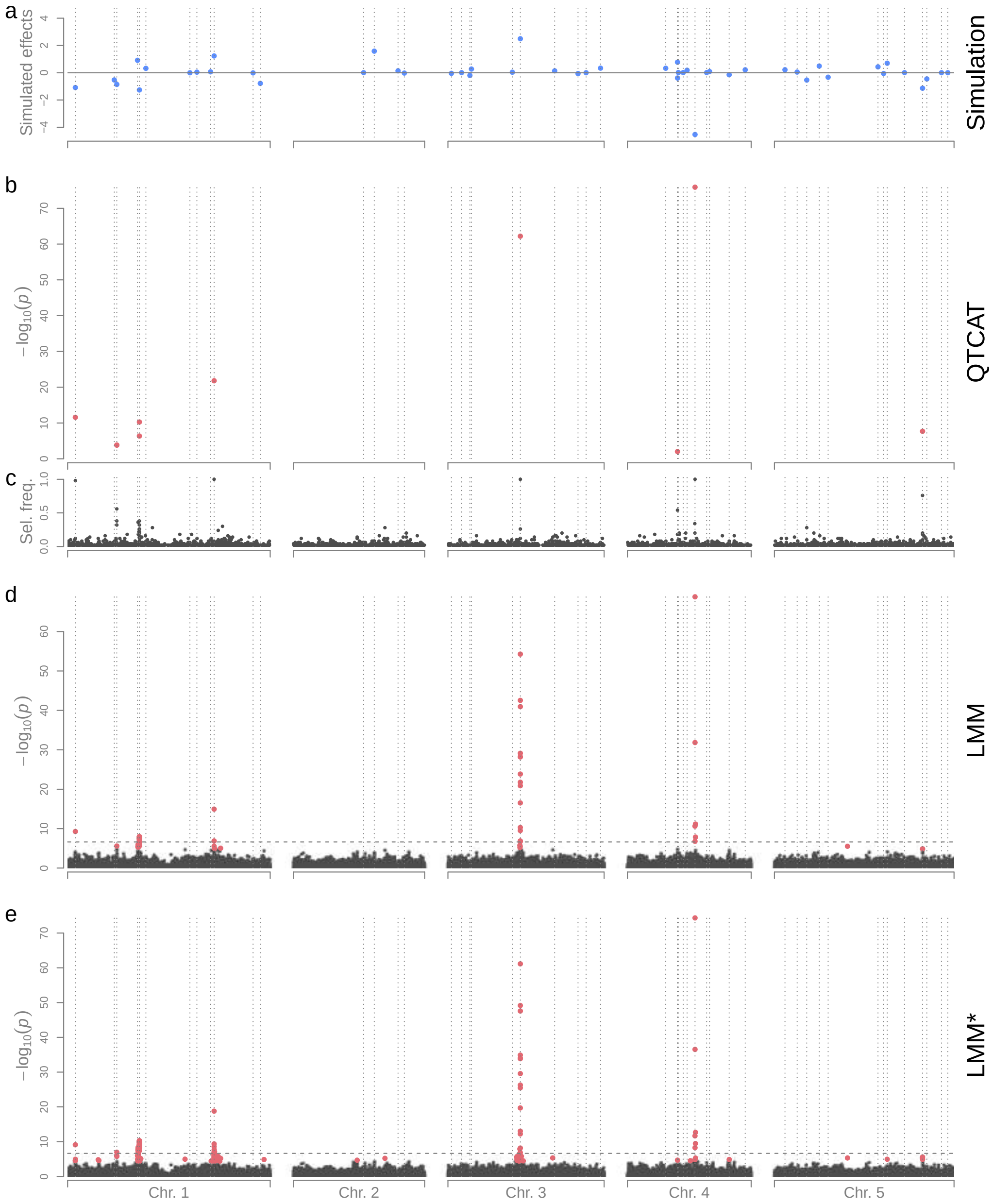

Supplementary Figure 31 Simulation of a GWA analysis based on a structured population with a heritability of 0.7 (run 31). (**a**) Simulation of 50 effects randomly drawn from a Gamma distribution and assigned to random markers. Markers with effect are highlighted with dashed lines. (**b**) Significant QTCs found by QTCAT. (**c**) LASSO selection frequency for each marker during the 50 iterations of QTCAT. (**d**) Manhattan plot of the LMM analysis. The horizontal dashed line depicts the significance threshold when controlling the multiple testing with FWER, whereas the red markers are significantly associated when controlling with FDR. (**e**) The Manhattan plot of the LMM* analysis. GRM was estimated without markers on the chromosome of the actual testing position. The results are shown as in (d).

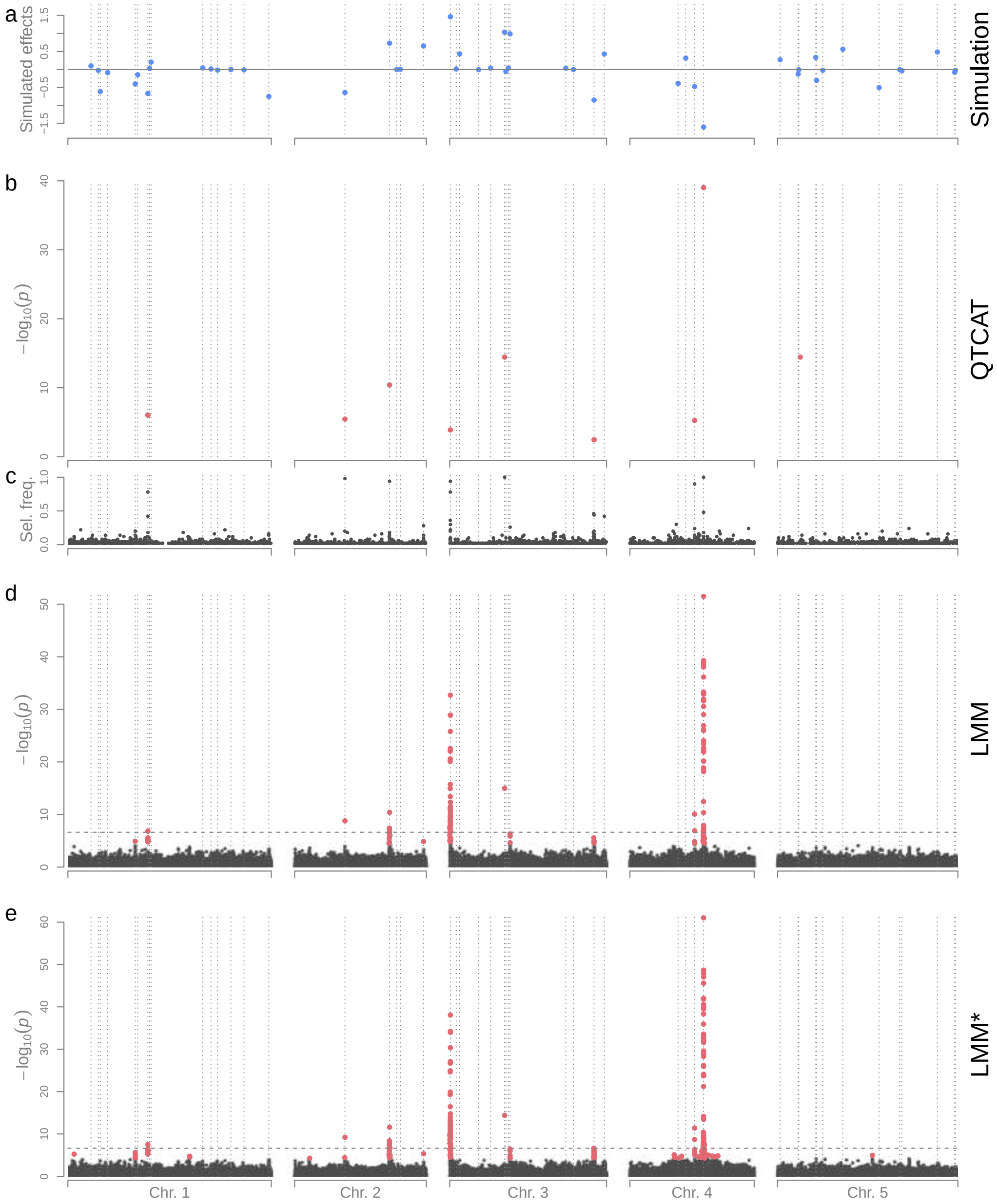

Supplementary Figure 32 Simulation of a GWA analysis based on a structured population with a heritability of 0.7 (run 32). (**a**) Simulation of 50 effects randomly drawn from a Gamma distribution and assigned to random markers. Markers with effect are highlighted with dashed lines. (**b**) Significant QTCs found by QTCAT. (**c**) LASSO selection frequency for each marker during the 50 iterations of QTCAT. (**d**) Manhattan plot of the LMM analysis. The horizontal dashed line depicts the significance threshold when controlling the multiple testing with FWER, whereas the red markers are significantly associated when controlling with FDR. (**e**) The Manhattan plot of the LMM* analysis. GRM was estimated without markers on the chromosome of the actual testing position. The results are shown as in (d).

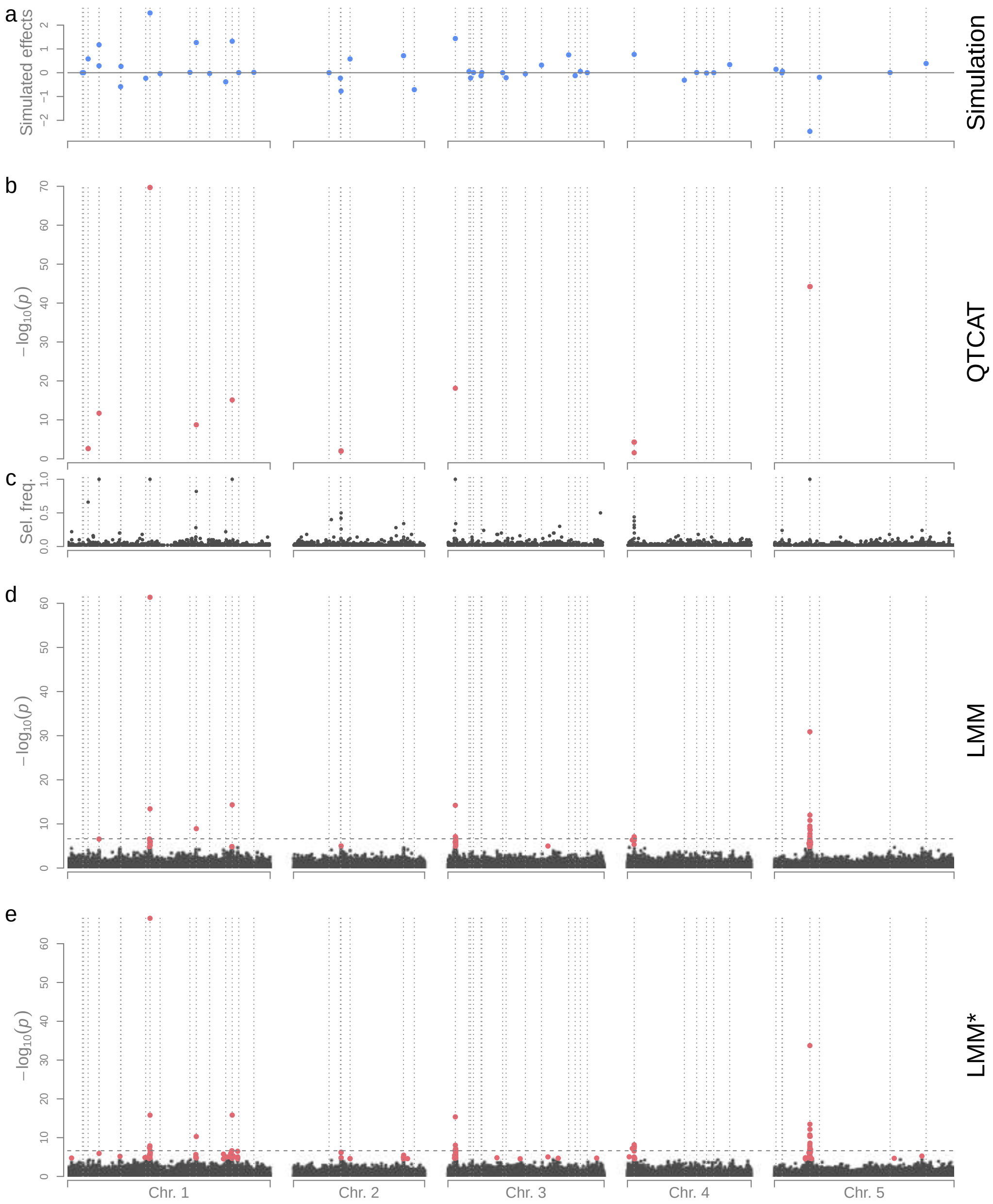

Supplementary Figure 33 Simulation of a GWA analysis based on a structured population with a heritability of 0.7 (run 33). (**a**) Simulation of 50 effects randomly drawn from a Gamma distribution and assigned to random markers. Markers with effect are highlighted with dashed lines. (**b**) Significant QTCs found by QTCAT. (**c**) LASSO selection frequency for each marker during the 50 iterations of QTCAT. (**d**) Manhattan plot of the LMM analysis. The horizontal dashed line depicts the significance threshold when controlling the multiple testing with FWER, whereas the red markers are significantly associated when controlling with FDR. (**e**) The Manhattan plot of the LMM* analysis. GRM was estimated without markers on the chromosome of the actual testing position. The results are shown as in (d).

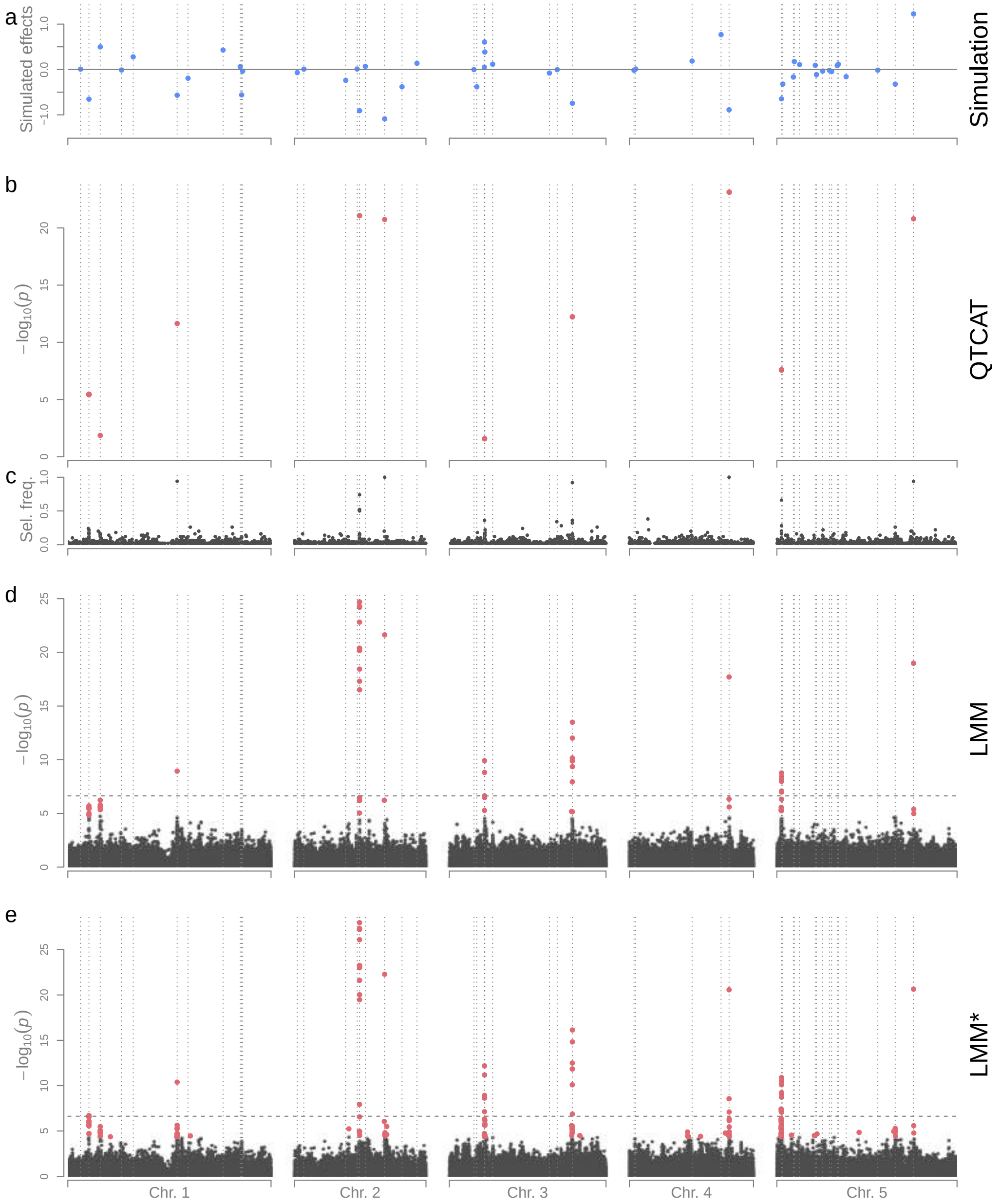

Supplementary Figure 34 Simulation of a GWA analysis based on a structured population with a heritability of 0.7 (run 34). (**a**) Simulation of 50 effects randomly drawn from a Gamma distribution and assigned to random markers. Markers with effect are highlighted with dashed lines. (**b**) Significant QTCs found by QTCAT. (**c**) LASSO selection frequency for each marker during the 50 iterations of QTCAT. (**d**) Manhattan plot of the LMM analysis. The horizontal dashed line depicts the significance threshold when controlling the multiple testing with FWER, whereas the red markers are significantly associated when controlling with FDR. (**e**) The Manhattan plot of the LMM* analysis. GRM was estimated without markers on the chromosome of the actual testing position. The results are shown as in (d).

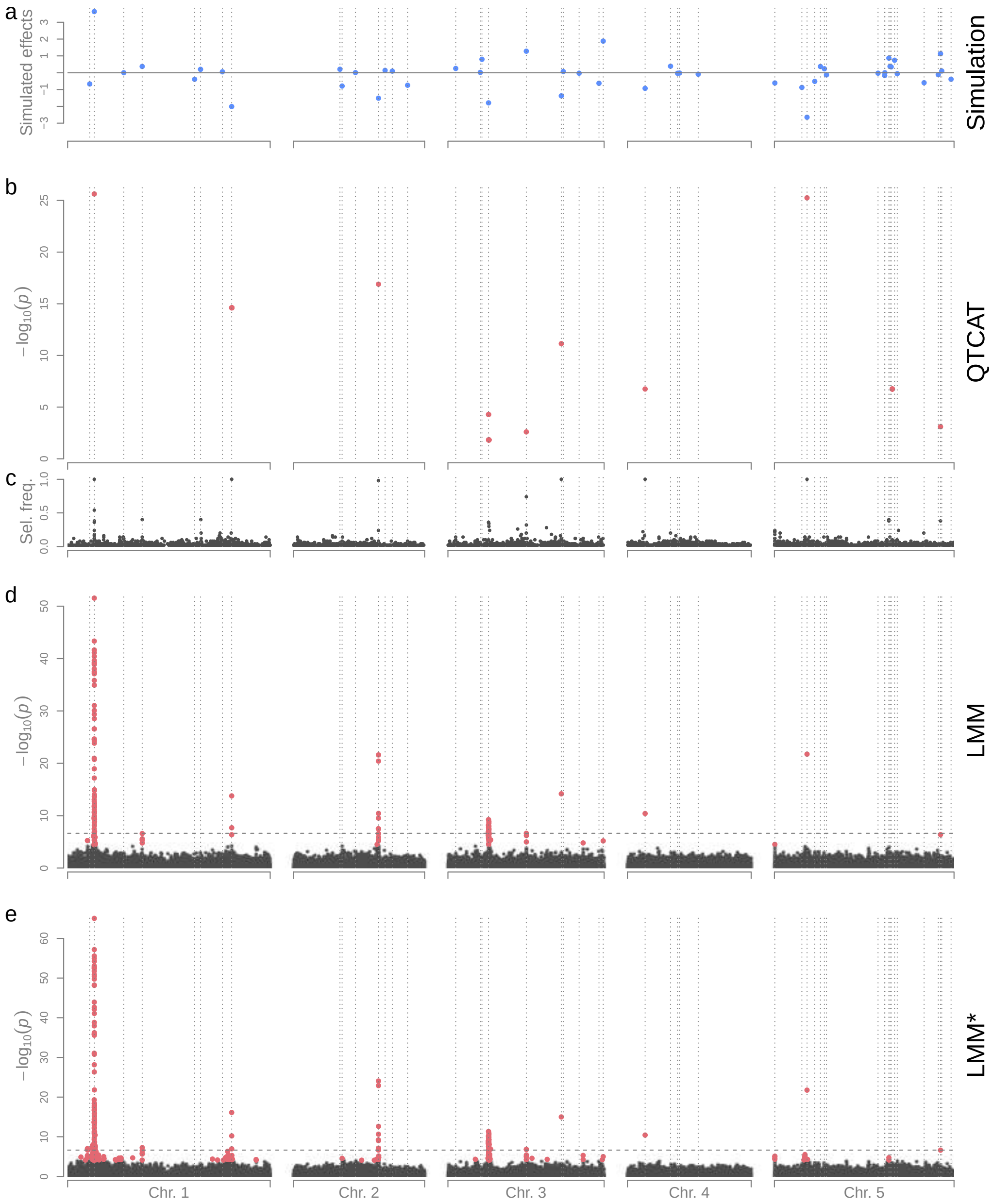

Supplementary Figure 35 Simulation of a GWA analysis based on a structured population with a heritability of 0.7 (run 35). (**a**) Simulation of 50 effects randomly drawn from a Gamma distribution and assigned to random markers. Markers with effect are highlighted with dashed lines. (**b**) Significant QTCs found by QTCAT. (**c**) LASSO selection frequency for each marker during the 50 iterations of QTCAT. (**d**) Manhattan plot of the LMM analysis. The horizontal dashed line depicts the significance threshold when controlling the multiple testing with FWER, whereas the red markers are significantly associated when controlling with FDR. (**e**) The Manhattan plot of the LMM* analysis. GRM was estimated without markers on the chromosome of the actual testing position. The results are shown as in (d).

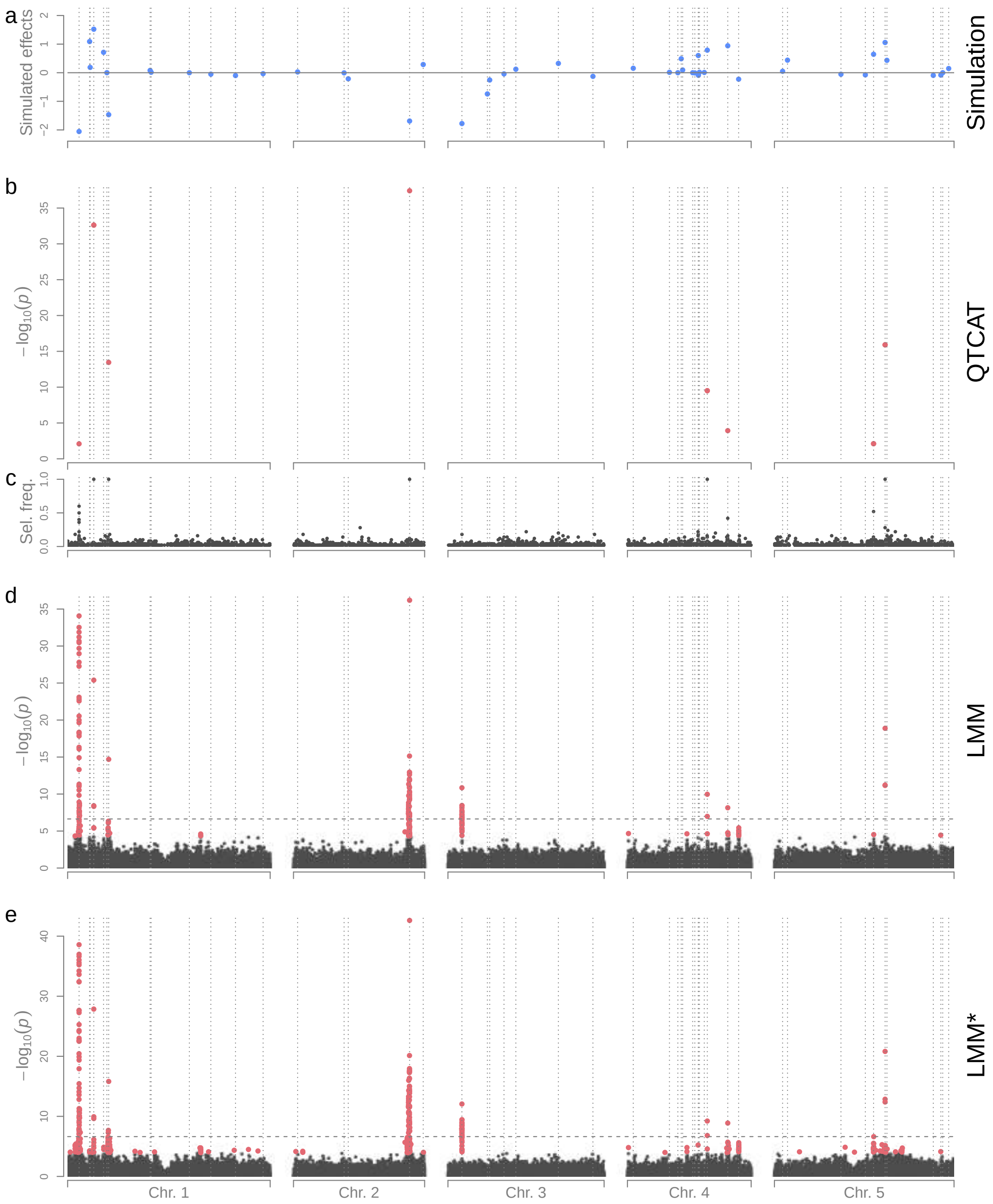

Supplementary Figure 36 Simulation of a GWA analysis based on a structured population with a heritability of 0.7 (run 36). (**a**) Simulation of 50 effects randomly drawn from a Gamma distribution and assigned to random markers. Markers with effect are highlighted with dashed lines. (**b**) Significant QTCs found by QTCAT. (**c**) LASSO selection frequency for each marker during the 50 iterations of QTCAT. (**d**) Manhattan plot of the LMM analysis. The horizontal dashed line depicts the significance threshold when controlling the multiple testing with FWER, whereas the red markers are significantly associated when controlling with FDR. (**e**) The Manhattan plot of the LMM* analysis. GRM was estimated without markers on the chromosome of the actual testing position. The results are shown as in (d).

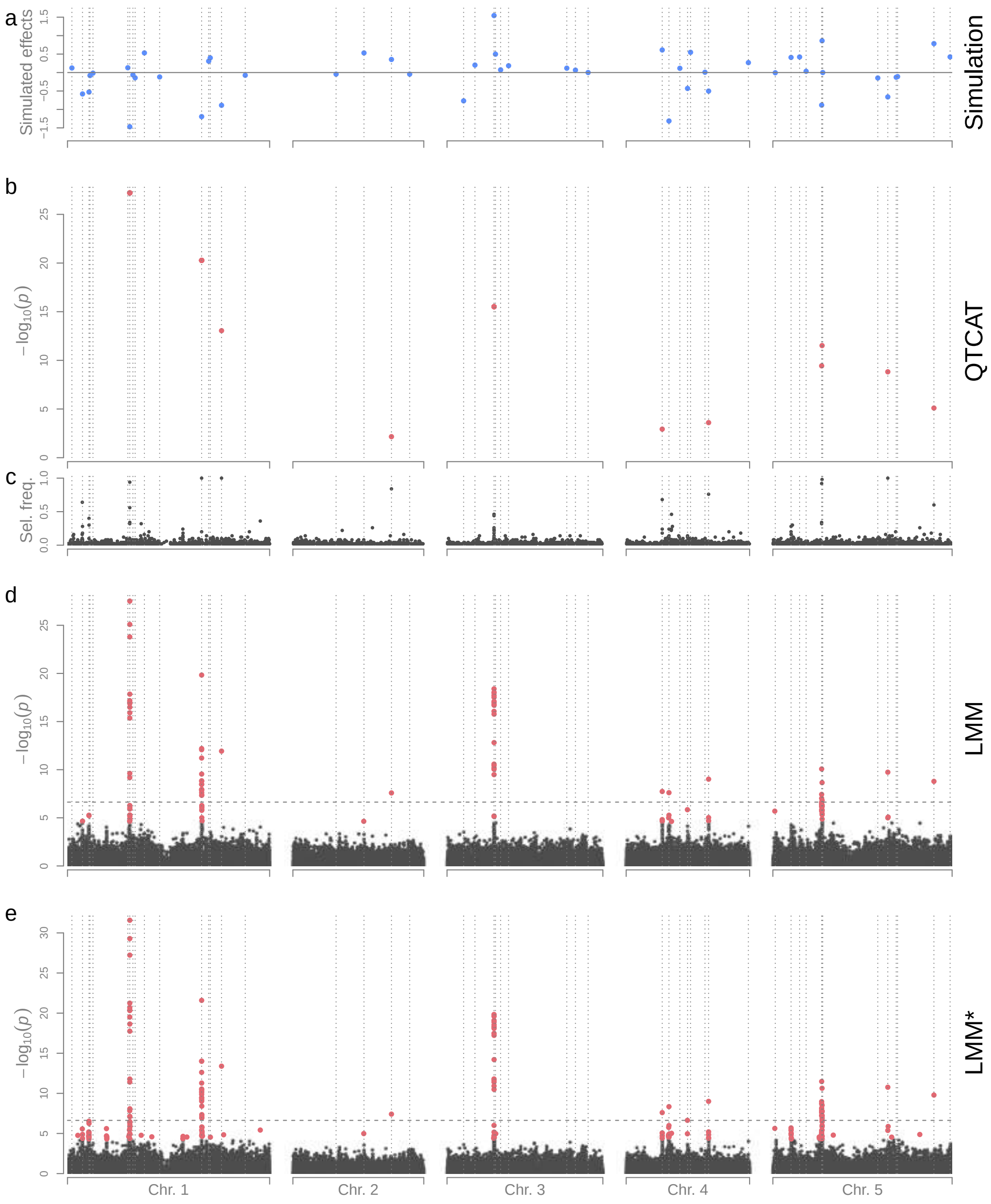

Supplementary Figure 37 Simulation of a GWA analysis based on a structured population with a heritability of 0.7 (run 37). (**a**) Simulation of 50 effects randomly drawn from a Gamma distribution and assigned to random markers. Markers with effect are highlighted with dashed lines. (**b**) Significant QTCs found by QTCAT. (**c**) LASSO selection frequency for each marker during the 50 iterations of QTCAT. (**d**) Manhattan plot of the LMM analysis. The horizontal dashed line depicts the significance threshold when controlling the multiple testing with FWER, whereas the red markers are significantly associated when controlling with FDR. (**e**) The Manhattan plot of the LMM* analysis. GRM was estimated without markers on the chromosome of the actual testing position. The results are shown as in (d).

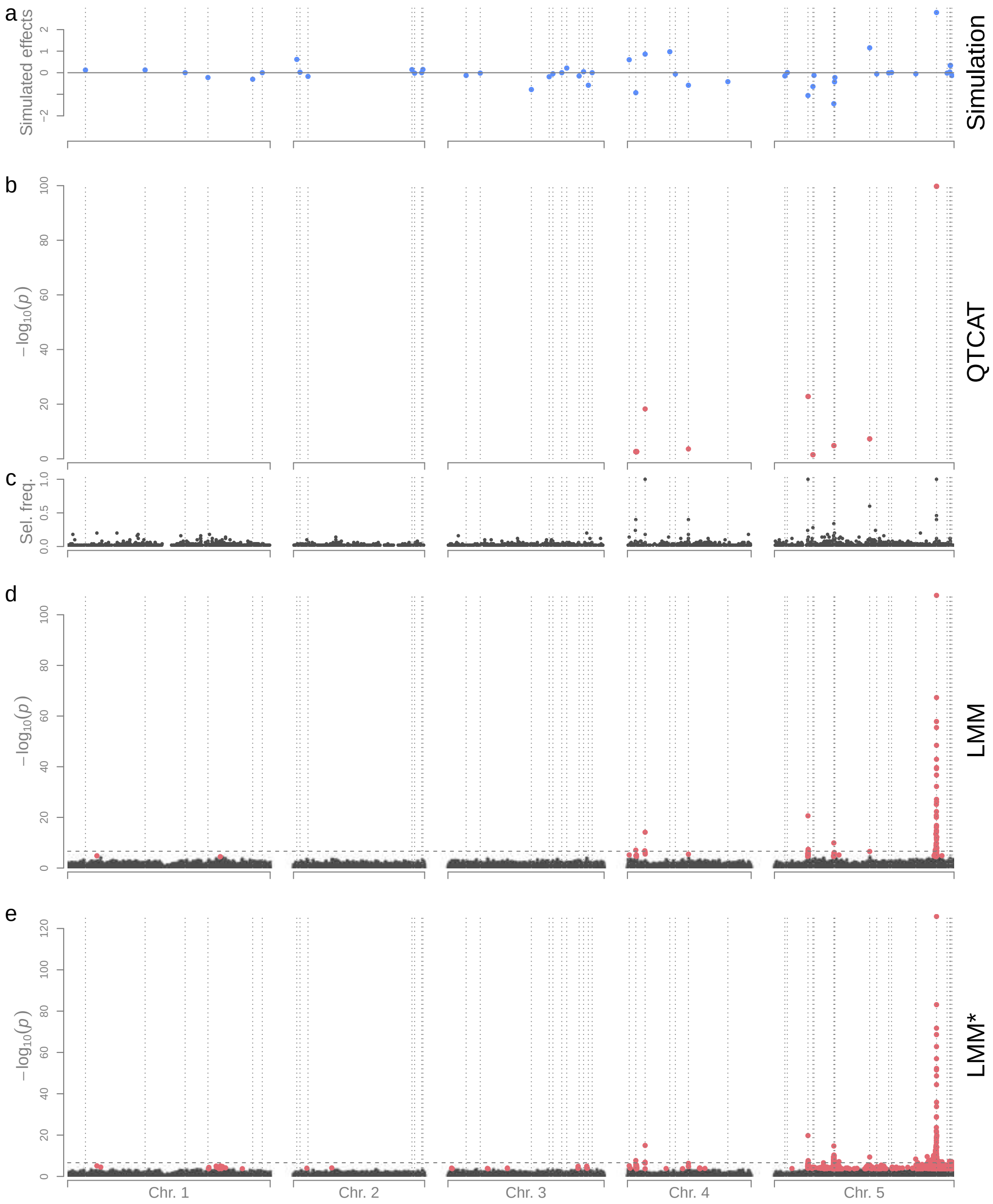

Supplementary Figure 38 Simulation of a GWA analysis based on a structured population with a heritability of 0.7 (run 38). (**a**) Simulation of 50 effects randomly drawn from a Gamma distribution and assigned to random markers. Markers with effect are highlighted with dashed lines. (**b**) Significant QTCs found by QTCAT. (**c**) LASSO selection frequency for each marker during the 50 iterations of QTCAT. (**d**) Manhattan plot of the LMM analysis. The horizontal dashed line depicts the significance threshold when controlling the multiple testing with FWER, whereas the red markers are significantly associated when controlling with FDR. (**e**) The Manhattan plot of the LMM* analysis. GRM was estimated without markers on the chromosome of the actual testing position. The results are shown as in (d).

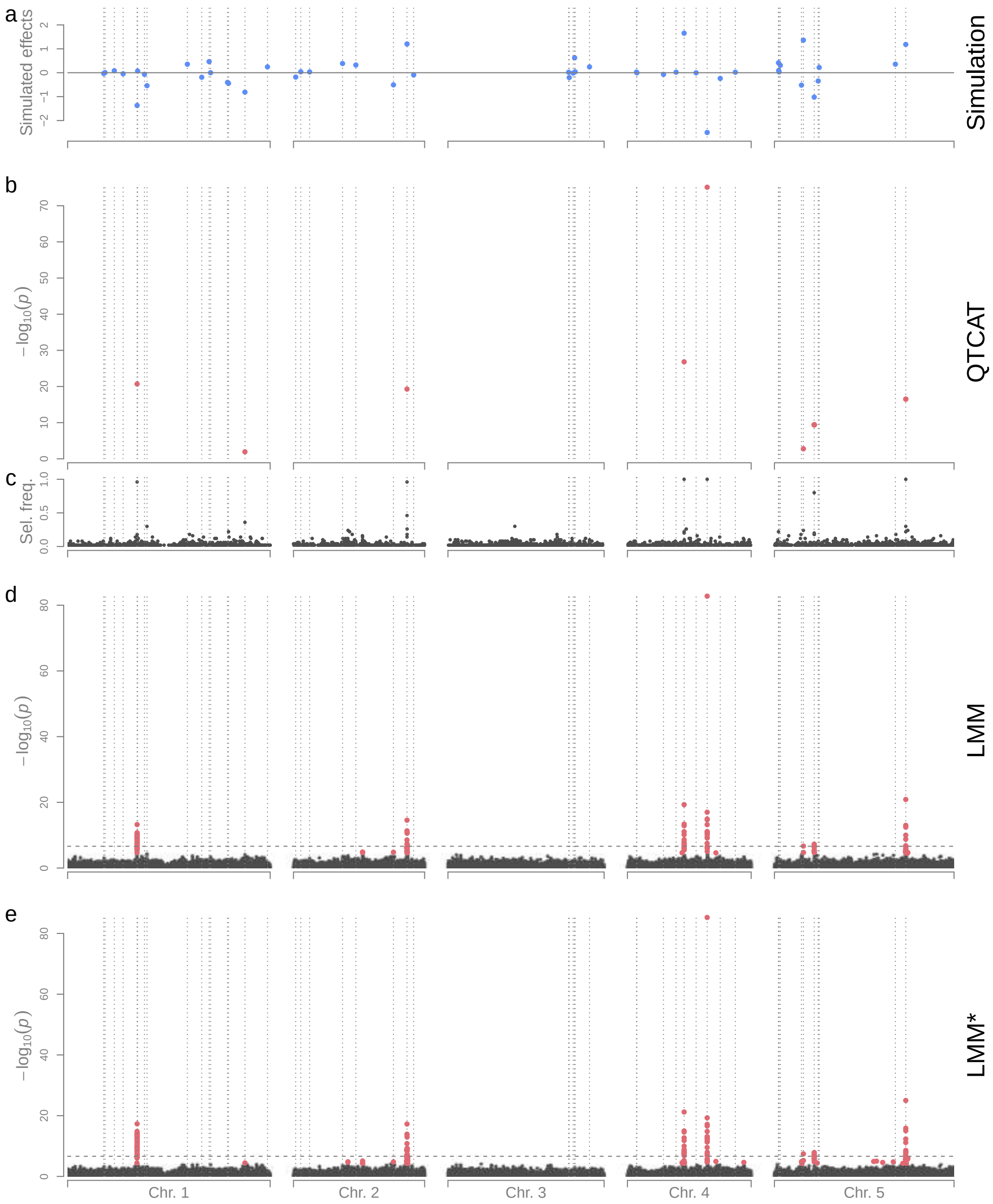

Supplementary Figure 39 Simulation of a GWA analysis based on a structured population with a heritability of 0.7 (run 39). (**a**) Simulation of 50 effects randomly drawn from a Gamma distribution and assigned to random markers. Markers with effect are highlighted with dashed lines. (**b**) Significant QTCs found by QTCAT. (**c**) LASSO selection frequency for each marker during the 50 iterations of QTCAT. (**d**) Manhattan plot of the LMM analysis. The horizontal dashed line depicts the significance threshold when controlling the multiple testing with FWER, whereas the red markers are significantly associated when controlling with FDR. (**e**) The Manhattan plot of the LMM* analysis. GRM was estimated without markers on the chromosome of the actual testing position. The results are shown as in (d).

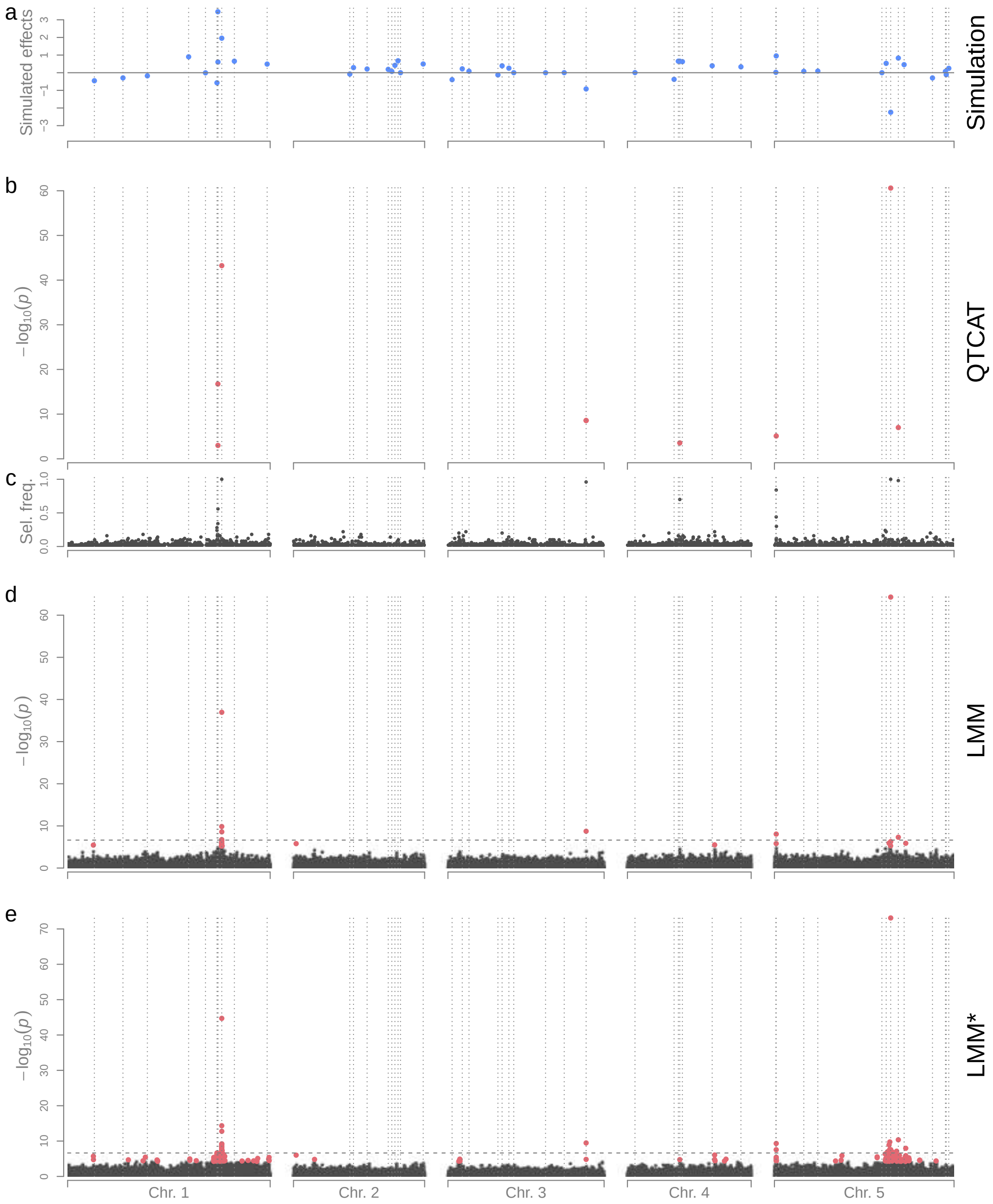

Supplementary Figure 40 Simulation of a GWA analysis based on a structured population with a heritability of 0.7 (run 40). (**a**) Simulation of 50 effects randomly drawn from a Gamma distribution and assigned to random markers. Markers with effect are highlighted with dashed lines. (**b**) Significant QTCs found by QTCAT. (**c**) LASSO selection frequency for each marker during the 50 iterations of QTCAT. (**d**) Manhattan plot of the LMM analysis. The horizontal dashed line depicts the significance threshold when controlling the multiple testing with FWER, whereas the red markers are significantly associated when controlling with FDR. (**e**) The Manhattan plot of the LMM* analysis. GRM was estimated without markers on the chromosome of the actual testing position. The results are shown as in (d).

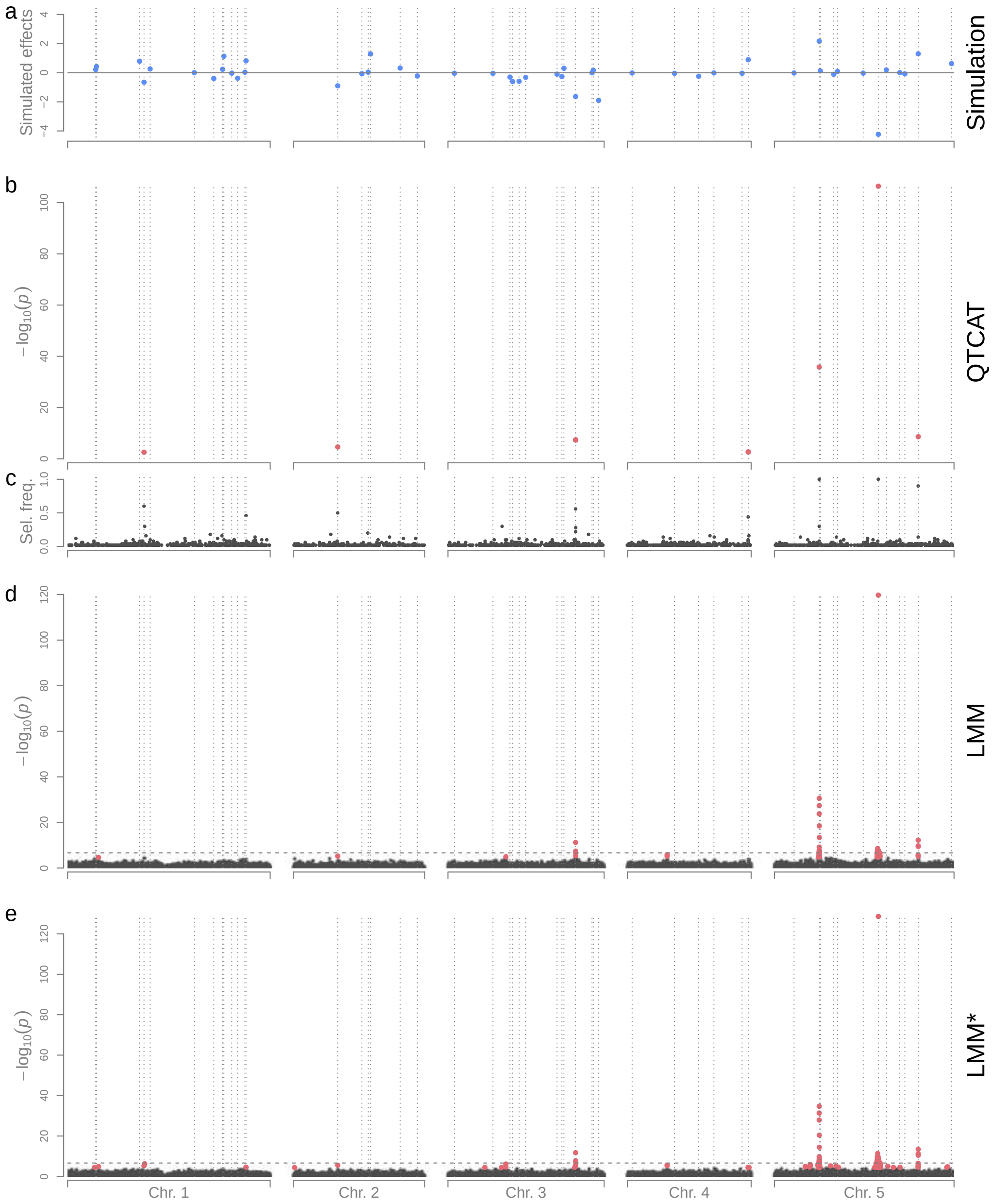

Supplementary Figure 41 Simulation of a GWA analysis based on a structured population with a heritability of 0.7 (run 41). (**a**) Simulation of 50 effects randomly drawn from a Gamma distribution and assigned to random markers. Markers with effect are highlighted with dashed lines. (**b**) Significant QTCs found by QTCAT. (**c**) LASSO selection frequency for each marker during the 50 iterations of QTCAT. (**d**) Manhattan plot of the LMM analysis. The horizontal dashed line depicts the significance threshold when controlling the multiple testing with FWER, whereas the red markers are significantly associated when controlling with FDR. (**e**) The Manhattan plot of the LMM* analysis. GRM was estimated without markers on the chromosome of the actual testing position. The results are shown as in (d).

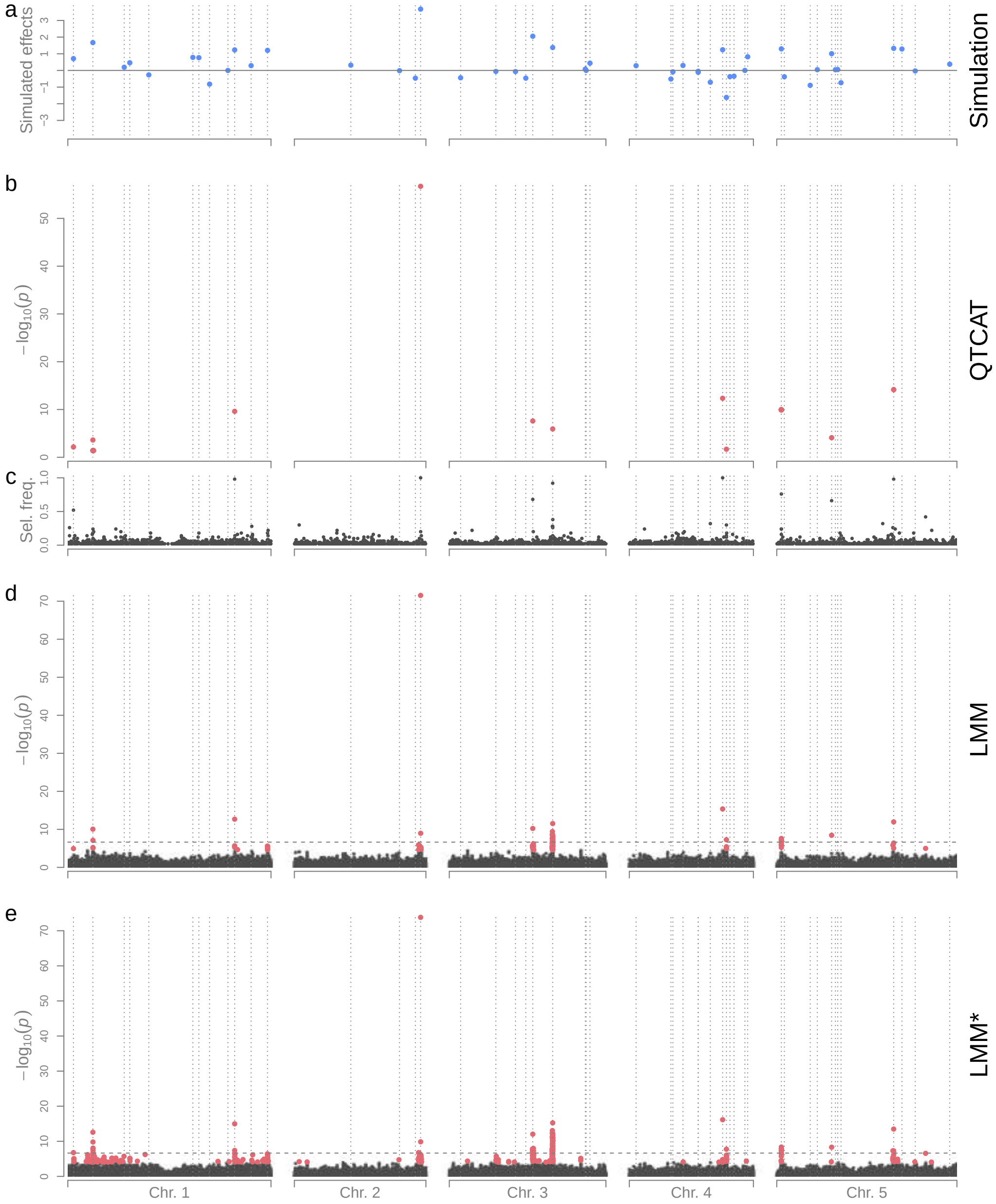

Supplementary Figure 42 Simulation of a GWA analysis based on a structured population with a heritability of 0.7 (run 42). (**a**) Simulation of 50 effects randomly drawn from a Gamma distribution and assigned to random markers. Markers with effect are highlighted with dashed lines. (**b**) Significant QTCs found by QTCAT. (**c**) LASSO selection frequency for each marker during the 50 iterations of QTCAT. (**d**) Manhattan plot of the LMM analysis. The horizontal dashed line depicts the significance threshold when controlling the multiple testing with FWER, whereas the red markers are significantly associated when controlling with FDR. (**e**) The Manhattan plot of the LMM* analysis. GRM was estimated without markers on the chromosome of the actual testing position. The results are shown as in (d).

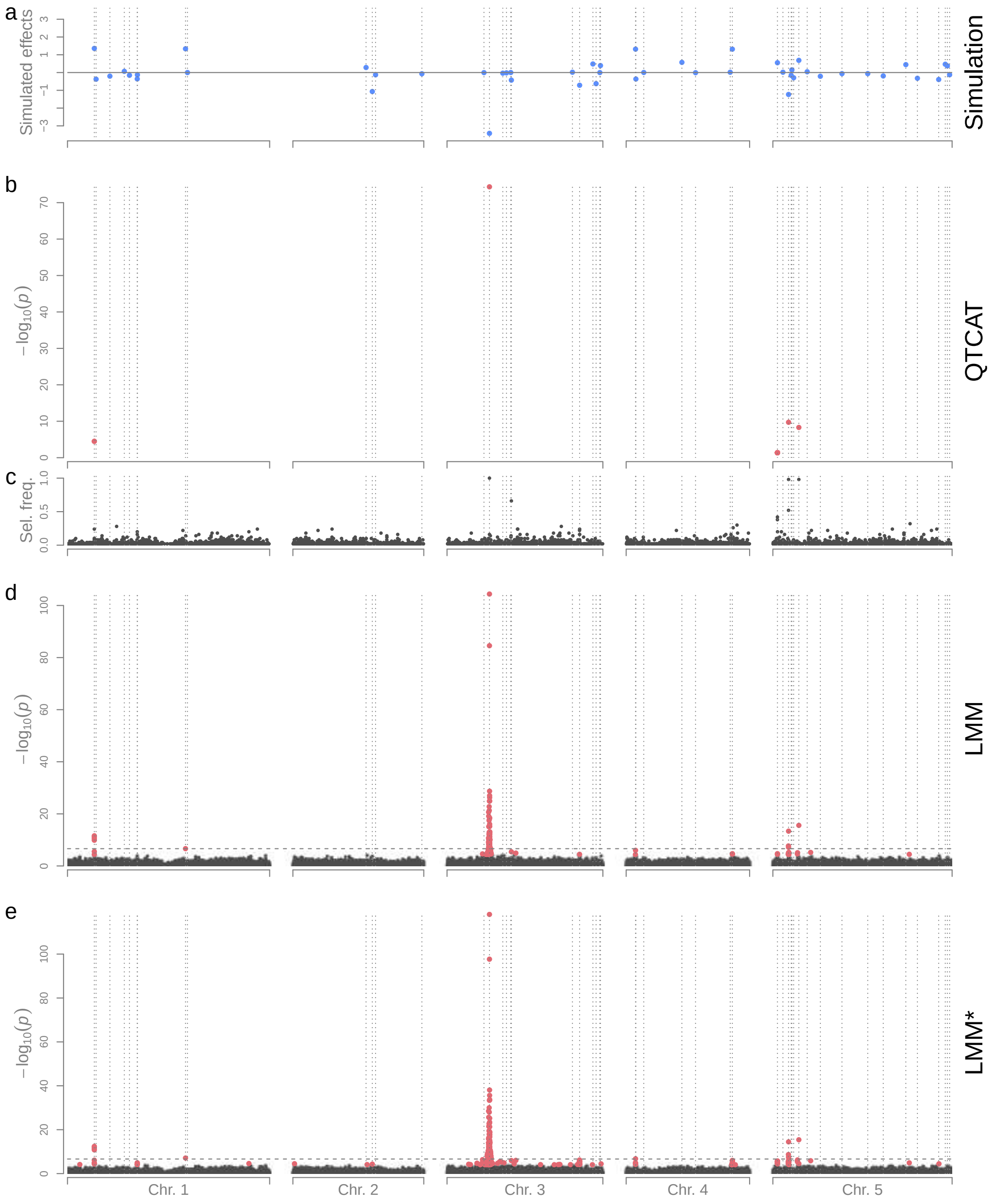

Supplementary Figure 43 Simulation of a GWA analysis based on a structured population with a heritability of 0.7 (run 43). (**a**) Simulation of 50 effects randomly drawn from a Gamma distribution and assigned to random markers. Markers with effect are highlighted with dashed lines. (**b**) Significant QTCs found by QTCAT. (**c**) LASSO selection frequency for each marker during the 50 iterations of QTCAT. (**d**) Manhattan plot of the LMM analysis. The horizontal dashed line depicts the significance threshold when controlling the multiple testing with FWER, whereas the red markers are significantly associated when controlling with FDR. (**e**) The Manhattan plot of the LMM* analysis. GRM was estimated without markers on the chromosome of the actual testing position. The results are shown as in (d).

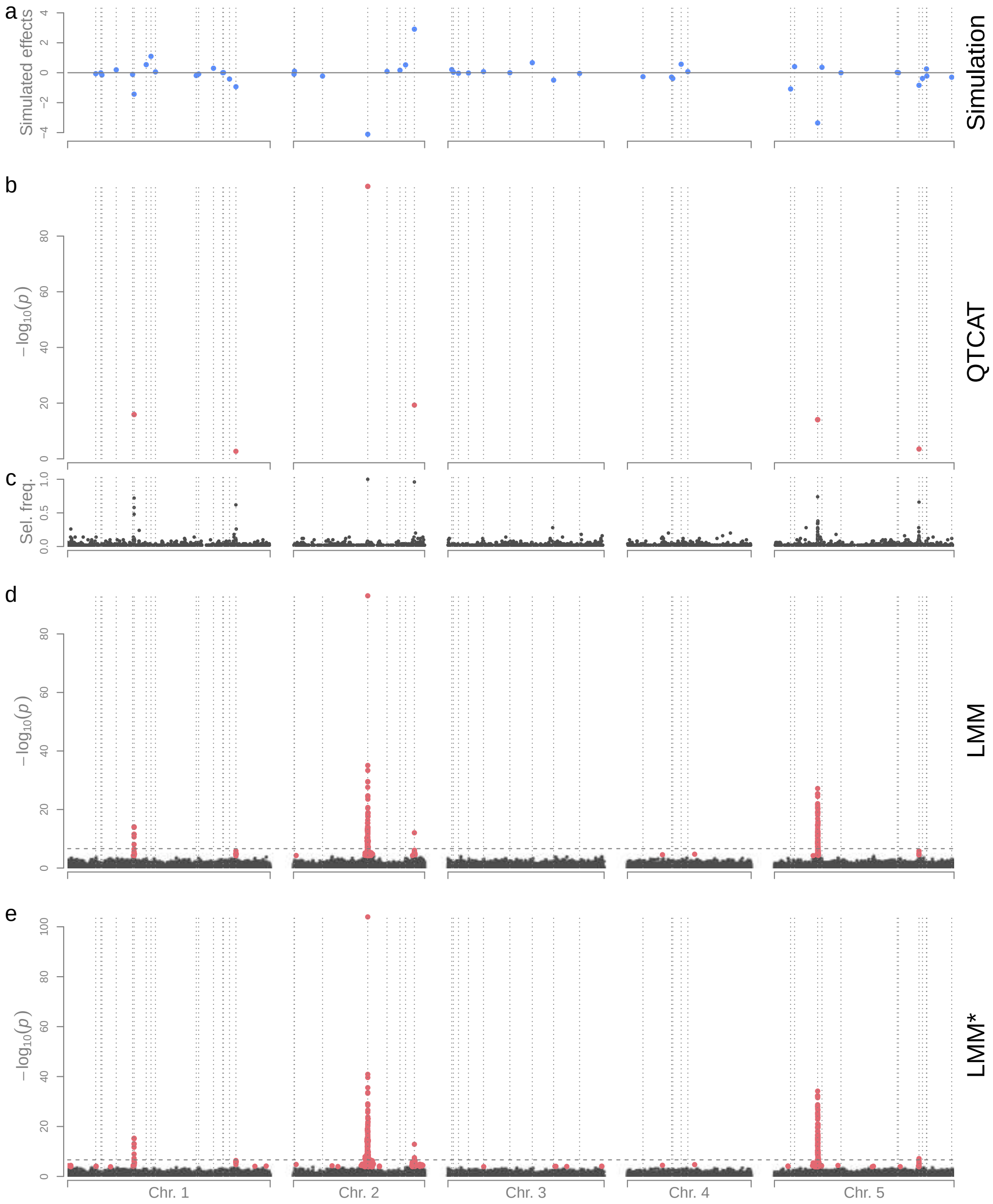

Supplementary Figure 44 Simulation of a GWA analysis based on a structured population with a heritability of 0.7 (run 44). (**a**) Simulation of 50 effects randomly drawn from a Gamma distribution and assigned to random markers. Markers with effect are highlighted with dashed lines. (**b**) Significant QTCs found by QTCAT. (**c**) LASSO selection frequency for each marker during the 50 iterations of QTCAT. (**d**) Manhattan plot of the LMM analysis. The horizontal dashed line depicts the significance threshold when controlling the multiple testing with FWER, whereas the red markers are significantly associated when controlling with FDR. (**e**) The Manhattan plot of the LMM* analysis. GRM was estimated without markers on the chromosome of the actual testing position. The results are shown as in (d).

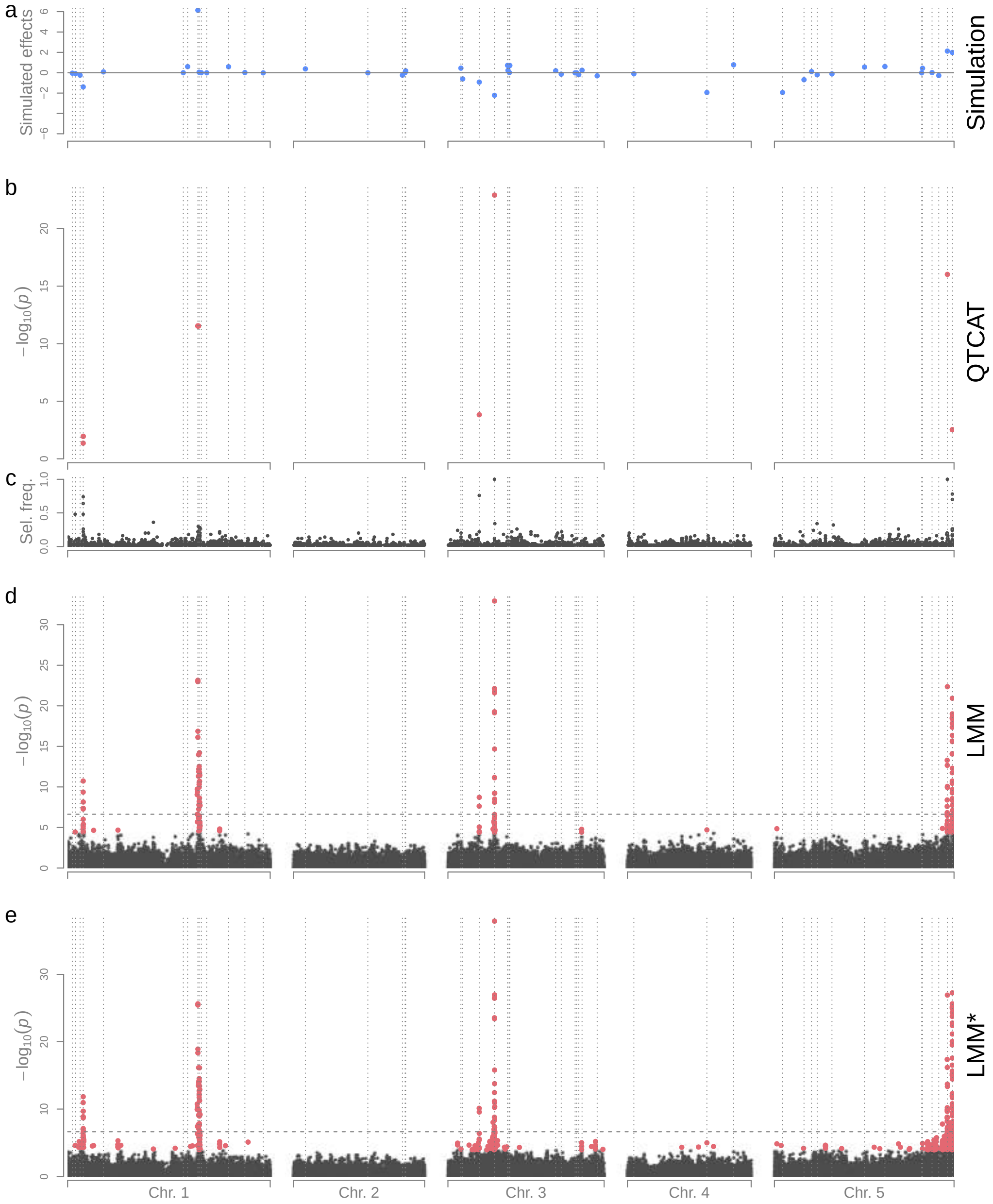

Supplementary Figure 45 Simulation of a GWA analysis based on a structured population with a heritability of 0.7 (run 45). (**a**) Simulation of 50 effects randomly drawn from a Gamma distribution and assigned to random markers. Markers with effect are highlighted with dashed lines. (**b**) Significant QTCs found by QTCAT. (**c**) LASSO selection frequency for each marker during the 50 iterations of QTCAT. (**d**) Manhattan plot of the LMM analysis. The horizontal dashed line depicts the significance threshold when controlling the multiple testing with FWER, whereas the red markers are significantly associated when controlling with FDR. (**e**) The Manhattan plot of the LMM* analysis. GRM was estimated without markers on the chromosome of the actual testing position. The results are shown as in (d).

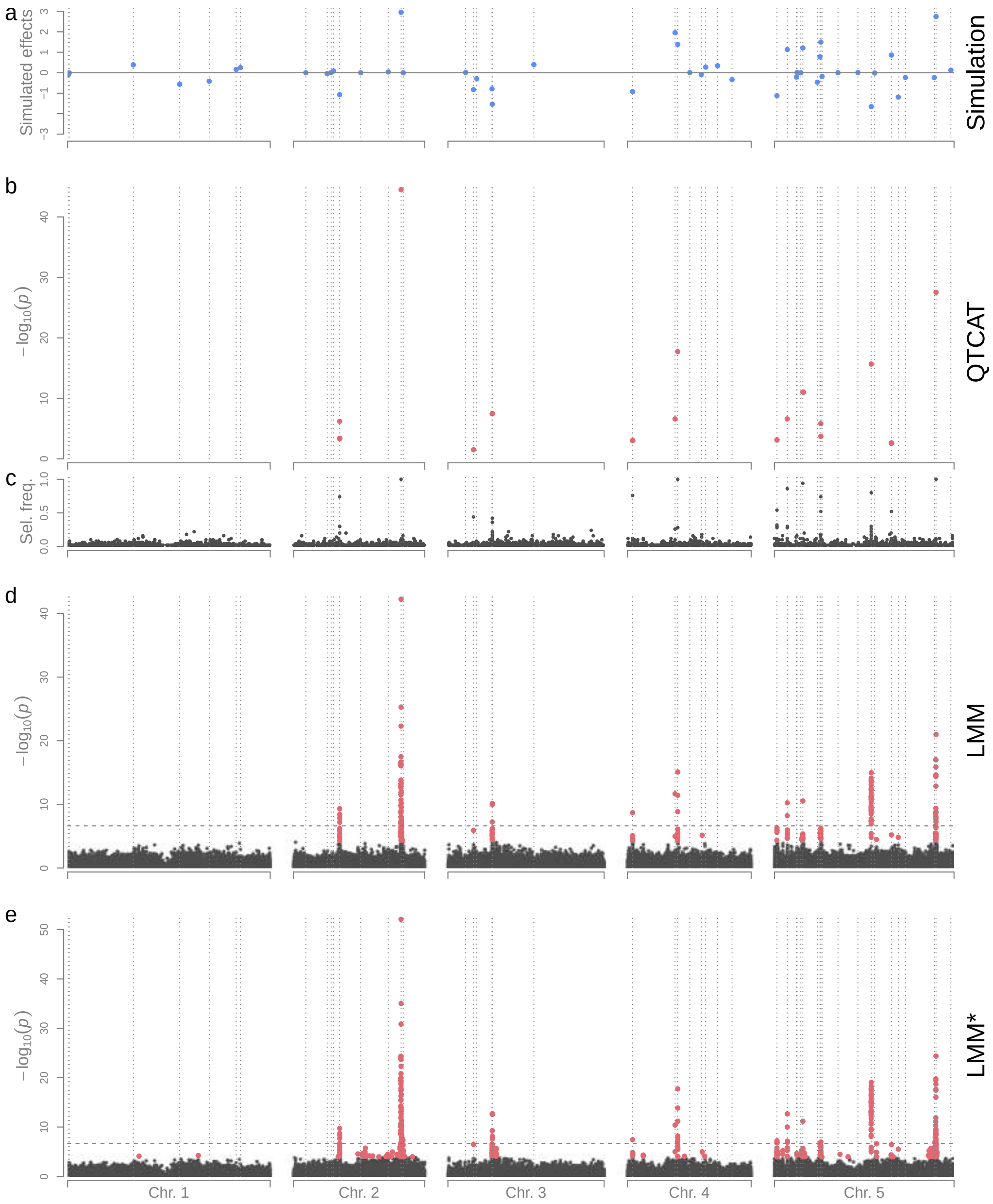

Supplementary Figure 46 Simulation of a GWA analysis based on a structured population with a heritability of 0.7 (run 46). (**a**) Simulation of 50 effects randomly drawn from a Gamma distribution and assigned to random markers. Markers with effect are highlighted with dashed lines. (**b**) Significant QTCs found by QTCAT. (**c**) LASSO selection frequency for each marker during the 50 iterations of QTCAT. (**d**) Manhattan plot of the LMM analysis. The horizontal dashed line depicts the significance threshold when controlling the multiple testing with FWER, whereas the red markers are significantly associated when controlling with FDR. (**e**) The Manhattan plot of the LMM* analysis. GRM was estimated without markers on the chromosome of the actual testing position. The results are shown as in (d).

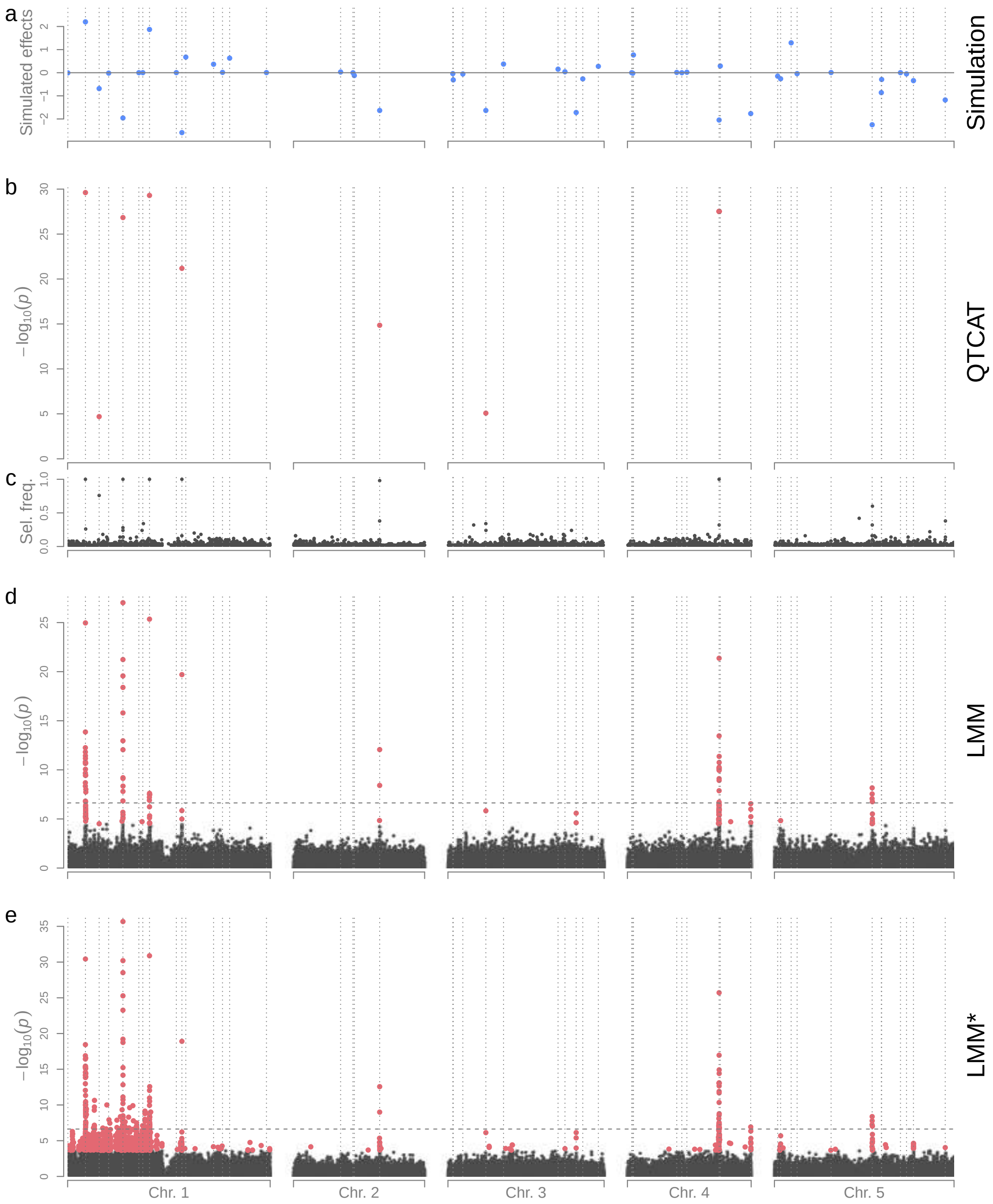

Supplementary Figure 47 Simulation of a GWA analysis based on a structured population with a heritability of 0.7 (run 47). (**a**) Simulation of 50 effects randomly drawn from a Gamma distribution and assigned to random markers. Markers with effect are highlighted with dashed lines. (**b**) Significant QTCs found by QTCAT. (**c**) LASSO selection frequency for each marker during the 50 iterations of QTCAT. (**d**) Manhattan plot of the LMM analysis. The horizontal dashed line depicts the significance threshold when controlling the multiple testing with FWER, whereas the red markers are significantly associated when controlling with FDR. (**e**) The Manhattan plot of the LMM* analysis. GRM was estimated without markers on the chromosome of the actual testing position. The results are shown as in (d).

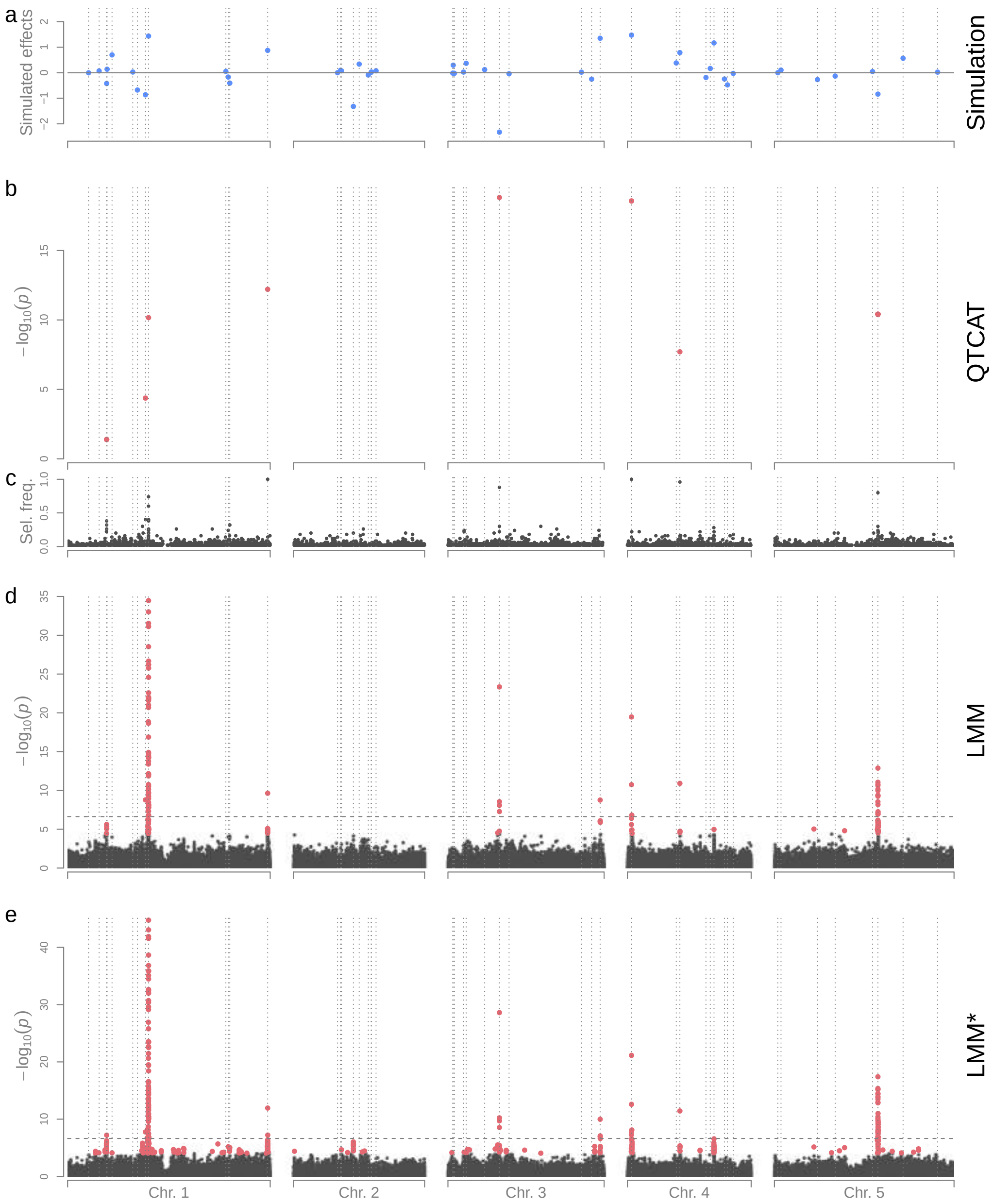

Supplementary Figure 48 Simulation of a GWA analysis based on a structured population with a heritability of 0.7 (run 48). (**a**) Simulation of 50 effects randomly drawn from a Gamma distribution and assigned to random markers. Markers with effect are highlighted with dashed lines. (**b**) Significant QTCs found by QTCAT. (**c**) LASSO selection frequency for each marker during the 50 iterations of QTCAT. (**d**) Manhattan plot of the LMM analysis. The horizontal dashed line depicts the significance threshold when controlling the multiple testing with FWER, whereas the red markers are significantly associated when controlling with FDR. (**e**) The Manhattan plot of the LMM* analysis. GRM was estimated without markers on the chromosome of the actual testing position. The results are shown as in (d).

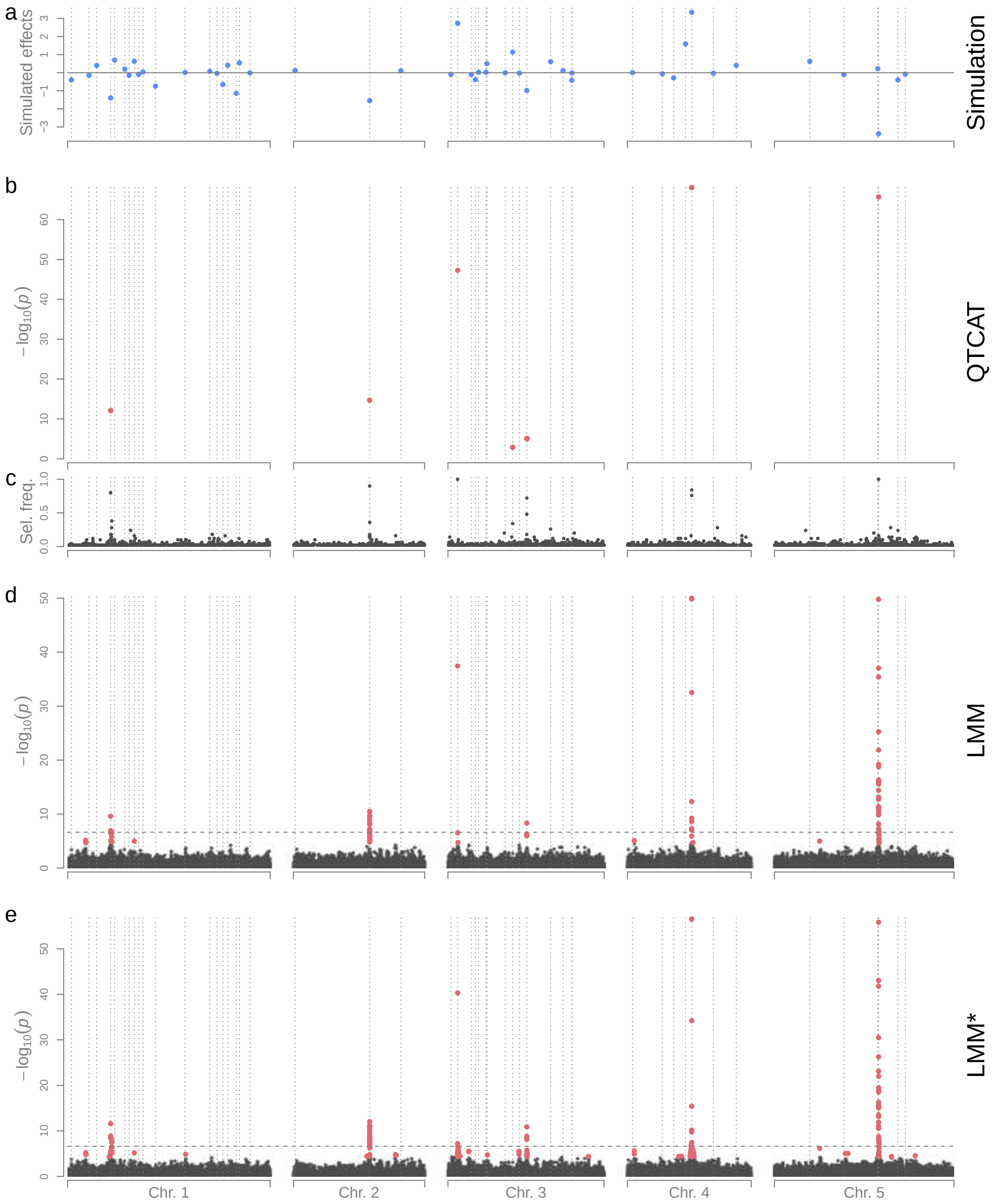

Supplementary Figure 49 Simulation of a GWA analysis based on a structured population with a heritability of 0.7 (run 49). (**a**) Simulation of 50 effects randomly drawn from a Gamma distribution and assigned to random markers. Markers with effect are highlighted with dashed lines. (**b**) Significant QTCs found by QTCAT. (**c**) LASSO selection frequency for each marker during the 50 iterations of QTCAT. (**d**) Manhattan plot of the LMM analysis. The horizontal dashed line depicts the significance threshold when controlling the multiple testing with FWER, whereas the red markers are significantly associated when controlling with FDR. (**e**) The Manhattan plot of the LMM* analysis. GRM was estimated without markers on the chromosome of the actual testing position. The results are shown as in (d).

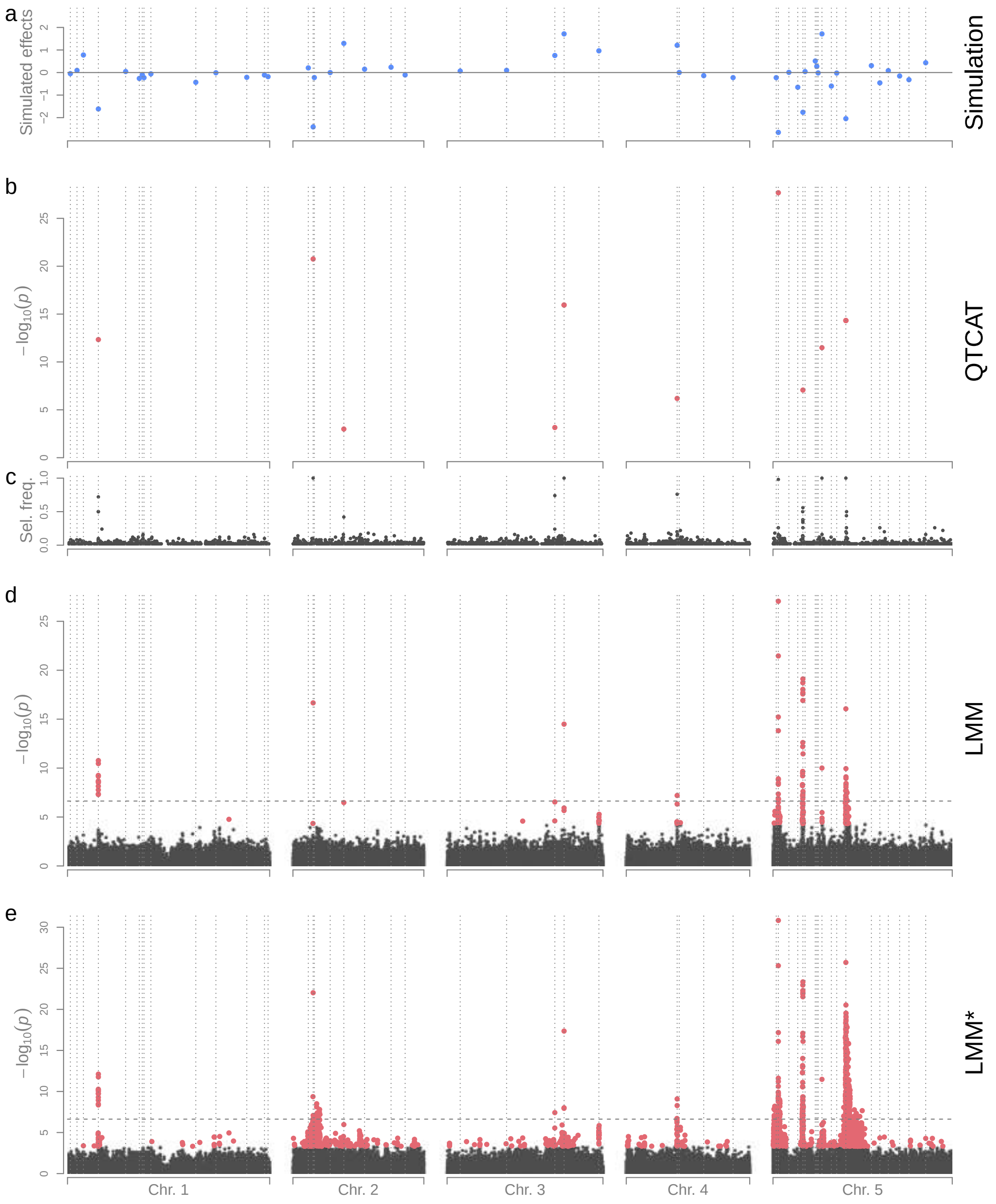

Supplementary Figure 50 Simulation of a GWA analysis based on a structured population with a heritability of 0.7 (run 50). (**a**) Simulation of 50 effects randomly drawn from a Gamma distribution and assigned to random markers. Markers with effect are highlighted with dashed lines. (**b**) Significant QTCs found by QTCAT. (**c**) LASSO selection frequency for each marker during the 50 iterations of QTCAT. (**d**) Manhattan plot of the LMM analysis. The horizontal dashed line depicts the significance threshold when controlling the multiple testing with FWER, whereas the red markers are significantly associated when controlling with FDR. (**e**) The Manhattan plot of the LMM* analysis. GRM was estimated without markers on the chromosome of the actual testing position. The results are shown as in (d).

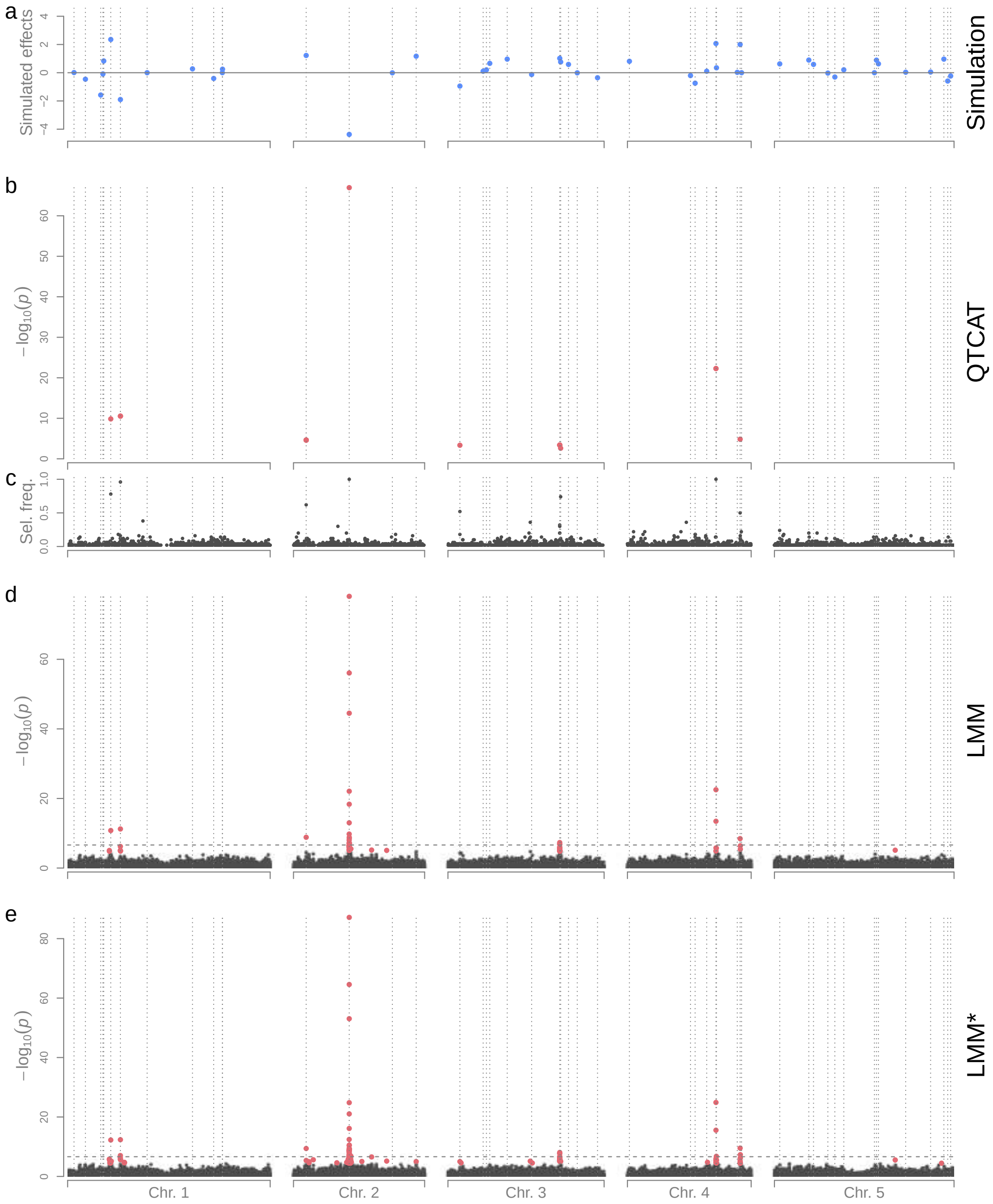

Supplementary Figure 51 Simulation of a GWA analysis based on a structured population with a heritability of 0.7 (run 51). (**a**) Simulation of 50 effects randomly drawn from a Gamma distribution and assigned to random markers. Markers with effect are highlighted with dashed lines. (**b**) Significant QTCs found by QTCAT. (**c**) LASSO selection frequency for each marker during the 50 iterations of QTCAT. (**d**) Manhattan plot of the LMM analysis. The horizontal dashed line depicts the significance threshold when controlling the multiple testing with FWER, whereas the red markers are significantly associated when controlling with FDR. (**e**) The Manhattan plot of the LMM* analysis. GRM was estimated without markers on the chromosome of the actual testing position. The results are shown as in (d).

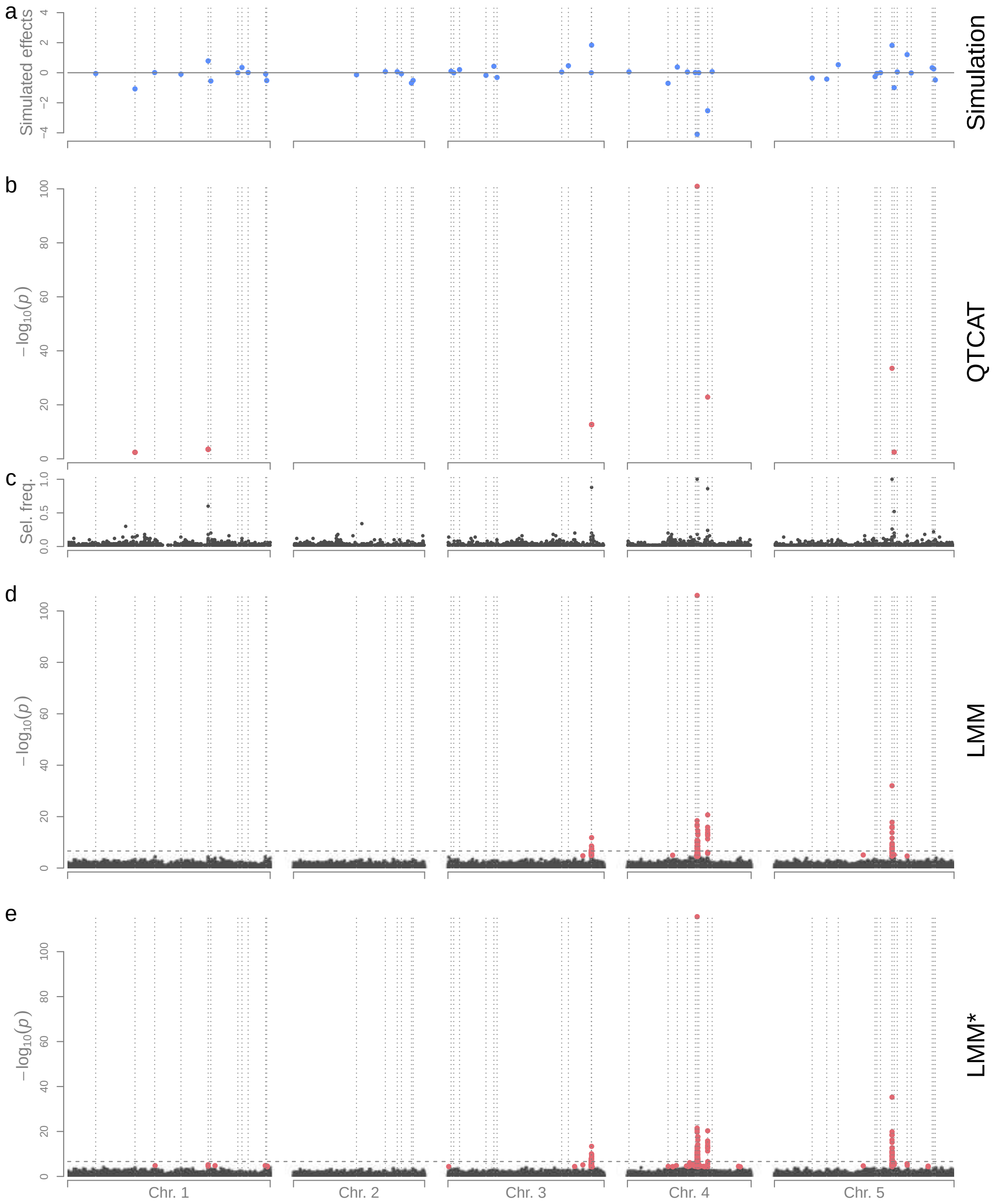

Supplementary Figure 52 Simulation of a GWA analysis based on a structured population with a heritability of 0.7 (run 52). (**a**) Simulation of 50 effects randomly drawn from a Gamma distribution and assigned to random markers. Markers with effect are highlighted with dashed lines. (**b**) Significant QTCs found by QTCAT. (**c**) LASSO selection frequency for each marker during the 50 iterations of QTCAT. (**d**) Manhattan plot of the LMM analysis. The horizontal dashed line depicts the significance threshold when controlling the multiple testing with FWER, whereas the red markers are significantly associated when controlling with FDR. (**e**) The Manhattan plot of the LMM* analysis. GRM was estimated without markers on the chromosome of the actual testing position. The results are shown as in (d).

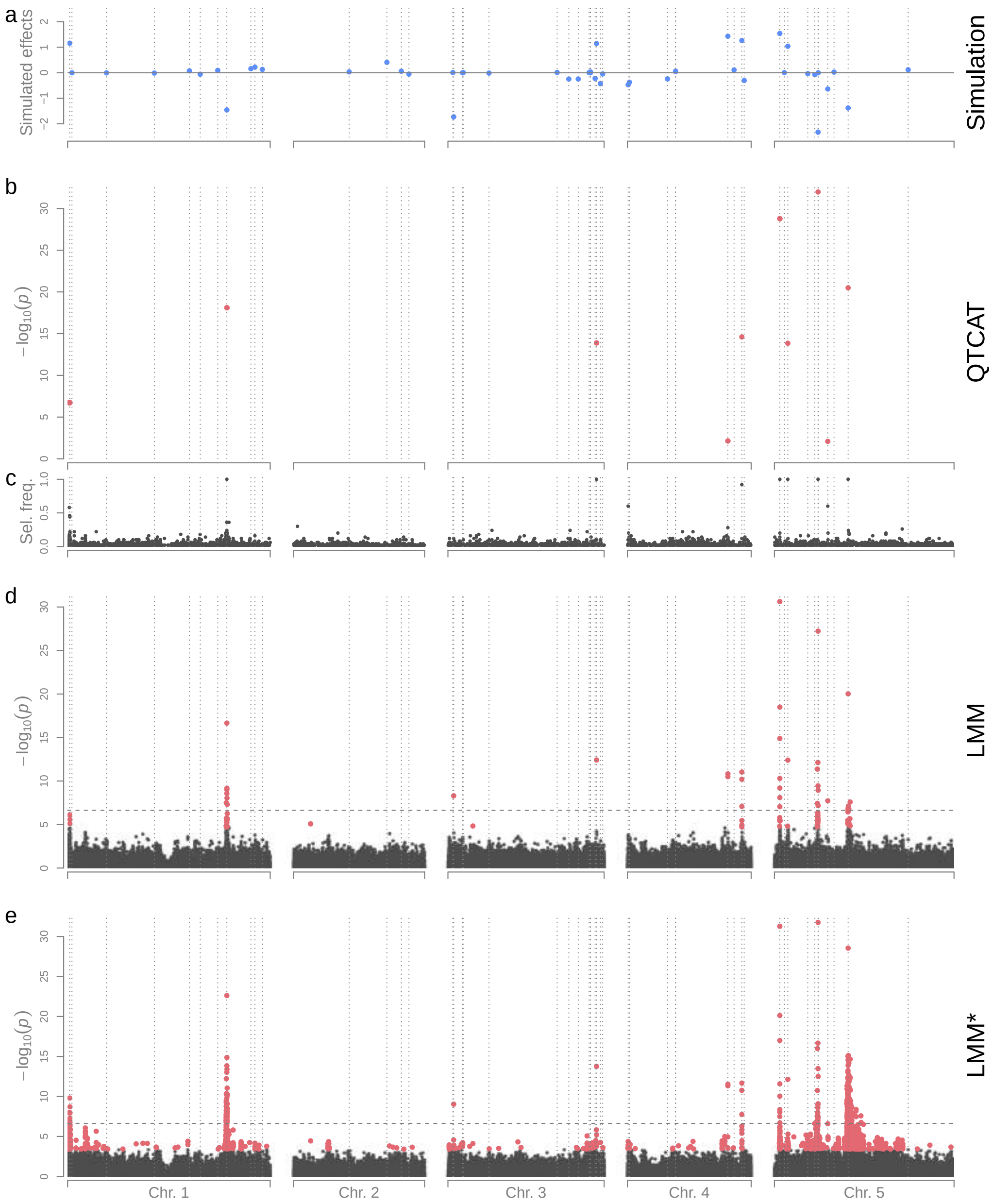

Supplementary Figure 53 Simulation of a GWA analysis based on a structured population with a heritability of 0.7 (run 53). (**a**) Simulation of 50 effects randomly drawn from a Gamma distribution and assigned to random markers. Markers with effect are highlighted with dashed lines. (**b**) Significant QTCs found by QTCAT. (**c**) LASSO selection frequency for each marker during the 50 iterations of QTCAT. (**d**) Manhattan plot of the LMM analysis. The horizontal dashed line depicts the significance threshold when controlling the multiple testing with FWER, whereas the red markers are significantly associated when controlling with FDR. (**e**) The Manhattan plot of the LMM* analysis. GRM was estimated without markers on the chromosome of the actual testing position. The results are shown as in (d).

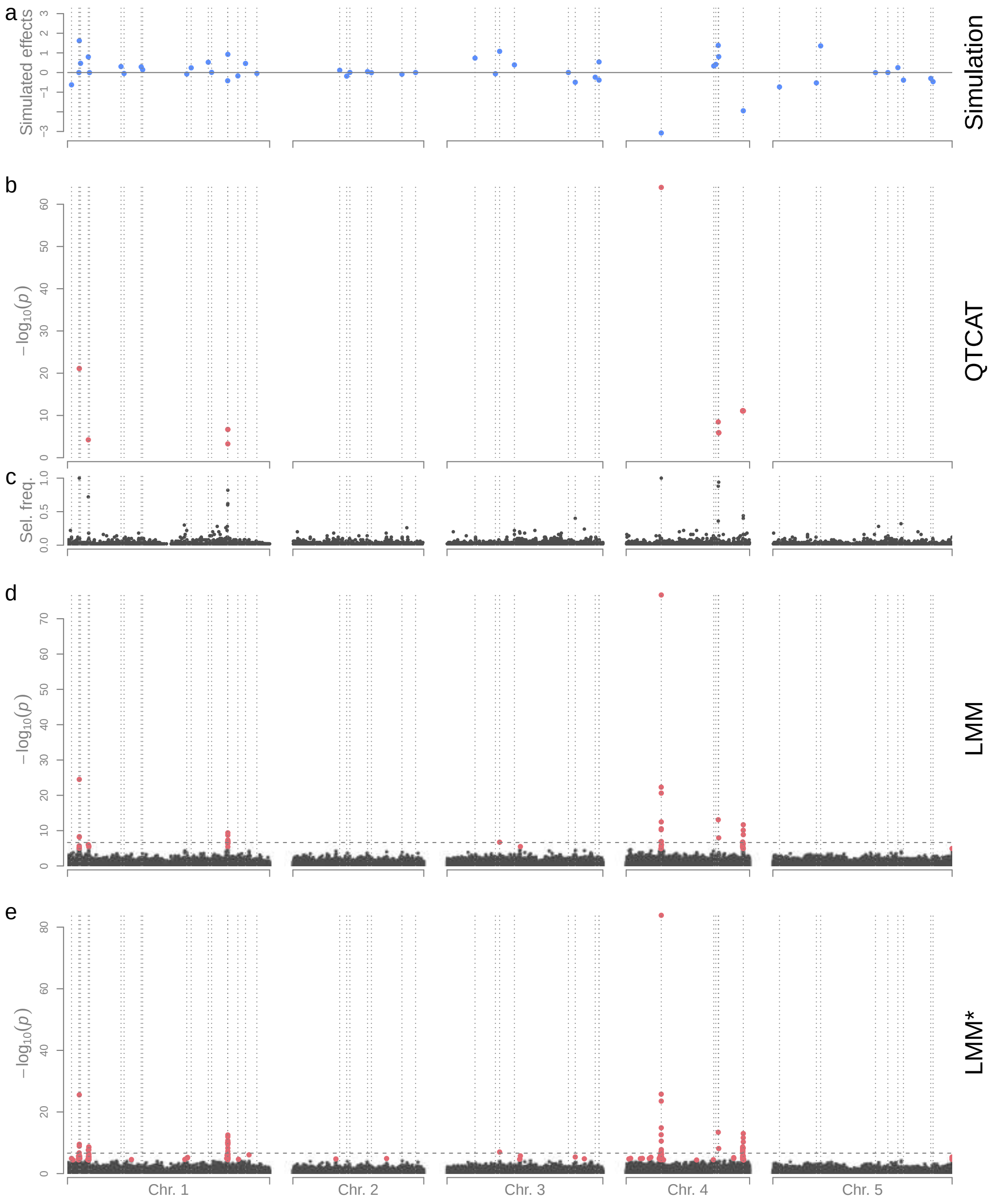

Supplementary Figure 54 Simulation of a GWA analysis based on a structured population with a heritability of 0.7 (run 54). (**a**) Simulation of 50 effects randomly drawn from a Gamma distribution and assigned to random markers. Markers with effect are highlighted with dashed lines. (**b**) Significant QTCs found by QTCAT. (**c**) LASSO selection frequency for each marker during the 50 iterations of QTCAT. (**d**) Manhattan plot of the LMM analysis. The horizontal dashed line depicts the significance threshold when controlling the multiple testing with FWER, whereas the red markers are significantly associated when controlling with FDR. (**e**) The Manhattan plot of the LMM* analysis. GRM was estimated without markers on the chromosome of the actual testing position. The results are shown as in (d).

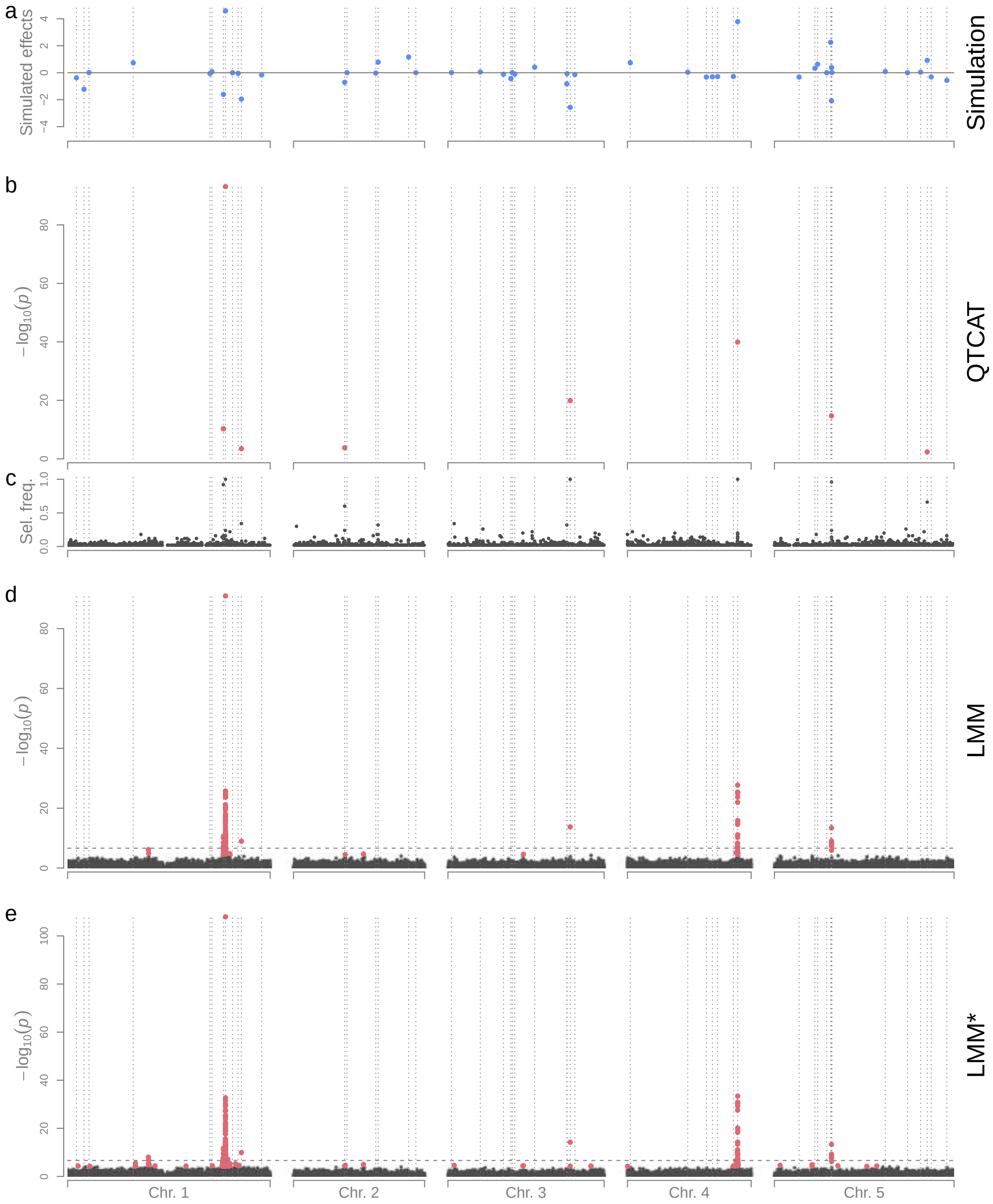

Supplementary Figure 55 Simulation of a GWA analysis based on a structured population with a heritability of 0.7 (run 55). (**a**) Simulation of 50 effects randomly drawn from a Gamma distribution and assigned to random markers. Markers with effect are highlighted with dashed lines. (**b**) Significant QTCs found by QTCAT. (**c**) LASSO selection frequency for each marker during the 50 iterations of QTCAT. (**d**) Manhattan plot of the LMM analysis. The horizontal dashed line depicts the significance threshold when controlling the multiple testing with FWER, whereas the red markers are significantly associated when controlling with FDR. (**e**) The Manhattan plot of the LMM* analysis. GRM was estimated without markers on the chromosome of the actual testing position. The results are shown as in (d).

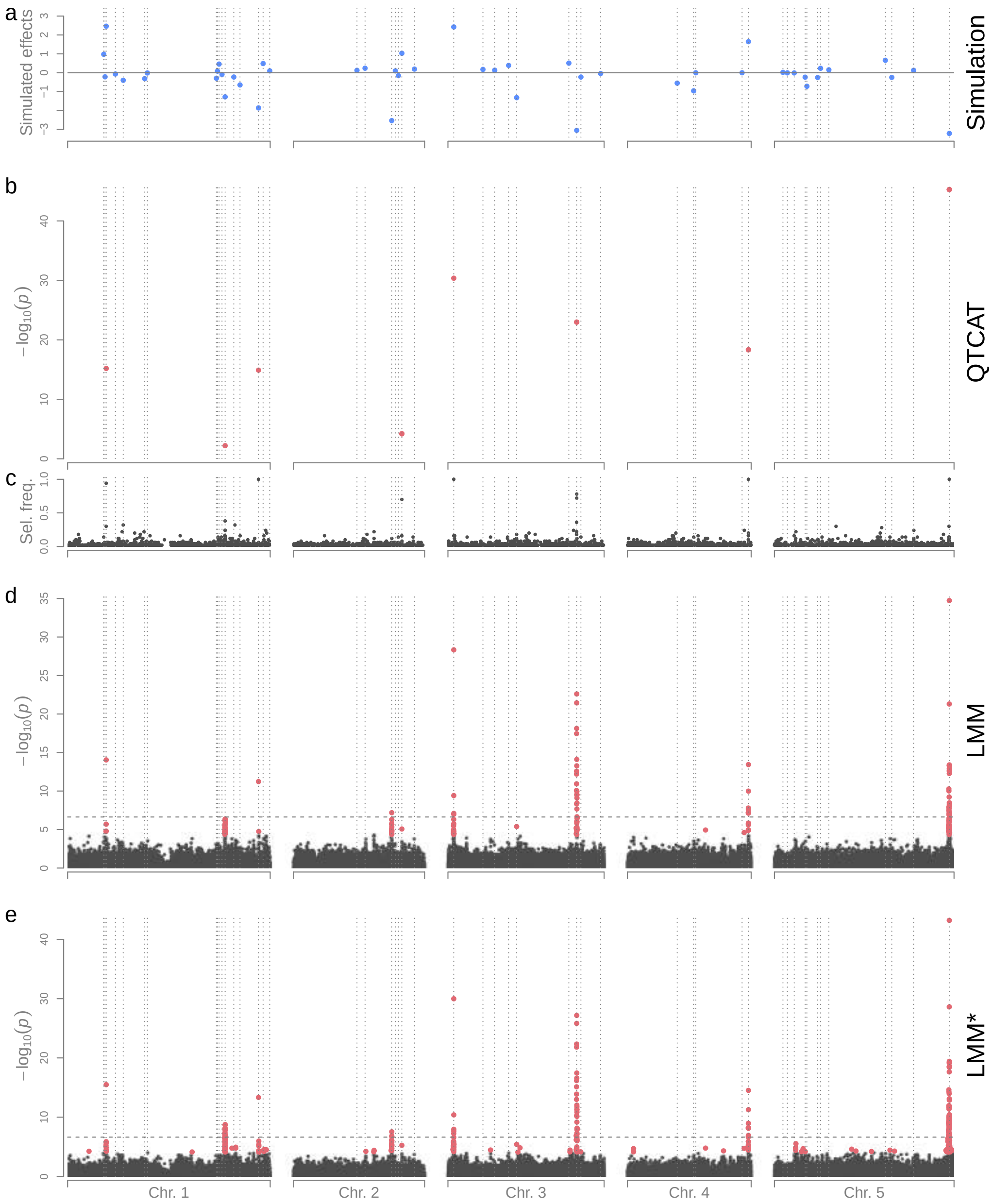

Supplementary Figure 56 Simulation of a GWA analysis based on a structured population with a heritability of 0.7 (run 56). (**a**) Simulation of 50 effects randomly drawn from a Gamma distribution and assigned to random markers. Markers with effect are highlighted with dashed lines. (**b**) Significant QTCs found by QTCAT. (**c**) LASSO selection frequency for each marker during the 50 iterations of QTCAT. (**d**) Manhattan plot of the LMM analysis. The horizontal dashed line depicts the significance threshold when controlling the multiple testing with FWER, whereas the red markers are significantly associated when controlling with FDR. (**e**) The Manhattan plot of the LMM* analysis. GRM was estimated without markers on the chromosome of the actual testing position. The results are shown as in (d).

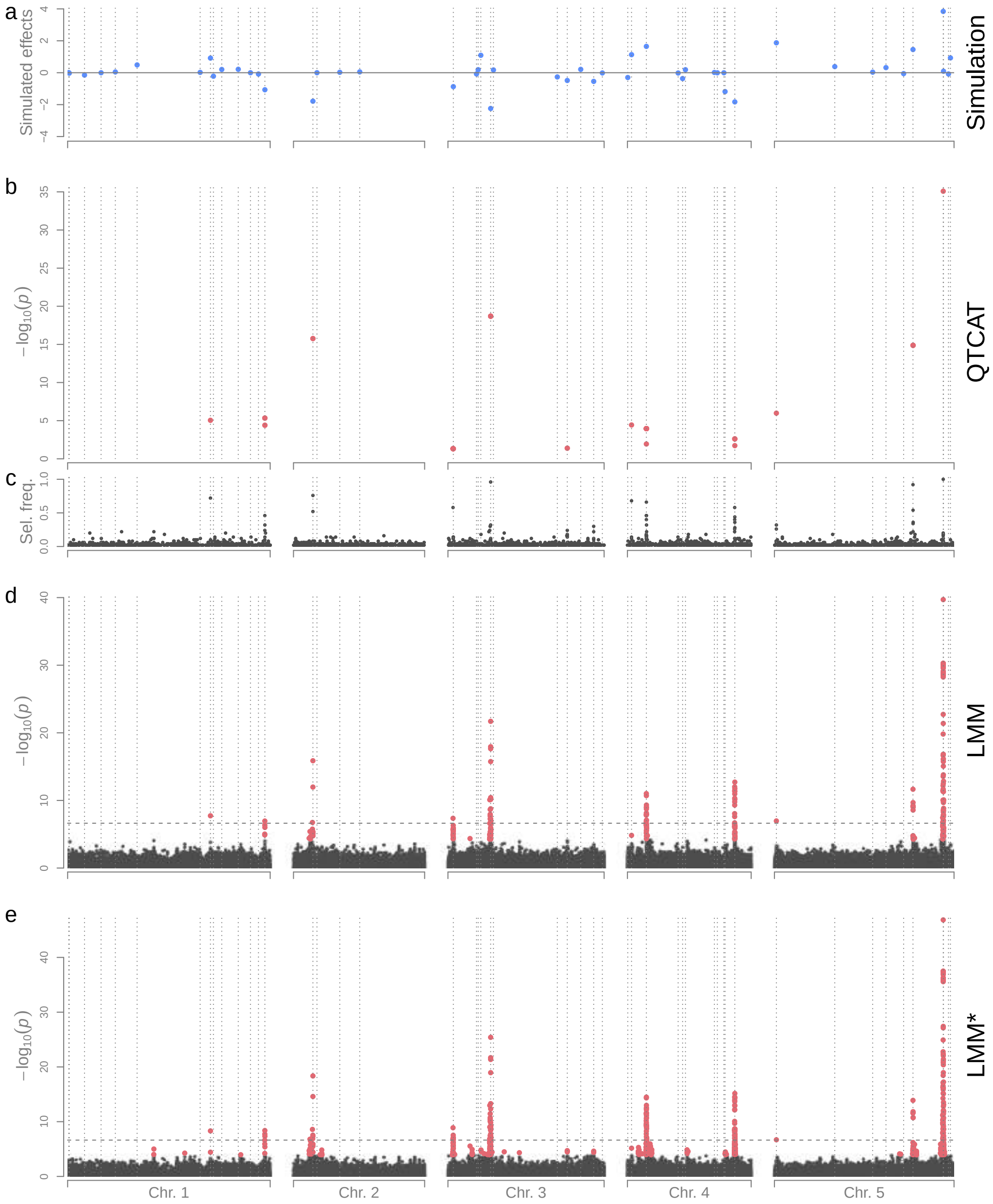

Supplementary Figure 57 Simulation of a GWA analysis based on a structured population with a heritability of 0.7 (run 57). (**a**) Simulation of 50 effects randomly drawn from a Gamma distribution and assigned to random markers. Markers with effect are highlighted with dashed lines. (**b**) Significant QTCs found by QTCAT. (**c**) LASSO selection frequency for each marker during the 50 iterations of QTCAT. (**d**) Manhattan plot of the LMM analysis. The horizontal dashed line depicts the significance threshold when controlling the multiple testing with FWER, whereas the red markers are significantly associated when controlling with FDR. (**e**) The Manhattan plot of the LMM* analysis. GRM was estimated without markers on the chromosome of the actual testing position. The results are shown as in (d).

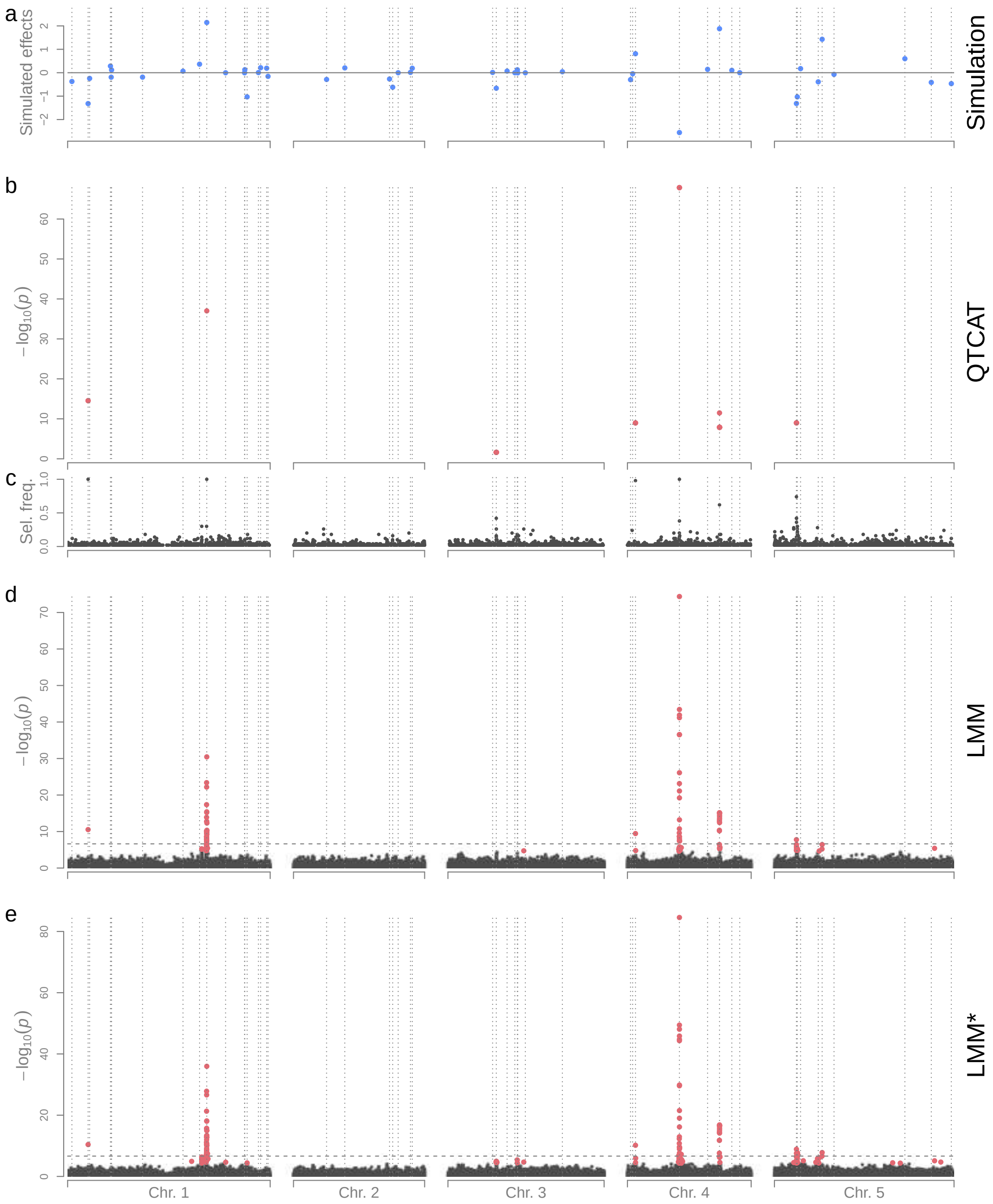

Supplementary Figure 58 Simulation of a GWA analysis based on a structured population with a heritability of 0.7 (run 58). (**a**) Simulation of 50 effects randomly drawn from a Gamma distribution and assigned to random markers. Markers with effect are highlighted with dashed lines. (**b**) Significant QTCs found by QTCAT. (**c**) LASSO selection frequency for each marker during the 50 iterations of QTCAT. (**d**) Manhattan plot of the LMM analysis. The horizontal dashed line depicts the significance threshold when controlling the multiple testing with FWER, whereas the red markers are significantly associated when controlling with FDR. (**e**) The Manhattan plot of the LMM* analysis. GRM was estimated without markers on the chromosome of the actual testing position. The results are shown as in (d).

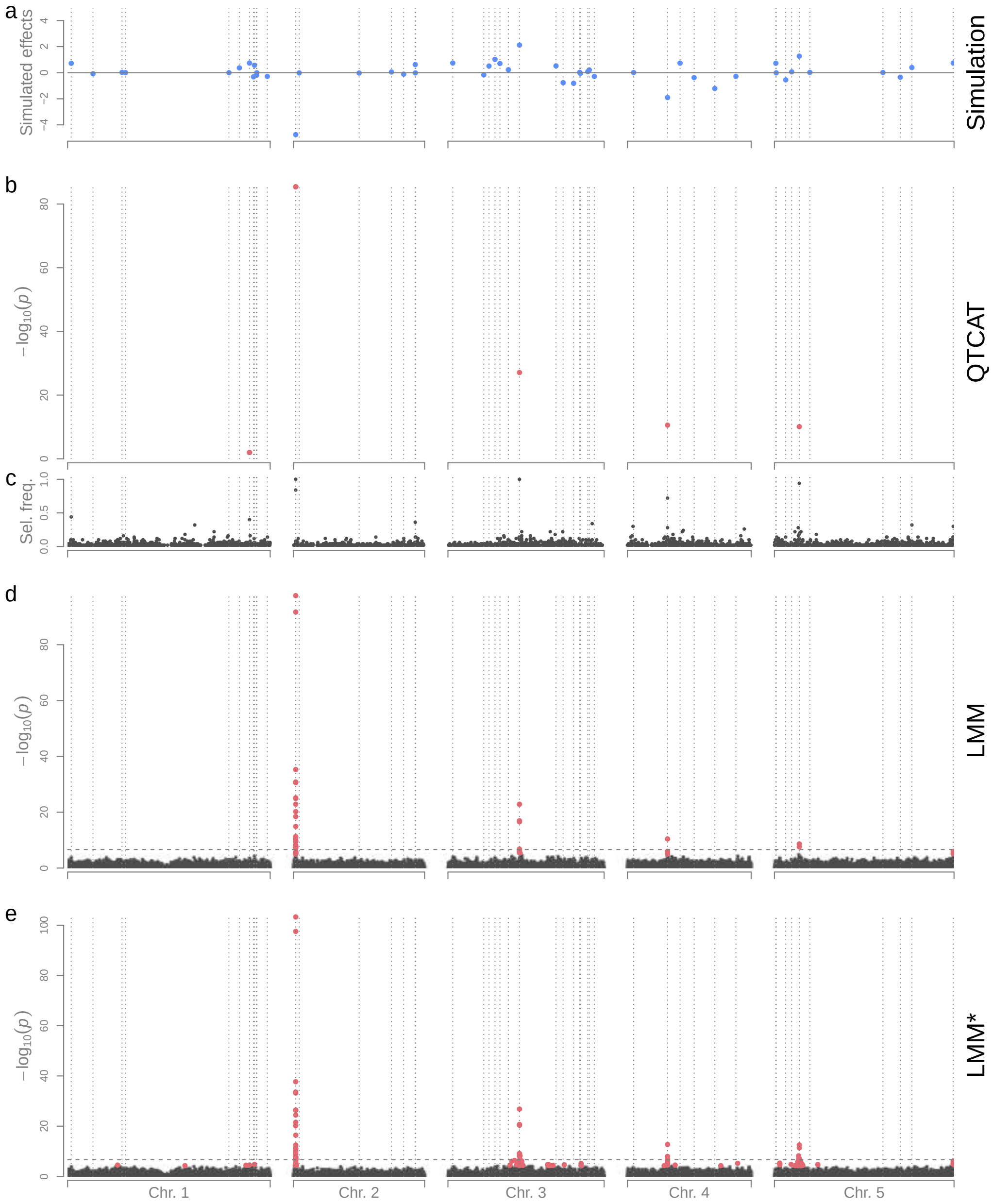

Supplementary Figure 59 Simulation of a GWA analysis based on a structured population with a heritability of 0.7 (run 59). (**a**) Simulation of 50 effects randomly drawn from a Gamma distribution and assigned to random markers. Markers with effect are highlighted with dashed lines. (**b**) Significant QTCs found by QTCAT. (**c**) LASSO selection frequency for each marker during the 50 iterations of QTCAT. (**d**) Manhattan plot of the LMM analysis. The horizontal dashed line depicts the significance threshold when controlling the multiple testing with FWER, whereas the red markers are significantly associated when controlling with FDR. (**e**) The Manhattan plot of the LMM* analysis. GRM was estimated without markers on the chromosome of the actual testing position. The results are shown as in (d).

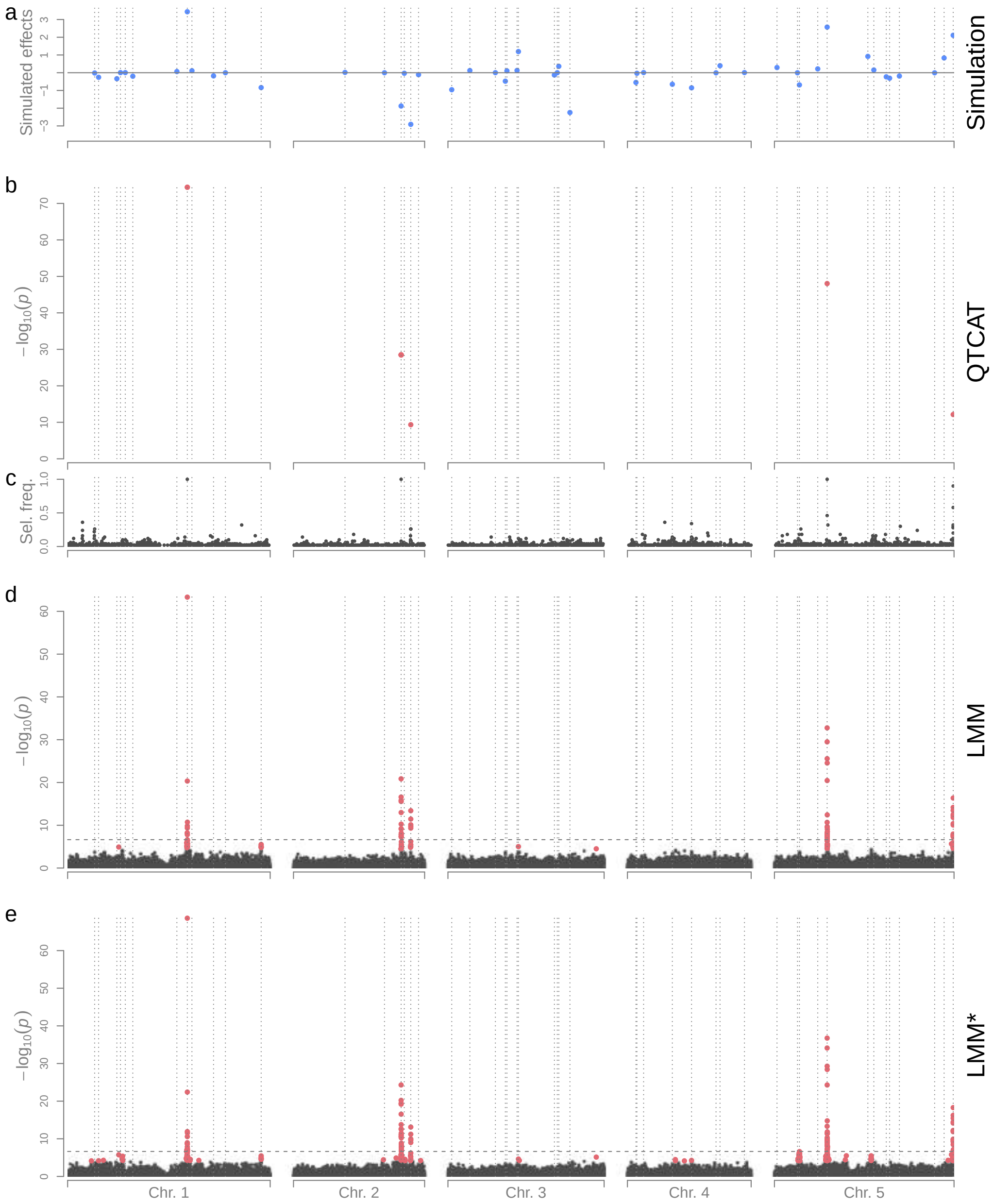

Supplementary Figure 60 Simulation of a GWA analysis based on a structured population with a heritability of 0.7 (run 60). (**a**) Simulation of 50 effects randomly drawn from a Gamma distribution and assigned to random markers. Markers with effect are highlighted with dashed lines. (**b**) Significant QTCs found by QTCAT. (**c**) LASSO selection frequency for each marker during the 50 iterations of QTCAT. (**d**) Manhattan plot of the LMM analysis. The horizontal dashed line depicts the significance threshold when controlling the multiple testing with FWER, whereas the red markers are significantly associated when controlling with FDR. (**e**) The Manhattan plot of the LMM* analysis. GRM was estimated without markers on the chromosome of the actual testing position. The results are shown as in (d).

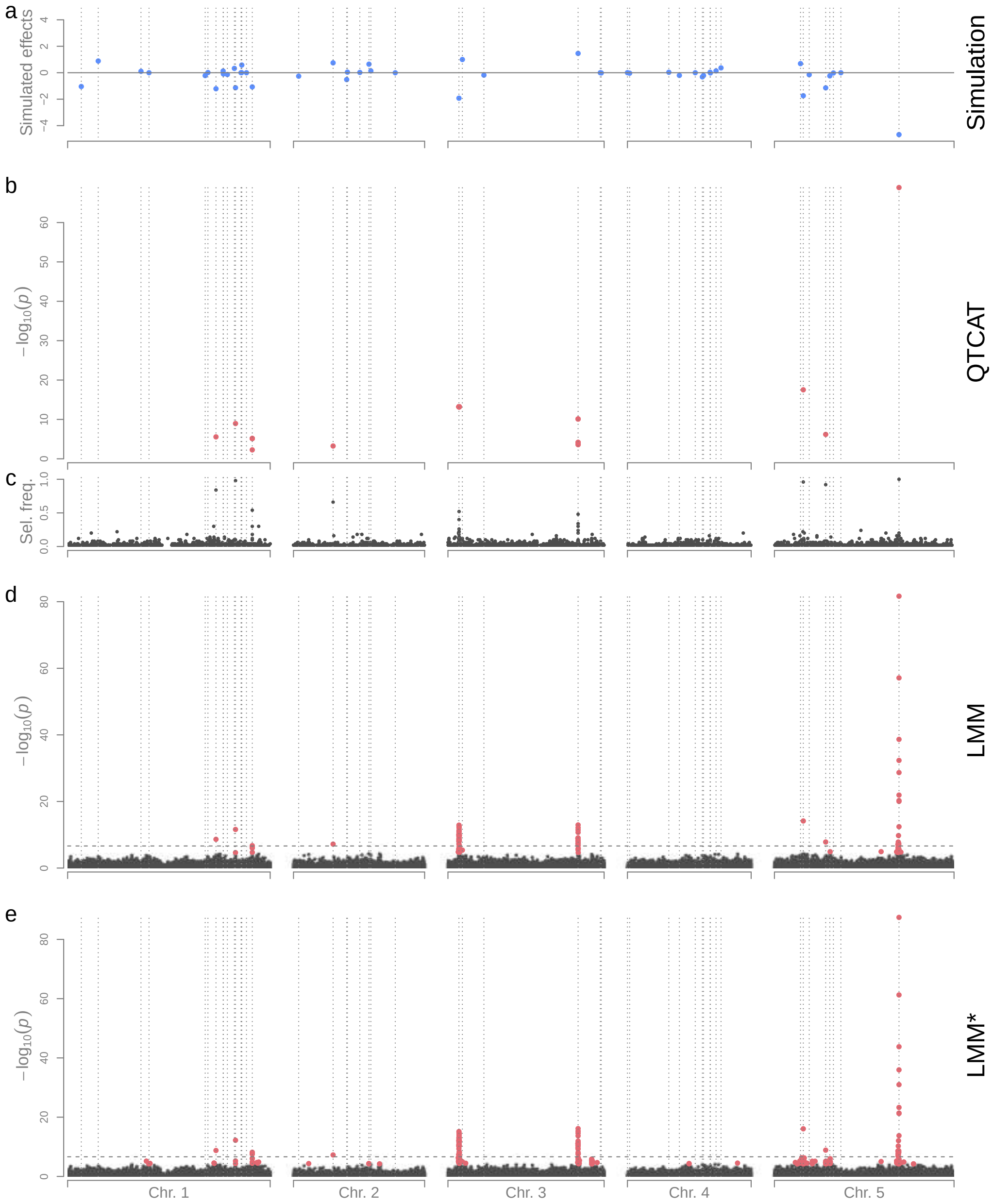

Supplementary Figure 61 Simulation of a GWA analysis based on a structured population with a heritability of 0.7 (run 61). (**a**) Simulation of 50 effects randomly drawn from a Gamma distribution and assigned to random markers. Markers with effect are highlighted with dashed lines. (**b**) Significant QTCs found by QTCAT. (**c**) LASSO selection frequency for each marker during the 50 iterations of QTCAT. (**d**) Manhattan plot of the LMM analysis. The horizontal dashed line depicts the significance threshold when controlling the multiple testing with FWER, whereas the red markers are significantly associated when controlling with FDR. (**e**) The Manhattan plot of the LMM* analysis. GRM was estimated without markers on the chromosome of the actual testing position. The results are shown as in (d).

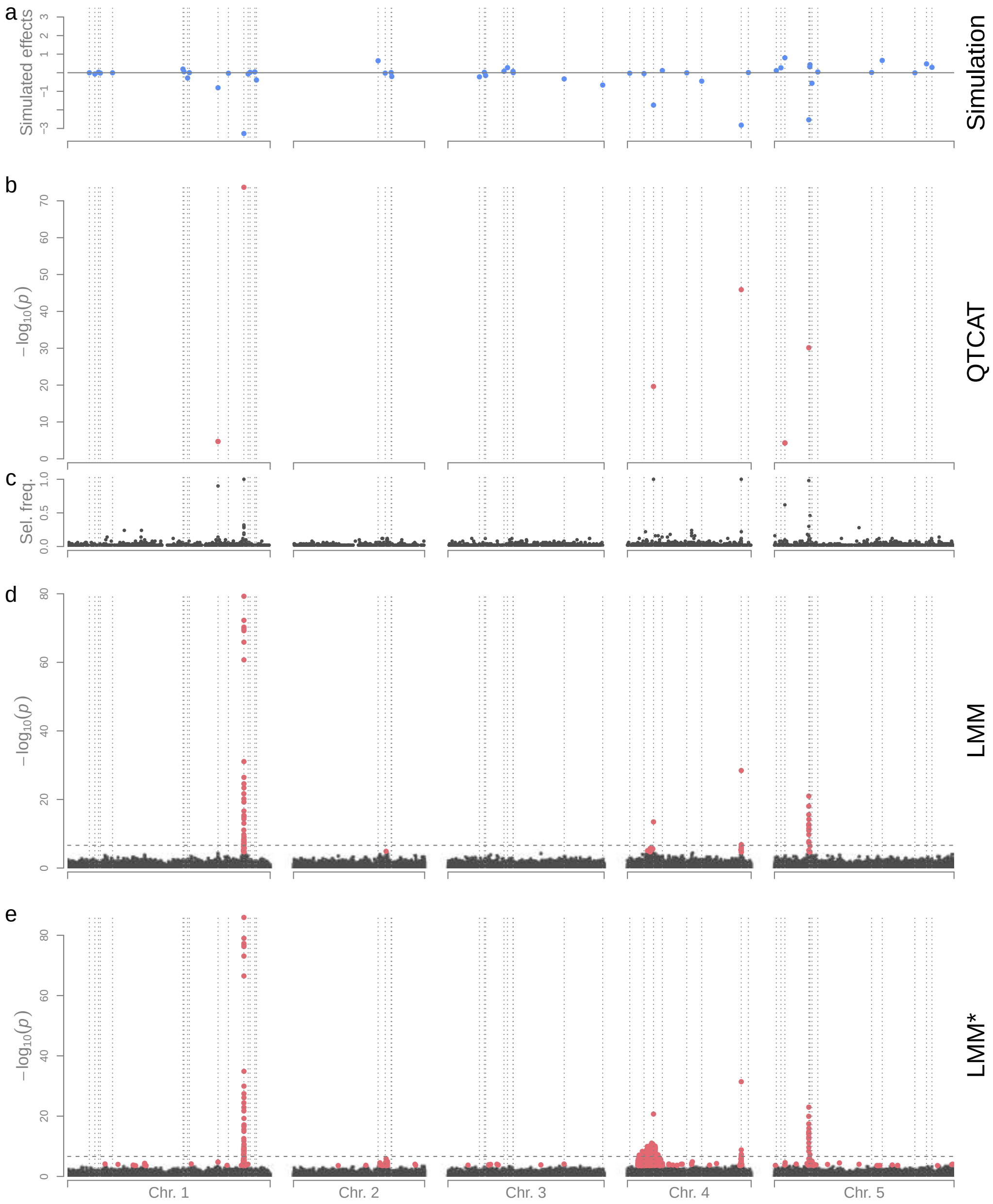

Supplementary Figure 62 Simulation of a GWA analysis based on a structured population with a heritability of 0.7 (run 62). (**a**) Simulation of 50 effects randomly drawn from a Gamma distribution and assigned to random markers. Markers with effect are highlighted with dashed lines. (**b**) Significant QTCs found by QTCAT. (**c**) LASSO selection frequency for each marker during the 50 iterations of QTCAT. (**d**) Manhattan plot of the LMM analysis. The horizontal dashed line depicts the significance threshold when controlling the multiple testing with FWER, whereas the red markers are significantly associated when controlling with FDR. (**e**) The Manhattan plot of the LMM* analysis. GRM was estimated without markers on the chromosome of the actual testing position. The results are shown as in (d).

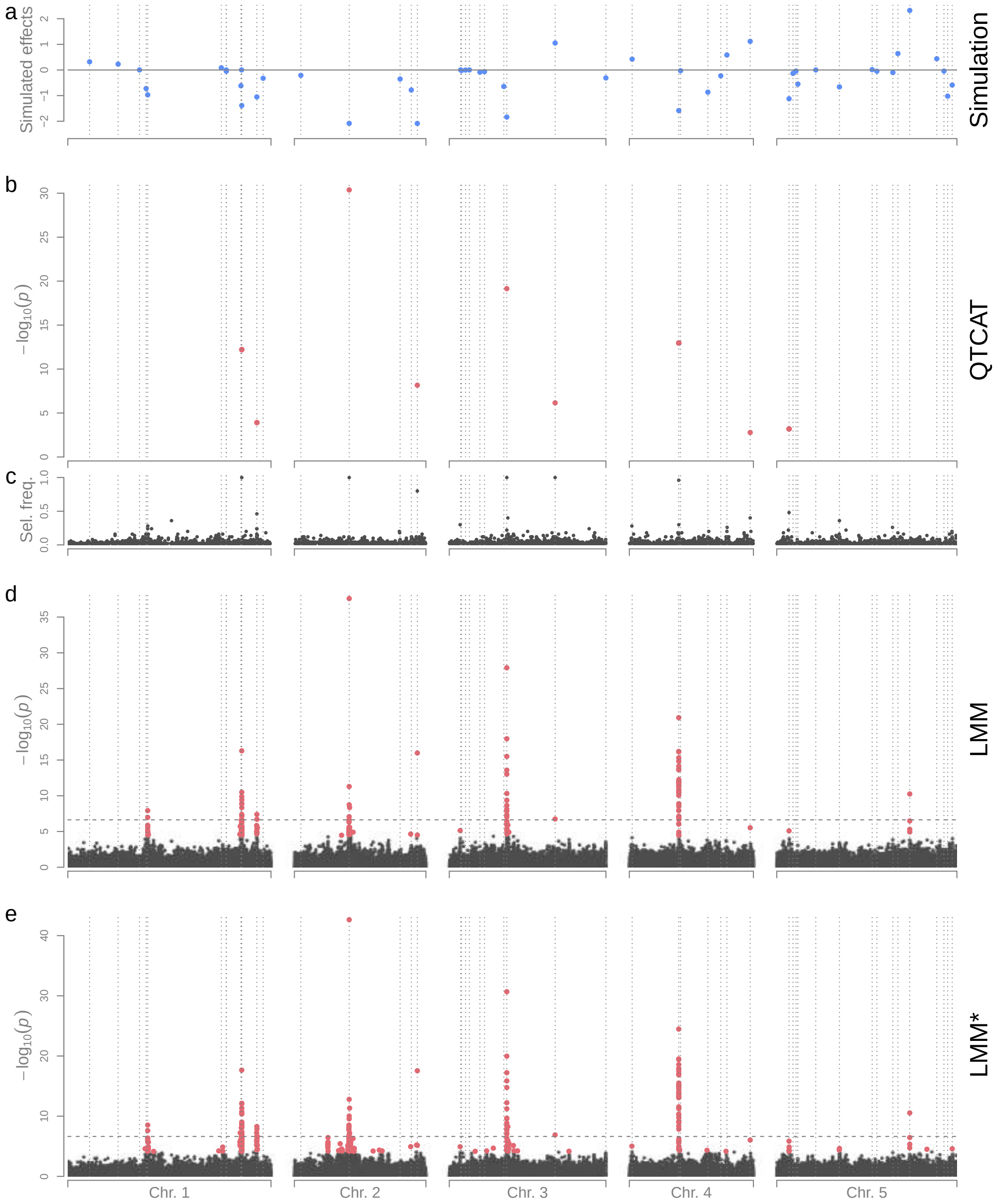

Supplementary Figure 63 Simulation of a GWA analysis based on a structured population with a heritability of 0.7 (run 63). (**a**) Simulation of 50 effects randomly drawn from a Gamma distribution and assigned to random markers. Markers with effect are highlighted with dashed lines. (**b**) Significant QTCs found by QTCAT. (**c**) LASSO selection frequency for each marker during the 50 iterations of QTCAT. (**d**) Manhattan plot of the LMM analysis. The horizontal dashed line depicts the significance threshold when controlling the multiple testing with FWER, whereas the red markers are significantly associated when controlling with FDR. (**e**) The Manhattan plot of the LMM* analysis. GRM was estimated without markers on the chromosome of the actual testing position. The results are shown as in (d).

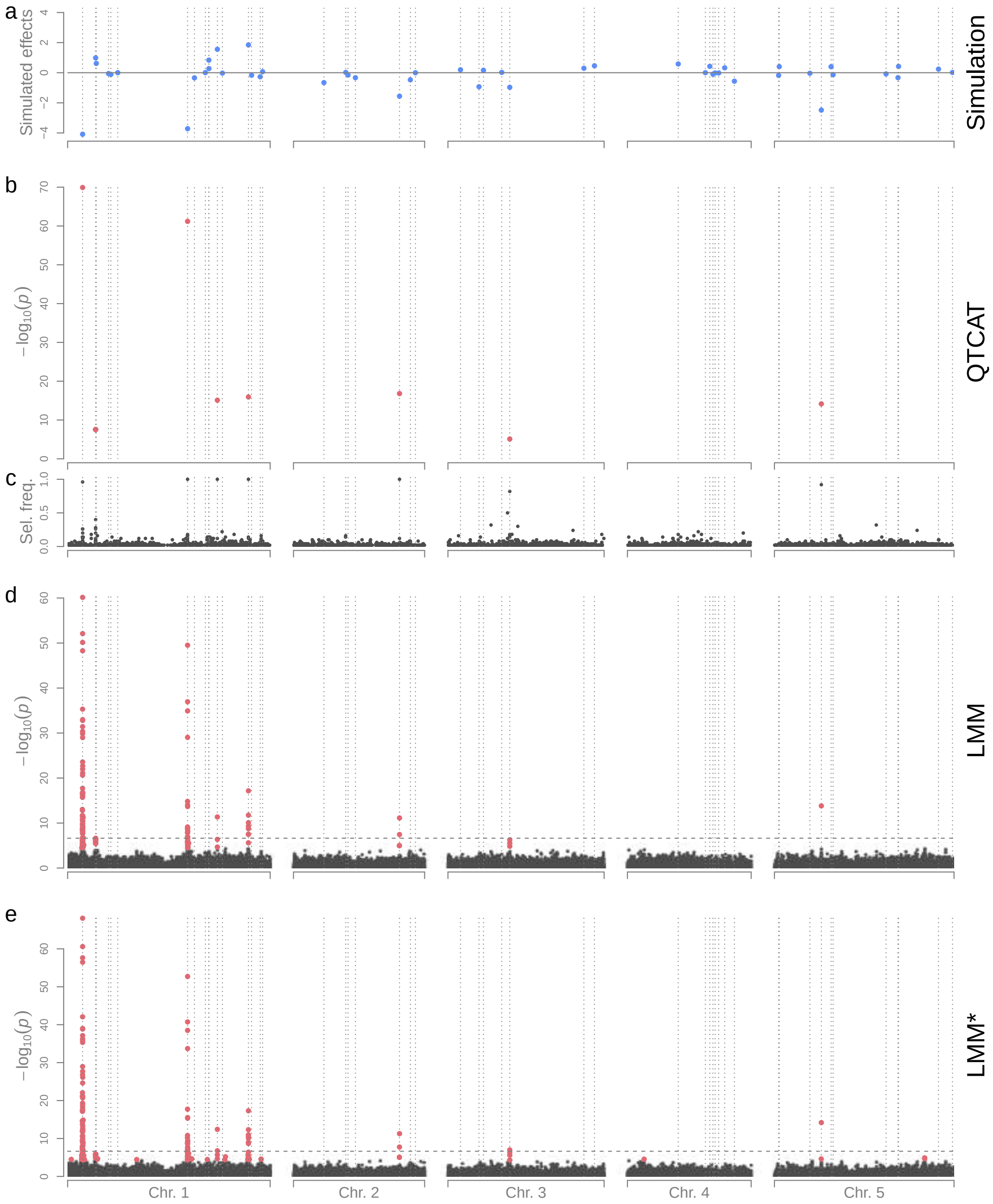

Supplementary Figure 64 Simulation of a GWA analysis based on a structured population with a heritability of 0.7 (run 64). (**a**) Simulation of 50 effects randomly drawn from a Gamma distribution and assigned to random markers. Markers with effect are highlighted with dashed lines. (**b**) Significant QTCs found by QTCAT. (**c**) LASSO selection frequency for each marker during the 50 iterations of QTCAT. (**d**) Manhattan plot of the LMM analysis. The horizontal dashed line depicts the significance threshold when controlling the multiple testing with FWER, whereas the red markers are significantly associated when controlling with FDR. (**e**) The Manhattan plot of the LMM* analysis. GRM was estimated without markers on the chromosome of the actual testing position. The results are shown as in (d).

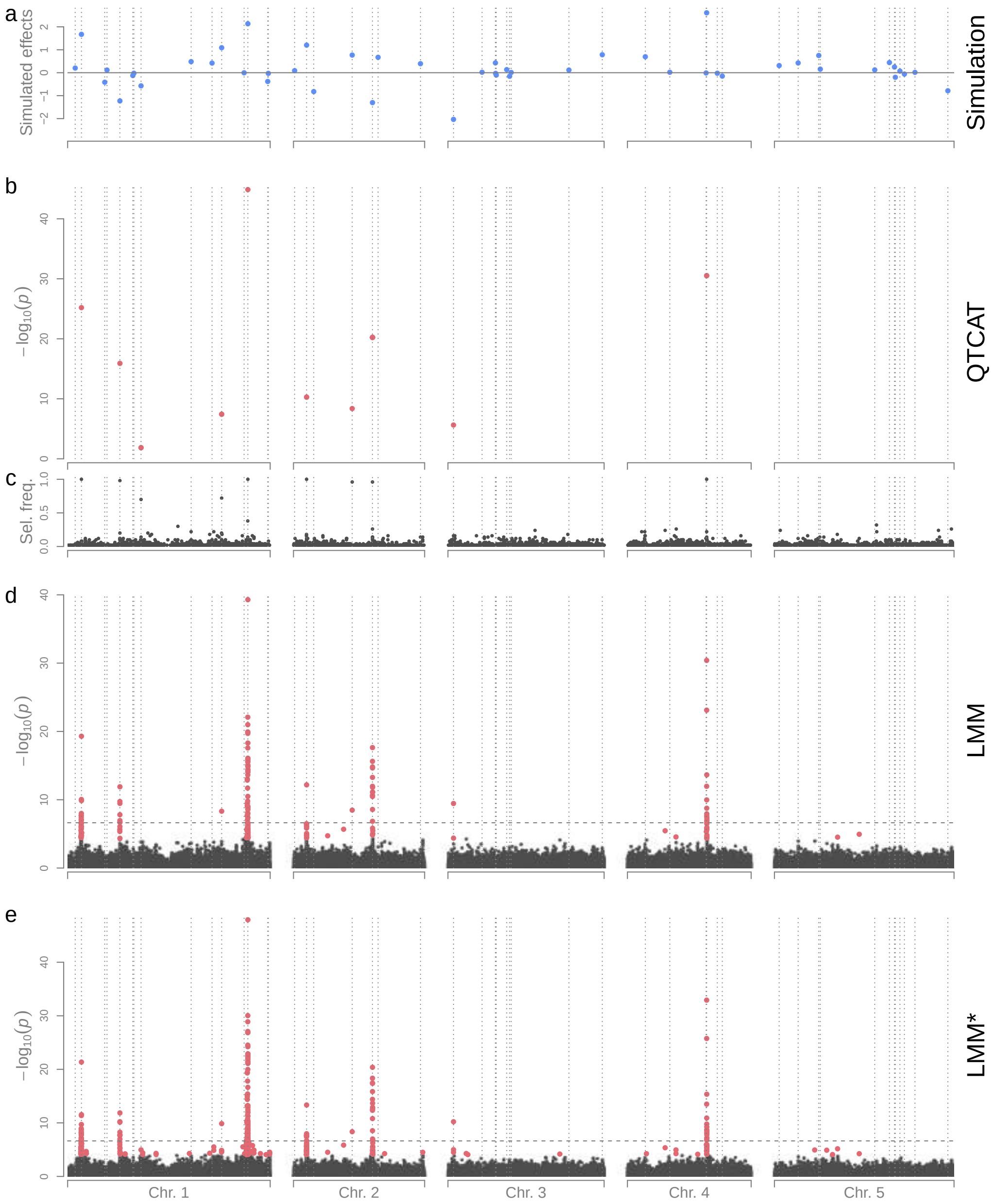

Supplementary Figure 65 Simulation of a GWA analysis based on a structured population with a heritability of 0.7 (run 65). (**a**) Simulation of 50 effects randomly drawn from a Gamma distribution and assigned to random markers. Markers with effect are highlighted with dashed lines. (**b**) Significant QTCs found by QTCAT. (**c**) LASSO selection frequency for each marker during the 50 iterations of QTCAT. (**d**) Manhattan plot of the LMM analysis. The horizontal dashed line depicts the significance threshold when controlling the multiple testing with FWER, whereas the red markers are significantly associated when controlling with FDR. (**e**) The Manhattan plot of the LMM* analysis. GRM was estimated without markers on the chromosome of the actual testing position. The results are shown as in (d).

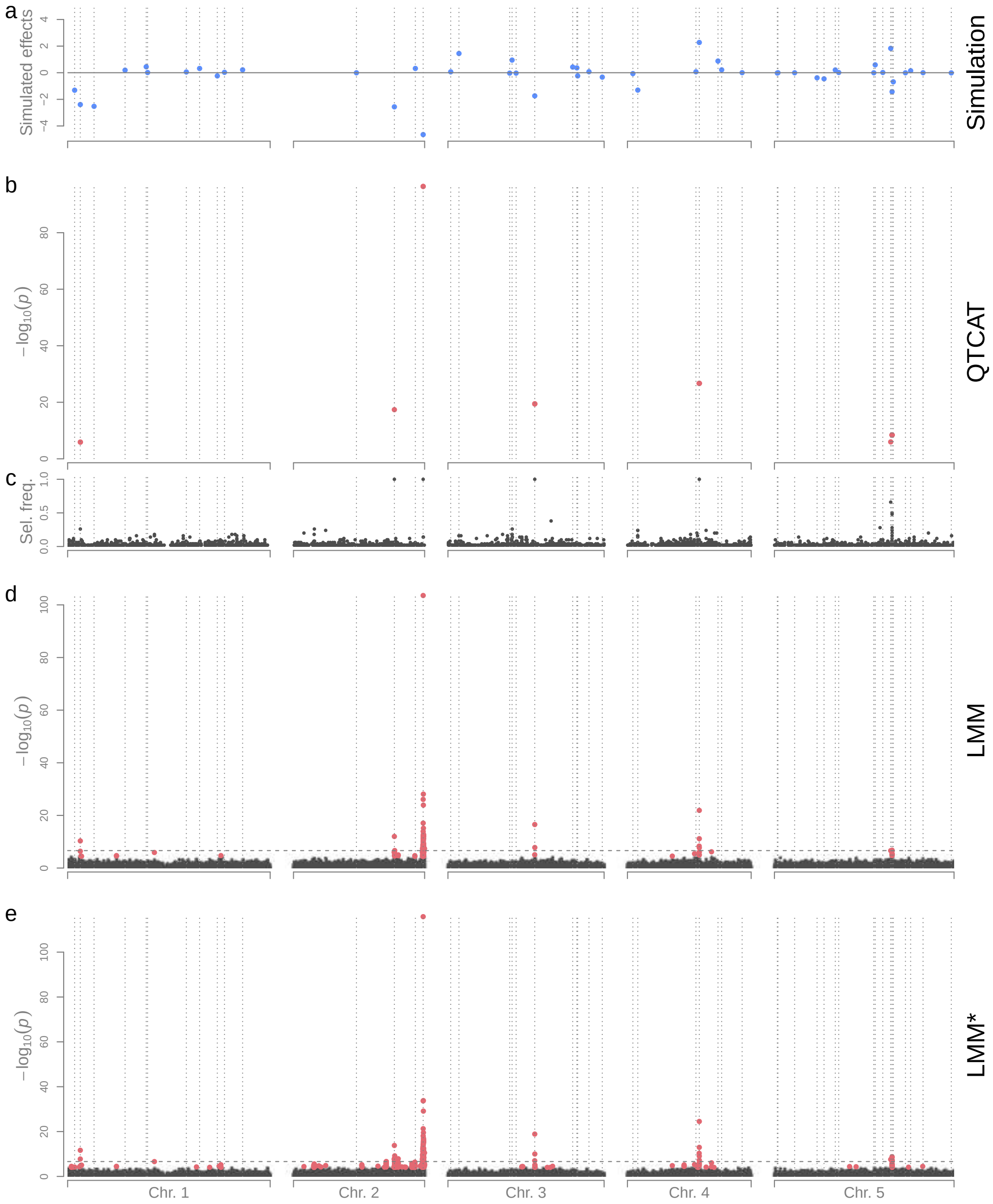

Supplementary Figure 66 Simulation of a GWA analysis based on a structured population with a heritability of 0.7 (run 66). (**a**) Simulation of 50 effects randomly drawn from a Gamma distribution and assigned to random markers. Markers with effect are highlighted with dashed lines. (**b**) Significant QTCs found by QTCAT. (**c**) LASSO selection frequency for each marker during the 50 iterations of QTCAT. (**d**) Manhattan plot of the LMM analysis. The horizontal dashed line depicts the significance threshold when controlling the multiple testing with FWER, whereas the red markers are significantly associated when controlling with FDR. (**e**) The Manhattan plot of the LMM* analysis. GRM was estimated without markers on the chromosome of the actual testing position. The results are shown as in (d).

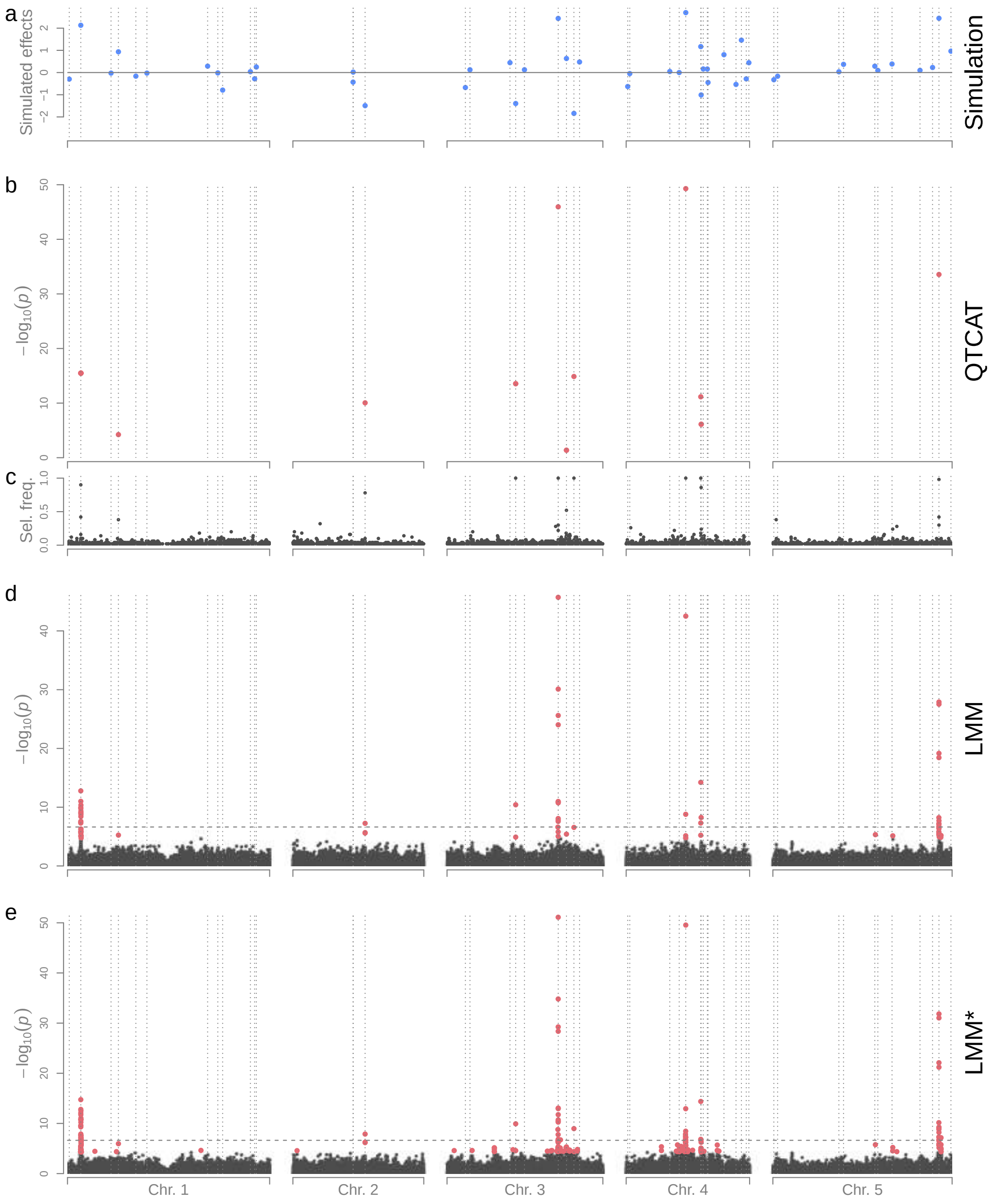

Supplementary Figure 67 Simulation of a GWA analysis based on a structured population with a heritability of 0.7 (run 67). (**a**) Simulation of 50 effects randomly drawn from a Gamma distribution and assigned to random markers. Markers with effect are highlighted with dashed lines. (**b**) Significant QTCs found by QTCAT. (**c**) LASSO selection frequency for each marker during the 50 iterations of QTCAT. (**d**) Manhattan plot of the LMM analysis. The horizontal dashed line depicts the significance threshold when controlling the multiple testing with FWER, whereas the red markers are significantly associated when controlling with FDR. (**e**) The Manhattan plot of the LMM* analysis. GRM was estimated without markers on the chromosome of the actual testing position. The results are shown as in (d).

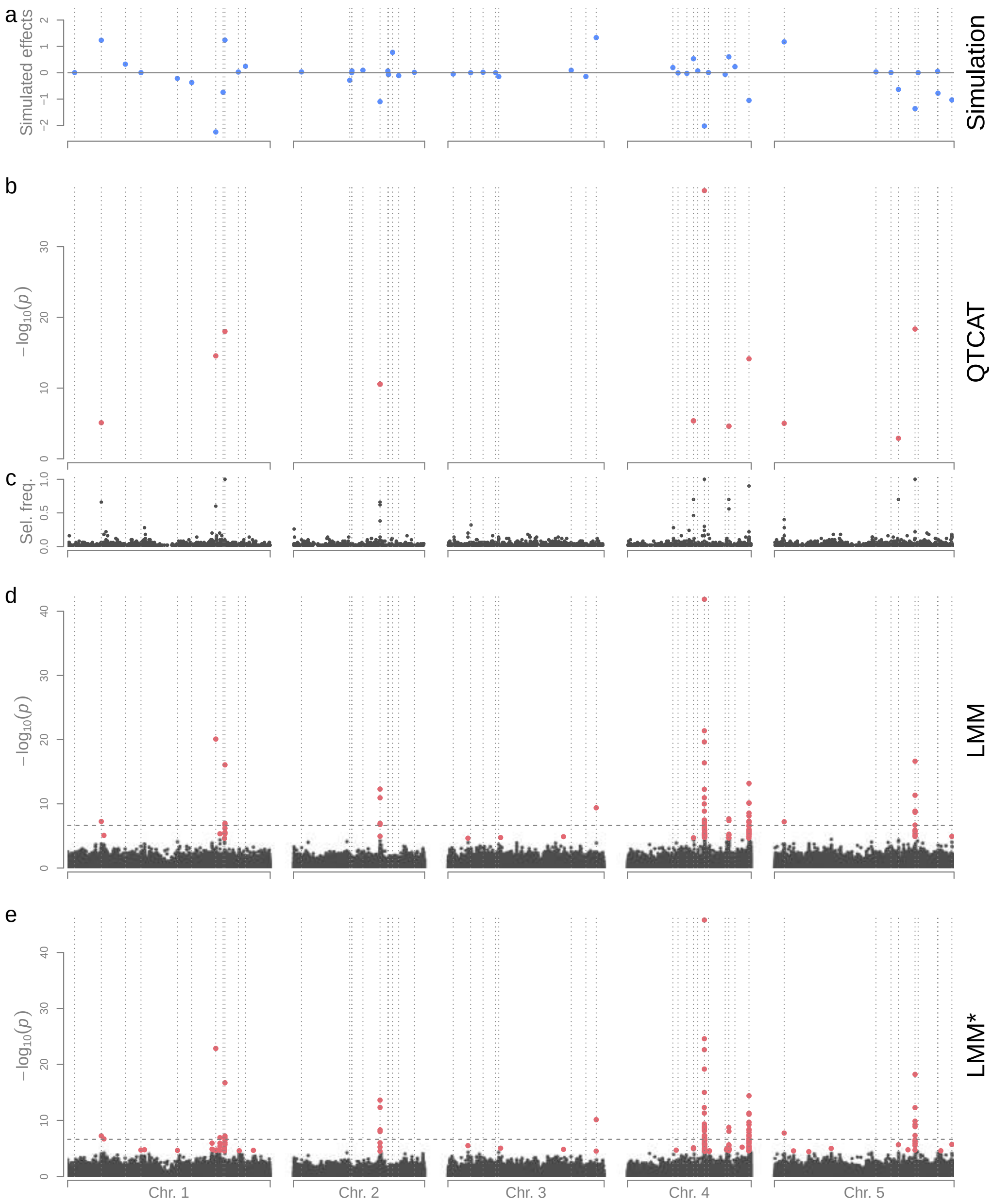

Supplementary Figure 68 Simulation of a GWA analysis based on a structured population with a heritability of 0.7 (run 68). (**a**) Simulation of 50 effects randomly drawn from a Gamma distribution and assigned to random markers. Markers with effect are highlighted with dashed lines. (**b**) Significant QTCs found by QTCAT. (**c**) LASSO selection frequency for each marker during the 50 iterations of QTCAT. (**d**) Manhattan plot of the LMM analysis. The horizontal dashed line depicts the significance threshold when controlling the multiple testing with FWER, whereas the red markers are significantly associated when controlling with FDR. (**e**) The Manhattan plot of the LMM* analysis. GRM was estimated without markers on the chromosome of the actual testing position. The results are shown as in (d).

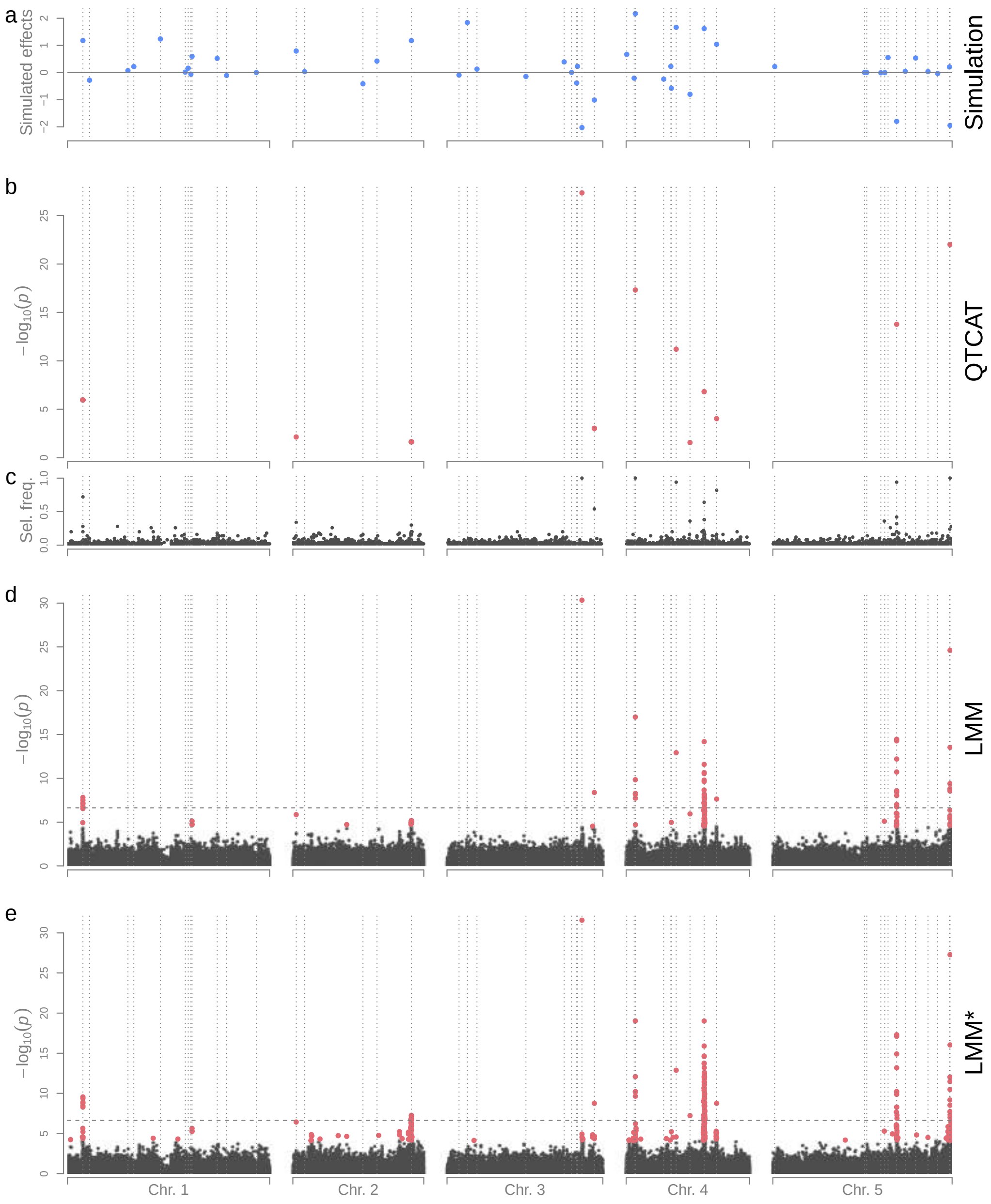

Supplementary Figure 69 Simulation of a GWA analysis based on a structured population with a heritability of 0.7 (run 69). (**a**) Simulation of 50 effects randomly drawn from a Gamma distribution and assigned to random markers. Markers with effect are highlighted with dashed lines. (**b**) Significant QTCs found by QTCAT. (**c**) LASSO selection frequency for each marker during the 50 iterations of QTCAT. (**d**) Manhattan plot of the LMM analysis. The horizontal dashed line depicts the significance threshold when controlling the multiple testing with FWER, whereas the red markers are significantly associated when controlling with FDR. (**e**) The Manhattan plot of the LMM* analysis. GRM was estimated without markers on the chromosome of the actual testing position. The results are shown as in (d).

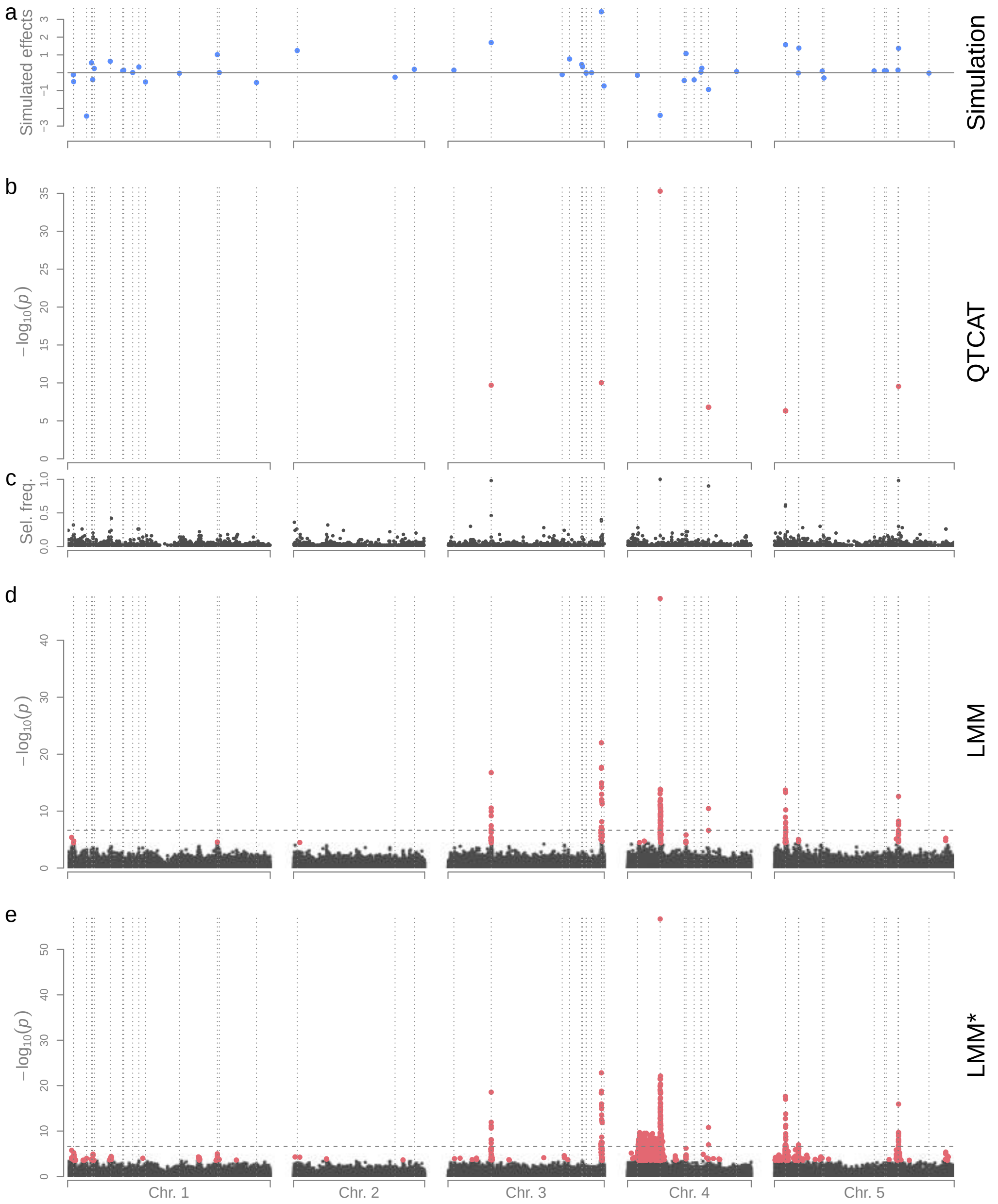

Supplementary Figure 70 Simulation of a GWA analysis based on a structured population with a heritability of 0.7 (run 70). (**a**) Simulation of 50 effects randomly drawn from a Gamma distribution and assigned to random markers. Markers with effect are highlighted with dashed lines. (**b**) Significant QTCs found by QTCAT. (**c**) LASSO selection frequency for each marker during the 50 iterations of QTCAT. (**d**) Manhattan plot of the LMM analysis. The horizontal dashed line depicts the significance threshold when controlling the multiple testing with FWER, whereas the red markers are significantly associated when controlling with FDR. (**e**) The Manhattan plot of the LMM* analysis. GRM was estimated without markers on the chromosome of the actual testing position. The results are shown as in (d).

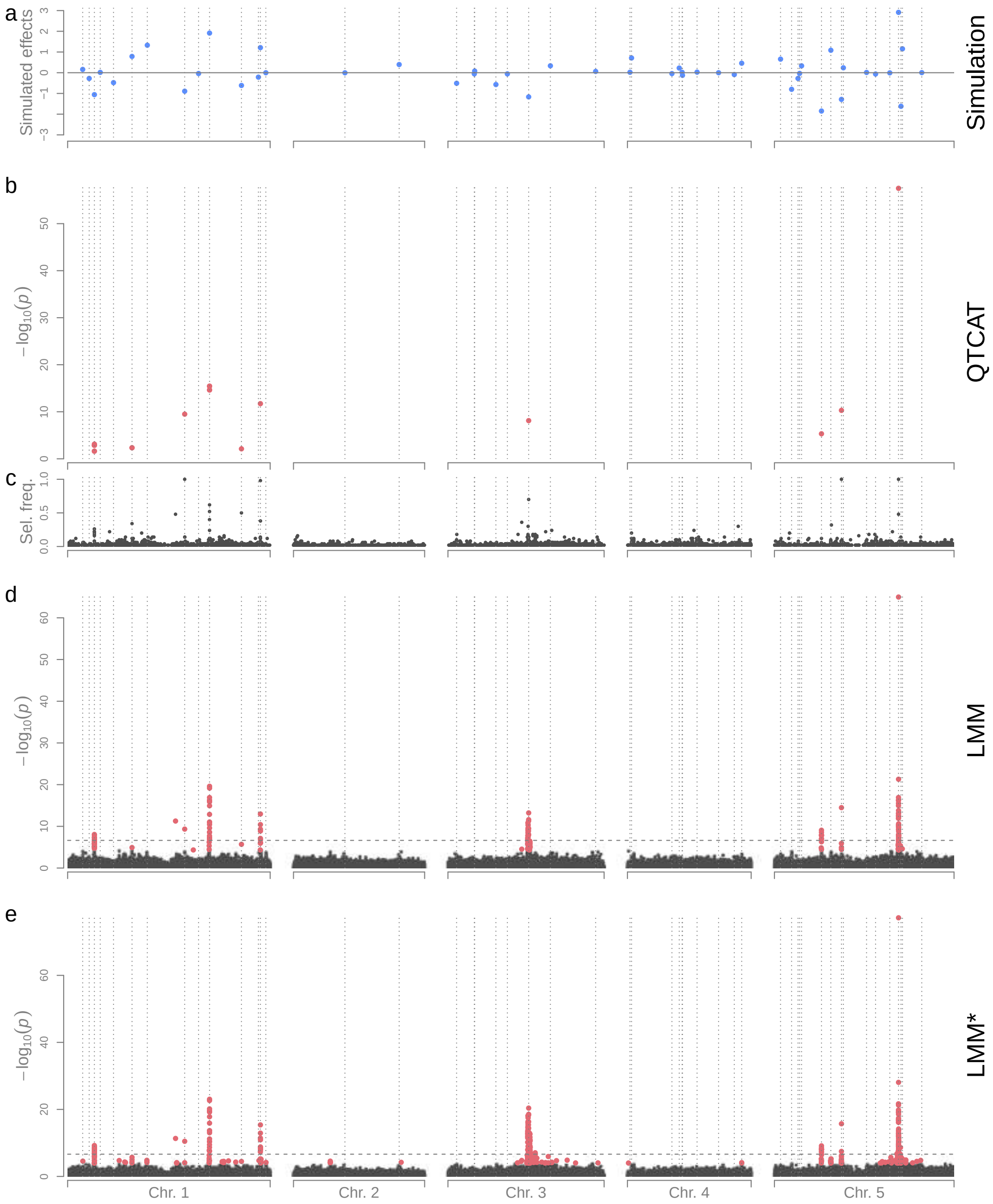

Supplementary Figure 71 Simulation of a GWA analysis based on a structured population with a heritability of 0.7 (run 71). (**a**) Simulation of 50 effects randomly drawn from a Gamma distribution and assigned to random markers. Markers with effect are highlighted with dashed lines. (**b**) Significant QTCs found by QTCAT. (**c**) LASSO selection frequency for each marker during the 50 iterations of QTCAT. (**d**) Manhattan plot of the LMM analysis. The horizontal dashed line depicts the significance threshold when controlling the multiple testing with FWER, whereas the red markers are significantly associated when controlling with FDR. (**e**) The Manhattan plot of the LMM* analysis. GRM was estimated without markers on the chromosome of the actual testing position. The results are shown as in (d).

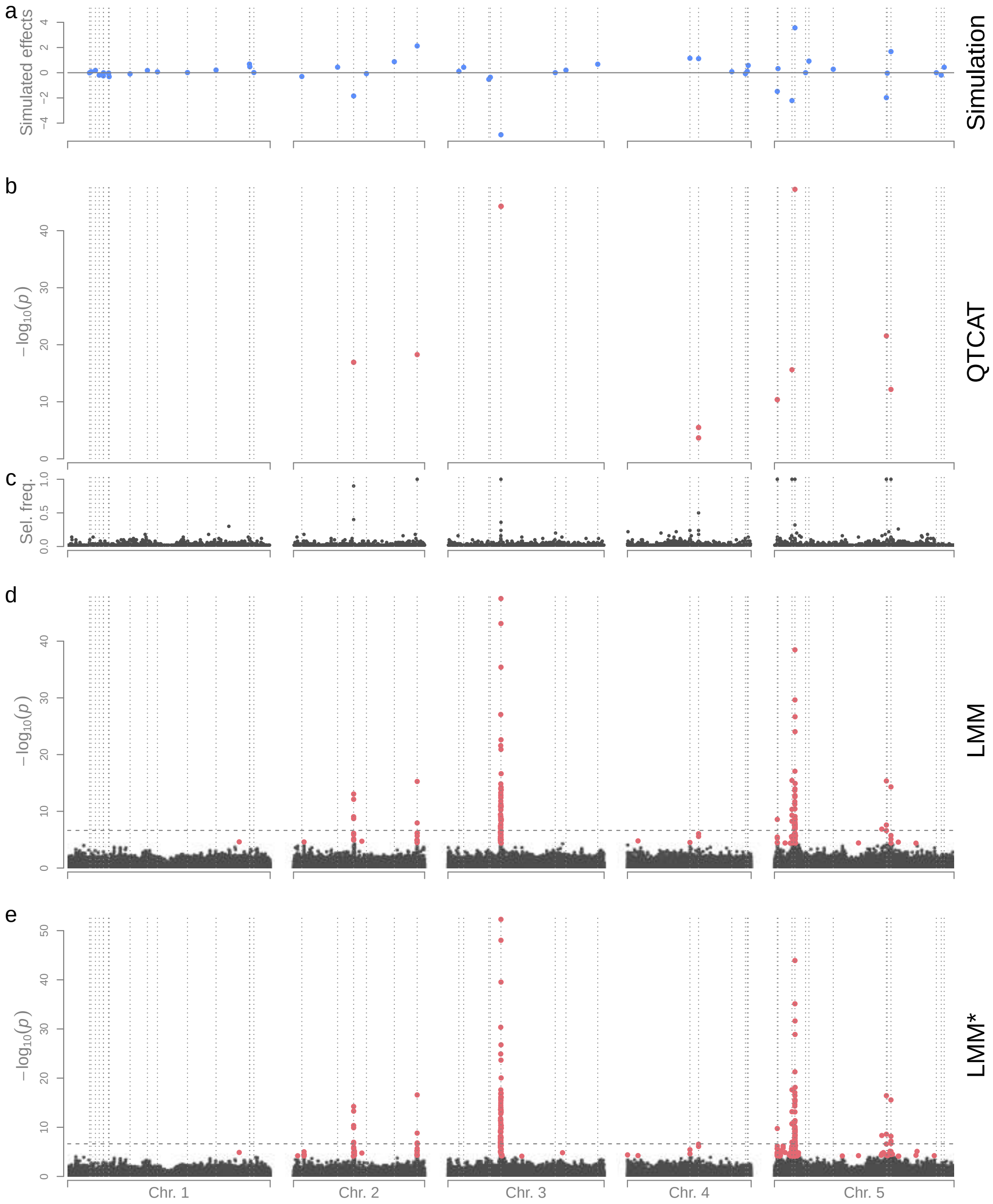

Supplementary Figure 72 Simulation of a GWA analysis based on a structured population with a heritability of 0.7 (run 72). (**a**) Simulation of 50 effects randomly drawn from a Gamma distribution and assigned to random markers. Markers with effect are highlighted with dashed lines. (**b**) Significant QTCs found by QTCAT. (**c**) LASSO selection frequency for each marker during the 50 iterations of QTCAT. (**d**) Manhattan plot of the LMM analysis. The horizontal dashed line depicts the significance threshold when controlling the multiple testing with FWER, whereas the red markers are significantly associated when controlling with FDR. (**e**) The Manhattan plot of the LMM* analysis. GRM was estimated without markers on the chromosome of the actual testing position. The results are shown as in (d).

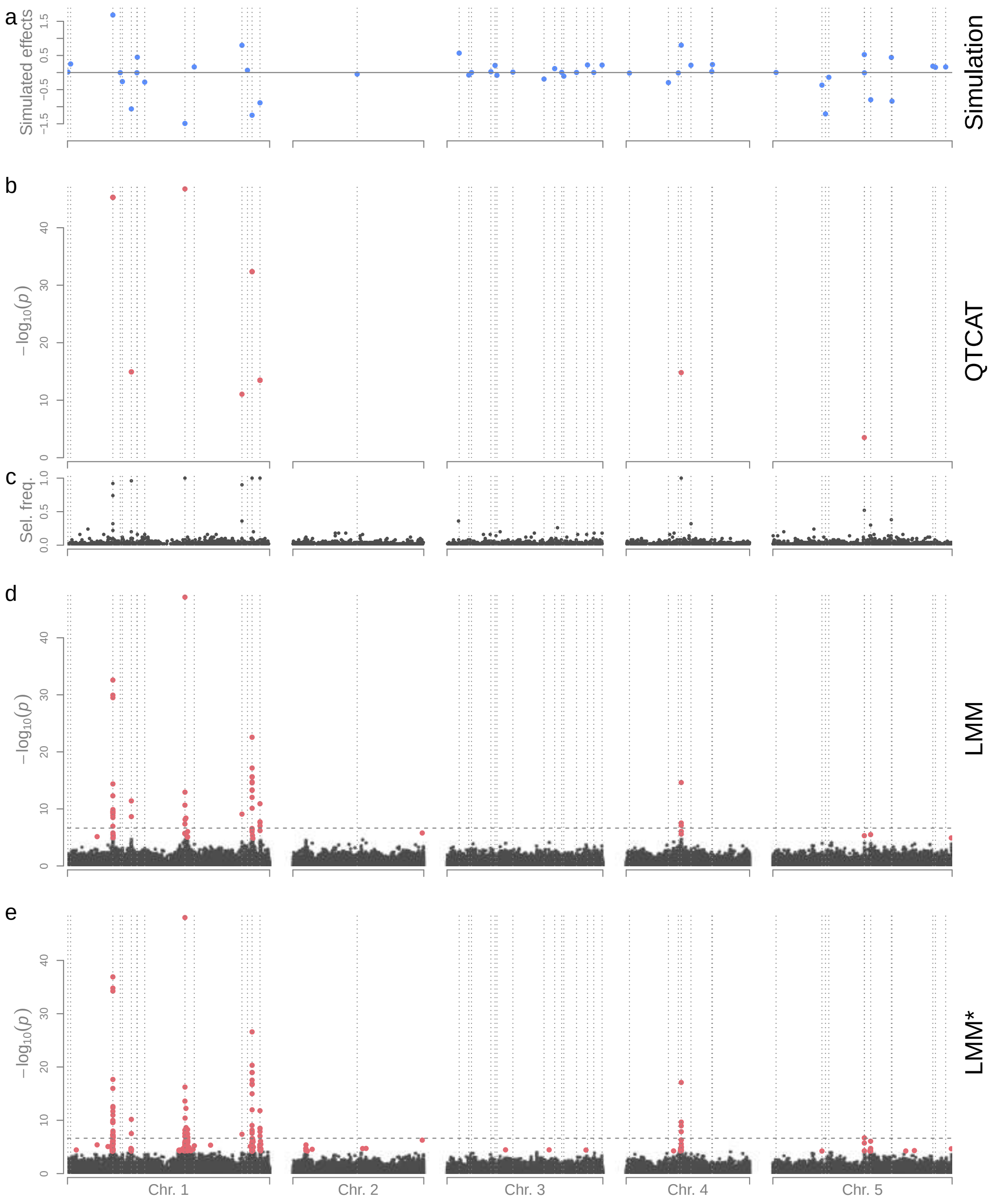

Supplementary Figure 73 Simulation of a GWA analysis based on a structured population with a heritability of 0.7 (run 73). (**a**) Simulation of 50 effects randomly drawn from a Gamma distribution and assigned to random markers. Markers with effect are highlighted with dashed lines. (**b**) Significant QTCs found by QTCAT. (**c**) LASSO selection frequency for each marker during the 50 iterations of QTCAT. (**d**) Manhattan plot of the LMM analysis. The horizontal dashed line depicts the significance threshold when controlling the multiple testing with FWER, whereas the red markers are significantly associated when controlling with FDR. (**e**) The Manhattan plot of the LMM* analysis. GRM was estimated without markers on the chromosome of the actual testing position. The results are shown as in (d).

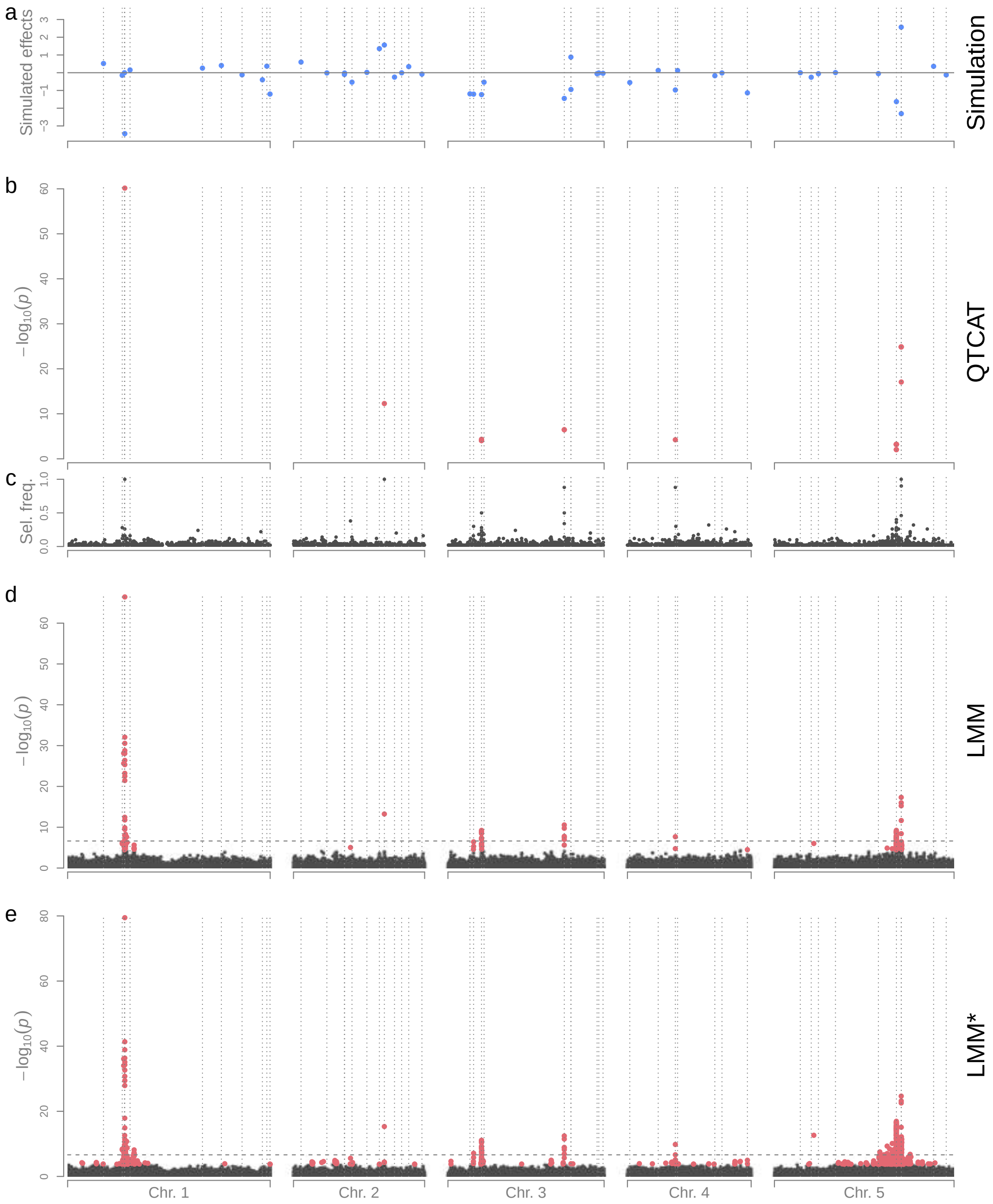

Supplementary Figure 74 Simulation of a GWA analysis based on a structured population with a heritability of 0.7 (run 74). (**a**) Simulation of 50 effects randomly drawn from a Gamma distribution and assigned to random markers. Markers with effect are highlighted with dashed lines. (**b**) Significant QTCs found by QTCAT. (**c**) LASSO selection frequency for each marker during the 50 iterations of QTCAT. (**d**) Manhattan plot of the LMM analysis. The horizontal dashed line depicts the significance threshold when controlling the multiple testing with FWER, whereas the red markers are significantly associated when controlling with FDR. (**e**) The Manhattan plot of the LMM* analysis. GRM was estimated without markers on the chromosome of the actual testing position. The results are shown as in (d).

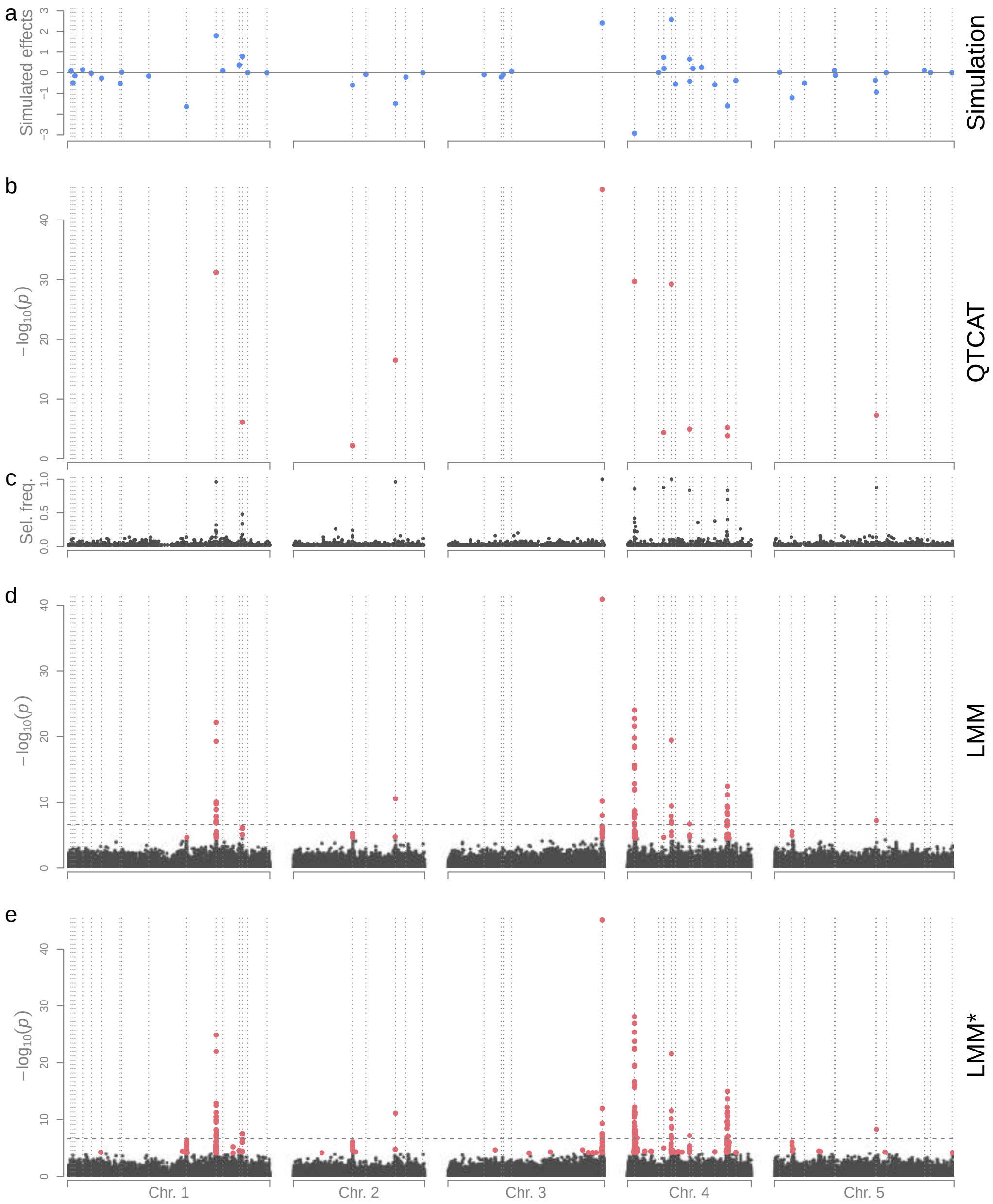

Supplementary Figure 75 Simulation of a GWA analysis based on a structured population with a heritability of 0.7 (run 75). (**a**) Simulation of 50 effects randomly drawn from a Gamma distribution and assigned to random markers. Markers with effect are highlighted with dashed lines. (**b**) Significant QTCs found by QTCAT. (**c**) LASSO selection frequency for each marker during the 50 iterations of QTCAT. (**d**) Manhattan plot of the LMM analysis. The horizontal dashed line depicts the significance threshold when controlling the multiple testing with FWER, whereas the red markers are significantly associated when controlling with FDR. (**e**) The Manhattan plot of the LMM* analysis. GRM was estimated without markers on the chromosome of the actual testing position. The results are shown as in (d).

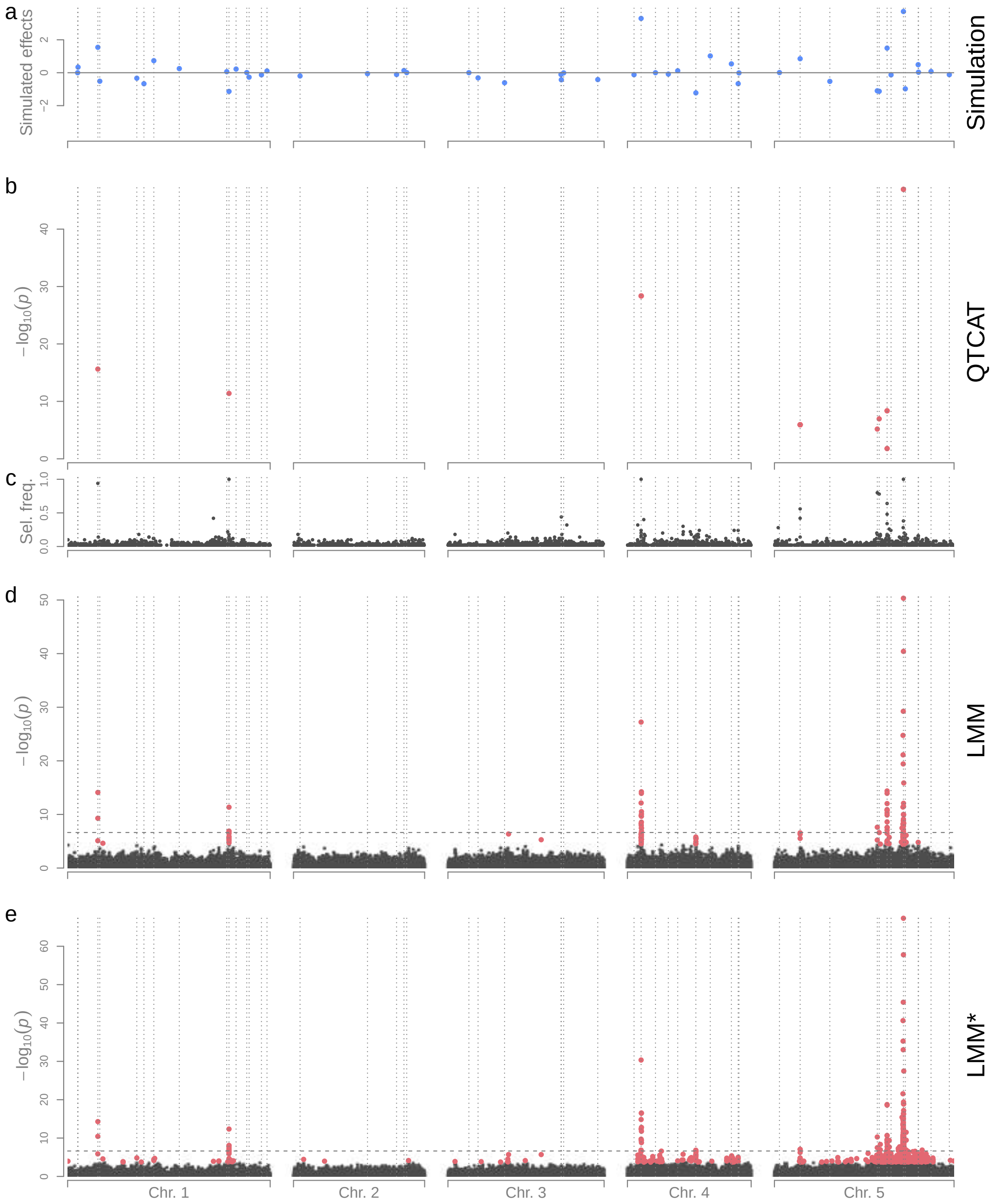

Supplementary Figure 76 Simulation of a GWA analysis based on a structured population with a heritability of 0.7 (run 76). (**a**) Simulation of 50 effects randomly drawn from a Gamma distribution and assigned to random markers. Markers with effect are highlighted with dashed lines. (**b**) Significant QTCs found by QTCAT. (**c**) LASSO selection frequency for each marker during the 50 iterations of QTCAT. (**d**) Manhattan plot of the LMM analysis. The horizontal dashed line depicts the significance threshold when controlling the multiple testing with FWER, whereas the red markers are significantly associated when controlling with FDR. (**e**) The Manhattan plot of the LMM* analysis. GRM was estimated without markers on the chromosome of the actual testing position. The results are shown as in (d).

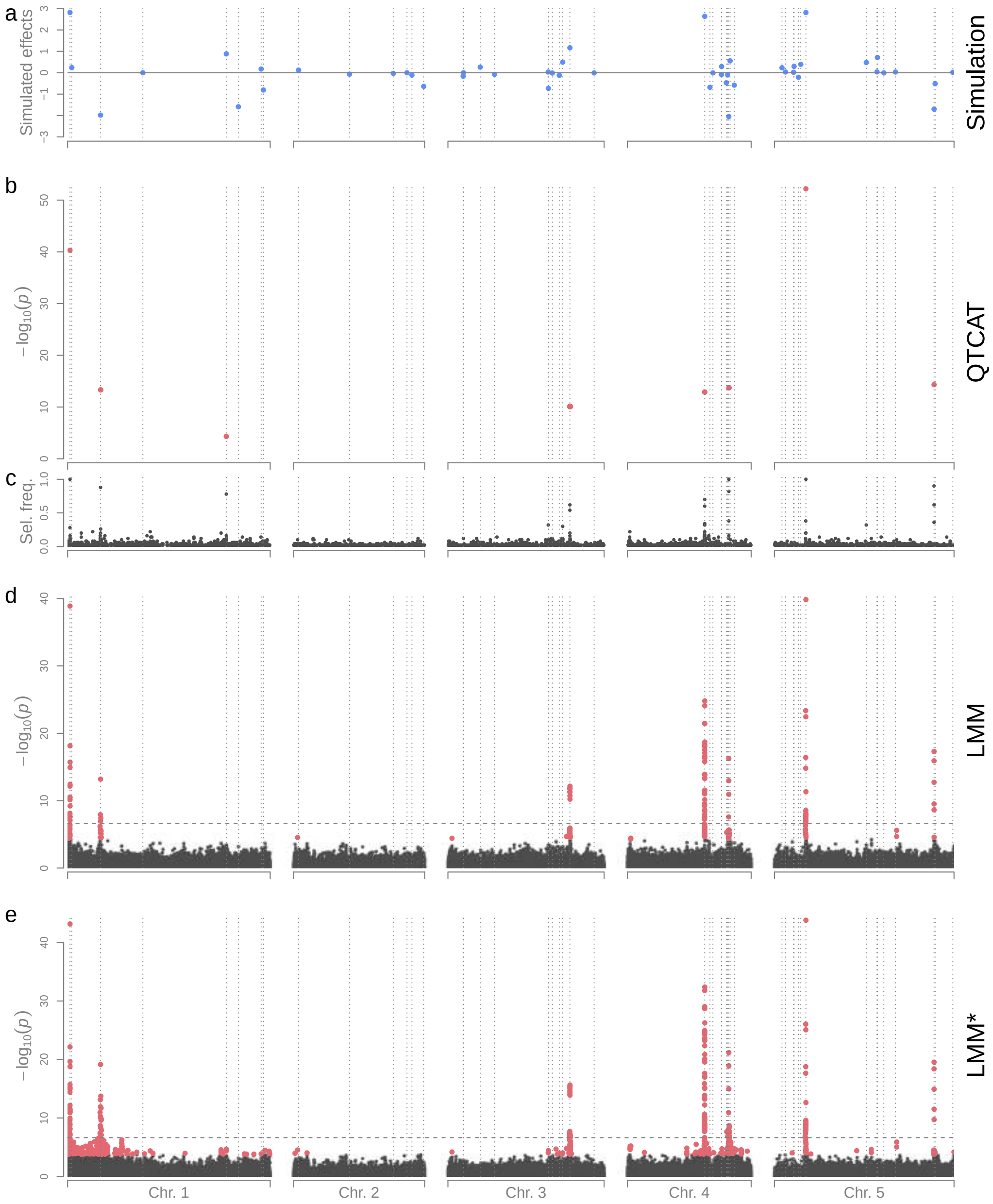

Supplementary Figure 77 Simulation of a GWA analysis based on a structured population with a heritability of 0.7 (run 77). (**a**) Simulation of 50 effects randomly drawn from a Gamma distribution and assigned to random markers. Markers with effect are highlighted with dashed lines. (**b**) Significant QTCs found by QTCAT. (**c**) LASSO selection frequency for each marker during the 50 iterations of QTCAT. (**d**) Manhattan plot of the LMM analysis. The horizontal dashed line depicts the significance threshold when controlling the multiple testing with FWER, whereas the red markers are significantly associated when controlling with FDR. (**e**) The Manhattan plot of the LMM* analysis. GRM was estimated without markers on the chromosome of the actual testing position. The results are shown as in (d).

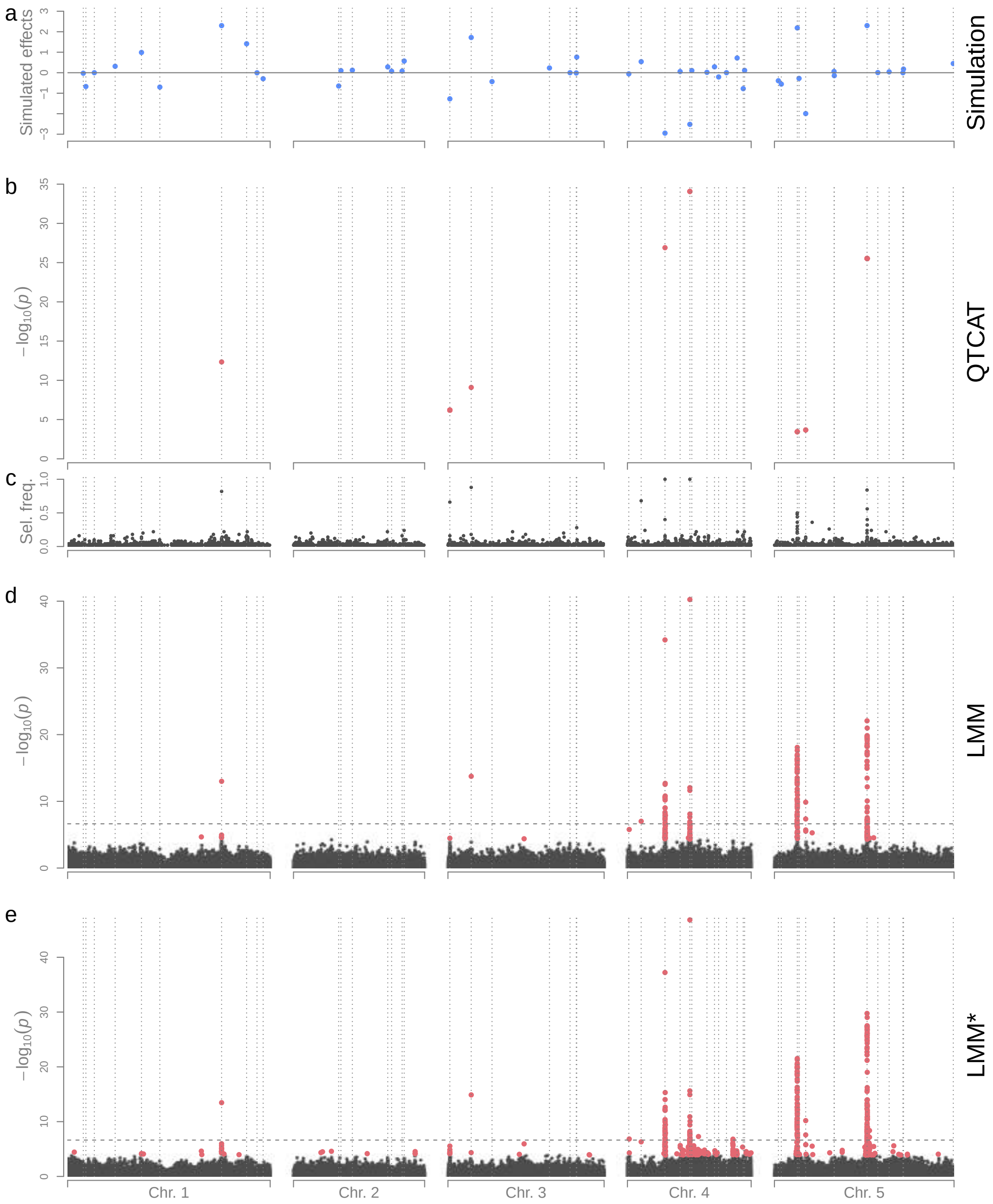

Supplementary Figure 78 Simulation of a GWA analysis based on a structured population with a heritability of 0.7 (run 78). (**a**) Simulation of 50 effects randomly drawn from a Gamma distribution and assigned to random markers. Markers with effect are highlighted with dashed lines. (**b**) Significant QTCs found by QTCAT. (**c**) LASSO selection frequency for each marker during the 50 iterations of QTCAT. (**d**) Manhattan plot of the LMM analysis. The horizontal dashed line depicts the significance threshold when controlling the multiple testing with FWER, whereas the red markers are significantly associated when controlling with FDR. (**e**) The Manhattan plot of the LMM* analysis. GRM was estimated without markers on the chromosome of the actual testing position. The results are shown as in (d).

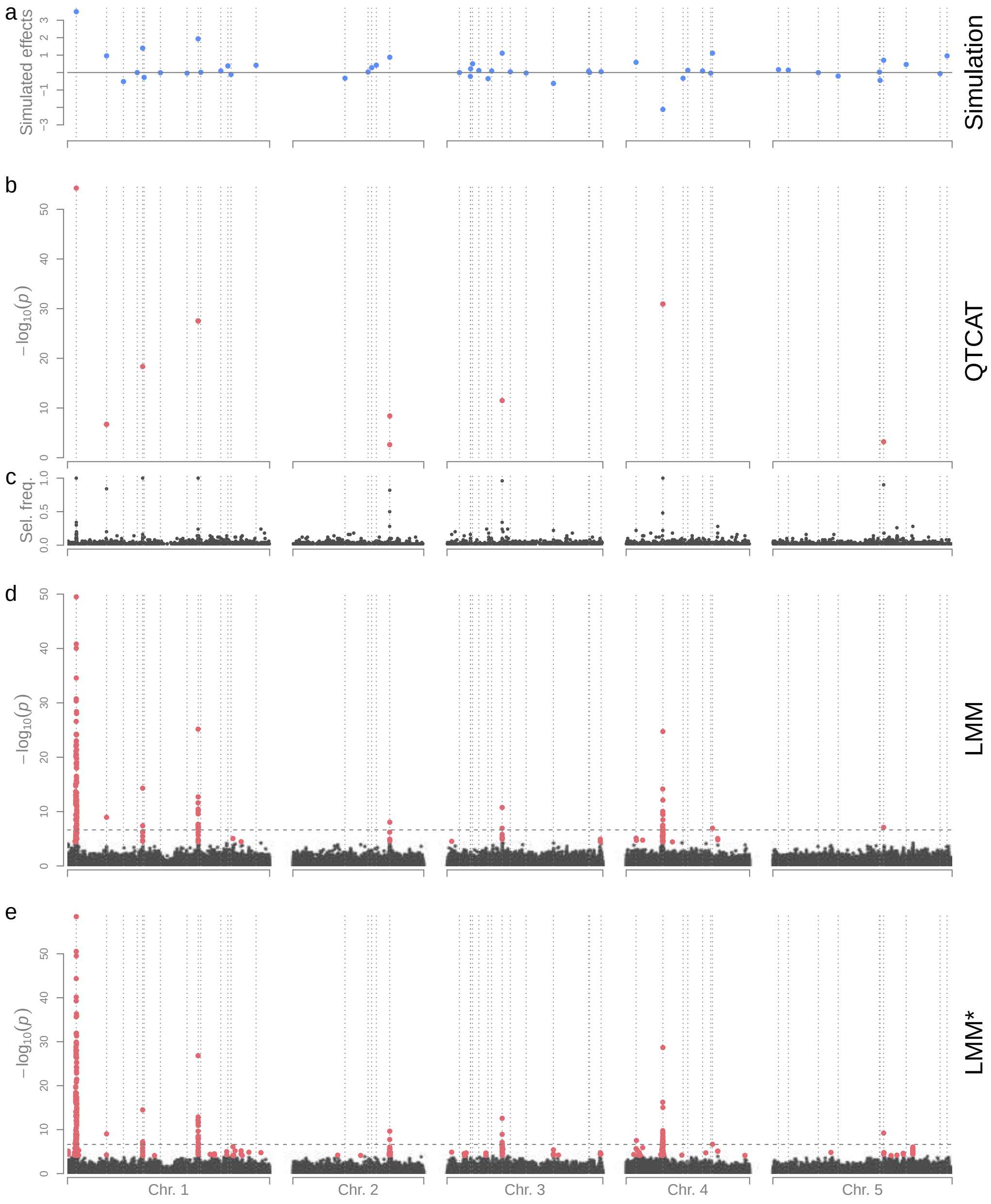

Supplementary Figure 79 Simulation of a GWA analysis based on a structured population with a heritability of 0.7 (run 79). (**a**) Simulation of 50 effects randomly drawn from a Gamma distribution and assigned to random markers. Markers with effect are highlighted with dashed lines. (**b**) Significant QTCs found by QTCAT. (**c**) LASSO selection frequency for each marker during the 50 iterations of QTCAT. (**d**) Manhattan plot of the LMM analysis. The horizontal dashed line depicts the significance threshold when controlling the multiple testing with FWER, whereas the red markers are significantly associated when controlling with FDR. (**e**) The Manhattan plot of the LMM* analysis. GRM was estimated without markers on the chromosome of the actual testing position. The results are shown as in (d).

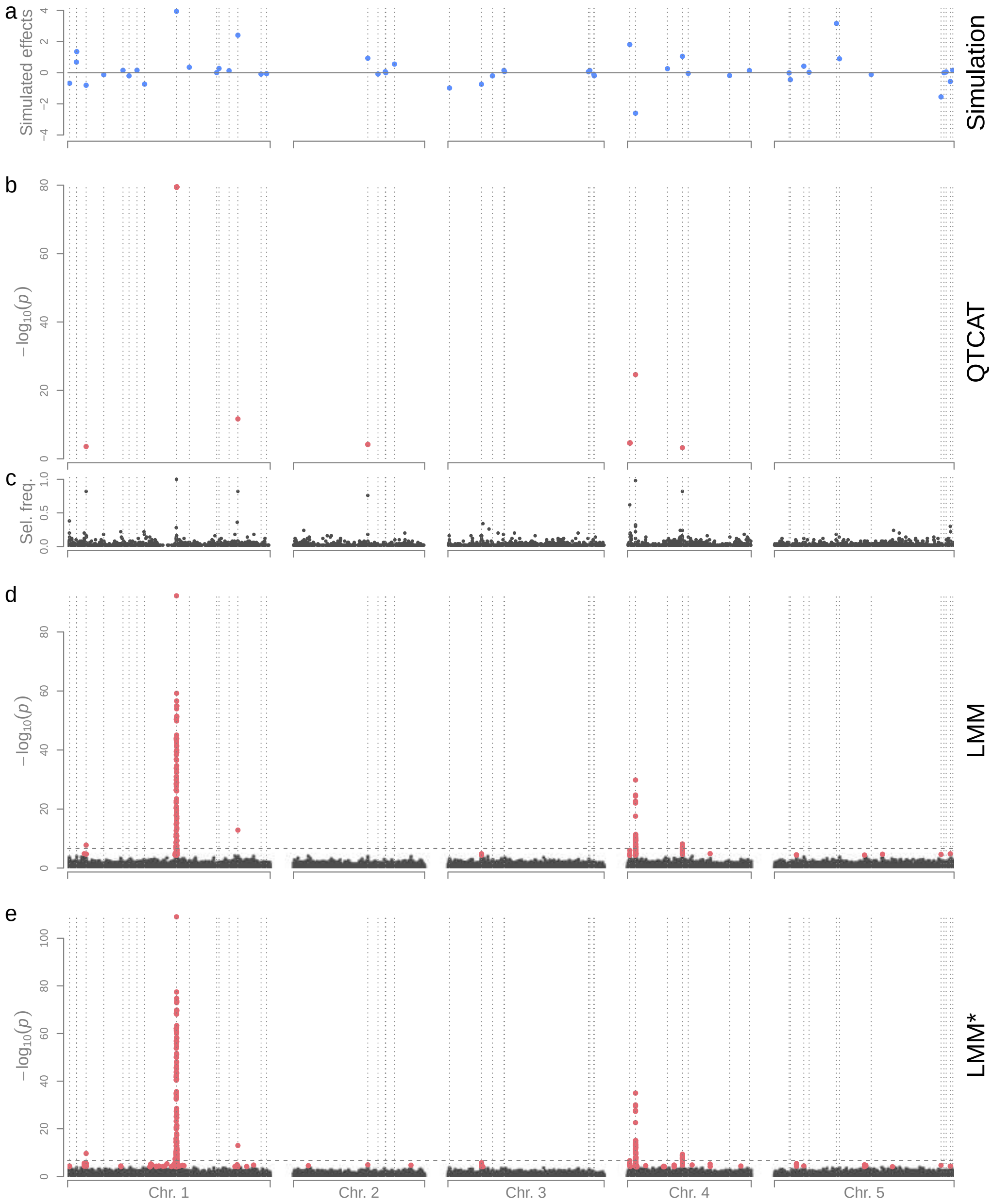

Supplementary Figure 80 Simulation of a GWA analysis based on a structured population with a heritability of 0.7 (run 80). (**a**) Simulation of 50 effects randomly drawn from a Gamma distribution and assigned to random markers. Markers with effect are highlighted with dashed lines. (**b**) Significant QTCs found by QTCAT. (**c**) LASSO selection frequency for each marker during the 50 iterations of QTCAT. (**d**) Manhattan plot of the LMM analysis. The horizontal dashed line depicts the significance threshold when controlling the multiple testing with FWER, whereas the red markers are significantly associated when controlling with FDR. (**e**) The Manhattan plot of the LMM* analysis. GRM was estimated without markers on the chromosome of the actual testing position. The results are shown as in (d).

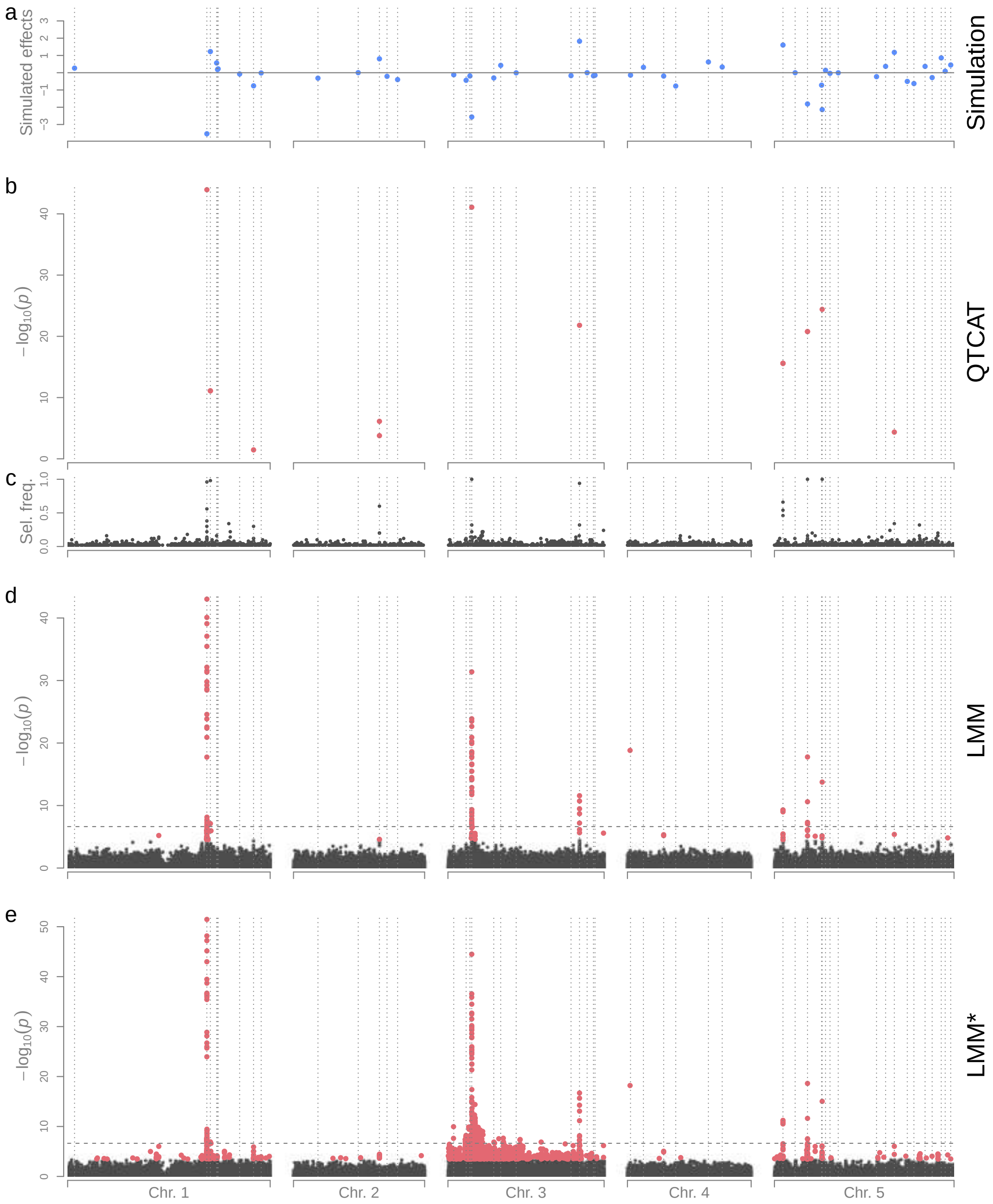

Supplementary Figure 81 Simulation of a GWA analysis based on a structured population with a heritability of 0.7 (run 81). (**a**) Simulation of 50 effects randomly drawn from a Gamma distribution and assigned to random markers. Markers with effect are highlighted with dashed lines. (**b**) Significant QTCs found by QTCAT. (**c**) LASSO selection frequency for each marker during the 50 iterations of QTCAT. (**d**) Manhattan plot of the LMM analysis. The horizontal dashed line depicts the significance threshold when controlling the multiple testing with FWER, whereas the red markers are significantly associated when controlling with FDR. (**e**) The Manhattan plot of the LMM* analysis. GRM was estimated without markers on the chromosome of the actual testing position. The results are shown as in (d).

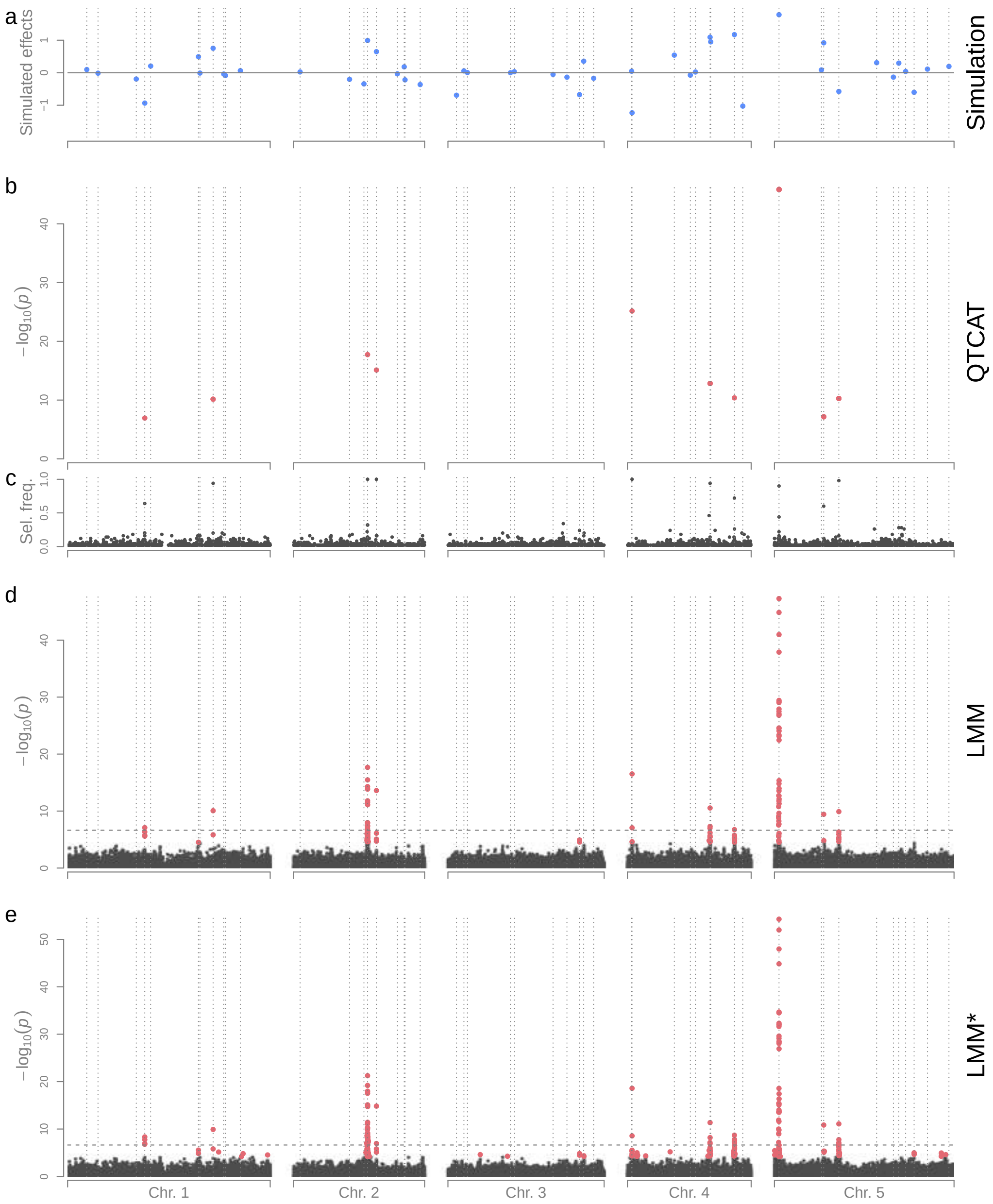

Supplementary Figure 82 Simulation of a GWA analysis based on a structured population with a heritability of 0.7 (run 82). (**a**) Simulation of 50 effects randomly drawn from a Gamma distribution and assigned to random markers. Markers with effect are highlighted with dashed lines. (**b**) Significant QTCs found by QTCAT. (**c**) LASSO selection frequency for each marker during the 50 iterations of QTCAT. (**d**) Manhattan plot of the LMM analysis. The horizontal dashed line depicts the significance threshold when controlling the multiple testing with FWER, whereas the red markers are significantly associated when controlling with FDR. (**e**) The Manhattan plot of the LMM* analysis. GRM was estimated without markers on the chromosome of the actual testing position. The results are shown as in (d).

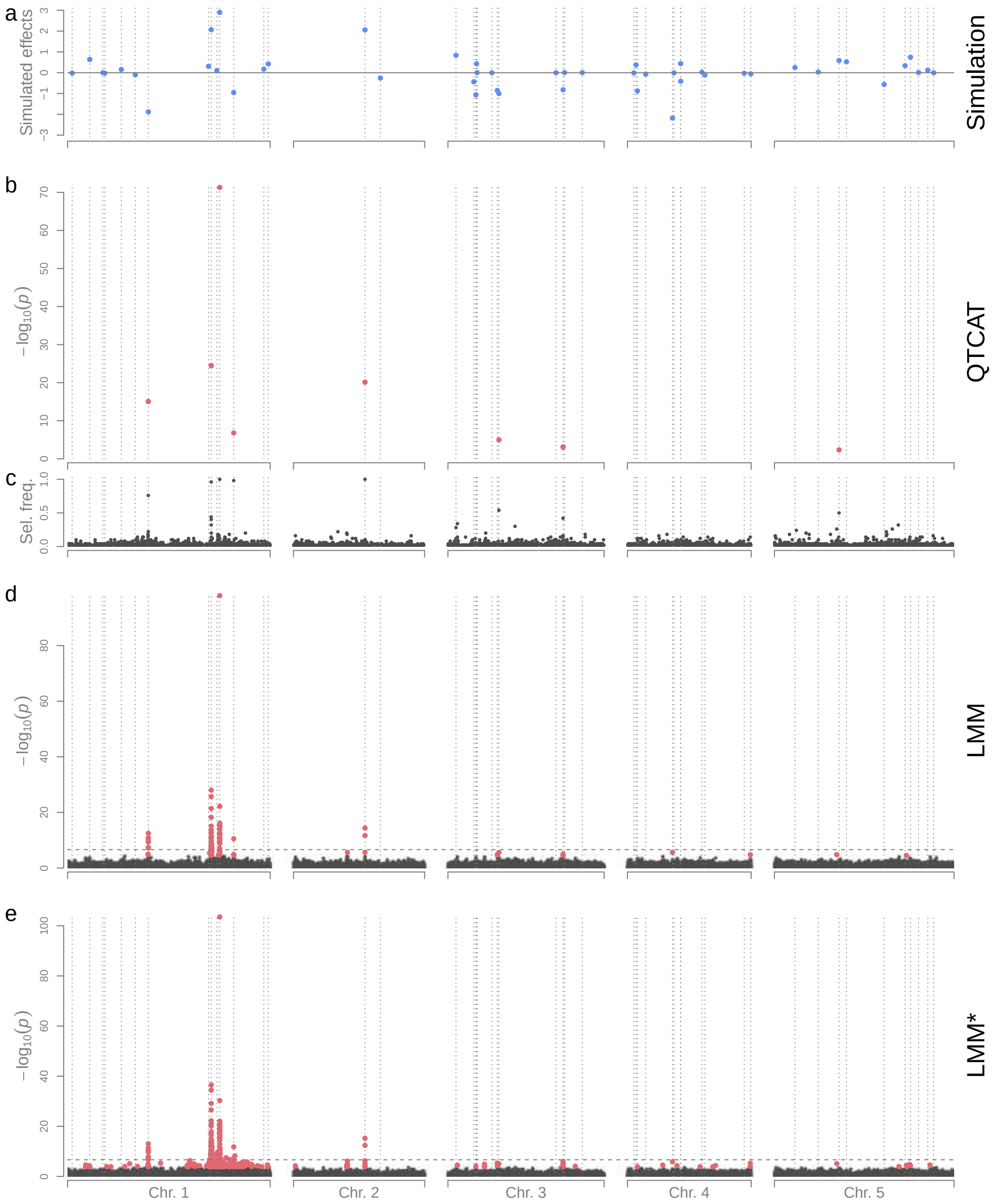

Supplementary Figure 83 Simulation of a GWA analysis based on a structured population with a heritability of 0.7 (run 83). (**a**) Simulation of 50 effects randomly drawn from a Gamma distribution and assigned to random markers. Markers with effect are highlighted with dashed lines. (**b**) Significant QTCs found by QTCAT. (**c**) LASSO selection frequency for each marker during the 50 iterations of QTCAT. (**d**) Manhattan plot of the LMM analysis. The horizontal dashed line depicts the significance threshold when controlling the multiple testing with FWER, whereas the red markers are significantly associated when controlling with FDR. (**e**) The Manhattan plot of the LMM* analysis. GRM was estimated without markers on the chromosome of the actual testing position. The results are shown as in (d).

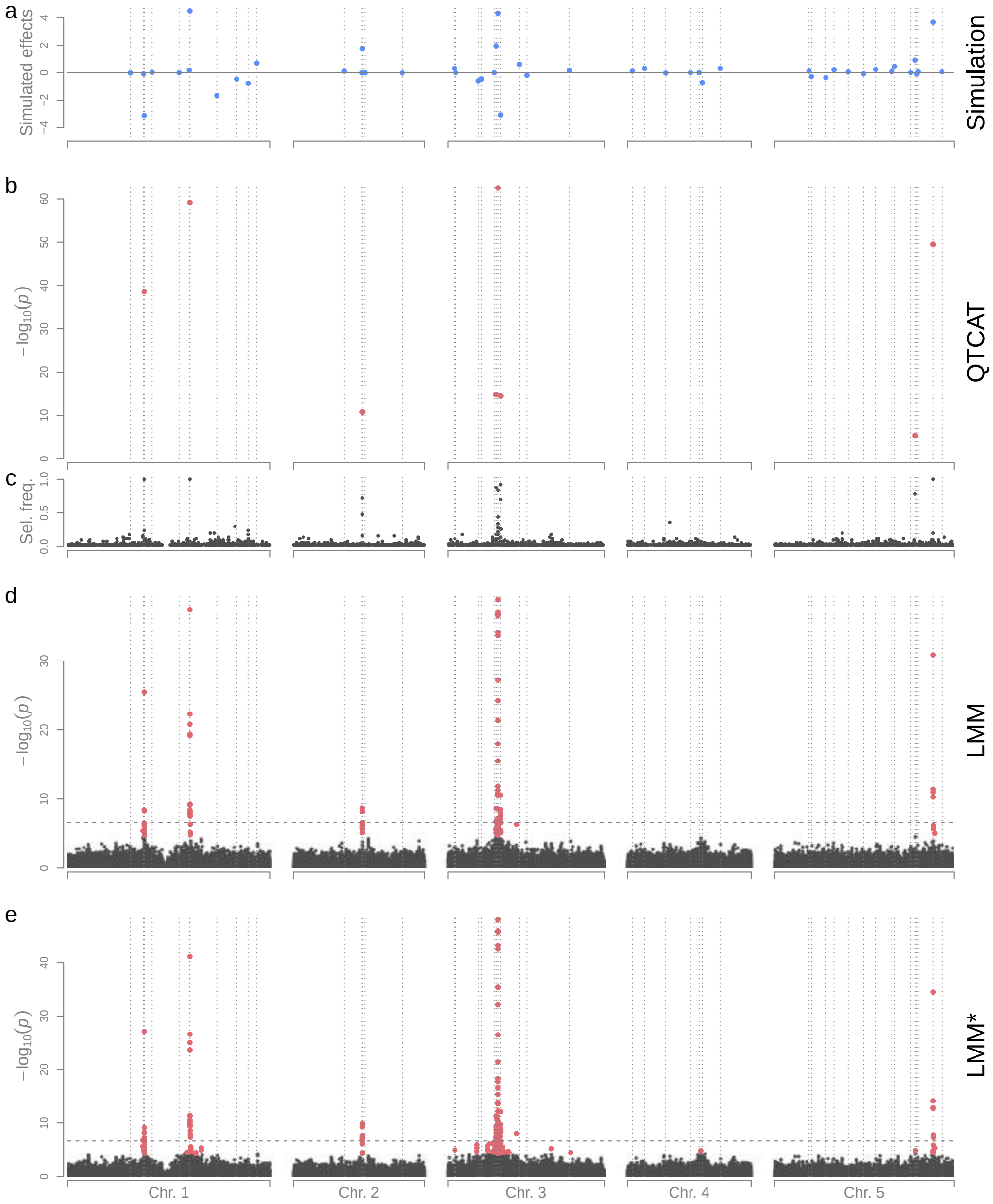

Supplementary Figure 84 Simulation of a GWA analysis based on a structured population with a heritability of 0.7 (run 84). (**a**) Simulation of 50 effects randomly drawn from a Gamma distribution and assigned to random markers. Markers with effect are highlighted with dashed lines. (**b**) Significant QTCs found by QTCAT. (**c**) LASSO selection frequency for each marker during the 50 iterations of QTCAT. (**d**) Manhattan plot of the LMM analysis. The horizontal dashed line depicts the significance threshold when controlling the multiple testing with FWER, whereas the red markers are significantly associated when controlling with FDR. (**e**) The Manhattan plot of the LMM* analysis. GRM was estimated without markers on the chromosome of the actual testing position. The results are shown as in (d).

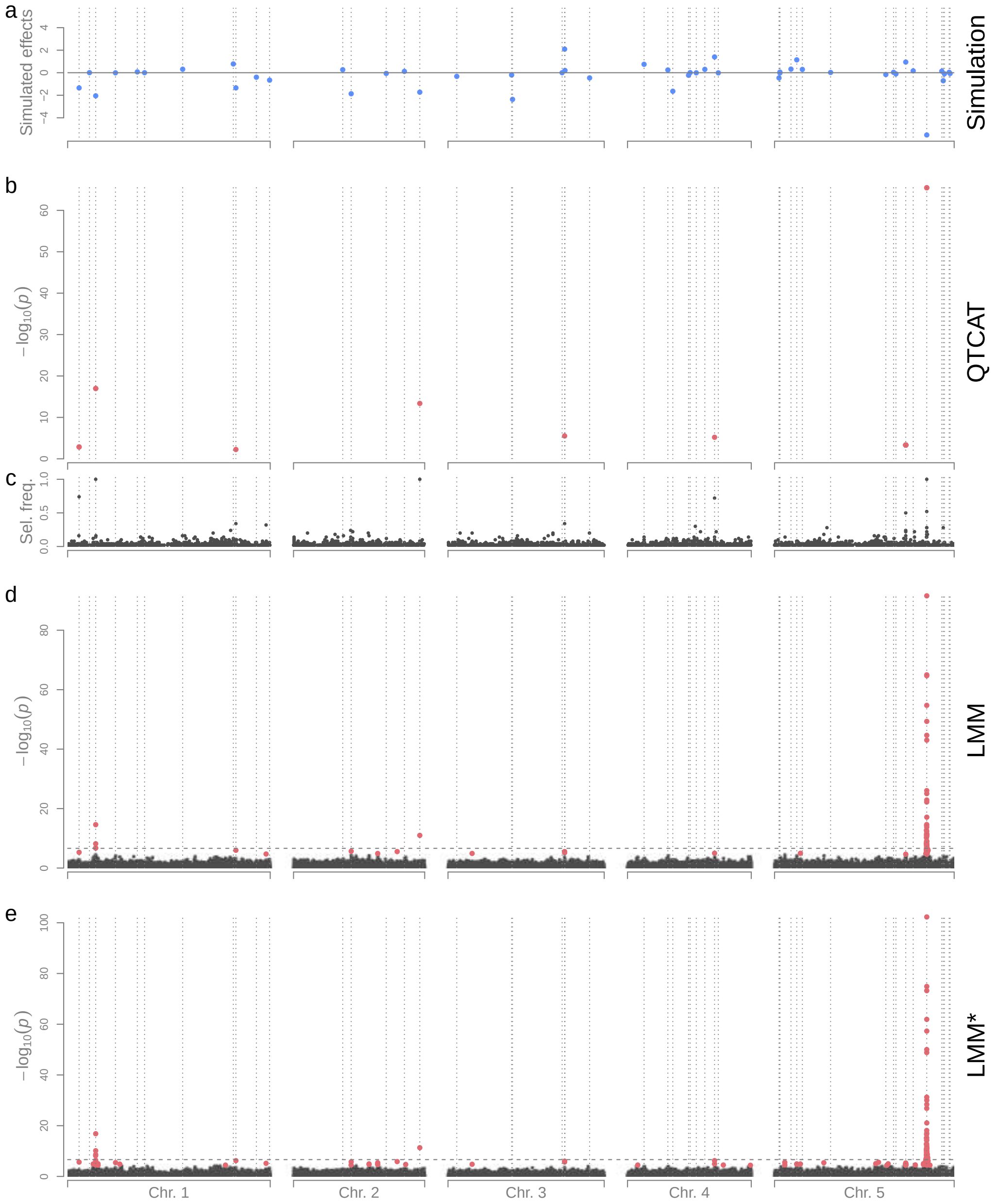

Supplementary Figure 85 Simulation of a GWA analysis based on a structured population with a heritability of 0.7 (run 85). (**a**) Simulation of 50 effects randomly drawn from a Gamma distribution and assigned to random markers. Markers with effect are highlighted with dashed lines. (**b**) Significant QTCs found by QTCAT. (**c**) LASSO selection frequency for each marker during the 50 iterations of QTCAT. (**d**) Manhattan plot of the LMM analysis. The horizontal dashed line depicts the significance threshold when controlling the multiple testing with FWER, whereas the red markers are significantly associated when controlling with FDR. (**e**) The Manhattan plot of the LMM* analysis. GRM was estimated without markers on the chromosome of the actual testing position. The results are shown as in (d).

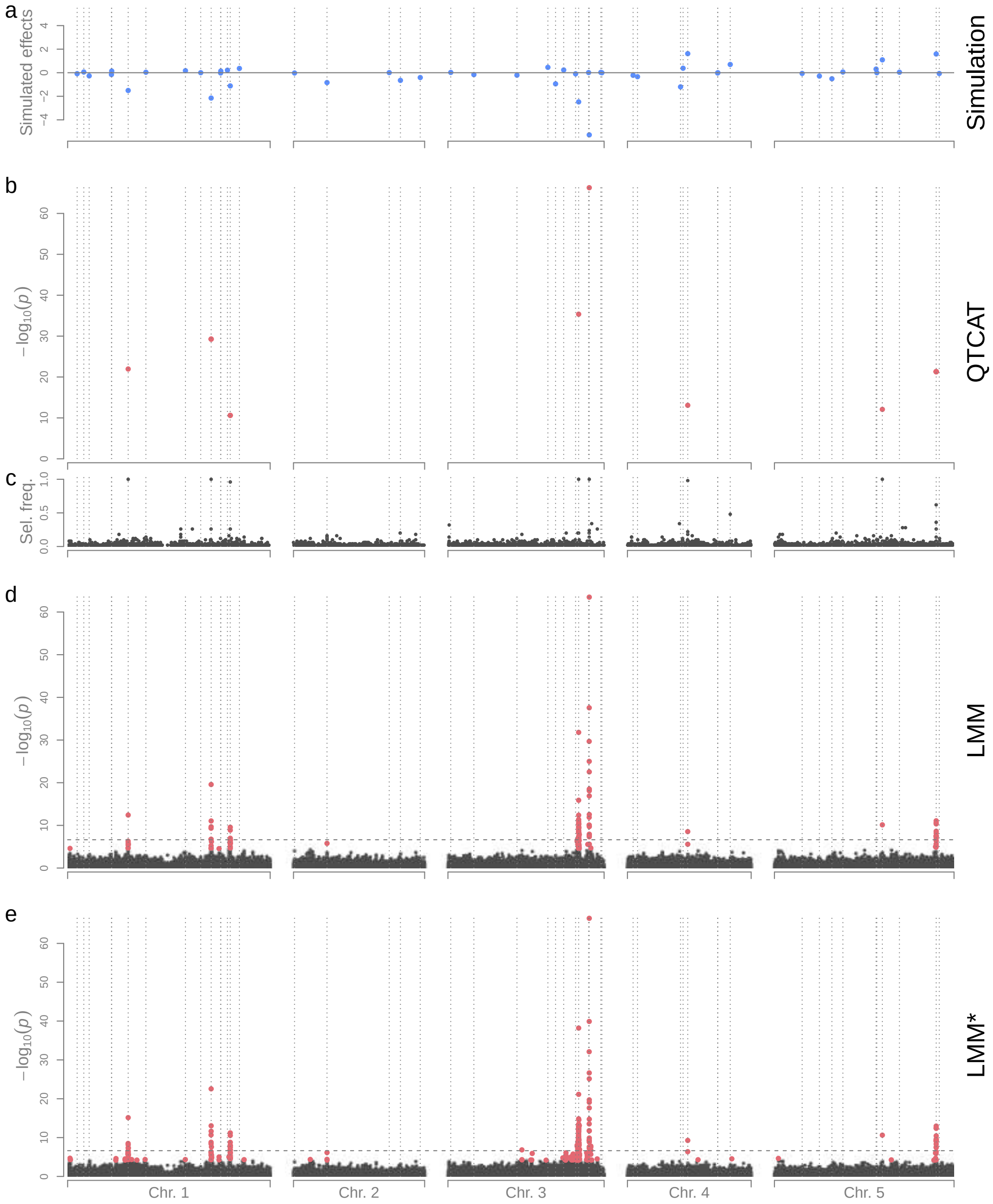

Supplementary Figure 86 Simulation of a GWA analysis based on a structured population with a heritability of 0.7 (run 86). (**a**) Simulation of 50 effects randomly drawn from a Gamma distribution and assigned to random markers. Markers with effect are highlighted with dashed lines. (**b**) Significant QTCs found by QTCAT. (**c**) LASSO selection frequency for each marker during the 50 iterations of QTCAT. (**d**) Manhattan plot of the LMM analysis. The horizontal dashed line depicts the significance threshold when controlling the multiple testing with FWER, whereas the red markers are significantly associated when controlling with FDR. (**e**) The Manhattan plot of the LMM* analysis. GRM was estimated without markers on the chromosome of the actual testing position. The results are shown as in (d).

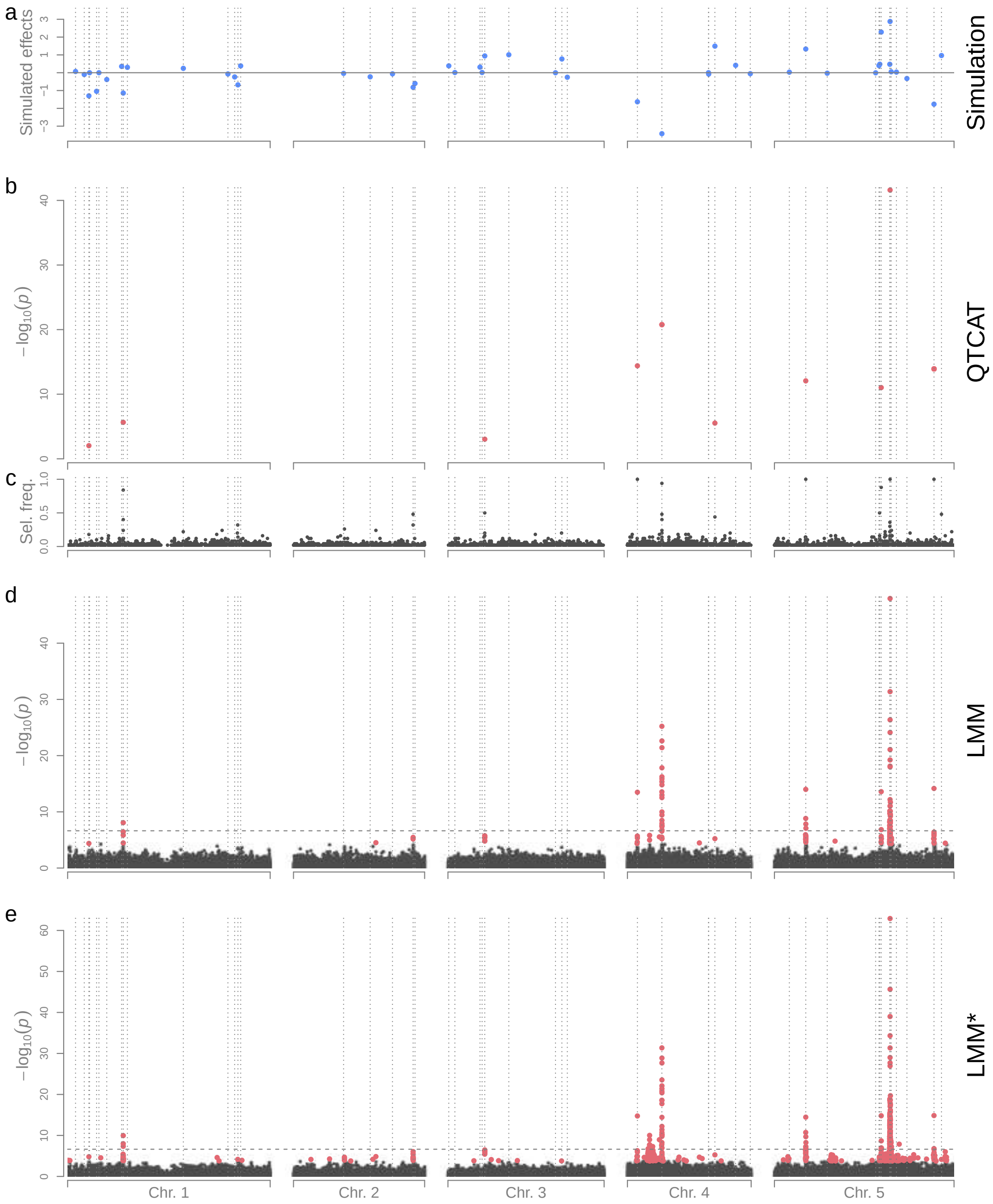

Supplementary Figure 87 Simulation of a GWA analysis based on a structured population with a heritability of 0.7 (run 87). (**a**) Simulation of 50 effects randomly drawn from a Gamma distribution and assigned to random markers. Markers with effect are highlighted with dashed lines. (**b**) Significant QTCs found by QTCAT. (**c**) LASSO selection frequency for each marker during the 50 iterations of QTCAT. (**d**) Manhattan plot of the LMM analysis. The horizontal dashed line depicts the significance threshold when controlling the multiple testing with FWER, whereas the red markers are significantly associated when controlling with FDR. (**e**) The Manhattan plot of the LMM* analysis. GRM was estimated without markers on the chromosome of the actual testing position. The results are shown as in (d).

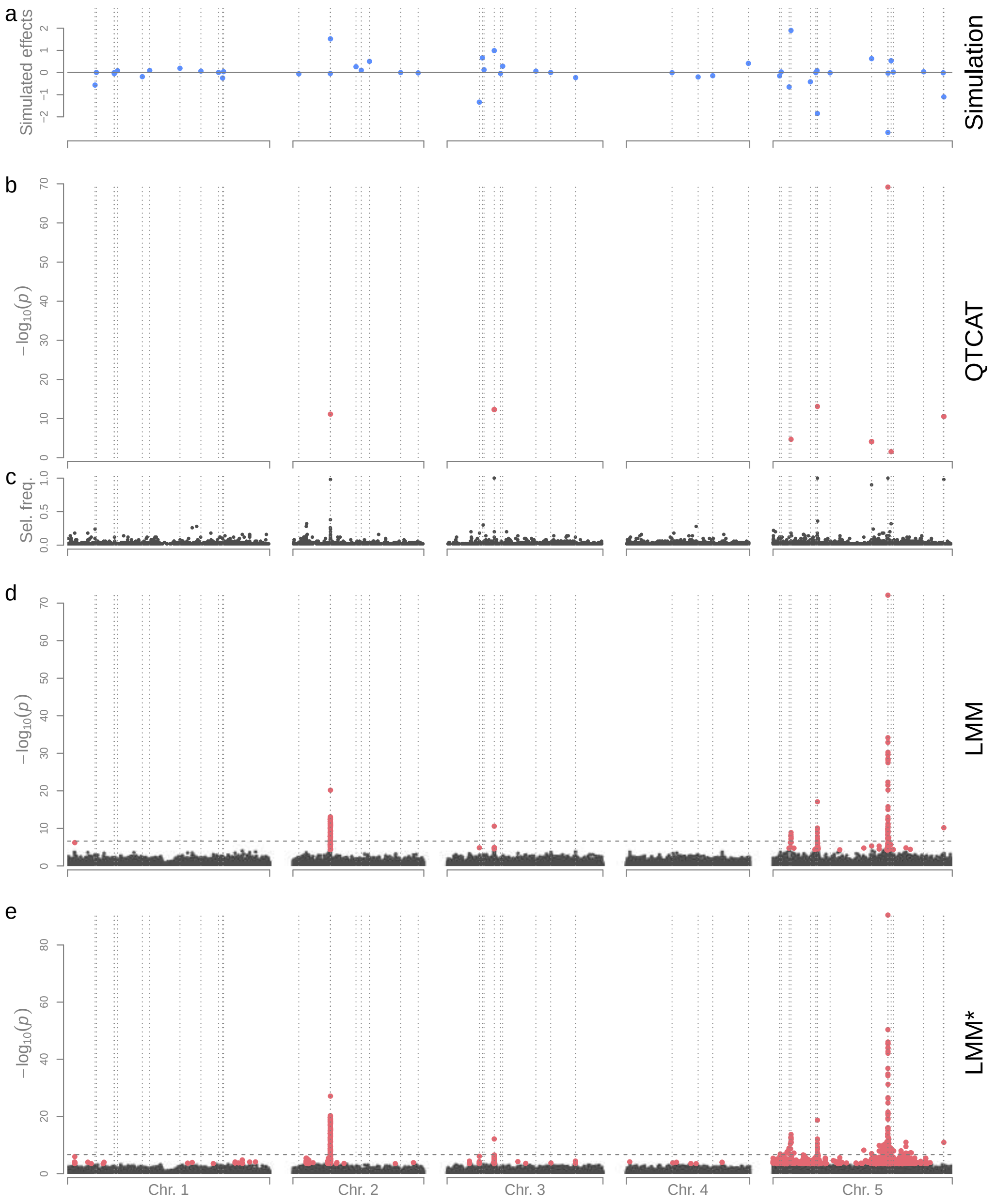

Supplementary Figure 88 Simulation of a GWA analysis based on a structured population with a heritability of 0.7 (run 88). (**a**) Simulation of 50 effects randomly drawn from a Gamma distribution and assigned to random markers. Markers with effect are highlighted with dashed lines. (**b**) Significant QTCs found by QTCAT. (**c**) LASSO selection frequency for each marker during the 50 iterations of QTCAT. (**d**) Manhattan plot of the LMM analysis. The horizontal dashed line depicts the significance threshold when controlling the multiple testing with FWER, whereas the red markers are significantly associated when controlling with FDR. (**e**) The Manhattan plot of the LMM* analysis. GRM was estimated without markers on the chromosome of the actual testing position. The results are shown as in (d).

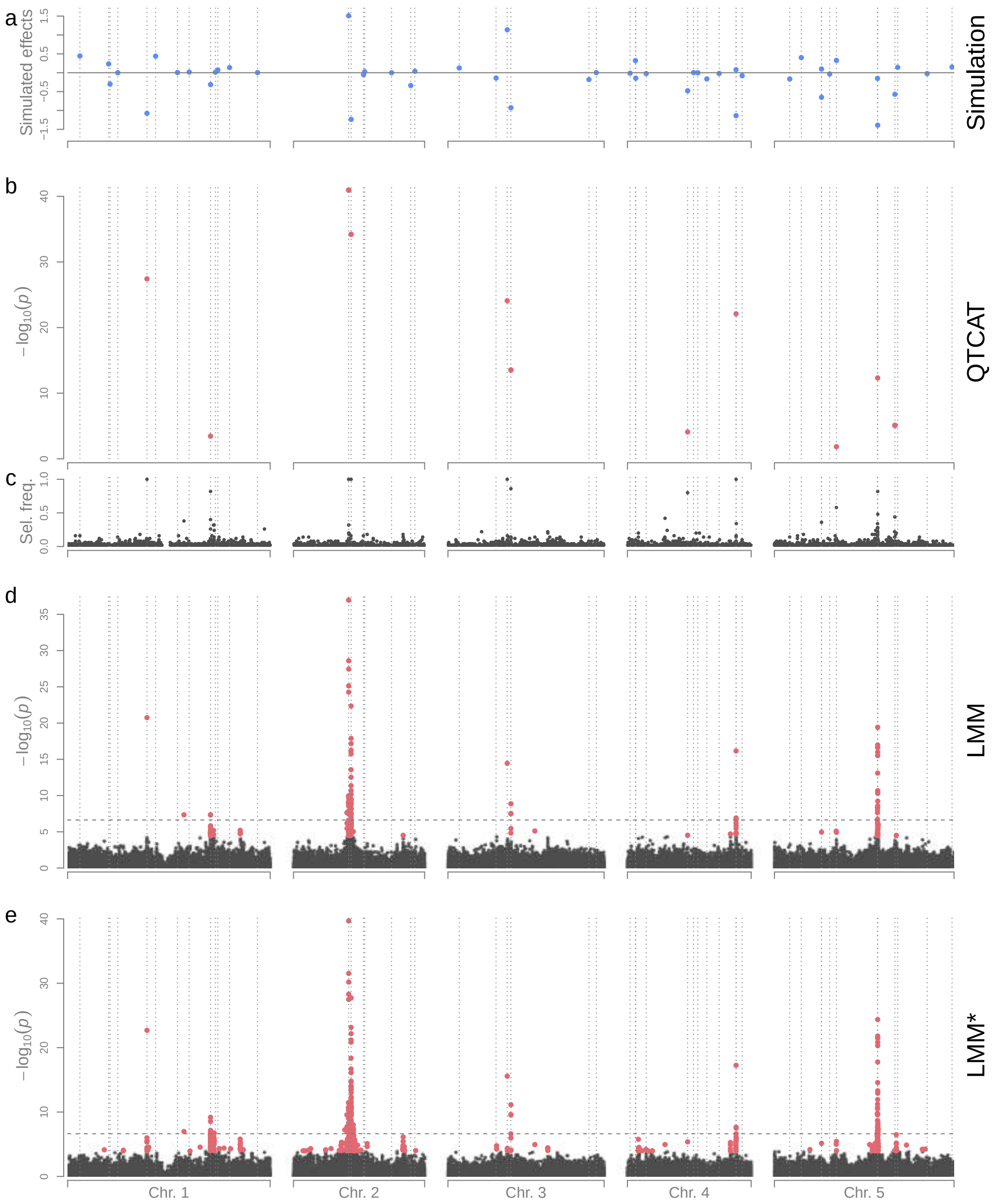

Supplementary Figure 89 Simulation of a GWA analysis based on a structured population with a heritability of 0.7 (run 89). (**a**) Simulation of 50 effects randomly drawn from a Gamma distribution and assigned to random markers. Markers with effect are highlighted with dashed lines. (**b**) Significant QTCs found by QTCAT. (**c**) LASSO selection frequency for each marker during the 50 iterations of QTCAT. (**d**) Manhattan plot of the LMM analysis. The horizontal dashed line depicts the significance threshold when controlling the multiple testing with FWER, whereas the red markers are significantly associated when controlling with FDR. (**e**) The Manhattan plot of the LMM* analysis. GRM was estimated without markers on the chromosome of the actual testing position. The results are shown as in (d).

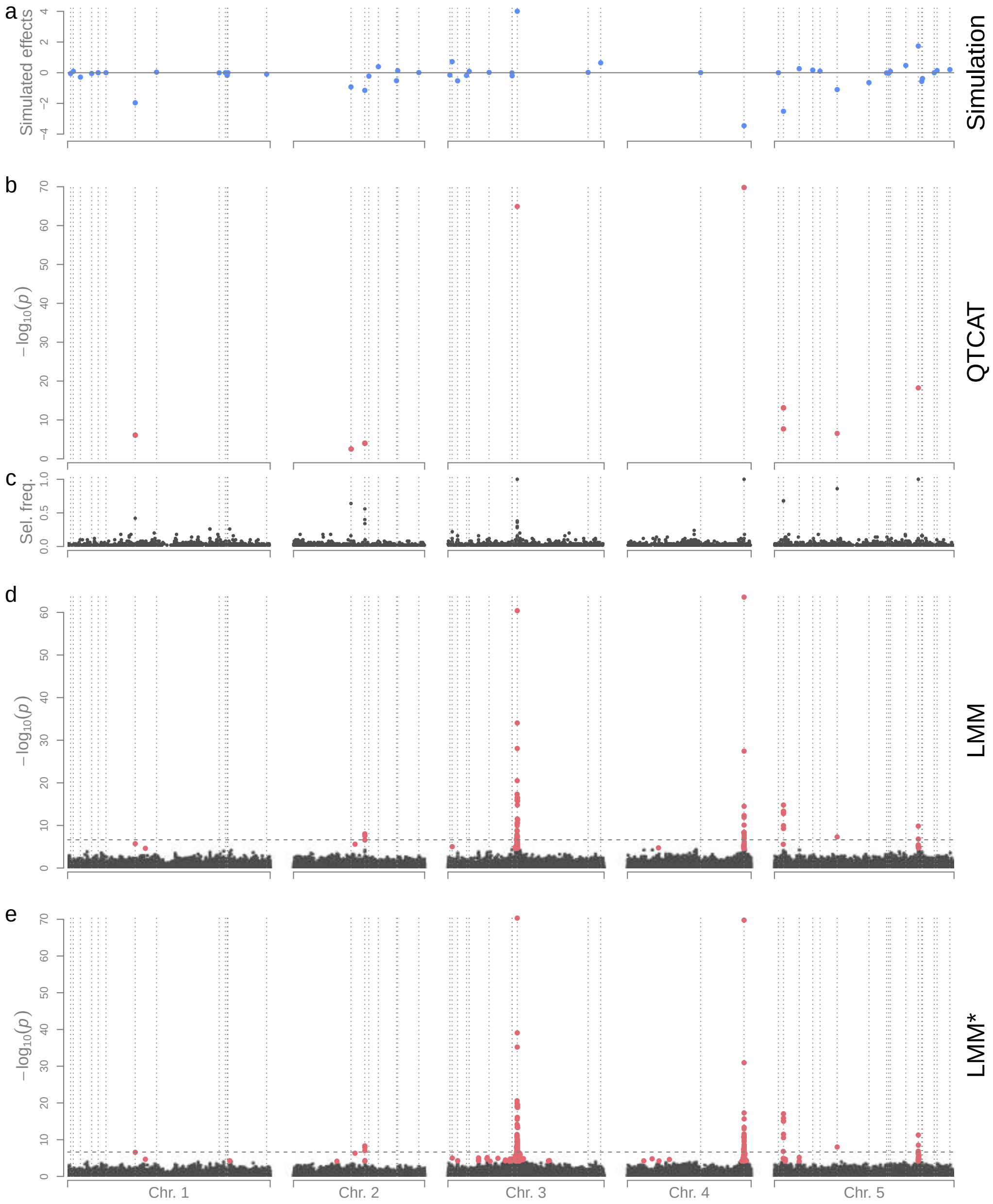

Supplementary Figure 90 Simulation of a GWA analysis based on a structured population with a heritability of 0.7 (run 90). (**a**) Simulation of 50 effects randomly drawn from a Gamma distribution and assigned to random markers. Markers with effect are highlighted with dashed lines. (**b**) Significant QTCs found by QTCAT. (**c**) LASSO selection frequency for each marker during the 50 iterations of QTCAT. (**d**) Manhattan plot of the LMM analysis. The horizontal dashed line depicts the significance threshold when controlling the multiple testing with FWER, whereas the red markers are significantly associated when controlling with FDR. (**e**) The Manhattan plot of the LMM* analysis. GRM was estimated without markers on the chromosome of the actual testing position. The results are shown as in (d).

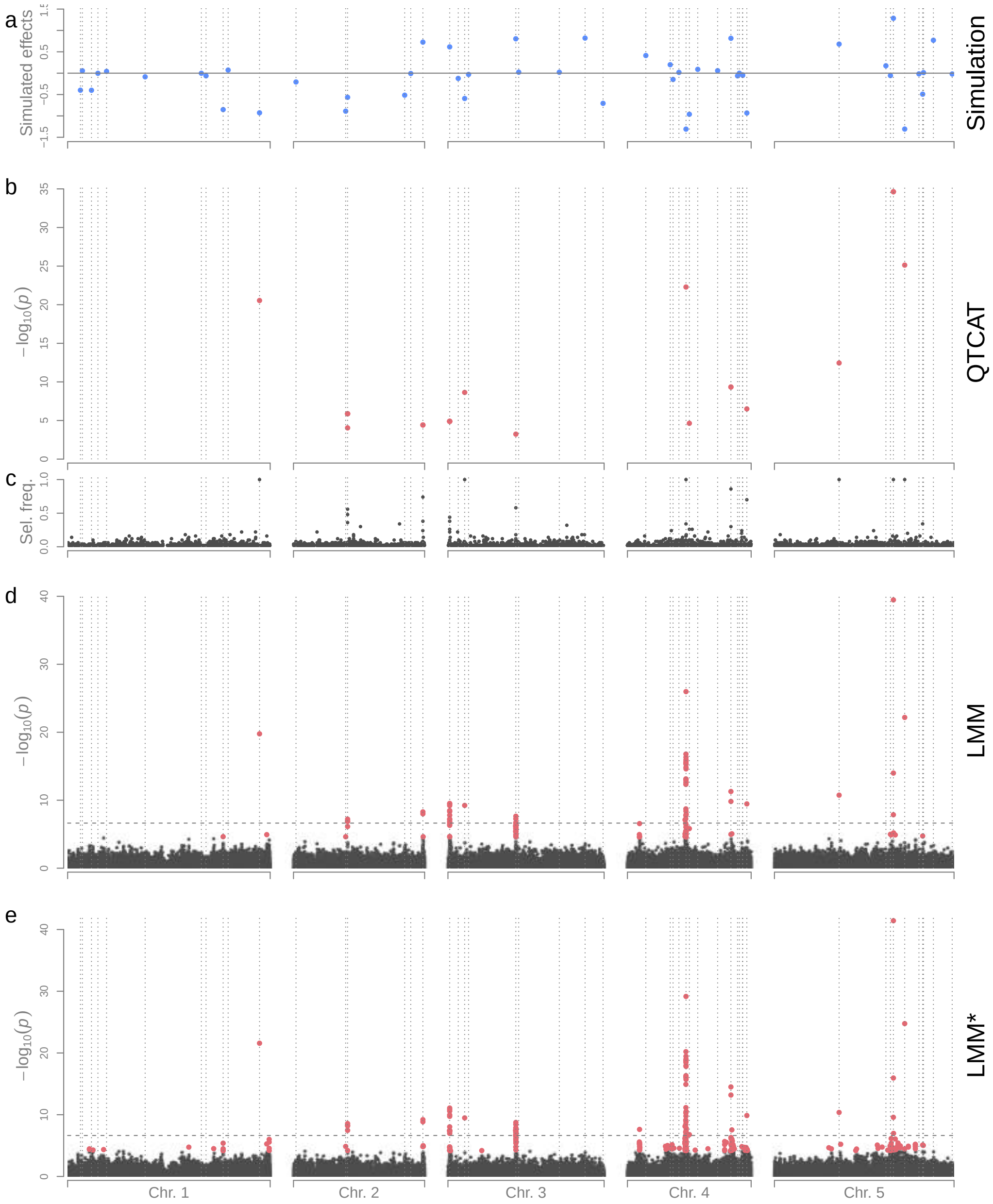

Supplementary Figure 91 Simulation of a GWA analysis based on a structured population with a heritability of 0.7 (run 91). (**a**) Simulation of 50 effects randomly drawn from a Gamma distribution and assigned to random markers. Markers with effect are highlighted with dashed lines. (**b**) Significant QTCs found by QTCAT. (**c**) LASSO selection frequency for each marker during the 50 iterations of QTCAT. (**d**) Manhattan plot of the LMM analysis. The horizontal dashed line depicts the significance threshold when controlling the multiple testing with FWER, whereas the red markers are significantly associated when controlling with FDR. (**e**) The Manhattan plot of the LMM* analysis. GRM was estimated without markers on the chromosome of the actual testing position. The results are shown as in (d).

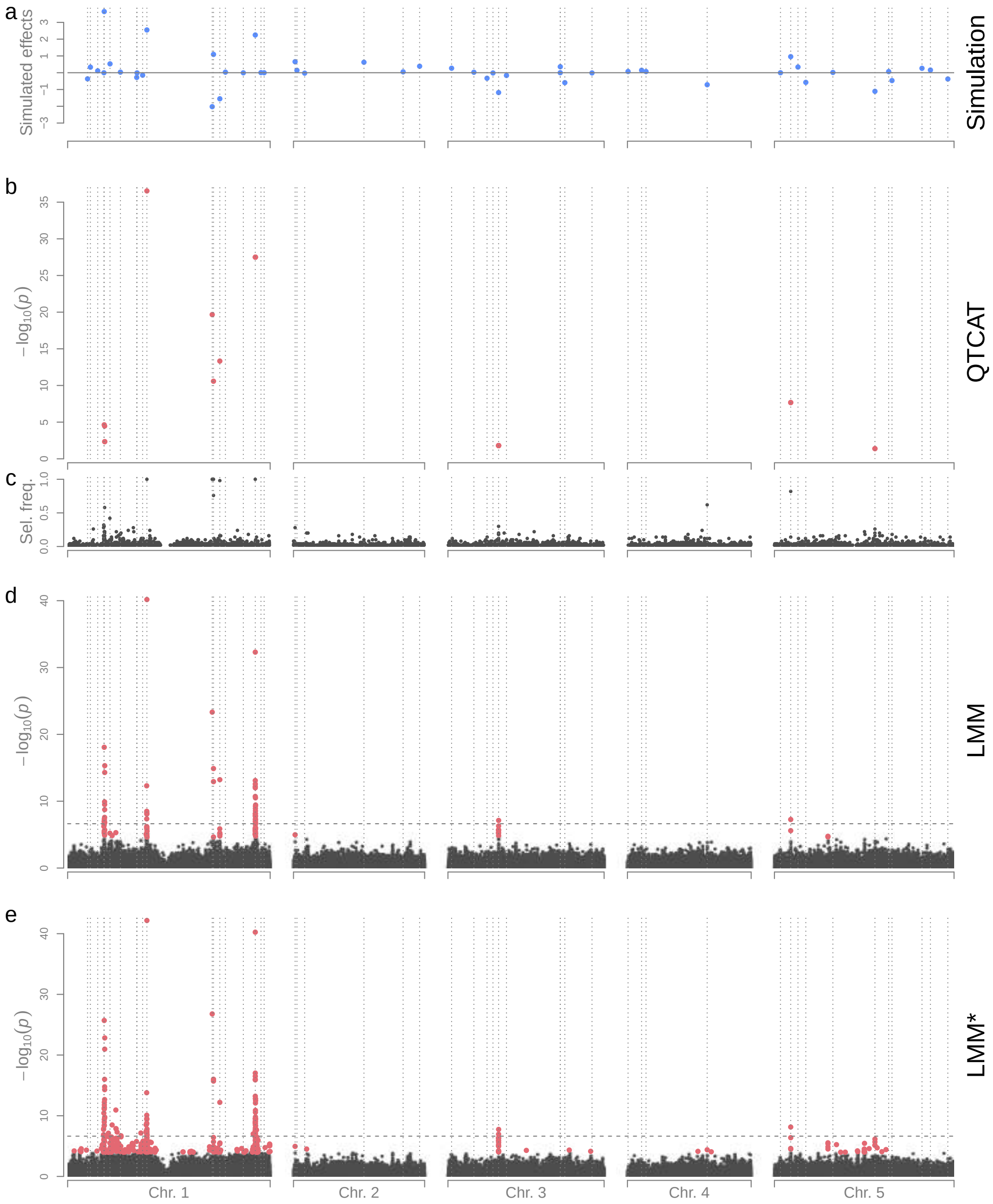

Supplementary Figure 92 Simulation of a GWA analysis based on a structured population with a heritability of 0.7 (run 92). (**a**) Simulation of 50 effects randomly drawn from a Gamma distribution and assigned to random markers. Markers with effect are highlighted with dashed lines. (**b**) Significant QTCs found by QTCAT. (**c**) LASSO selection frequency for each marker during the 50 iterations of QTCAT. (**d**) Manhattan plot of the LMM analysis. The horizontal dashed line depicts the significance threshold when controlling the multiple testing with FWER, whereas the red markers are significantly associated when controlling with FDR. (**e**) The Manhattan plot of the LMM* analysis. GRM was estimated without markers on the chromosome of the actual testing position. The results are shown as in (d).

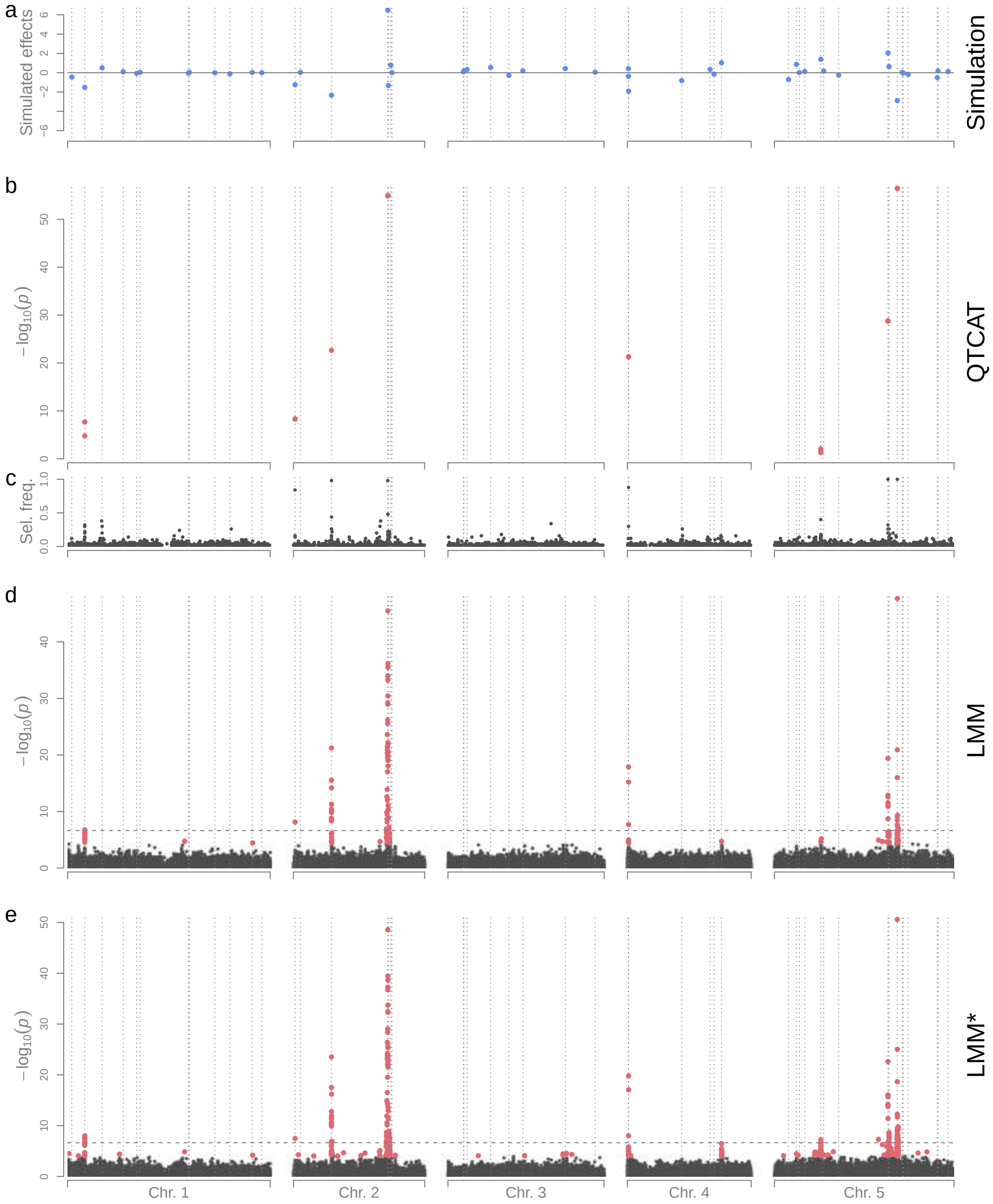

Supplementary Figure 93 Simulation of a GWA analysis based on a structured population with a heritability of 0.7 (run 93). (**a**) Simulation of 50 effects randomly drawn from a Gamma distribution and assigned to random markers. Markers with effect are highlighted with dashed lines. (**b**) Significant QTCs found by QTCAT. (**c**) LASSO selection frequency for each marker during the 50 iterations of QTCAT. (**d**) Manhattan plot of the LMM analysis. The horizontal dashed line depicts the significance threshold when controlling the multiple testing with FWER, whereas the red markers are significantly associated when controlling with FDR. (**e**) The Manhattan plot of the LMM* analysis. GRM was estimated without markers on the chromosome of the actual testing position. The results are shown as in (d).

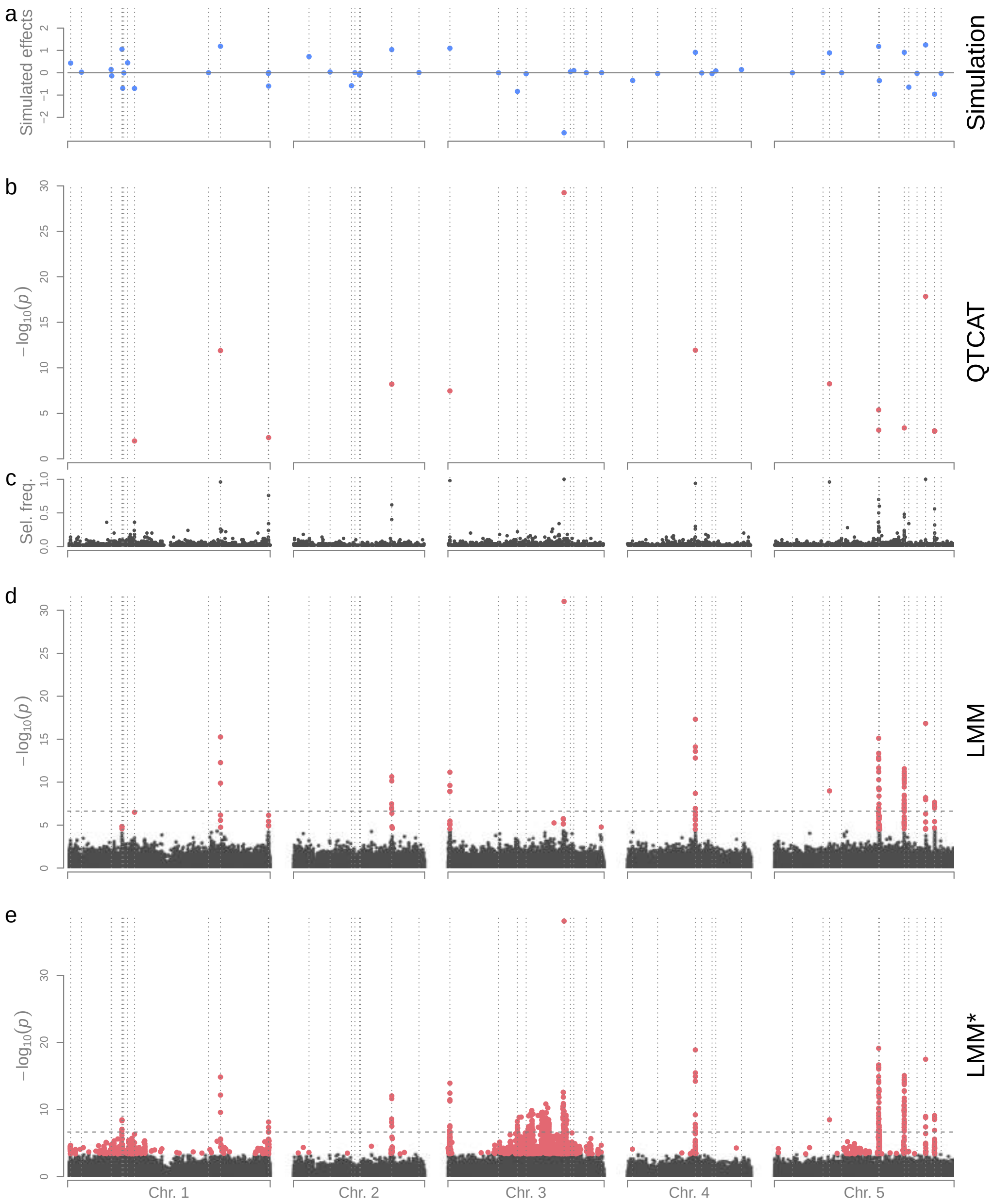

Supplementary Figure 94 Simulation of a GWA analysis based on a structured population with a heritability of 0.7 (run 94). (**a**) Simulation of 50 effects randomly drawn from a Gamma distribution and assigned to random markers. Markers with effect are highlighted with dashed lines. (**b**) Significant QTCs found by QTCAT. (**c**) LASSO selection frequency for each marker during the 50 iterations of QTCAT. (**d**) Manhattan plot of the LMM analysis. The horizontal dashed line depicts the significance threshold when controlling the multiple testing with FWER, whereas the red markers are significantly associated when controlling with FDR. (**e**) The Manhattan plot of the LMM* analysis. GRM was estimated without markers on the chromosome of the actual testing position. The results are shown as in (d).

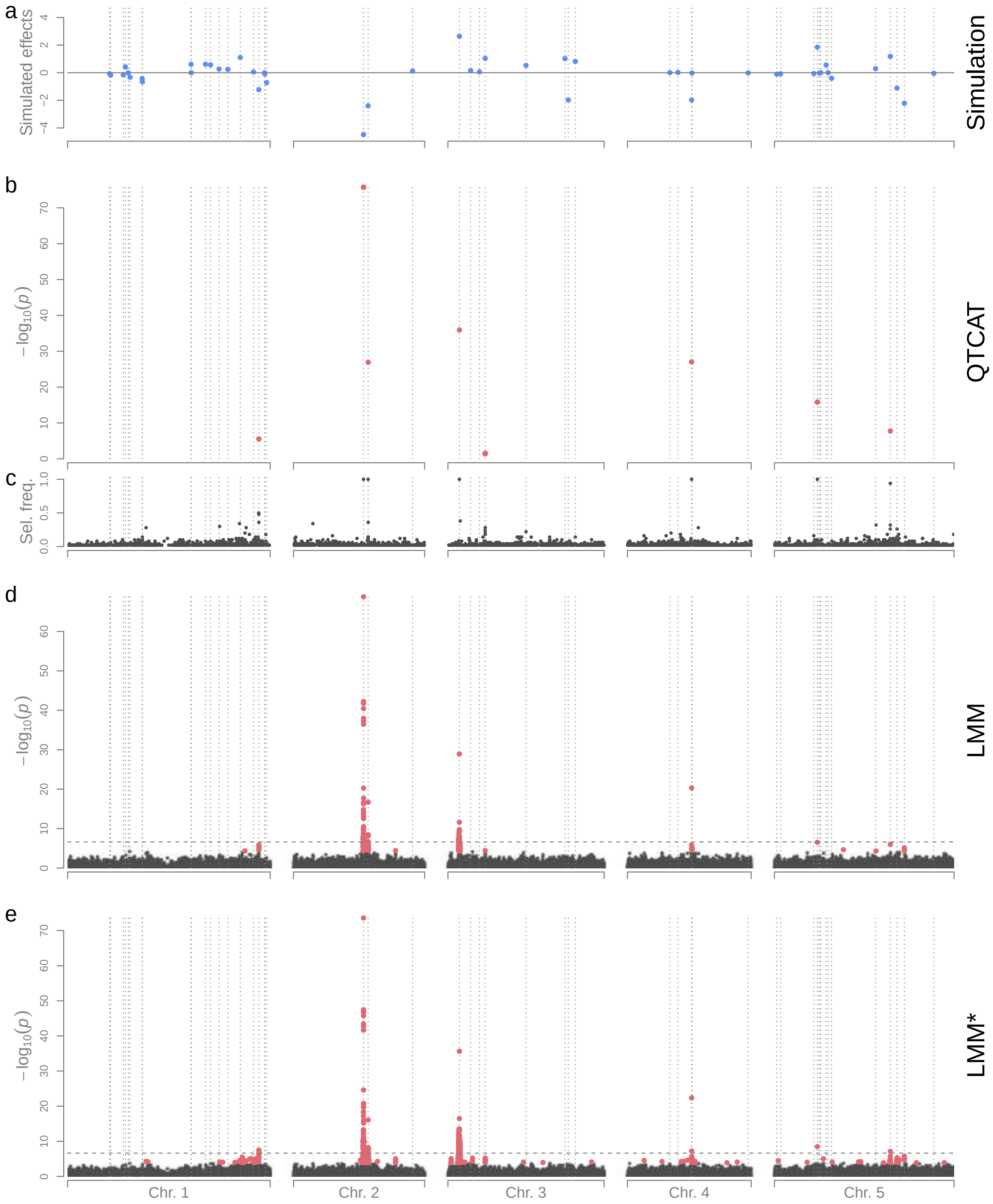

Supplementary Figure 95 Simulation of a GWA analysis based on a structured population with a heritability of 0.7 (run 95). (**a**) Simulation of 50 effects randomly drawn from a Gamma distribution and assigned to random markers. Markers with effect are highlighted with dashed lines. (**b**) Significant QTCs found by QTCAT. (**c**) LASSO selection frequency for each marker during the 50 iterations of QTCAT. (**d**) Manhattan plot of the LMM analysis. The horizontal dashed line depicts the significance threshold when controlling the multiple testing with FWER, whereas the red markers are significantly associated when controlling with FDR. (**e**) The Manhattan plot of the LMM* analysis. GRM was estimated without markers on the chromosome of the actual testing position. The results are shown as in (d).

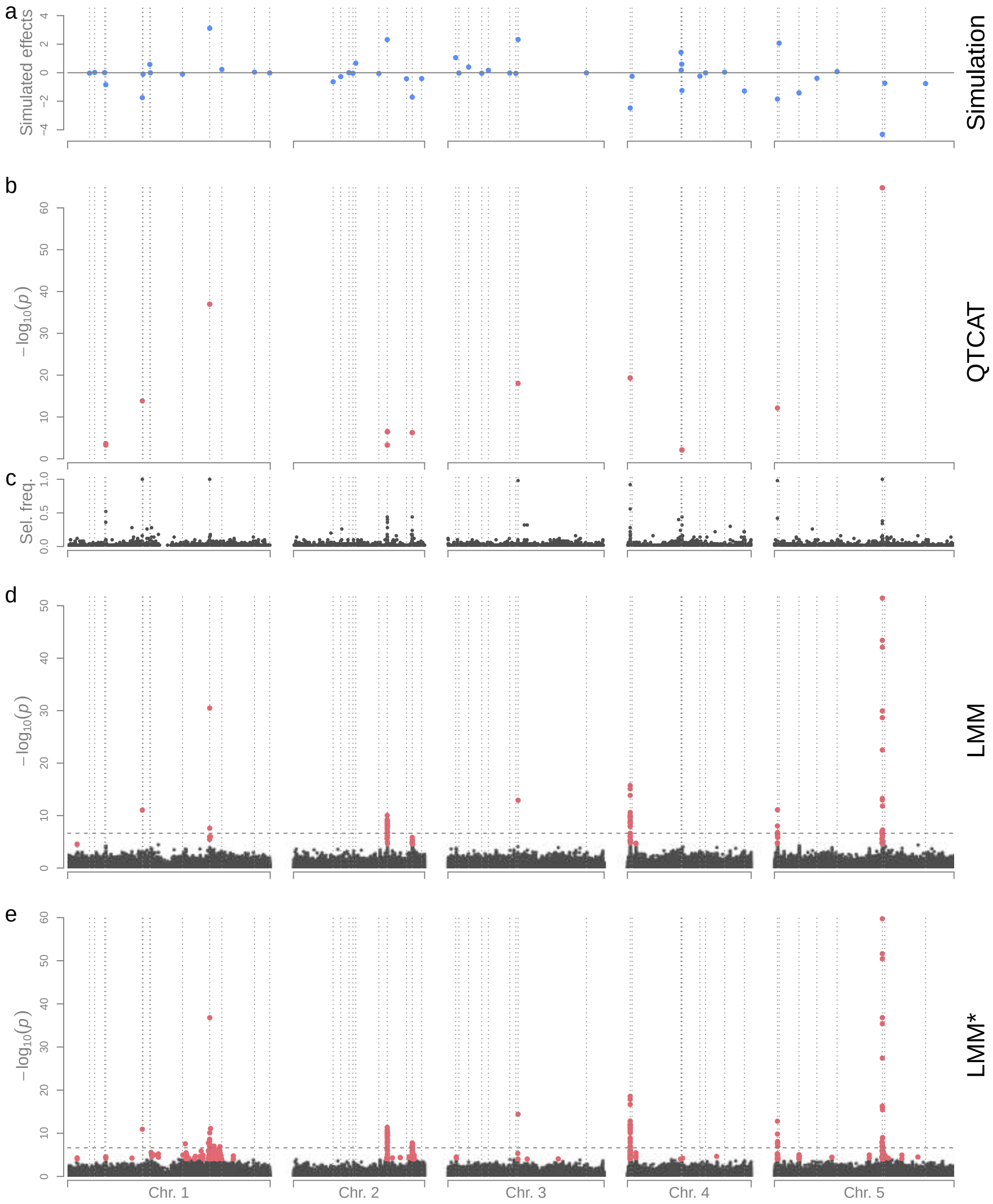

Supplementary Figure 96 Simulation of a GWA analysis based on a structured population with a heritability of 0.7 (run 96). (**a**) Simulation of 50 effects randomly drawn from a Gamma distribution and assigned to random markers. Markers with effect are highlighted with dashed lines. (**b**) Significant QTCs found by QTCAT. (**c**) LASSO selection frequency for each marker during the 50 iterations of QTCAT. (**d**) Manhattan plot of the LMM analysis. The horizontal dashed line depicts the significance threshold when controlling the multiple testing with FWER, whereas the red markers are significantly associated when controlling with FDR. (**e**) The Manhattan plot of the LMM* analysis. GRM was estimated without markers on the chromosome of the actual testing position. The results are shown as in (d).

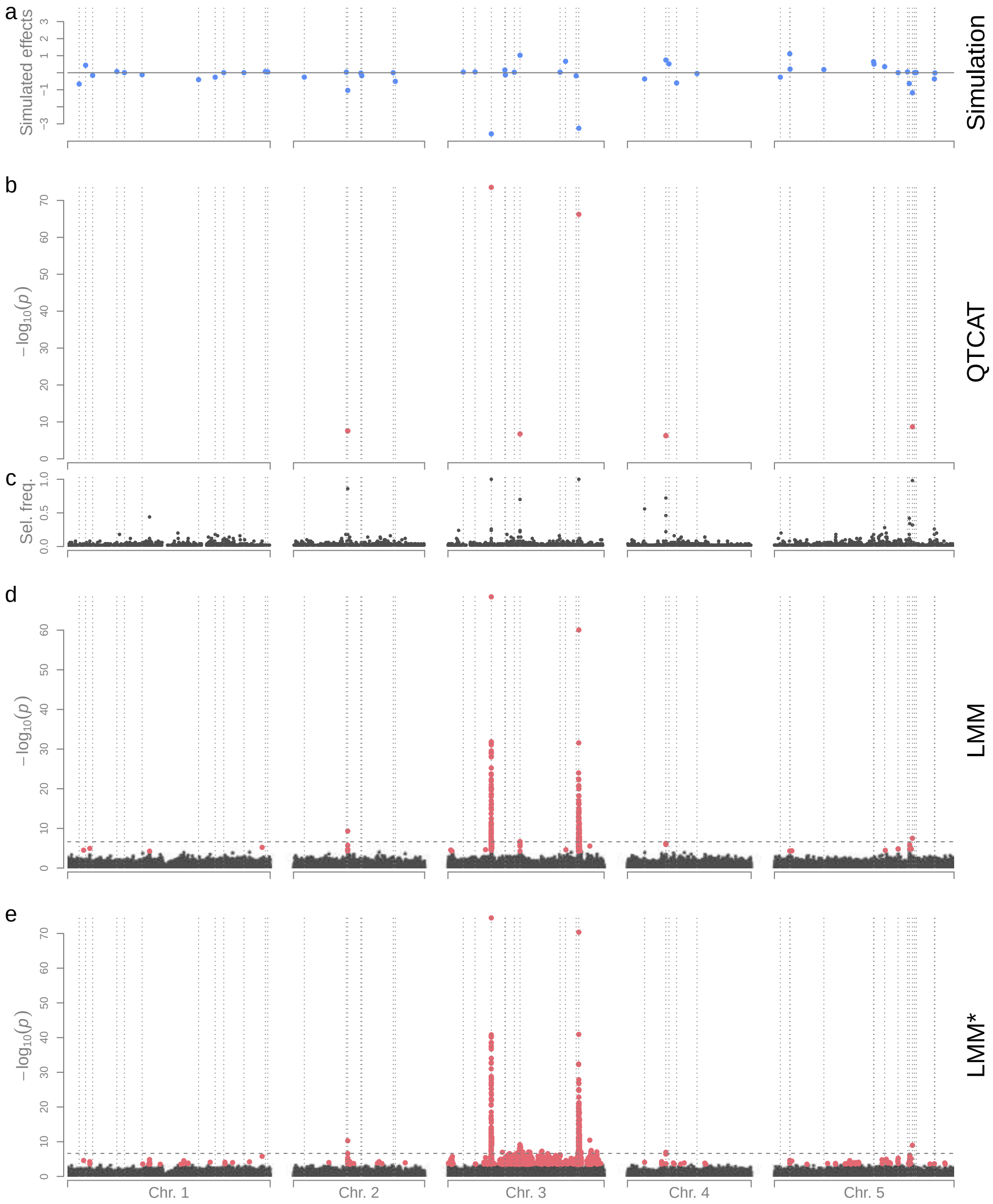

Supplementary Figure 97 Simulation of a GWA analysis based on a structured population with a heritability of 0.7 (run 97). (**a**) Simulation of 50 effects randomly drawn from a Gamma distribution and assigned to random markers. Markers with effect are highlighted with dashed lines. (**b**) Significant QTCs found by QTCAT. (**c**) LASSO selection frequency for each marker during the 50 iterations of QTCAT. (**d**) Manhattan plot of the LMM analysis. The horizontal dashed line depicts the significance threshold when controlling the multiple testing with FWER, whereas the red markers are significantly associated when controlling with FDR. (**e**) The Manhattan plot of the LMM* analysis. GRM was estimated without markers on the chromosome of the actual testing position. The results are shown as in (d).

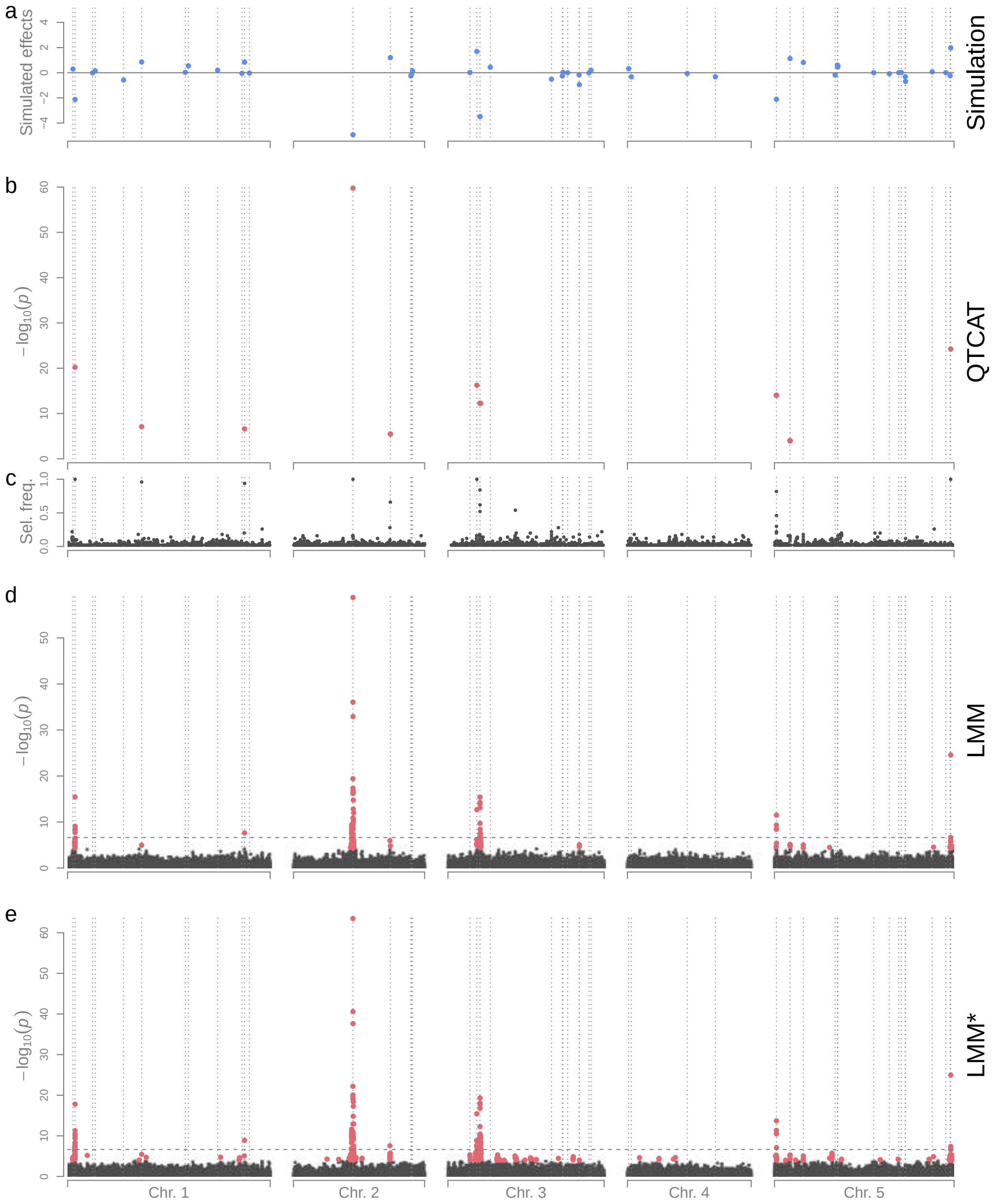

Supplementary Figure 98 Simulation of a GWA analysis based on a structured population with a heritability of 0.7 (run 98). (**a**) Simulation of 50 effects randomly drawn from a Gamma distribution and assigned to random markers. Markers with effect are highlighted with dashed lines. (**b**) Significant QTCs found by QTCAT. (**c**) LASSO selection frequency for each marker during the 50 iterations of QTCAT. (**d**) Manhattan plot of the LMM analysis. The horizontal dashed line depicts the significance threshold when controlling the multiple testing with FWER, whereas the red markers are significantly associated when controlling with FDR. (**e**) The Manhattan plot of the LMM* analysis. GRM was estimated without markers on the chromosome of the actual testing position. The results are shown as in (d).

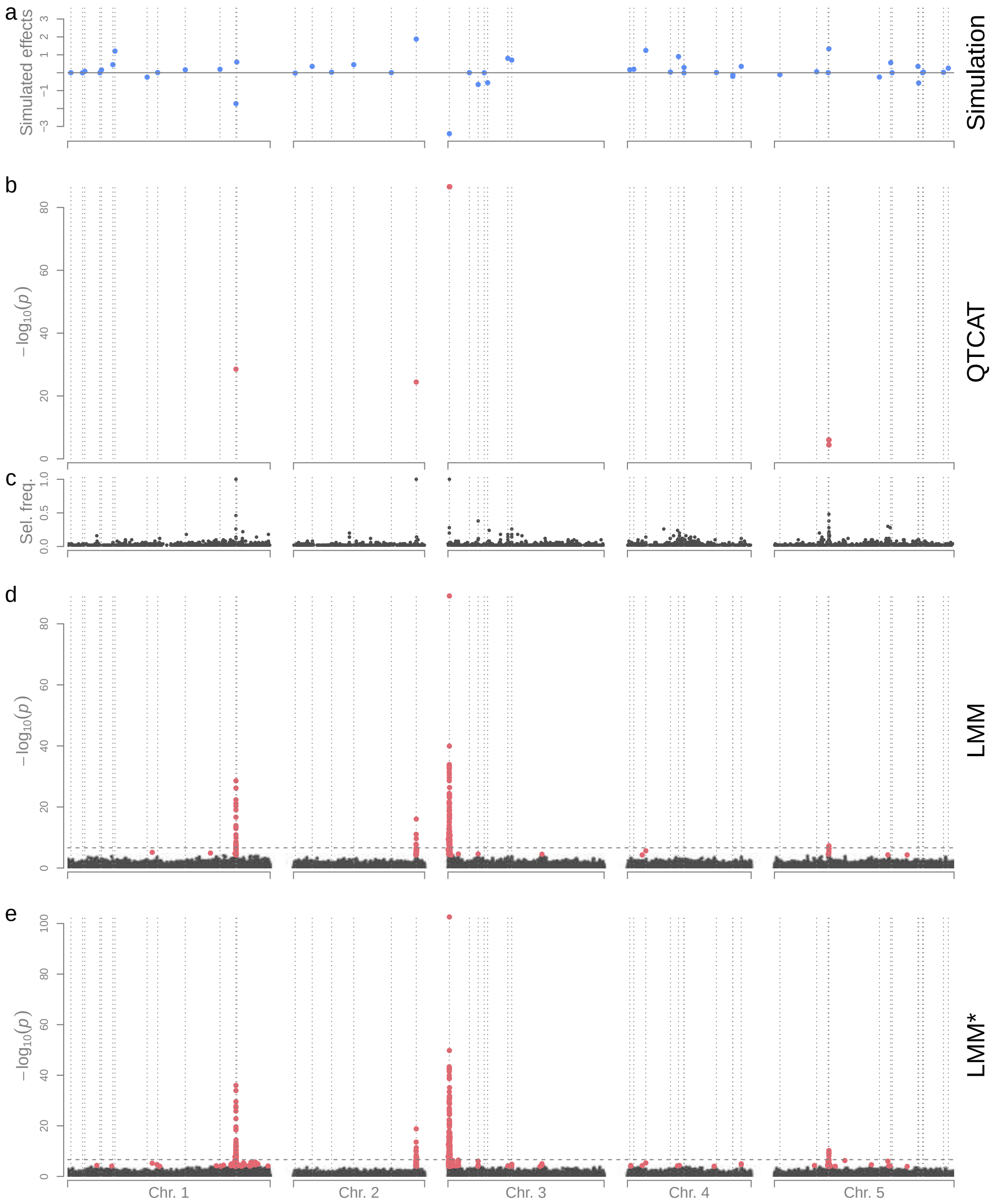

Supplementary Figure 99 Simulation of a GWA analysis based on a structured population with a heritability of 0.7 (run 99). (**a**) Simulation of 50 effects randomly drawn from a Gamma distribution and assigned to random markers. Markers with effect are highlighted with dashed lines. (**b**) Significant QTCs found by QTCAT. (**c**) LASSO selection frequency for each marker during the 50 iterations of QTCAT. (**d**) Manhattan plot of the LMM analysis. The horizontal dashed line depicts the significance threshold when controlling the multiple testing with FWER, whereas the red markers are significantly associated when controlling with FDR. (**e**) The Manhattan plot of the LMM* analysis. GRM was estimated without markers on the chromosome of the actual testing position. The results are shown as in (d).

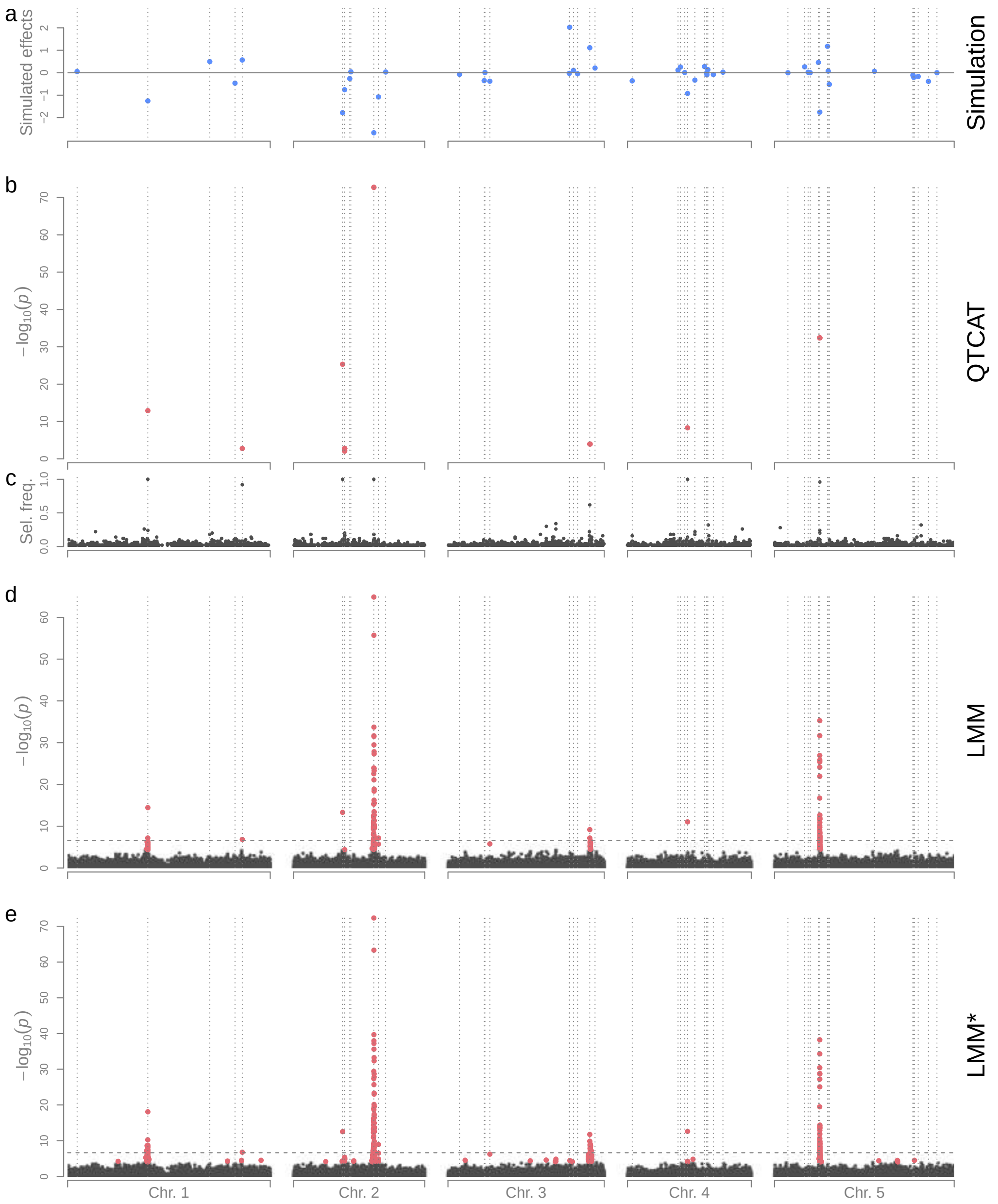

Supplementary Figure 100 Simulation of a GWA analysis based on a structured population with a heritability of 0.7 (run 100). (**a**) Simulation of 50 effects randomly drawn from a Gamma distribution and assigned to random markers. Markers with effect are highlighted with dashed lines. (**b**) Significant QTCs found by QTCAT. (**c**) LASSO selection frequency for each marker during the 50 iterations of QTCAT. (**d**) Manhattan plot of the LMM analysis. The horizontal dashed line depicts the significance threshold when controlling the multiple testing with FWER, whereas the red markers are significantly associated when controlling with FDR. (**e**) The Manhattan plot of the LMM* analysis. GRM was estimated without markers on the chromosome of the actual testing position. The results are shown as in (d).

Simulation with heritability of 0.7 and 50 effects randomly drawn from a normal distribution

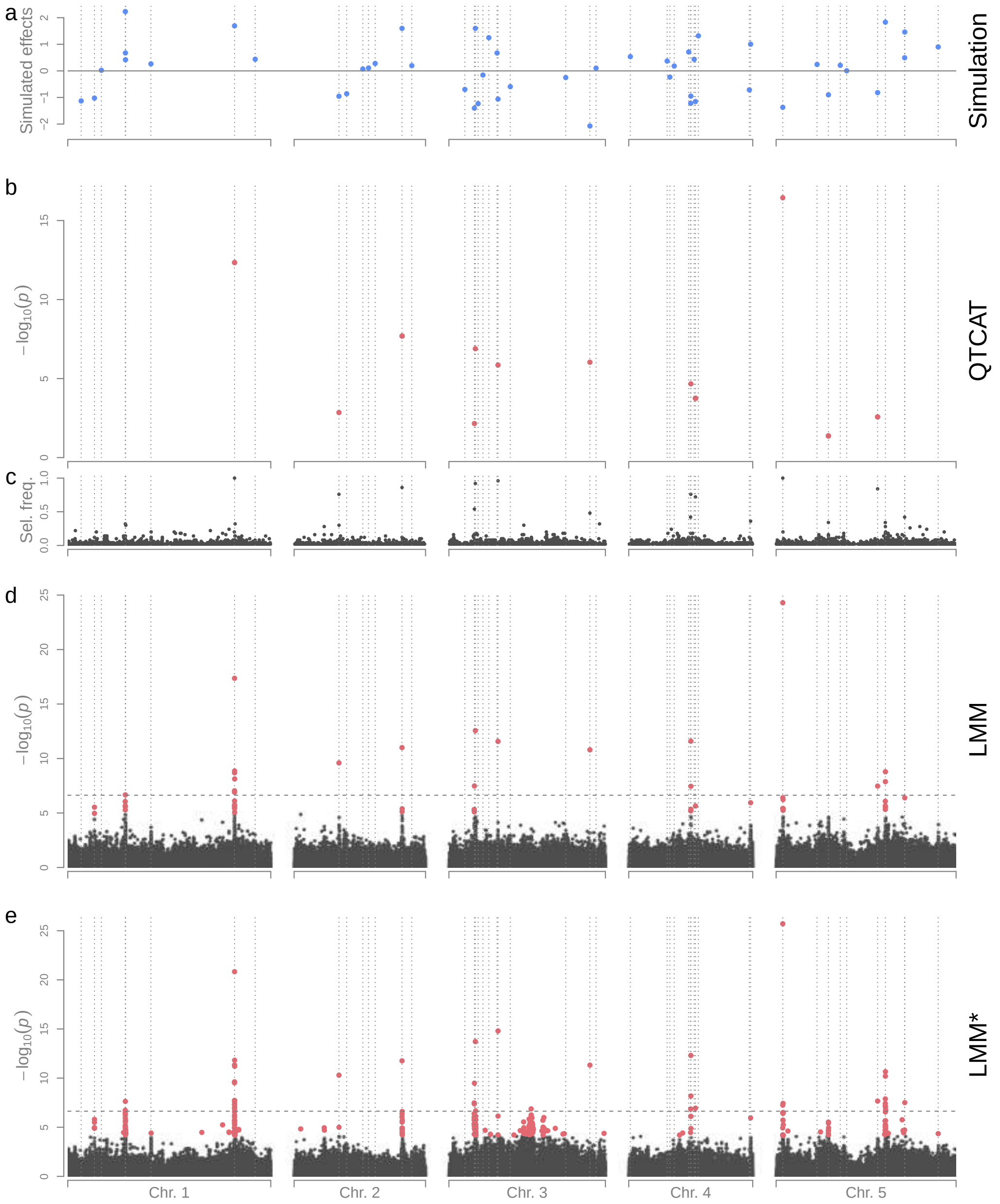

Supplementary Figure 101 Simulation of a GWA analysis based on a structured population with a heritability of 0.7 (run 1). (**a**) Simulation of 50 effects randomly drawn from a normal distribution and assigned to random markers. Markers with effect are highlighted with dashed lines. (**b**) Significant QTCs found by QTCAT. (**c**) LASSO selection frequency for each marker during the 50 iterations of QTCAT. (**d**) Manhattan plot of the LMM analysis. The horizontal dashed line depicts the significance threshold when controlling the multiple testing with FWER, whereas the red markers are significantly associated when controlling with FDR. (**e**) The Manhattan plot of the LMM* analysis. GRM was estimated without markers on the chromosome of the actual testing position. The results are shown as in (d).

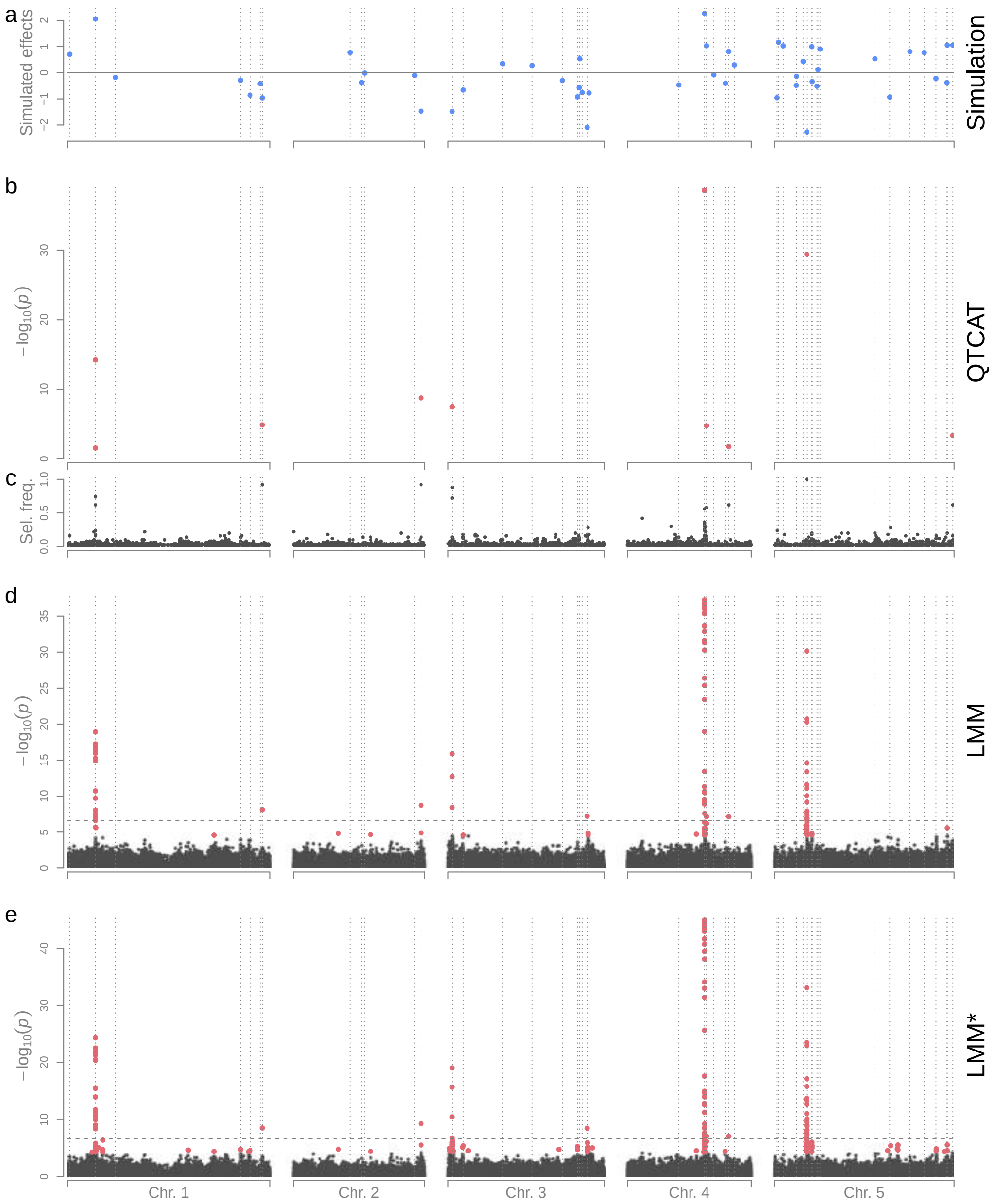

Supplementary Figure 102 Simulation of a GWA analysis based on a structured population with a heritability of 0.7 (run 2). (**a**) Simulation of 50 effects randomly drawn from a normal distribution and assigned to random markers. Markers with effect are highlighted with dashed lines. (**b**) Significant QTCs found by QTCAT. (**c**) LASSO selection frequency for each marker during the 50 iterations of QTCAT. (**d**) Manhattan plot of the LMM analysis. The horizontal dashed line depicts the significance threshold when controlling the multiple testing with FWER, whereas the red markers are significantly associated when controlling with FDR. (**e**) The Manhattan plot of the LMM* analysis. GRM was estimated without markers on the chromosome of the actual testing position. The results are shown as in (d).

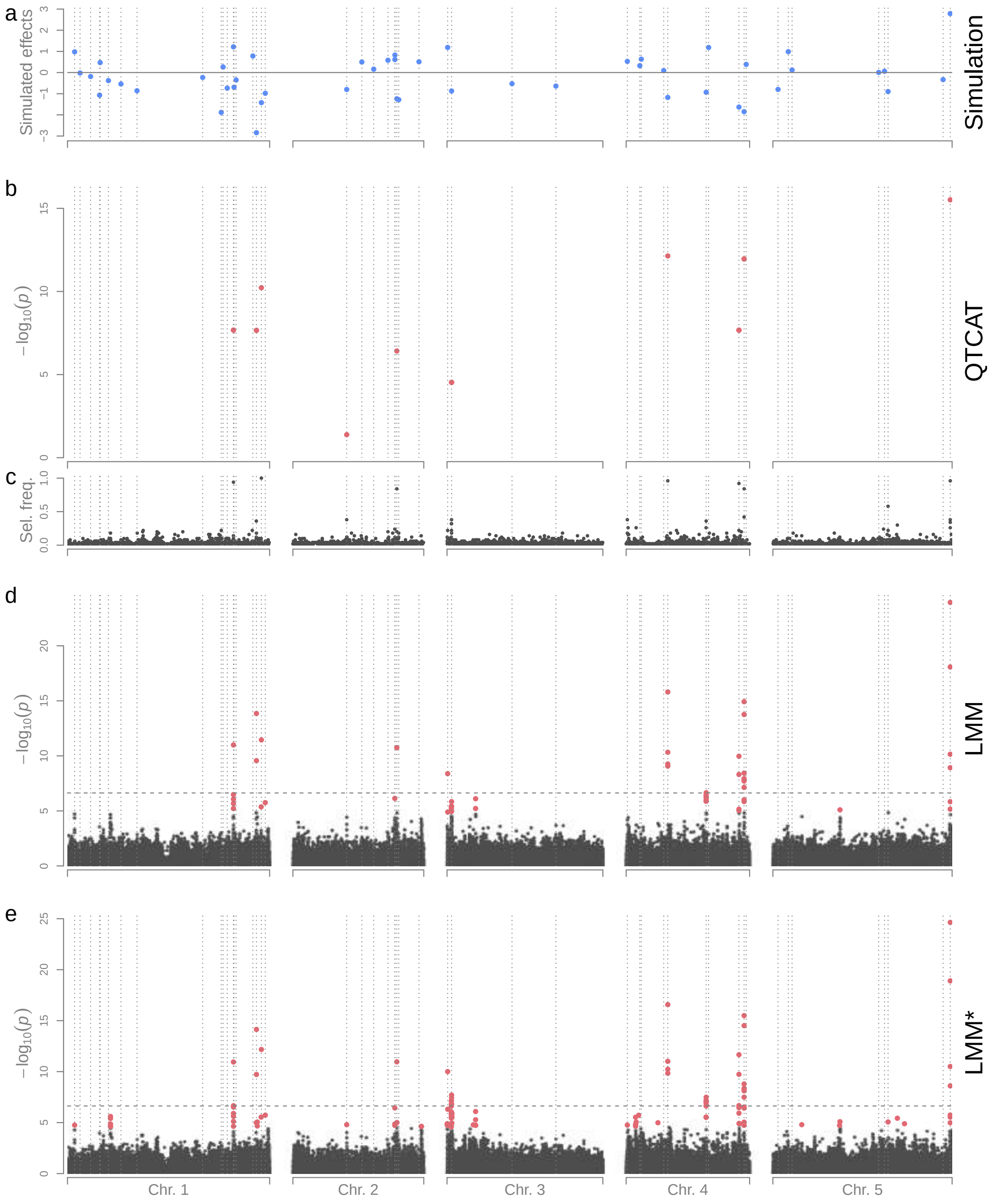

Supplementary Figure 103 Simulation of a GWA analysis based on a structured population with a heritability of 0.7 (run 3). (**a**) Simulation of 50 effects randomly drawn from a normal distribution and assigned to random markers. Markers with effect are highlighted with dashed lines. (**b**) Significant QTCs found by QTCAT. (**c**) LASSO selection frequency for each marker during the 50 iterations of QTCAT. (**d**) Manhattan plot of the LMM analysis. The horizontal dashed line depicts the significance threshold when controlling the multiple testing with FWER, whereas the red markers are significantly associated when controlling with FDR. (**e**) The Manhattan plot of the LMM* analysis. GRM was estimated without markers on the chromosome of the actual testing position. The results are shown as in (d).

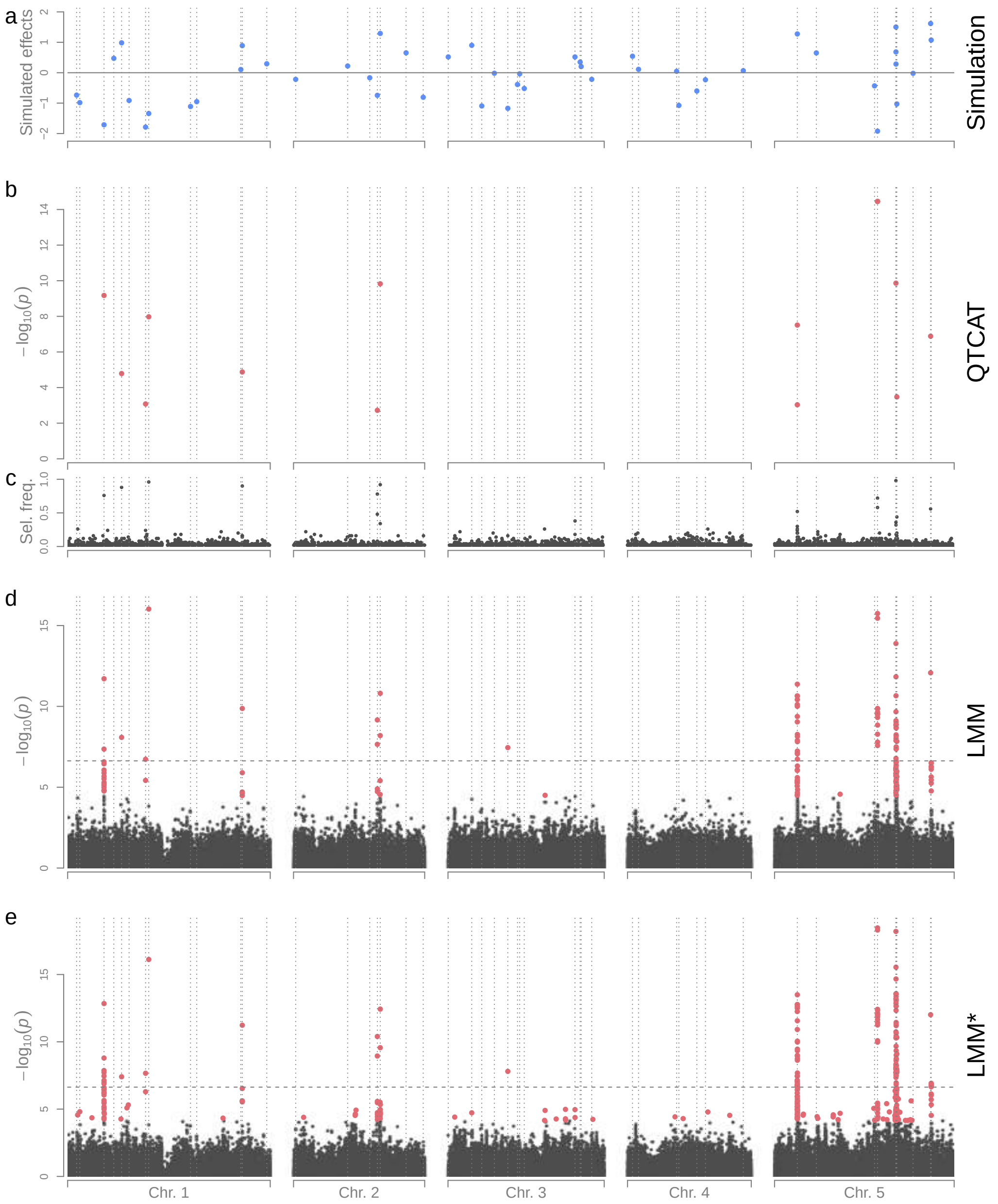

Supplementary Figure 104 Simulation of a GWA analysis based on a structured population with a heritability of 0.7 (run 4). (**a**) Simulation of 50 effects randomly drawn from a normal distribution and assigned to random markers. Markers with effect are highlighted with dashed lines. (**b**) Significant QTCs found by QTCAT. (**c**) LASSO selection frequency for each marker during the 50 iterations of QTCAT. (**d**) Manhattan plot of the LMM analysis. The horizontal dashed line depicts the significance threshold when controlling the multiple testing with FWER, whereas the red markers are significantly associated when controlling with FDR. (**e**) The Manhattan plot of the LMM* analysis. GRM was estimated without markers on the chromosome of the actual testing position. The results are shown as in (d).

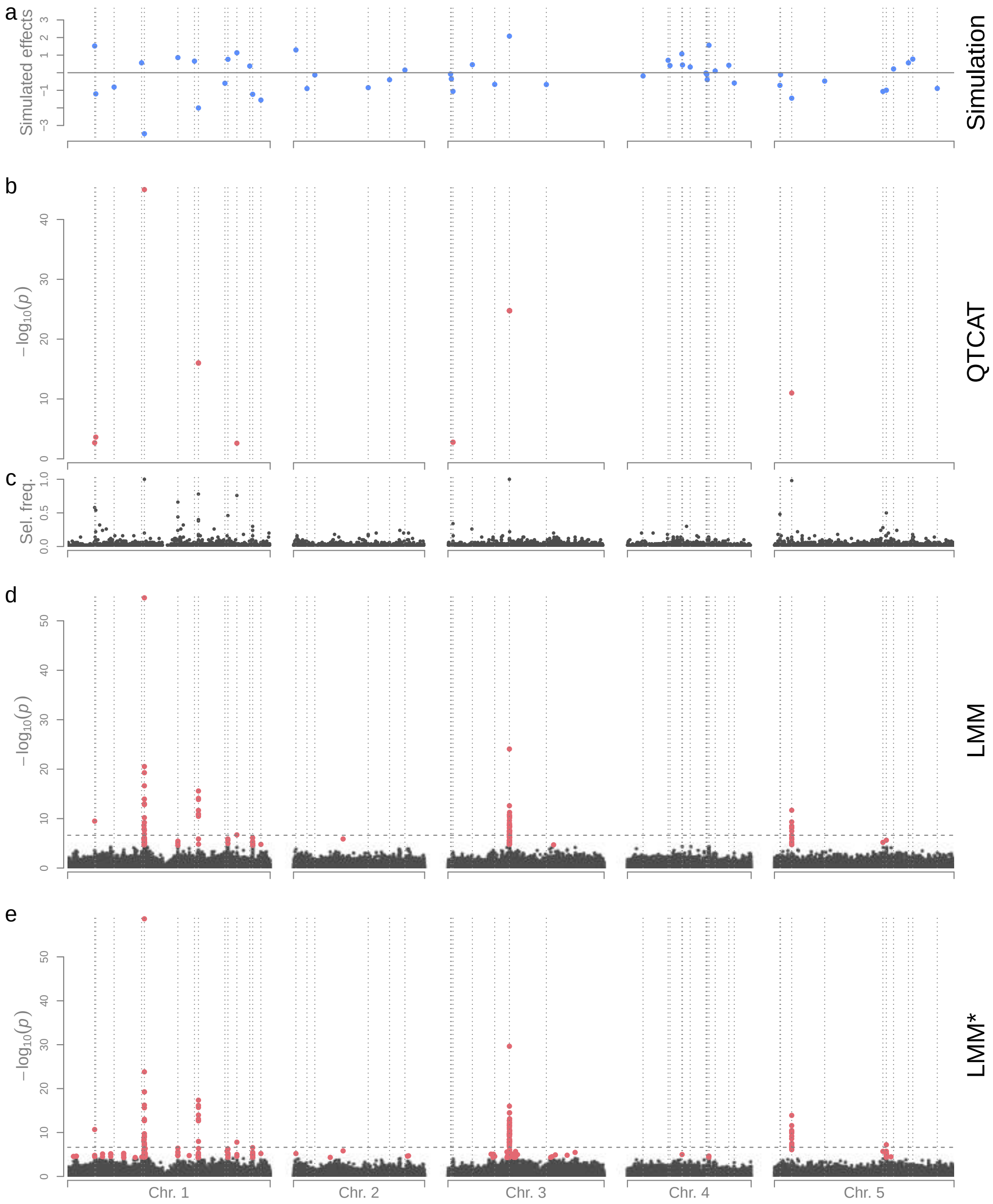

Supplementary Figure 105 Simulation of a GWA analysis based on a structured population with a heritability of 0.7 (run 5). (**a**) Simulation of 50 effects randomly drawn from a normal distribution and assigned to random markers. Markers with effect are highlighted with dashed lines. (**b**) Significant QTCs found by QTCAT. (**c**) LASSO selection frequency for each marker during the 50 iterations of QTCAT. (**d**) Manhattan plot of the LMM analysis. The horizontal dashed line depicts the significance threshold when controlling the multiple testing with FWER, whereas the red markers are significantly associated when controlling with FDR. (**e**) The Manhattan plot of the LMM* analysis. GRM was estimated without markers on the chromosome of the actual testing position. The results are shown as in (d).

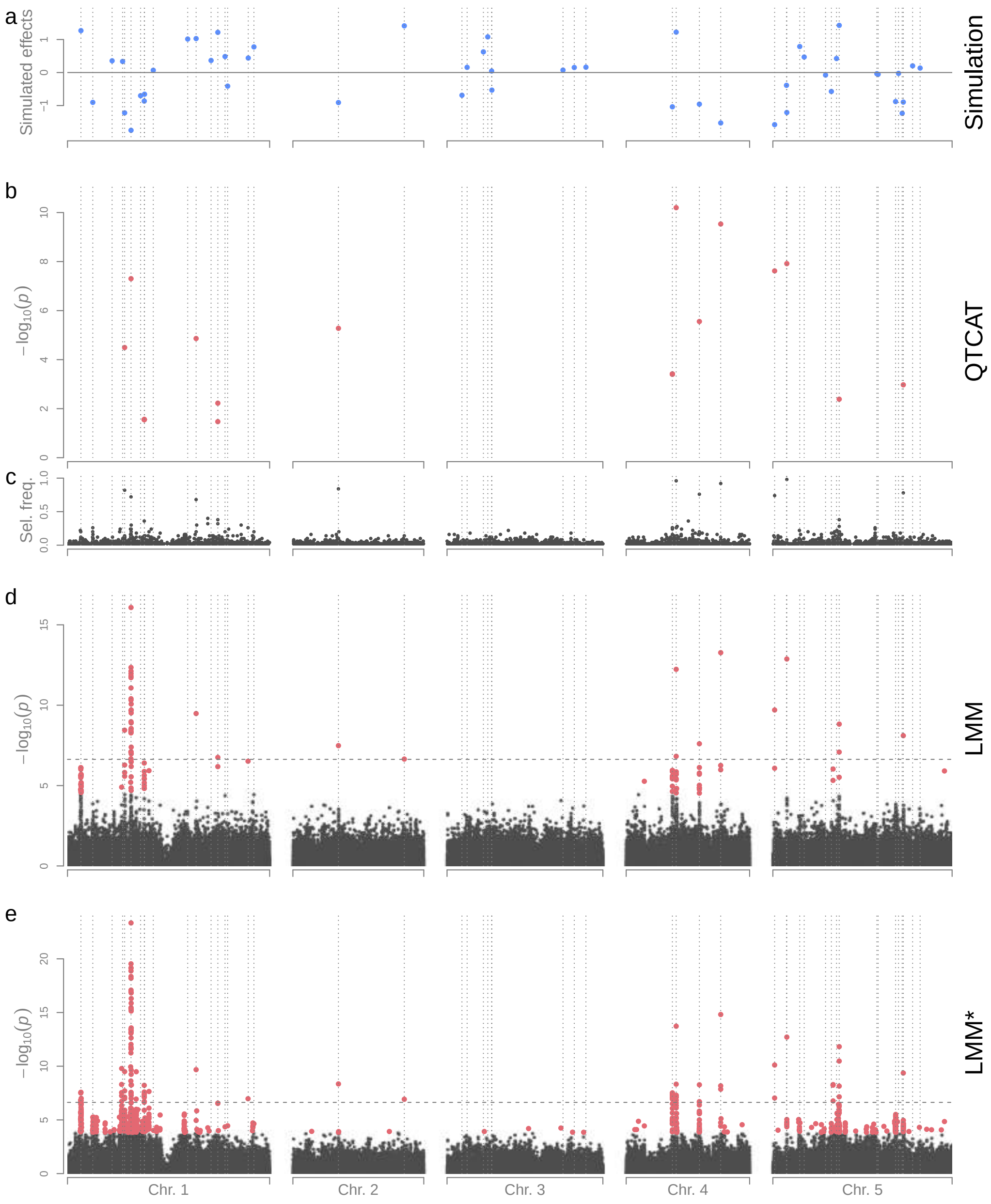

Supplementary Figure 106 Simulation of a GWA analysis based on a structured population with a heritability of 0.7 (run 6). (**a**) Simulation of 50 effects randomly drawn from a normal distribution and assigned to random markers. Markers with effect are highlighted with dashed lines. (**b**) Significant QTCs found by QTCAT. (**c**) LASSO selection frequency for each marker during the 50 iterations of QTCAT. (**d**) Manhattan plot of the LMM analysis. The horizontal dashed line depicts the significance threshold when controlling the multiple testing with FWER, whereas the red markers are significantly associated when controlling with FDR. (**e**) The Manhattan plot of the LMM* analysis. GRM was estimated without markers on the chromosome of the actual testing position. The results are shown as in (d).

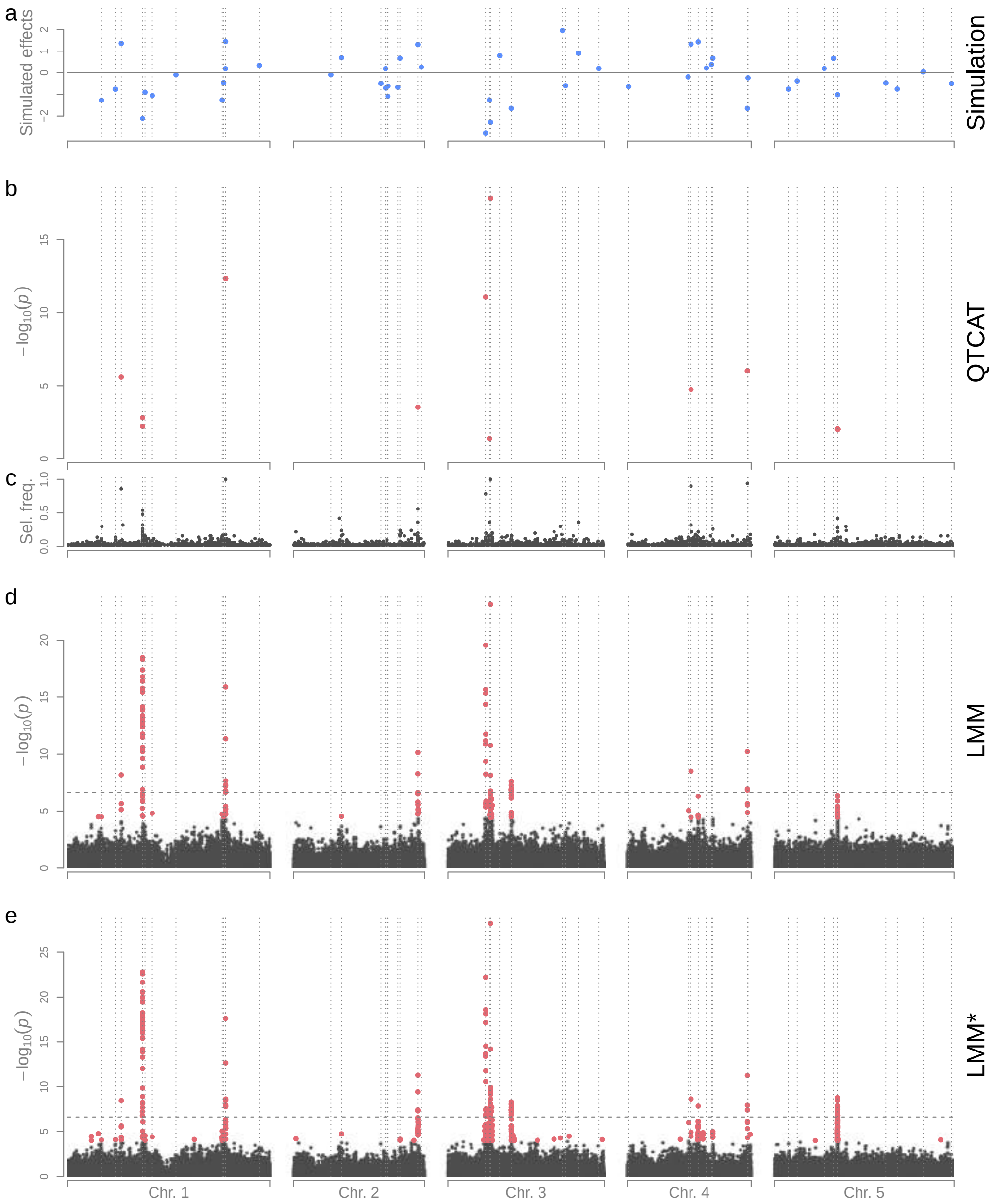

Supplementary Figure 107 Simulation of a GWA analysis based on a structured population with a heritability of 0.7 (run 7). (**a**) Simulation of 50 effects randomly drawn from a normal distribution and assigned to random markers. Markers with effect are highlighted with dashed lines. (**b**) Significant QTCs found by QTCAT. (**c**) LASSO selection frequency for each marker during the 50 iterations of QTCAT. (**d**) Manhattan plot of the LMM analysis. The horizontal dashed line depicts the significance threshold when controlling the multiple testing with FWER, whereas the red markers are significantly associated when controlling with FDR. (**e**) The Manhattan plot of the LMM* analysis. GRM was estimated without markers on the chromosome of the actual testing position. The results are shown as in (d).

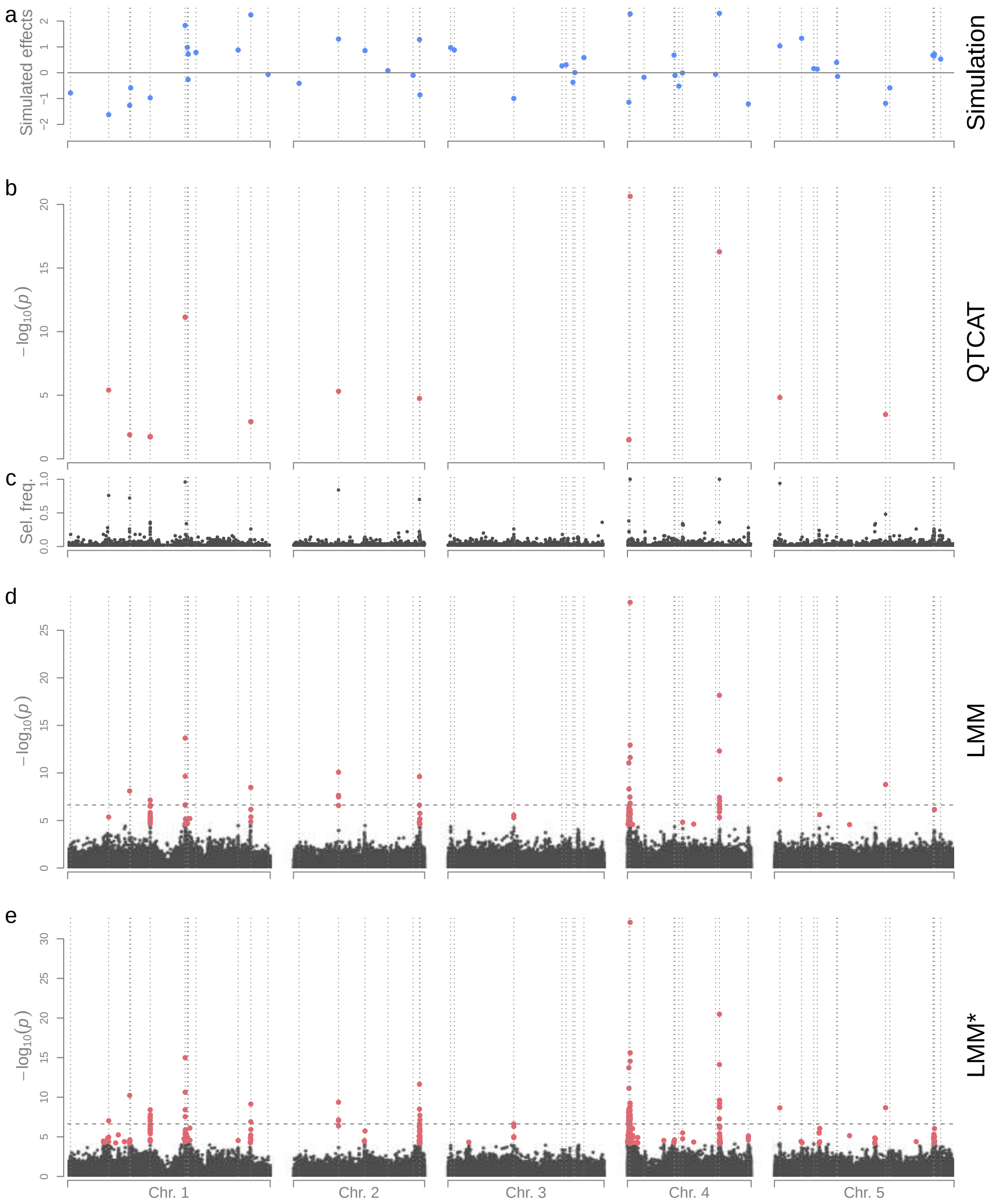

Supplementary Figure 108 Simulation of a GWA analysis based on a structured population with a heritability of 0.7 (run 8). (**a**) Simulation of 50 effects randomly drawn from a normal distribution and assigned to random markers. Markers with effect are highlighted with dashed lines. (**b**) Significant QTCs found by QTCAT. (**c**) LASSO selection frequency for each marker during the 50 iterations of QTCAT. (**d**) Manhattan plot of the LMM analysis. The horizontal dashed line depicts the significance threshold when controlling the multiple testing with FWER, whereas the red markers are significantly associated when controlling with FDR. (**e**) The Manhattan plot of the LMM* analysis. GRM was estimated without markers on the chromosome of the actual testing position. The results are shown as in (d).

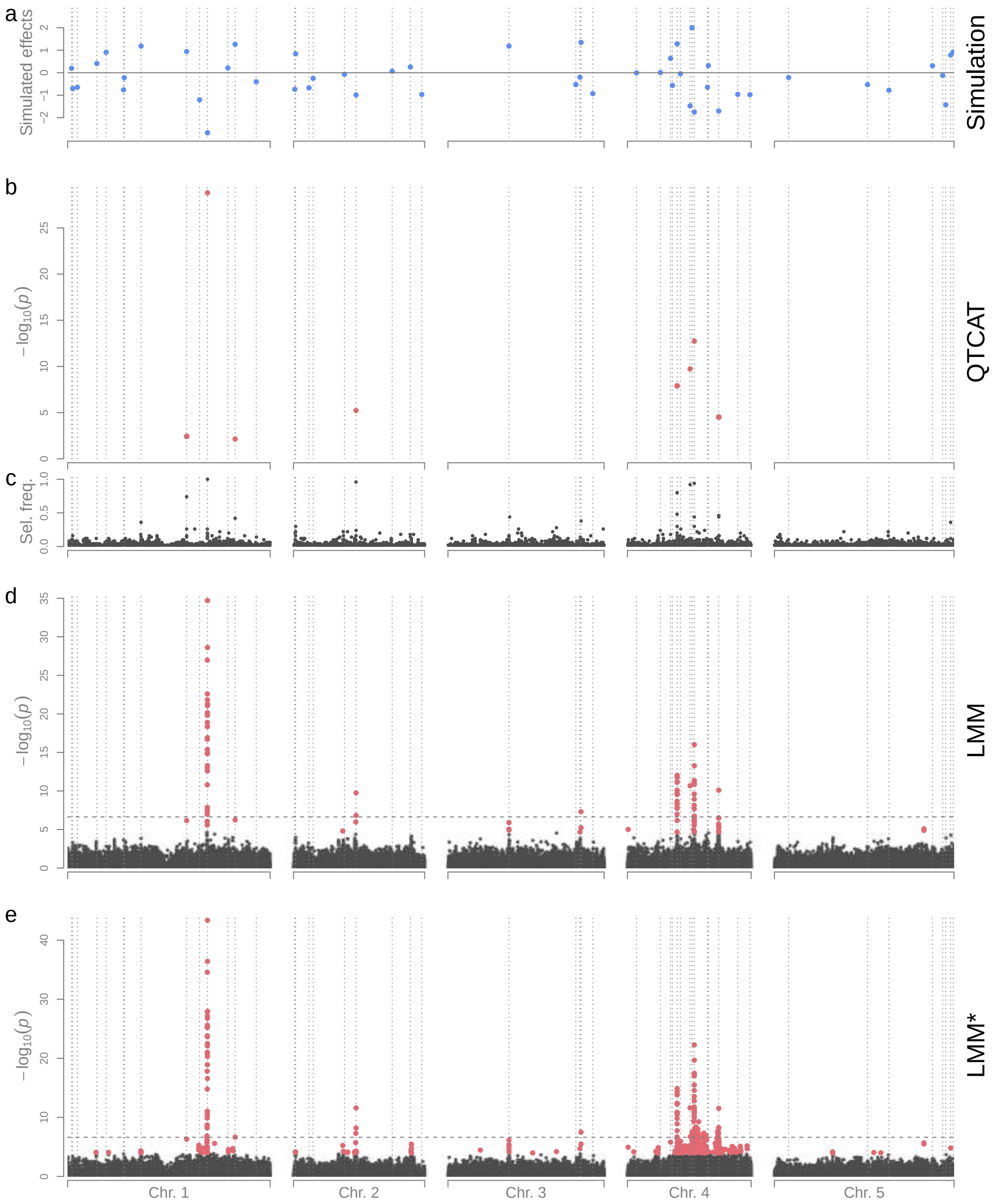

Supplementary Figure 109 Simulation of a GWA analysis based on a structured population with a heritability of 0.7 (run 9). (**a**) Simulation of 50 effects randomly drawn from a normal distribution and assigned to random markers. Markers with effect are highlighted with dashed lines. (**b**) Significant QTCs found by QTCAT. (**c**) LASSO selection frequency for each marker during the 50 iterations of QTCAT. (**d**) Manhattan plot of the LMM analysis. The horizontal dashed line depicts the significance threshold when controlling the multiple testing with FWER, whereas the red markers are significantly associated when controlling with FDR. (**e**) The Manhattan plot of the LMM* analysis. GRM was estimated without markers on the chromosome of the actual testing position. The results are shown as in (d).

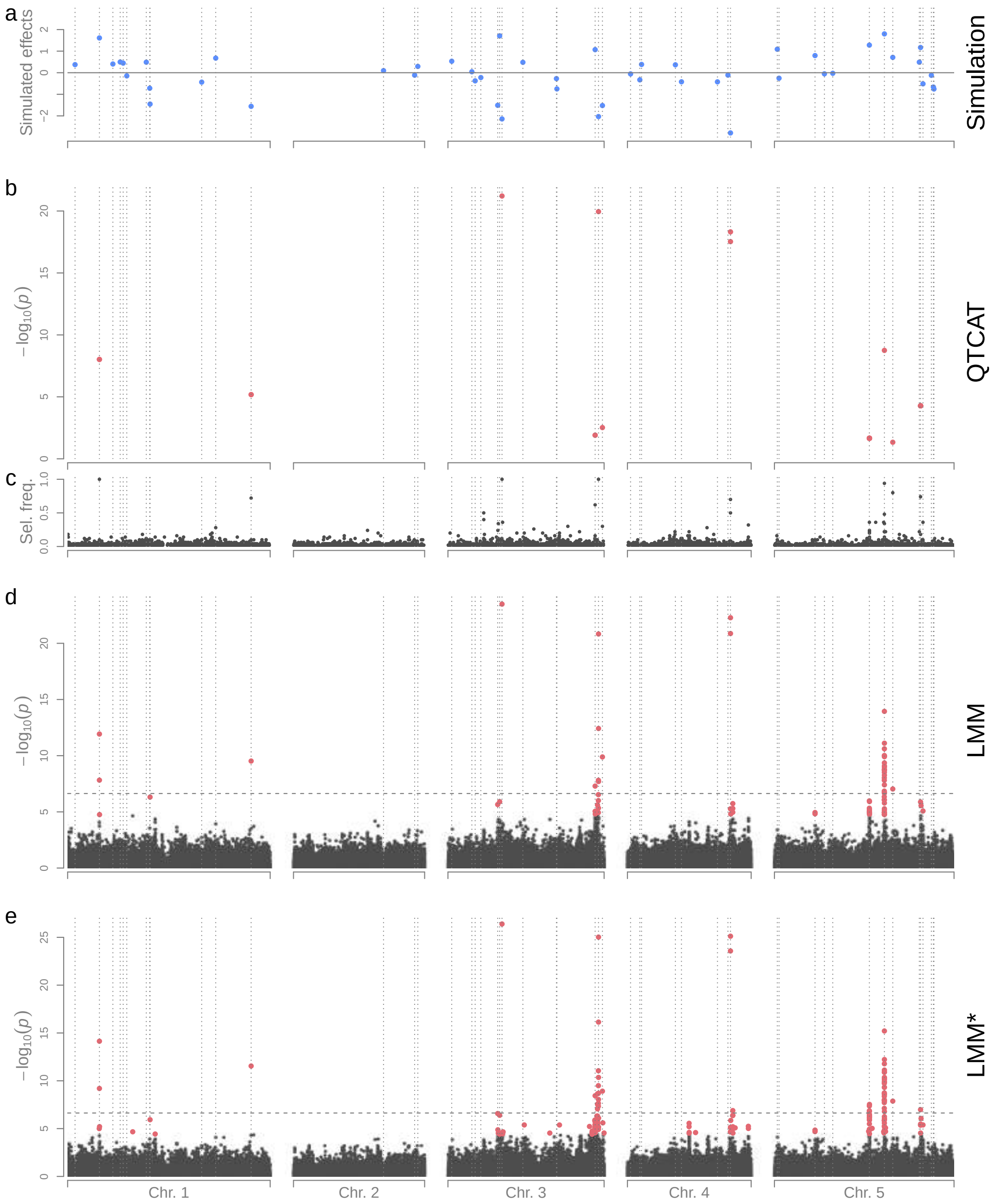

Supplementary Figure 110 Simulation of a GWA analysis based on a structured population with a heritability of 0.7 (run 10). (**a**) Simulation of 50 effects randomly drawn from a normal distribution and assigned to random markers. Markers with effect are highlighted with dashed lines. (**b**) Significant QTCs found by QTCAT. (**c**) LASSO selection frequency for each marker during the 50 iterations of QTCAT. (**d**) Manhattan plot of the LMM analysis. The horizontal dashed line depicts the significance threshold when controlling the multiple testing with FWER, whereas the red markers are significantly associated when controlling with FDR. (**e**) The Manhattan plot of the LMM* analysis. GRM was estimated without markers on the chromosome of the actual testing position. The results are shown as in (d).

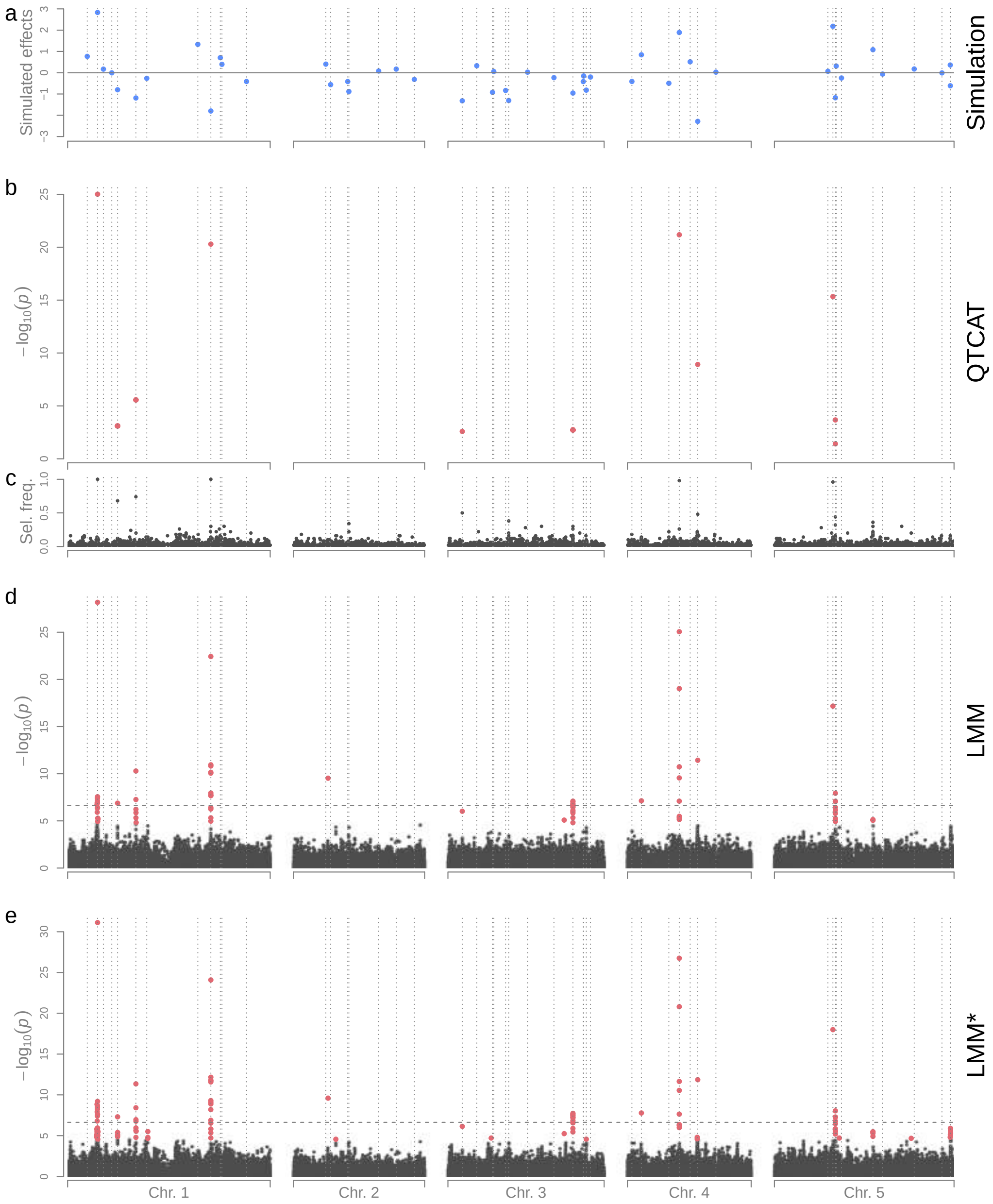

Supplementary Figure 111 Simulation of a GWA analysis based on a structured population with a heritability of 0.7 (run 11). (**a**) Simulation of 50 effects randomly drawn from a normal distribution and assigned to random markers. Markers with effect are highlighted with dashed lines. (**b**) Significant QTCs found by QTCAT. (**c**) LASSO selection frequency for each marker during the 50 iterations of QTCAT. (**d**) Manhattan plot of the LMM analysis. The horizontal dashed line depicts the significance threshold when controlling the multiple testing with FWER, whereas the red markers are significantly associated when controlling with FDR. (**e**) The Manhattan plot of the LMM* analysis. GRM was estimated without markers on the chromosome of the actual testing position. The results are shown as in (d).

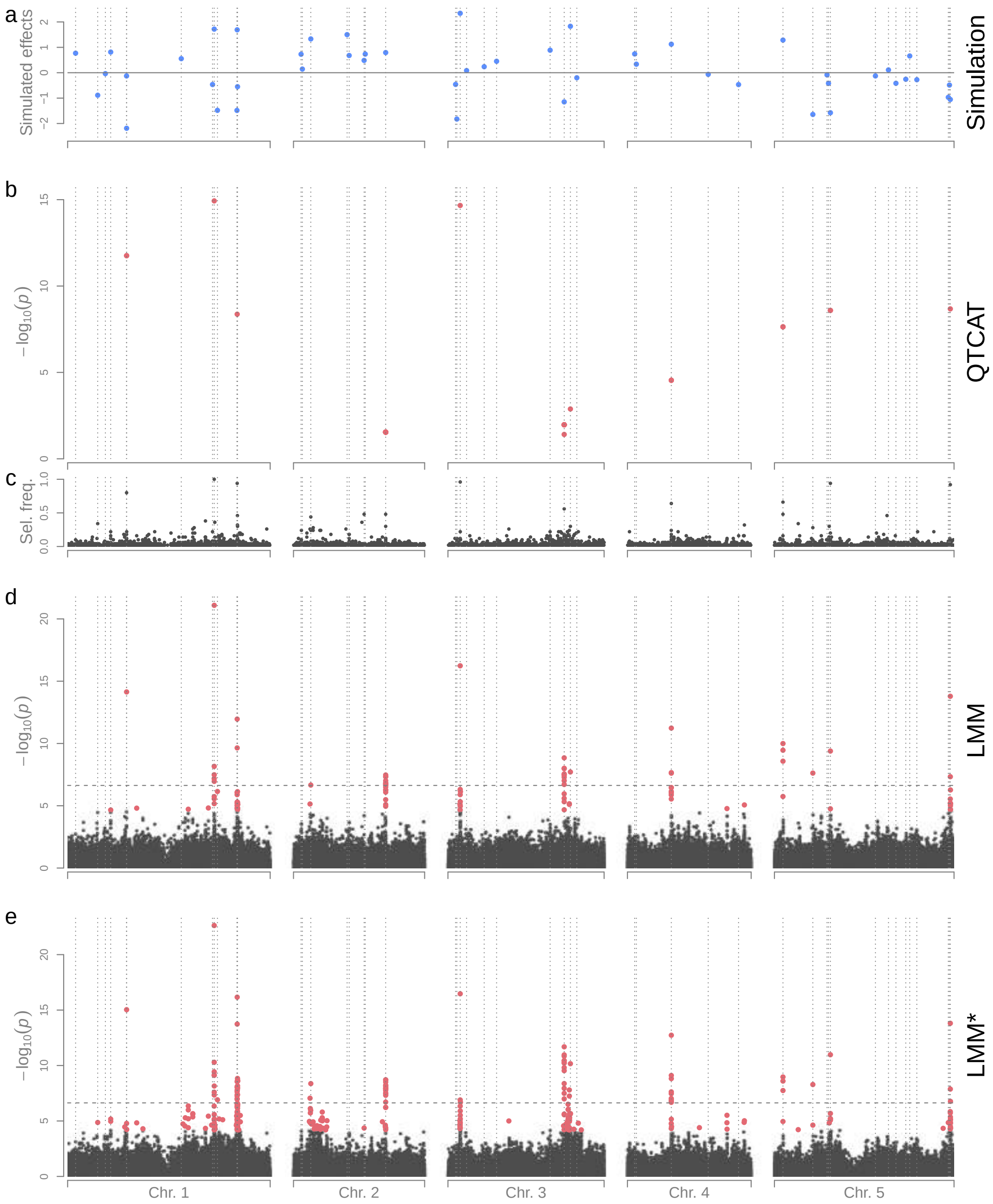

Supplementary Figure 112 Simulation of a GWA analysis based on a structured population with a heritability of 0.7 (run 12). (**a**) Simulation of 50 effects randomly drawn from a normal distribution and assigned to random markers. Markers with effect are highlighted with dashed lines. (**b**) Significant QTCs found by QTCAT. (**c**) LASSO selection frequency for each marker during the 50 iterations of QTCAT. (**d**) Manhattan plot of the LMM analysis. The horizontal dashed line depicts the significance threshold when controlling the multiple testing with FWER, whereas the red markers are significantly associated when controlling with FDR. (**e**) The Manhattan plot of the LMM* analysis. GRM was estimated without markers on the chromosome of the actual testing position. The results are shown as in (d).

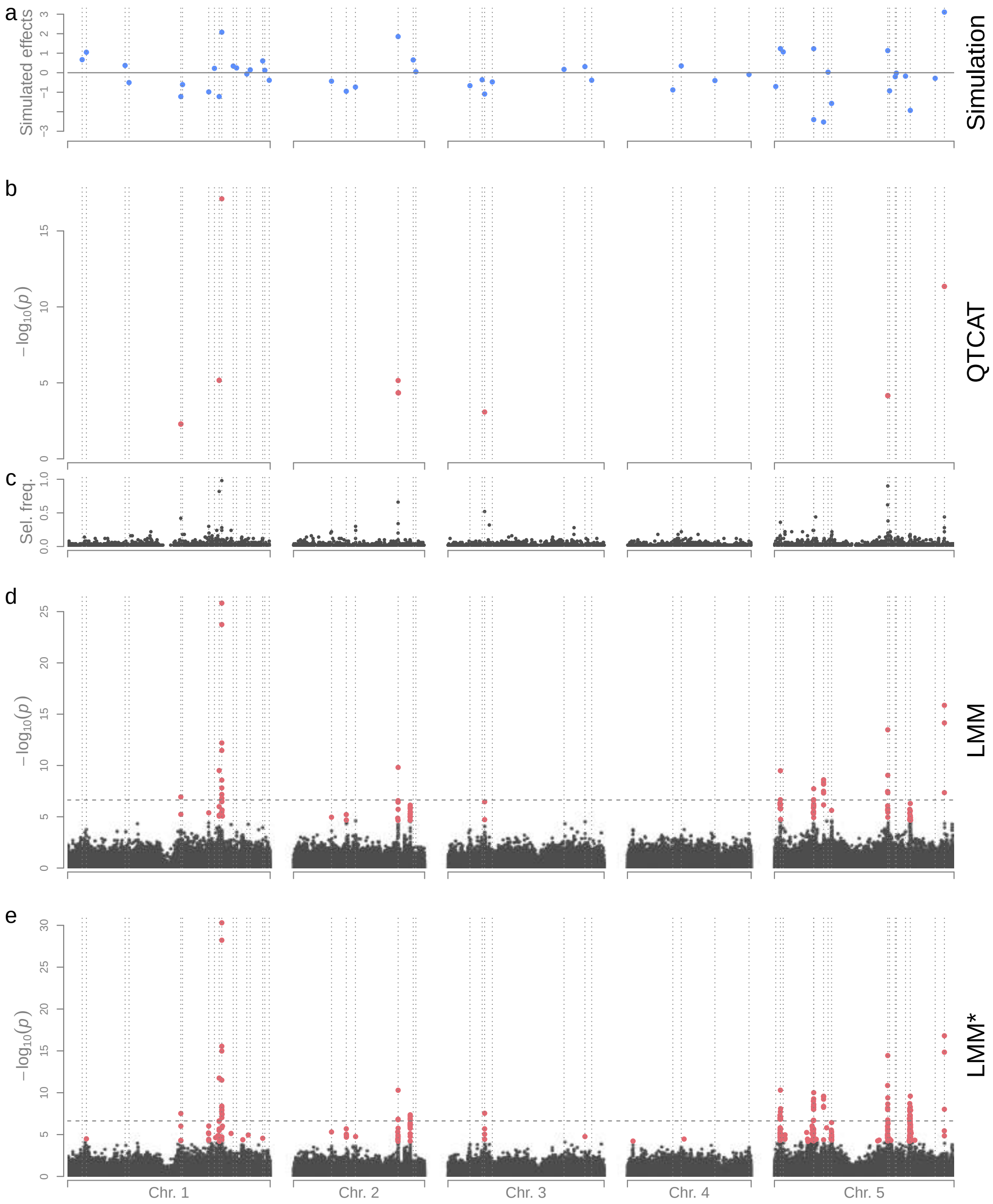

Supplementary Figure 113 Simulation of a GWA analysis based on a structured population with a heritability of 0.7 (run 13). (**a**) Simulation of 50 effects randomly drawn from a normal distribution and assigned to random markers. Markers with effect are highlighted with dashed lines. (**b**) Significant QTCs found by QTCAT. (**c**) LASSO selection frequency for each marker during the 50 iterations of QTCAT. (**d**) Manhattan plot of the LMM analysis. The horizontal dashed line depicts the significance threshold when controlling the multiple testing with FWER, whereas the red markers are significantly associated when controlling with FDR. (**e**) The Manhattan plot of the LMM* analysis. GRM was estimated without markers on the chromosome of the actual testing position. The results are shown as in (d).

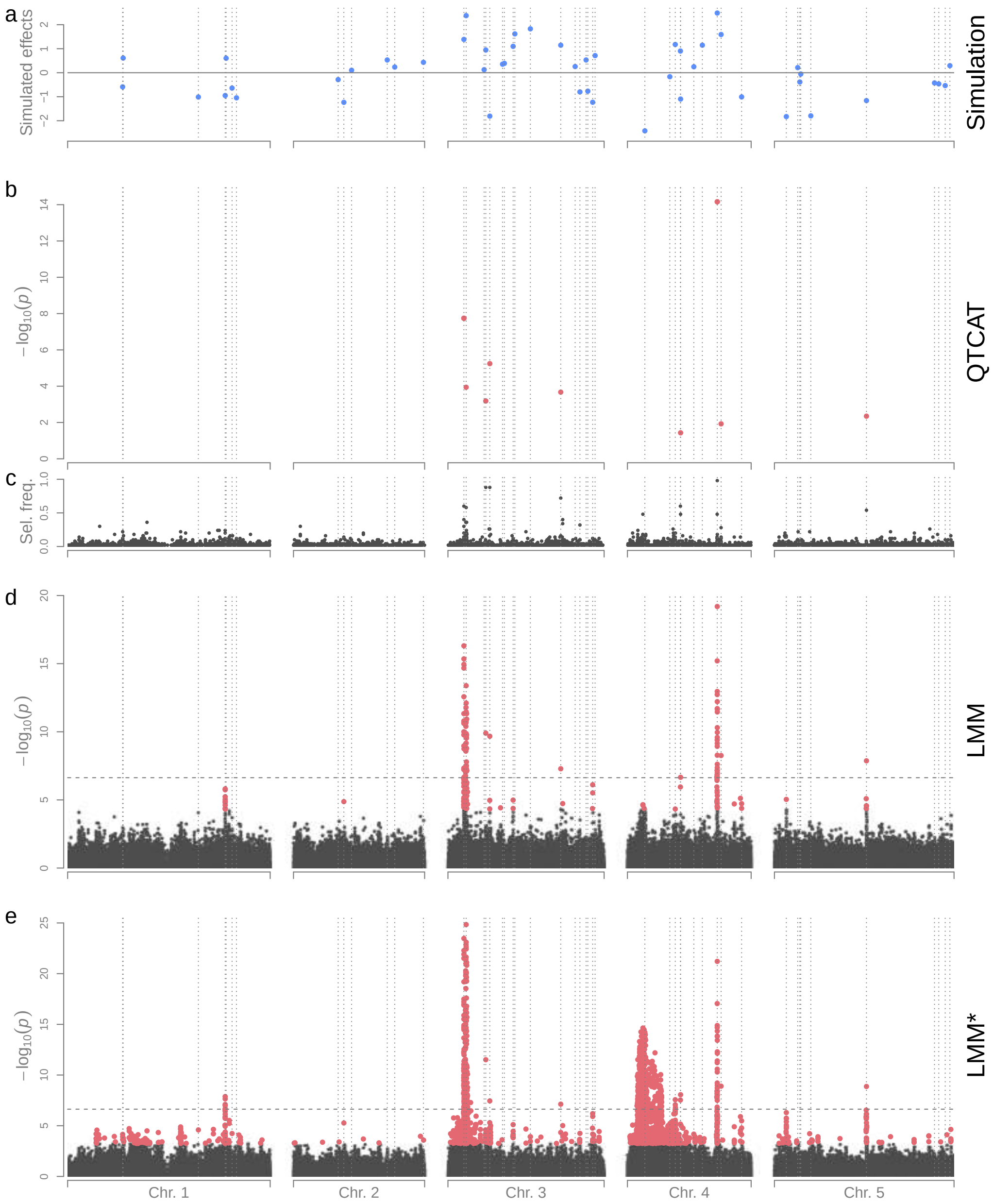

Supplementary Figure 114 Simulation of a GWA analysis based on a structured population with a heritability of 0.7 (run 14). (**a**) Simulation of 50 effects randomly drawn from a normal distribution and assigned to random markers. Markers with effect are highlighted with dashed lines. (**b**) Significant QTCs found by QTCAT. (**c**) LASSO selection frequency for each marker during the 50 iterations of QTCAT. (**d**) Manhattan plot of the LMM analysis. The horizontal dashed line depicts the significance threshold when controlling the multiple testing with FWER, whereas the red markers are significantly associated when controlling with FDR. (**e**) The Manhattan plot of the LMM* analysis. GRM was estimated without markers on the chromosome of the actual testing position. The results are shown as in (d).

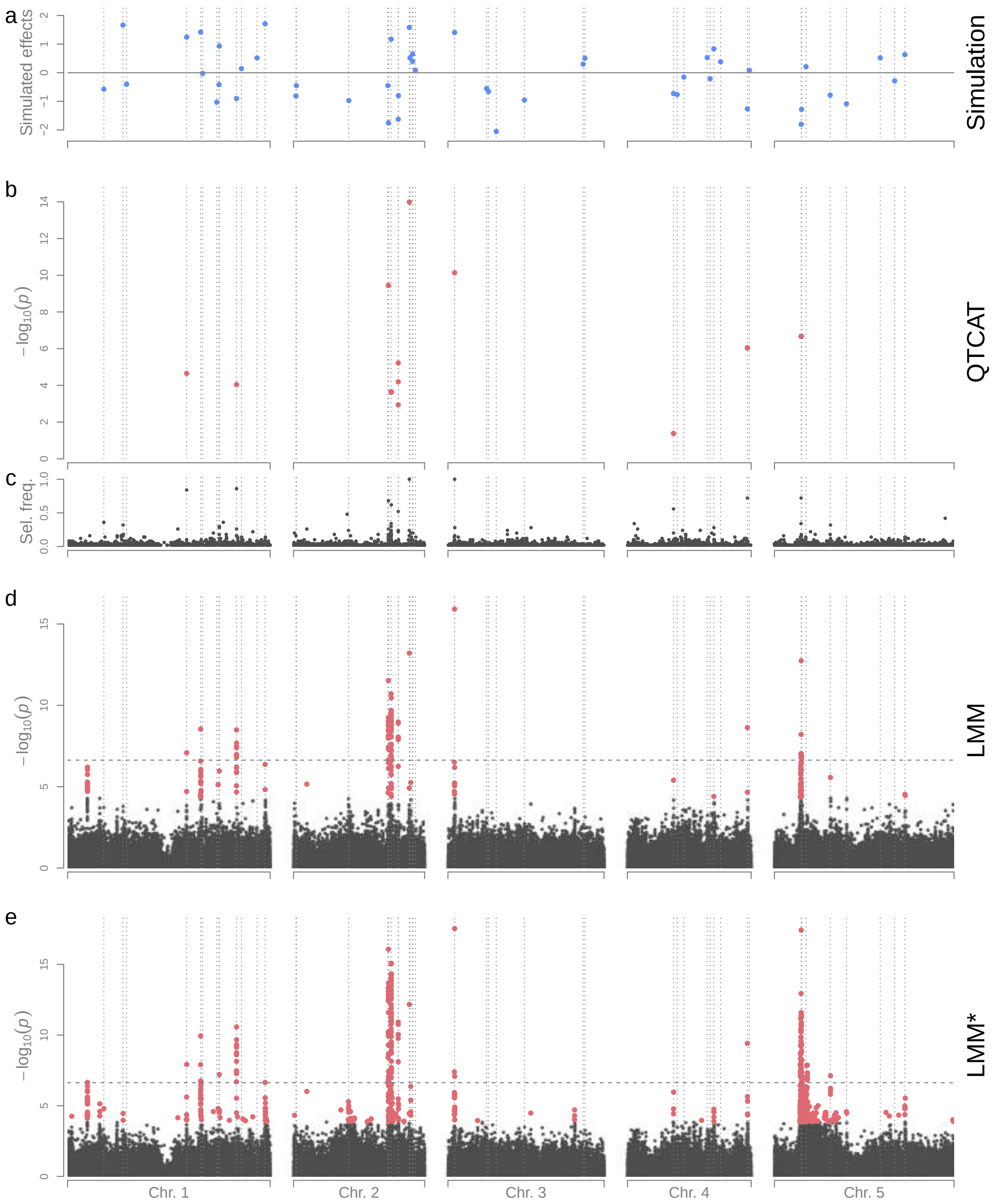

Supplementary Figure 115 Simulation of a GWA analysis based on a structured population with a heritability of 0.7 (run 15). (**a**) Simulation of 50 effects randomly drawn from a normal distribution and assigned to random markers. Markers with effect are highlighted with dashed lines. (**b**) Significant QTCs found by QTCAT. (**c**) LASSO selection frequency for each marker during the 50 iterations of QTCAT. (**d**) Manhattan plot of the LMM analysis. The horizontal dashed line depicts the significance threshold when controlling the multiple testing with FWER, whereas the red markers are significantly associated when controlling with FDR. (**e**) The Manhattan plot of the LMM* analysis. GRM was estimated without markers on the chromosome of the actual testing position. The results are shown as in (d).

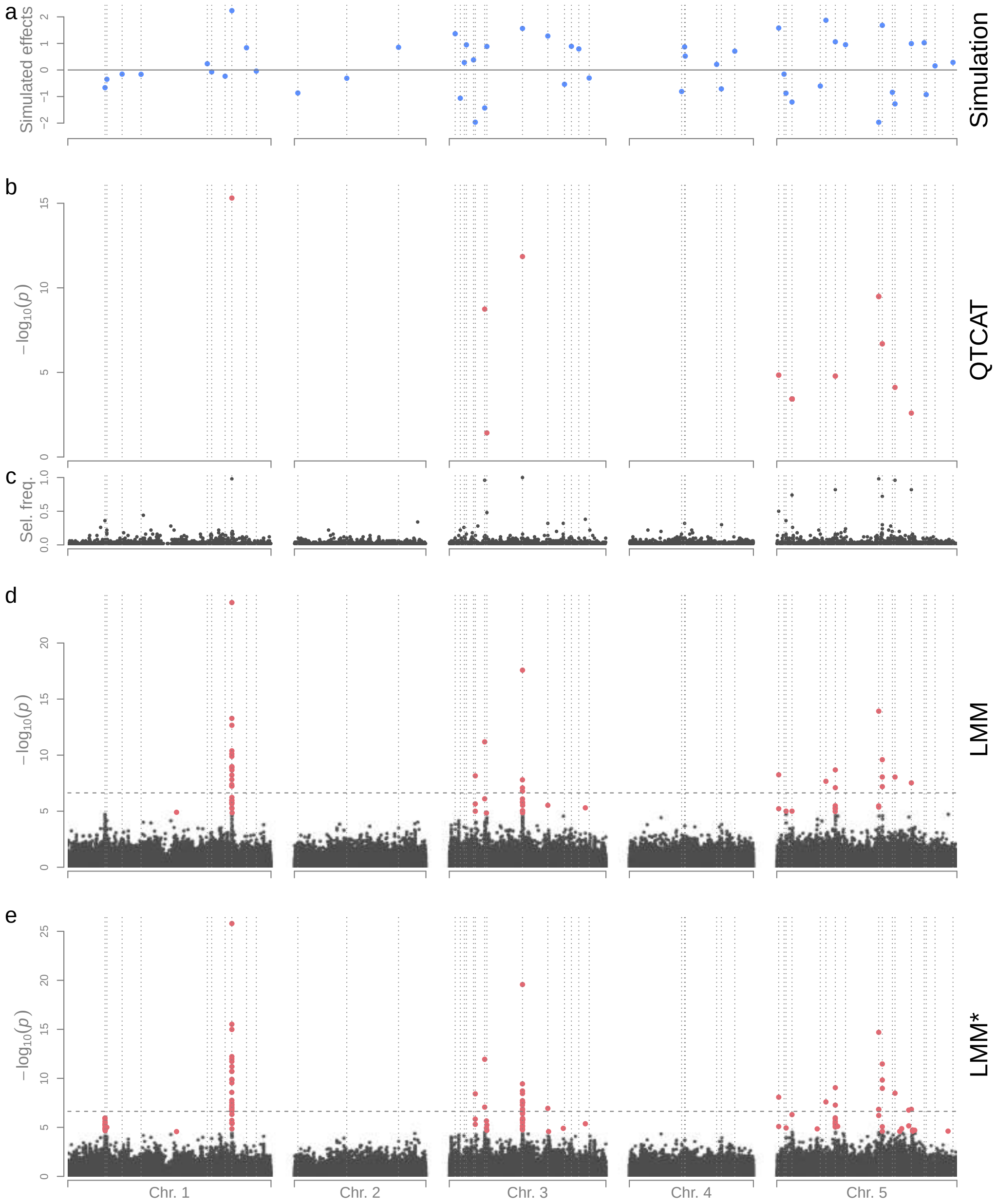

Supplementary Figure 116 Simulation of a GWA analysis based on a structured population with a heritability of 0.7 (run 16). (**a**) Simulation of 50 effects randomly drawn from a normal distribution and assigned to random markers. Markers with effect are highlighted with dashed lines. (**b**) Significant QTCs found by QTCAT. (**c**) LASSO selection frequency for each marker during the 50 iterations of QTCAT. (**d**) Manhattan plot of the LMM analysis. The horizontal dashed line depicts the significance threshold when controlling the multiple testing with FWER, whereas the red markers are significantly associated when controlling with FDR. (**e**) The Manhattan plot of the LMM* analysis. GRM was estimated without markers on the chromosome of the actual testing position. The results are shown as in (d).

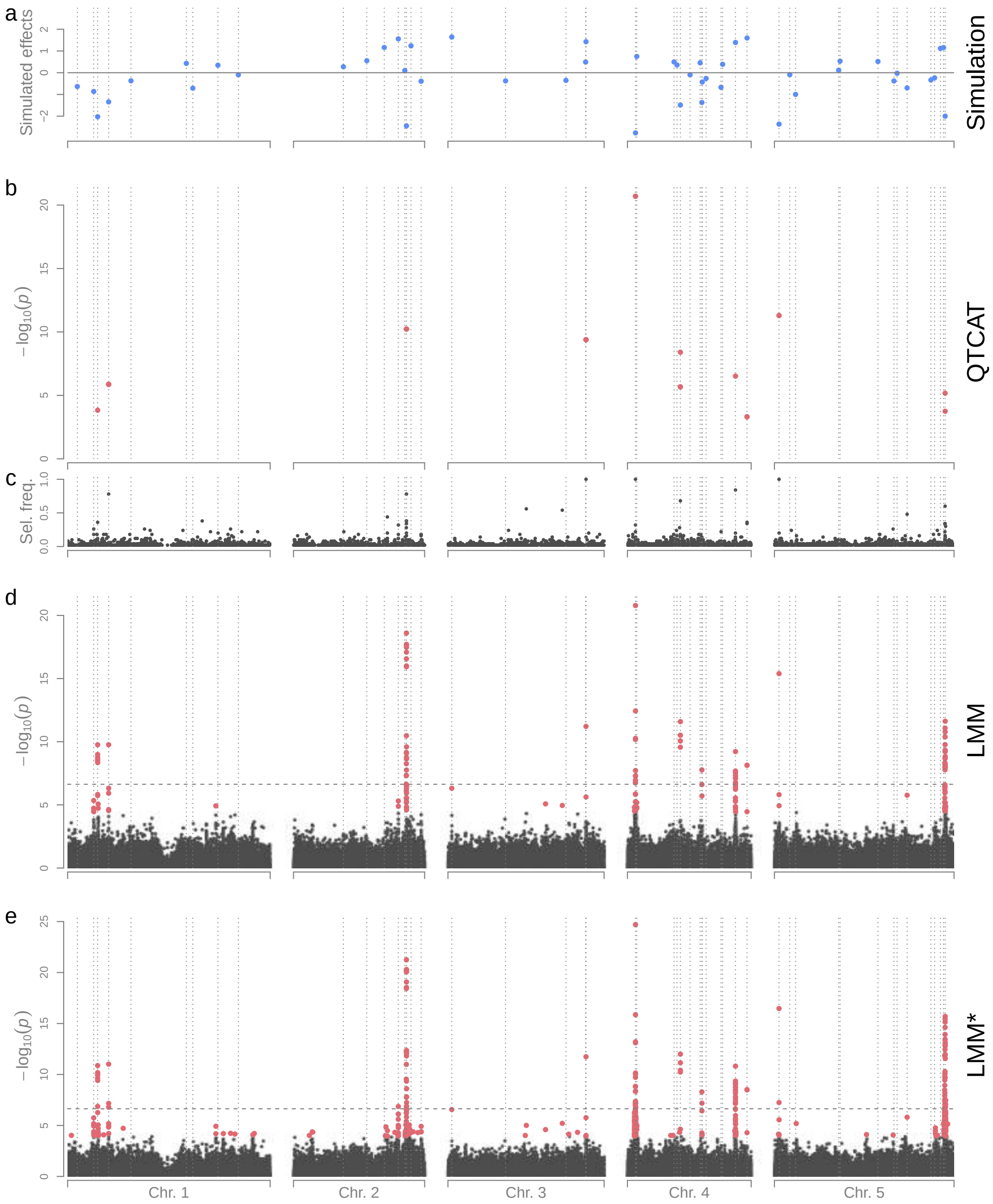

Supplementary Figure 117 Simulation of a GWA analysis based on a structured population with a heritability of 0.7 (run 17). (**a**) Simulation of 50 effects randomly drawn from a normal distribution and assigned to random markers. Markers with effect are highlighted with dashed lines. (**b**) Significant QTCs found by QTCAT. (**c**) LASSO selection frequency for each marker during the 50 iterations of QTCAT. (**d**) Manhattan plot of the LMM analysis. The horizontal dashed line depicts the significance threshold when controlling the multiple testing with FWER, whereas the red markers are significantly associated when controlling with FDR. (**e**) The Manhattan plot of the LMM* analysis. GRM was estimated without markers on the chromosome of the actual testing position. The results are shown as in (d).

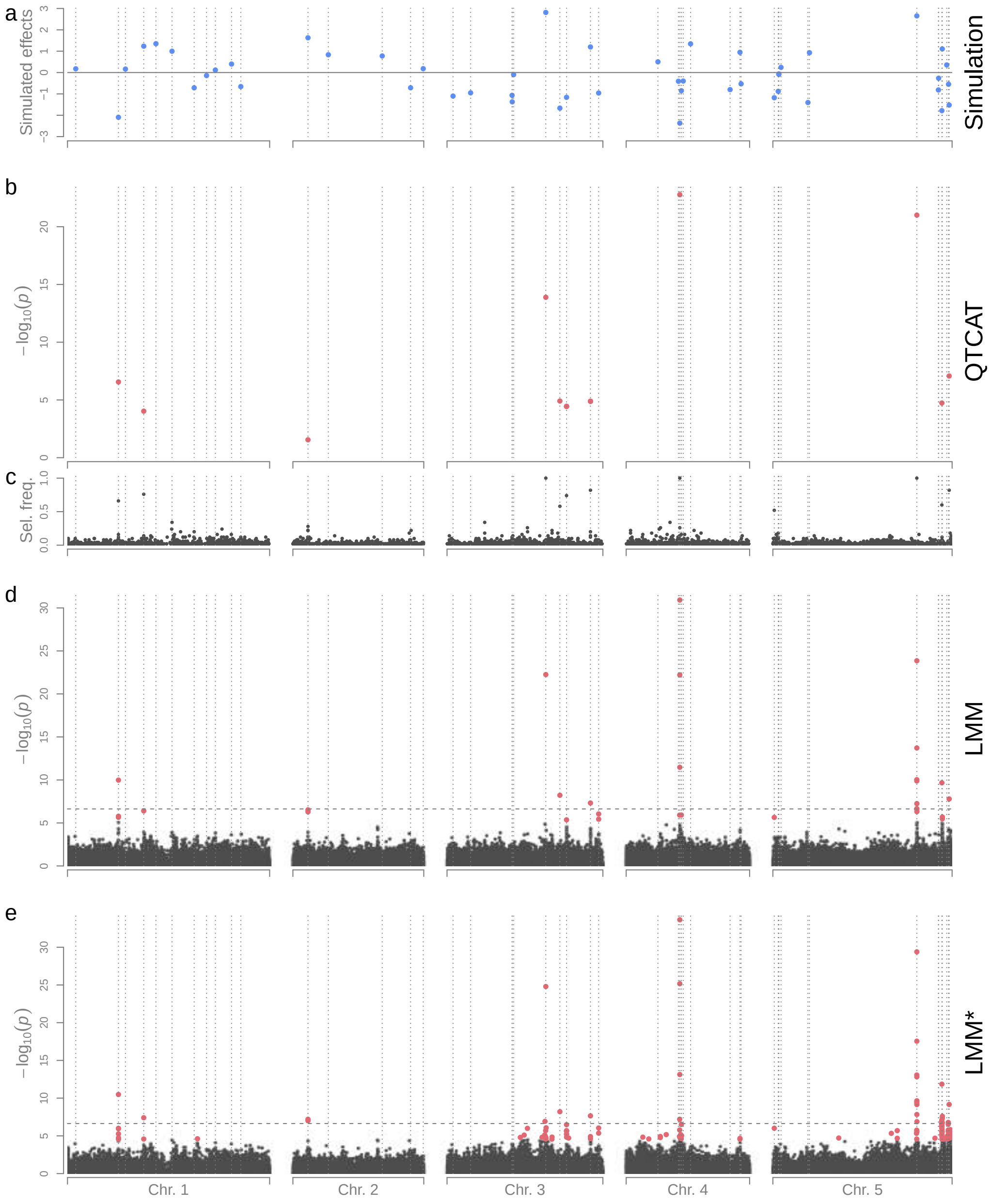

Supplementary Figure 118 Simulation of a GWA analysis based on a structured population with a heritability of 0.7 (run 18). (**a**) Simulation of 50 effects randomly drawn from a normal distribution and assigned to random markers. Markers with effect are highlighted with dashed lines. (**b**) Significant QTCs found by QTCAT. (**c**) LASSO selection frequency for each marker during the 50 iterations of QTCAT. (**d**) Manhattan plot of the LMM analysis. The horizontal dashed line depicts the significance threshold when controlling the multiple testing with FWER, whereas the red markers are significantly associated when controlling with FDR. (**e**) The Manhattan plot of the LMM* analysis. GRM was estimated without markers on the chromosome of the actual testing position. The results are shown as in (d).

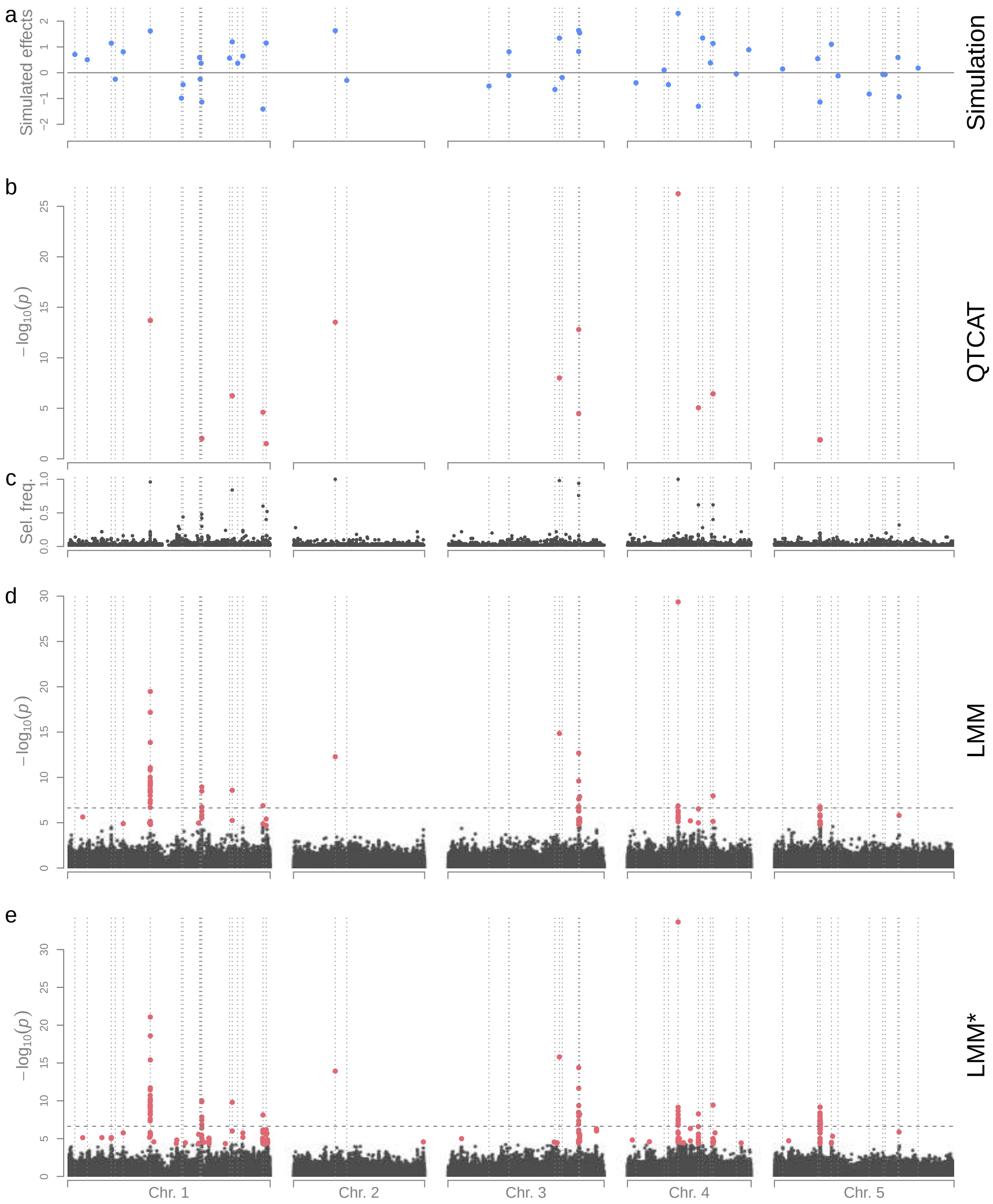

Supplementary Figure 119 Simulation of a GWA analysis based on a structured population with a heritability of 0.7 (run 19). (**a**) Simulation of 50 effects randomly drawn from a normal distribution and assigned to random markers. Markers with effect are highlighted with dashed lines. (**b**) Significant QTCs found by QTCAT. (**c**) LASSO selection frequency for each marker during the 50 iterations of QTCAT. (**d**) Manhattan plot of the LMM analysis. The horizontal dashed line depicts the significance threshold when controlling the multiple testing with FWER, whereas the red markers are significantly associated when controlling with FDR. (**e**) The Manhattan plot of the LMM* analysis. GRM was estimated without markers on the chromosome of the actual testing position. The results are shown as in (d).

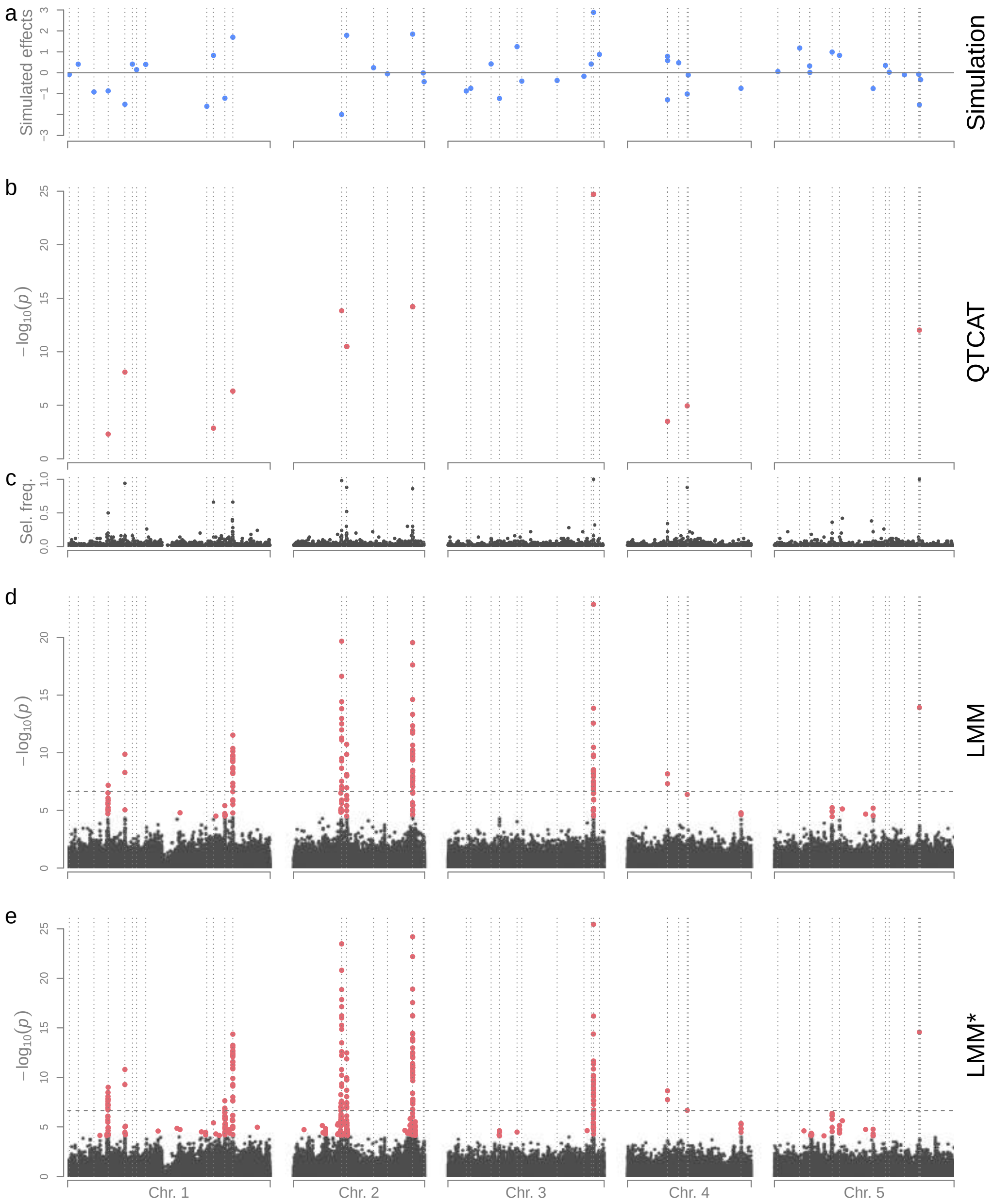

Supplementary Figure 120 Simulation of a GWA analysis based on a structured population with a heritability of 0.7 (run 20). (**a**) Simulation of 50 effects randomly drawn from a normal distribution and assigned to random markers. Markers with effect are highlighted with dashed lines. (**b**) Significant QTCs found by QTCAT. (**c**) LASSO selection frequency for each marker during the 50 iterations of QTCAT. (**d**) Manhattan plot of the LMM analysis. The horizontal dashed line depicts the significance threshold when controlling the multiple testing with FWER, whereas the red markers are significantly associated when controlling with FDR. (**e**) The Manhattan plot of the LMM* analysis. GRM was estimated without markers on the chromosome of the actual testing position. The results are shown as in (d).

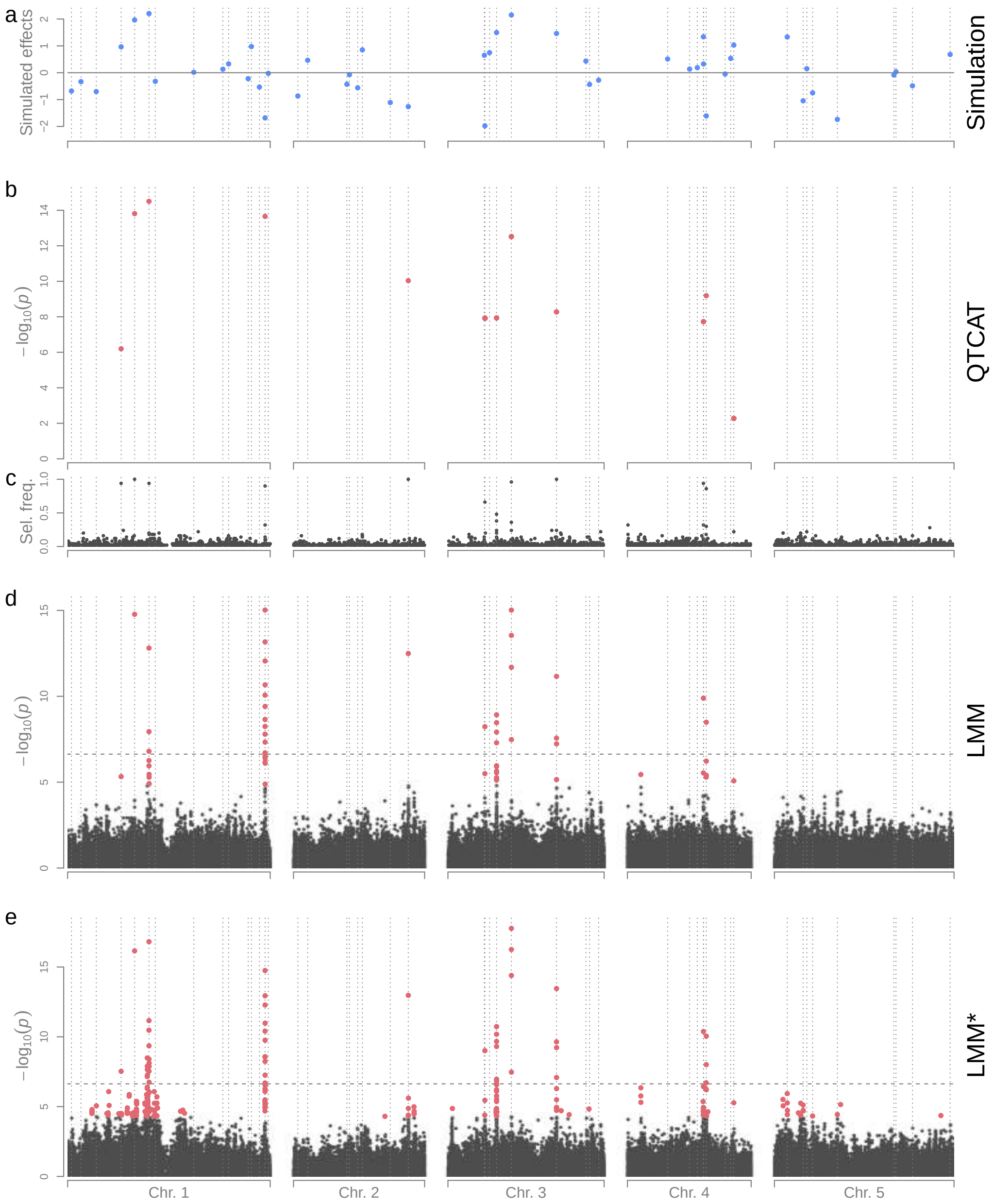

Supplementary Figure 121 Simulation of a GWA analysis based on a structured population with a heritability of 0.7 (run 21). (**a**) Simulation of 50 effects randomly drawn from a normal distribution and assigned to random markers. Markers with effect are highlighted with dashed lines. (**b**) Significant QTCs found by QTCAT. (**c**) LASSO selection frequency for each marker during the 50 iterations of QTCAT. (**d**) Manhattan plot of the LMM analysis. The horizontal dashed line depicts the significance threshold when controlling the multiple testing with FWER, whereas the red markers are significantly associated when controlling with FDR. (**e**) The Manhattan plot of the LMM* analysis. GRM was estimated without markers on the chromosome of the actual testing position. The results are shown as in (d).

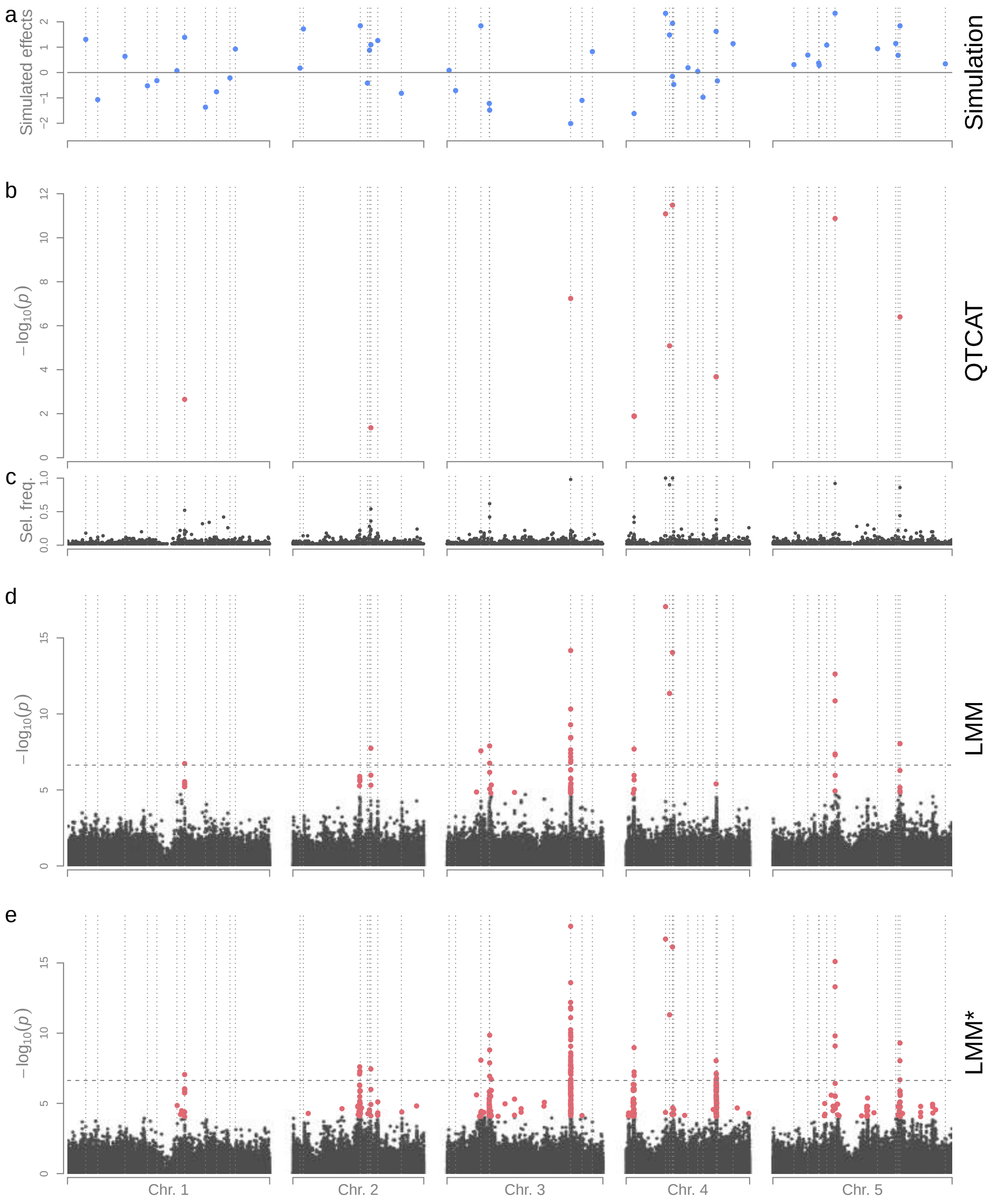

Supplementary Figure 122 Simulation of a GWA analysis based on a structured population with a heritability of 0.7 (run 22). (**a**) Simulation of 50 effects randomly drawn from a normal distribution and assigned to random markers. Markers with effect are highlighted with dashed lines. (**b**) Significant QTCs found by QTCAT. (**c**) LASSO selection frequency for each marker during the 50 iterations of QTCAT. (**d**) Manhattan plot of the LMM analysis. The horizontal dashed line depicts the significance threshold when controlling the multiple testing with FWER, whereas the red markers are significantly associated when controlling with FDR. (**e**) The Manhattan plot of the LMM* analysis. GRM was estimated without markers on the chromosome of the actual testing position. The results are shown as in (d).

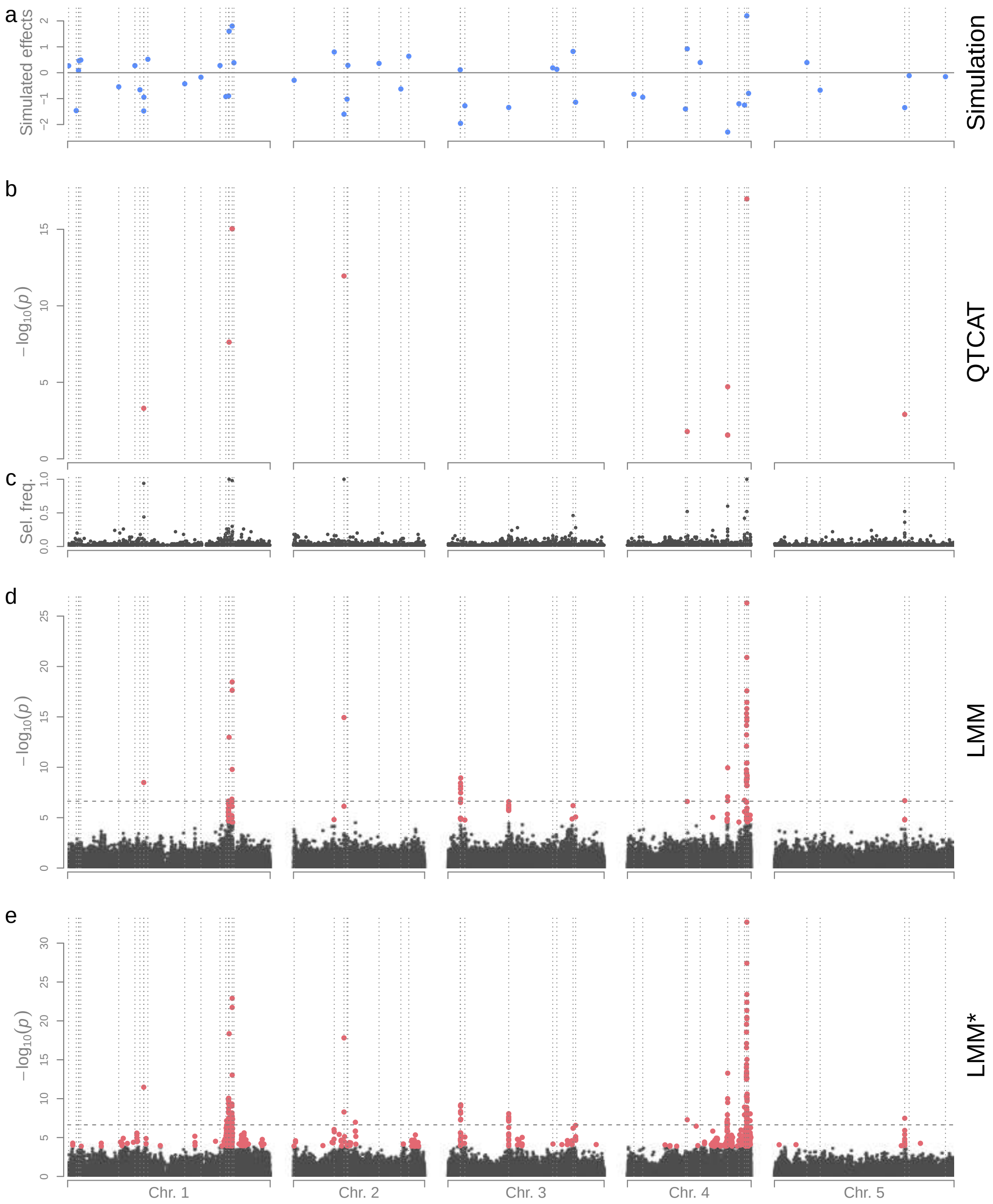

Supplementary Figure 123 Simulation of a GWA analysis based on a structured population with a heritability of 0.7 (run 23). (**a**) Simulation of 50 effects randomly drawn from a normal distribution and assigned to random markers. Markers with effect are highlighted with dashed lines. (**b**) Significant QTCs found by QTCAT. (**c**) LASSO selection frequency for each marker during the 50 iterations of QTCAT. (**d**) Manhattan plot of the LMM analysis. The horizontal dashed line depicts the significance threshold when controlling the multiple testing with FWER, whereas the red markers are significantly associated when controlling with FDR. (**e**) The Manhattan plot of the LMM* analysis. GRM was estimated without markers on the chromosome of the actual testing position. The results are shown as in (d).

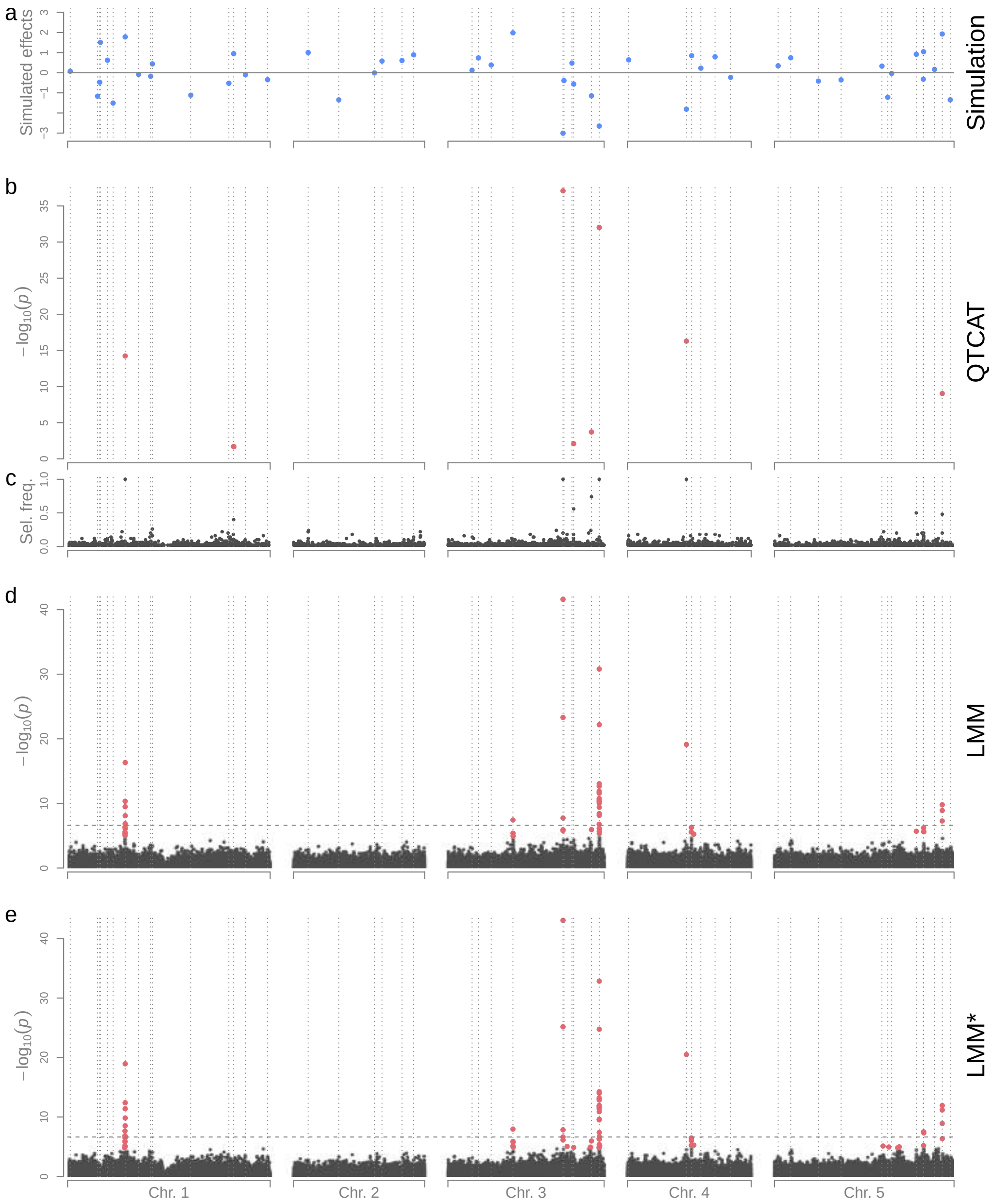

Supplementary Figure 124 Simulation of a GWA analysis based on a structured population with a heritability of 0.7 (run 24). (**a**) Simulation of 50 effects randomly drawn from a normal distribution and assigned to random markers. Markers with effect are highlighted with dashed lines. (**b**) Significant QTCs found by QTCAT. (**c**) LASSO selection frequency for each marker during the 50 iterations of QTCAT. (**d**) Manhattan plot of the LMM analysis. The horizontal dashed line depicts the significance threshold when controlling the multiple testing with FWER, whereas the red markers are significantly associated when controlling with FDR. (**e**) The Manhattan plot of the LMM* analysis. GRM was estimated without markers on the chromosome of the actual testing position. The results are shown as in (d).

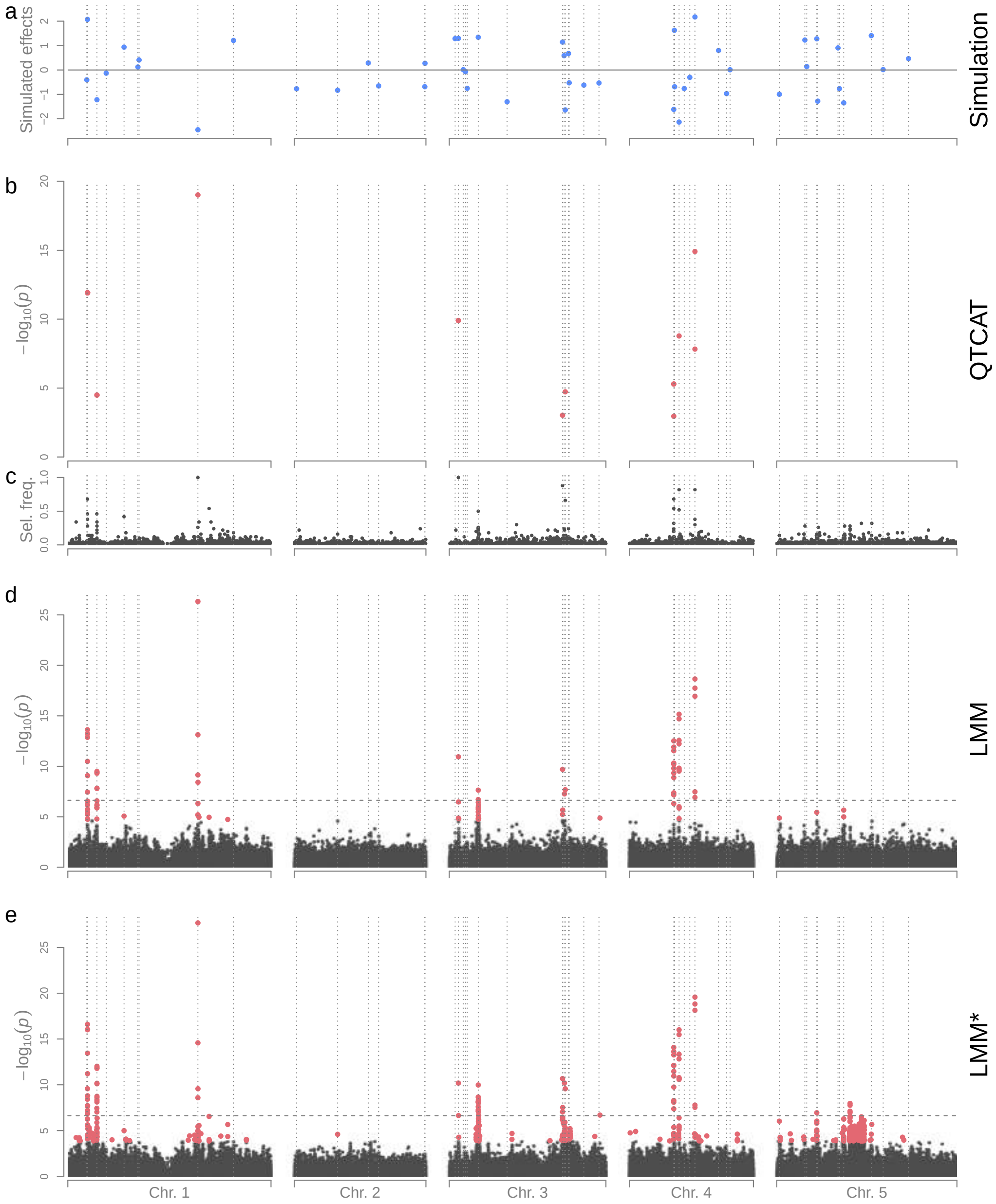

Supplementary Figure 125 Simulation of a GWA analysis based on a structured population with a heritability of 0.7 (run 25). (**a**) Simulation of 50 effects randomly drawn from a normal distribution and assigned to random markers. Markers with effect are highlighted with dashed lines. (**b**) Significant QTCs found by QTCAT. (**c**) LASSO selection frequency for each marker during the 50 iterations of QTCAT. (**d**) Manhattan plot of the LMM analysis. The horizontal dashed line depicts the significance threshold when controlling the multiple testing with FWER, whereas the red markers are significantly associated when controlling with FDR. (**e**) The Manhattan plot of the LMM* analysis. GRM was estimated without markers on the chromosome of the actual testing position. The results are shown as in (d).

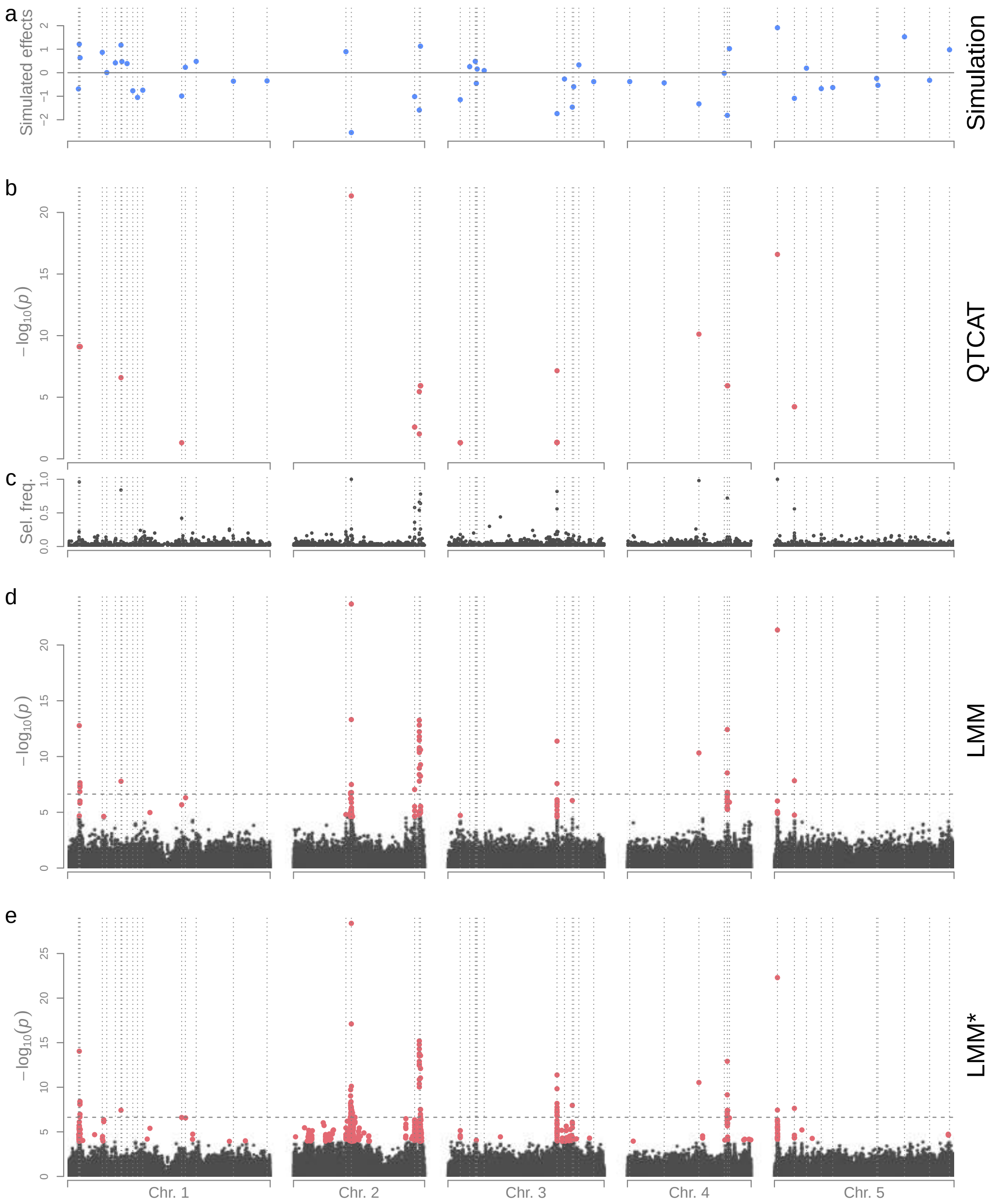

Supplementary Figure 126 Simulation of a GWA analysis based on a structured population with a heritability of 0.7 (run 26). (**a**) Simulation of 50 effects randomly drawn from a normal distribution and assigned to random markers. Markers with effect are highlighted with dashed lines. (**b**) Significant QTCs found by QTCAT. (**c**) LASSO selection frequency for each marker during the 50 iterations of QTCAT. (**d**) Manhattan plot of the LMM analysis. The horizontal dashed line depicts the significance threshold when controlling the multiple testing with FWER, whereas the red markers are significantly associated when controlling with FDR. (**e**) The Manhattan plot of the LMM* analysis. GRM was estimated without markers on the chromosome of the actual testing position. The results are shown as in (d).

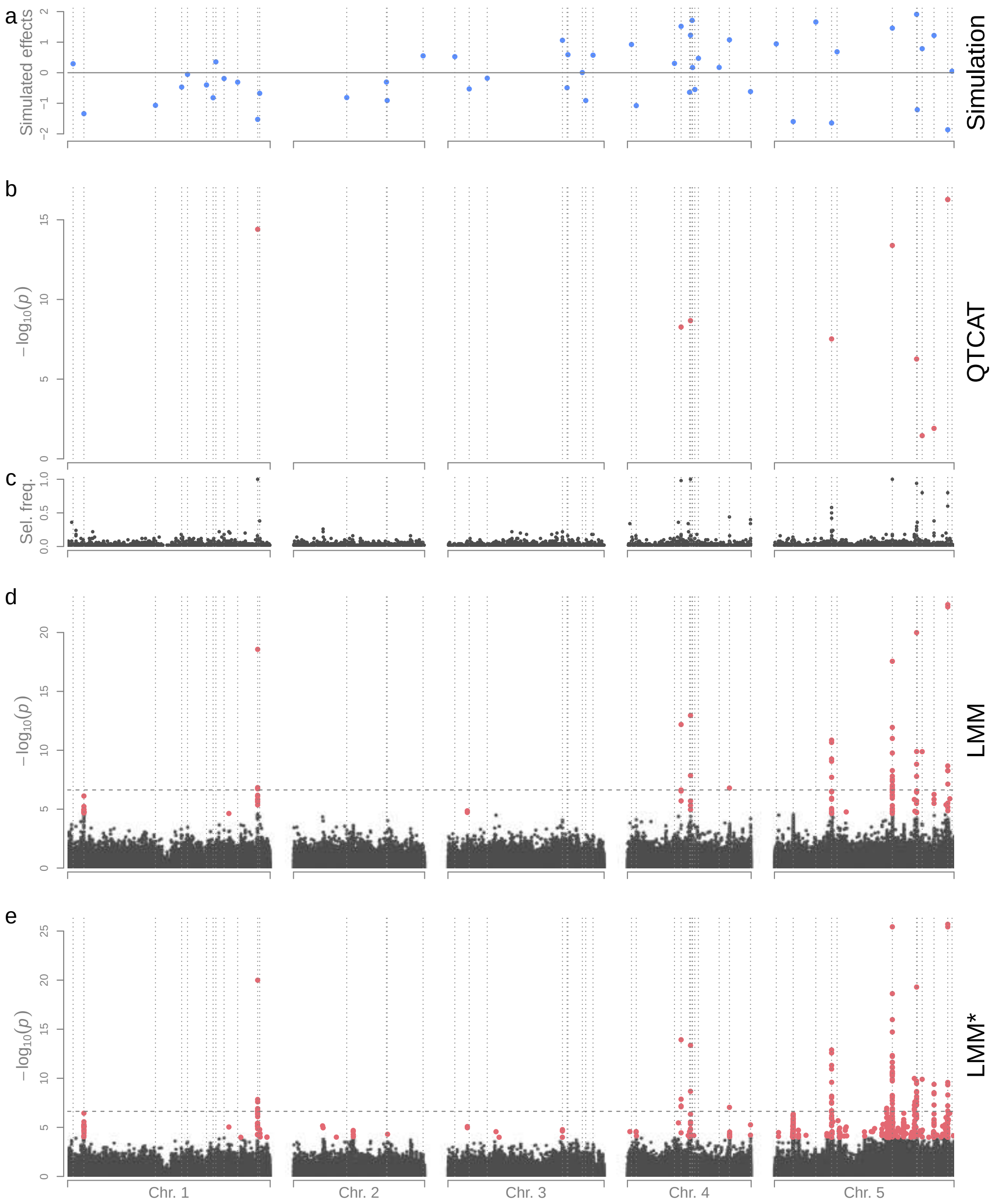

Supplementary Figure 127 Simulation of a GWA analysis based on a structured population with a heritability of 0.7 (run 27). (**a**) Simulation of 50 effects randomly drawn from a normal distribution and assigned to random markers. Markers with effect are highlighted with dashed lines. (**b**) Significant QTCs found by QTCAT. (**c**) LASSO selection frequency for each marker during the 50 iterations of QTCAT. (**d**) Manhattan plot of the LMM analysis. The horizontal dashed line depicts the significance threshold when controlling the multiple testing with FWER, whereas the red markers are significantly associated when controlling with FDR. (**e**) The Manhattan plot of the LMM* analysis. GRM was estimated without markers on the chromosome of the actual testing position. The results are shown as in (d).

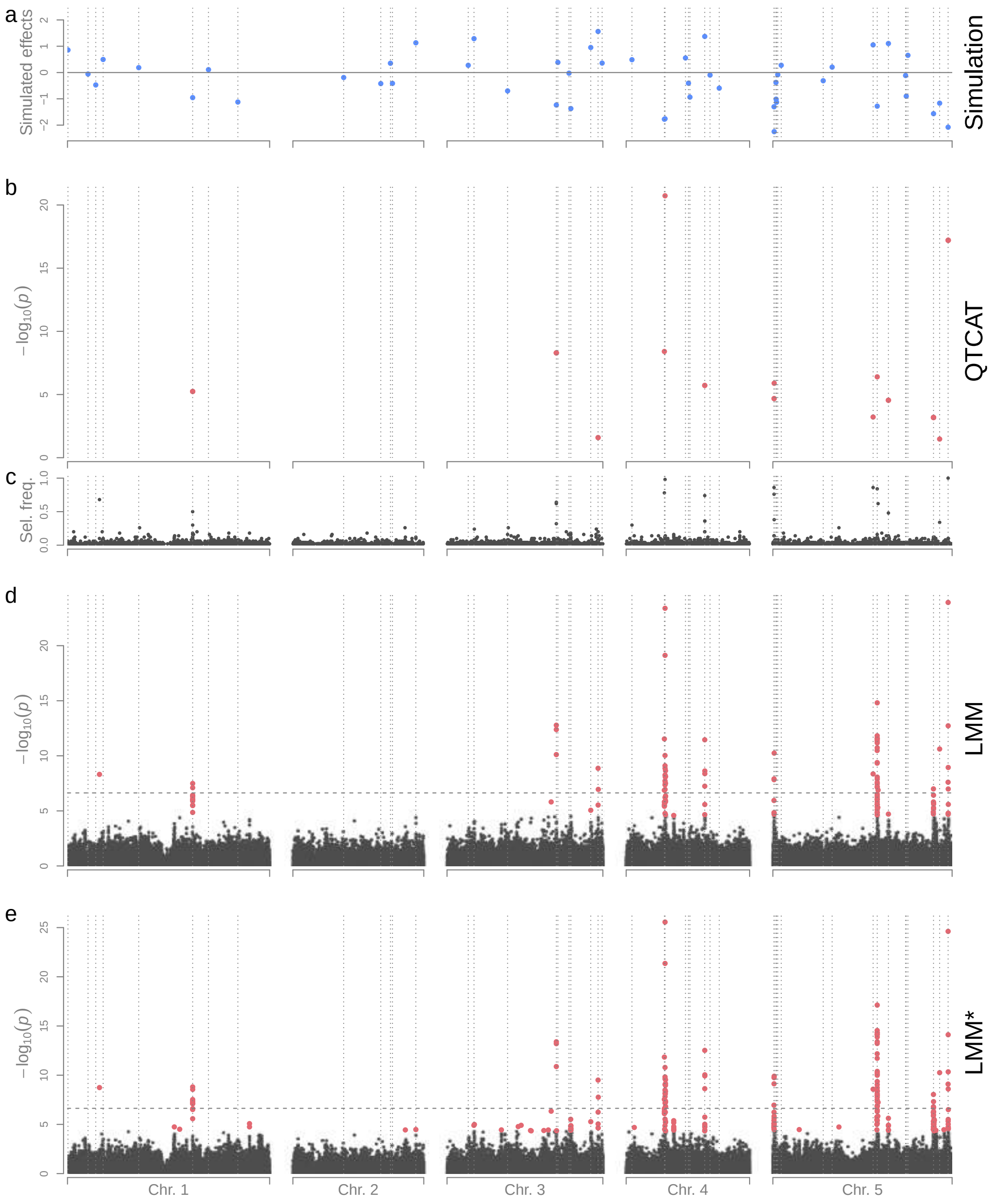

Supplementary Figure 128 Simulation of a GWA analysis based on a structured population with a heritability of 0.7 (run 28). (**a**) Simulation of 50 effects randomly drawn from a normal distribution and assigned to random markers. Markers with effect are highlighted with dashed lines. (**b**) Significant QTCs found by QTCAT. (**c**) LASSO selection frequency for each marker during the 50 iterations of QTCAT. (**d**) Manhattan plot of the LMM analysis. The horizontal dashed line depicts the significance threshold when controlling the multiple testing with FWER, whereas the red markers are significantly associated when controlling with FDR. (**e**) The Manhattan plot of the LMM* analysis. GRM was estimated without markers on the chromosome of the actual testing position. The results are shown as in (d).

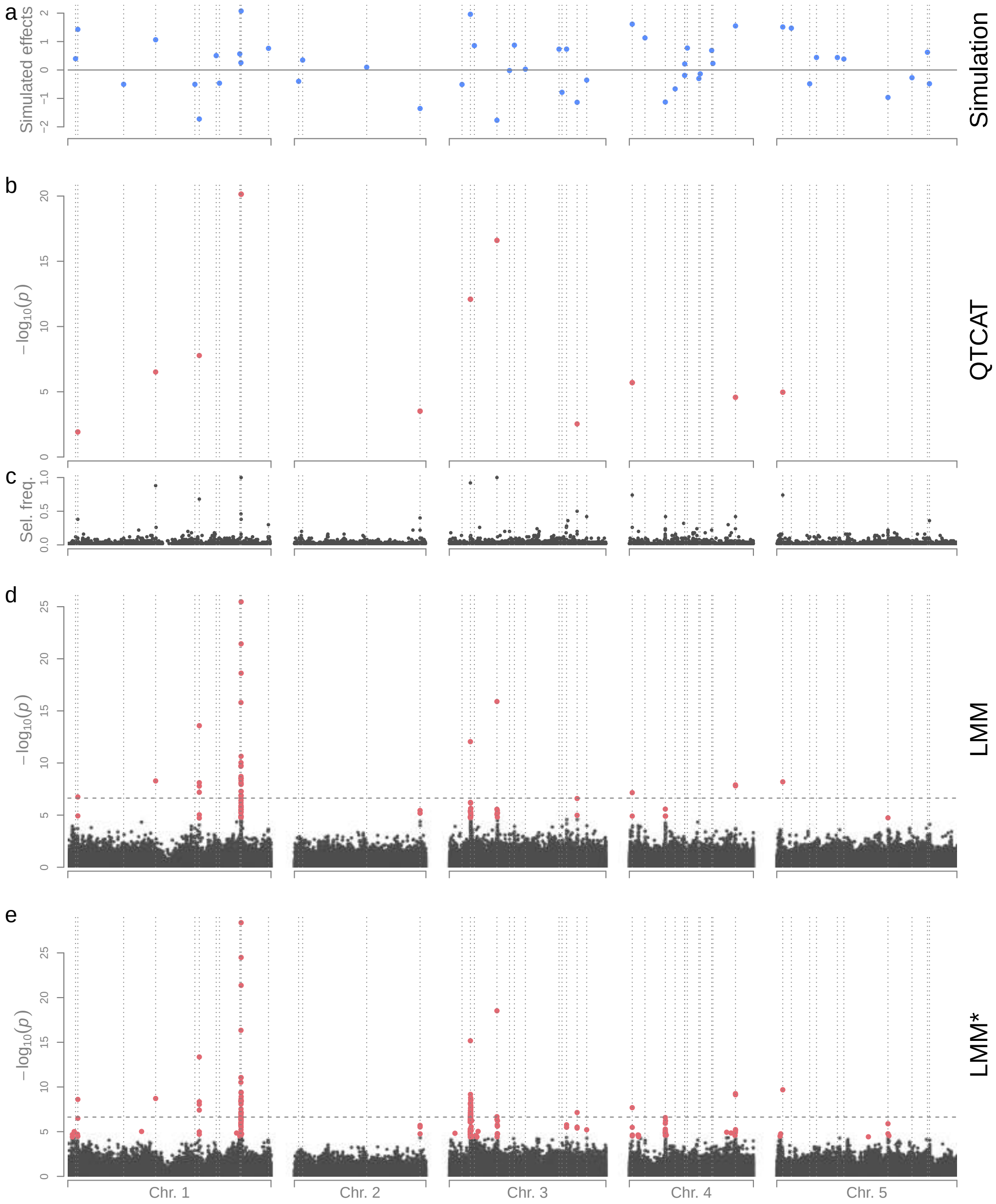

Supplementary Figure 129 Simulation of a GWA analysis based on a structured population with a heritability of 0.7 (run 29). (**a**) Simulation of 50 effects randomly drawn from a normal distribution and assigned to random markers. Markers with effect are highlighted with dashed lines. (**b**) Significant QTCs found by QTCAT. (**c**) LASSO selection frequency for each marker during the 50 iterations of QTCAT. (**d**) Manhattan plot of the LMM analysis. The horizontal dashed line depicts the significance threshold when controlling the multiple testing with FWER, whereas the red markers are significantly associated when controlling with FDR. (**e**) The Manhattan plot of the LMM* analysis. GRM was estimated without markers on the chromosome of the actual testing position. The results are shown as in (d).

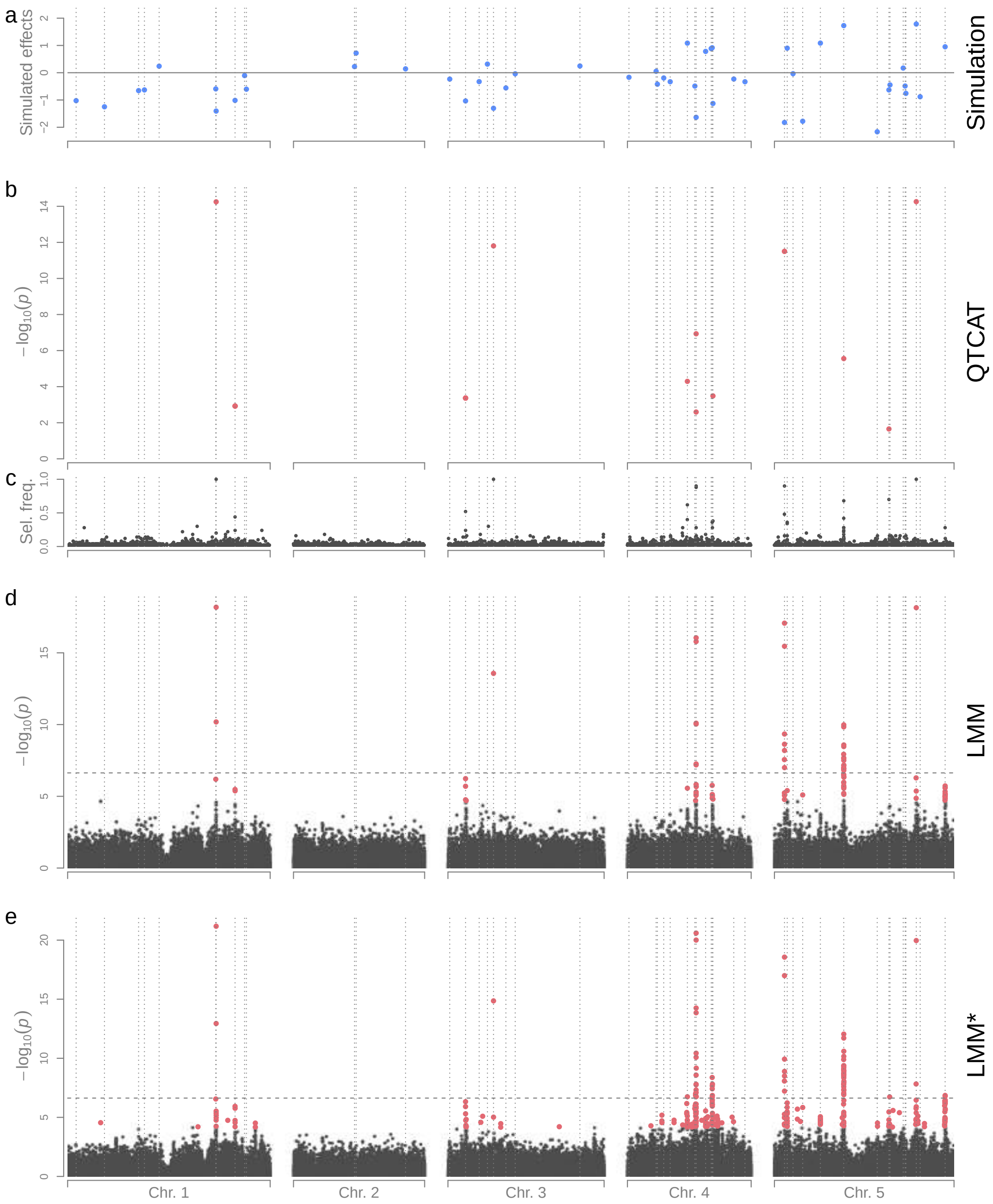

Supplementary Figure 130 Simulation of a GWA analysis based on a structured population with a heritability of 0.7 (run 30). (**a**) Simulation of 50 effects randomly drawn from a normal distribution and assigned to random markers. Markers with effect are highlighted with dashed lines. (**b**) Significant QTCs found by QTCAT. (**c**) LASSO selection frequency for each marker during the 50 iterations of QTCAT. (**d**) Manhattan plot of the LMM analysis. The horizontal dashed line depicts the significance threshold when controlling the multiple testing with FWER, whereas the red markers are significantly associated when controlling with FDR. (**e**) The Manhattan plot of the LMM* analysis. GRM was estimated without markers on the chromosome of the actual testing position. The results are shown as in (d).

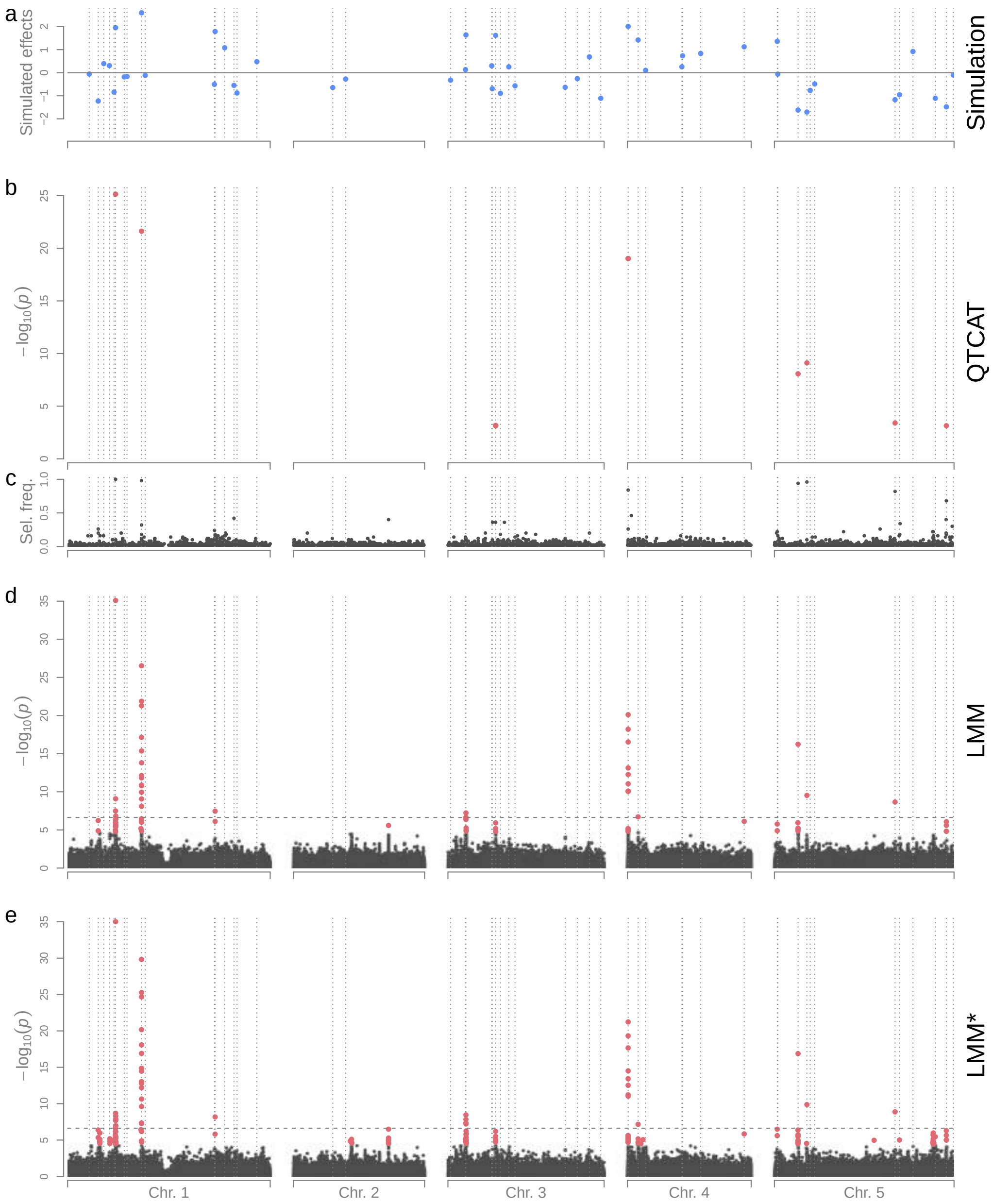

Supplementary Figure 131 Simulation of a GWA analysis based on a structured population with a heritability of 0.7 (run 31). (**a**) Simulation of 50 effects randomly drawn from a normal distribution and assigned to random markers. Markers with effect are highlighted with dashed lines. (**b**) Significant QTCs found by QTCAT. (**c**) LASSO selection frequency for each marker during the 50 iterations of QTCAT. (**d**) Manhattan plot of the LMM analysis. The horizontal dashed line depicts the significance threshold when controlling the multiple testing with FWER, whereas the red markers are significantly associated when controlling with FDR. (**e**) The Manhattan plot of the LMM* analysis. GRM was estimated without markers on the chromosome of the actual testing position. The results are shown as in (d).

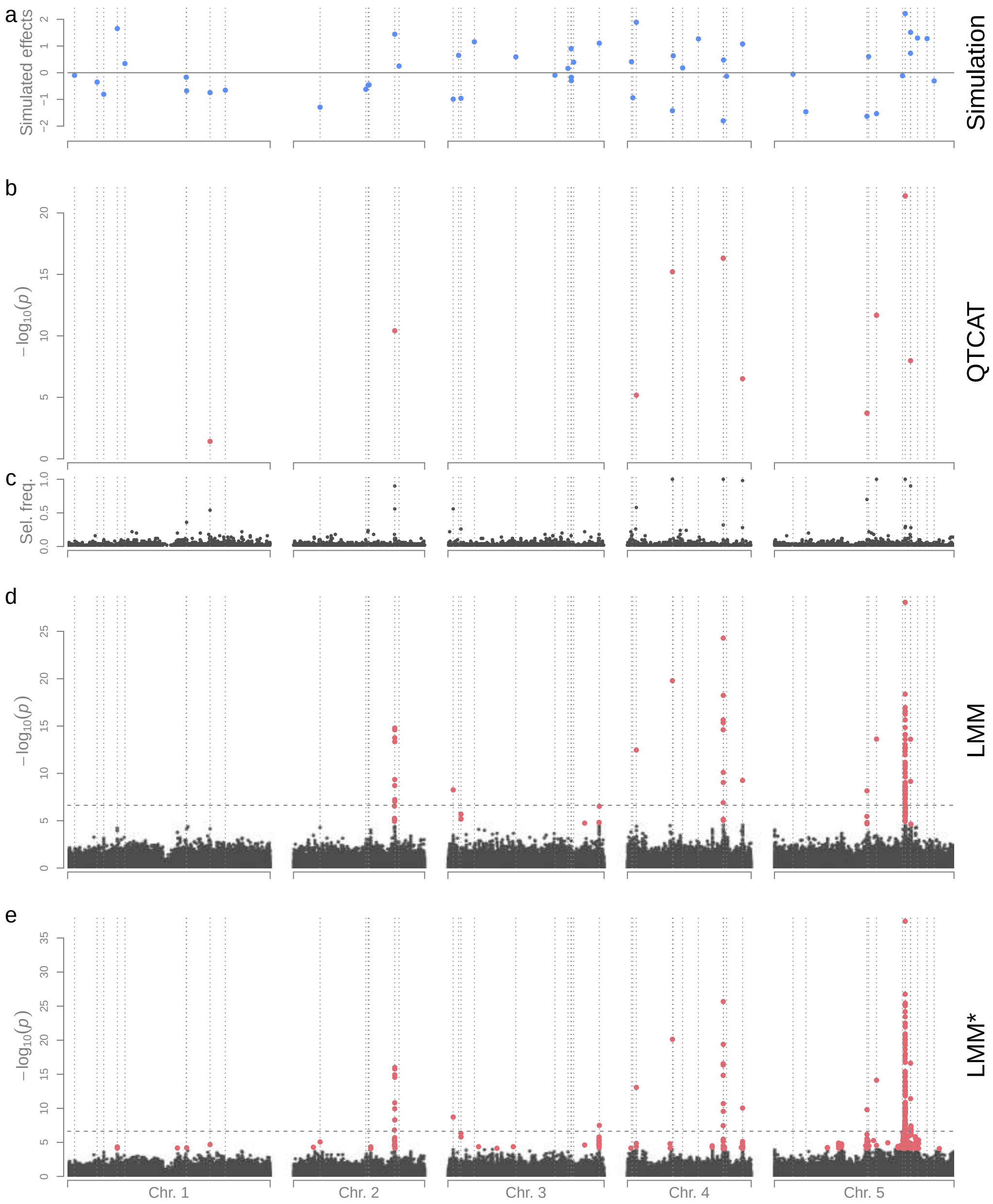

Supplementary Figure 132 Simulation of a GWA analysis based on a structured population with a heritability of 0.7 (run 32). (**a**) Simulation of 50 effects randomly drawn from a normal distribution and assigned to random markers. Markers with effect are highlighted with dashed lines. (**b**) Significant QTCs found by QTCAT. (**c**) LASSO selection frequency for each marker during the 50 iterations of QTCAT. (**d**) Manhattan plot of the LMM analysis. The horizontal dashed line depicts the significance threshold when controlling the multiple testing with FWER, whereas the red markers are significantly associated when controlling with FDR. (**e**) The Manhattan plot of the LMM* analysis. GRM was estimated without markers on the chromosome of the actual testing position. The results are shown as in (d).

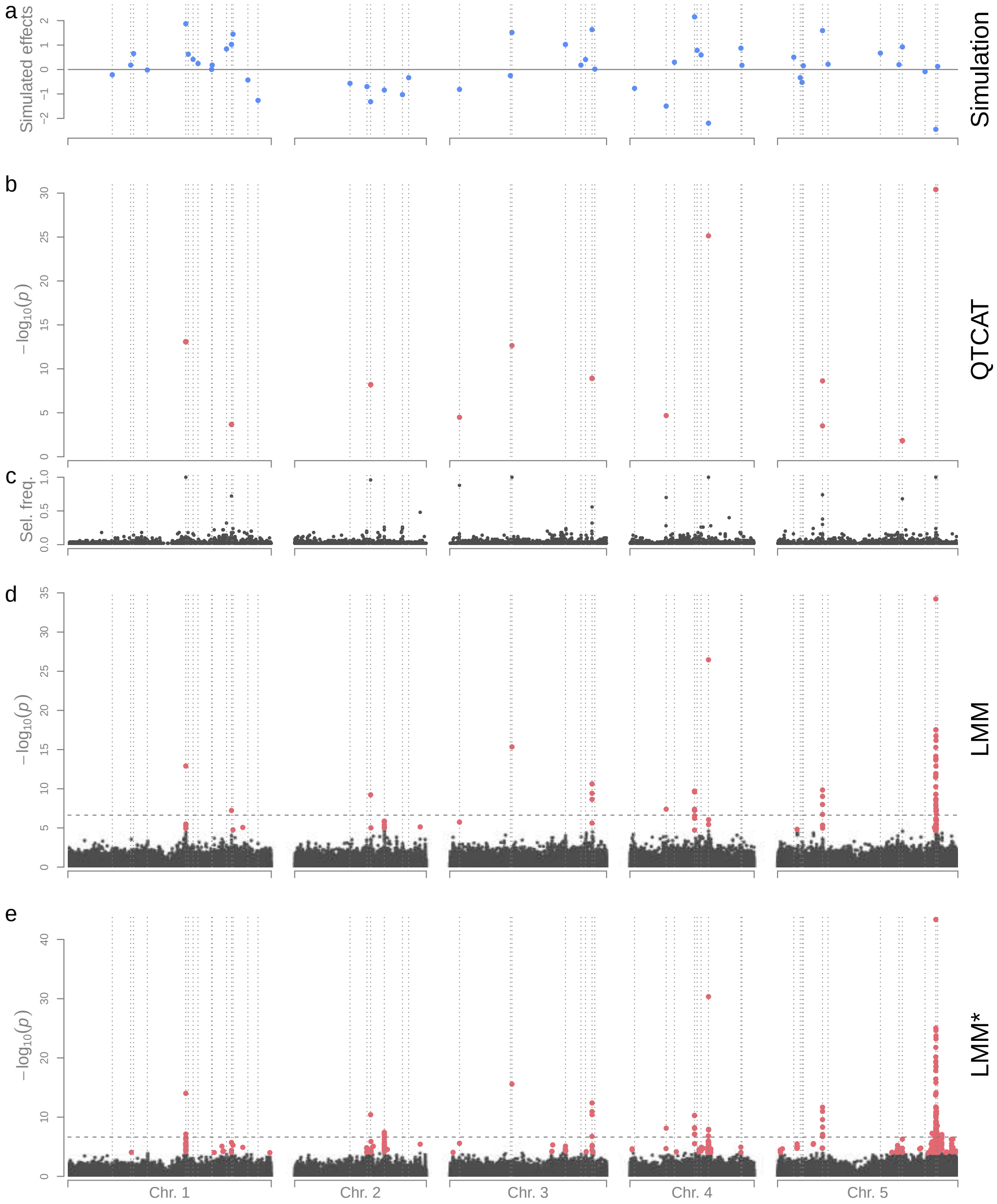

Supplementary Figure 133 Simulation of a GWA analysis based on a structured population with a heritability of 0.7 (run 33). (**a**) Simulation of 50 effects randomly drawn from a normal distribution and assigned to random markers. Markers with effect are highlighted with dashed lines. (**b**) Significant QTCs found by QTCAT. (**c**) LASSO selection frequency for each marker during the 50 iterations of QTCAT. (**d**) Manhattan plot of the LMM analysis. The horizontal dashed line depicts the significance threshold when controlling the multiple testing with FWER, whereas the red markers are significantly associated when controlling with FDR. (**e**) The Manhattan plot of the LMM* analysis. GRM was estimated without markers on the chromosome of the actual testing position. The results are shown as in (d).

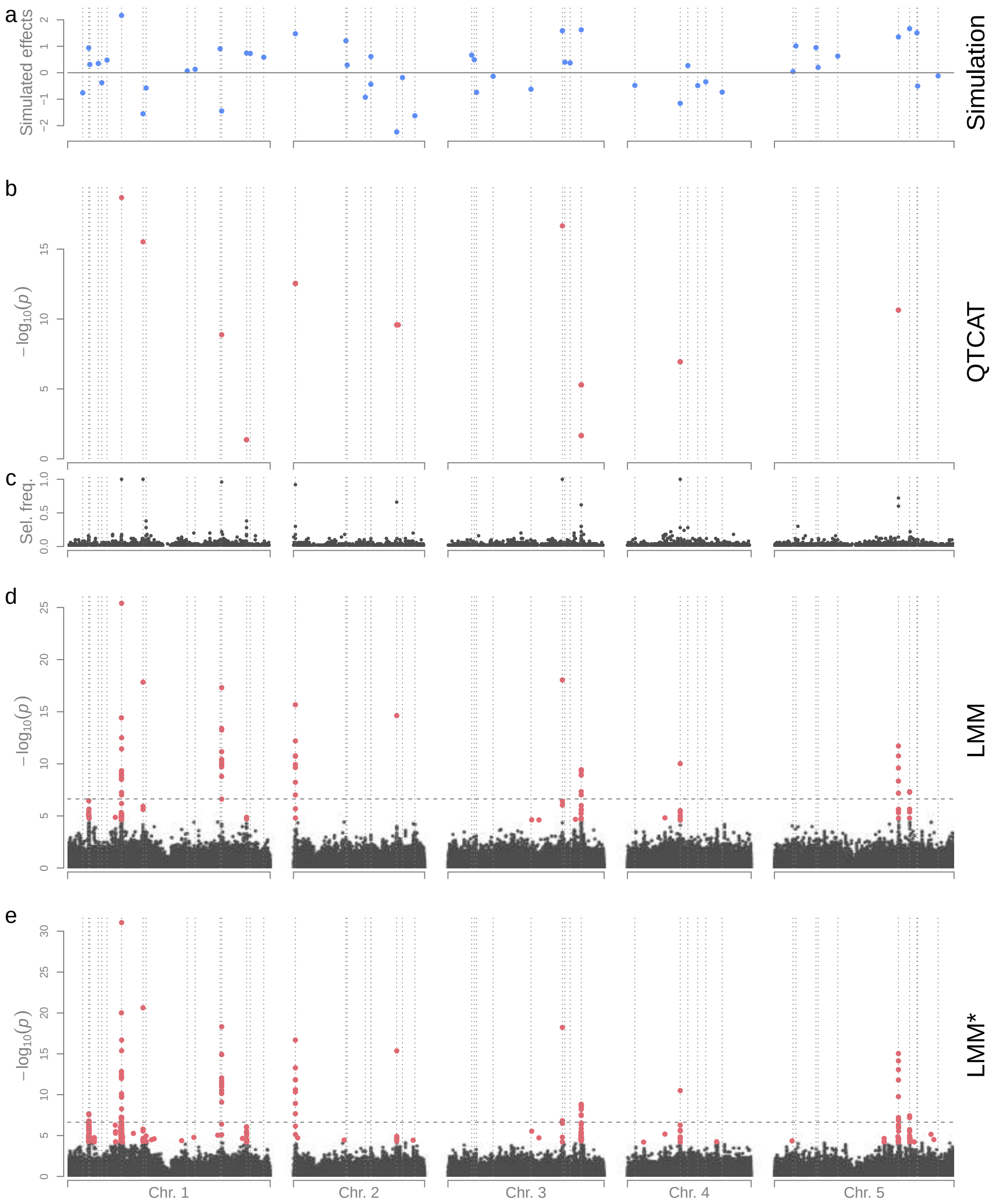

Supplementary Figure 134 Simulation of a GWA analysis based on a structured population with a heritability of 0.7 (run 34). (**a**) Simulation of 50 effects randomly drawn from a normal distribution and assigned to random markers. Markers with effect are highlighted with dashed lines. (**b**) Significant QTCs found by QTCAT. (**c**) LASSO selection frequency for each marker during the 50 iterations of QTCAT. (**d**) Manhattan plot of the LMM analysis. The horizontal dashed line depicts the significance threshold when controlling the multiple testing with FWER, whereas the red markers are significantly associated when controlling with FDR. (**e**) The Manhattan plot of the LMM* analysis. GRM was estimated without markers on the chromosome of the actual testing position. The results are shown as in (d).

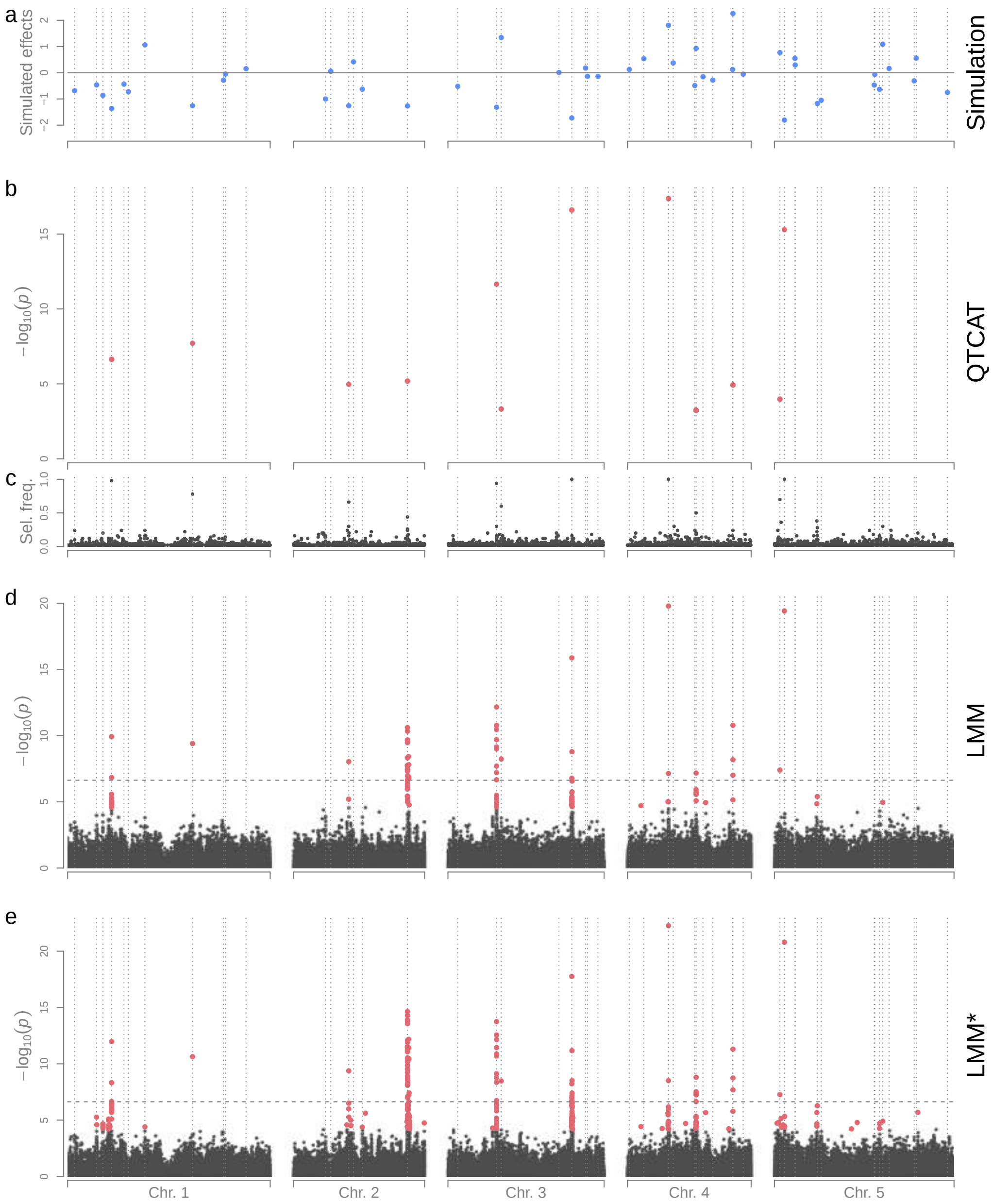

Supplementary Figure 135 Simulation of a GWA analysis based on a structured population with a heritability of 0.7 (run 35). (**a**) Simulation of 50 effects randomly drawn from a normal distribution and assigned to random markers. Markers with effect are highlighted with dashed lines. (**b**) Significant QTCs found by QTCAT. (**c**) LASSO selection frequency for each marker during the 50 iterations of QTCAT. (**d**) Manhattan plot of the LMM analysis. The horizontal dashed line depicts the significance threshold when controlling the multiple testing with FWER, whereas the red markers are significantly associated when controlling with FDR. (**e**) The Manhattan plot of the LMM* analysis. GRM was estimated without markers on the chromosome of the actual testing position. The results are shown as in (d).

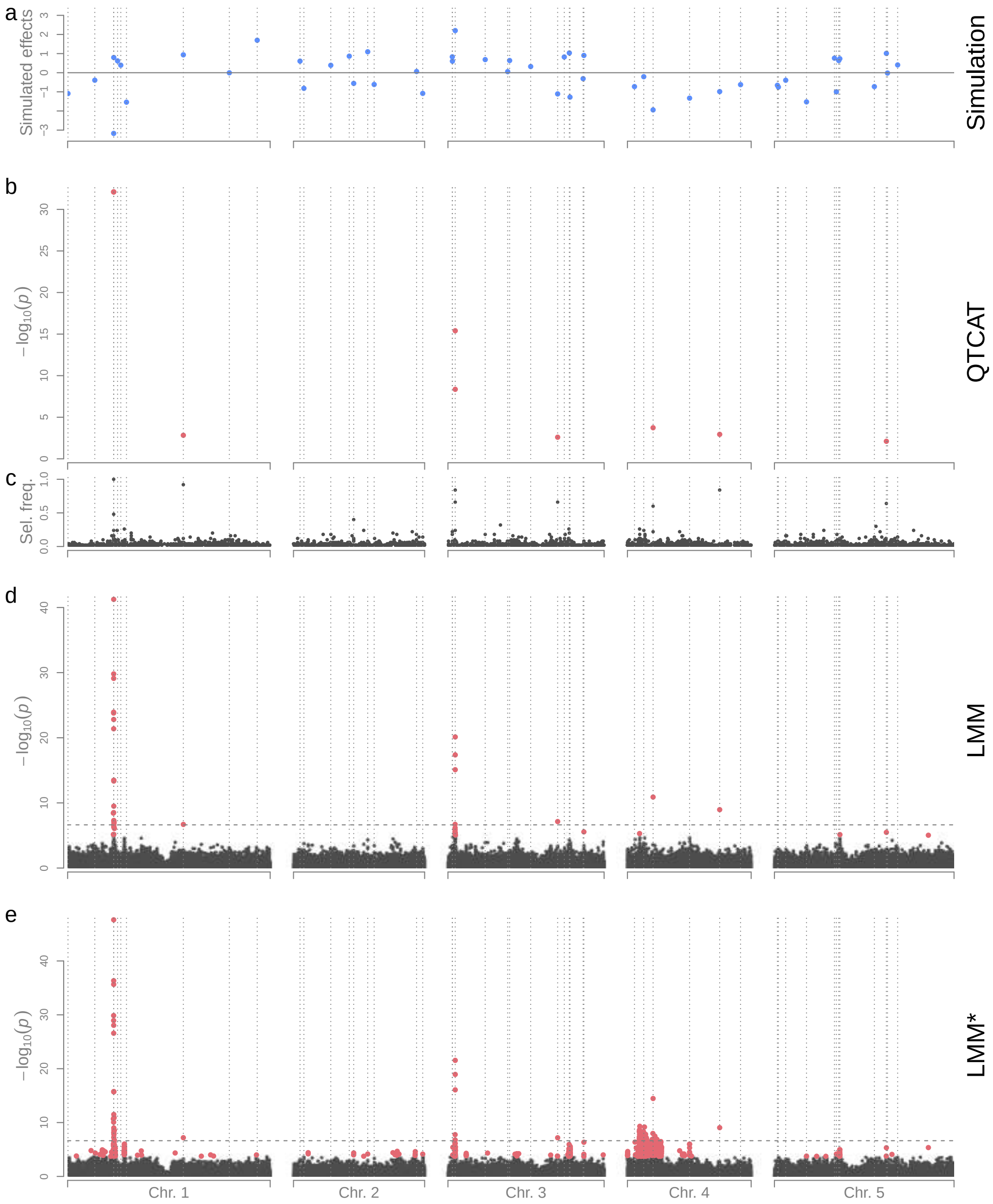

Supplementary Figure 136 Simulation of a GWA analysis based on a structured population with a heritability of 0.7 (run 36). (**a**) Simulation of 50 effects randomly drawn from a normal distribution and assigned to random markers. Markers with effect are highlighted with dashed lines. (**b**) Significant QTCs found by QTCAT. (**c**) LASSO selection frequency for each marker during the 50 iterations of QTCAT. (**d**) Manhattan plot of the LMM analysis. The horizontal dashed line depicts the significance threshold when controlling the multiple testing with FWER, whereas the red markers are significantly associated when controlling with FDR. (**e**) The Manhattan plot of the LMM* analysis. GRM was estimated without markers on the chromosome of the actual testing position. The results are shown as in (d).

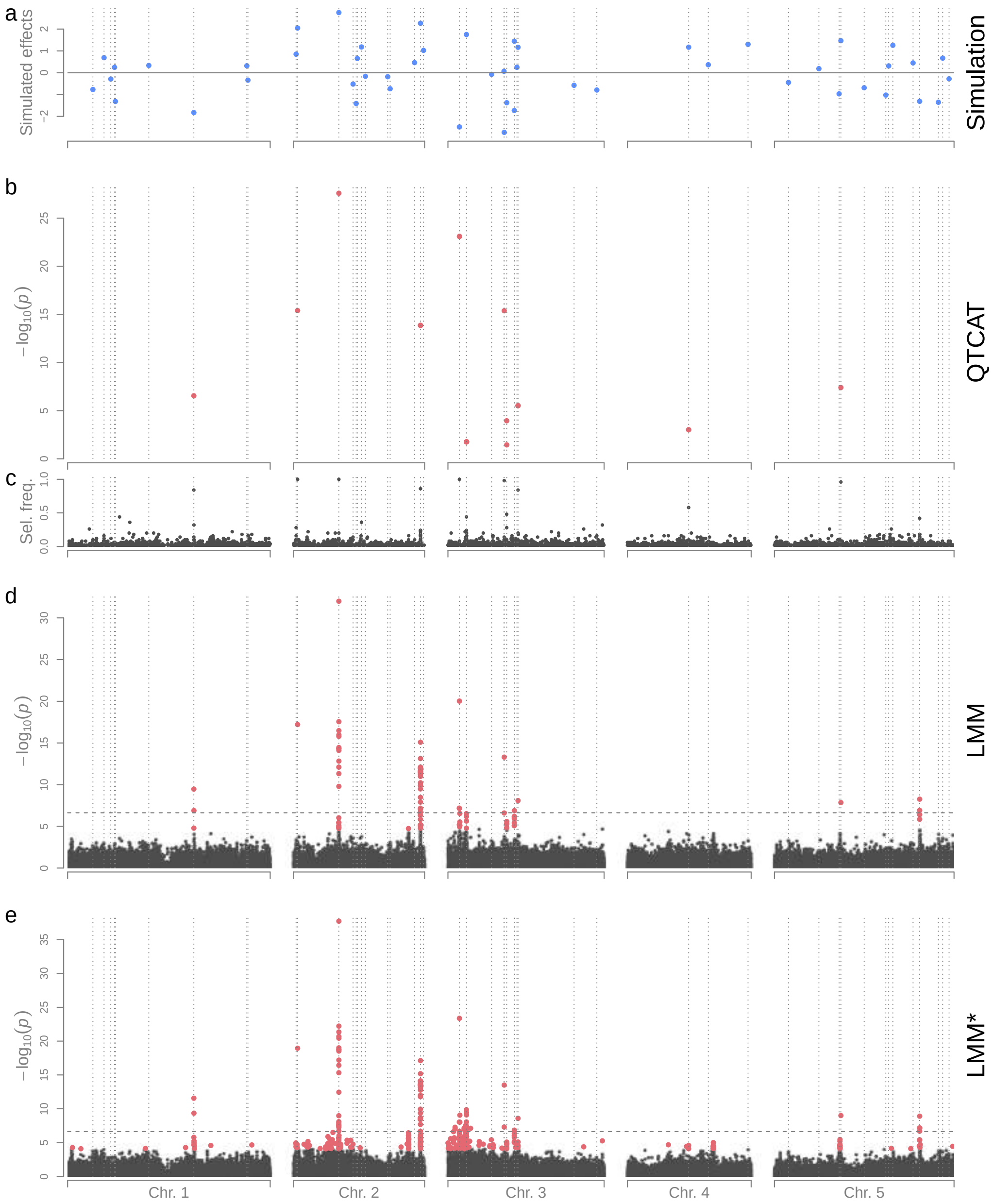

Supplementary Figure 137 Simulation of a GWA analysis based on a structured population with a heritability of 0.7 (run 37). (**a**) Simulation of 50 effects randomly drawn from a normal distribution and assigned to random markers. Markers with effect are highlighted with dashed lines. (**b**) Significant QTCs found by QTCAT. (**c**) LASSO selection frequency for each marker during the 50 iterations of QTCAT. (**d**) Manhattan plot of the LMM analysis. The horizontal dashed line depicts the significance threshold when controlling the multiple testing with FWER, whereas the red markers are significantly associated when controlling with FDR. (**e**) The Manhattan plot of the LMM* analysis. GRM was estimated without markers on the chromosome of the actual testing position. The results are shown as in (d).

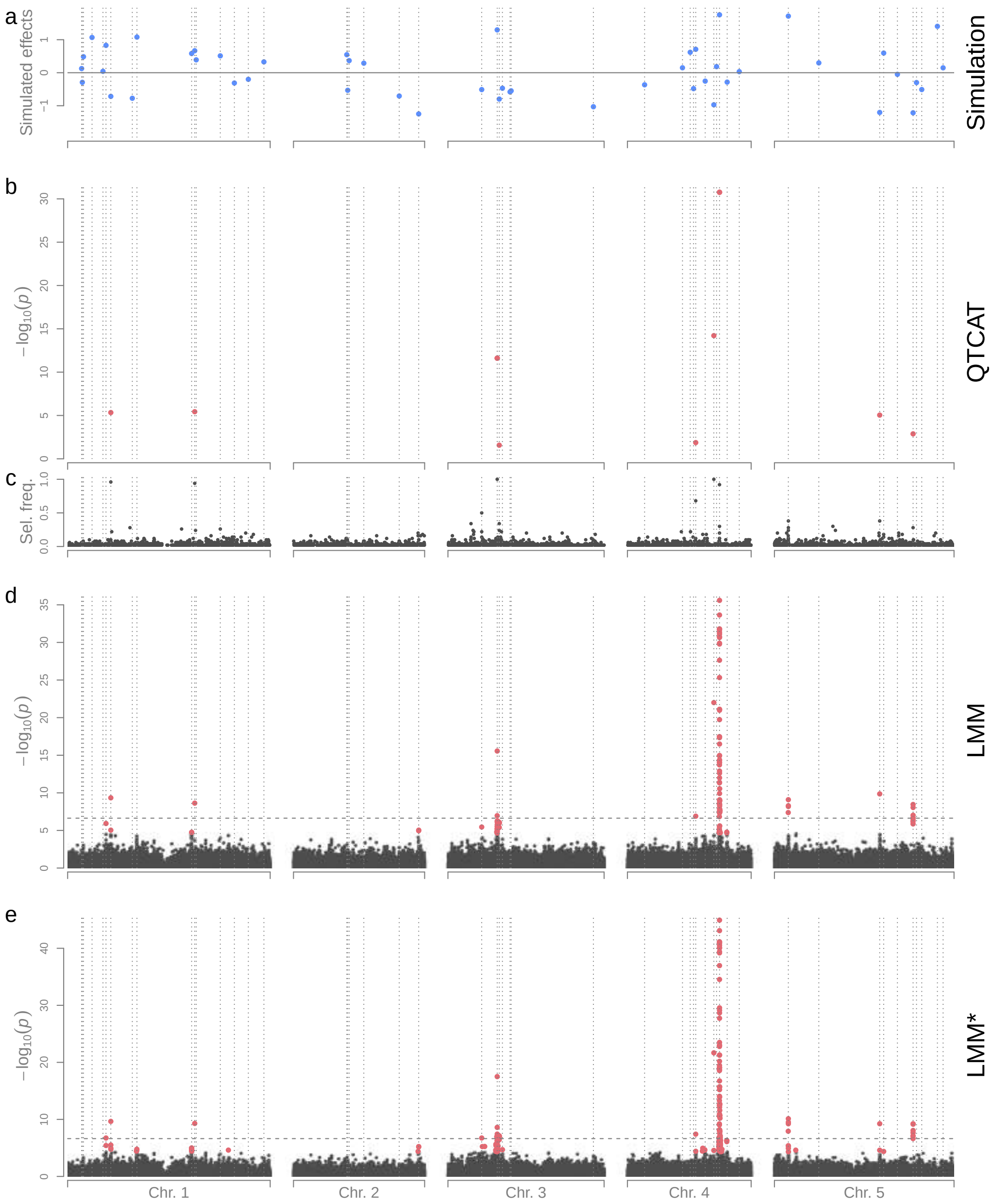

Supplementary Figure 138 Simulation of a GWA analysis based on a structured population with a heritability of 0.7 (run 38). (**a**) Simulation of 50 effects randomly drawn from a normal distribution and assigned to random markers. Markers with effect are highlighted with dashed lines. (**b**) Significant QTCs found by QTCAT. (**c**) LASSO selection frequency for each marker during the 50 iterations of QTCAT. (**d**) Manhattan plot of the LMM analysis. The horizontal dashed line depicts the significance threshold when controlling the multiple testing with FWER, whereas the red markers are significantly associated when controlling with FDR. (**e**) The Manhattan plot of the LMM* analysis. GRM was estimated without markers on the chromosome of the actual testing position. The results are shown as in (d).

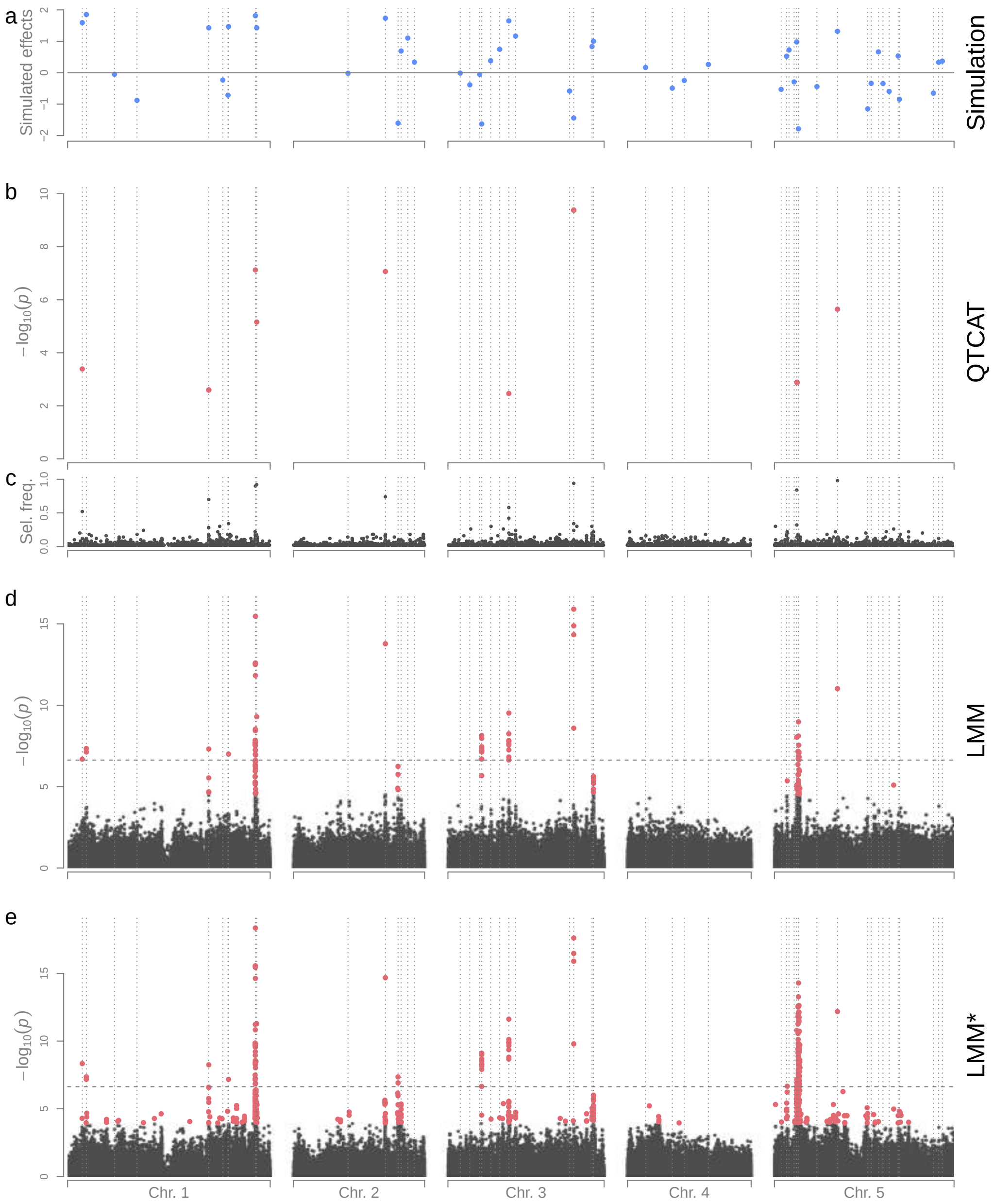

Supplementary Figure 139 Simulation of a GWA analysis based on a structured population with a heritability of 0.7 (run 39). (**a**) Simulation of 50 effects randomly drawn from a normal distribution and assigned to random markers. Markers with effect are highlighted with dashed lines. (**b**) Significant QTCs found by QTCAT. (**c**) LASSO selection frequency for each marker during the 50 iterations of QTCAT. (**d**) Manhattan plot of the LMM analysis. The horizontal dashed line depicts the significance threshold when controlling the multiple testing with FWER, whereas the red markers are significantly associated when controlling with FDR. (**e**) The Manhattan plot of the LMM* analysis. GRM was estimated without markers on the chromosome of the actual testing position. The results are shown as in (d).

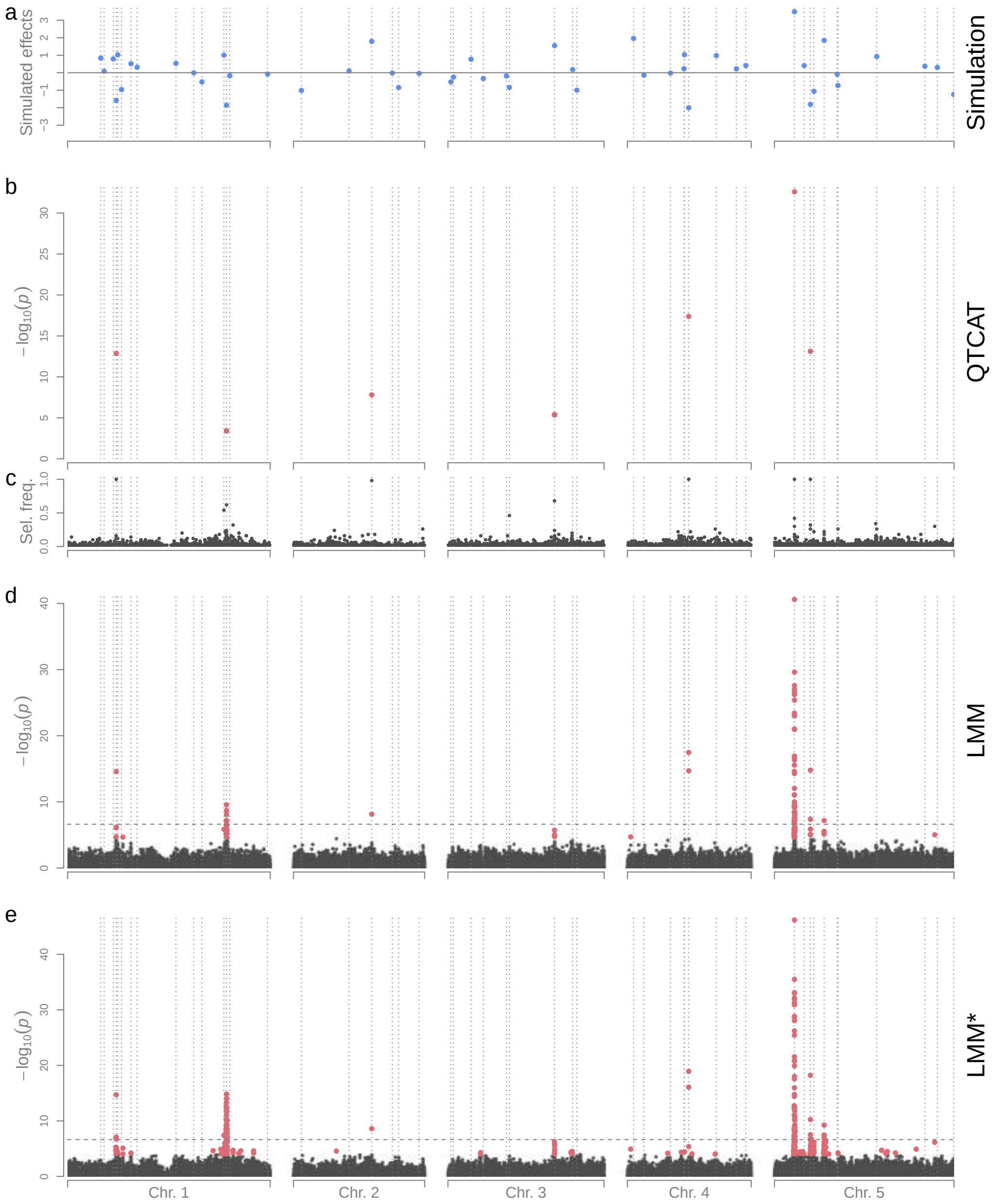

Supplementary Figure 140 Simulation of a GWA analysis based on a structured population with a heritability of 0.7 (run 40). (**a**) Simulation of 50 effects randomly drawn from a normal distribution and assigned to random markers. Markers with effect are highlighted with dashed lines. (**b**) Significant QTCs found by QTCAT. (**c**) LASSO selection frequency for each marker during the 50 iterations of QTCAT. (**d**) Manhattan plot of the LMM analysis. The horizontal dashed line depicts the significance threshold when controlling the multiple testing with FWER, whereas the red markers are significantly associated when controlling with FDR. (**e**) The Manhattan plot of the LMM* analysis. GRM was estimated without markers on the chromosome of the actual testing position. The results are shown as in (d).

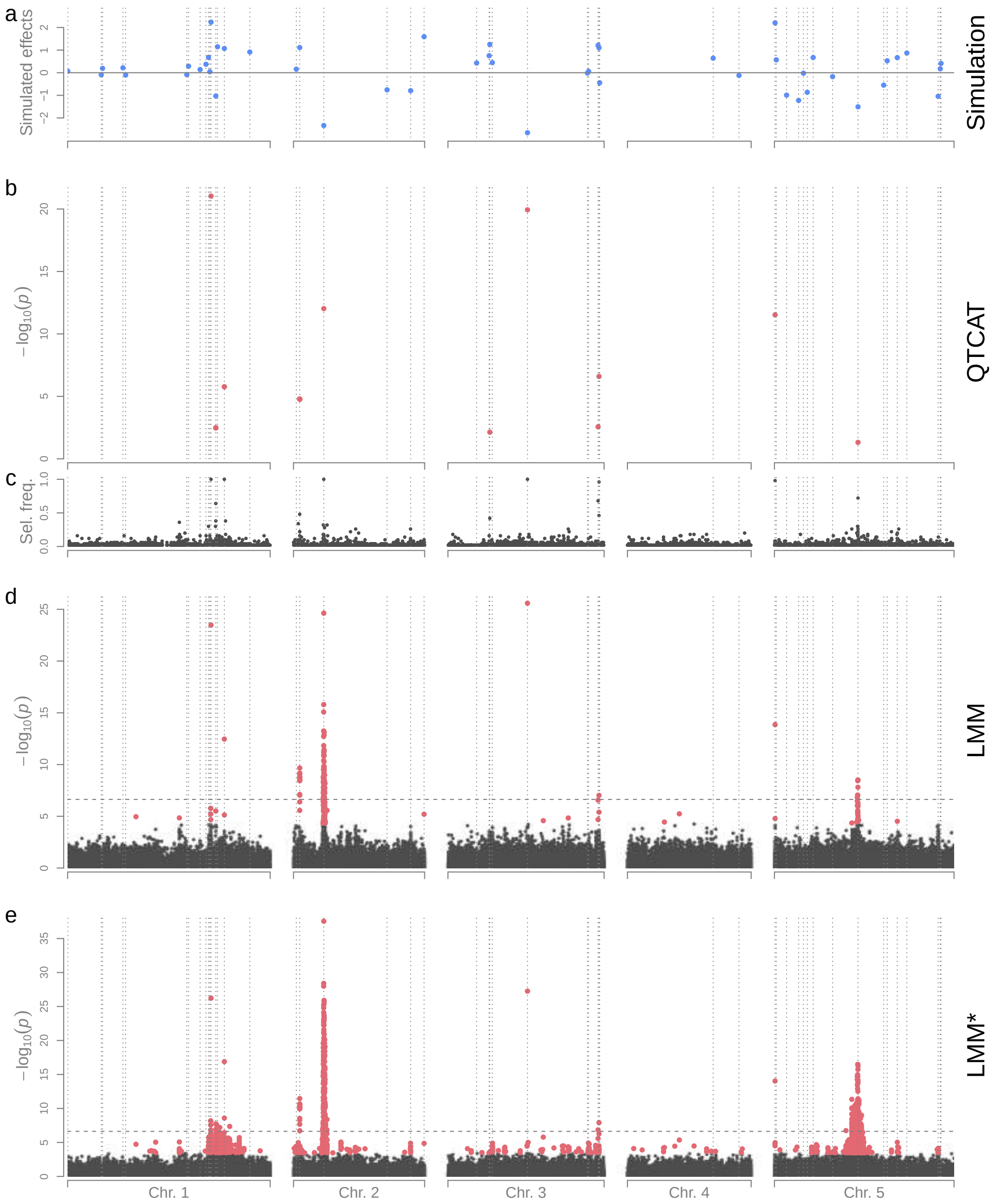

Supplementary Figure 141 Simulation of a GWA analysis based on a structured population with a heritability of 0.7 (run 41). (**a**) Simulation of 50 effects randomly drawn from a normal distribution and assigned to random markers. Markers with effect are highlighted with dashed lines. (**b**) Significant QTCs found by QTCAT. (**c**) LASSO selection frequency for each marker during the 50 iterations of QTCAT. (**d**) Manhattan plot of the LMM analysis. The horizontal dashed line depicts the significance threshold when controlling the multiple testing with FWER, whereas the red markers are significantly associated when controlling with FDR. (**e**) The Manhattan plot of the LMM* analysis. GRM was estimated without markers on the chromosome of the actual testing position. The results are shown as in (d).

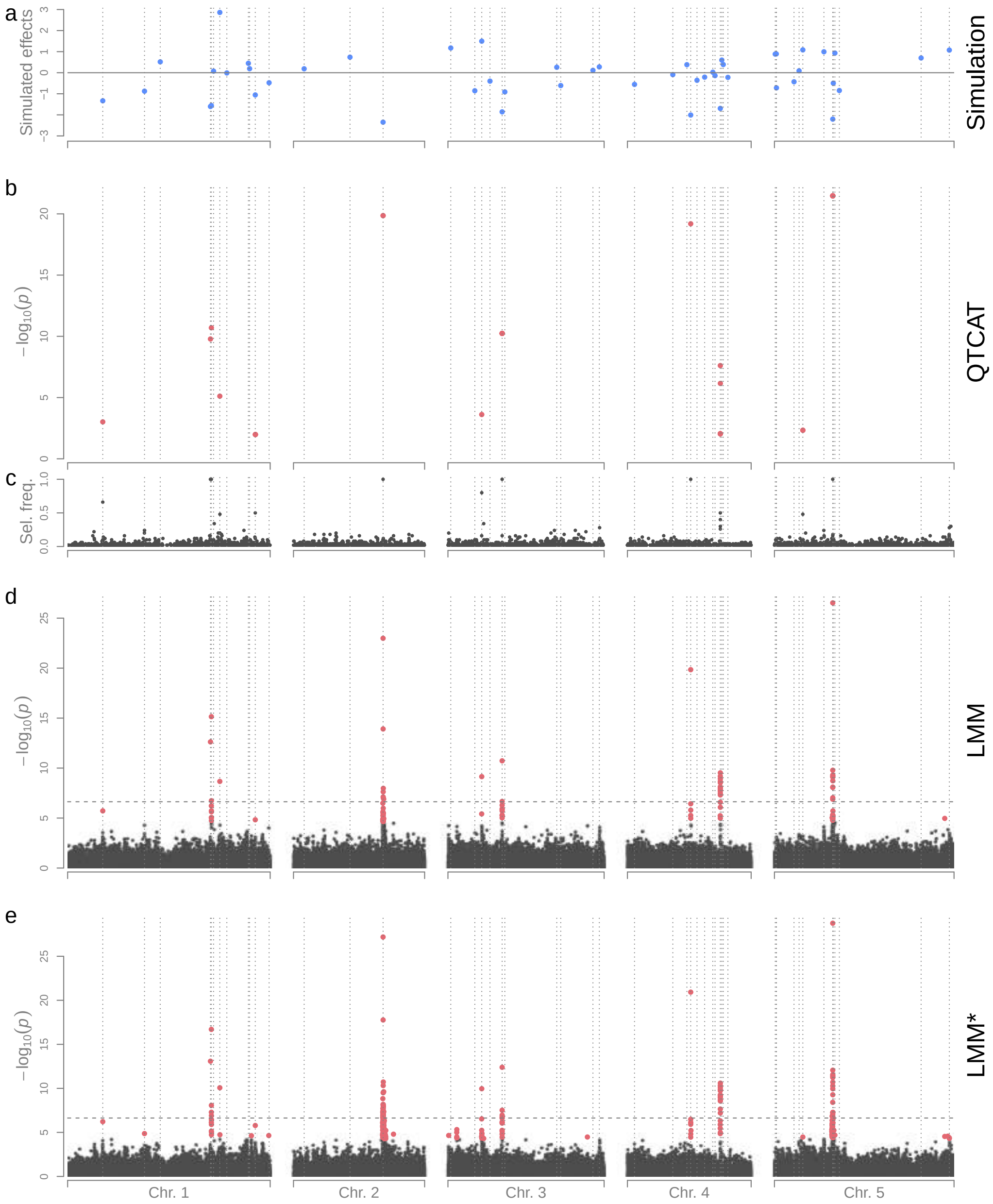

Supplementary Figure 142 Simulation of a GWA analysis based on a structured population with a heritability of 0.7 (run 42). (**a**) Simulation of 50 effects randomly drawn from a normal distribution and assigned to random markers. Markers with effect are highlighted with dashed lines. (**b**) Significant QTCs found by QTCAT. (**c**) LASSO selection frequency for each marker during the 50 iterations of QTCAT. (**d**) Manhattan plot of the LMM analysis. The horizontal dashed line depicts the significance threshold when controlling the multiple testing with FWER, whereas the red markers are significantly associated when controlling with FDR. (**e**) The Manhattan plot of the LMM* analysis. GRM was estimated without markers on the chromosome of the actual testing position. The results are shown as in (d).

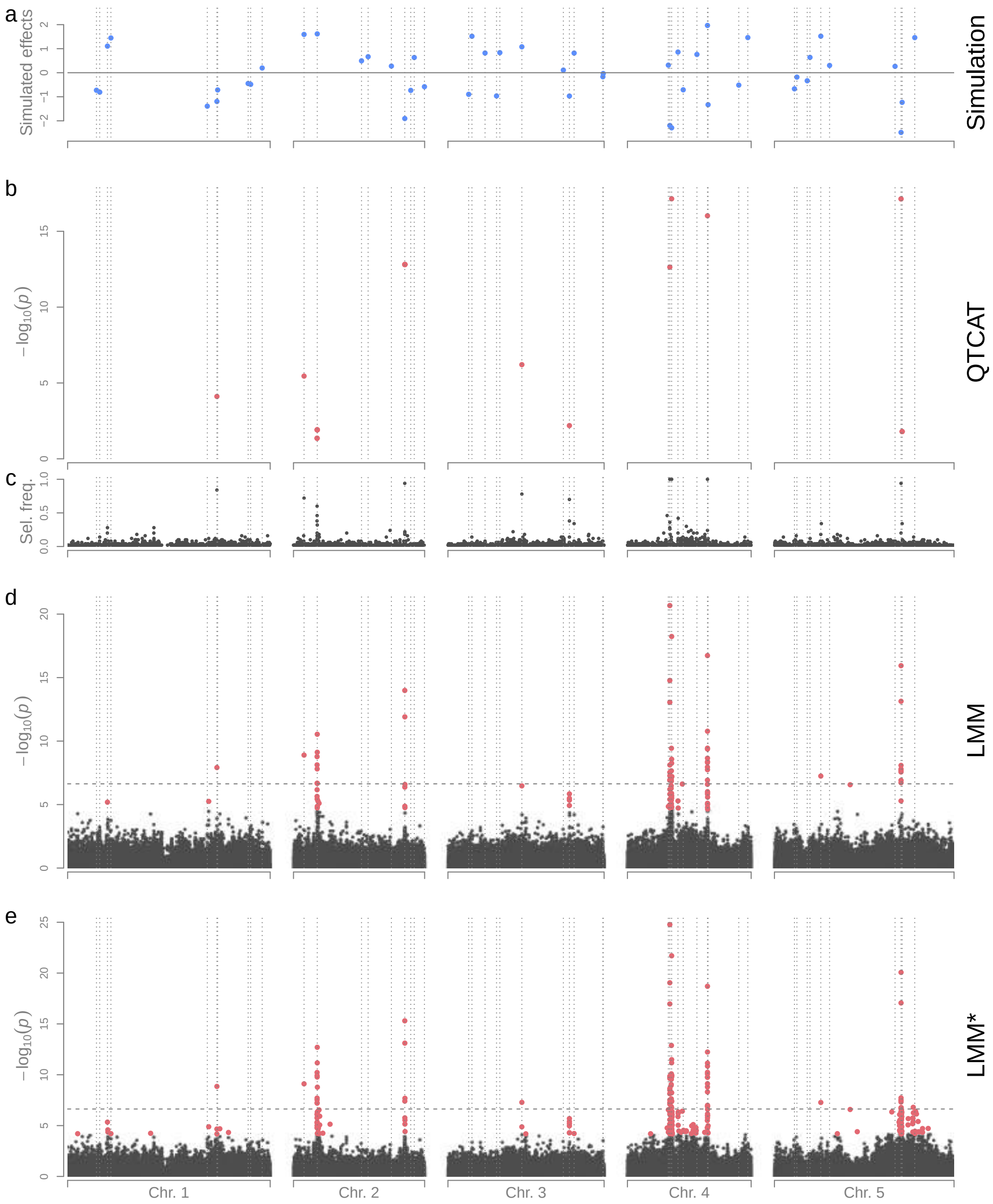

Supplementary Figure 143 Simulation of a GWA analysis based on a structured population with a heritability of 0.7 (run 43). (**a**) Simulation of 50 effects randomly drawn from a normal distribution and assigned to random markers. Markers with effect are highlighted with dashed lines. (**b**) Significant QTCs found by QTCAT. (**c**) LASSO selection frequency for each marker during the 50 iterations of QTCAT. (**d**) Manhattan plot of the LMM analysis. The horizontal dashed line depicts the significance threshold when controlling the multiple testing with FWER, whereas the red markers are significantly associated when controlling with FDR. (**e**) The Manhattan plot of the LMM* analysis. GRM was estimated without markers on the chromosome of the actual testing position. The results are shown as in (d).

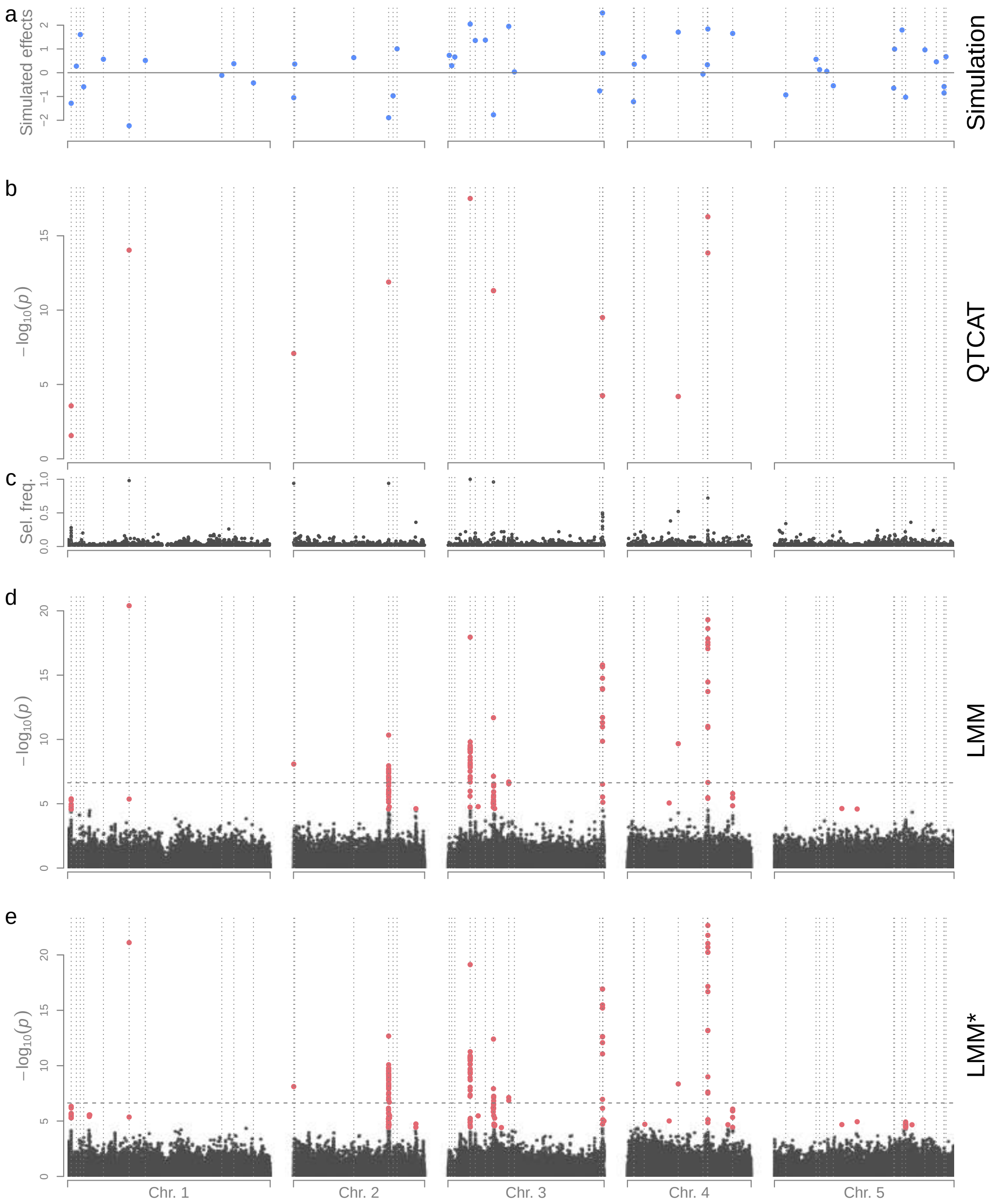

Supplementary Figure 144 Simulation of a GWA analysis based on a structured population with a heritability of 0.7 (run 44). (**a**) Simulation of 50 effects randomly drawn from a normal distribution and assigned to random markers. Markers with effect are highlighted with dashed lines. (**b**) Significant QTCs found by QTCAT. (**c**) LASSO selection frequency for each marker during the 50 iterations of QTCAT. (**d**) Manhattan plot of the LMM analysis. The horizontal dashed line depicts the significance threshold when controlling the multiple testing with FWER, whereas the red markers are significantly associated when controlling with FDR. (**e**) The Manhattan plot of the LMM* analysis. GRM was estimated without markers on the chromosome of the actual testing position. The results are shown as in (d).

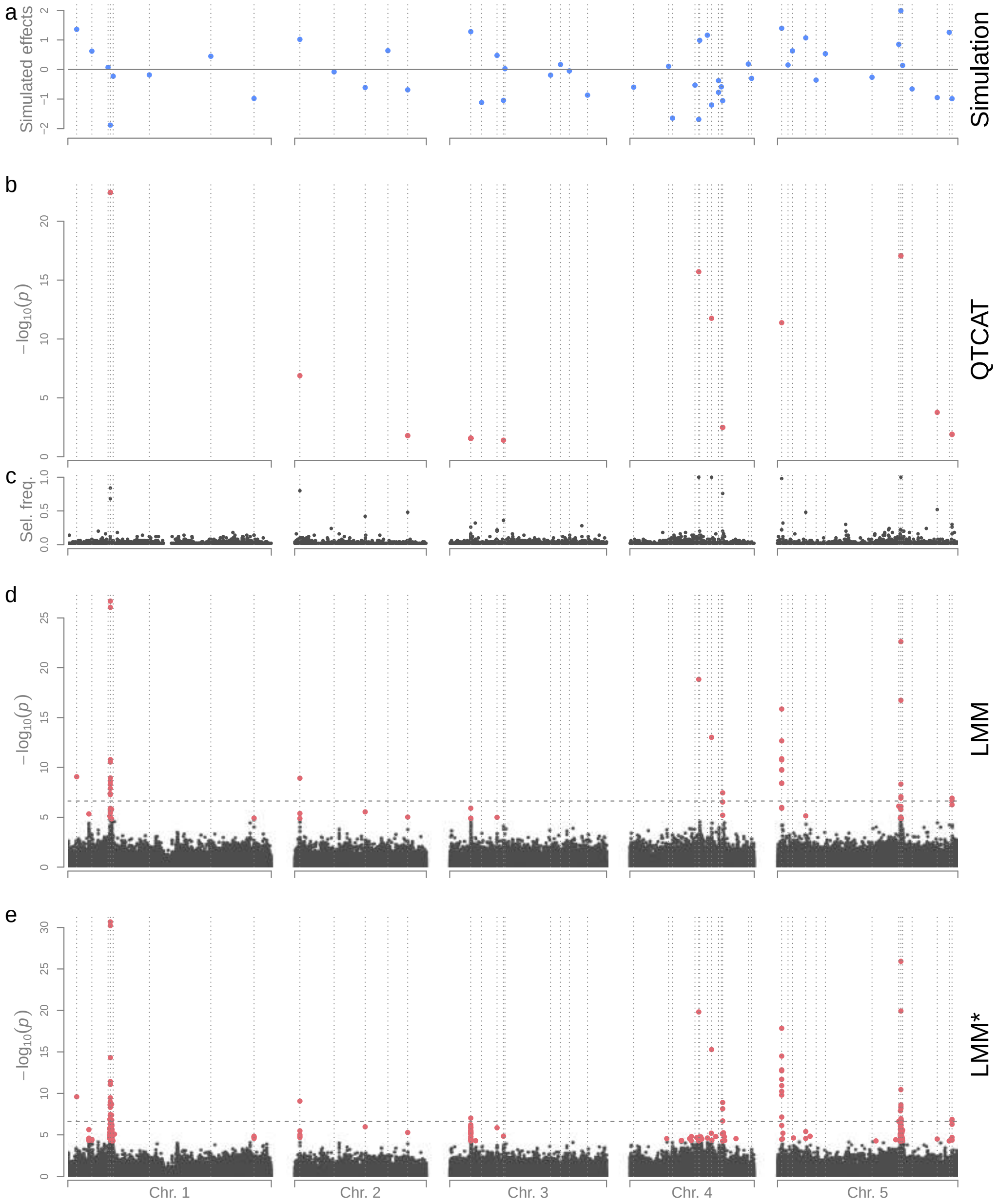

Supplementary Figure 145 Simulation of a GWA analysis based on a structured population with a heritability of 0.7 (run 45). (**a**) Simulation of 50 effects randomly drawn from a normal distribution and assigned to random markers. Markers with effect are highlighted with dashed lines. (**b**) Significant QTCs found by QTCAT. (**c**) LASSO selection frequency for each marker during the 50 iterations of QTCAT. (**d**) Manhattan plot of the LMM analysis. The horizontal dashed line depicts the significance threshold when controlling the multiple testing with FWER, whereas the red markers are significantly associated when controlling with FDR. (**e**) The Manhattan plot of the LMM* analysis. GRM was estimated without markers on the chromosome of the actual testing position. The results are shown as in (d).

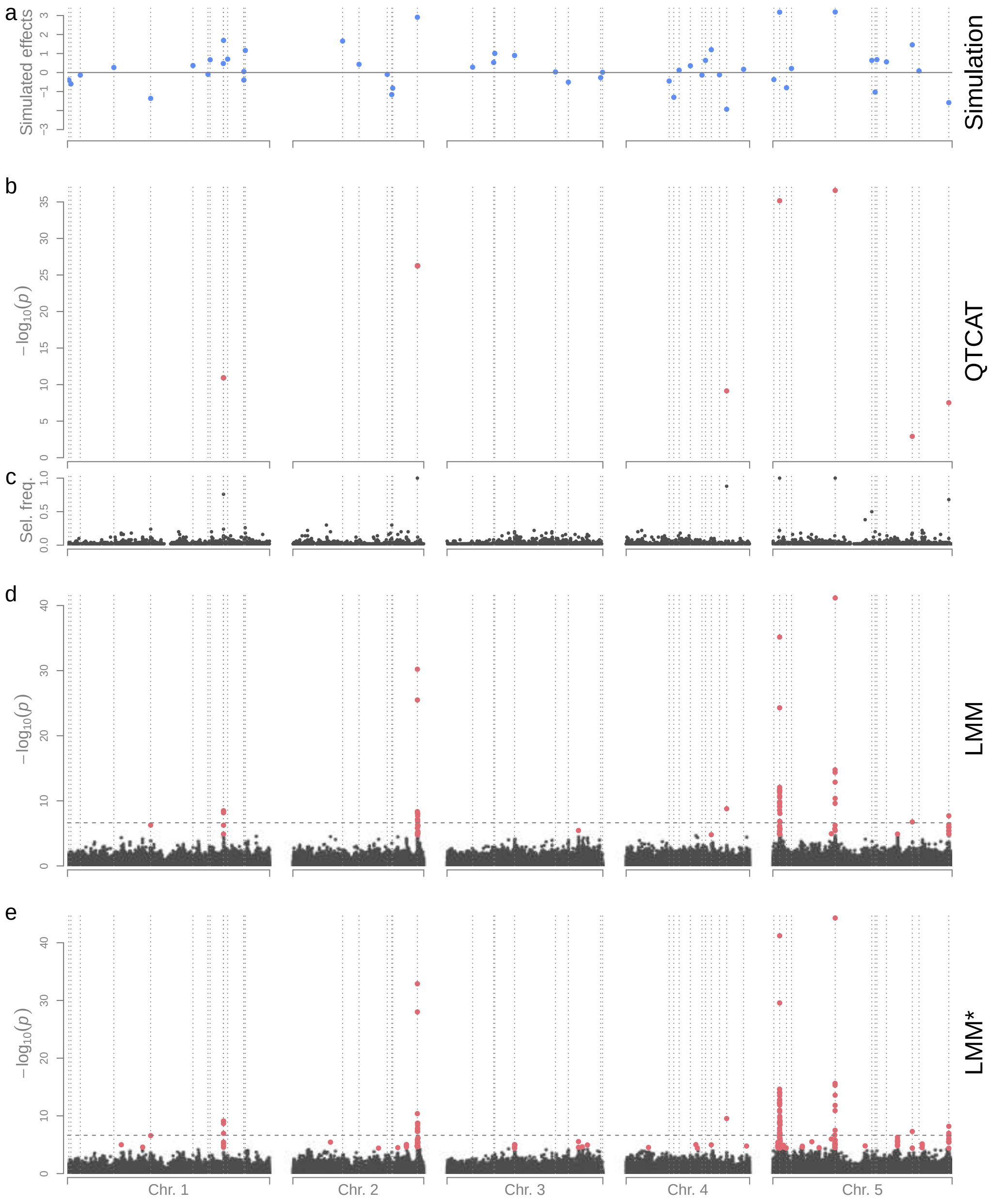

Supplementary Figure 146 Simulation of a GWA analysis based on a structured population with a heritability of 0.7 (run 46). (**a**) Simulation of 50 effects randomly drawn from a normal distribution and assigned to random markers. Markers with effect are highlighted with dashed lines. (**b**) Significant QTCs found by QTCAT. (**c**) LASSO selection frequency for each marker during the 50 iterations of QTCAT. (**d**) Manhattan plot of the LMM analysis. The horizontal dashed line depicts the significance threshold when controlling the multiple testing with FWER, whereas the red markers are significantly associated when controlling with FDR. (**e**) The Manhattan plot of the LMM* analysis. GRM was estimated without markers on the chromosome of the actual testing position. The results are shown as in (d).

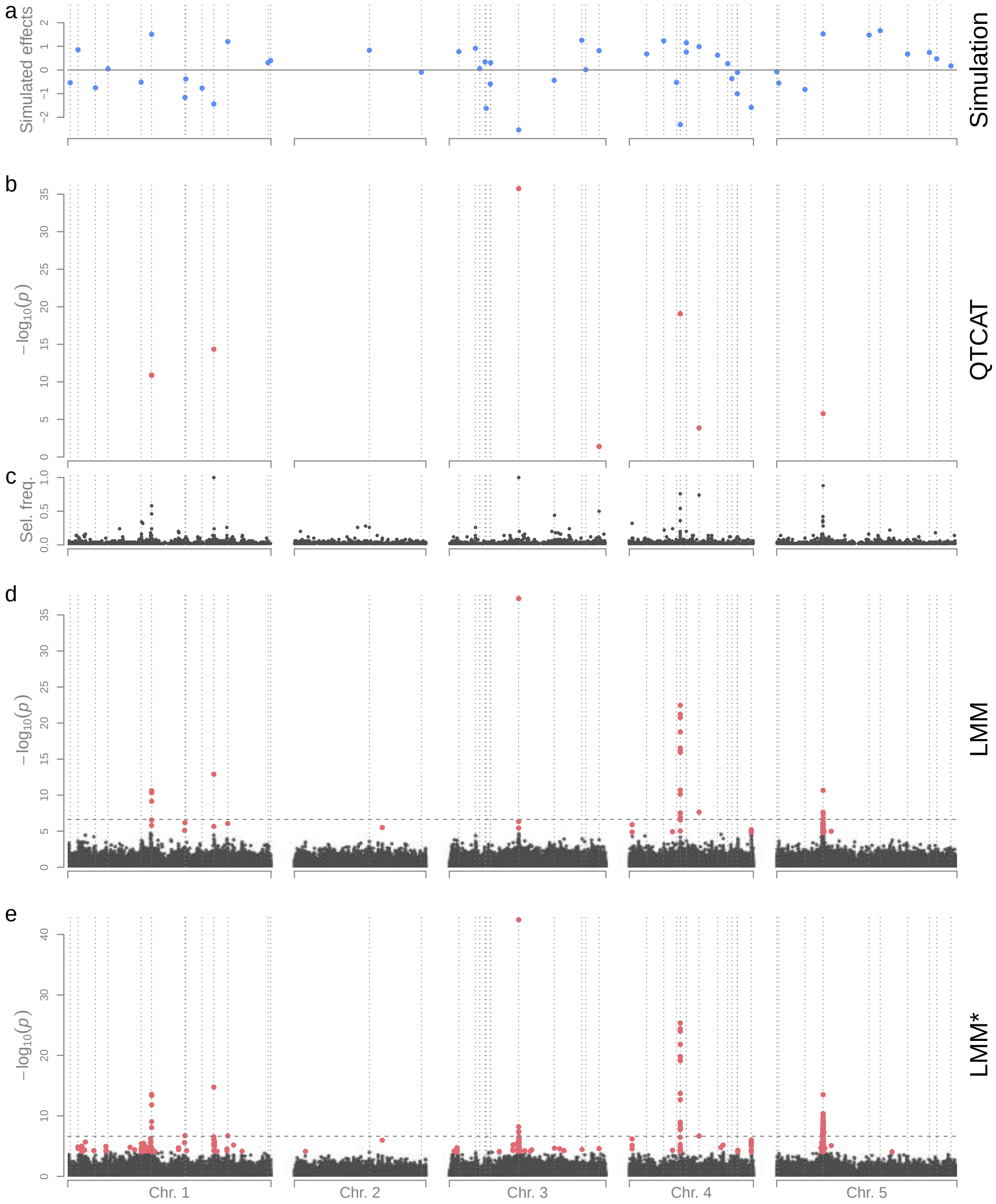

Supplementary Figure 147 Simulation of a GWA analysis based on a structured population with a heritability of 0.7 (run 47). (**a**) Simulation of 50 effects randomly drawn from a normal distribution and assigned to random markers. Markers with effect are highlighted with dashed lines. (**b**) Significant QTCs found by QTCAT. (**c**) LASSO selection frequency for each marker during the 50 iterations of QTCAT. (**d**) Manhattan plot of the LMM analysis. The horizontal dashed line depicts the significance threshold when controlling the multiple testing with FWER, whereas the red markers are significantly associated when controlling with FDR. (**e**) The Manhattan plot of the LMM* analysis. GRM was estimated without markers on the chromosome of the actual testing position. The results are shown as in (d).

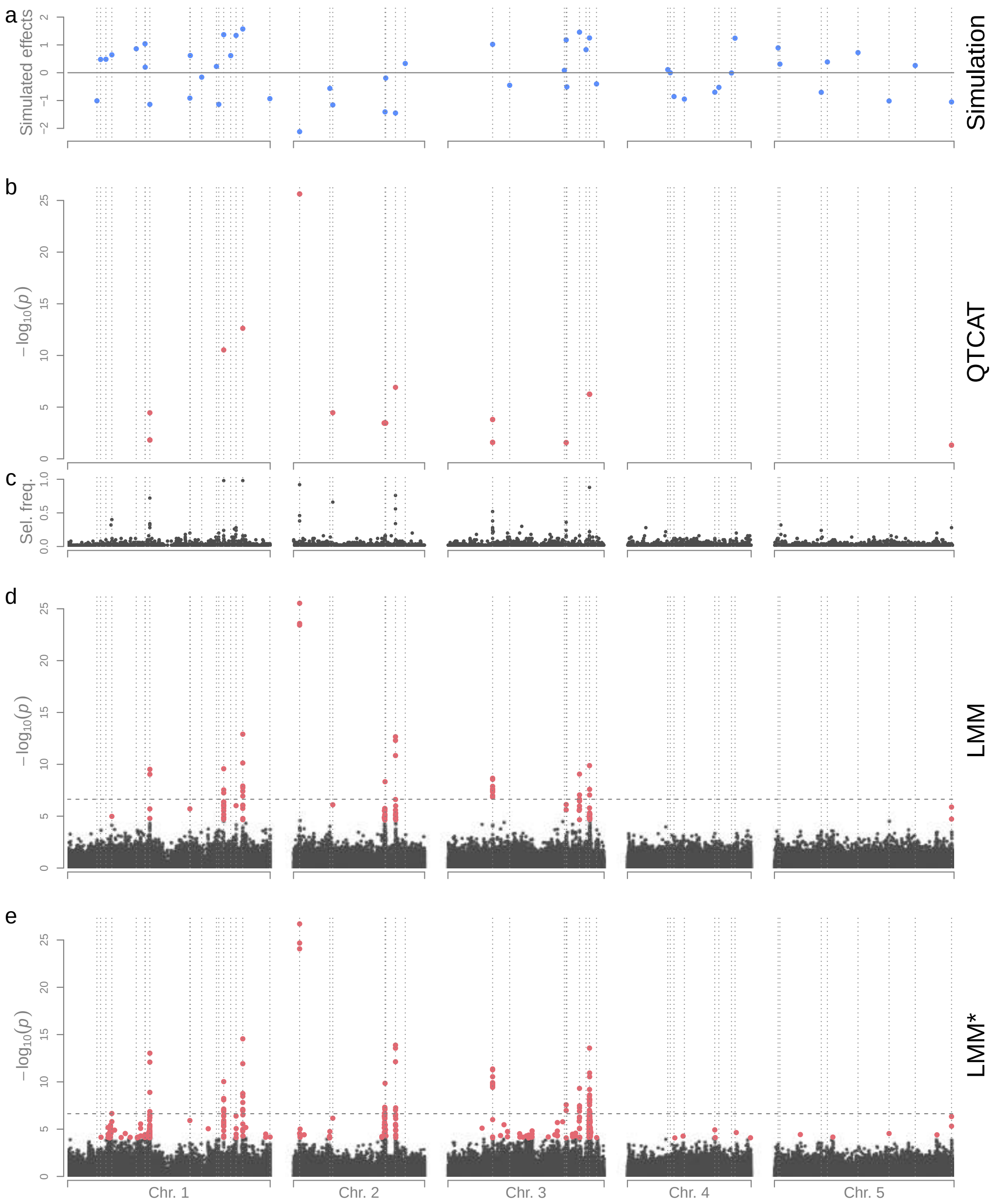

Supplementary Figure 148 Simulation of a GWA analysis based on a structured population with a heritability of 0.7 (run 48). (**a**) Simulation of 50 effects randomly drawn from a normal distribution and assigned to random markers. Markers with effect are highlighted with dashed lines. (**b**) Significant QTCs found by QTCAT. (**c**) LASSO selection frequency for each marker during the 50 iterations of QTCAT. (**d**) Manhattan plot of the LMM analysis. The horizontal dashed line depicts the significance threshold when controlling the multiple testing with FWER, whereas the red markers are significantly associated when controlling with FDR. (**e**) The Manhattan plot of the LMM* analysis. GRM was estimated without markers on the chromosome of the actual testing position. The results are shown as in (d).

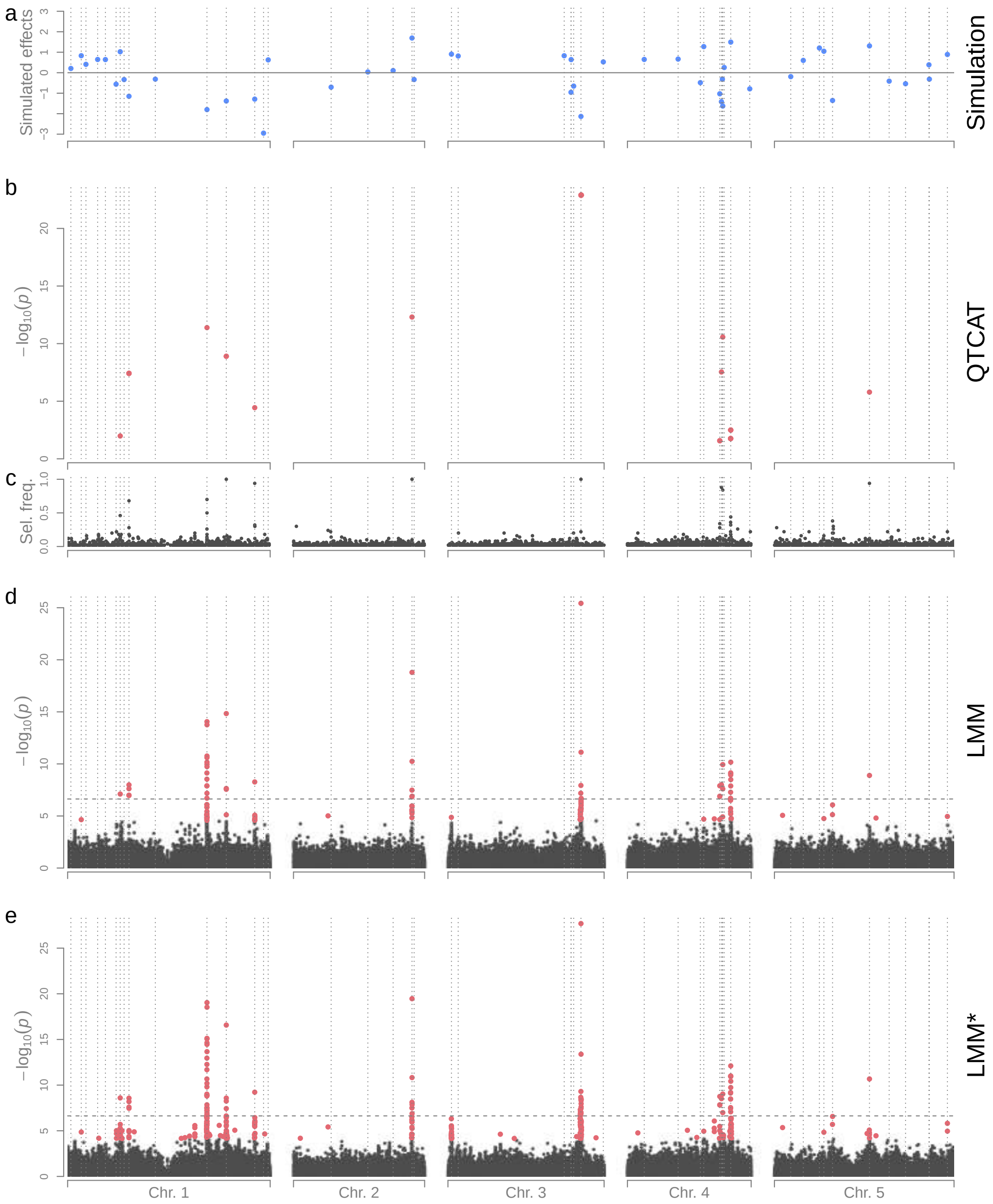

Supplementary Figure 149 Simulation of a GWA analysis based on a structured population with a heritability of 0.7 (run 49). (**a**) Simulation of 50 effects randomly drawn from a normal distribution and assigned to random markers. Markers with effect are highlighted with dashed lines. (**b**) Significant QTCs found by QTCAT. (**c**) LASSO selection frequency for each marker during the 50 iterations of QTCAT. (**d**) Manhattan plot of the LMM analysis. The horizontal dashed line depicts the significance threshold when controlling the multiple testing with FWER, whereas the red markers are significantly associated when controlling with FDR. (**e**) The Manhattan plot of the LMM* analysis. GRM was estimated without markers on the chromosome of the actual testing position. The results are shown as in (d).

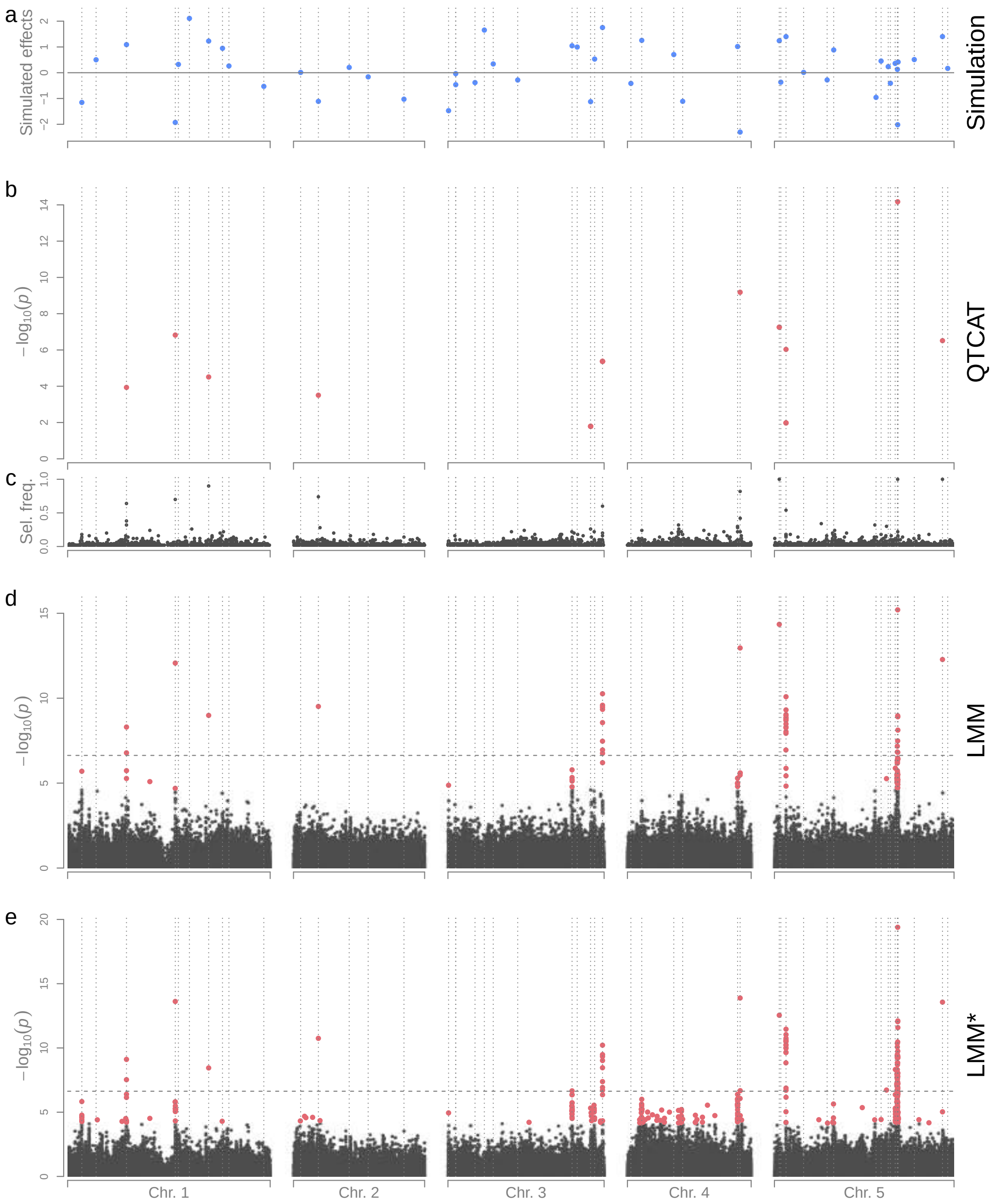

Supplementary Figure 150 Simulation of a GWA analysis based on a structured population with a heritability of 0.7 (run 50). (**a**) Simulation of 50 effects randomly drawn from a normal distribution and assigned to random markers. Markers with effect are highlighted with dashed lines. (**b**) Significant QTCs found by QTCAT. (**c**) LASSO selection frequency for each marker during the 50 iterations of QTCAT. (**d**) Manhattan plot of the LMM analysis. The horizontal dashed line depicts the significance threshold when controlling the multiple testing with FWER, whereas the red markers are significantly associated when controlling with FDR. (**e**) The Manhattan plot of the LMM* analysis. GRM was estimated without markers on the chromosome of the actual testing position. The results are shown as in (d).

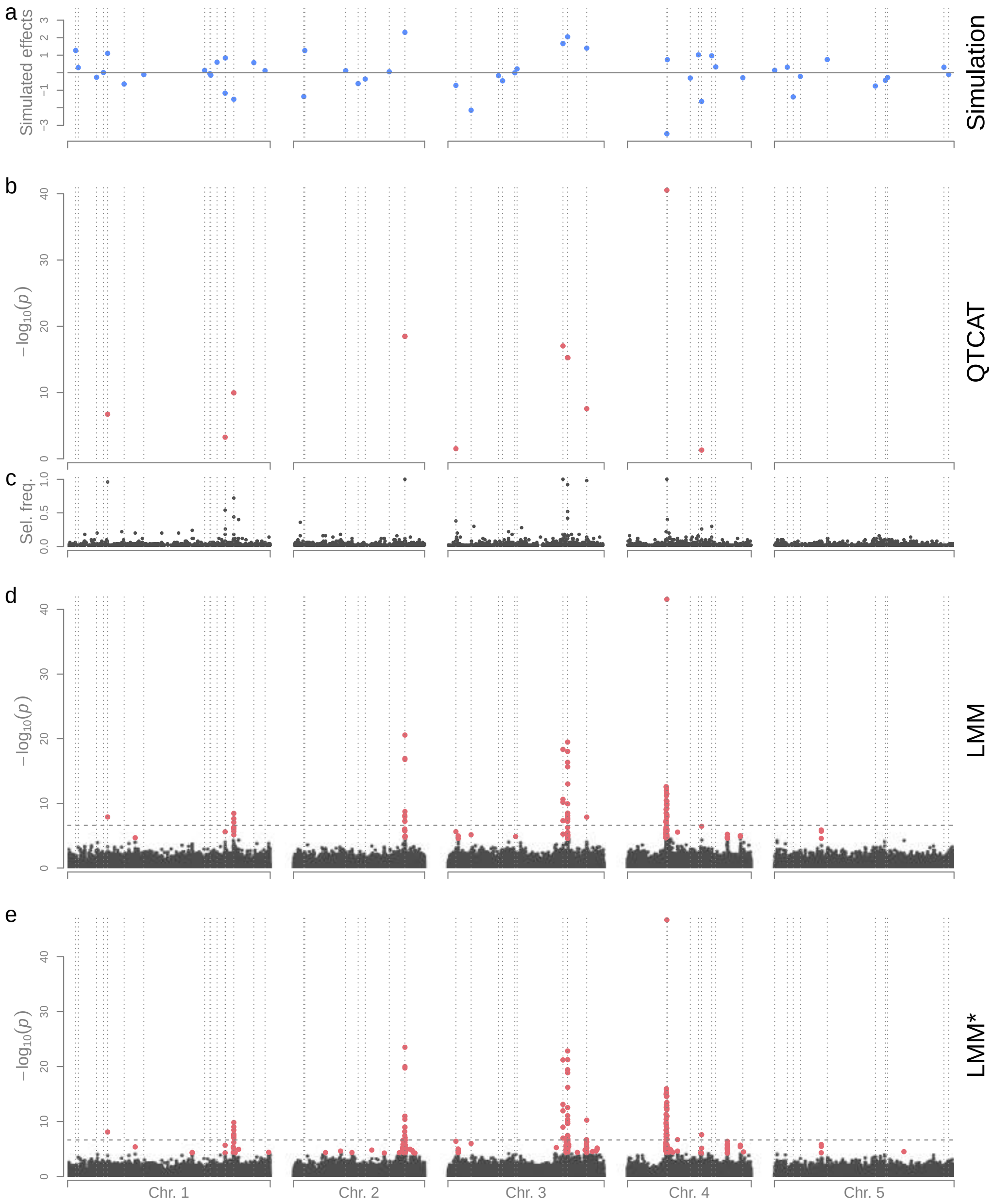

Supplementary Figure 151 Simulation of a GWA analysis based on a structured population with a heritability of 0.7 (run 51). (**a**) Simulation of 50 effects randomly drawn from a normal distribution and assigned to random markers. Markers with effect are highlighted with dashed lines. (**b**) Significant QTCs found by QTCAT. (**c**) LASSO selection frequency for each marker during the 50 iterations of QTCAT. (**d**) Manhattan plot of the LMM analysis. The horizontal dashed line depicts the significance threshold when controlling the multiple testing with FWER, whereas the red markers are significantly associated when controlling with FDR. (**e**) The Manhattan plot of the LMM* analysis. GRM was estimated without markers on the chromosome of the actual testing position. The results are shown as in (d).

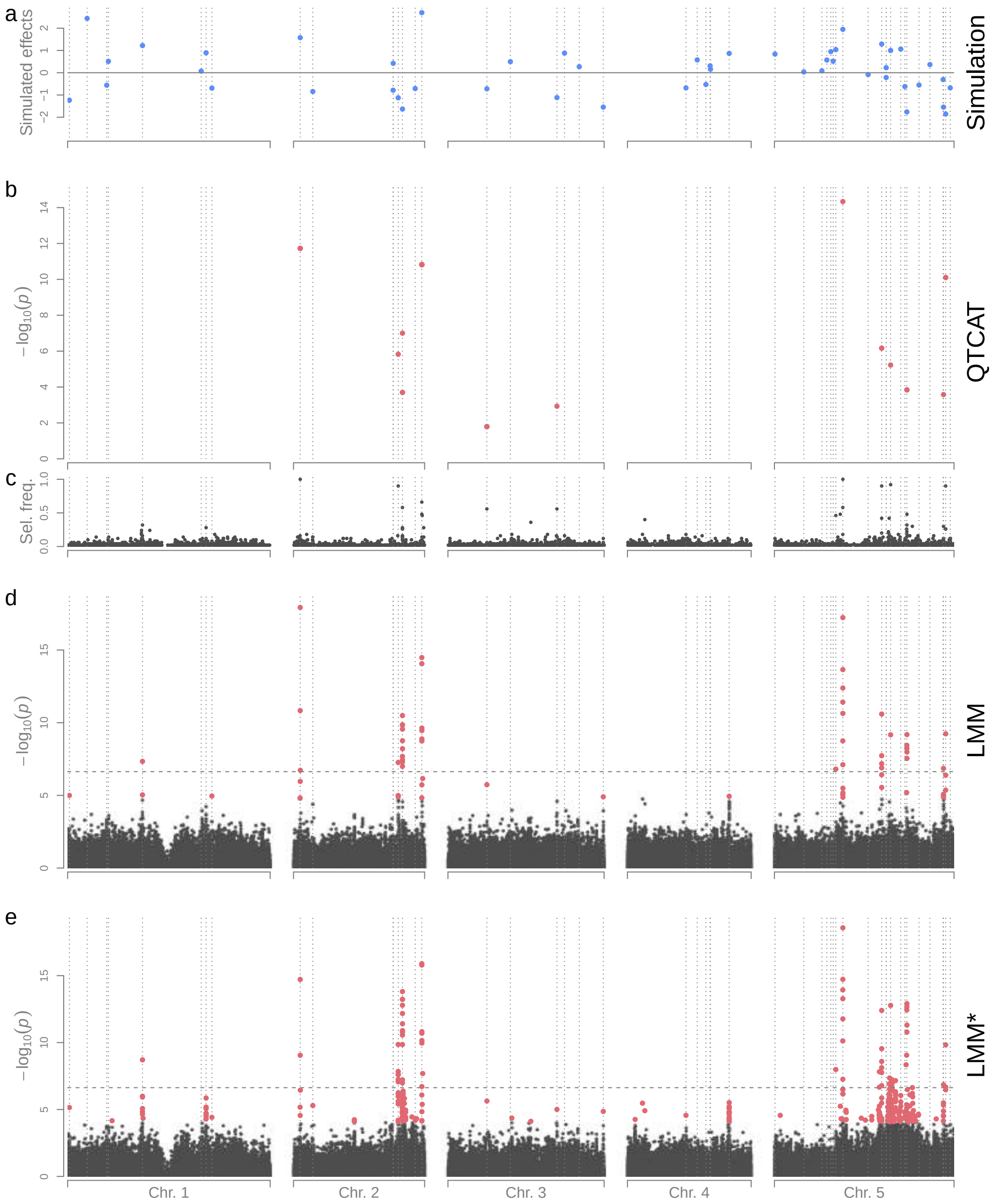

Supplementary Figure 152 Simulation of a GWA analysis based on a structured population with a heritability of 0.7 (run 52). (**a**) Simulation of 50 effects randomly drawn from a normal distribution and assigned to random markers. Markers with effect are highlighted with dashed lines. (**b**) Significant QTCs found by QTCAT. (**c**) LASSO selection frequency for each marker during the 50 iterations of QTCAT. (**d**) Manhattan plot of the LMM analysis. The horizontal dashed line depicts the significance threshold when controlling the multiple testing with FWER, whereas the red markers are significantly associated when controlling with FDR. (**e**) The Manhattan plot of the LMM* analysis. GRM was estimated without markers on the chromosome of the actual testing position. The results are shown as in (d).

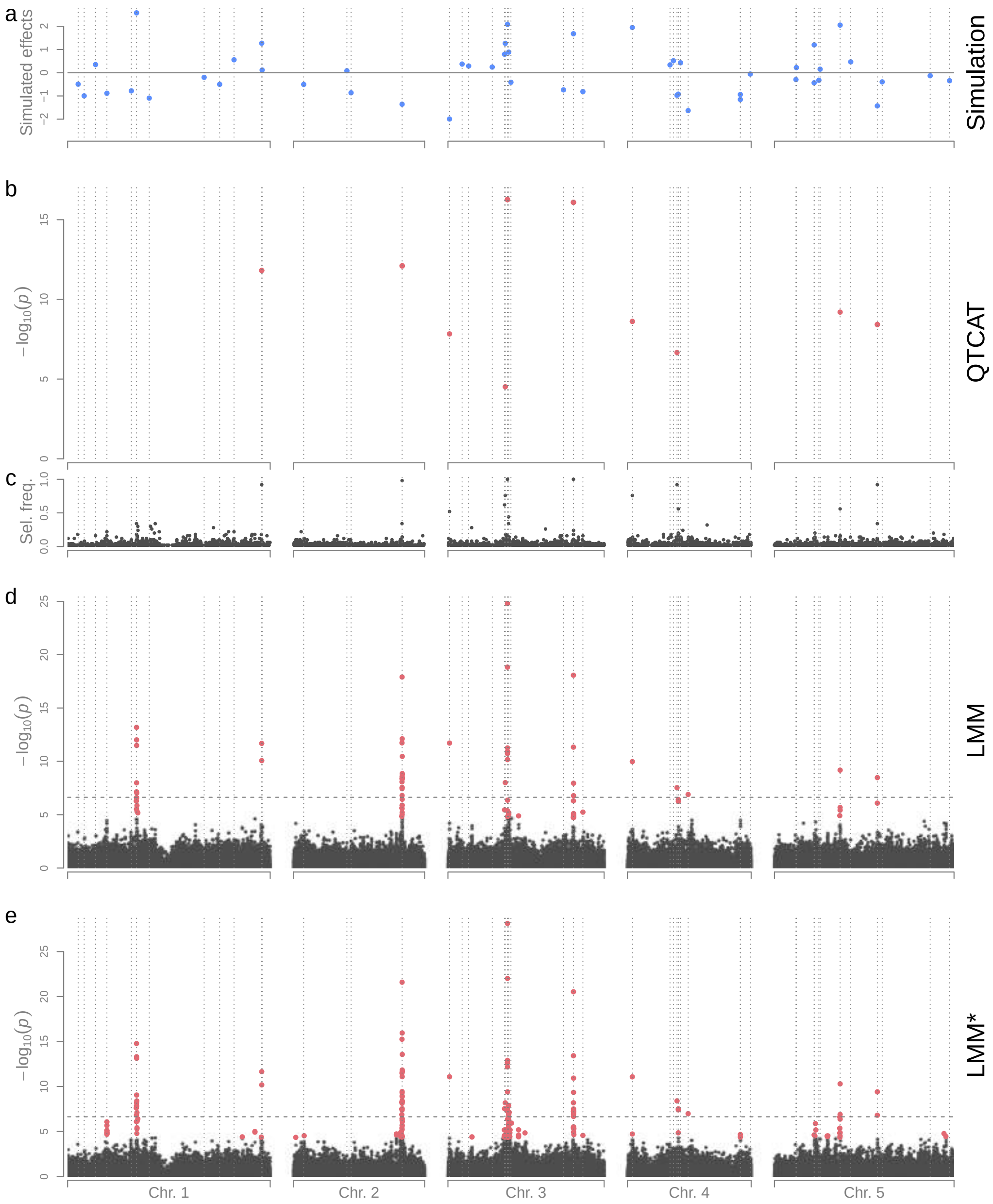

Supplementary Figure 153 Simulation of a GWA analysis based on a structured population with a heritability of 0.7 (run 53). (**a**) Simulation of 50 effects randomly drawn from a normal distribution and assigned to random markers. Markers with effect are highlighted with dashed lines. (**b**) Significant QTCs found by QTCAT. (**c**) LASSO selection frequency for each marker during the 50 iterations of QTCAT. (**d**) Manhattan plot of the LMM analysis. The horizontal dashed line depicts the significance threshold when controlling the multiple testing with FWER, whereas the red markers are significantly associated when controlling with FDR. (**e**) The Manhattan plot of the LMM* analysis. GRM was estimated without markers on the chromosome of the actual testing position. The results are shown as in (d).

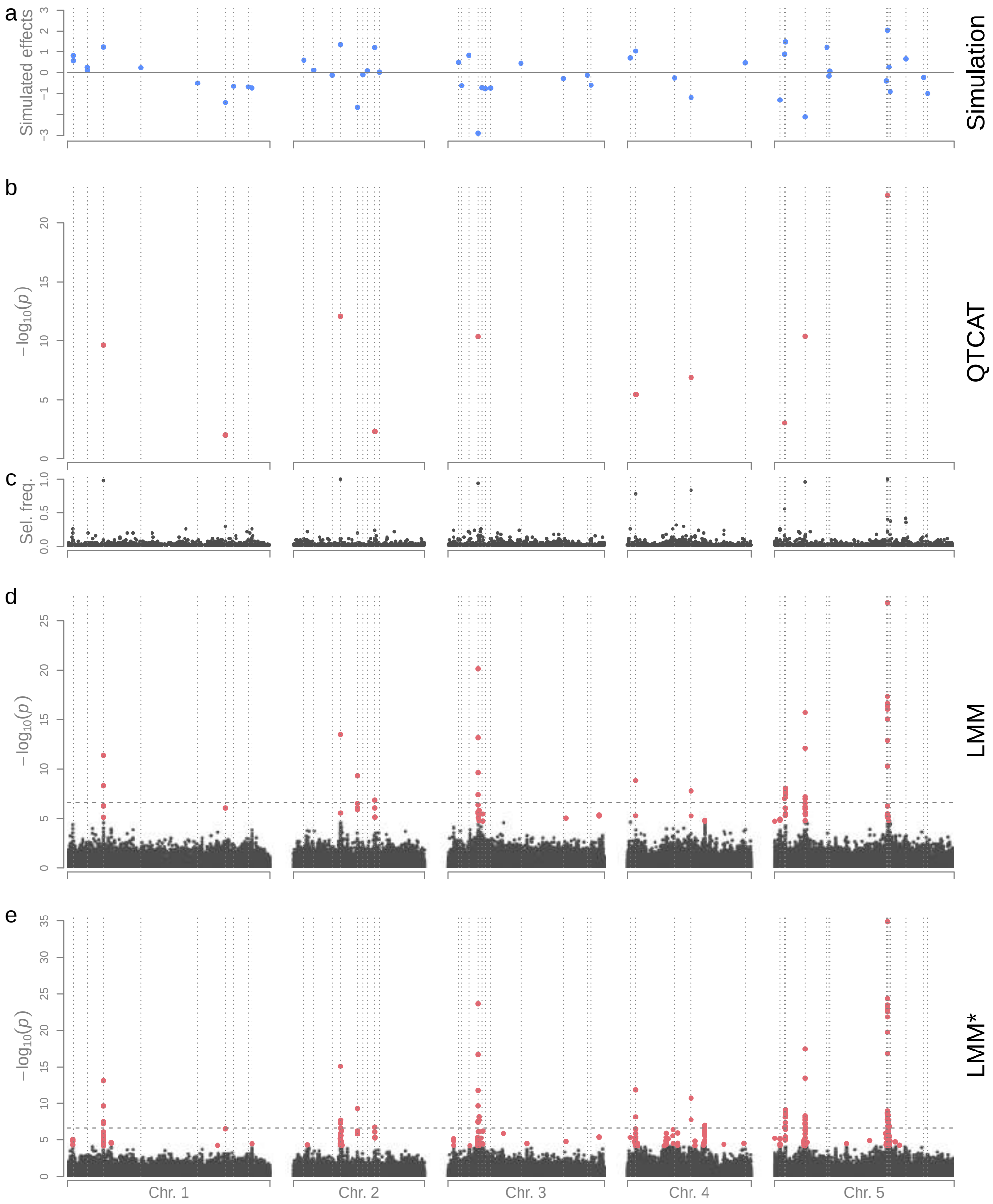

Supplementary Figure 154 Simulation of a GWA analysis based on a structured population with a heritability of 0.7 (run 54). (**a**) Simulation of 50 effects randomly drawn from a normal distribution and assigned to random markers. Markers with effect are highlighted with dashed lines. (**b**) Significant QTCs found by QTCAT. (**c**) LASSO selection frequency for each marker during the 50 iterations of QTCAT. (**d**) Manhattan plot of the LMM analysis. The horizontal dashed line depicts the significance threshold when controlling the multiple testing with FWER, whereas the red markers are significantly associated when controlling with FDR. (**e**) The Manhattan plot of the LMM* analysis. GRM was estimated without markers on the chromosome of the actual testing position. The results are shown as in (d).

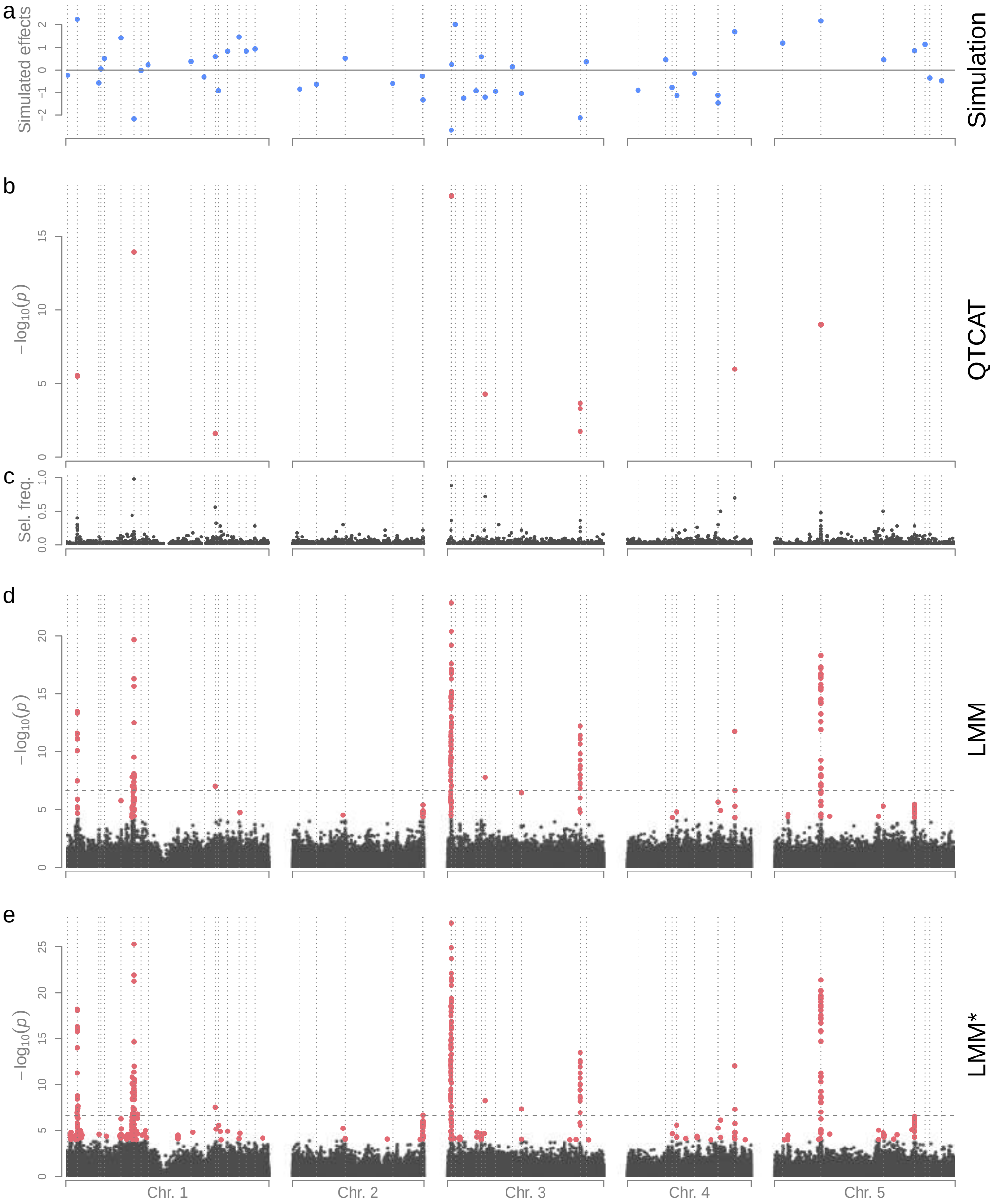

Supplementary Figure 155 Simulation of a GWA analysis based on a structured population with a heritability of 0.7 (run 55). (**a**) Simulation of 50 effects randomly drawn from a normal distribution and assigned to random markers. Markers with effect are highlighted with dashed lines. (**b**) Significant QTCs found by QTCAT. (**c**) LASSO selection frequency for each marker during the 50 iterations of QTCAT. (**d**) Manhattan plot of the LMM analysis. The horizontal dashed line depicts the significance threshold when controlling the multiple testing with FWER, whereas the red markers are significantly associated when controlling with FDR. (**e**) The Manhattan plot of the LMM* analysis. GRM was estimated without markers on the chromosome of the actual testing position. The results are shown as in (d).

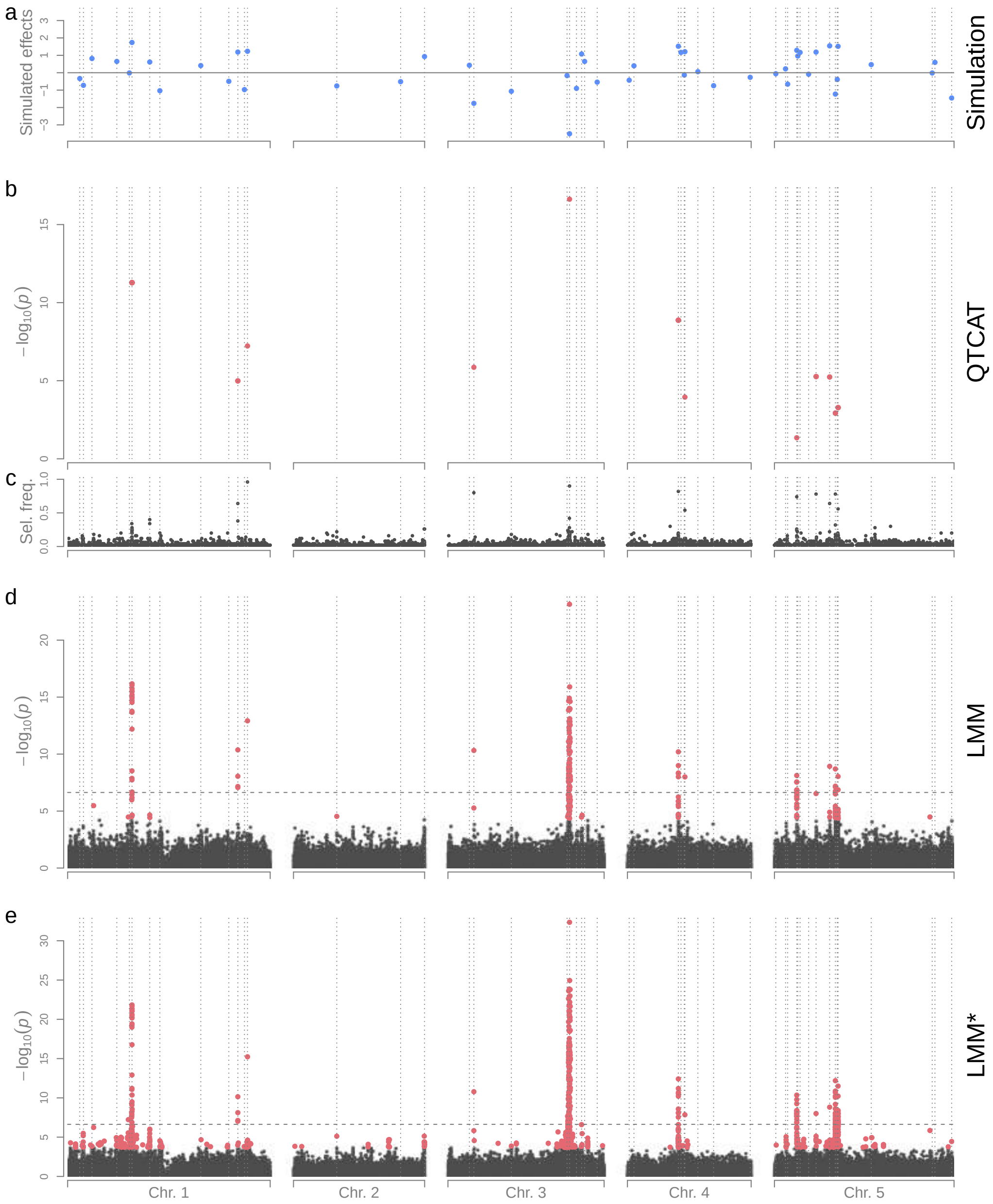

Supplementary Figure 156 Simulation of a GWA analysis based on a structured population with a heritability of 0.7 (run 56). (**a**) Simulation of 50 effects randomly drawn from a normal distribution and assigned to random markers. Markers with effect are highlighted with dashed lines. (**b**) Significant QTCs found by QTCAT. (**c**) LASSO selection frequency for each marker during the 50 iterations of QTCAT. (**d**) Manhattan plot of the LMM analysis. The horizontal dashed line depicts the significance threshold when controlling the multiple testing with FWER, whereas the red markers are significantly associated when controlling with FDR. (**e**) The Manhattan plot of the LMM* analysis. GRM was estimated without markers on the chromosome of the actual testing position. The results are shown as in (d).

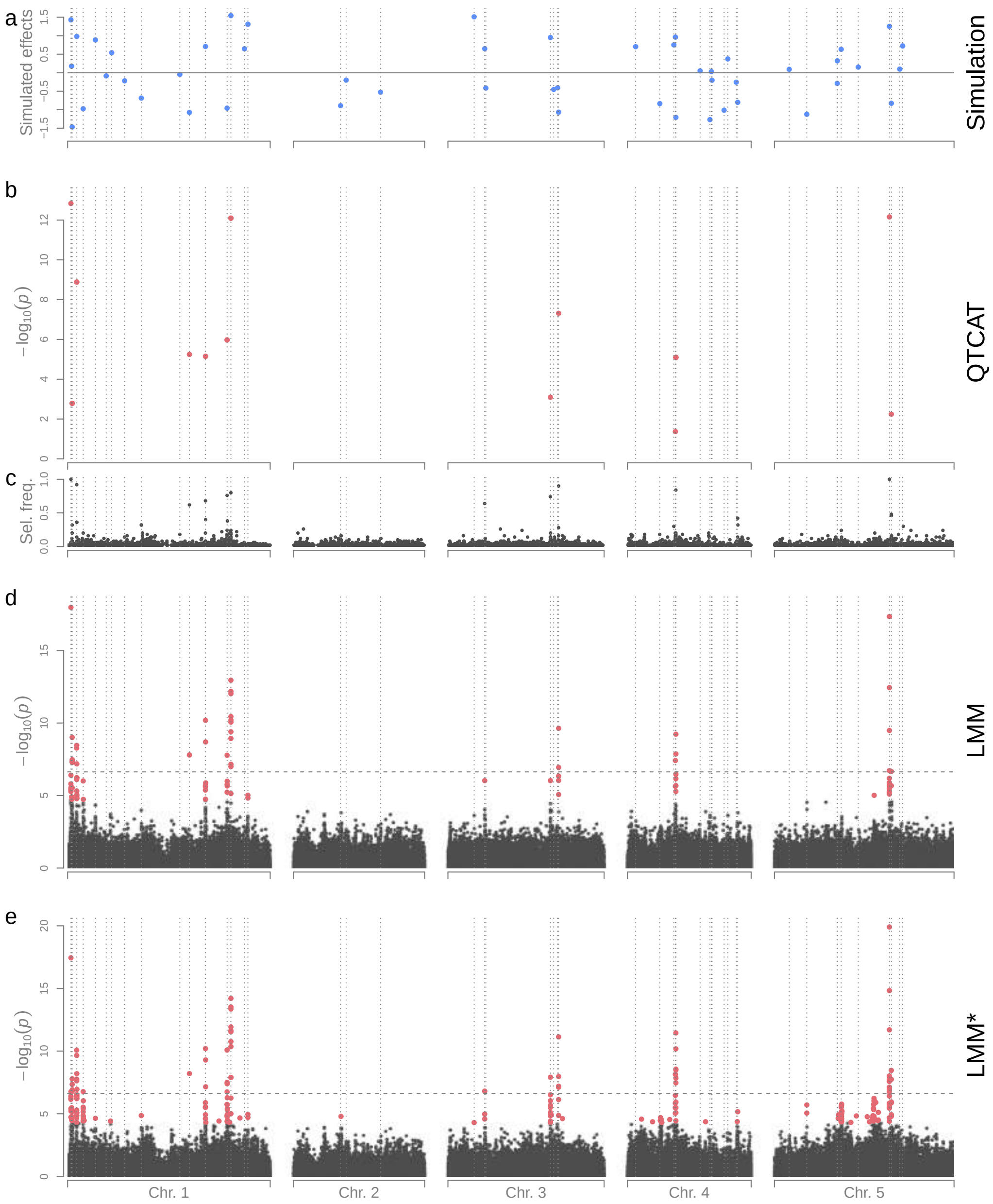

Supplementary Figure 157 Simulation of a GWA analysis based on a structured population with a heritability of 0.7 (run 57). (**a**) Simulation of 50 effects randomly drawn from a normal distribution and assigned to random markers. Markers with effect are highlighted with dashed lines. (**b**) Significant QTCs found by QTCAT. (**c**) LASSO selection frequency for each marker during the 50 iterations of QTCAT. (**d**) Manhattan plot of the LMM analysis. The horizontal dashed line depicts the significance threshold when controlling the multiple testing with FWER, whereas the red markers are significantly associated when controlling with FDR. (**e**) The Manhattan plot of the LMM* analysis. GRM was estimated without markers on the chromosome of the actual testing position. The results are shown as in (d).

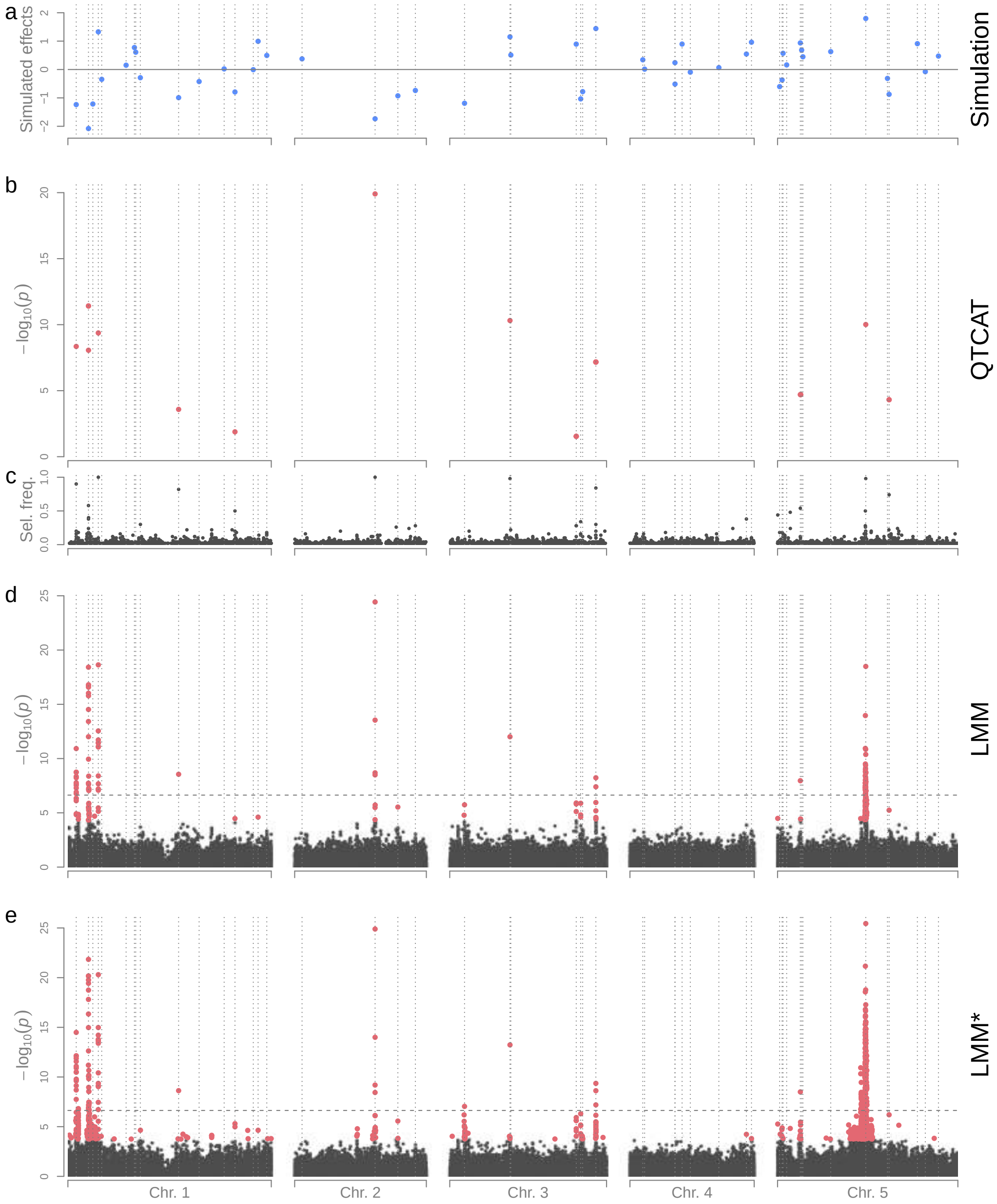

Supplementary Figure 158 Simulation of a GWA analysis based on a structured population with a heritability of 0.7 (run 58). (**a**) Simulation of 50 effects randomly drawn from a normal distribution and assigned to random markers. Markers with effect are highlighted with dashed lines. (**b**) Significant QTCs found by QTCAT. (**c**) LASSO selection frequency for each marker during the 50 iterations of QTCAT. (**d**) Manhattan plot of the LMM analysis. The horizontal dashed line depicts the significance threshold when controlling the multiple testing with FWER, whereas the red markers are significantly associated when controlling with FDR. (**e**) The Manhattan plot of the LMM* analysis. GRM was estimated without markers on the chromosome of the actual testing position. The results are shown as in (d).

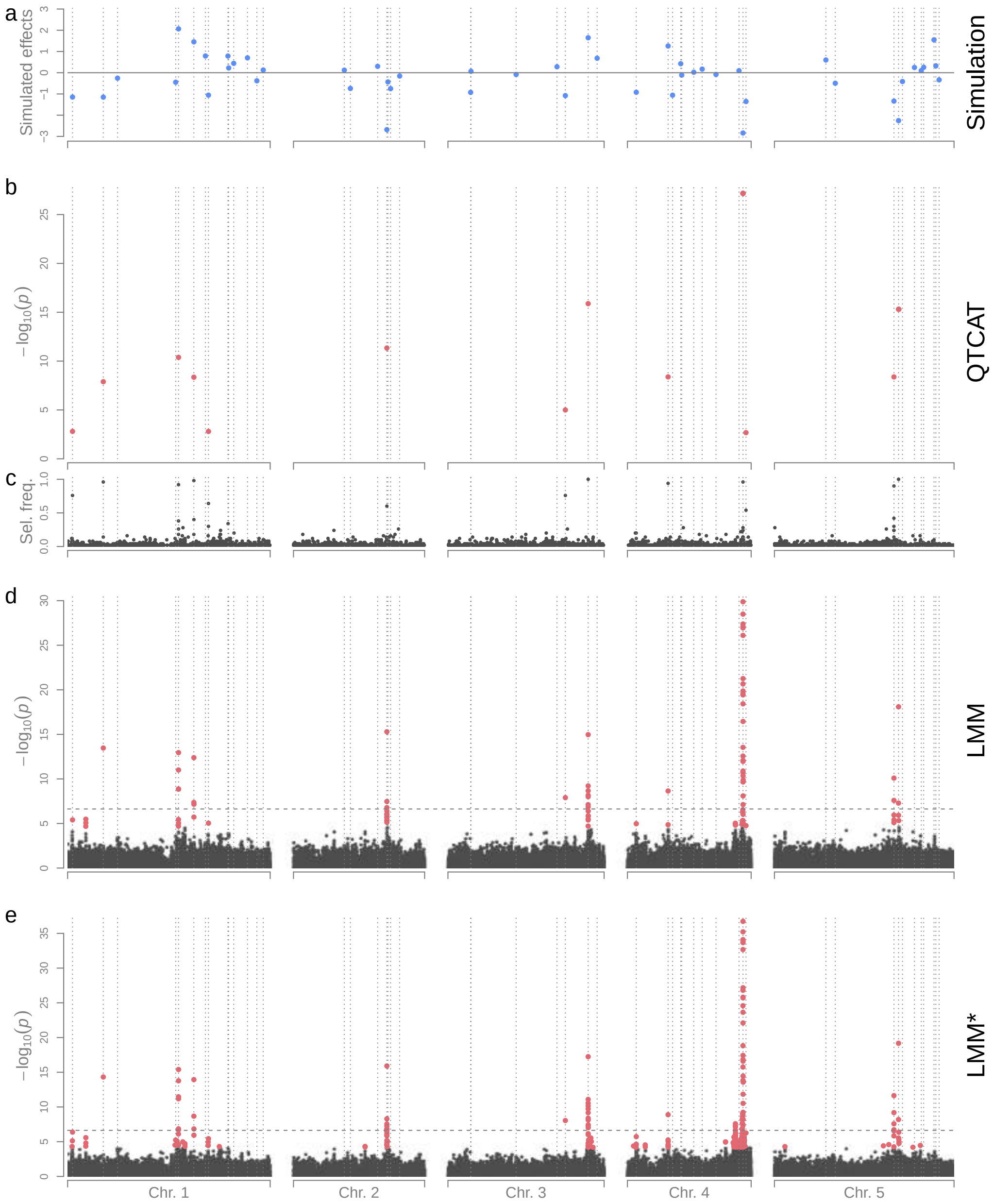

Supplementary Figure 159 Simulation of a GWA analysis based on a structured population with a heritability of 0.7 (run 59). (**a**) Simulation of 50 effects randomly drawn from a normal distribution and assigned to random markers. Markers with effect are highlighted with dashed lines. (**b**) Significant QTCs found by QTCAT. (**c**) LASSO selection frequency for each marker during the 50 iterations of QTCAT. (**d**) Manhattan plot of the LMM analysis. The horizontal dashed line depicts the significance threshold when controlling the multiple testing with FWER, whereas the red markers are significantly associated when controlling with FDR. (**e**) The Manhattan plot of the LMM* analysis. GRM was estimated without markers on the chromosome of the actual testing position. The results are shown as in (d).

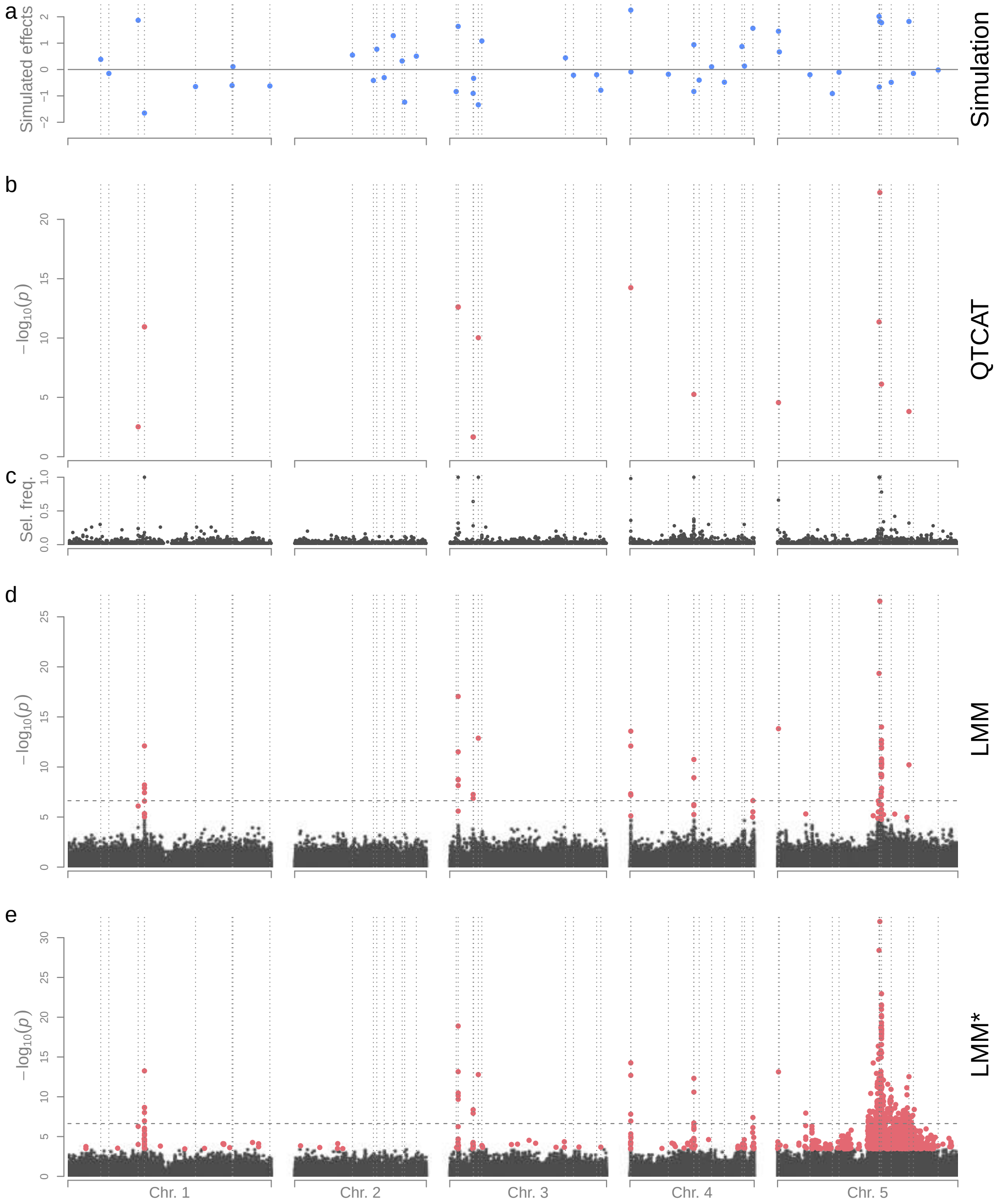

Supplementary Figure 160 Simulation of a GWA analysis based on a structured population with a heritability of 0.7 (run 60). (**a**) Simulation of 50 effects randomly drawn from a normal distribution and assigned to random markers. Markers with effect are highlighted with dashed lines. (**b**) Significant QTCs found by QTCAT. (**c**) LASSO selection frequency for each marker during the 50 iterations of QTCAT. (**d**) Manhattan plot of the LMM analysis. The horizontal dashed line depicts the significance threshold when controlling the multiple testing with FWER, whereas the red markers are significantly associated when controlling with FDR. (**e**) The Manhattan plot of the LMM* analysis. GRM was estimated without markers on the chromosome of the actual testing position. The results are shown as in (d).

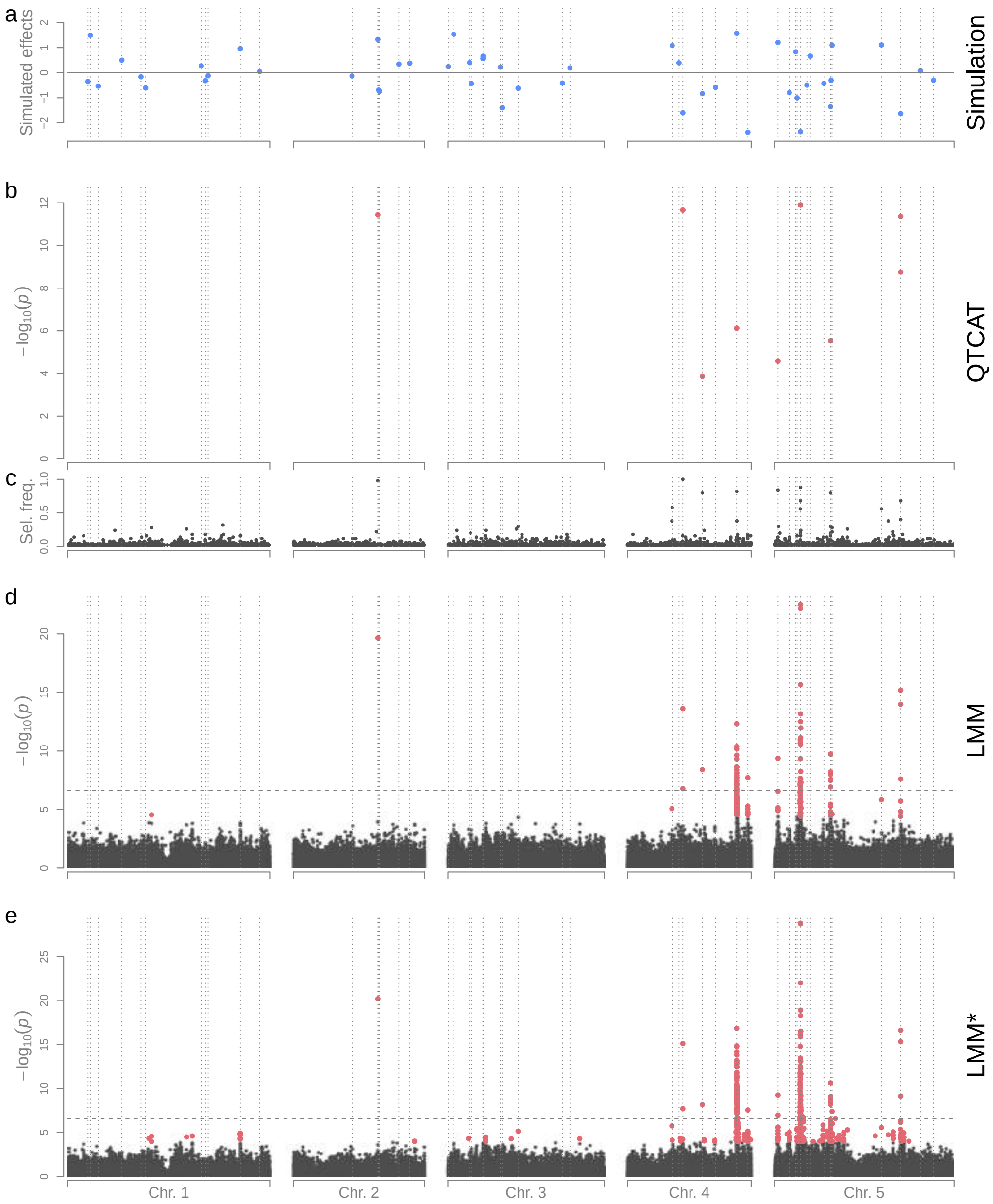

Supplementary Figure 161 Simulation of a GWA analysis based on a structured population with a heritability of 0.7 (run 61). (**a**) Simulation of 50 effects randomly drawn from a normal distribution and assigned to random markers. Markers with effect are highlighted with dashed lines. (**b**) Significant QTCs found by QTCAT. (**c**) LASSO selection frequency for each marker during the 50 iterations of QTCAT. (**d**) Manhattan plot of the LMM analysis. The horizontal dashed line depicts the significance threshold when controlling the multiple testing with FWER, whereas the red markers are significantly associated when controlling with FDR. (**e**) The Manhattan plot of the LMM* analysis. GRM was estimated without markers on the chromosome of the actual testing position. The results are shown as in (d).

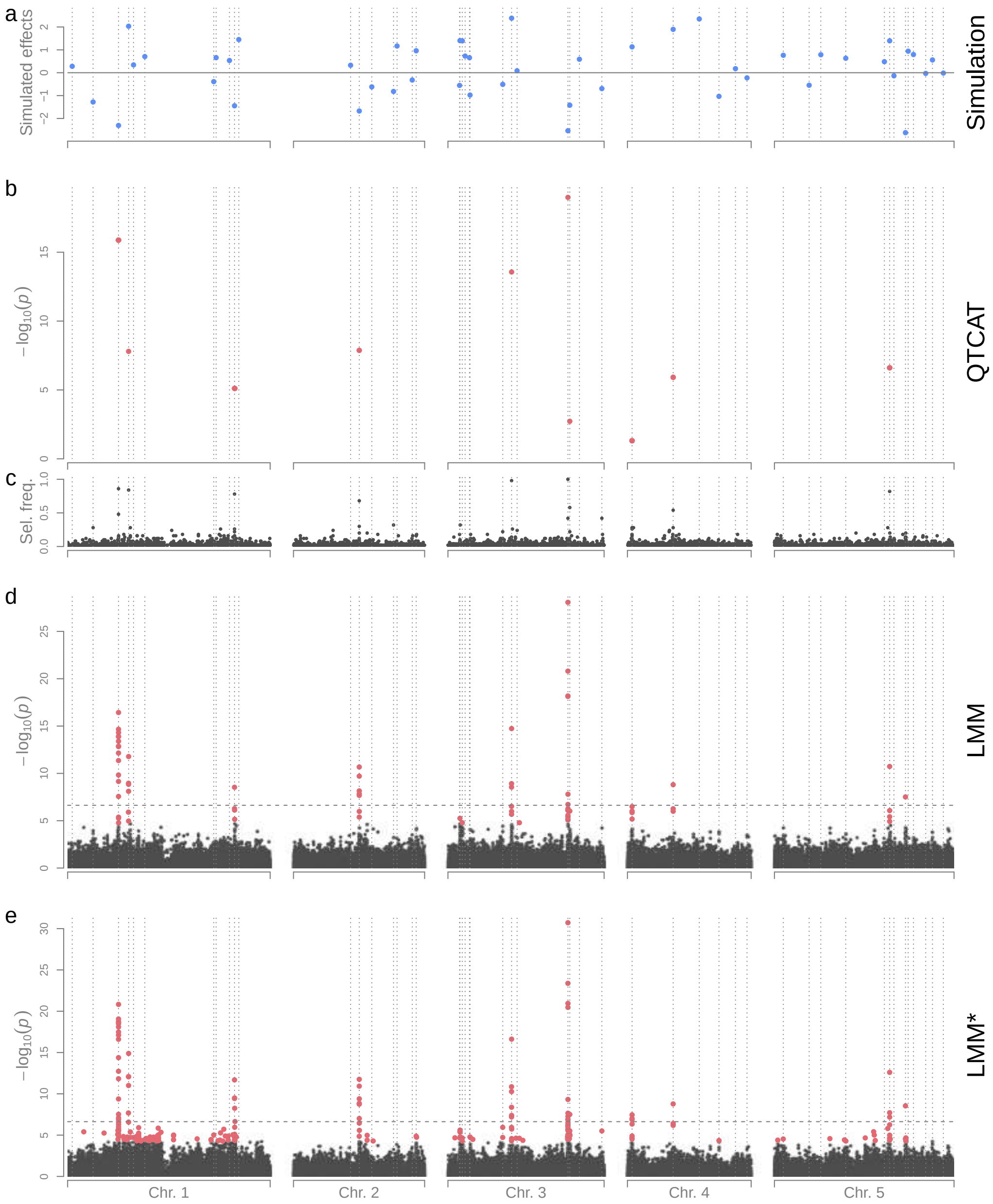

Supplementary Figure 162 Simulation of a GWA analysis based on a structured population with a heritability of 0.7 (run 62). (**a**) Simulation of 50 effects randomly drawn from a normal distribution and assigned to random markers. Markers with effect are highlighted with dashed lines. (**b**) Significant QTCs found by QTCAT. (**c**) LASSO selection frequency for each marker during the 50 iterations of QTCAT. (**d**) Manhattan plot of the LMM analysis. The horizontal dashed line depicts the significance threshold when controlling the multiple testing with FWER, whereas the red markers are significantly associated when controlling with FDR. (**e**) The Manhattan plot of the LMM* analysis. GRM was estimated without markers on the chromosome of the actual testing position. The results are shown as in (d).

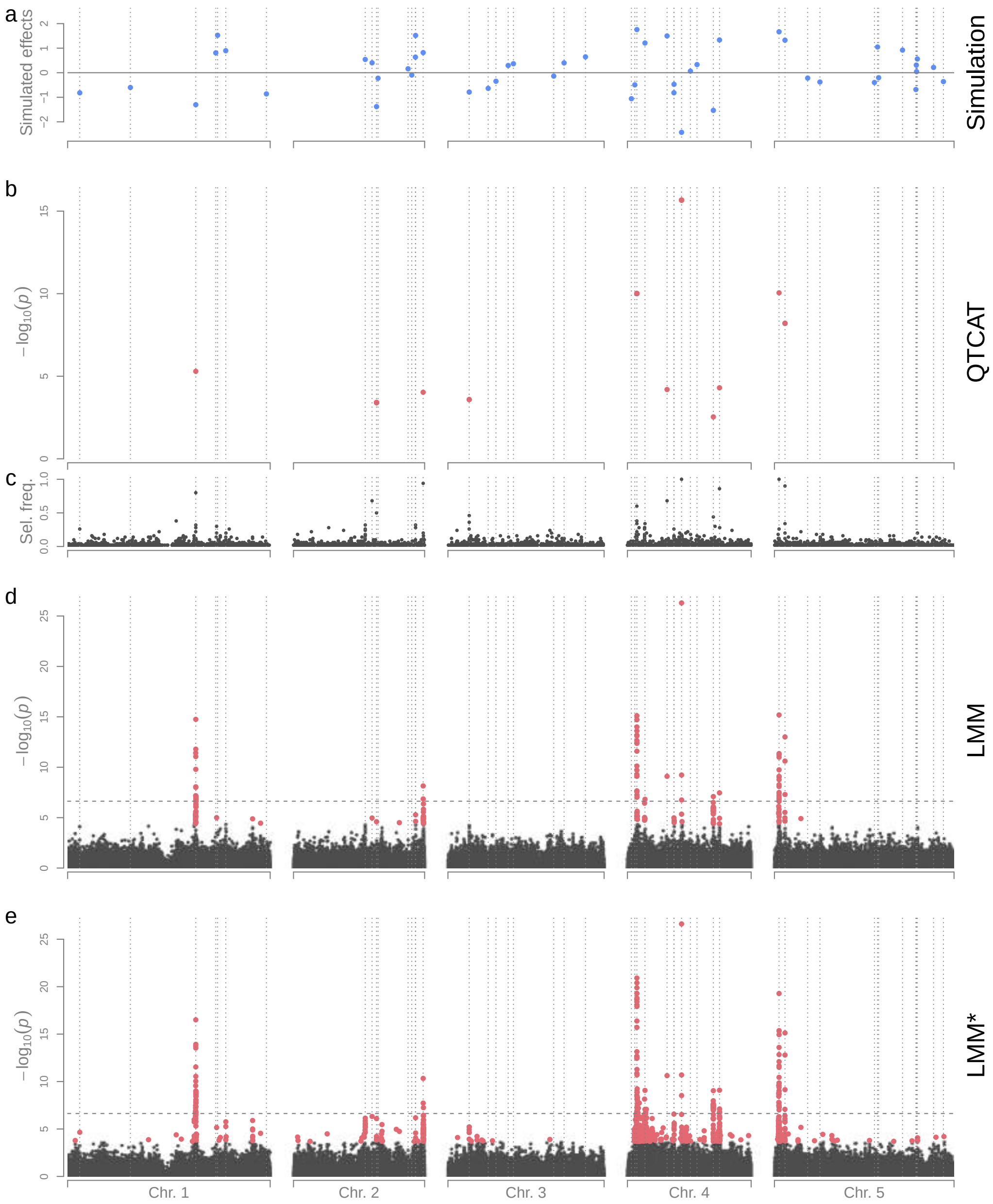

Supplementary Figure 163 Simulation of a GWA analysis based on a structured population with a heritability of 0.7 (run 63). (**a**) Simulation of 50 effects randomly drawn from a normal distribution and assigned to random markers. Markers with effect are highlighted with dashed lines. (**b**) Significant QTCs found by QTCAT. (**c**) LASSO selection frequency for each marker during the 50 iterations of QTCAT. (**d**) Manhattan plot of the LMM analysis. The horizontal dashed line depicts the significance threshold when controlling the multiple testing with FWER, whereas the red markers are significantly associated when controlling with FDR. (**e**) The Manhattan plot of the LMM* analysis. GRM was estimated without markers on the chromosome of the actual testing position. The results are shown as in (d).

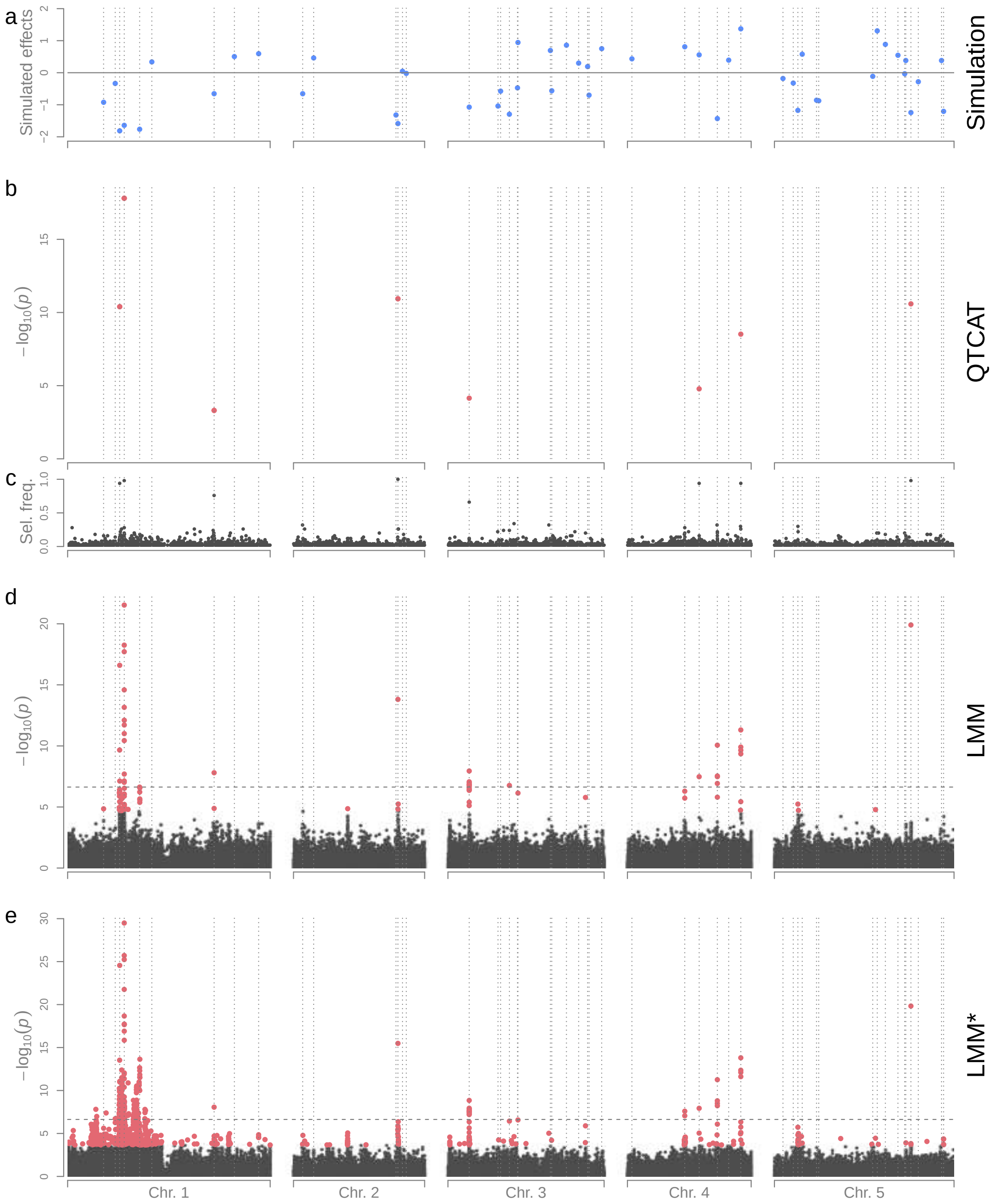

Supplementary Figure 164 Simulation of a GWA analysis based on a structured population with a heritability of 0.7 (run 64). (**a**) Simulation of 50 effects randomly drawn from a normal distribution and assigned to random markers. Markers with effect are highlighted with dashed lines. (**b**) Significant QTCs found by QTCAT. (**c**) LASSO selection frequency for each marker during the 50 iterations of QTCAT. (**d**) Manhattan plot of the LMM analysis. The horizontal dashed line depicts the significance threshold when controlling the multiple testing with FWER, whereas the red markers are significantly associated when controlling with FDR. (**e**) The Manhattan plot of the LMM* analysis. GRM was estimated without markers on the chromosome of the actual testing position. The results are shown as in (d).

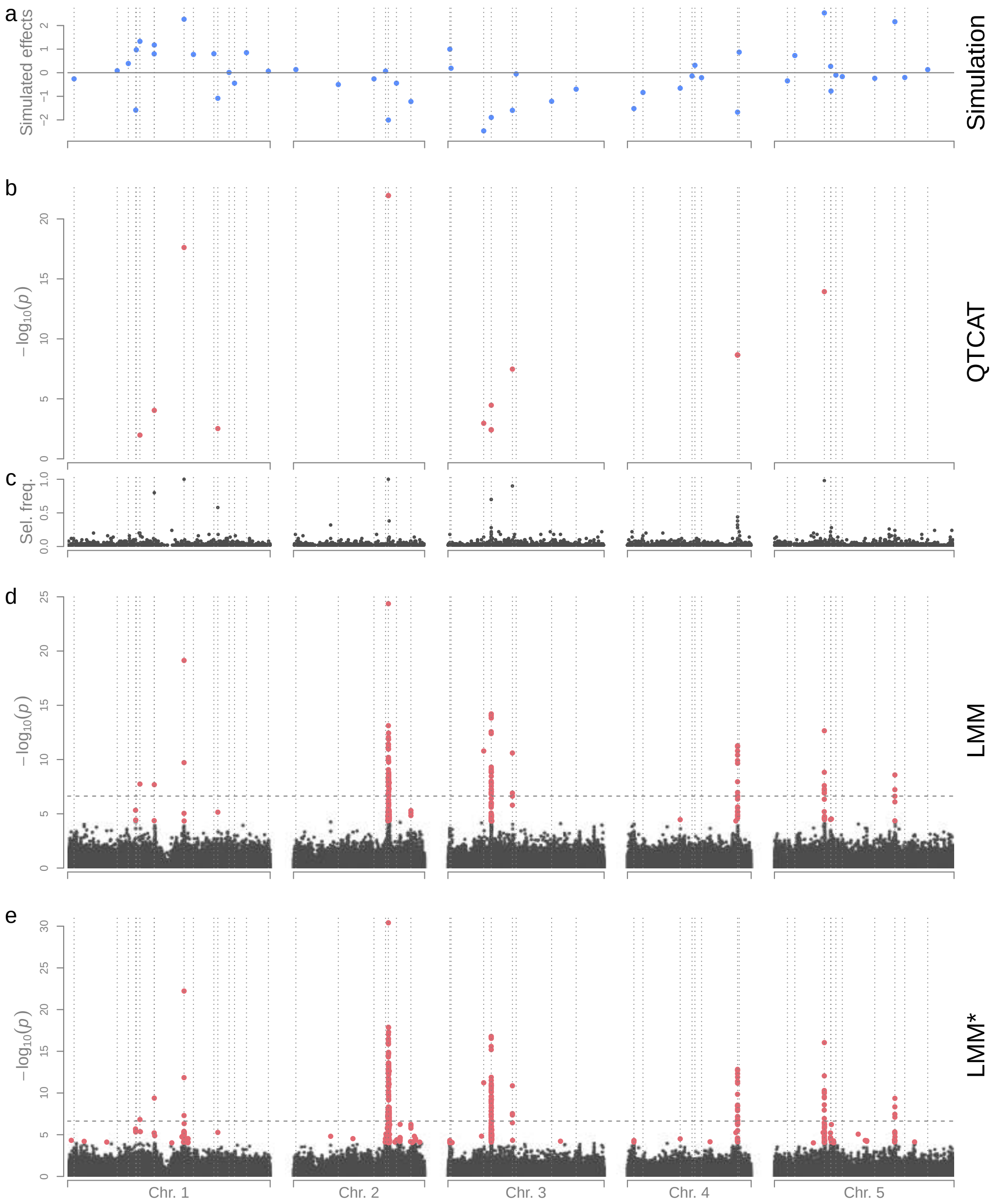

Supplementary Figure 165 Simulation of a GWA analysis based on a structured population with a heritability of 0.7 (run 65). (**a**) Simulation of 50 effects randomly drawn from a normal distribution and assigned to random markers. Markers with effect are highlighted with dashed lines. (**b**) Significant QTCs found by QTCAT. (**c**) LASSO selection frequency for each marker during the 50 iterations of QTCAT. (**d**) Manhattan plot of the LMM analysis. The horizontal dashed line depicts the significance threshold when controlling the multiple testing with FWER, whereas the red markers are significantly associated when controlling with FDR. (**e**) The Manhattan plot of the LMM* analysis. GRM was estimated without markers on the chromosome of the actual testing position. The results are shown as in (d).

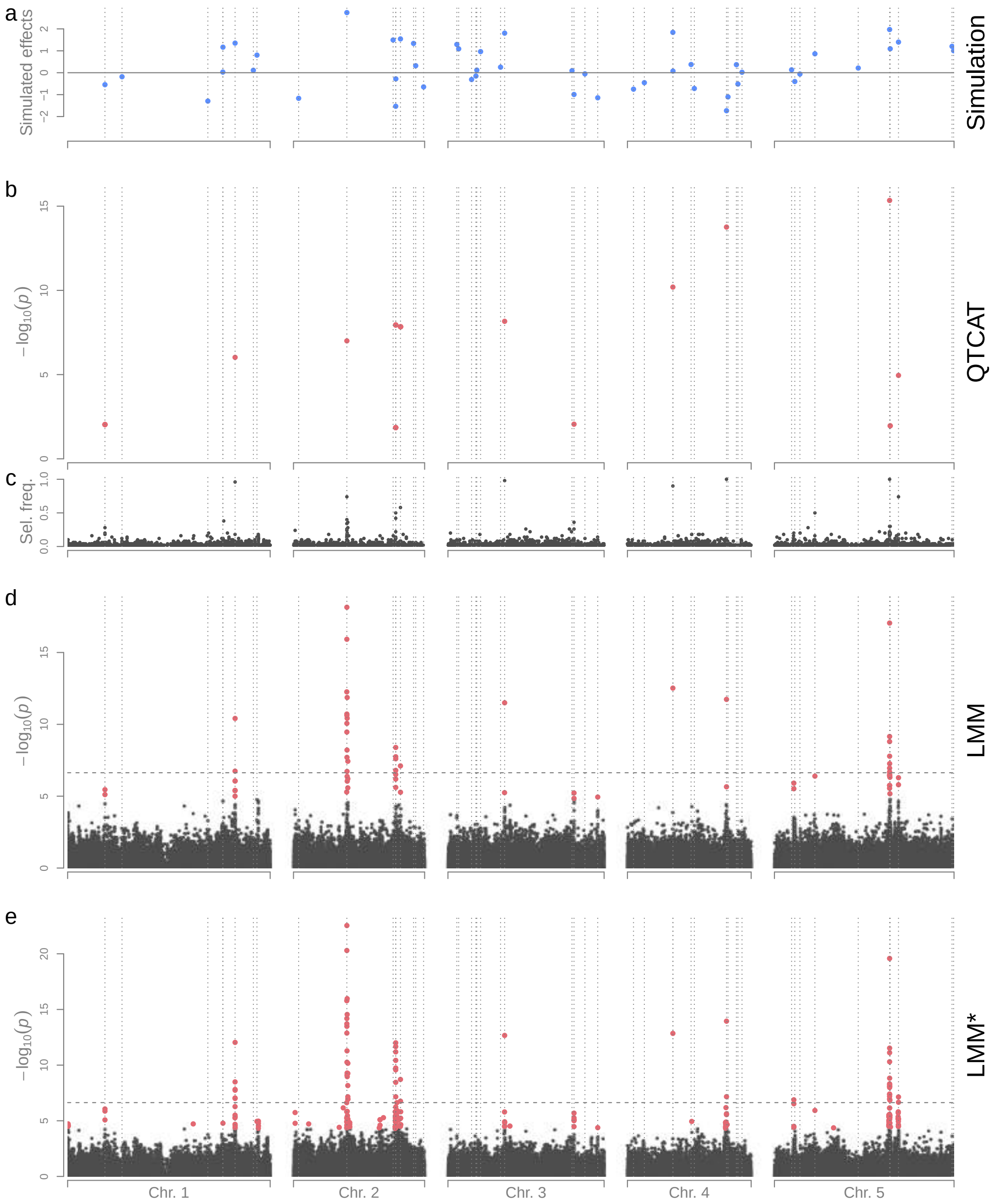

Supplementary Figure 166 Simulation of a GWA analysis based on a structured population with a heritability of 0.7 (run 66). (**a**) Simulation of 50 effects randomly drawn from a normal distribution and assigned to random markers. Markers with effect are highlighted with dashed lines. (**b**) Significant QTCs found by QTCAT. (**c**) LASSO selection frequency for each marker during the 50 iterations of QTCAT. (**d**) Manhattan plot of the LMM analysis. The horizontal dashed line depicts the significance threshold when controlling the multiple testing with FWER, whereas the red markers are significantly associated when controlling with FDR. (**e**) The Manhattan plot of the LMM* analysis. GRM was estimated without markers on the chromosome of the actual testing position. The results are shown as in (d).

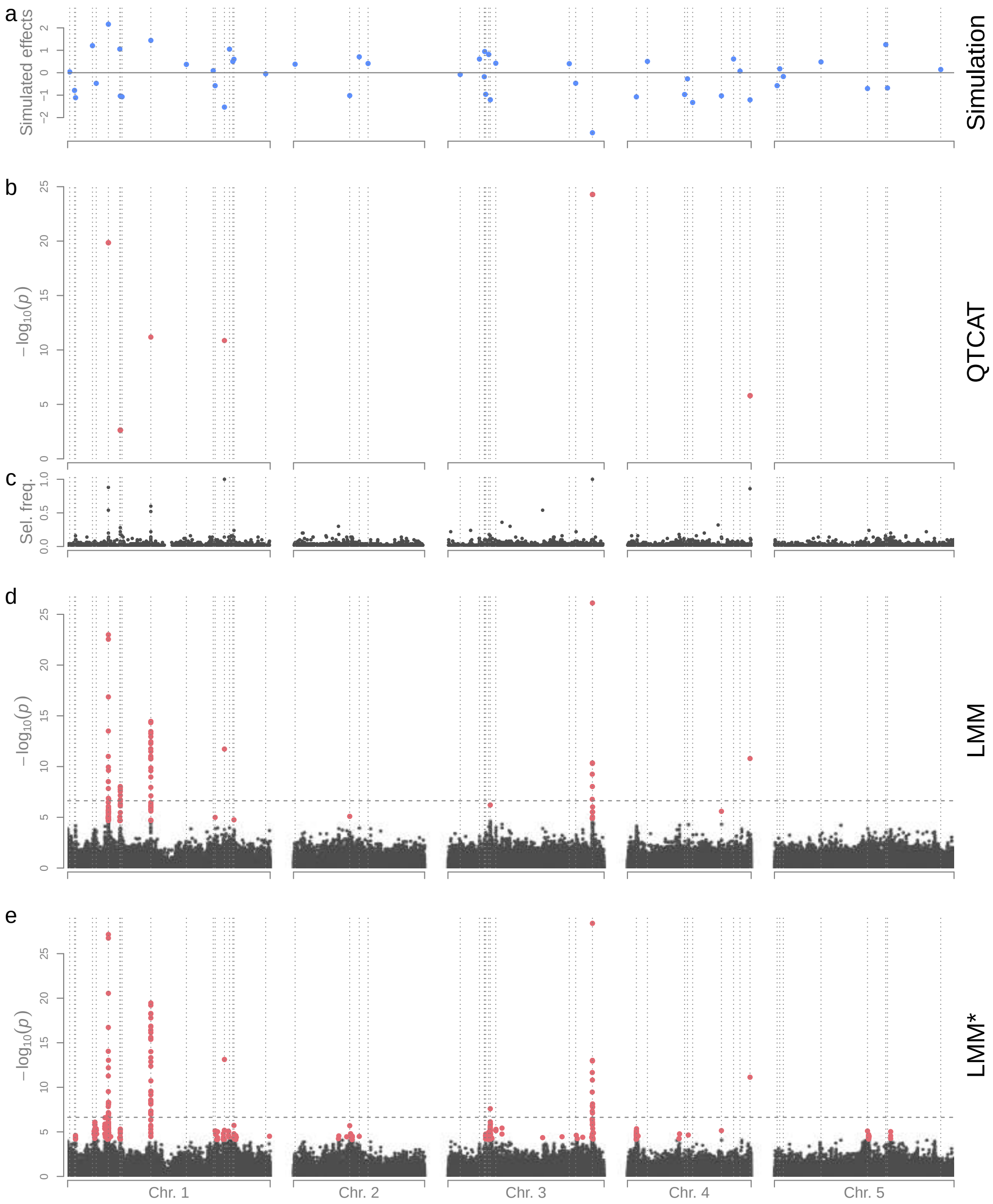

Supplementary Figure 167 Simulation of a GWA analysis based on a structured population with a heritability of 0.7 (run 67). (**a**) Simulation of 50 effects randomly drawn from a normal distribution and assigned to random markers. Markers with effect are highlighted with dashed lines. (**b**) Significant QTCs found by QTCAT. (**c**) LASSO selection frequency for each marker during the 50 iterations of QTCAT. (**d**) Manhattan plot of the LMM analysis. The horizontal dashed line depicts the significance threshold when controlling the multiple testing with FWER, whereas the red markers are significantly associated when controlling with FDR. (**e**) The Manhattan plot of the LMM* analysis. GRM was estimated without markers on the chromosome of the actual testing position. The results are shown as in (d).

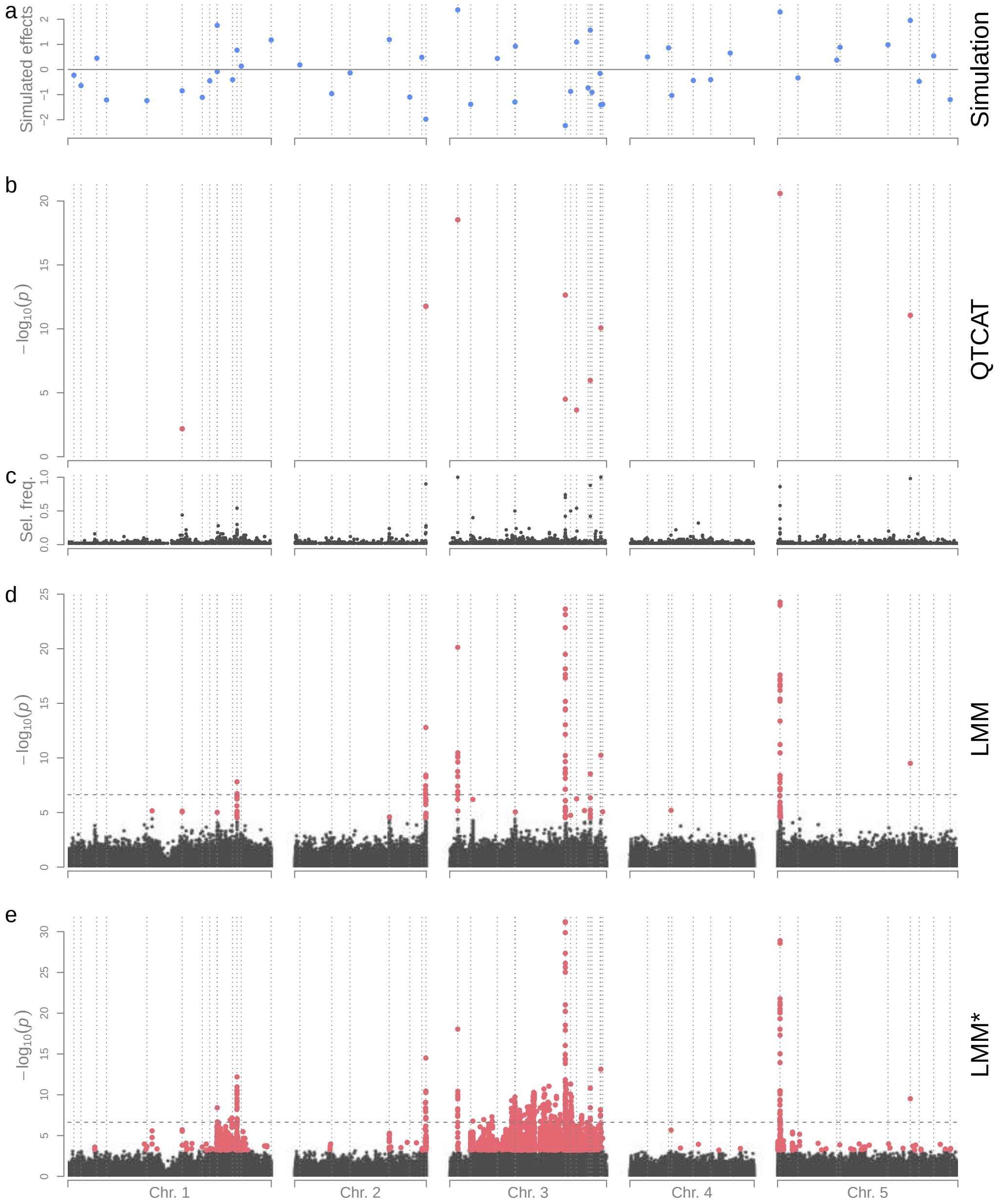

Supplementary Figure 168 Simulation of a GWA analysis based on a structured population with a heritability of 0.7 (run 68). (**a**) Simulation of 50 effects randomly drawn from a normal distribution and assigned to random markers. Markers with effect are highlighted with dashed lines. (**b**) Significant QTCs found by QTCAT. (**c**) LASSO selection frequency for each marker during the 50 iterations of QTCAT. (**d**) Manhattan plot of the LMM analysis. The horizontal dashed line depicts the significance threshold when controlling the multiple testing with FWER, whereas the red markers are significantly associated when controlling with FDR. (**e**) The Manhattan plot of the LMM* analysis. GRM was estimated without markers on the chromosome of the actual testing position. The results are shown as in (d).

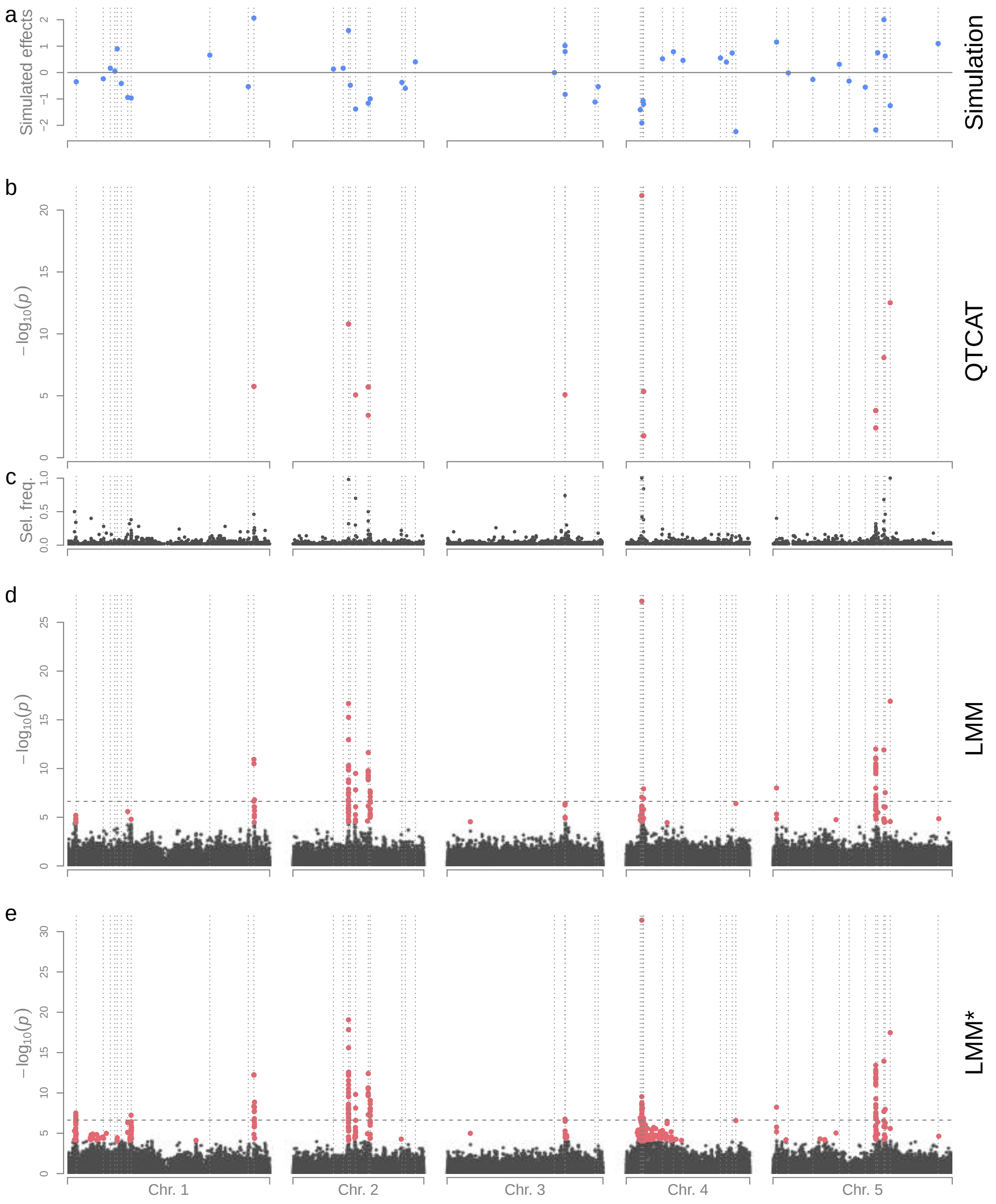

Supplementary Figure 169 Simulation of a GWA analysis based on a structured population with a heritability of 0.7 (run 69). (**a**) Simulation of 50 effects randomly drawn from a normal distribution and assigned to random markers. Markers with effect are highlighted with dashed lines. (**b**) Significant QTCs found by QTCAT. (**c**) LASSO selection frequency for each marker during the 50 iterations of QTCAT. (**d**) Manhattan plot of the LMM analysis. The horizontal dashed line depicts the significance threshold when controlling the multiple testing with FWER, whereas the red markers are significantly associated when controlling with FDR. (**e**) The Manhattan plot of the LMM* analysis. GRM was estimated without markers on the chromosome of the actual testing position. The results are shown as in (d).

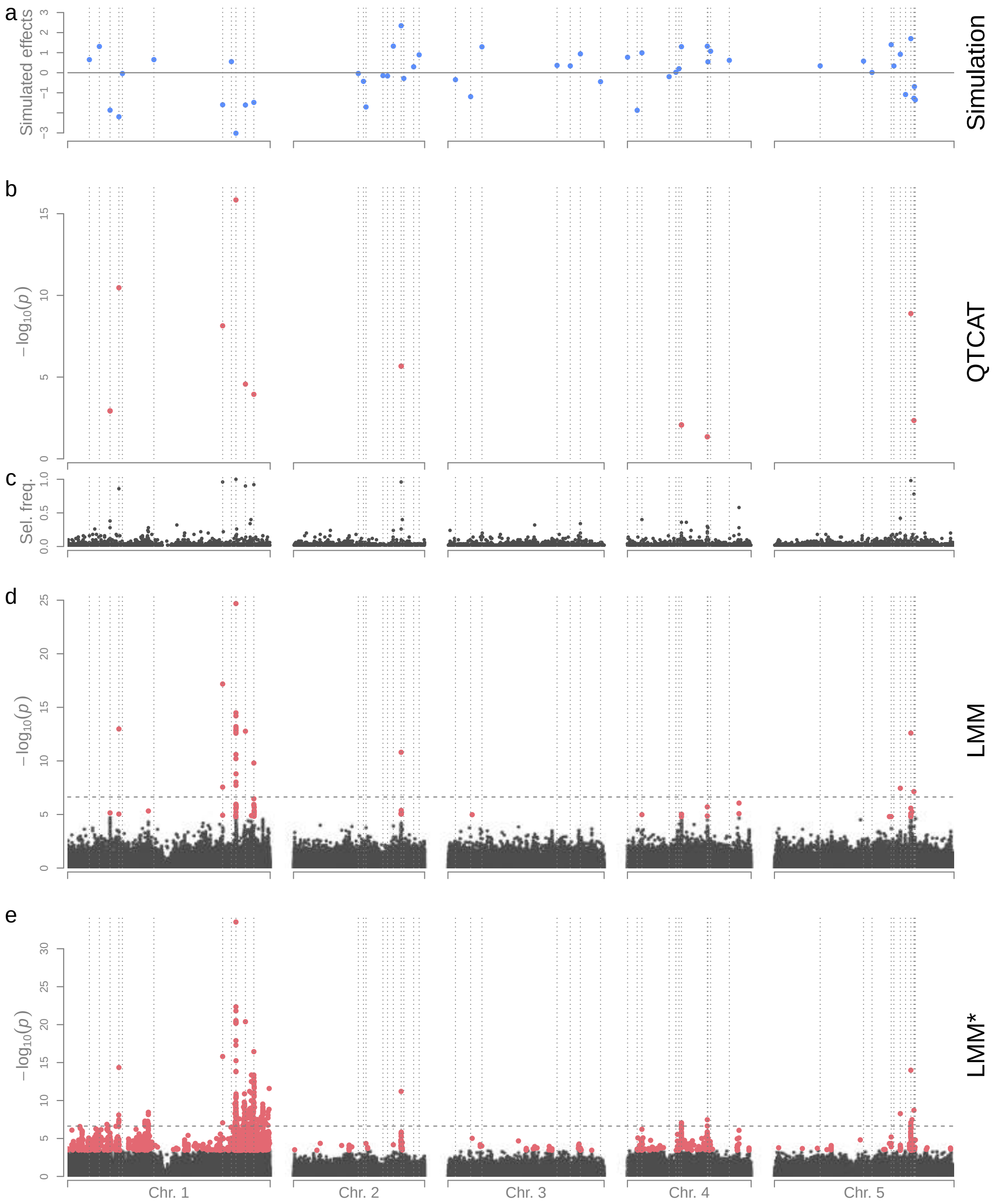

Supplementary Figure 170 Simulation of a GWA analysis based on a structured population with a heritability of 0.7 (run 70). (**a**) Simulation of 50 effects randomly drawn from a normal distribution and assigned to random markers. Markers with effect are highlighted with dashed lines. (**b**) Significant QTCs found by QTCAT. (**c**) LASSO selection frequency for each marker during the 50 iterations of QTCAT. (**d**) Manhattan plot of the LMM analysis. The horizontal dashed line depicts the significance threshold when controlling the multiple testing with FWER, whereas the red markers are significantly associated when controlling with FDR. (**e**) The Manhattan plot of the LMM* analysis. GRM was estimated without markers on the chromosome of the actual testing position. The results are shown as in (d).

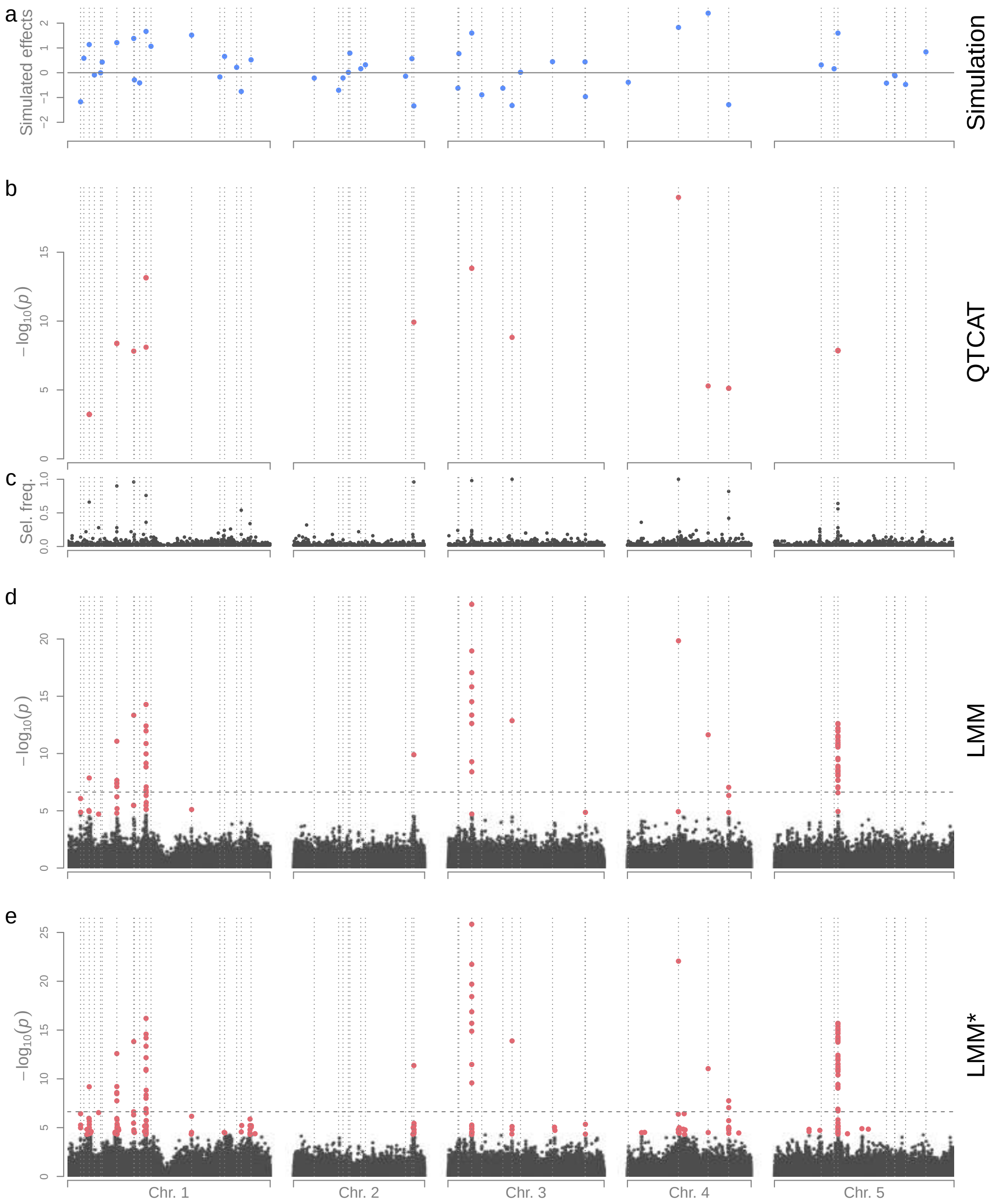

Supplementary Figure 171 Simulation of a GWA analysis based on a structured population with a heritability of 0.7 (run 71). (**a**) Simulation of 50 effects randomly drawn from a normal distribution and assigned to random markers. Markers with effect are highlighted with dashed lines. (**b**) Significant QTCs found by QTCAT. (**c**) LASSO selection frequency for each marker during the 50 iterations of QTCAT. (**d**) Manhattan plot of the LMM analysis. The horizontal dashed line depicts the significance threshold when controlling the multiple testing with FWER, whereas the red markers are significantly associated when controlling with FDR. (**e**) The Manhattan plot of the LMM* analysis. GRM was estimated without markers on the chromosome of the actual testing position. The results are shown as in (d).

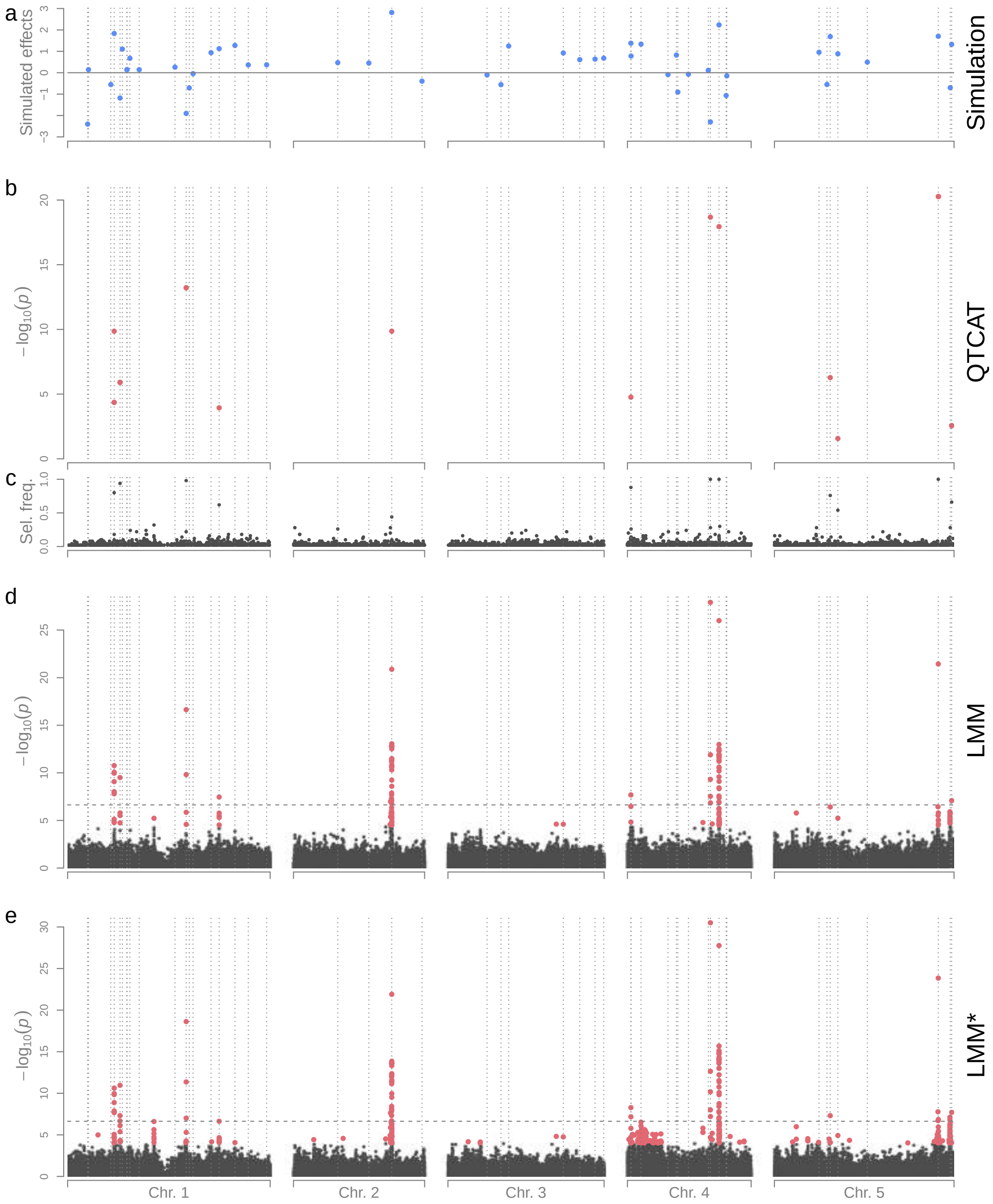

Supplementary Figure 172 Simulation of a GWA analysis based on a structured population with a heritability of 0.7 (run 72). (**a**) Simulation of 50 effects randomly drawn from a normal distribution and assigned to random markers. Markers with effect are highlighted with dashed lines. (**b**) Significant QTCs found by QTCAT. (**c**) LASSO selection frequency for each marker during the 50 iterations of QTCAT. (**d**) Manhattan plot of the LMM analysis. The horizontal dashed line depicts the significance threshold when controlling the multiple testing with FWER, whereas the red markers are significantly associated when controlling with FDR. (**e**) The Manhattan plot of the LMM* analysis. GRM was estimated without markers on the chromosome of the actual testing position. The results are shown as in (d).

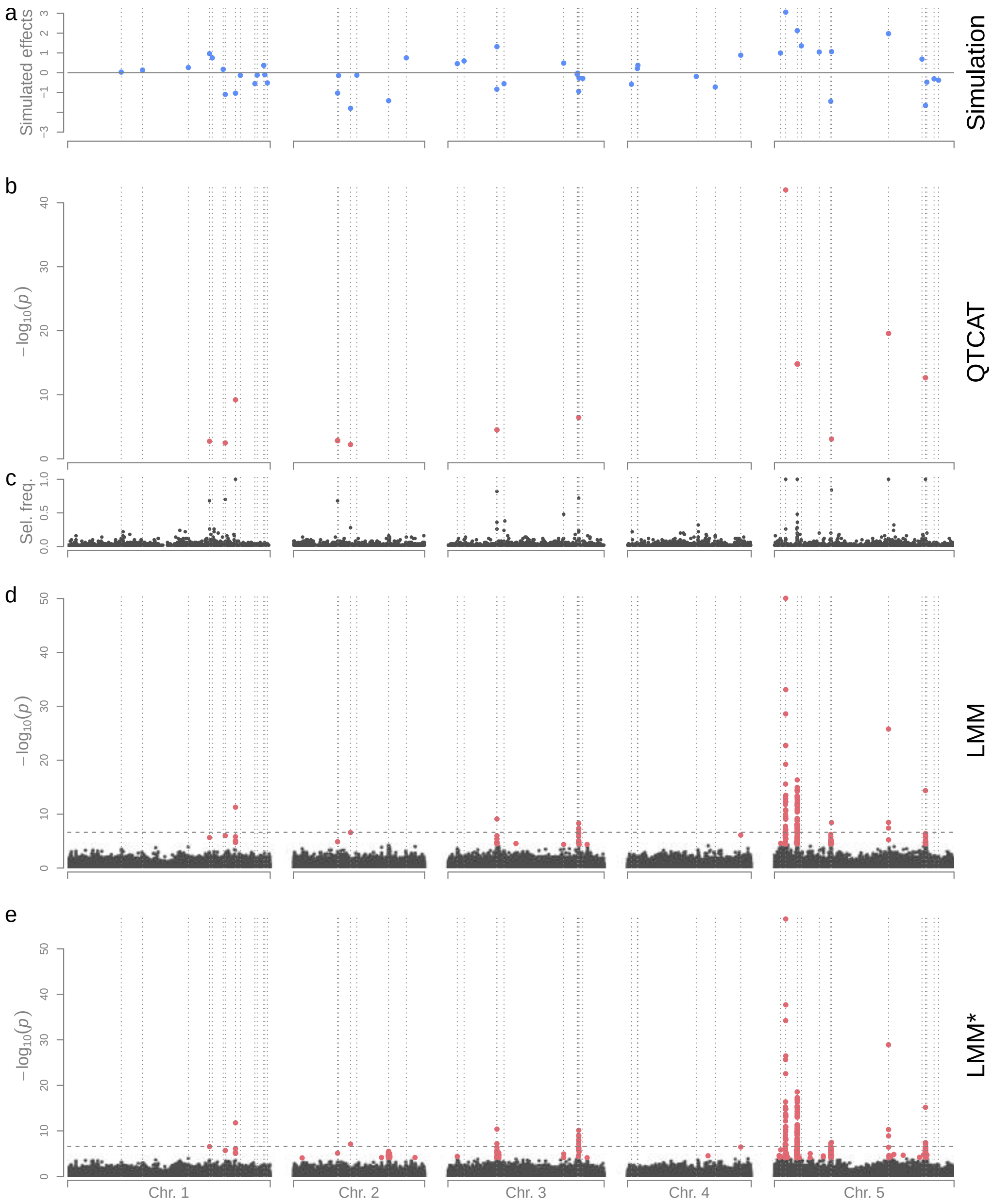

Supplementary Figure 173 Simulation of a GWA analysis based on a structured population with a heritability of 0.7 (run 73). (**a**) Simulation of 50 effects randomly drawn from a normal distribution and assigned to random markers. Markers with effect are highlighted with dashed lines. (**b**) Significant QTCs found by QTCAT. (**c**) LASSO selection frequency for each marker during the 50 iterations of QTCAT. (**d**) Manhattan plot of the LMM analysis. The horizontal dashed line depicts the significance threshold when controlling the multiple testing with FWER, whereas the red markers are significantly associated when controlling with FDR. (**e**) The Manhattan plot of the LMM* analysis. GRM was estimated without markers on the chromosome of the actual testing position. The results are shown as in (d).

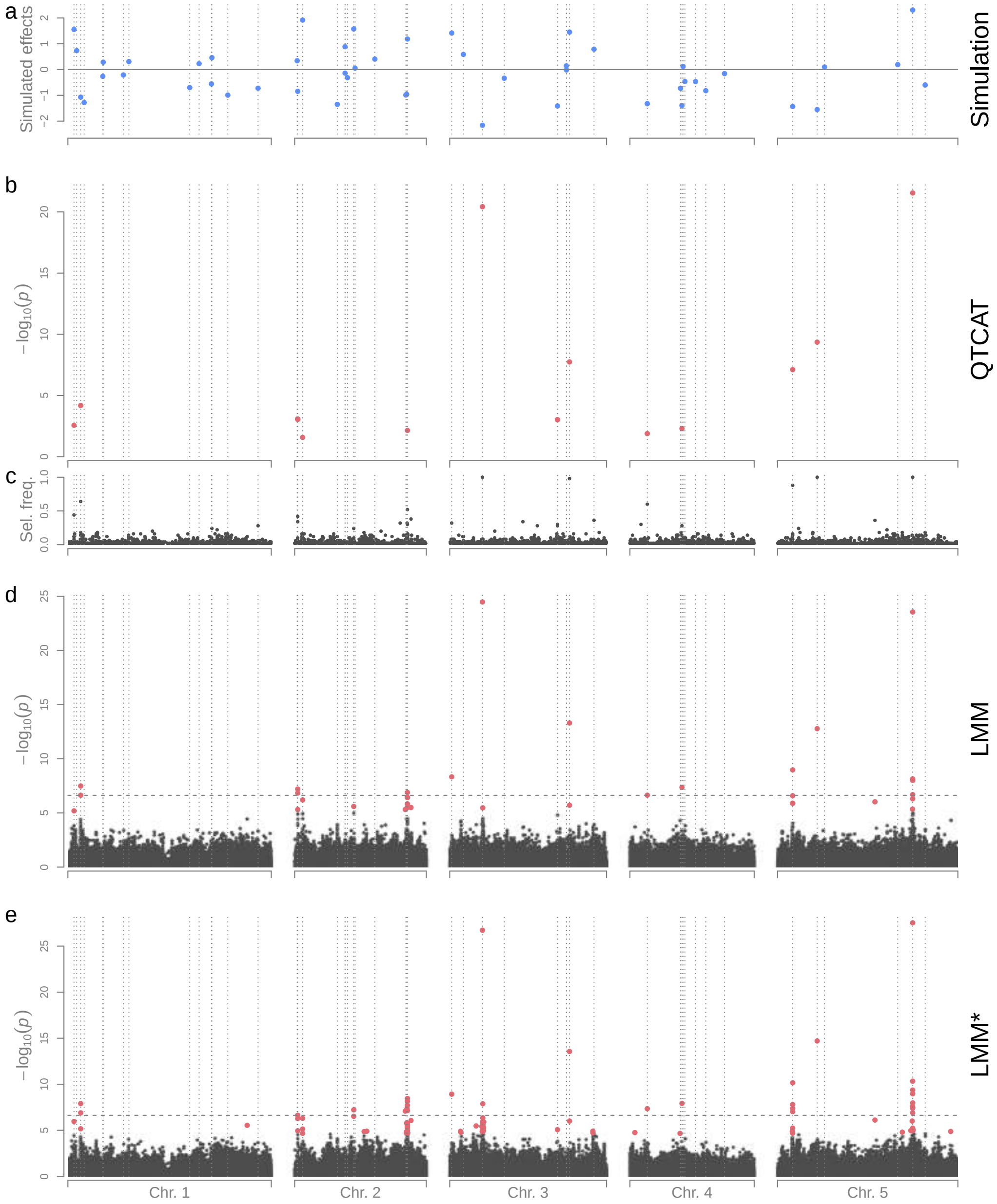

Supplementary Figure 174 Simulation of a GWA analysis based on a structured population with a heritability of 0.7 (run 74). (**a**) Simulation of 50 effects randomly drawn from a normal distribution and assigned to random markers. Markers with effect are highlighted with dashed lines. (**b**) Significant QTCs found by QTCAT. (**c**) LASSO selection frequency for each marker during the 50 iterations of QTCAT. (**d**) Manhattan plot of the LMM analysis. The horizontal dashed line depicts the significance threshold when controlling the multiple testing with FWER, whereas the red markers are significantly associated when controlling with FDR. (**e**) The Manhattan plot of the LMM* analysis. GRM was estimated without markers on the chromosome of the actual testing position. The results are shown as in (d).

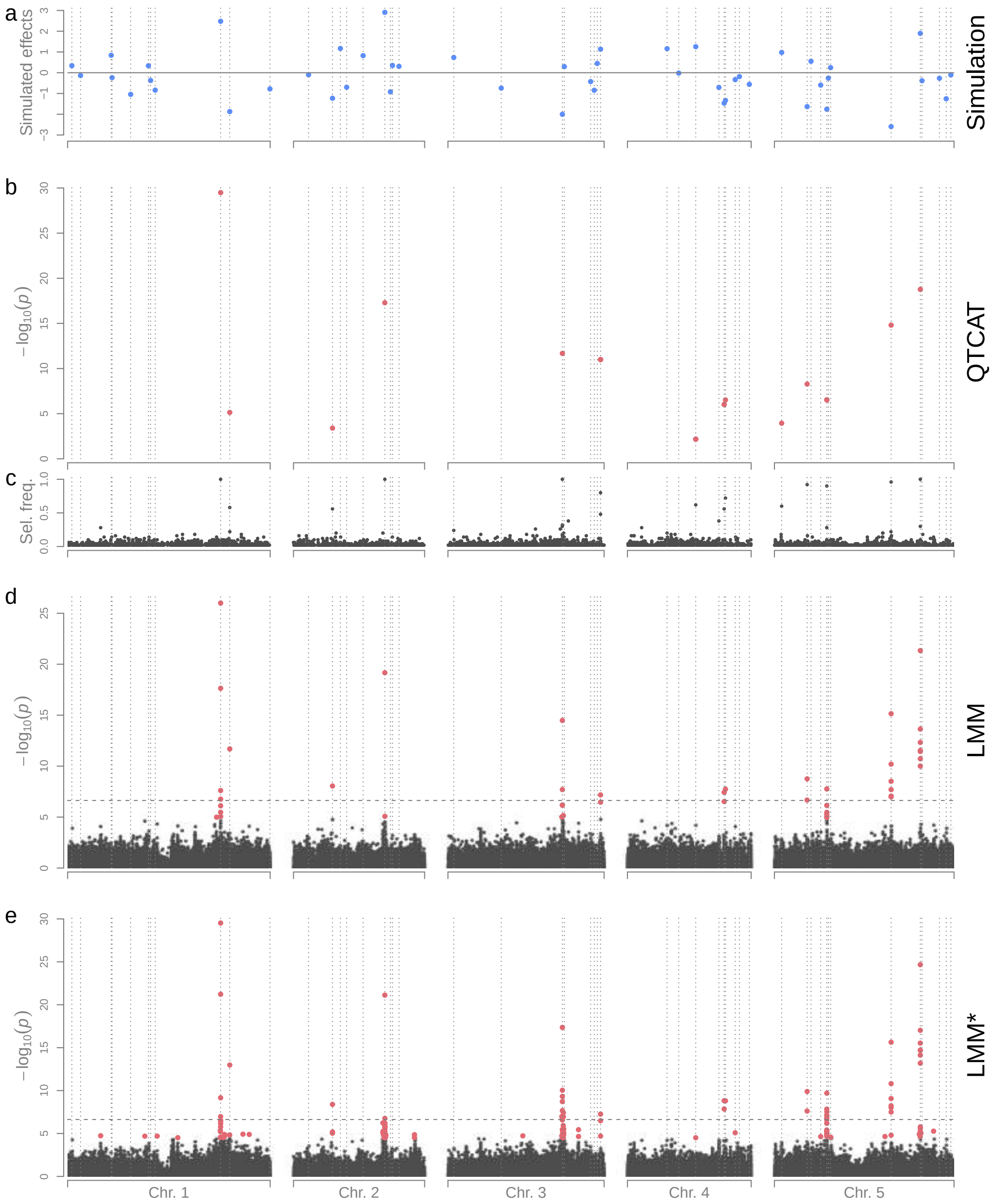

Supplementary Figure 175 Simulation of a GWA analysis based on a structured population with a heritability of 0.7 (run 75). (**a**) Simulation of 50 effects randomly drawn from a normal distribution and assigned to random markers. Markers with effect are highlighted with dashed lines. (**b**) Significant QTCs found by QTCAT. (**c**) LASSO selection frequency for each marker during the 50 iterations of QTCAT. (**d**) Manhattan plot of the LMM analysis. The horizontal dashed line depicts the significance threshold when controlling the multiple testing with FWER, whereas the red markers are significantly associated when controlling with FDR. (**e**) The Manhattan plot of the LMM* analysis. GRM was estimated without markers on the chromosome of the actual testing position. The results are shown as in (d).

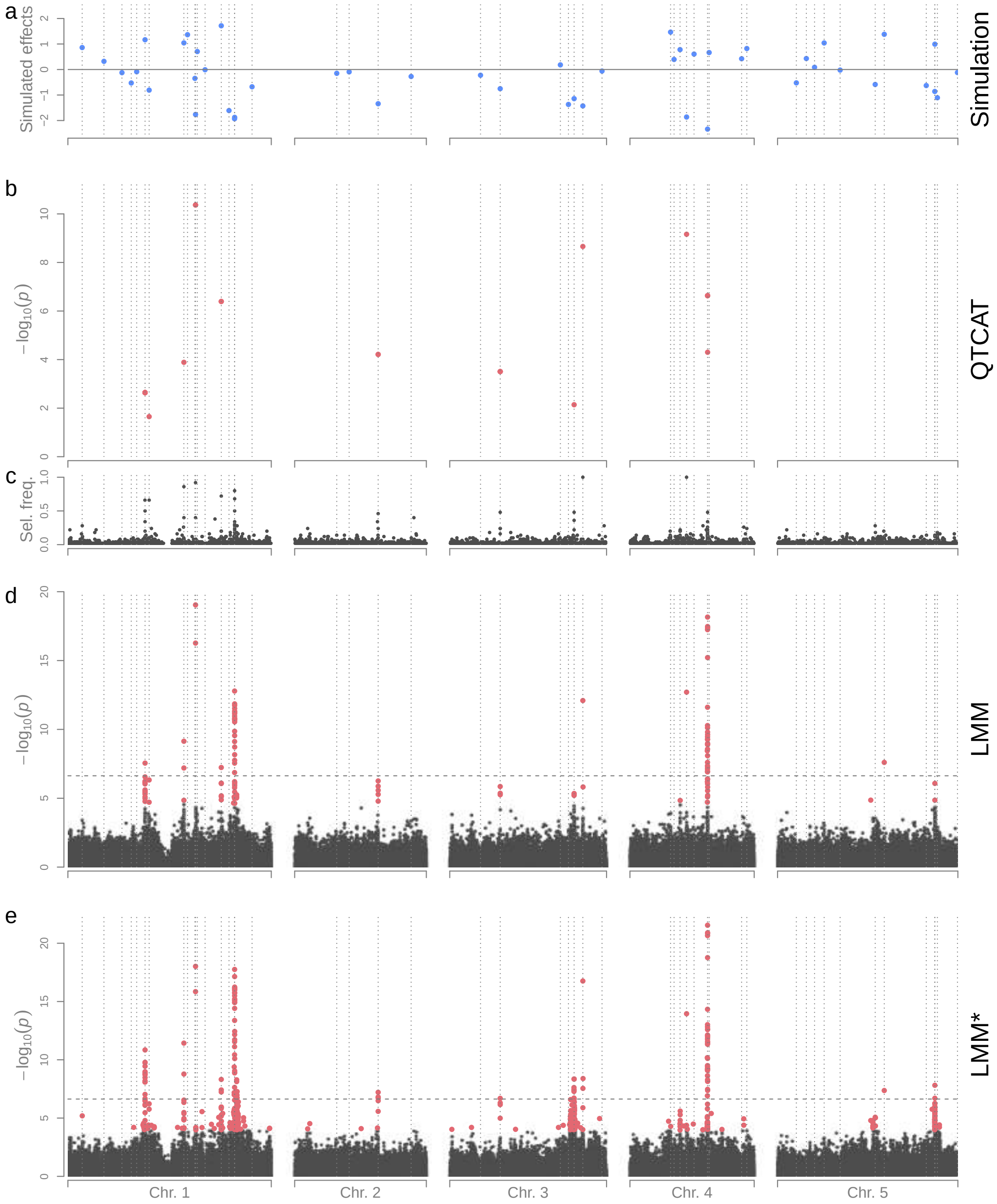

Supplementary Figure 176 Simulation of a GWA analysis based on a structured population with a heritability of 0.7 (run 76). (**a**) Simulation of 50 effects randomly drawn from a normal distribution and assigned to random markers. Markers with effect are highlighted with dashed lines. (**b**) Significant QTCs found by QTCAT. (**c**) LASSO selection frequency for each marker during the 50 iterations of QTCAT. (**d**) Manhattan plot of the LMM analysis. The horizontal dashed line depicts the significance threshold when controlling the multiple testing with FWER, whereas the red markers are significantly associated when controlling with FDR. (**e**) The Manhattan plot of the LMM* analysis. GRM was estimated without markers on the chromosome of the actual testing position. The results are shown as in (d).

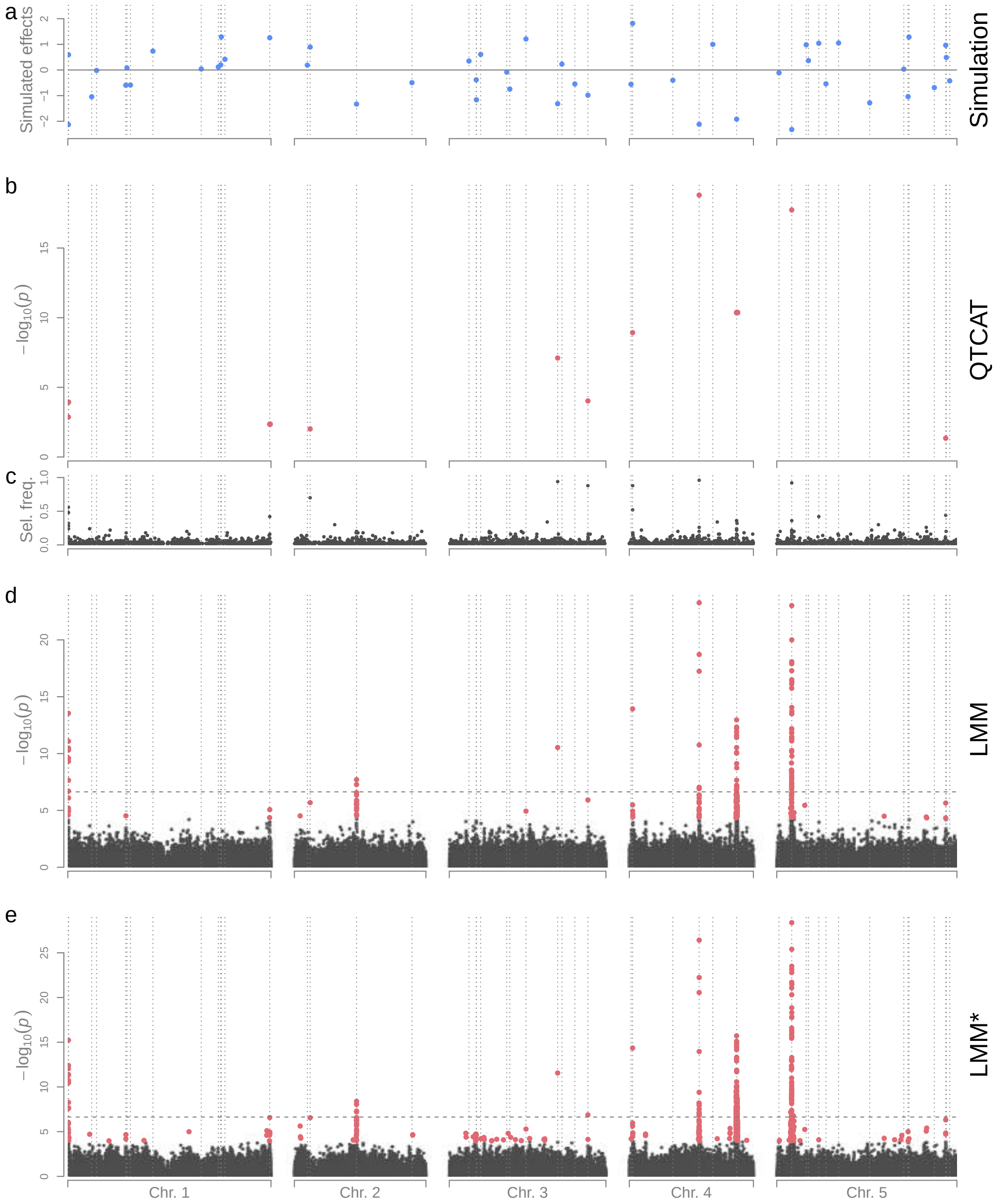

Supplementary Figure 177 Simulation of a GWA analysis based on a structured population with a heritability of 0.7 (run 77). (**a**) Simulation of 50 effects randomly drawn from a normal distribution and assigned to random markers. Markers with effect are highlighted with dashed lines. (**b**) Significant QTCs found by QTCAT. (**c**) LASSO selection frequency for each marker during the 50 iterations of QTCAT. (**d**) Manhattan plot of the LMM analysis. The horizontal dashed line depicts the significance threshold when controlling the multiple testing with FWER, whereas the red markers are significantly associated when controlling with FDR. (**e**) The Manhattan plot of the LMM* analysis. GRM was estimated without markers on the chromosome of the actual testing position. The results are shown as in (d).

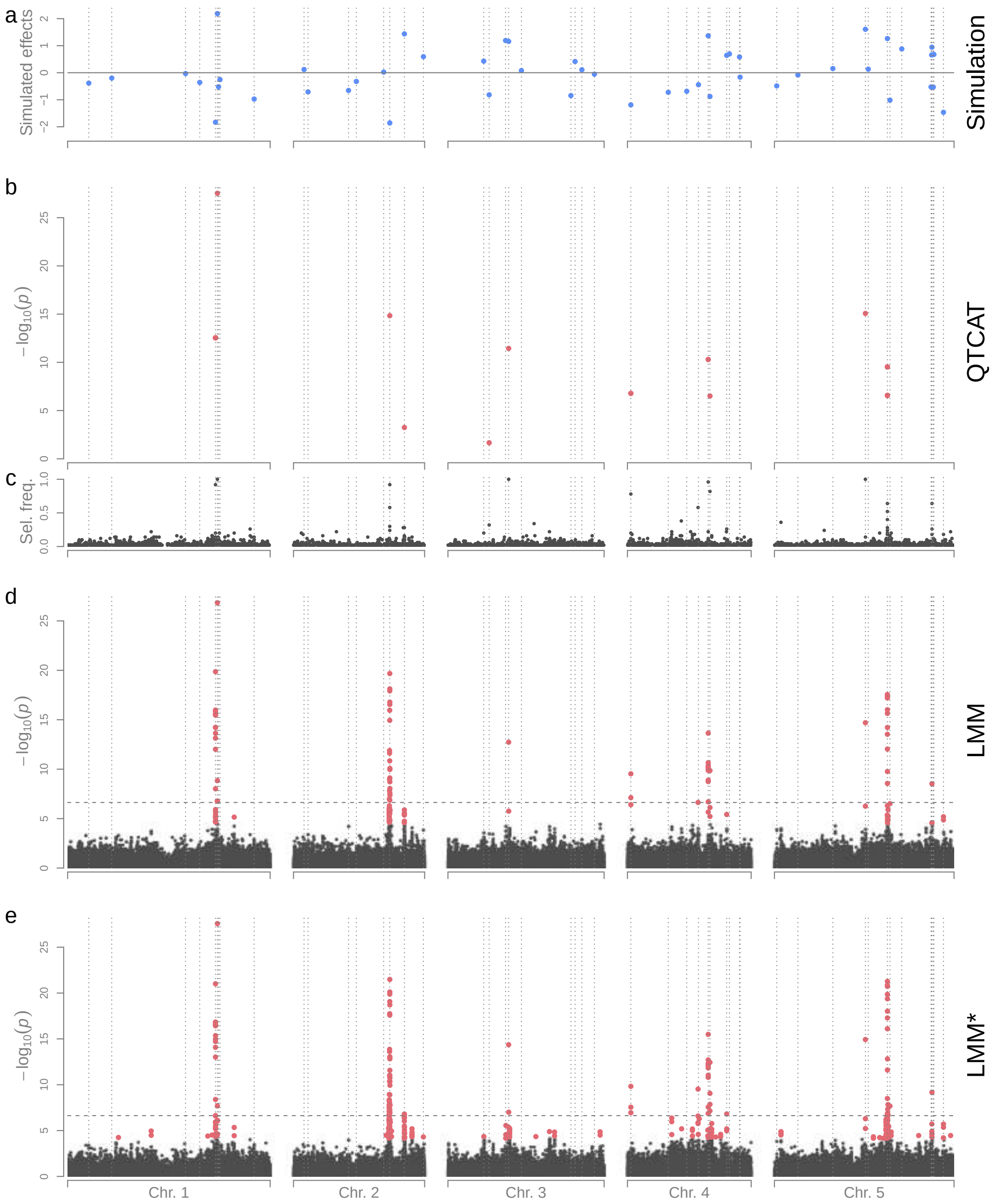

Supplementary Figure 178 Simulation of a GWA analysis based on a structured population with a heritability of 0.7 (run 78). (**a**) Simulation of 50 effects randomly drawn from a normal distribution and assigned to random markers. Markers with effect are highlighted with dashed lines. (**b**) Significant QTCs found by QTCAT. (**c**) LASSO selection frequency for each marker during the 50 iterations of QTCAT. (**d**) Manhattan plot of the LMM analysis. The horizontal dashed line depicts the significance threshold when controlling the multiple testing with FWER, whereas the red markers are significantly associated when controlling with FDR. (**e**) The Manhattan plot of the LMM* analysis. GRM was estimated without markers on the chromosome of the actual testing position. The results are shown as in (d).

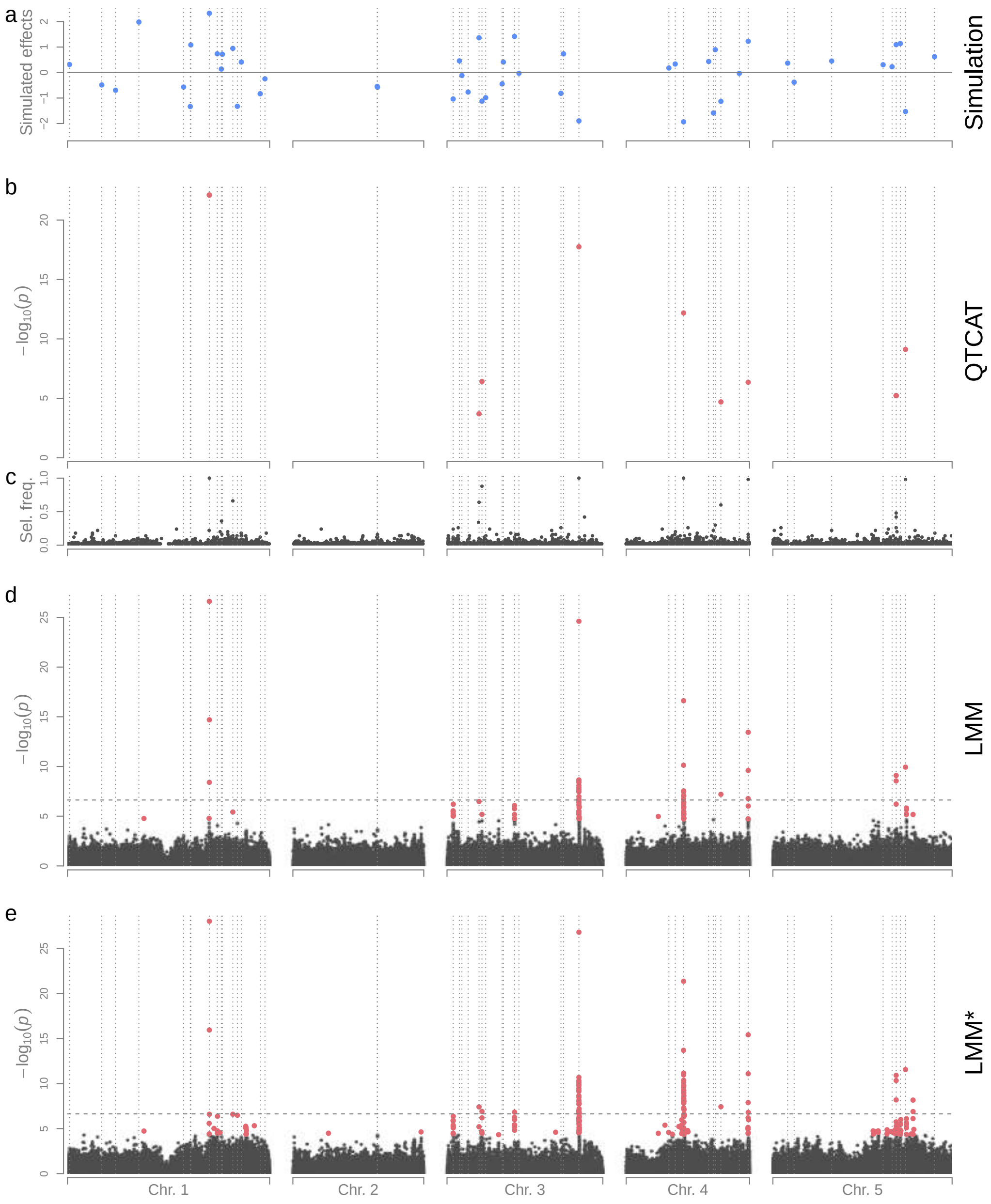

Supplementary Figure 179 Simulation of a GWA analysis based on a structured population with a heritability of 0.7 (run 79). (**a**) Simulation of 50 effects randomly drawn from a normal distribution and assigned to random markers. Markers with effect are highlighted with dashed lines. (**b**) Significant QTCs found by QTCAT. (**c**) LASSO selection frequency for each marker during the 50 iterations of QTCAT. (**d**) Manhattan plot of the LMM analysis. The horizontal dashed line depicts the significance threshold when controlling the multiple testing with FWER, whereas the red markers are significantly associated when controlling with FDR. (**e**) The Manhattan plot of the LMM* analysis. GRM was estimated without markers on the chromosome of the actual testing position. The results are shown as in (d).

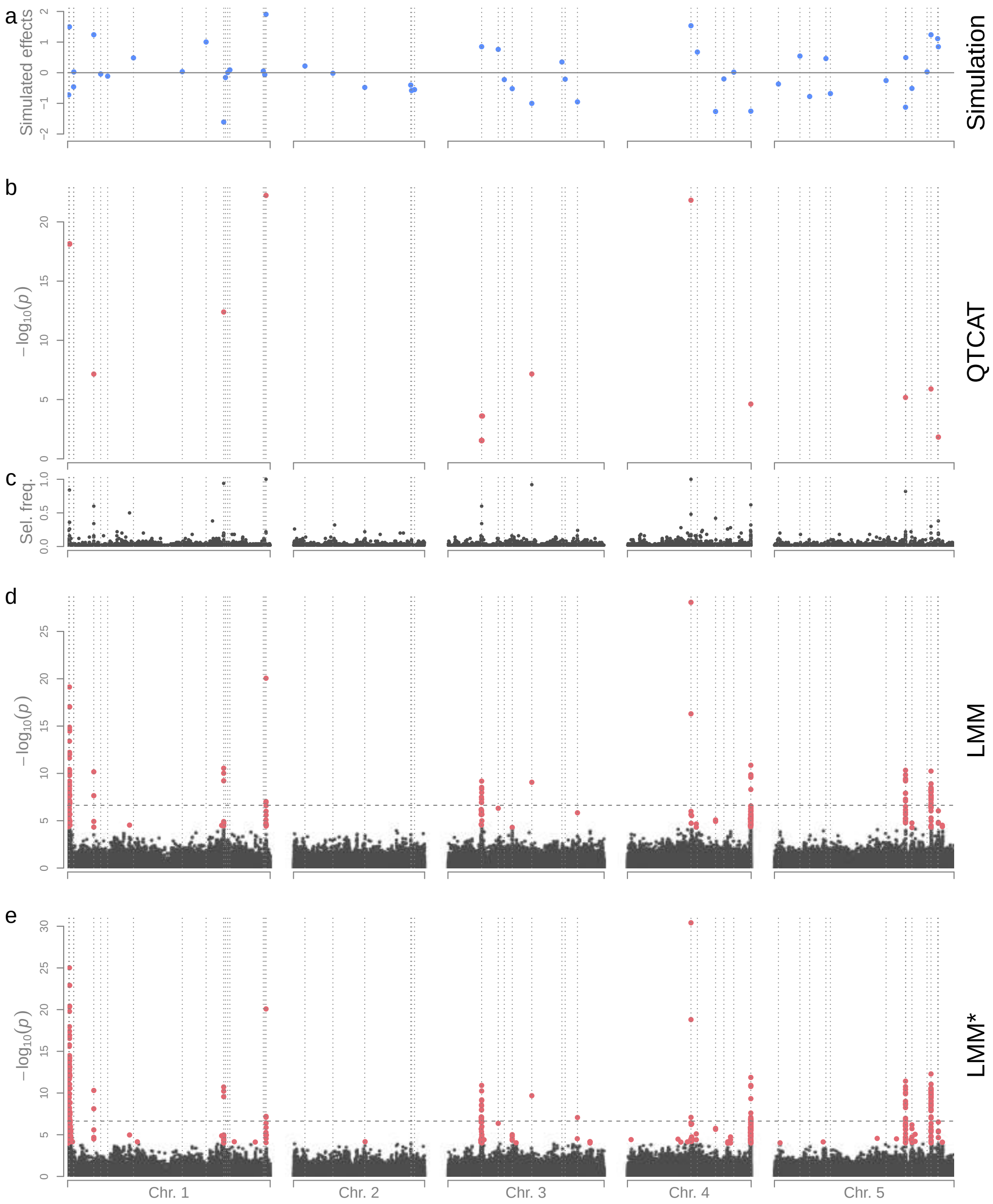

Supplementary Figure 180 Simulation of a GWA analysis based on a structured population with a heritability of 0.7 (run 80). (**a**) Simulation of 50 effects randomly drawn from a normal distribution and assigned to random markers. Markers with effect are highlighted with dashed lines. (**b**) Significant QTCs found by QTCAT. (**c**) LASSO selection frequency for each marker during the 50 iterations of QTCAT. (**d**) Manhattan plot of the LMM analysis. The horizontal dashed line depicts the significance threshold when controlling the multiple testing with FWER, whereas the red markers are significantly associated when controlling with FDR. (**e**) The Manhattan plot of the LMM* analysis. GRM was estimated without markers on the chromosome of the actual testing position. The results are shown as in (d).

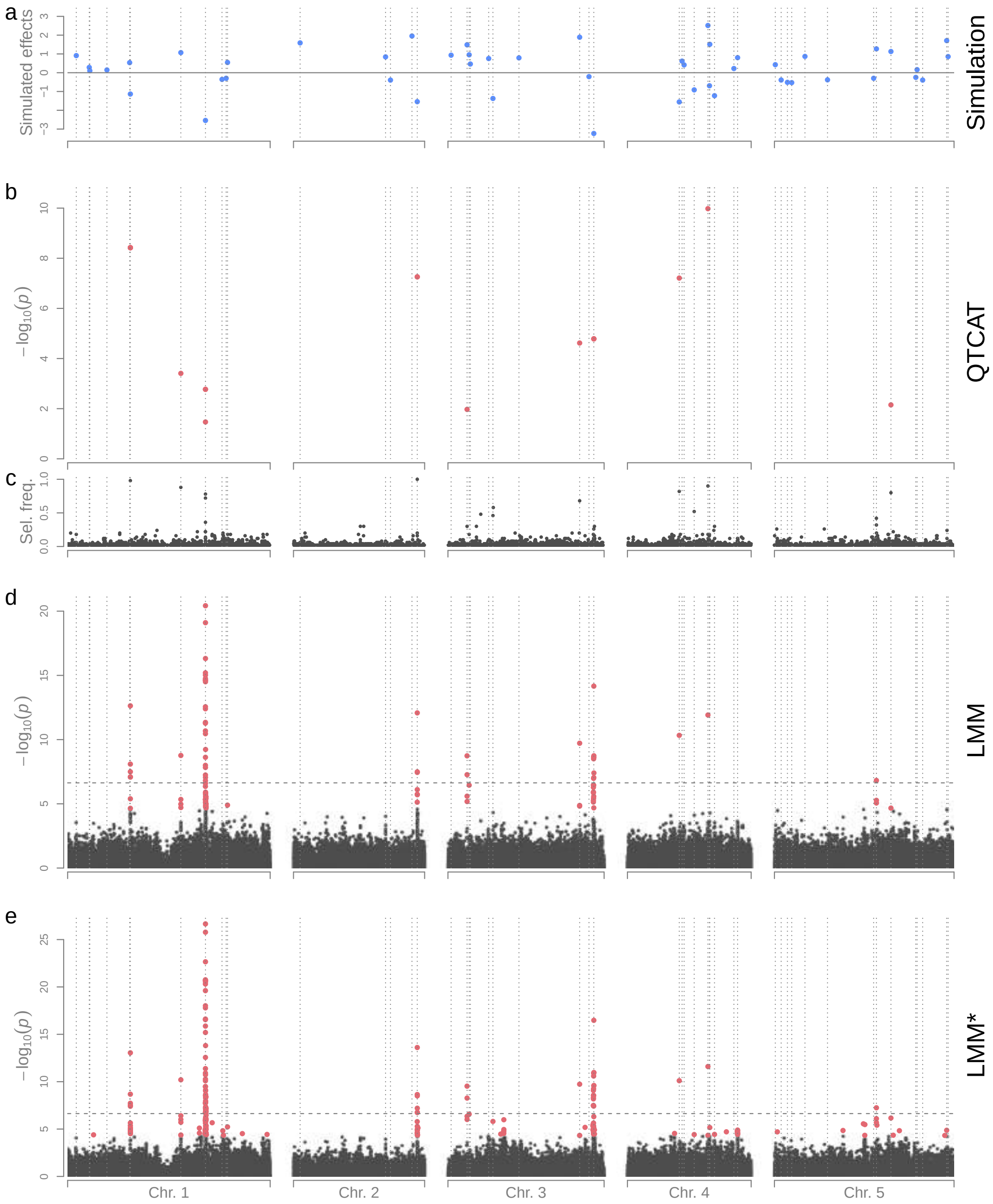

Supplementary Figure 181 Simulation of a GWA analysis based on a structured population with a heritability of 0.7 (run 81). (**a**) Simulation of 50 effects randomly drawn from a normal distribution and assigned to random markers. Markers with effect are highlighted with dashed lines. (**b**) Significant QTCs found by QTCAT. (**c**) LASSO selection frequency for each marker during the 50 iterations of QTCAT. (**d**) Manhattan plot of the LMM analysis. The horizontal dashed line depicts the significance threshold when controlling the multiple testing with FWER, whereas the red markers are significantly associated when controlling with FDR. (**e**) The Manhattan plot of the LMM* analysis. GRM was estimated without markers on the chromosome of the actual testing position. The results are shown as in (d).

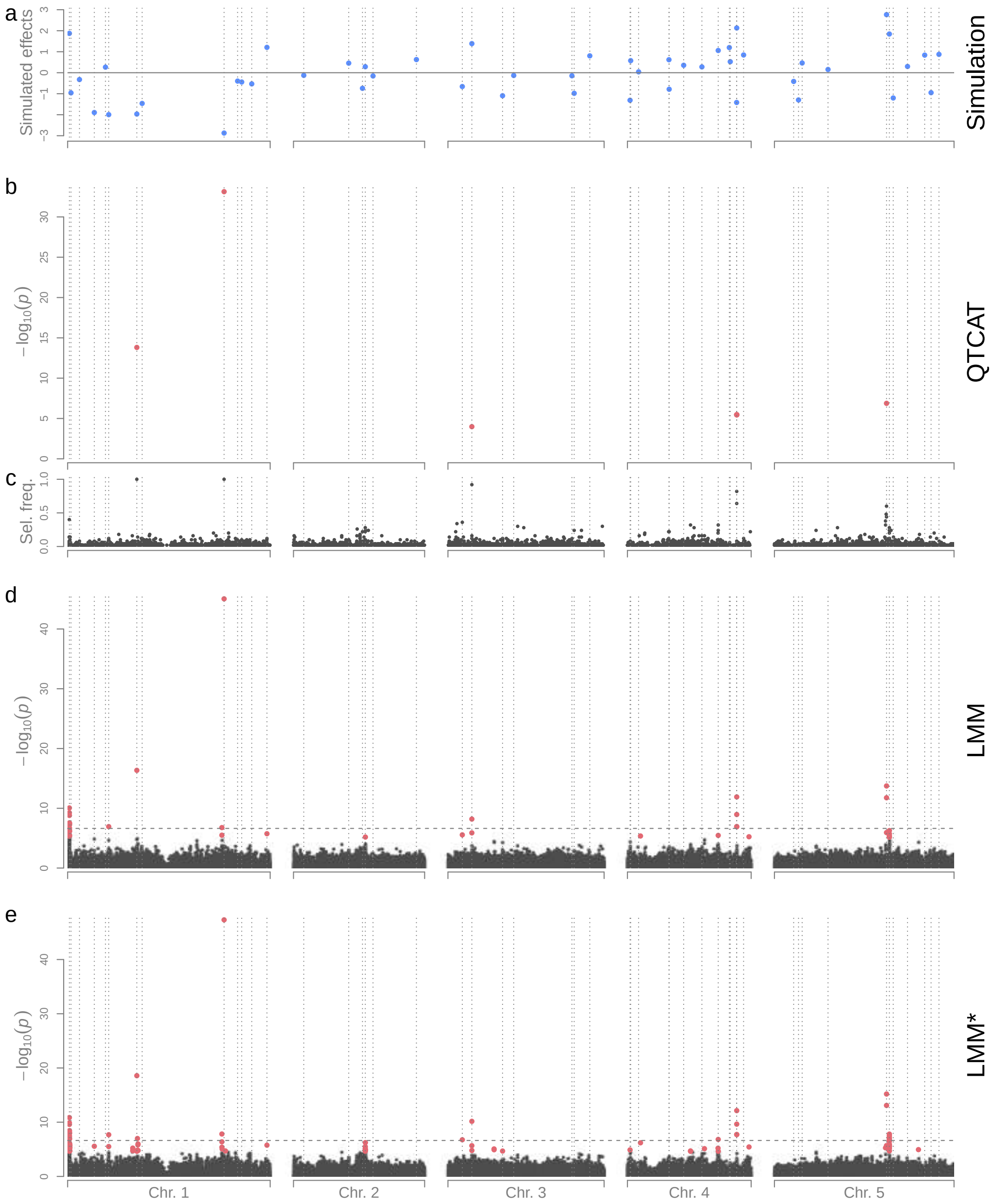

Supplementary Figure 182 Simulation of a GWA analysis based on a structured population with a heritability of 0.7 (run 82). (**a**) Simulation of 50 effects randomly drawn from a normal distribution and assigned to random markers. Markers with effect are highlighted with dashed lines. (**b**) Significant QTCs found by QTCAT. (**c**) LASSO selection frequency for each marker during the 50 iterations of QTCAT. (**d**) Manhattan plot of the LMM analysis. The horizontal dashed line depicts the significance threshold when controlling the multiple testing with FWER, whereas the red markers are significantly associated when controlling with FDR. (**e**) The Manhattan plot of the LMM* analysis. GRM was estimated without markers on the chromosome of the actual testing position. The results are shown as in (d).

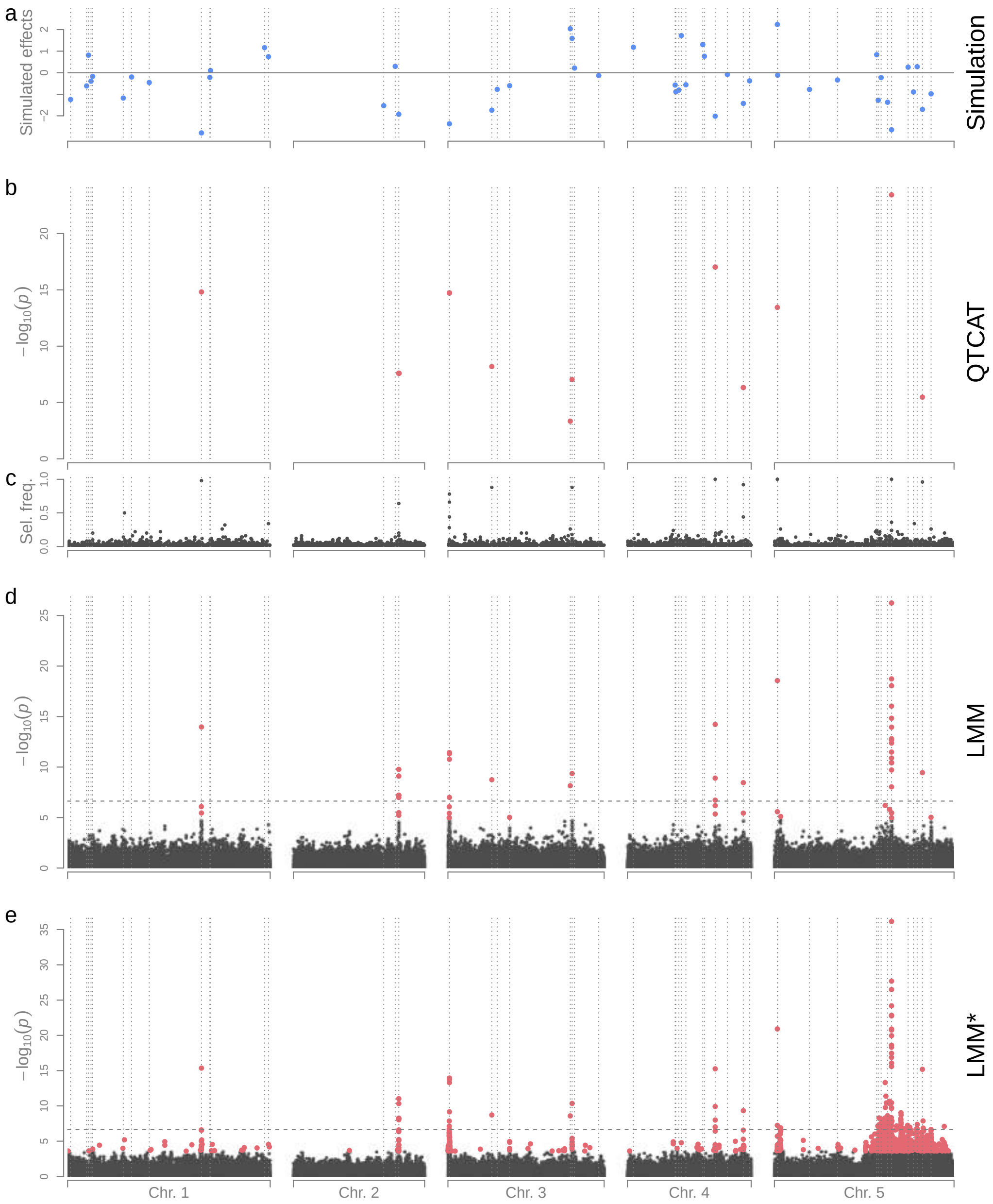

Supplementary Figure 183 Simulation of a GWA analysis based on a structured population with a heritability of 0.7 (run 83). (**a**) Simulation of 50 effects randomly drawn from a normal distribution and assigned to random markers. Markers with effect are highlighted with dashed lines. (**b**) Significant QTCs found by QTCAT. (**c**) LASSO selection frequency for each marker during the 50 iterations of QTCAT. (**d**) Manhattan plot of the LMM analysis. The horizontal dashed line depicts the significance threshold when controlling the multiple testing with FWER, whereas the red markers are significantly associated when controlling with FDR. (**e**) The Manhattan plot of the LMM* analysis. GRM was estimated without markers on the chromosome of the actual testing position. The results are shown as in (d).

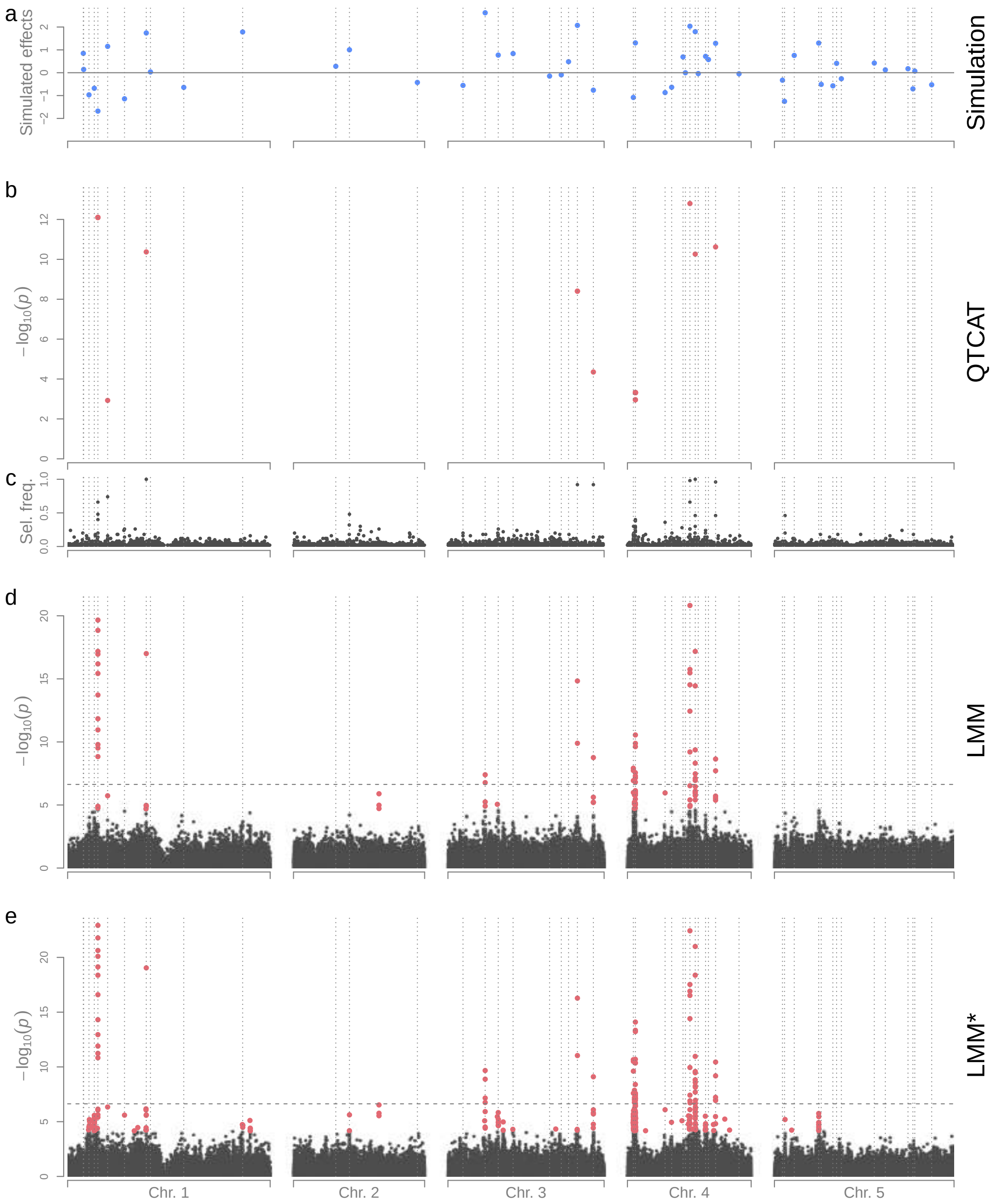

Supplementary Figure 184 Simulation of a GWA analysis based on a structured population with a heritability of 0.7 (run 84). (**a**) Simulation of 50 effects randomly drawn from a normal distribution and assigned to random markers. Markers with effect are highlighted with dashed lines. (**b**) Significant QTCs found by QTCAT. (**c**) LASSO selection frequency for each marker during the 50 iterations of QTCAT. (**d**) Manhattan plot of the LMM analysis. The horizontal dashed line depicts the significance threshold when controlling the multiple testing with FWER, whereas the red markers are significantly associated when controlling with FDR. (**e**) The Manhattan plot of the LMM* analysis. GRM was estimated without markers on the chromosome of the actual testing position. The results are shown as in (d).

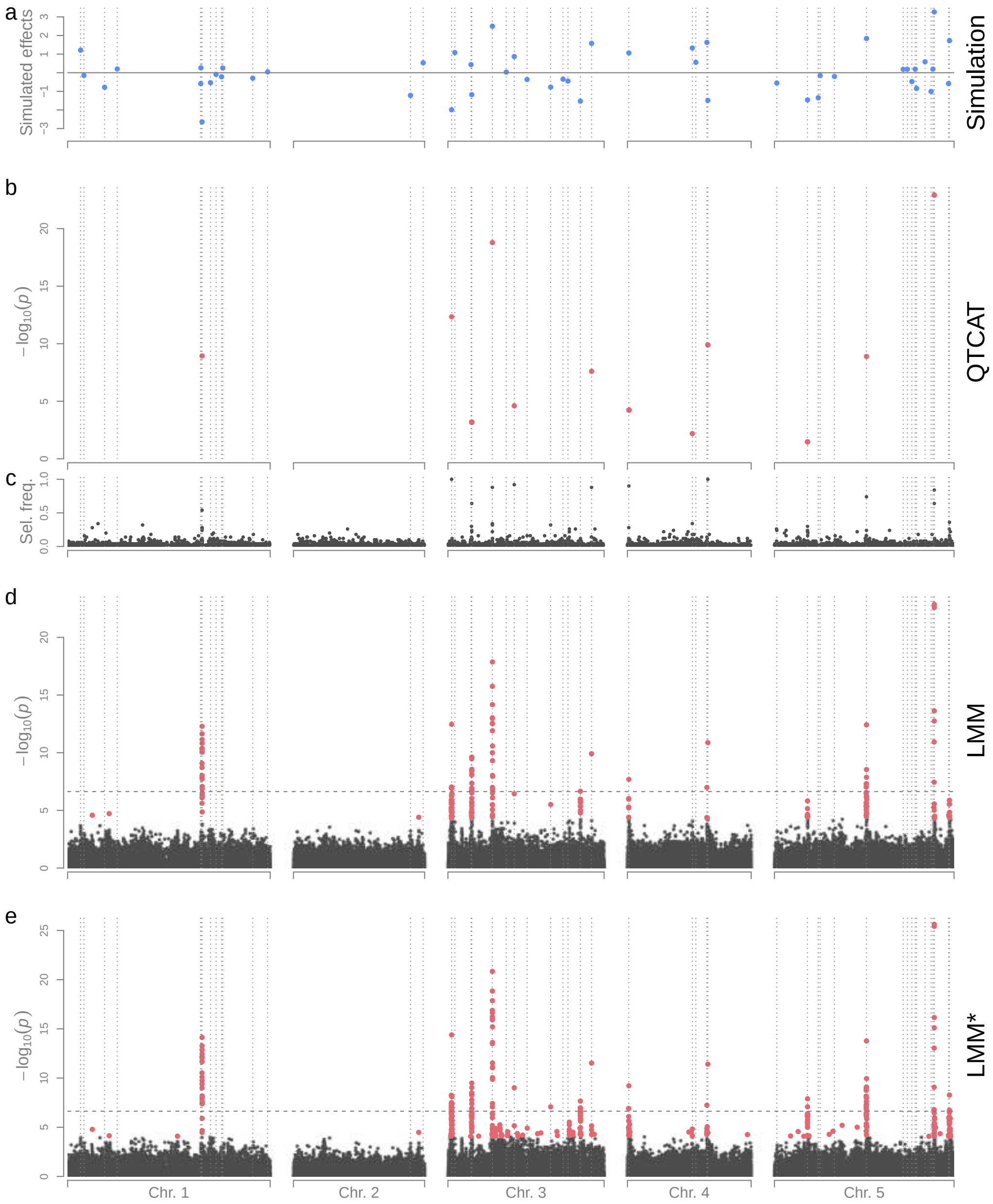

Supplementary Figure 185 Simulation of a GWA analysis based on a structured population with a heritability of 0.7 (run 85). (**a**) Simulation of 50 effects randomly drawn from a normal distribution and assigned to random markers. Markers with effect are highlighted with dashed lines. (**b**) Significant QTCs found by QTCAT. (**c**) LASSO selection frequency for each marker during the 50 iterations of QTCAT. (**d**) Manhattan plot of the LMM analysis. The horizontal dashed line depicts the significance threshold when controlling the multiple testing with FWER, whereas the red markers are significantly associated when controlling with FDR. (**e**) The Manhattan plot of the LMM* analysis. GRM was estimated without markers on the chromosome of the actual testing position. The results are shown as in (d).

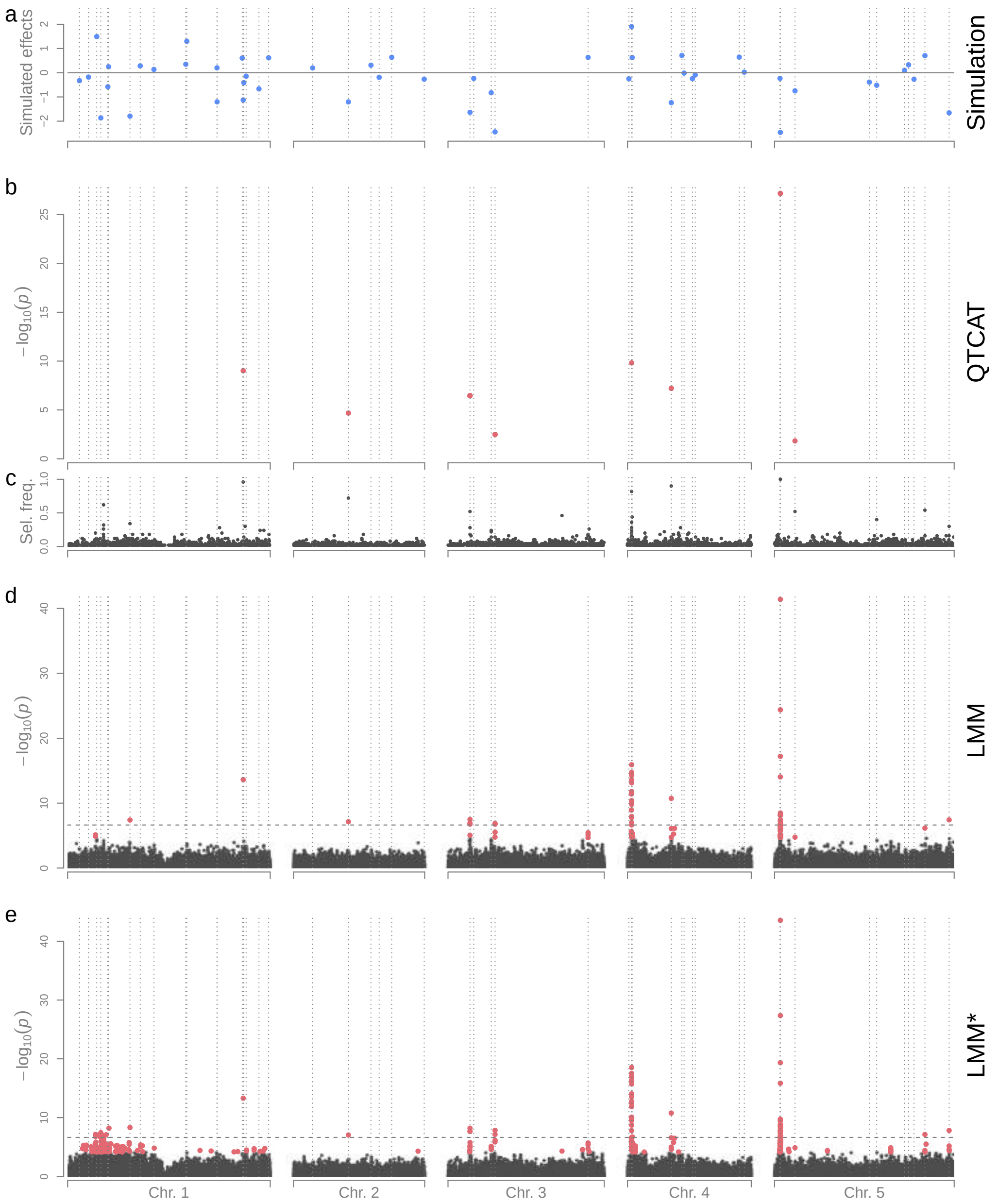

Supplementary Figure 186 Simulation of a GWA analysis based on a structured population with a heritability of 0.7 (run 86). (**a**) Simulation of 50 effects randomly drawn from a normal distribution and assigned to random markers. Markers with effect are highlighted with dashed lines. (**b**) Significant QTCs found by QTCAT. (**c**) LASSO selection frequency for each marker during the 50 iterations of QTCAT. (**d**) Manhattan plot of the LMM analysis. The horizontal dashed line depicts the significance threshold when controlling the multiple testing with FWER, whereas the red markers are significantly associated when controlling with FDR. (**e**) The Manhattan plot of the LMM* analysis. GRM was estimated without markers on the chromosome of the actual testing position. The results are shown as in (d).

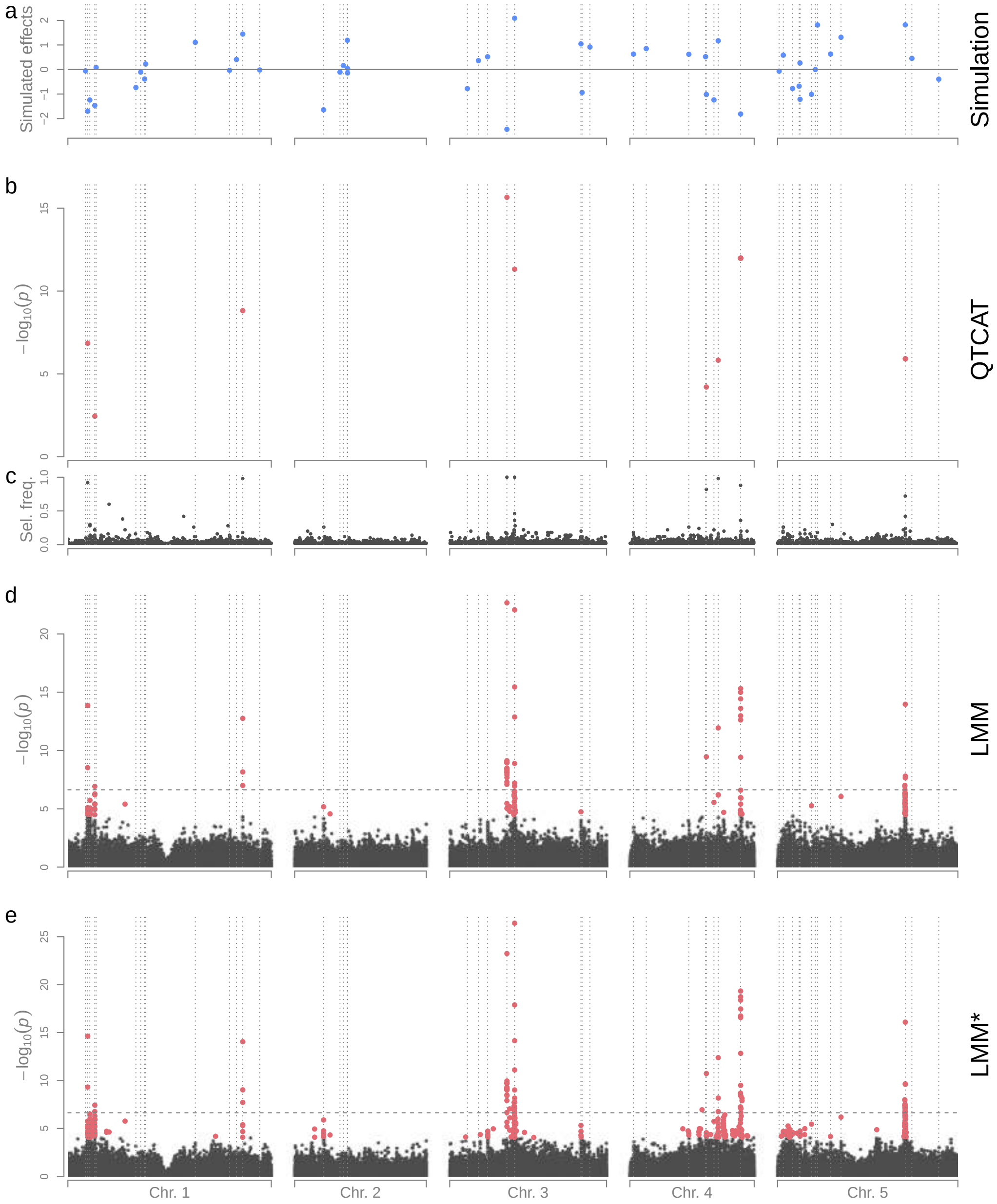

Supplementary Figure 187 Simulation of a GWA analysis based on a structured population with a heritability of 0.7 (run 87). (**a**) Simulation of 50 effects randomly drawn from a normal distribution and assigned to random markers. Markers with effect are highlighted with dashed lines. (**b**) Significant QTCs found by QTCAT. (**c**) LASSO selection frequency for each marker during the 50 iterations of QTCAT. (**d**) Manhattan plot of the LMM analysis. The horizontal dashed line depicts the significance threshold when controlling the multiple testing with FWER, whereas the red markers are significantly associated when controlling with FDR. (**e**) The Manhattan plot of the LMM* analysis. GRM was estimated without markers on the chromosome of the actual testing position. The results are shown as in (d).

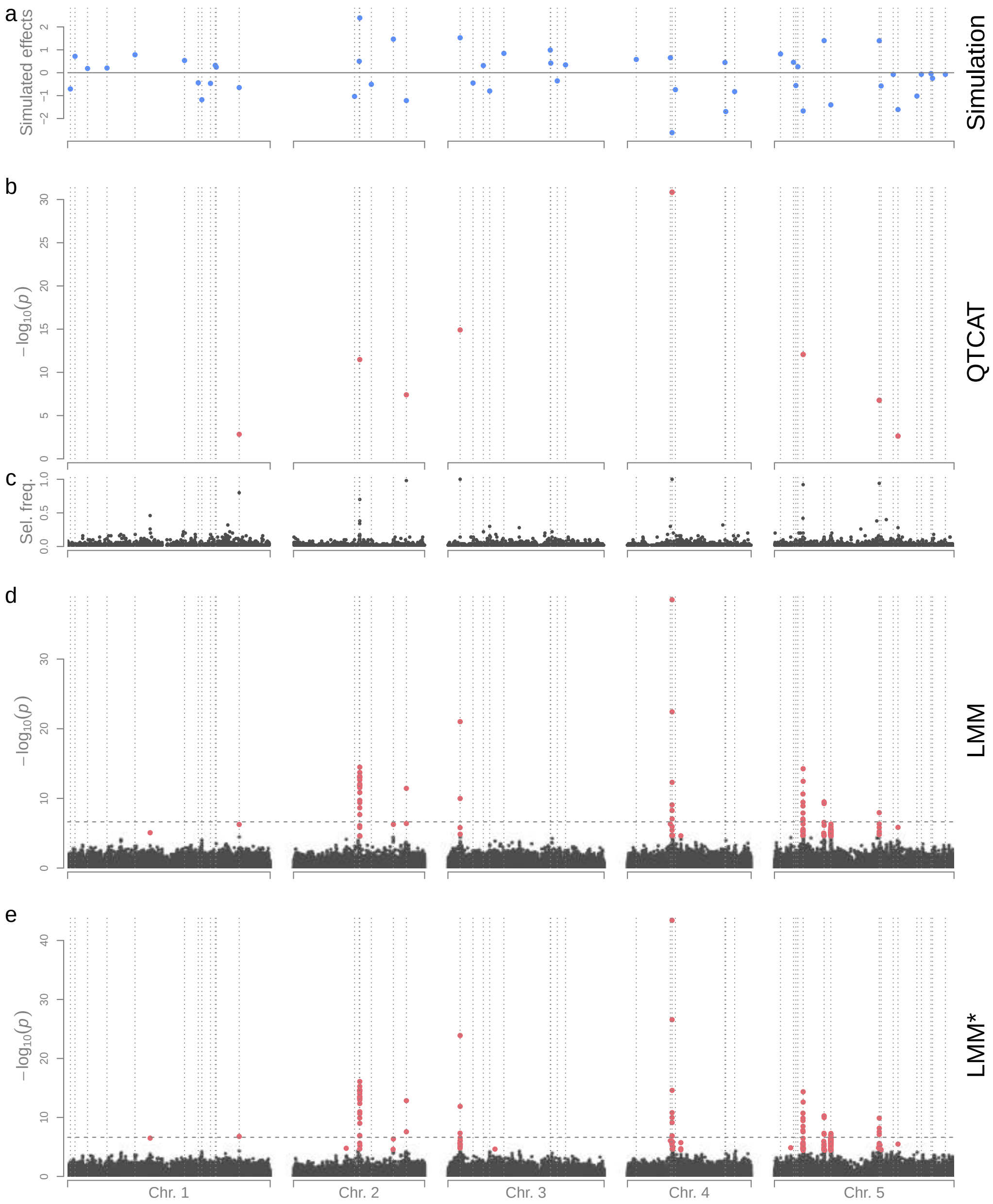

Supplementary Figure 188 Simulation of a GWA analysis based on a structured population with a heritability of 0.7 (run 88). (**a**) Simulation of 50 effects randomly drawn from a normal distribution and assigned to random markers. Markers with effect are highlighted with dashed lines. (**b**) Significant QTCs found by QTCAT. (**c**) LASSO selection frequency for each marker during the 50 iterations of QTCAT. (**d**) Manhattan plot of the LMM analysis. The horizontal dashed line depicts the significance threshold when controlling the multiple testing with FWER, whereas the red markers are significantly associated when controlling with FDR. (**e**) The Manhattan plot of the LMM* analysis. GRM was estimated without markers on the chromosome of the actual testing position. The results are shown as in (d).

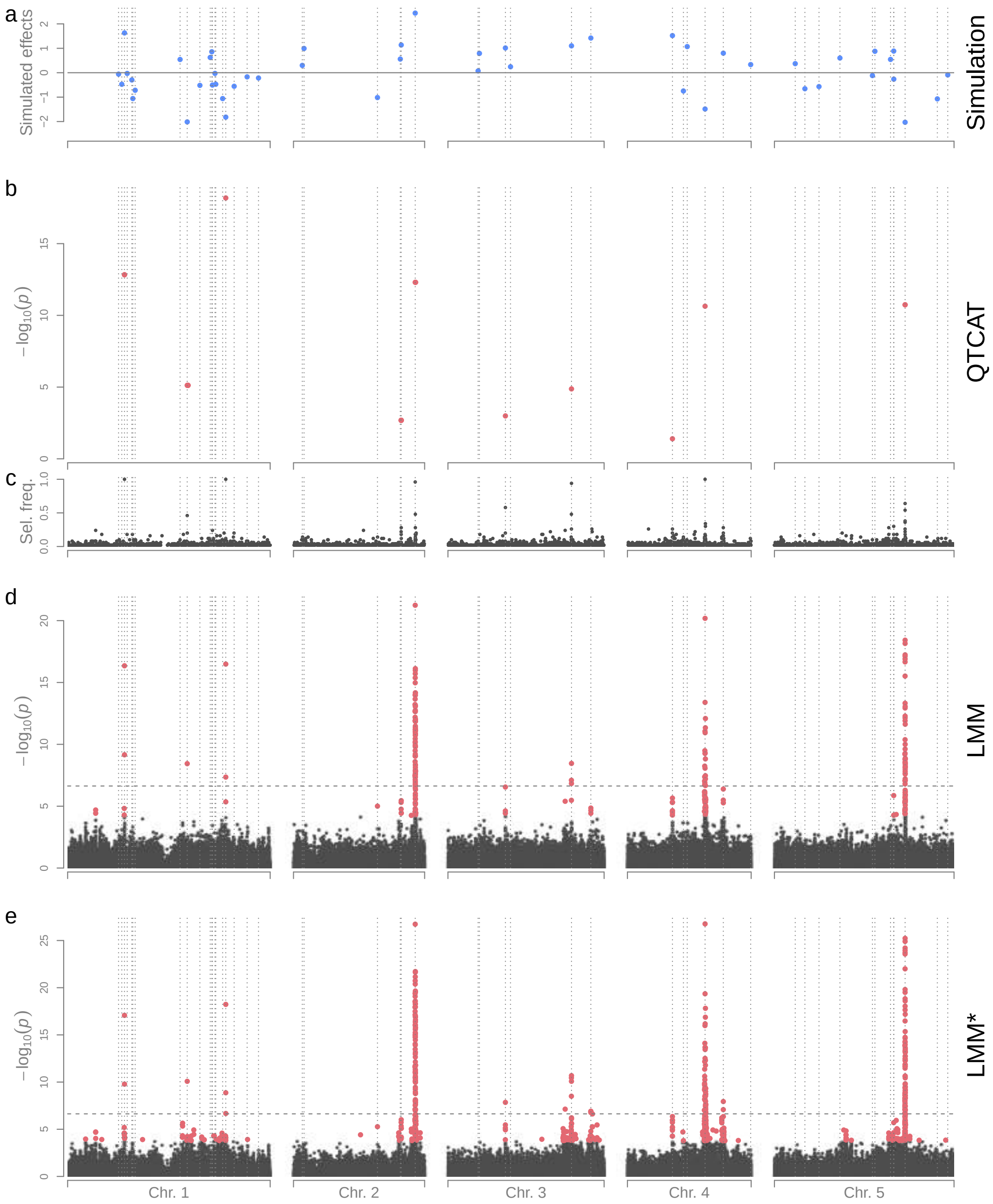

Supplementary Figure 189 Simulation of a GWA analysis based on a structured population with a heritability of 0.7 (run 89). (**a**) Simulation of 50 effects randomly drawn from a normal distribution and assigned to random markers. Markers with effect are highlighted with dashed lines. (**b**) Significant QTCs found by QTCAT. (**c**) LASSO selection frequency for each marker during the 50 iterations of QTCAT. (**d**) Manhattan plot of the LMM analysis. The horizontal dashed line depicts the significance threshold when controlling the multiple testing with FWER, whereas the red markers are significantly associated when controlling with FDR. (**e**) The Manhattan plot of the LMM* analysis. GRM was estimated without markers on the chromosome of the actual testing position. The results are shown as in (d).

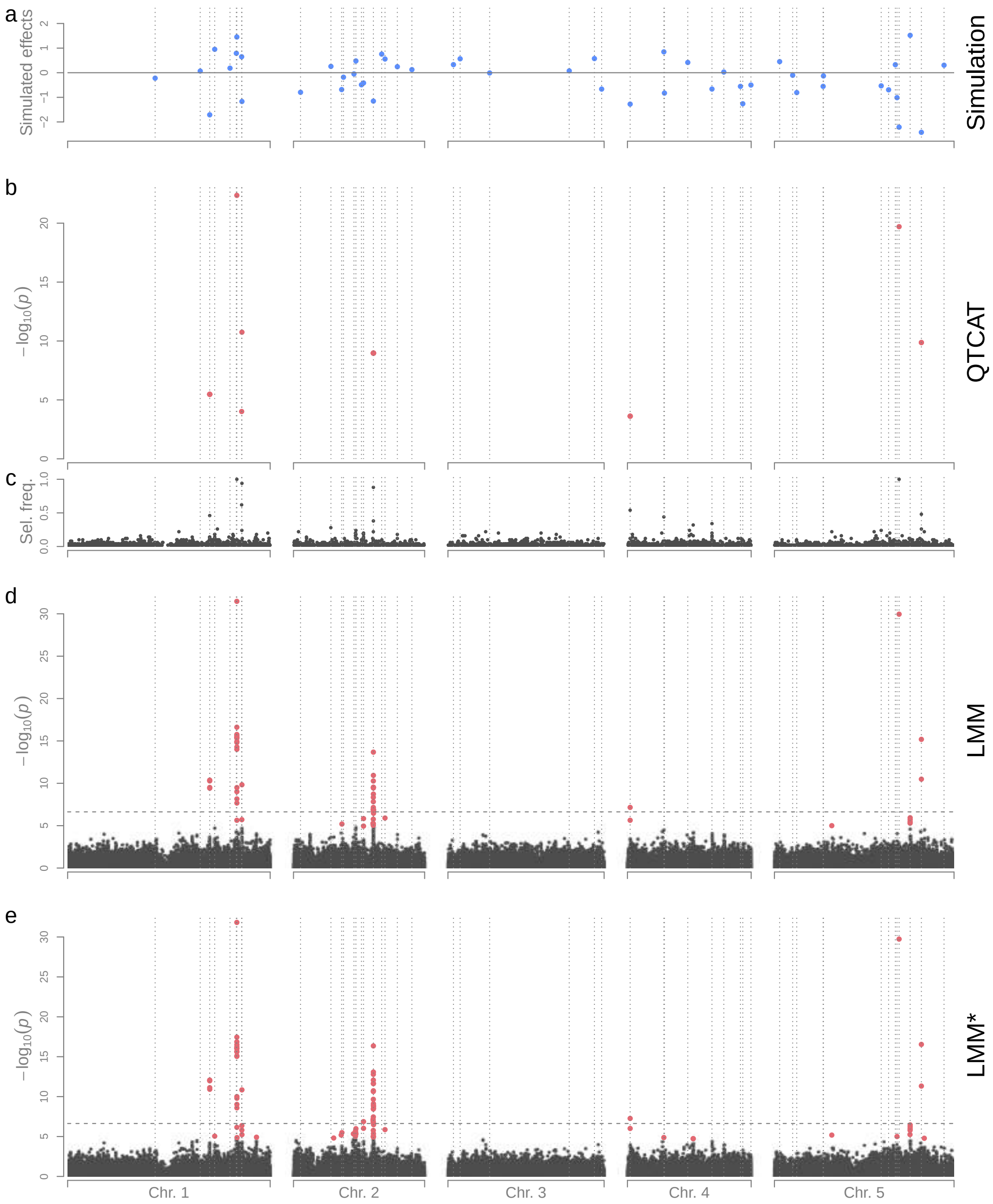

Supplementary Figure 190 Simulation of a GWA analysis based on a structured population with a heritability of 0.7 (run 90). (**a**) Simulation of 50 effects randomly drawn from a normal distribution and assigned to random markers. Markers with effect are highlighted with dashed lines. (**b**) Significant QTCs found by QTCAT. (**c**) LASSO selection frequency for each marker during the 50 iterations of QTCAT. (**d**) Manhattan plot of the LMM analysis. The horizontal dashed line depicts the significance threshold when controlling the multiple testing with FWER, whereas the red markers are significantly associated when controlling with FDR. (**e**) The Manhattan plot of the LMM* analysis. GRM was estimated without markers on the chromosome of the actual testing position. The results are shown as in (d).

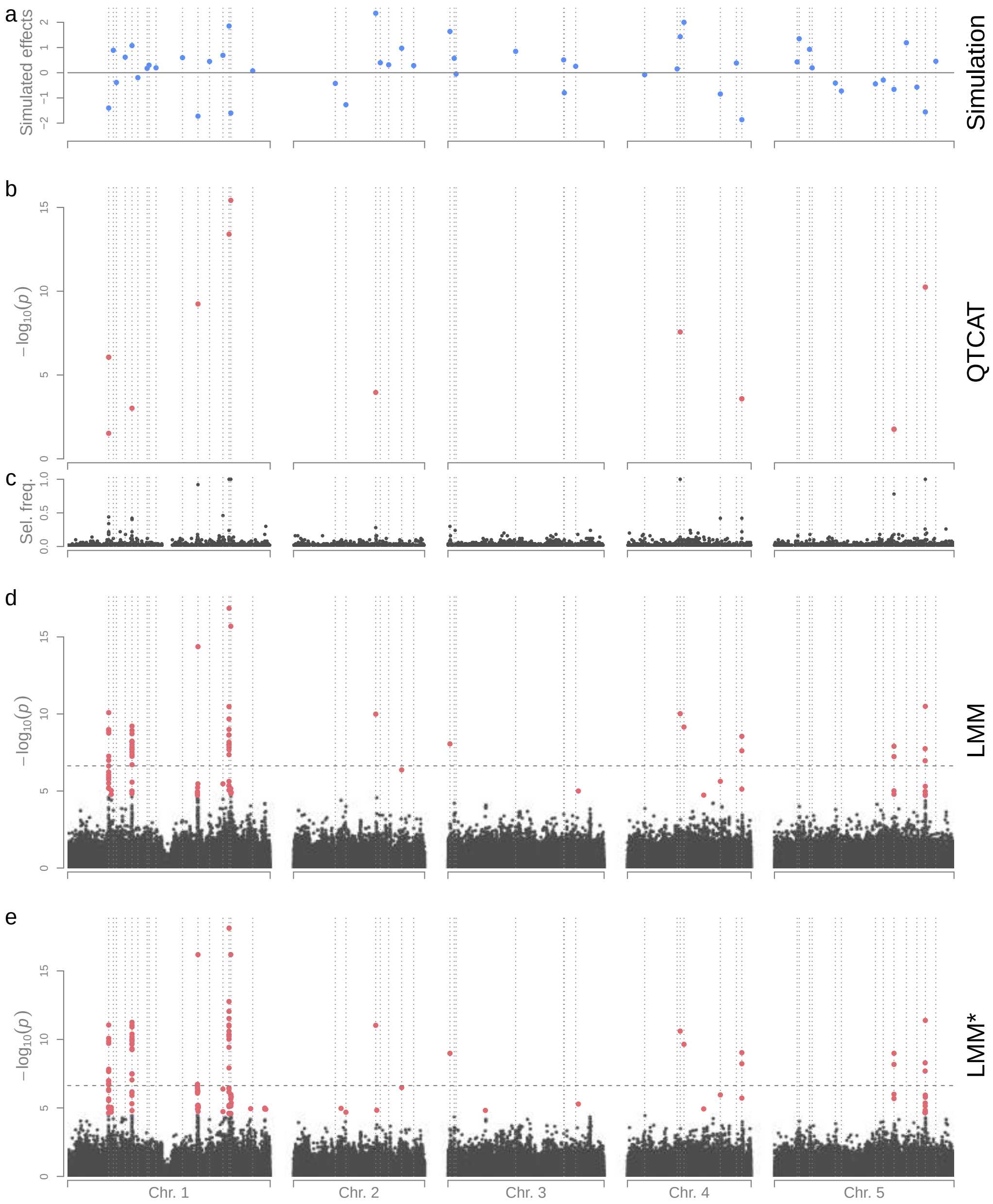

Supplementary Figure 191 Simulation of a GWA analysis based on a structured population with a heritability of 0.7 (run 91). (**a**) Simulation of 50 effects randomly drawn from a normal distribution and assigned to random markers. Markers with effect are highlighted with dashed lines. (**b**) Significant QTCs found by QTCAT. (**c**) LASSO selection frequency for each marker during the 50 iterations of QTCAT. (**d**) Manhattan plot of the LMM analysis. The horizontal dashed line depicts the significance threshold when controlling the multiple testing with FWER, whereas the red markers are significantly associated when controlling with FDR. (**e**) The Manhattan plot of the LMM* analysis. GRM was estimated without markers on the chromosome of the actual testing position. The results are shown as in (d).

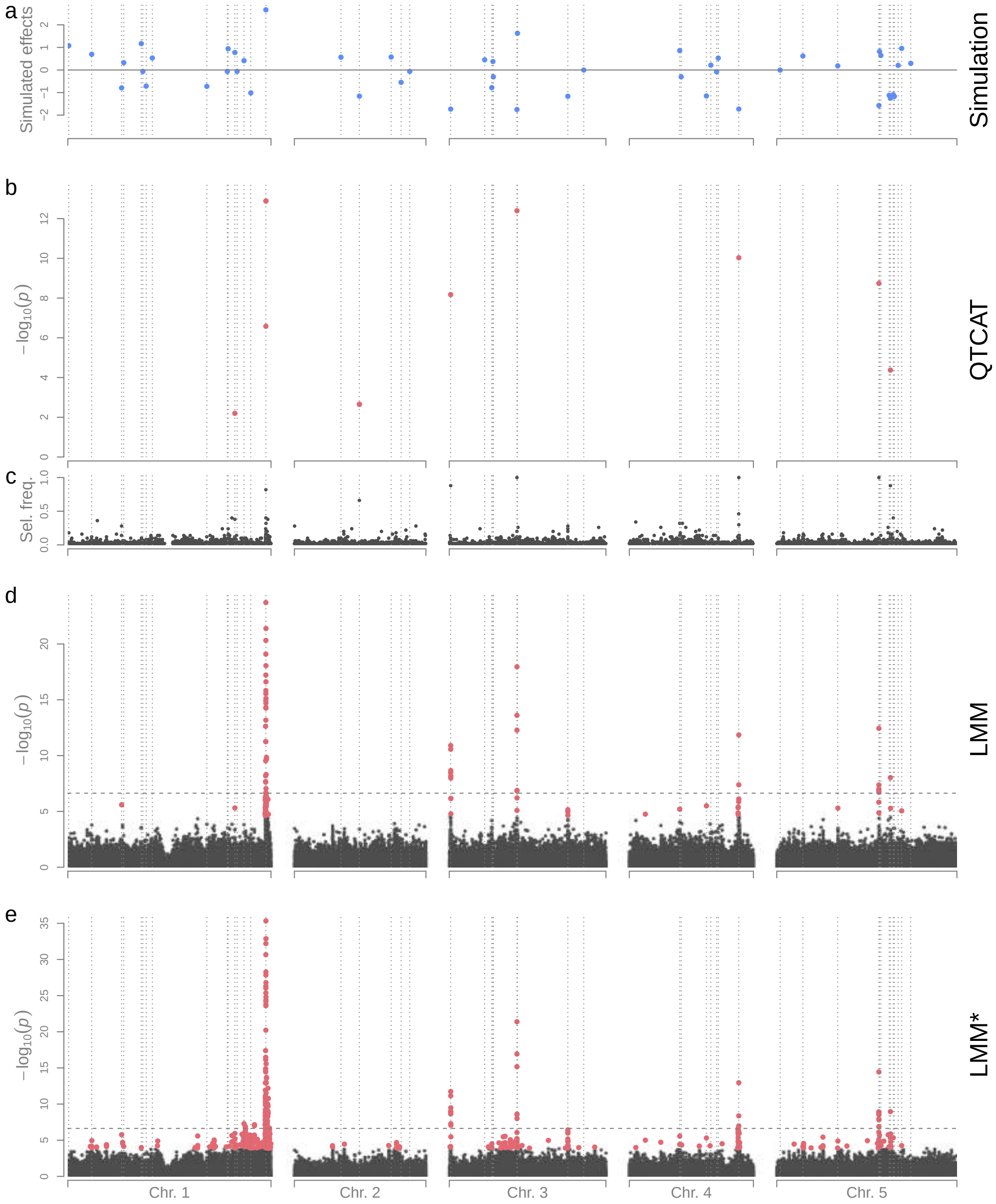

Supplementary Figure 192 Simulation of a GWA analysis based on a structured population with a heritability of 0.7 (run 92). (**a**) Simulation of 50 effects randomly drawn from a normal distribution and assigned to random markers. Markers with effect are highlighted with dashed lines. (**b**) Significant QTCs found by QTCAT. (**c**) LASSO selection frequency for each marker during the 50 iterations of QTCAT. (**d**) Manhattan plot of the LMM analysis. The horizontal dashed line depicts the significance threshold when controlling the multiple testing with FWER, whereas the red markers are significantly associated when controlling with FDR. (**e**) The Manhattan plot of the LMM* analysis. GRM was estimated without markers on the chromosome of the actual testing position. The results are shown as in (d).

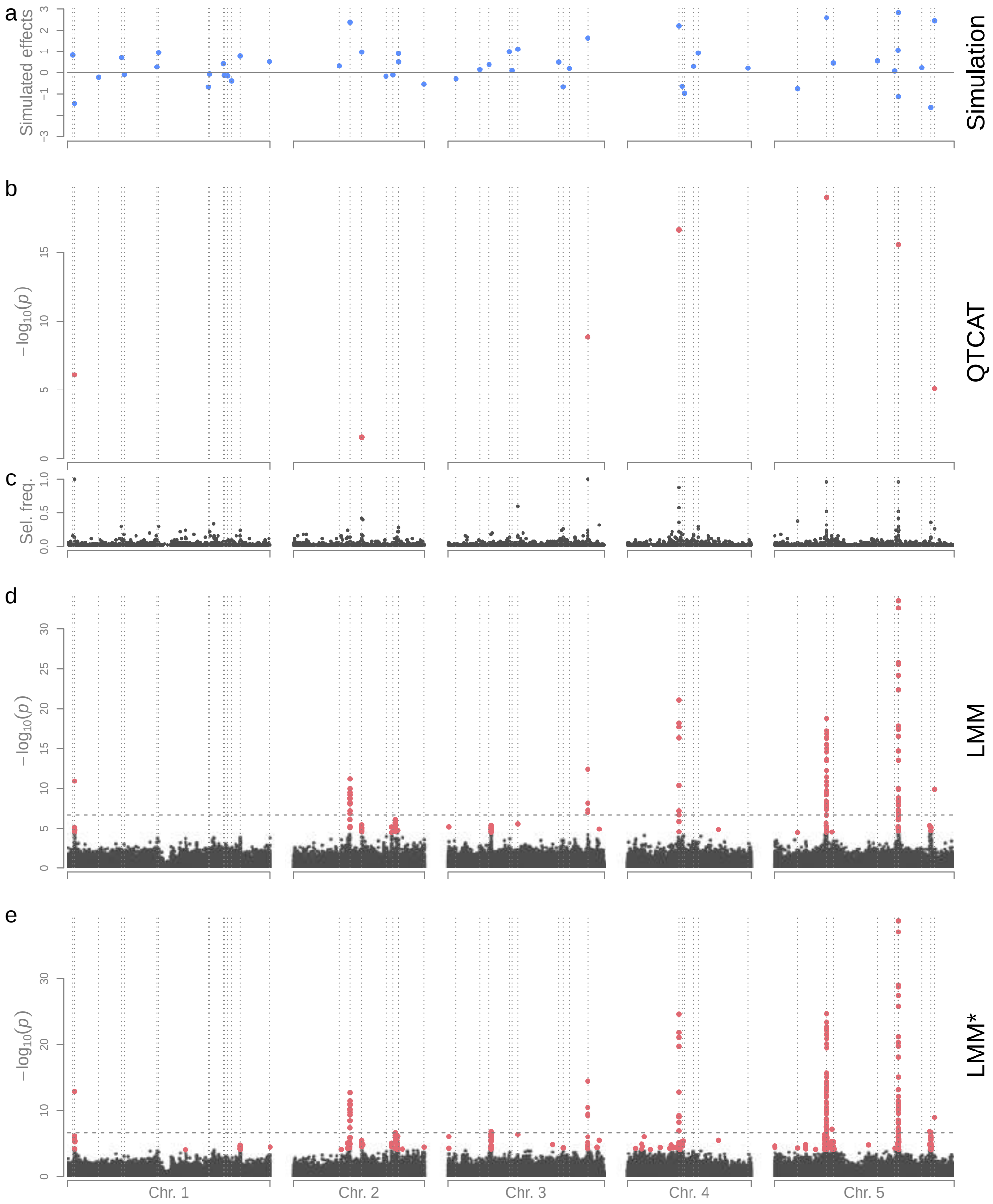

Supplementary Figure 193 Simulation of a GWA analysis based on a structured population with a heritability of 0.7 (run 93). (**a**) Simulation of 50 effects randomly drawn from a normal distribution and assigned to random markers. Markers with effect are highlighted with dashed lines. (**b**) Significant QTCs found by QTCAT. (**c**) LASSO selection frequency for each marker during the 50 iterations of QTCAT. (**d**) Manhattan plot of the LMM analysis. The horizontal dashed line depicts the significance threshold when controlling the multiple testing with FWER, whereas the red markers are significantly associated when controlling with FDR. (**e**) The Manhattan plot of the LMM* analysis. GRM was estimated without markers on the chromosome of the actual testing position. The results are shown as in (d).

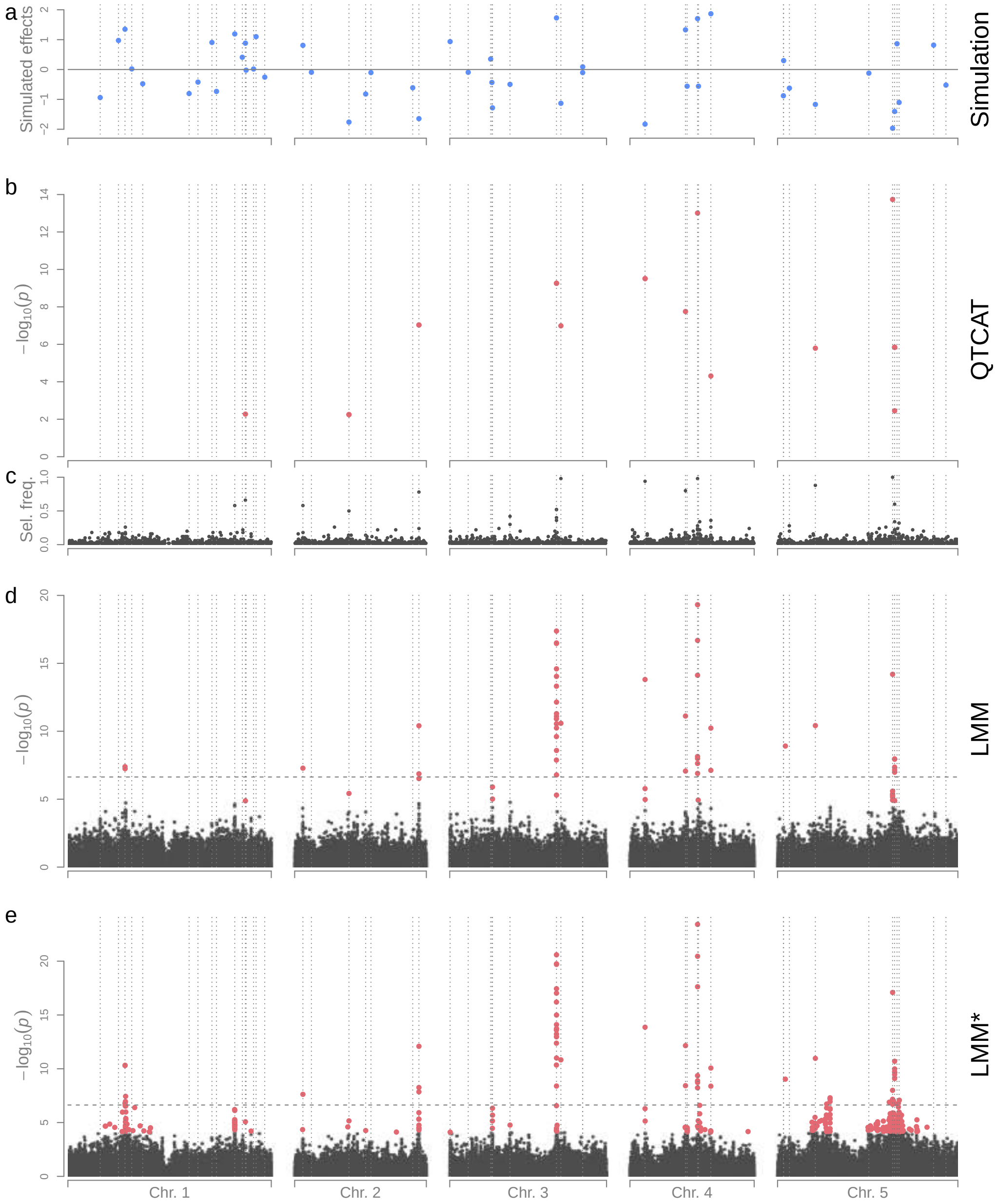

Supplementary Figure 194 Simulation of a GWA analysis based on a structured population with a heritability of 0.7 (run 94). (**a**) Simulation of 50 effects randomly drawn from a normal distribution and assigned to random markers. Markers with effect are highlighted with dashed lines. (**b**) Significant QTCs found by QTCAT. (**c**) LASSO selection frequency for each marker during the 50 iterations of QTCAT. (**d**) Manhattan plot of the LMM analysis. The horizontal dashed line depicts the significance threshold when controlling the multiple testing with FWER, whereas the red markers are significantly associated when controlling with FDR. (**e**) The Manhattan plot of the LMM* analysis. GRM was estimated without markers on the chromosome of the actual testing position. The results are shown as in (d).

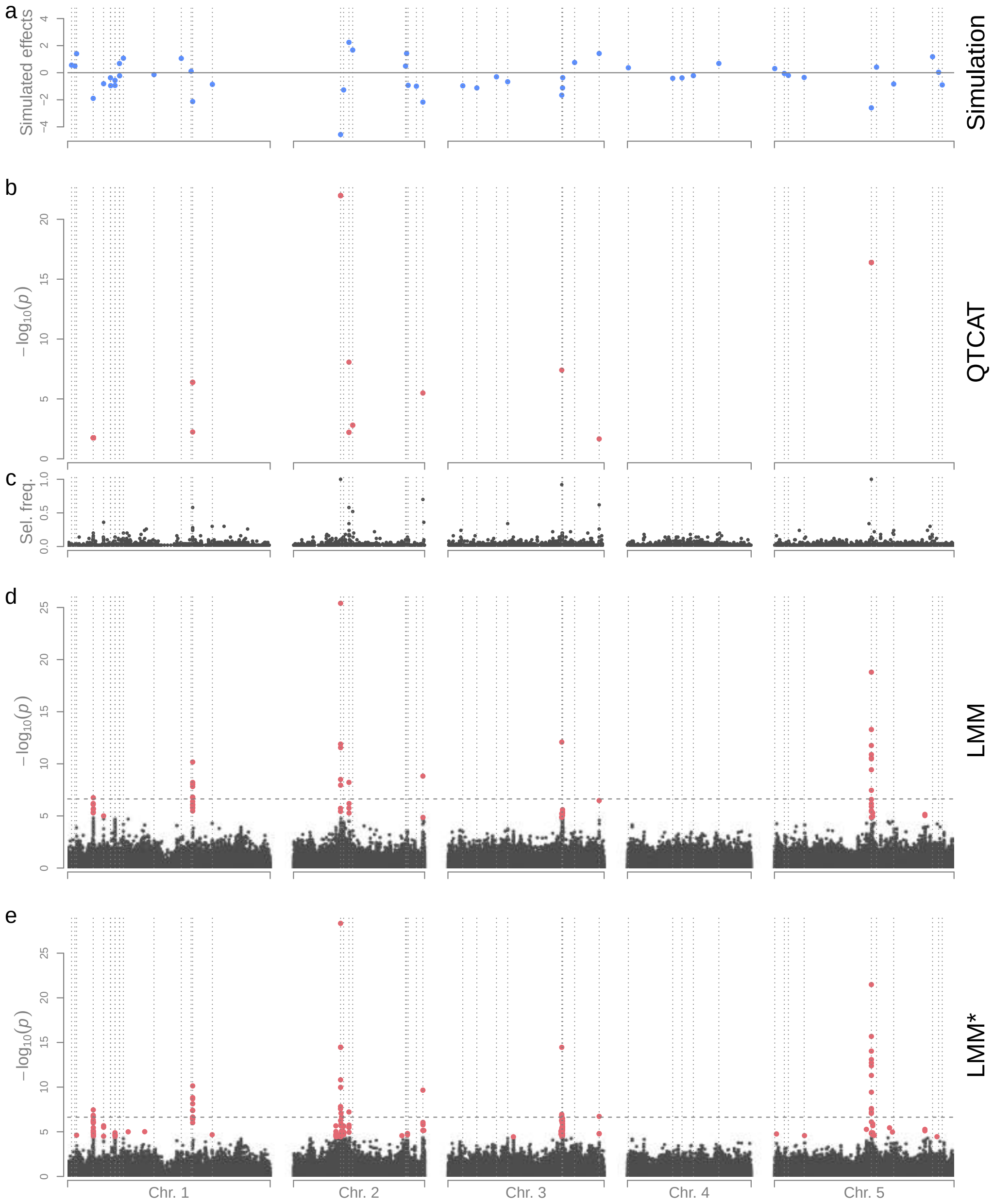

Supplementary Figure 195 Simulation of a GWA analysis based on a structured population with a heritability of 0.7 (run 95). (**a**) Simulation of 50 effects randomly drawn from a normal distribution and assigned to random markers. Markers with effect are highlighted with dashed lines. (**b**) Significant QTCs found by QTCAT. (**c**) LASSO selection frequency for each marker during the 50 iterations of QTCAT. (**d**) Manhattan plot of the LMM analysis. The horizontal dashed line depicts the significance threshold when controlling the multiple testing with FWER, whereas the red markers are significantly associated when controlling with FDR. (**e**) The Manhattan plot of the LMM* analysis. GRM was estimated without markers on the chromosome of the actual testing position. The results are shown as in (d).

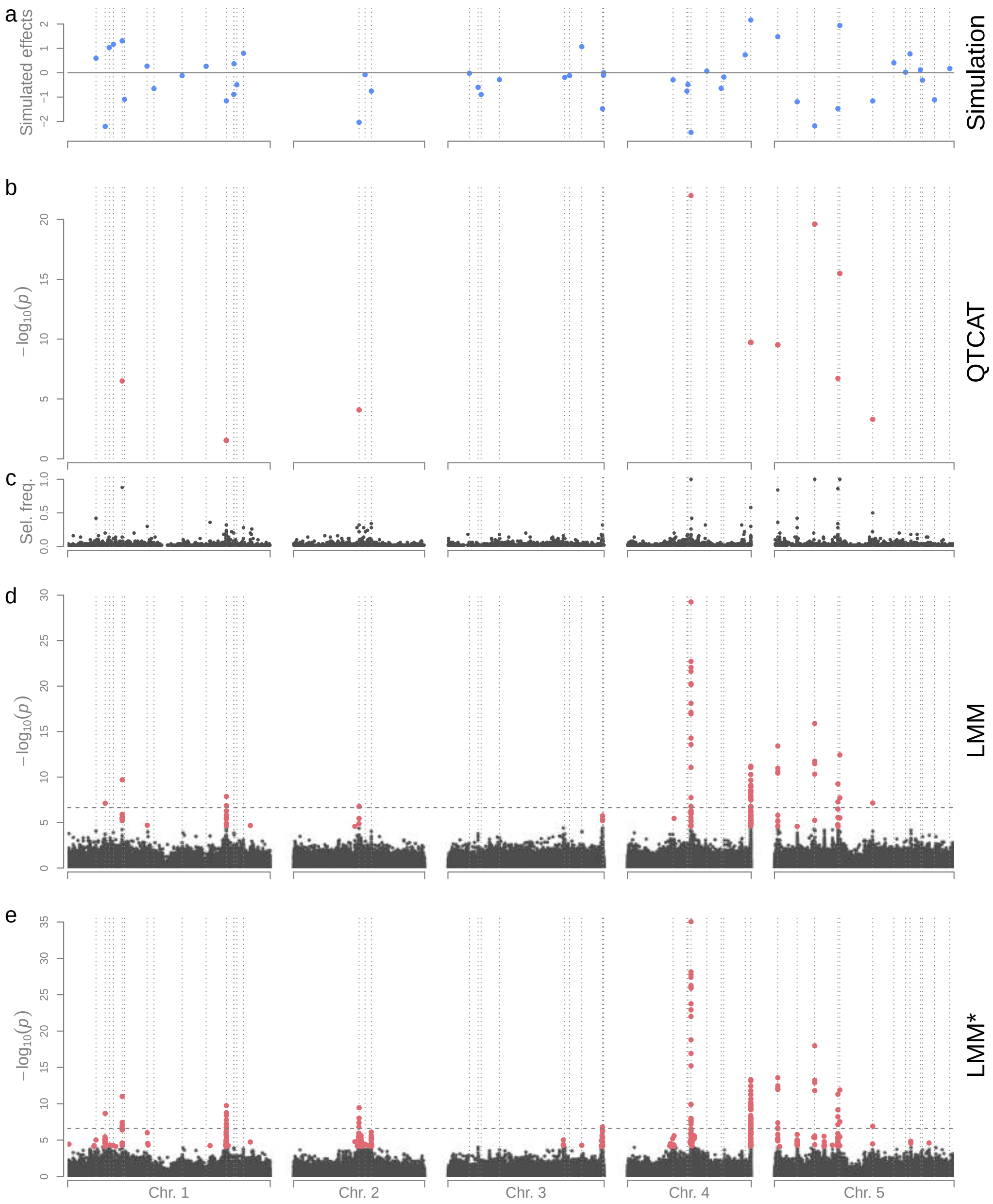

Supplementary Figure 196 Simulation of a GWA analysis based on a structured population with a heritability of 0.7 (run 96). (**a**) Simulation of 50 effects randomly drawn from a normal distribution and assigned to random markers. Markers with effect are highlighted with dashed lines. (**b**) Significant QTCs found by QTCAT. (**c**) LASSO selection frequency for each marker during the 50 iterations of QTCAT. (**d**) Manhattan plot of the LMM analysis. The horizontal dashed line depicts the significance threshold when controlling the multiple testing with FWER, whereas the red markers are significantly associated when controlling with FDR. (**e**) The Manhattan plot of the LMM* analysis. GRM was estimated without markers on the chromosome of the actual testing position. The results are shown as in (d).

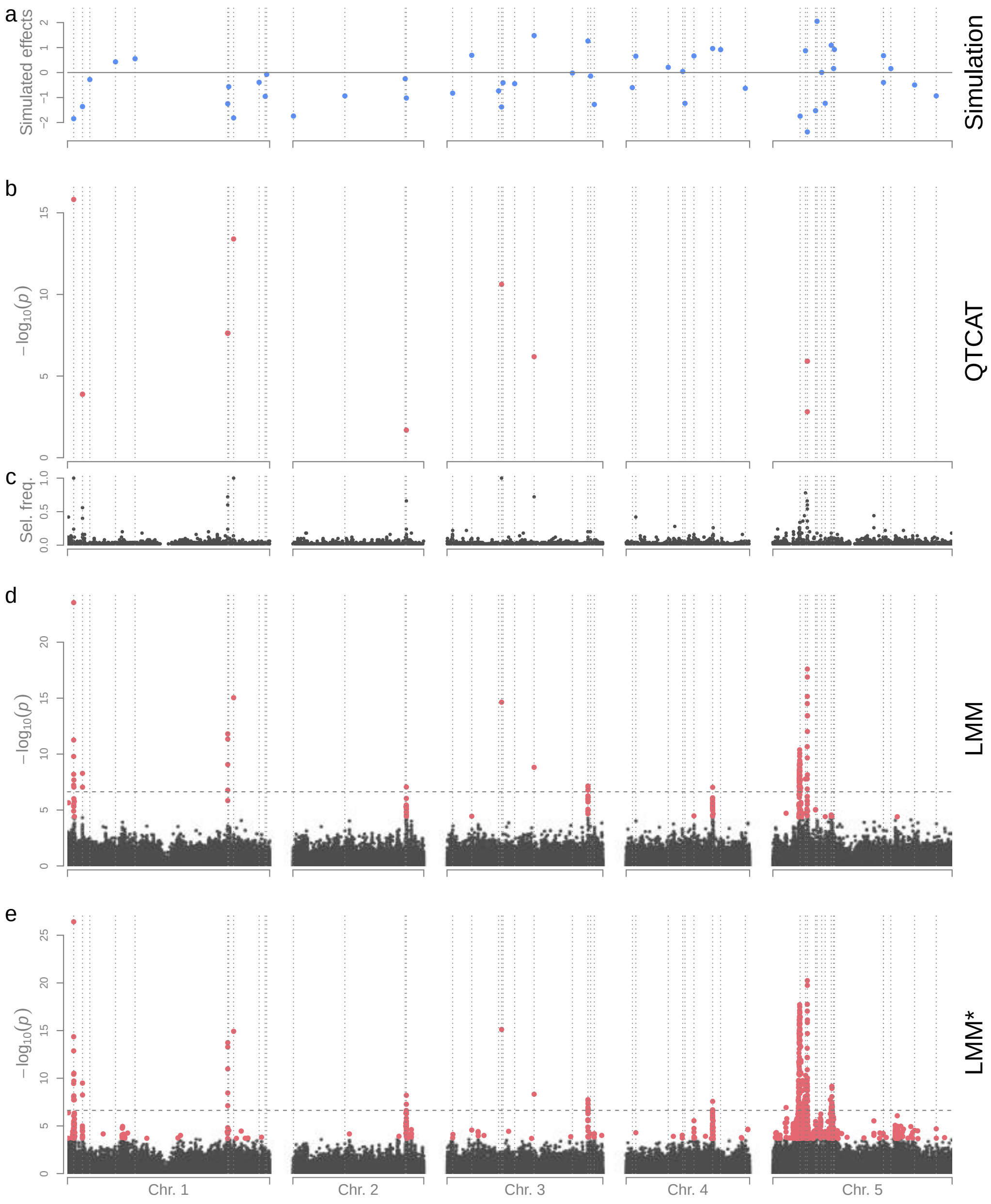

Supplementary Figure 197 Simulation of a GWA analysis based on a structured population with a heritability of 0.7 (run 97). (**a**) Simulation of 50 effects randomly drawn from a normal distribution and assigned to random markers. Markers with effect are highlighted with dashed lines. (**b**) Significant QTCs found by QTCAT. (**c**) LASSO selection frequency for each marker during the 50 iterations of QTCAT. (**d**) Manhattan plot of the LMM analysis. The horizontal dashed line depicts the significance threshold when controlling the multiple testing with FWER, whereas the red markers are significantly associated when controlling with FDR. (**e**) The Manhattan plot of the LMM* analysis. GRM was estimated without markers on the chromosome of the actual testing position. The results are shown as in (d).

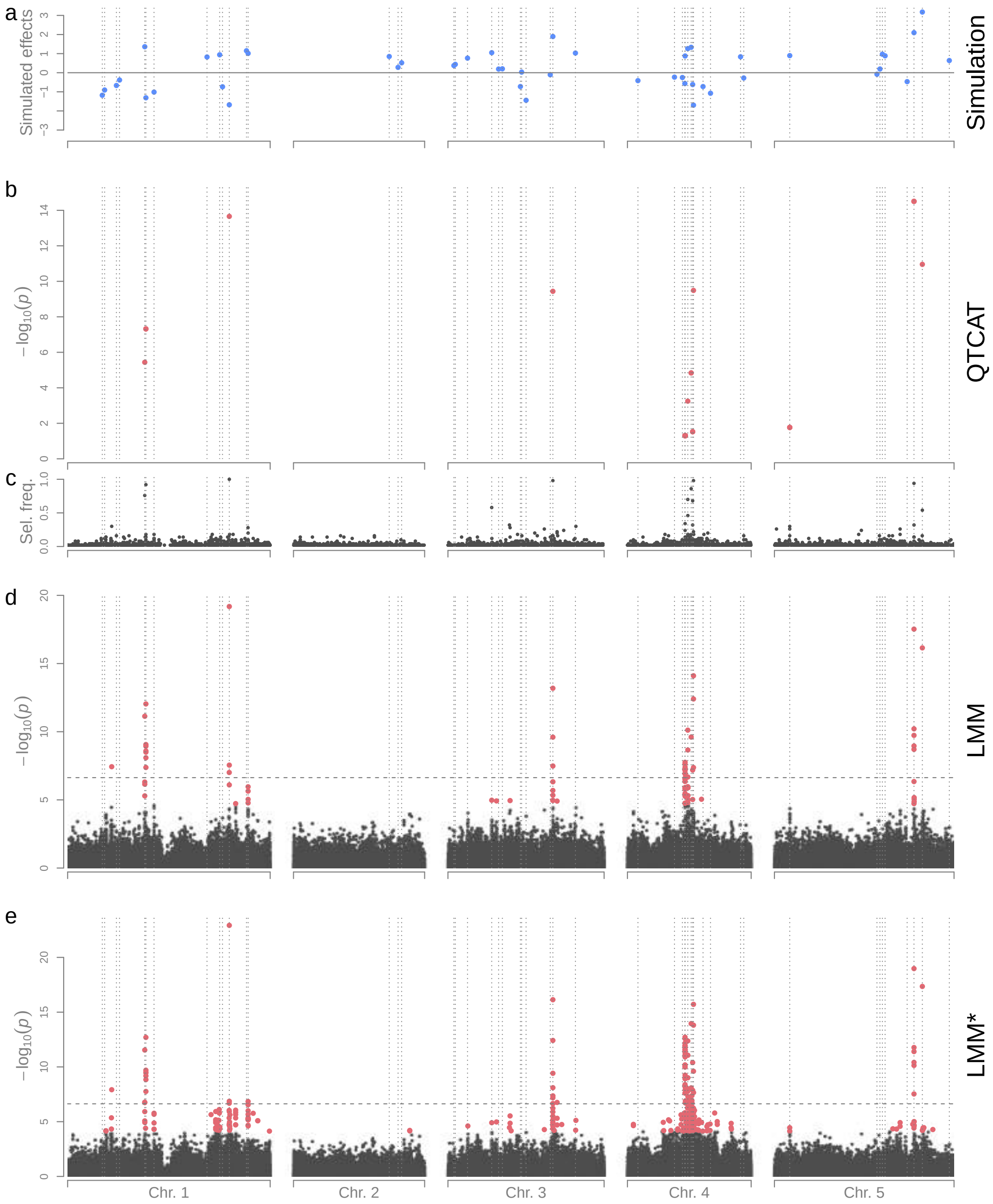

Supplementary Figure 198 Simulation of a GWA analysis based on a structured population with a heritability of 0.7 (run 98). (**a**) Simulation of 50 effects randomly drawn from a normal distribution and assigned to random markers. Markers with effect are highlighted with dashed lines. (**b**) Significant QTCs found by QTCAT. (**c**) LASSO selection frequency for each marker during the 50 iterations of QTCAT. (**d**) Manhattan plot of the LMM analysis. The horizontal dashed line depicts the significance threshold when controlling the multiple testing with FWER, whereas the red markers are significantly associated when controlling with FDR. (**e**) The Manhattan plot of the LMM* analysis. GRM was estimated without markers on the chromosome of the actual testing position. The results are shown as in (d).

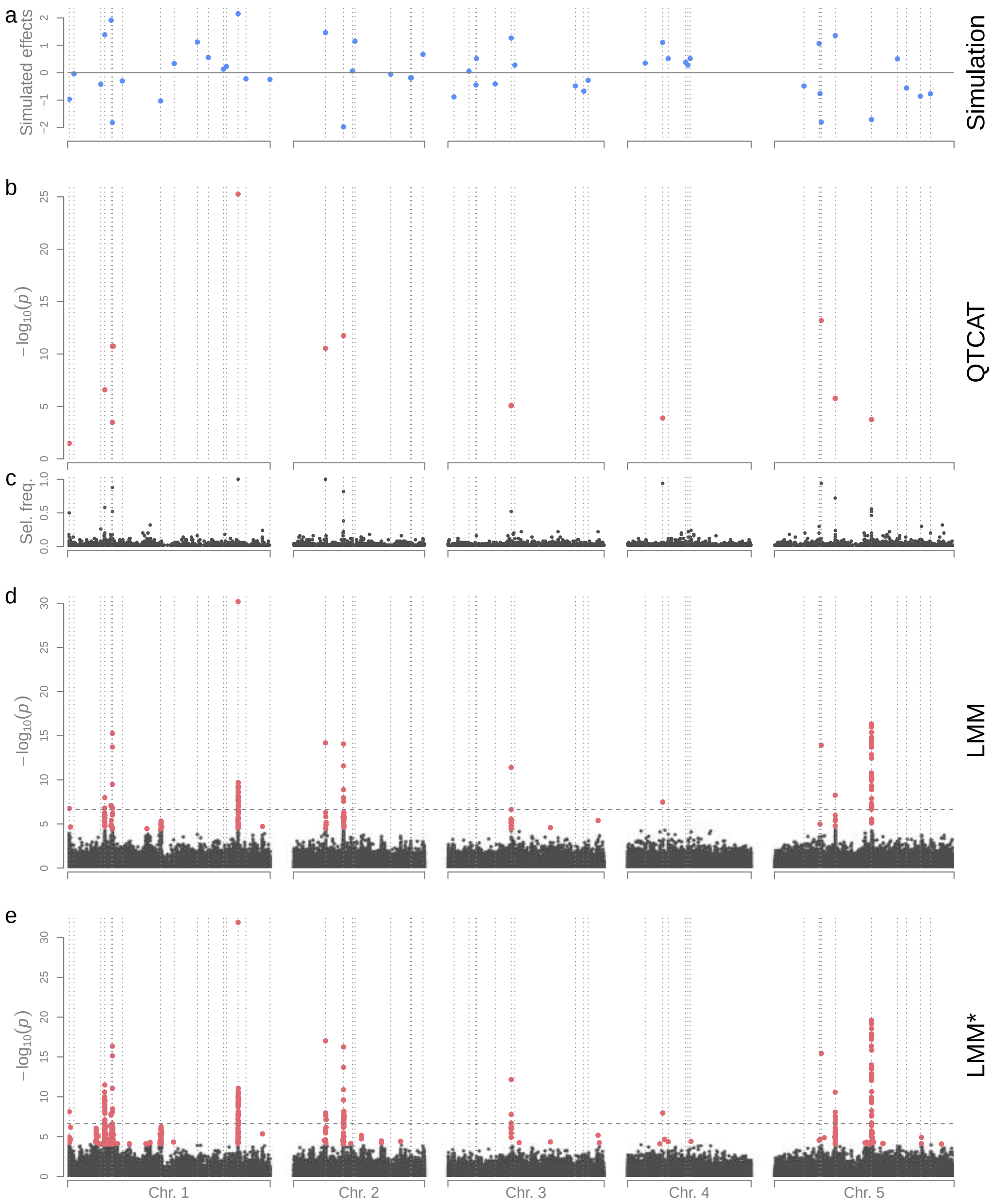

Supplementary Figure 199 Simulation of a GWA analysis based on a structured population with a heritability of 0.7 (run 99). (**a**) Simulation of 50 effects randomly drawn from a normal distribution and assigned to random markers. Markers with effect are highlighted with dashed lines. (**b**) Significant QTCs found by QTCAT. (**c**) LASSO selection frequency for each marker during the 50 iterations of QTCAT. (**d**) Manhattan plot of the LMM analysis. The horizontal dashed line depicts the significance threshold when controlling the multiple testing with FWER, whereas the red markers are significantly associated when controlling with FDR. (**e**) The Manhattan plot of the LMM* analysis. GRM was estimated without markers on the chromosome of the actual testing position. The results are shown as in (d).

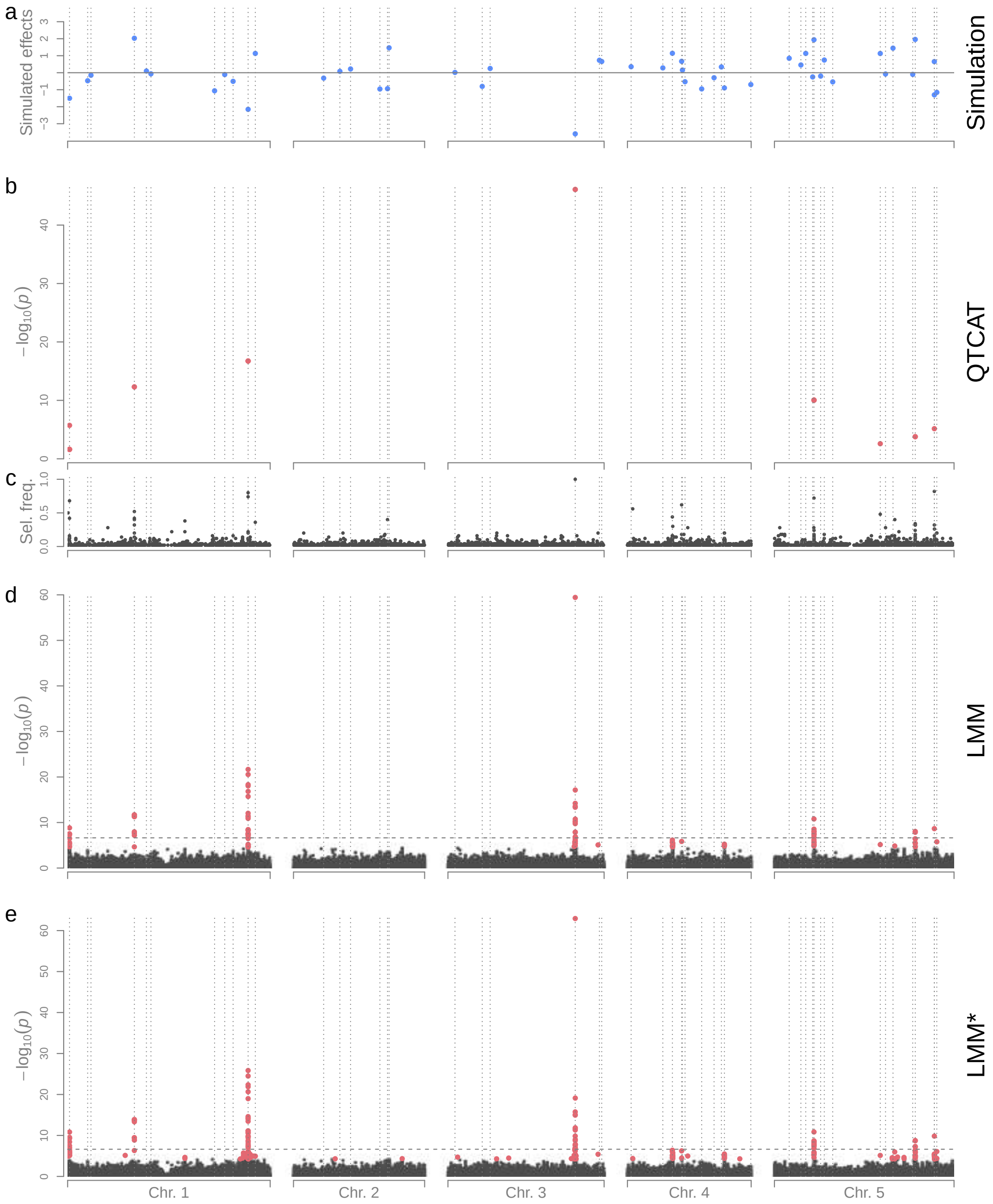

Supplementary Figure 200 Simulation of a GWA analysis based on a structured population with a heritability of 0.7 (run 100). (**a**) Simulation of 50 effects randomly drawn from a normal distribution and assigned to random markers. Markers with effect are highlighted with dashed lines. (**b**) Significant QTCs found by QTCAT. (**c**) LASSO selection frequency for each marker during the 50 iterations of QTCAT. (**d**) Manhattan plot of the LMM analysis. The horizontal dashed line depicts the significance threshold when controlling the multiple testing with FWER, whereas the red markers are significantly associated when controlling with FDR. (**e**) The Manhattan plot of the LMM* analysis. GRM was estimated without markers on the chromosome of the actual testing position. The results are shown as in (d).

Simulation with heritability of 0.4 and 150 effects randomly drawn from a normal distribution

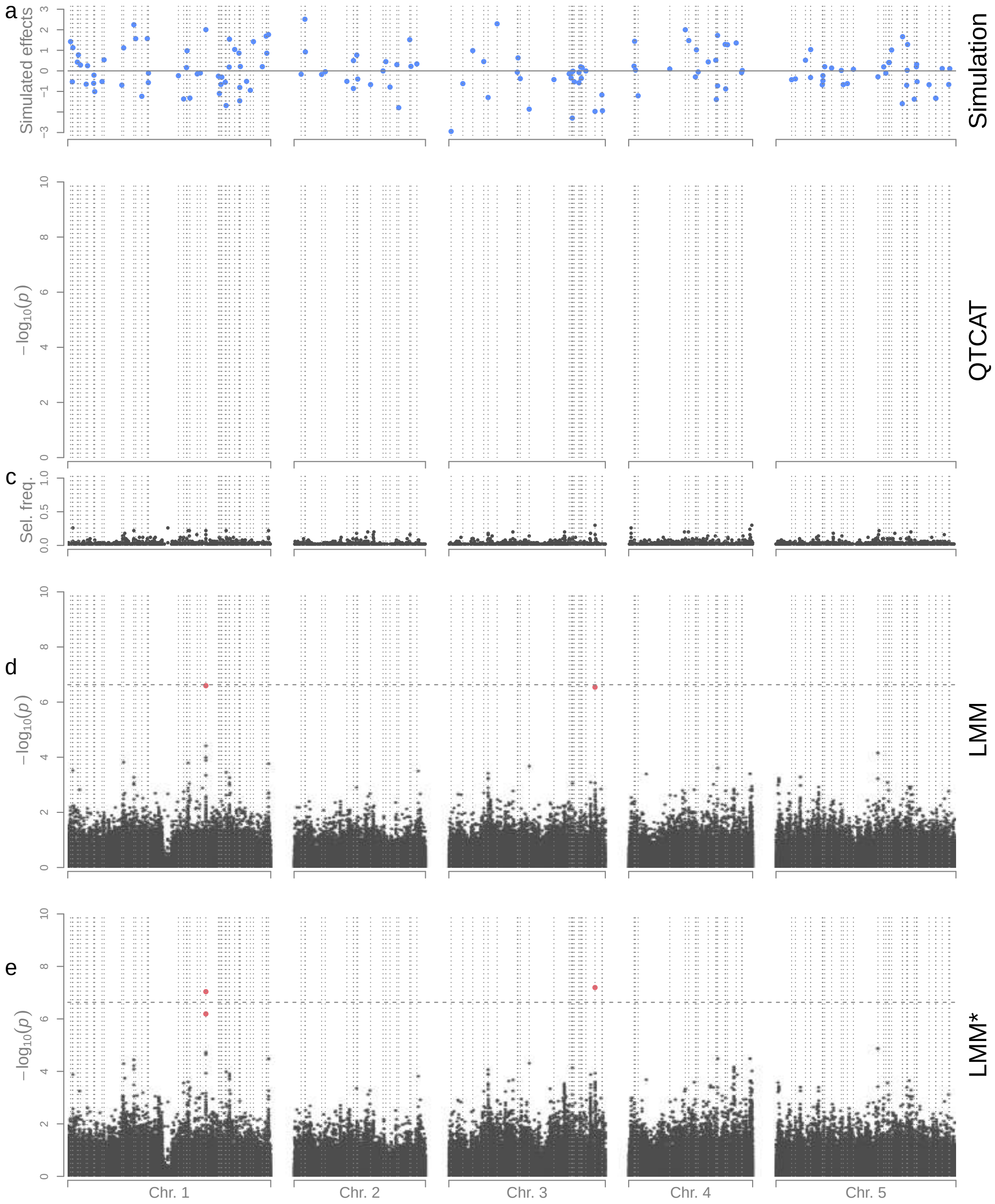

Supplementary Figure 201 Simulation of a GWA analysis based on a structured population with a heritability of 0.4 (run 1). (**a**) Simulation of 150 effects randomly drawn from a normal distribution and assigned to random markers. Markers with effect are highlighted with dashed lines. (**b**) Significant QTCs found by QTCAT. (**c**) LASSO selection frequency for each marker during the 50 iterations of QTCAT. (**d**) Manhattan plot of the LMM analysis. The horizontal dashed line depicts the significance threshold when controlling the multiple testing with FWER, whereas the red markers are significantly associated when controlling with FDR. (**e**) The Manhattan plot of the LMM* analysis. GRM was estimated without markers on the chromosome of the actual testing position. The results are shown as in (d).

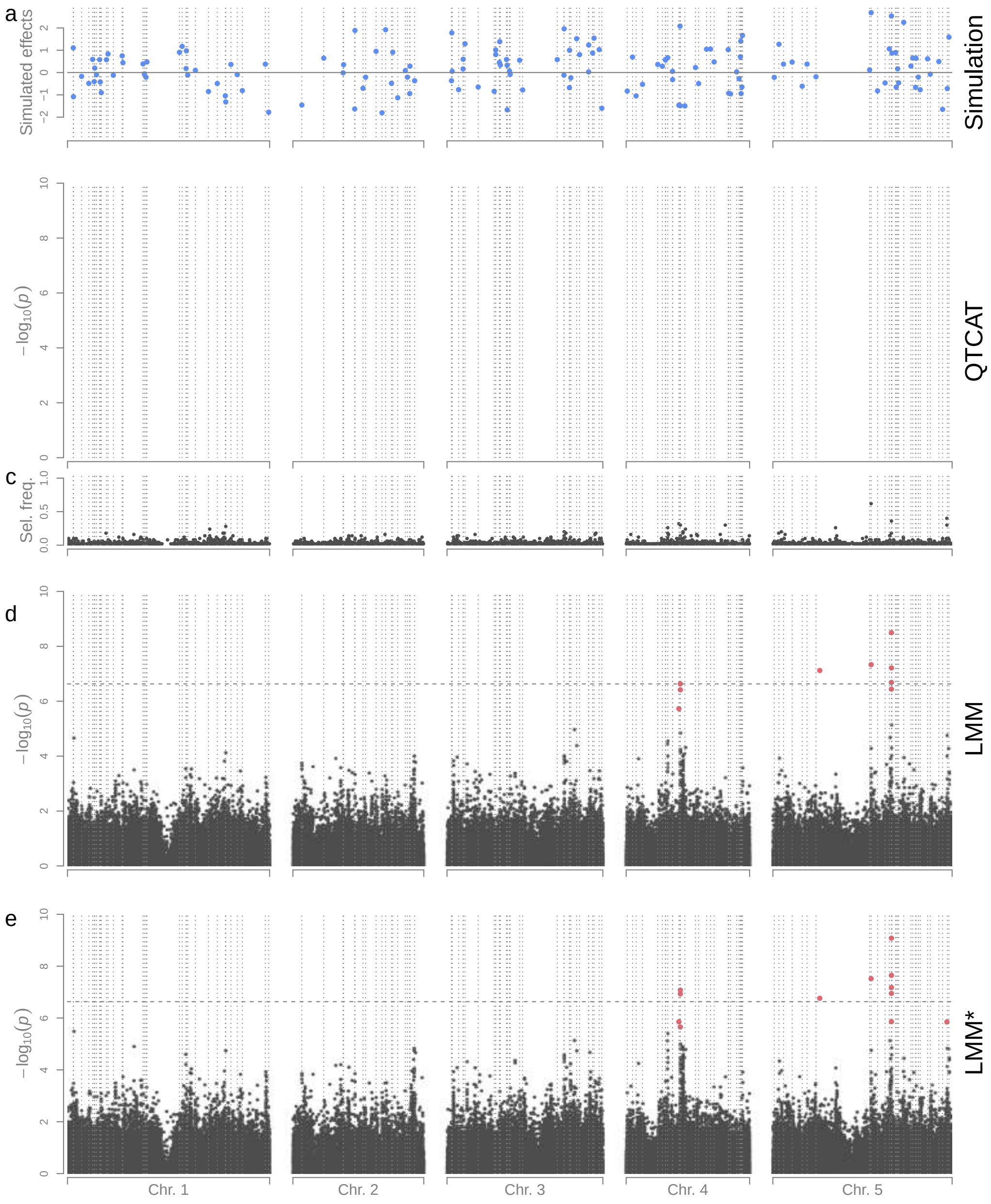

Supplementary Figure 202 Simulation of a GWA analysis based on a structured population with a heritability of 0.4 (run 2). (**a**) Simulation of 150 effects randomly drawn from a normal distribution and assigned to random markers. Markers with effect are highlighted with dashed lines. (**b**) Significant QTCs found by QTCAT. (**c**) LASSO selection frequency for each marker during the 50 iterations of QTCAT. (**d**) Manhattan plot of the LMM analysis. The horizontal dashed line depicts the significance threshold when controlling the multiple testing with FWER, whereas the red markers are significantly associated when controlling with FDR. (**e**) The Manhattan plot of the LMM* analysis. GRM was estimated without markers on the chromosome of the actual testing position. The results are shown as in (d).

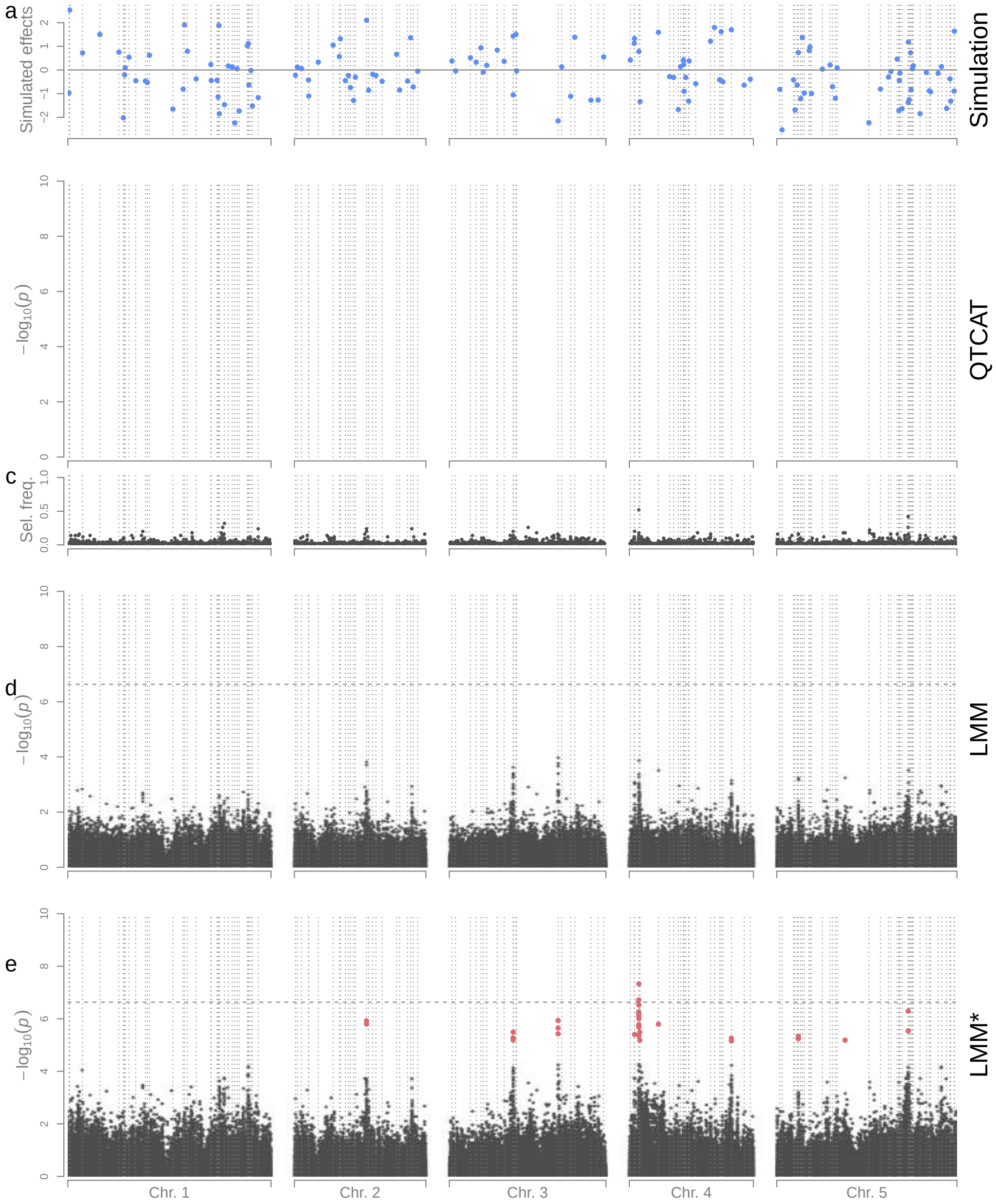

Supplementary Figure 203 Simulation of a GWA analysis based on a structured population with a heritability of 0.4 (run 3). (**a**) Simulation of 150 effects randomly drawn from a normal distribution and assigned to random markers. Markers with effect are highlighted with dashed lines. (**b**) Significant QTCs found by QTCAT. (**c**) LASSO selection frequency for each marker during the 50 iterations of QTCAT. (**d**) Manhattan plot of the LMM analysis. The horizontal dashed line depicts the significance threshold when controlling the multiple testing with FWER, whereas the red markers are significantly associated when controlling with FDR. (**e**) The Manhattan plot of the LMM* analysis. GRM was estimated without markers on the chromosome of the actual testing position. The results are shown as in (d).

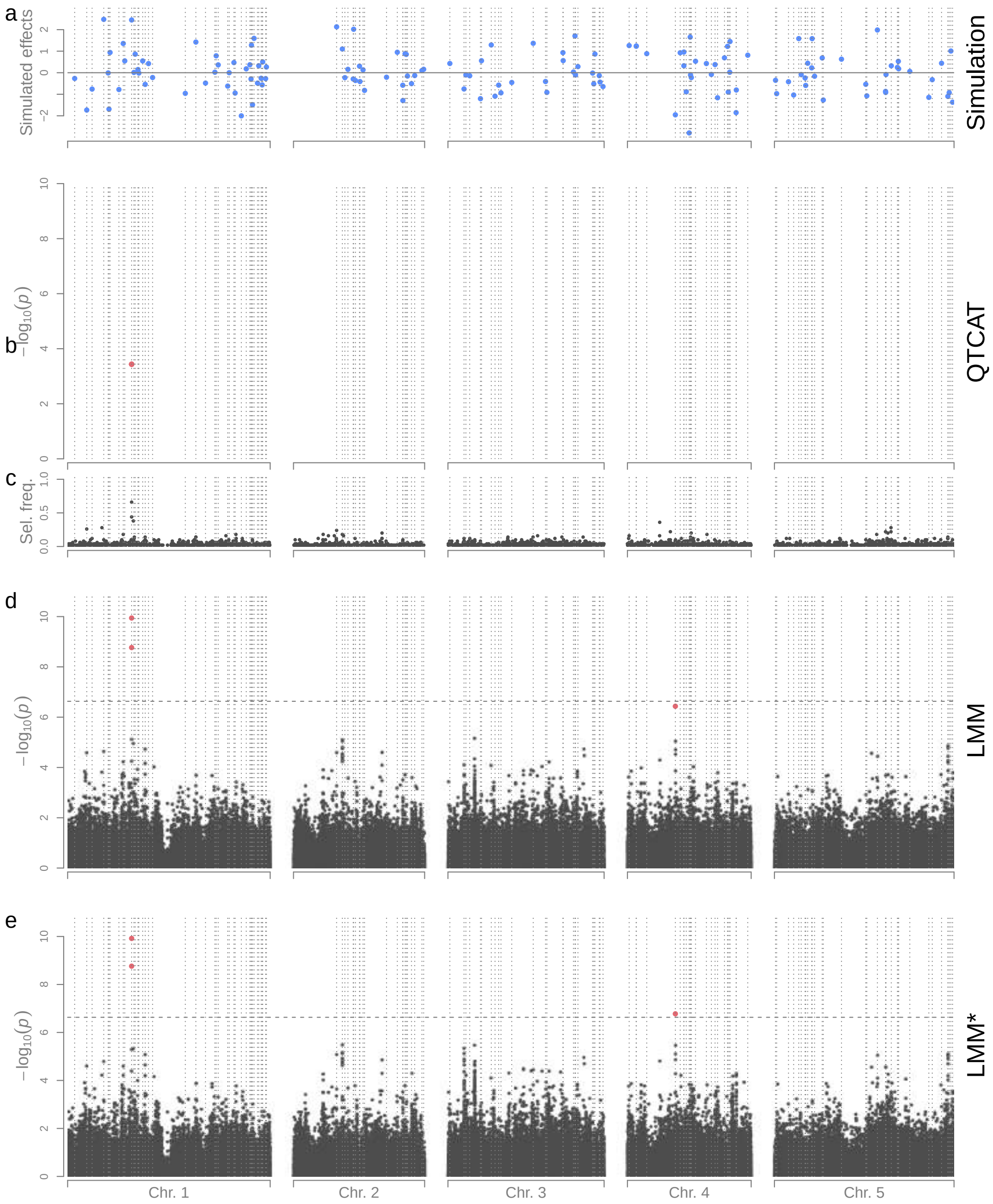

Supplementary Figure 204 Simulation of a GWA analysis based on a structured population with a heritability of 0.4 (run 4). (**a**) Simulation of 150 effects randomly drawn from a normal distribution and assigned to random markers. Markers with effect are highlighted with dashed lines. (**b**) Significant QTCs found by QTCAT. (**c**) LASSO selection frequency for each marker during the 50 iterations of QTCAT. (**d**) Manhattan plot of the LMM analysis. The horizontal dashed line depicts the significance threshold when controlling the multiple testing with FWER, whereas the red markers are significantly associated when controlling with FDR. (**e**) The Manhattan plot of the LMM* analysis. GRM was estimated without markers on the chromosome of the actual testing position. The results are shown as in (d).

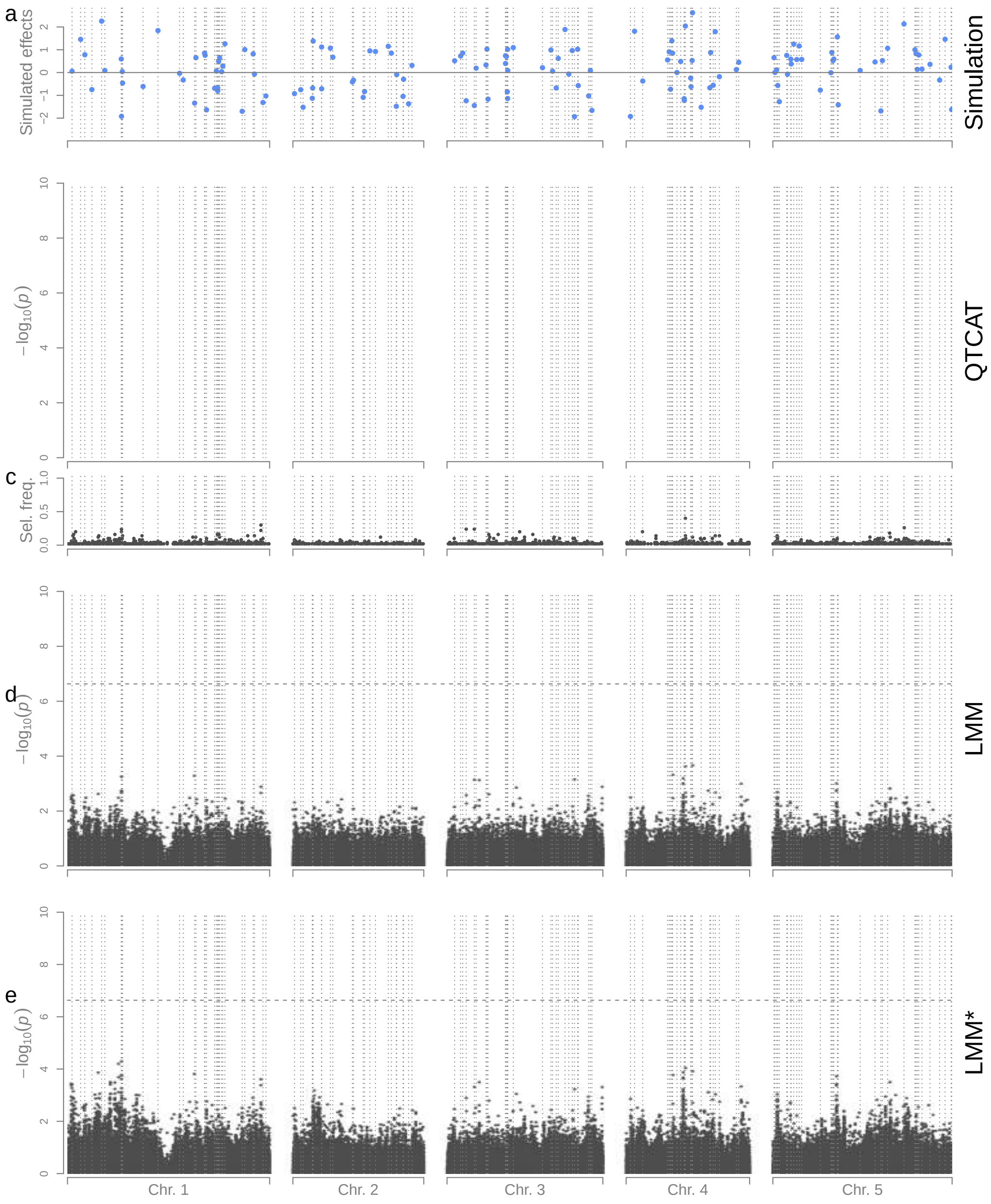

Supplementary Figure 205 Simulation of a GWA analysis based on a structured population with a heritability of 0.4 (run 5). (**a**) Simulation of 150 effects randomly drawn from a normal distribution and assigned to random markers. Markers with effect are highlighted with dashed lines. (**b**) Significant QTCs found by QTCAT. (**c**) LASSO selection frequency for each marker during the 50 iterations of QTCAT. (**d**) Manhattan plot of the LMM analysis. The horizontal dashed line depicts the significance threshold when controlling the multiple testing with FWER, whereas the red markers are significantly associated when controlling with FDR. (**e**) The Manhattan plot of the LMM* analysis. GRM was estimated without markers on the chromosome of the actual testing position. The results are shown as in (d).

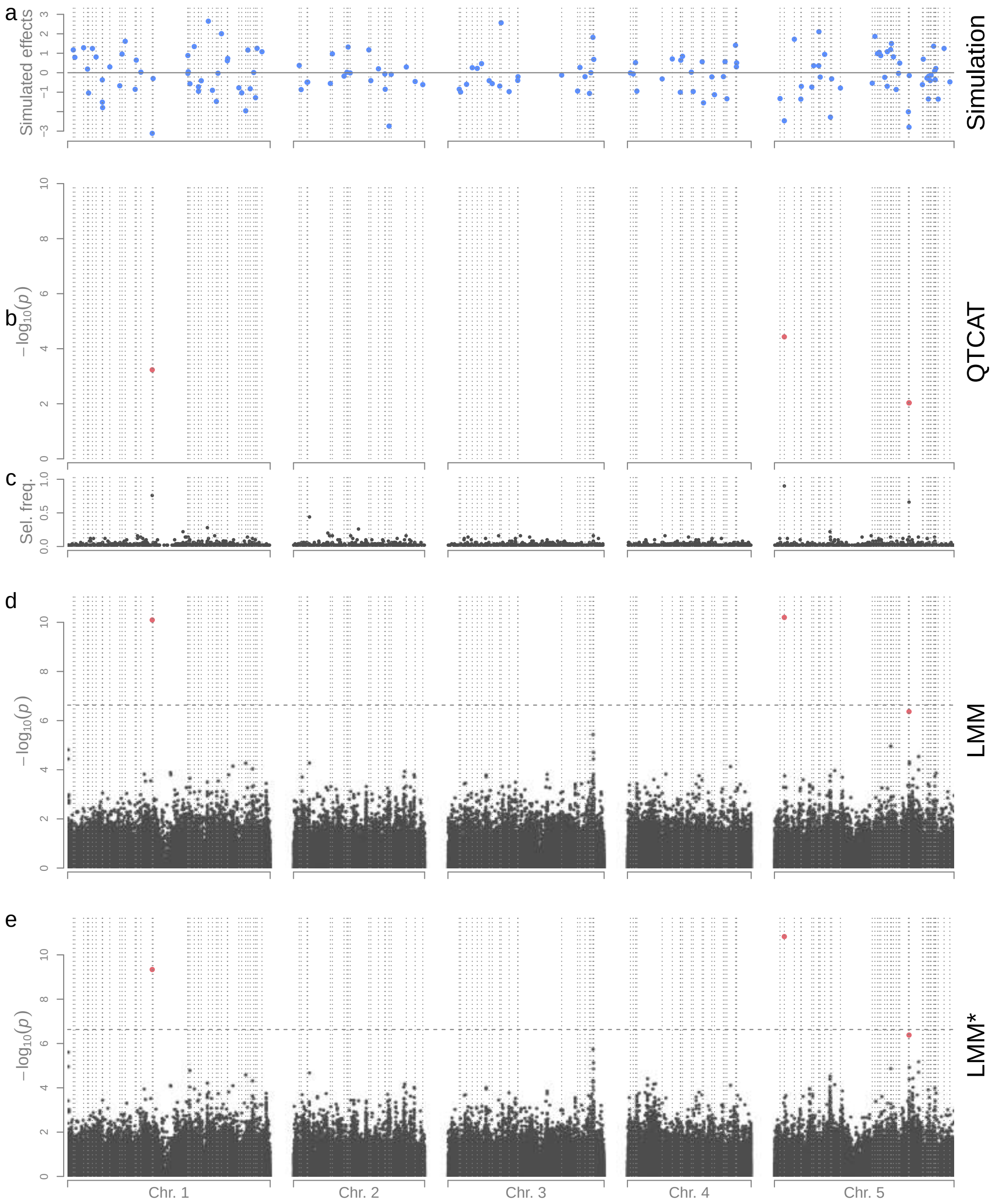

Supplementary Figure 206 Simulation of a GWA analysis based on a structured population with a heritability of 0.4 (run 6). (**a**) Simulation of 150 effects randomly drawn from a normal distribution and assigned to random markers. Markers with effect are highlighted with dashed lines. (**b**) Significant QTCs found by QTCAT. (**c**) LASSO selection frequency for each marker during the 50 iterations of QTCAT. (**d**) Manhattan plot of the LMM analysis. The horizontal dashed line depicts the significance threshold when controlling the multiple testing with FWER, whereas the red markers are significantly associated when controlling with FDR. (**e**) The Manhattan plot of the LMM* analysis. GRM was estimated without markers on the chromosome of the actual testing position. The results are shown as in (d).

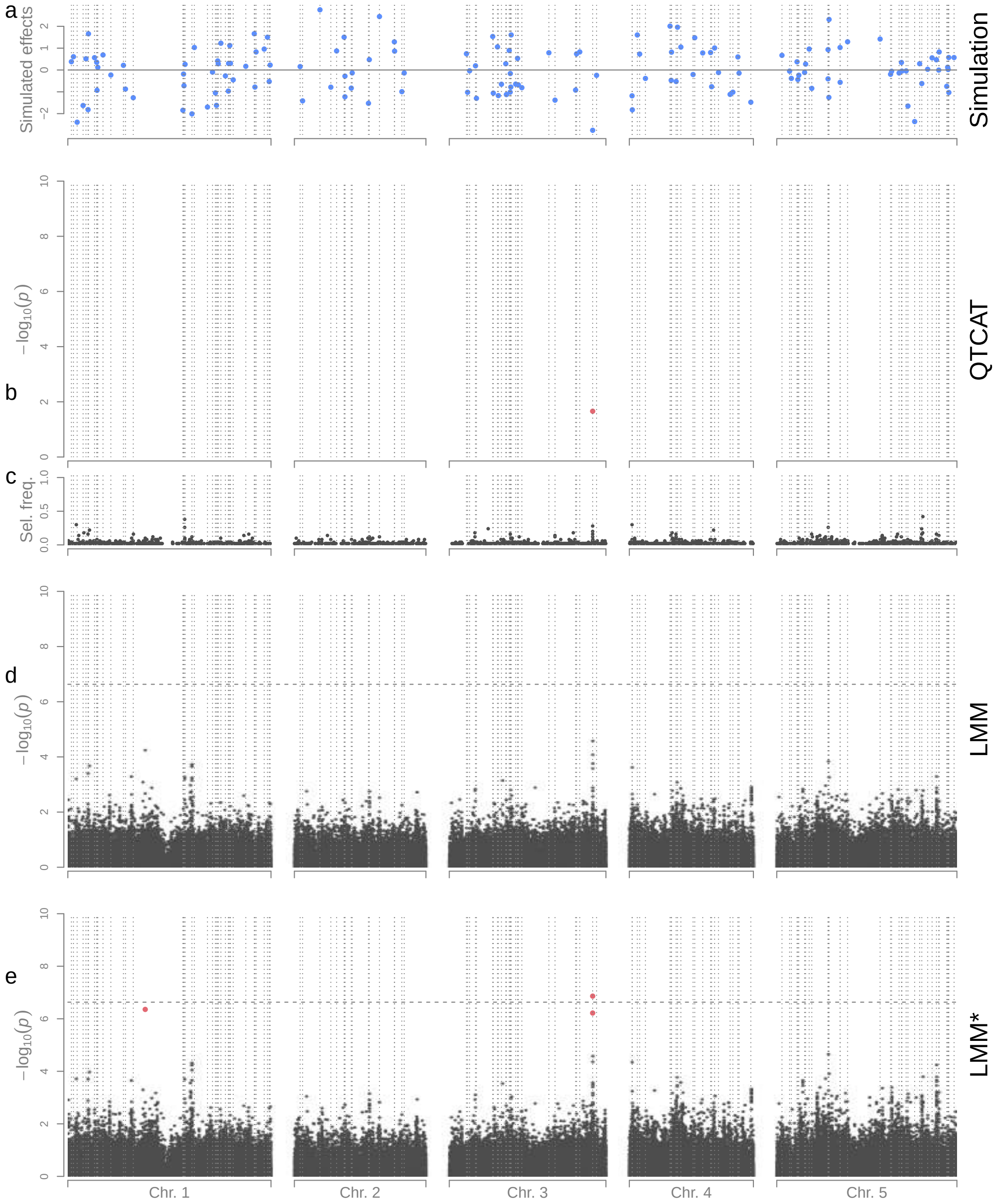

Supplementary Figure 207 Simulation of a GWA analysis based on a structured population with a heritability of 0.4 (run 7). (**a**) Simulation of 150 effects randomly drawn from a normal distribution and assigned to random markers. Markers with effect are highlighted with dashed lines. (**b**) Significant QTCs found by QTCAT. (**c**) LASSO selection frequency for each marker during the 50 iterations of QTCAT. (**d**) Manhattan plot of the LMM analysis. The horizontal dashed line depicts the significance threshold when controlling the multiple testing with FWER, whereas the red markers are significantly associated when controlling with FDR. (**e**) The Manhattan plot of the LMM* analysis. GRM was estimated without markers on the chromosome of the actual testing position. The results are shown as in (d).

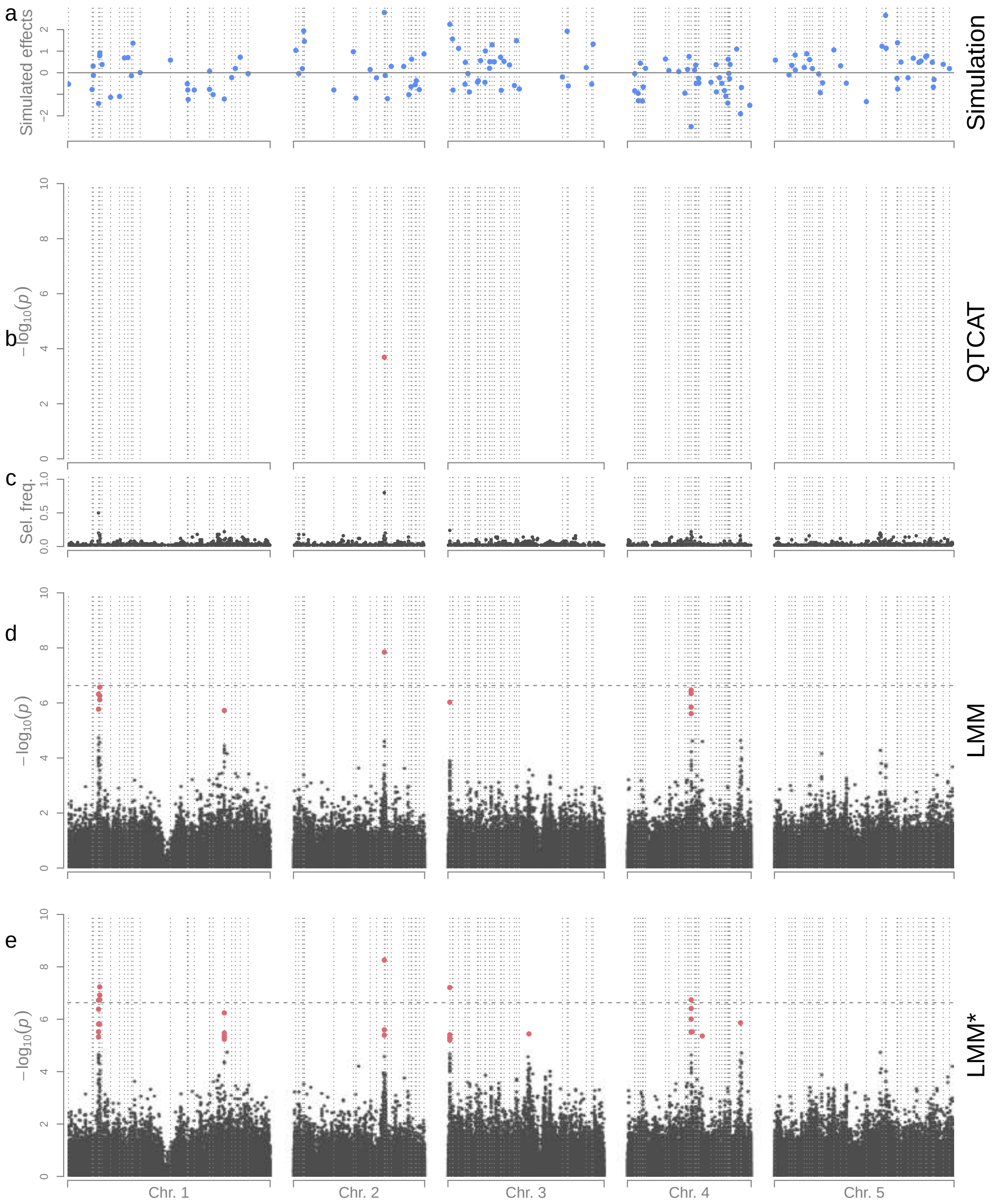

Supplementary Figure 208 Simulation of a GWA analysis based on a structured population with a heritability of 0.4 (run 8). (**a**) Simulation of 150 effects randomly drawn from a normal distribution and assigned to random markers. Markers with effect are highlighted with dashed lines. (**b**) Significant QTCs found by QTCAT. (**c**) LASSO selection frequency for each marker during the 50 iterations of QTCAT. (**d**) Manhattan plot of the LMM analysis. The horizontal dashed line depicts the significance threshold when controlling the multiple testing with FWER, whereas the red markers are significantly associated when controlling with FDR. (**e**) The Manhattan plot of the LMM* analysis. GRM was estimated without markers on the chromosome of the actual testing position. The results are shown as in (d).

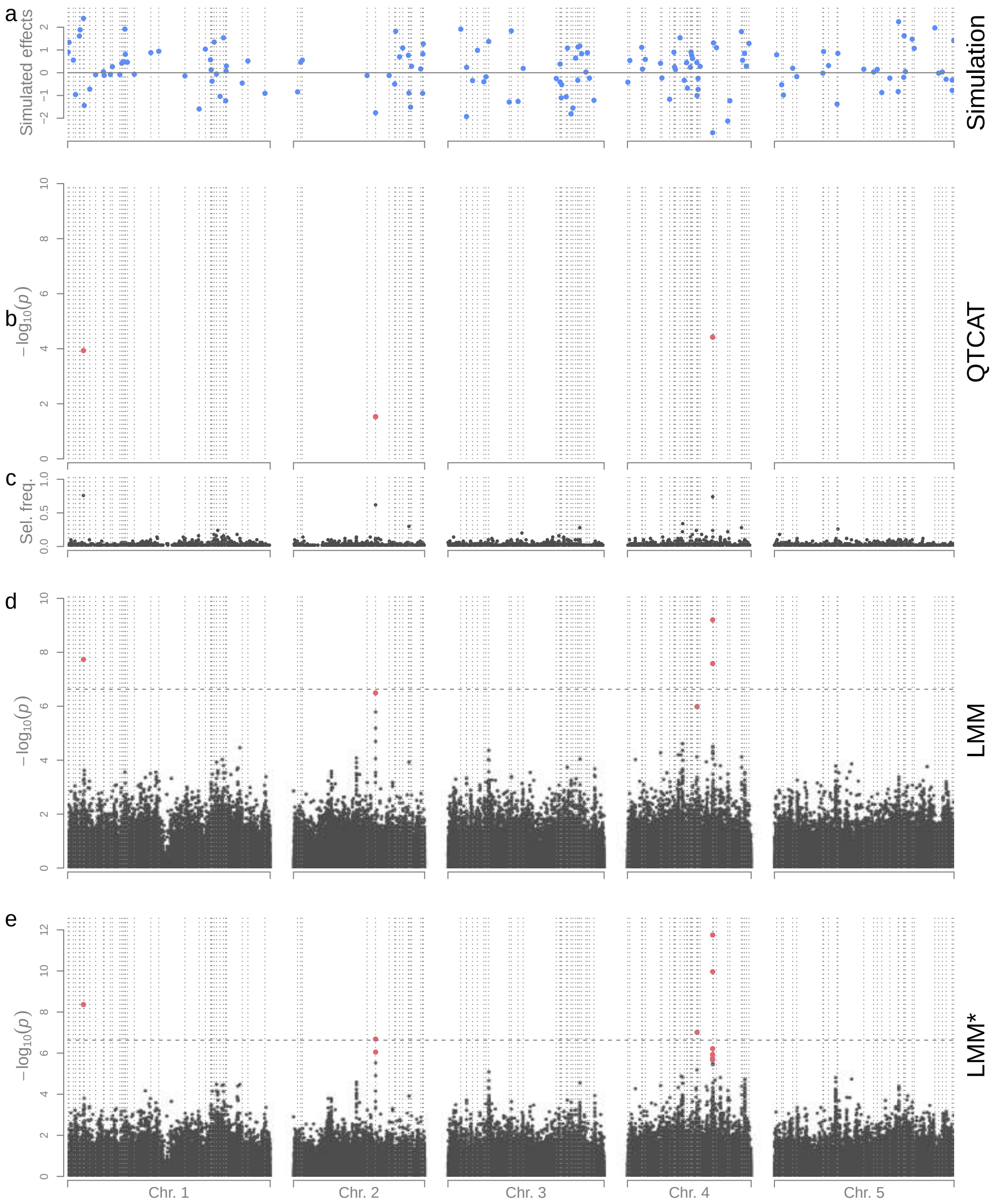

Supplementary Figure 209 Simulation of a GWA analysis based on a structured population with a heritability of 0.4 (run 9). (**a**) Simulation of 150 effects randomly drawn from a normal distribution and assigned to random markers. Markers with effect are highlighted with dashed lines. (**b**) Significant QTCs found by QTCAT. (**c**) LASSO selection frequency for each marker during the 50 iterations of QTCAT. (**d**) Manhattan plot of the LMM analysis. The horizontal dashed line depicts the significance threshold when controlling the multiple testing with FWER, whereas the red markers are significantly associated when controlling with FDR. (**e**) The Manhattan plot of the LMM* analysis. GRM was estimated without markers on the chromosome of the actual testing position. The results are shown as in (d).

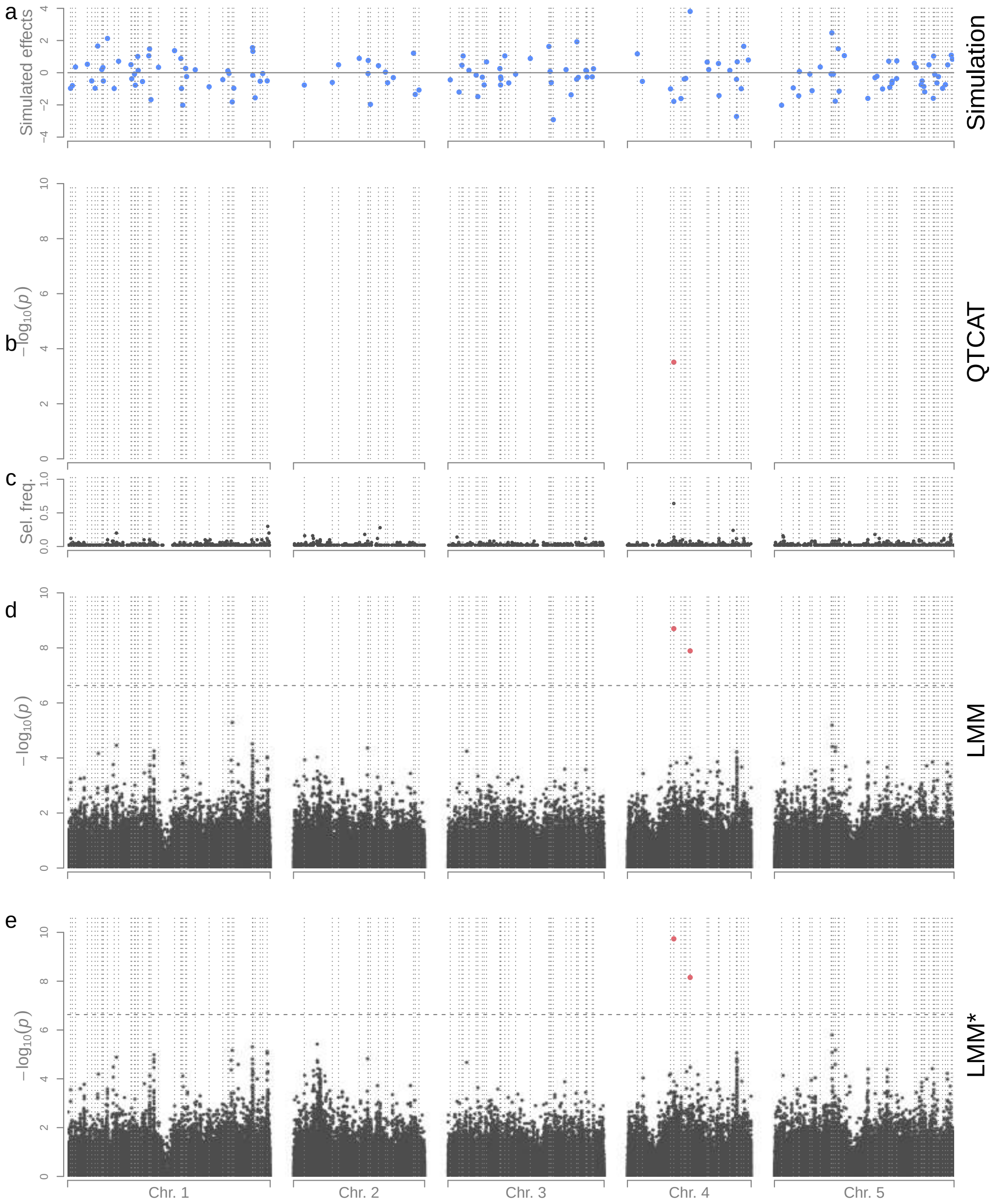

Supplementary Figure 210 Simulation of a GWA analysis based on a structured population with a heritability of 0.4 (run 10). (**a**) Simulation of 150 effects randomly drawn from a normal distribution and assigned to random markers. Markers with effect are highlighted with dashed lines. (**b**) Significant QTCs found by QTCAT. (**c**) LASSO selection frequency for each marker during the 50 iterations of QTCAT. (**d**) Manhattan plot of the LMM analysis. The horizontal dashed line depicts the significance threshold when controlling the multiple testing with FWER, whereas the red markers are significantly associated when controlling with FDR. (**e**) The Manhattan plot of the LMM* analysis. GRM was estimated without markers on the chromosome of the actual testing position. The results are shown as in (d).

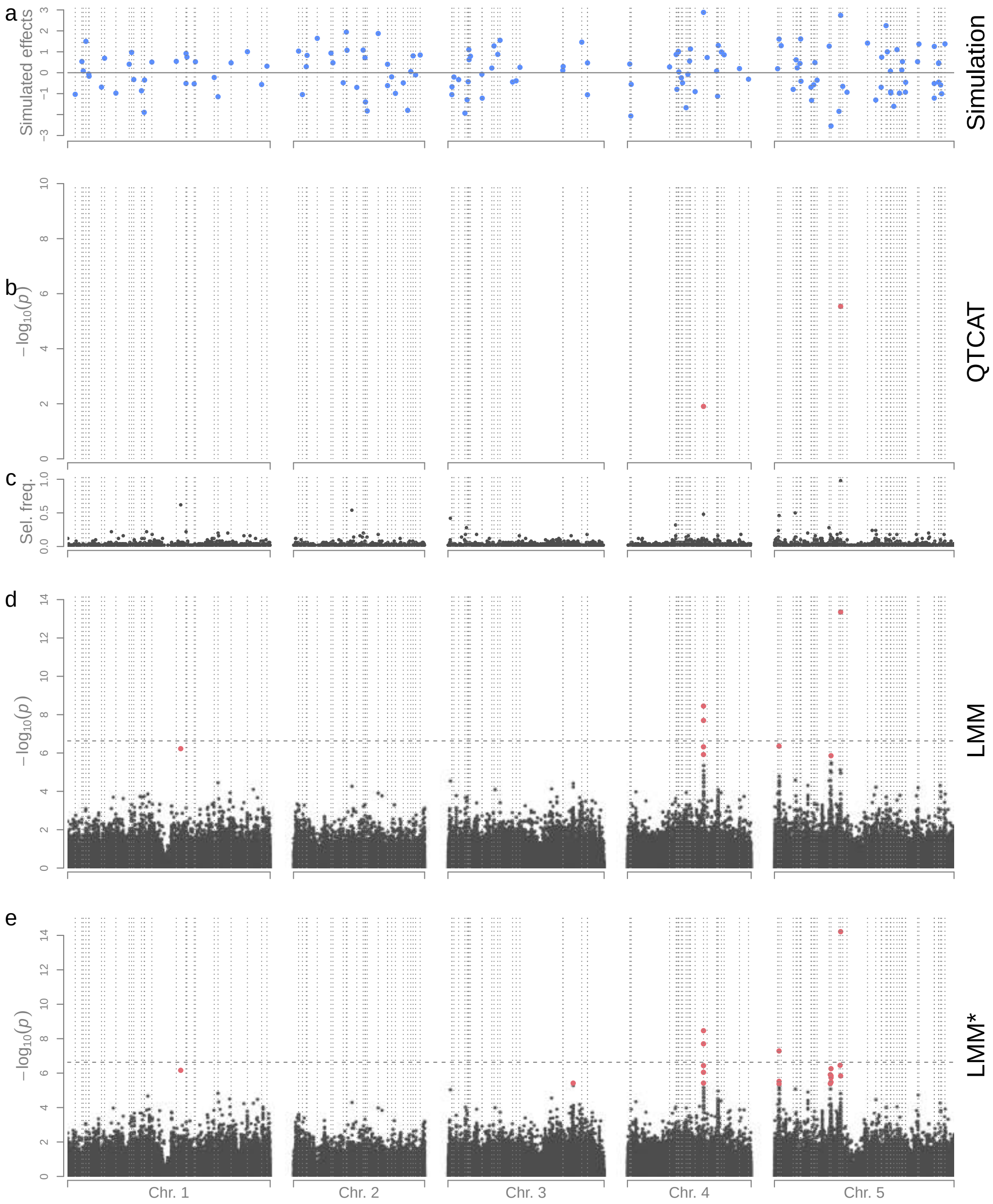

Supplementary Figure 211 Simulation of a GWA analysis based on a structured population with a heritability of 0.4 (run 11). (**a**) Simulation of 150 effects randomly drawn from a normal distribution and assigned to random markers. Markers with effect are highlighted with dashed lines. (**b**) Significant QTCs found by QTCAT. (**c**) LASSO selection frequency for each marker during the 50 iterations of QTCAT. (**d**) Manhattan plot of the LMM analysis. The horizontal dashed line depicts the significance threshold when controlling the multiple testing with FWER, whereas the red markers are significantly associated when controlling with FDR. (**e**) The Manhattan plot of the LMM* analysis. GRM was estimated without markers on the chromosome of the actual testing position. The results are shown as in (d).

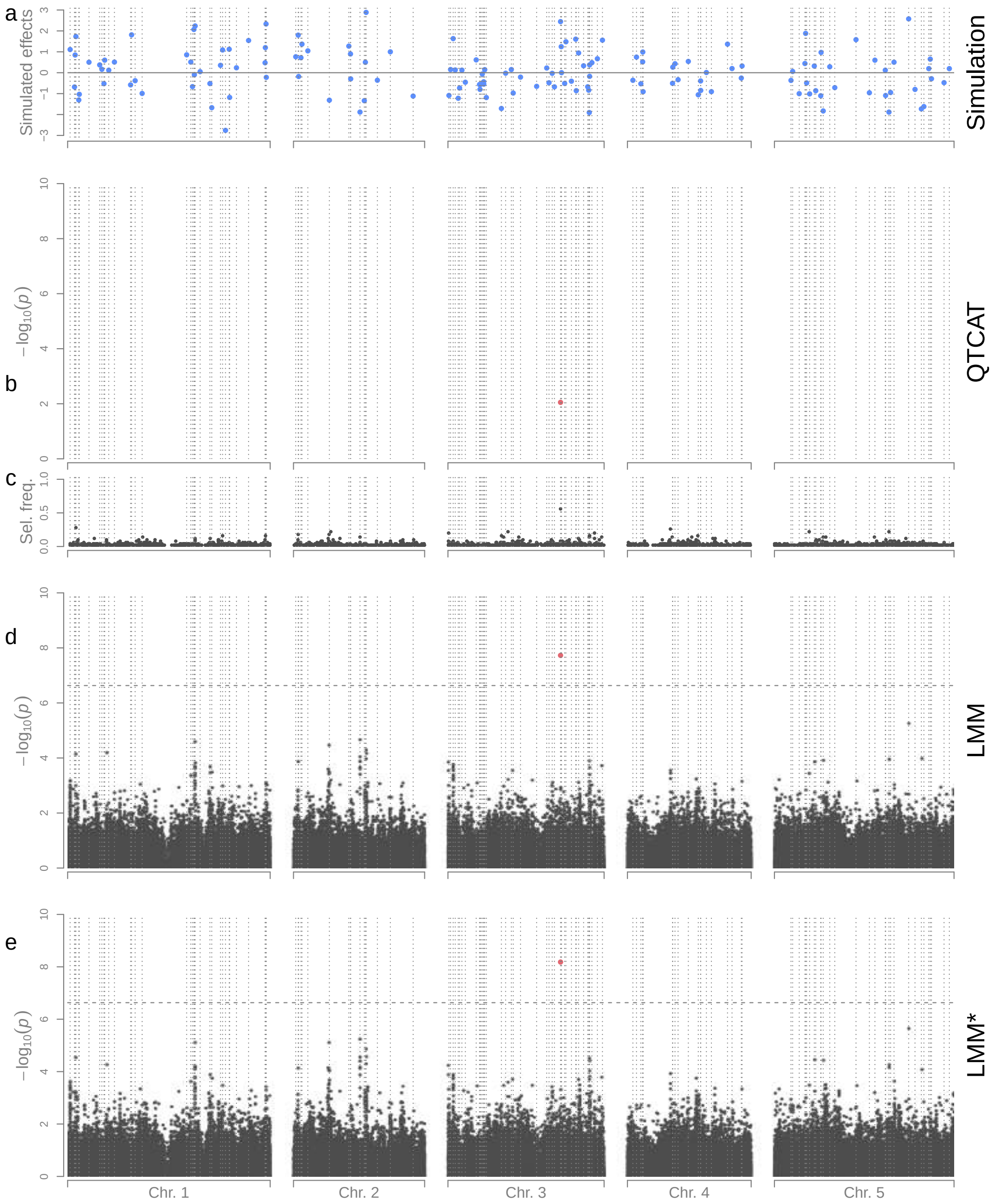

Supplementary Figure 212 Simulation of a GWA analysis based on a structured population with a heritability of 0.4 (run 12). (**a**) Simulation of 150 effects randomly drawn from a normal distribution and assigned to random markers. Markers with effect are highlighted with dashed lines. (**b**) Significant QTCs found by QTCAT. (**c**) LASSO selection frequency for each marker during the 50 iterations of QTCAT. (**d**) Manhattan plot of the LMM analysis. The horizontal dashed line depicts the significance threshold when controlling the multiple testing with FWER, whereas the red markers are significantly associated when controlling with FDR. (**e**) The Manhattan plot of the LMM* analysis. GRM was estimated without markers on the chromosome of the actual testing position. The results are shown as in (d).

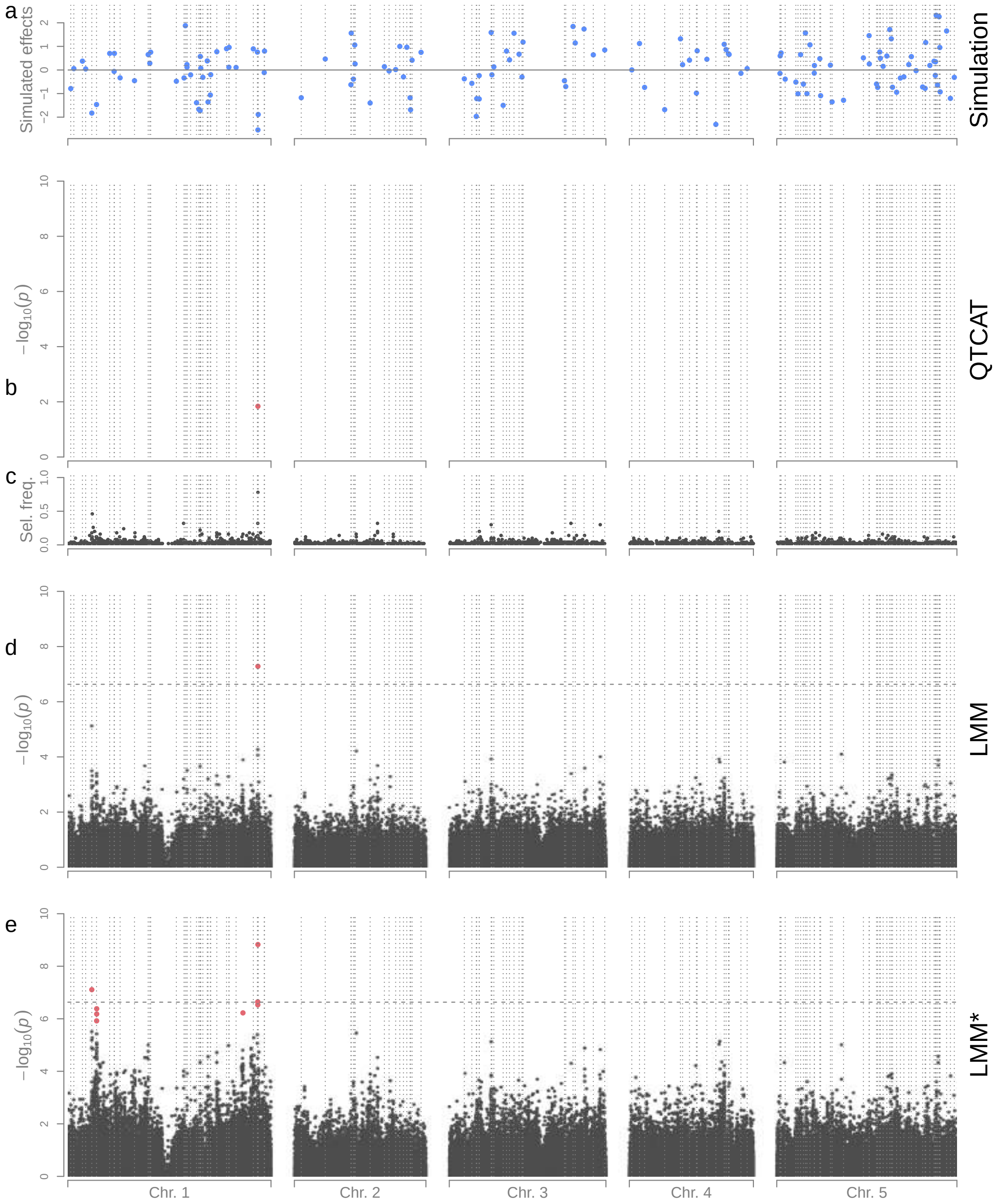

Supplementary Figure 213 Simulation of a GWA analysis based on a structured population with a heritability of 0.4 (run 13). (**a**) Simulation of 150 effects randomly drawn from a normal distribution and assigned to random markers. Markers with effect are highlighted with dashed lines. (**b**) Significant QTCs found by QTCAT. (**c**) LASSO selection frequency for each marker during the 50 iterations of QTCAT. (**d**) Manhattan plot of the LMM analysis. The horizontal dashed line depicts the significance threshold when controlling the multiple testing with FWER, whereas the red markers are significantly associated when controlling with FDR. (**e**) The Manhattan plot of the LMM* analysis. GRM was estimated without markers on the chromosome of the actual testing position. The results are shown as in (d).

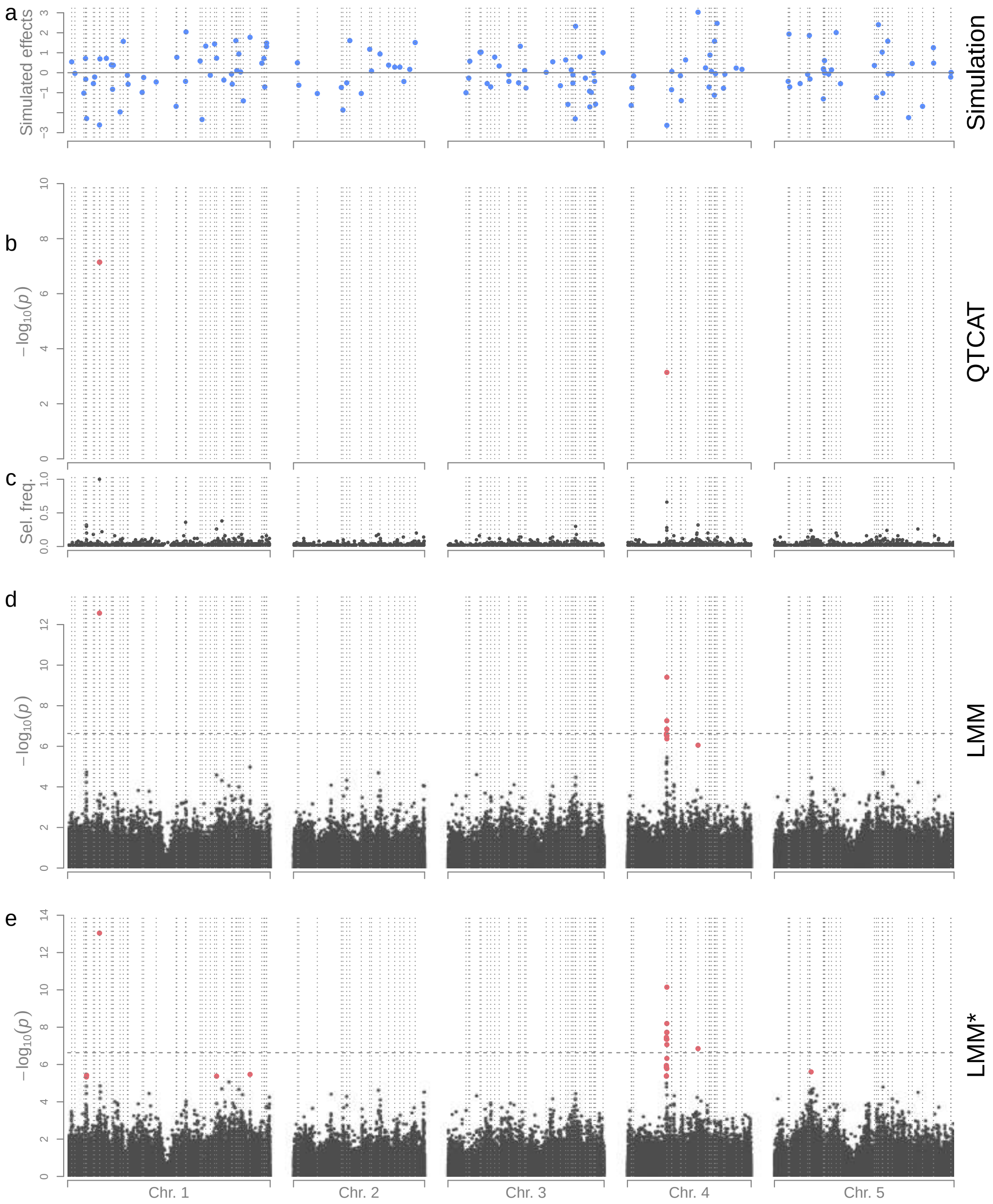

Supplementary Figure 214 Simulation of a GWA analysis based on a structured population with a heritability of 0.4 (run 14). (**a**) Simulation of 150 effects randomly drawn from a normal distribution and assigned to random markers. Markers with effect are highlighted with dashed lines. (**b**) Significant QTCs found by QTCAT. (**c**) LASSO selection frequency for each marker during the 50 iterations of QTCAT. (**d**) Manhattan plot of the LMM analysis. The horizontal dashed line depicts the significance threshold when controlling the multiple testing with FWER, whereas the red markers are significantly associated when controlling with FDR. (**e**) The Manhattan plot of the LMM* analysis. GRM was estimated without markers on the chromosome of the actual testing position. The results are shown as in (d).

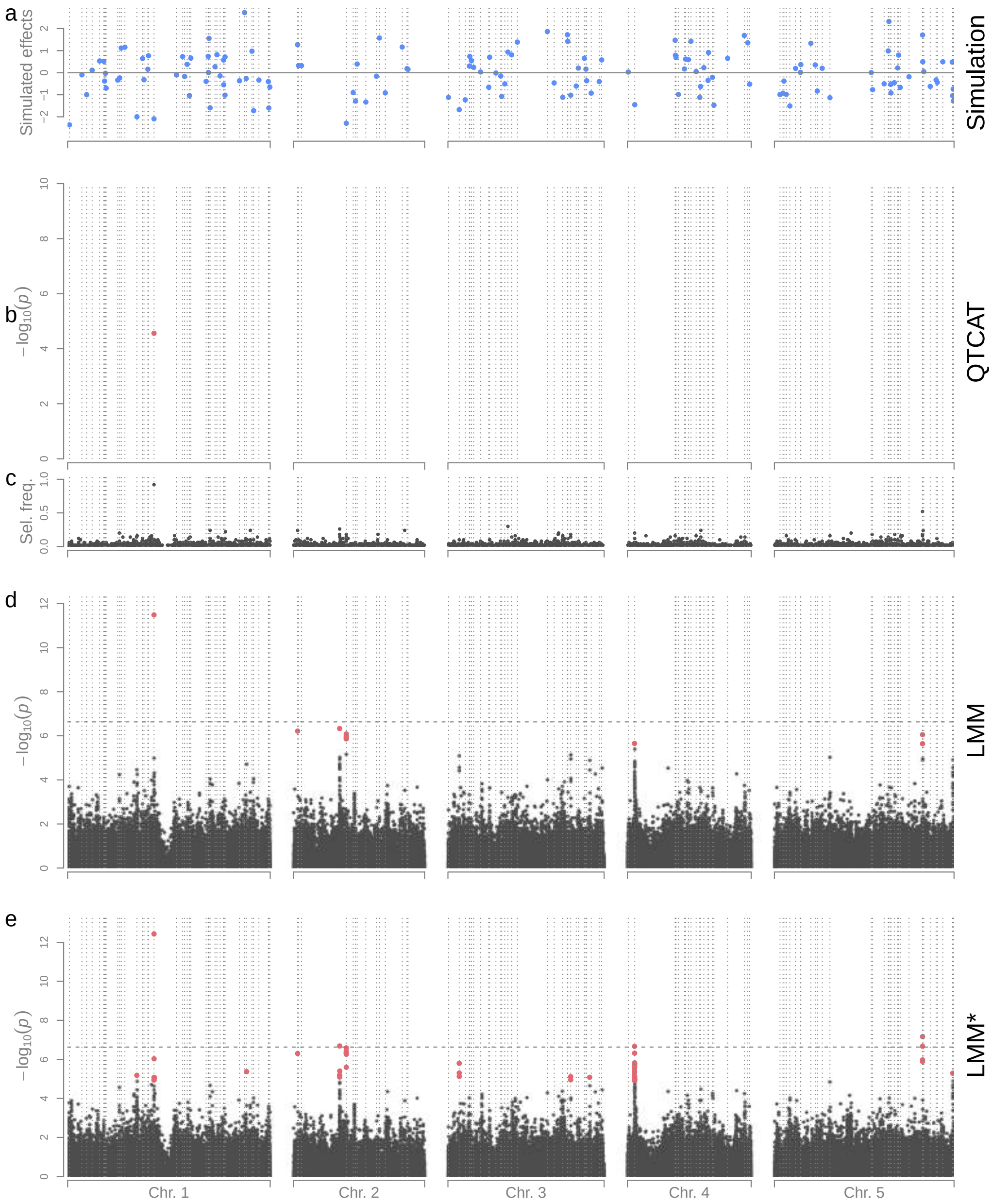

Supplementary Figure 215 Simulation of a GWA analysis based on a structured population with a heritability of 0.4 (run 15). (**a**) Simulation of 150 effects randomly drawn from a normal distribution and assigned to random markers. Markers with effect are highlighted with dashed lines. (**b**) Significant QTCs found by QTCAT. (**c**) LASSO selection frequency for each marker during the 50 iterations of QTCAT. (**d**) Manhattan plot of the LMM analysis. The horizontal dashed line depicts the significance threshold when controlling the multiple testing with FWER, whereas the red markers are significantly associated when controlling with FDR. (**e**) The Manhattan plot of the LMM* analysis. GRM was estimated without markers on the chromosome of the actual testing position. The results are shown as in (d).

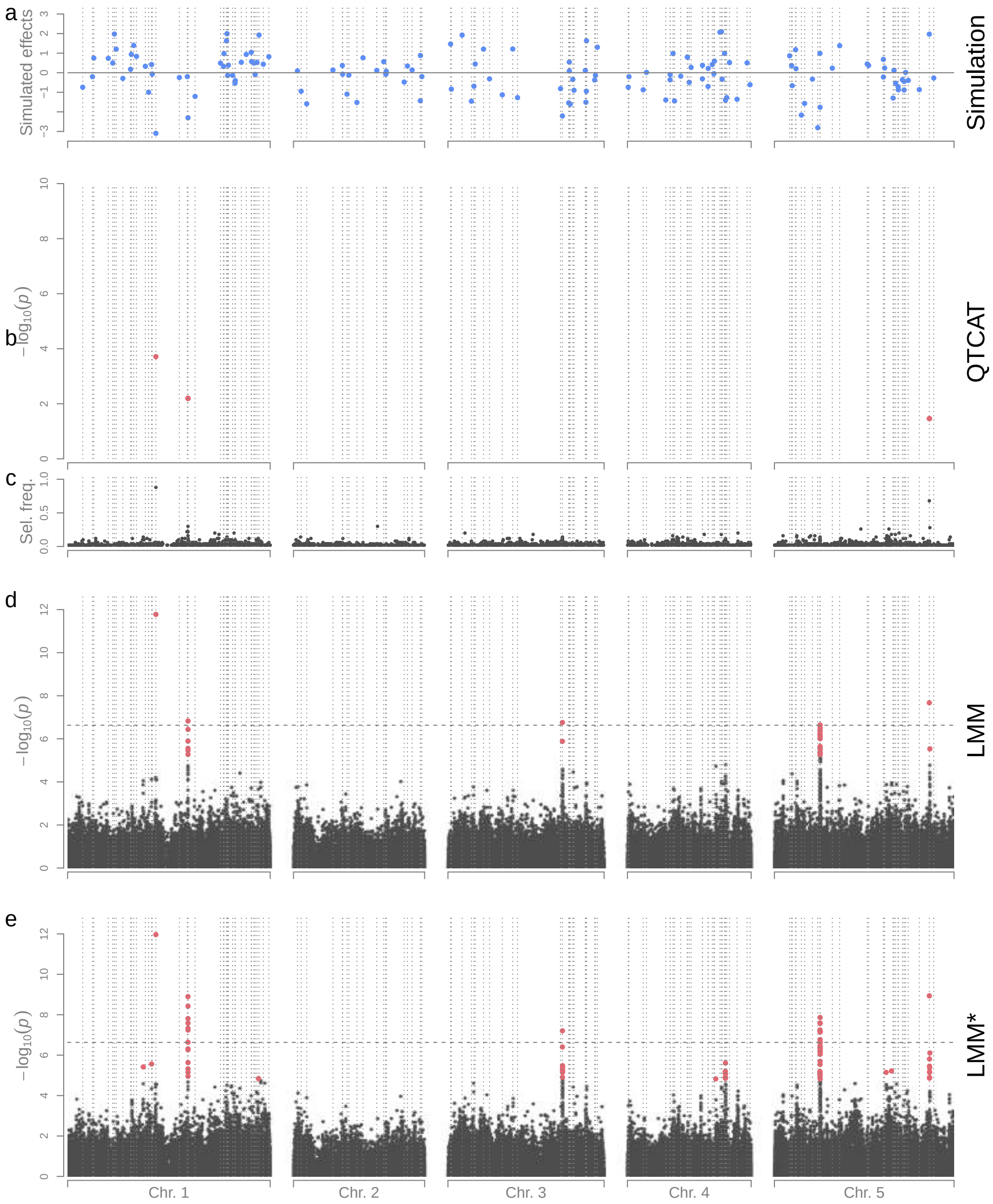

Supplementary Figure 216 Simulation of a GWA analysis based on a structured population with a heritability of 0.4 (run 16). (**a**) Simulation of 150 effects randomly drawn from a normal distribution and assigned to random markers. Markers with effect are highlighted with dashed lines. (**b**) Significant QTCs found by QTCAT. (**c**) LASSO selection frequency for each marker during the 50 iterations of QTCAT. (**d**) Manhattan plot of the LMM analysis. The horizontal dashed line depicts the significance threshold when controlling the multiple testing with FWER, whereas the red markers are significantly associated when controlling with FDR. (**e**) The Manhattan plot of the LMM* analysis. GRM was estimated without markers on the chromosome of the actual testing position. The results are shown as in (d).

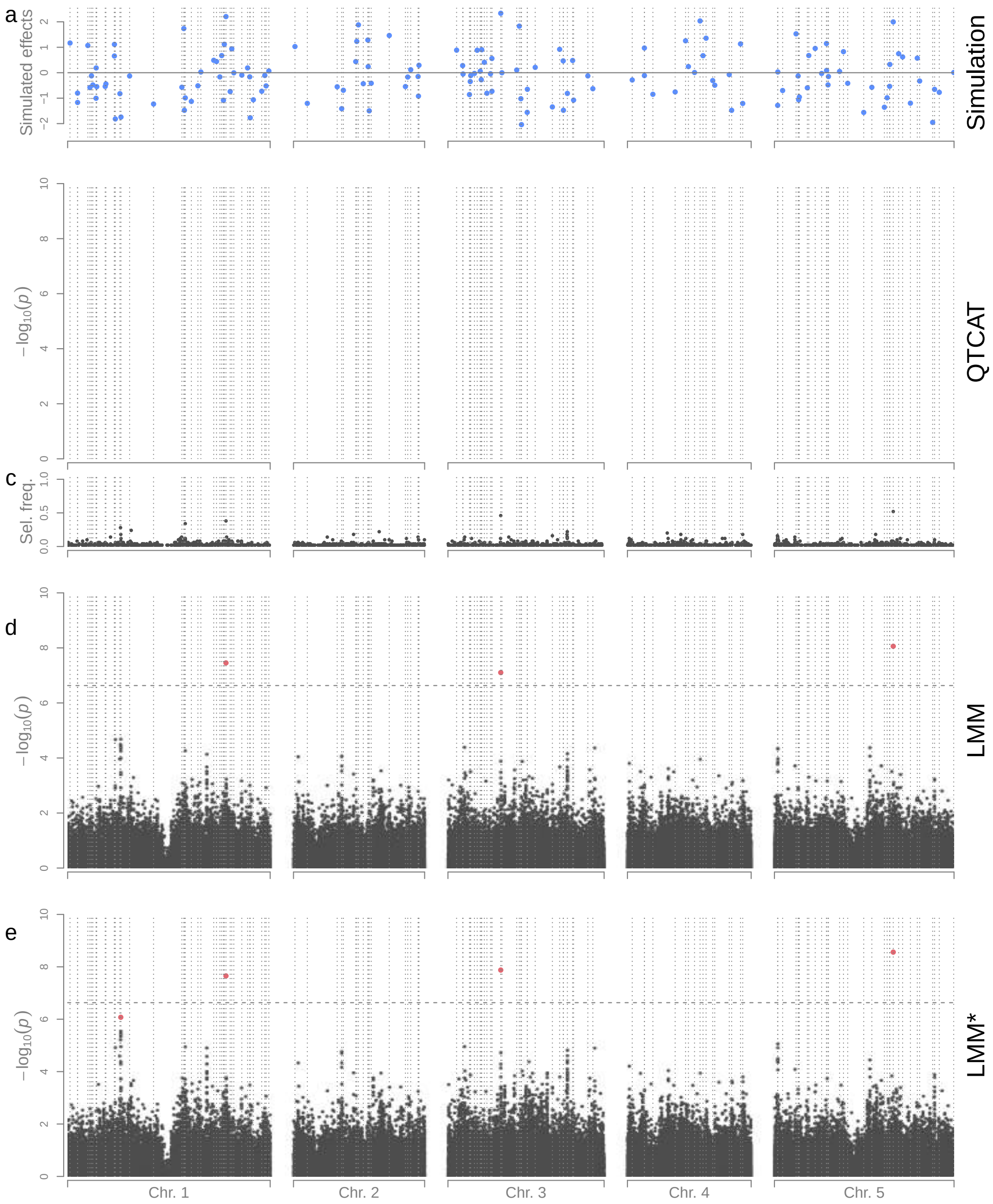

Supplementary Figure 217 Simulation of a GWA analysis based on a structured population with a heritability of 0.4 (run 17). (**a**) Simulation of 150 effects randomly drawn from a normal distribution and assigned to random markers. Markers with effect are highlighted with dashed lines. (**b**) Significant QTCs found by QTCAT. (**c**) LASSO selection frequency for each marker during the 50 iterations of QTCAT. (**d**) Manhattan plot of the LMM analysis. The horizontal dashed line depicts the significance threshold when controlling the multiple testing with FWER, whereas the red markers are significantly associated when controlling with FDR. (**e**) The Manhattan plot of the LMM* analysis. GRM was estimated without markers on the chromosome of the actual testing position. The results are shown as in (d).

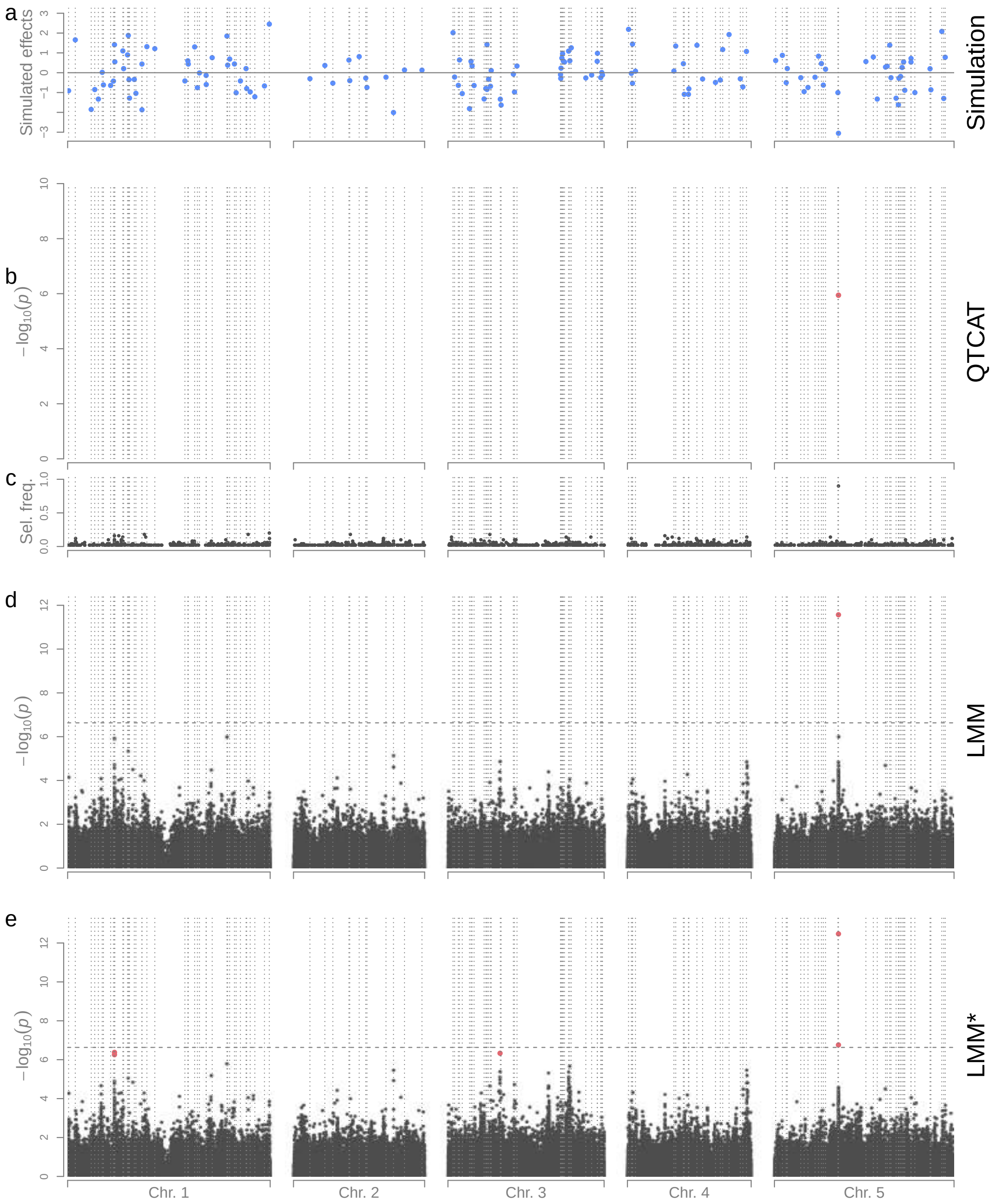

Supplementary Figure 218 Simulation of a GWA analysis based on a structured population with a heritability of 0.4 (run 18). (**a**) Simulation of 150 effects randomly drawn from a normal distribution and assigned to random markers. Markers with effect are highlighted with dashed lines. (**b**) Significant QTCs found by QTCAT. (**c**) LASSO selection frequency for each marker during the 50 iterations of QTCAT. (**d**) Manhattan plot of the LMM analysis. The horizontal dashed line depicts the significance threshold when controlling the multiple testing with FWER, whereas the red markers are significantly associated when controlling with FDR. (**e**) The Manhattan plot of the LMM* analysis. GRM was estimated without markers on the chromosome of the actual testing position. The results are shown as in (d).

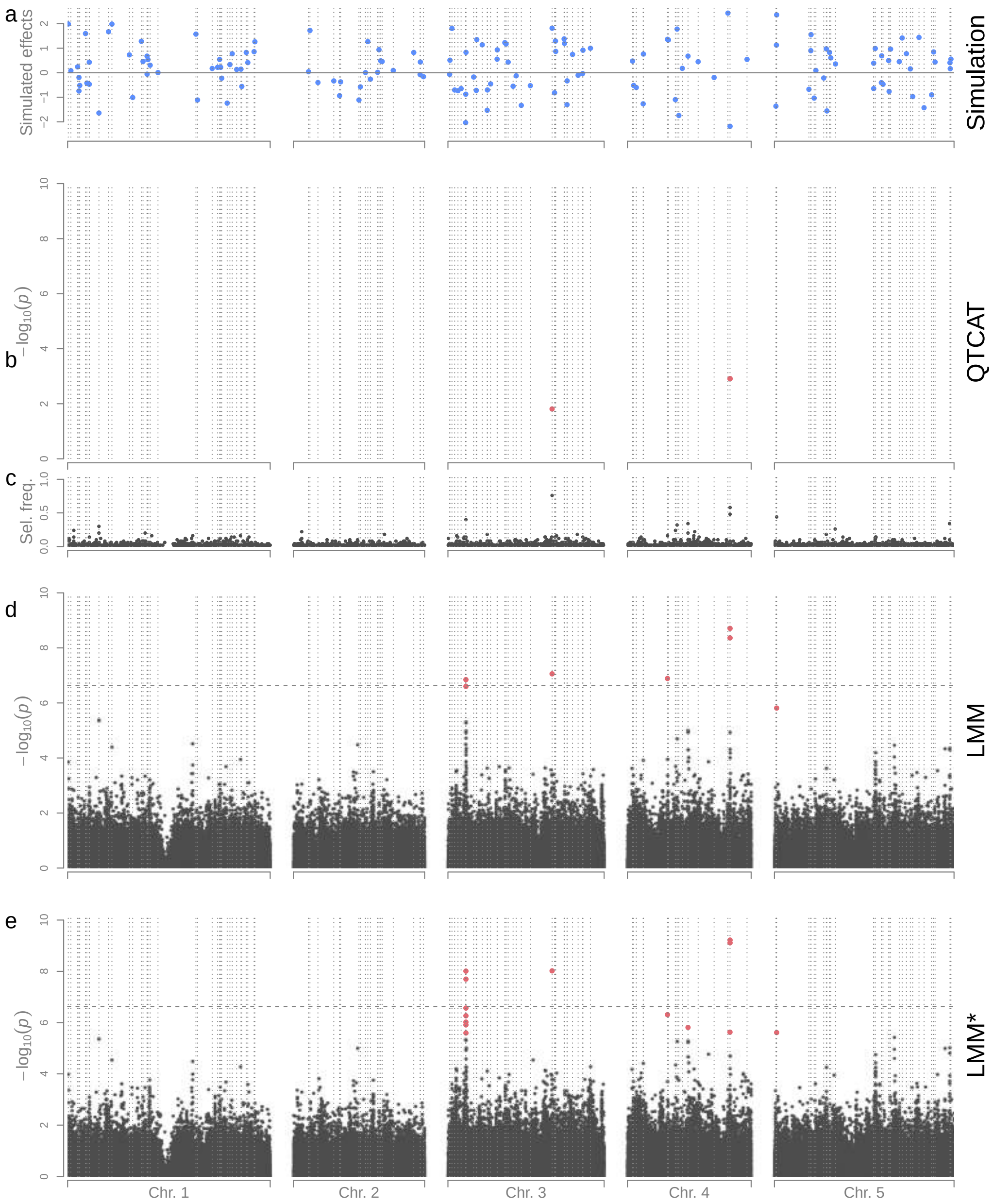

Supplementary Figure 219 Simulation of a GWA analysis based on a structured population with a heritability of 0.4 (run 19). (**a**) Simulation of 150 effects randomly drawn from a normal distribution and assigned to random markers. Markers with effect are highlighted with dashed lines. (**b**) Significant QTCs found by QTCAT. (**c**) LASSO selection frequency for each marker during the 50 iterations of QTCAT. (**d**) Manhattan plot of the LMM analysis. The horizontal dashed line depicts the significance threshold when controlling the multiple testing with FWER, whereas the red markers are significantly associated when controlling with FDR. (**e**) The Manhattan plot of the LMM* analysis. GRM was estimated without markers on the chromosome of the actual testing position. The results are shown as in (d).

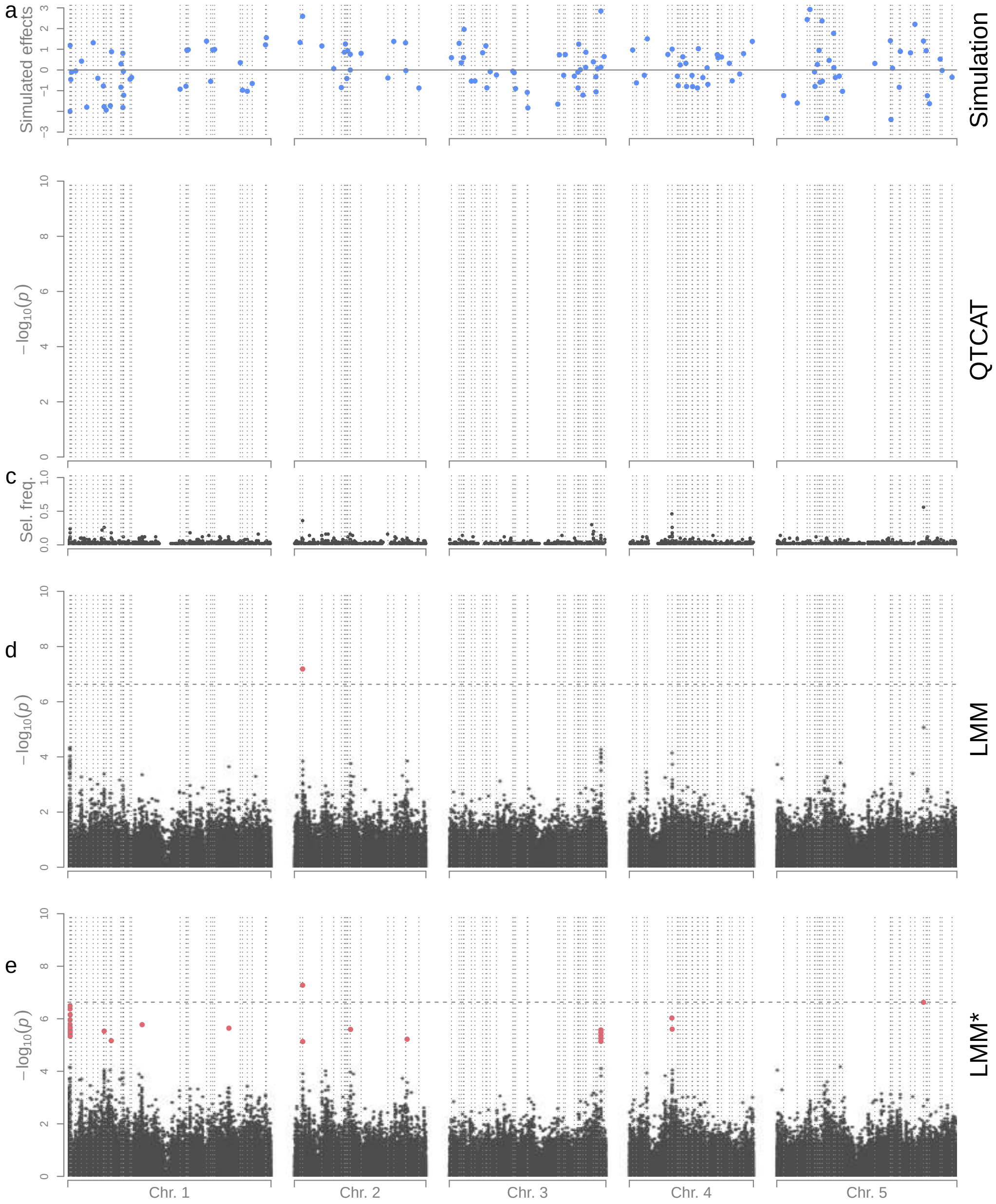

Supplementary Figure 220 Simulation of a GWA analysis based on a structured population with a heritability of 0.4 (run 20). (**a**) Simulation of 150 effects randomly drawn from a normal distribution and assigned to random markers. Markers with effect are highlighted with dashed lines. (**b**) Significant QTCs found by QTCAT. (**c**) LASSO selection frequency for each marker during the 50 iterations of QTCAT. (**d**) Manhattan plot of the LMM analysis. The horizontal dashed line depicts the significance threshold when controlling the multiple testing with FWER, whereas the red markers are significantly associated when controlling with FDR. (**e**) The Manhattan plot of the LMM* analysis. GRM was estimated without markers on the chromosome of the actual testing position. The results are shown as in (d).

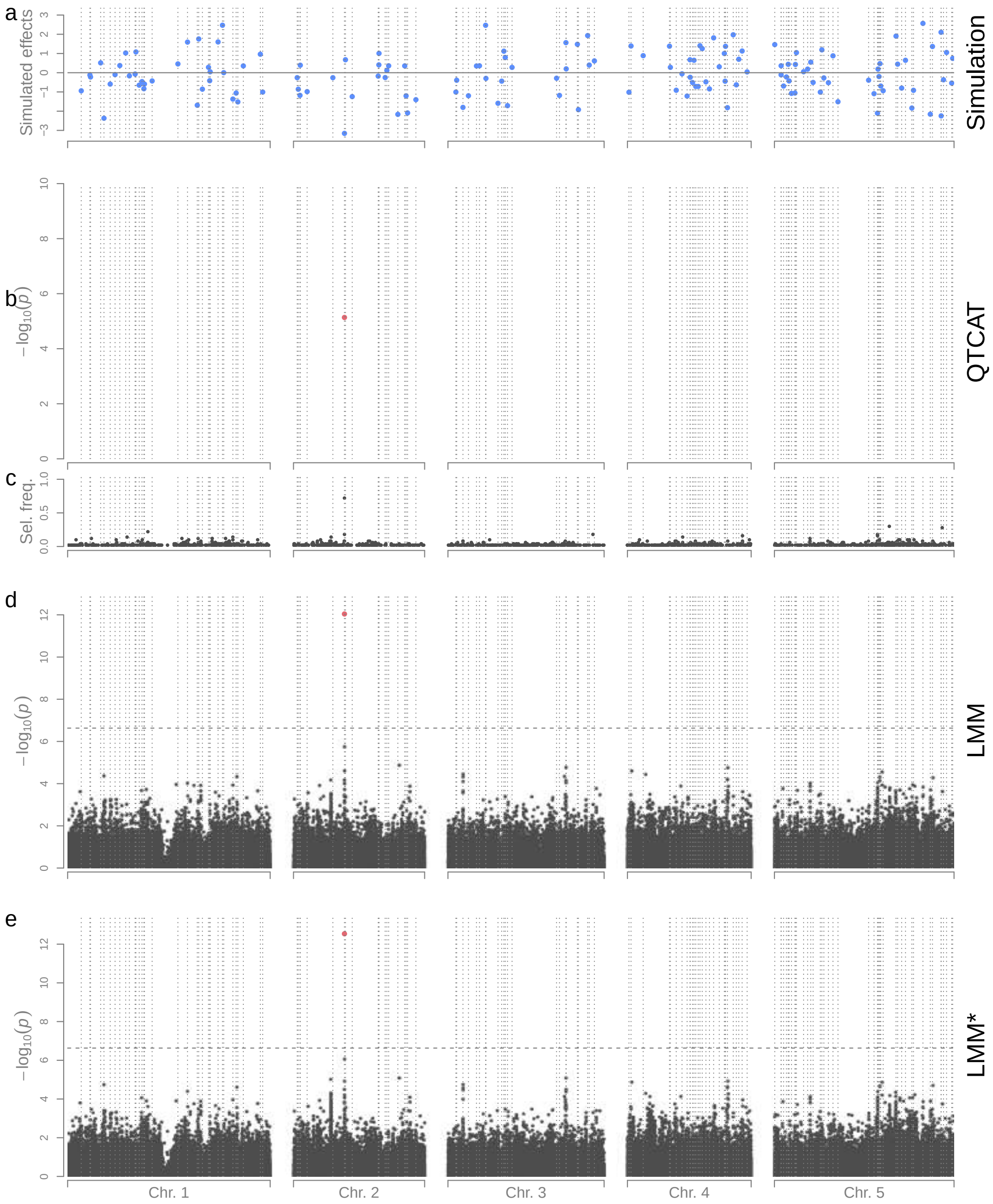

Supplementary Figure 221 Simulation of a GWA analysis based on a structured population with a heritability of 0.4 (run 21). (**a**) Simulation of 150 effects randomly drawn from a normal distribution and assigned to random markers. Markers with effect are highlighted with dashed lines. (**b**) Significant QTCs found by QTCAT. (**c**) LASSO selection frequency for each marker during the 50 iterations of QTCAT. (**d**) Manhattan plot of the LMM analysis. The horizontal dashed line depicts the significance threshold when controlling the multiple testing with FWER, whereas the red markers are significantly associated when controlling with FDR. (**e**) The Manhattan plot of the LMM* analysis. GRM was estimated without markers on the chromosome of the actual testing position. The results are shown as in (d).

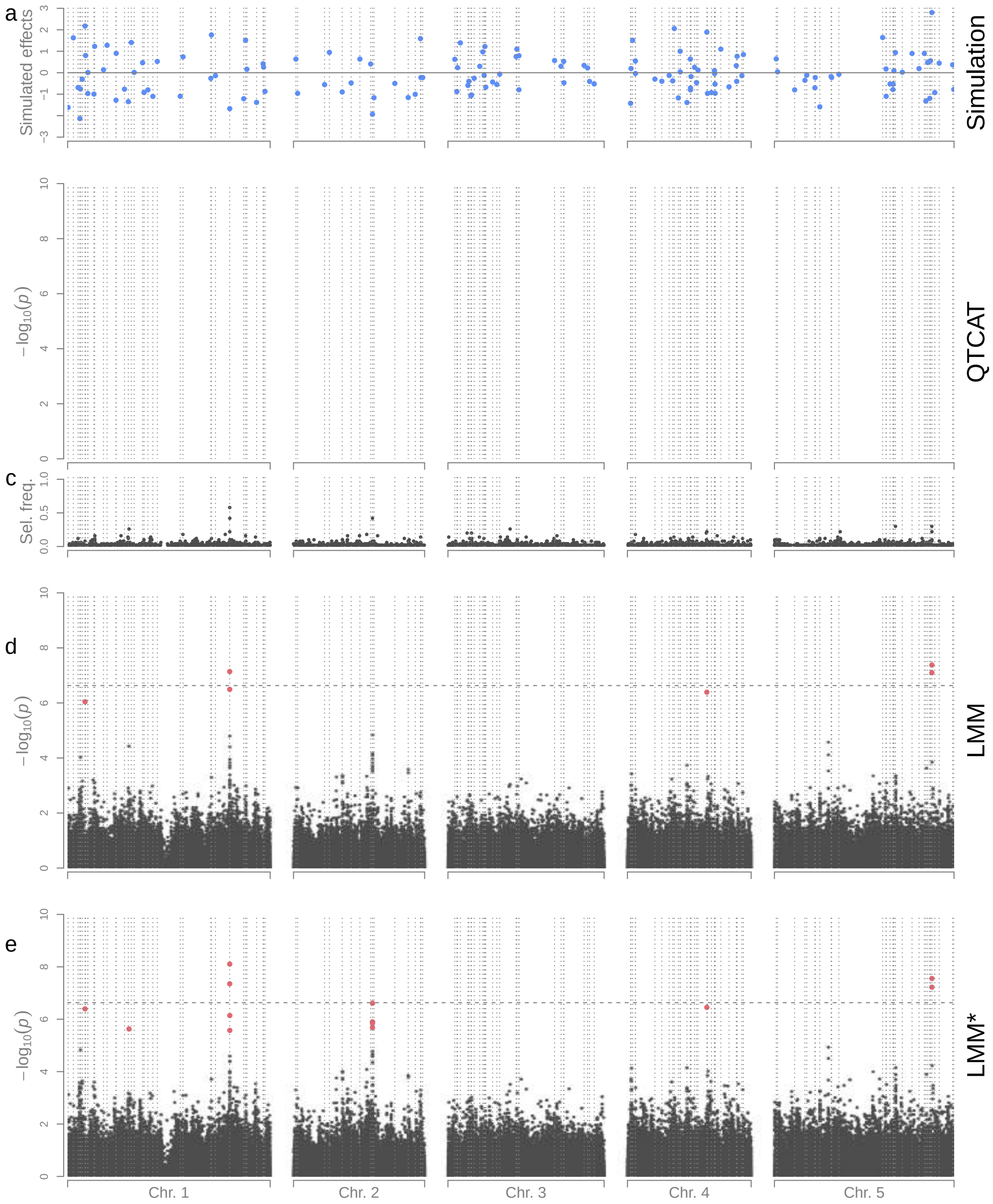

Supplementary Figure 222 Simulation of a GWA analysis based on a structured population with a heritability of 0.4 (run 22). (**a**) Simulation of 150 effects randomly drawn from a normal distribution and assigned to random markers. Markers with effect are highlighted with dashed lines. (**b**) Significant QTCs found by QTCAT. (**c**) LASSO selection frequency for each marker during the 50 iterations of QTCAT. (**d**) Manhattan plot of the LMM analysis. The horizontal dashed line depicts the significance threshold when controlling the multiple testing with FWER, whereas the red markers are significantly associated when controlling with FDR. (**e**) The Manhattan plot of the LMM* analysis. GRM was estimated without markers on the chromosome of the actual testing position. The results are shown as in (d).

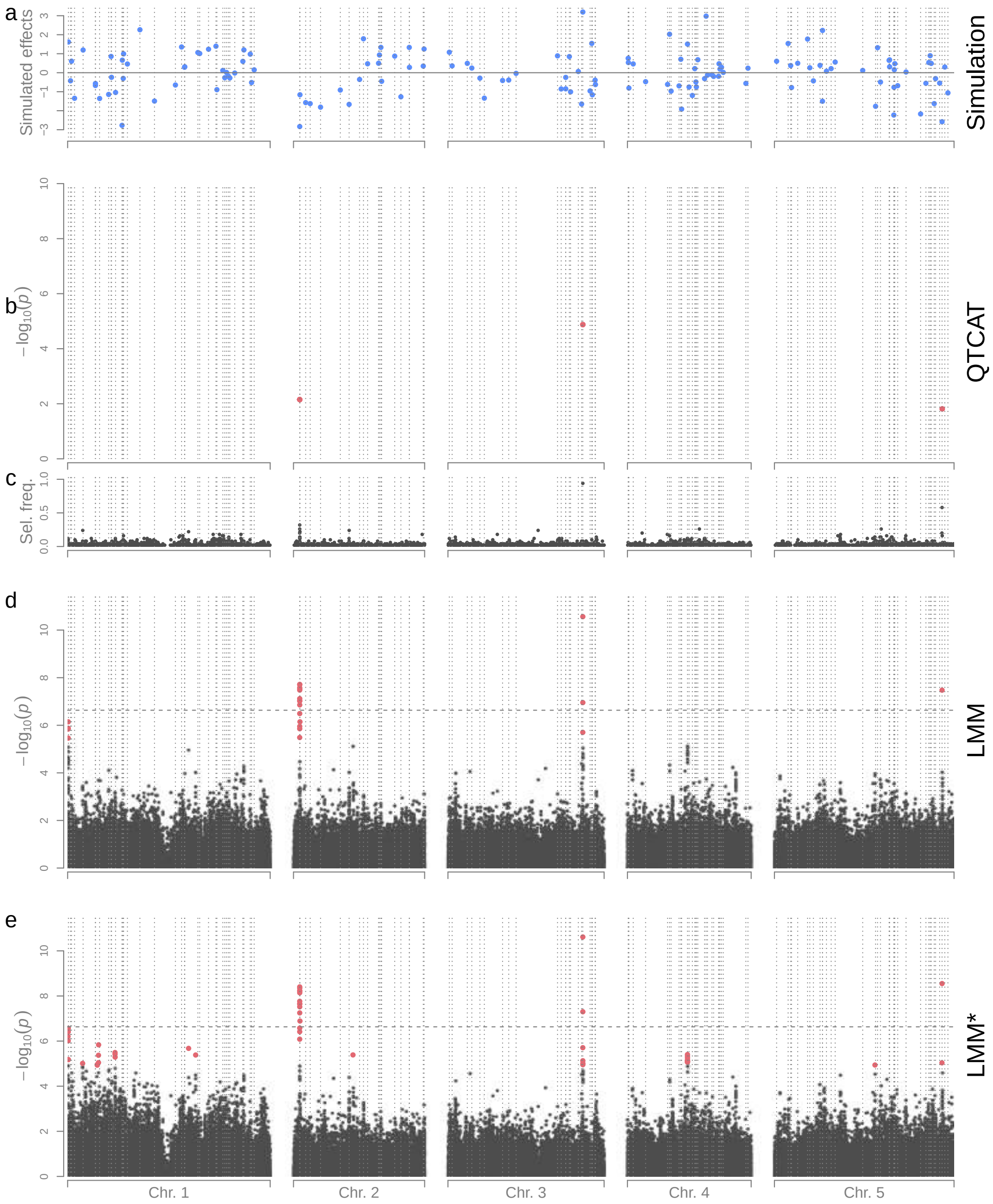

Supplementary Figure 223 Simulation of a GWA analysis based on a structured population with a heritability of 0.4 (run 23). (**a**) Simulation of 150 effects randomly drawn from a normal distribution and assigned to random markers. Markers with effect are highlighted with dashed lines. (**b**) Significant QTCs found by QTCAT. (**c**) LASSO selection frequency for each marker during the 50 iterations of QTCAT. (**d**) Manhattan plot of the LMM analysis. The horizontal dashed line depicts the significance threshold when controlling the multiple testing with FWER, whereas the red markers are significantly associated when controlling with FDR. (**e**) The Manhattan plot of the LMM* analysis. GRM was estimated without markers on the chromosome of the actual testing position. The results are shown as in (d).

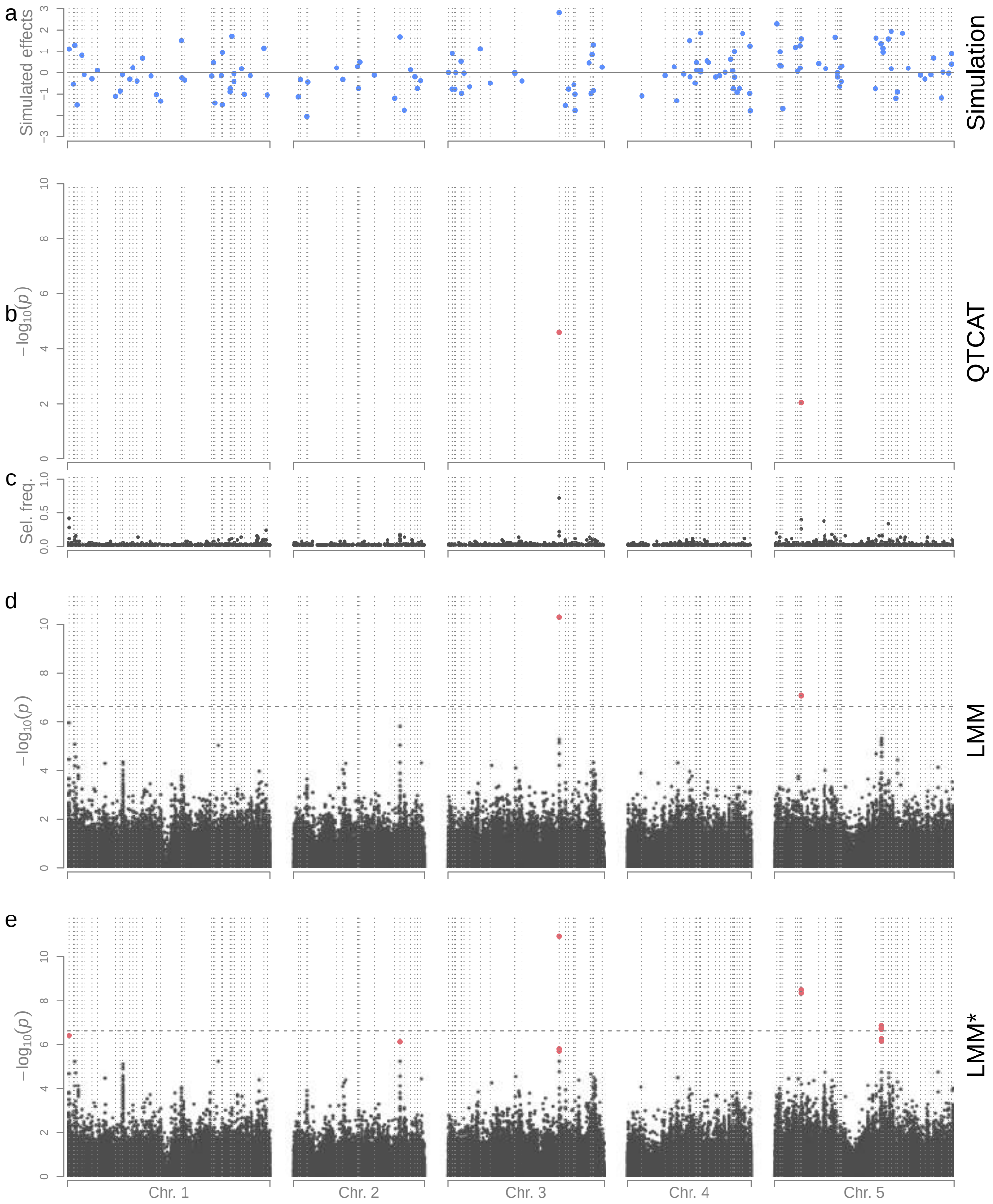

Supplementary Figure 224 Simulation of a GWA analysis based on a structured population with a heritability of 0.4 (run 24). (**a**) Simulation of 150 effects randomly drawn from a normal distribution and assigned to random markers. Markers with effect are highlighted with dashed lines. (**b**) Significant QTCs found by QTCAT. (**c**) LASSO selection frequency for each marker during the 50 iterations of QTCAT. (**d**) Manhattan plot of the LMM analysis. The horizontal dashed line depicts the significance threshold when controlling the multiple testing with FWER, whereas the red markers are significantly associated when controlling with FDR. (**e**) The Manhattan plot of the LMM* analysis. GRM was estimated without markers on the chromosome of the actual testing position. The results are shown as in (d).

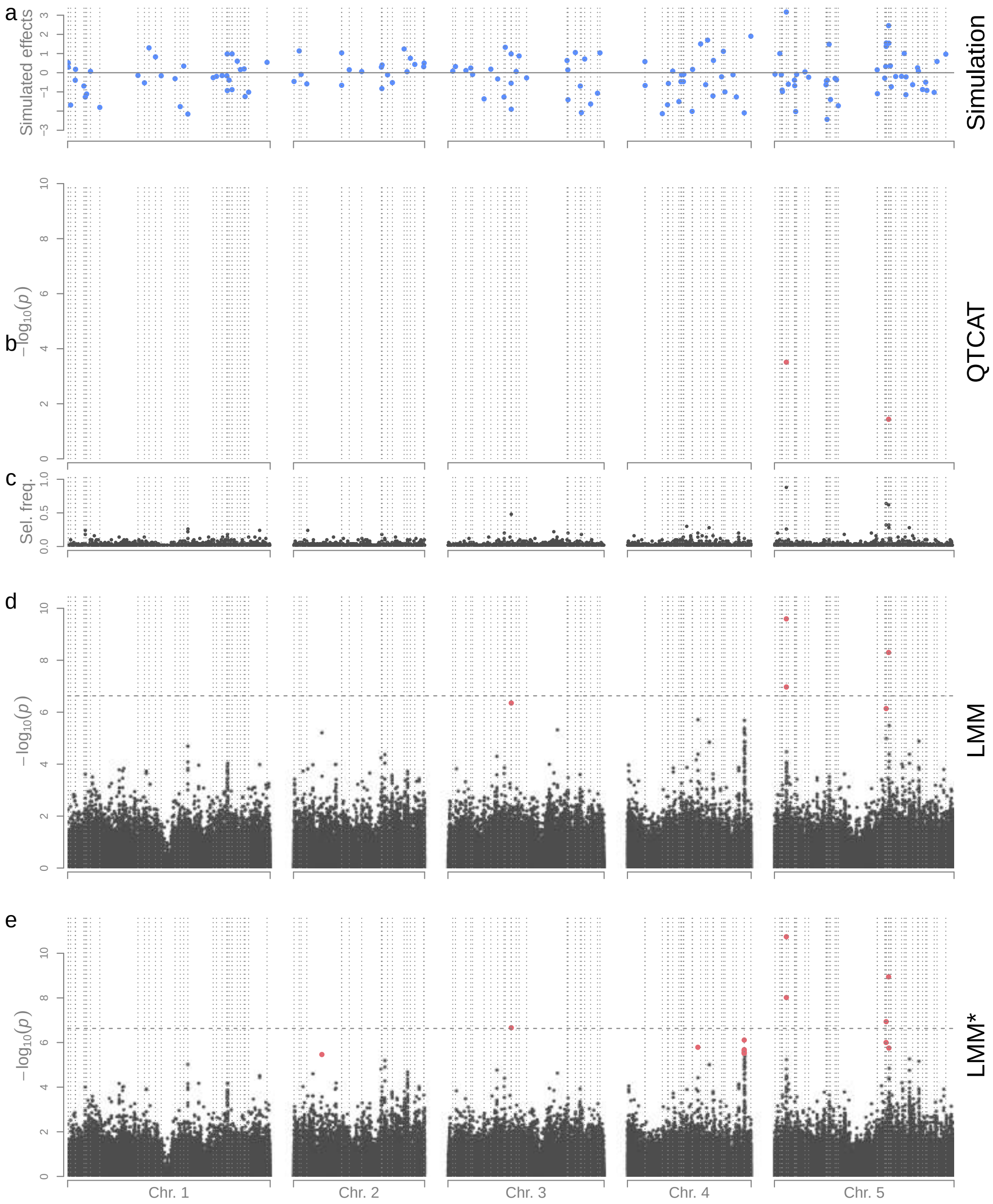

Supplementary Figure 225 Simulation of a GWA analysis based on a structured population with a heritability of 0.4 (run 25). (**a**) Simulation of 150 effects randomly drawn from a normal distribution and assigned to random markers. Markers with effect are highlighted with dashed lines. (**b**) Significant QTCs found by QTCAT. (**c**) LASSO selection frequency for each marker during the 50 iterations of QTCAT. (**d**) Manhattan plot of the LMM analysis. The horizontal dashed line depicts the significance threshold when controlling the multiple testing with FWER, whereas the red markers are significantly associated when controlling with FDR. (**e**) The Manhattan plot of the LMM* analysis. GRM was estimated without markers on the chromosome of the actual testing position. The results are shown as in (d).

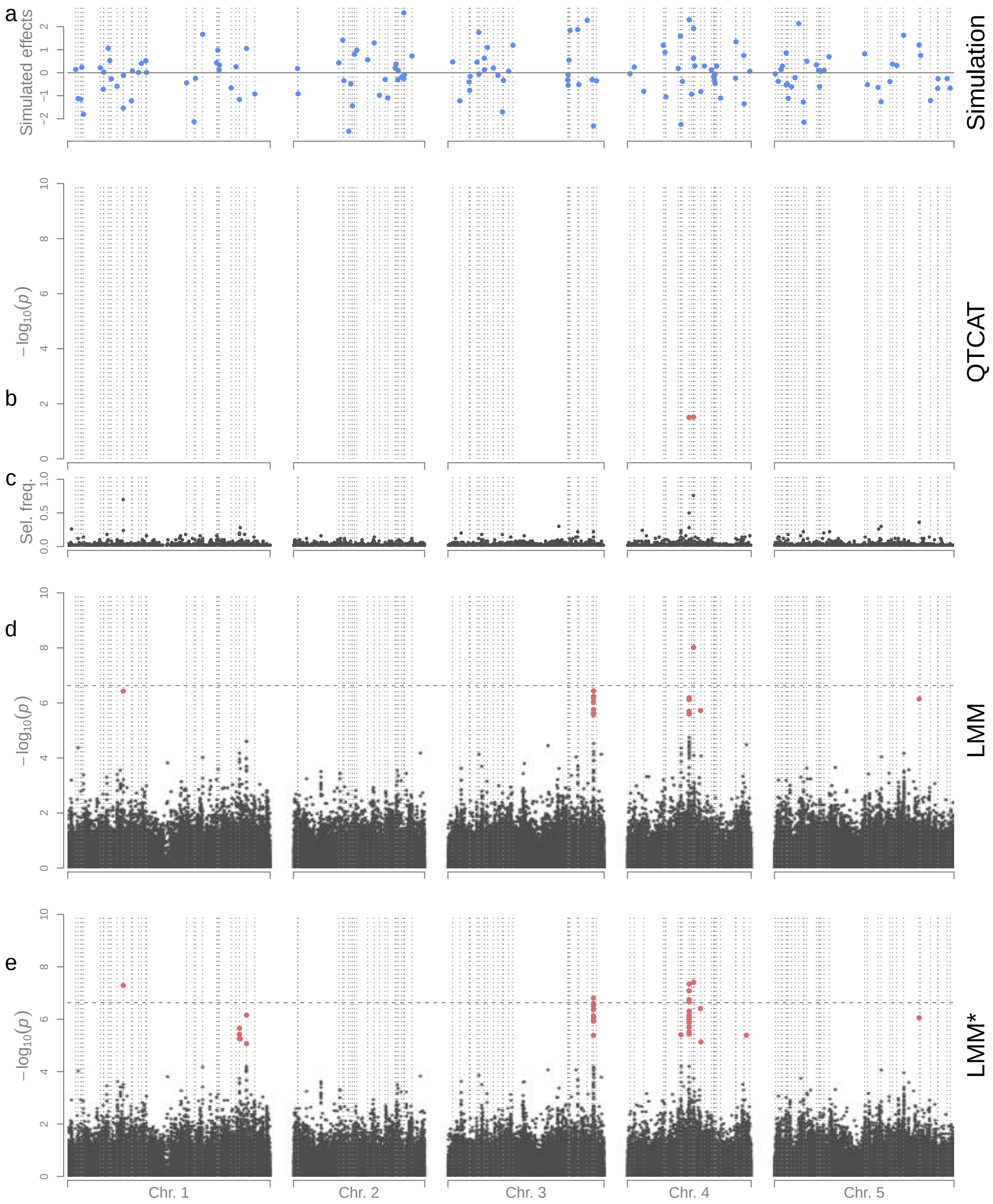

Supplementary Figure 226 Simulation of a GWA analysis based on a structured population with a heritability of 0.4 (run 26). (**a**) Simulation of 150 effects randomly drawn from a normal distribution and assigned to random markers. Markers with effect are highlighted with dashed lines. (**b**) Significant QTCs found by QTCAT. (**c**) LASSO selection frequency for each marker during the 50 iterations of QTCAT. (**d**) Manhattan plot of the LMM analysis. The horizontal dashed line depicts the significance threshold when controlling the multiple testing with FWER, whereas the red markers are significantly associated when controlling with FDR. (**e**) The Manhattan plot of the LMM* analysis. GRM was estimated without markers on the chromosome of the actual testing position. The results are shown as in (d).

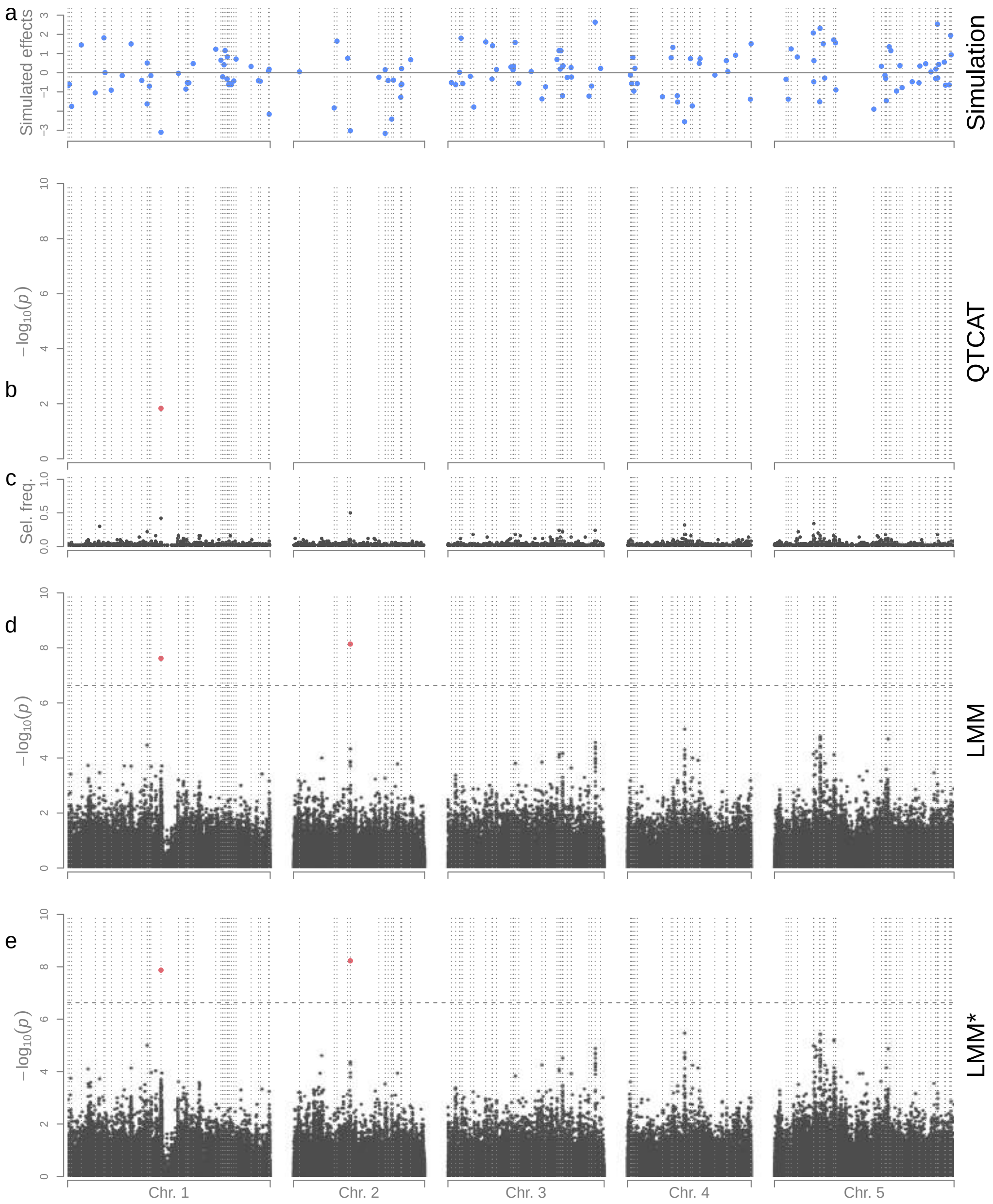

Supplementary Figure 227 Simulation of a GWA analysis based on a structured population with a heritability of 0.4 (run 27). (**a**) Simulation of 150 effects randomly drawn from a normal distribution and assigned to random markers. Markers with effect are highlighted with dashed lines. (**b**) Significant QTCs found by QTCAT. (**c**) LASSO selection frequency for each marker during the 50 iterations of QTCAT. (**d**) Manhattan plot of the LMM analysis. The horizontal dashed line depicts the significance threshold when controlling the multiple testing with FWER, whereas the red markers are significantly associated when controlling with FDR. (**e**) The Manhattan plot of the LMM* analysis. GRM was estimated without markers on the chromosome of the actual testing position. The results are shown as in (d).

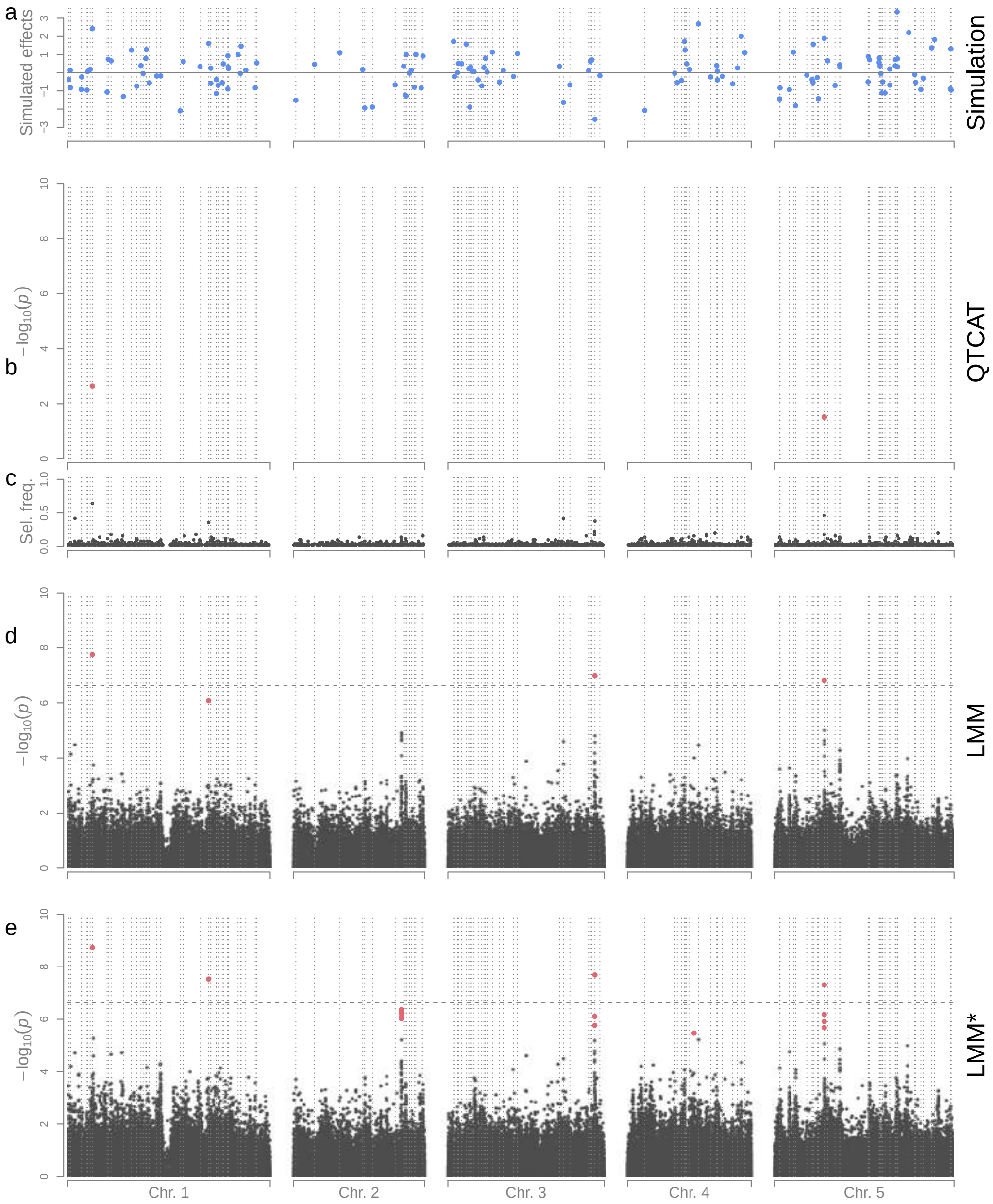

Supplementary Figure 228 Simulation of a GWA analysis based on a structured population with a heritability of 0.4 (run 28). (**a**) Simulation of 150 effects randomly drawn from a normal distribution and assigned to random markers. Markers with effect are highlighted with dashed lines. (**b**) Significant QTCs found by QTCAT. (**c**) LASSO selection frequency for each marker during the 50 iterations of QTCAT. (**d**) Manhattan plot of the LMM analysis. The horizontal dashed line depicts the significance threshold when controlling the multiple testing with FWER, whereas the red markers are significantly associated when controlling with FDR. (**e**) The Manhattan plot of the LMM* analysis. GRM was estimated without markers on the chromosome of the actual testing position. The results are shown as in (d).

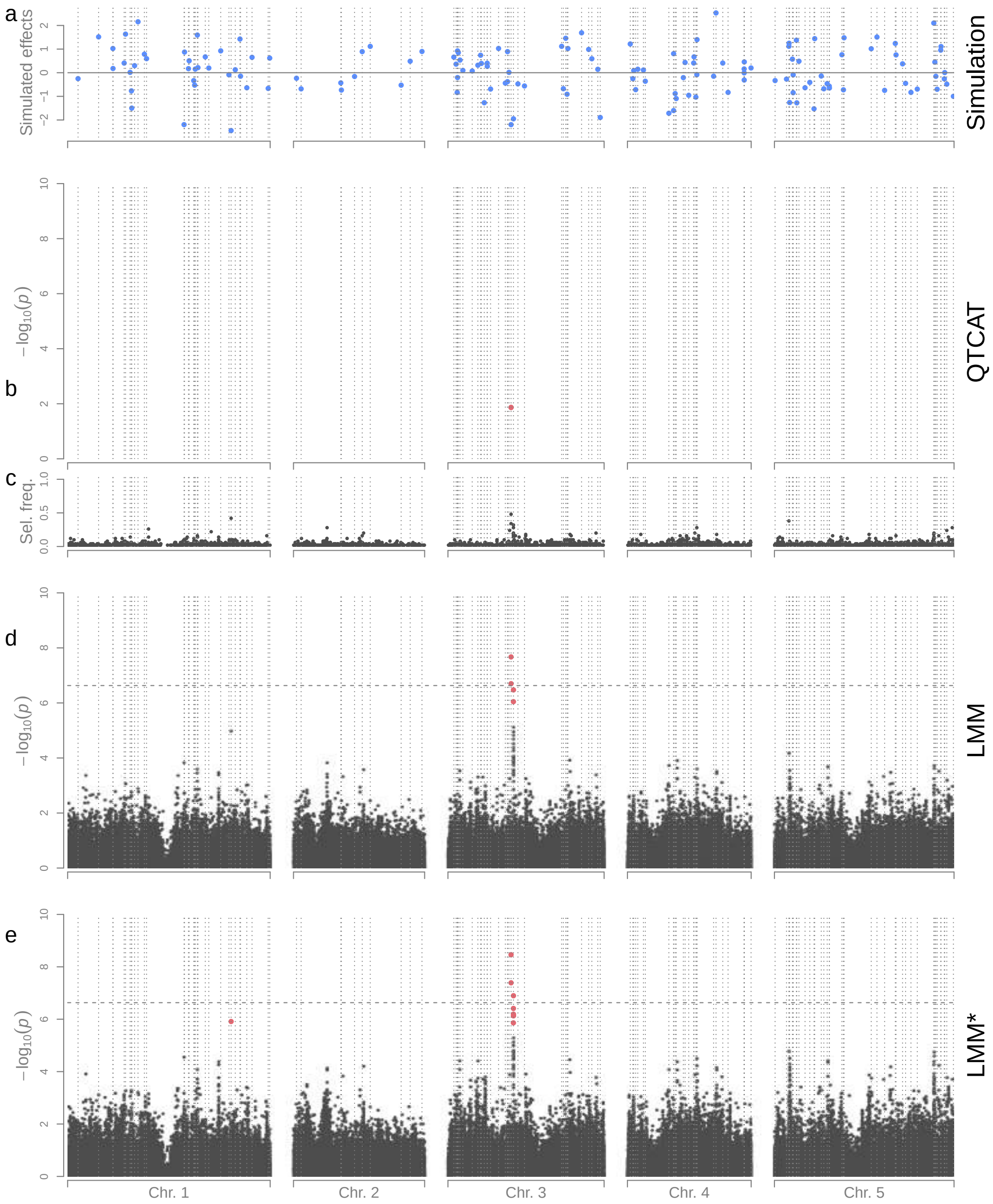

Supplementary Figure 229 Simulation of a GWA analysis based on a structured population with a heritability of 0.4 (run 29). (**a**) Simulation of 150 effects randomly drawn from a normal distribution and assigned to random markers. Markers with effect are highlighted with dashed lines. (**b**) Significant QTCs found by QTCAT. (**c**) LASSO selection frequency for each marker during the 50 iterations of QTCAT. (**d**) Manhattan plot of the LMM analysis. The horizontal dashed line depicts the significance threshold when controlling the multiple testing with FWER, whereas the red markers are significantly associated when controlling with FDR. (**e**) The Manhattan plot of the LMM* analysis. GRM was estimated without markers on the chromosome of the actual testing position. The results are shown as in (d).

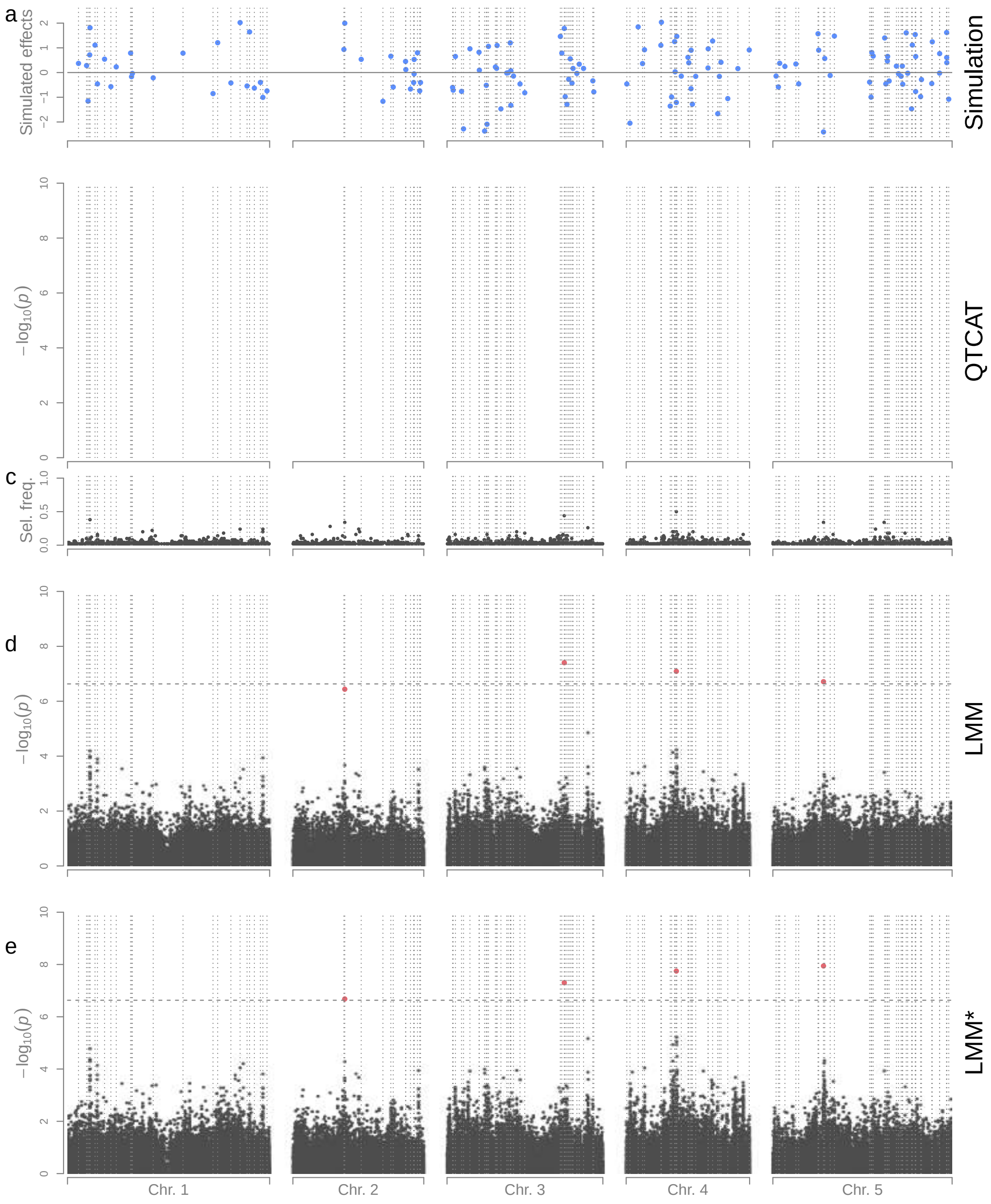

Supplementary Figure 230 Simulation of a GWA analysis based on a structured population with a heritability of 0.4 (run 30). (**a**) Simulation of 150 effects randomly drawn from a normal distribution and assigned to random markers. Markers with effect are highlighted with dashed lines. (**b**) Significant QTCs found by QTCAT. (**c**) LASSO selection frequency for each marker during the 50 iterations of QTCAT. (**d**) Manhattan plot of the LMM analysis. The horizontal dashed line depicts the significance threshold when controlling the multiple testing with FWER, whereas the red markers are significantly associated when controlling with FDR. (**e**) The Manhattan plot of the LMM* analysis. GRM was estimated without markers on the chromosome of the actual testing position. The results are shown as in (d).

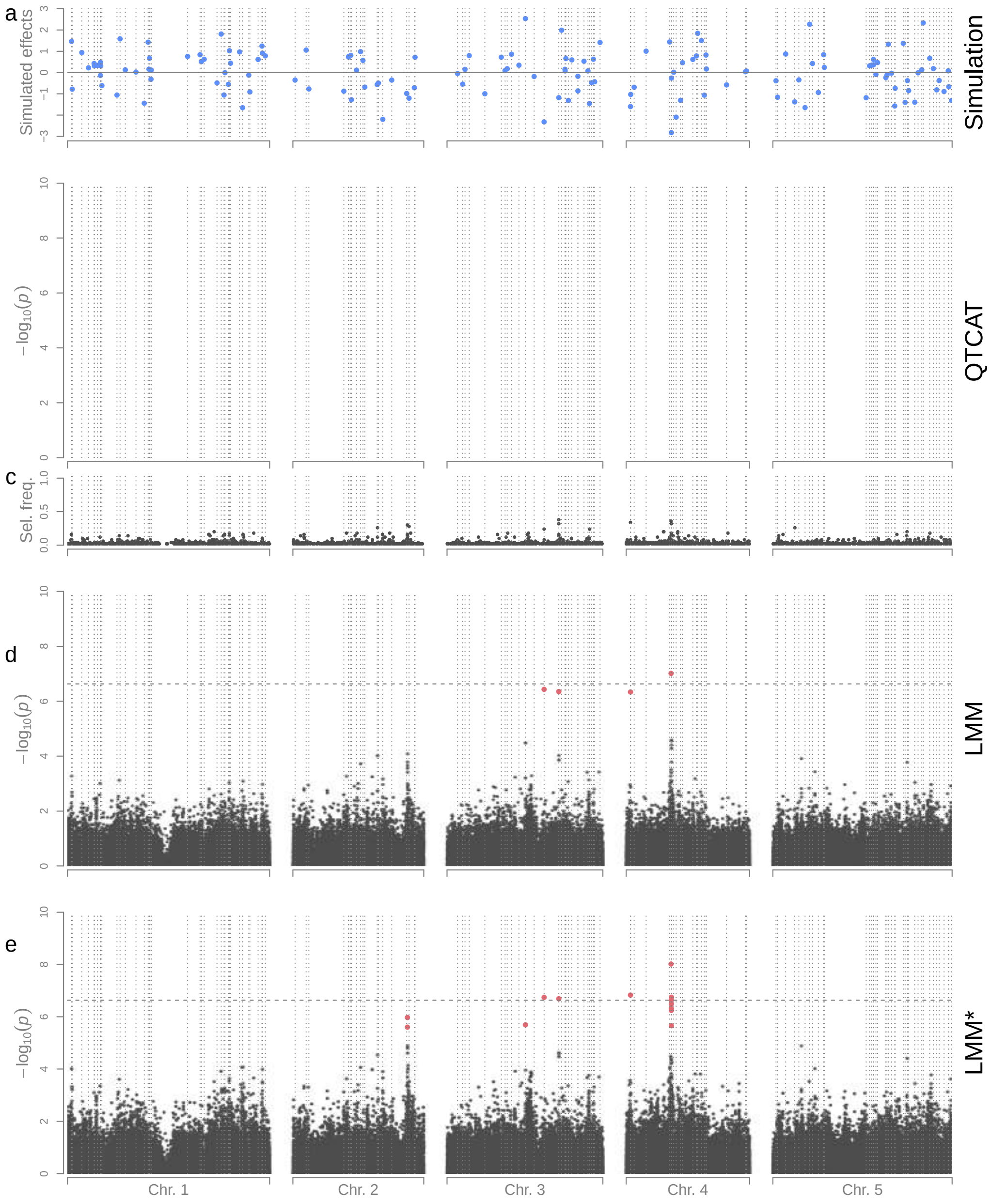

Supplementary Figure 231 Simulation of a GWA analysis based on a structured population with a heritability of 0.4 (run 31). (**a**) Simulation of 150 effects randomly drawn from a normal distribution and assigned to random markers. Markers with effect are highlighted with dashed lines. (**b**) Significant QTCs found by QTCAT. (**c**) LASSO selection frequency for each marker during the 50 iterations of QTCAT. (**d**) Manhattan plot of the LMM analysis. The horizontal dashed line depicts the significance threshold when controlling the multiple testing with FWER, whereas the red markers are significantly associated when controlling with FDR. (**e**) The Manhattan plot of the LMM* analysis. GRM was estimated without markers on the chromosome of the actual testing position. The results are shown as in (d).

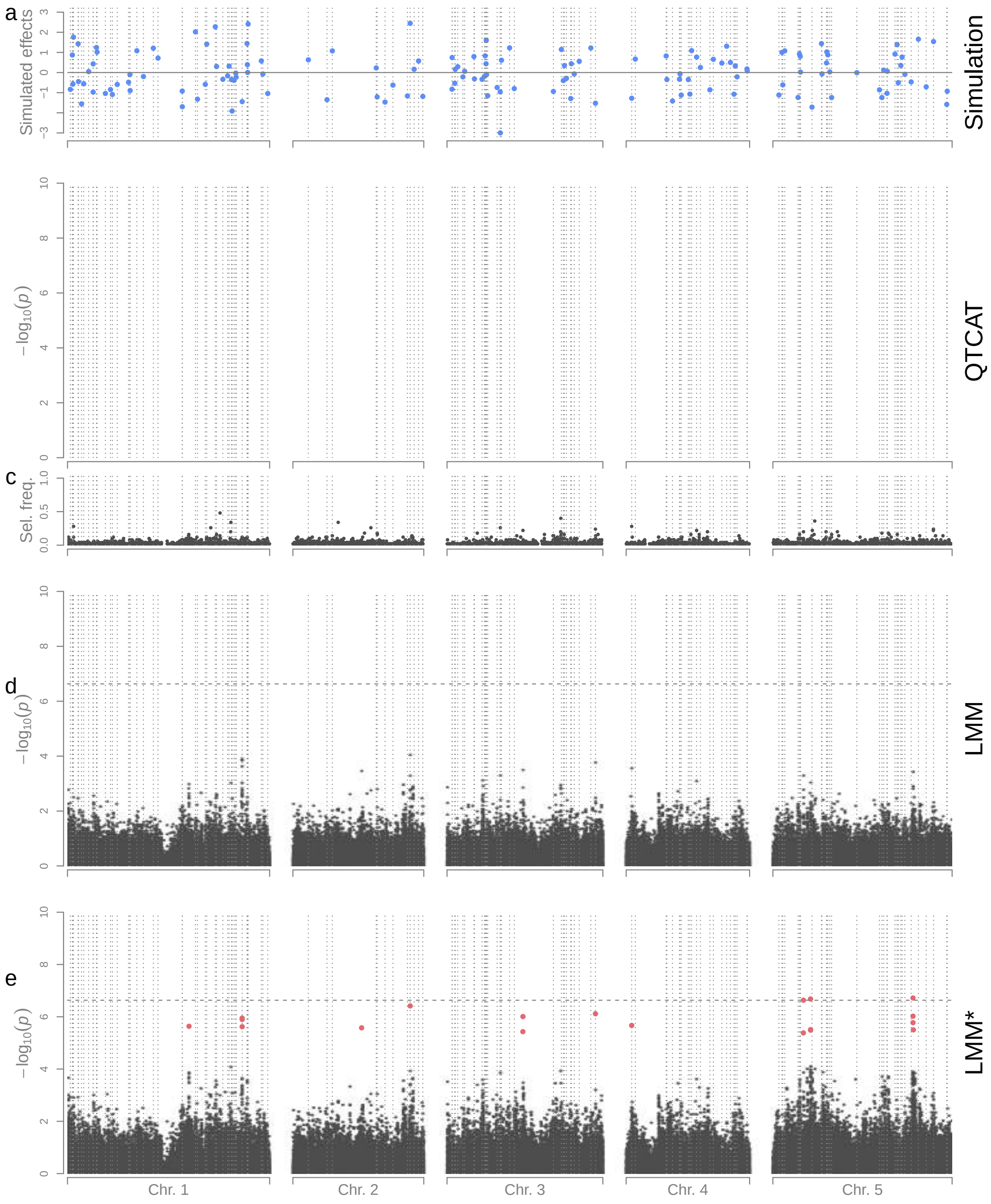

Supplementary Figure 232 Simulation of a GWA analysis based on a structured population with a heritability of 0.4 (run 32). (**a**) Simulation of 150 effects randomly drawn from a normal distribution and assigned to random markers. Markers with effect are highlighted with dashed lines. (**b**) Significant QTCs found by QTCAT. (**c**) LASSO selection frequency for each marker during the 50 iterations of QTCAT. (**d**) Manhattan plot of the LMM analysis. The horizontal dashed line depicts the significance threshold when controlling the multiple testing with FWER, whereas the red markers are significantly associated when controlling with FDR. (**e**) The Manhattan plot of the LMM* analysis. GRM was estimated without markers on the chromosome of the actual testing position. The results are shown as in (d).

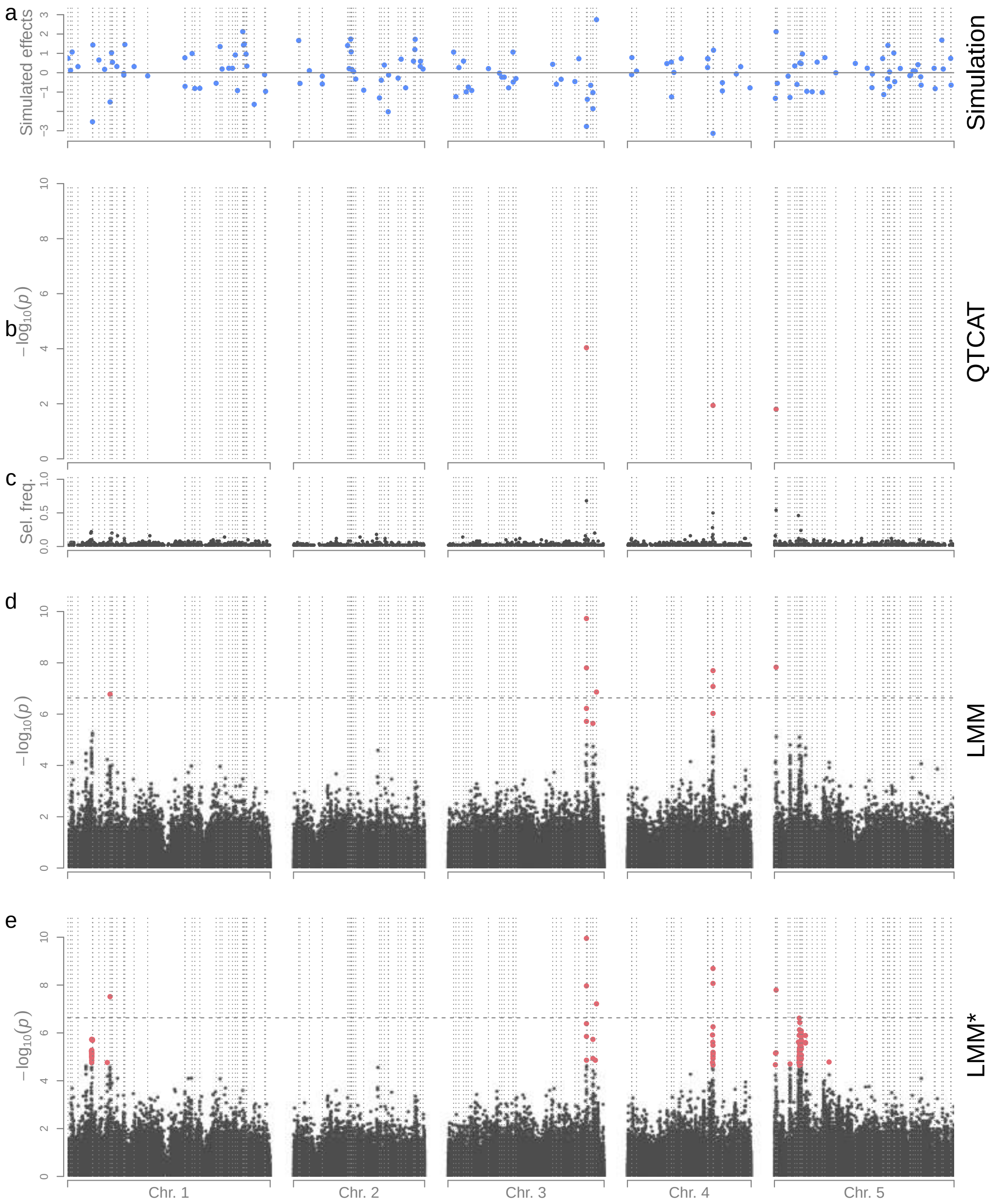

Supplementary Figure 233 Simulation of a GWA analysis based on a structured population with a heritability of 0.4 (run 33). (**a**) Simulation of 150 effects randomly drawn from a normal distribution and assigned to random markers. Markers with effect are highlighted with dashed lines. (**b**) Significant QTCs found by QTCAT. (**c**) LASSO selection frequency for each marker during the 50 iterations of QTCAT. (**d**) Manhattan plot of the LMM analysis. The horizontal dashed line depicts the significance threshold when controlling the multiple testing with FWER, whereas the red markers are significantly associated when controlling with FDR. (**e**) The Manhattan plot of the LMM* analysis. GRM was estimated without markers on the chromosome of the actual testing position. The results are shown as in (d).

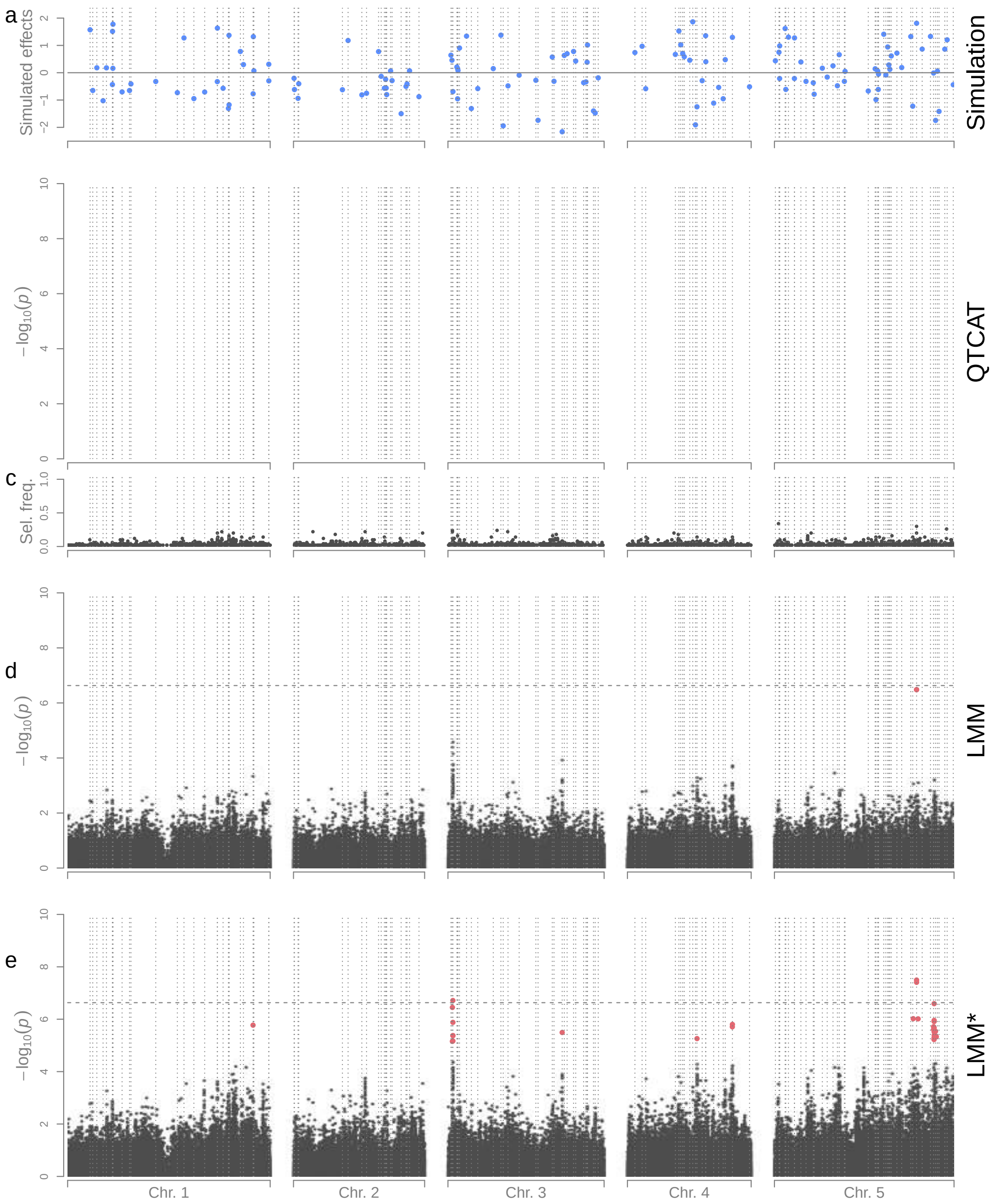

Supplementary Figure 234 Simulation of a GWA analysis based on a structured population with a heritability of 0.4 (run 34). (**a**) Simulation of 150 effects randomly drawn from a normal distribution and assigned to random markers. Markers with effect are highlighted with dashed lines. (**b**) Significant QTCs found by QTCAT. (**c**) LASSO selection frequency for each marker during the 50 iterations of QTCAT. (**d**) Manhattan plot of the LMM analysis. The horizontal dashed line depicts the significance threshold when controlling the multiple testing with FWER, whereas the red markers are significantly associated when controlling with FDR. (**e**) The Manhattan plot of the LMM* analysis. GRM was estimated without markers on the chromosome of the actual testing position. The results are shown as in (d).

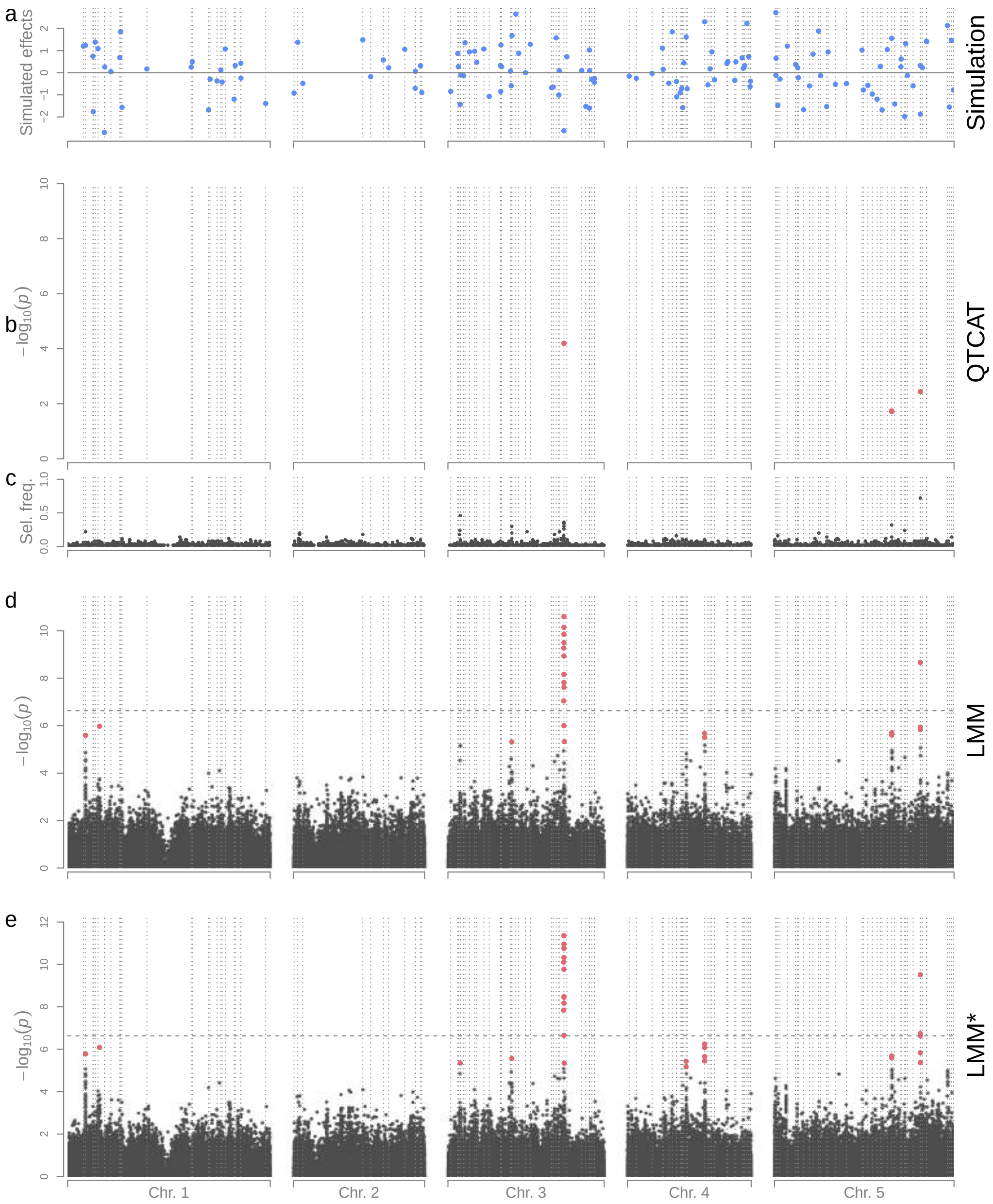

Supplementary Figure 235 Simulation of a GWA analysis based on a structured population with a heritability of 0.4 (run 35). (**a**) Simulation of 150 effects randomly drawn from a normal distribution and assigned to random markers. Markers with effect are highlighted with dashed lines. (**b**) Significant QTCs found by QTCAT. (**c**) LASSO selection frequency for each marker during the 50 iterations of QTCAT. (**d**) Manhattan plot of the LMM analysis. The horizontal dashed line depicts the significance threshold when controlling the multiple testing with FWER, whereas the red markers are significantly associated when controlling with FDR. (**e**) The Manhattan plot of the LMM* analysis. GRM was estimated without markers on the chromosome of the actual testing position. The results are shown as in (d).

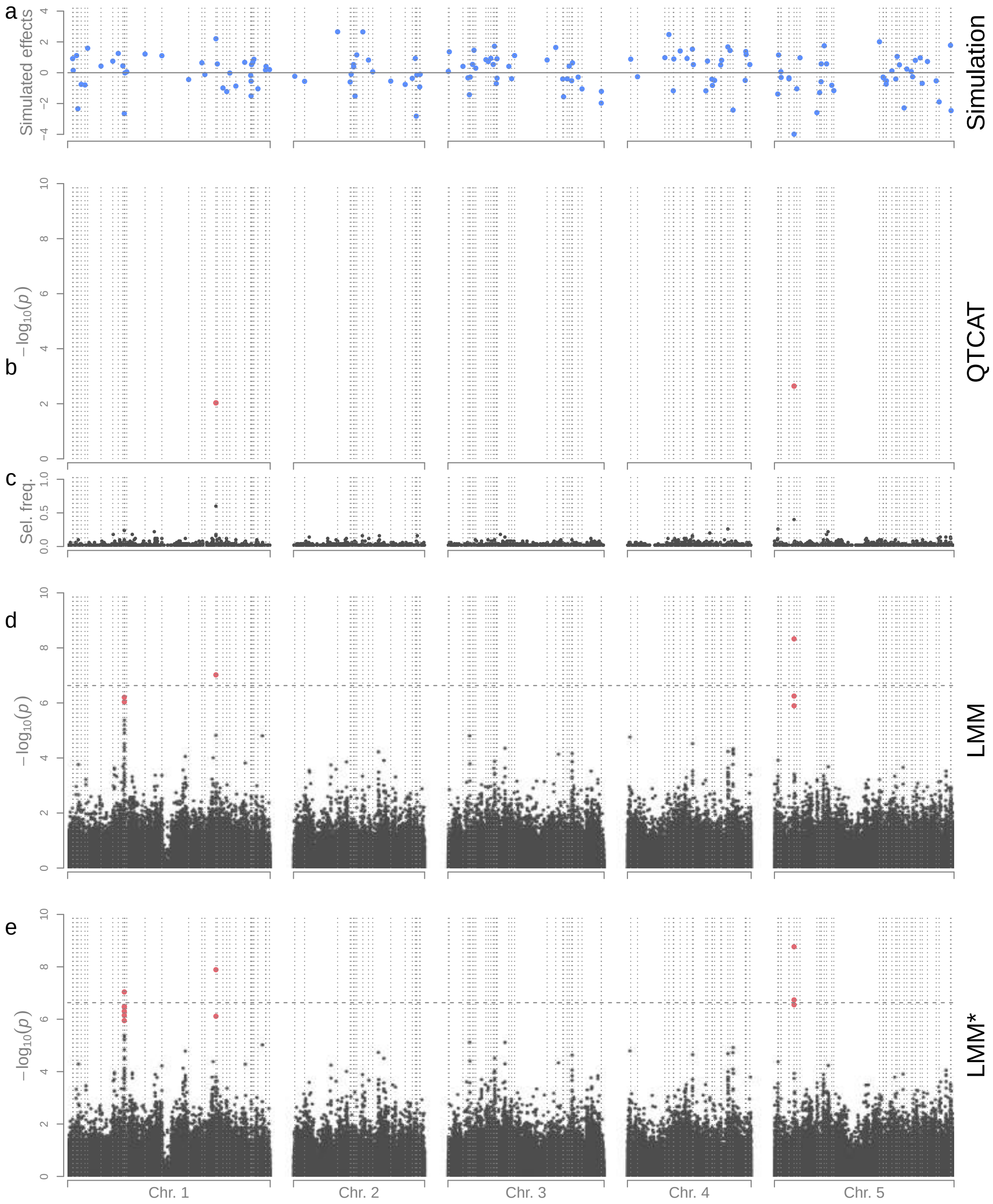

Supplementary Figure 236 Simulation of a GWA analysis based on a structured population with a heritability of 0.4 (run 36). (**a**) Simulation of 150 effects randomly drawn from a normal distribution and assigned to random markers. Markers with effect are highlighted with dashed lines. (**b**) Significant QTCs found by QTCAT. (**c**) LASSO selection frequency for each marker during the 50 iterations of QTCAT. (**d**) Manhattan plot of the LMM analysis. The horizontal dashed line depicts the significance threshold when controlling the multiple testing with FWER, whereas the red markers are significantly associated when controlling with FDR. (**e**) The Manhattan plot of the LMM* analysis. GRM was estimated without markers on the chromosome of the actual testing position. The results are shown as in (d).

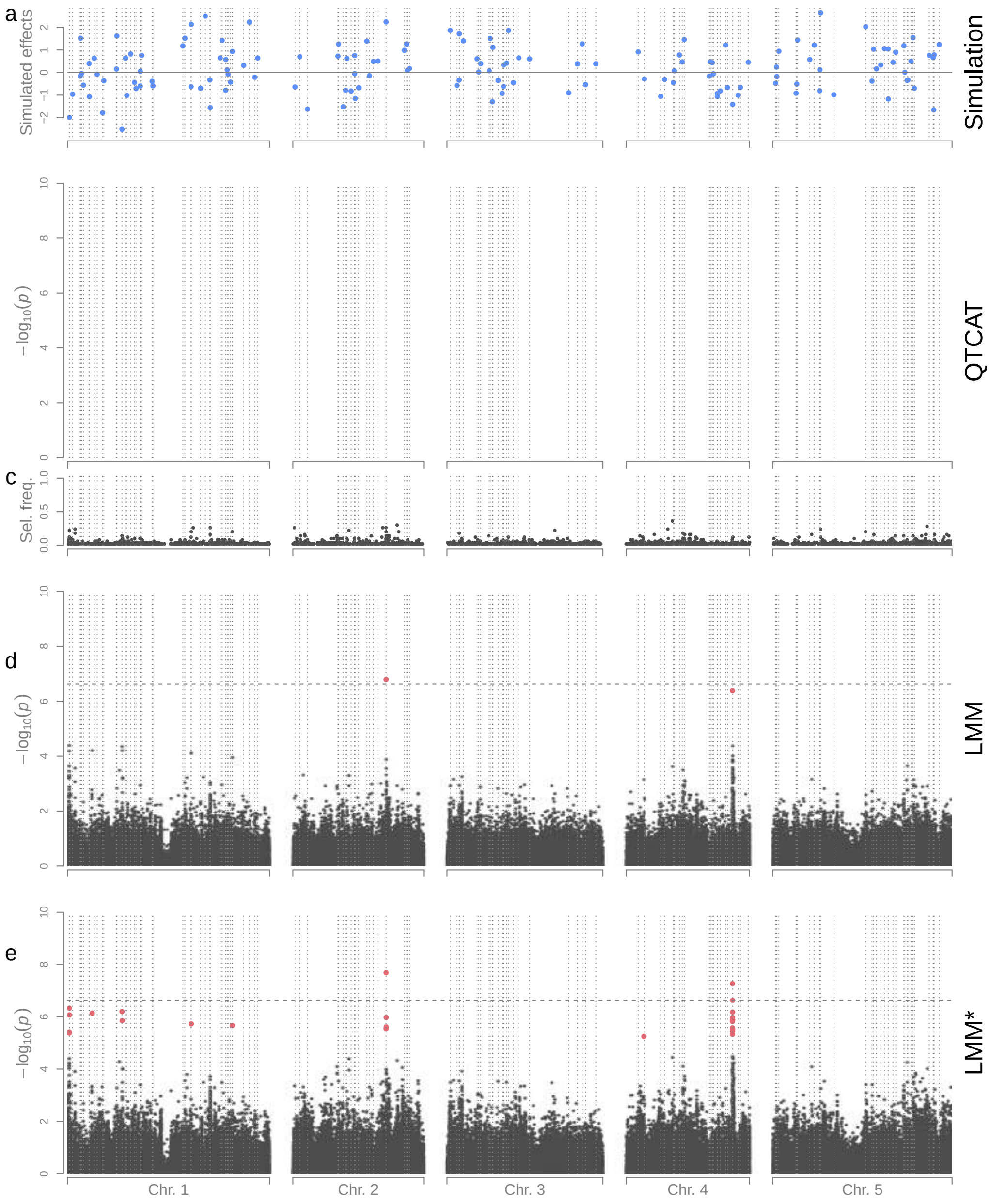

Supplementary Figure 237 Simulation of a GWA analysis based on a structured population with a heritability of 0.4 (run 37). (**a**) Simulation of 150 effects randomly drawn from a normal distribution and assigned to random markers. Markers with effect are highlighted with dashed lines. (**b**) Significant QTCs found by QTCAT. (**c**) LASSO selection frequency for each marker during the 50 iterations of QTCAT. (**d**) Manhattan plot of the LMM analysis. The horizontal dashed line depicts the significance threshold when controlling the multiple testing with FWER, whereas the red markers are significantly associated when controlling with FDR. (**e**) The Manhattan plot of the LMM* analysis. GRM was estimated without markers on the chromosome of the actual testing position. The results are shown as in (d).

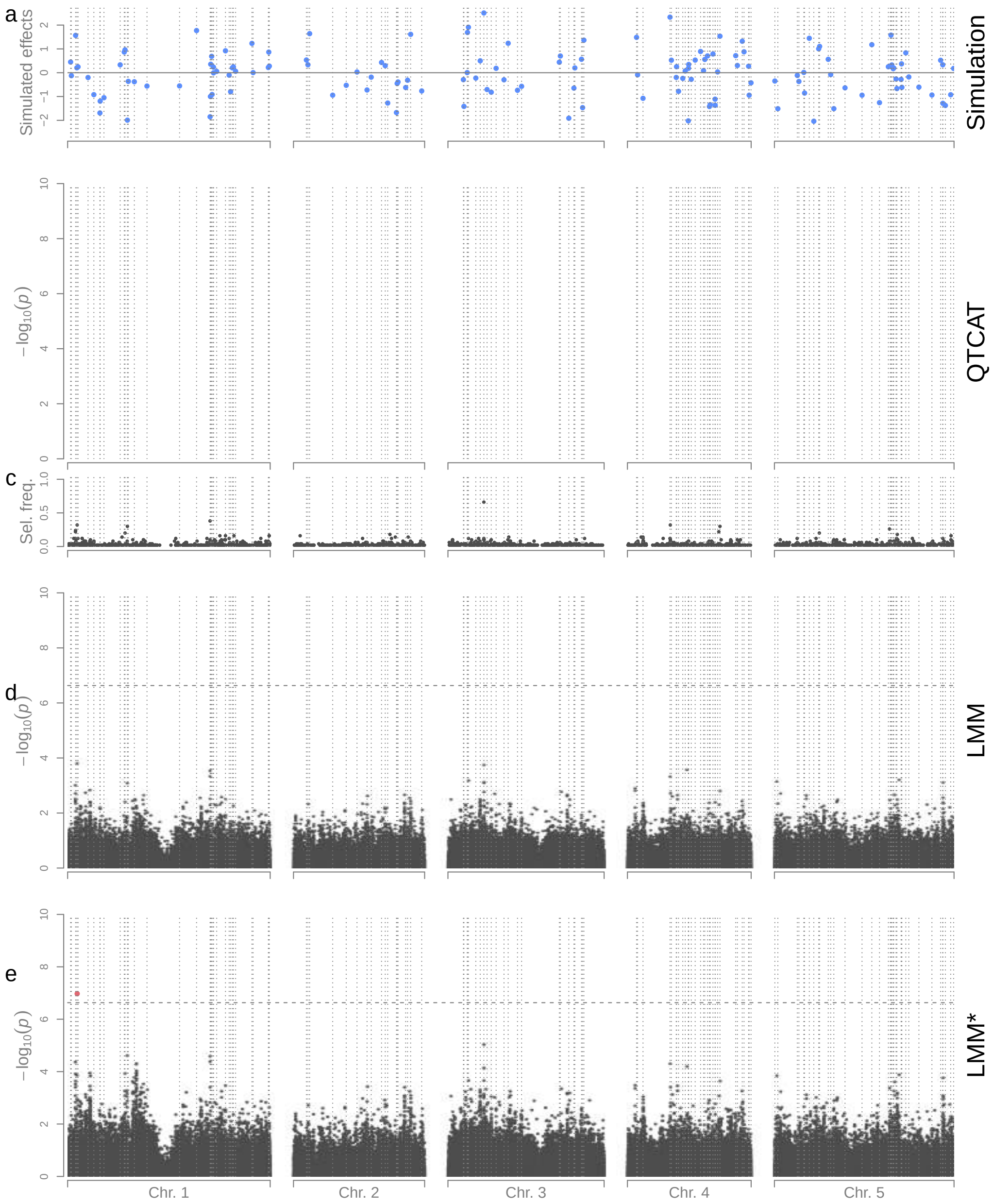

Supplementary Figure 238 Simulation of a GWA analysis based on a structured population with a heritability of 0.4 (run 38). (**a**) Simulation of 150 effects randomly drawn from a normal distribution and assigned to random markers. Markers with effect are highlighted with dashed lines. (**b**) Significant QTCs found by QTCAT. (**c**) LASSO selection frequency for each marker during the 50 iterations of QTCAT. (**d**) Manhattan plot of the LMM analysis. The horizontal dashed line depicts the significance threshold when controlling the multiple testing with FWER, whereas the red markers are significantly associated when controlling with FDR. (**e**) The Manhattan plot of the LMM* analysis. GRM was estimated without markers on the chromosome of the actual testing position. The results are shown as in (d).

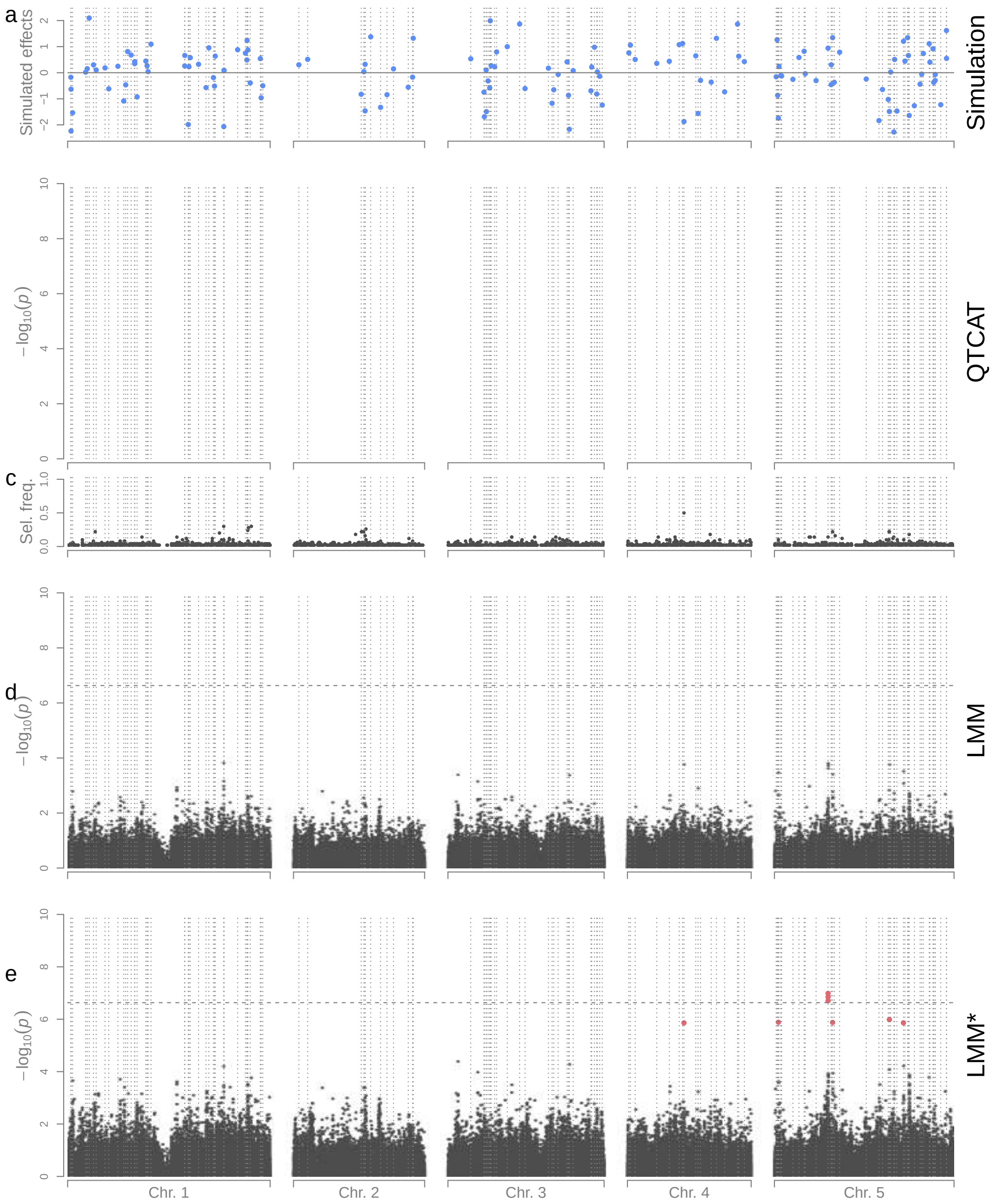

Supplementary Figure 239 Simulation of a GWA analysis based on a structured population with a heritability of 0.4 (run 39). (**a**) Simulation of 150 effects randomly drawn from a normal distribution and assigned to random markers. Markers with effect are highlighted with dashed lines. (**b**) Significant QTCs found by QTCAT. (**c**) LASSO selection frequency for each marker during the 50 iterations of QTCAT. (**d**) Manhattan plot of the LMM analysis. The horizontal dashed line depicts the significance threshold when controlling the multiple testing with FWER, whereas the red markers are significantly associated when controlling with FDR. (**e**) The Manhattan plot of the LMM* analysis. GRM was estimated without markers on the chromosome of the actual testing position. The results are shown as in (d).

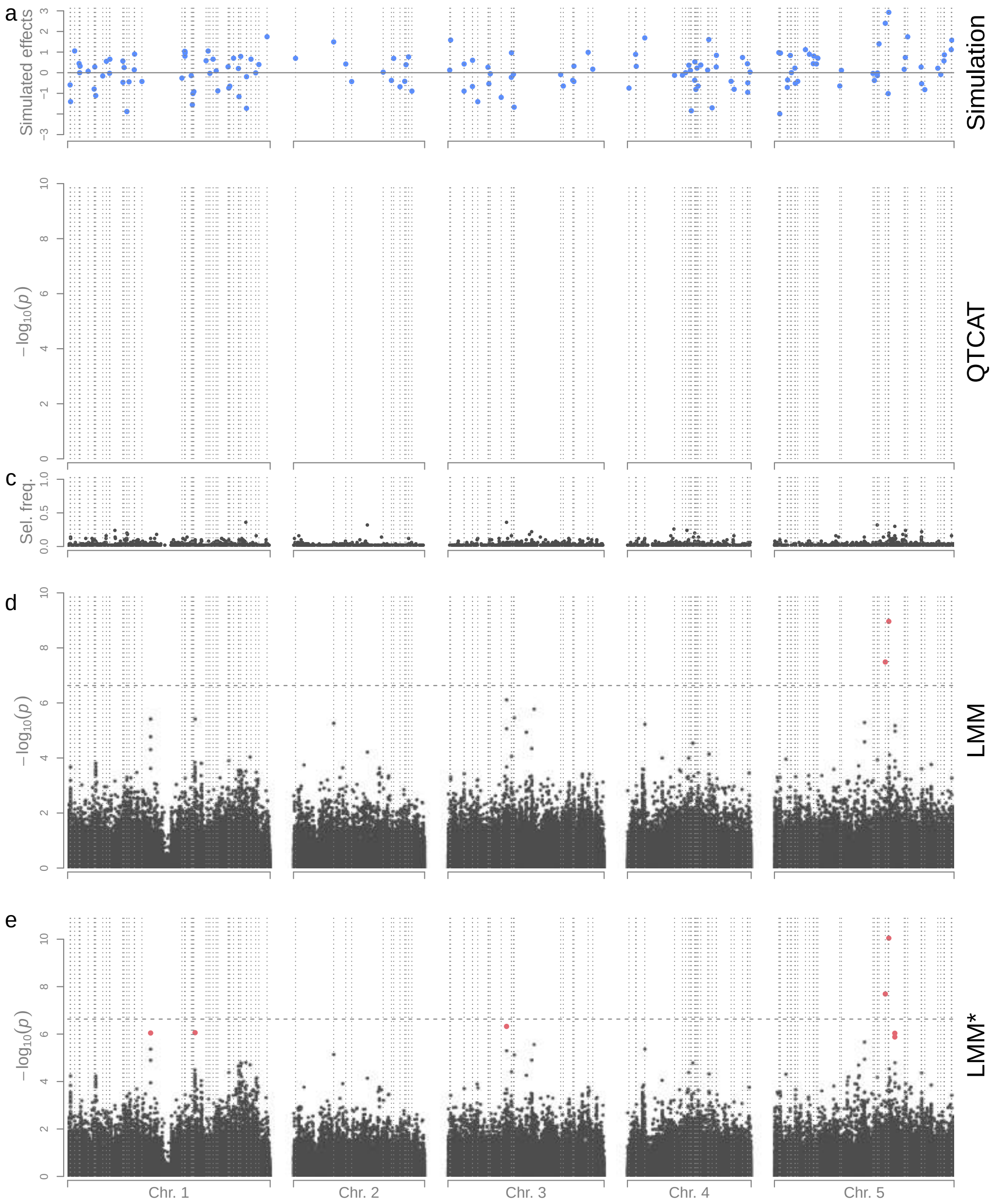

Supplementary Figure 240 Simulation of a GWA analysis based on a structured population with a heritability of 0.4 (run 40). (**a**) Simulation of 150 effects randomly drawn from a normal distribution and assigned to random markers. Markers with effect are highlighted with dashed lines. (**b**) Significant QTCs found by QTCAT. (**c**) LASSO selection frequency for each marker during the 50 iterations of QTCAT. (**d**) Manhattan plot of the LMM analysis. The horizontal dashed line depicts the significance threshold when controlling the multiple testing with FWER, whereas the red markers are significantly associated when controlling with FDR. (**e**) The Manhattan plot of the LMM* analysis. GRM was estimated without markers on the chromosome of the actual testing position. The results are shown as in (d).

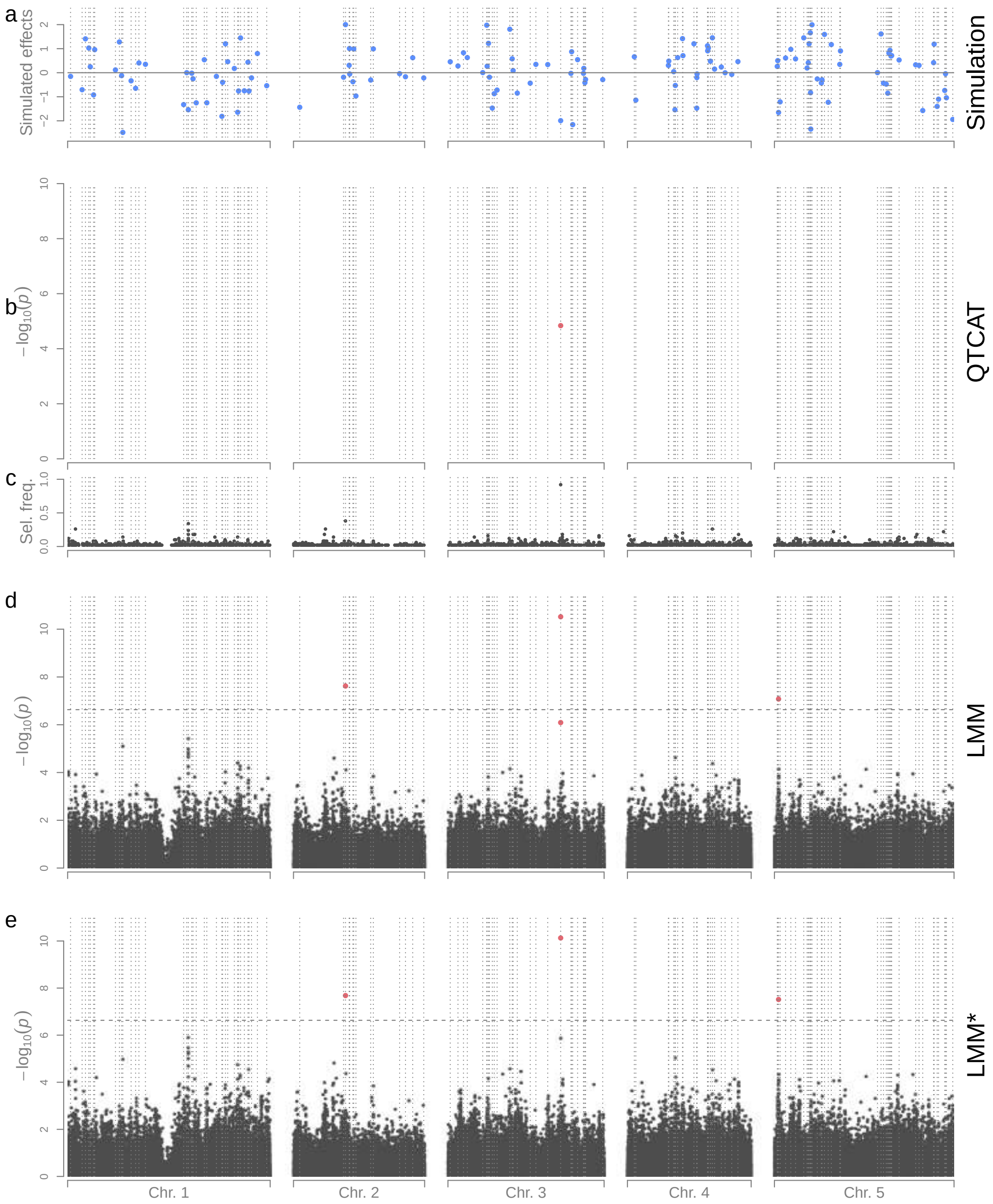

Supplementary Figure 241 Simulation of a GWA analysis based on a structured population with a heritability of 0.4 (run 41). (**a**) Simulation of 150 effects randomly drawn from a normal distribution and assigned to random markers. Markers with effect are highlighted with dashed lines. (**b**) Significant QTCs found by QTCAT. (**c**) LASSO selection frequency for each marker during the 50 iterations of QTCAT. (**d**) Manhattan plot of the LMM analysis. The horizontal dashed line depicts the significance threshold when controlling the multiple testing with FWER, whereas the red markers are significantly associated when controlling with FDR. (**e**) The Manhattan plot of the LMM* analysis. GRM was estimated without markers on the chromosome of the actual testing position. The results are shown as in (d).

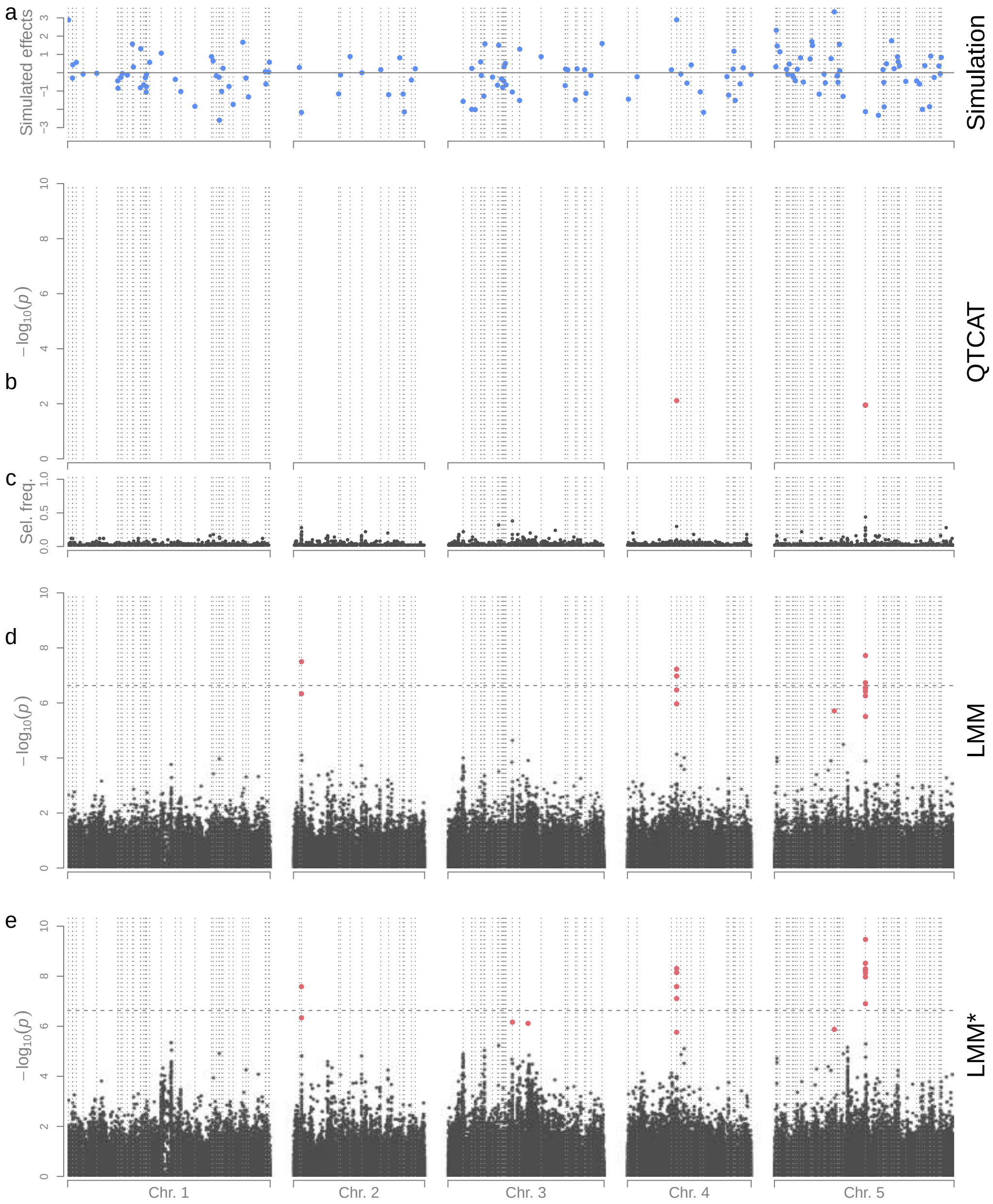

Supplementary Figure 242 Simulation of a GWA analysis based on a structured population with a heritability of 0.4 (run 42). (**a**) Simulation of 150 effects randomly drawn from a normal distribution and assigned to random markers. Markers with effect are highlighted with dashed lines. (**b**) Significant QTCs found by QTCAT. (**c**) LASSO selection frequency for each marker during the 50 iterations of QTCAT. (**d**) Manhattan plot of the LMM analysis. The horizontal dashed line depicts the significance threshold when controlling the multiple testing with FWER, whereas the red markers are significantly associated when controlling with FDR. (**e**) The Manhattan plot of the LMM* analysis. GRM was estimated without markers on the chromosome of the actual testing position. The results are shown as in (d).

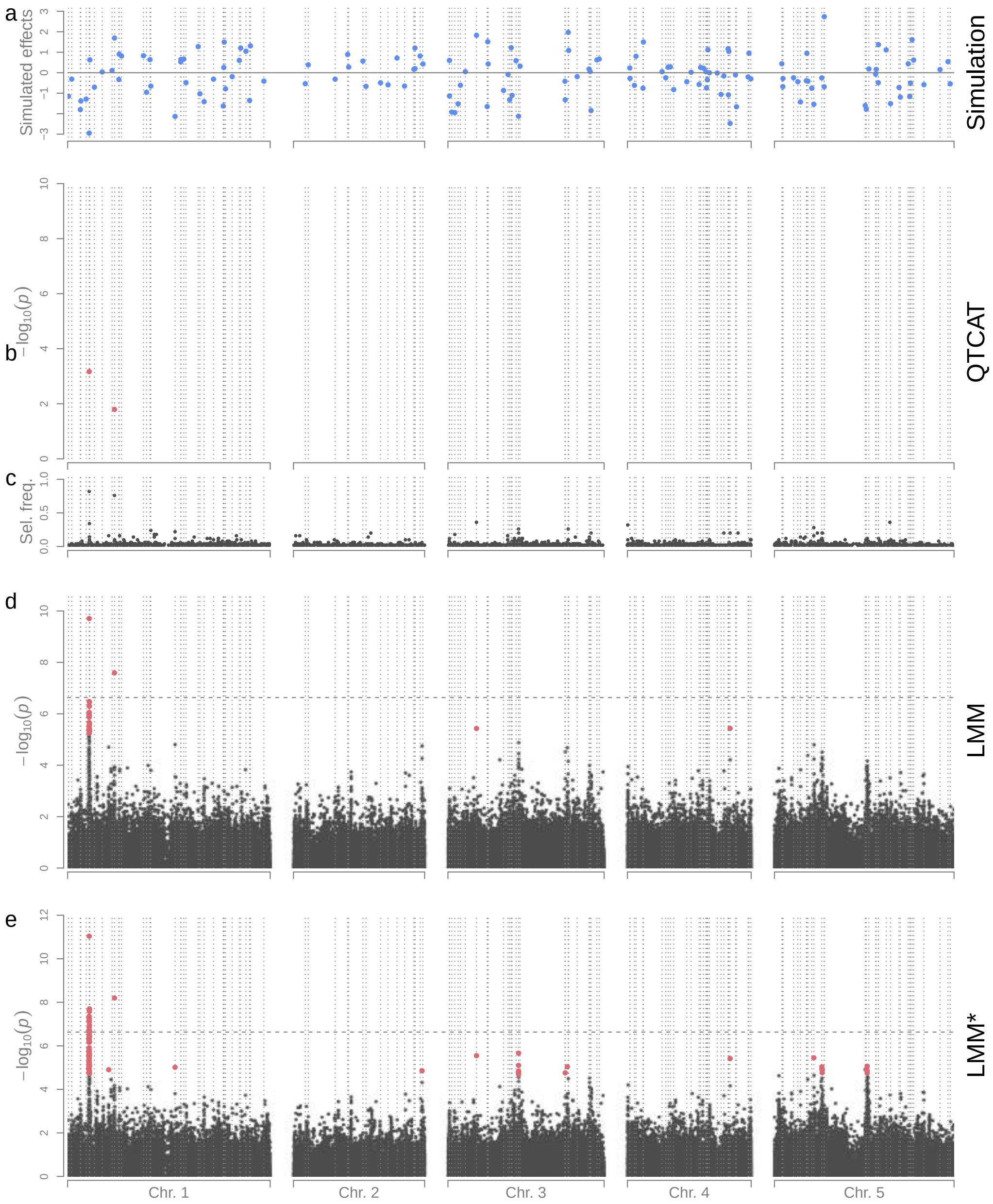

Supplementary Figure 243 Simulation of a GWA analysis based on a structured population with a heritability of 0.4 (run 43). (**a**) Simulation of 150 effects randomly drawn from a normal distribution and assigned to random markers. Markers with effect are highlighted with dashed lines. (**b**) Significant QTCs found by QTCAT. (**c**) LASSO selection frequency for each marker during the 50 iterations of QTCAT. (**d**) Manhattan plot of the LMM analysis. The horizontal dashed line depicts the significance threshold when controlling the multiple testing with FWER, whereas the red markers are significantly associated when controlling with FDR. (**e**) The Manhattan plot of the LMM* analysis. GRM was estimated without markers on the chromosome of the actual testing position. The results are shown as in (d).

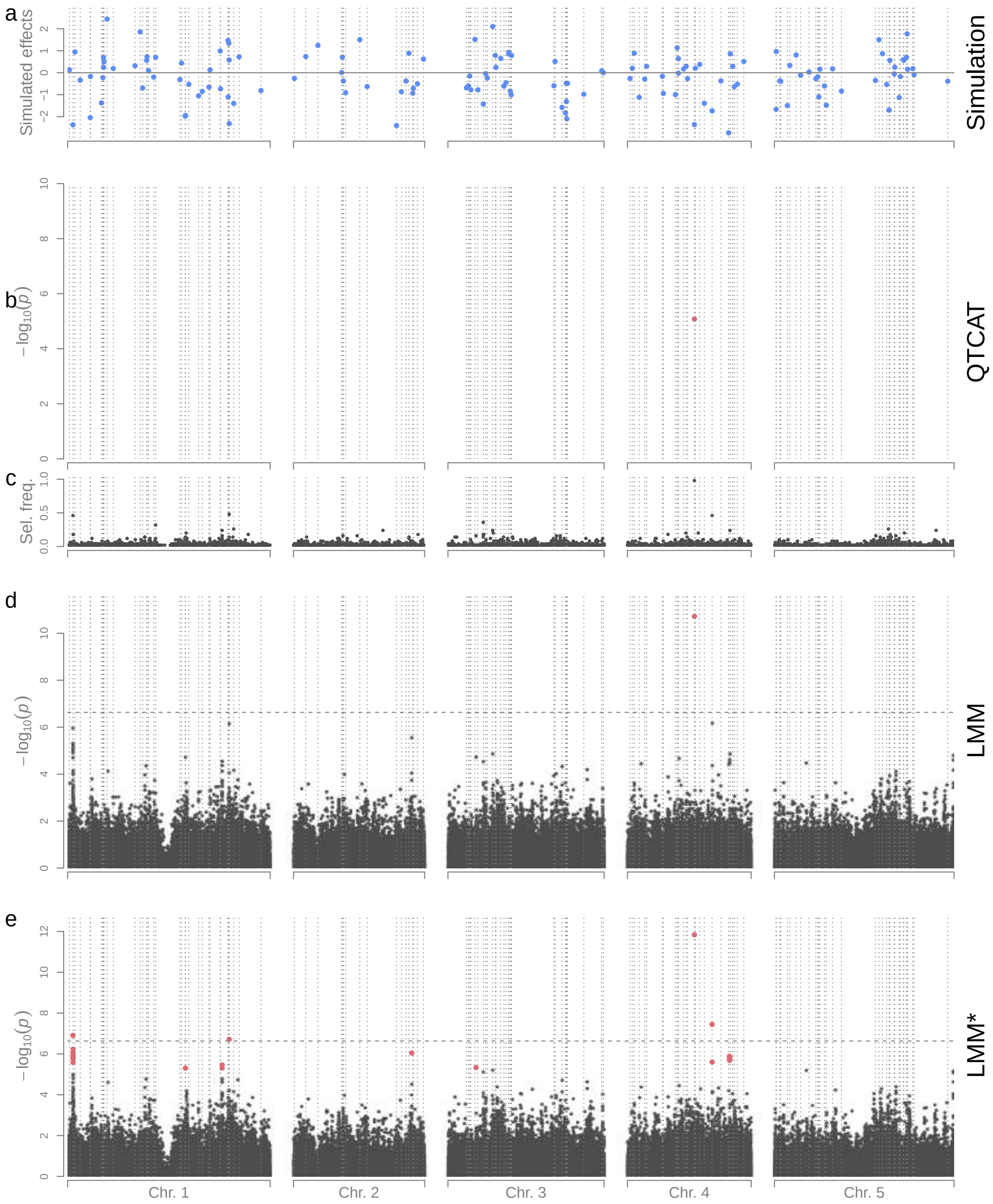

Supplementary Figure 244 Simulation of a GWA analysis based on a structured population with a heritability of 0.4 (run 44). (**a**) Simulation of 150 effects randomly drawn from a normal distribution and assigned to random markers. Markers with effect are highlighted with dashed lines. (**b**) Significant QTCs found by QTCAT. (**c**) LASSO selection frequency for each marker during the 50 iterations of QTCAT. (**d**) Manhattan plot of the LMM analysis. The horizontal dashed line depicts the significance threshold when controlling the multiple testing with FWER, whereas the red markers are significantly associated when controlling with FDR. (**e**) The Manhattan plot of the LMM* analysis. GRM was estimated without markers on the chromosome of the actual testing position. The results are shown as in (d).

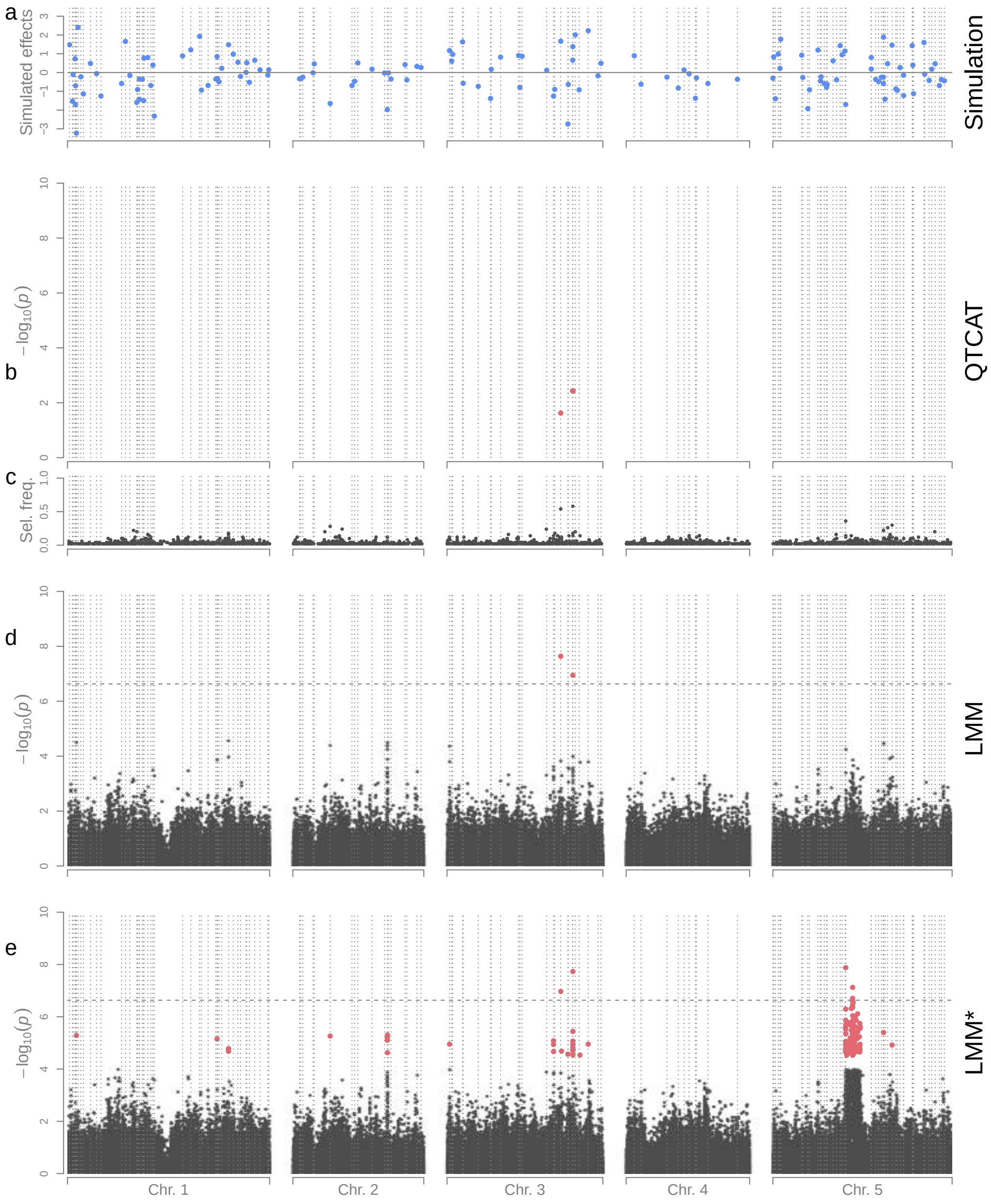

Supplementary Figure 245 Simulation of a GWA analysis based on a structured population with a heritability of 0.4 (run 45). (**a**) Simulation of 150 effects randomly drawn from a normal distribution and assigned to random markers. Markers with effect are highlighted with dashed lines. (**b**) Significant QTCs found by QTCAT. (**c**) LASSO selection frequency for each marker during the 50 iterations of QTCAT. (**d**) Manhattan plot of the LMM analysis. The horizontal dashed line depicts the significance threshold when controlling the multiple testing with FWER, whereas the red markers are significantly associated when controlling with FDR. (**e**) The Manhattan plot of the LMM* analysis. GRM was estimated without markers on the chromosome of the actual testing position. The results are shown as in (d).

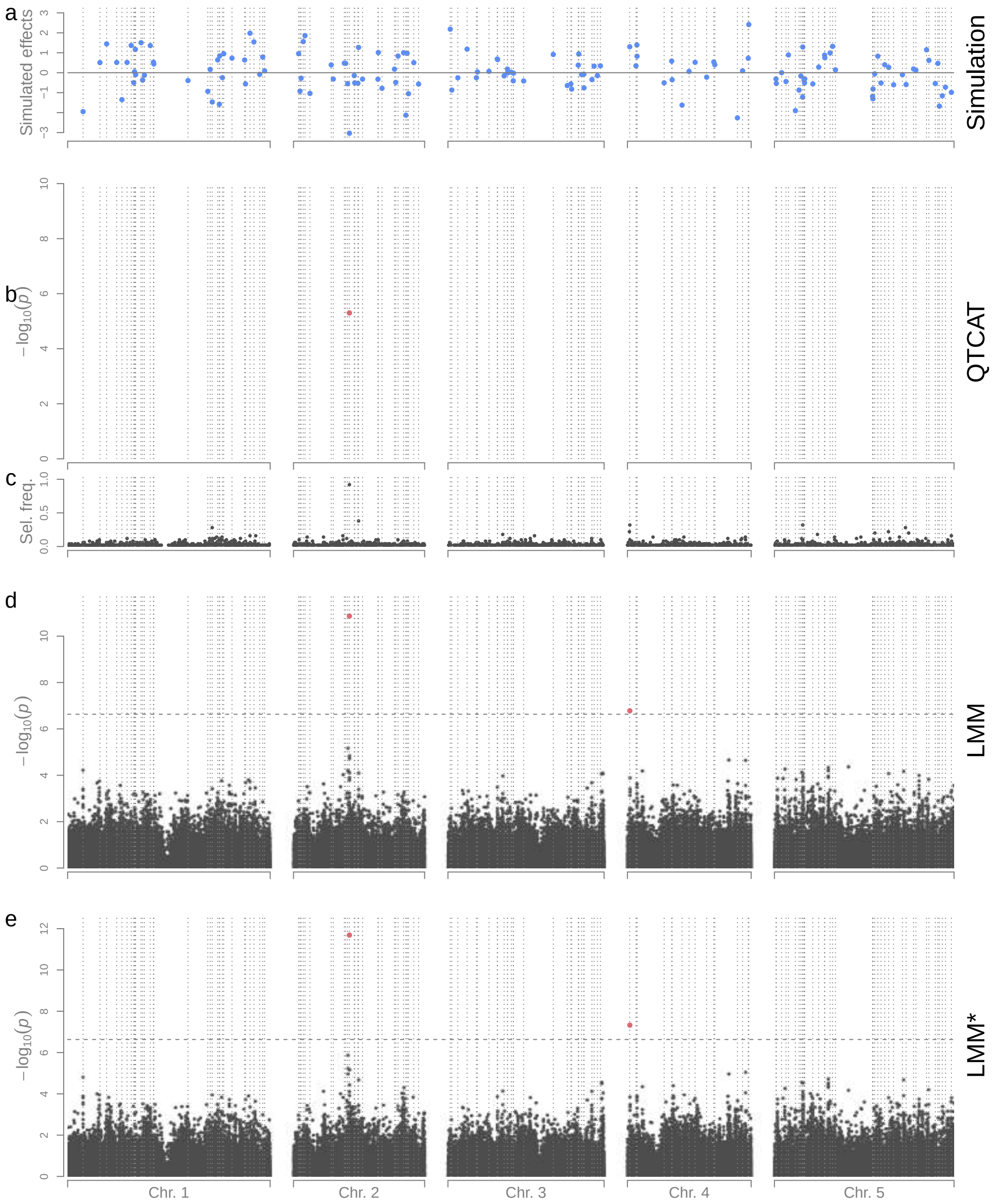

Supplementary Figure 246 Simulation of a GWA analysis based on a structured population with a heritability of 0.4 (run 46). (**a**) Simulation of 150 effects randomly drawn from a normal distribution and assigned to random markers. Markers with effect are highlighted with dashed lines. (**b**) Significant QTCs found by QTCAT. (**c**) LASSO selection frequency for each marker during the 50 iterations of QTCAT. (**d**) Manhattan plot of the LMM analysis. The horizontal dashed line depicts the significance threshold when controlling the multiple testing with FWER, whereas the red markers are significantly associated when controlling with FDR. (**e**) The Manhattan plot of the LMM* analysis. GRM was estimated without markers on the chromosome of the actual testing position. The results are shown as in (d).

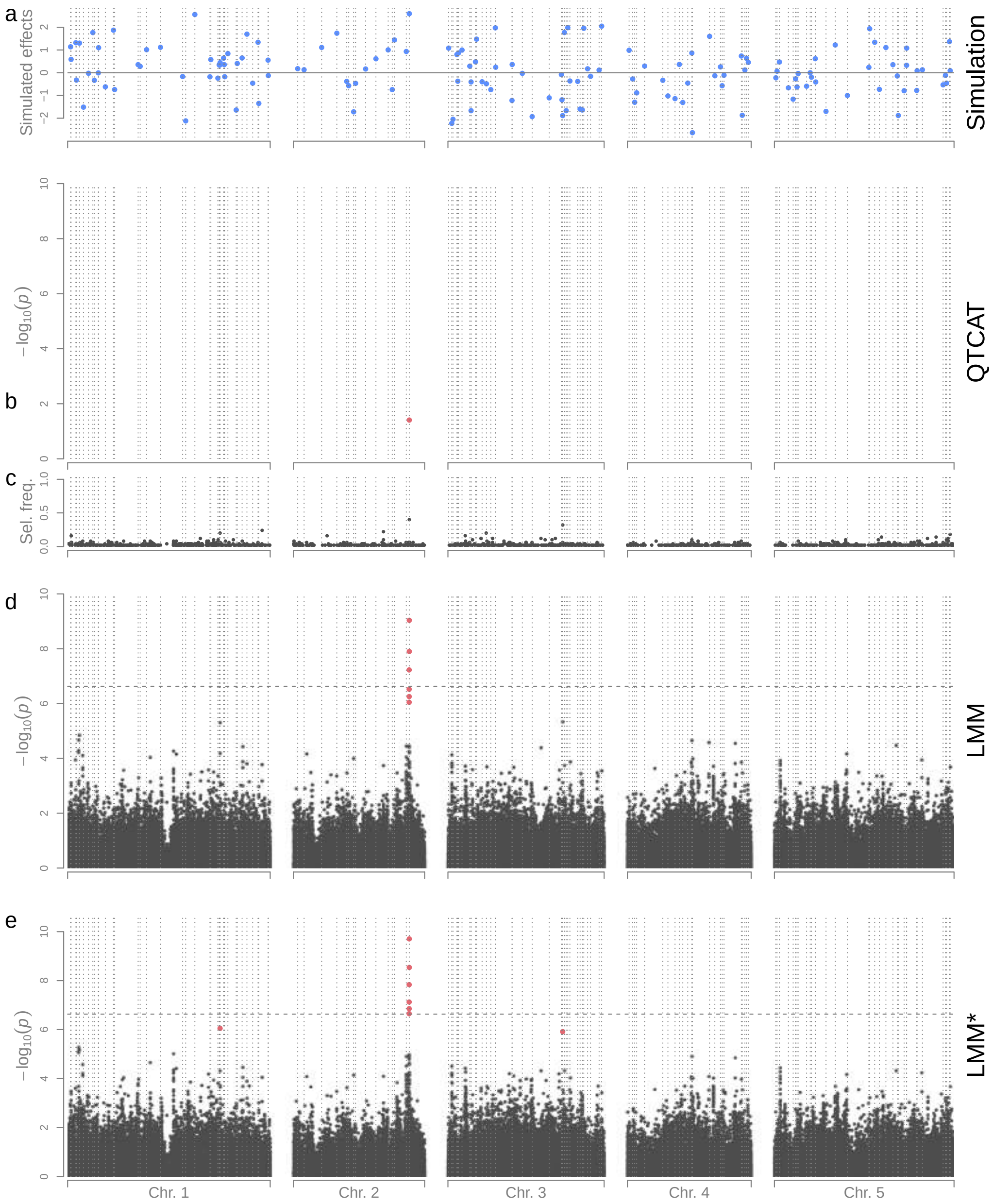

Supplementary Figure 247 Simulation of a GWA analysis based on a structured population with a heritability of 0.4 (run 47). (**a**) Simulation of 150 effects randomly drawn from a normal distribution and assigned to random markers. Markers with effect are highlighted with dashed lines. (**b**) Significant QTCs found by QTCAT. (**c**) LASSO selection frequency for each marker during the 50 iterations of QTCAT. (**d**) Manhattan plot of the LMM analysis. The horizontal dashed line depicts the significance threshold when controlling the multiple testing with FWER, whereas the red markers are significantly associated when controlling with FDR. (**e**) The Manhattan plot of the LMM* analysis. GRM was estimated without markers on the chromosome of the actual testing position. The results are shown as in (d).

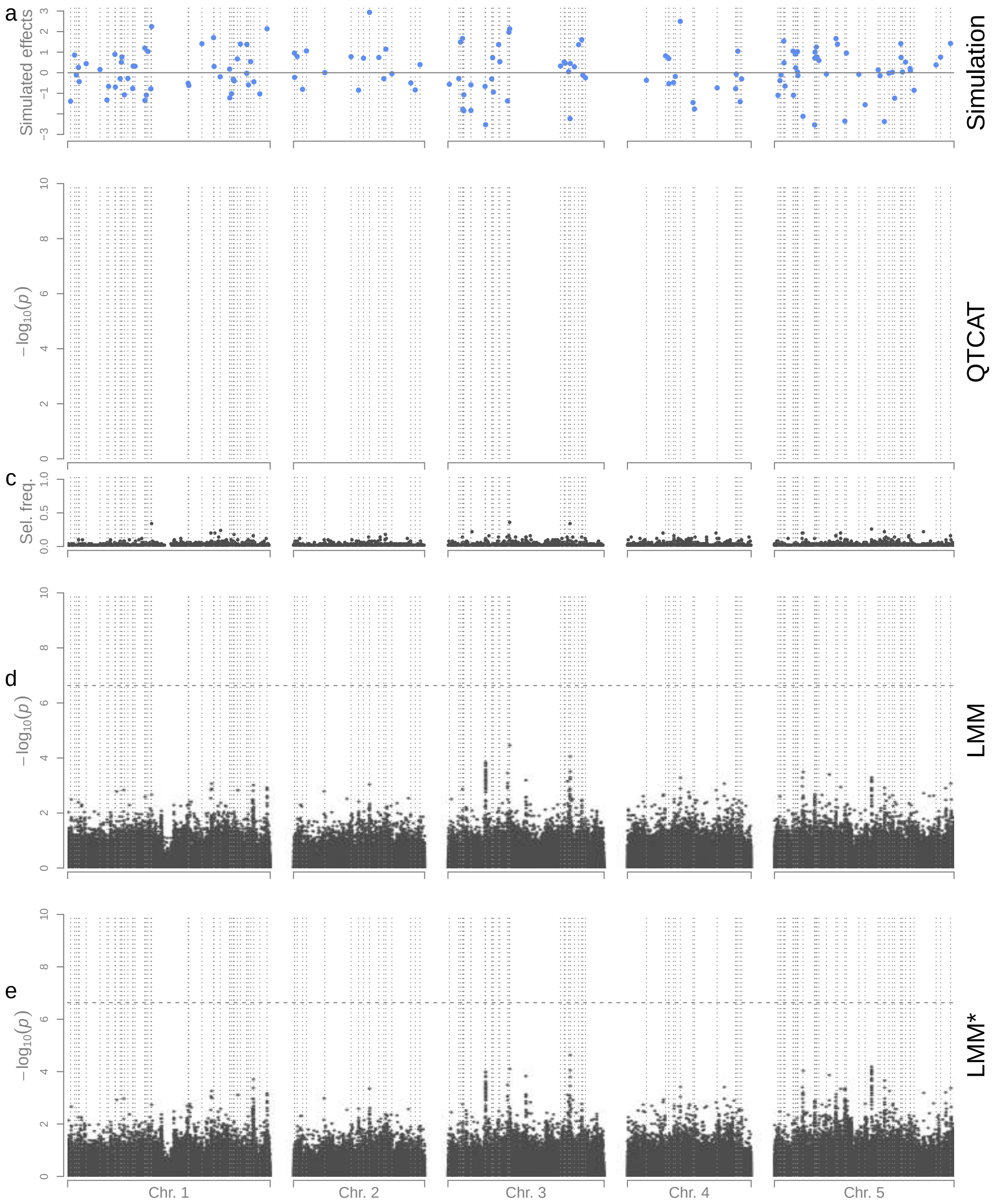

Supplementary Figure 248 Simulation of a GWA analysis based on a structured population with a heritability of 0.4 (run 48). (**a**) Simulation of 150 effects randomly drawn from a normal distribution and assigned to random markers. Markers with effect are highlighted with dashed lines. (**b**) Significant QTCs found by QTCAT. (**c**) LASSO selection frequency for each marker during the 50 iterations of QTCAT. (**d**) Manhattan plot of the LMM analysis. The horizontal dashed line depicts the significance threshold when controlling the multiple testing with FWER, whereas the red markers are significantly associated when controlling with FDR. (**e**) The Manhattan plot of the LMM* analysis. GRM was estimated without markers on the chromosome of the actual testing position. The results are shown as in (d).

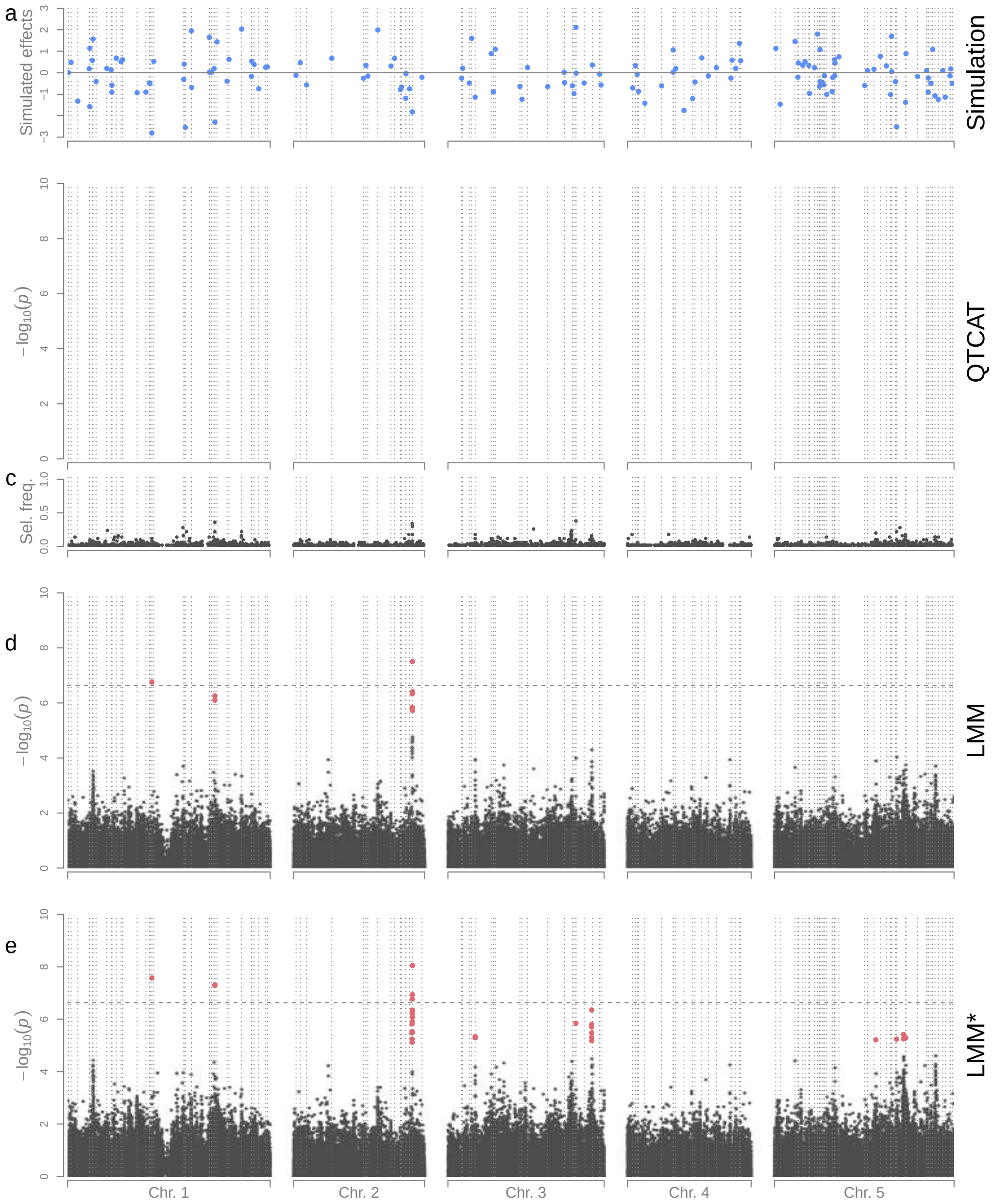

Supplementary Figure 249 Simulation of a GWA analysis based on a structured population with a heritability of 0.4 (run 49). (**a**) Simulation of 150 effects randomly drawn from a normal distribution and assigned to random markers. Markers with effect are highlighted with dashed lines. (**b**) Significant QTCs found by QTCAT. (**c**) LASSO selection frequency for each marker during the 50 iterations of QTCAT. (**d**) Manhattan plot of the LMM analysis. The horizontal dashed line depicts the significance threshold when controlling the multiple testing with FWER, whereas the red markers are significantly associated when controlling with FDR. (**e**) The Manhattan plot of the LMM* analysis. GRM was estimated without markers on the chromosome of the actual testing position. The results are shown as in (d).

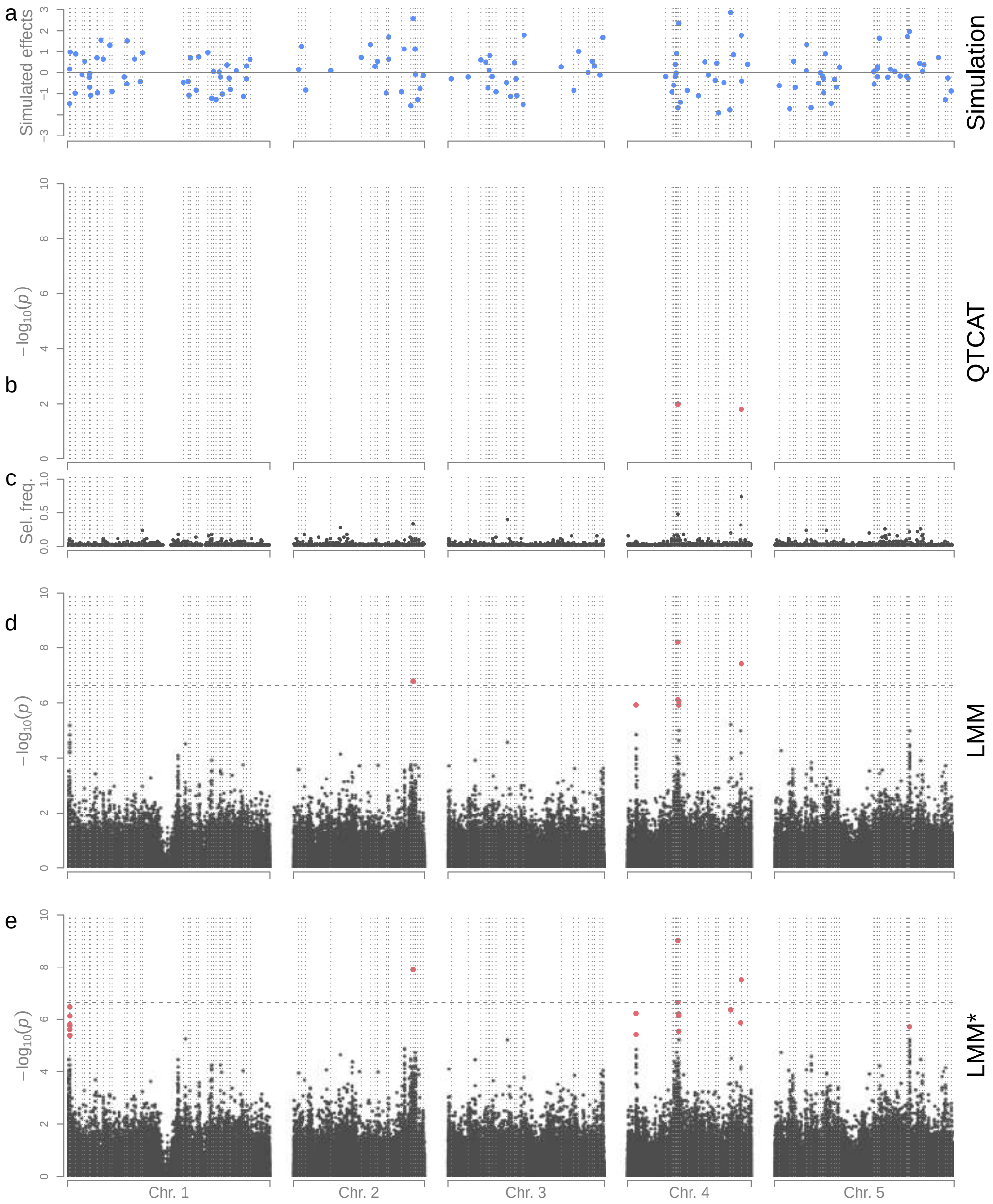

Supplementary Figure 250 Simulation of a GWA analysis based on a structured population with a heritability of 0.4 (run 50). (**a**) Simulation of 150 effects randomly drawn from a normal distribution and assigned to random markers. Markers with effect are highlighted with dashed lines. (**b**) Significant QTCs found by QTCAT. (**c**) LASSO selection frequency for each marker during the 50 iterations of QTCAT. (**d**) Manhattan plot of the LMM analysis. The horizontal dashed line depicts the significance threshold when controlling the multiple testing with FWER, whereas the red markers are significantly associated when controlling with FDR. (**e**) The Manhattan plot of the LMM* analysis. GRM was estimated without markers on the chromosome of the actual testing position. The results are shown as in (d).

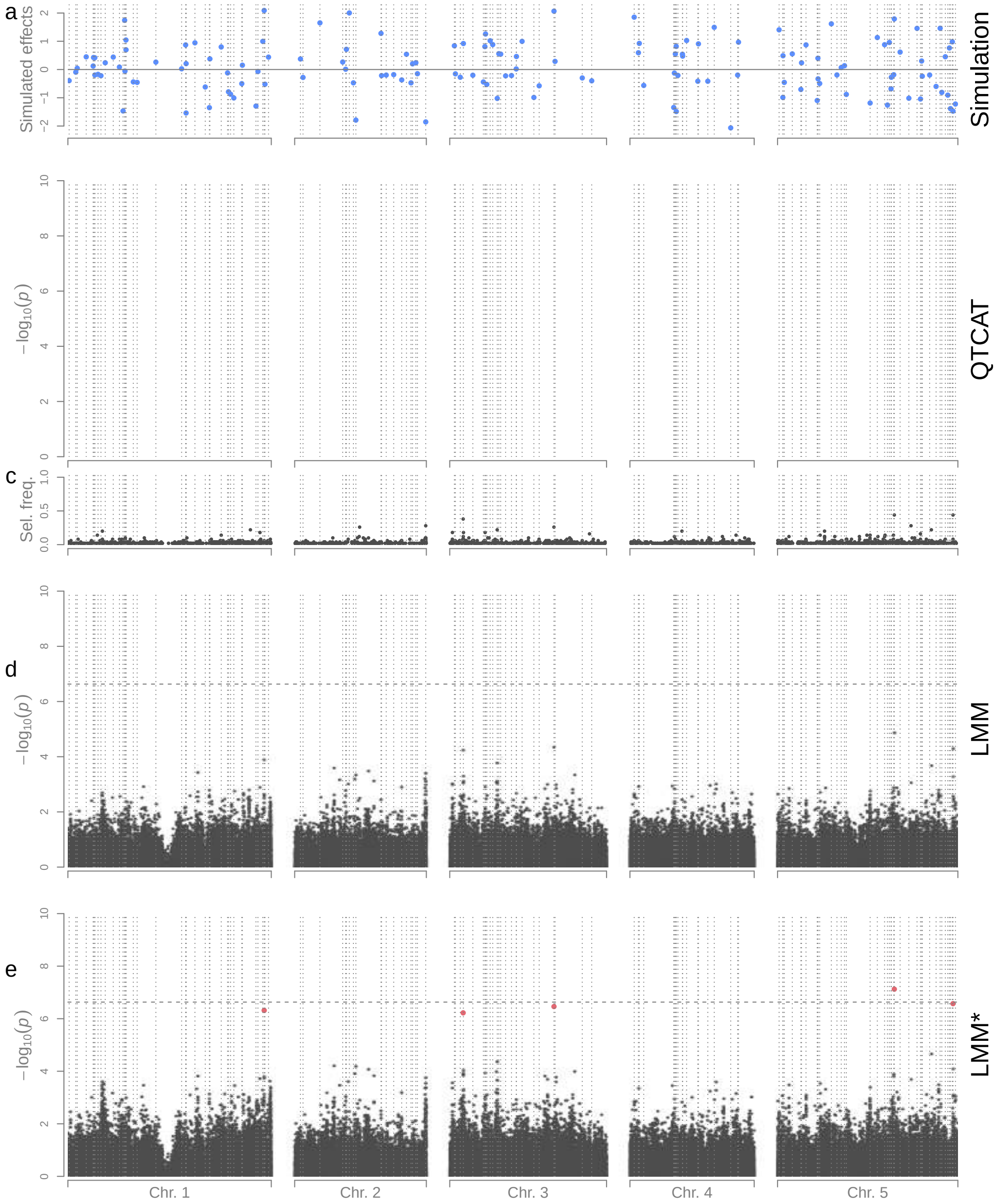

Supplementary Figure 251 Simulation of a GWA analysis based on a structured population with a heritability of 0.4 (run 51). (**a**) Simulation of 150 effects randomly drawn from a normal distribution and assigned to random markers. Markers with effect are highlighted with dashed lines. (**b**) Significant QTCs found by QTCAT. (**c**) LASSO selection frequency for each marker during the 50 iterations of QTCAT. (**d**) Manhattan plot of the LMM analysis. The horizontal dashed line depicts the significance threshold when controlling the multiple testing with FWER, whereas the red markers are significantly associated when controlling with FDR. (**e**) The Manhattan plot of the LMM* analysis. GRM was estimated without markers on the chromosome of the actual testing position. The results are shown as in (d).

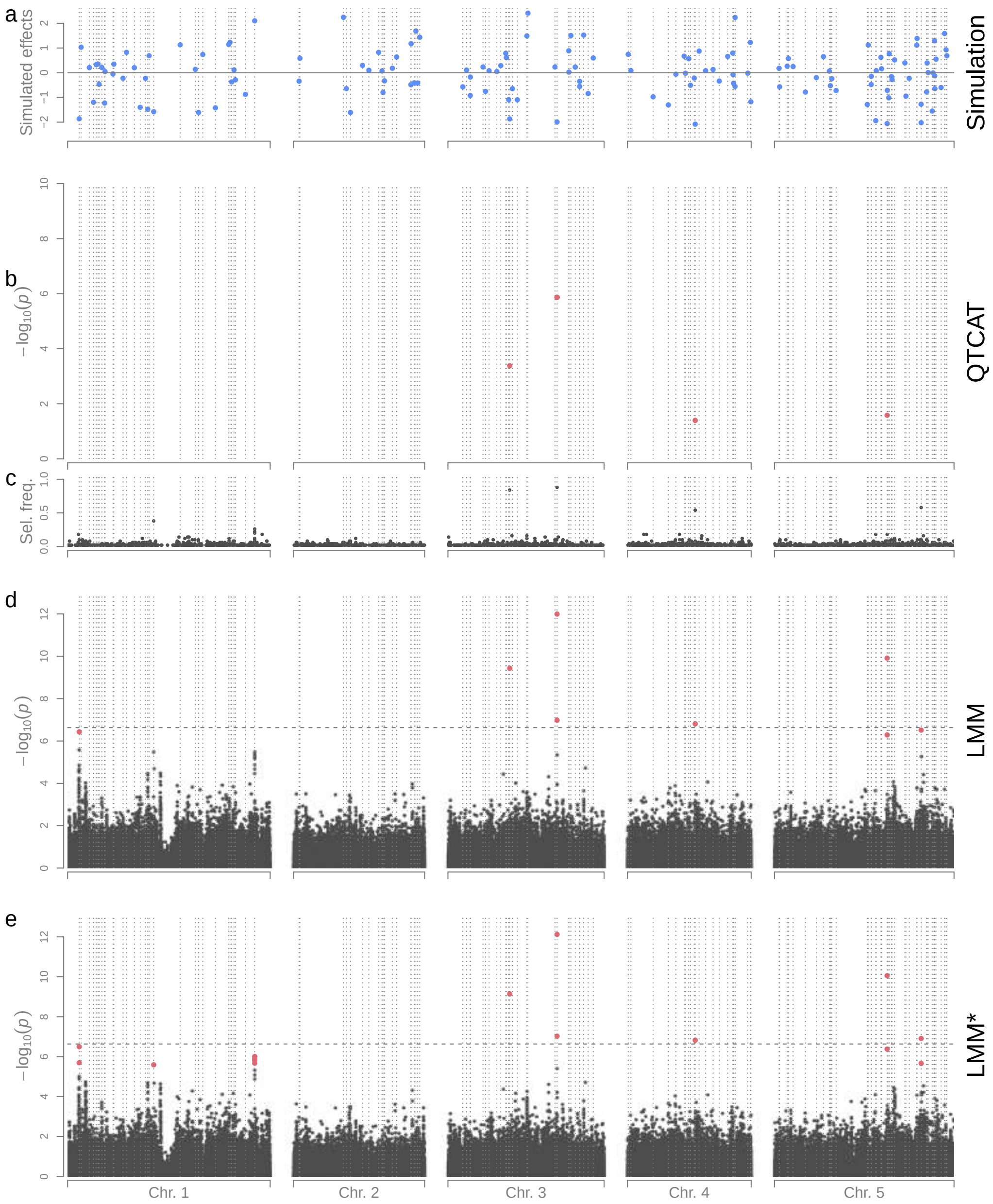

Supplementary Figure 252 Simulation of a GWA analysis based on a structured population with a heritability of 0.4 (run 52). (**a**) Simulation of 150 effects randomly drawn from a normal distribution and assigned to random markers. Markers with effect are highlighted with dashed lines. (**b**) Significant QTCs found by QTCAT. (**c**) LASSO selection frequency for each marker during the 50 iterations of QTCAT. (**d**) Manhattan plot of the LMM analysis. The horizontal dashed line depicts the significance threshold when controlling the multiple testing with FWER, whereas the red markers are significantly associated when controlling with FDR. (**e**) The Manhattan plot of the LMM* analysis. GRM was estimated without markers on the chromosome of the actual testing position. The results are shown as in (d).

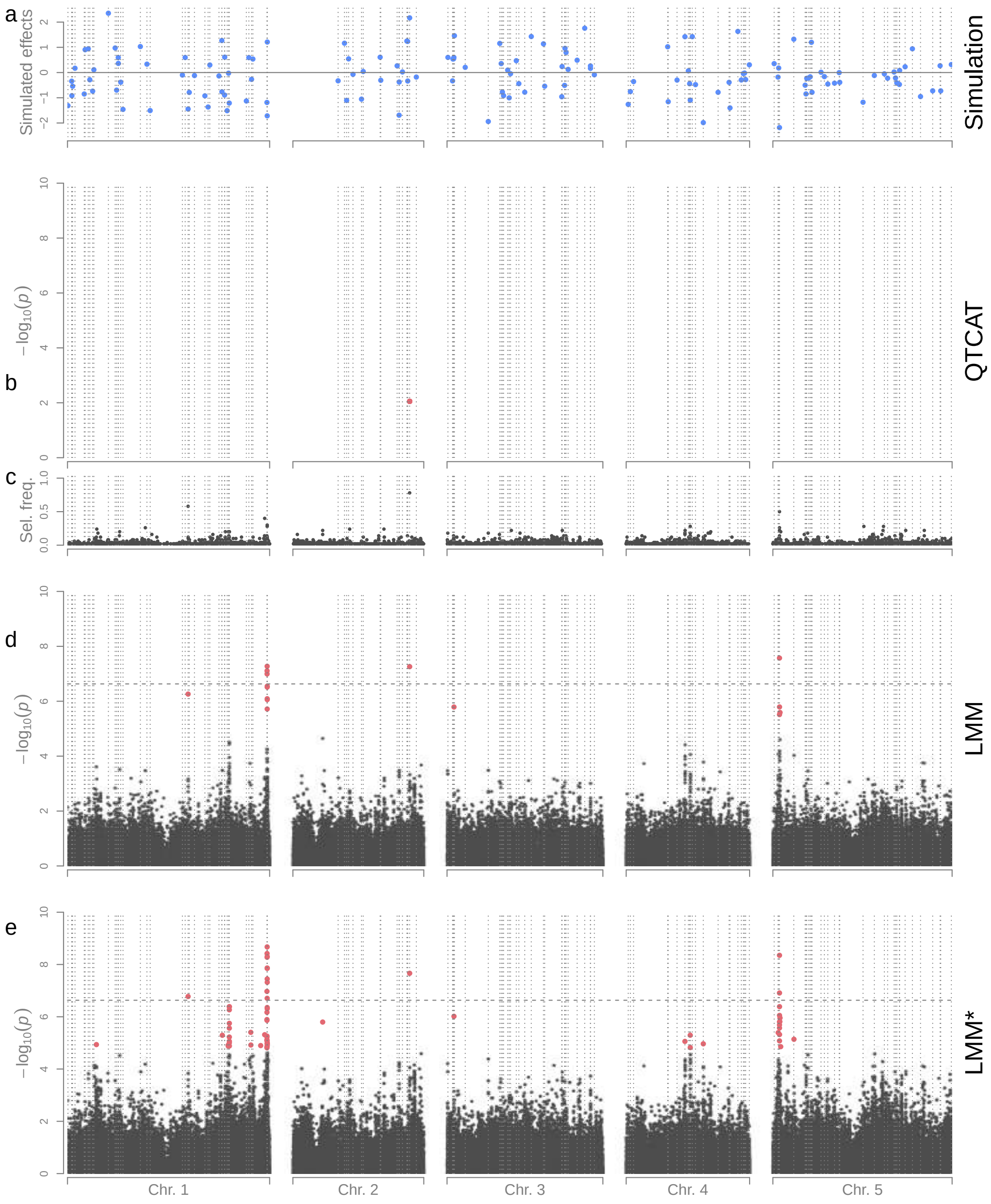

Supplementary Figure 253 Simulation of a GWA analysis based on a structured population with a heritability of 0.4 (run 53). (**a**) Simulation of 150 effects randomly drawn from a normal distribution and assigned to random markers. Markers with effect are highlighted with dashed lines. (**b**) Significant QTCs found by QTCAT. (**c**) LASSO selection frequency for each marker during the 50 iterations of QTCAT. (**d**) Manhattan plot of the LMM analysis. The horizontal dashed line depicts the significance threshold when controlling the multiple testing with FWER, whereas the red markers are significantly associated when controlling with FDR. (**e**) The Manhattan plot of the LMM* analysis. GRM was estimated without markers on the chromosome of the actual testing position. The results are shown as in (d).

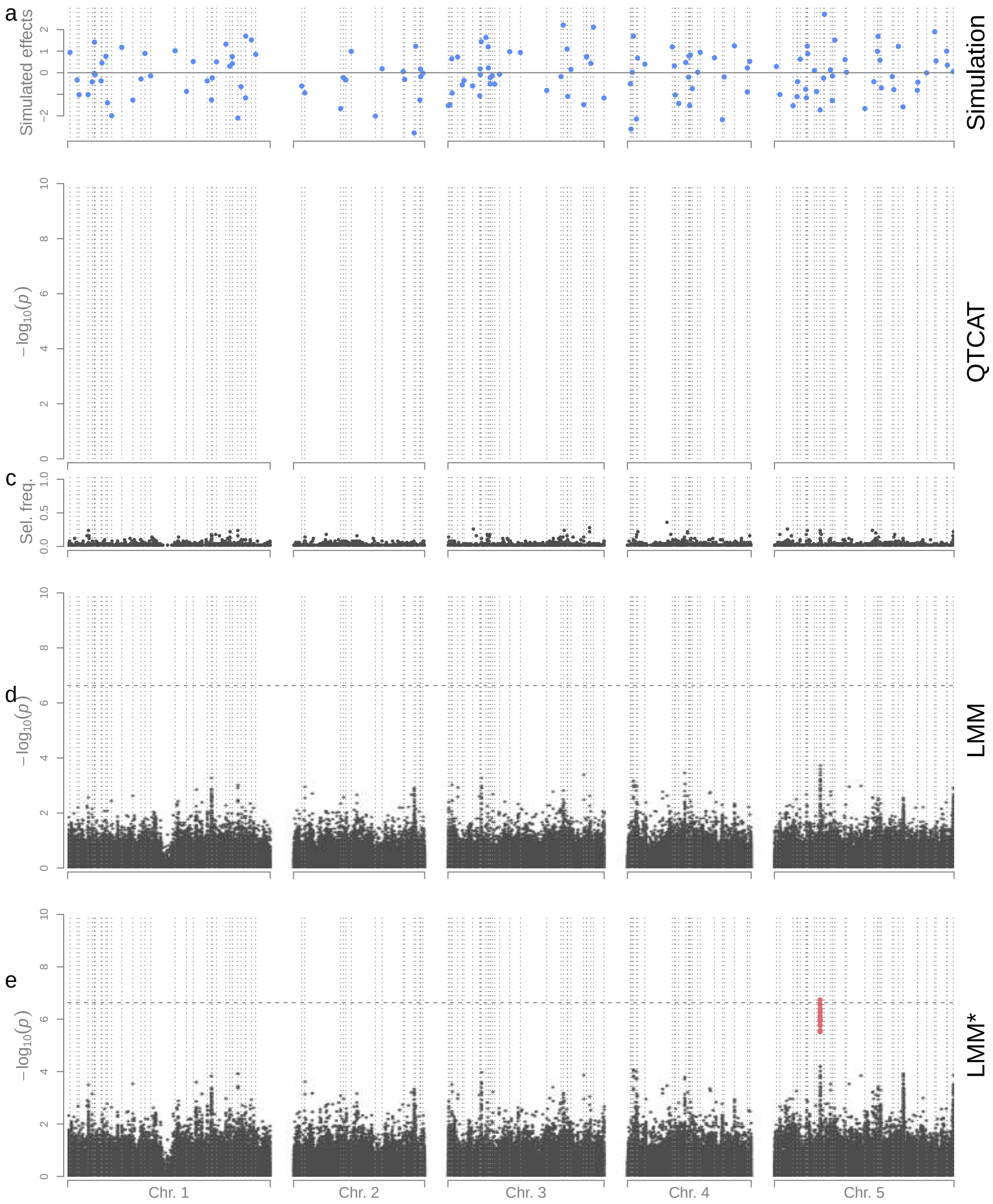

Supplementary Figure 254 Simulation of a GWA analysis based on a structured population with a heritability of 0.4 (run 54). (**a**) Simulation of 150 effects randomly drawn from a normal distribution and assigned to random markers. Markers with effect are highlighted with dashed lines. (**b**) Significant QTCs found by QTCAT. (**c**) LASSO selection frequency for each marker during the 50 iterations of QTCAT. (**d**) Manhattan plot of the LMM analysis. The horizontal dashed line depicts the significance threshold when controlling the multiple testing with FWER, whereas the red markers are significantly associated when controlling with FDR. (**e**) The Manhattan plot of the LMM* analysis. GRM was estimated without markers on the chromosome of the actual testing position. The results are shown as in (d).

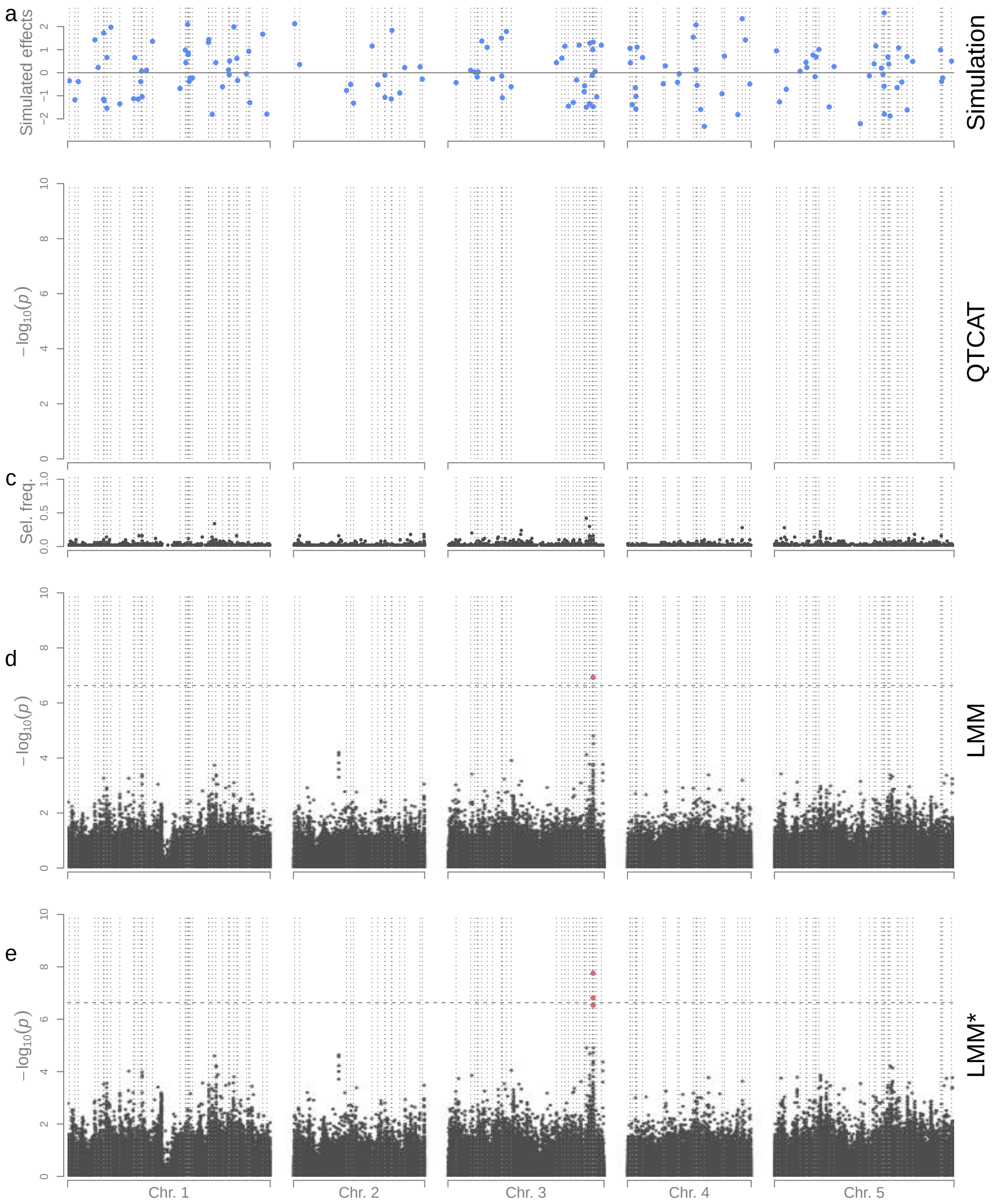

Supplementary Figure 255 Simulation of a GWA analysis based on a structured population with a heritability of 0.4 (run 55). (**a**) Simulation of 150 effects randomly drawn from a normal distribution and assigned to random markers. Markers with effect are highlighted with dashed lines. (**b**) Significant QTCs found by QTCAT. (**c**) LASSO selection frequency for each marker during the 50 iterations of QTCAT. (**d**) Manhattan plot of the LMM analysis. The horizontal dashed line depicts the significance threshold when controlling the multiple testing with FWER, whereas the red markers are significantly associated when controlling with FDR. (**e**) The Manhattan plot of the LMM* analysis. GRM was estimated without markers on the chromosome of the actual testing position. The results are shown as in (d).

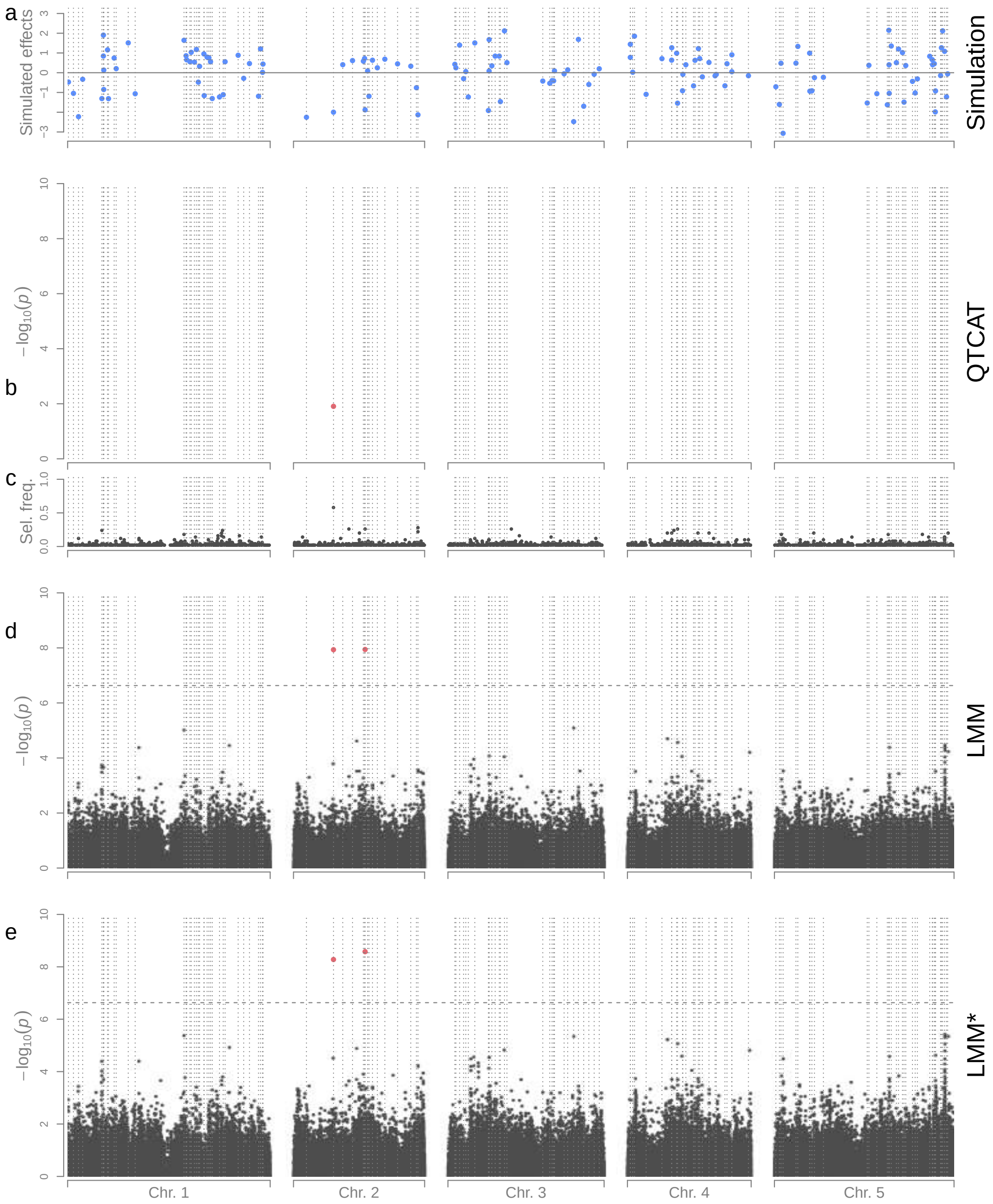

Supplementary Figure 256 Simulation of a GWA analysis based on a structured population with a heritability of 0.4 (run 56). (**a**) Simulation of 150 effects randomly drawn from a normal distribution and assigned to random markers. Markers with effect are highlighted with dashed lines. (**b**) Significant QTCs found by QTCAT. (**c**) LASSO selection frequency for each marker during the 50 iterations of QTCAT. (**d**) Manhattan plot of the LMM analysis. The horizontal dashed line depicts the significance threshold when controlling the multiple testing with FWER, whereas the red markers are significantly associated when controlling with FDR. (**e**) The Manhattan plot of the LMM* analysis. GRM was estimated without markers on the chromosome of the actual testing position. The results are shown as in (d).

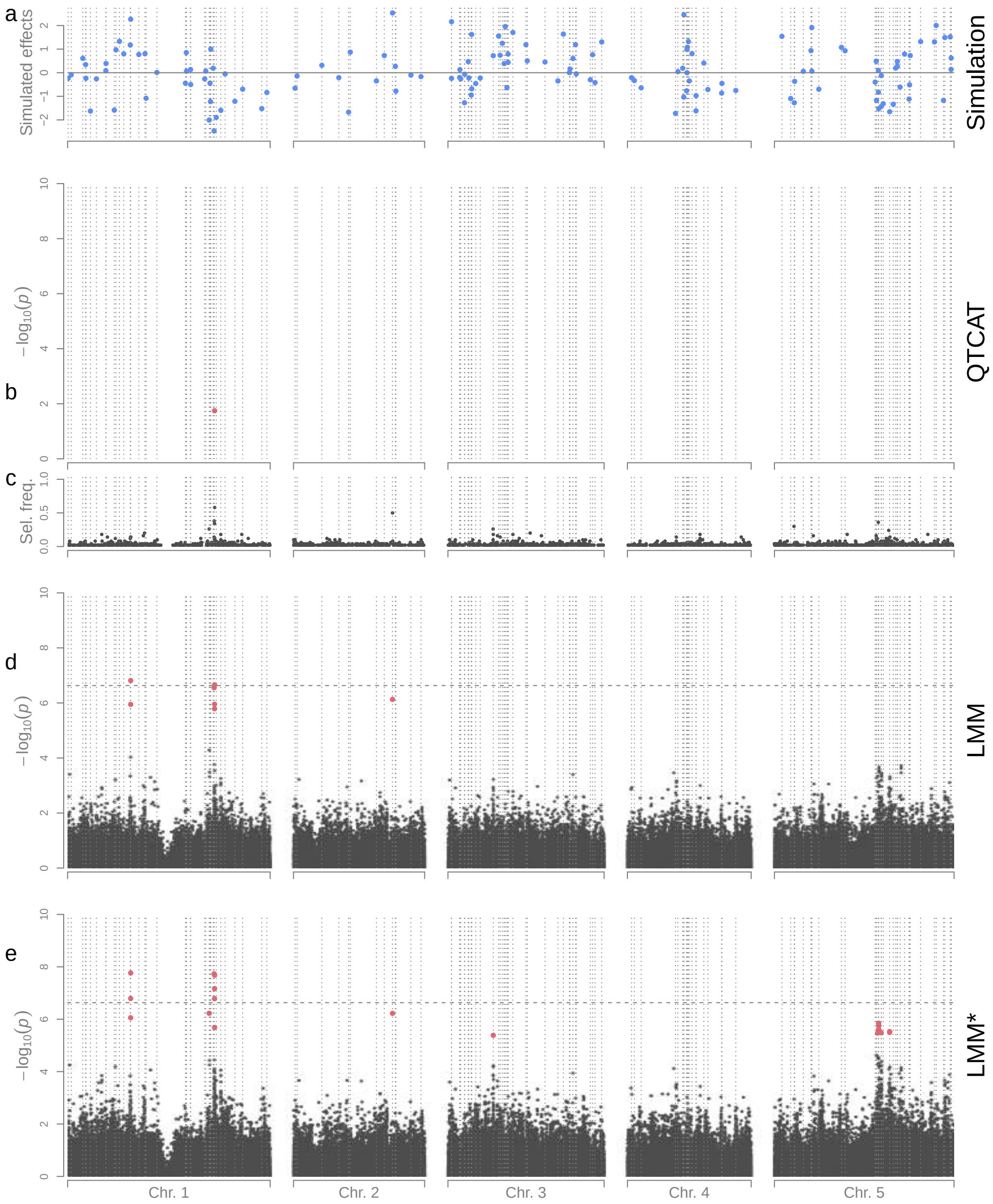

Supplementary Figure 257 Simulation of a GWA analysis based on a structured population with a heritability of 0.4 (run 57). (**a**) Simulation of 150 effects randomly drawn from a normal distribution and assigned to random markers. Markers with effect are highlighted with dashed lines. (**b**) Significant QTCs found by QTCAT. (**c**) LASSO selection frequency for each marker during the 50 iterations of QTCAT. (**d**) Manhattan plot of the LMM analysis. The horizontal dashed line depicts the significance threshold when controlling the multiple testing with FWER, whereas the red markers are significantly associated when controlling with FDR. (**e**) The Manhattan plot of the LMM* analysis. GRM was estimated without markers on the chromosome of the actual testing position. The results are shown as in (d).

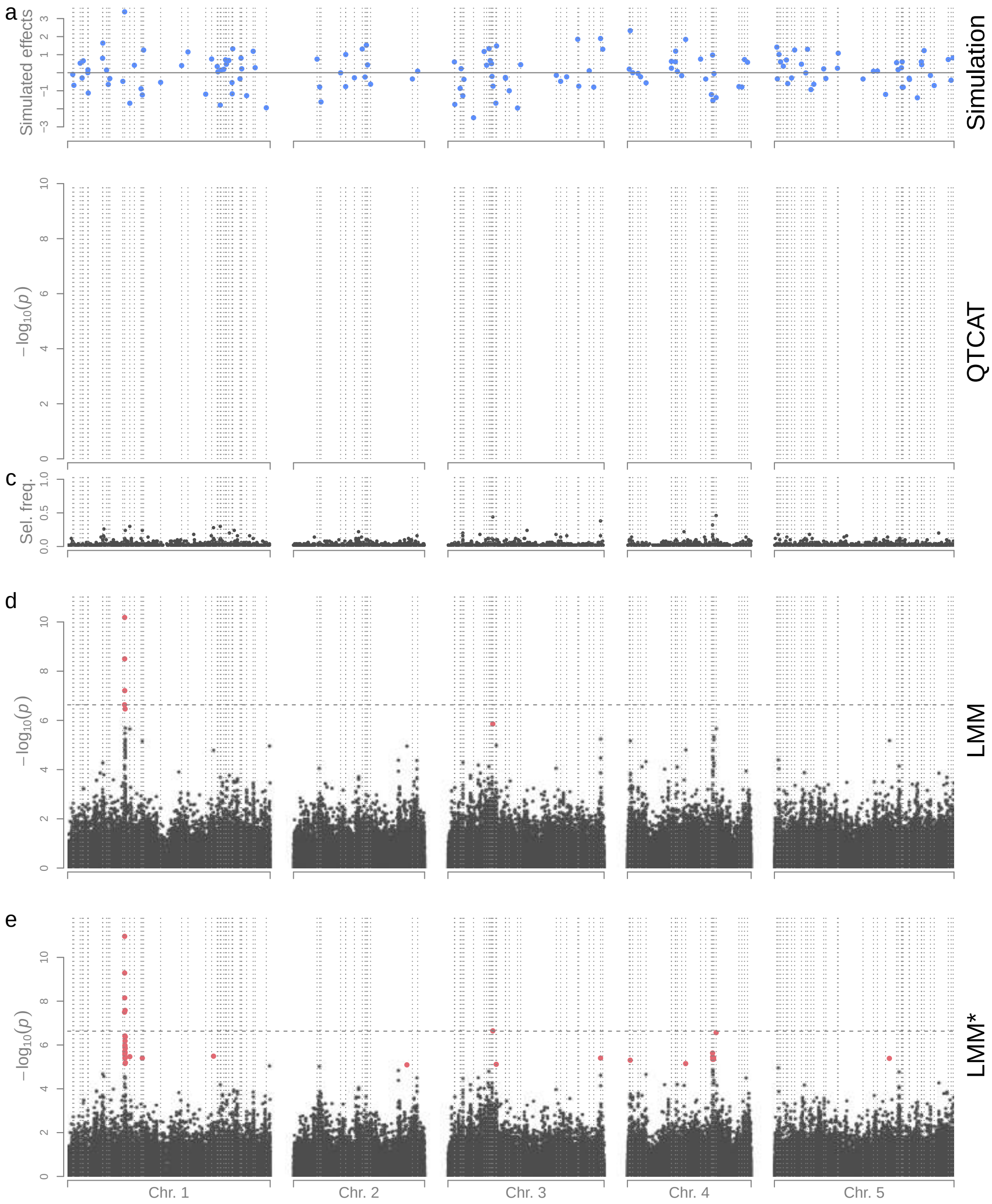

Supplementary Figure 258 Simulation of a GWA analysis based on a structured population with a heritability of 0.4 (run 58). (**a**) Simulation of 150 effects randomly drawn from a normal distribution and assigned to random markers. Markers with effect are highlighted with dashed lines. (**b**) Significant QTCs found by QTCAT. (**c**) LASSO selection frequency for each marker during the 50 iterations of QTCAT. (**d**) Manhattan plot of the LMM analysis. The horizontal dashed line depicts the significance threshold when controlling the multiple testing with FWER, whereas the red markers are significantly associated when controlling with FDR. (**e**) The Manhattan plot of the LMM* analysis. GRM was estimated without markers on the chromosome of the actual testing position. The results are shown as in (d).

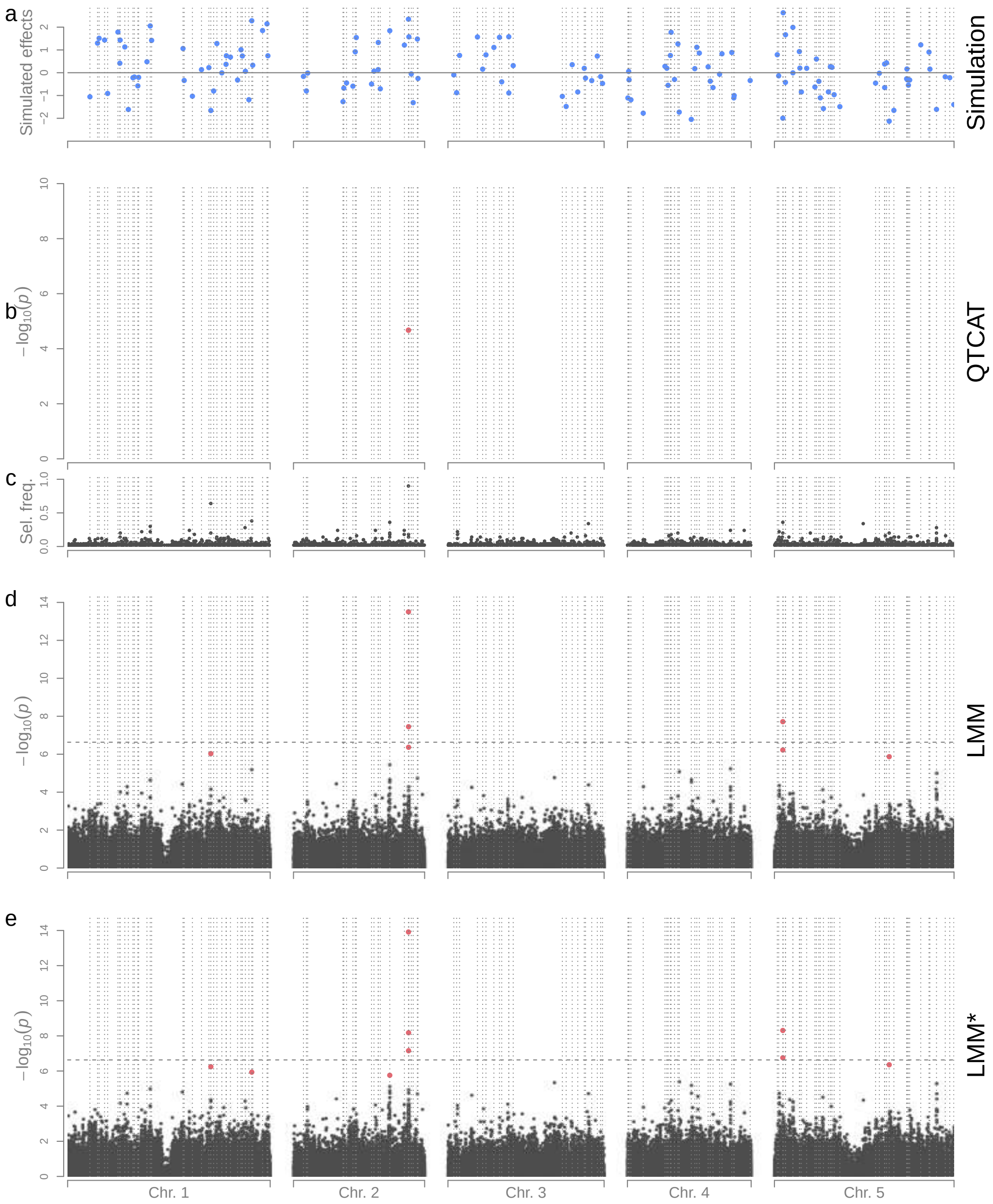

Supplementary Figure 259 Simulation of a GWA analysis based on a structured population with a heritability of 0.4 (run 59). (**a**) Simulation of 150 effects randomly drawn from a normal distribution and assigned to random markers. Markers with effect are highlighted with dashed lines. (**b**) Significant QTCs found by QTCAT. (**c**) LASSO selection frequency for each marker during the 50 iterations of QTCAT. (**d**) Manhattan plot of the LMM analysis. The horizontal dashed line depicts the significance threshold when controlling the multiple testing with FWER, whereas the red markers are significantly associated when controlling with FDR. (**e**) The Manhattan plot of the LMM* analysis. GRM was estimated without markers on the chromosome of the actual testing position. The results are shown as in (d).

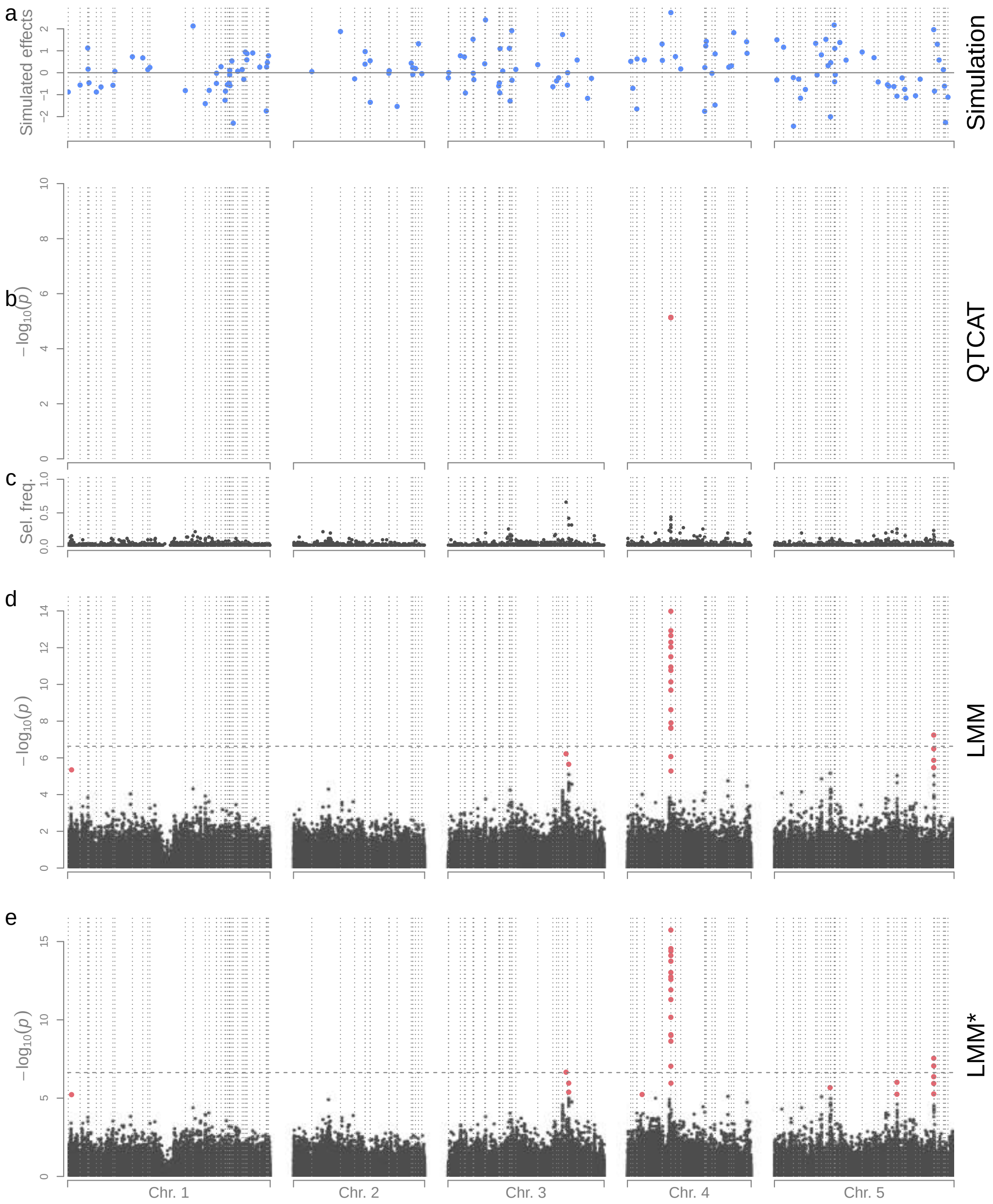

Supplementary Figure 260 Simulation of a GWA analysis based on a structured population with a heritability of 0.4 (run 60). (**a**) Simulation of 150 effects randomly drawn from a normal distribution and assigned to random markers. Markers with effect are highlighted with dashed lines. (**b**) Significant QTCs found by QTCAT. (**c**) LASSO selection frequency for each marker during the 50 iterations of QTCAT. (**d**) Manhattan plot of the LMM analysis. The horizontal dashed line depicts the significance threshold when controlling the multiple testing with FWER, whereas the red markers are significantly associated when controlling with FDR. (**e**) The Manhattan plot of the LMM* analysis. GRM was estimated without markers on the chromosome of the actual testing position. The results are shown as in (d).

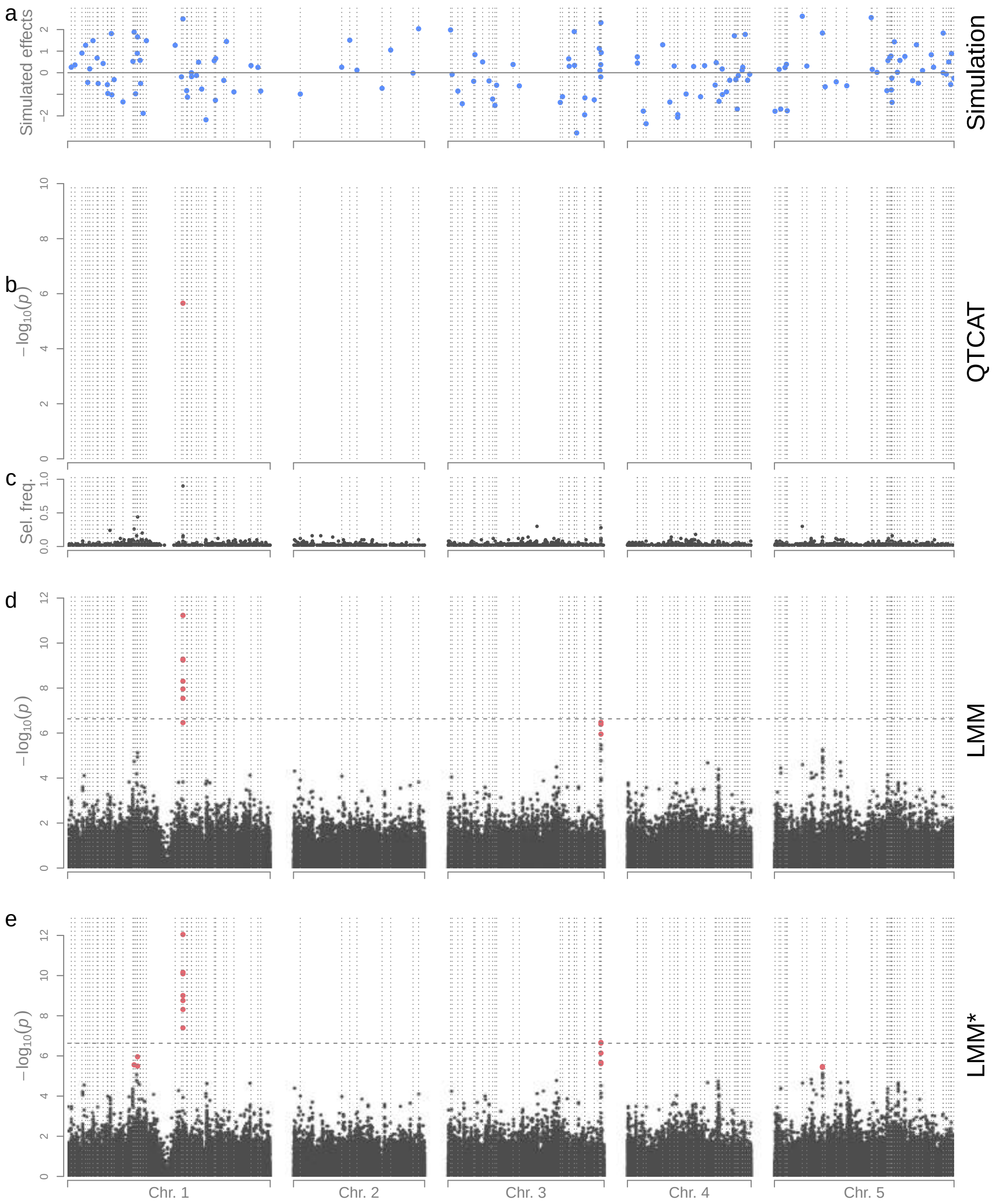

Supplementary Figure 261 Simulation of a GWA analysis based on a structured population with a heritability of 0.4 (run 61). (**a**) Simulation of 150 effects randomly drawn from a normal distribution and assigned to random markers. Markers with effect are highlighted with dashed lines. (**b**) Significant QTCs found by QTCAT. (**c**) LASSO selection frequency for each marker during the 50 iterations of QTCAT. (**d**) Manhattan plot of the LMM analysis. The horizontal dashed line depicts the significance threshold when controlling the multiple testing with FWER, whereas the red markers are significantly associated when controlling with FDR. (**e**) The Manhattan plot of the LMM* analysis. GRM was estimated without markers on the chromosome of the actual testing position. The results are shown as in (d).

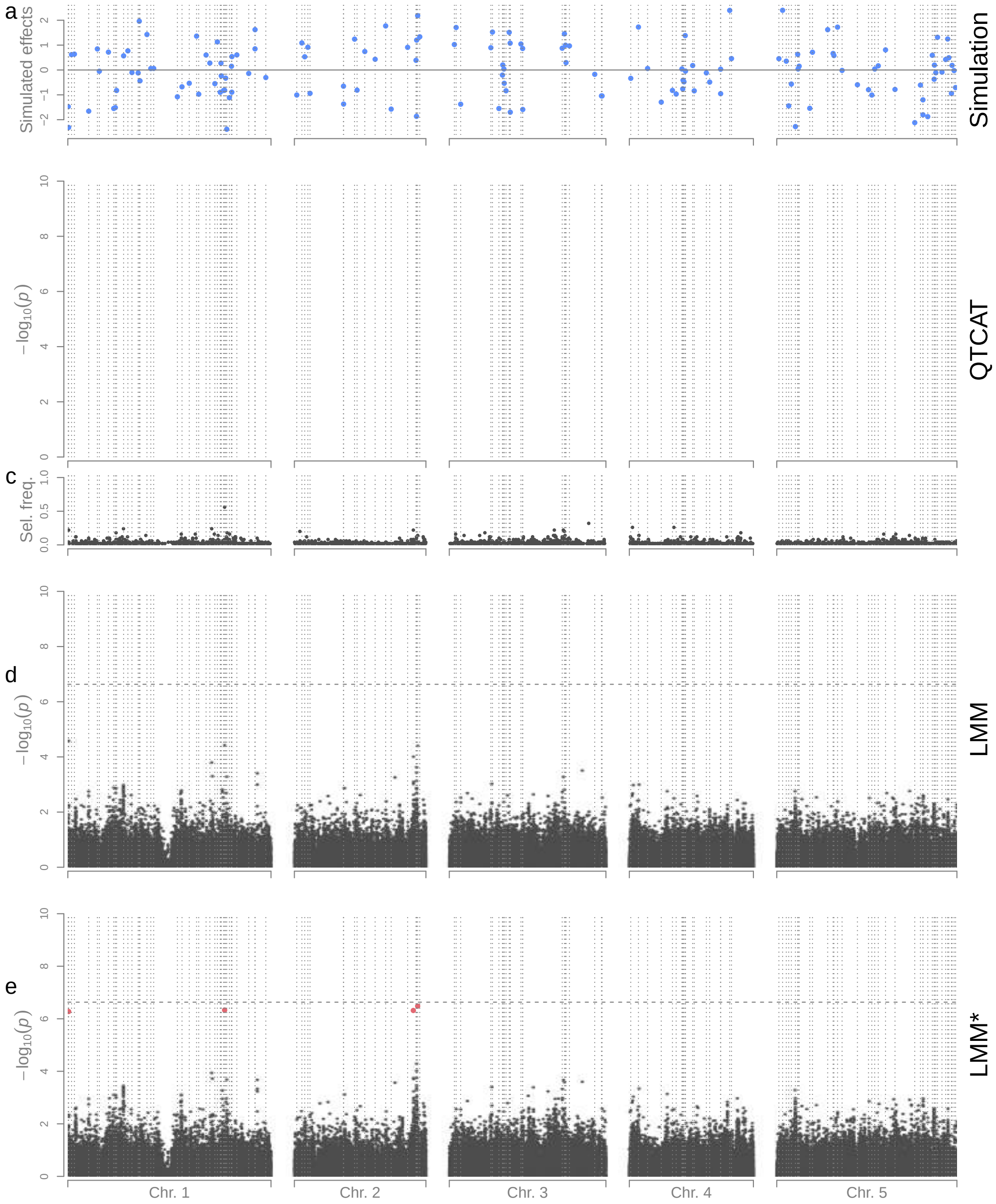

Supplementary Figure 262 Simulation of a GWA analysis based on a structured population with a heritability of 0.4 (run 62). (**a**) Simulation of 150 effects randomly drawn from a normal distribution and assigned to random markers. Markers with effect are highlighted with dashed lines. (**b**) Significant QTCs found by QTCAT. (**c**) LASSO selection frequency for each marker during the 50 iterations of QTCAT. (**d**) Manhattan plot of the LMM analysis. The horizontal dashed line depicts the significance threshold when controlling the multiple testing with FWER, whereas the red markers are significantly associated when controlling with FDR. (**e**) The Manhattan plot of the LMM* analysis. GRM was estimated without markers on the chromosome of the actual testing position. The results are shown as in (d).

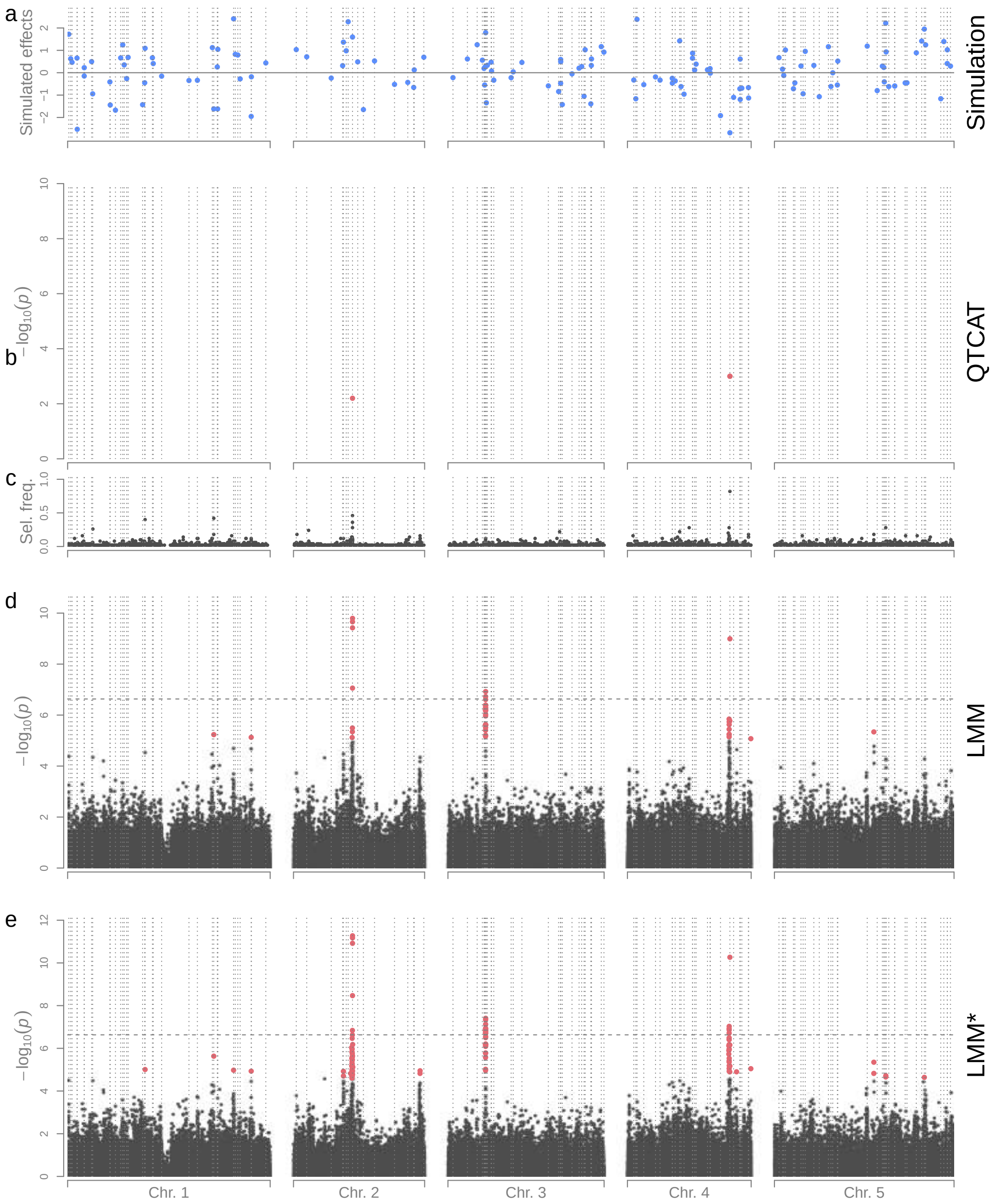

Supplementary Figure 263 Simulation of a GWA analysis based on a structured population with a heritability of 0.4 (run 63). (**a**) Simulation of 150 effects randomly drawn from a normal distribution and assigned to random markers. Markers with effect are highlighted with dashed lines. (**b**) Significant QTCs found by QTCAT. (**c**) LASSO selection frequency for each marker during the 50 iterations of QTCAT. (**d**) Manhattan plot of the LMM analysis. The horizontal dashed line depicts the significance threshold when controlling the multiple testing with FWER, whereas the red markers are significantly associated when controlling with FDR. (**e**) The Manhattan plot of the LMM* analysis. GRM was estimated without markers on the chromosome of the actual testing position. The results are shown as in (d).

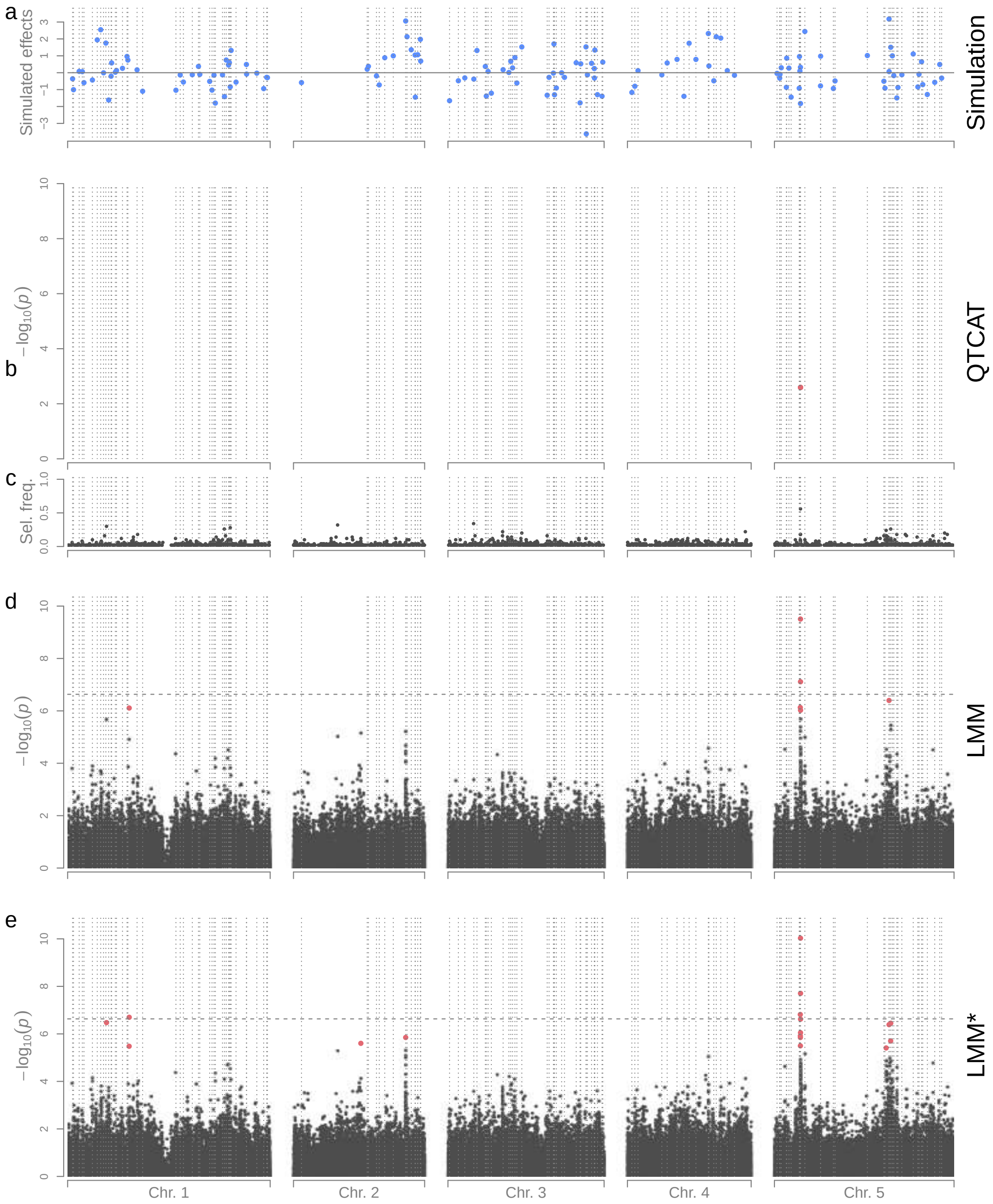

Supplementary Figure 264 Simulation of a GWA analysis based on a structured population with a heritability of 0.4 (run 64). (**a**) Simulation of 150 effects randomly drawn from a normal distribution and assigned to random markers. Markers with effect are highlighted with dashed lines. (**b**) Significant QTCs found by QTCAT. (**c**) LASSO selection frequency for each marker during the 50 iterations of QTCAT. (**d**) Manhattan plot of the LMM analysis. The horizontal dashed line depicts the significance threshold when controlling the multiple testing with FWER, whereas the red markers are significantly associated when controlling with FDR. (**e**) The Manhattan plot of the LMM* analysis. GRM was estimated without markers on the chromosome of the actual testing position. The results are shown as in (d).

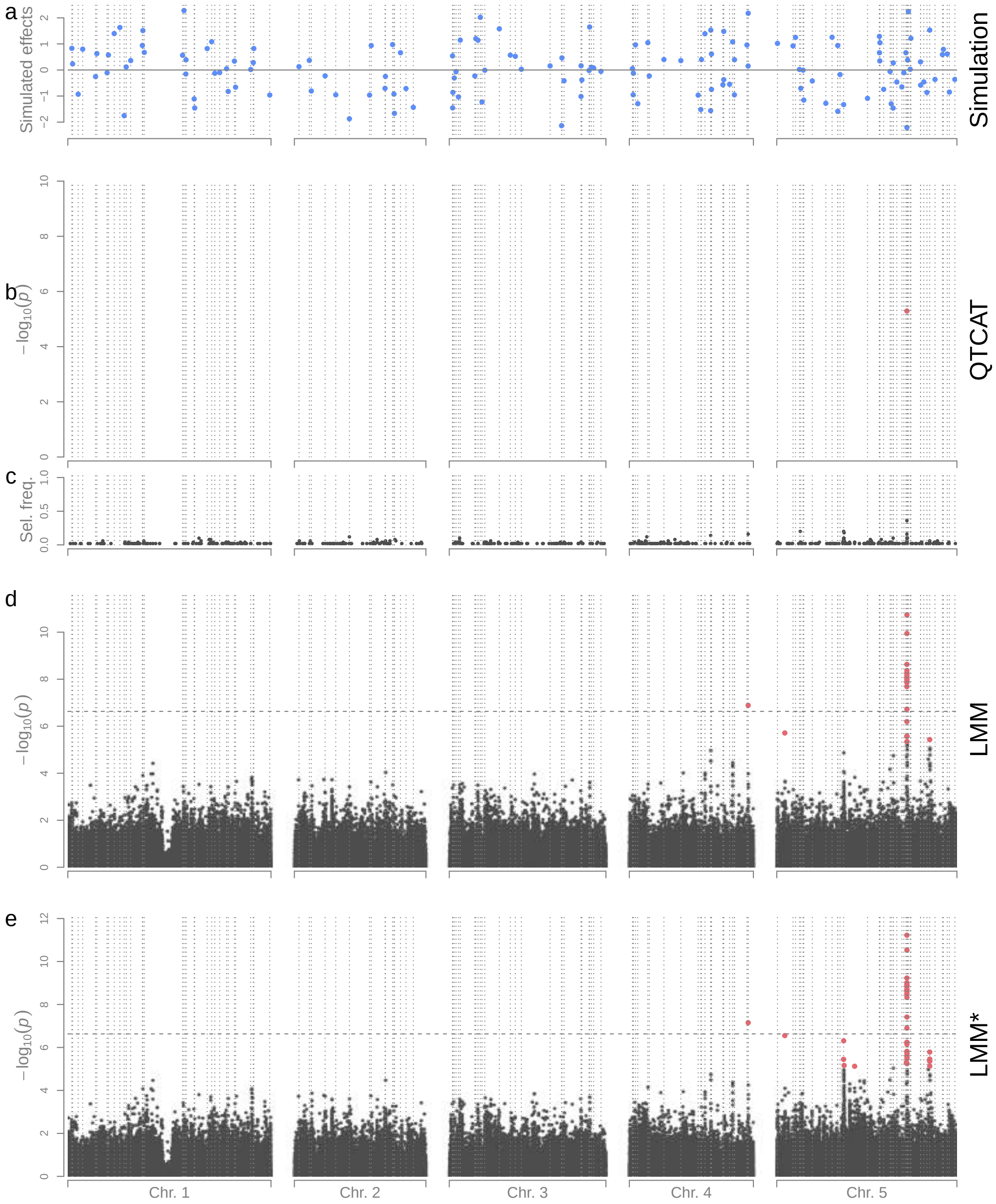

Supplementary Figure 265 Simulation of a GWA analysis based on a structured population with a heritability of 0.4 (run 65). (**a**) Simulation of 150 effects randomly drawn from a normal distribution and assigned to random markers. Markers with effect are highlighted with dashed lines. (**b**) Significant QTCs found by QTCAT. (**c**) LASSO selection frequency for each marker during the 50 iterations of QTCAT. (**d**) Manhattan plot of the LMM analysis. The horizontal dashed line depicts the significance threshold when controlling the multiple testing with FWER, whereas the red markers are significantly associated when controlling with FDR. (**e**) The Manhattan plot of the LMM* analysis. GRM was estimated without markers on the chromosome of the actual testing position. The results are shown as in (d).

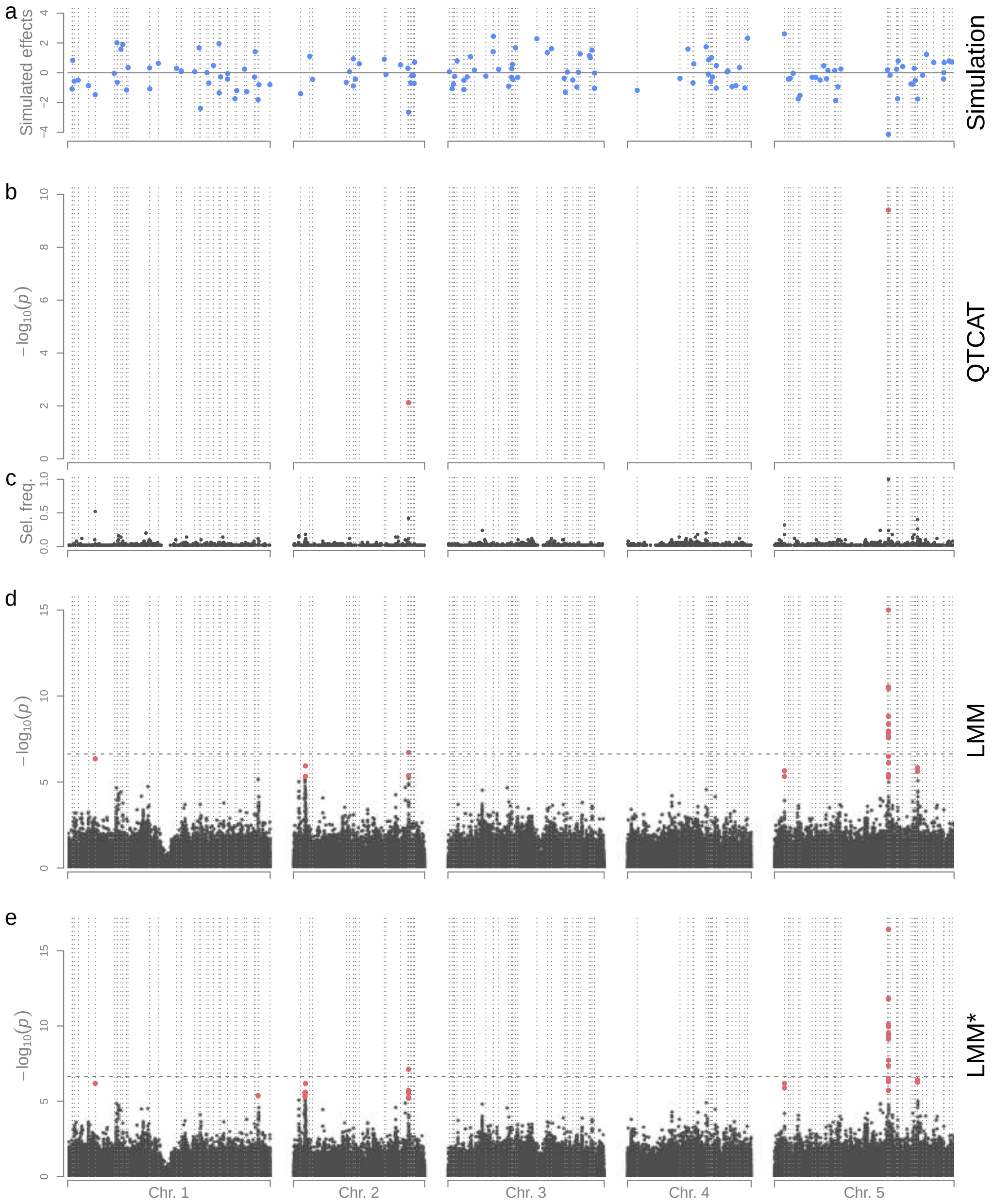

Supplementary Figure 266 Simulation of a GWA analysis based on a structured population with a heritability of 0.4 (run 66). (**a**) Simulation of 150 effects randomly drawn from a normal distribution and assigned to random markers. Markers with effect are highlighted with dashed lines. (**b**) Significant QTCs found by QTCAT. (**c**) LASSO selection frequency for each marker during the 50 iterations of QTCAT. (**d**) Manhattan plot of the LMM analysis. The horizontal dashed line depicts the significance threshold when controlling the multiple testing with FWER, whereas the red markers are significantly associated when controlling with FDR. (**e**) The Manhattan plot of the LMM* analysis. GRM was estimated without markers on the chromosome of the actual testing position. The results are shown as in (d).

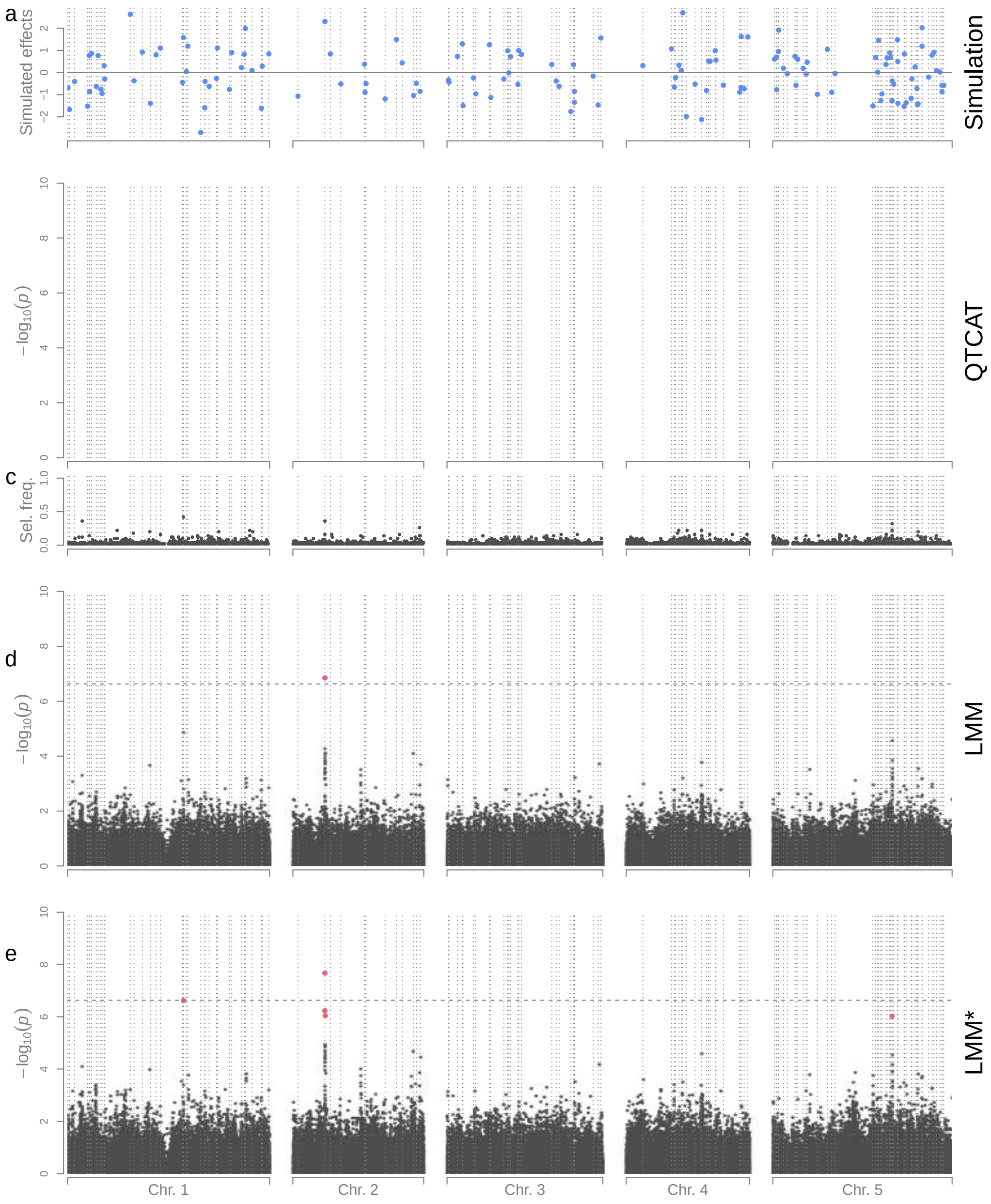

Supplementary Figure 267 Simulation of a GWA analysis based on a structured population with a heritability of 0.4 (run 67). (**a**) Simulation of 150 effects randomly drawn from a normal distribution and assigned to random markers. Markers with effect are highlighted with dashed lines. (**b**) Significant QTCs found by QTCAT. (**c**) LASSO selection frequency for each marker during the 50 iterations of QTCAT. (**d**) Manhattan plot of the LMM analysis. The horizontal dashed line depicts the significance threshold when controlling the multiple testing with FWER, whereas the red markers are significantly associated when controlling with FDR. (**e**) The Manhattan plot of the LMM* analysis. GRM was estimated without markers on the chromosome of the actual testing position. The results are shown as in (d).

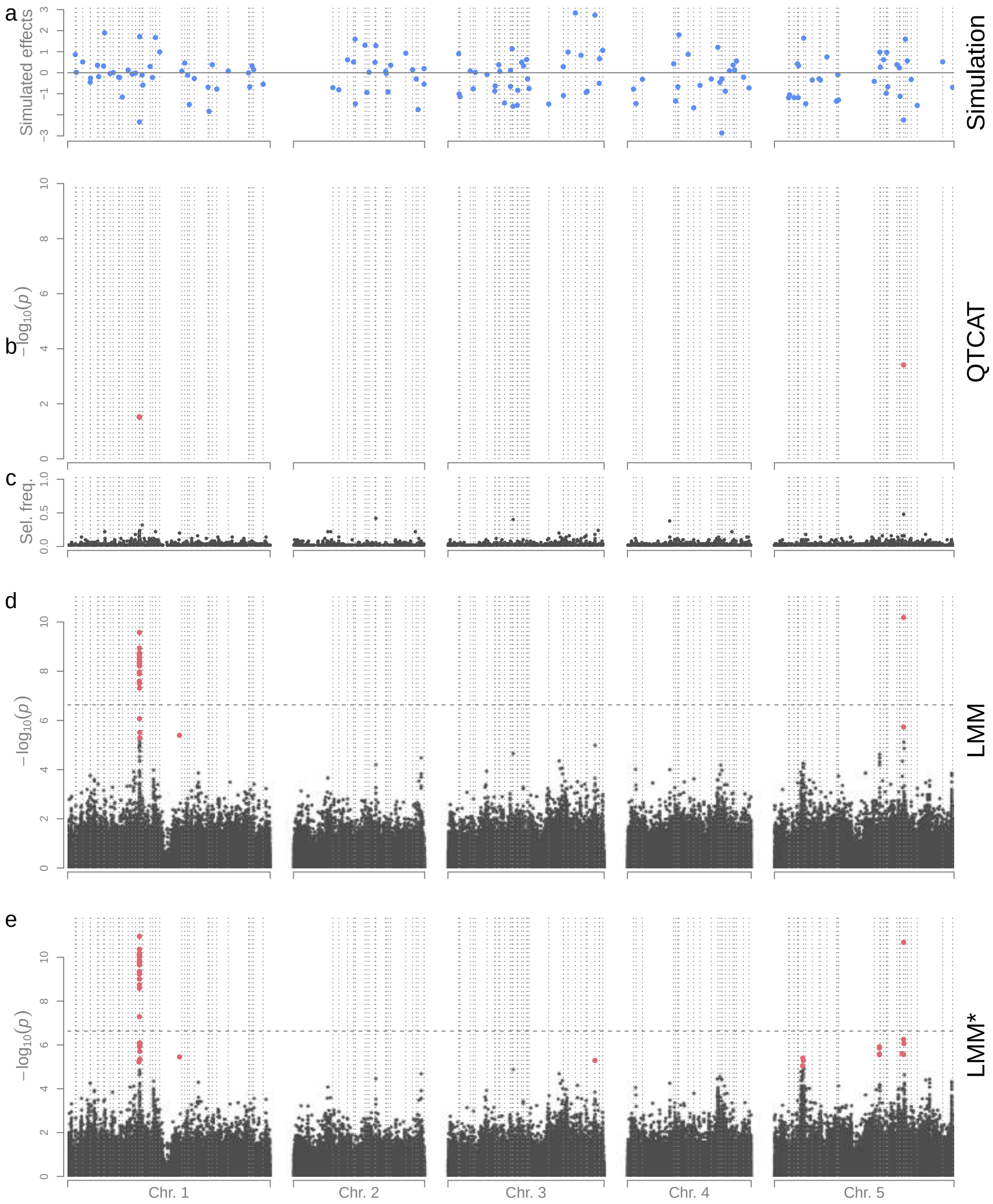

Supplementary Figure 268 Simulation of a GWA analysis based on a structured population with a heritability of 0.4 (run 68). (**a**) Simulation of 150 effects randomly drawn from a normal distribution and assigned to random markers. Markers with effect are highlighted with dashed lines. (**b**) Significant QTCs found by QTCAT. (**c**) LASSO selection frequency for each marker during the 50 iterations of QTCAT. (**d**) Manhattan plot of the LMM analysis. The horizontal dashed line depicts the significance threshold when controlling the multiple testing with FWER, whereas the red markers are significantly associated when controlling with FDR. (**e**) The Manhattan plot of the LMM* analysis. GRM was estimated without markers on the chromosome of the actual testing position. The results are shown as in (d).

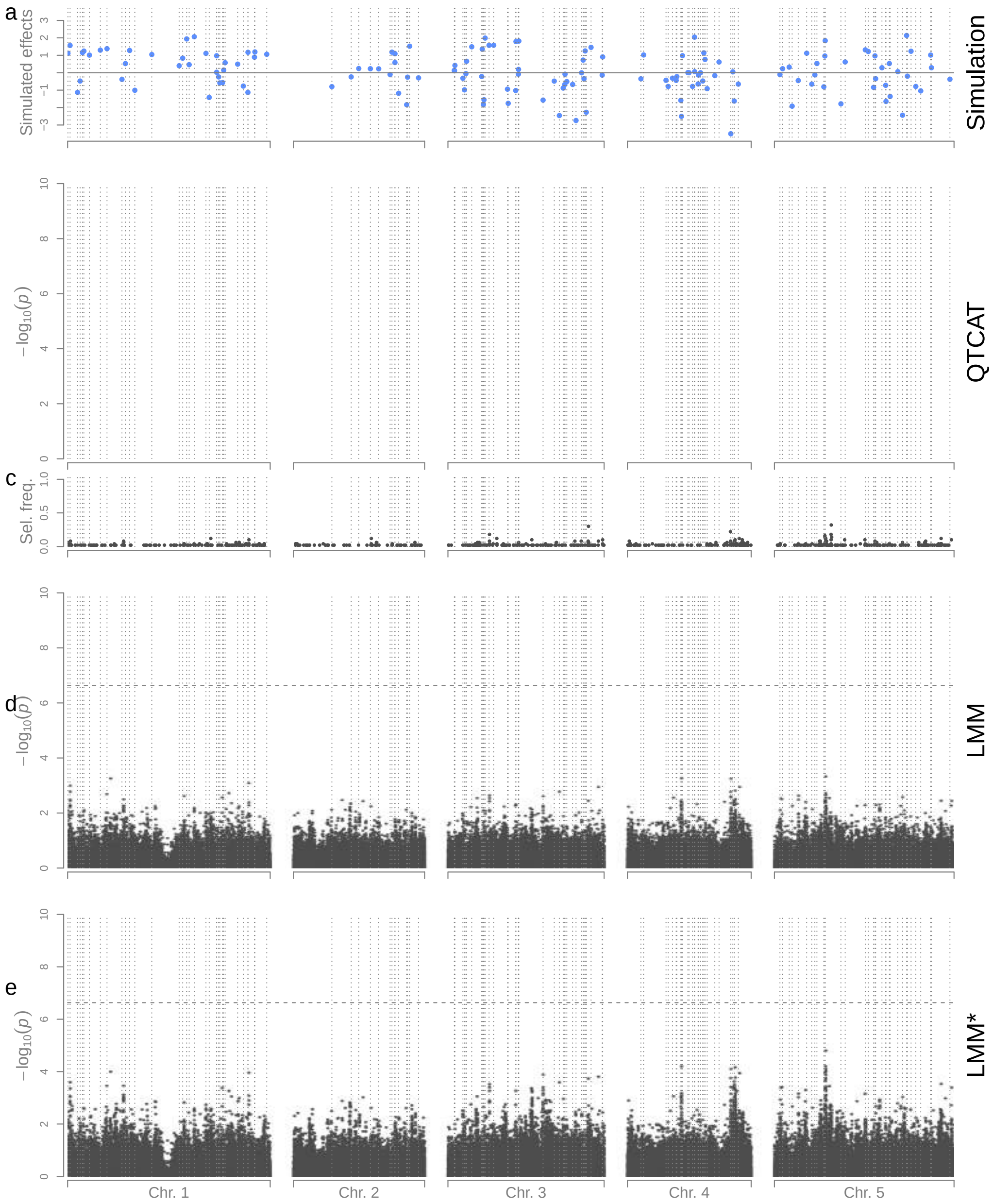

Supplementary Figure 269 Simulation of a GWA analysis based on a structured population with a heritability of 0.4 (run 69). (**a**) Simulation of 150 effects randomly drawn from a normal distribution and assigned to random markers. Markers with effect are highlighted with dashed lines. (**b**) Significant QTCs found by QTCAT. (**c**) LASSO selection frequency for each marker during the 50 iterations of QTCAT. (**d**) Manhattan plot of the LMM analysis. The horizontal dashed line depicts the significance threshold when controlling the multiple testing with FWER, whereas the red markers are significantly associated when controlling with FDR. (**e**) The Manhattan plot of the LMM* analysis. GRM was estimated without markers on the chromosome of the actual testing position. The results are shown as in (d).

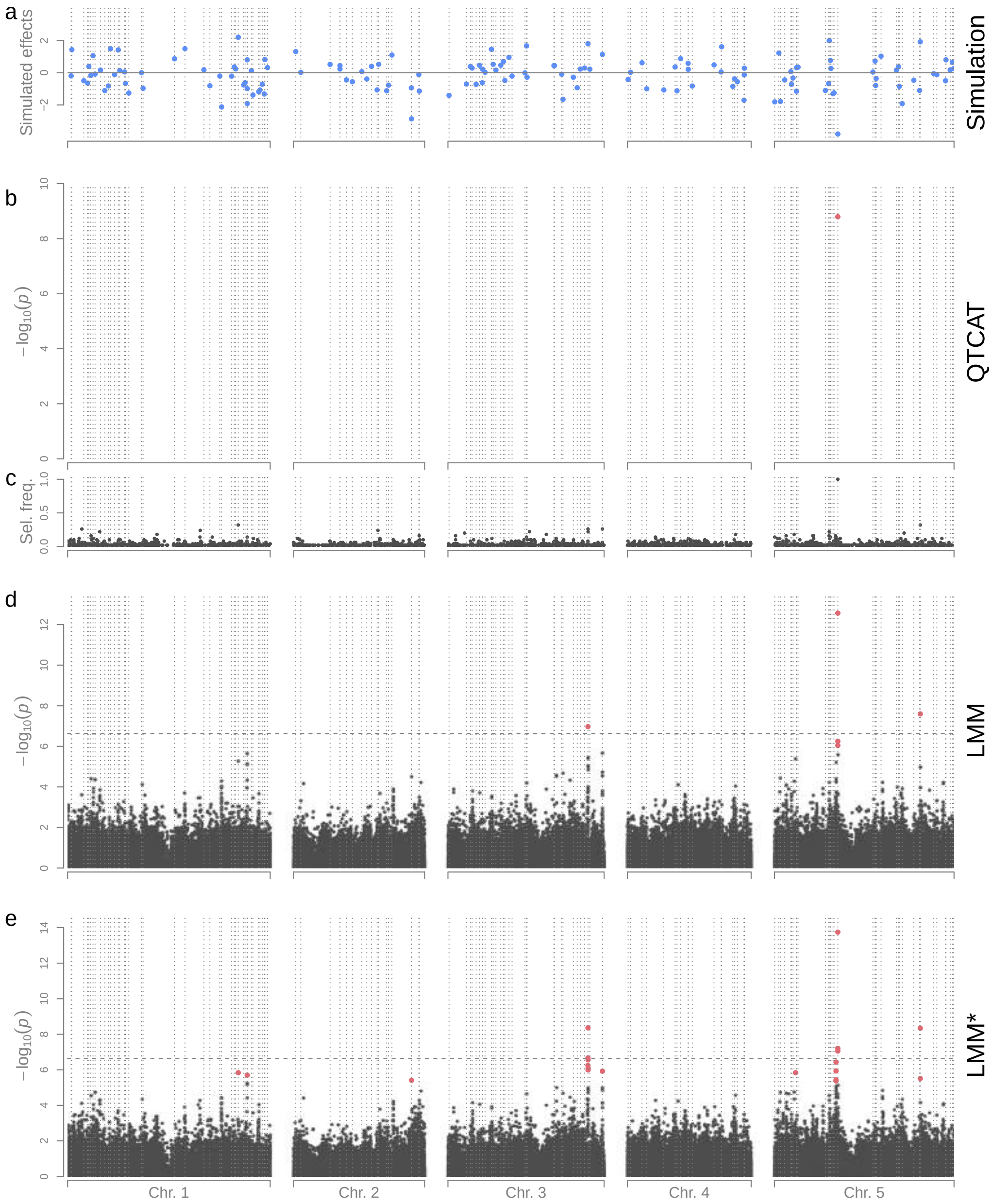

Supplementary Figure 270 Simulation of a GWA analysis based on a structured population with a heritability of 0.4 (run 70). (**a**) Simulation of 150 effects randomly drawn from a normal distribution and assigned to random markers. Markers with effect are highlighted with dashed lines. (**b**) Significant QTCs found by QTCAT. (**c**) LASSO selection frequency for each marker during the 50 iterations of QTCAT. (**d**) Manhattan plot of the LMM analysis. The horizontal dashed line depicts the significance threshold when controlling the multiple testing with FWER, whereas the red markers are significantly associated when controlling with FDR. (**e**) The Manhattan plot of the LMM* analysis. GRM was estimated without markers on the chromosome of the actual testing position. The results are shown as in (d).

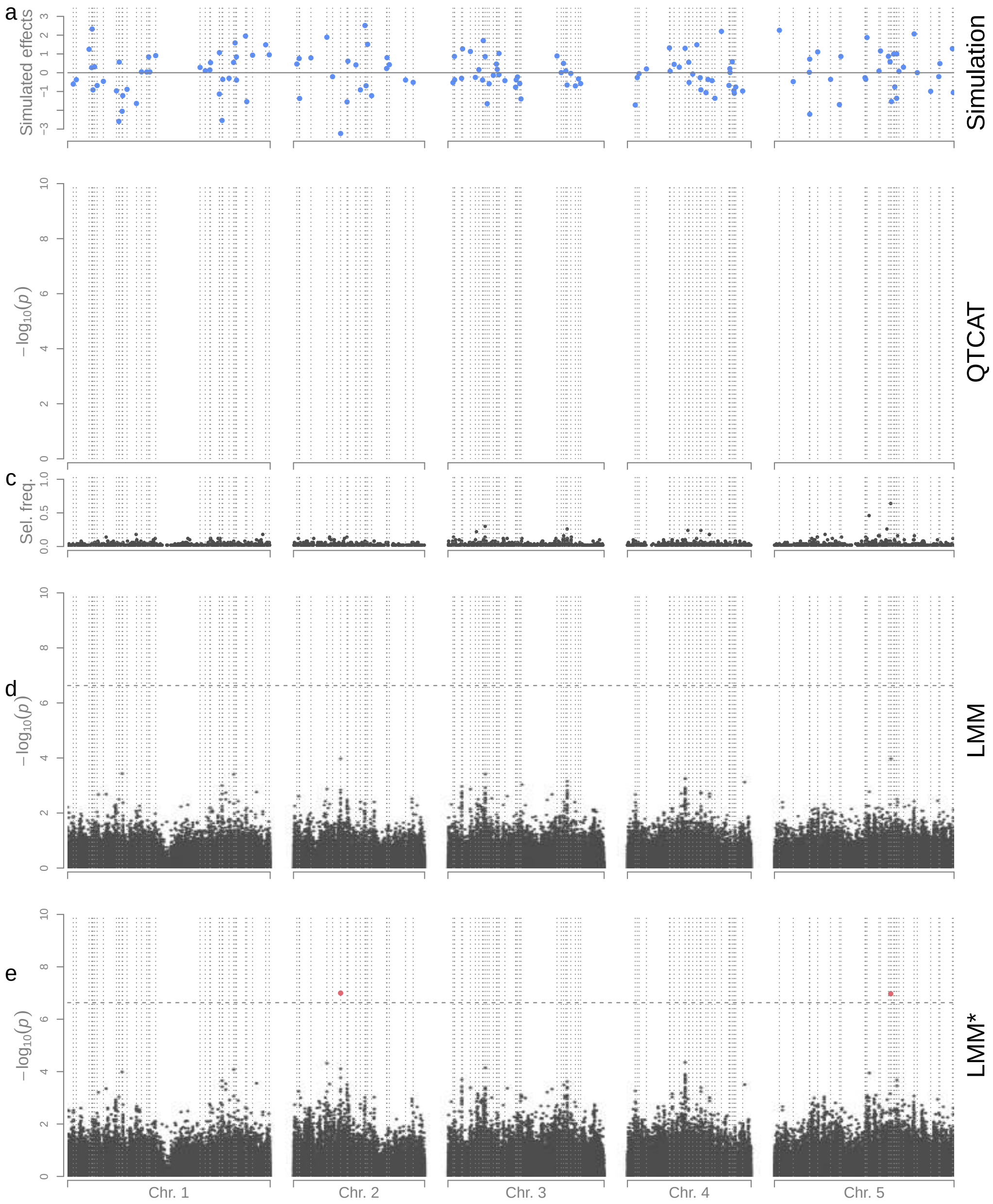

Supplementary Figure 271 Simulation of a GWA analysis based on a structured population with a heritability of 0.4 (run 71). (**a**) Simulation of 150 effects randomly drawn from a normal distribution and assigned to random markers. Markers with effect are highlighted with dashed lines. (**b**) Significant QTCs found by QTCAT. (**c**) LASSO selection frequency for each marker during the 50 iterations of QTCAT. (**d**) Manhattan plot of the LMM analysis. The horizontal dashed line depicts the significance threshold when controlling the multiple testing with FWER, whereas the red markers are significantly associated when controlling with FDR. (**e**) The Manhattan plot of the LMM* analysis. GRM was estimated without markers on the chromosome of the actual testing position. The results are shown as in (d).

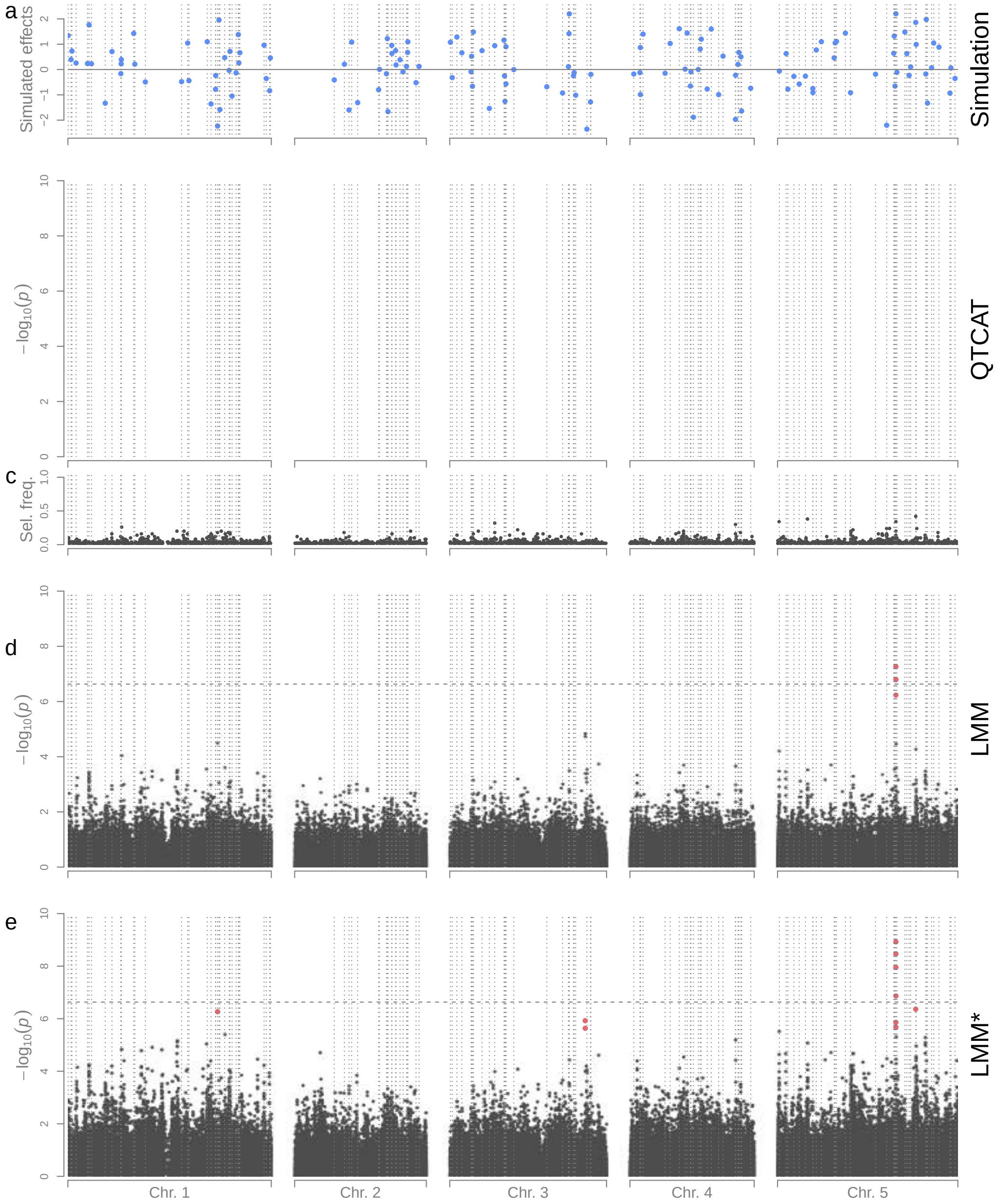

Supplementary Figure 272 Simulation of a GWA analysis based on a structured population with a heritability of 0.4 (run 72). (**a**) Simulation of 150 effects randomly drawn from a normal distribution and assigned to random markers. Markers with effect are highlighted with dashed lines. (**b**) Significant QTCs found by QTCAT. (**c**) LASSO selection frequency for each marker during the 50 iterations of QTCAT. (**d**) Manhattan plot of the LMM analysis. The horizontal dashed line depicts the significance threshold when controlling the multiple testing with FWER, whereas the red markers are significantly associated when controlling with FDR. (**e**) The Manhattan plot of the LMM* analysis. GRM was estimated without markers on the chromosome of the actual testing position. The results are shown as in (d).

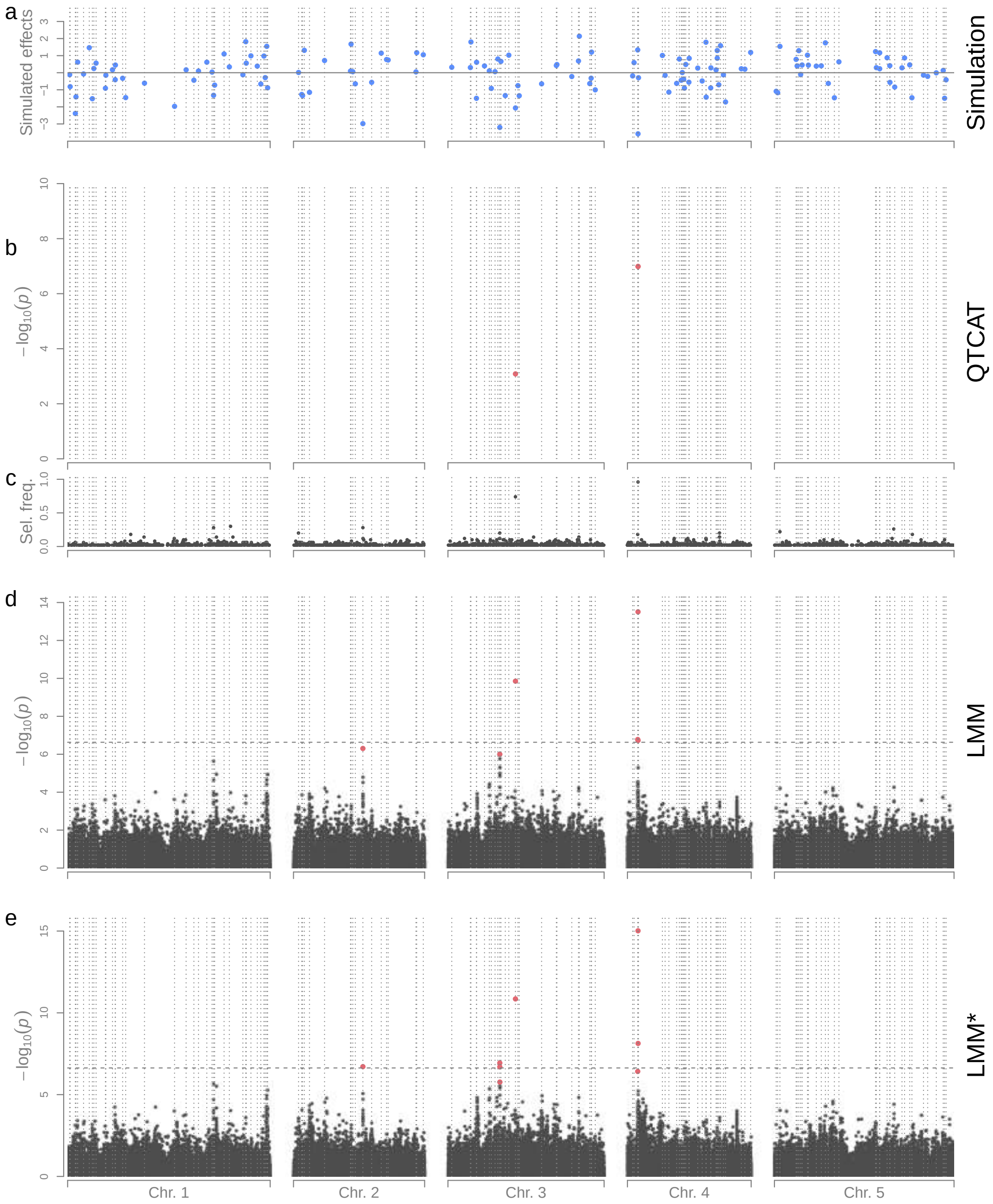

Supplementary Figure 273 Simulation of a GWA analysis based on a structured population with a heritability of 0.4 (run 73). (**a**) Simulation of 150 effects randomly drawn from a normal distribution and assigned to random markers. Markers with effect are highlighted with dashed lines. (**b**) Significant QTCs found by QTCAT. (**c**) LASSO selection frequency for each marker during the 50 iterations of QTCAT. (**d**) Manhattan plot of the LMM analysis. The horizontal dashed line depicts the significance threshold when controlling the multiple testing with FWER, whereas the red markers are significantly associated when controlling with FDR. (**e**) The Manhattan plot of the LMM* analysis. GRM was estimated without markers on the chromosome of the actual testing position. The results are shown as in (d).

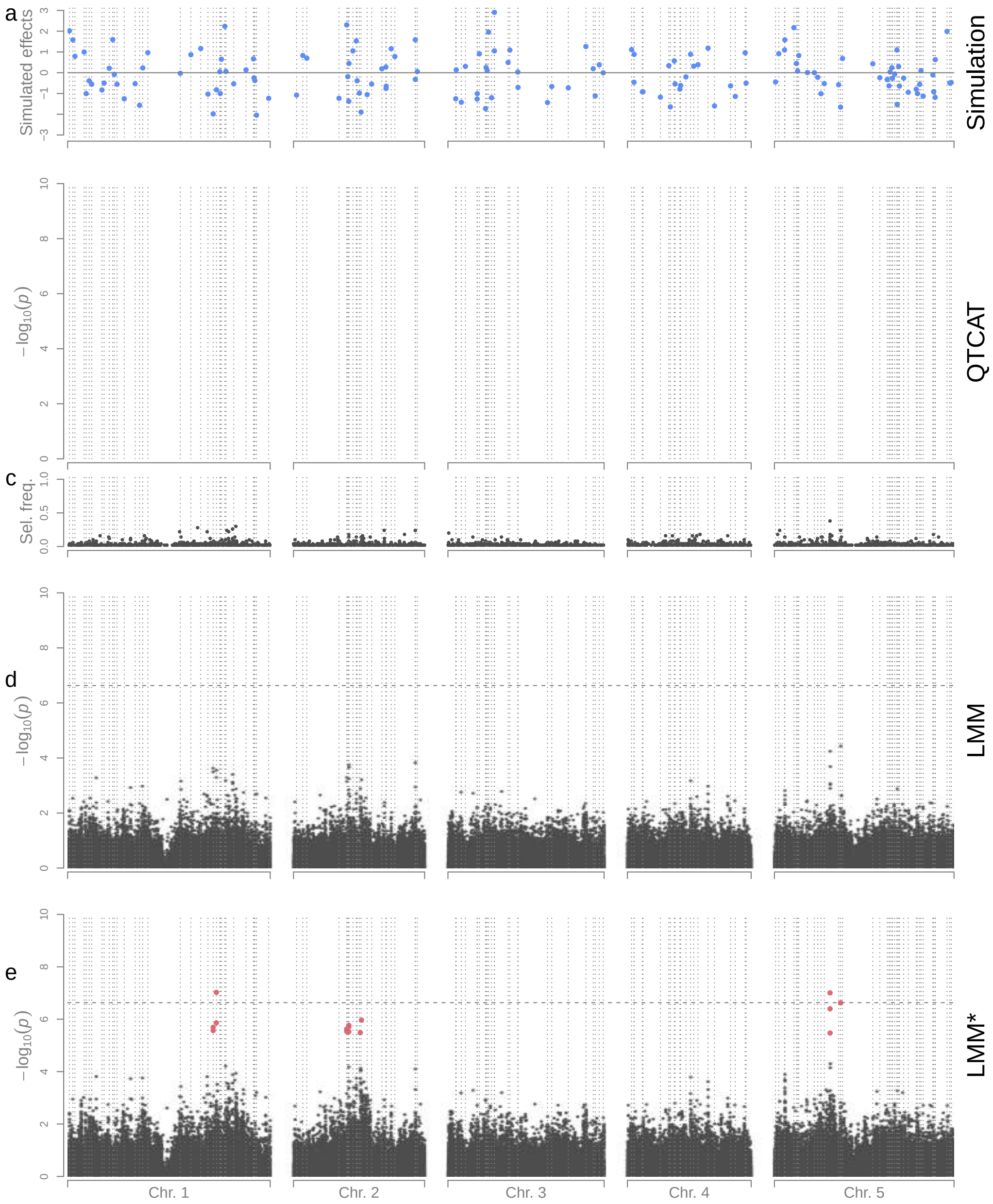

Supplementary Figure 274 Simulation of a GWA analysis based on a structured population with a heritability of 0.4 (run 74). (**a**) Simulation of 150 effects randomly drawn from a normal distribution and assigned to random markers. Markers with effect are highlighted with dashed lines. (**b**) Significant QTCs found by QTCAT. (**c**) LASSO selection frequency for each marker during the 50 iterations of QTCAT. (**d**) Manhattan plot of the LMM analysis. The horizontal dashed line depicts the significance threshold when controlling the multiple testing with FWER, whereas the red markers are significantly associated when controlling with FDR. (**e**) The Manhattan plot of the LMM* analysis. GRM was estimated without markers on the chromosome of the actual testing position. The results are shown as in (d).

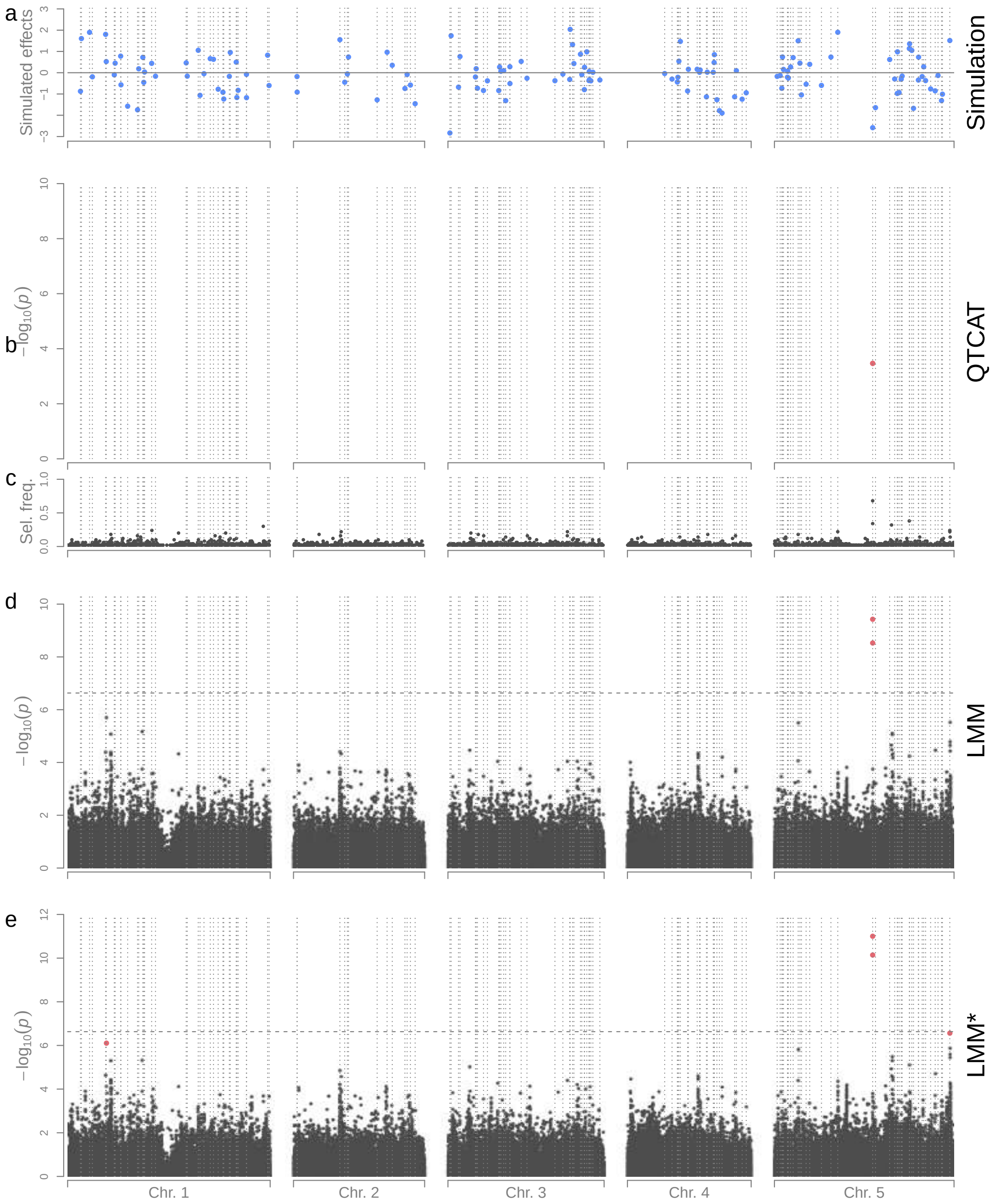

Supplementary Figure 275 Simulation of a GWA analysis based on a structured population with a heritability of 0.4 (run 75). (**a**) Simulation of 150 effects randomly drawn from a normal distribution and assigned to random markers. Markers with effect are highlighted with dashed lines. (**b**) Significant QTCs found by QTCAT. (**c**) LASSO selection frequency for each marker during the 50 iterations of QTCAT. (**d**) Manhattan plot of the LMM analysis. The horizontal dashed line depicts the significance threshold when controlling the multiple testing with FWER, whereas the red markers are significantly associated when controlling with FDR. (**e**) The Manhattan plot of the LMM* analysis. GRM was estimated without markers on the chromosome of the actual testing position. The results are shown as in (d).

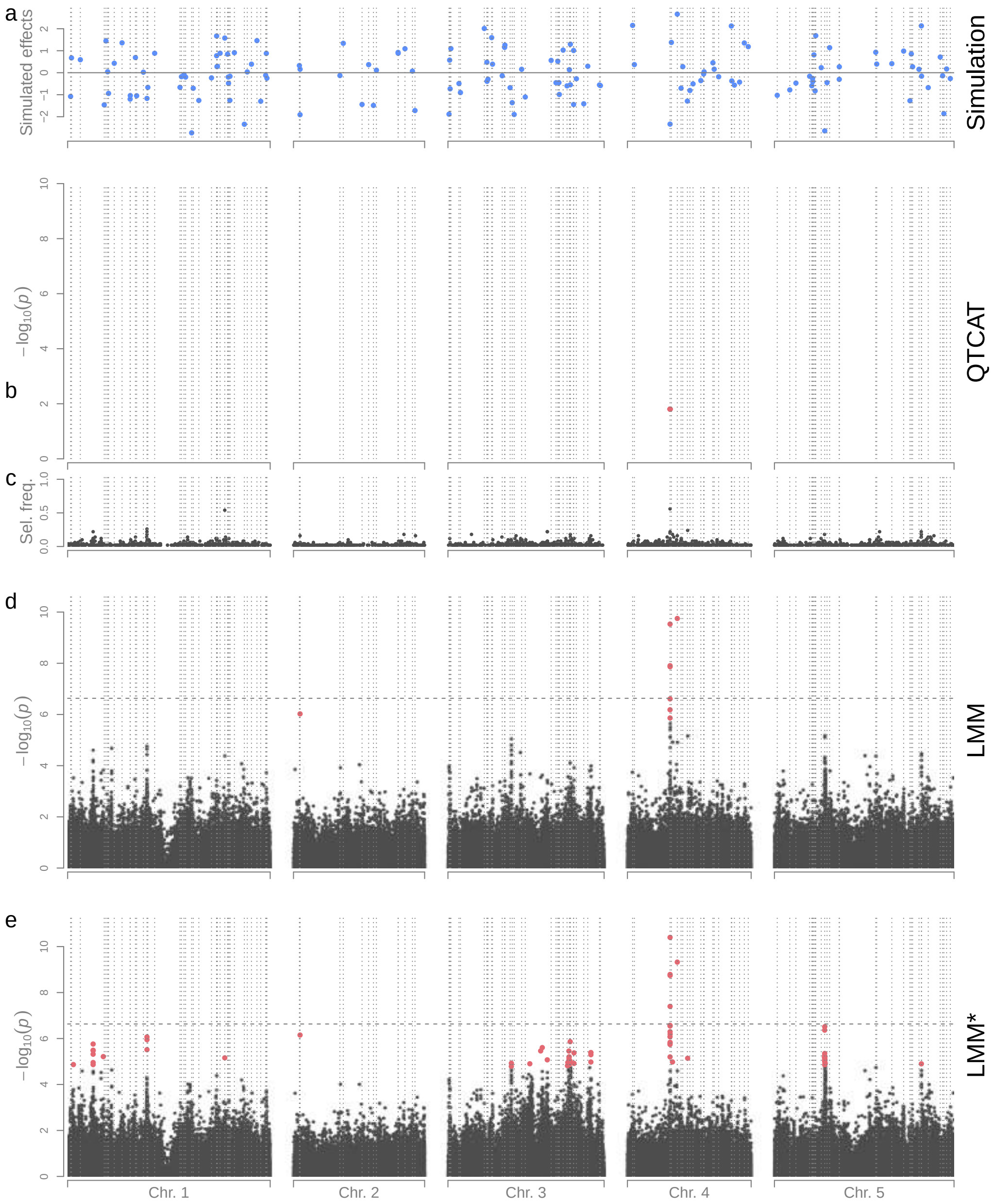

Supplementary Figure 276 Simulation of a GWA analysis based on a structured population with a heritability of 0.4 (run 76). (**a**) Simulation of 150 effects randomly drawn from a normal distribution and assigned to random markers. Markers with effect are highlighted with dashed lines. (**b**) Significant QTCs found by QTCAT. (**c**) LASSO selection frequency for each marker during the 50 iterations of QTCAT. (**d**) Manhattan plot of the LMM analysis. The horizontal dashed line depicts the significance threshold when controlling the multiple testing with FWER, whereas the red markers are significantly associated when controlling with FDR. (**e**) The Manhattan plot of the LMM* analysis. GRM was estimated without markers on the chromosome of the actual testing position. The results are shown as in (d).

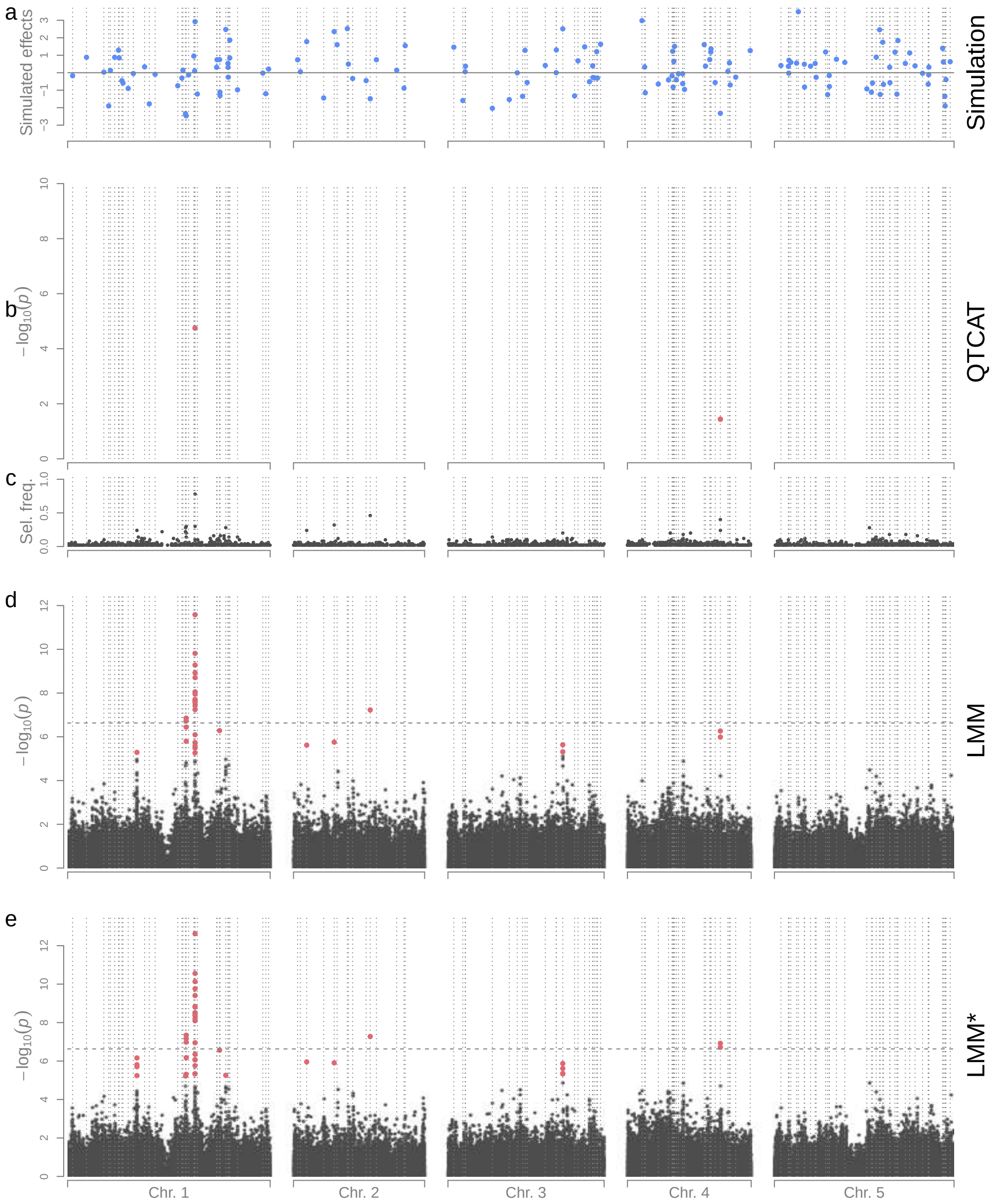

Supplementary Figure 277 Simulation of a GWA analysis based on a structured population with a heritability of 0.4 (run 77). (**a**) Simulation of 150 effects randomly drawn from a normal distribution and assigned to random markers. Markers with effect are highlighted with dashed lines. (**b**) Significant QTCs found by QTCAT. (**c**) LASSO selection frequency for each marker during the 50 iterations of QTCAT. (**d**) Manhattan plot of the LMM analysis. The horizontal dashed line depicts the significance threshold when controlling the multiple testing with FWER, whereas the red markers are significantly associated when controlling with FDR. (**e**) The Manhattan plot of the LMM* analysis. GRM was estimated without markers on the chromosome of the actual testing position. The results are shown as in (d).

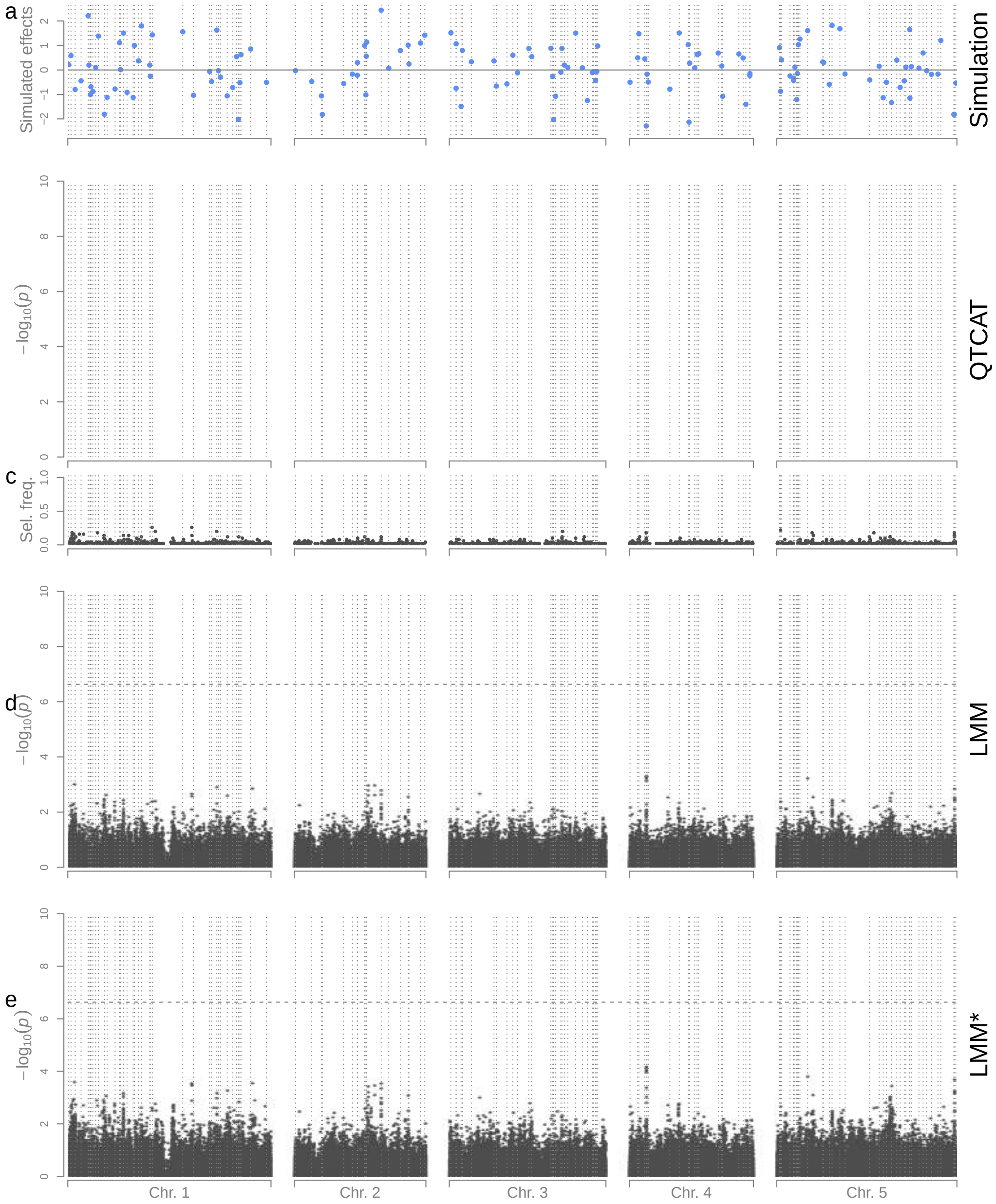

Supplementary Figure 278 Simulation of a GWA analysis based on a structured population with a heritability of 0.4 (run 78). (**a**) Simulation of 150 effects randomly drawn from a normal distribution and assigned to random markers. Markers with effect are highlighted with dashed lines. (**b**) Significant QTCs found by QTCAT. (**c**) LASSO selection frequency for each marker during the 50 iterations of QTCAT. (**d**) Manhattan plot of the LMM analysis. The horizontal dashed line depicts the significance threshold when controlling the multiple testing with FWER, whereas the red markers are significantly associated when controlling with FDR. (**e**) The Manhattan plot of the LMM* analysis. GRM was estimated without markers on the chromosome of the actual testing position. The results are shown as in (d).

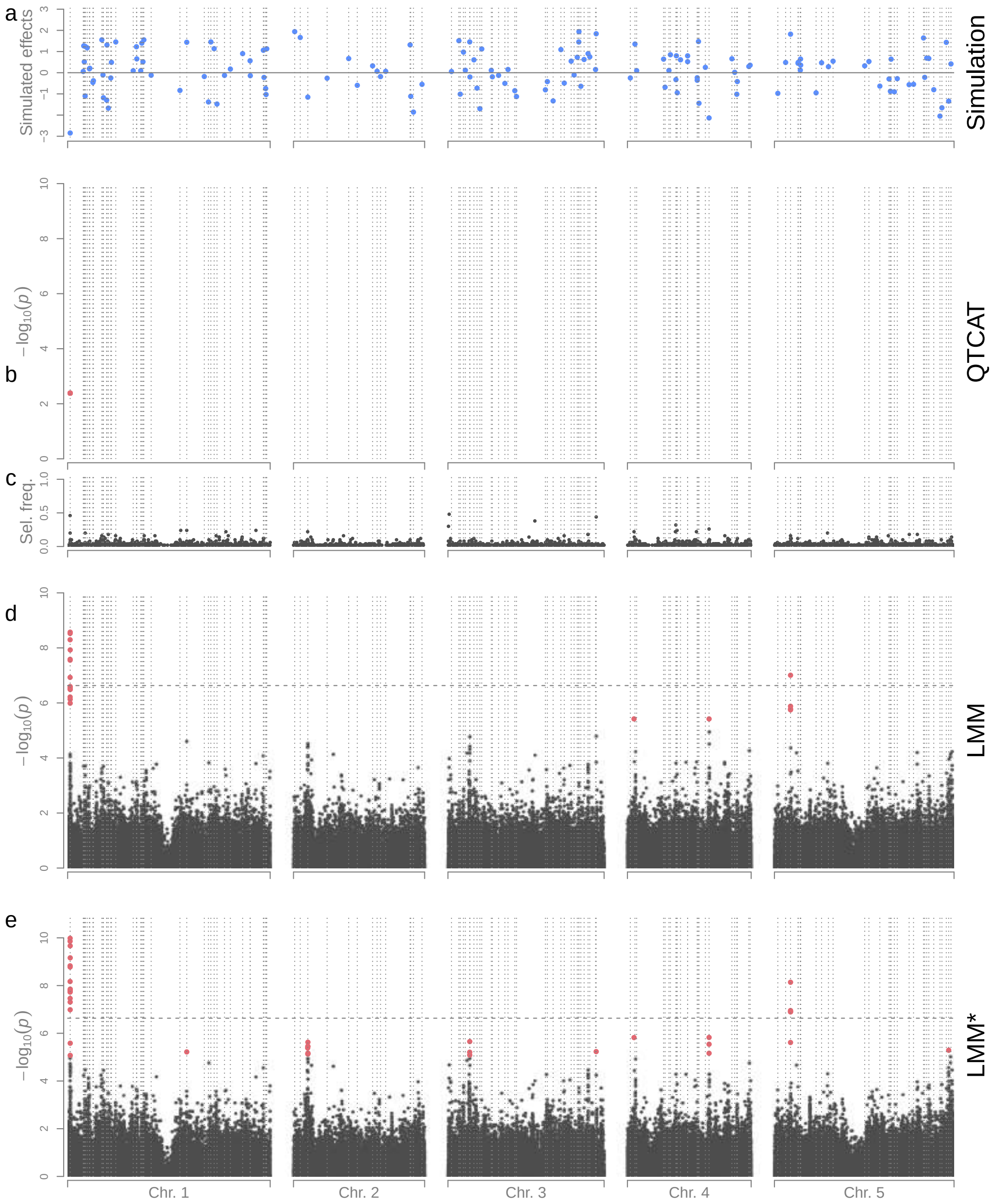

Supplementary Figure 279 Simulation of a GWA analysis based on a structured population with a heritability of 0.4 (run 79). (**a**) Simulation of 150 effects randomly drawn from a normal distribution and assigned to random markers. Markers with effect are highlighted with dashed lines. (**b**) Significant QTCs found by QTCAT. (**c**) LASSO selection frequency for each marker during the 50 iterations of QTCAT. (**d**) Manhattan plot of the LMM analysis. The horizontal dashed line depicts the significance threshold when controlling the multiple testing with FWER, whereas the red markers are significantly associated when controlling with FDR. (**e**) The Manhattan plot of the LMM* analysis. GRM was estimated without markers on the chromosome of the actual testing position. The results are shown as in (d).

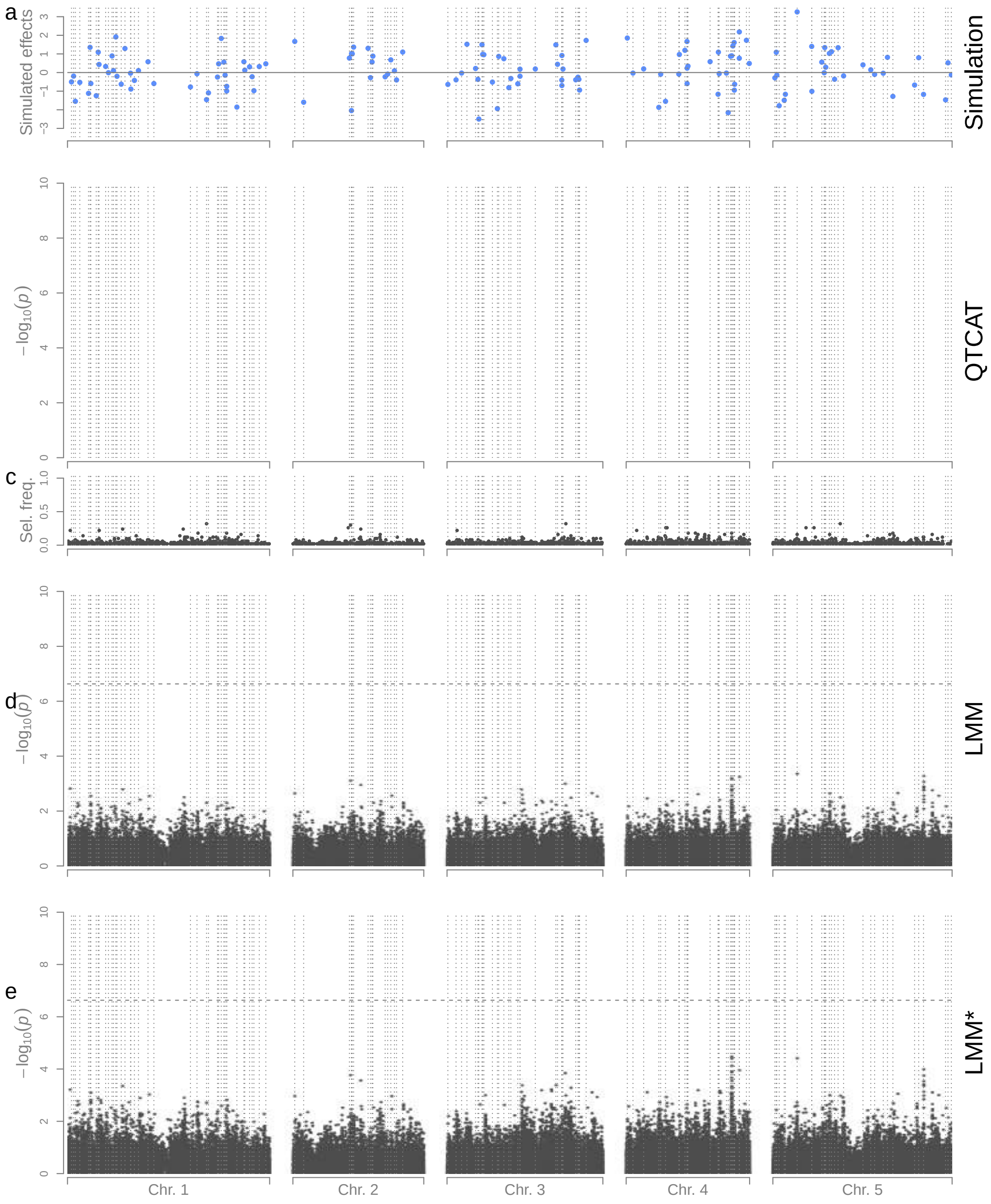

Supplementary Figure 280 Simulation of a GWA analysis based on a structured population with a heritability of 0.4 (run 80). (**a**) Simulation of 150 effects randomly drawn from a normal distribution and assigned to random markers. Markers with effect are highlighted with dashed lines. (**b**) Significant QTCs found by QTCAT. (**c**) LASSO selection frequency for each marker during the 50 iterations of QTCAT. (**d**) Manhattan plot of the LMM analysis. The horizontal dashed line depicts the significance threshold when controlling the multiple testing with FWER, whereas the red markers are significantly associated when controlling with FDR. (**e**) The Manhattan plot of the LMM* analysis. GRM was estimated without markers on the chromosome of the actual testing position. The results are shown as in (d).

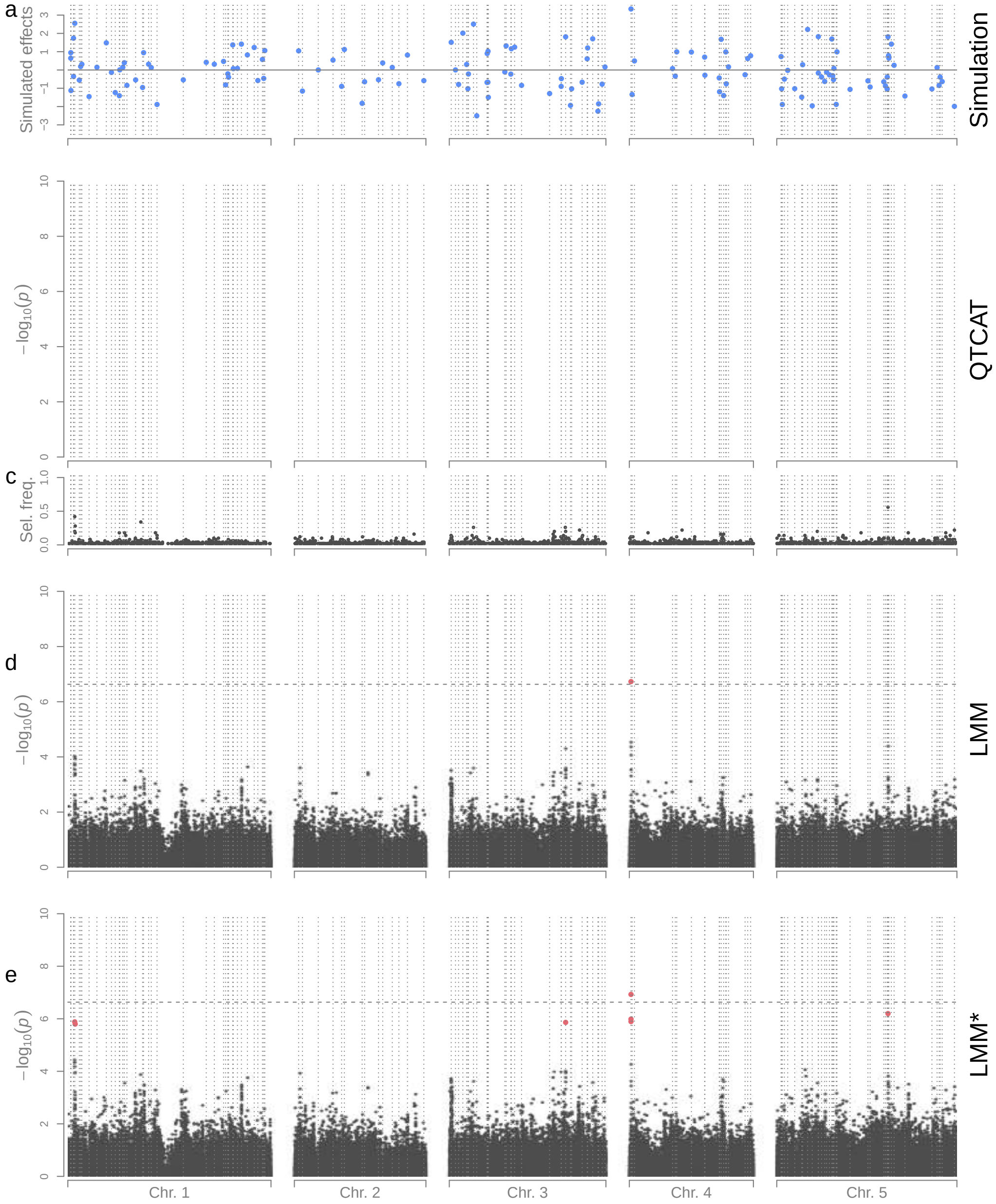

Supplementary Figure 281 Simulation of a GWA analysis based on a structured population with a heritability of 0.4 (run 81). (**a**) Simulation of 150 effects randomly drawn from a normal distribution and assigned to random markers. Markers with effect are highlighted with dashed lines. (**b**) Significant QTCs found by QTCAT. (**c**) LASSO selection frequency for each marker during the 50 iterations of QTCAT. (**d**) Manhattan plot of the LMM analysis. The horizontal dashed line depicts the significance threshold when controlling the multiple testing with FWER, whereas the red markers are significantly associated when controlling with FDR. (**e**) The Manhattan plot of the LMM* analysis. GRM was estimated without markers on the chromosome of the actual testing position. The results are shown as in (d).

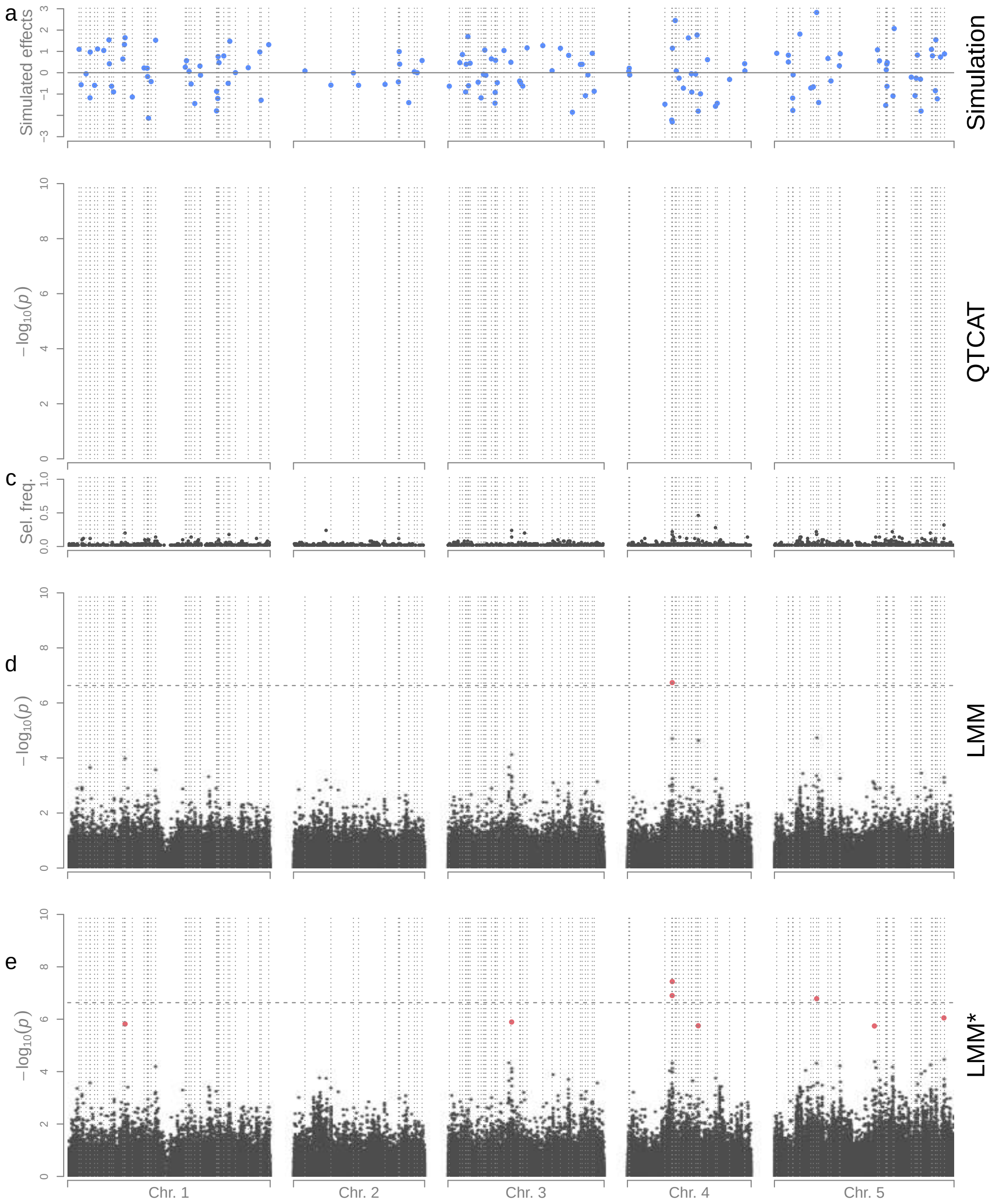

Supplementary Figure 282 Simulation of a GWA analysis based on a structured population with a heritability of 0.4 (run 82). (**a**) Simulation of 150 effects randomly drawn from a normal distribution and assigned to random markers. Markers with effect are highlighted with dashed lines. (**b**) Significant QTCs found by QTCAT. (**c**) LASSO selection frequency for each marker during the 50 iterations of QTCAT. (**d**) Manhattan plot of the LMM analysis. The horizontal dashed line depicts the significance threshold when controlling the multiple testing with FWER, whereas the red markers are significantly associated when controlling with FDR. (**e**) The Manhattan plot of the LMM* analysis. GRM was estimated without markers on the chromosome of the actual testing position. The results are shown as in (d).

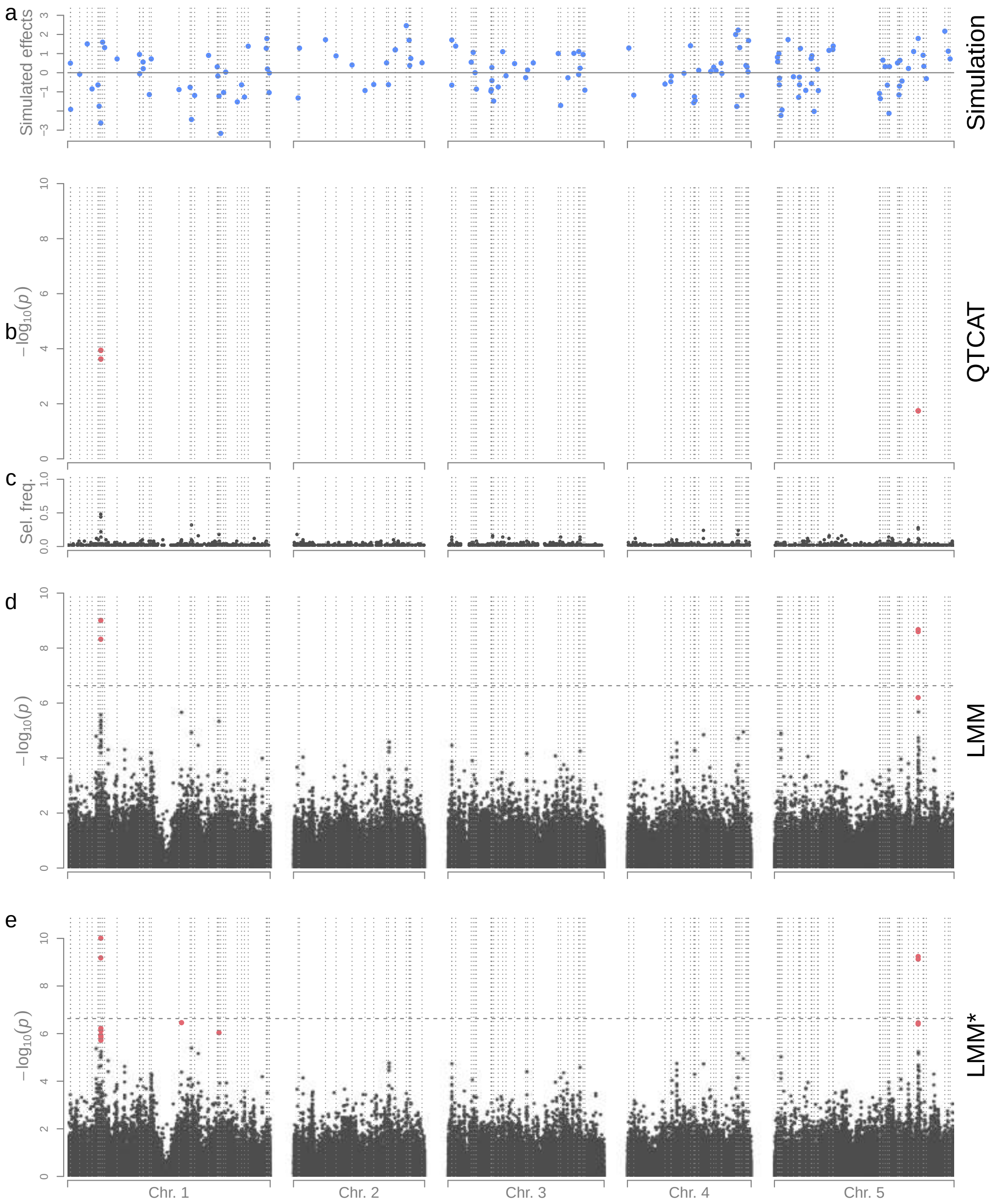

Supplementary Figure 283 Simulation of a GWA analysis based on a structured population with a heritability of 0.4 (run 83). (**a**) Simulation of 150 effects randomly drawn from a normal distribution and assigned to random markers. Markers with effect are highlighted with dashed lines. (**b**) Significant QTCs found by QTCAT. (**c**) LASSO selection frequency for each marker during the 50 iterations of QTCAT. (**d**) Manhattan plot of the LMM analysis. The horizontal dashed line depicts the significance threshold when controlling the multiple testing with FWER, whereas the red markers are significantly associated when controlling with FDR. (**e**) The Manhattan plot of the LMM* analysis. GRM was estimated without markers on the chromosome of the actual testing position. The results are shown as in (d).

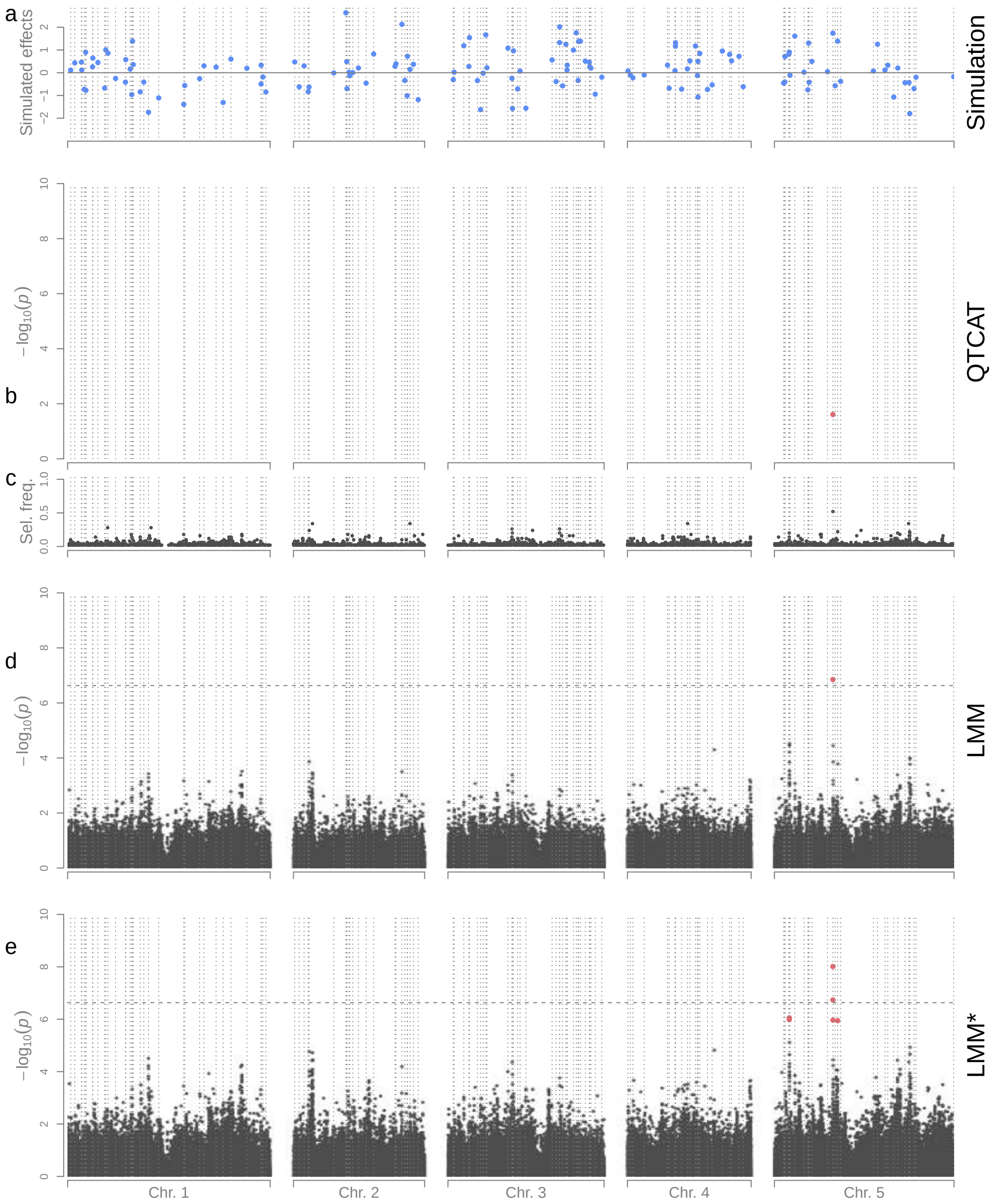

Supplementary Figure 284 Simulation of a GWA analysis based on a structured population with a heritability of 0.4 (run 84). (**a**) Simulation of 150 effects randomly drawn from a normal distribution and assigned to random markers. Markers with effect are highlighted with dashed lines. (**b**) Significant QTCs found by QTCAT. (**c**) LASSO selection frequency for each marker during the 50 iterations of QTCAT. (**d**) Manhattan plot of the LMM analysis. The horizontal dashed line depicts the significance threshold when controlling the multiple testing with FWER, whereas the red markers are significantly associated when controlling with FDR. (**e**) The Manhattan plot of the LMM* analysis. GRM was estimated without markers on the chromosome of the actual testing position. The results are shown as in (d).

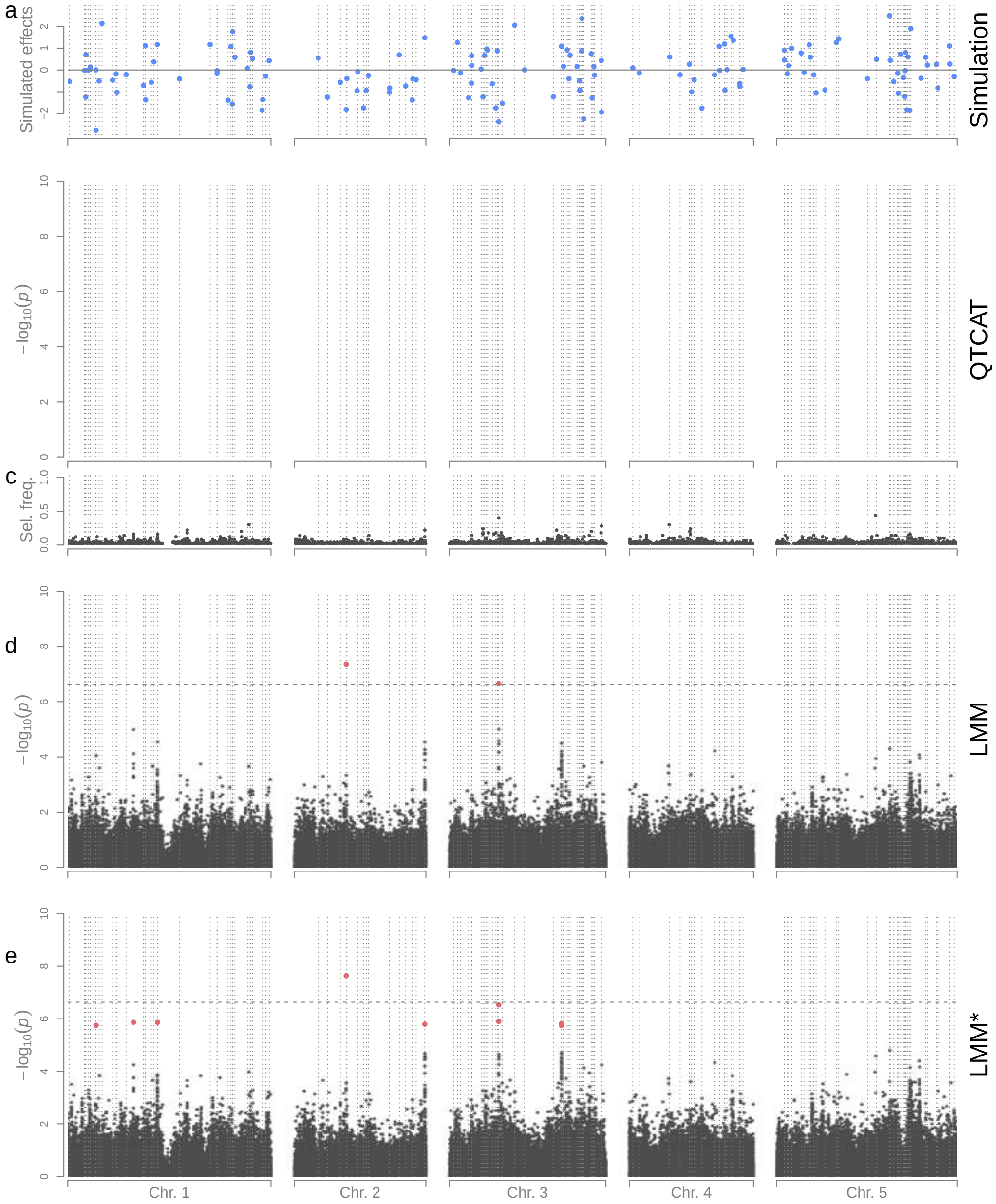

Supplementary Figure 285 Simulation of a GWA analysis based on a structured population with a heritability of 0.4 (run 85). (**a**) Simulation of 150 effects randomly drawn from a normal distribution and assigned to random markers. Markers with effect are highlighted with dashed lines. (**b**) Significant QTCs found by QTCAT. (**c**) LASSO selection frequency for each marker during the 50 iterations of QTCAT. (**d**) Manhattan plot of the LMM analysis. The horizontal dashed line depicts the significance threshold when controlling the multiple testing with FWER, whereas the red markers are significantly associated when controlling with FDR. (**e**) The Manhattan plot of the LMM* analysis. GRM was estimated without markers on the chromosome of the actual testing position. The results are shown as in (d).

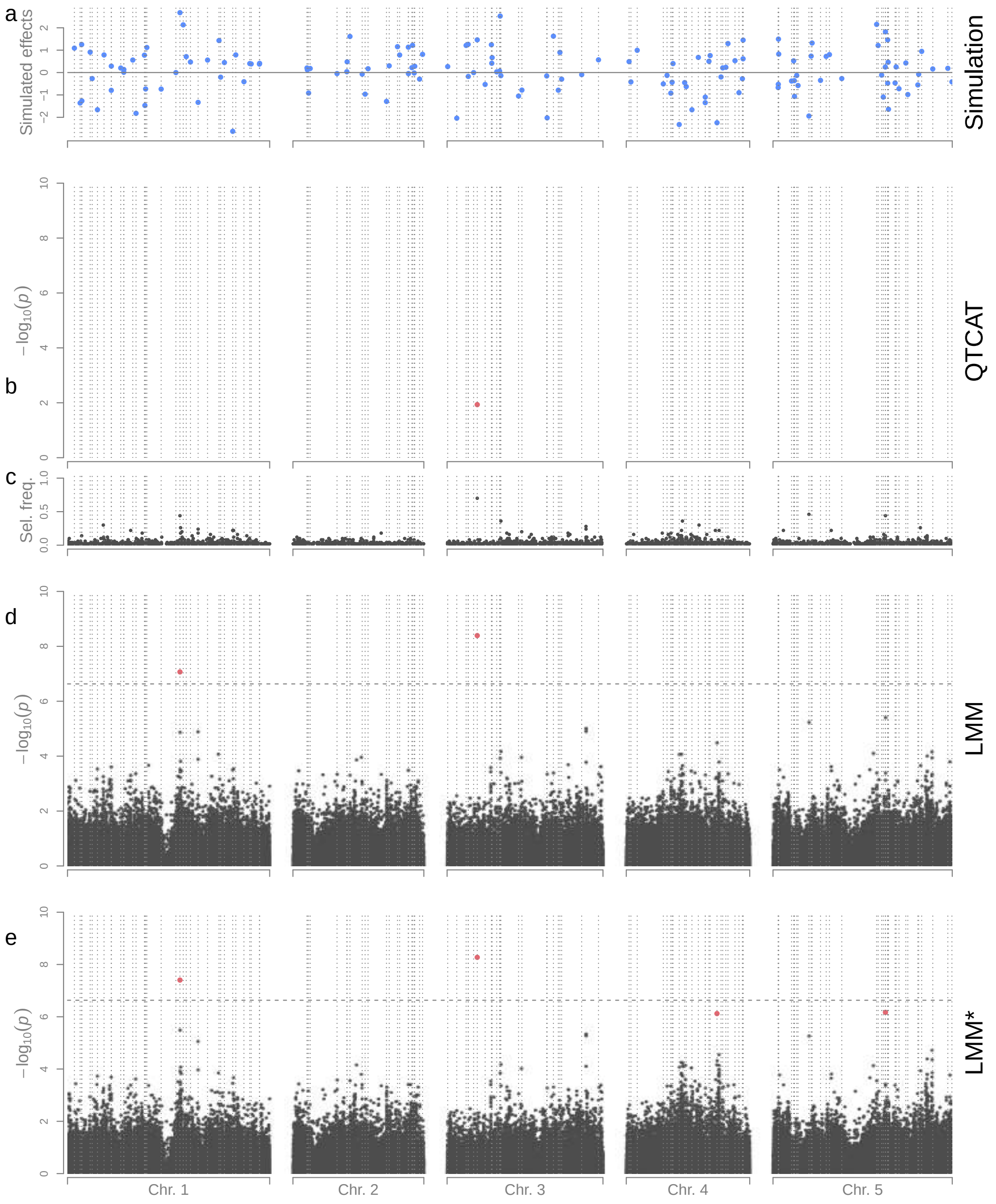

Supplementary Figure 286 Simulation of a GWA analysis based on a structured population with a heritability of 0.4 (run 86). (**a**) Simulation of 150 effects randomly drawn from a normal distribution and assigned to random markers. Markers with effect are highlighted with dashed lines. (**b**) Significant QTCs found by QTCAT. (**c**) LASSO selection frequency for each marker during the 50 iterations of QTCAT. (**d**) Manhattan plot of the LMM analysis. The horizontal dashed line depicts the significance threshold when controlling the multiple testing with FWER, whereas the red markers are significantly associated when controlling with FDR. (**e**) The Manhattan plot of the LMM* analysis. GRM was estimated without markers on the chromosome of the actual testing position. The results are shown as in (d).

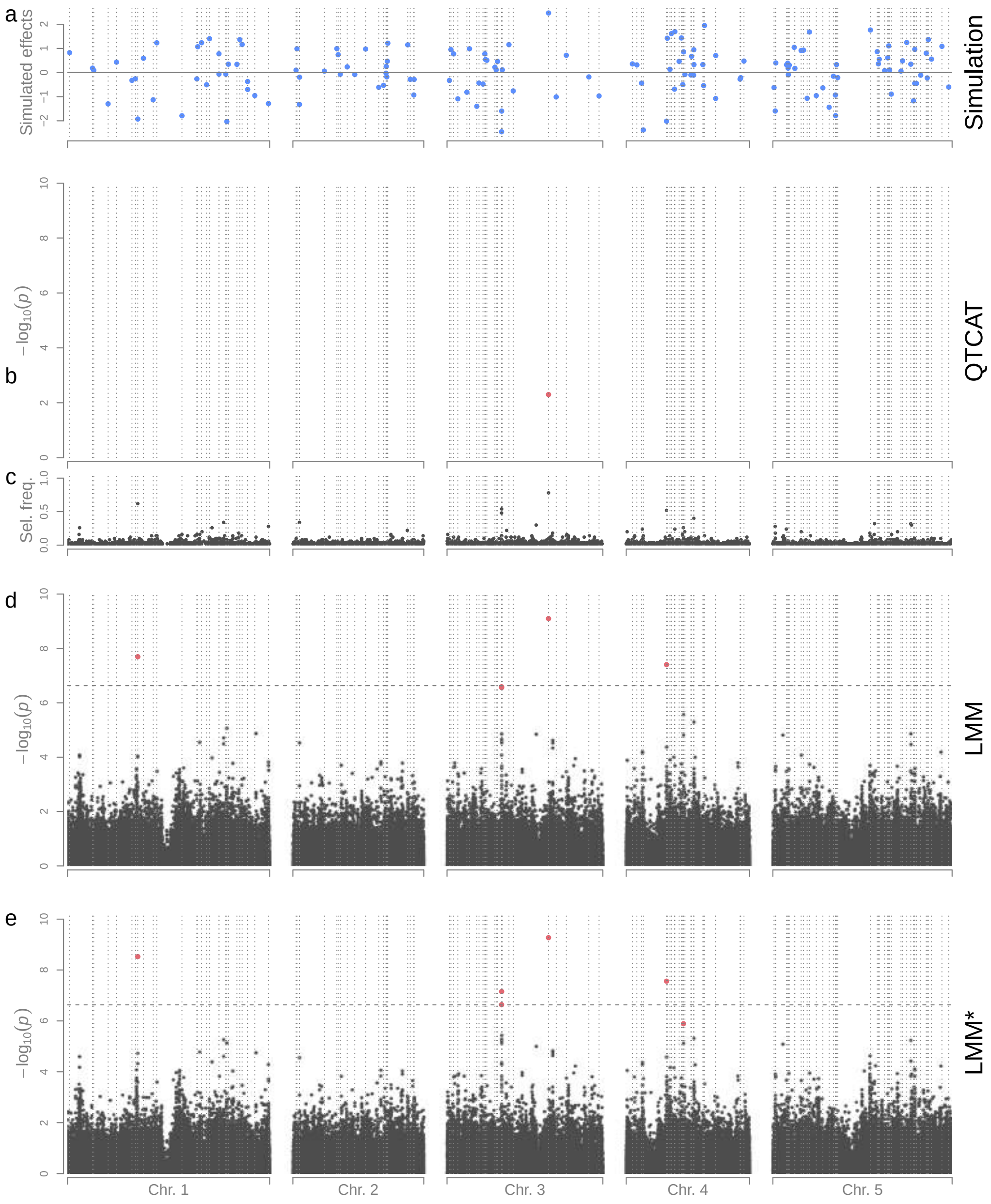

Supplementary Figure 287 Simulation of a GWA analysis based on a structured population with a heritability of 0.4 (run 87). (**a**) Simulation of 150 effects randomly drawn from a normal distribution and assigned to random markers. Markers with effect are highlighted with dashed lines. (**b**) Significant QTCs found by QTCAT. (**c**) LASSO selection frequency for each marker during the 50 iterations of QTCAT. (**d**) Manhattan plot of the LMM analysis. The horizontal dashed line depicts the significance threshold when controlling the multiple testing with FWER, whereas the red markers are significantly associated when controlling with FDR. (**e**) The Manhattan plot of the LMM* analysis. GRM was estimated without markers on the chromosome of the actual testing position. The results are shown as in (d).

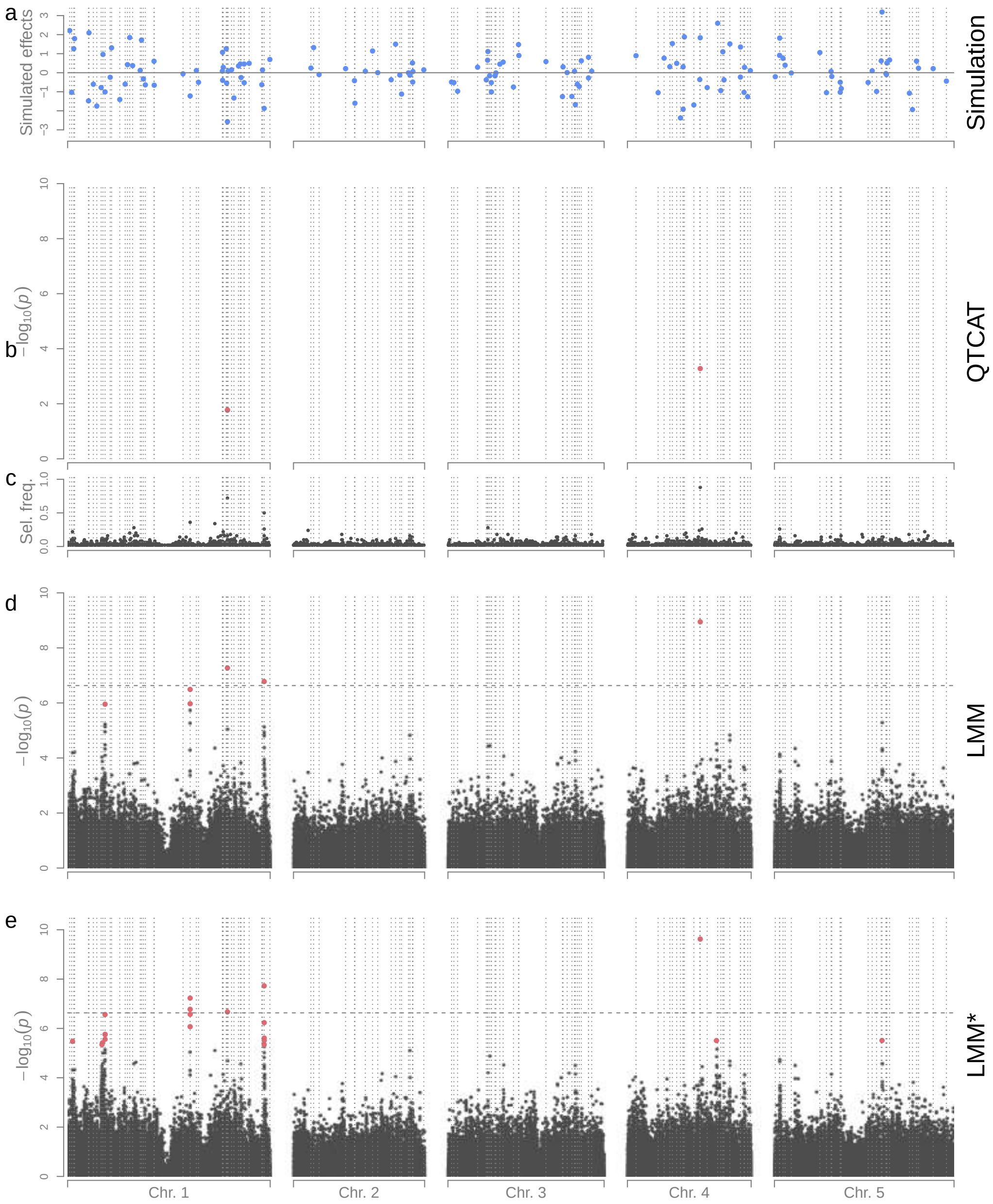

Supplementary Figure 288 Simulation of a GWA analysis based on a structured population with a heritability of 0.4 (run 88). (**a**) Simulation of 150 effects randomly drawn from a normal distribution and assigned to random markers. Markers with effect are highlighted with dashed lines. (**b**) Significant QTCs found by QTCAT. (**c**) LASSO selection frequency for each marker during the 50 iterations of QTCAT. (**d**) Manhattan plot of the LMM analysis. The horizontal dashed line depicts the significance threshold when controlling the multiple testing with FWER, whereas the red markers are significantly associated when controlling with FDR. (**e**) The Manhattan plot of the LMM* analysis. GRM was estimated without markers on the chromosome of the actual testing position. The results are shown as in (d).

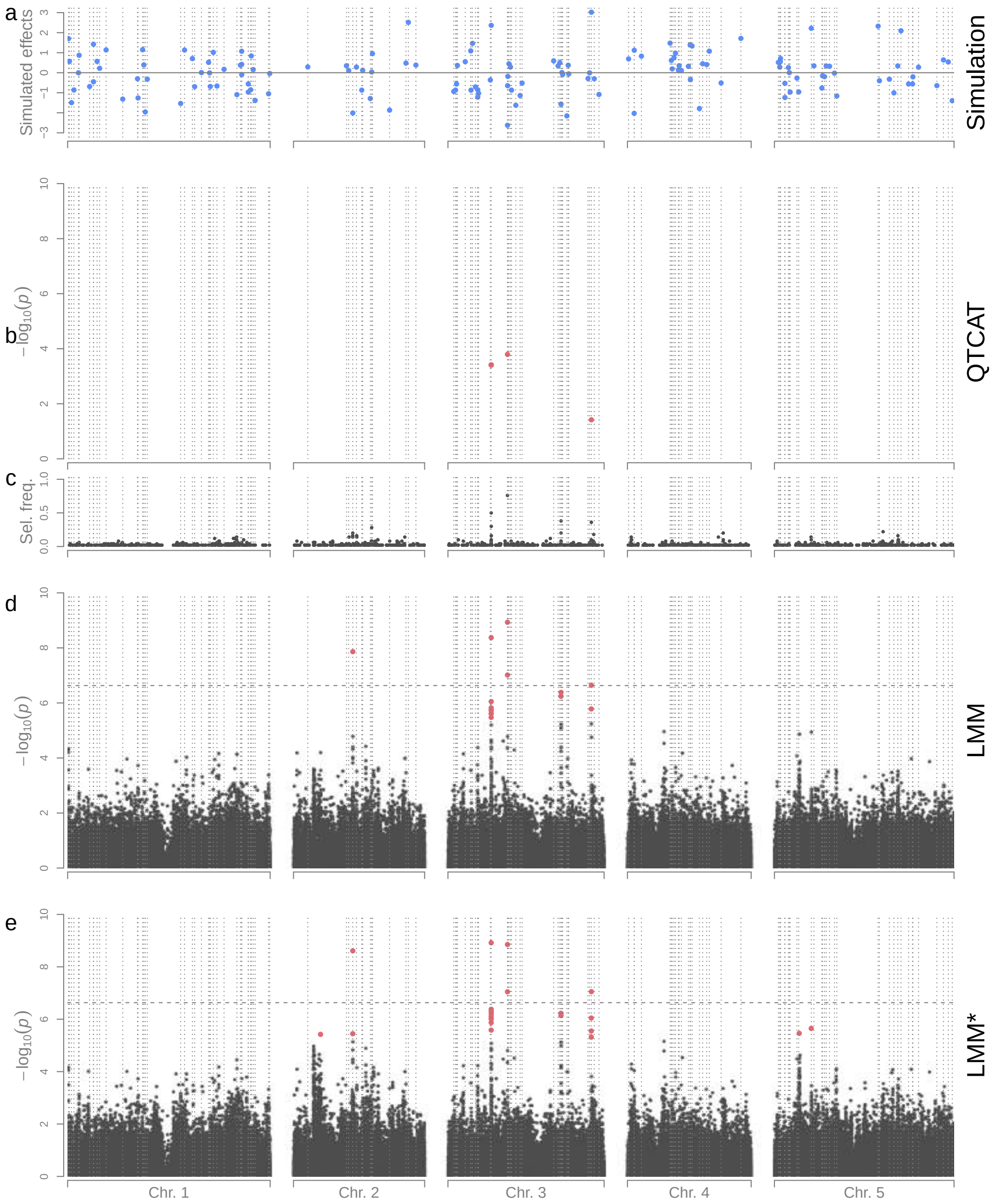

Supplementary Figure 289 Simulation of a GWA analysis based on a structured population with a heritability of 0.4 (run 89). (**a**) Simulation of 150 effects randomly drawn from a normal distribution and assigned to random markers. Markers with effect are highlighted with dashed lines. (**b**) Significant QTCs found by QTCAT. (**c**) LASSO selection frequency for each marker during the 50 iterations of QTCAT. (**d**) Manhattan plot of the LMM analysis. The horizontal dashed line depicts the significance threshold when controlling the multiple testing with FWER, whereas the red markers are significantly associated when controlling with FDR. (**e**) The Manhattan plot of the LMM* analysis. GRM was estimated without markers on the chromosome of the actual testing position. The results are shown as in (d).

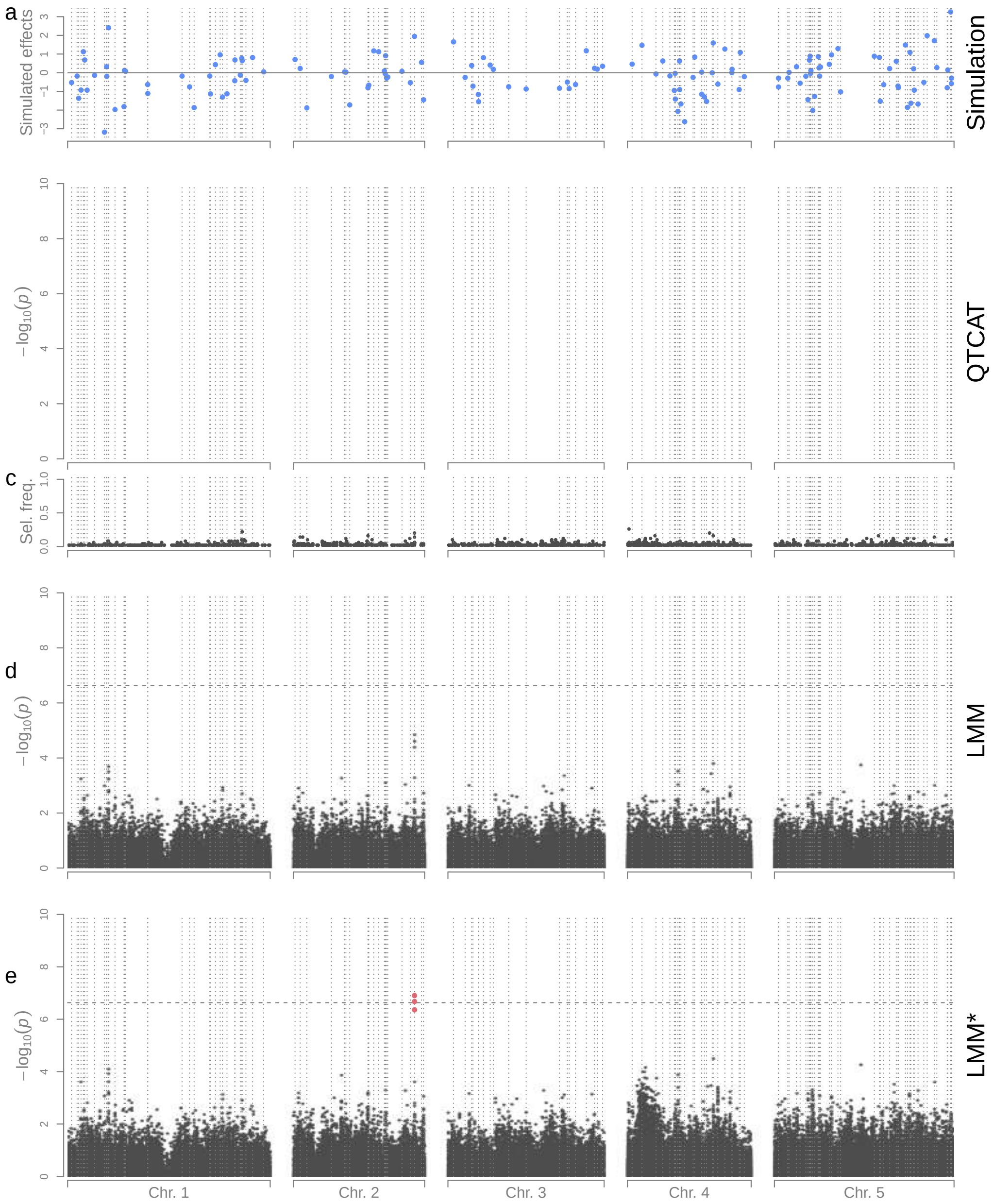

Supplementary Figure 290 Simulation of a GWA analysis based on a structured population with a heritability of 0.4 (run 90). (**a**) Simulation of 150 effects randomly drawn from a normal distribution and assigned to random markers. Markers with effect are highlighted with dashed lines. (**b**) Significant QTCs found by QTCAT. (**c**) LASSO selection frequency for each marker during the 50 iterations of QTCAT. (**d**) Manhattan plot of the LMM analysis. The horizontal dashed line depicts the significance threshold when controlling the multiple testing with FWER, whereas the red markers are significantly associated when controlling with FDR. (**e**) The Manhattan plot of the LMM* analysis. GRM was estimated without markers on the chromosome of the actual testing position. The results are shown as in (d).

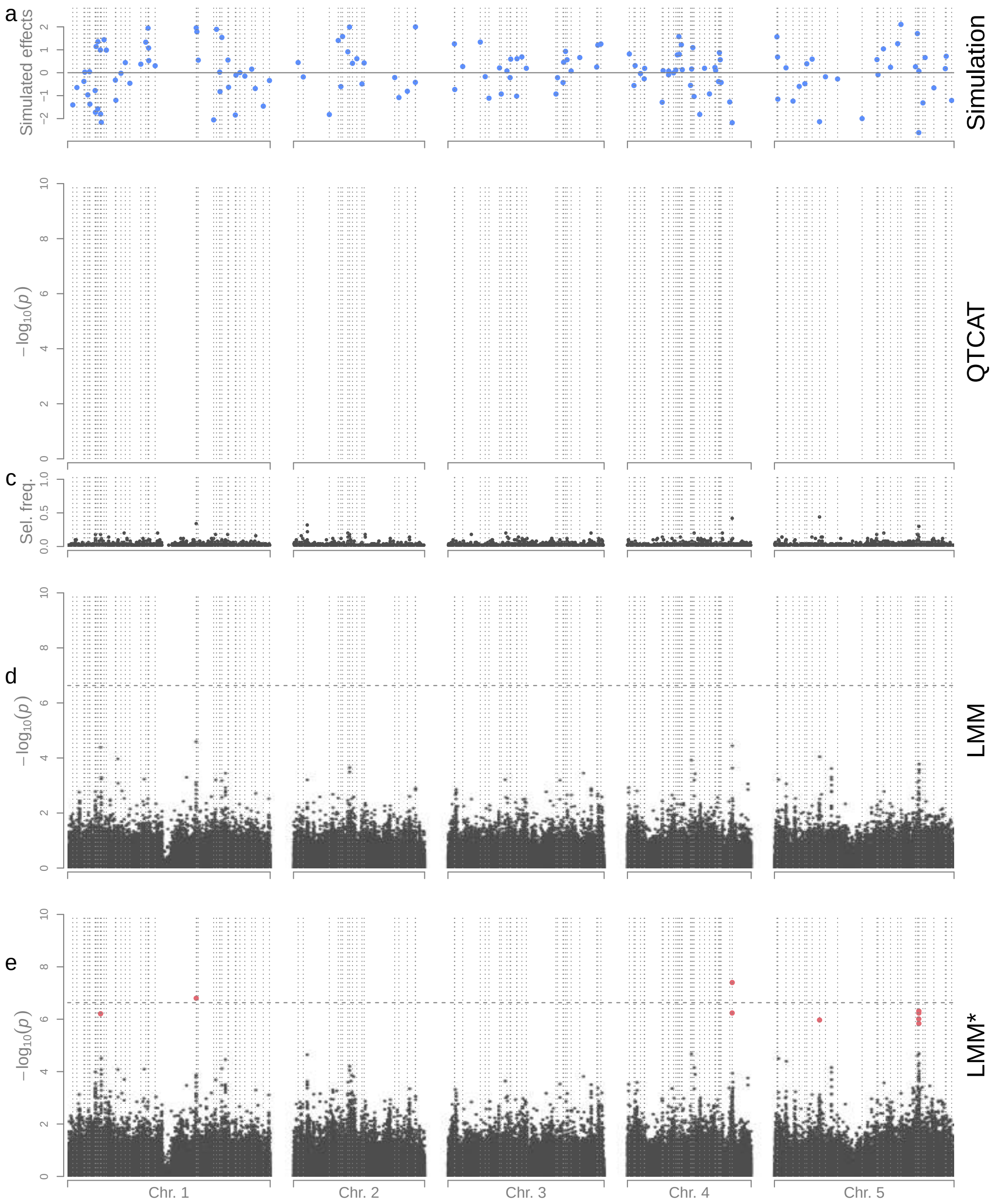

Supplementary Figure 291 Simulation of a GWA analysis based on a structured population with a heritability of 0.4 (run 91). (**a**) Simulation of 150 effects randomly drawn from a normal distribution and assigned to random markers. Markers with effect are highlighted with dashed lines. (**b**) Significant QTCs found by QTCAT. (**c**) LASSO selection frequency for each marker during the 50 iterations of QTCAT. (**d**) Manhattan plot of the LMM analysis. The horizontal dashed line depicts the significance threshold when controlling the multiple testing with FWER, whereas the red markers are significantly associated when controlling with FDR. (**e**) The Manhattan plot of the LMM* analysis. GRM was estimated without markers on the chromosome of the actual testing position. The results are shown as in (d).

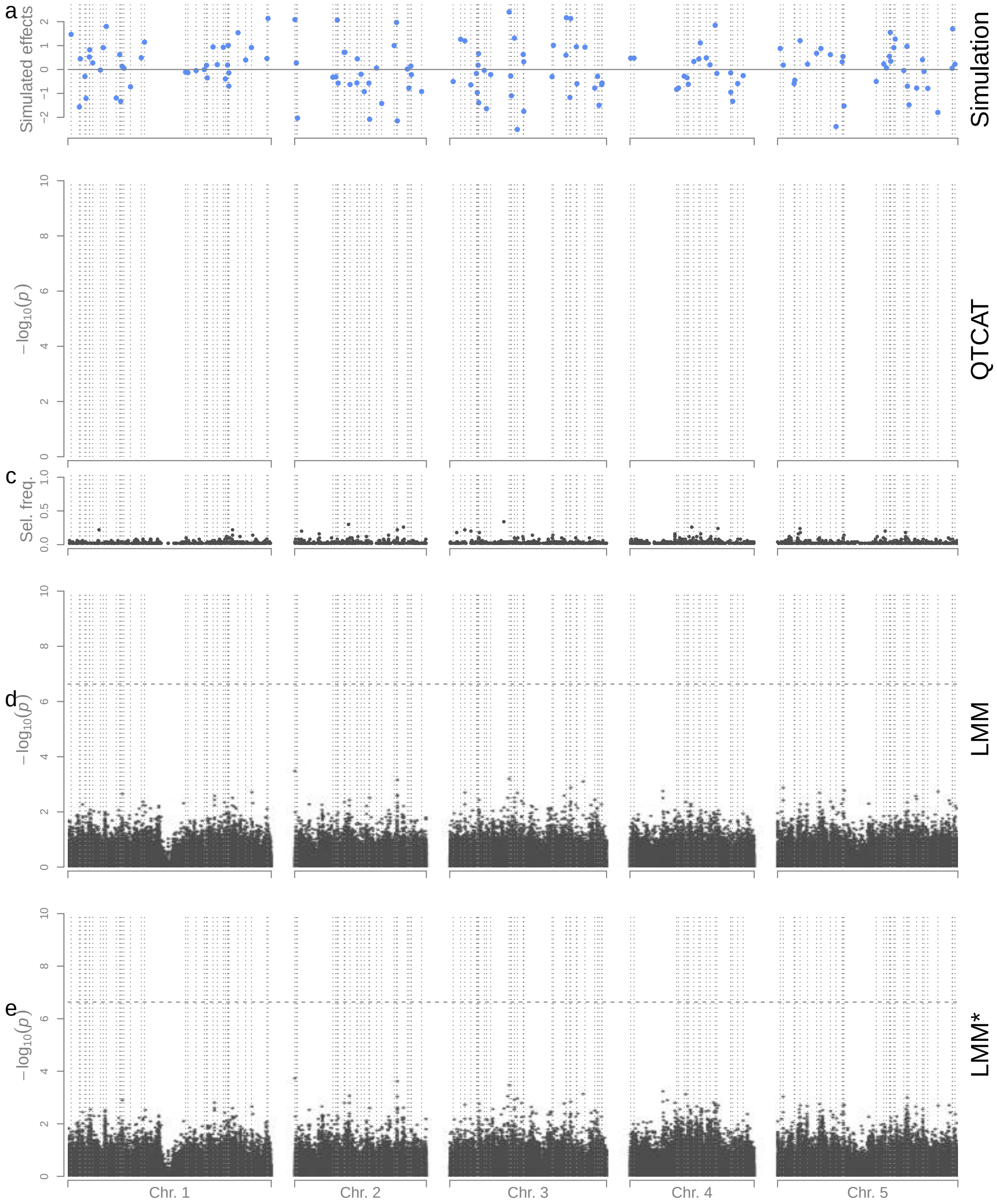

Supplementary Figure 292 Simulation of a GWA analysis based on a structured population with a heritability of 0.4 (run 92). (**a**) Simulation of 150 effects randomly drawn from a normal distribution and assigned to random markers. Markers with effect are highlighted with dashed lines. (**b**) Significant QTCs found by QTCAT. (**c**) LASSO selection frequency for each marker during the 50 iterations of QTCAT. (**d**) Manhattan plot of the LMM analysis. The horizontal dashed line depicts the significance threshold when controlling the multiple testing with FWER, whereas the red markers are significantly associated when controlling with FDR. (**e**) The Manhattan plot of the LMM* analysis. GRM was estimated without markers on the chromosome of the actual testing position. The results are shown as in (d).

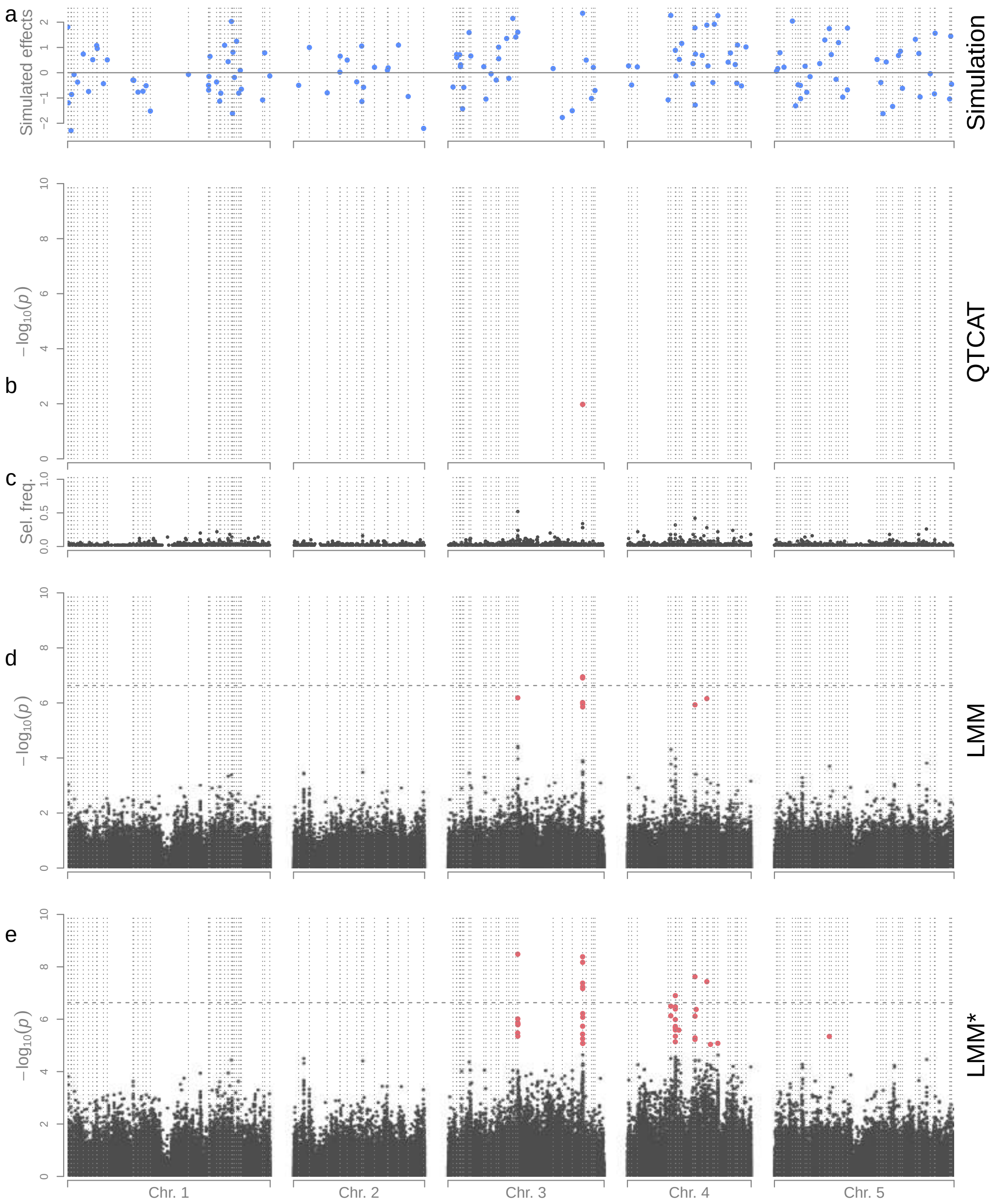

Supplementary Figure 293 Simulation of a GWA analysis based on a structured population with a heritability of 0.4 (run 93). (**a**) Simulation of 150 effects randomly drawn from a normal distribution and assigned to random markers. Markers with effect are highlighted with dashed lines. (**b**) Significant QTCs found by QTCAT. (**c**) LASSO selection frequency for each marker during the 50 iterations of QTCAT. (**d**) Manhattan plot of the LMM analysis. The horizontal dashed line depicts the significance threshold when controlling the multiple testing with FWER, whereas the red markers are significantly associated when controlling with FDR. (**e**) The Manhattan plot of the LMM* analysis. GRM was estimated without markers on the chromosome of the actual testing position. The results are shown as in (d).

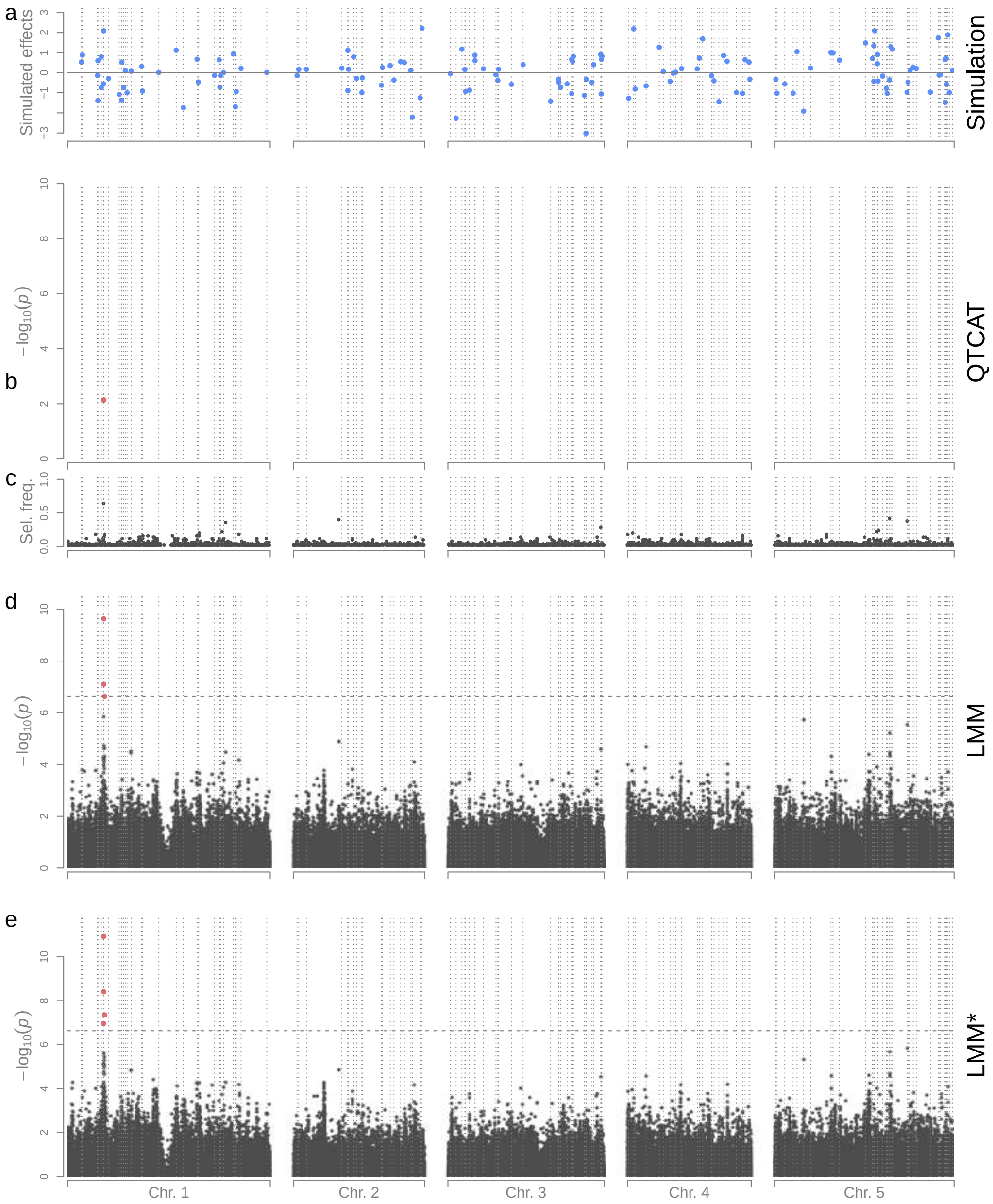

Supplementary Figure 294 Simulation of a GWA analysis based on a structured population with a heritability of 0.4 (run 94). (**a**) Simulation of 150 effects randomly drawn from a normal distribution and assigned to random markers. Markers with effect are highlighted with dashed lines. (**b**) Significant QTCs found by QTCAT. (**c**) LASSO selection frequency for each marker during the 50 iterations of QTCAT. (**d**) Manhattan plot of the LMM analysis. The horizontal dashed line depicts the significance threshold when controlling the multiple testing with FWER, whereas the red markers are significantly associated when controlling with FDR. (**e**) The Manhattan plot of the LMM* analysis. GRM was estimated without markers on the chromosome of the actual testing position. The results are shown as in (d).

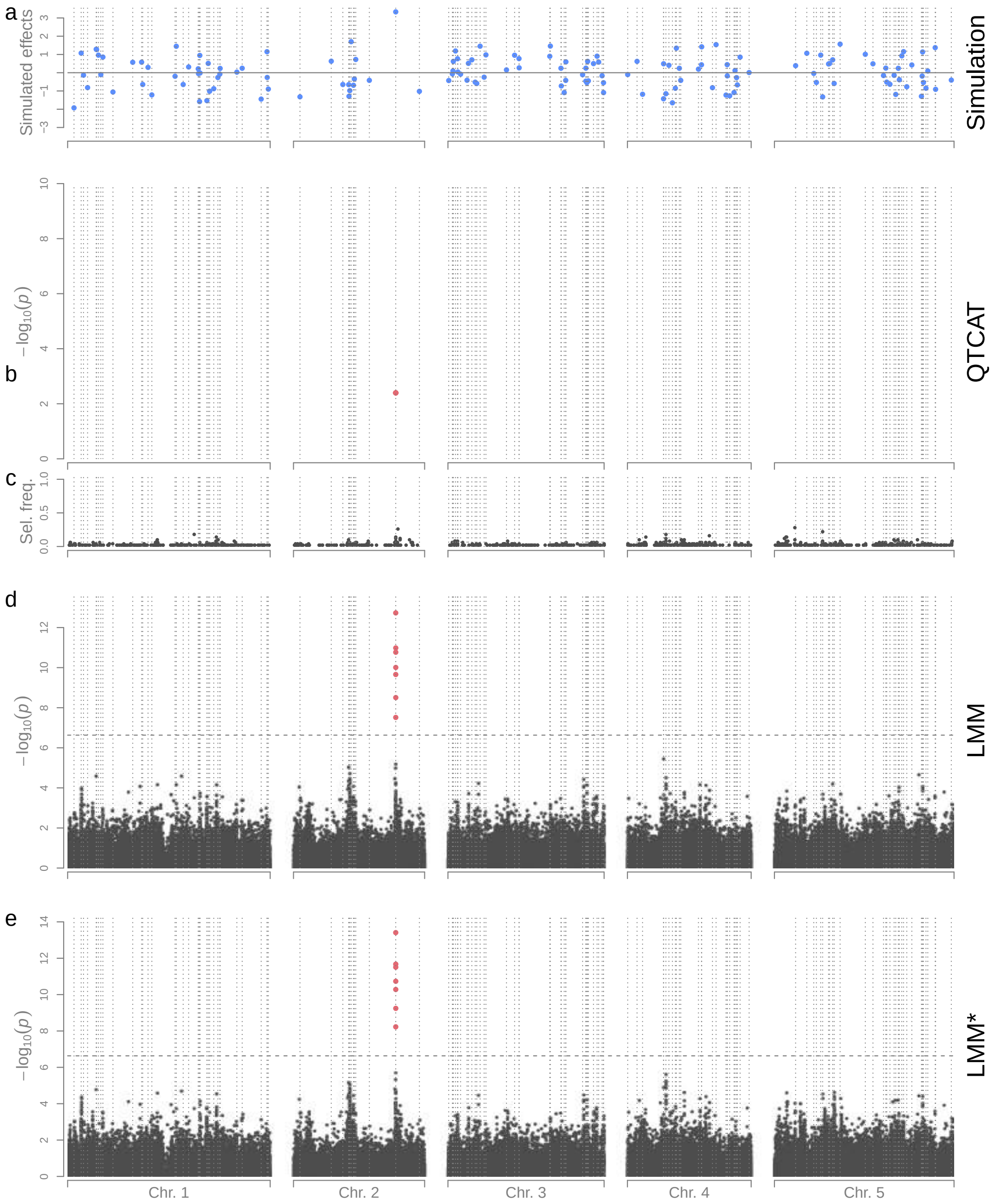

Supplementary Figure 295 Simulation of a GWA analysis based on a structured population with a heritability of 0.4 (run 95). (**a**) Simulation of 150 effects randomly drawn from a normal distribution and assigned to random markers. Markers with effect are highlighted with dashed lines. (**b**) Significant QTCs found by QTCAT. (**c**) LASSO selection frequency for each marker during the 50 iterations of QTCAT. (**d**) Manhattan plot of the LMM analysis. The horizontal dashed line depicts the significance threshold when controlling the multiple testing with FWER, whereas the red markers are significantly associated when controlling with FDR. (**e**) The Manhattan plot of the LMM* analysis. GRM was estimated without markers on the chromosome of the actual testing position. The results are shown as in (d).

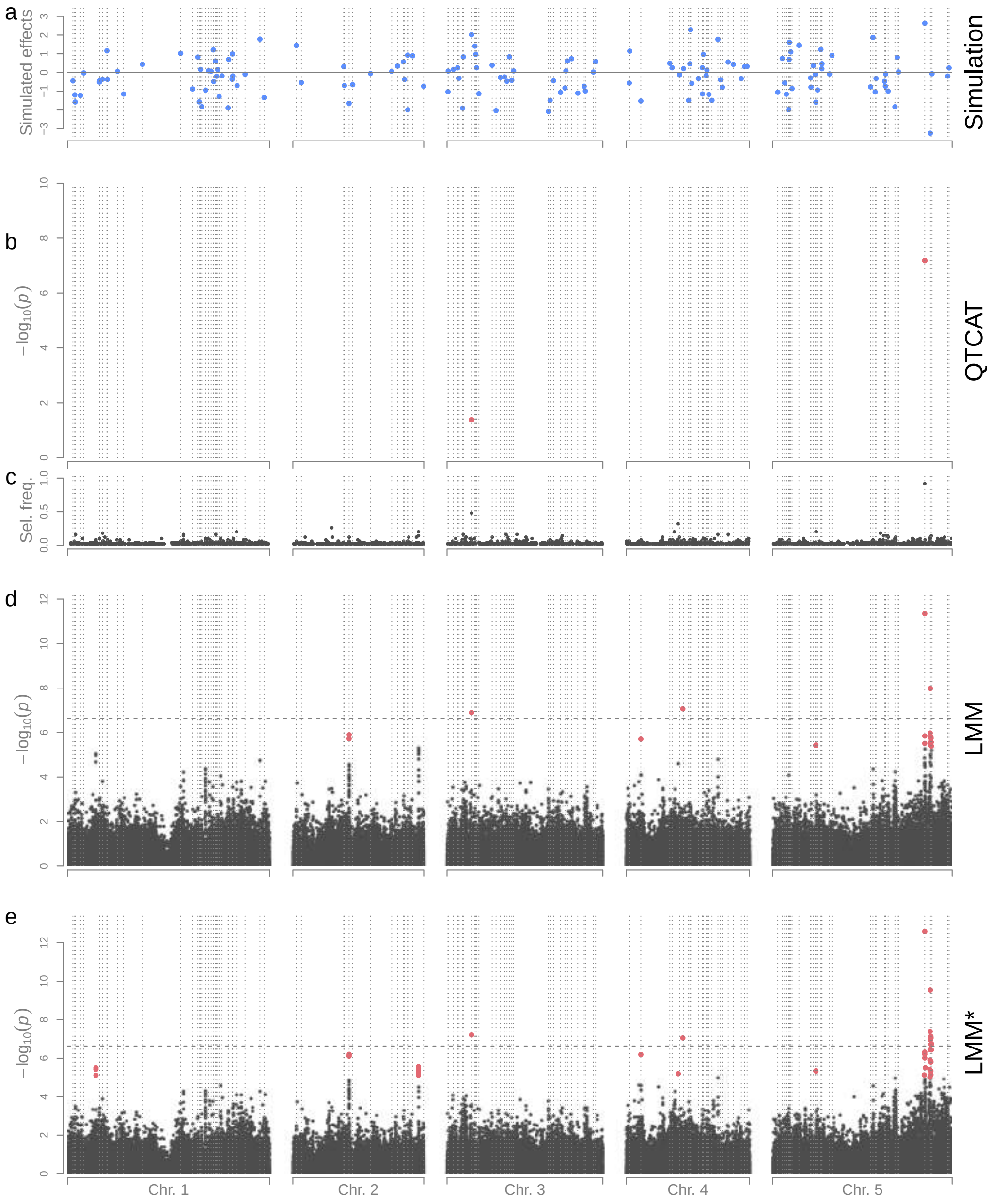

Supplementary Figure 296 Simulation of a GWA analysis based on a structured population with a heritability of 0.4 (run 96). (**a**) Simulation of 150 effects randomly drawn from a normal distribution and assigned to random markers. Markers with effect are highlighted with dashed lines. (**b**) Significant QTCs found by QTCAT. (**c**) LASSO selection frequency for each marker during the 50 iterations of QTCAT. (**d**) Manhattan plot of the LMM analysis. The horizontal dashed line depicts the significance threshold when controlling the multiple testing with FWER, whereas the red markers are significantly associated when controlling with FDR. (**e**) The Manhattan plot of the LMM* analysis. GRM was estimated without markers on the chromosome of the actual testing position. The results are shown as in (d).

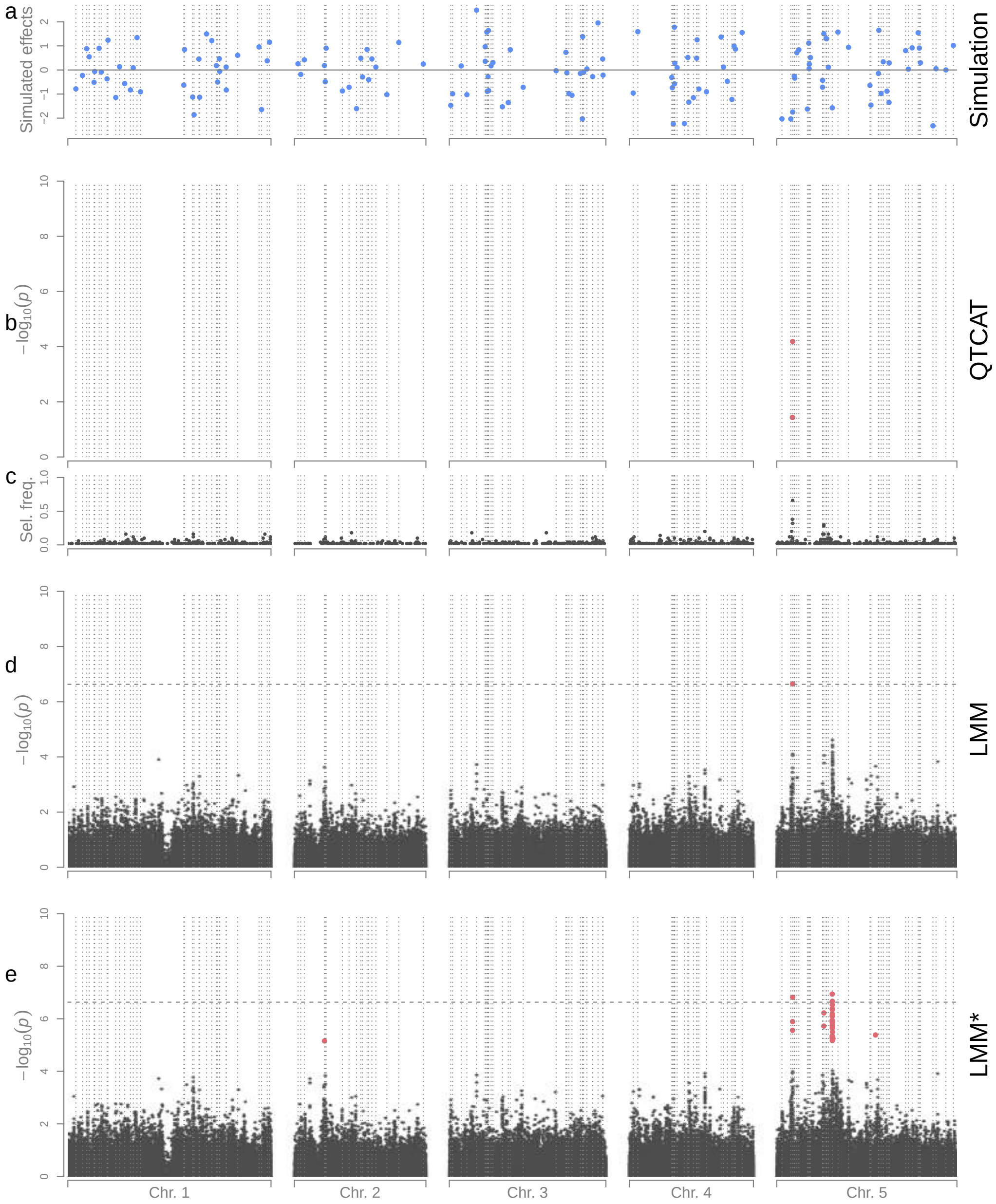

Supplementary Figure 297 Simulation of a GWA analysis based on a structured population with a heritability of 0.4 (run 97). (**a**) Simulation of 150 effects randomly drawn from a normal distribution and assigned to random markers. Markers with effect are highlighted with dashed lines. (**b**) Significant QTCs found by QTCAT. (**c**) LASSO selection frequency for each marker during the 50 iterations of QTCAT. (**d**) Manhattan plot of the LMM analysis. The horizontal dashed line depicts the significance threshold when controlling the multiple testing with FWER, whereas the red markers are significantly associated when controlling with FDR. (**e**) The Manhattan plot of the LMM* analysis. GRM was estimated without markers on the chromosome of the actual testing position. The results are shown as in (d).

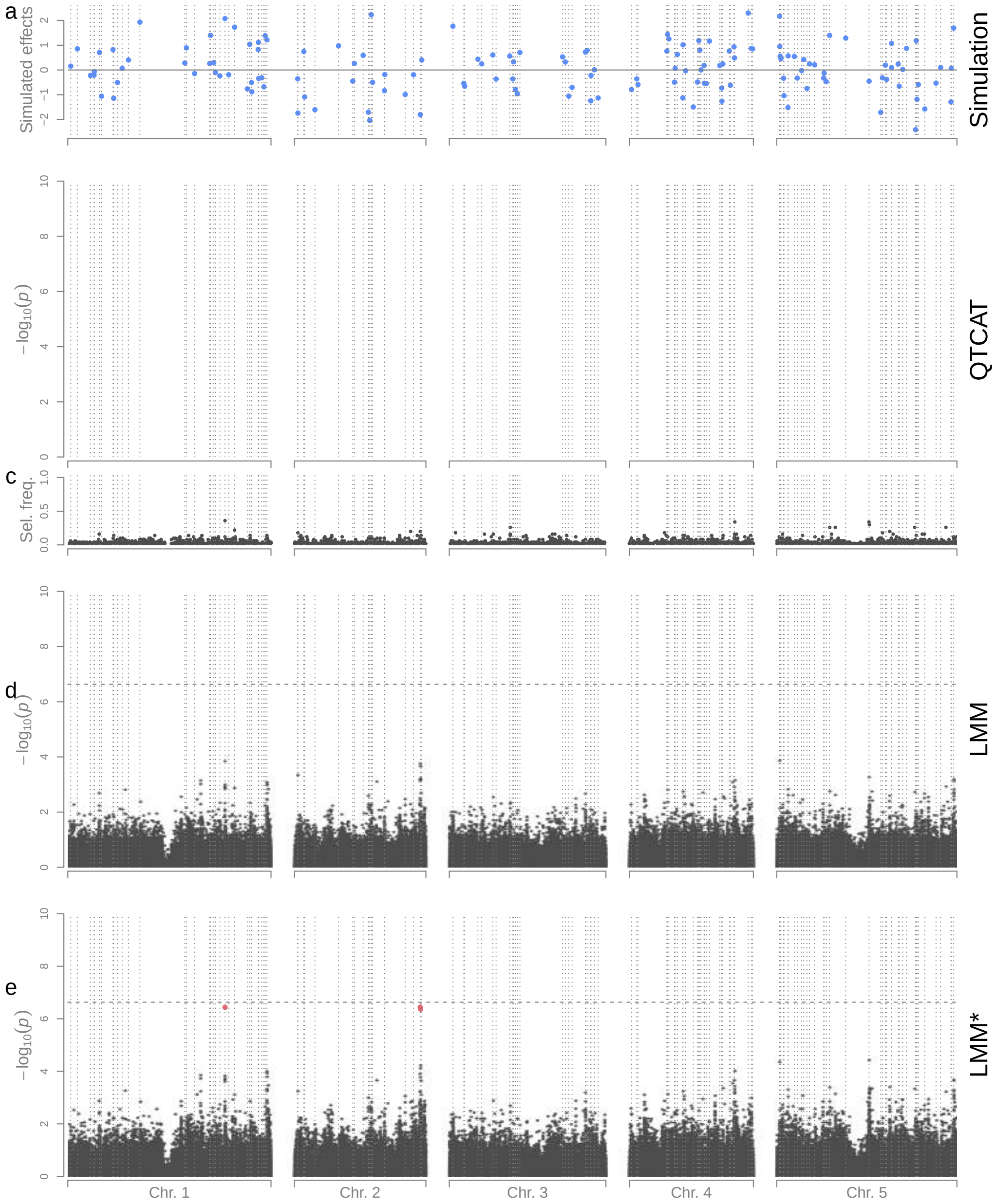

Supplementary Figure 298 Simulation of a GWA analysis based on a structured population with a heritability of 0.4 (run 98). (**a**) Simulation of 150 effects randomly drawn from a normal distribution and assigned to random markers. Markers with effect are highlighted with dashed lines. (**b**) Significant QTCs found by QTCAT. (**c**) LASSO selection frequency for each marker during the 50 iterations of QTCAT. (**d**) Manhattan plot of the LMM analysis. The horizontal dashed line depicts the significance threshold when controlling the multiple testing with FWER, whereas the red markers are significantly associated when controlling with FDR. (**e**) The Manhattan plot of the LMM* analysis. GRM was estimated without markers on the chromosome of the actual testing position. The results are shown as in (d).

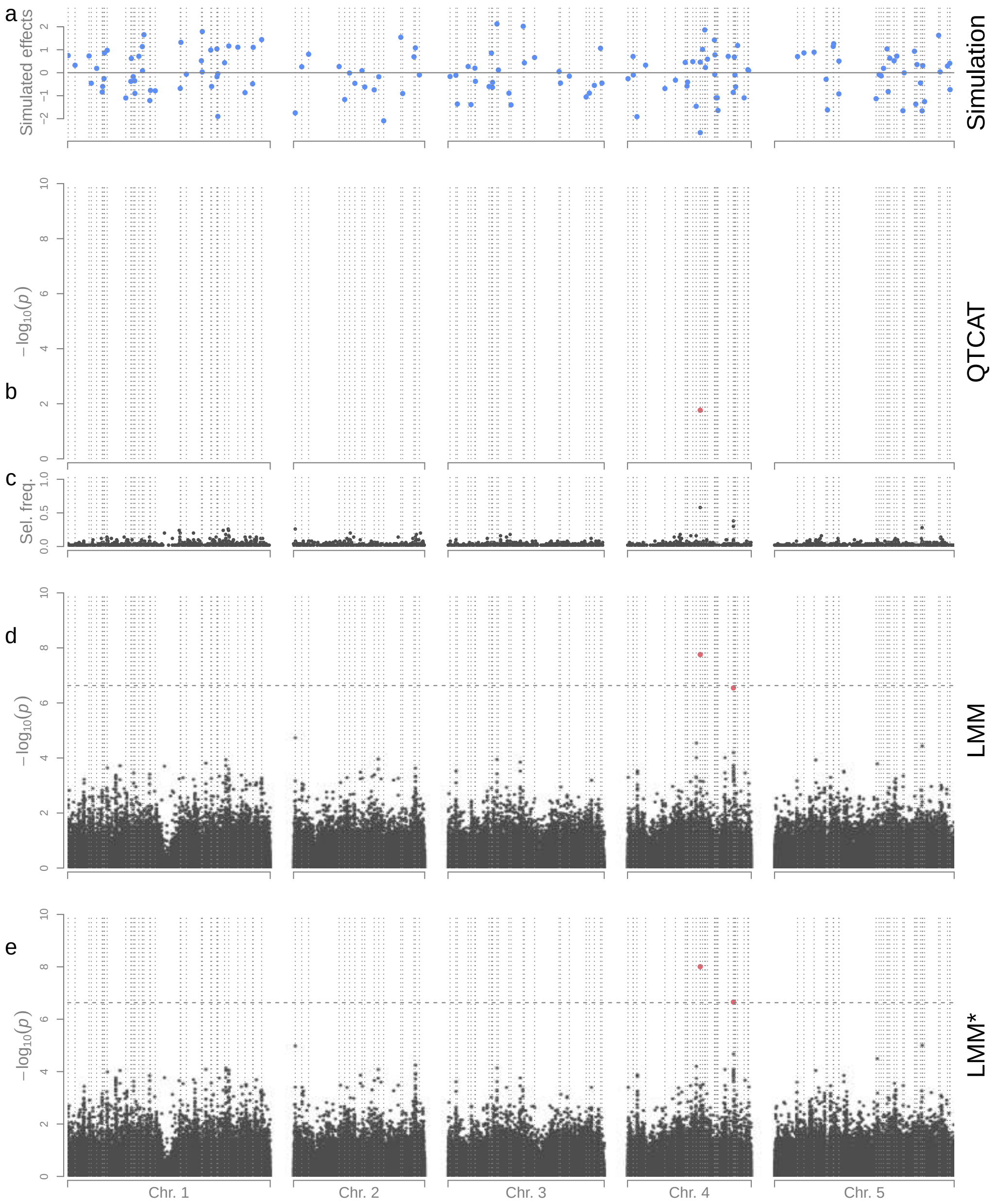

Supplementary Figure 299 Simulation of a GWA analysis based on a structured population with a heritability of 0.4 (run 99). (**a**) Simulation of 150 effects randomly drawn from a normal distribution and assigned to random markers. Markers with effect are highlighted with dashed lines. (**b**) Significant QTCs found by QTCAT. (**c**) LASSO selection frequency for each marker during the 50 iterations of QTCAT. (**d**) Manhattan plot of the LMM analysis. The horizontal dashed line depicts the significance threshold when controlling the multiple testing with FWER, whereas the red markers are significantly associated when controlling with FDR. (**e**) The Manhattan plot of the LMM* analysis. GRM was estimated without markers on the chromosome of the actual testing position. The results are shown as in (d).

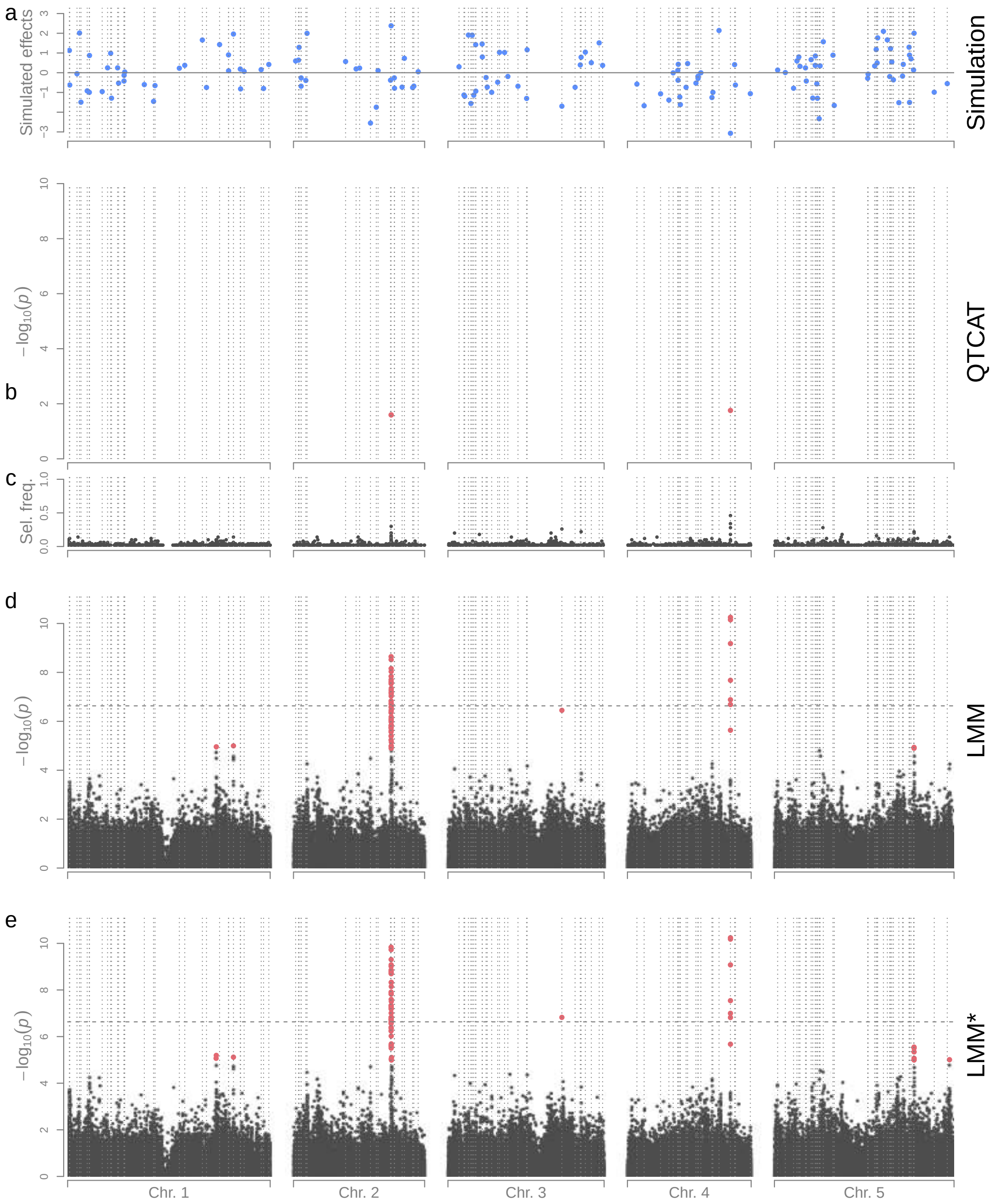

Supplementary Figure 300 Simulation of a GWA analysis based on a structured population with a heritability of 0.4 (run 100). (**a**) Simulation of 150 effects randomly drawn from a normal distribution and assigned to random markers. Markers with effect are highlighted with dashed lines. (**b**) Significant QTCs found by QTCAT. (**c**) LASSO selection frequency for each marker during the 50 iterations of QTCAT. (**d**) Manhattan plot of the LMM analysis. The horizontal dashed line depicts the significance threshold when controlling the multiple testing with FWER, whereas the red markers are significantly associated when controlling with FDR. (**e**) The Manhattan plot of the LMM* analysis. GRM was estimated without markers on the chromosome of the actual testing position. The results are shown as in (d).

GWA analysis for simulation of unstructured populations

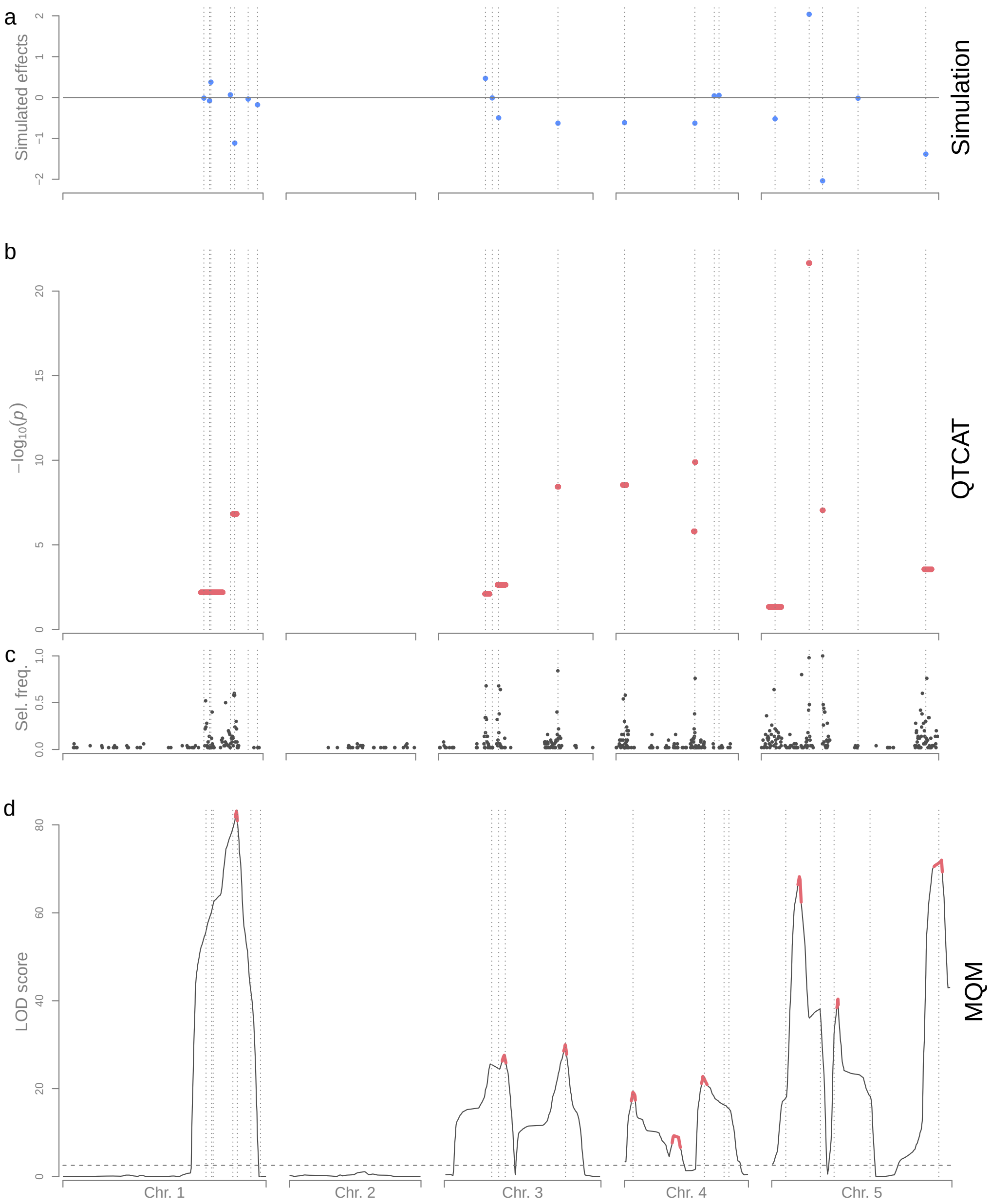

Supplementary Figure 301 Simulation of a GWA analysis based on a unstructured population with a heritability of 0.7 (run 1). (**a**) Simulated of 20 effects randomly drawn from a Gamma distribution and assigned them randomly to markers. Simulated effects randomly drawn from a Gamma distribution. We assigned effects to 20 markers. Markers with an effect are highlighted in (**b–d**) with dashed lines. (**b**) Significant QTCs found by QTCAT. (**c**) The selection frequency of the LASSO for each marker during the 50 iterations of QTCAT. (**d**) MQM LOD score plot, the horizontal dashed line is a simulation based perumtation test FDR. The red colored areas represent the LOD-intervals.

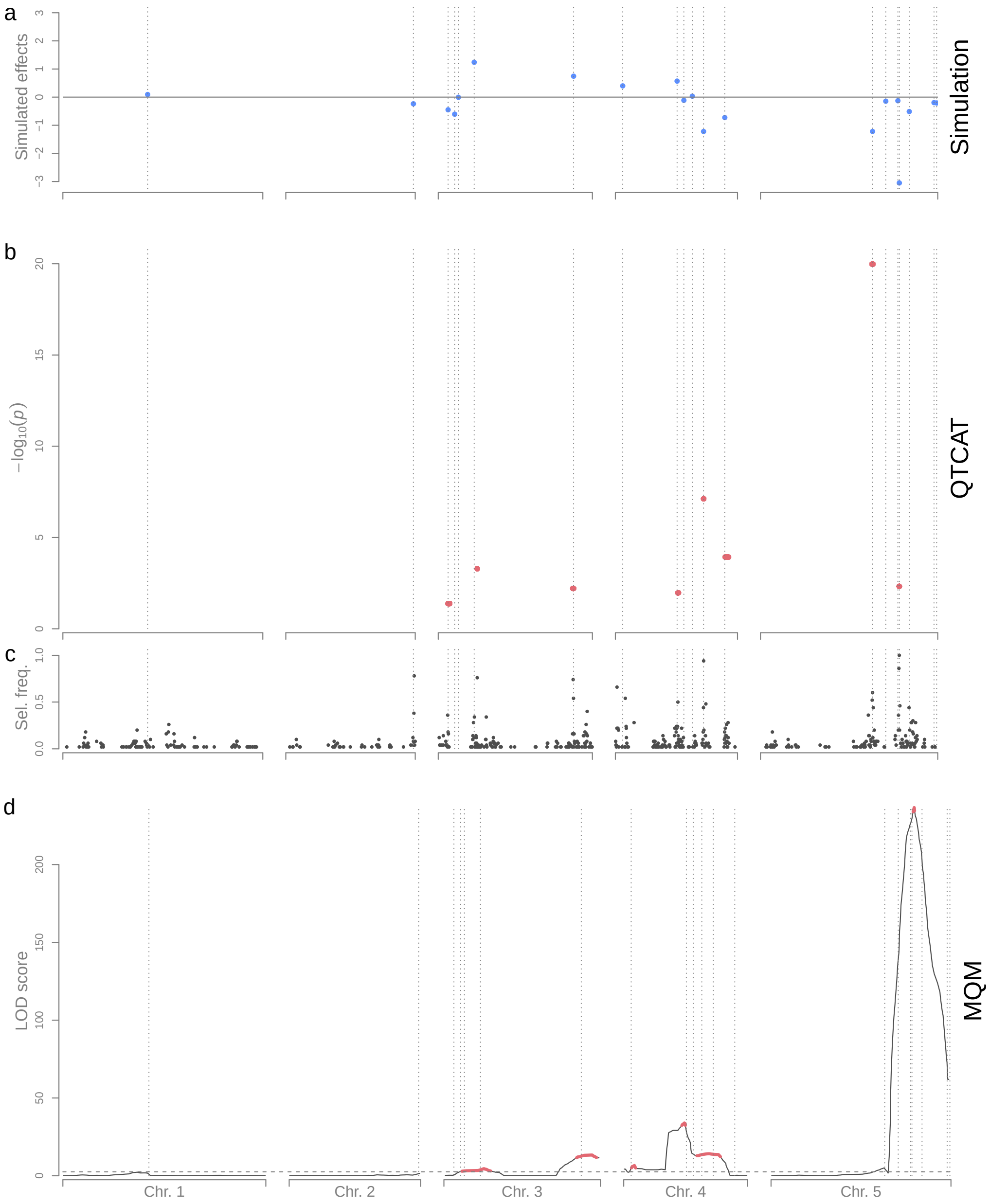

Supplementary Figure 302 Simulation of a GWA analysis based on a unstructured population with a heritability of 0.7 (run 2). (**a**) Simulated of 20 effects randomly drawn from a Gamma distribution and assigned them randomly to markers. Simulated effects randomly drawn from a Gamma distribution. We assigned effects to 20 markers. Markers with an effect are highlighted in (**b–d**) with dashed lines. (**b**) Significant QTCs found by QTCAT. (**c**) The selection frequency of the LASSO for each marker during the 50 iterations of QTCAT. (**d**) MQM LOD score plot, the horizontal dashed line is a simulation based perumtation test FDR. The red colored areas represent the LOD-intervals.

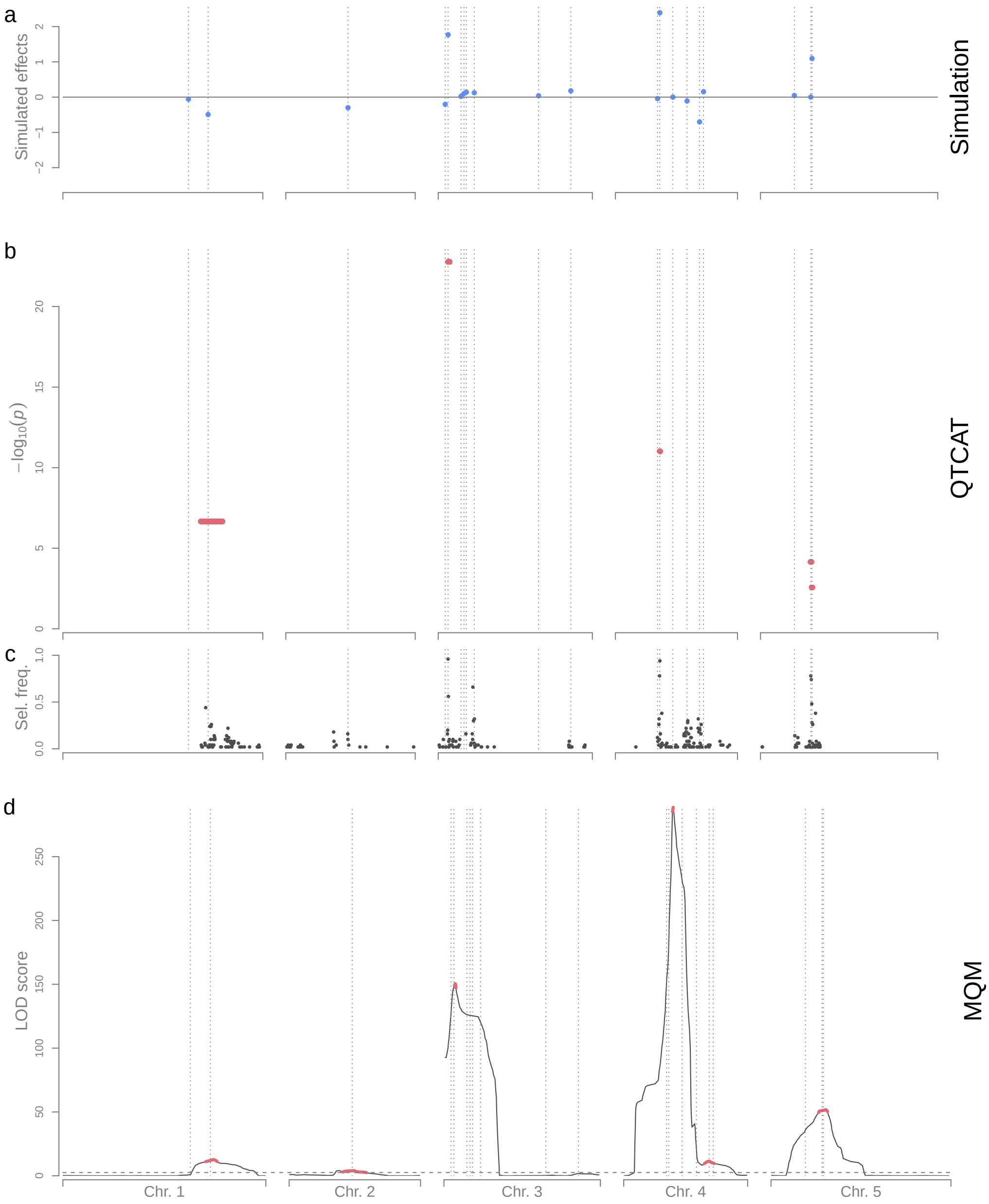

Supplementary Figure 303 Simulation of a GWA analysis based on a unstructured population with a heritability of 0.7 (run 3). (**a**) Simulated of 20 effects randomly drawn from a Gamma distribution and assigned them randomly to markers. Simulated effects randomly drawn from a Gamma distribution. We assigned effects to 20 markers. Markers with an effect are highlighted in (**b**–**d**) with dashed lines. (**b**) Significant QTCs found by QTCAT. (**c**) The selection frequency of the LASSO for each marker during the 50 iterations of QTCAT. (**d**) MQM LOD score plot, the horizontal dashed line is a simulation based perumtation test FDR. The red colored areas represent the LOD-intervals.

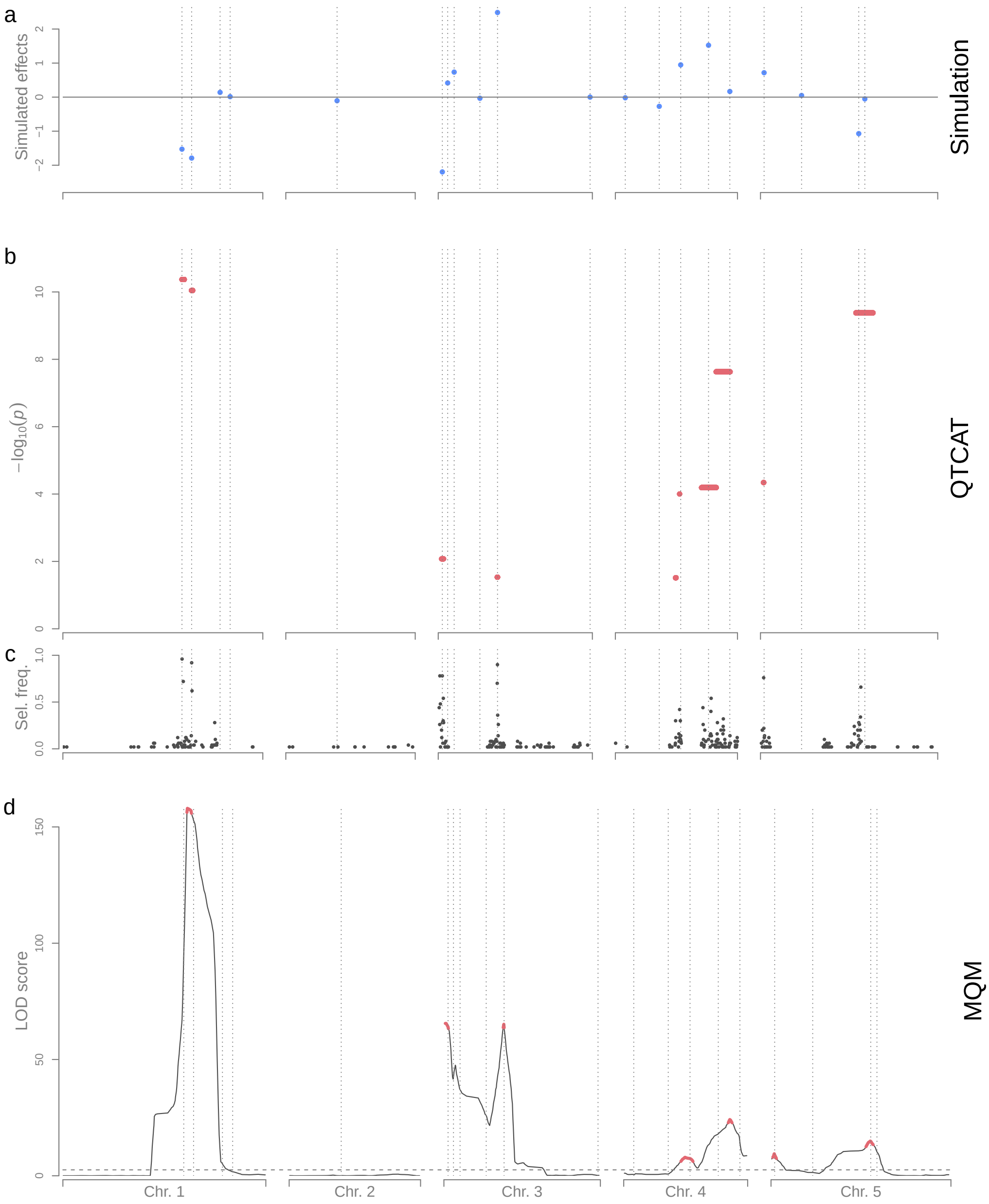

Supplementary Figure 304 Simulation of a GWA analysis based on a unstructured population with a heritability of 0.7 (run 4). (**a**) Simulated of 20 effects randomly drawn from a Gamma distribution and assigned them randomly to markers. Simulated effects randomly drawn from a Gamma distribution. We assigned effects to 20 markers. Markers with an effect are highlighted in (**b–d**) with dashed lines. (**b**) Significant QTCs found by QTCAT. (**c**) The selection frequency of the LASSO for each marker during the 50 iterations of QTCAT. (**d**) MQM LOD score plot, the horizontal dashed line is a simulation based perumtation test FDR. The red colored areas represent the LOD-intervals.

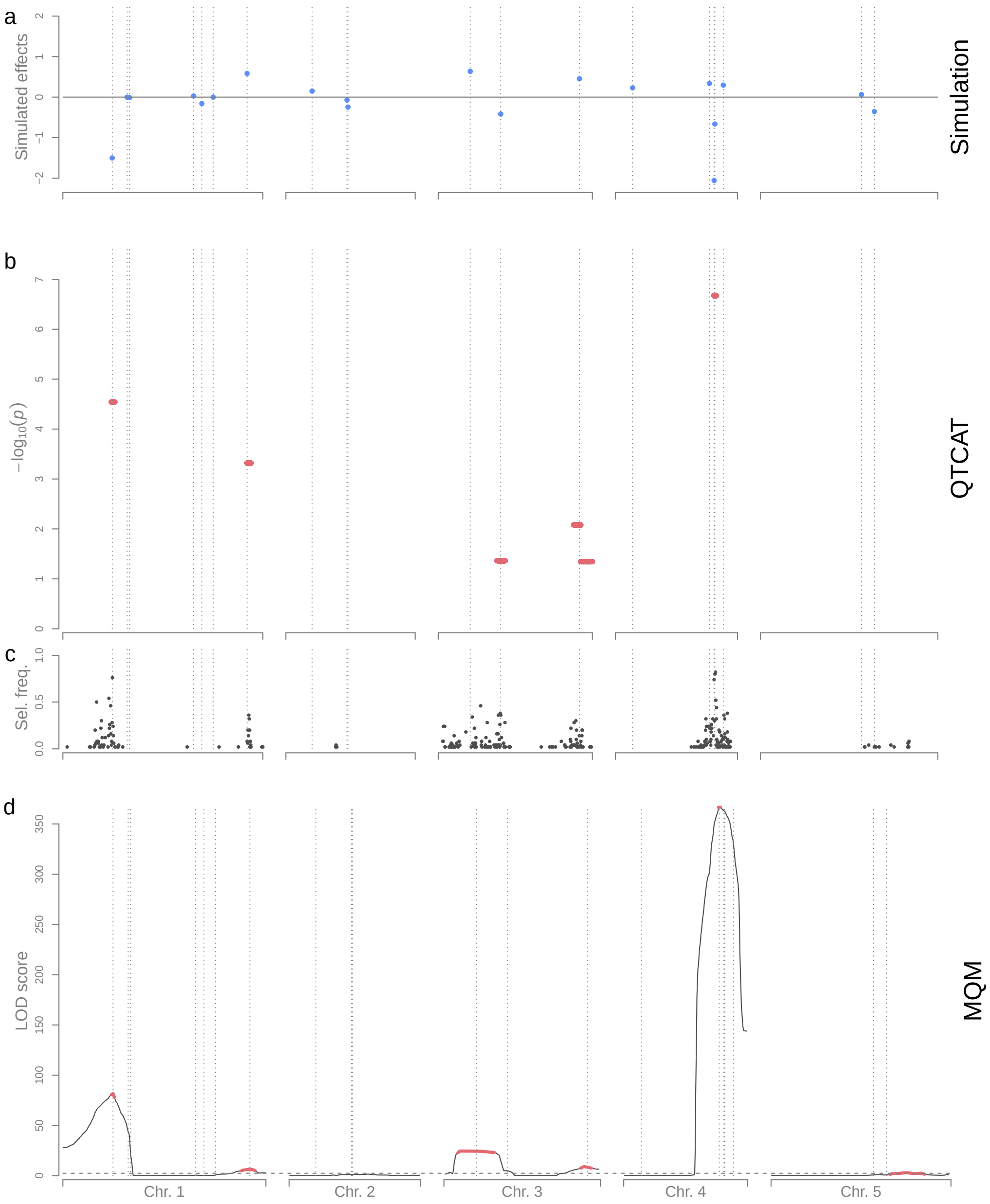

Supplementary Figure 305 Simulation of a GWA analysis based on a unstructured population with a heritability of 0.7 (run 5). (**a**) Simulated of 20 effects randomly drawn from a Gamma distribution and assigned them randomly to markers. Simulated effects randomly drawn from a Gamma distribution. We assigned effects to 20 markers. Markers with an effect are highlighted in (**b**–**d**) with dashed lines. (**b**) Significant QTCs found by QTCAT. (**c**) The selection frequency of the LASSO for each marker during the 50 iterations of QTCAT. (**d**) MQM LOD score plot, the horizontal dashed line is a simulation based perumtation test FDR. The red colored areas represent the LOD-intervals.

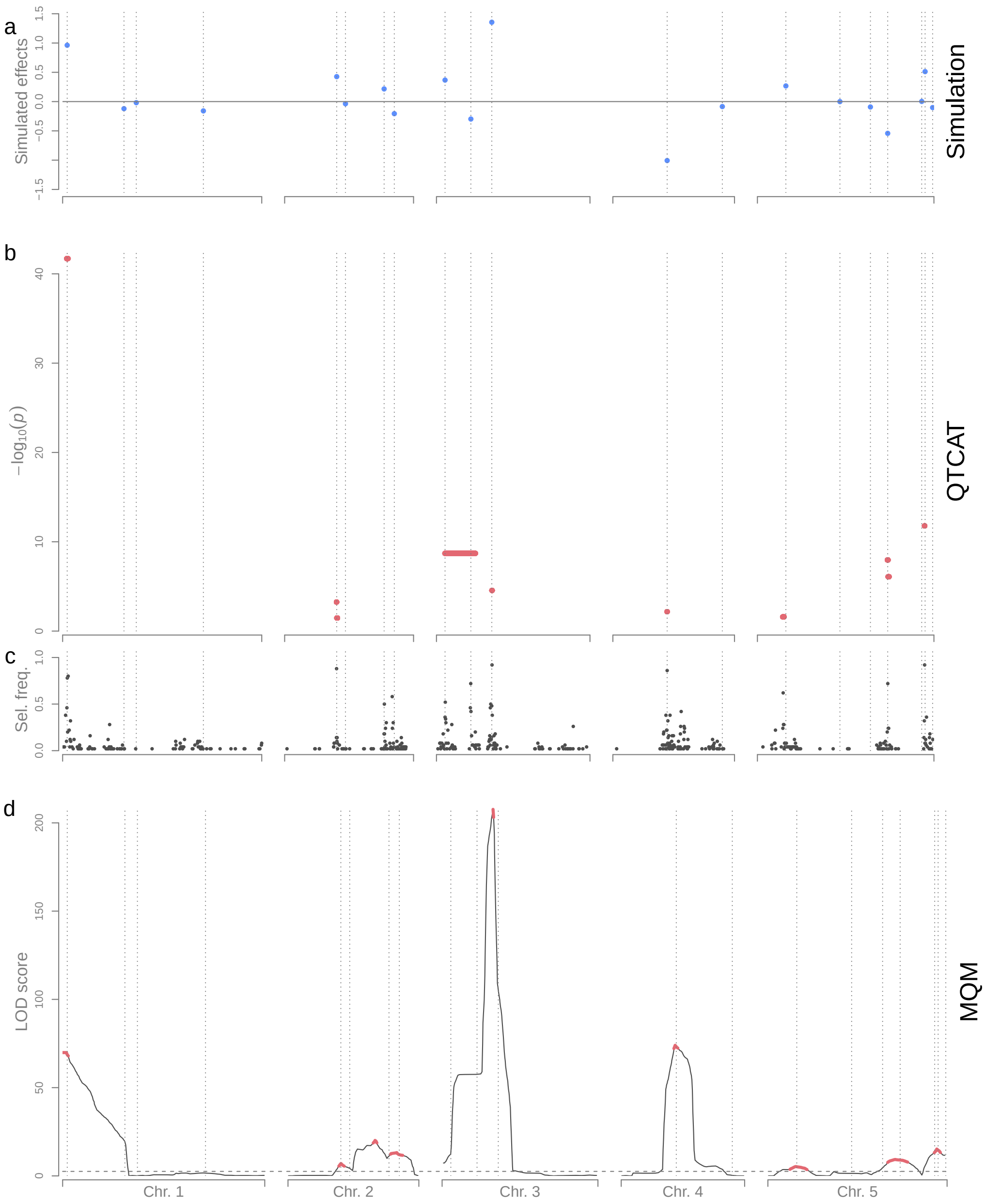

Supplementary Figure 306 Simulation of a GWA analysis based on a unstructured population with a heritability of 0.7 (run 6). (**a**) Simulated of 20 effects randomly drawn from a Gamma distribution and assigned them randomly to markers. Simulated effects randomly drawn from a Gamma distribution. We assigned effects to 20 markers. Markers with an effect are highlighted in (**b–d**) with dashed lines. (**b**) Significant QTCs found by QTCAT. (**c**) The selection frequency of the LASSO for each marker during the 50 iterations of QTCAT. (**d**) MQM LOD score plot, the horizontal dashed line is a simulation based perumtation test FDR. The red colored areas represent the LOD-intervals.

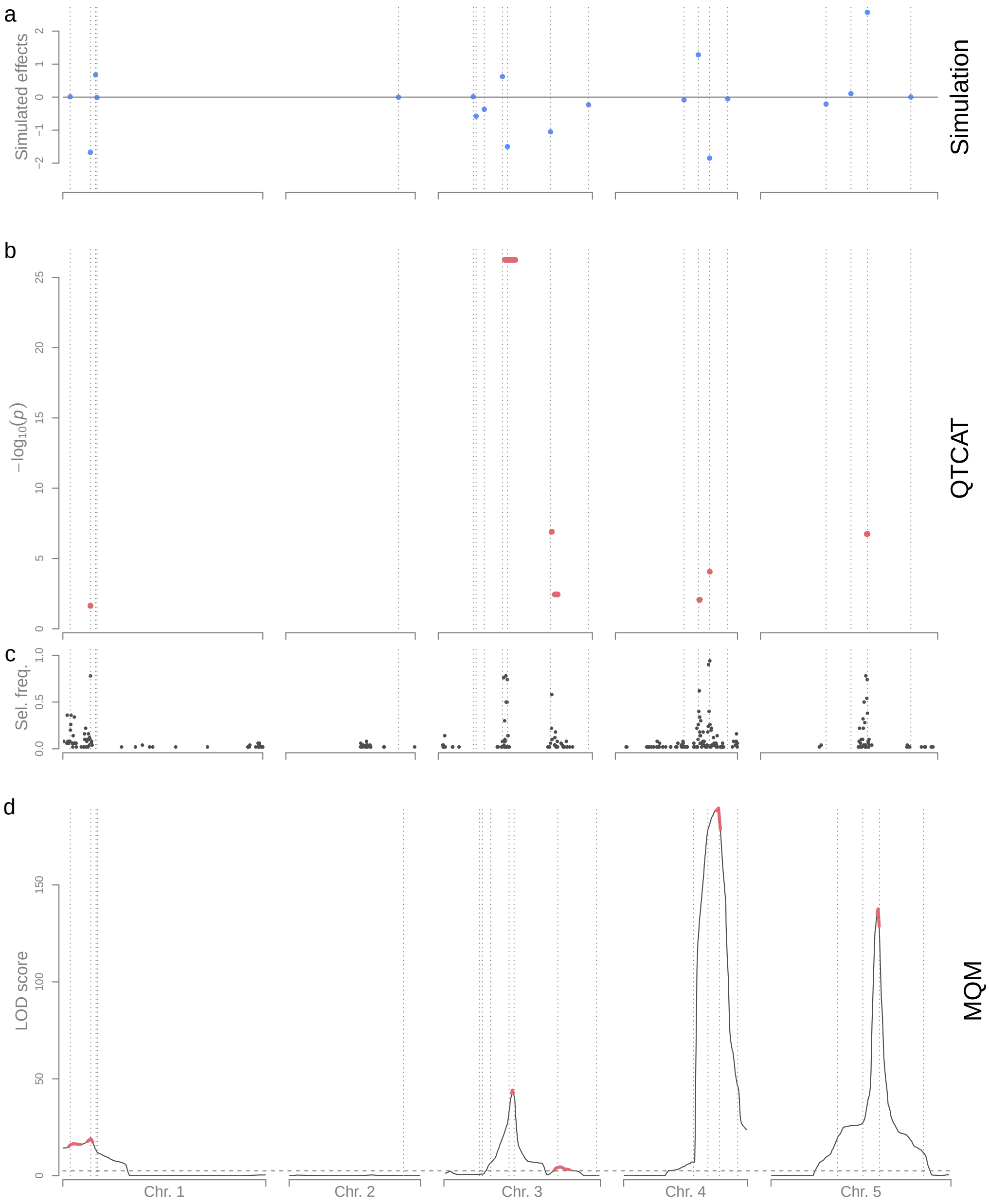

Supplementary Figure 307 Simulation of a GWA analysis based on a unstructured population with a heritability of 0.7 (run 7). (**a**) Simulated of 20 effects randomly drawn from a Gamma distribution and assigned them randomly to markers. Simulated effects randomly drawn from a Gamma distribution. We assigned effects to 20 markers. Markers with an effect are highlighted in (**b**–**d**) with dashed lines. (**b**) Significant QTCs found by QTCAT. (**c**) The selection frequency of the LASSO for each marker during the 50 iterations of QTCAT. (**d**) MQM LOD score plot, the horizontal dashed line is a simulation based perumtation test FDR. The red colored areas represent the LOD-intervals.

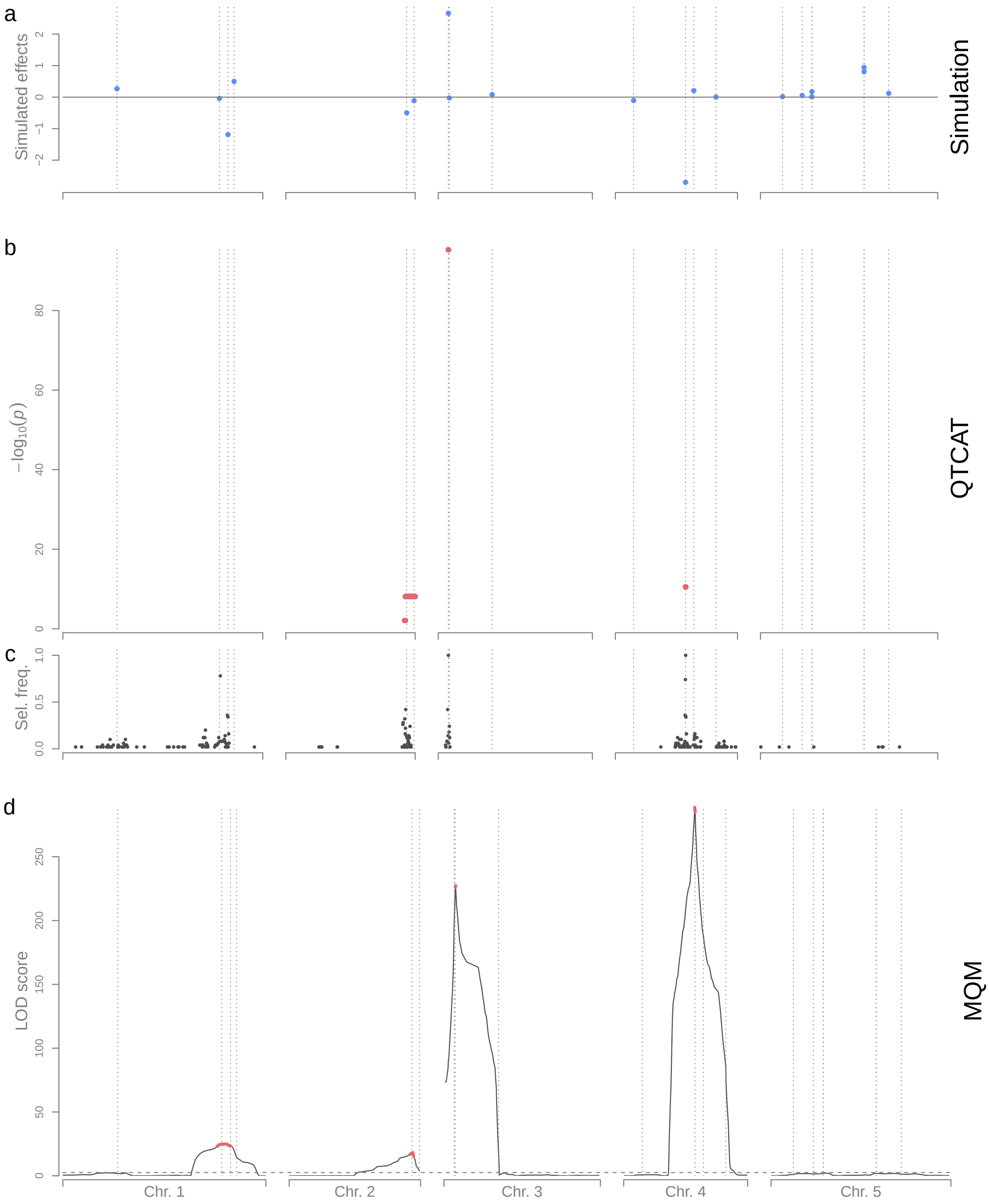

Supplementary Figure 308 Simulation of a GWA analysis based on a unstructured population with a heritability of 0.7 (run 8). (**a**) Simulated of 20 effects randomly drawn from a Gamma distribution and assigned them randomly to markers. Simulated effects randomly drawn from a Gamma distribution. We assigned effects to 20 markers. Markers with an effect are highlighted in (**b–d**) with dashed lines. (**b**) Significant QTCs found by QTCAT. (**c**) The selection frequency of the LASSO for each marker during the 50 iterations of QTCAT. (**d**) MQM LOD score plot, the horizontal dashed line is a simulation based perumtation test FDR. The red colored areas represent the LOD-intervals.

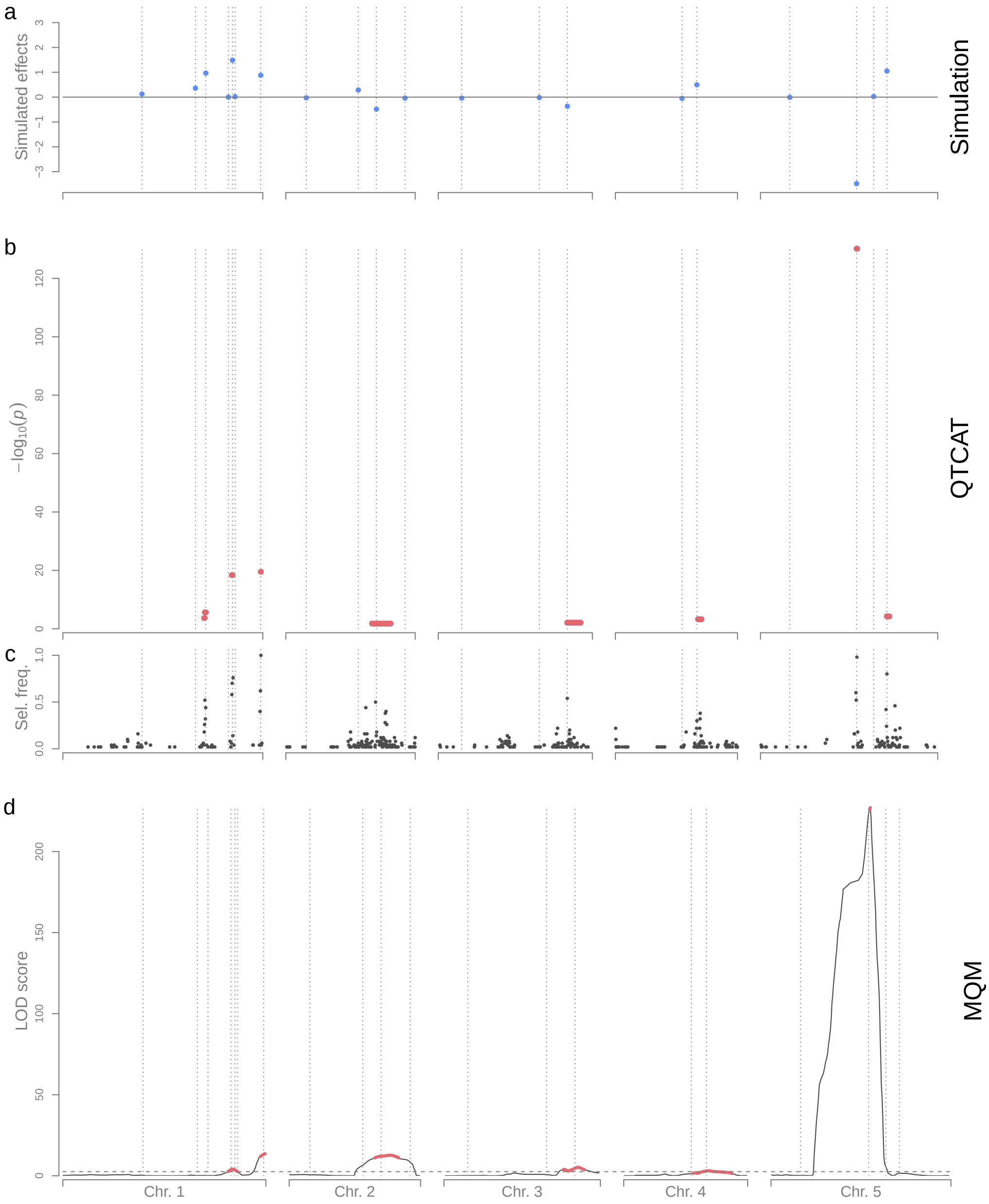

Supplementary Figure 309 Simulation of a GWA analysis based on a unstructured population with a heritability of 0.7 (run 9). (**a**) Simulated of 20 effects randomly drawn from a Gamma distribution and assigned them randomly to markers. Simulated effects randomly drawn from a Gamma distribution. We assigned effects to 20 markers. Markers with an effect are highlighted in (**b**–**d**) with dashed lines. (**b**) Significant QTCs found by QTCAT. (**c**) The selection frequency of the LASSO for each marker during the 50 iterations of QTCAT. (**d**) MQM LOD score plot, the horizontal dashed line is a simulation based perumtation test FDR. The red colored areas represent the LOD-intervals.

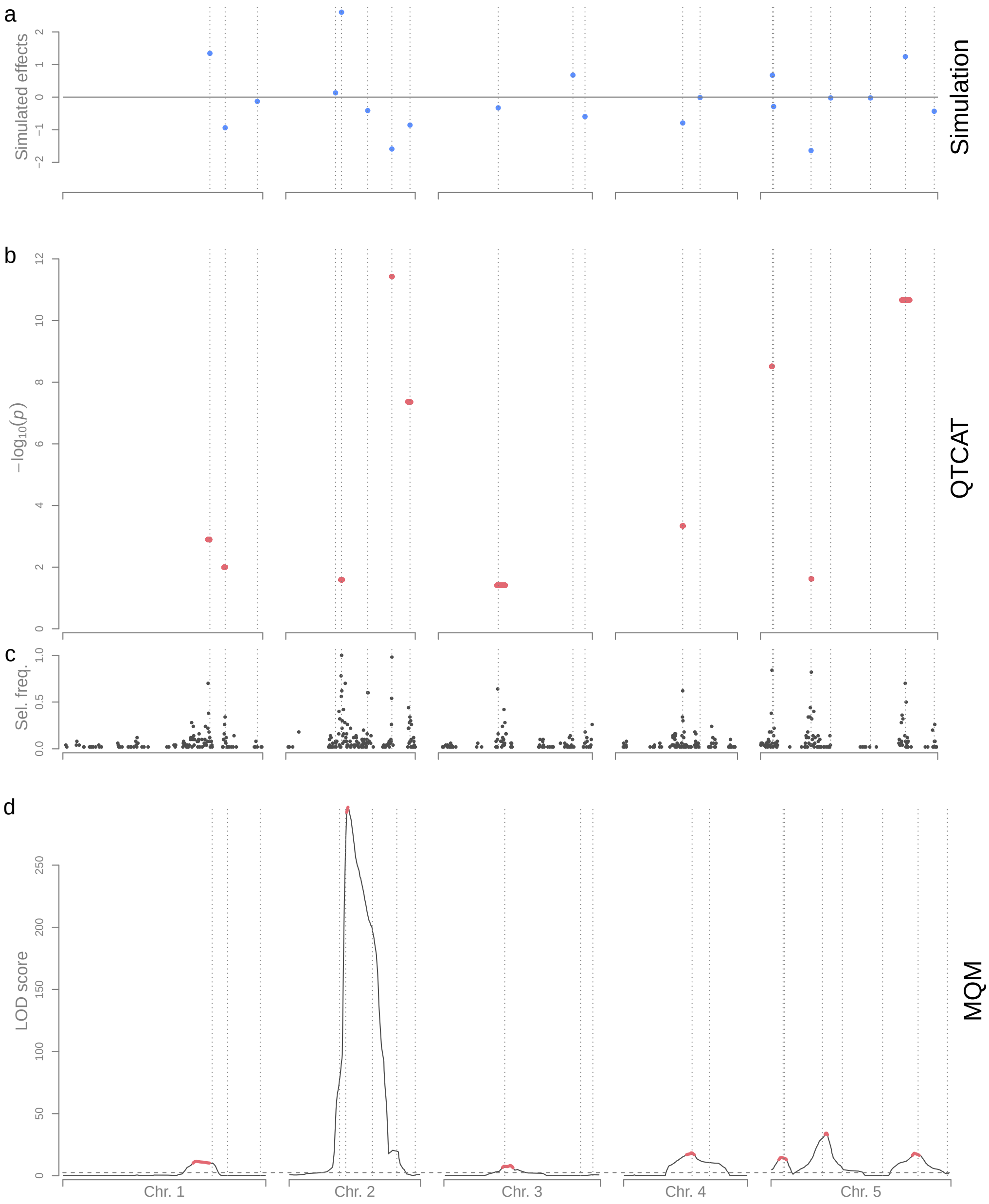

Supplementary Figure 310 Simulation of a GWA analysis based on a unstructured population with a heritability of 0.7 (run 10). (**a**) Simulated of 20 effects randomly drawn from a Gamma distribution and assigned them randomly to markers. Simulated effects randomly drawn from a Gamma distribution. We assigned effects to 20 markers. Markers with an effect are highlighted in (**b**–**d**) with dashed lines. (**b**) Significant QTCs found by QTCAT. (**c**) The selection frequency of the LASSO for each marker during the 50 iterations of QTCAT. (**d**) MQM LOD score plot, the horizontal dashed line is a simulation based perumtation test FDR. The red colored areas represent the LOD-intervals.

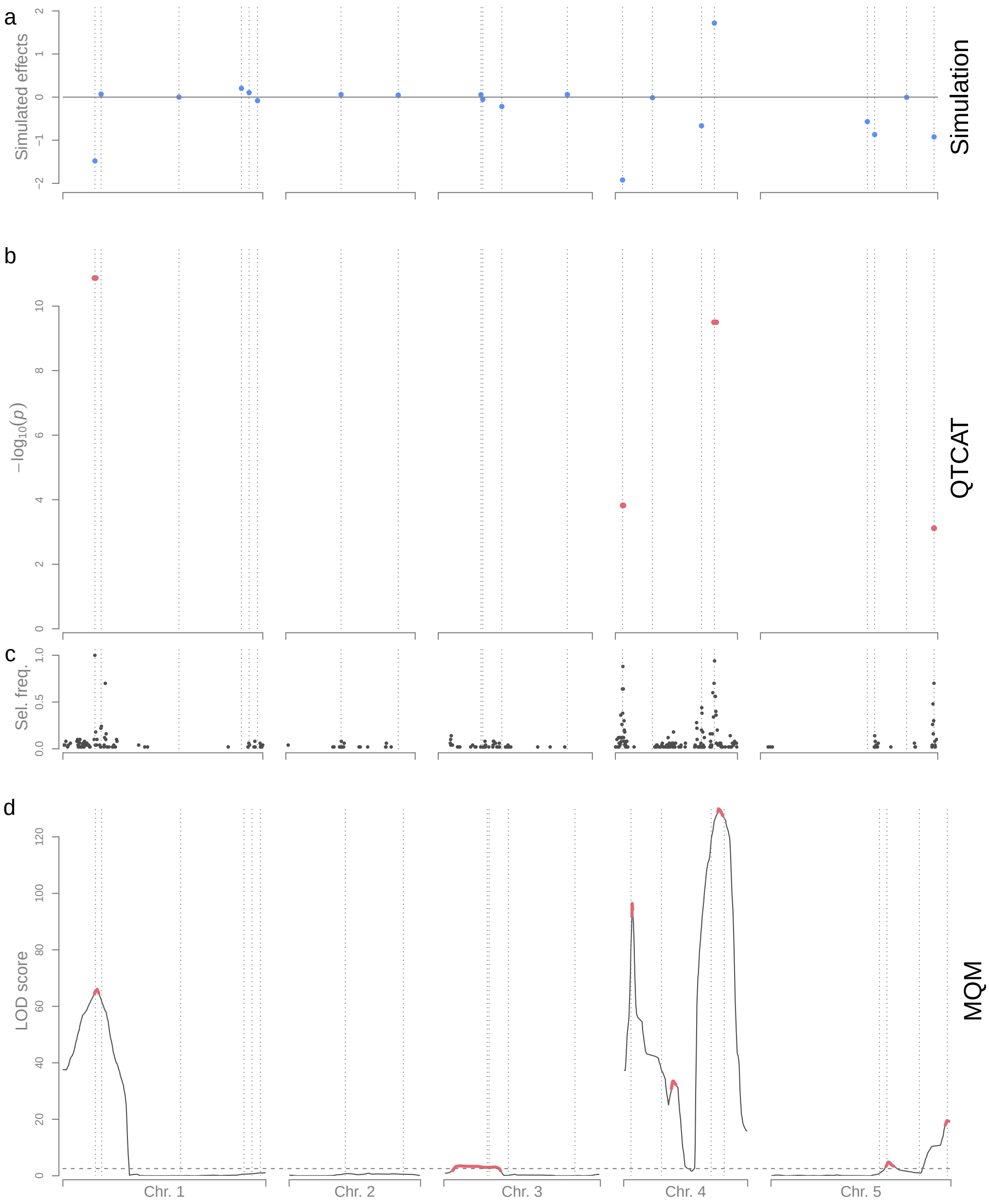

Supplementary Figure 311 Simulation of a GWA analysis based on a unstructured population with a heritability of 0.7 (run 11). (**a**) Simulated of 20 effects randomly drawn from a Gamma distribution and assigned them randomly to markers. Simulated effects randomly drawn from a Gamma distribution. We assigned effects to 20 markers. Markers with an effect are highlighted in (**b**–**d**) with dashed lines. (**b**) Significant QTCs found by QTCAT. (**c**) The selection frequency of the LASSO for each marker during the 50 iterations of QTCAT. (**d**) MQM LOD score plot, the horizontal dashed line is a simulation based perumtation test FDR. The red colored areas represent the LOD-intervals.

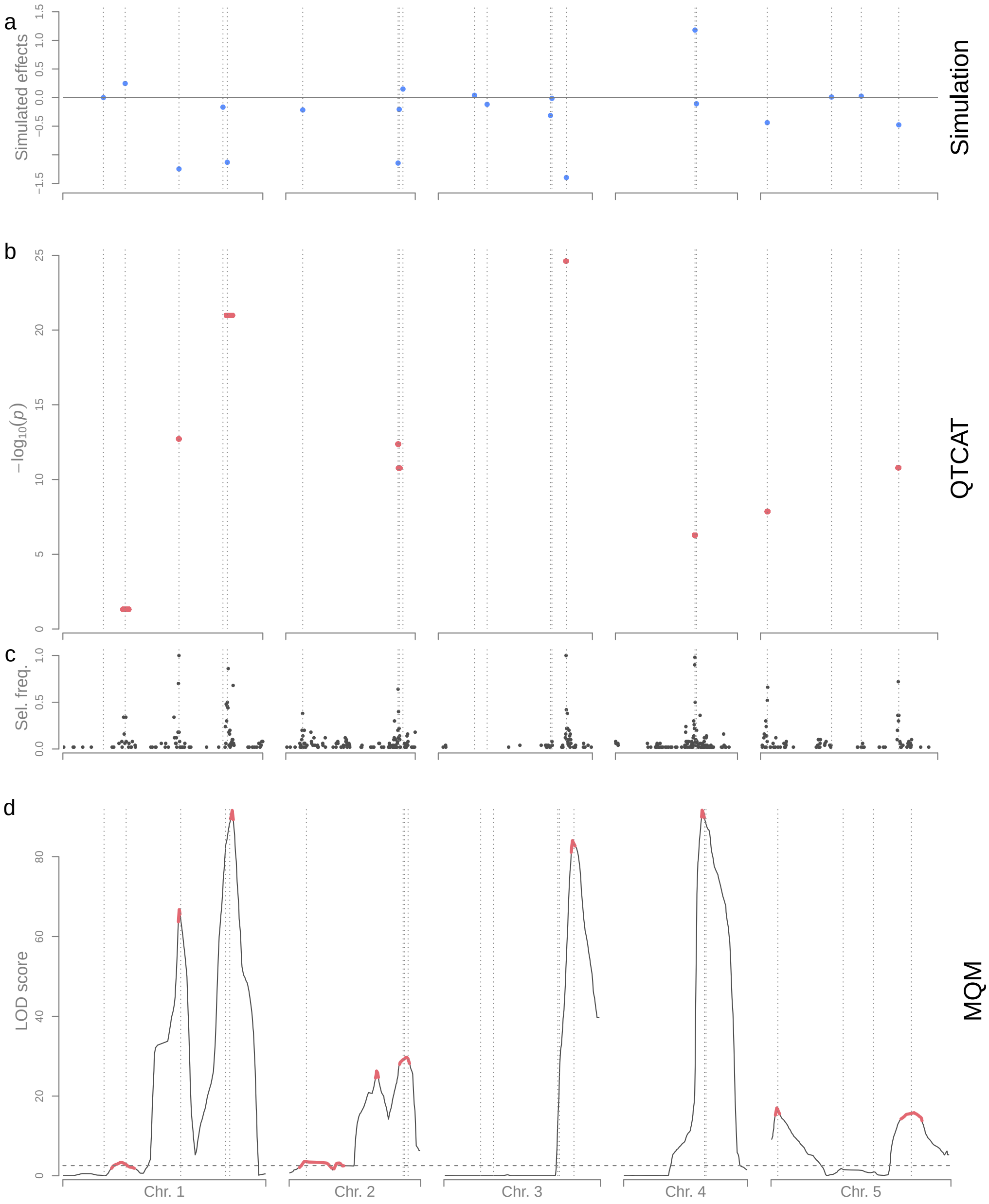

Supplementary Figure 312 Simulation of a GWA analysis based on a unstructured population with a heritability of 0.7 (run 12). (**a**) Simulated of 20 effects randomly drawn from a Gamma distribution and assigned them randomly to markers. Simulated effects randomly drawn from a Gamma distribution. We assigned effects to 20 markers. Markers with an effect are highlighted in (**b**–**d**) with dashed lines. (**b**) Significant QTCs found by QTCAT. (**c**) The selection frequency of the LASSO for each marker during the 50 iterations of QTCAT. (**d**) MQM LOD score plot, the horizontal dashed line is a simulation based perumtation test FDR. The red colored areas represent the LOD-intervals.

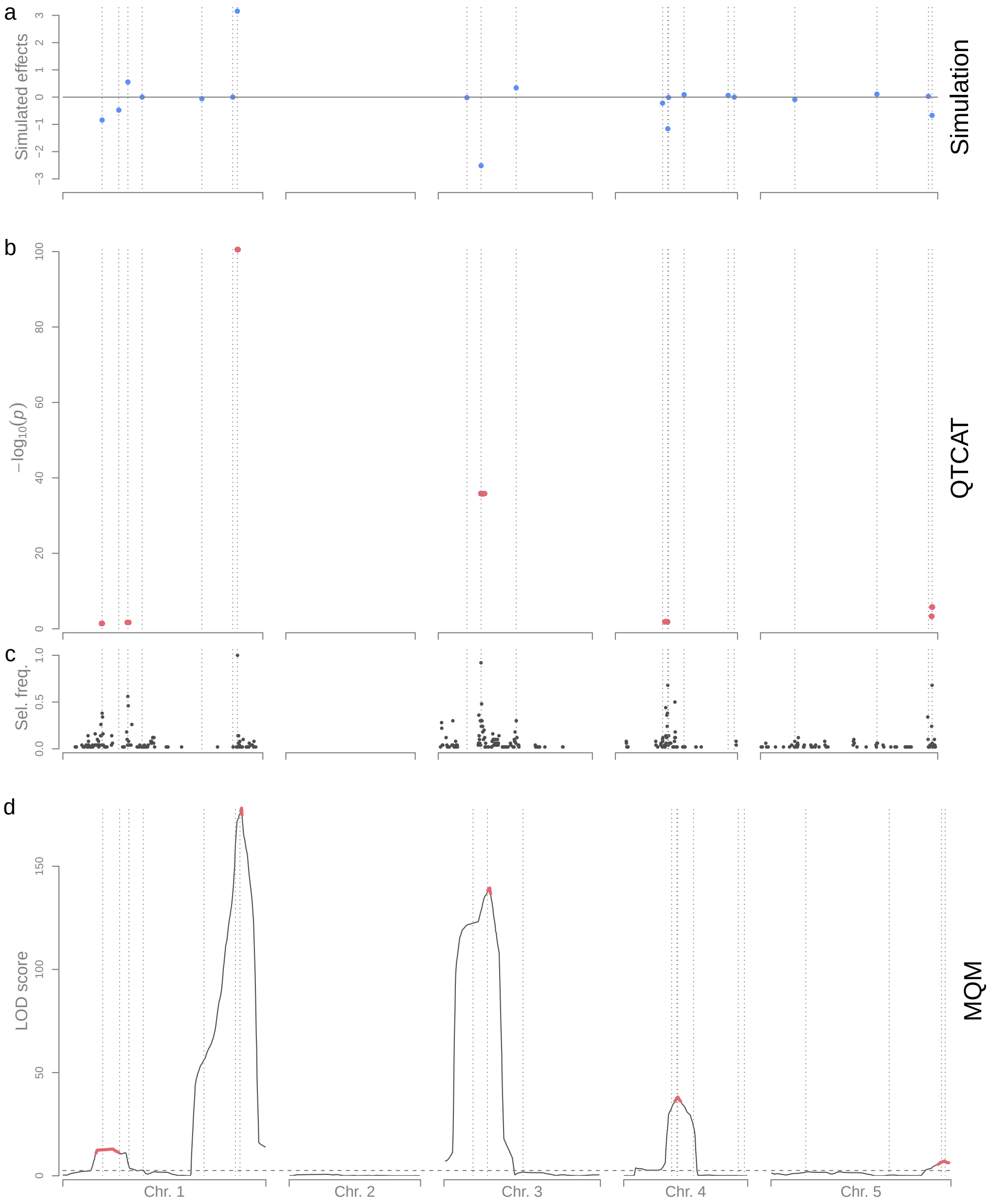

Supplementary Figure 313 Simulation of a GWA analysis based on a unstructured population with a heritability of 0.7 (run 13). (**a**) Simulated of 20 effects randomly drawn from a Gamma distribution and assigned them randomly to markers. Simulated effects randomly drawn from a Gamma distribution. We assigned effects to 20 markers. Markers with an effect are highlighted in (**b–d**) with dashed lines. (**b**) Significant QTCs found by QTCAT. (**c**) The selection frequency of the LASSO for each marker during the 50 iterations of QTCAT. (**d**) MQM LOD score plot, the horizontal dashed line is a simulation based perumtation test FDR. The red colored areas represent the LOD-intervals.

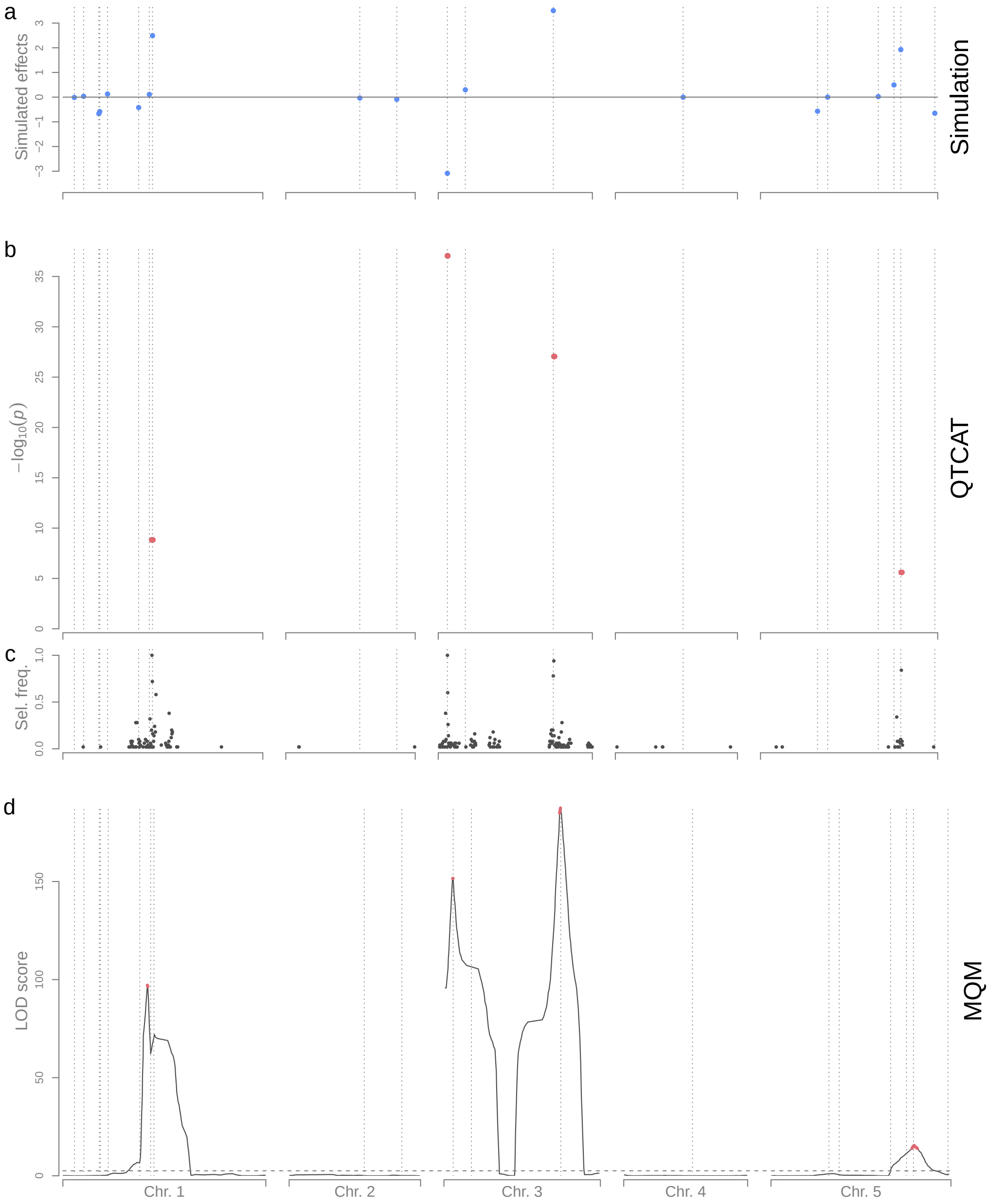

Supplementary Figure 314 Simulation of a GWA analysis based on a unstructured population with a heritability of 0.7 (run 14). (**a**) Simulated of 20 effects randomly drawn from a Gamma distribution and assigned them randomly to markers. Simulated effects randomly drawn from a Gamma distribution. We assigned effects to 20 markers. Markers with an effect are highlighted in (**b**–**d**) with dashed lines. (**b**) Significant QTCs found by QTCAT. (**c**) The selection frequency of the LASSO for each marker during the 50 iterations of QTCAT. (**d**) MQM LOD score plot, the horizontal dashed line is a simulation based perumtation test FDR. The red colored areas represent the LOD-intervals.

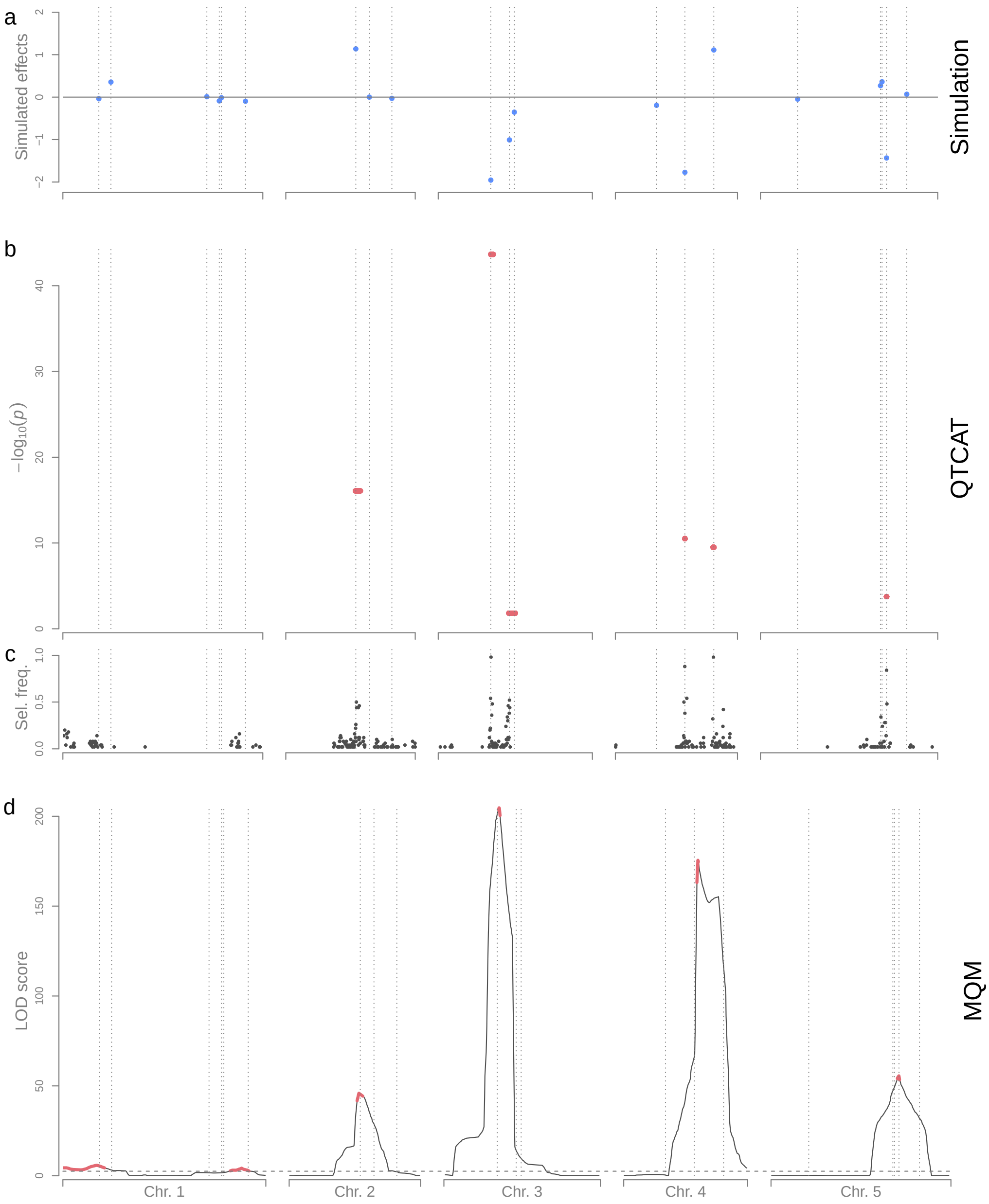

Supplementary Figure 315 Simulation of a GWA analysis based on a unstructured population with a heritability of 0.7 (run 15). (**a**) Simulated of 20 effects randomly drawn from a Gamma distribution and assigned them randomly to markers. Simulated effects randomly drawn from a Gamma distribution. We assigned effects to 20 markers. Markers with an effect are highlighted in (**b**–**d**) with dashed lines. (**b**) Significant QTCs found by QTCAT. (**c**) The selection frequency of the LASSO for each marker during the 50 iterations of QTCAT. (**d**) MQM LOD score plot, the horizontal dashed line is a simulation based perumtation test FDR. The red colored areas represent the LOD-intervals.

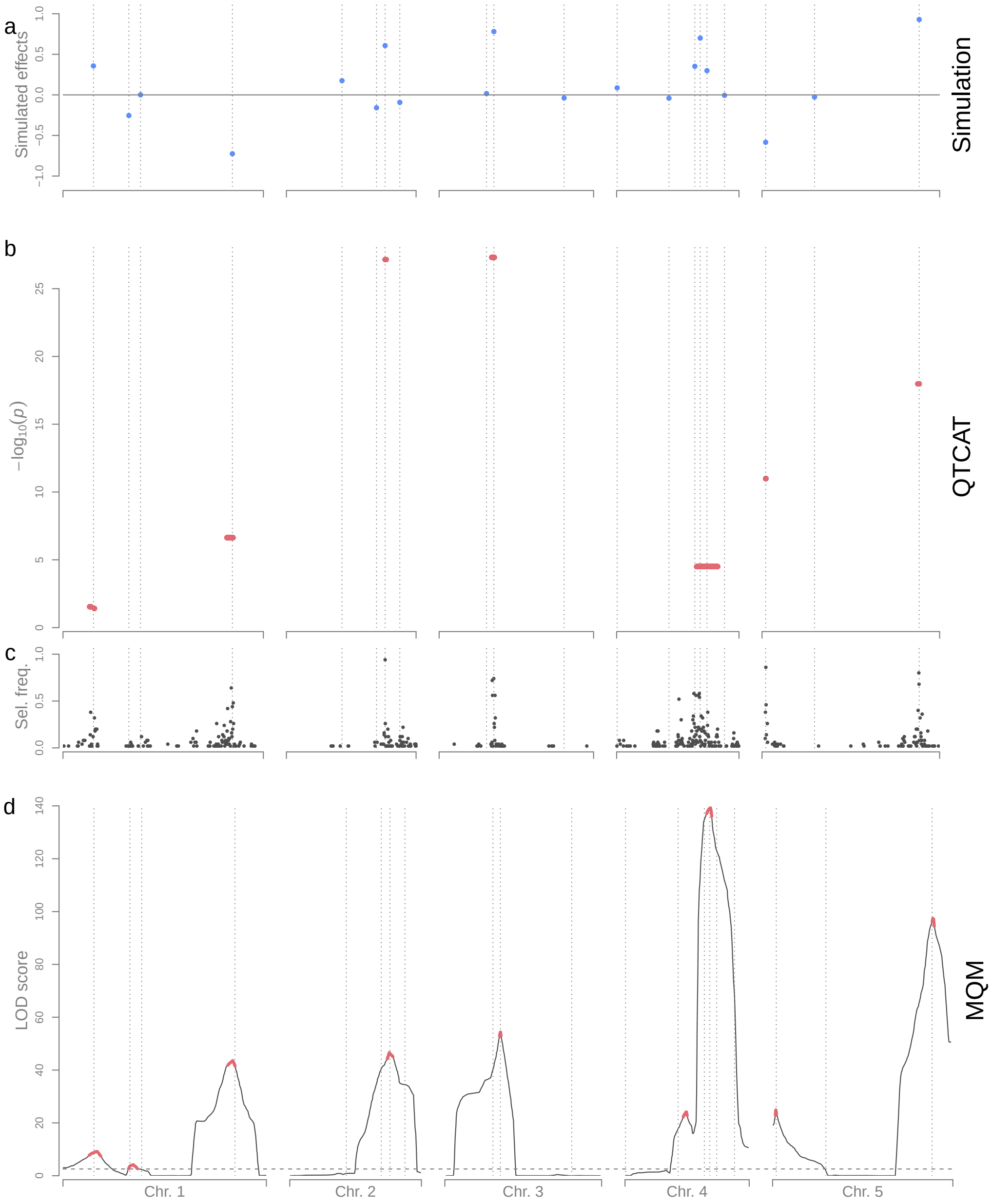

Supplementary Figure 316 Simulation of a GWA analysis based on a unstructured population with a heritability of 0.7 (run 16). (**a**) Simulated of 20 effects randomly drawn from a Gamma distribution and assigned them randomly to markers. Simulated effects randomly drawn from a Gamma distribution. We assigned effects to 20 markers. Markers with an effect are highlighted in (**b**–**d**) with dashed lines. (**b**) Significant QTCs found by QTCAT. (**c**) The selection frequency of the LASSO for each marker during the 50 iterations of QTCAT. (**d**) MQM LOD score plot, the horizontal dashed line is a simulation based perumtation test FDR. The red colored areas represent the LOD-intervals.

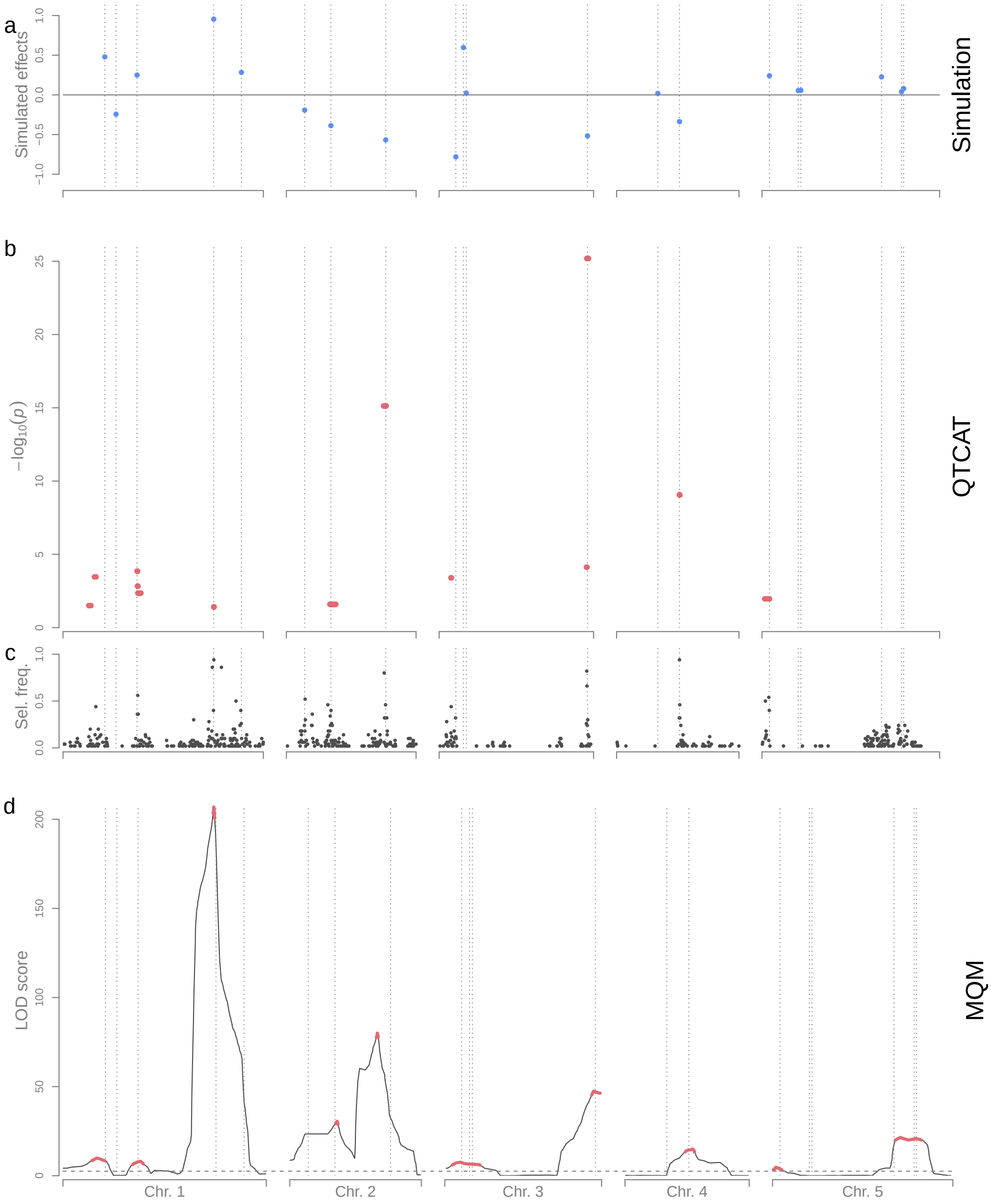

Supplementary Figure 317 Simulation of a GWA analysis based on a unstructured population with a heritability of 0.7 (run 17). (**a**) Simulated of 20 effects randomly drawn from a Gamma distribution and assigned them randomly to markers. Simulated effects randomly drawn from a Gamma distribution. We assigned effects to 20 markers. Markers with an effect are highlighted in (**b**–**d**) with dashed lines. (**b**) Significant QTCs found by QTCAT. (**c**) The selection frequency of the LASSO for each marker during the 50 iterations of QTCAT. (**d**) MQM LOD score plot, the horizontal dashed line is a simulation based perumtation test FDR. The red colored areas represent the LOD-intervals.

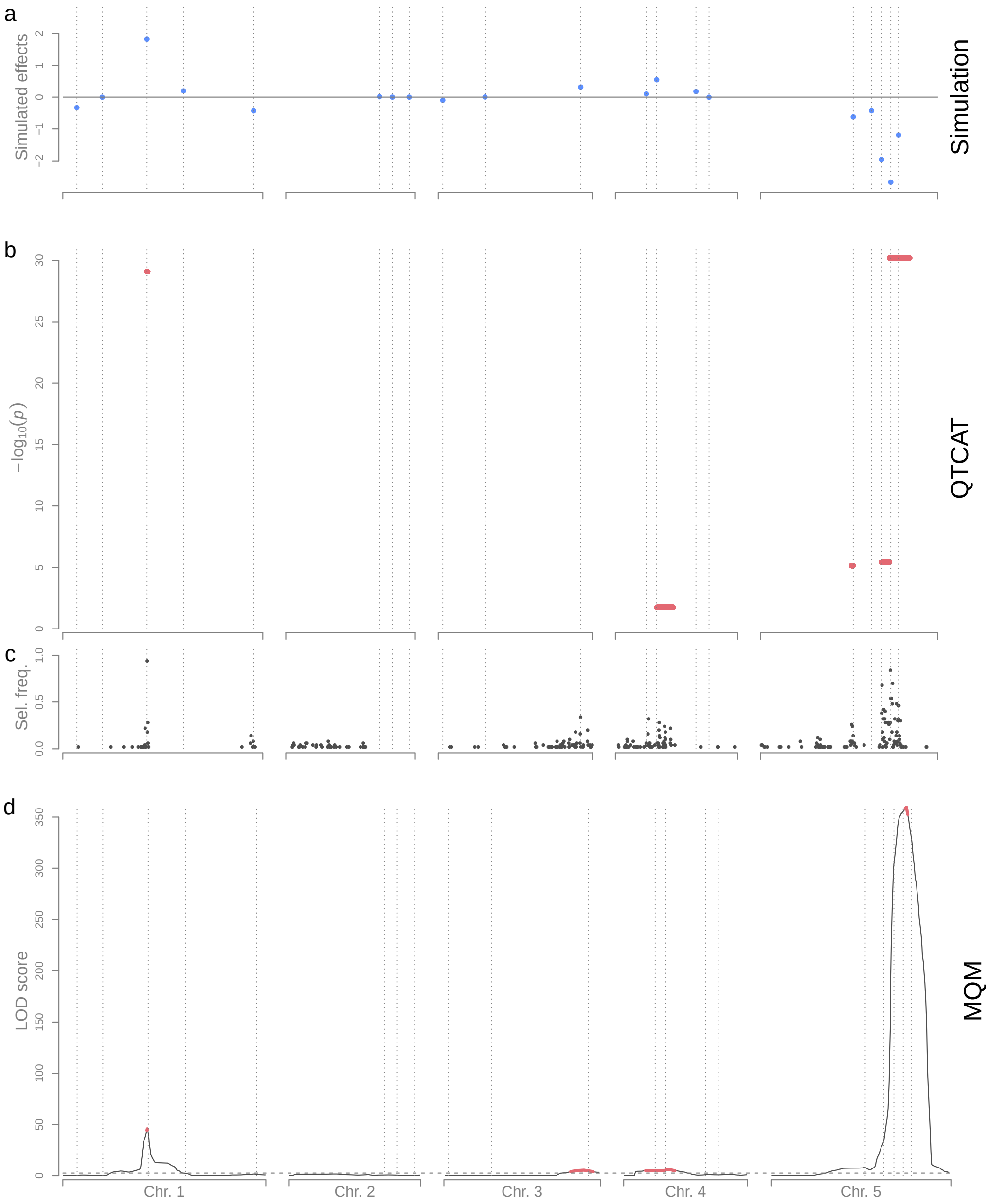

Supplementary Figure 318 Simulation of a GWA analysis based on a unstructured population with a heritability of 0.7 (run 18). (**a**) Simulated of 20 effects randomly drawn from a Gamma distribution and assigned them randomly to markers. Simulated effects randomly drawn from a Gamma distribution. We assigned effects to 20 markers. Markers with an effect are highlighted in (**b–d**) with dashed lines. (**b**) Significant QTCs found by QTCAT. (**c**) The selection frequency of the LASSO for each marker during the 50 iterations of QTCAT. (**d**) MQM LOD score plot, the horizontal dashed line is a simulation based perumtation test FDR. The red colored areas represent the LOD-intervals.

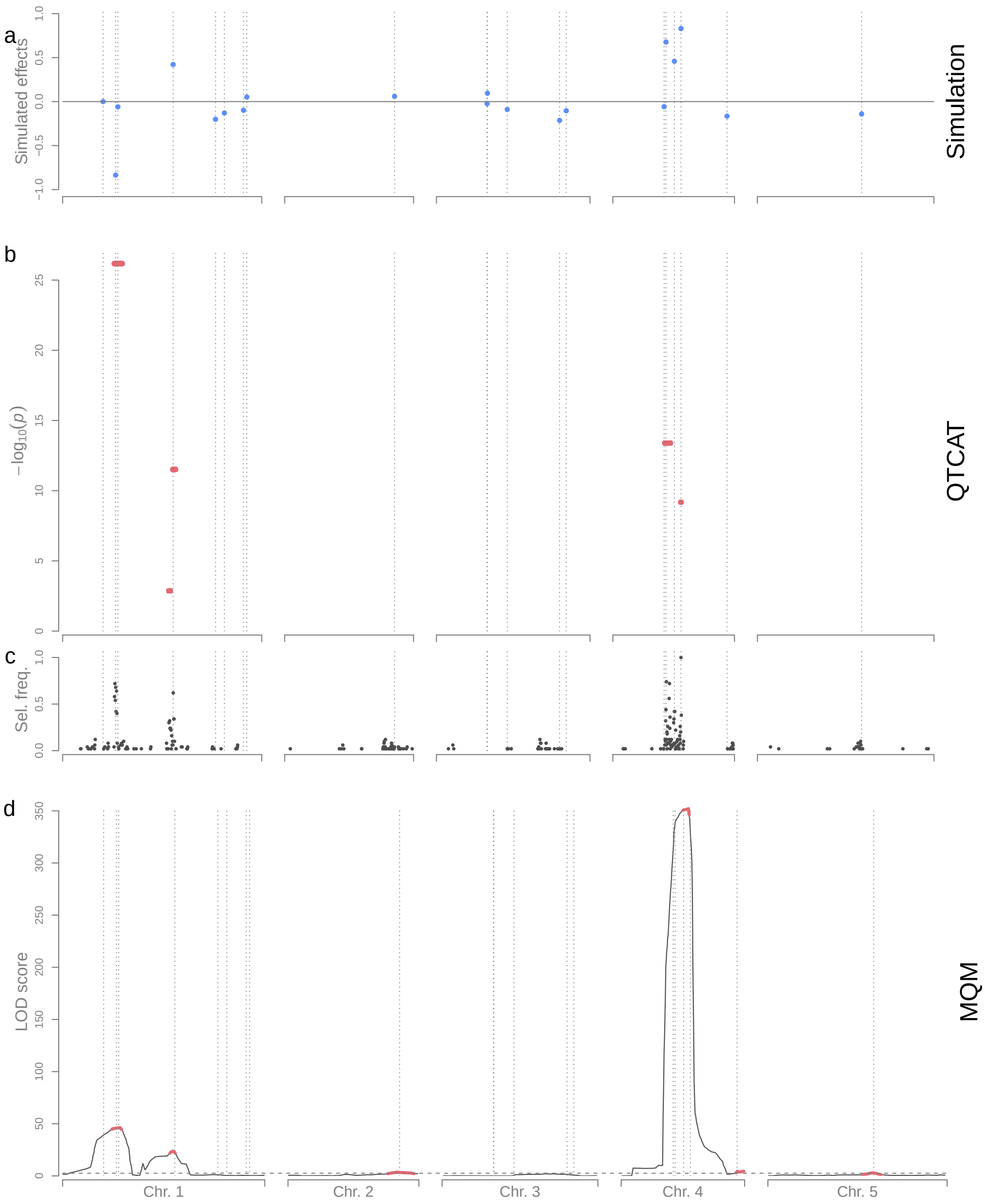

Supplementary Figure 319 Simulation of a GWA analysis based on a unstructured population with a heritability of 0.7 (run 19). (**a**) Simulated of 20 effects randomly drawn from a Gamma distribution and assigned them randomly to markers. Simulated effects randomly drawn from a Gamma distribution. We assigned effects to 20 markers. Markers with an effect are highlighted in (**b–d**) with dashed lines. (**b**) Significant QTCs found by QTCAT. (**c**) The selection frequency of the LASSO for each marker during the 50 iterations of QTCAT. (**d**) MQM LOD score plot, the horizontal dashed line is a simulation based perumtation test FDR. The red colored areas represent the LOD-intervals.

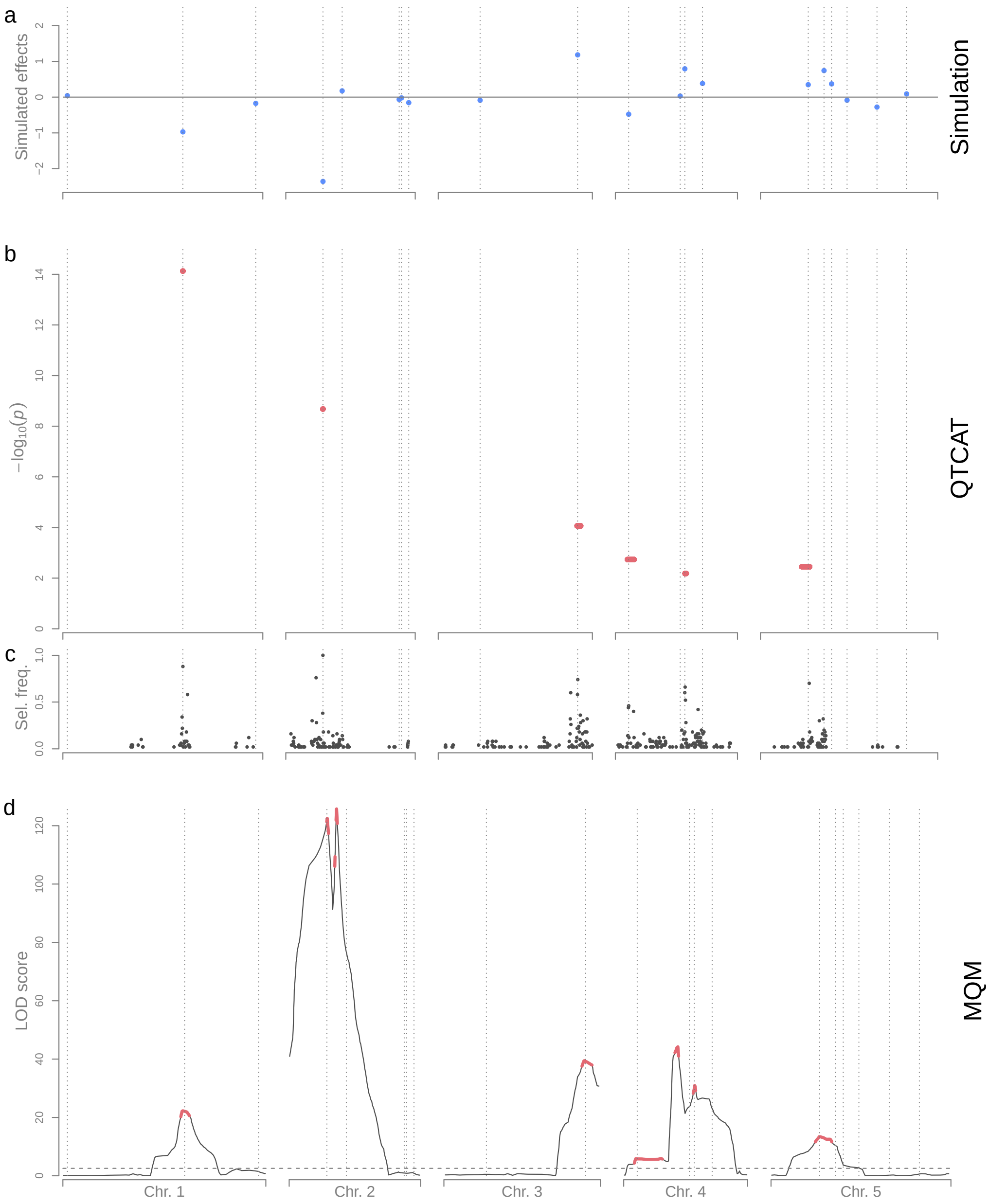

Supplementary Figure 320 Simulation of a GWA analysis based on a unstructured population with a heritability of 0.7 (run 20). (**a**) Simulated of 20 effects randomly drawn from a Gamma distribution and assigned them randomly to markers. Simulated effects randomly drawn from a Gamma distribution. We assigned effects to 20 markers. Markers with an effect are highlighted in (**b–d**) with dashed lines. (**b**) Significant QTCs found by QTCAT. (**c**) The selection frequency of the LASSO for each marker during the 50 iterations of QTCAT. (**d**) MQM LOD score plot, the horizontal dashed line is a simulation based perumtation test FDR. The red colored areas represent the LOD-intervals.

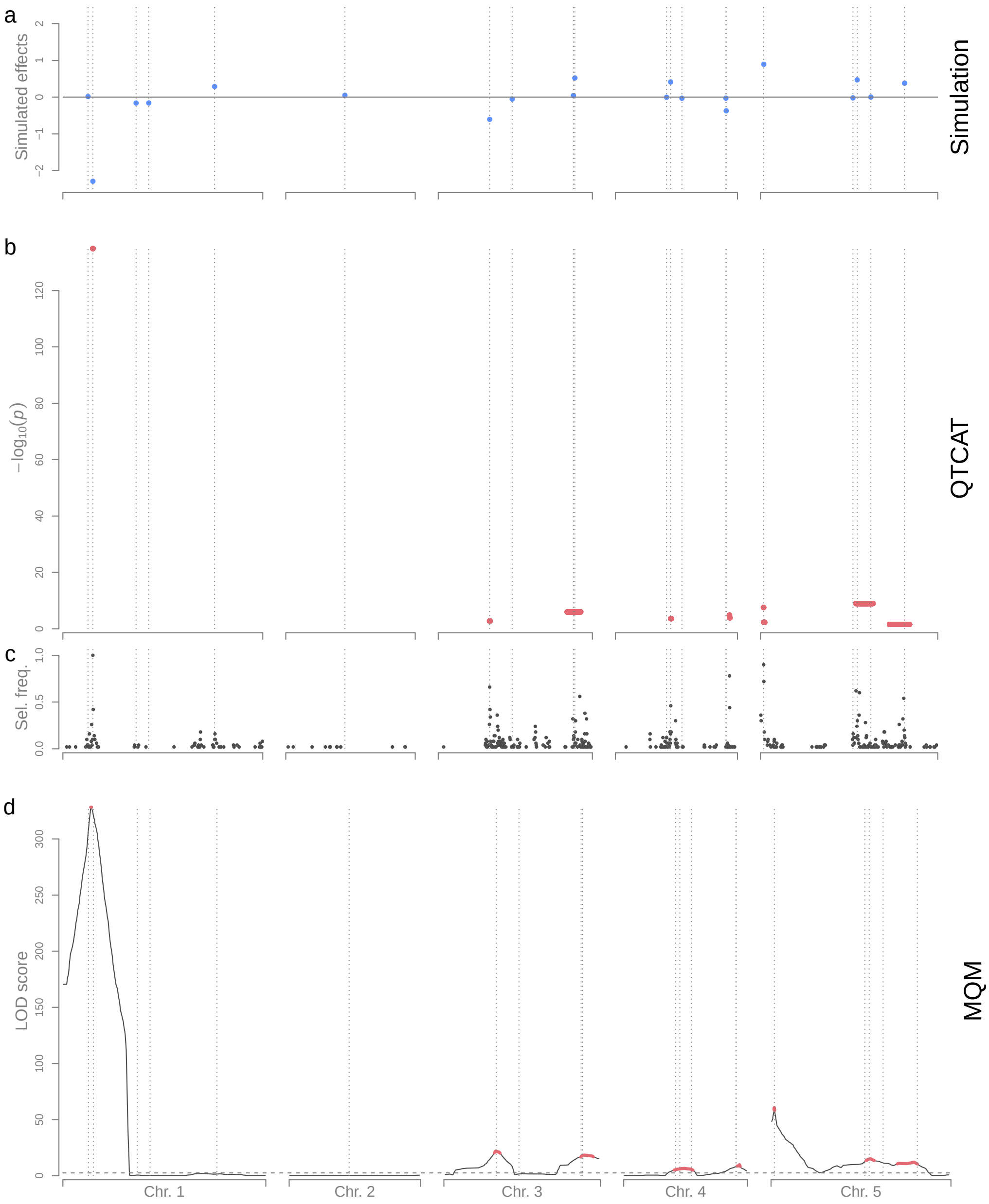

Supplementary Figure 321 Simulation of a GWA analysis based on a unstructured population with a heritability of 0.7 (run 21). (**a**) Simulated of 20 effects randomly drawn from a Gamma distribution and assigned them randomly to markers. Simulated effects randomly drawn from a Gamma distribution. We assigned effects to 20 markers. Markers with an effect are highlighted in (**b**–**d**) with dashed lines. (**b**) Significant QTCs found by QTCAT. (**c**) The selection frequency of the LASSO for each marker during the 50 iterations of QTCAT. (**d**) MQM LOD score plot, the horizontal dashed line is a simulation based perumtation test FDR. The red colored areas represent the LOD-intervals.

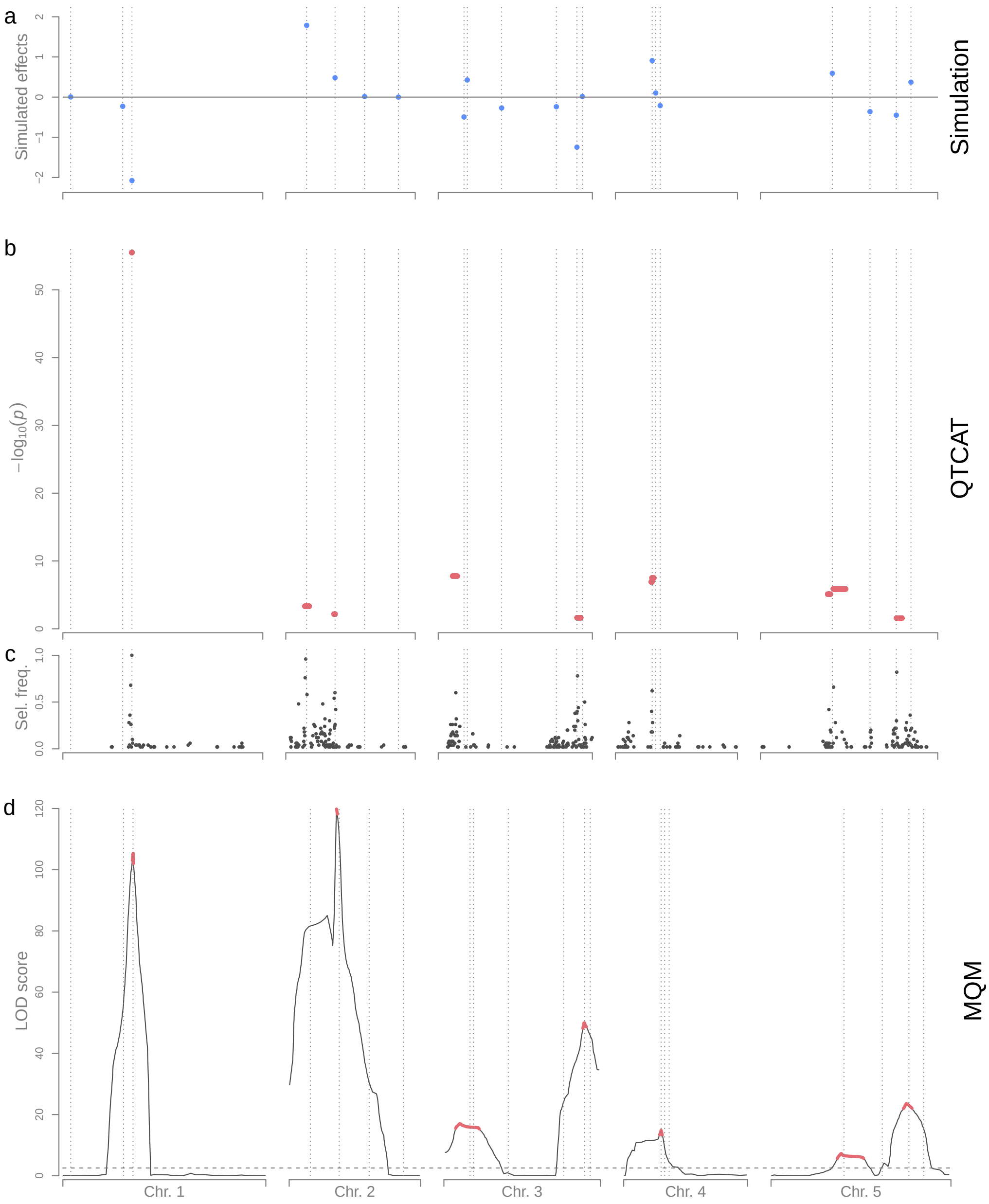

Supplementary Figure 322 Simulation of a GWA analysis based on a unstructured population with a heritability of 0.7 (run 22). (**a**) Simulated of 20 effects randomly drawn from a Gamma distribution and assigned them randomly to markers. Simulated effects randomly drawn from a Gamma distribution. We assigned effects to 20 markers. Markers with an effect are highlighted in (**b**–**d**) with dashed lines. (**b**) Significant QTCs found by QTCAT. (**c**) The selection frequency of the LASSO for each marker during the 50 iterations of QTCAT. (**d**) MQM LOD score plot, the horizontal dashed line is a simulation based perumtation test FDR. The red colored areas represent the LOD-intervals.

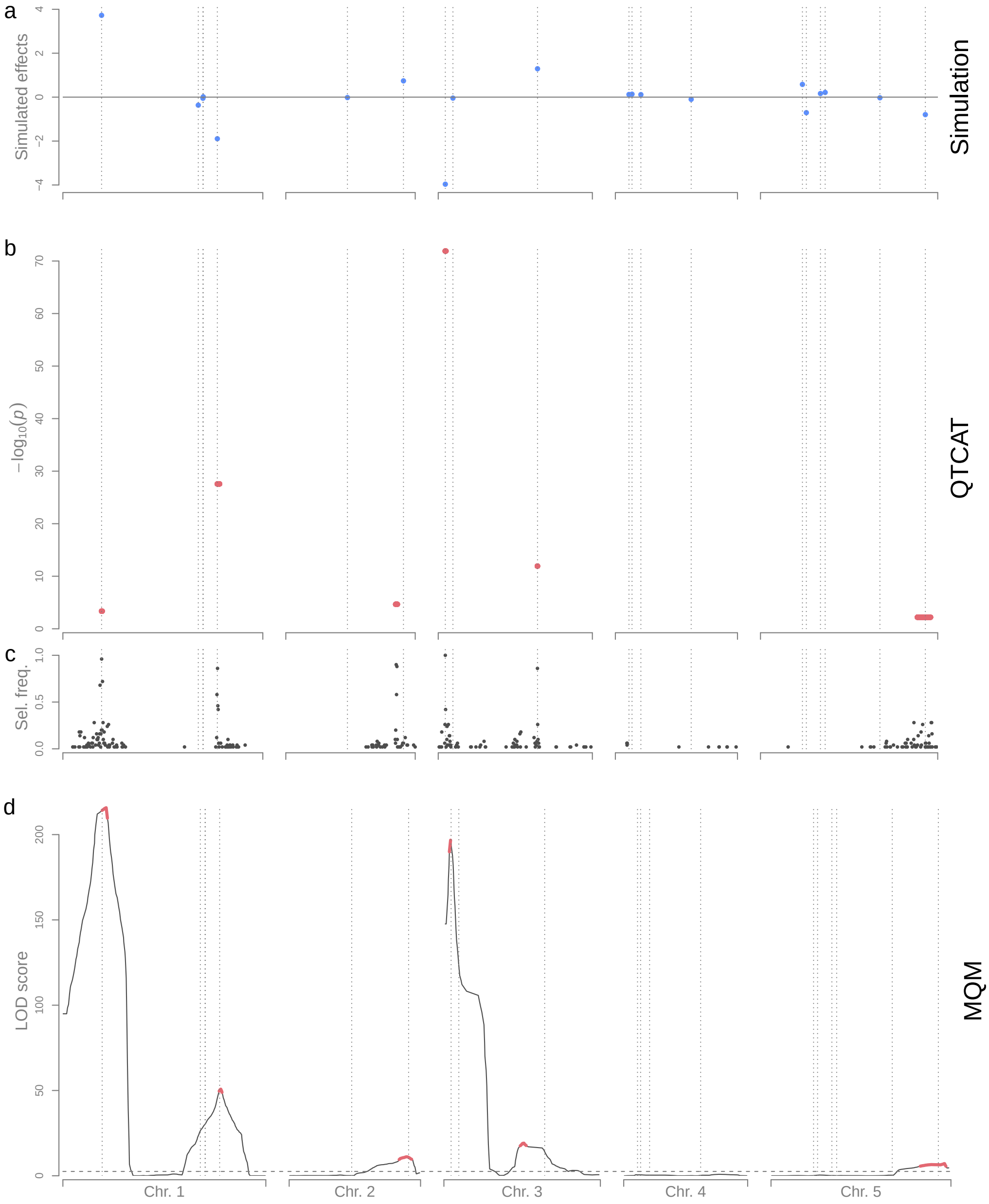

Supplementary Figure 323 Simulation of a GWA analysis based on a unstructured population with a heritability of 0.7 (run 23). (**a**) Simulated of 20 effects randomly drawn from a Gamma distribution and assigned them randomly to markers. Simulated effects randomly drawn from a Gamma distribution. We assigned effects to 20 markers. Markers with an effect are highlighted in (**b**–**d**) with dashed lines. (**b**) Significant QTCs found by QTCAT. (**c**) The selection frequency of the LASSO for each marker during the 50 iterations of QTCAT. (**d**) MQM LOD score plot, the horizontal dashed line is a simulation based perumtation test FDR. The red colored areas represent the LOD-intervals.

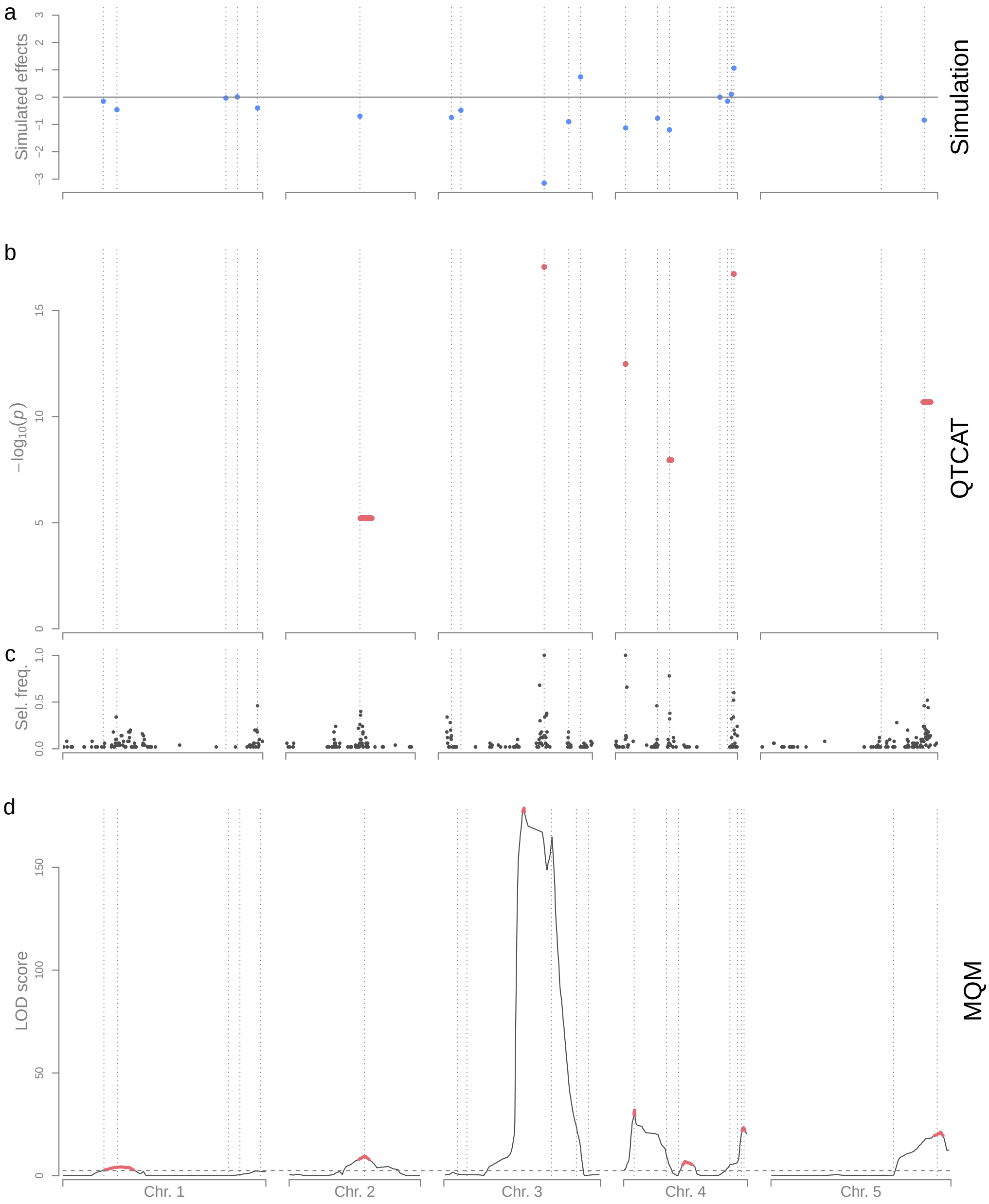

Supplementary Figure 324 Simulation of a GWA analysis based on a unstructured population with a heritability of 0.7 (run 24). (**a**) Simulated of 20 effects randomly drawn from a Gamma distribution and assigned them randomly to markers. Simulated effects randomly drawn from a Gamma distribution. We assigned effects to 20 markers. Markers with an effect are highlighted in (**b–d**) with dashed lines. (**b**) Significant QTCs found by QTCAT. (**c**) The selection frequency of the LASSO for each marker during the 50 iterations of QTCAT. (**d**) MQM LOD score plot, the horizontal dashed line is a simulation based perumtation test FDR. The red colored areas represent the LOD-intervals.

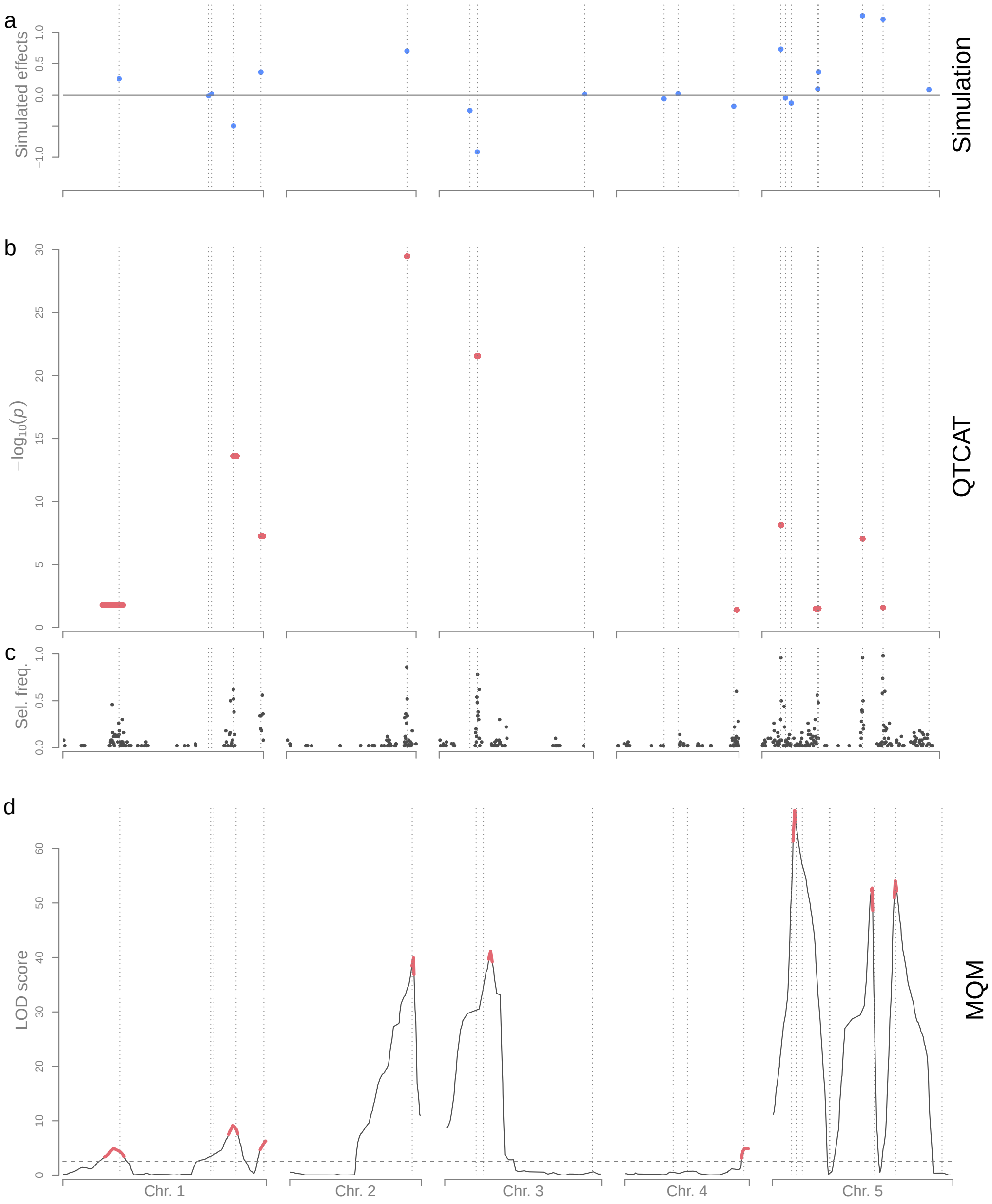

Supplementary Figure 325 Simulation of a GWA analysis based on a unstructured population with a heritability of 0.7 (run 25). (**a**) Simulated of 20 effects randomly drawn from a Gamma distribution and assigned them randomly to markers. Simulated effects randomly drawn from a Gamma distribution. We assigned effects to 20 markers. Markers with an effect are highlighted in (**b–d**) with dashed lines. (**b**) Significant QTCs found by QTCAT. (**c**) The selection frequency of the LASSO for each marker during the 50 iterations of QTCAT. (**d**) MQM LOD score plot, the horizontal dashed line is a simulation based perumtation test FDR. The red colored areas represent the LOD-intervals.

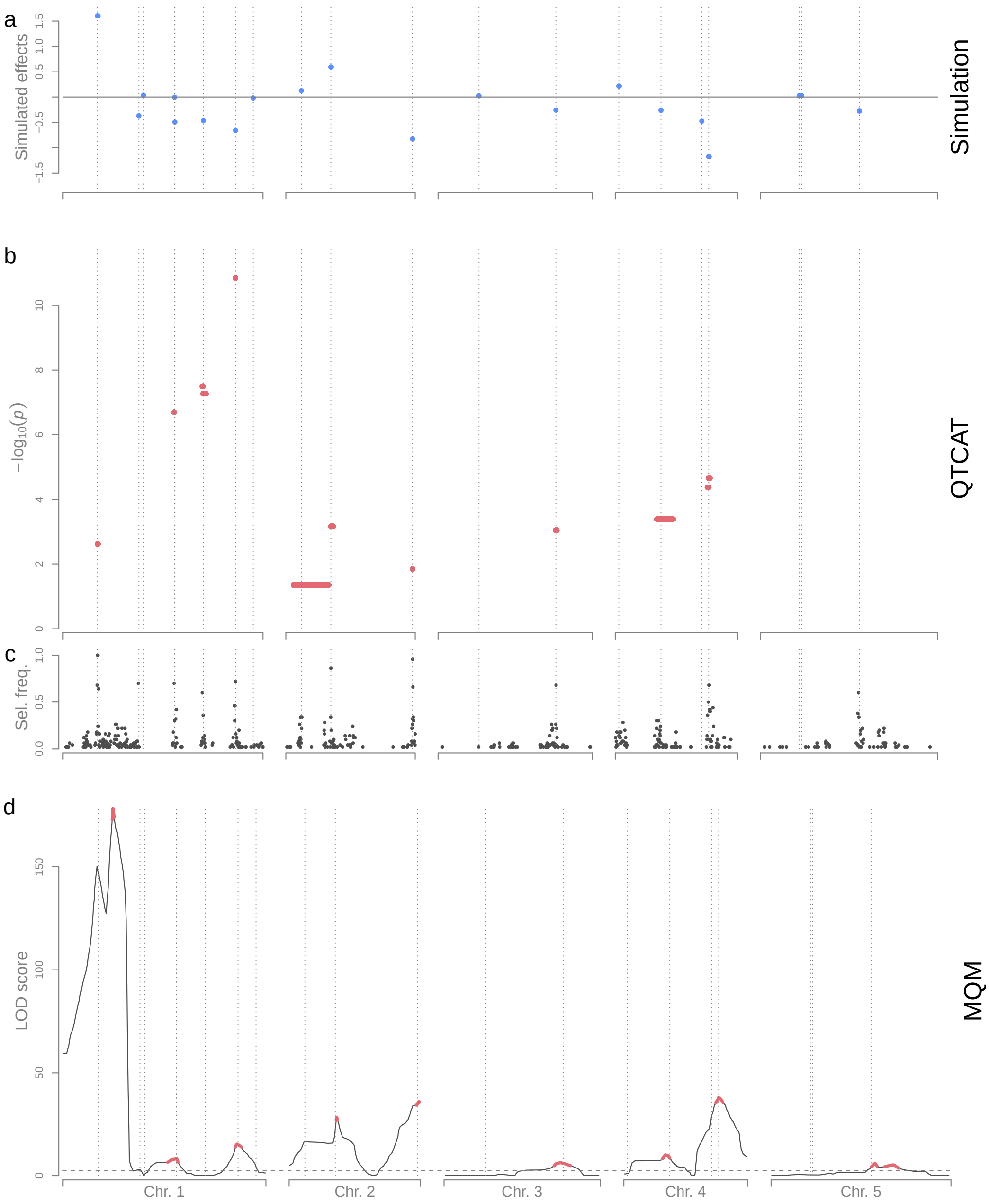

Supplementary Figure 326 Simulation of a GWA analysis based on a unstructured population with a heritability of 0.7 (run 26). (**a**) Simulated of 20 effects randomly drawn from a Gamma distribution and assigned them randomly to markers. Simulated effects randomly drawn from a Gamma distribution. We assigned effects to 20 markers. Markers with an effect are highlighted in (**b–d**) with dashed lines. (**b**) Significant QTCs found by QTCAT. (**c**) The selection frequency of the LASSO for each marker during the 50 iterations of QTCAT. (**d**) MQM LOD score plot, the horizontal dashed line is a simulation based perumtation test FDR. The red colored areas represent the LOD-intervals.

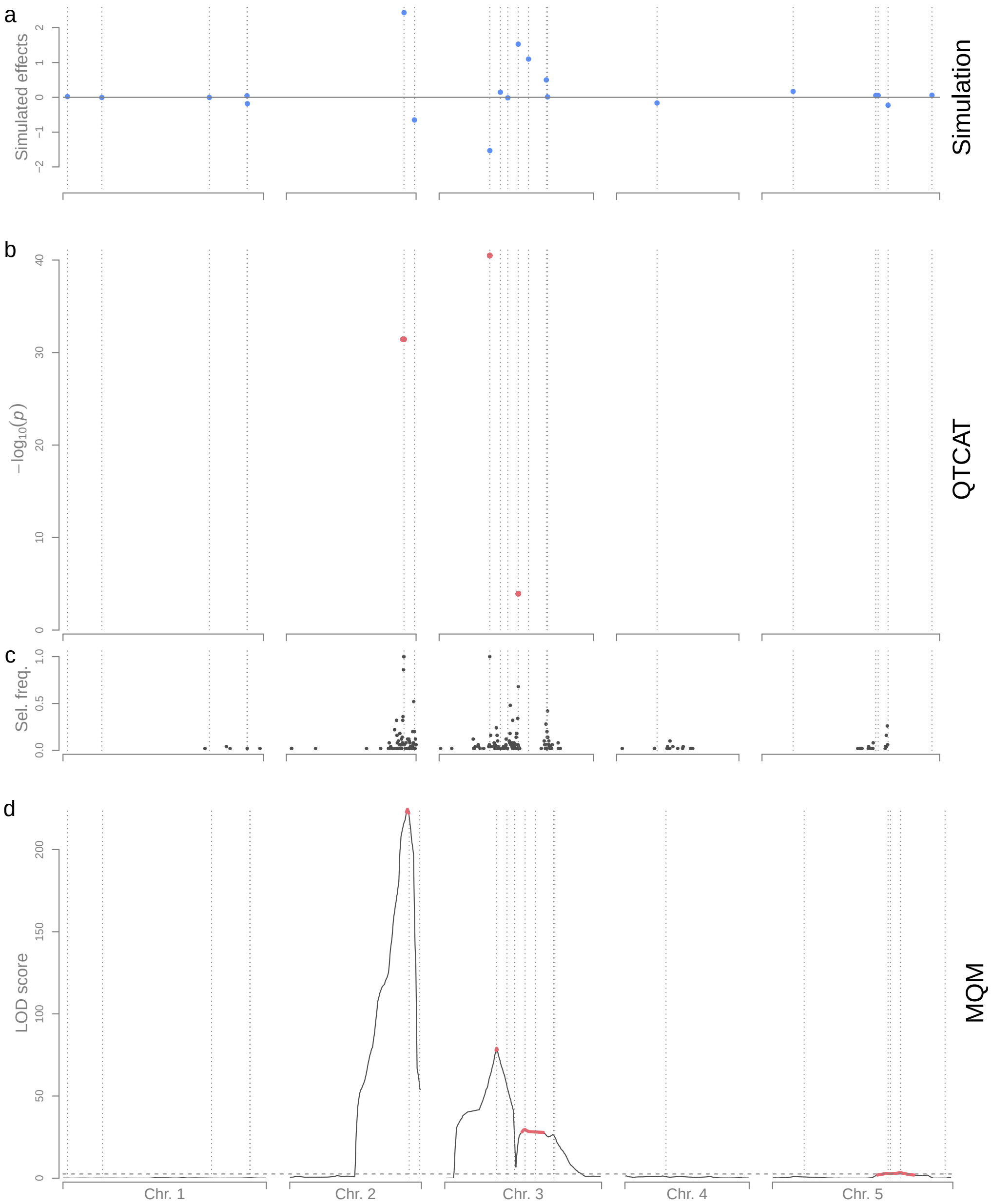

Supplementary Figure 327 Simulation of a GWA analysis based on a unstructured population with a heritability of 0.7 (run 27). (**a**) Simulated of 20 effects randomly drawn from a Gamma distribution and assigned them randomly to markers. Simulated effects randomly drawn from a Gamma distribution. We assigned effects to 20 markers. Markers with an effect are highlighted in (**b–d**) with dashed lines. (**b**) Significant QTCs found by QTCAT. (**c**) The selection frequency of the LASSO for each marker during the 50 iterations of QTCAT. (**d**) MQM LOD score plot, the horizontal dashed line is a simulation based perumtation test FDR. The red colored areas represent the LOD-intervals.

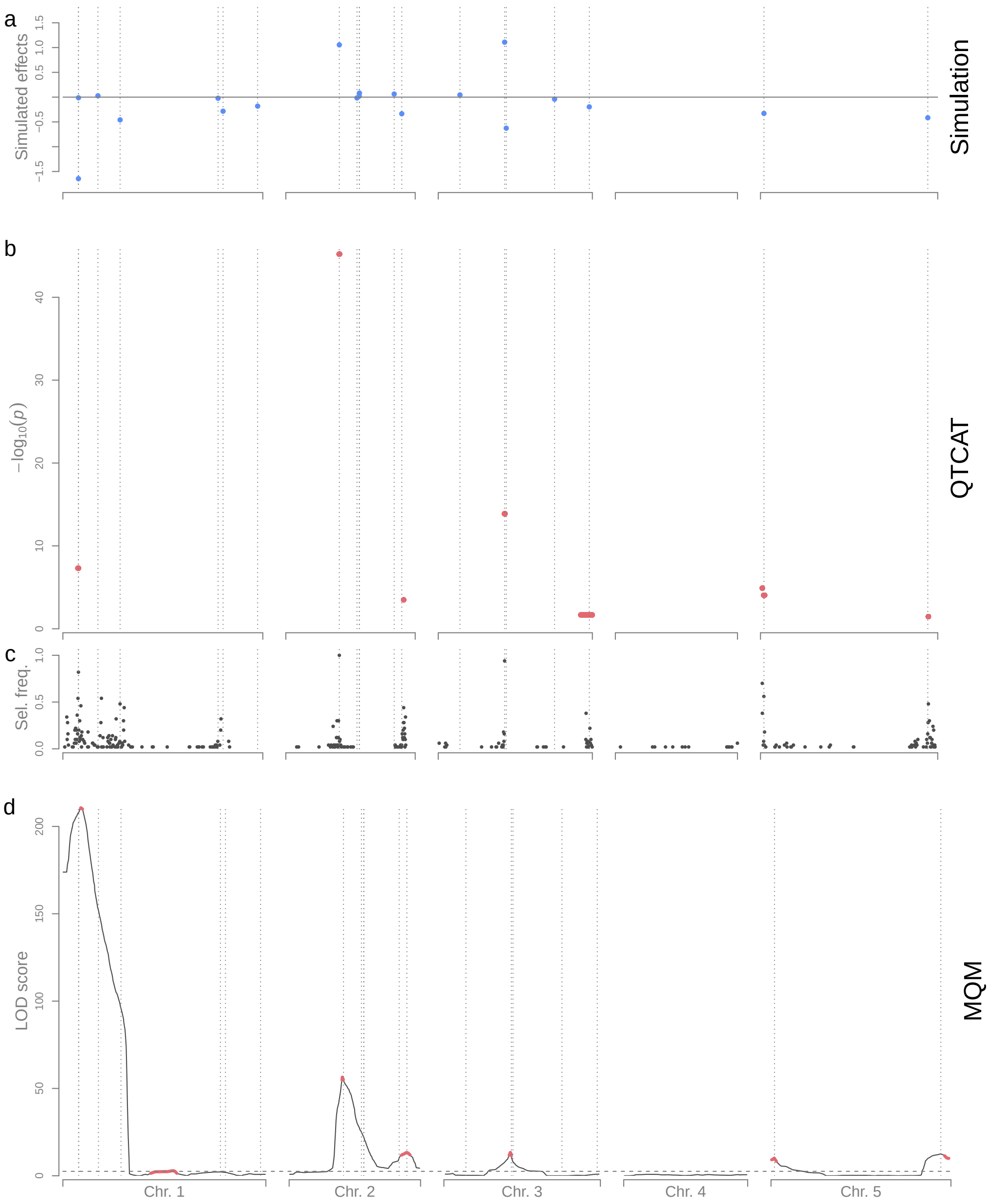

Supplementary Figure 328 Simulation of a GWA analysis based on a unstructured population with a heritability of 0.7 (run 28). (**a**) Simulated of 20 effects randomly drawn from a Gamma distribution and assigned them randomly to markers. Simulated effects randomly drawn from a Gamma distribution. We assigned effects to 20 markers. Markers with an effect are highlighted in (**b–d**) with dashed lines. (**b**) Significant QTCs found by QTCAT. (**c**) The selection frequency of the LASSO for each marker during the 50 iterations of QTCAT. (**d**) MQM LOD score plot, the horizontal dashed line is a simulation based perumtation test FDR. The red colored areas represent the LOD-intervals.

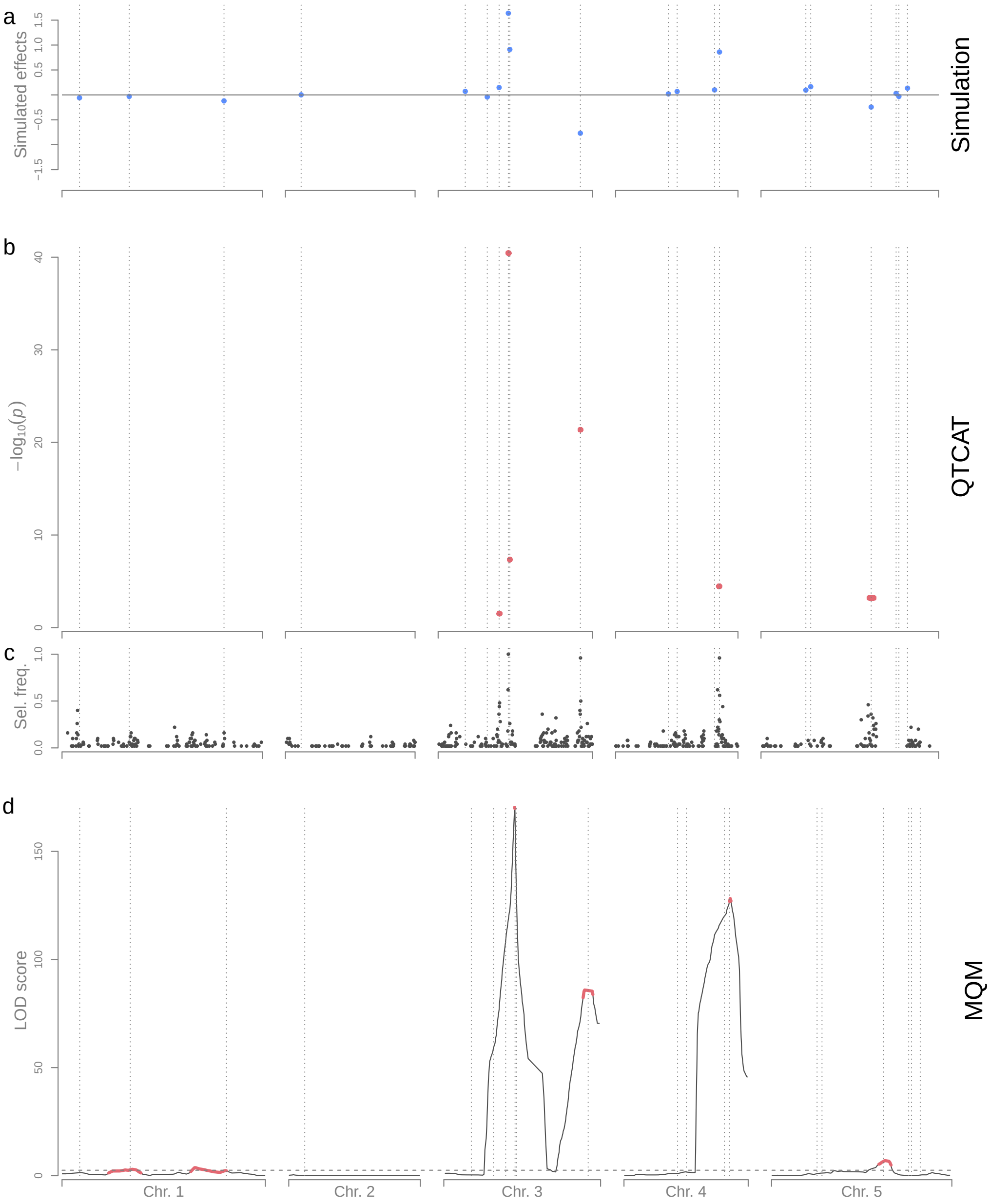

Supplementary Figure 329 Simulation of a GWA analysis based on a unstructured population with a heritability of 0.7 (run 29). (**a**) Simulated of 20 effects randomly drawn from a Gamma distribution and assigned them randomly to markers. Simulated effects randomly drawn from a Gamma distribution. We assigned effects to 20 markers. Markers with an effect are highlighted in (**b**–**d**) with dashed lines. (**b**) Significant QTCs found by QTCAT. (**c**) The selection frequency of the LASSO for each marker during the 50 iterations of QTCAT. (**d**) MQM LOD score plot, the horizontal dashed line is a simulation based perumtation test FDR. The red colored areas represent the LOD-intervals.

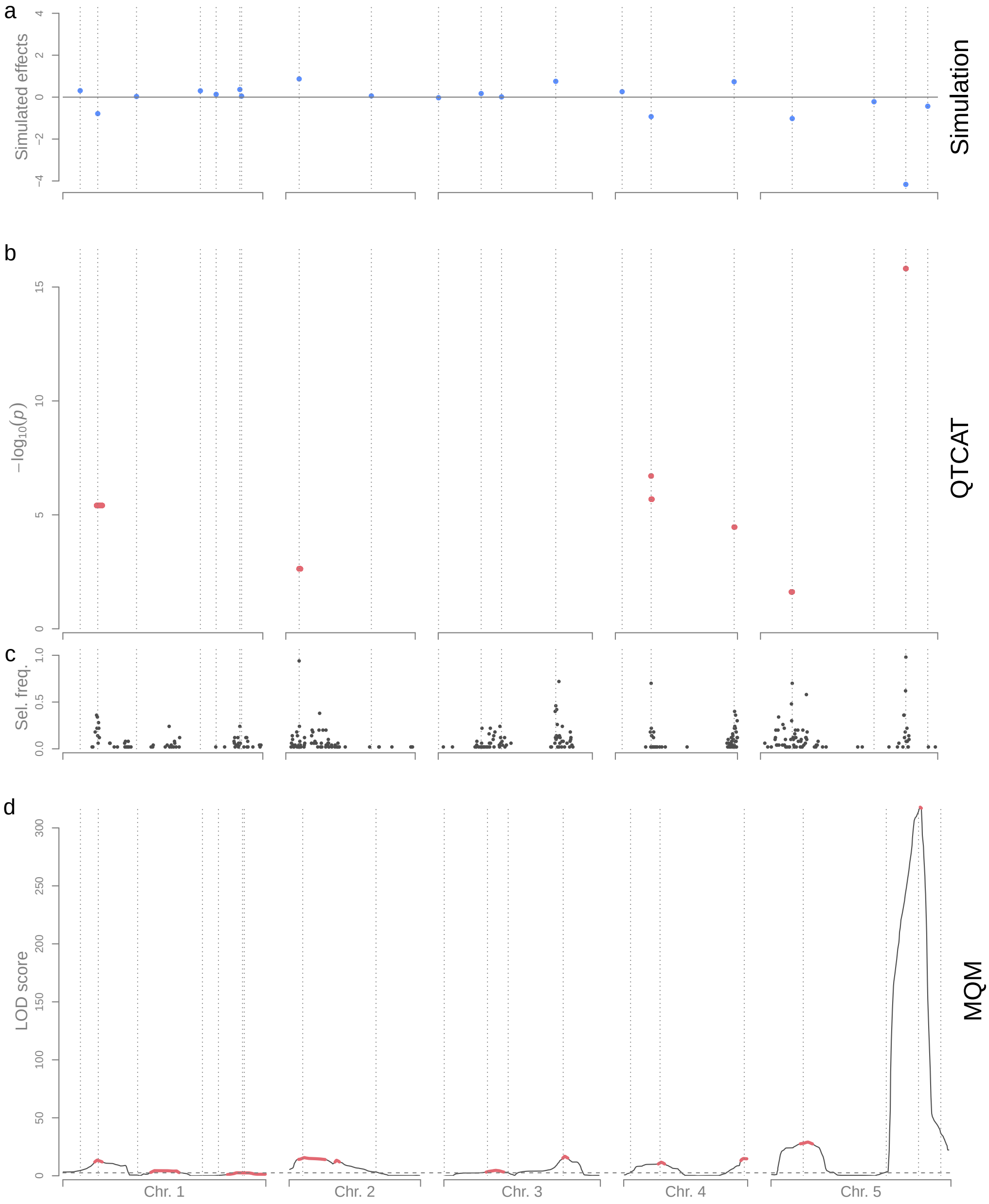

Supplementary Figure 330 Simulation of a GWA analysis based on a unstructured population with a heritability of 0.7 (run 30). (**a**) Simulated of 20 effects randomly drawn from a Gamma distribution and assigned them randomly to markers. Simulated effects randomly drawn from a Gamma distribution. We assigned effects to 20 markers. Markers with an effect are highlighted in (**b–d**) with dashed lines. (**b**) Significant QTCs found by QTCAT. (**c**) The selection frequency of the LASSO for each marker during the 50 iterations of QTCAT. (**d**) MQM LOD score plot, the horizontal dashed line is a simulation based perumtation test FDR. The red colored areas represent the LOD-intervals.

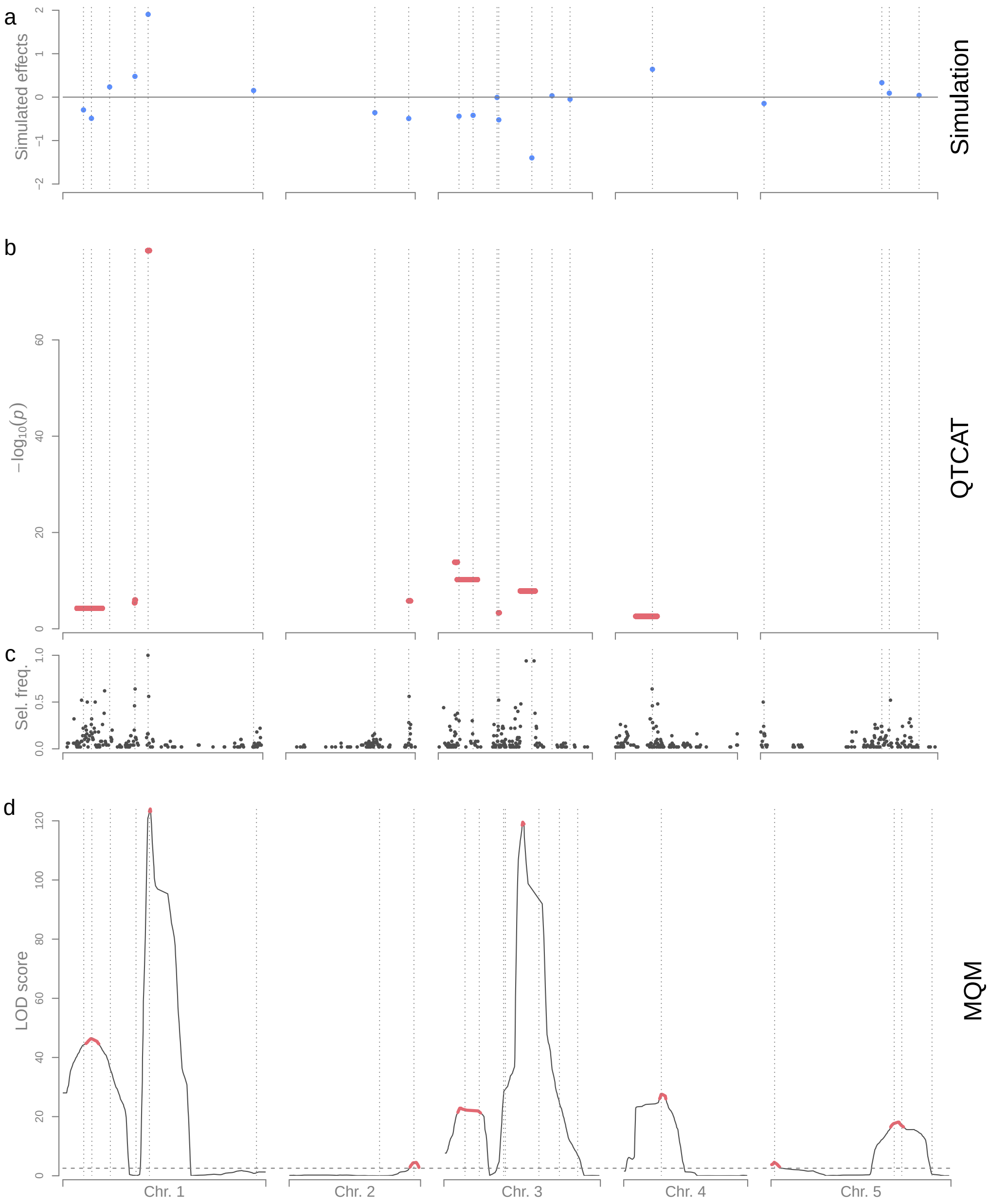

Supplementary Figure 331 Simulation of a GWA analysis based on a unstructured population with a heritability of 0.7 (run 31). (**a**) Simulated of 20 effects randomly drawn from a Gamma distribution and assigned them randomly to markers. Simulated effects randomly drawn from a Gamma distribution. We assigned effects to 20 markers. Markers with an effect are highlighted in (**b–d**) with dashed lines. (**b**) Significant QTCs found by QTCAT. (**c**) The selection frequency of the LASSO for each marker during the 50 iterations of QTCAT. (**d**) MQM LOD score plot, the horizontal dashed line is a simulation based perumtation test FDR. The red colored areas represent the LOD-intervals.

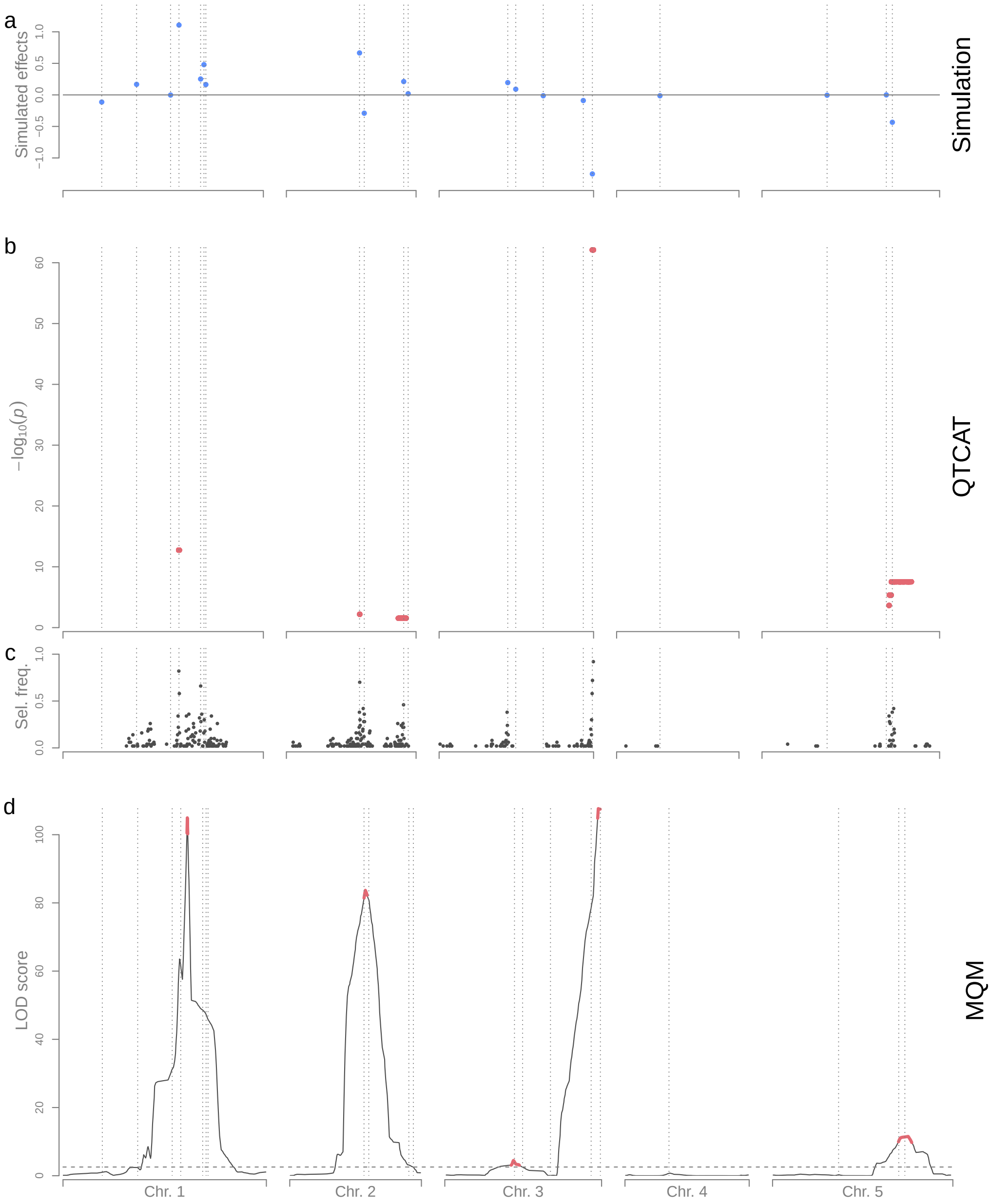

Supplementary Figure 332 Simulation of a GWA analysis based on a unstructured population with a heritability of 0.7 (run 32). (**a**) Simulated of 20 effects randomly drawn from a Gamma distribution and assigned them randomly to markers. Simulated effects randomly drawn from a Gamma distribution. We assigned effects to 20 markers. Markers with an effect are highlighted in (**b–d**) with dashed lines. (**b**) Significant QTCs found by QTCAT. (**c**) The selection frequency of the LASSO for each marker during the 50 iterations of QTCAT. (**d**) MQM LOD score plot, the horizontal dashed line is a simulation based perumtation test FDR. The red colored areas represent the LOD-intervals.

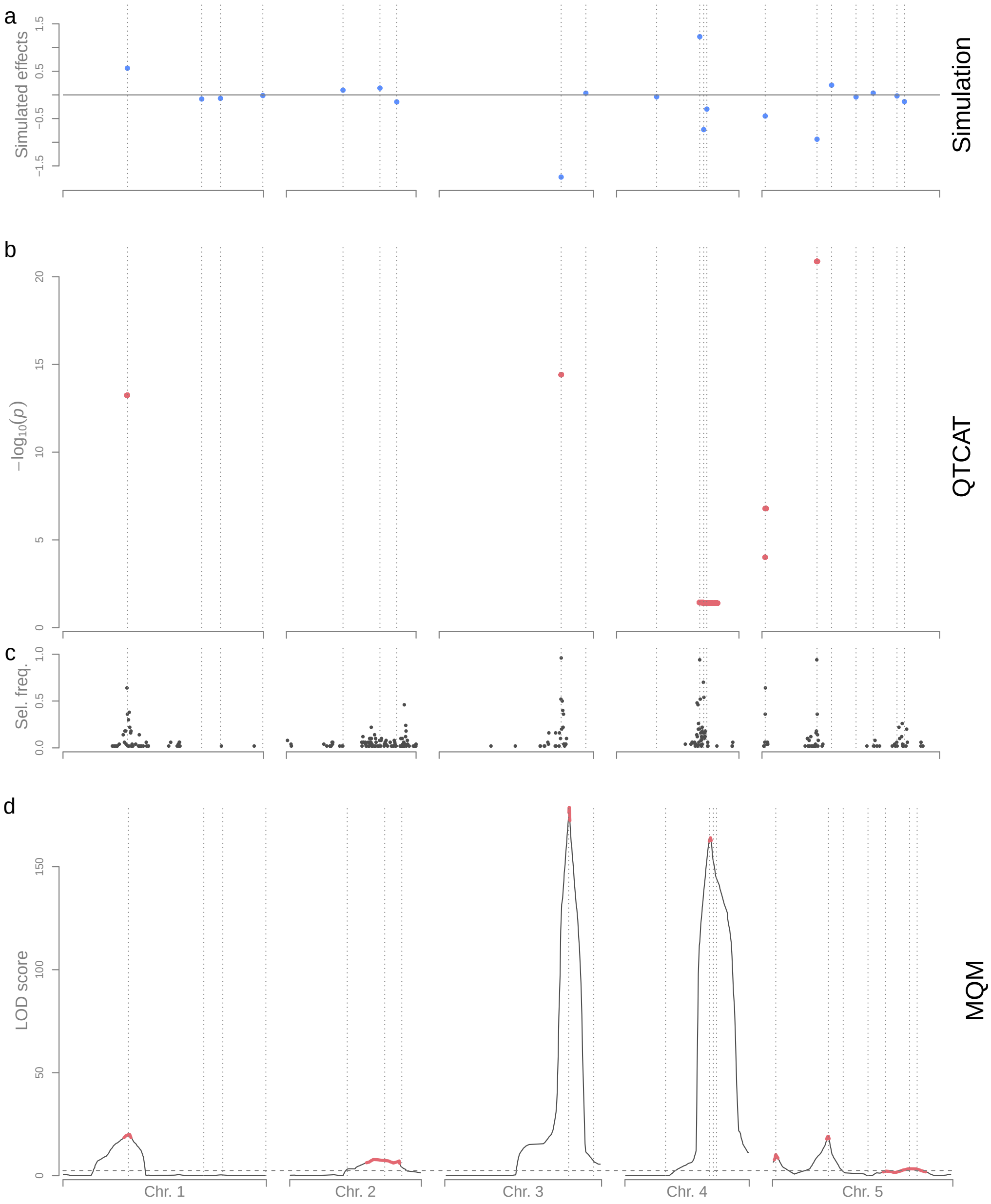

Supplementary Figure 333 Simulation of a GWA analysis based on a unstructured population with a heritability of 0.7 (run 33). (**a**) Simulated of 20 effects randomly drawn from a Gamma distribution and assigned them randomly to markers. Simulated effects randomly drawn from a Gamma distribution. We assigned effects to 20 markers. Markers with an effect are highlighted in (**b**–**d**) with dashed lines. (**b**) Significant QTCs found by QTCAT. (**c**) The selection frequency of the LASSO for each marker during the 50 iterations of QTCAT. (**d**) MQM LOD score plot, the horizontal dashed line is a simulation based perumtation test FDR. The red colored areas represent the LOD-intervals.

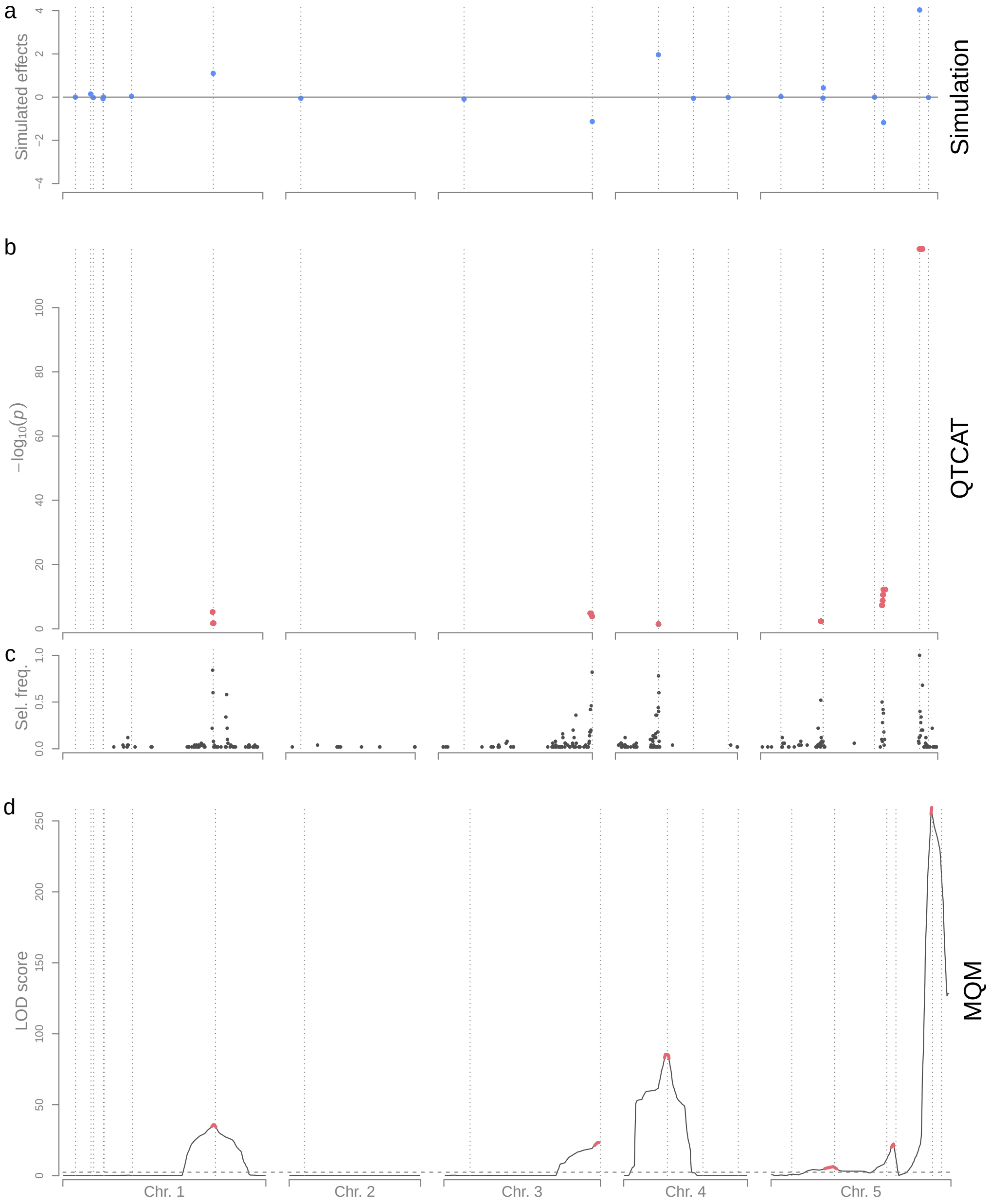

Supplementary Figure 334 Simulation of a GWA analysis based on a unstructured population with a heritability of 0.7 (run 34). (**a**) Simulated of 20 effects randomly drawn from a Gamma distribution and assigned them randomly to markers. Simulated effects randomly drawn from a Gamma distribution. We assigned effects to 20 markers. Markers with an effect are highlighted in (**b–d**) with dashed lines. (**b**) Significant QTCs found by QTCAT. (**c**) The selection frequency of the LASSO for each marker during the 50 iterations of QTCAT. (**d**) MQM LOD score plot, the horizontal dashed line is a simulation based perumtation test FDR. The red colored areas represent the LOD-intervals.

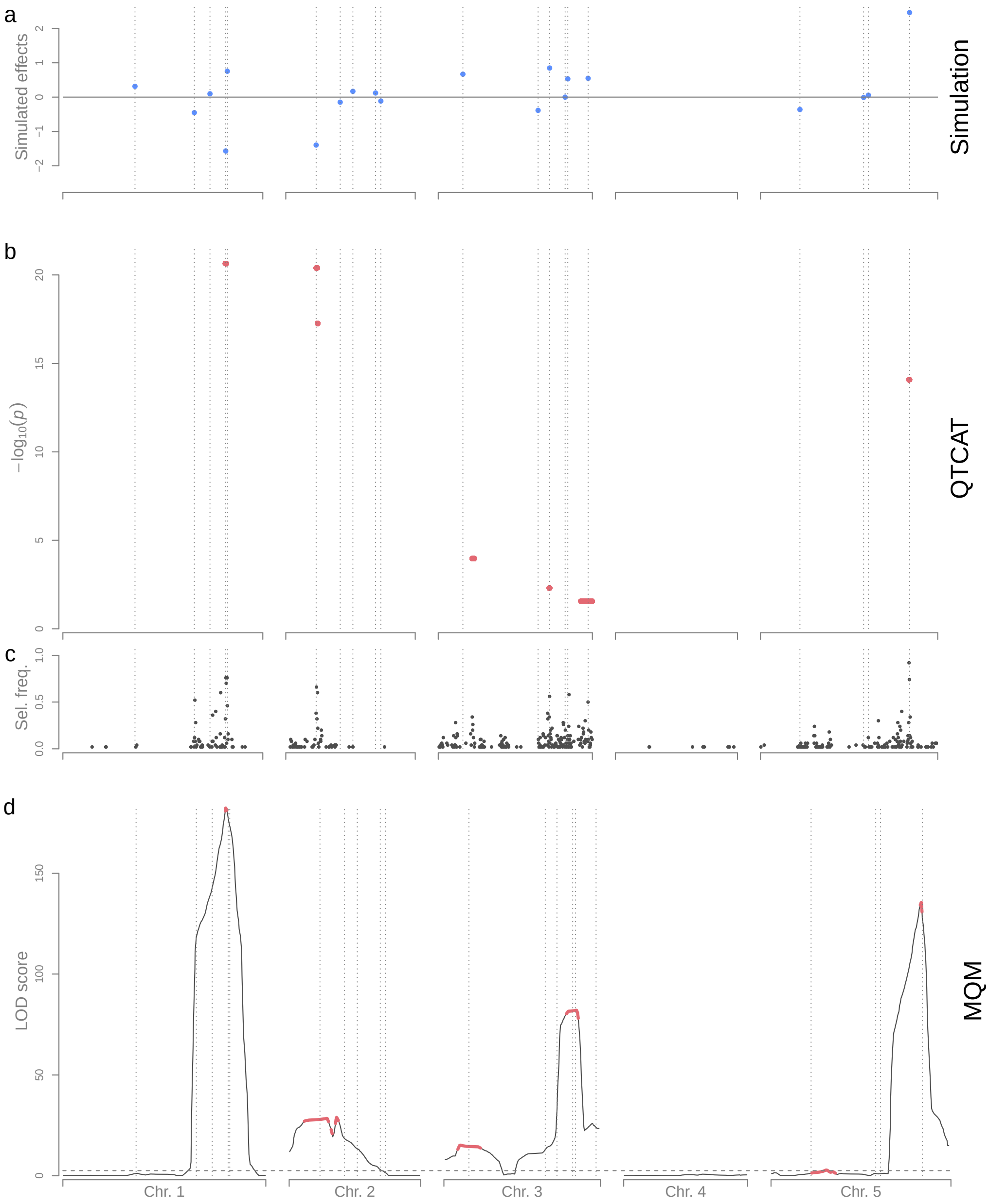

Supplementary Figure 335 Simulation of a GWA analysis based on a unstructured population with a heritability of 0.7 (run 35). (**a**) Simulated of 20 effects randomly drawn from a Gamma distribution and assigned them randomly to markers. Simulated effects randomly drawn from a Gamma distribution. We assigned effects to 20 markers. Markers with an effect are highlighted in (**b–d**) with dashed lines. (**b**) Significant QTCs found by QTCAT. (**c**) The selection frequency of the LASSO for each marker during the 50 iterations of QTCAT. (**d**) MQM LOD score plot, the horizontal dashed line is a simulation based perumtation test FDR. The red colored areas represent the LOD-intervals.

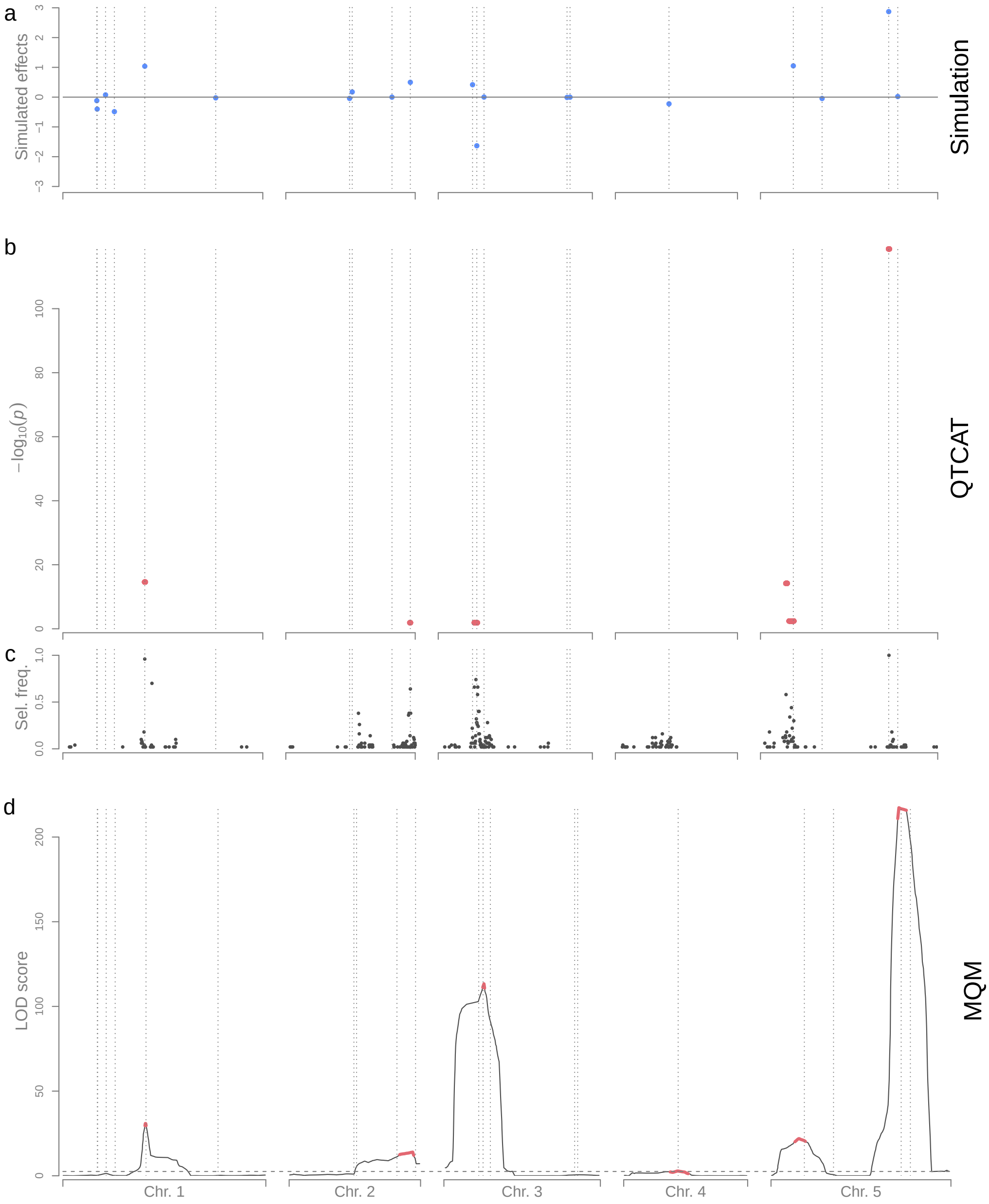

Supplementary Figure 336 Simulation of a GWA analysis based on a unstructured population with a heritability of 0.7 (run 36). (**a**) Simulated of 20 effects randomly drawn from a Gamma distribution and assigned them randomly to markers. Simulated effects randomly drawn from a Gamma distribution. We assigned effects to 20 markers. Markers with an effect are highlighted in (**b–d**) with dashed lines. (**b**) Significant QTCs found by QTCAT. (**c**) The selection frequency of the LASSO for each marker during the 50 iterations of QTCAT. (**d**) MQM LOD score plot, the horizontal dashed line is a simulation based perumtation test FDR. The red colored areas represent the LOD-intervals.

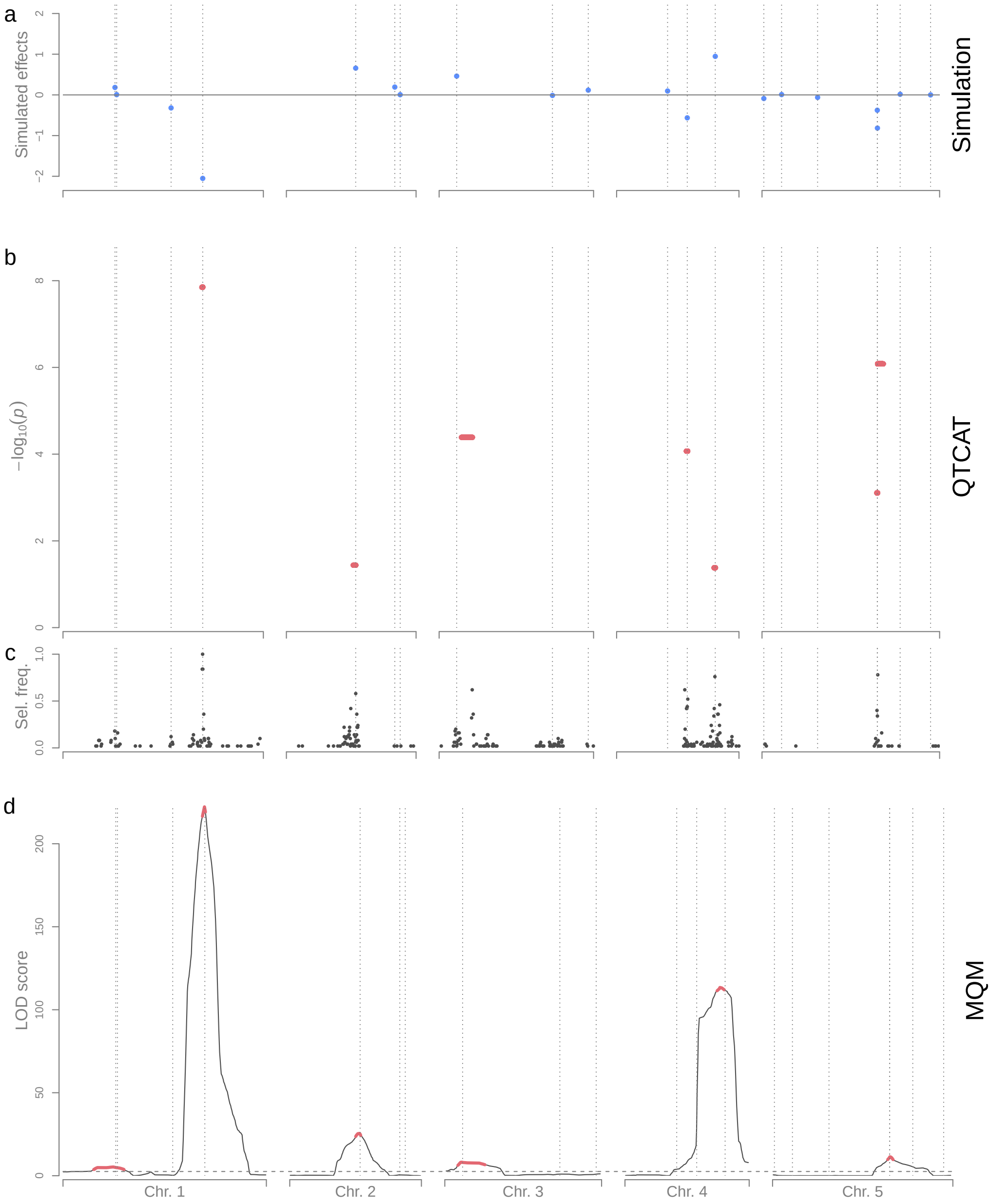

Supplementary Figure 337 Simulation of a GWA analysis based on a unstructured population with a heritability of 0.7 (run 37). (**a**) Simulated of 20 effects randomly drawn from a Gamma distribution and assigned them randomly to markers. Simulated effects randomly drawn from a Gamma distribution. We assigned effects to 20 markers. Markers with an effect are highlighted in (**b**–**d**) with dashed lines. (**b**) Significant QTCs found by QTCAT. (**c**) The selection frequency of the LASSO for each marker during the 50 iterations of QTCAT. (**d**) MQM LOD score plot, the horizontal dashed line is a simulation based perumtation test FDR. The red colored areas represent the LOD-intervals.

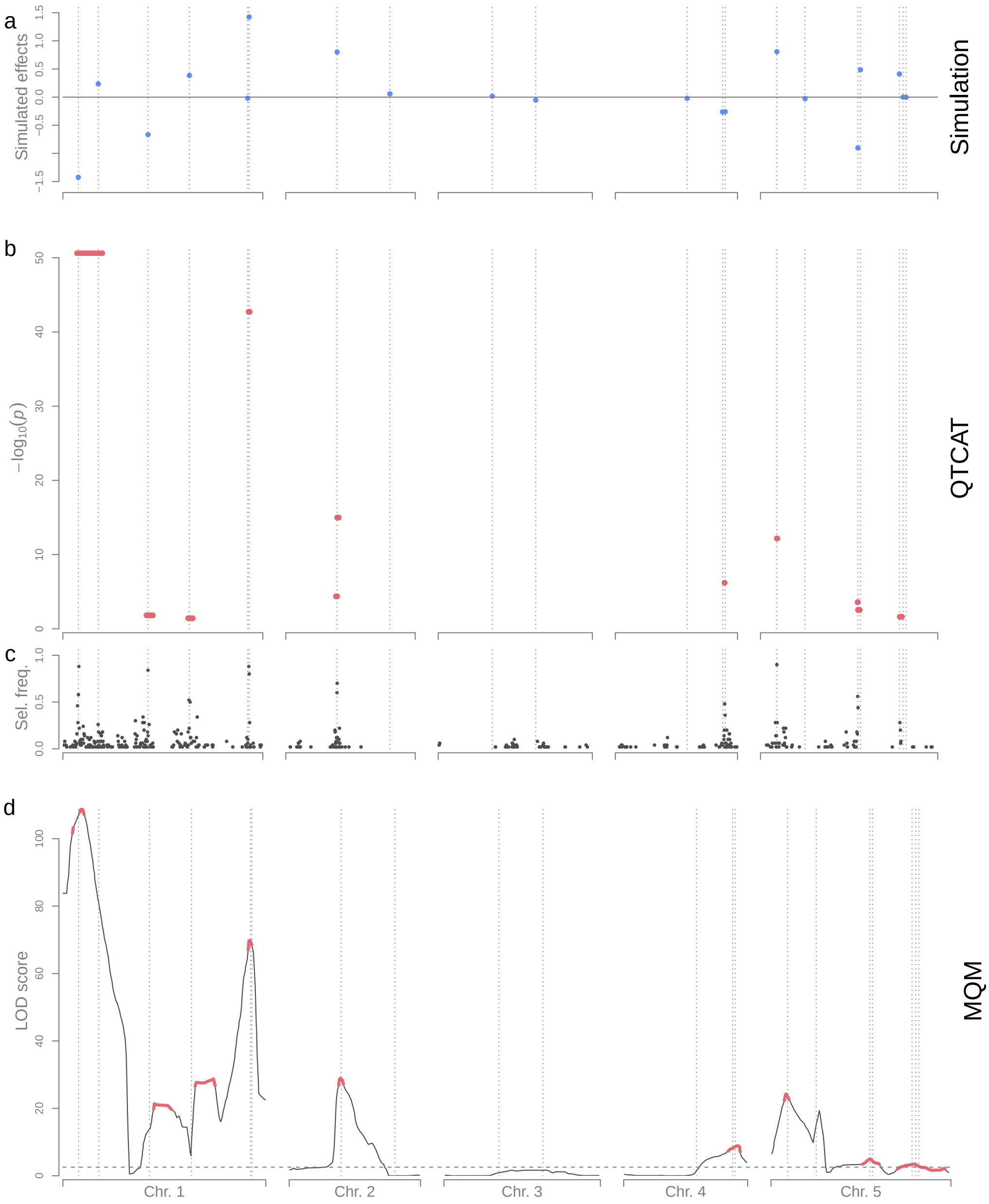

Supplementary Figure 338 Simulation of a GWA analysis based on a unstructured population with a heritability of 0.7 (run 38). (**a**) Simulated of 20 effects randomly drawn from a Gamma distribution and assigned them randomly to markers. Simulated effects randomly drawn from a Gamma distribution. We assigned effects to 20 markers. Markers with an effect are highlighted in (**b–d**) with dashed lines. (**b**) Significant QTCs found by QTCAT. (**c**) The selection frequency of the LASSO for each marker during the 50 iterations of QTCAT. (**d**) MQM LOD score plot, the horizontal dashed line is a simulation based perumtation test FDR. The red colored areas represent the LOD-intervals.

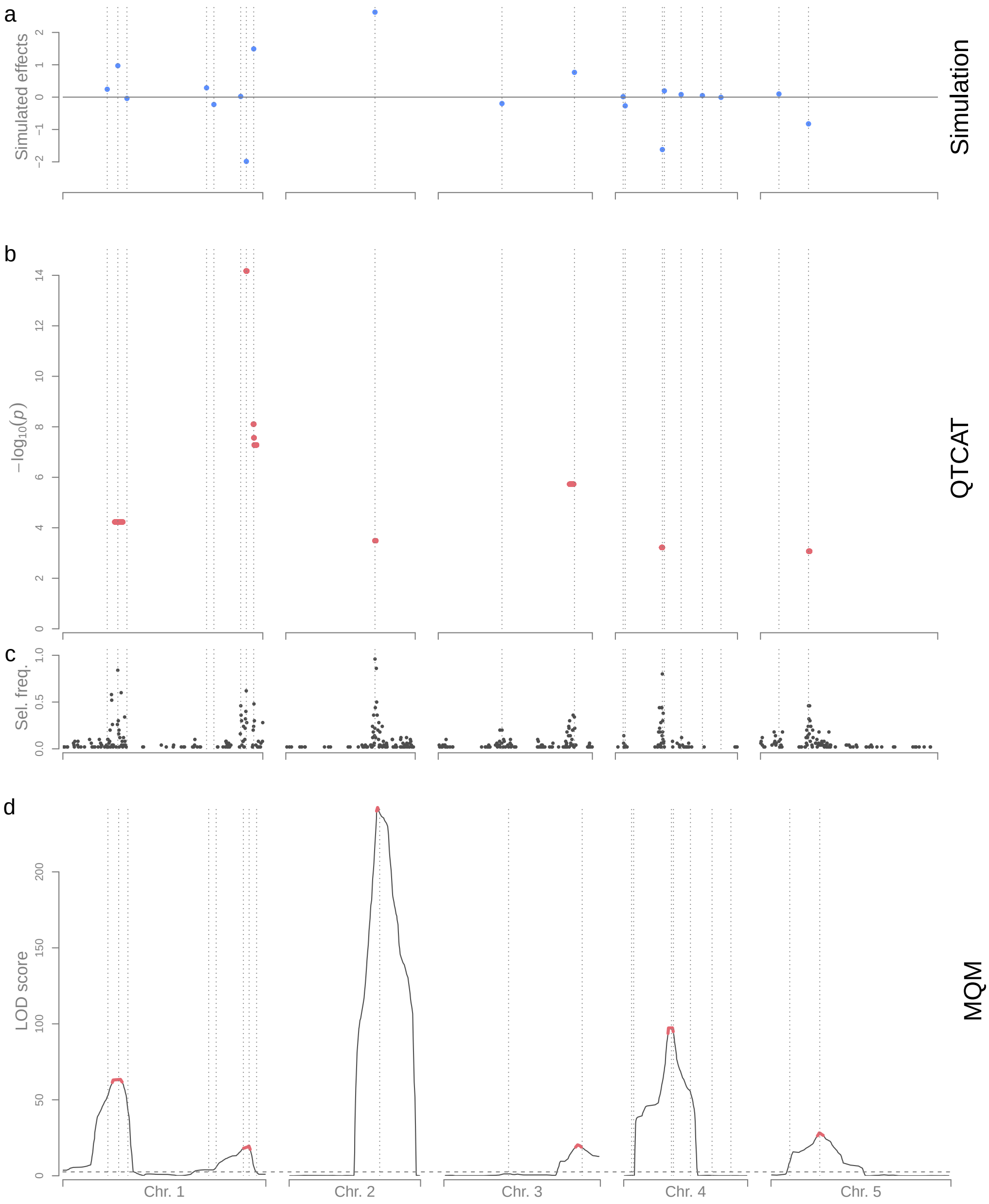

Supplementary Figure 339 Simulation of a GWA analysis based on a unstructured population with a heritability of 0.7 (run 39). (**a**) Simulated of 20 effects randomly drawn from a Gamma distribution and assigned them randomly to markers. Simulated effects randomly drawn from a Gamma distribution. We assigned effects to 20 markers. Markers with an effect are highlighted in (**b–d**) with dashed lines. (**b**) Significant QTCs found by QTCAT. (**c**) The selection frequency of the LASSO for each marker during the 50 iterations of QTCAT. (**d**) MQM LOD score plot, the horizontal dashed line is a simulation based perumtation test FDR. The red colored areas represent the LOD-intervals.

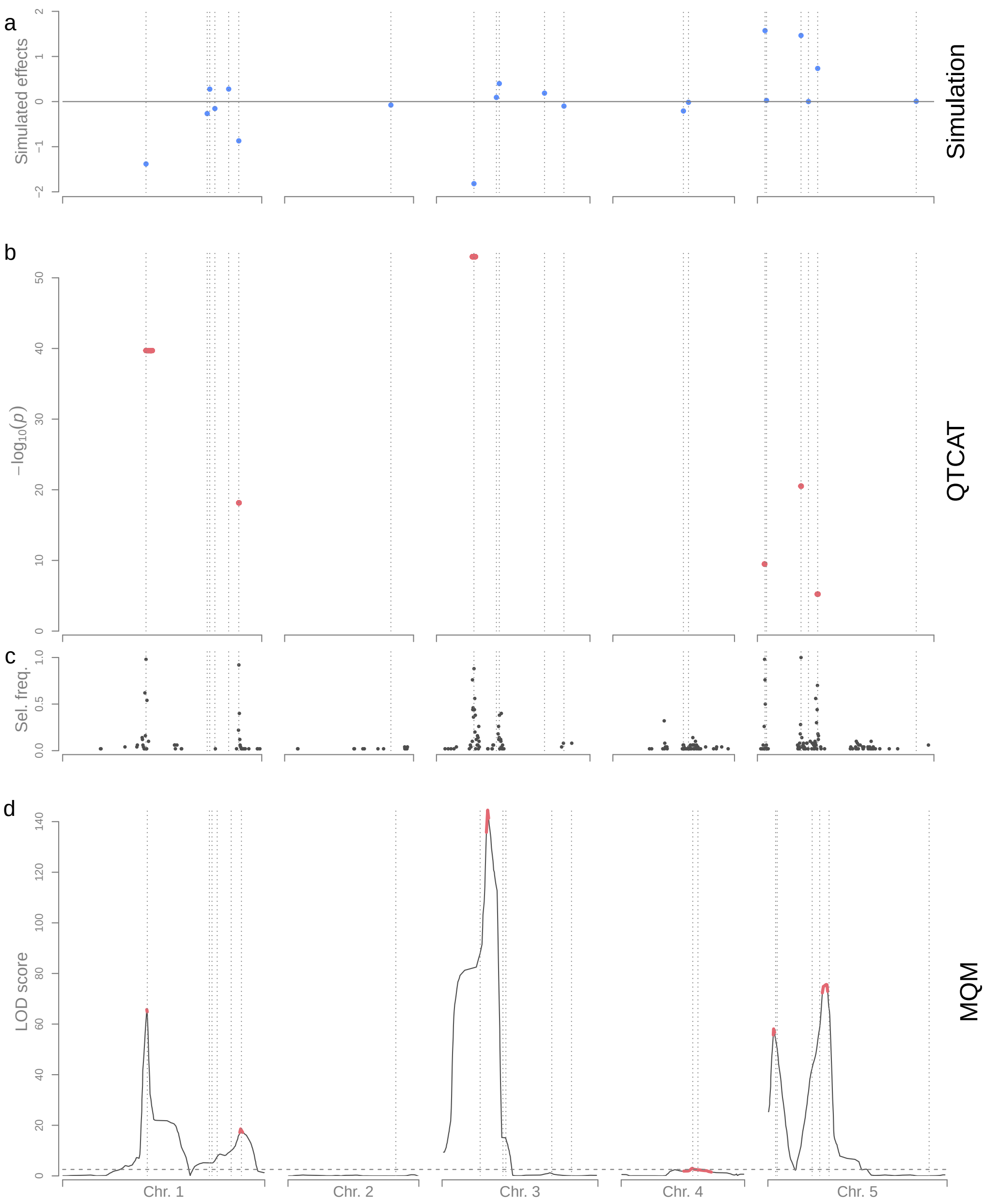

Supplementary Figure 340 Simulation of a GWA analysis based on a unstructured population with a heritability of 0.7 (run 40). (**a**) Simulated of 20 effects randomly drawn from a Gamma distribution and assigned them randomly to markers. Simulated effects randomly drawn from a Gamma distribution. We assigned effects to 20 markers. Markers with an effect are highlighted in (**b–d**) with dashed lines. (**b**) Significant QTCs found by QTCAT. (**c**) The selection frequency of the LASSO for each marker during the 50 iterations of QTCAT. (**d**) MQM LOD score plot, the horizontal dashed line is a simulation based perumtation test FDR. The red colored areas represent the LOD-intervals.

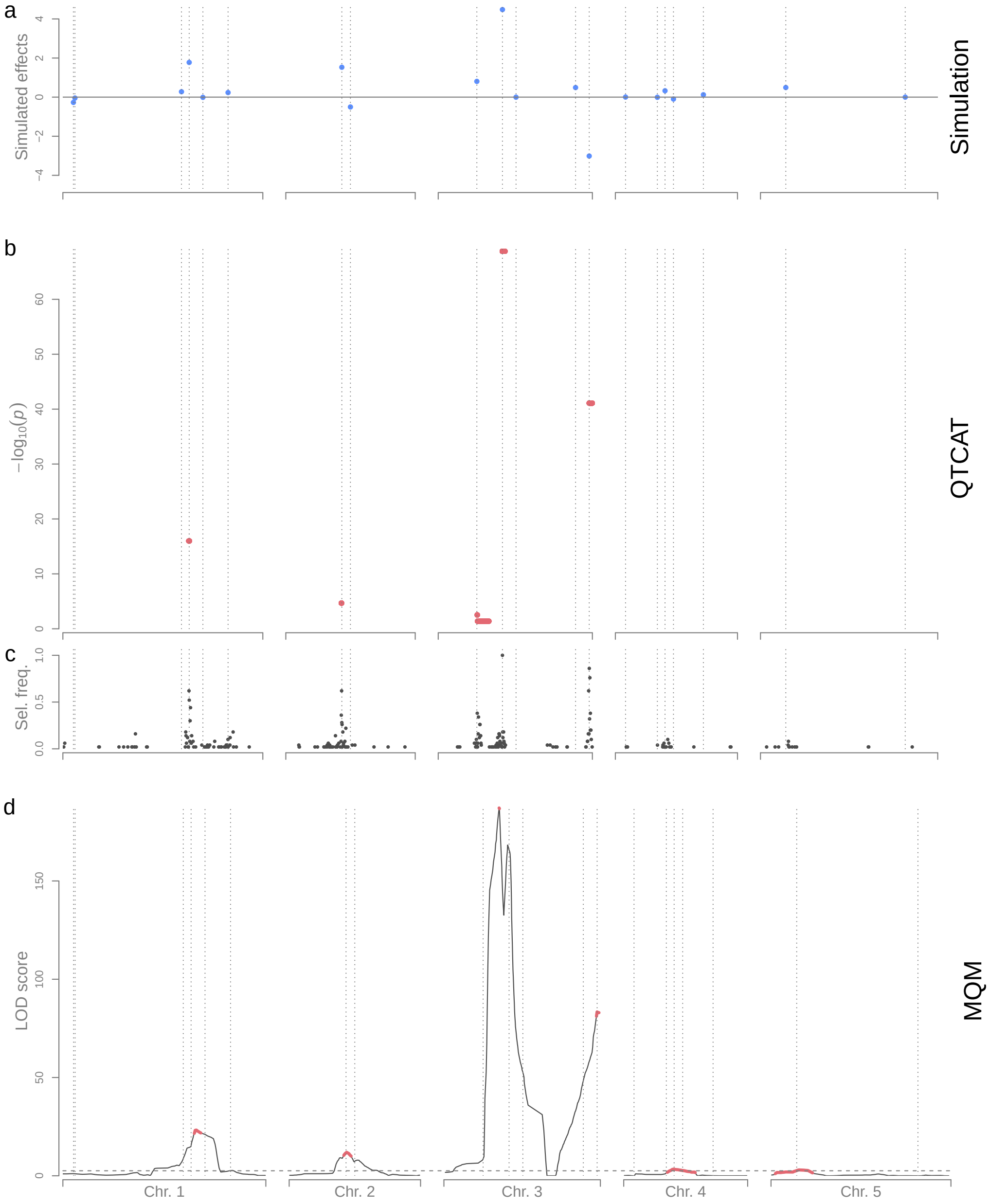

Supplementary Figure 341 Simulation of a GWA analysis based on a unstructured population with a heritability of 0.7 (run 41). (**a**) Simulated of 20 effects randomly drawn from a Gamma distribution and assigned them randomly to markers. Simulated effects randomly drawn from a Gamma distribution. We assigned effects to 20 markers. Markers with an effect are highlighted in (**b**–**d**) with dashed lines. (**b**) Significant QTCs found by QTCAT. (**c**) The selection frequency of the LASSO for each marker during the 50 iterations of QTCAT. (**d**) MQM LOD score plot, the horizontal dashed line is a simulation based perumtation test FDR. The red colored areas represent the LOD-intervals.

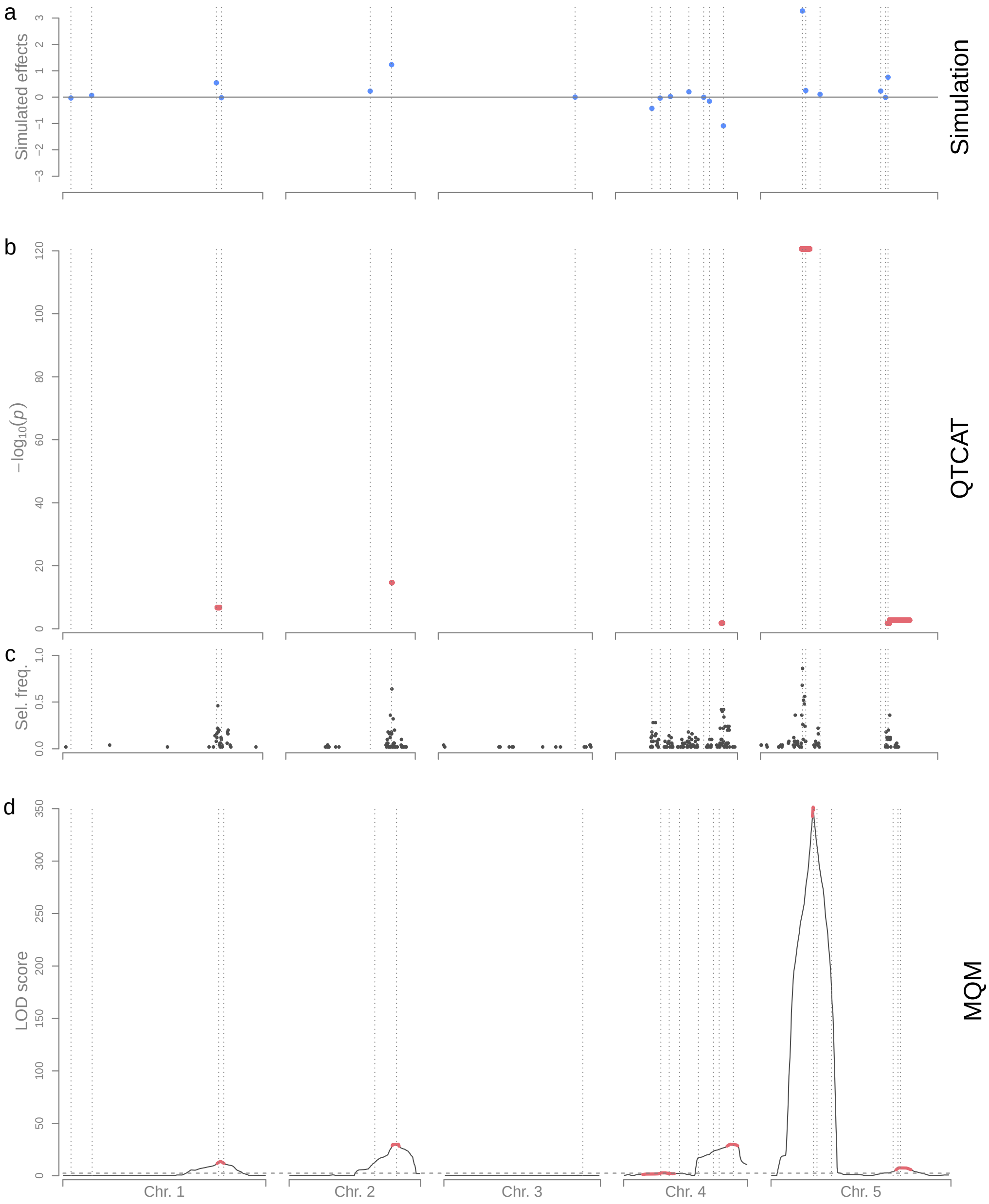

Supplementary Figure 342 Simulation of a GWA analysis based on a unstructured population with a heritability of 0.7 (run 42). (**a**) Simulated of 20 effects randomly drawn from a Gamma distribution and assigned them randomly to markers. Simulated effects randomly drawn from a Gamma distribution. We assigned effects to 20 markers. Markers with an effect are highlighted in (**b–d**) with dashed lines. (**b**) Significant QTCs found by QTCAT. (**c**) The selection frequency of the LASSO for each marker during the 50 iterations of QTCAT. (**d**) MQM LOD score plot, the horizontal dashed line is a simulation based perumtation test FDR. The red colored areas represent the LOD-intervals.

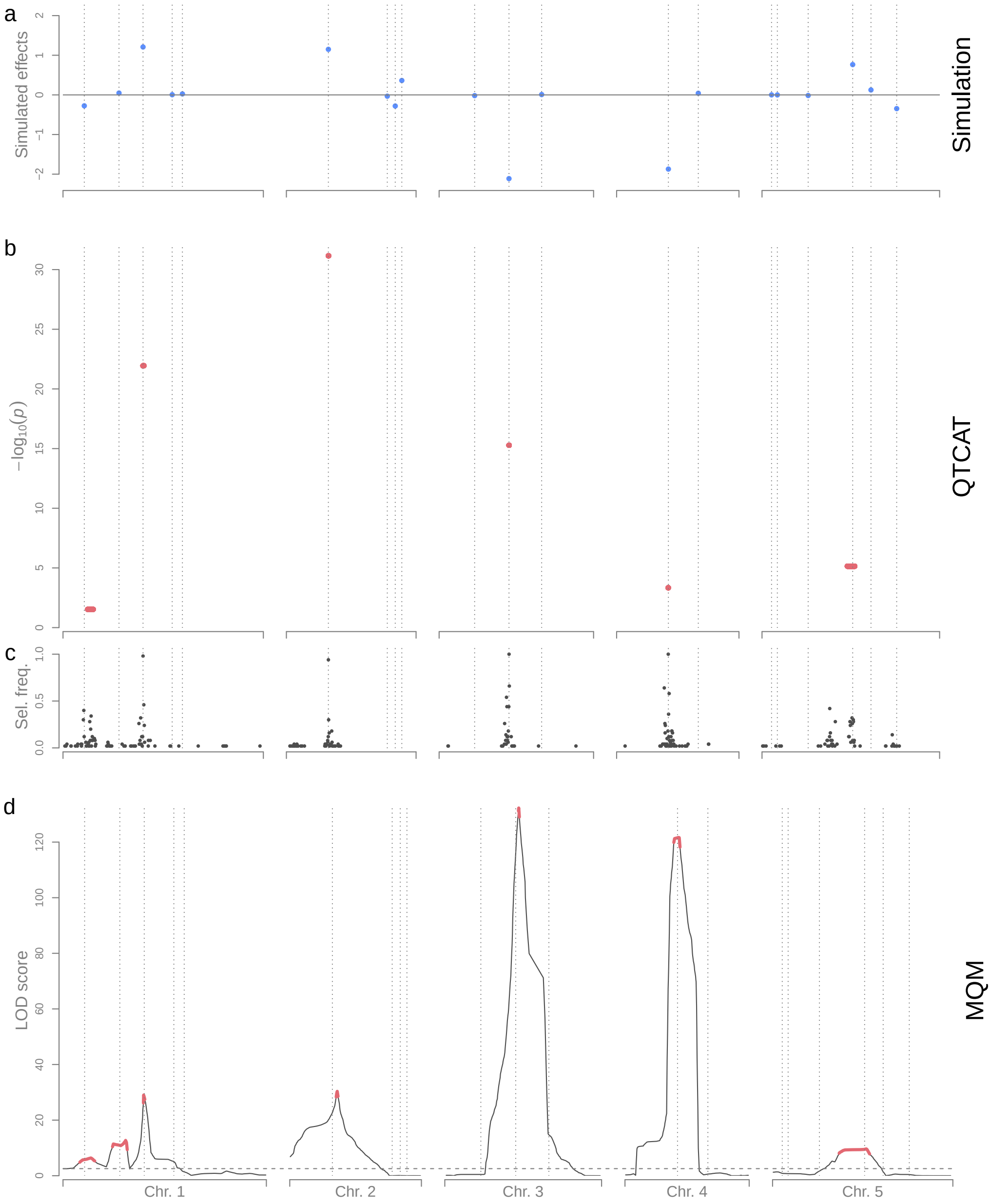

Supplementary Figure 343 Simulation of a GWA analysis based on a unstructured population with a heritability of 0.7 (run 43). (**a**) Simulated of 20 effects randomly drawn from a Gamma distribution and assigned them randomly to markers. Simulated effects randomly drawn from a Gamma distribution. We assigned effects to 20 markers. Markers with an effect are highlighted in (**b**–**d**) with dashed lines. (**b**) Significant QTCs found by QTCAT. (**c**) The selection frequency of the LASSO for each marker during the 50 iterations of QTCAT. (**d**) MQM LOD score plot, the horizontal dashed line is a simulation based perumtation test FDR. The red colored areas represent the LOD-intervals.

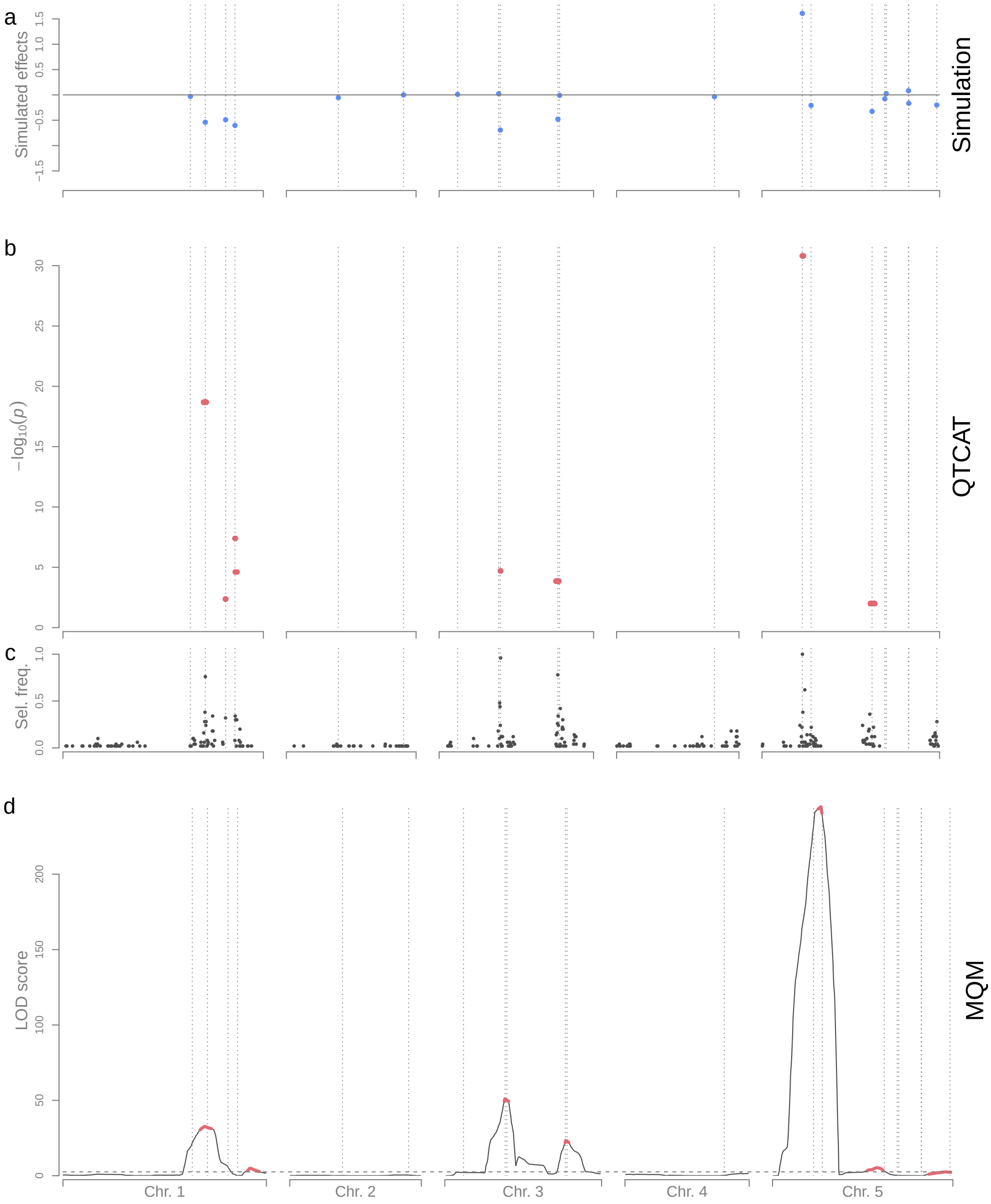

Supplementary Figure 344 Simulation of a GWA analysis based on a unstructured population with a heritability of 0.7 (run 44). (**a**) Simulated of 20 effects randomly drawn from a Gamma distribution and assigned them randomly to markers. Simulated effects randomly drawn from a Gamma distribution. We assigned effects to 20 markers. Markers with an effect are highlighted in (**b–d**) with dashed lines. (**b**) Significant QTCs found by QTCAT. (**c**) The selection frequency of the LASSO for each marker during the 50 iterations of QTCAT. (**d**) MQM LOD score plot, the horizontal dashed line is a simulation based perumtation test FDR. The red colored areas represent the LOD-intervals.

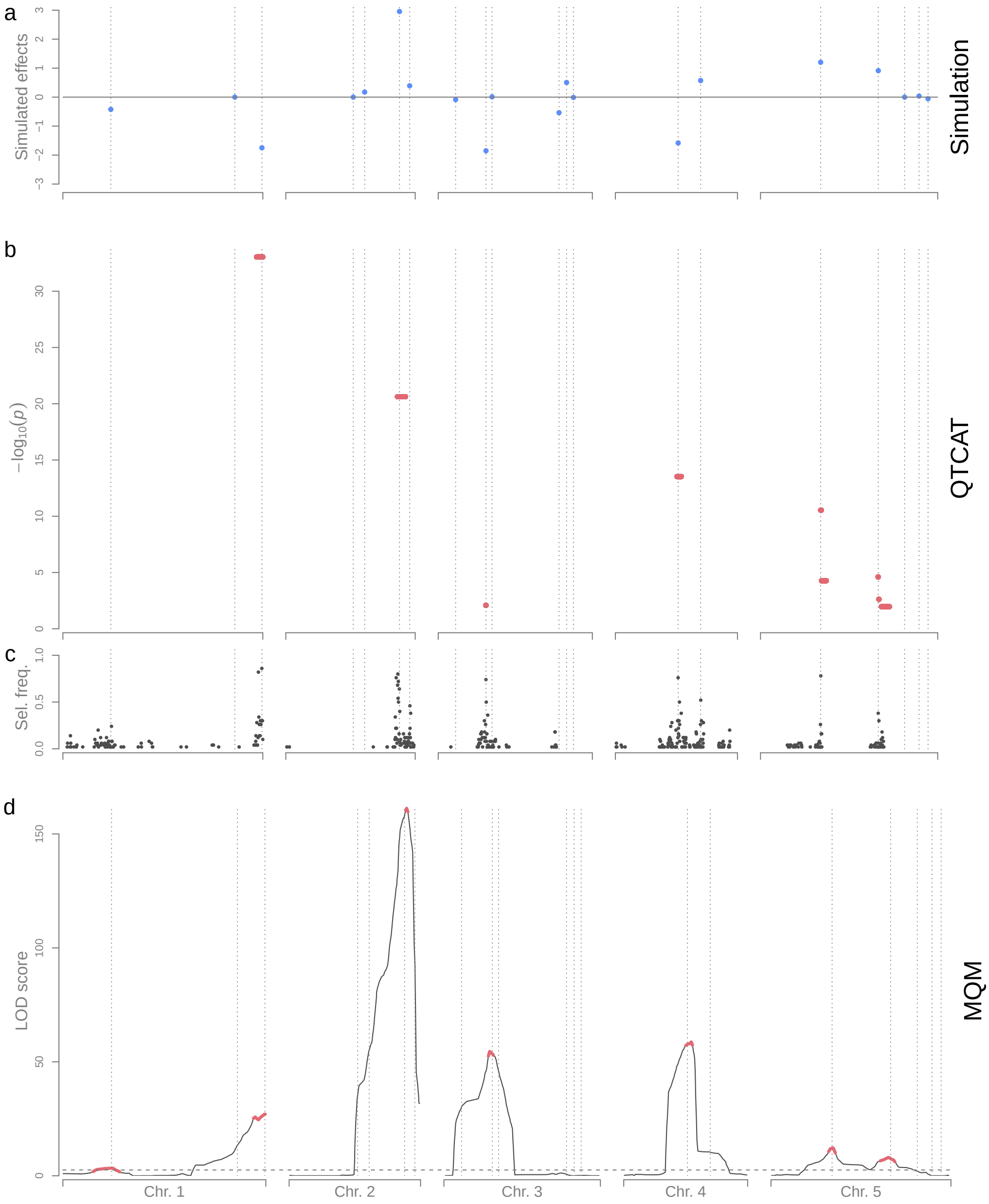

Supplementary Figure 345 Simulation of a GWA analysis based on a unstructured population with a heritability of 0.7 (run 45). (**a**) Simulated of 20 effects randomly drawn from a Gamma distribution and assigned them randomly to markers. Simulated effects randomly drawn from a Gamma distribution. We assigned effects to 20 markers. Markers with an effect are highlighted in (**b–d**) with dashed lines. (**b**) Significant QTCs found by QTCAT. (**c**) The selection frequency of the LASSO for each marker during the 50 iterations of QTCAT. (**d**) MQM LOD score plot, the horizontal dashed line is a simulation based perumtation test FDR. The red colored areas represent the LOD-intervals.

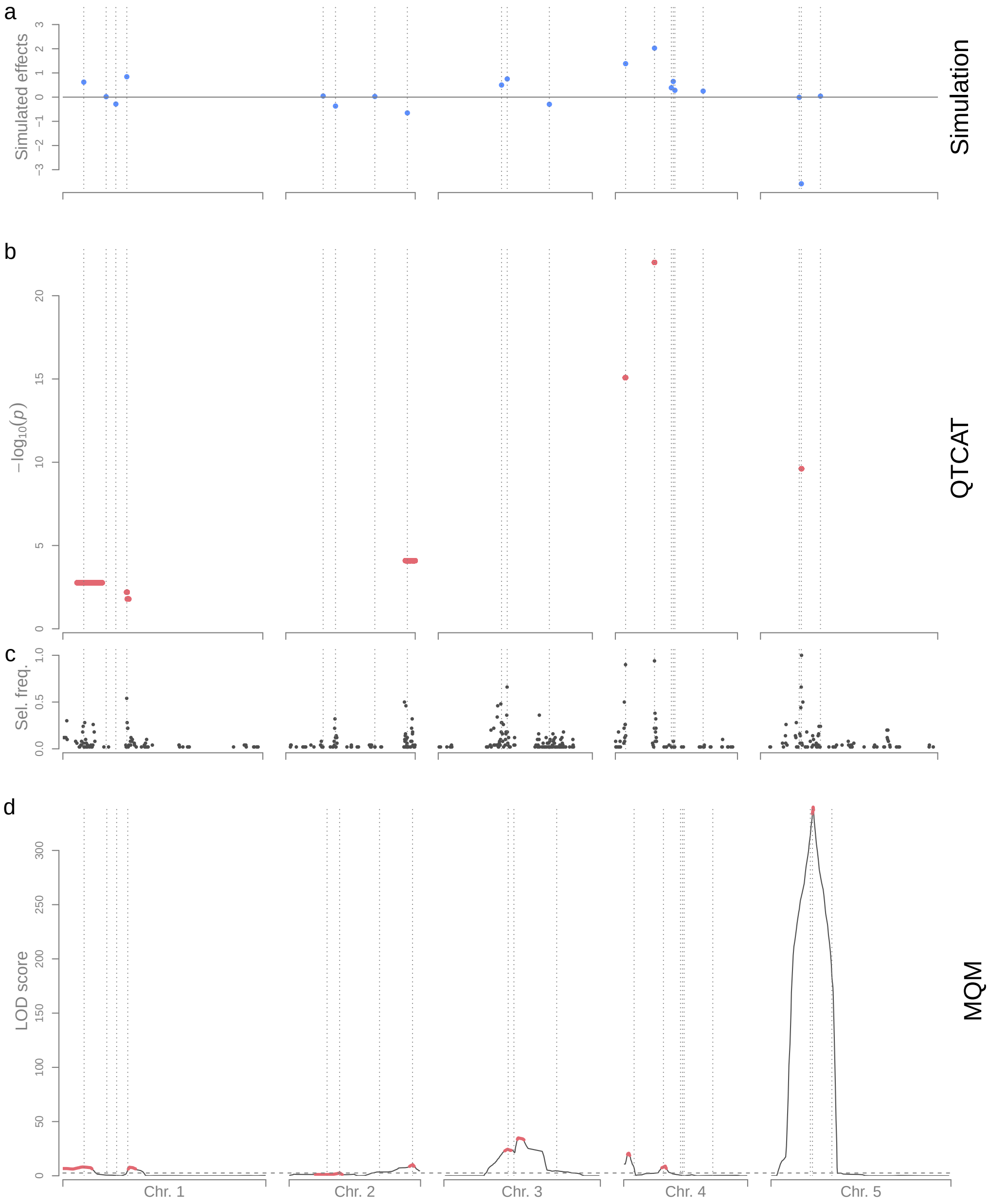

Supplementary Figure 346 Simulation of a GWA analysis based on a unstructured population with a heritability of 0.7 (run 46). (**a**) Simulated of 20 effects randomly drawn from a Gamma distribution and assigned them randomly to markers. Simulated effects randomly drawn from a Gamma distribution. We assigned effects to 20 markers. Markers with an effect are highlighted in (**b**–**d**) with dashed lines. (**b**) Significant QTCs found by QTCAT. (**c**) The selection frequency of the LASSO for each marker during the 50 iterations of QTCAT. (**d**) MQM LOD score plot, the horizontal dashed line is a simulation based perumtation test FDR. The red colored areas represent the LOD-intervals.

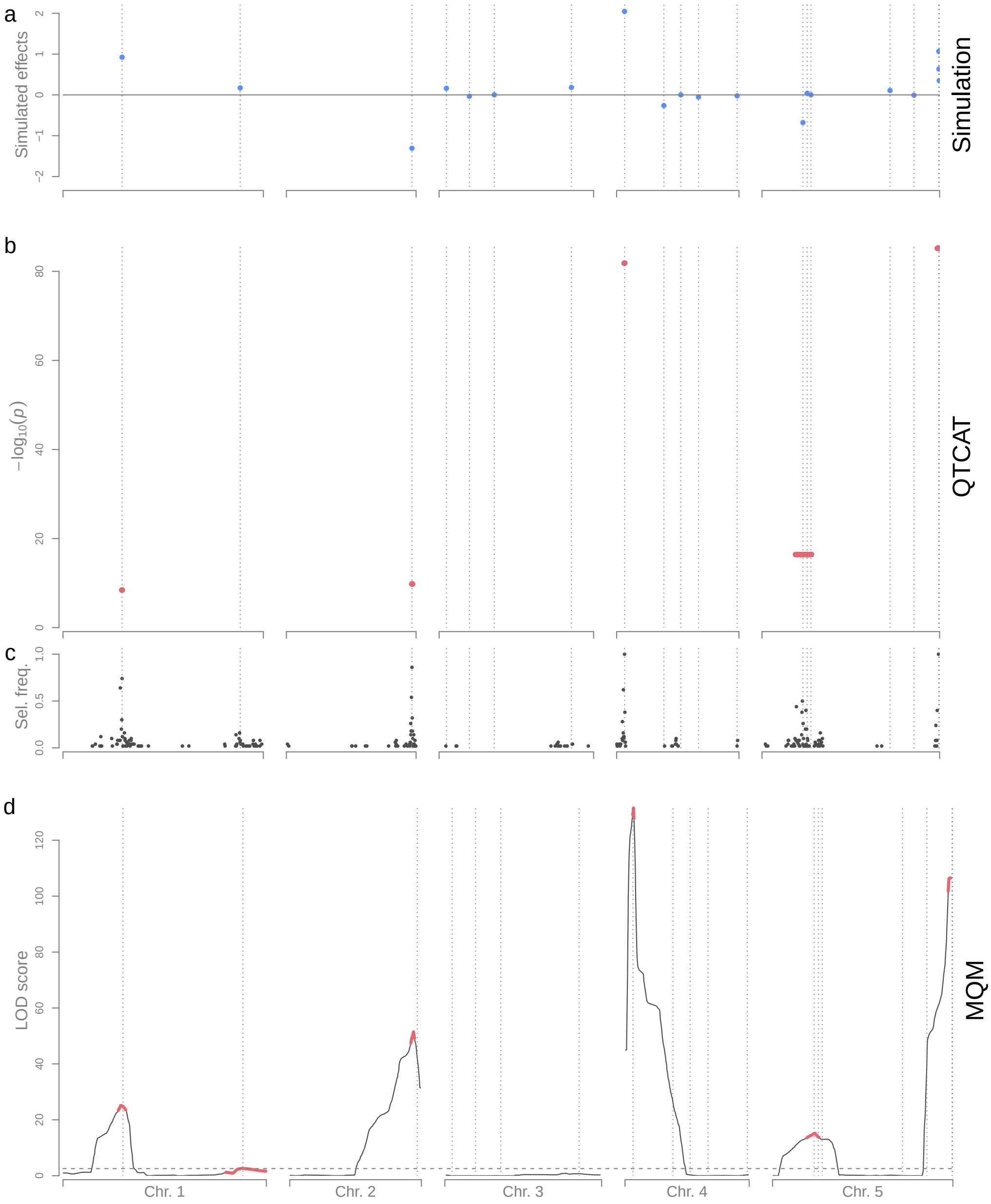

Supplementary Figure 347 Simulation of a GWA analysis based on a unstructured population with a heritability of 0.7 (run 47). (**a**) Simulated of 20 effects randomly drawn from a Gamma distribution and assigned them randomly to markers. Simulated effects randomly drawn from a Gamma distribution. We assigned effects to 20 markers. Markers with an effect are highlighted in (**b–d**) with dashed lines. (**b**) Significant QTCs found by QTCAT. (**c**) The selection frequency of the LASSO for each marker during the 50 iterations of QTCAT. (**d**) MQM LOD score plot, the horizontal dashed line is a simulation based perumtation test FDR. The red colored areas represent the LOD-intervals.

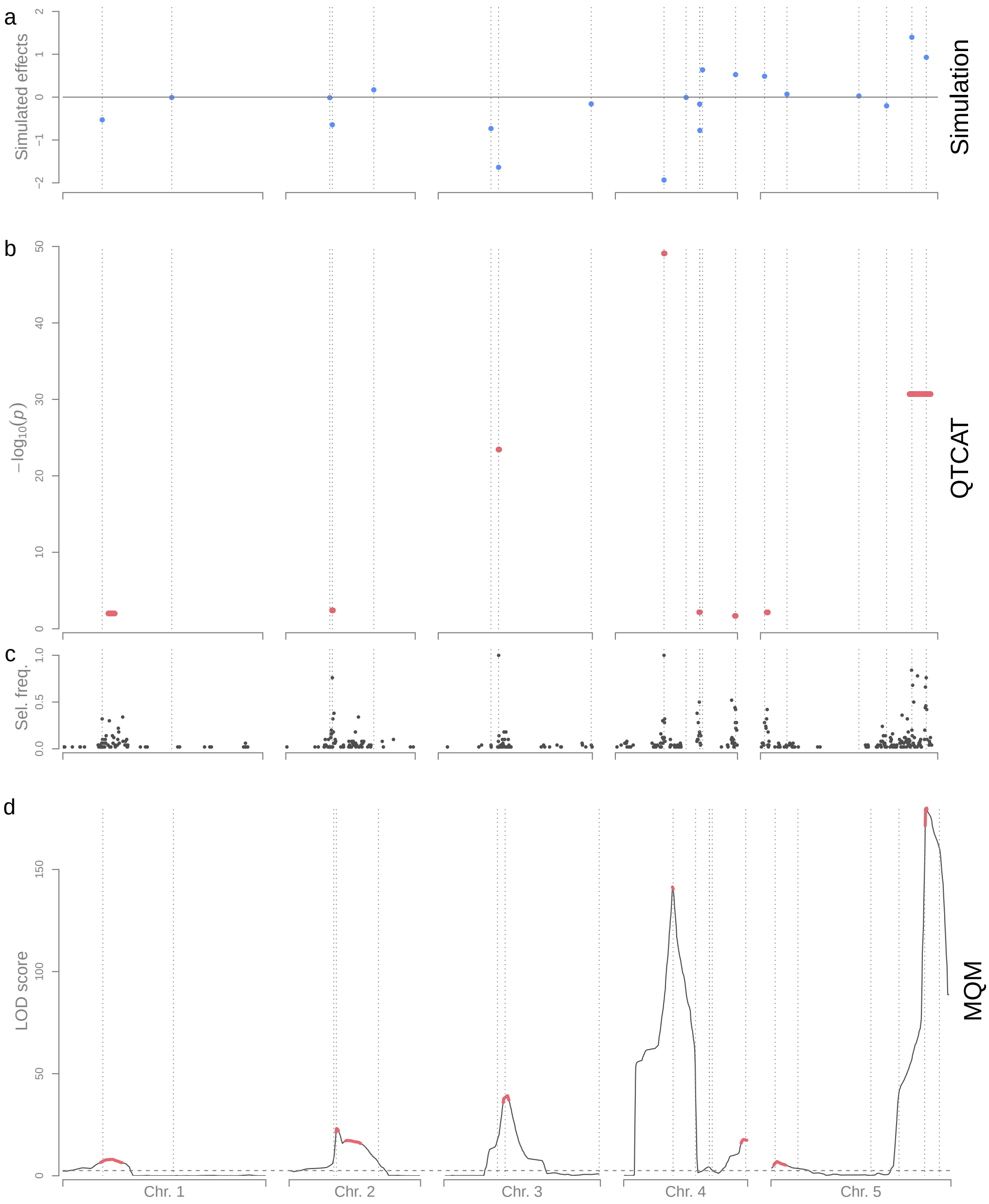

Supplementary Figure 348 Simulation of a GWA analysis based on a unstructured population with a heritability of 0.7 (run 48). (**a**) Simulated of 20 effects randomly drawn from a Gamma distribution and assigned them randomly to markers. Simulated effects randomly drawn from a Gamma distribution. We assigned effects to 20 markers. Markers with an effect are highlighted in (**b**–**d**) with dashed lines. (**b**) Significant QTCs found by QTCAT. (**c**) The selection frequency of the LASSO for each marker during the 50 iterations of QTCAT. (**d**) MQM LOD score plot, the horizontal dashed line is a simulation based perumtation test FDR. The red colored areas represent the LOD-intervals.

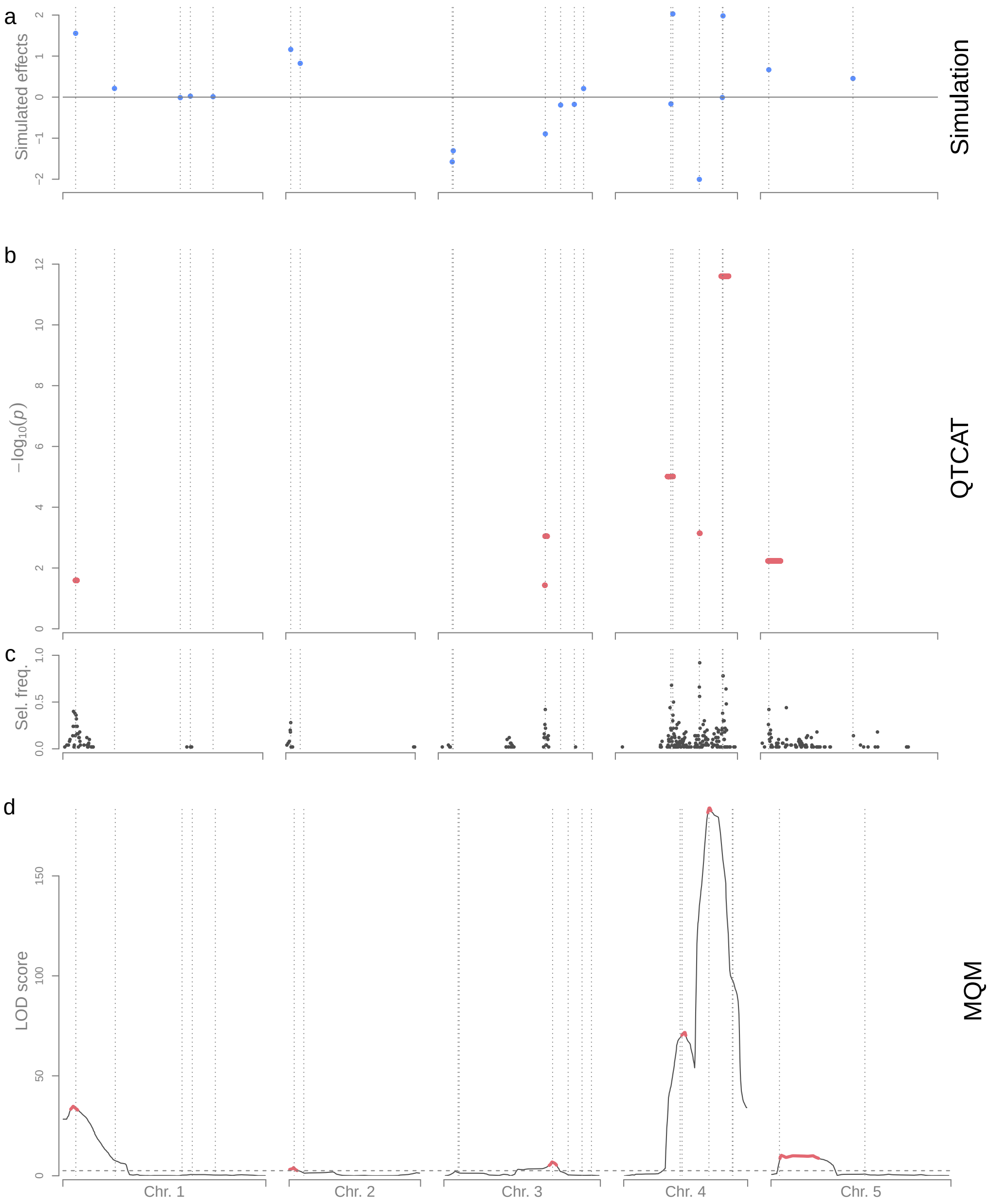

Supplementary Figure 349 Simulation of a GWA analysis based on a unstructured population with a heritability of 0.7 (run 49). (**a**) Simulated of 20 effects randomly drawn from a Gamma distribution and assigned them randomly to markers. Simulated effects randomly drawn from a Gamma distribution. We assigned effects to 20 markers. Markers with an effect are highlighted in (**b–d**) with dashed lines. (**b**) Significant QTCs found by QTCAT. (**c**) The selection frequency of the LASSO for each marker during the 50 iterations of QTCAT. (**d**) MQM LOD score plot, the horizontal dashed line is a simulation based perumtation test FDR. The red colored areas represent the LOD-intervals.

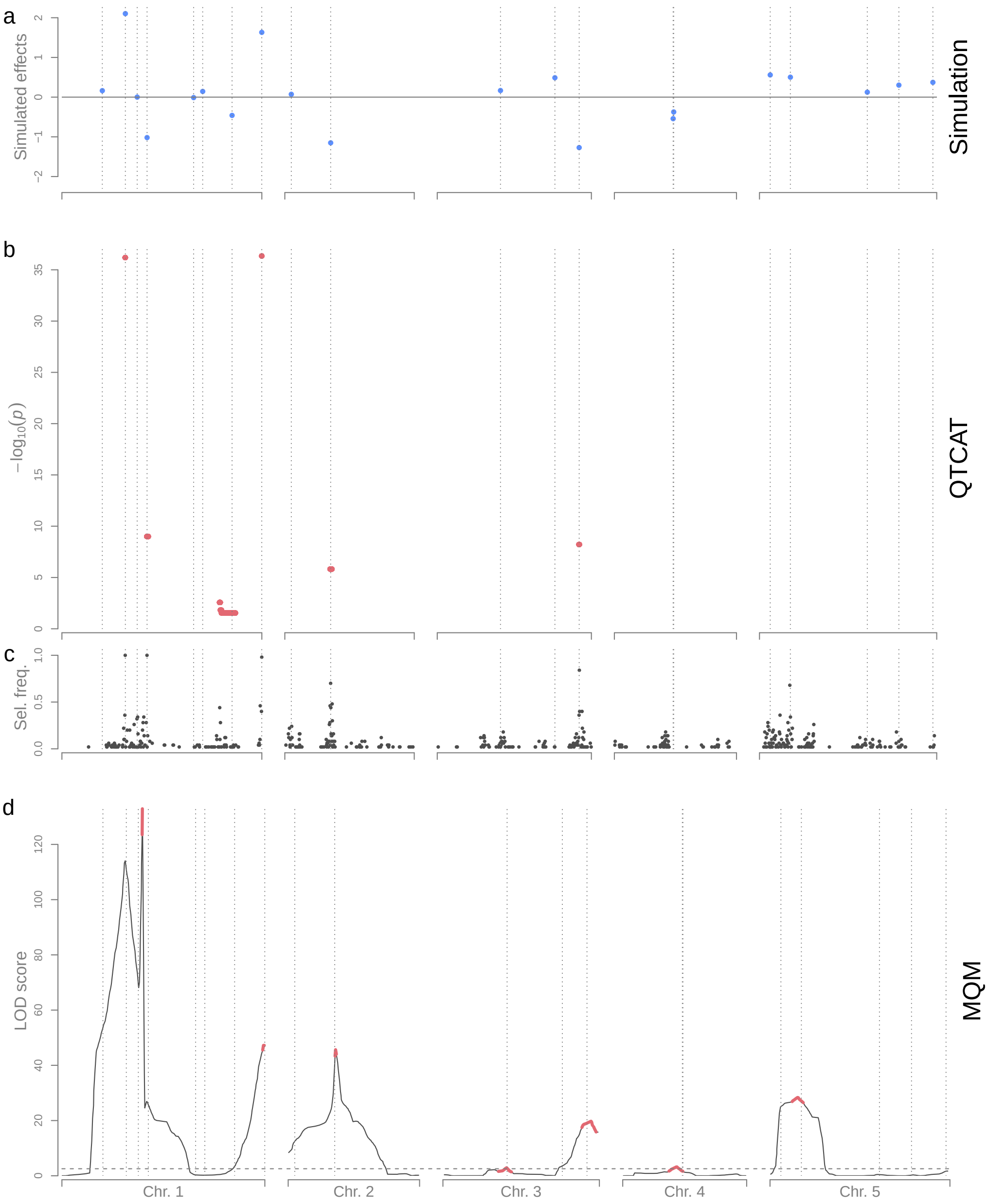

Supplementary Figure 350 Simulation of a GWA analysis based on a unstructured population with a heritability of 0.7 (run 50). (**a**) Simulated of 20 effects randomly drawn from a Gamma distribution and assigned them randomly to markers. Simulated effects randomly drawn from a Gamma distribution. We assigned effects to 20 markers. Markers with an effect are highlighted in (**b**–**d**) with dashed lines. (**b**) Significant QTCs found by QTCAT. (**c**) The selection frequency of the LASSO for each marker during the 50 iterations of QTCAT. (**d**) MQM LOD score plot, the horizontal dashed line is a simulation based perumtation test FDR. The red colored areas represent the LOD-intervals.

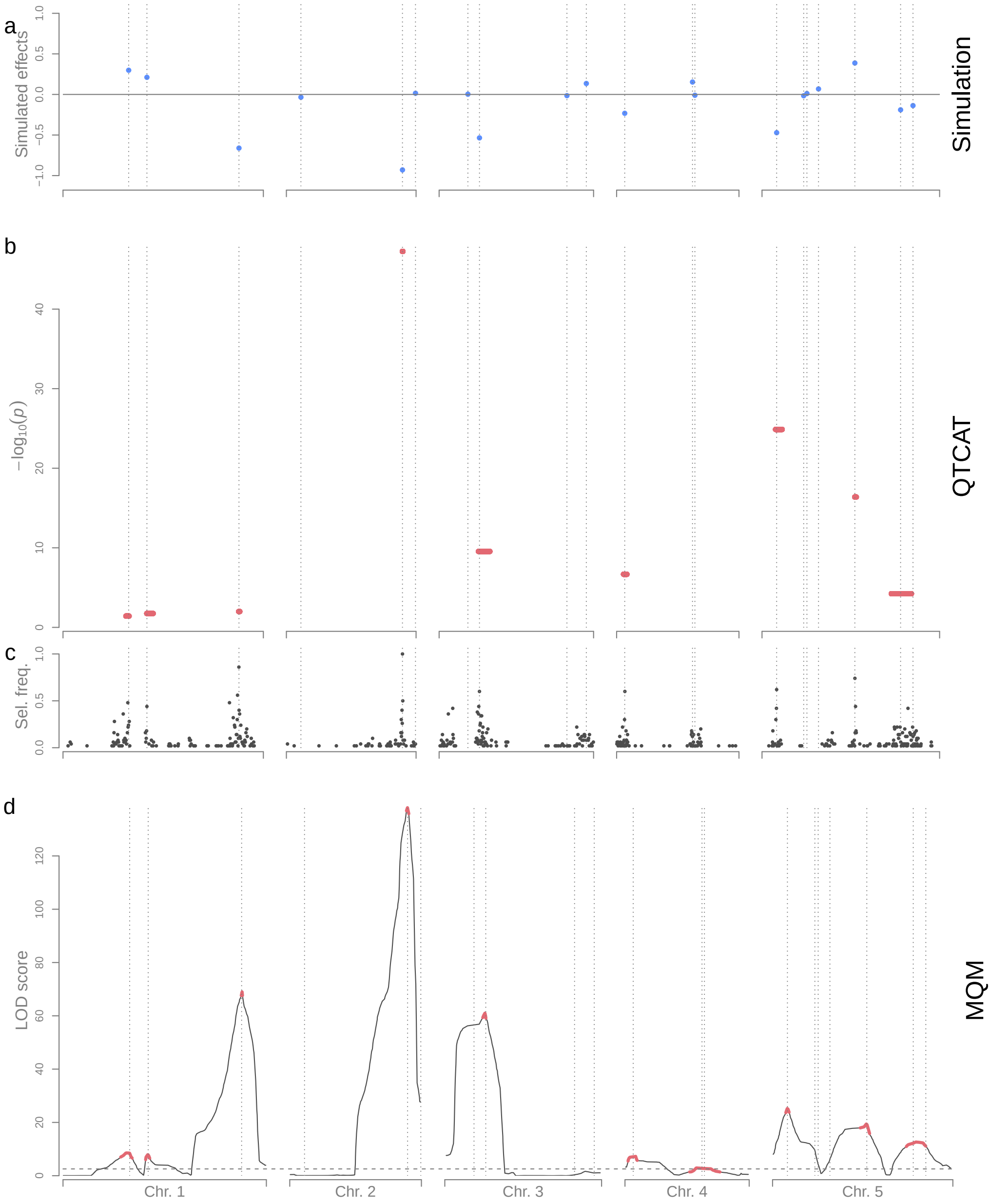

Supplementary Figure 351 Simulation of a GWA analysis based on a unstructured population with a heritability of 0.7 (run 51). (**a**) Simulated of 20 effects randomly drawn from a Gamma distribution and assigned them randomly to markers. Simulated effects randomly drawn from a Gamma distribution. We assigned effects to 20 markers. Markers with an effect are highlighted in (**b–d**) with dashed lines. (**b**) Significant QTCs found by QTCAT. (**c**) The selection frequency of the LASSO for each marker during the 50 iterations of QTCAT. (**d**) MQM LOD score plot, the horizontal dashed line is a simulation based perumtation test FDR. The red colored areas represent the LOD-intervals.

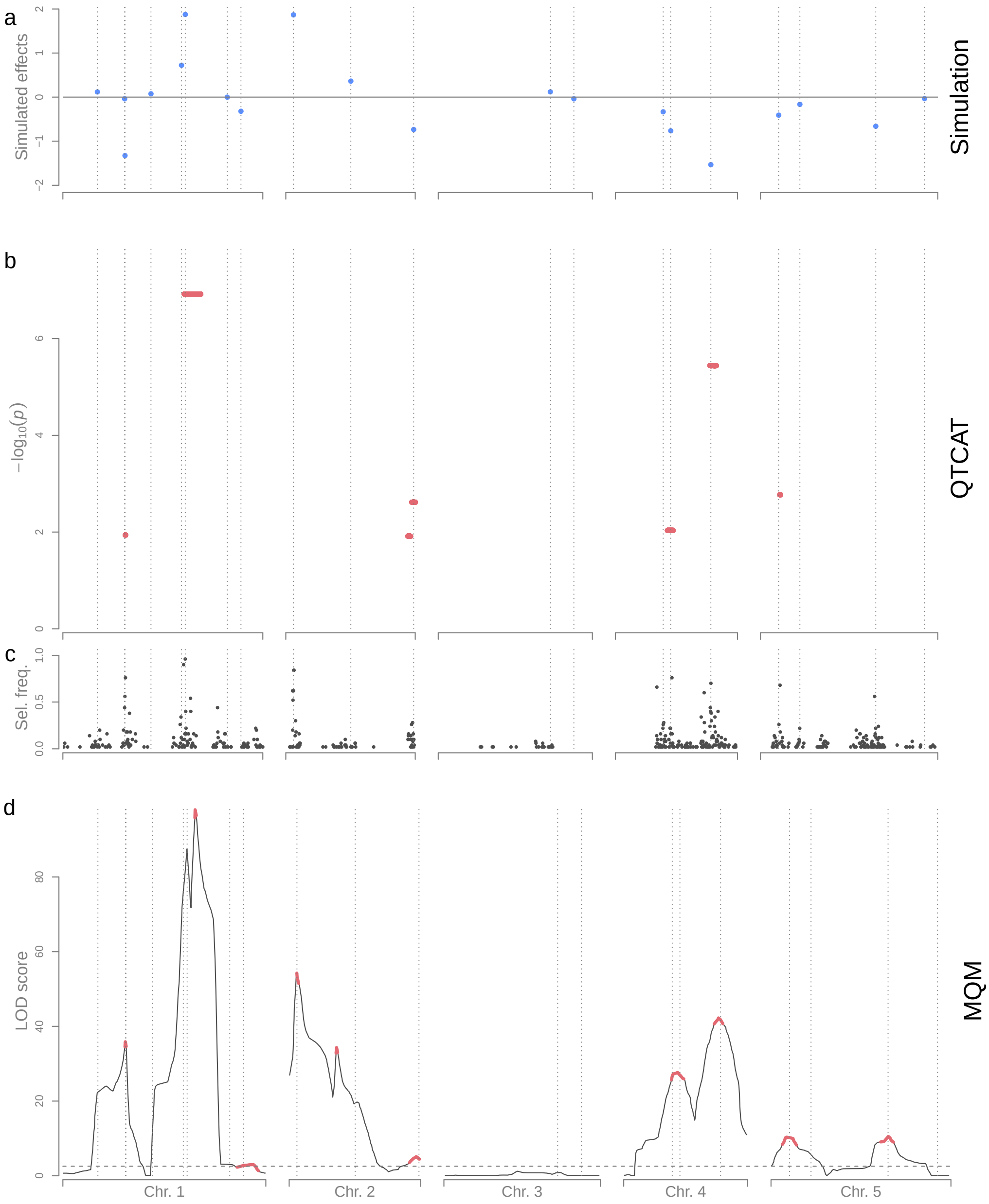

Supplementary Figure 352 Simulation of a GWA analysis based on a unstructured population with a heritability of 0.7 (run 52). (**a**) Simulated of 20 effects randomly drawn from a Gamma distribution and assigned them randomly to markers. Simulated effects randomly drawn from a Gamma distribution. We assigned effects to 20 markers. Markers with an effect are highlighted in (**b**–**d**) with dashed lines. (**b**) Significant QTCs found by QTCAT. (**c**) The selection frequency of the LASSO for each marker during the 50 iterations of QTCAT. (**d**) MQM LOD score plot, the horizontal dashed line is a simulation based perumtation test FDR. The red colored areas represent the LOD-intervals.

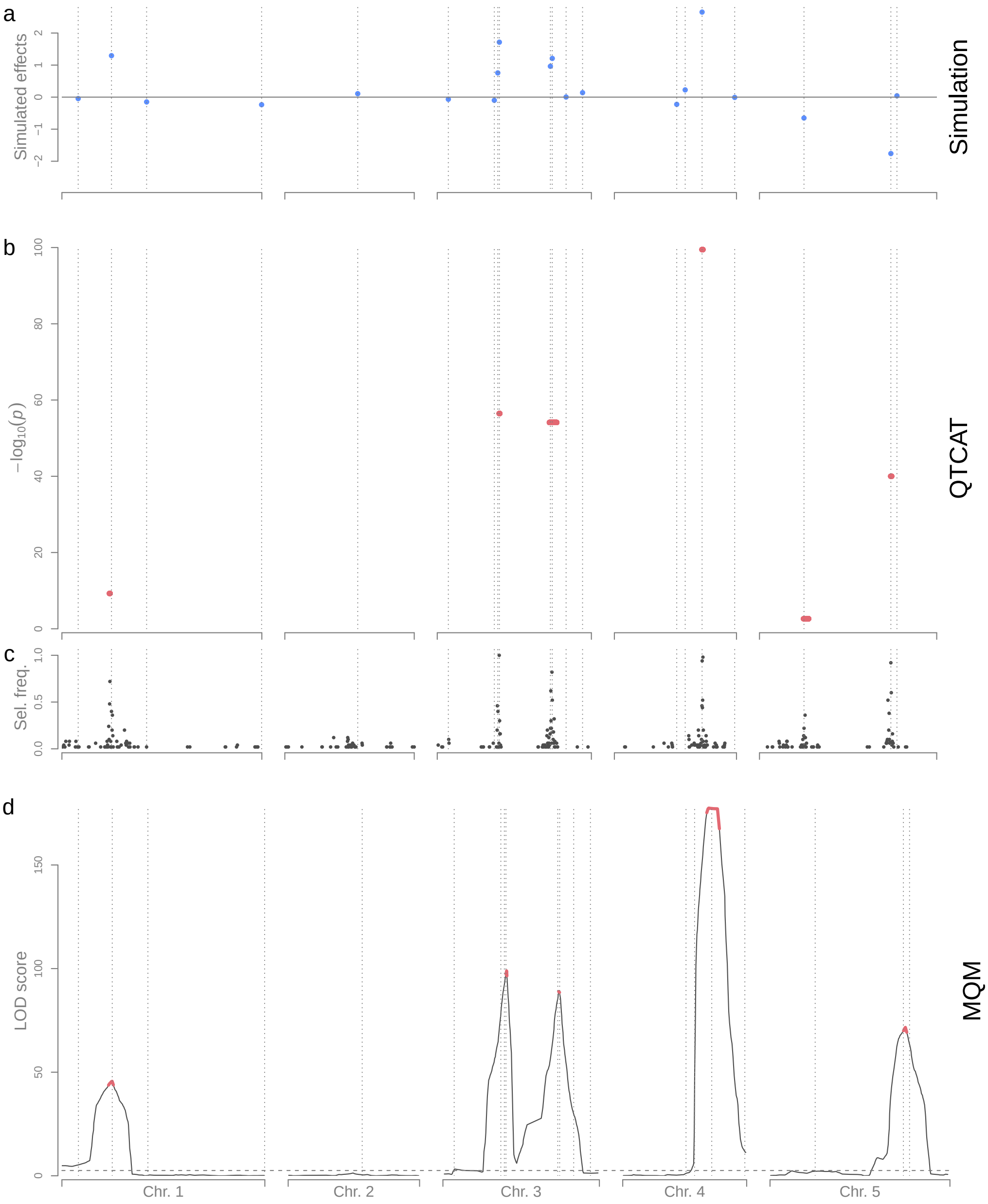

Supplementary Figure 353 Simulation of a GWA analysis based on a unstructured population with a heritability of 0.7 (run 53). (**a**) Simulated of 20 effects randomly drawn from a Gamma distribution and assigned them randomly to markers. Simulated effects randomly drawn from a Gamma distribution. We assigned effects to 20 markers. Markers with an effect are highlighted in (**b**–**d**) with dashed lines. (**b**) Significant QTCs found by QTCAT. (**c**) The selection frequency of the LASSO for each marker during the 50 iterations of QTCAT. (**d**) MQM LOD score plot, the horizontal dashed line is a simulation based perumtation test FDR. The red colored areas represent the LOD-intervals.

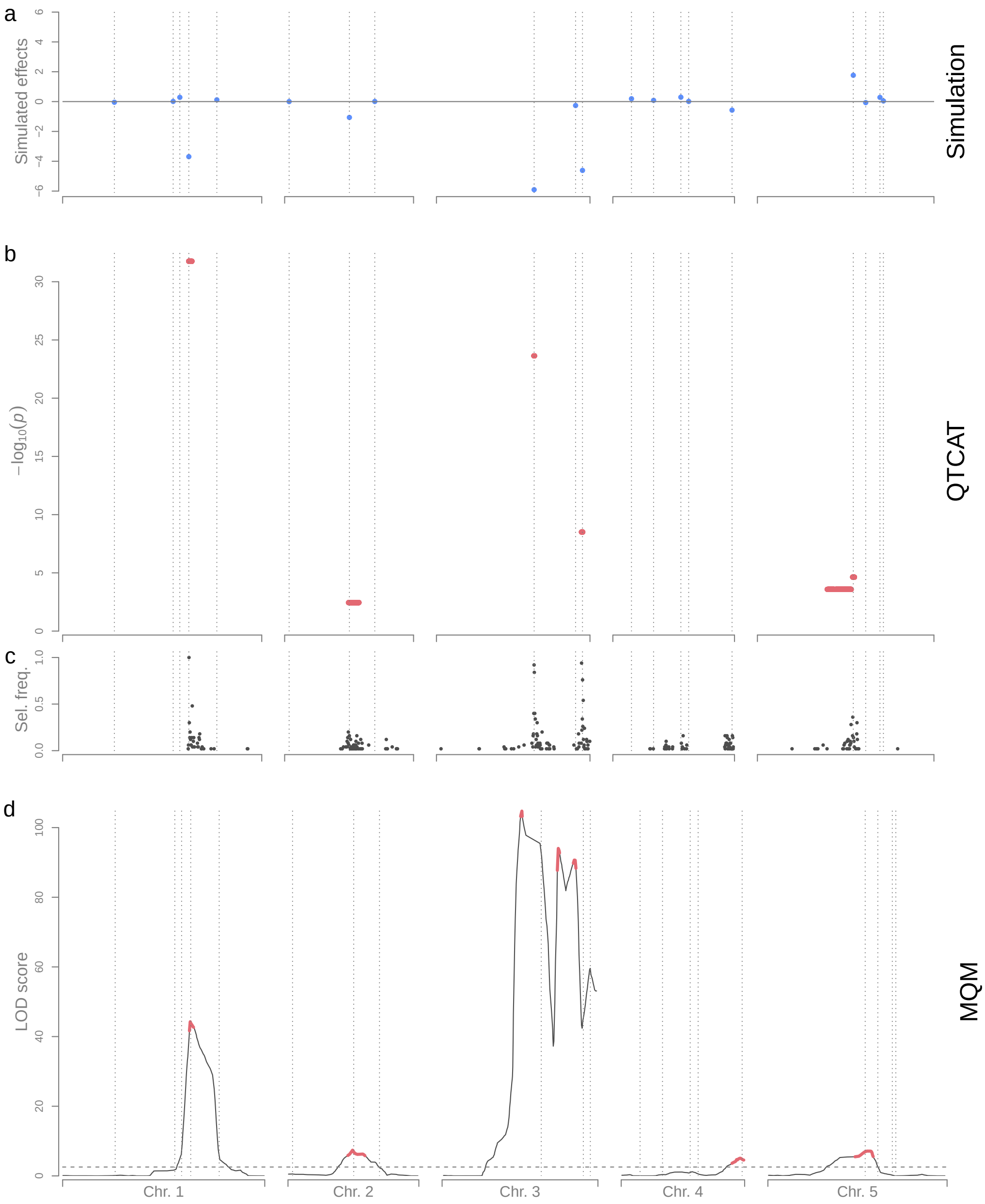

Supplementary Figure 354 Simulation of a GWA analysis based on a unstructured population with a heritability of 0.7 (run 54). (**a**) Simulated of 20 effects randomly drawn from a Gamma distribution and assigned them randomly to markers. Simulated effects randomly drawn from a Gamma distribution. We assigned effects to 20 markers. Markers with an effect are highlighted in (**b–d**) with dashed lines. (**b**) Significant QTCs found by QTCAT. (**c**) The selection frequency of the LASSO for each marker during the 50 iterations of QTCAT. (**d**) MQM LOD score plot, the horizontal dashed line is a simulation based perumtation test FDR. The red colored areas represent the LOD-intervals.

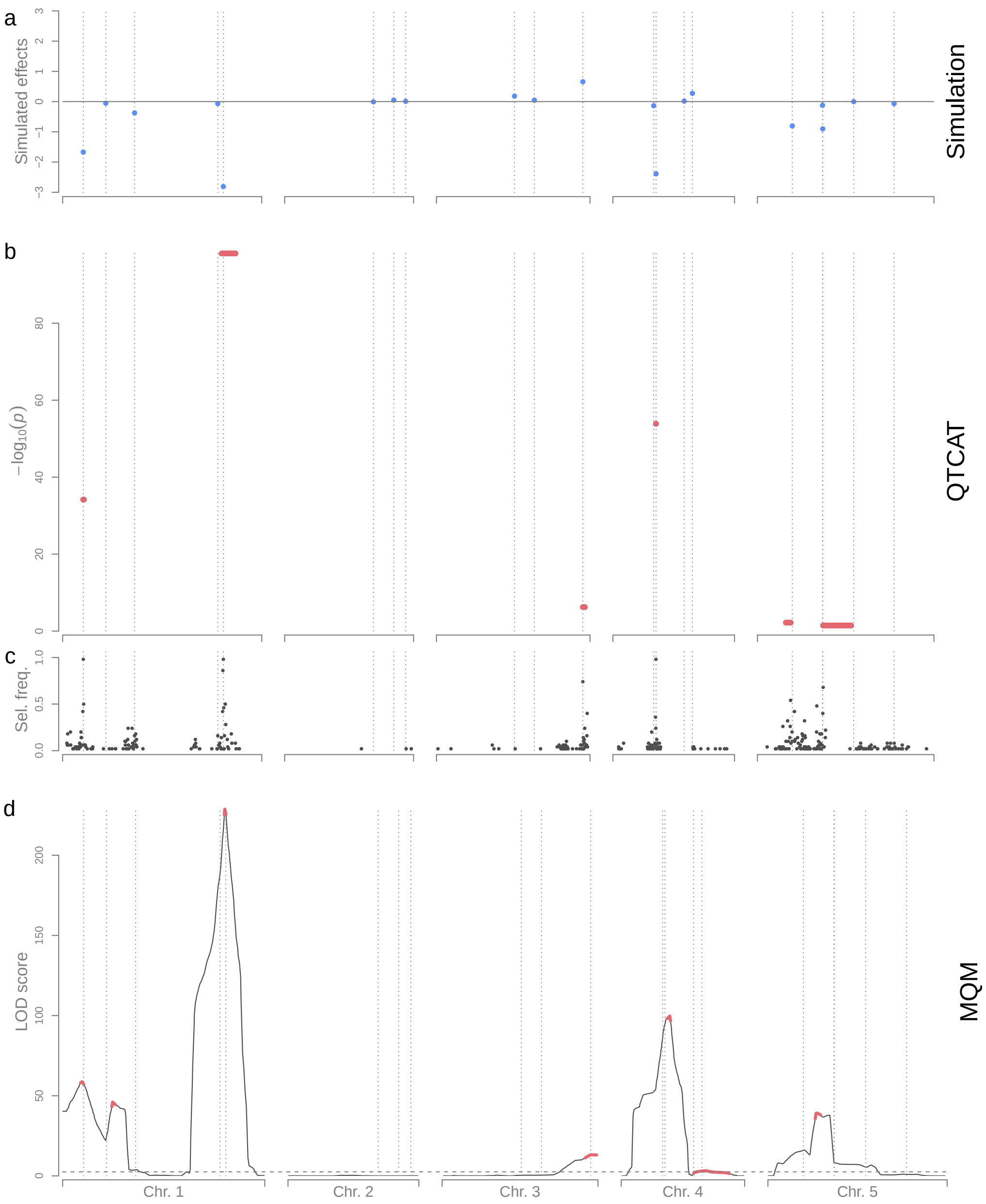

Supplementary Figure 355 Simulation of a GWA analysis based on a unstructured population with a heritability of 0.7 (run 55). (**a**) Simulated of 20 effects randomly drawn from a Gamma distribution and assigned them randomly to markers. Simulated effects randomly drawn from a Gamma distribution. We assigned effects to 20 markers. Markers with an effect are highlighted in (**b–d**) with dashed lines. (**b**) Significant QTCs found by QTCAT. (**c**) The selection frequency of the LASSO for each marker during the 50 iterations of QTCAT. (**d**) MQM LOD score plot, the horizontal dashed line is a simulation based perumtation test FDR. The red colored areas represent the LOD-intervals.

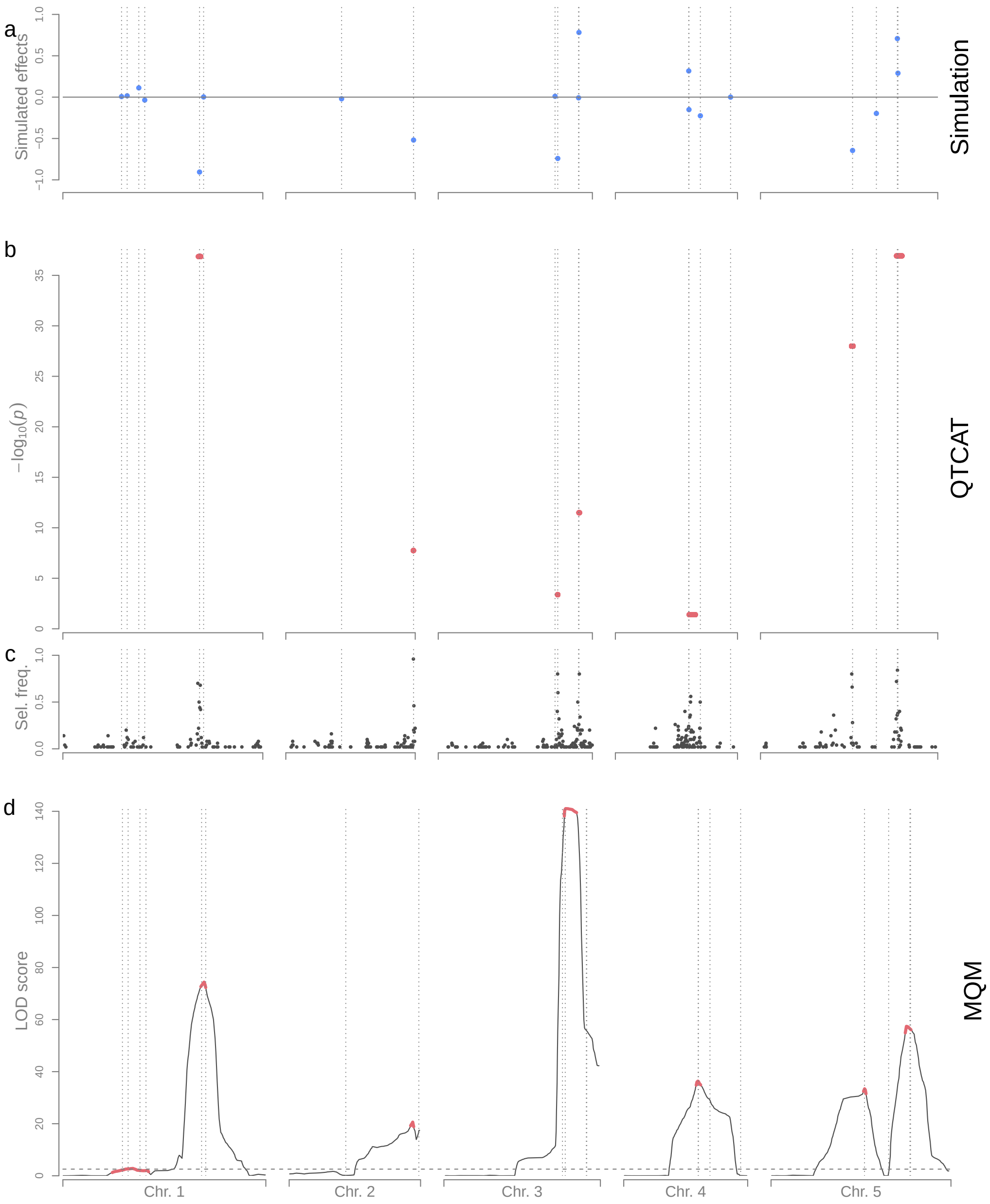

Supplementary Figure 356 Simulation of a GWA analysis based on a unstructured population with a heritability of 0.7 (run 56). (**a**) Simulated of 20 effects randomly drawn from a Gamma distribution and assigned them randomly to markers. Simulated effects randomly drawn from a Gamma distribution. We assigned effects to 20 markers. Markers with an effect are highlighted in (**b**–**d**) with dashed lines. (**b**) Significant QTCs found by QTCAT. (**c**) The selection frequency of the LASSO for each marker during the 50 iterations of QTCAT. (**d**) MQM LOD score plot, the horizontal dashed line is a simulation based perumtation test FDR. The red colored areas represent the LOD-intervals.

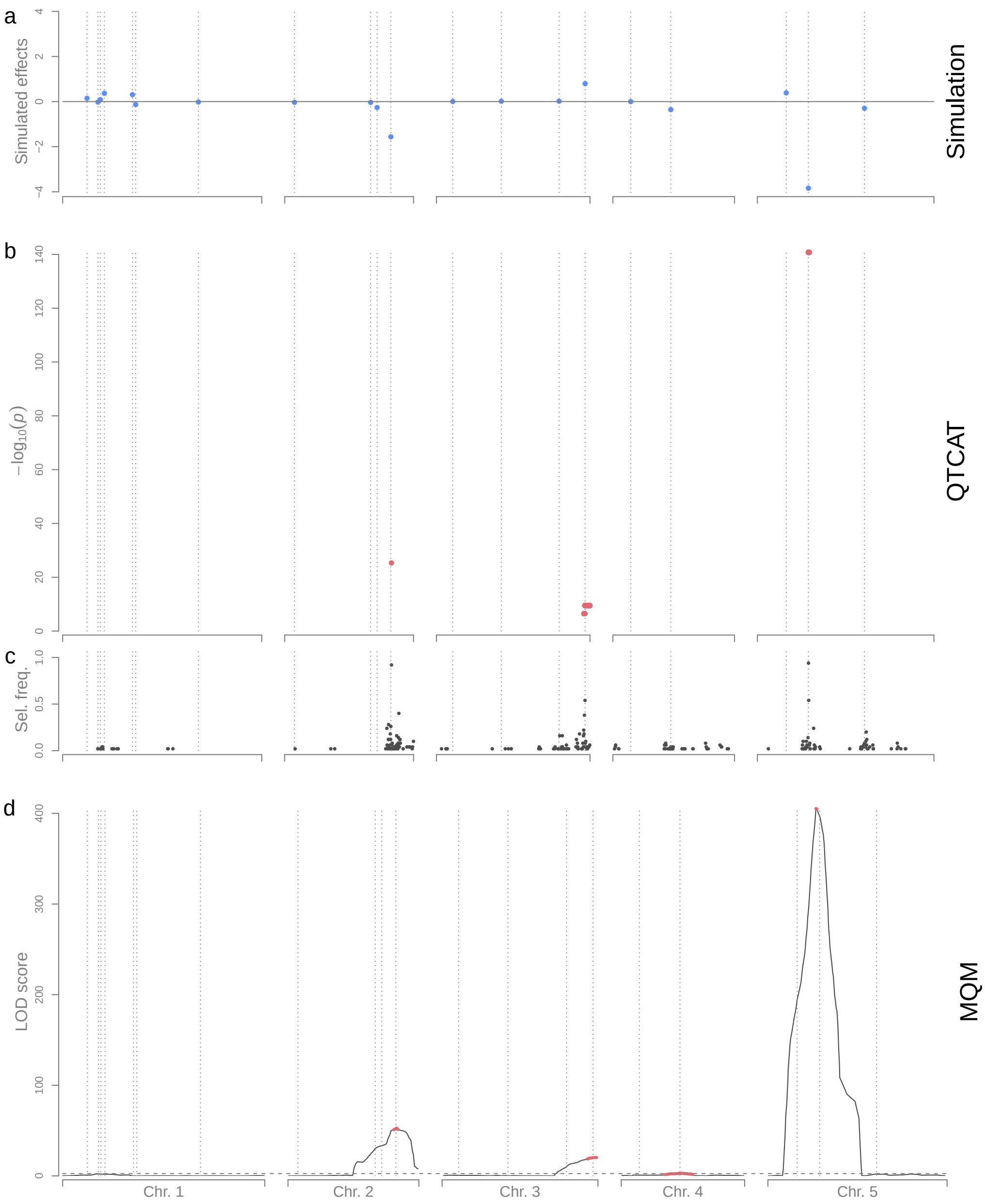

Supplementary Figure 357 Simulation of a GWA analysis based on a unstructured population with a heritability of 0.7 (run 57). (**a**) Simulated of 20 effects randomly drawn from a Gamma distribution and assigned them randomly to markers. Simulated effects randomly drawn from a Gamma distribution. We assigned effects to 20 markers. Markers with an effect are highlighted in (**b–d**) with dashed lines. (**b**) Significant QTCs found by QTCAT. (**c**) The selection frequency of the LASSO for each marker during the 50 iterations of QTCAT. (**d**) MQM LOD score plot, the horizontal dashed line is a simulation based perumtation test FDR. The red colored areas represent the LOD-intervals.

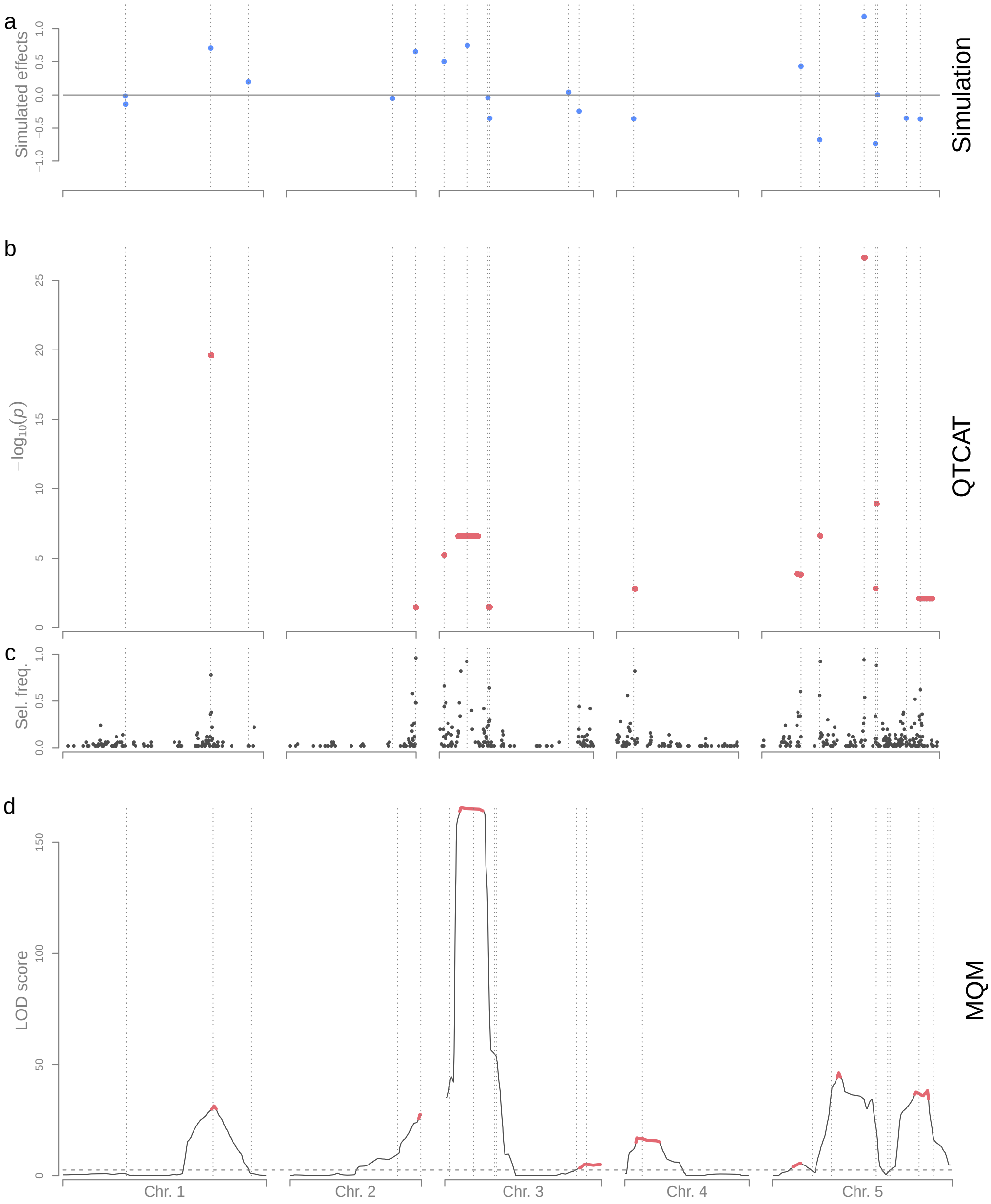

Supplementary Figure 358 Simulation of a GWA analysis based on a unstructured population with a heritability of 0.7 (run 58). (**a**) Simulated of 20 effects randomly drawn from a Gamma distribution and assigned them randomly to markers. Simulated effects randomly drawn from a Gamma distribution. We assigned effects to 20 markers. Markers with an effect are highlighted in (**b**–**d**) with dashed lines. (**b**) Significant QTCs found by QTCAT. (**c**) The selection frequency of the LASSO for each marker during the 50 iterations of QTCAT. (**d**) MQM LOD score plot, the horizontal dashed line is a simulation based perumtation test FDR. The red colored areas represent the LOD-intervals.

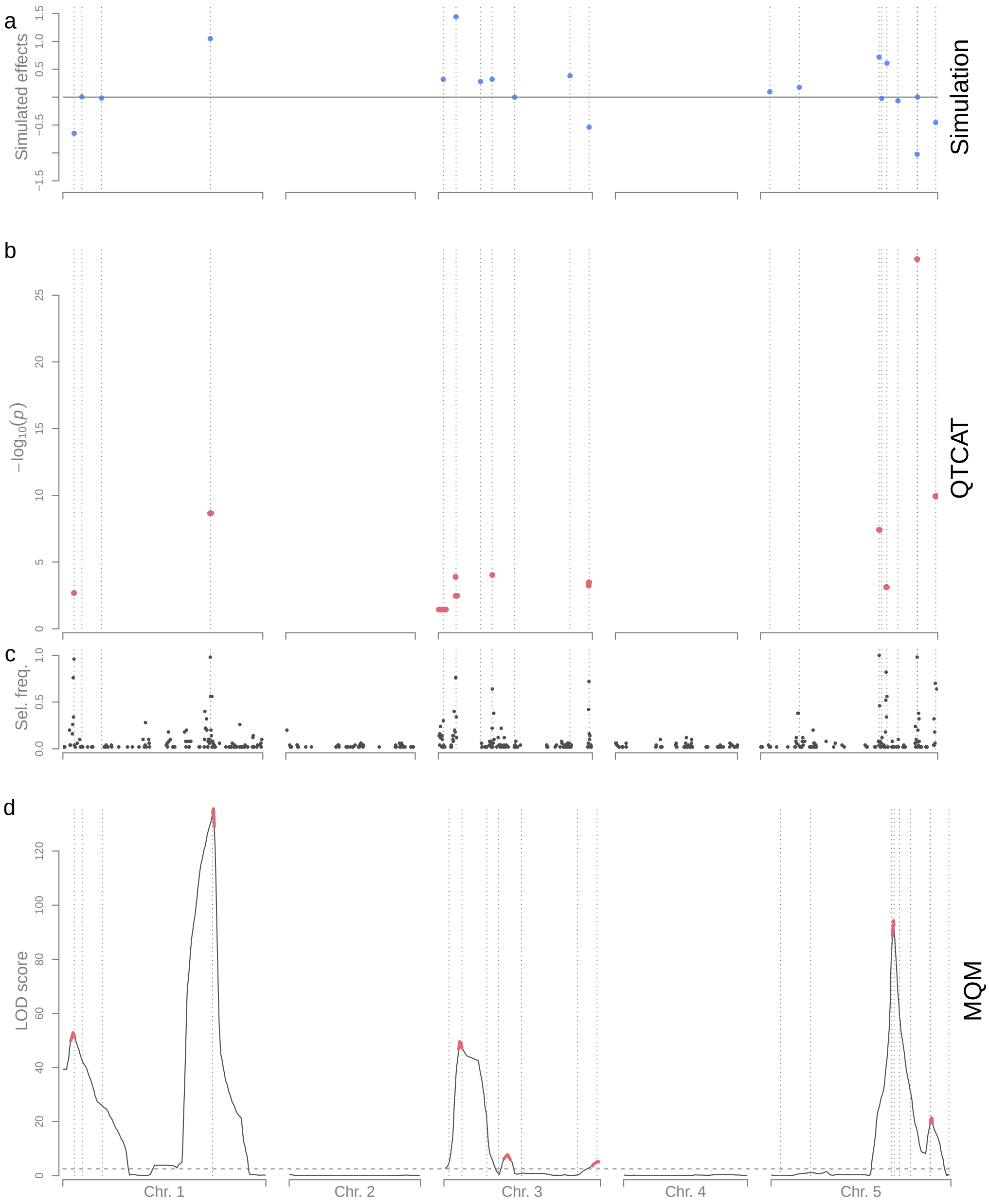

Supplementary Figure 359 Simulation of a GWA analysis based on a unstructured population with a heritability of 0.7 (run 59). (**a**) Simulated of 20 effects randomly drawn from a Gamma distribution and assigned them randomly to markers. Simulated effects randomly drawn from a Gamma distribution. We assigned effects to 20 markers. Markers with an effect are highlighted in (**b–d**) with dashed lines. (**b**) Significant QTCs found by QTCAT. (**c**) The selection frequency of the LASSO for each marker during the 50 iterations of QTCAT. (**d**) MQM LOD score plot, the horizontal dashed line is a simulation based perumtation test FDR. The red colored areas represent the LOD-intervals.

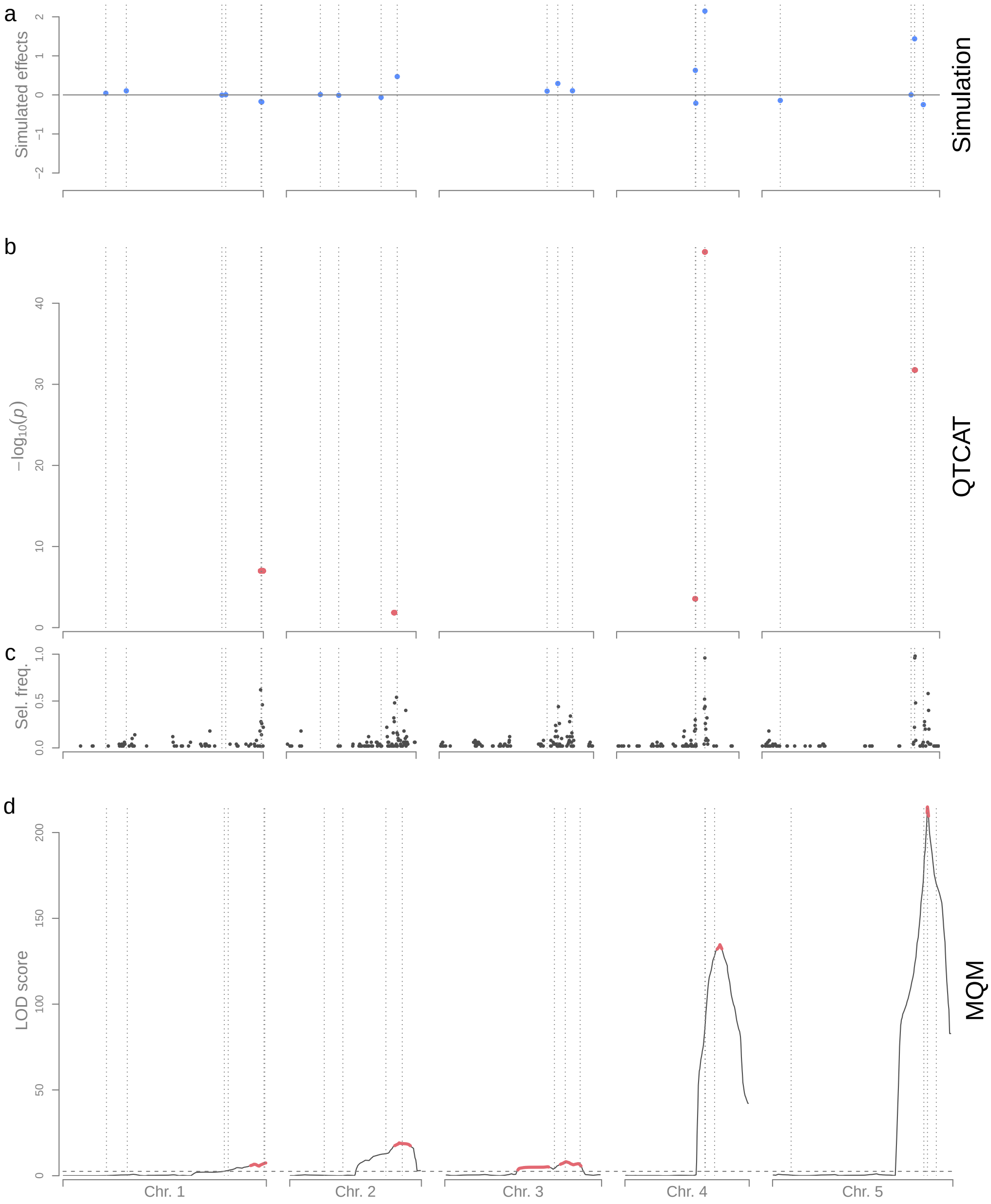

Supplementary Figure 360 Simulation of a GWA analysis based on a unstructured population with a heritability of 0.7 (run 60). (**a**) Simulated of 20 effects randomly drawn from a Gamma distribution and assigned them randomly to markers. Simulated effects randomly drawn from a Gamma distribution. We assigned effects to 20 markers. Markers with an effect are highlighted in (**b**–**d**) with dashed lines. (**b**) Significant QTCs found by QTCAT. (**c**) The selection frequency of the LASSO for each marker during the 50 iterations of QTCAT. (**d**) MQM LOD score plot, the horizontal dashed line is a simulation based perumtation test FDR. The red colored areas represent the LOD-intervals.

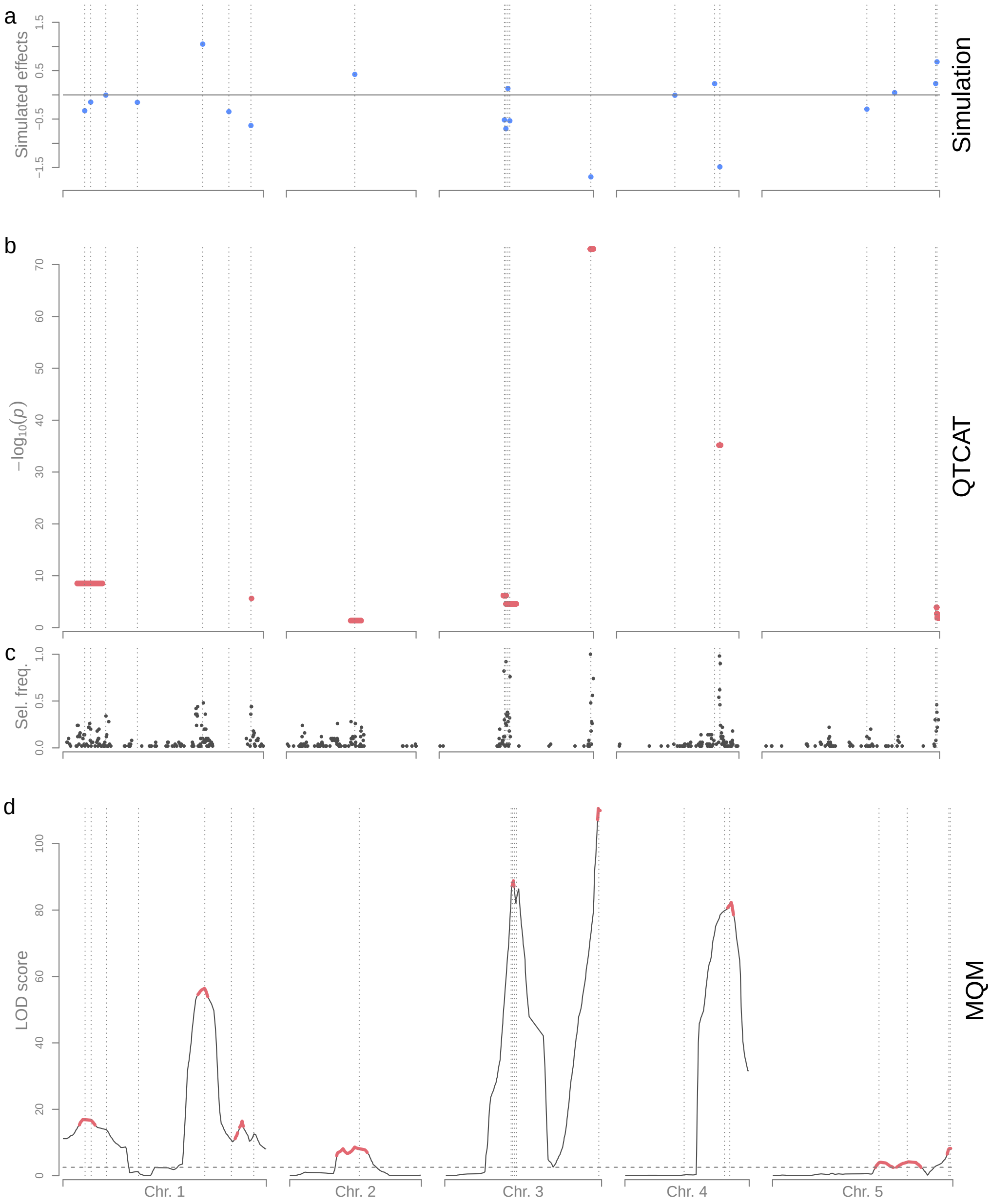

Supplementary Figure 361 Simulation of a GWA analysis based on a unstructured population with a heritability of 0.7 (run 61). (**a**) Simulated of 20 effects randomly drawn from a Gamma distribution and assigned them randomly to markers. Simulated effects randomly drawn from a Gamma distribution. We assigned effects to 20 markers. Markers with an effect are highlighted in (**b–d**) with dashed lines. (**b**) Significant QTCs found by QTCAT. (**c**) The selection frequency of the LASSO for each marker during the 50 iterations of QTCAT. (**d**) MQM LOD score plot, the horizontal dashed line is a simulation based perumtation test FDR. The red colored areas represent the LOD-intervals.

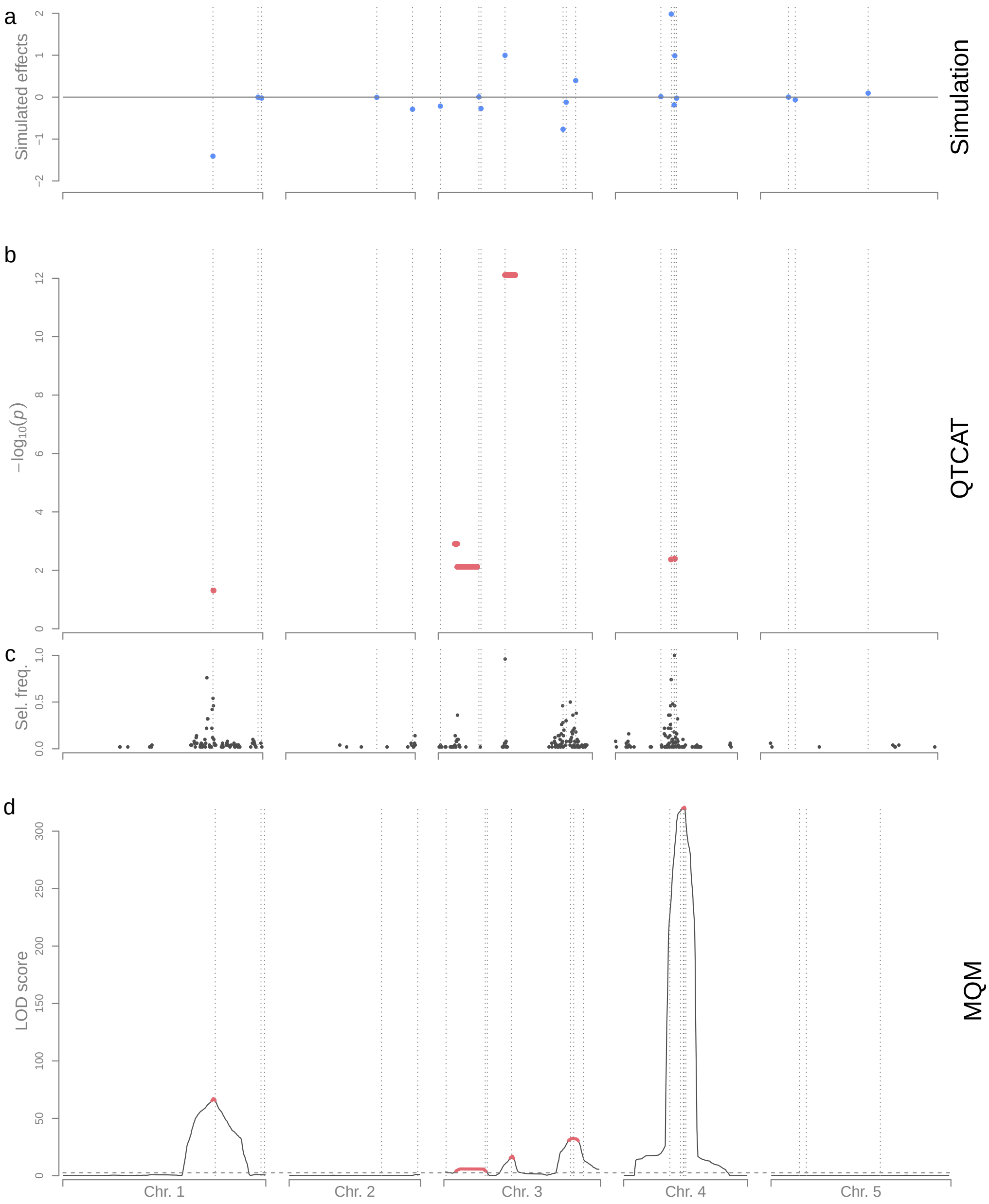

Supplementary Figure 362 Simulation of a GWA analysis based on a unstructured population with a heritability of 0.7 (run 62). (**a**) Simulated of 20 effects randomly drawn from a Gamma distribution and assigned them randomly to markers. Simulated effects randomly drawn from a Gamma distribution. We assigned effects to 20 markers. Markers with an effect are highlighted in (**b**–**d**) with dashed lines. (**b**) Significant QTCs found by QTCAT. (**c**) The selection frequency of the LASSO for each marker during the 50 iterations of QTCAT. (**d**) MQM LOD score plot, the horizontal dashed line is a simulation based perumtation test FDR. The red colored areas represent the LOD-intervals.

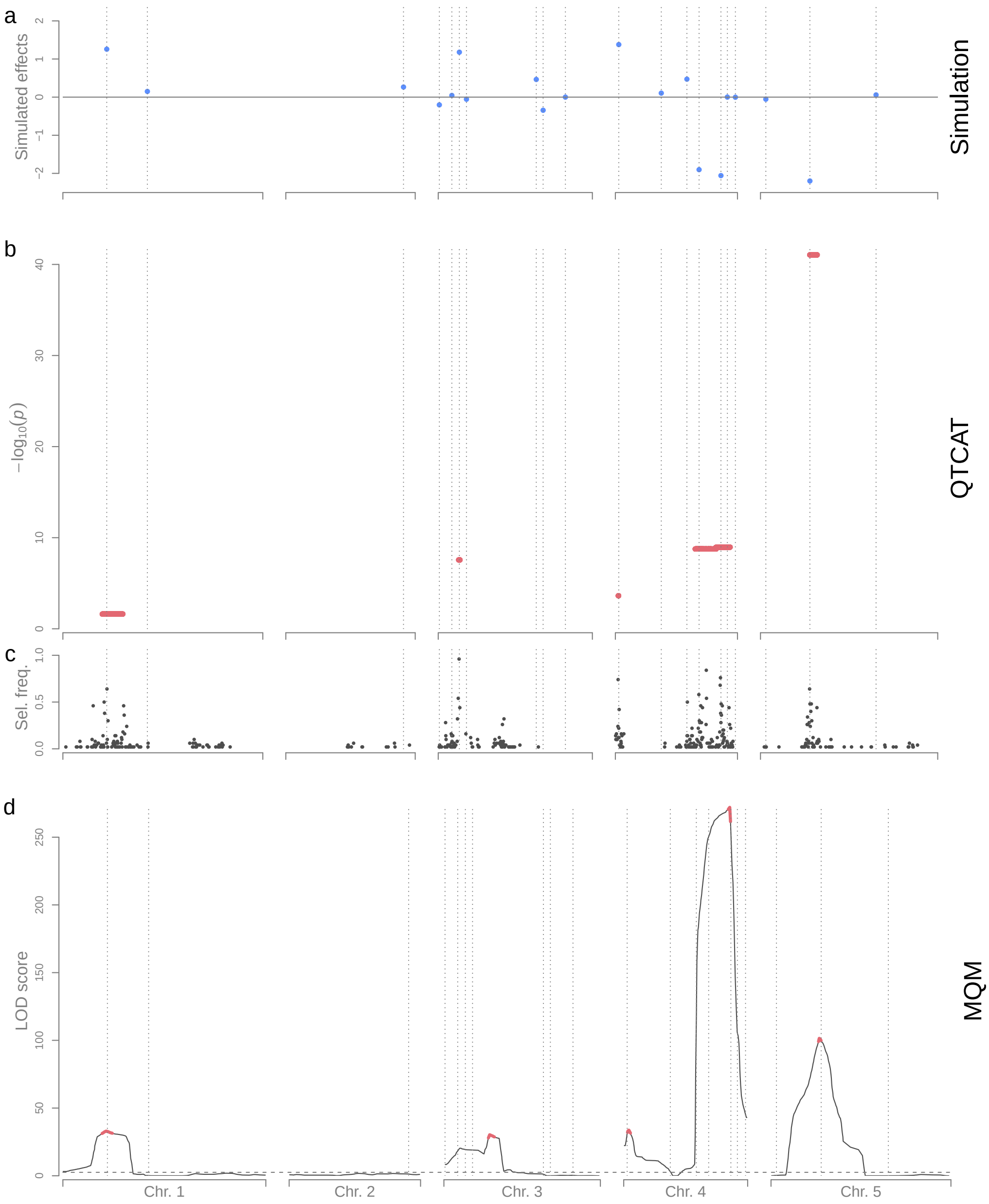

Supplementary Figure 363 Simulation of a GWA analysis based on a unstructured population with a heritability of 0.7 (run 63). (**a**) Simulated of 20 effects randomly drawn from a Gamma distribution and assigned them randomly to markers. Simulated effects randomly drawn from a Gamma distribution. We assigned effects to 20 markers. Markers with an effect are highlighted in (**b–d**) with dashed lines. (**b**) Significant QTCs found by QTCAT. (**c**) The selection frequency of the LASSO for each marker during the 50 iterations of QTCAT. (**d**) MQM LOD score plot, the horizontal dashed line is a simulation based perumtation test FDR. The red colored areas represent the LOD-intervals.

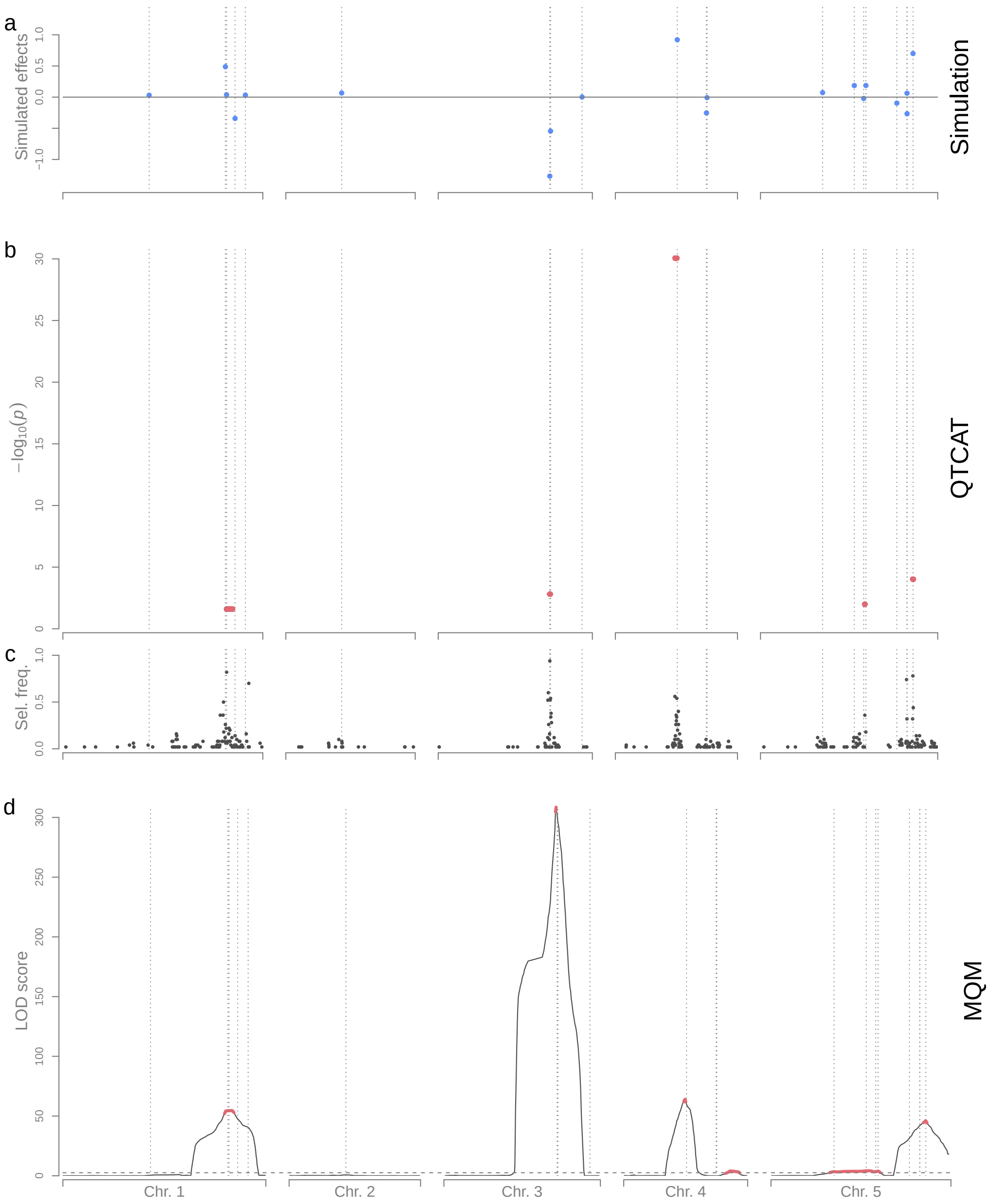

Supplementary Figure 364 Simulation of a GWA analysis based on a unstructured population with a heritability of 0.7 (run 64). (**a**) Simulated of 20 effects randomly drawn from a Gamma distribution and assigned them randomly to markers. Simulated effects randomly drawn from a Gamma distribution. We assigned effects to 20 markers. Markers with an effect are highlighted in (**b–d**) with dashed lines. (**b**) Significant QTCs found by QTCAT. (**c**) The selection frequency of the LASSO for each marker during the 50 iterations of QTCAT. (**d**) MQM LOD score plot, the horizontal dashed line is a simulation based perumtation test FDR. The red colored areas represent the LOD-intervals.

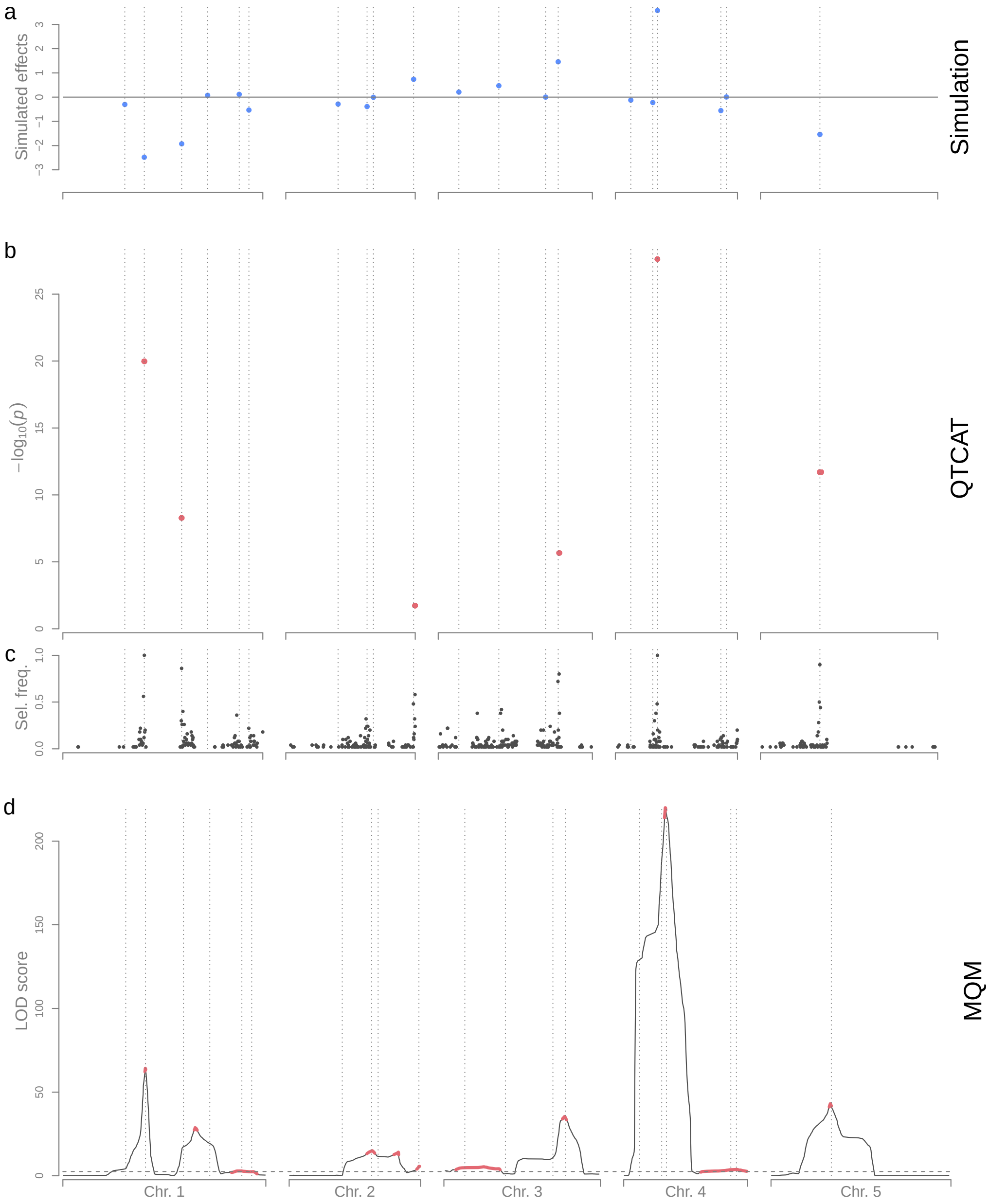

Supplementary Figure 365 Simulation of a GWA analysis based on a unstructured population with a heritability of 0.7 (run 65). (**a**) Simulated of 20 effects randomly drawn from a Gamma distribution and assigned them randomly to markers. Simulated effects randomly drawn from a Gamma distribution. We assigned effects to 20 markers. Markers with an effect are highlighted in (**b**–**d**) with dashed lines. (**b**) Significant QTCs found by QTCAT. (**c**) The selection frequency of the LASSO for each marker during the 50 iterations of QTCAT. (**d**) MQM LOD score plot, the horizontal dashed line is a simulation based perumtation test FDR. The red colored areas represent the LOD-intervals.

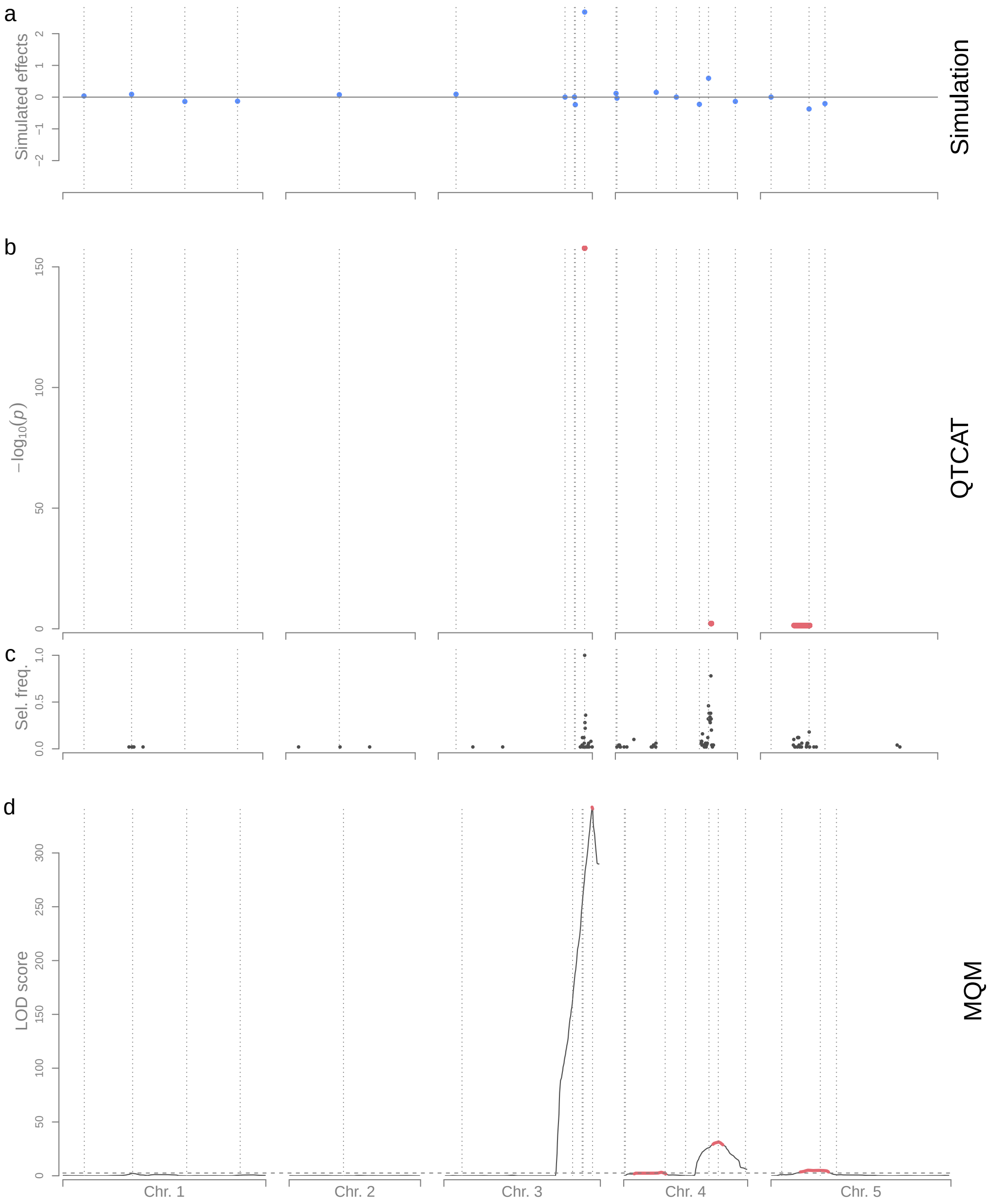

Supplementary Figure 366 Simulation of a GWA analysis based on a unstructured population with a heritability of 0.7 (run 66). (**a**) Simulated of 20 effects randomly drawn from a Gamma distribution and assigned them randomly to markers. Simulated effects randomly drawn from a Gamma distribution. We assigned effects to 20 markers. Markers with an effect are highlighted in (**b**–**d**) with dashed lines. (**b**) Significant QTCs found by QTCAT. (**c**) The selection frequency of the LASSO for each marker during the 50 iterations of QTCAT. (**d**) MQM LOD score plot, the horizontal dashed line is a simulation based perumtation test FDR. The red colored areas represent the LOD-intervals.

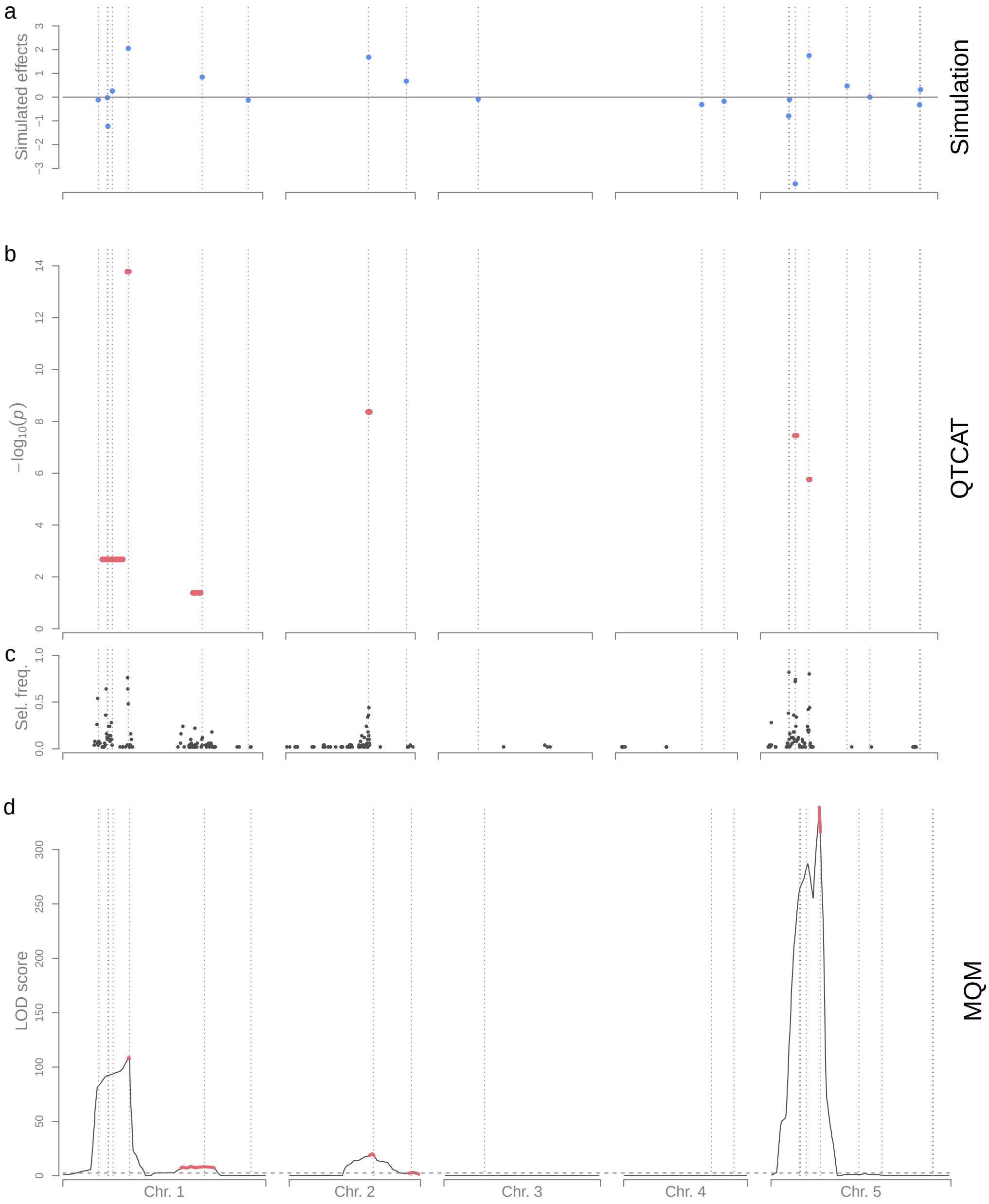

Supplementary Figure 367 Simulation of a GWA analysis based on a unstructured population with a heritability of 0.7 (run 67). (**a**) Simulated of 20 effects randomly drawn from a Gamma distribution and assigned them randomly to markers. Simulated effects randomly drawn from a Gamma distribution. We assigned effects to 20 markers. Markers with an effect are highlighted in (**b–d**) with dashed lines. (**b**) Significant QTCs found by QTCAT. (**c**) The selection frequency of the LASSO for each marker during the 50 iterations of QTCAT. (**d**) MQM LOD score plot, the horizontal dashed line is a simulation based perumtation test FDR. The red colored areas represent the LOD-intervals.

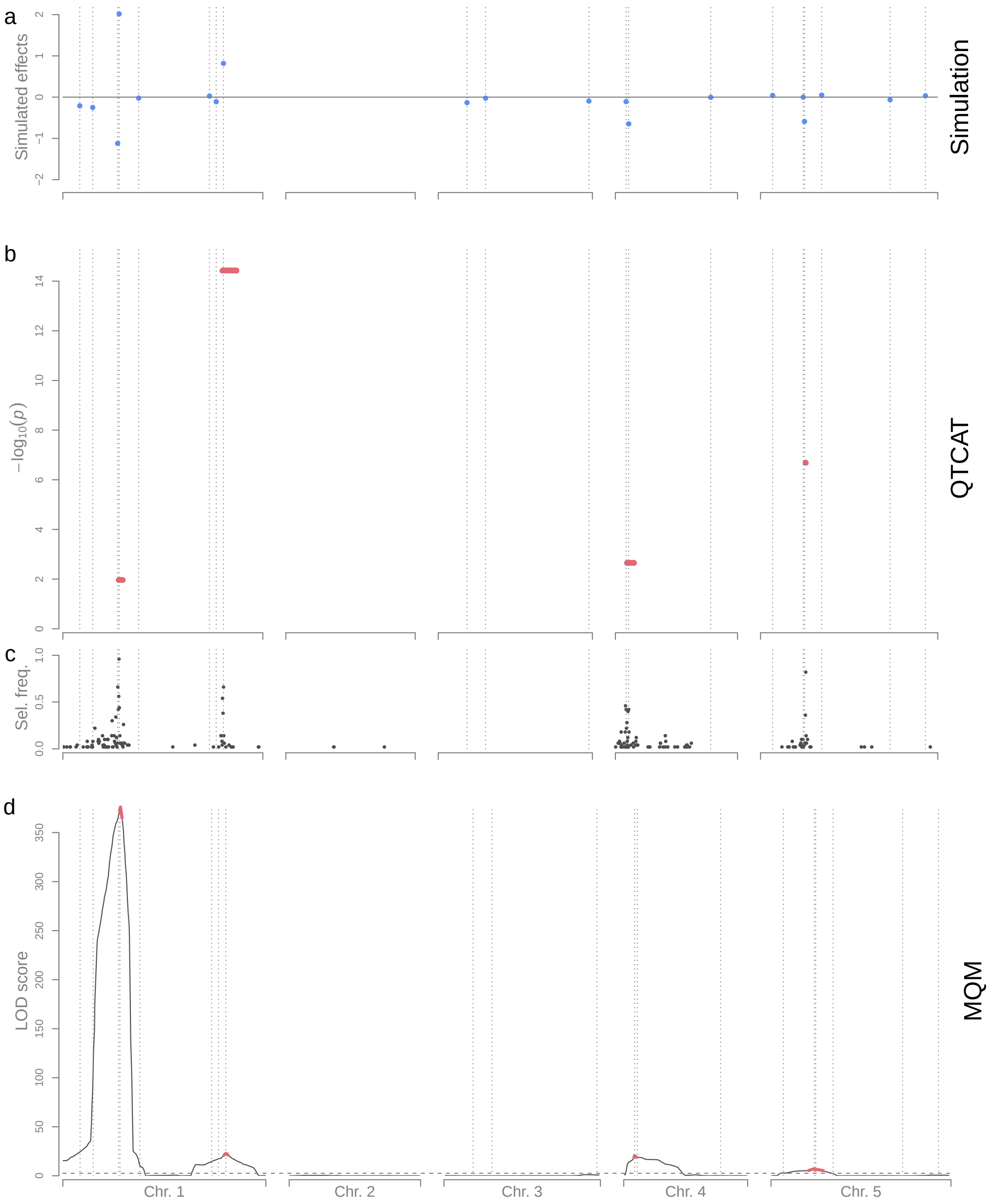

Supplementary Figure 368 Simulation of a GWA analysis based on a unstructured population with a heritability of 0.7 (run 68). (**a**) Simulated of 20 effects randomly drawn from a Gamma distribution and assigned them randomly to markers. Simulated effects randomly drawn from a Gamma distribution. We assigned effects to 20 markers. Markers with an effect are highlighted in (**b–d**) with dashed lines. (**b**) Significant QTCs found by QTCAT. (**c**) The selection frequency of the LASSO for each marker during the 50 iterations of QTCAT. (**d**) MQM LOD score plot, the horizontal dashed line is a simulation based perumtation test FDR. The red colored areas represent the LOD-intervals.

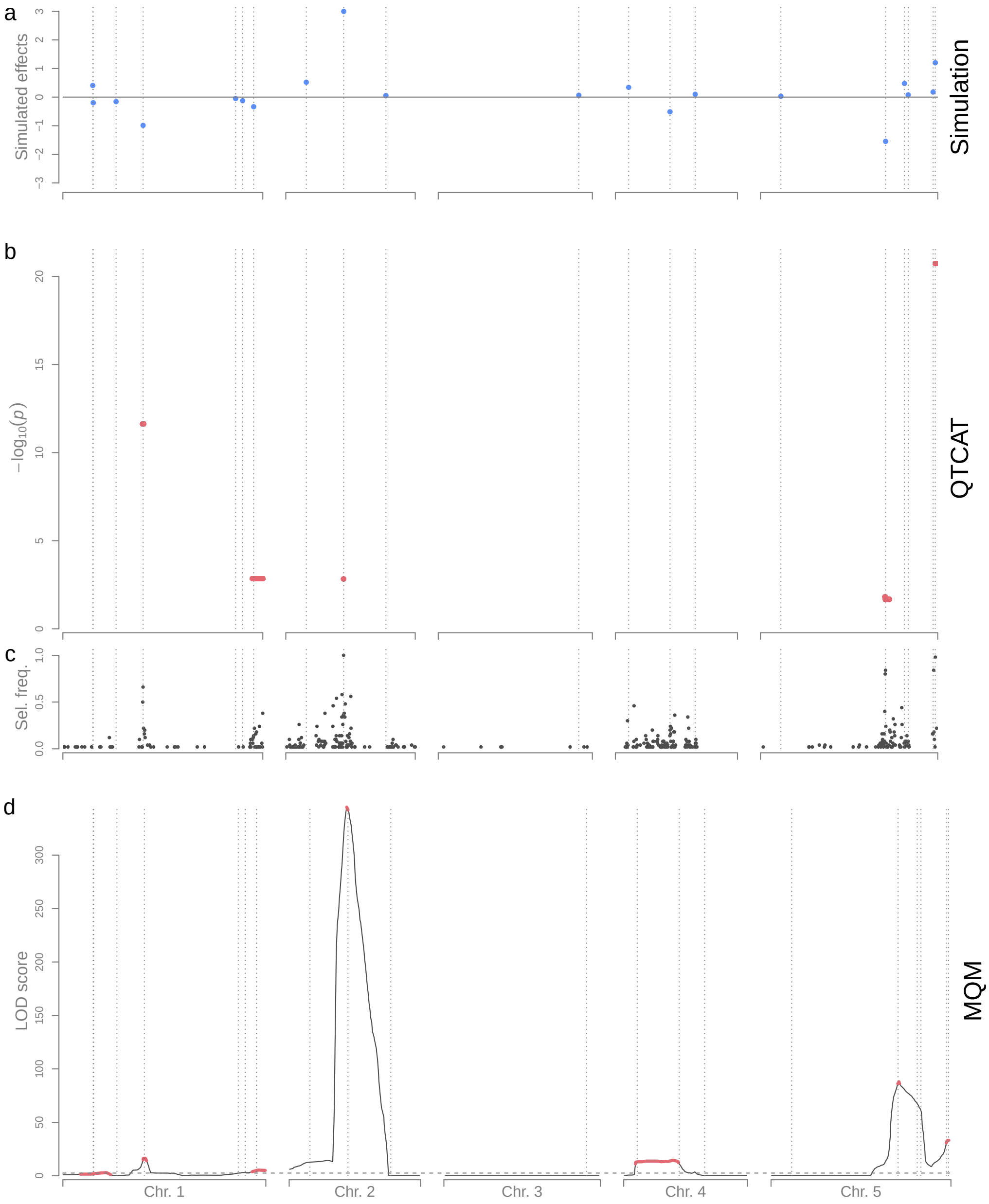

Supplementary Figure 369 Simulation of a GWA analysis based on a unstructured population with a heritability of 0.7 (run 69). (**a**) Simulated of 20 effects randomly drawn from a Gamma distribution and assigned them randomly to markers. Simulated effects randomly drawn from a Gamma distribution. We assigned effects to 20 markers. Markers with an effect are highlighted in (**b–d**) with dashed lines. (**b**) Significant QTCs found by QTCAT. (**c**) The selection frequency of the LASSO for each marker during the 50 iterations of QTCAT. (**d**) MQM LOD score plot, the horizontal dashed line is a simulation based perumtation test FDR. The red colored areas represent the LOD-intervals.

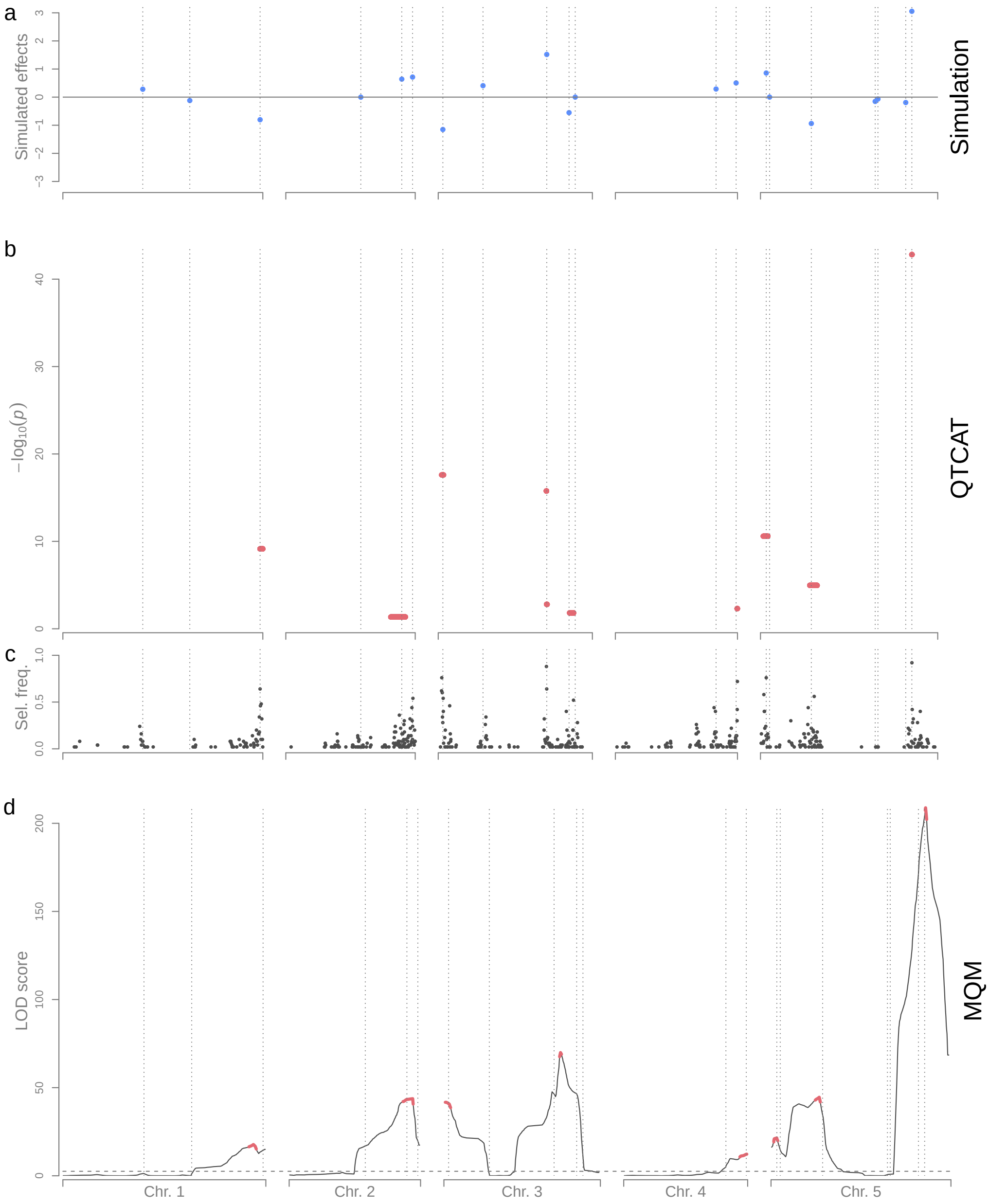

Supplementary Figure 370 Simulation of a GWA analysis based on a unstructured population with a heritability of 0.7 (run 70). (**a**) Simulated of 20 effects randomly drawn from a Gamma distribution and assigned them randomly to markers. Simulated effects randomly drawn from a Gamma distribution. We assigned effects to 20 markers. Markers with an effect are highlighted in (**b**–**d**) with dashed lines. (**b**) Significant QTCs found by QTCAT. (**c**) The selection frequency of the LASSO for each marker during the 50 iterations of QTCAT. (**d**) MQM LOD score plot, the horizontal dashed line is a simulation based perumtation test FDR. The red colored areas represent the LOD-intervals.

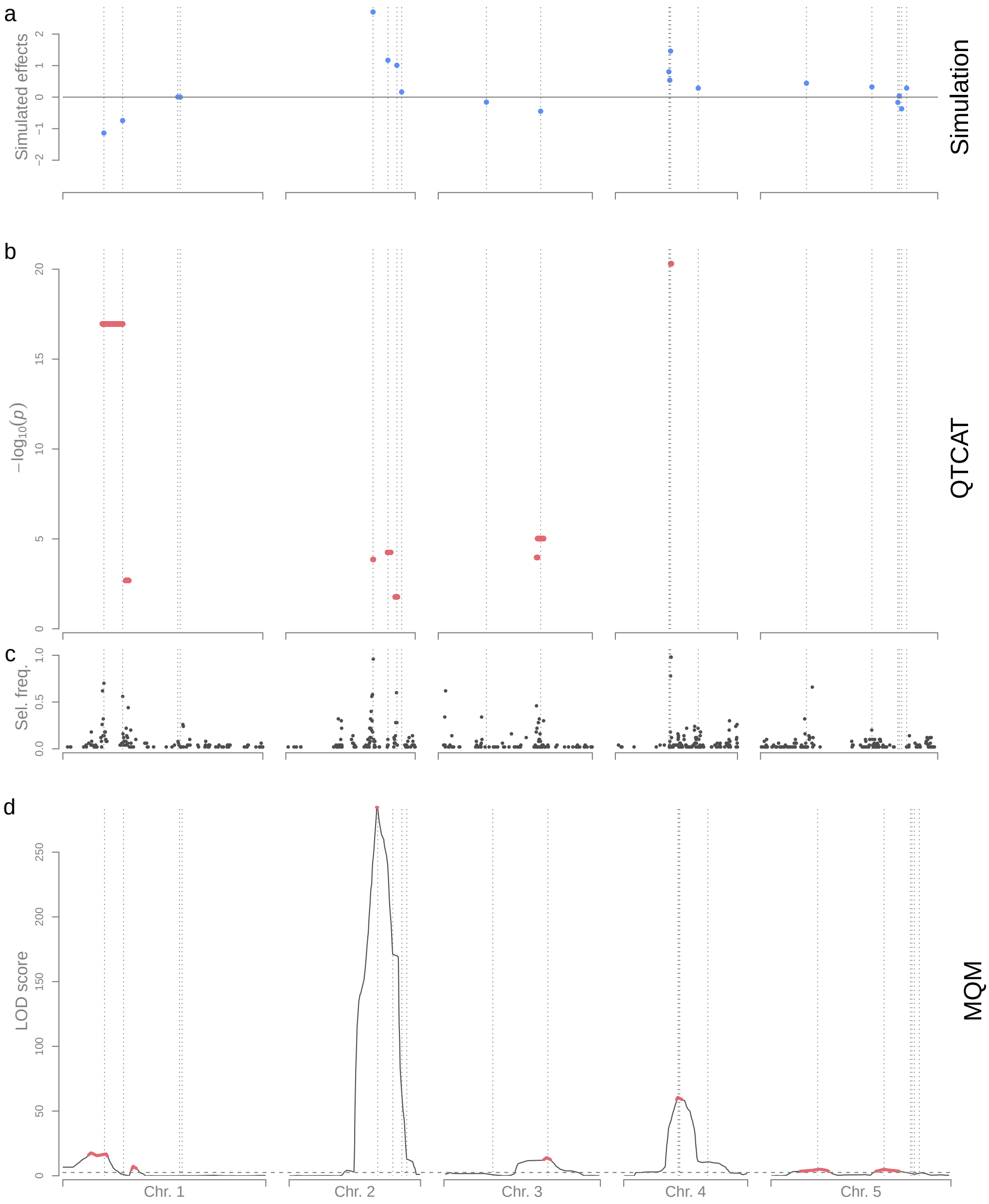

Supplementary Figure 371 Simulation of a GWA analysis based on a unstructured population with a heritability of 0.7 (run 71). (**a**) Simulated of 20 effects randomly drawn from a Gamma distribution and assigned them randomly to markers. Simulated effects randomly drawn from a Gamma distribution. We assigned effects to 20 markers. Markers with an effect are highlighted in (**b**–**d**) with dashed lines. (**b**) Significant QTCs found by QTCAT. (**c**) The selection frequency of the LASSO for each marker during the 50 iterations of QTCAT. (**d**) MQM LOD score plot, the horizontal dashed line is a simulation based perumtation test FDR. The red colored areas represent the LOD-intervals.

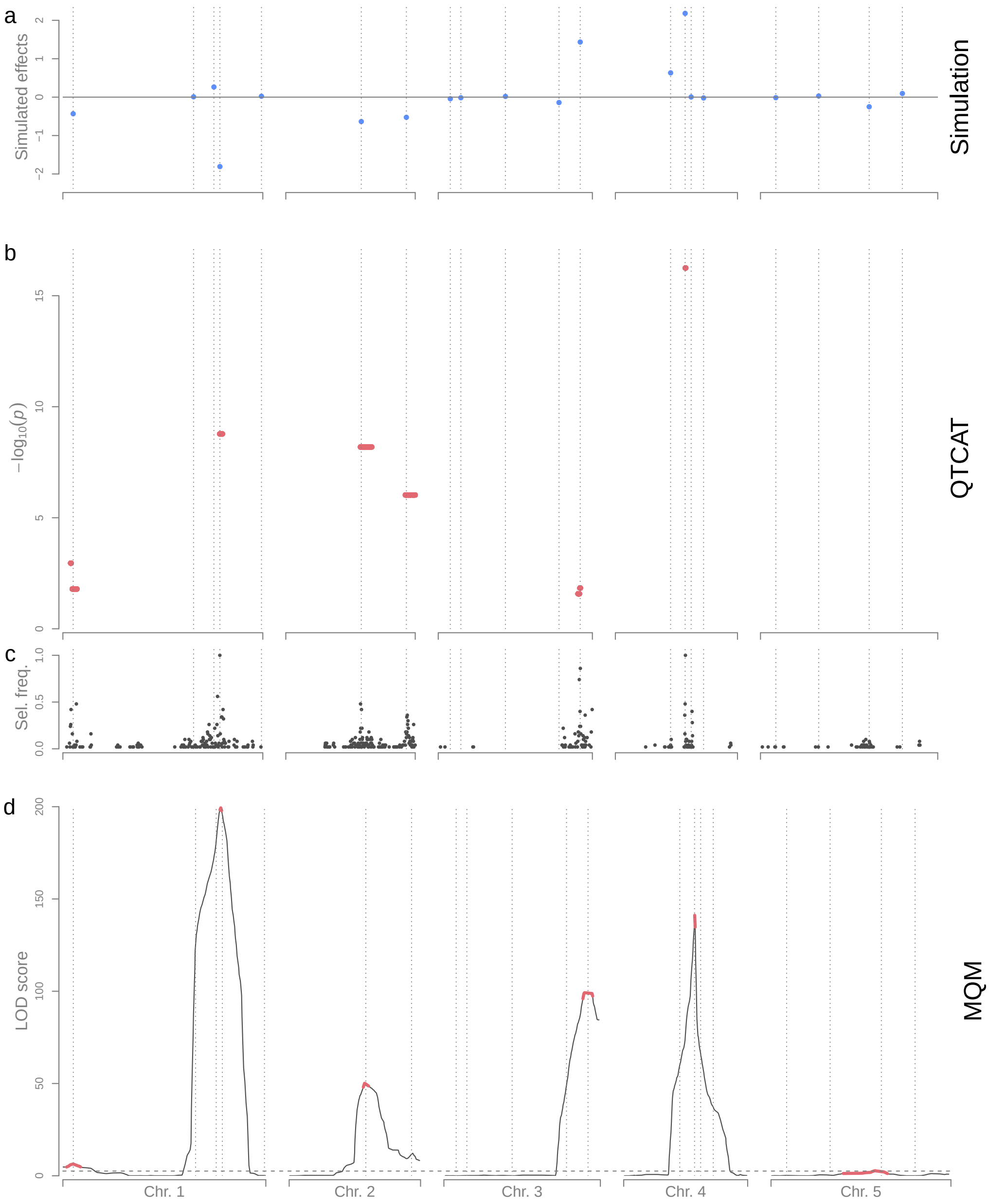

Supplementary Figure 372 Simulation of a GWA analysis based on a unstructured population with a heritability of 0.7 (run 72). (**a**) Simulated of 20 effects randomly drawn from a Gamma distribution and assigned them randomly to markers. Simulated effects randomly drawn from a Gamma distribution. We assigned effects to 20 markers. Markers with an effect are highlighted in (**b–d**) with dashed lines. (**b**) Significant QTCs found by QTCAT. (**c**) The selection frequency of the LASSO for each marker during the 50 iterations of QTCAT. (**d**) MQM LOD score plot, the horizontal dashed line is a simulation based perumtation test FDR. The red colored areas represent the LOD-intervals.

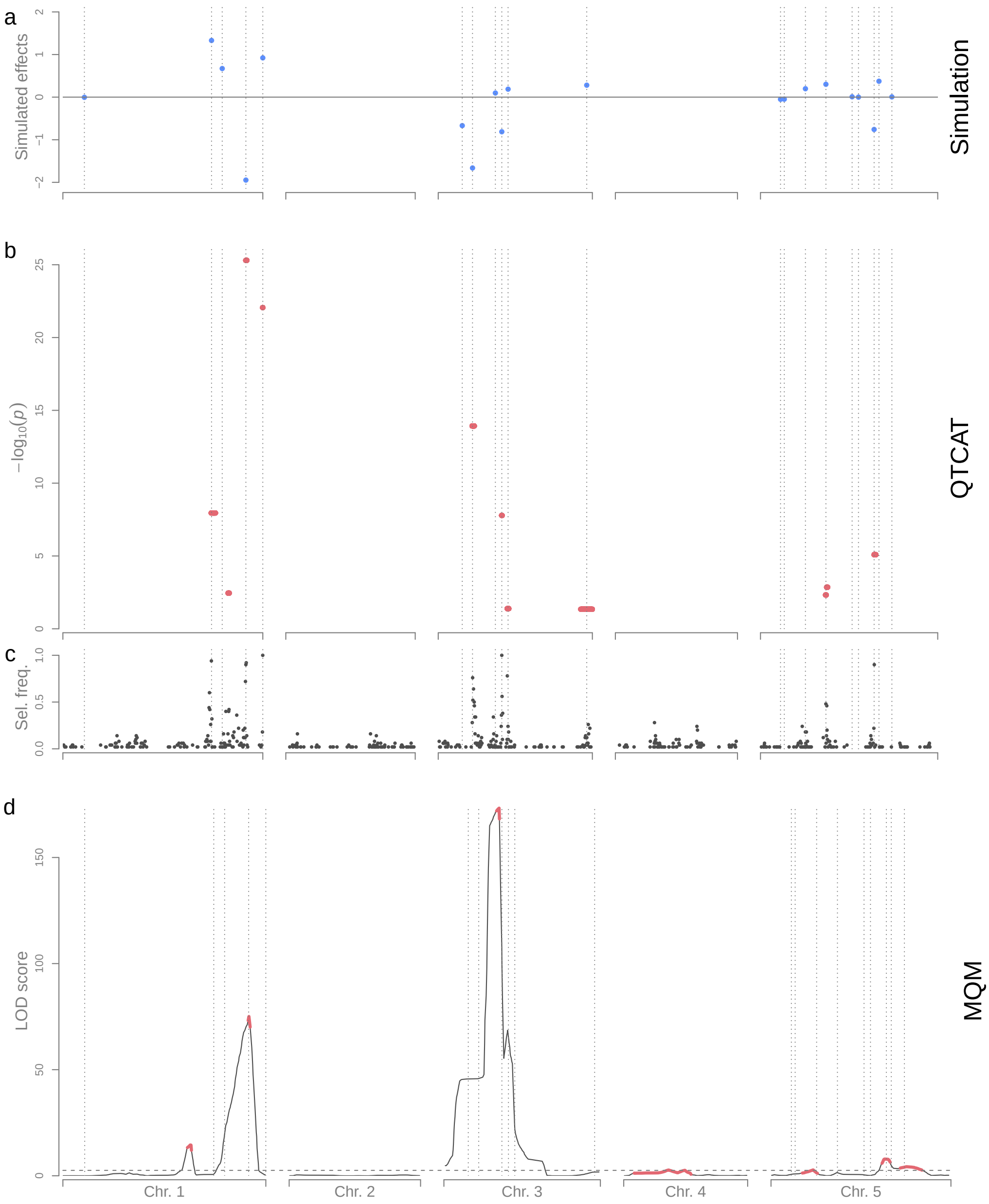

Supplementary Figure 373 Simulation of a GWA analysis based on a unstructured population with a heritability of 0.7 (run 73). (**a**) Simulated of 20 effects randomly drawn from a Gamma distribution and assigned them randomly to markers. Simulated effects randomly drawn from a Gamma distribution. We assigned effects to 20 markers. Markers with an effect are highlighted in (**b**–**d**) with dashed lines. (**b**) Significant QTCs found by QTCAT. (**c**) The selection frequency of the LASSO for each marker during the 50 iterations of QTCAT. (**d**) MQM LOD score plot, the horizontal dashed line is a simulation based perumtation test FDR. The red colored areas represent the LOD-intervals.

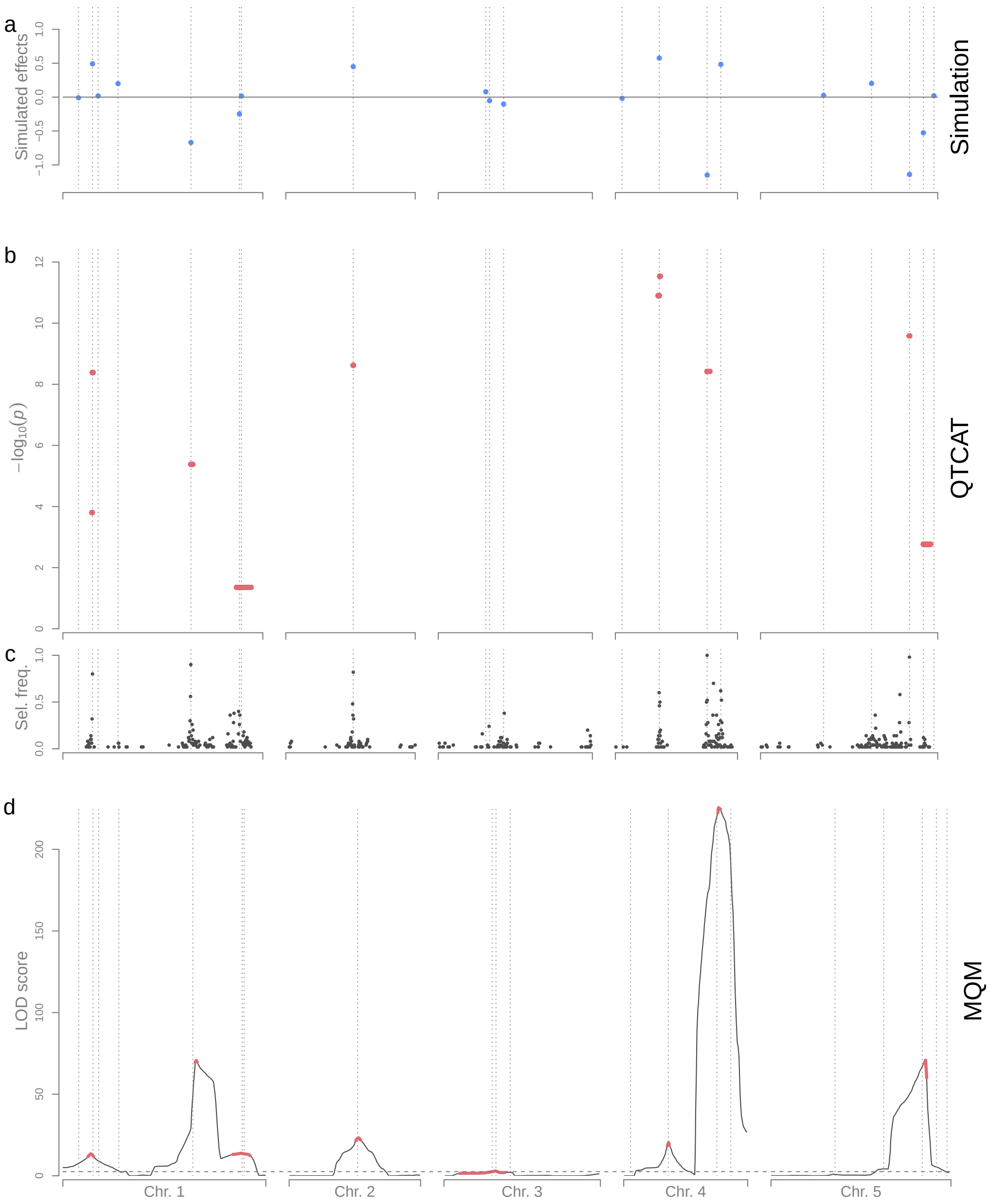

Supplementary Figure 374 Simulation of a GWA analysis based on a unstructured population with a heritability of 0.7 (run 74). (**a**) Simulated of 20 effects randomly drawn from a Gamma distribution and assigned them randomly to markers. Simulated effects randomly drawn from a Gamma distribution. We assigned effects to 20 markers. Markers with an effect are highlighted in (**b**–**d**) with dashed lines. (**b**) Significant QTCs found by QTCAT. (**c**) The selection frequency of the LASSO for each marker during the 50 iterations of QTCAT. (**d**) MQM LOD score plot, the horizontal dashed line is a simulation based perumtation test FDR. The red colored areas represent the LOD-intervals.

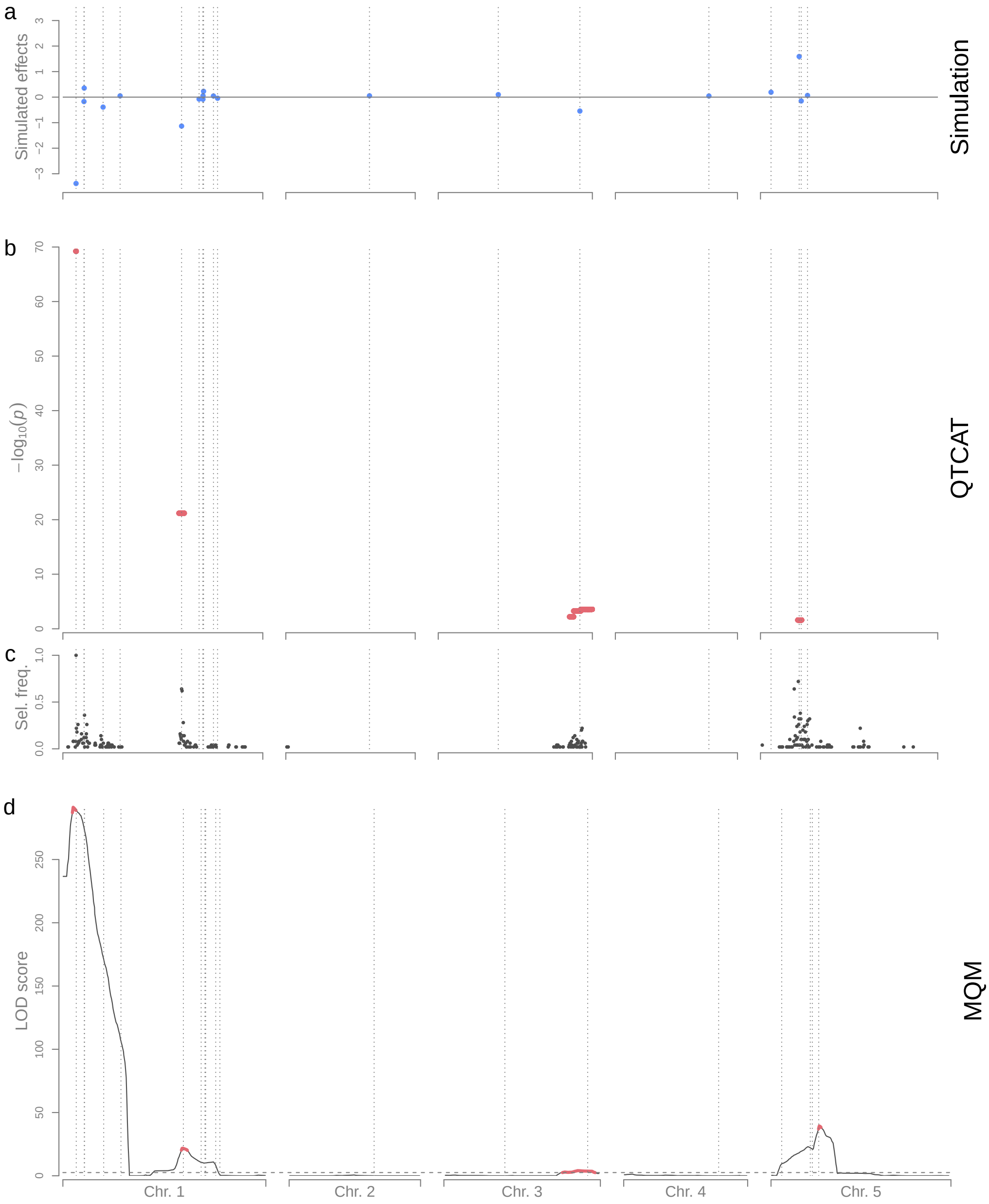

Supplementary Figure 375 Simulation of a GWA analysis based on a unstructured population with a heritability of 0.7 (run 75). (**a**) Simulated of 20 effects randomly drawn from a Gamma distribution and assigned them randomly to markers. Simulated effects randomly drawn from a Gamma distribution. We assigned effects to 20 markers. Markers with an effect are highlighted in (**b–d**) with dashed lines. (**b**) Significant QTCs found by QTCAT. (**c**) The selection frequency of the LASSO for each marker during the 50 iterations of QTCAT. (**d**) MQM LOD score plot, the horizontal dashed line is a simulation based perumtation test FDR. The red colored areas represent the LOD-intervals.

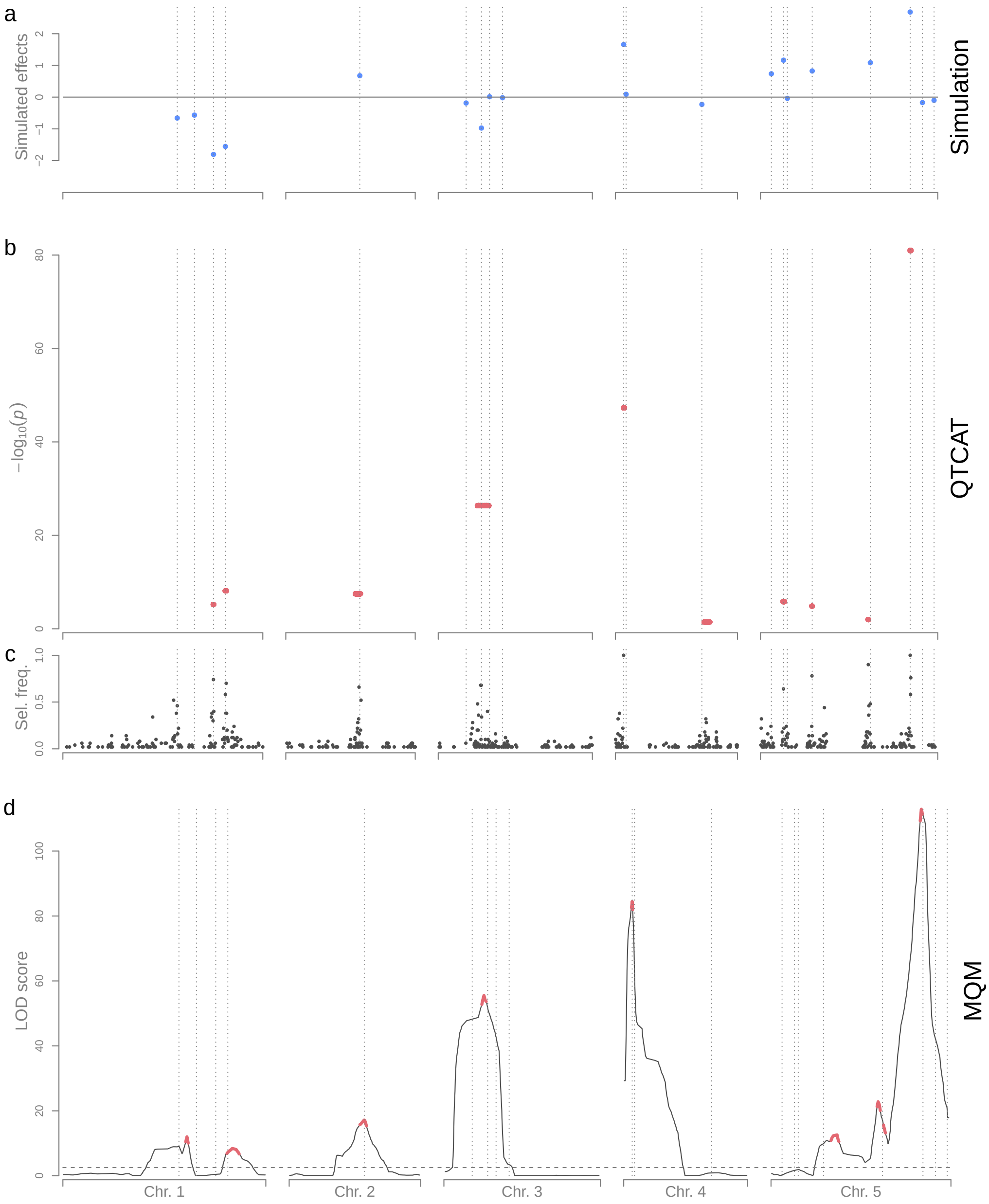

Supplementary Figure 376 Simulation of a GWA analysis based on a unstructured population with a heritability of 0.7 (run 76). (**a**) Simulated of 20 effects randomly drawn from a Gamma distribution and assigned them randomly to markers. Simulated effects randomly drawn from a Gamma distribution. We assigned effects to 20 markers. Markers with an effect are highlighted in (**b–d**) with dashed lines. (**b**) Significant QTCs found by QTCAT. (**c**) The selection frequency of the LASSO for each marker during the 50 iterations of QTCAT. (**d**) MQM LOD score plot, the horizontal dashed line is a simulation based perumtation test FDR. The red colored areas represent the LOD-intervals.

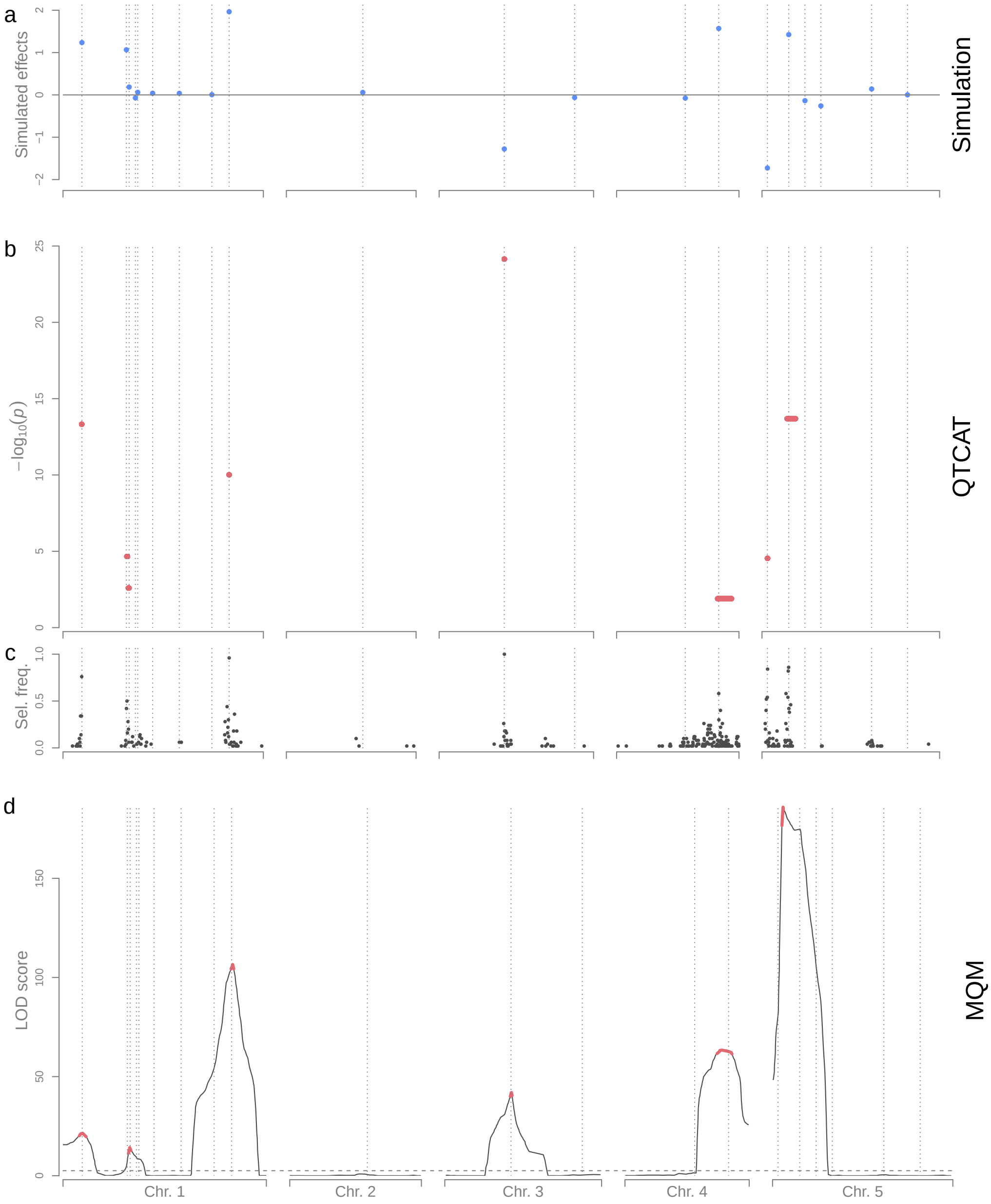

Supplementary Figure 377 Simulation of a GWA analysis based on a unstructured population with a heritability of 0.7 (run 77). (**a**) Simulated of 20 effects randomly drawn from a Gamma distribution and assigned them randomly to markers. Simulated effects randomly drawn from a Gamma distribution. We assigned effects to 20 markers. Markers with an effect are highlighted in (**b–d**) with dashed lines. (**b**) Significant QTCs found by QTCAT. (**c**) The selection frequency of the LASSO for each marker during the 50 iterations of QTCAT. (**d**) MQM LOD score plot, the horizontal dashed line is a simulation based perumtation test FDR. The red colored areas represent the LOD-intervals.

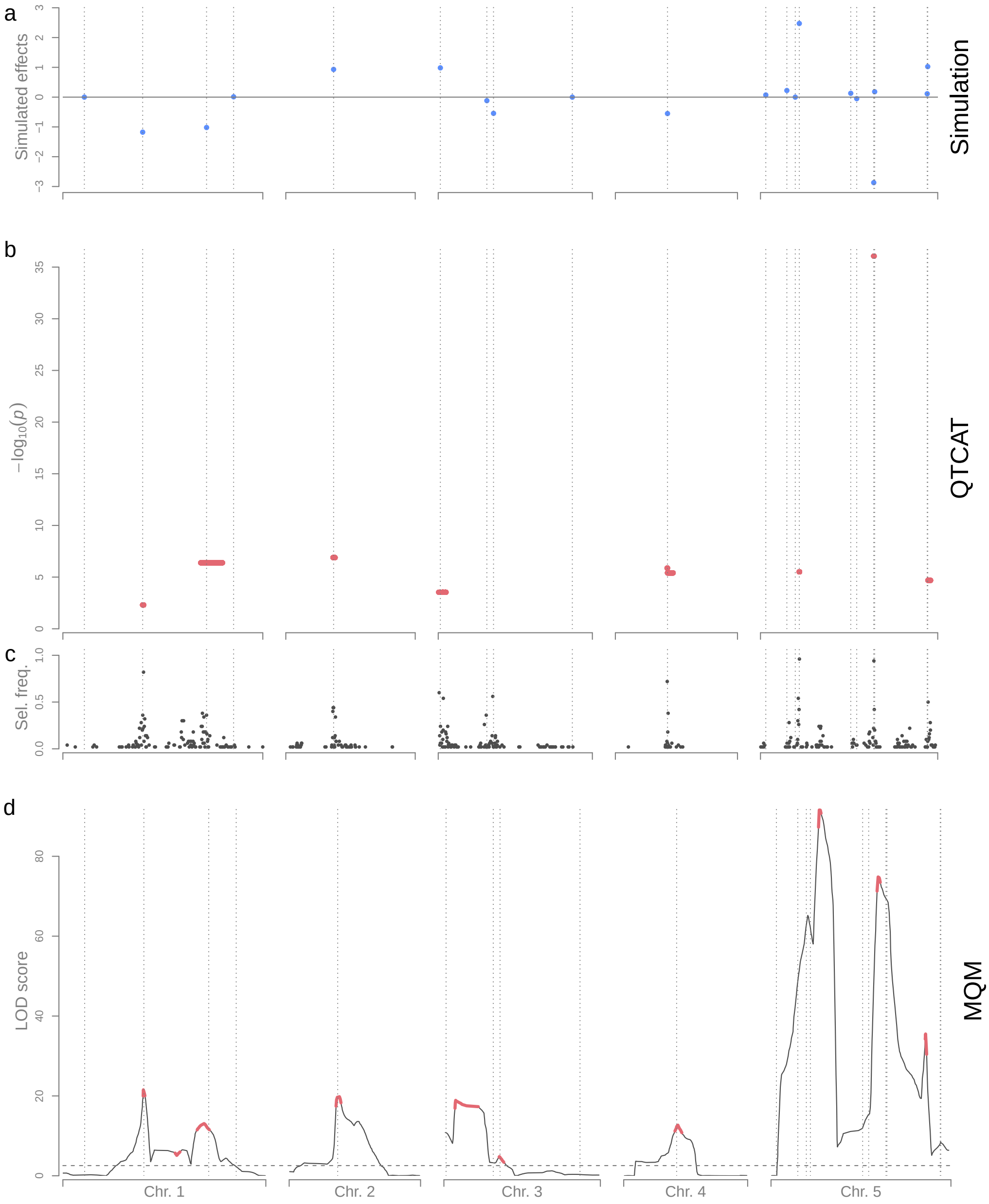

Supplementary Figure 378 Simulation of a GWA analysis based on a unstructured population with a heritability of 0.7 (run 78). (**a**) Simulated of 20 effects randomly drawn from a Gamma distribution and assigned them randomly to markers. Simulated effects randomly drawn from a Gamma distribution. We assigned effects to 20 markers. Markers with an effect are highlighted in (**b–d**) with dashed lines. (**b**) Significant QTCs found by QTCAT. (**c**) The selection frequency of the LASSO for each marker during the 50 iterations of QTCAT. (**d**) MQM LOD score plot, the horizontal dashed line is a simulation based perumtation test FDR. The red colored areas represent the LOD-intervals.

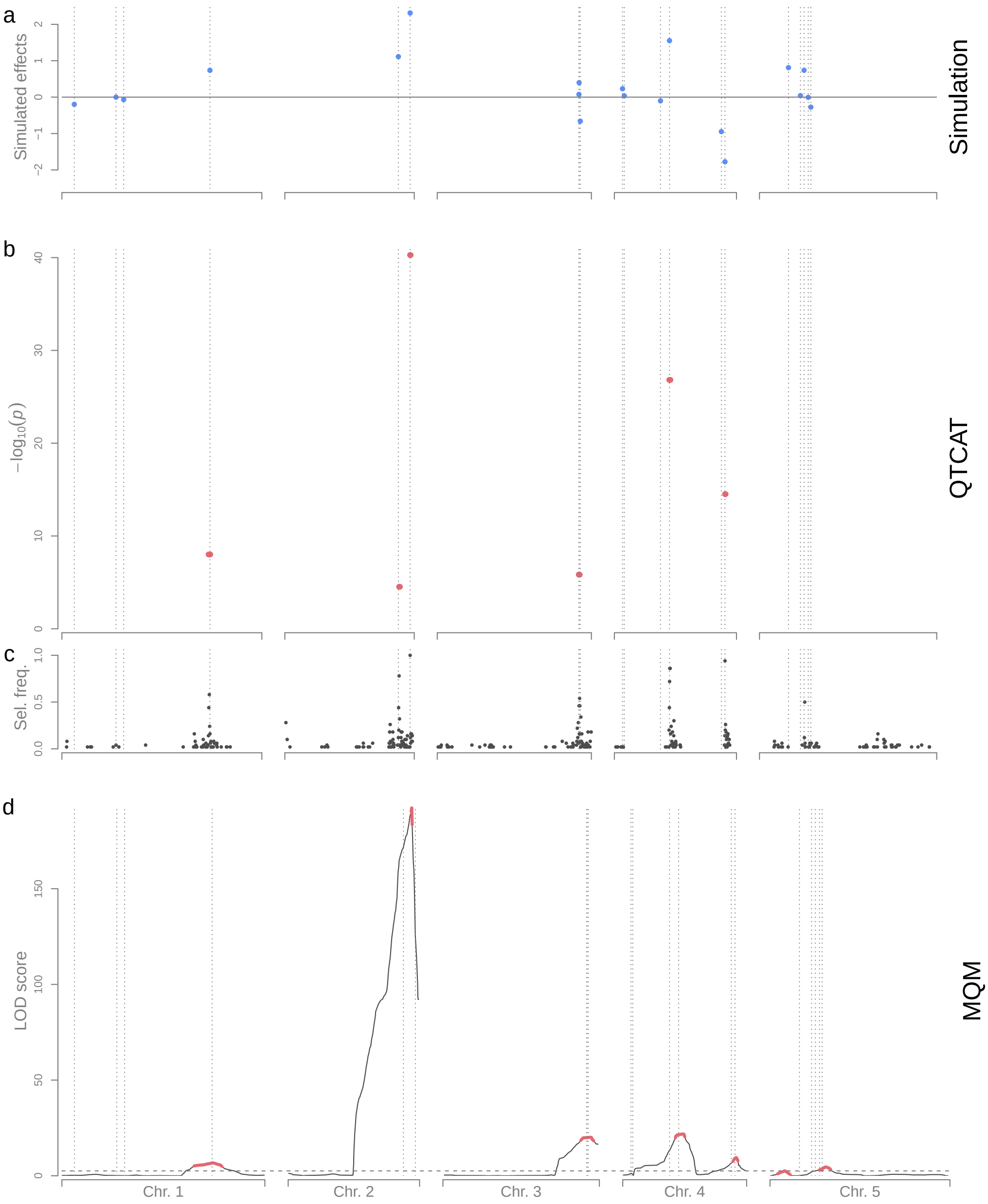

Supplementary Figure 379 Simulation of a GWA analysis based on a unstructured population with a heritability of 0.7 (run 79). (**a**) Simulated of 20 effects randomly drawn from a Gamma distribution and assigned them randomly to markers. Simulated effects randomly drawn from a Gamma distribution. We assigned effects to 20 markers. Markers with an effect are highlighted in (**b**–**d**) with dashed lines. (**b**) Significant QTCs found by QTCAT. (**c**) The selection frequency of the LASSO for each marker during the 50 iterations of QTCAT. (**d**) MQM LOD score plot, the horizontal dashed line is a simulation based perumtation test FDR. The red colored areas represent the LOD-intervals.

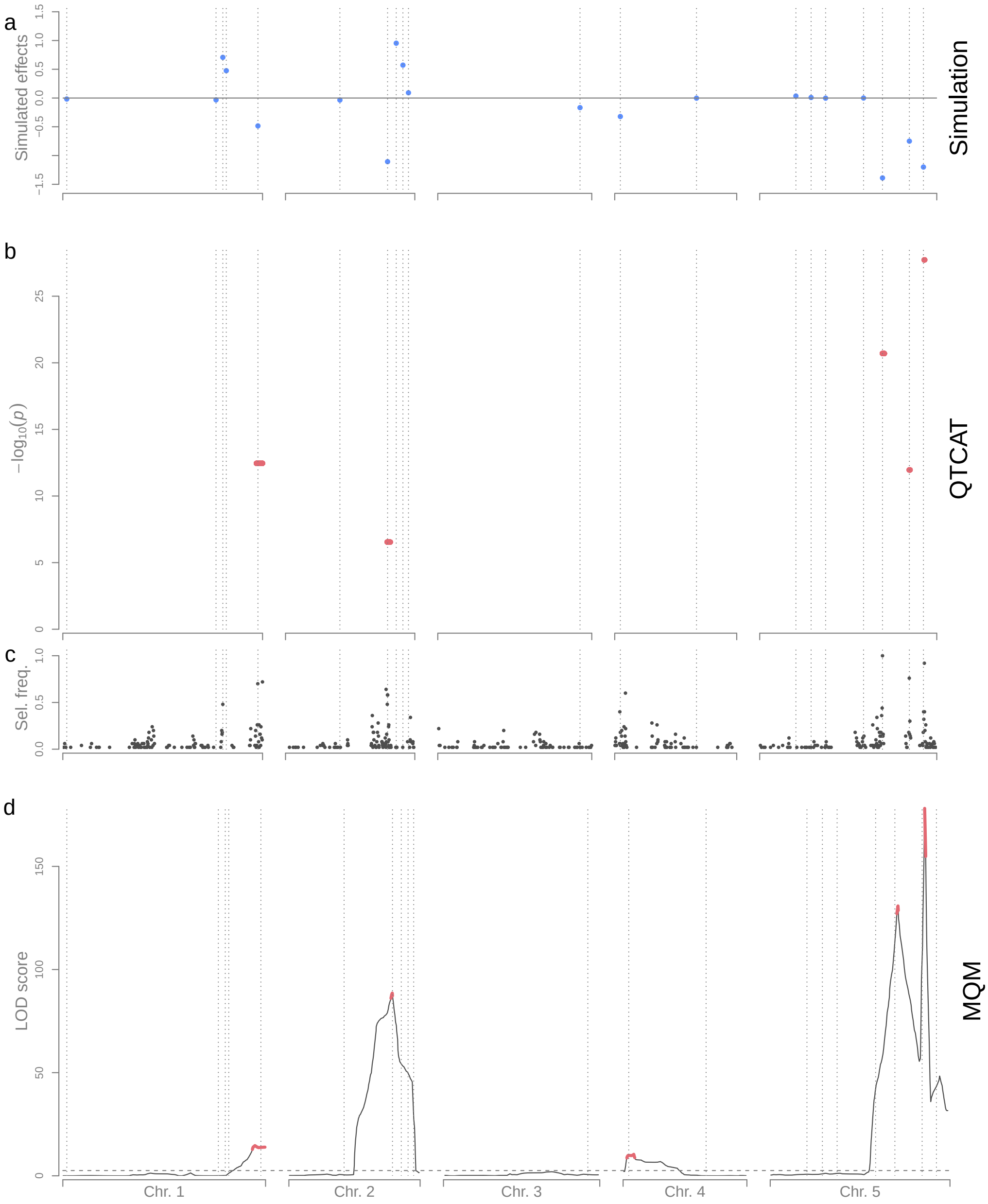

Supplementary Figure 380 Simulation of a GWA analysis based on a unstructured population with a heritability of 0.7 (run 80). (**a**) Simulated of 20 effects randomly drawn from a Gamma distribution and assigned them randomly to markers. Simulated effects randomly drawn from a Gamma distribution. We assigned effects to 20 markers. Markers with an effect are highlighted in (**b–d**) with dashed lines. (**b**) Significant QTCs found by QTCAT. (**c**) The selection frequency of the LASSO for each marker during the 50 iterations of QTCAT. (**d**) MQM LOD score plot, the horizontal dashed line is a simulation based perumtation test FDR. The red colored areas represent the LOD-intervals.

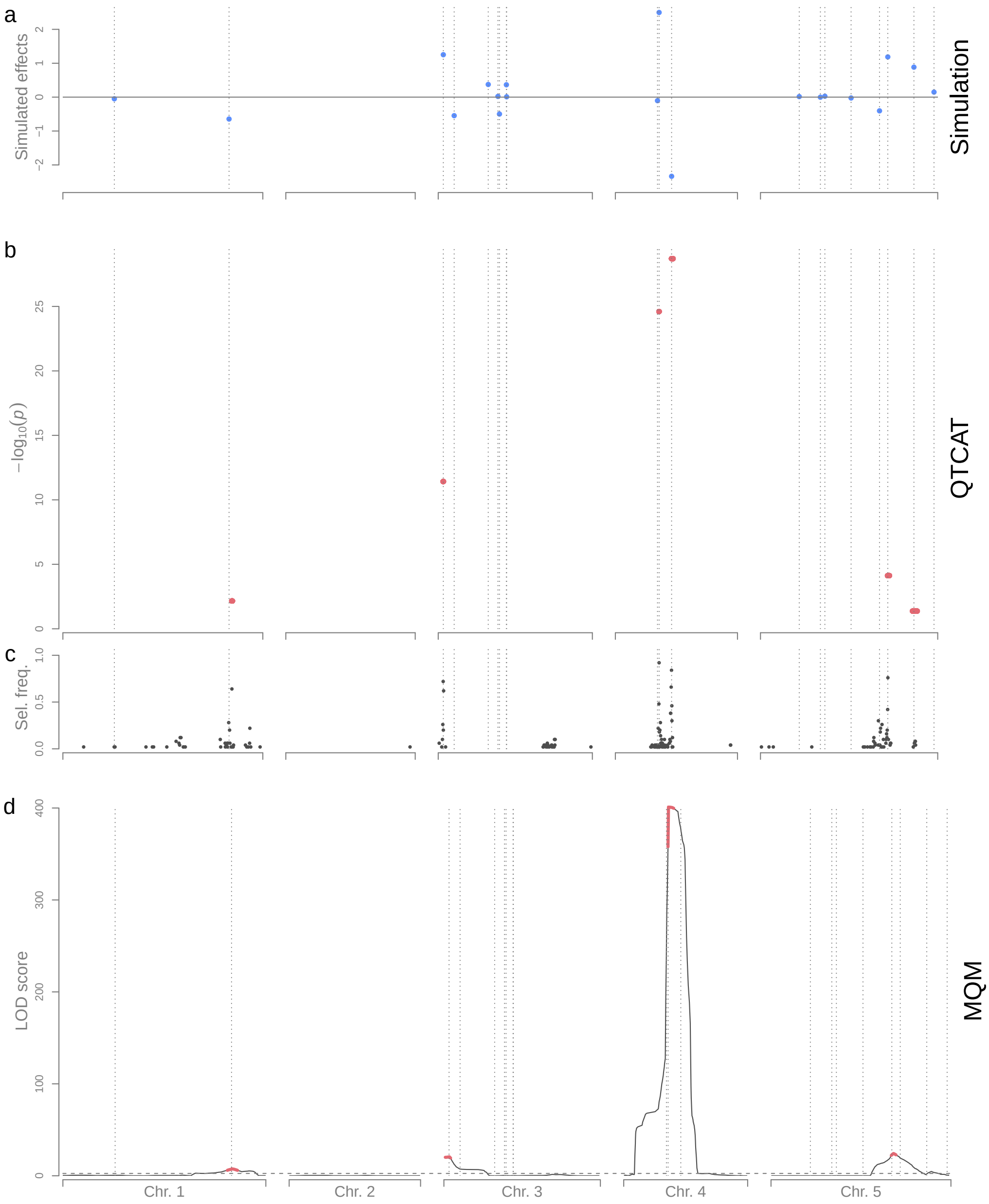

Supplementary Figure 381 Simulation of a GWA analysis based on a unstructured population with a heritability of 0.7 (run 81). (**a**) Simulated of 20 effects randomly drawn from a Gamma distribution and assigned them randomly to markers. Simulated effects randomly drawn from a Gamma distribution. We assigned effects to 20 markers. Markers with an effect are highlighted in (**b**–**d**) with dashed lines. (**b**) Significant QTCs found by QTCAT. (**c**) The selection frequency of the LASSO for each marker during the 50 iterations of QTCAT. (**d**) MQM LOD score plot, the horizontal dashed line is a simulation based perumtation test FDR. The red colored areas represent the LOD-intervals.

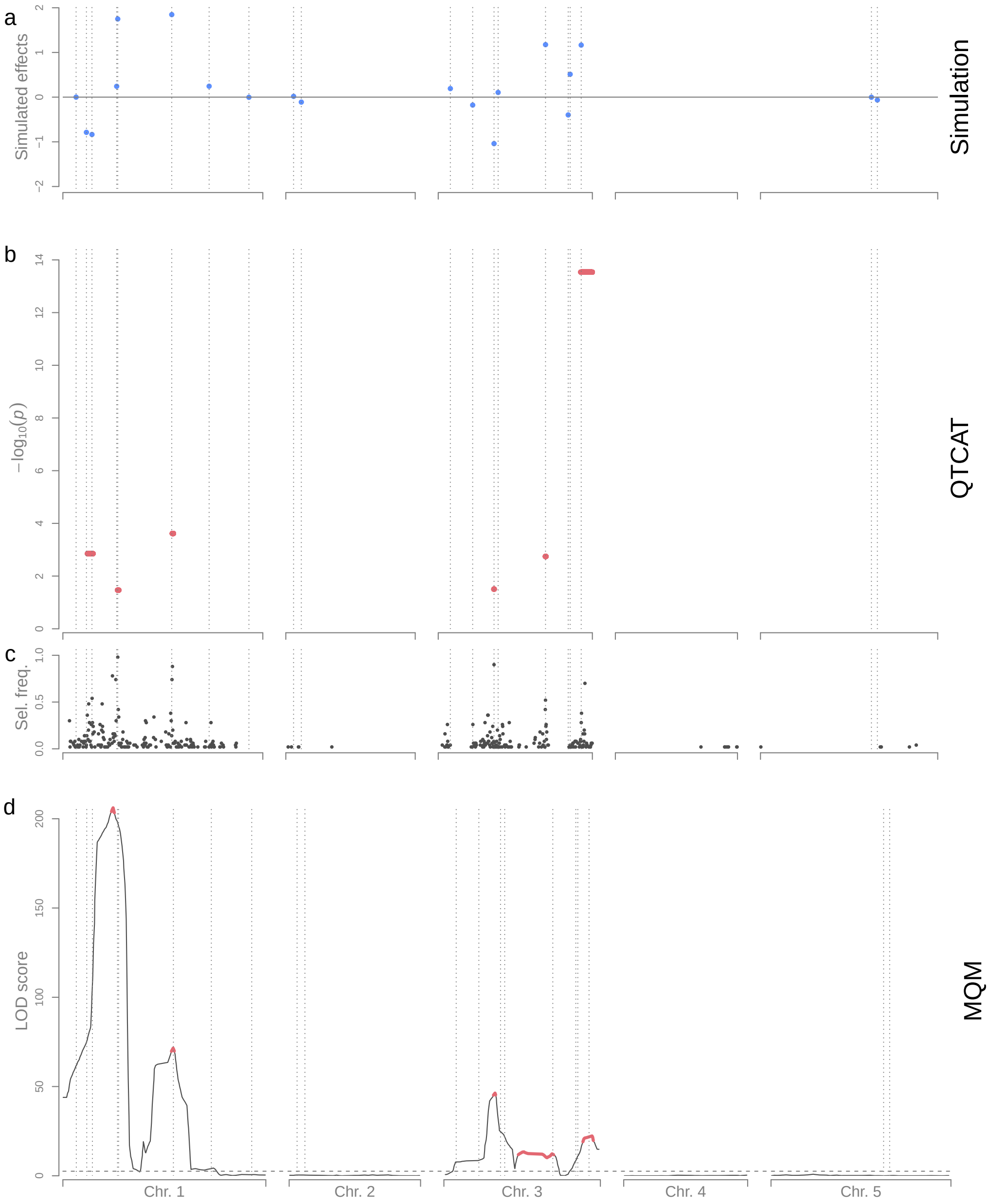

Supplementary Figure 382 Simulation of a GWA analysis based on a unstructured population with a heritability of 0.7 (run 82). (**a**) Simulated of 20 effects randomly drawn from a Gamma distribution and assigned them randomly to markers. Simulated effects randomly drawn from a Gamma distribution. We assigned effects to 20 markers. Markers with an effect are highlighted in (**b–d**) with dashed lines. (**b**) Significant QTCs found by QTCAT. (**c**) The selection frequency of the LASSO for each marker during the 50 iterations of QTCAT. (**d**) MQM LOD score plot, the horizontal dashed line is a simulation based perumtation test FDR. The red colored areas represent the LOD-intervals.

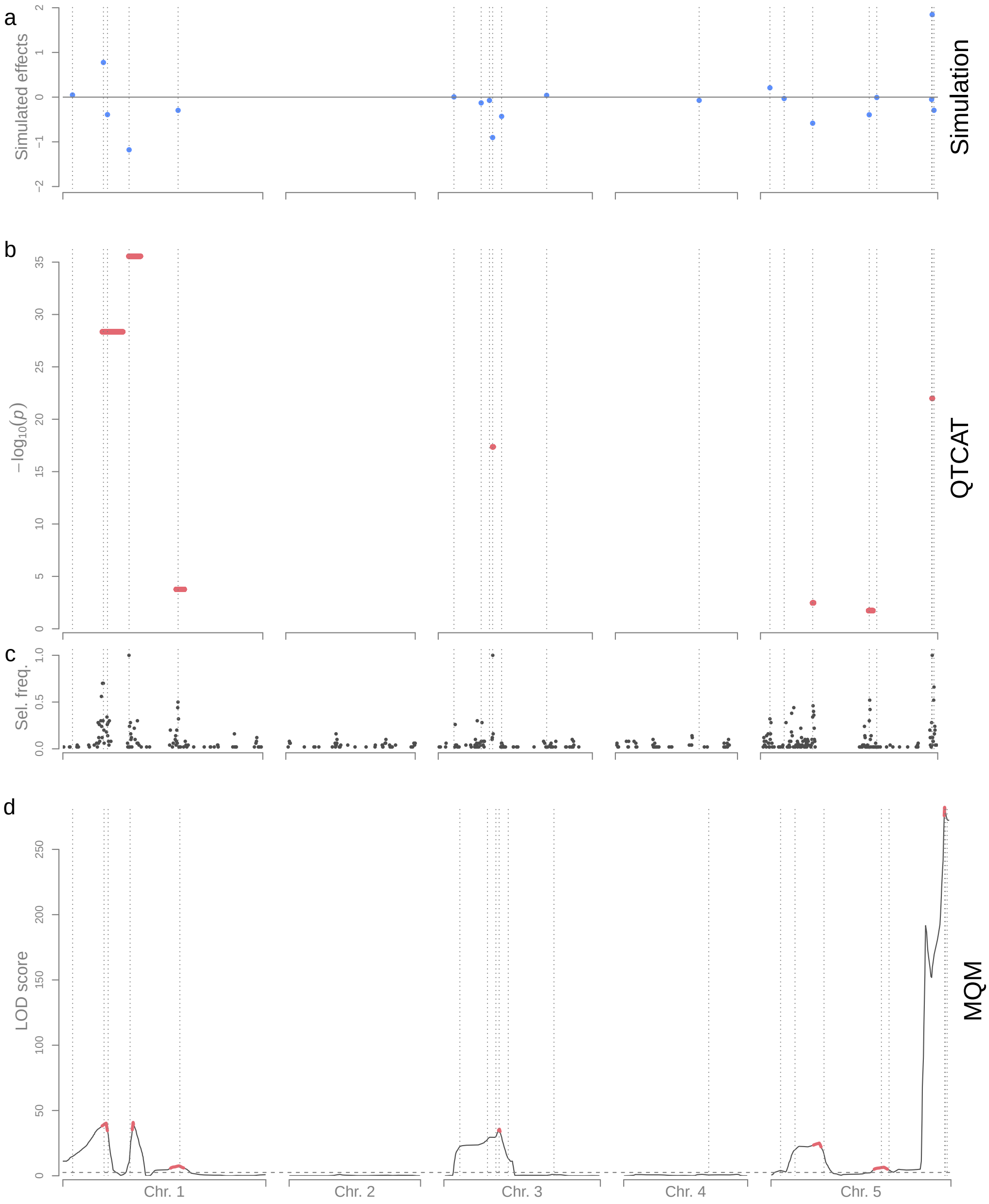

Supplementary Figure 383 Simulation of a GWA analysis based on a unstructured population with a heritability of 0.7 (run 83). (**a**) Simulated of 20 effects randomly drawn from a Gamma distribution and assigned them randomly to markers. Simulated effects randomly drawn from a Gamma distribution. We assigned effects to 20 markers. Markers with an effect are highlighted in (**b**–**d**) with dashed lines. (**b**) Significant QTCs found by QTCAT. (**c**) The selection frequency of the LASSO for each marker during the 50 iterations of QTCAT. (**d**) MQM LOD score plot, the horizontal dashed line is a simulation based perumtation test FDR. The red colored areas represent the LOD-intervals.

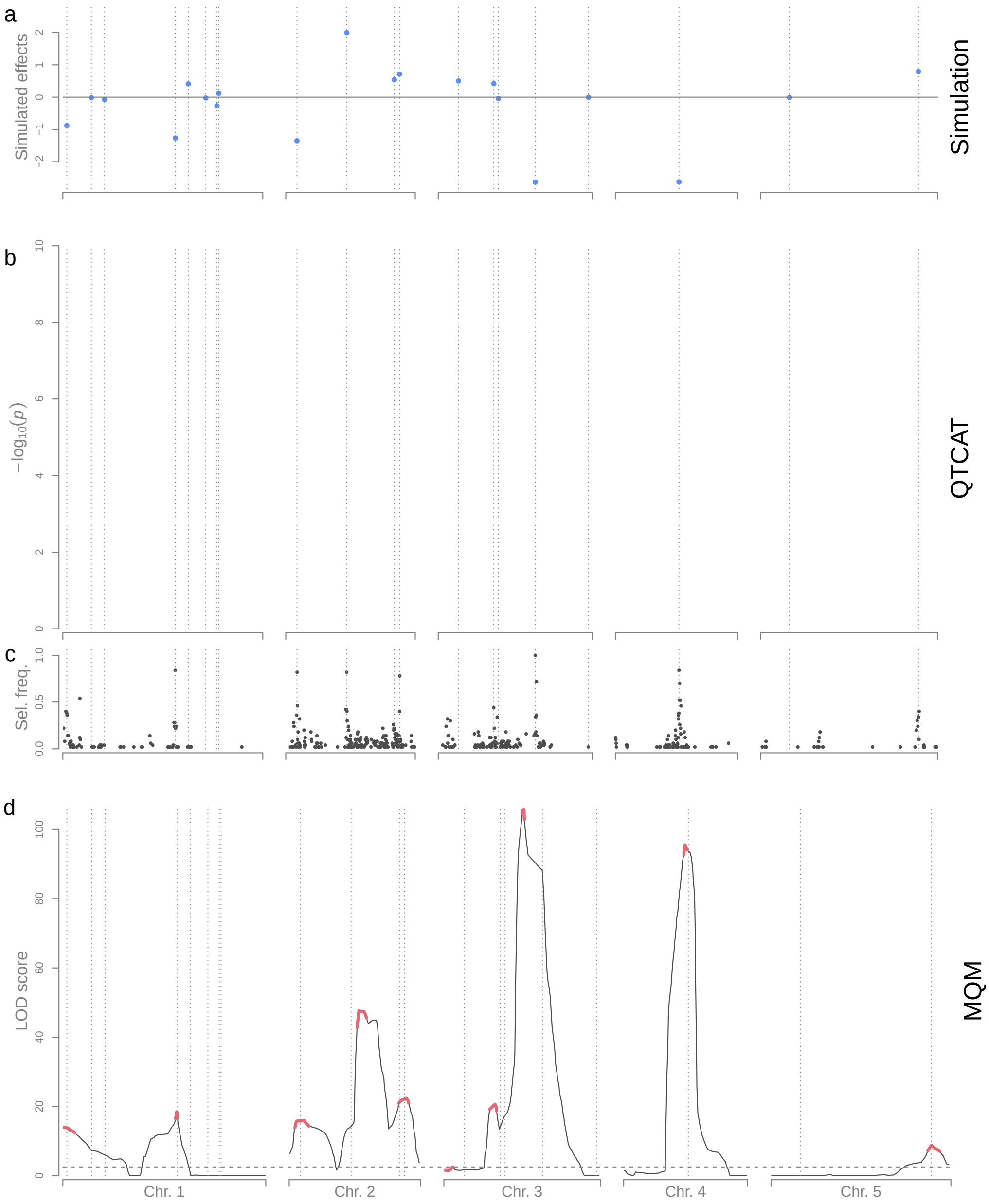

Supplementary Figure 384 Simulation of a GWA analysis based on a unstructured population with a heritability of 0.7 (run 84). (**a**) Simulated of 20 effects randomly drawn from a Gamma distribution and assigned them randomly to markers. Simulated effects randomly drawn from a Gamma distribution. We assigned effects to 20 markers. Markers with an effect are highlighted in (**b–d**) with dashed lines. (**b**) Significant QTCs found by QTCAT. (**c**) The selection frequency of the LASSO for each marker during the 50 iterations of QTCAT. (**d**) MQM LOD score plot, the horizontal dashed line is a simulation based perumtation test FDR. The red colored areas represent the LOD-intervals.

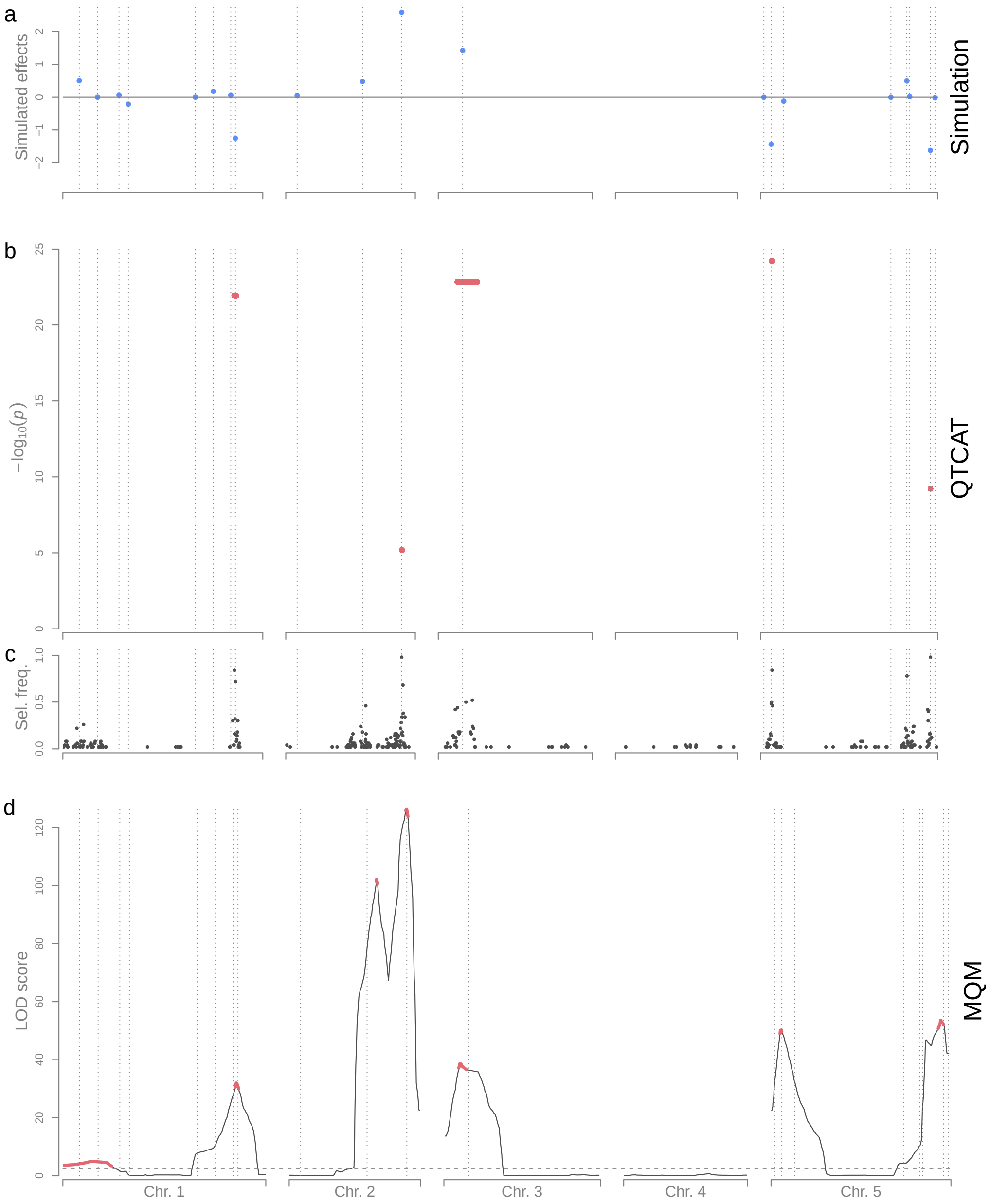

Supplementary Figure 385 Simulation of a GWA analysis based on a unstructured population with a heritability of 0.7 (run 85). (**a**) Simulated of 20 effects randomly drawn from a Gamma distribution and assigned them randomly to markers. Simulated effects randomly drawn from a Gamma distribution. We assigned effects to 20 markers. Markers with an effect are highlighted in (**b**–**d**) with dashed lines. (**b**) Significant QTCs found by QTCAT. (**c**) The selection frequency of the LASSO for each marker during the 50 iterations of QTCAT. (**d**) MQM LOD score plot, the horizontal dashed line is a simulation based perumtation test FDR. The red colored areas represent the LOD-intervals.

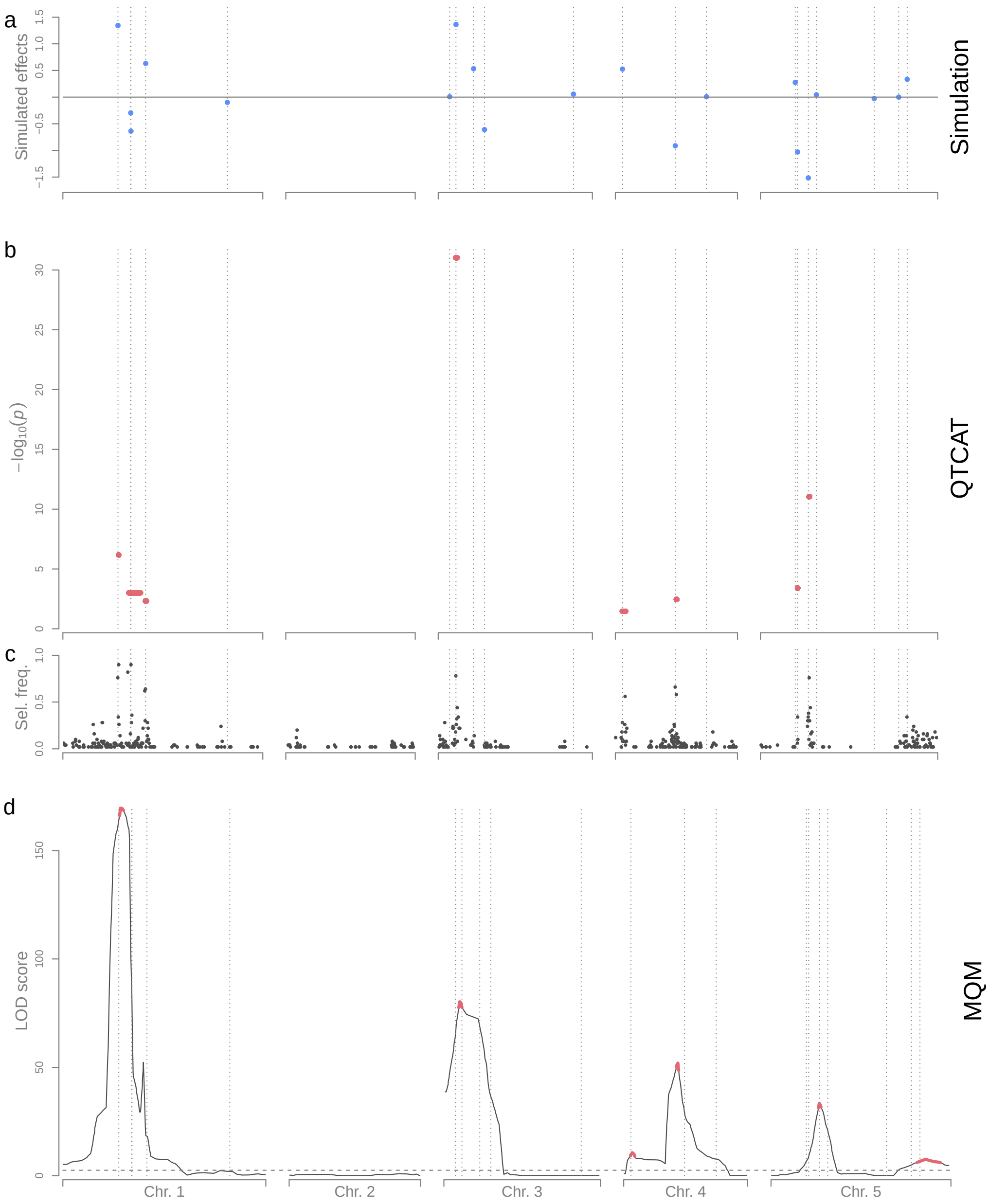

Supplementary Figure 386 Simulation of a GWA analysis based on a unstructured population with a heritability of 0.7 (run 86). (**a**) Simulated of 20 effects randomly drawn from a Gamma distribution and assigned them randomly to markers. Simulated effects randomly drawn from a Gamma distribution. We assigned effects to 20 markers. Markers with an effect are highlighted in (**b–d**) with dashed lines. (**b**) Significant QTCs found by QTCAT. (**c**) The selection frequency of the LASSO for each marker during the 50 iterations of QTCAT. (**d**) MQM LOD score plot, the horizontal dashed line is a simulation based perumtation test FDR. The red colored areas represent the LOD-intervals.

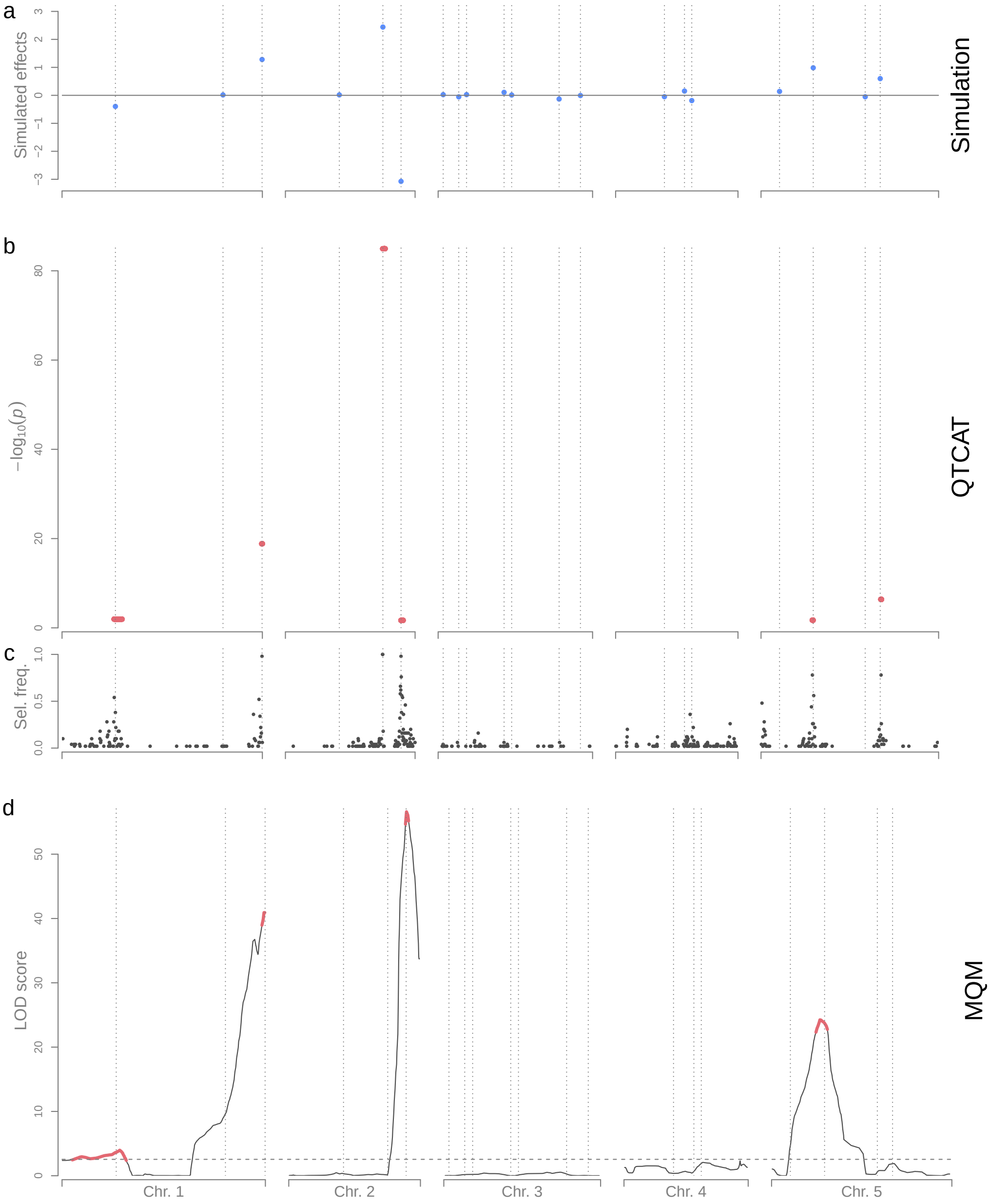

Supplementary Figure 387 Simulation of a GWA analysis based on a unstructured population with a heritability of 0.7 (run 87). (**a**) Simulated of 20 effects randomly drawn from a Gamma distribution and assigned them randomly to markers. Simulated effects randomly drawn from a Gamma distribution. We assigned effects to 20 markers. Markers with an effect are highlighted in (**b–d**) with dashed lines. (**b**) Significant QTCs found by QTCAT. (**c**) The selection frequency of the LASSO for each marker during the 50 iterations of QTCAT. (**d**) MQM LOD score plot, the horizontal dashed line is a simulation based perumtation test FDR. The red colored areas represent the LOD-intervals.

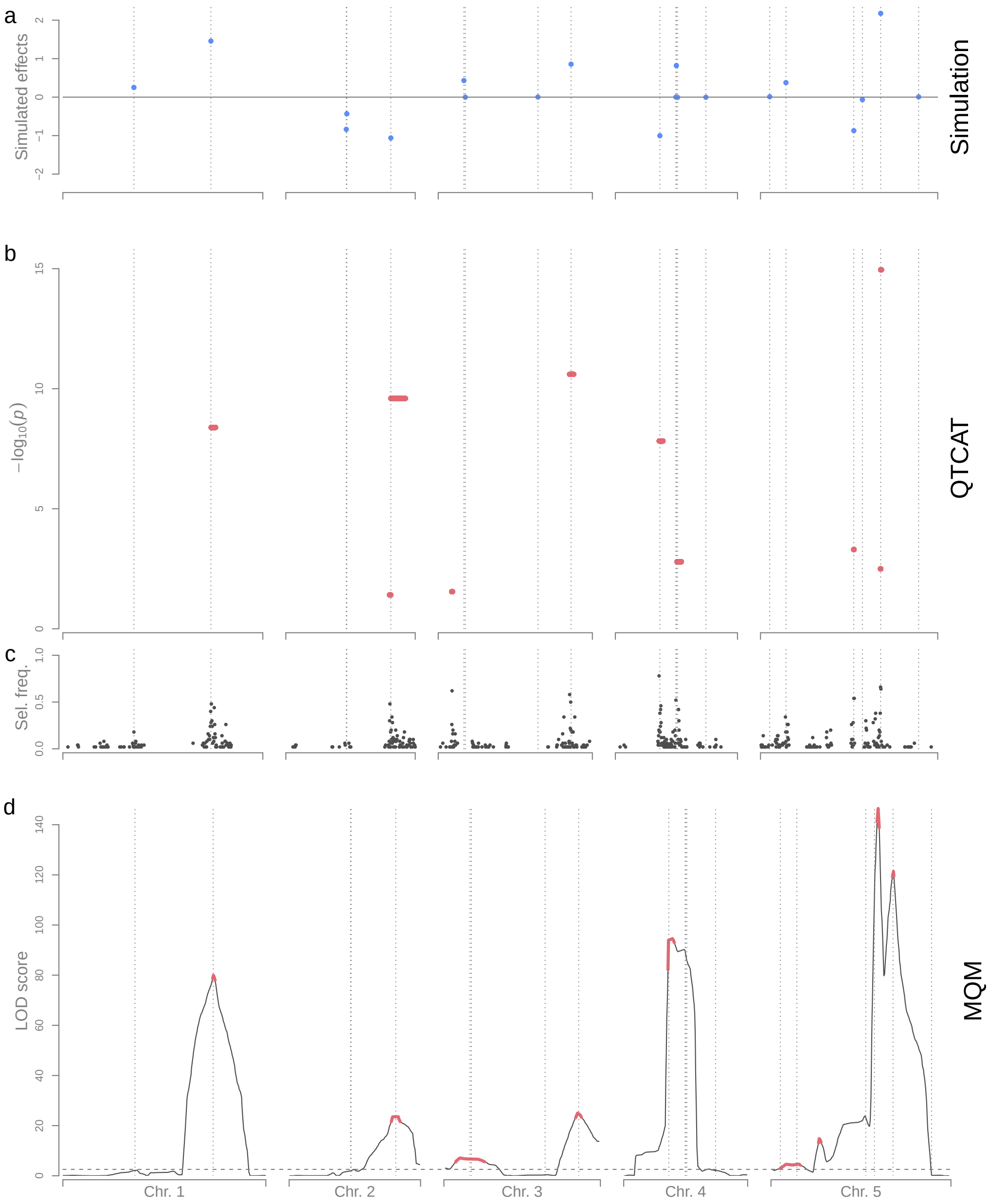

Supplementary Figure 388 Simulation of a GWA analysis based on a unstructured population with a heritability of 0.7 (run 88). (**a**) Simulated of 20 effects randomly drawn from a Gamma distribution and assigned them randomly to markers. Simulated effects randomly drawn from a Gamma distribution. We assigned effects to 20 markers. Markers with an effect are highlighted in (**b–d**) with dashed lines. (**b**) Significant QTCs found by QTCAT. (**c**) The selection frequency of the LASSO for each marker during the 50 iterations of QTCAT. (**d**) MQM LOD score plot, the horizontal dashed line is a simulation based perumtation test FDR. The red colored areas represent the LOD-intervals.

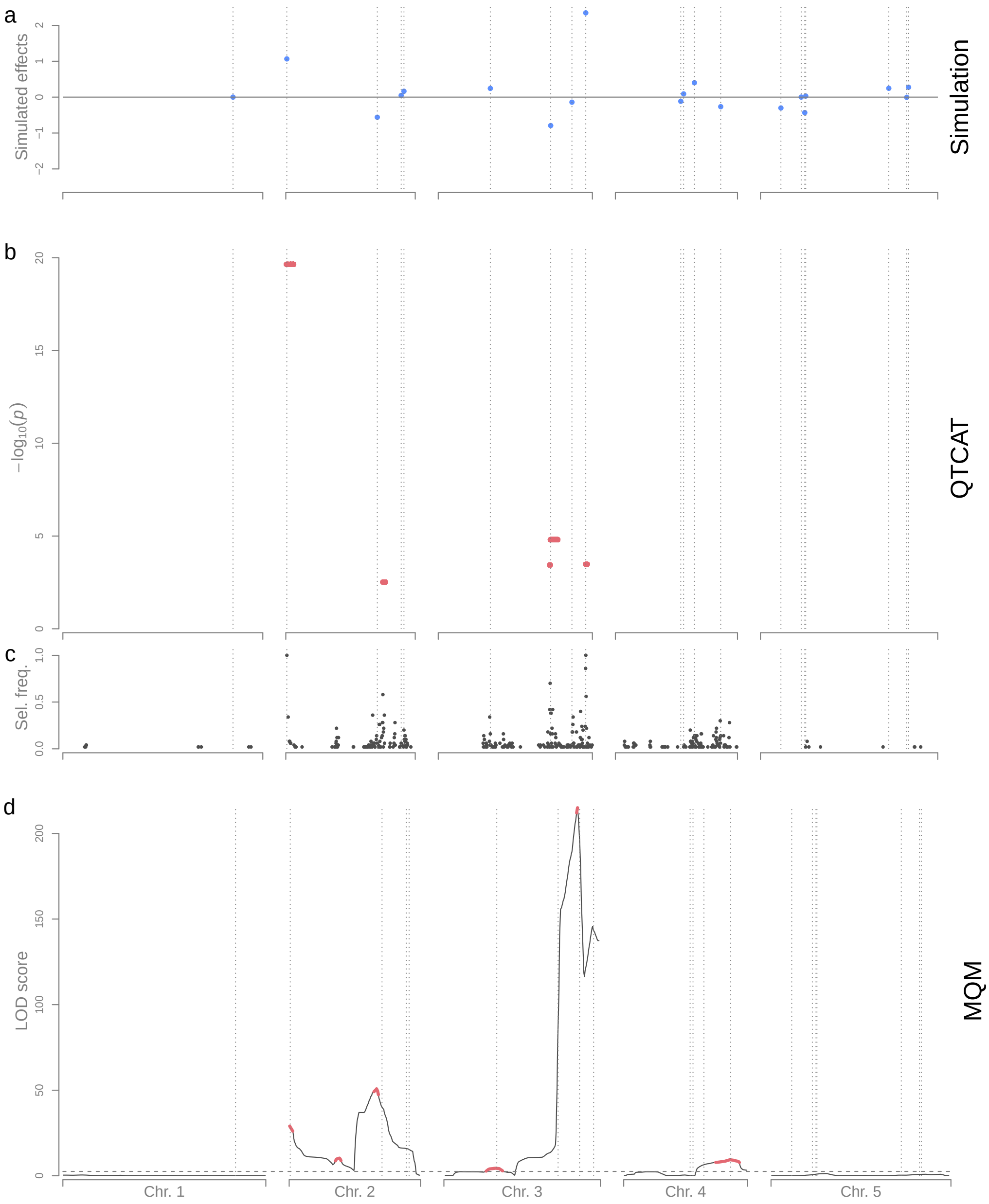

Supplementary Figure 389 Simulation of a GWA analysis based on a unstructured population with a heritability of 0.7 (run 89). (**a**) Simulated of 20 effects randomly drawn from a Gamma distribution and assigned them randomly to markers. Simulated effects randomly drawn from a Gamma distribution. We assigned effects to 20 markers. Markers with an effect are highlighted in (**b**–**d**) with dashed lines. (**b**) Significant QTCs found by QTCAT. (**c**) The selection frequency of the LASSO for each marker during the 50 iterations of QTCAT. (**d**) MQM LOD score plot, the horizontal dashed line is a simulation based perumtation test FDR. The red colored areas represent the LOD-intervals.

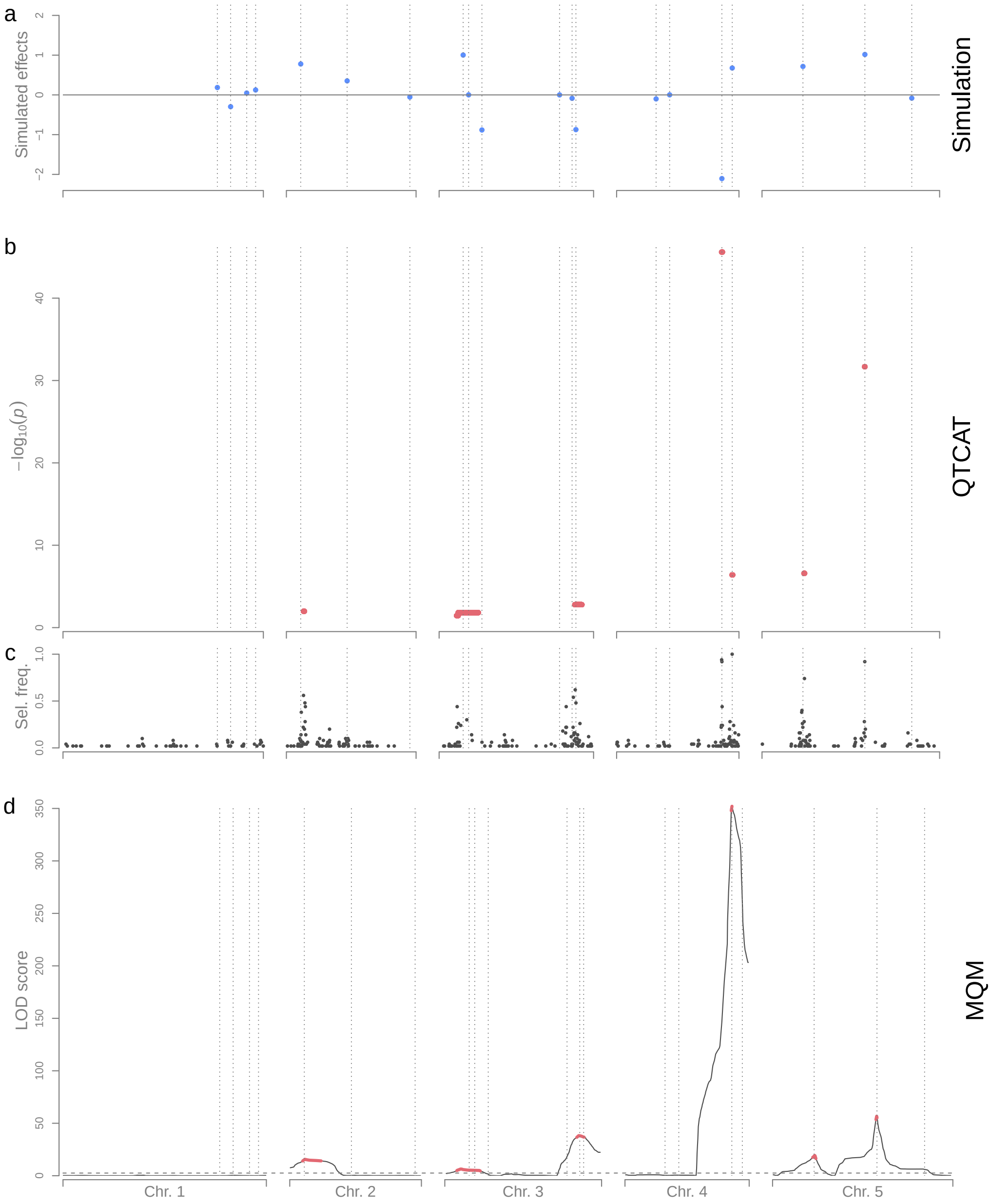

Supplementary Figure 390 Simulation of a GWA analysis based on a unstructured population with a heritability of 0.7 (run 90). (**a**) Simulated of 20 effects randomly drawn from a Gamma distribution and assigned them randomly to markers. Simulated effects randomly drawn from a Gamma distribution. We assigned effects to 20 markers. Markers with an effect are highlighted in (**b–d**) with dashed lines. (**b**) Significant QTCs found by QTCAT. (**c**) The selection frequency of the LASSO for each marker during the 50 iterations of QTCAT. (**d**) MQM LOD score plot, the horizontal dashed line is a simulation based perumtation test FDR. The red colored areas represent the LOD-intervals.

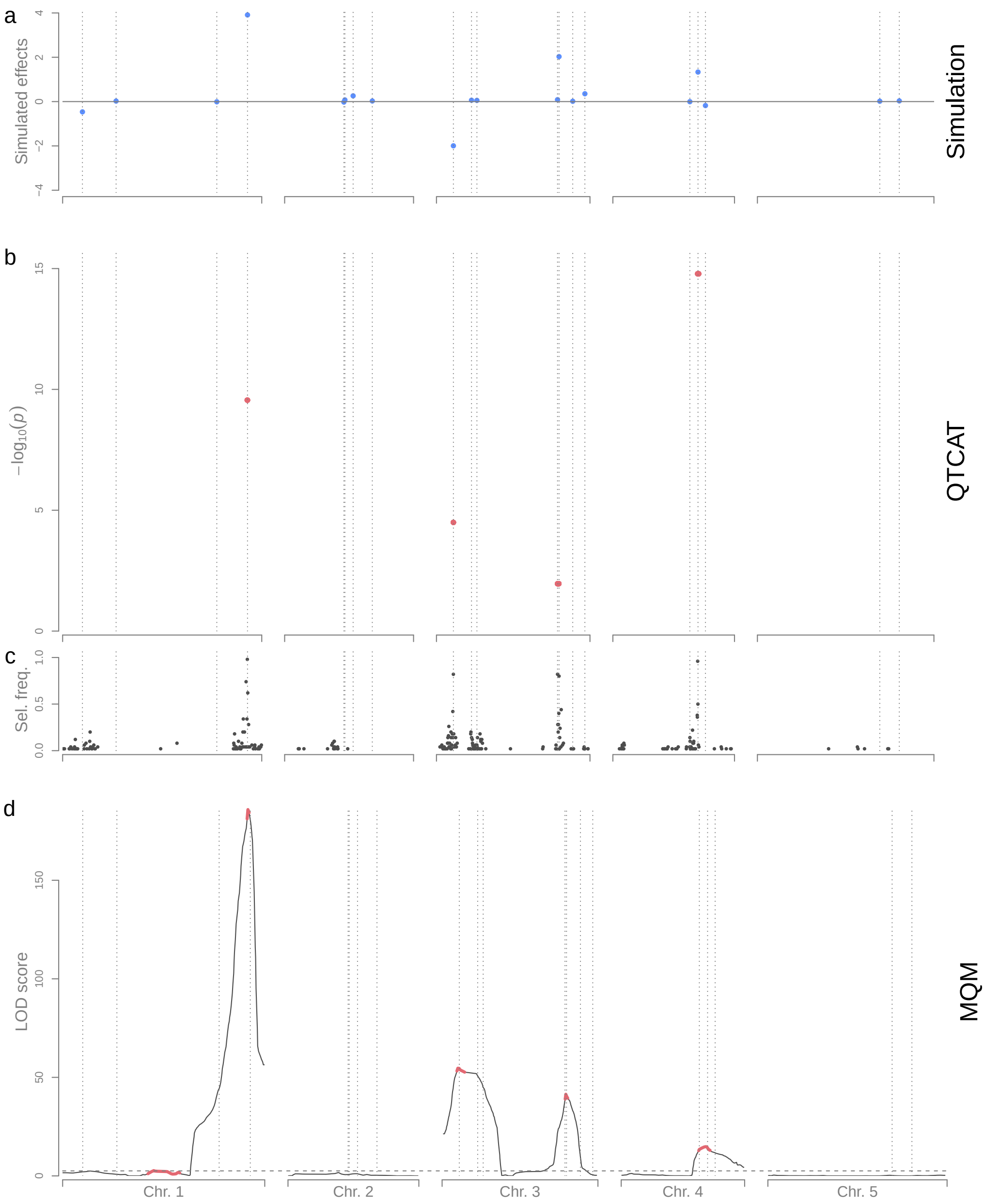

Supplementary Figure 391 Simulation of a GWA analysis based on a unstructured population with a heritability of 0.7 (run 91). (**a**) Simulated of 20 effects randomly drawn from a Gamma distribution and assigned them randomly to markers. Simulated effects randomly drawn from a Gamma distribution. We assigned effects to 20 markers. Markers with an effect are highlighted in (**b–d**) with dashed lines. (**b**) Significant QTCs found by QTCAT. (**c**) The selection frequency of the LASSO for each marker during the 50 iterations of QTCAT. (**d**) MQM LOD score plot, the horizontal dashed line is a simulation based perumtation test FDR. The red colored areas represent the LOD-intervals.

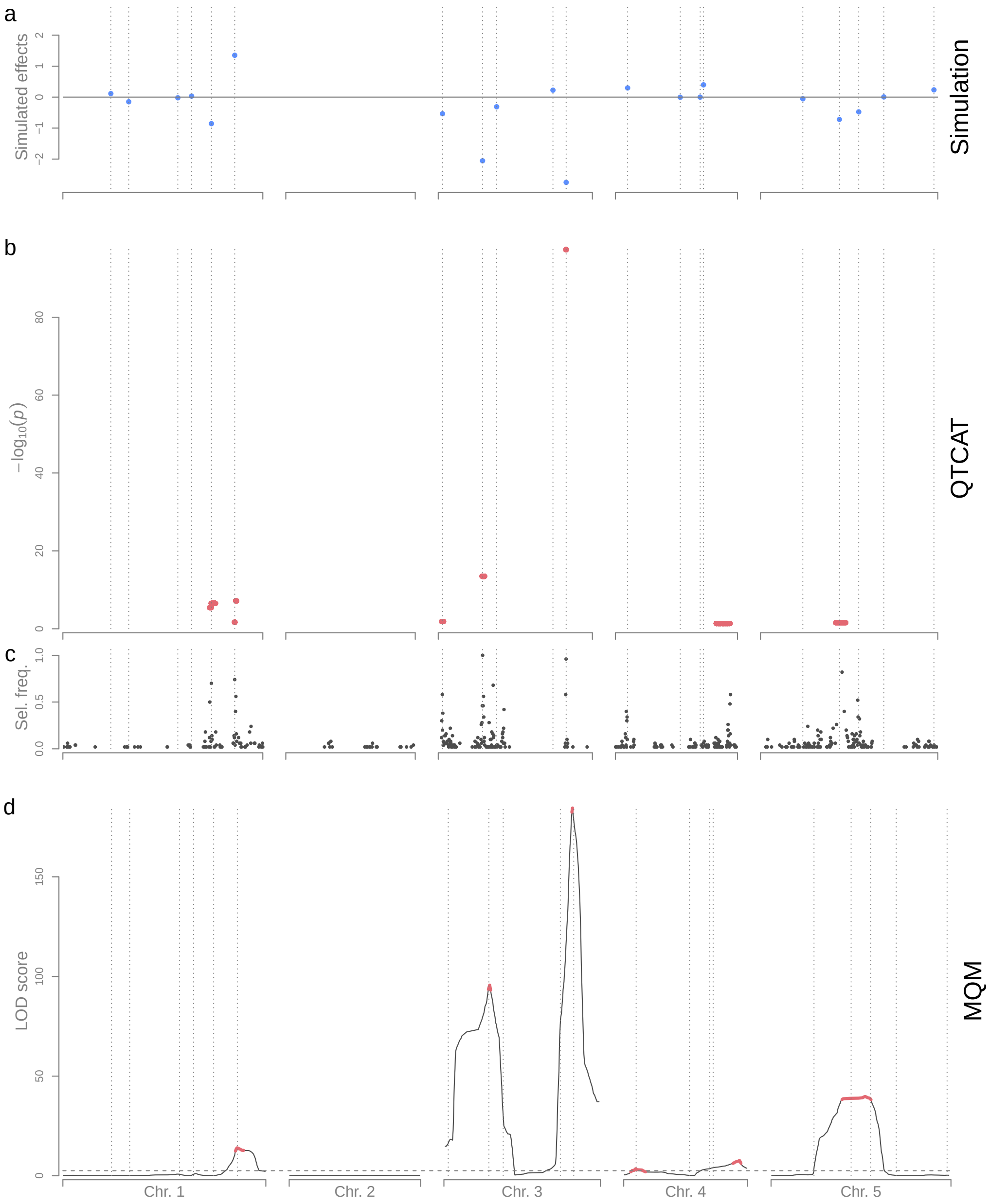

Supplementary Figure 392 Simulation of a GWA analysis based on a unstructured population with a heritability of 0.7 (run 92). (**a**) Simulated of 20 effects randomly drawn from a Gamma distribution and assigned them randomly to markers. Simulated effects randomly drawn from a Gamma distribution. We assigned effects to 20 markers. Markers with an effect are highlighted in (**b**–**d**) with dashed lines. (**b**) Significant QTCs found by QTCAT. (**c**) The selection frequency of the LASSO for each marker during the 50 iterations of QTCAT. (**d**) MQM LOD score plot, the horizontal dashed line is a simulation based perumtation test FDR. The red colored areas represent the LOD-intervals.

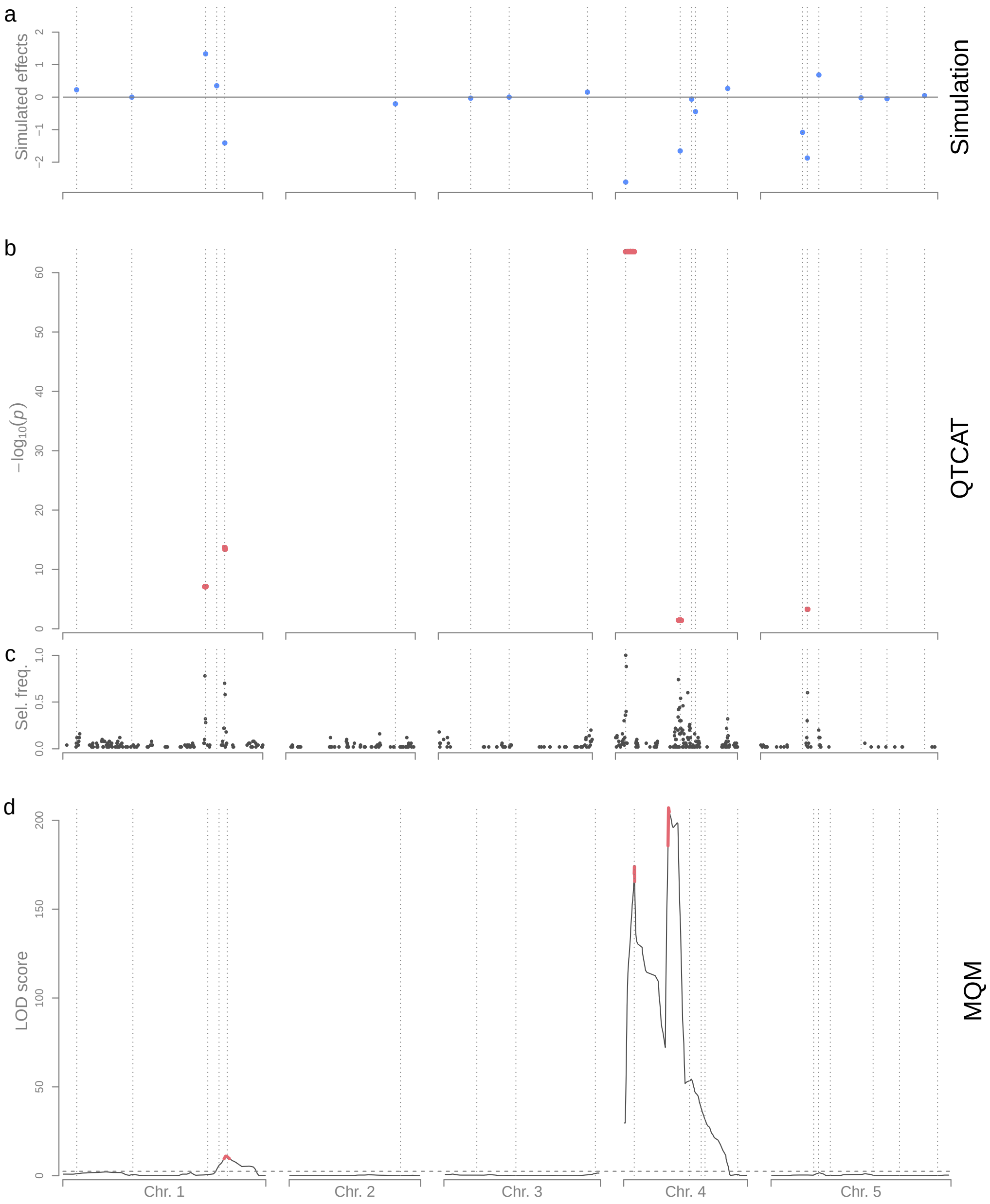

Supplementary Figure 393 Simulation of a GWA analysis based on a unstructured population with a heritability of 0.7 (run 93). (**a**) Simulated of 20 effects randomly drawn from a Gamma distribution and assigned them randomly to markers. Simulated effects randomly drawn from a Gamma distribution. We assigned effects to 20 markers. Markers with an effect are highlighted in (**b–d**) with dashed lines. (**b**) Significant QTCs found by QTCAT. (**c**) The selection frequency of the LASSO for each marker during the 50 iterations of QTCAT. (**d**) MQM LOD score plot, the horizontal dashed line is a simulation based perumtation test FDR. The red colored areas represent the LOD-intervals.

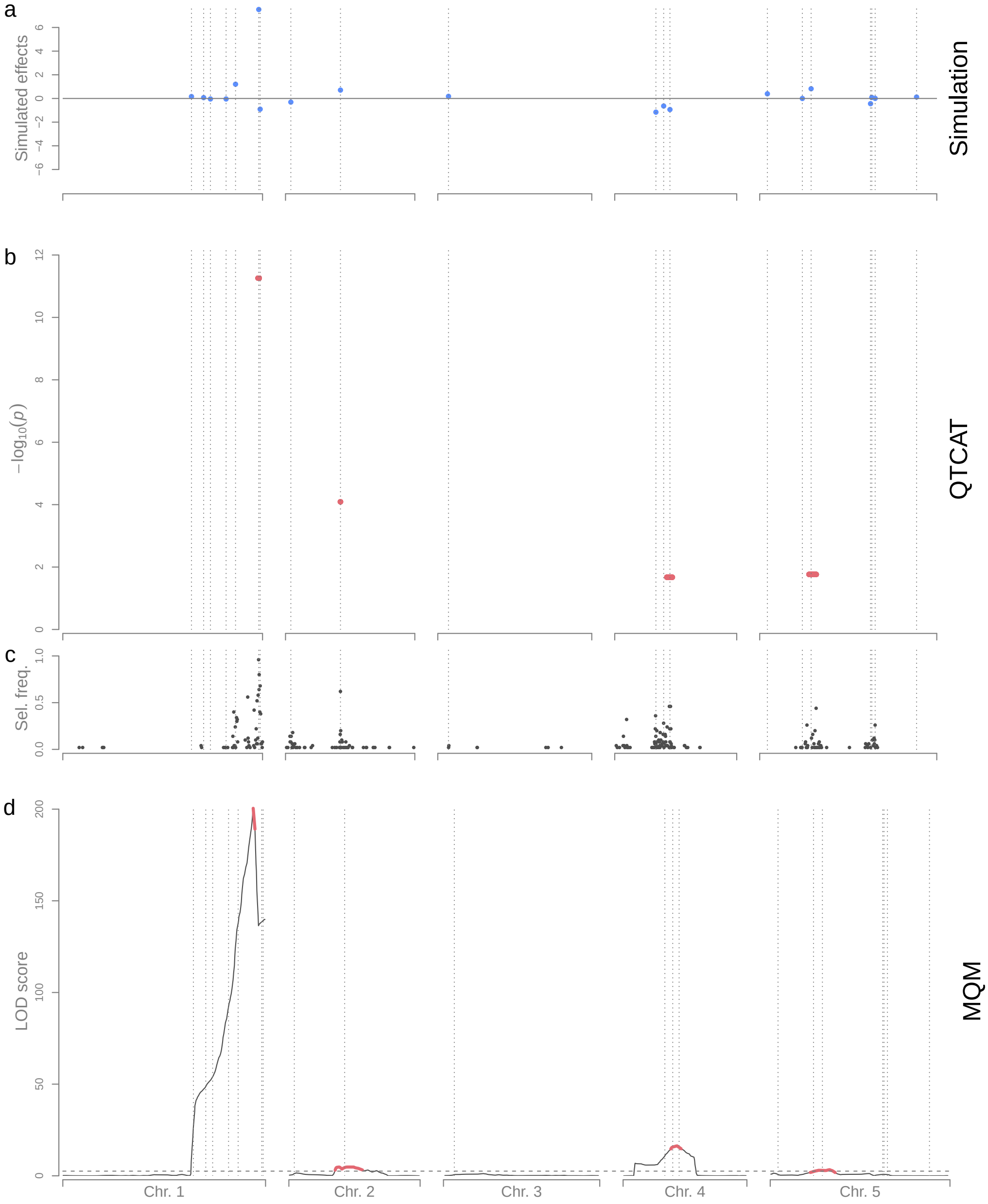

Supplementary Figure 394 Simulation of a GWA analysis based on a unstructured population with a heritability of 0.7 (run 94). (**a**) Simulated of 20 effects randomly drawn from a Gamma distribution and assigned them randomly to markers. Simulated effects randomly drawn from a Gamma distribution. We assigned effects to 20 markers. Markers with an effect are highlighted in (**b**–**d**) with dashed lines. (**b**) Significant QTCs found by QTCAT. (**c**) The selection frequency of the LASSO for each marker during the 50 iterations of QTCAT. (**d**) MQM LOD score plot, the horizontal dashed line is a simulation based perumtation test FDR. The red colored areas represent the LOD-intervals.

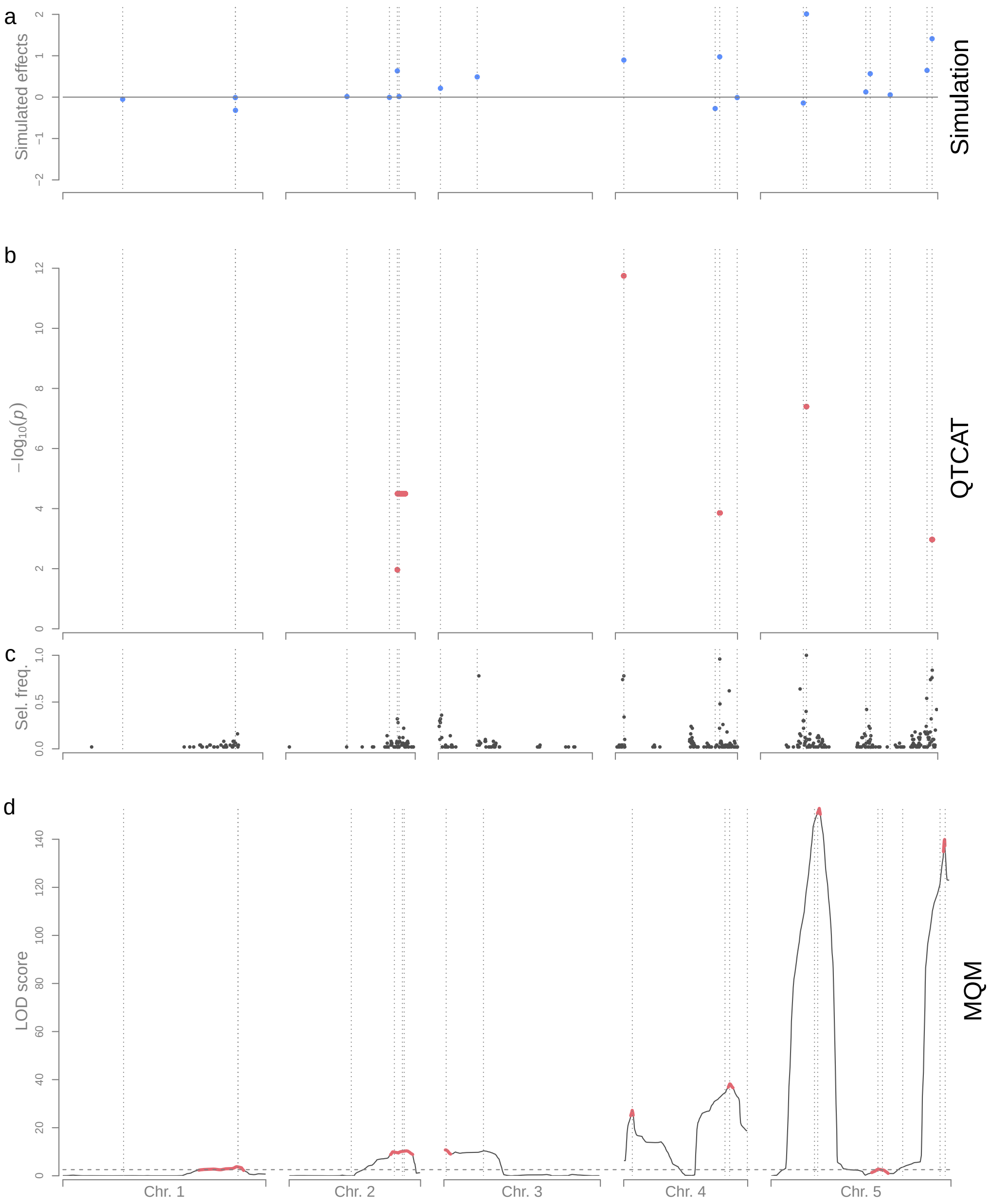

Supplementary Figure 395 Simulation of a GWA analysis based on a unstructured population with a heritability of 0.7 (run 95). (**a**) Simulated of 20 effects randomly drawn from a Gamma distribution and assigned them randomly to markers. Simulated effects randomly drawn from a Gamma distribution. We assigned effects to 20 markers. Markers with an effect are highlighted in (**b–d**) with dashed lines. (**b**) Significant QTCs found by QTCAT. (**c**) The selection frequency of the LASSO for each marker during the 50 iterations of QTCAT. (**d**) MQM LOD score plot, the horizontal dashed line is a simulation based perumtation test FDR. The red colored areas represent the LOD-intervals.

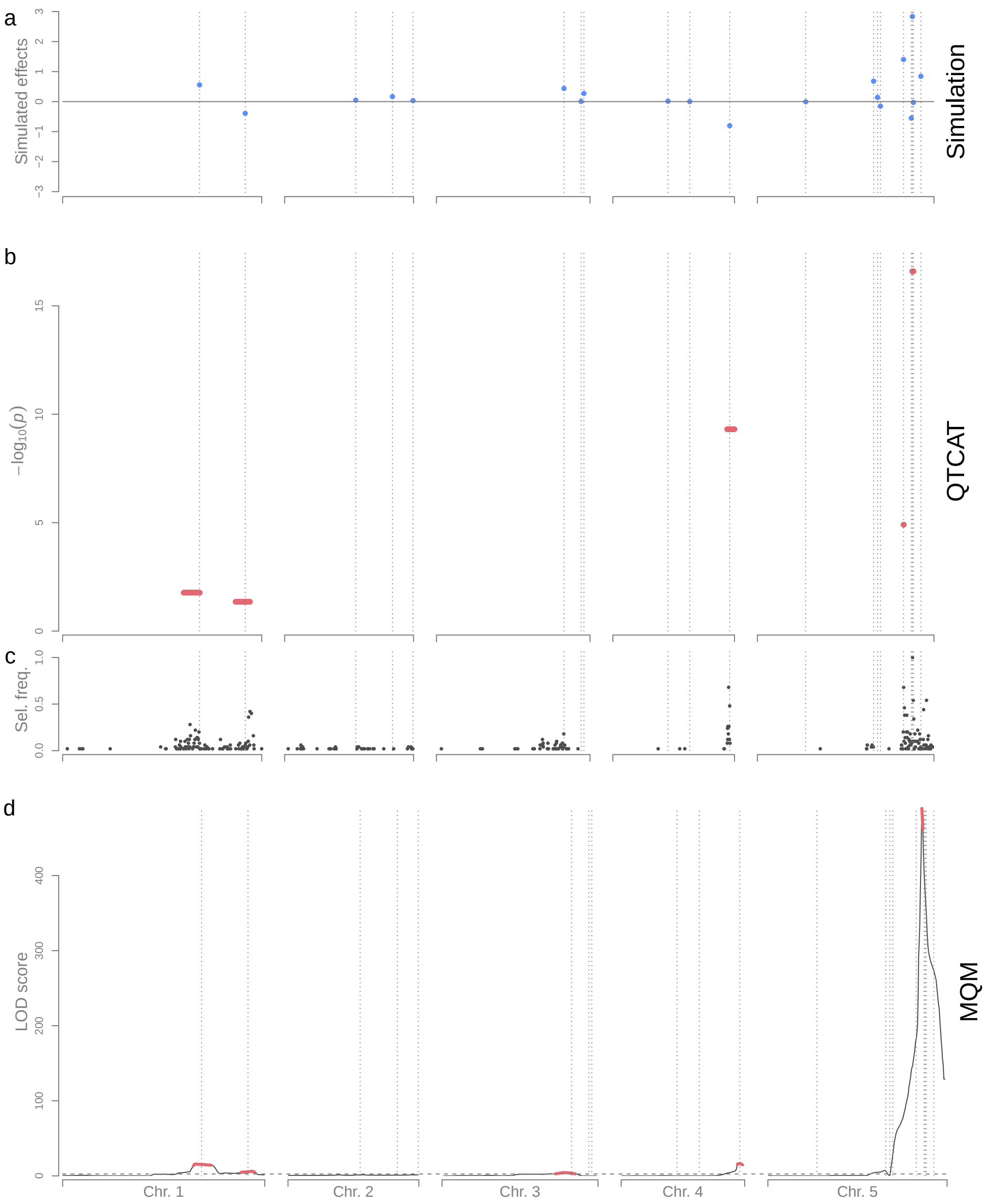

Supplementary Figure 396 Simulation of a GWA analysis based on a unstructured population with a heritability of 0.7 (run 96). (**a**) Simulated of 20 effects randomly drawn from a Gamma distribution and assigned them randomly to markers. Simulated effects randomly drawn from a Gamma distribution. We assigned effects to 20 markers. Markers with an effect are highlighted in (**b–d**) with dashed lines. (**b**) Significant QTCs found by QTCAT. (**c**) The selection frequency of the LASSO for each marker during the 50 iterations of QTCAT. (**d**) MQM LOD score plot, the horizontal dashed line is a simulation based perumtation test FDR. The red colored areas represent the LOD-intervals.

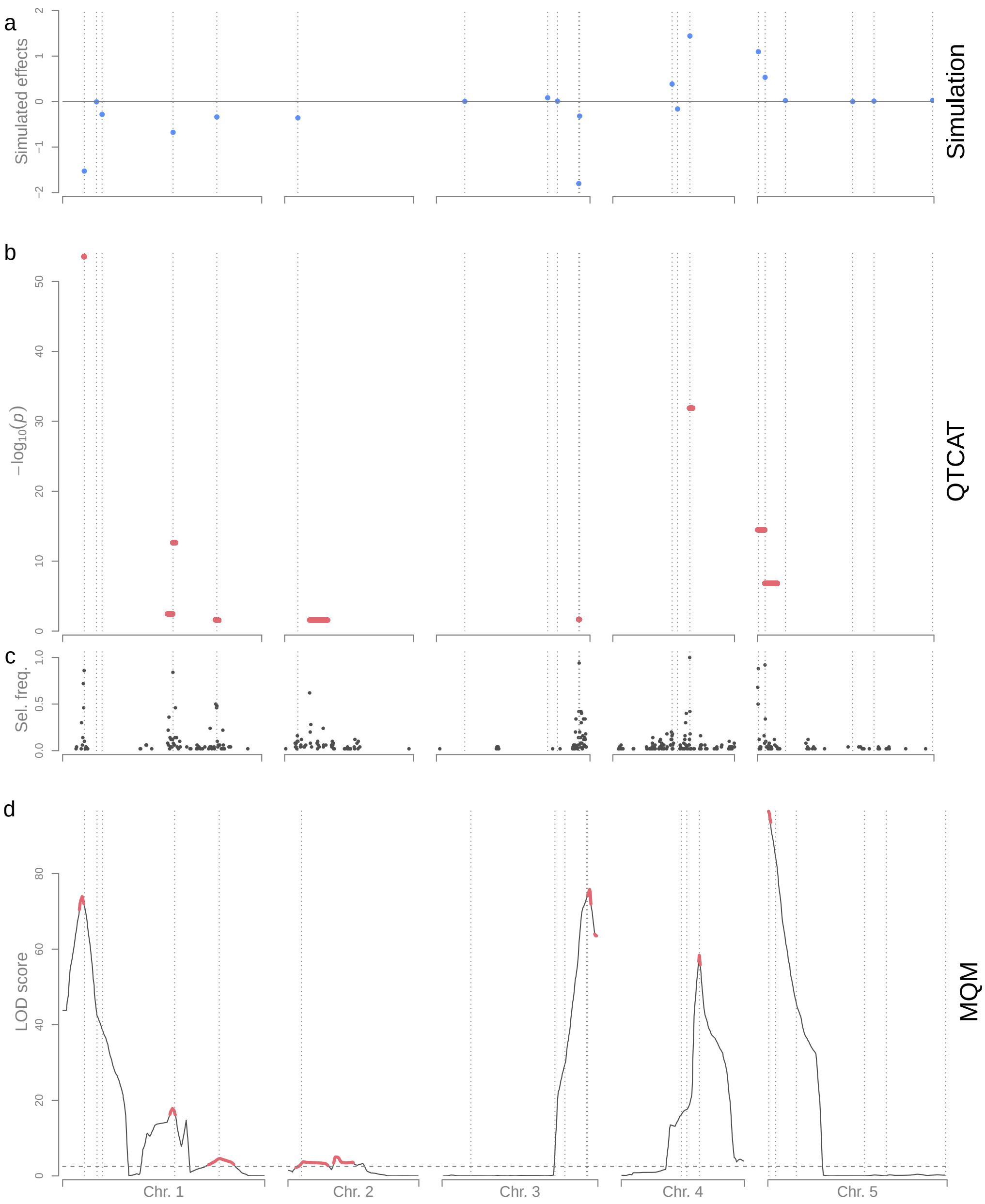

Supplementary Figure 397 Simulation of a GWA analysis based on a unstructured population with a heritability of 0.7 (run 97). (**a**) Simulated of 20 effects randomly drawn from a Gamma distribution and assigned them randomly to markers. Simulated effects randomly drawn from a Gamma distribution. We assigned effects to 20 markers. Markers with an effect are highlighted in (**b**–**d**) with dashed lines. (**b**) Significant QTCs found by QTCAT. (**c**) The selection frequency of the LASSO for each marker during the 50 iterations of QTCAT. (**d**) MQM LOD score plot, the horizontal dashed line is a simulation based perumtation test FDR. The red colored areas represent the LOD-intervals.

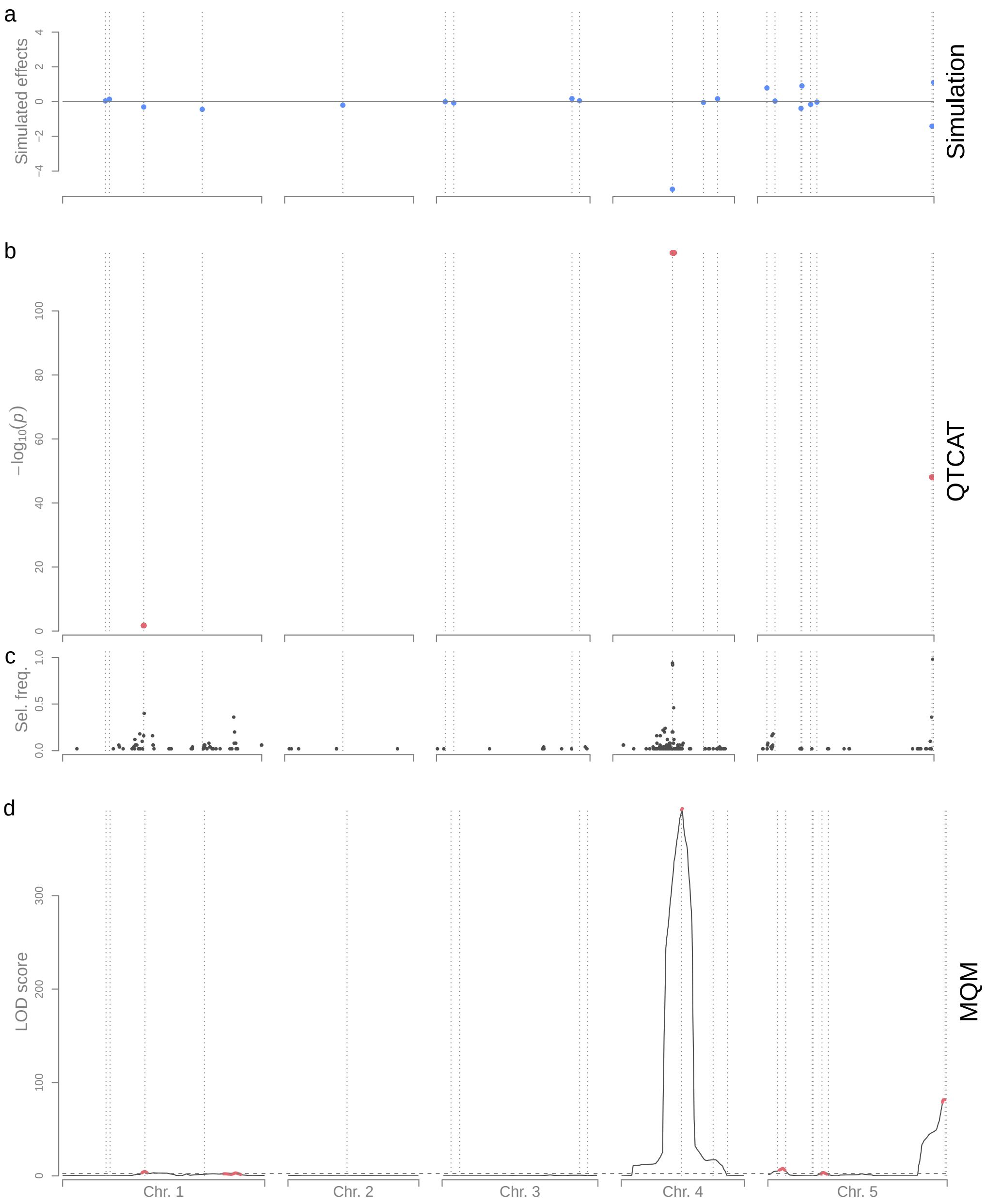

Supplementary Figure 398 Simulation of a GWA analysis based on a unstructured population with a heritability of 0.7 (run 98). (**a**) Simulated of 20 effects randomly drawn from a Gamma distribution and assigned them randomly to markers. Simulated effects randomly drawn from a Gamma distribution. We assigned effects to 20 markers. Markers with an effect are highlighted in (**b–d**) with dashed lines. (**b**) Significant QTCs found by QTCAT. (**c**) The selection frequency of the LASSO for each marker during the 50 iterations of QTCAT. (**d**) MQM LOD score plot, the horizontal dashed line is a simulation based perumtation test FDR. The red colored areas represent the LOD-intervals.

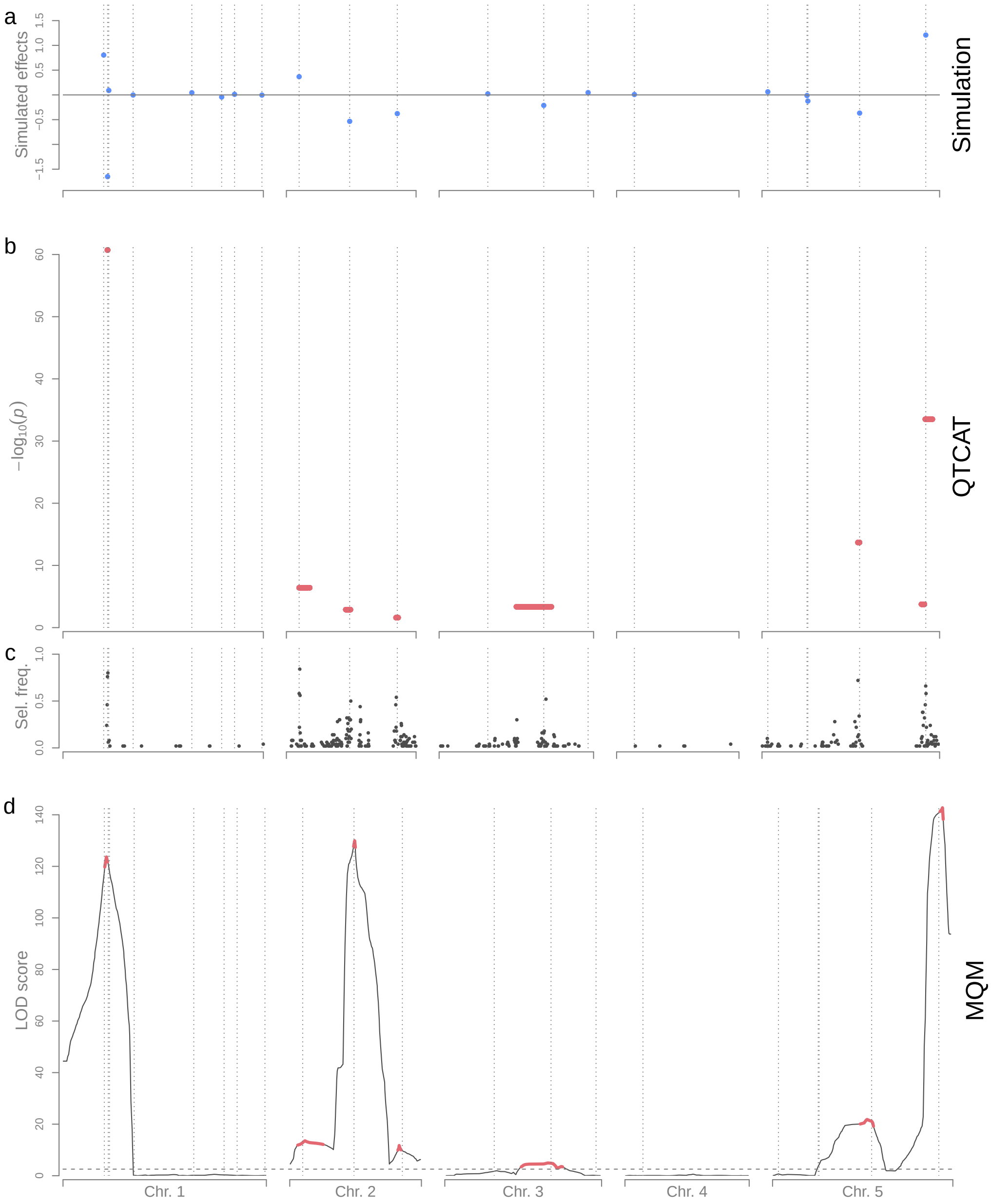

Supplementary Figure 399 Simulation of a GWA analysis based on a unstructured population with a heritability of 0.7 (run 99). (**a**) Simulated of 20 effects randomly drawn from a Gamma distribution and assigned them randomly to markers. Simulated effects randomly drawn from a Gamma distribution. We assigned effects to 20 markers. Markers with an effect are highlighted in (**b**–**d**) with dashed lines. (**b**) Significant QTCs found by QTCAT. (**c**) The selection frequency of the LASSO for each marker during the 50 iterations of QTCAT. (**d**) MQM LOD score plot, the horizontal dashed line is a simulation based perumtation test FDR. The red colored areas represent the LOD-intervals.

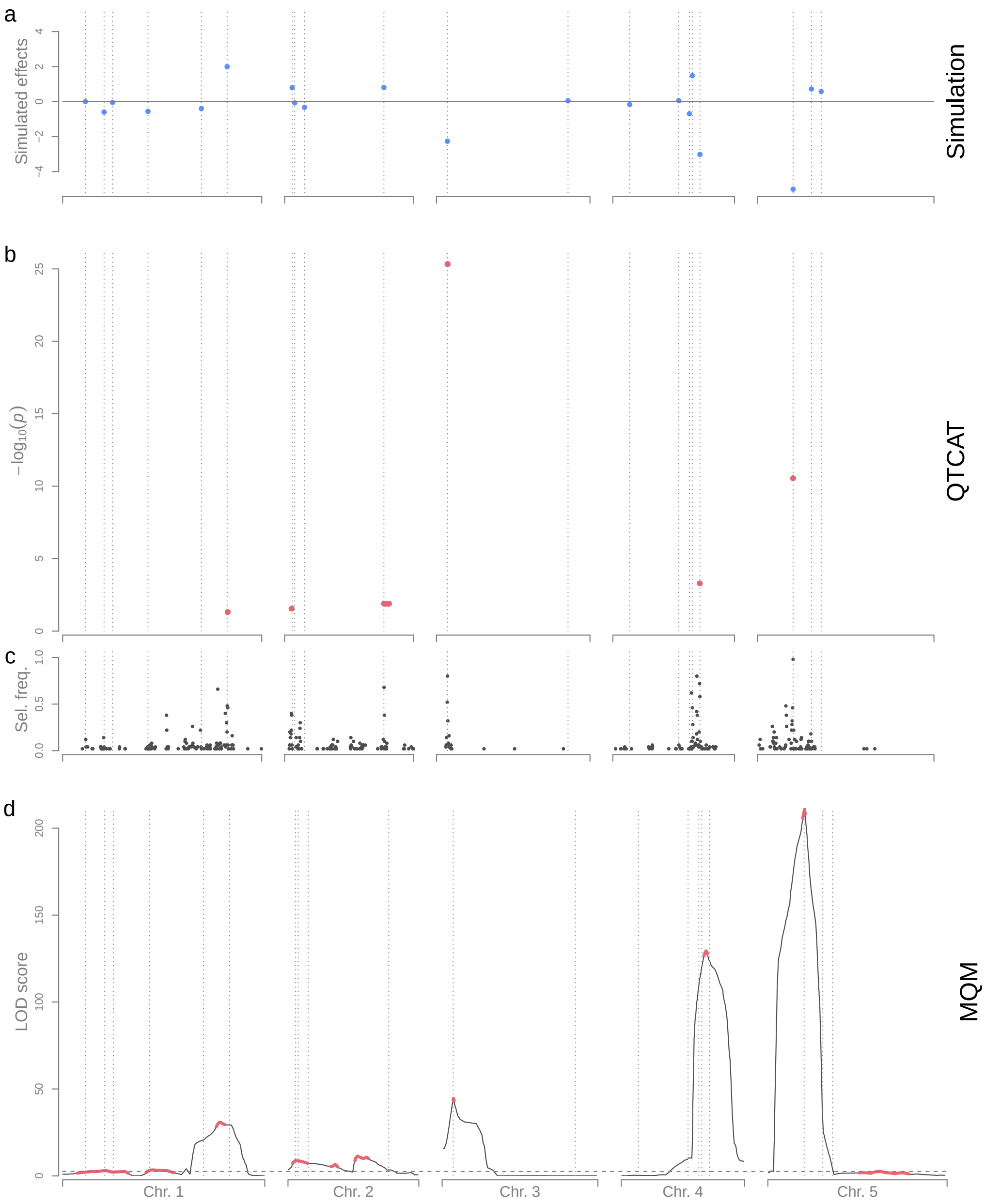

Supplementary Figure 400 Simulation of a GWA analysis based on a unstructured population with a heritability of 0.7 (run 100). (**a**) Simulated of 20 effects randomly drawn from a Gamma distribution and assigned them randomly to markers. Simulated effects randomly drawn from a Gamma distribution. We assigned effects to 20 markers. Markers with an effect are highlighted in (**b–d**) with dashed lines. (**b**) Significant QTCs found by QTCAT. (**c**) The selection frequency of the LASSO for each marker during the 50 iterations of QTCAT. (**d**) MQM LOD score plot, the horizontal dashed line is a simulation based perumtation test FDR. The red colored areas represent the LOD-intervals.

SNP distribution around the *PEPR2* gene

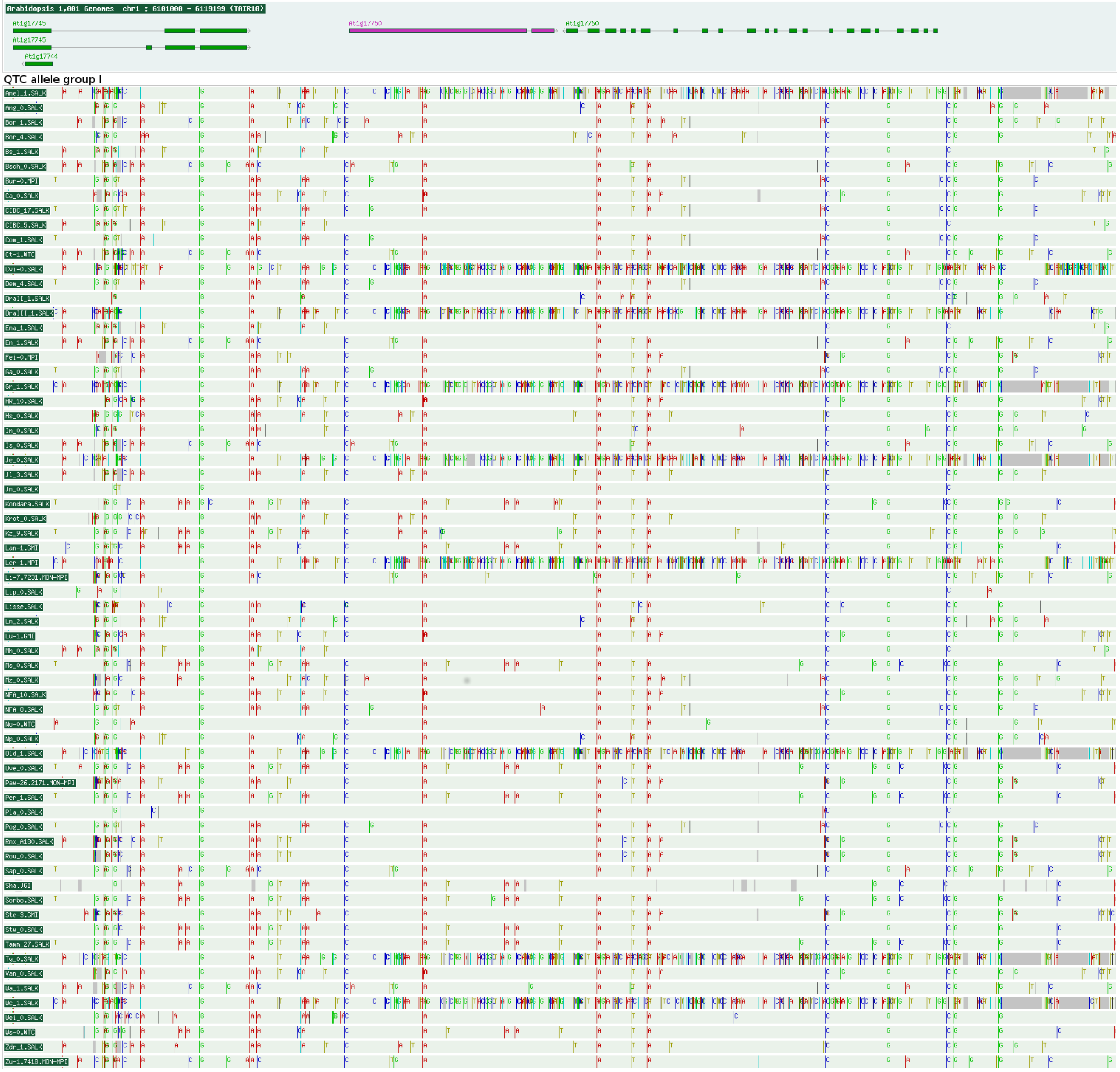

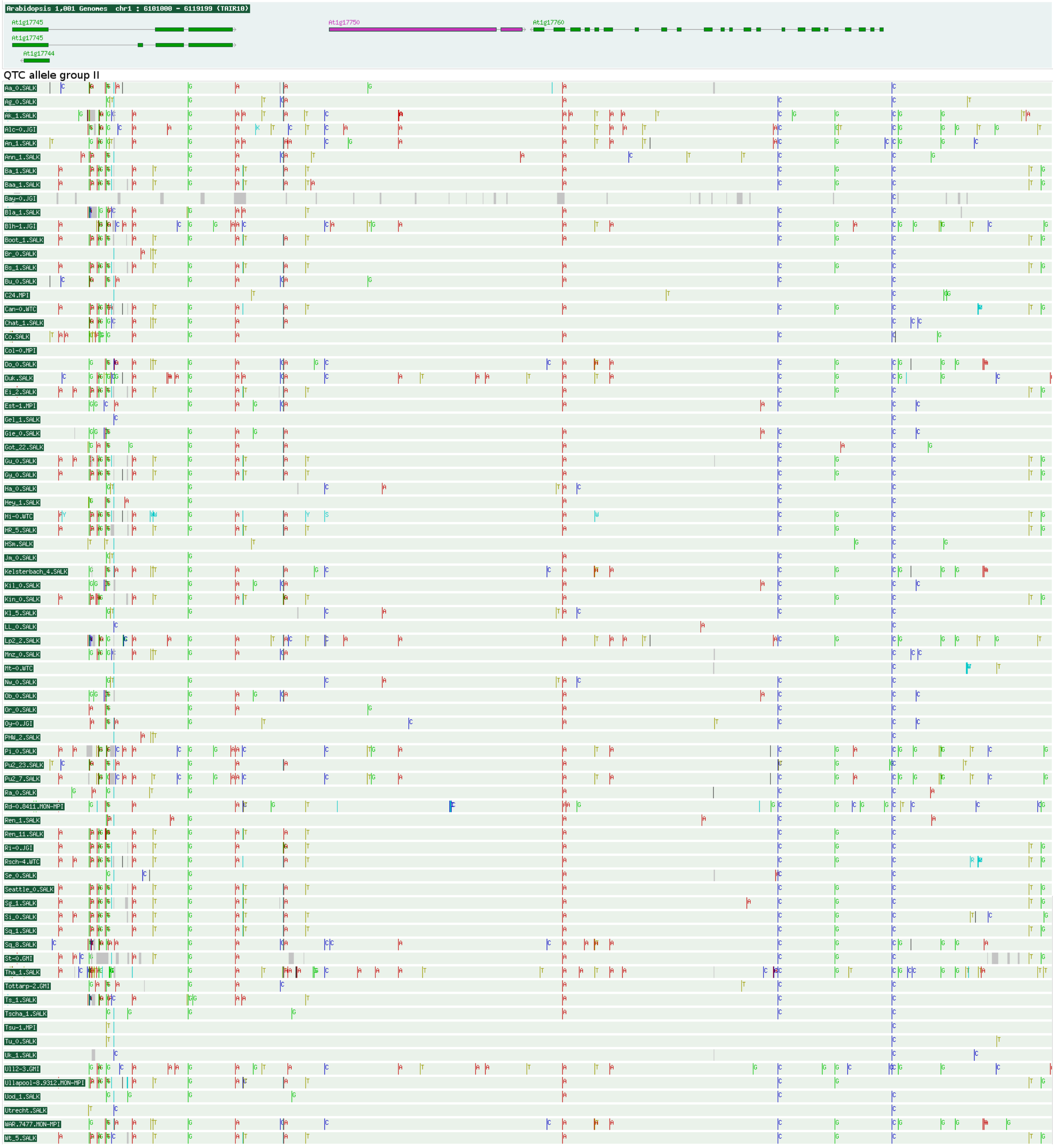

Supplementary Figure 401 SNP distribution around the *PEPR2* gene. The accessions which are part of the 1001 genome project and are included in the RegMap population used in this study are shown (screen-shot taken from: http://signal.salk.edu/atg1001/3.0/gebrowser.php). The accessions are sorted according to the QTC allele groups.

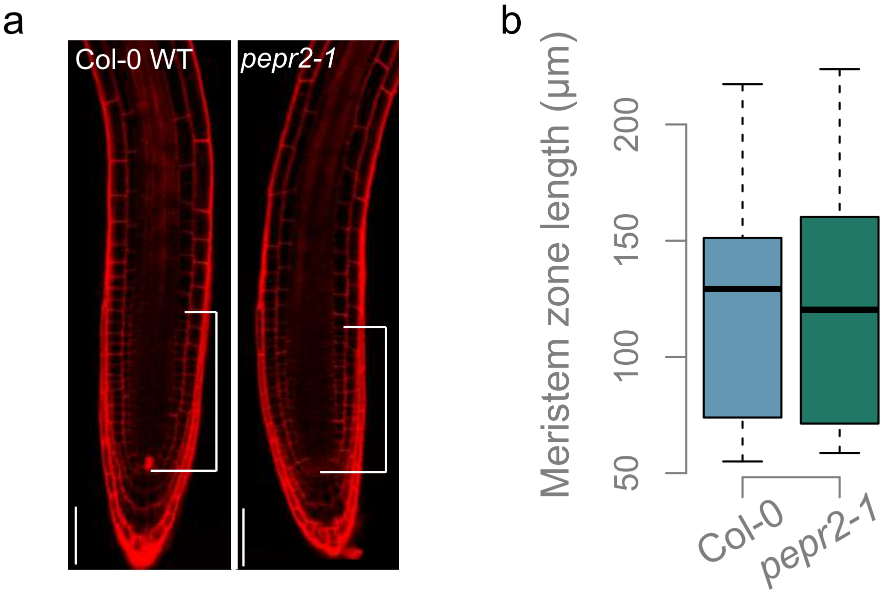

Supplementary Figure 402 Root meristem zone lengths after five days of stratification. (**a**) The root middle sections of 3 days-old Col-0 and *pepr2-1* mutant seedlings. White bars indicate meristem zone length (scale bar: 50 μm). (**b**) Boxplot of meristem zone lengths of Col-0 and *pepr2-1*.

GWA analysis of mouse high density lipoprotein levels

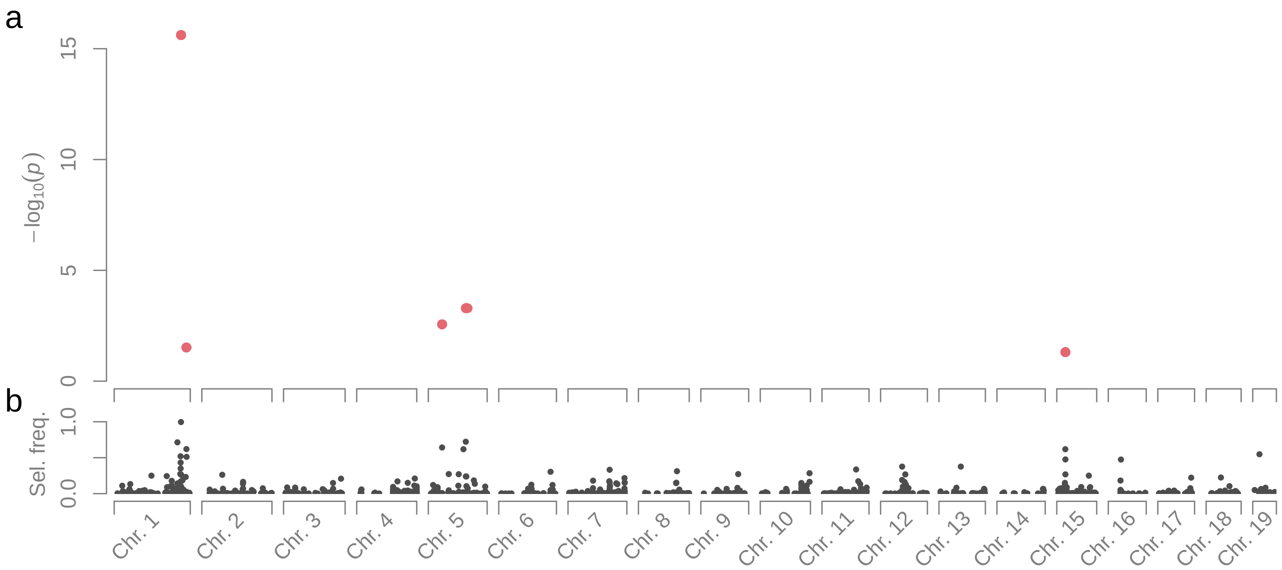

Supplementary Figure 403 QTCT analysis of high density lipoprotein in mouse. (**a**) Significant QTCs found for high density lipoprotein levels. (**b**) Selection frequency of markers during the 500 sample splits of QTCAT.

GWA analysis of human multiple sclerosis

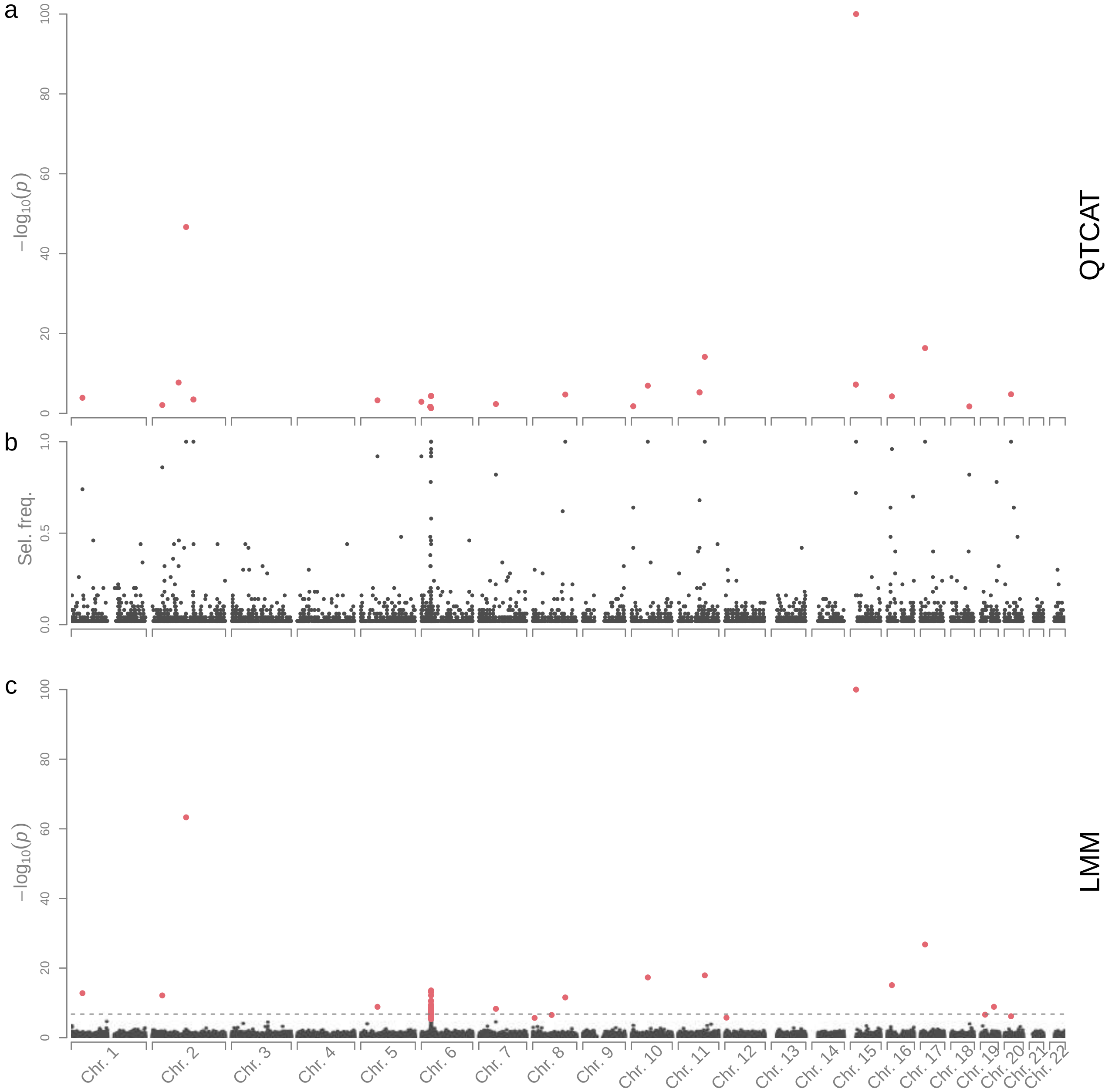

Supplementary Figure 404 GWA analysis of multiple sclerosis in human. (**a**) Significant QTCs found for multiple sclerosis case-control phenotypes. (**b**) Selection frequency of markers during the 500 sample splits of QTCAT. (**c**) Manhattan plot of the LMM analysis. The horizontal dashed line depicts the significance threshold when controlling multiple testing with FWER, whereas the red markers are significantly associated when controlling with FDR.

Number of markers and distance between the markers of a QTC

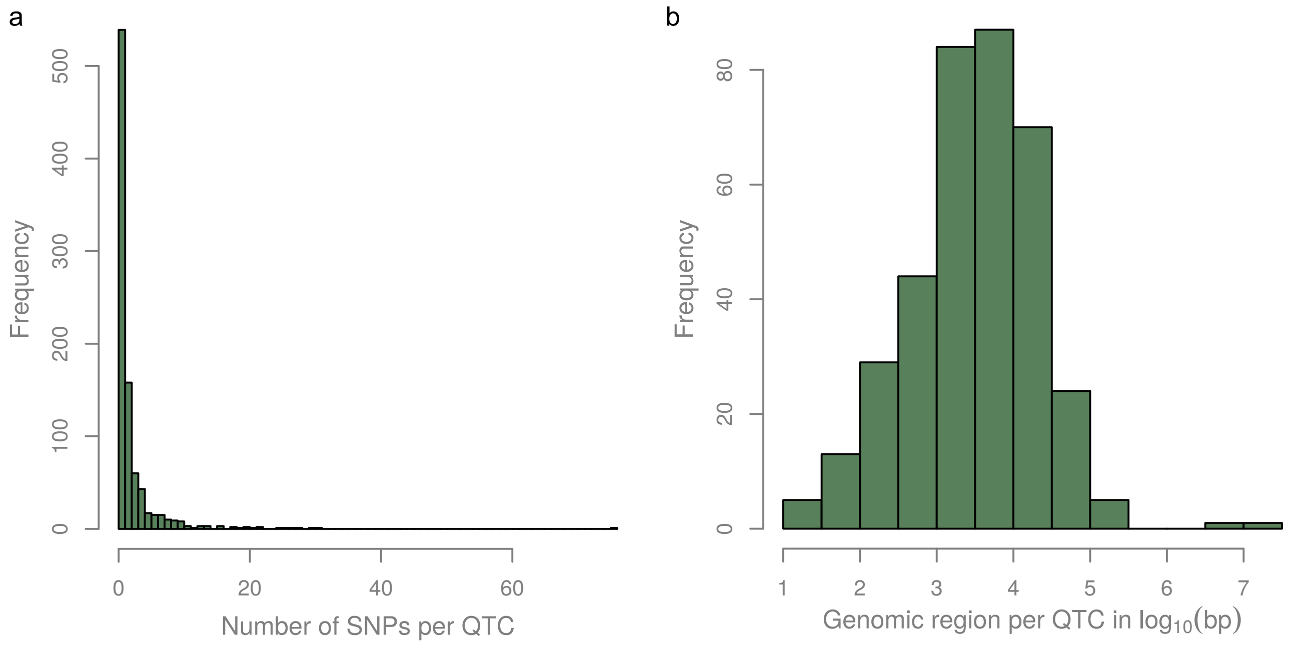

Supplementary Figure 405 Number of markers and maximum pair-wise distance between the markers of a QTC (Phenotype 1). (**a**) The number of markers per QTC. (**b**) The maximum pair-wise distance between the markers of a QTC containing two or more markers.

Supplementary Note 1

Physically, closely linked markers are highly correlated due to the lack of sufficient recombination between them. In addition to correlations based on physical linkage, population structure introduces correlations between markers even if they are weakly linked or not linked at all. Occasionally these correlations can lead to wrong associations between markers and phenotypes and thus need to be accounted for in any testing procedure.

In contrast to other methods, Quantitative Trait Cluster Association Test (QTCAT) is not correcting for population structure using similarity between individuals but accounts for the correlation between markers during the association procedure (similarity between rows (individuals) vs. columns (markers) of the marker matrix). Thereby QTCAT has an increased detection power as false positive markers can be controlled by this in a very efficient manner.

In a first step a hierarchical clustering based on the pairwise correlations between all markers is generated. As hierarchical clustering for large numbers of markers is computationally expensive, an approximated hierarchical clustering is calculated.

In a second step this hierarchical structure is used for the inference testing procedure. All markers as well as all possible clusters of markers in the hierarchical clustering are tested for a significant association to the phenotype. Beginning at the root of the hierarchical clustering, where all markers are joined, the clusters are tested for significant association to the phenotype conditionally to the rest of the genome. As long as this test yields significant associations, the algorithm moves down in the hierarchical clustering structure to test the sub-clusters of the actual cluster for their association to the phenotype.

In some cases this testing procedure will stop before the single marker level of the hierarchical structure is reached. In such a case, the outcome of the test is not the association of a single marker, but the association of a cluster of markers, called Quantitative Trait Cluster (QTC). This happens if markers are so strongly correlated to each other that they cannot be distinguished in respect to their individual effects on the phenotype.

Typically such sets of markers are physically linked, i.e. they reside in the same region. Markers, which are correlated due to population structure, are most often much weaker correlated. In consequence, the testing procedure will find stronger associations between the phenotype and clusters of physically linked markers as compared to markers that are correlated due to population structure. In rare cases, where population structure introduced highly correlated markers in unlinked regions (e.g. co-selection of two regions), these will be reported as one cluster (which will then span multiple regions in the genome) if associated to the phenotypes. However, this is not a weakness of QTCAT, in fact, if such markers exists they are by definition not distinguishable for their individual contribution to the phenotypes and thus they actually should be reported together. Moreover, if markers at different regions are strongly correlated but are still distinguishable, they are reported as independent QTCs. This helps to overcome problems like "ghost QTLs" which can appear in populations with strong linkage.

This second step is implemented in a iterative sample-splitting algorithm which is described in more detail after an introduction to the hierarchical clustering algorithm.

These statistical methods are not limited to associations between phenotypes and genetic markers. Therefore, we refer to them as response variable (phenotype) and covariates (markers).

Approximated hierarchical clustering

The goal of this algorithm is a computationally feasible approximation of an hierarchical clustering of a large number of covariates. Standard algorithms for hierarchical clustering are not applicable, mainly for two reasons: (i) the fastest hierarchical clustering algorithms have a runtime of $\mathcal{O}(n^2)$; and (ii) their computation speed relies on precomputed distances between all covariates, which have a memory usage of $\mathcal{O}(n^2)$. If the number of covariates is large, algorithms which scale quadratically in computing time and memory usage become

computationally very demanding. Therefore, a more efficient implementation is needed.

The algorithm we developed is an approximation which is able to process the problem in a practical computing time in combination with a small memory footprint (Algorithm 1). The algorithm consists of three steps: (i) removal of redundant covariates through identification of identical covariates; (ii) K-medoids clustering; and (iii) agglomerative hierarchical clustering.

Identification of identical markers

The first step is the identification of Z clusters of identical covariates. For each cluster of identical covariates, one covariate $i_z \in C_z$ is selected resulting in a subset of Z covariates $i_z = i_1, ..., i_Z$. These covariants are referred to as representative covariates. Note, this step does not remove any information of the association test, however, avoids redundant computation.

K-medoids clustering

The second step of the clustering procedure is the clustering of the representative covariates $\boldsymbol{G}^{(z)}$ into K clusters, $\{i_1, ..., i_Z\} = C_1 \cup ... \cup C_K$. These clusters are non-overlapping $C_k \cap C_{k'} = \emptyset$ with $k \neq k'$. The K clusters are generated by minimizing an objective function of the absolute correlation of representative covariates to a medoid M,

$$O = \sum_{k=1}^{K} \sum_{i_z \in \{i_1,...,i_Z\}} d(\boldsymbol{G}_{i_z}(k), M_k).$$

Hence, all representative covariates are partitioned into K clusters, where the number of clusters K is usually selected in the range of a few dozen. This step is performed to reduce the number of representative covariates per cluster, so that the next clustering step can be performed on smaller subsets of covariants instead of clustering all covariants at once.

Agglomerative hierarchical clustering

The third step includes hierarchical clustering of each of the K clusters. A hierarchy $\mathcal{T}_k$ is a set of clusters $\{C_h\}$ with $C_h \subseteq C_k$. On the basis of this hierarchy every representative covariate represents a distinct cluster. All pairs of clusters fulfil the following condition:

$$C_h, C_{h'} \in \mathcal{T}_k, \qquad (C_h \subset C_{h'}) \vee (C_h \supset C_{h'}) \vee (C_h \cap C_{h'} = \emptyset).$$

In this way, K independent hierarchical structures $\mathcal{T}_k$ are generated. Finally, these structures are joined at their roots into one hierarchical structure $\mathcal{T}$, of all representative covariates.

Clustering similarity function

Hierarchical clustering in general has no specific requirements regarding its similarity function. However, our approximation relies on initial K-medoids clustering, which typically relies on similarity function, which needs to fulfill the conditions of a metric space. Here k-medoids clustering in QTCAT is based on the similarity $d(\boldsymbol{G}_i, \boldsymbol{G}_{i'}) = |1 - r_{\boldsymbol{G}_i\boldsymbol{G}_{i'}}|$ with Pearson correlation,

$$r_{\boldsymbol{G}_i\boldsymbol{G}_{i'}} = \frac{\sum_{j=1}^{N}(\boldsymbol{G}_{ji} - \bar{\boldsymbol{G}}_i)(\boldsymbol{G}_{ji'} - \bar{\boldsymbol{G}}_{i'})}{\sqrt{\sum_{j=1}^{N}(\boldsymbol{G}_{ji} - \bar{\boldsymbol{G}}_i)^2 \sum_{j=1}^{N}(\boldsymbol{G}_{ji'} - \bar{\boldsymbol{G}}_{i'})^2}},$$

Algorithm 1 Three-step clustering

Input: Covariate matrix, K (where K is usually < 100).

First step:

1. Cluster all covariates with perfect correlation.
2. Select one representative covariate per cluster.

Second step:

1. Cluster the representative covariates into K clusters.

Third step:

1. Hierarchical clustering in each of the K clusters of the second step.
2. Joining of the K hierarchical clustering structures at their roots.

Output: Hierarchical structure of representative covariates,
perfectly correlated clusters of all covariates.

with the covariate $\{i, i'\} \subseteq \{1, ..., P\}$ were P is the number of covariates and the restriction $i \neq i'$. $\boldsymbol{G}$ is the dummy coded matrix of all covariates.

A metric relies on the following conditions: (i) $d(\boldsymbol{G}_i, \boldsymbol{G}_{i'}) \geq 0$ (non-negativity); (ii) $d(\boldsymbol{G}_i, \boldsymbol{G}_{i'}) = d(\boldsymbol{G}_{i'}, \boldsymbol{G}_i)$ (symmetric relation); (iii) $d(\boldsymbol{G}_i, \boldsymbol{G}_{i'}) = 0$ if and only if $\boldsymbol{G}_i = \boldsymbol{G}_{i'}$ (identity of indiscernible); (iv) $d(\boldsymbol{G}_i, \boldsymbol{G}_{i'}) \leq d(\boldsymbol{G}_i, \boldsymbol{G}_{i''}) + d(\boldsymbol{G}_{i'}, \boldsymbol{G}_{i''})$ (triangle inequality). The first two conditions are fulfilled by the above mentioned similarity function. The third condition is not met, however, perfect absolute correlation implies similarity independent of the sign of the correlation which is intended for dummy-coded covariates. Coding of alleles with either 0 or 1 at a marker is thereby interchangeably without affecting the similarity to other markers. The forth condition, the triangle inequality, is a key condition of k-medoids clustering. It guarantees that all covariates which are closely related to one medoid are themselves related. It cannot be formally be proven, however, we could confirm in a simulation test that it is fulfilled if more than 40 observations per covariate are considered. Therefore, this similarity function is valid for k-medoids clustering.

Hierarchical Inference Testing

In the following, the HIT algorithm of QTCAT is described (Algorithm 2), which is based on the idea of repeated sample-splitting as introduced in Mandozzi and Bühlmann[1]. The HIT algorithm is based on four steps: (i) sample-splitting; (ii) screening of representative covariates for an active set of covariates; (iii) significance testing; and (iv) aggregation of the results of the individual sample-splittings. This allows for detection of clusters of highly correlated covariates which are associated to the response variable.

Sample-splitting

The N observations (i.e. the sampled individuals) is B times randomly split into two groups $G_{b(1)}$ and $G_{b(2)}$, with $b = \{1, ..., B\}$. Such that $\{1, ..., N\} = G_{b(1)} \cup G_{b(2)}$ and $G_{b(1)} \cap G_{b(2)} = \emptyset$. The group sizes $g_1 = |G_{b(1)}|$ and $g_2 = |G_{b(2)}|$ are set to be $g_1 \leq g_2$.

Screening of representative covariates

For every sample split, the first group $G_{b(1)}$ of observation is screened for an active set of representative covariates. For this the following model is assumed:

$$\boldsymbol{y}_{G_{b(1)}} = \boldsymbol{X}_{G_{b(1)}}\boldsymbol{\beta} + \boldsymbol{\varepsilon}$$

with response variable of the sample split $\boldsymbol{y}_{G_{b(1)}}$ and the design matrix of the sample split $\boldsymbol{X}_{G_{b(1)}}$. This is done via the LASSO framework where λ is chosen per 10-fold cross-validation. In this way B active sets $\hat{S}_b$ of representative covariates are selected.

Algorithm 2 Hierarchical inference testing

Input: Response variable, representative covariates matrix,
hierarchical structure of representatives, number of sample splitting (B).
First step:

1. B random sample-splitting in two groups (Group I and II).

Second step:

1. Selection of an active set of representative covariates via LASSO for each of Group I.

Third step:

1. Testing significance for each active set in respective Group II at every node in the hierarchy.
2. Multiplicity adjustment of p-values.

Fourth step:

1. Aggregating results of all sample splits.
2. Hierarchical adjustment of p-values.

Output: p-value for every node in the hierarchy.

Significance testing

Since the active sets $\hat{S}_b$ are estimated in the first group $G_{b(1)}$ of the sample-splitting and the group sizes need to be restricted to $g_1 \leq g_2$. Hence the selected covariates are not high-dimensional any more $|\hat{S}_b| \leq g_2$. This enables testing the active sets $\hat{S}_b$ with sequential F-tests in the second groups $G_{b(2)}$. The hypothesis tested is $H_0^{(C\cap\hat{S}_b)}$ where $C \in \mathcal{T}$ is any given cluster. The p-value of these tests, $p^{(C\cap\hat{S}_b)}$, is multiplicity adjusted and thereafter, assigned to all representative covariates which are members of the cluster C. This is done even though only the representative covariates in $C \cap \hat{S}_b$ are tested. If the intersection is empty, $C \cap \hat{S}_b = \emptyset$, the p-value of this representative covariates cluster C is reported to be one. In short:

$$p_{\text{adj}}^{(C,b)} = \begin{cases} \min\left(p^{(C\cap\hat{S}_b)}\frac{|\hat{S}_b|}{|C\cap\hat{S}_b|}, 1\right) & \text{if } C\cap\hat{S}_b \neq \emptyset \\ 1 & \text{if } C\cap\hat{S}_b = \emptyset \end{cases}$$

Aggregation of the results of the sample splits

In the last step, all p-values $p_{\text{adj}}^{(C,1)}, ..., p_{\text{adj}}^{(C,B)}$ for each cluster C are aggregated using a procedure developed by Meinshausen et al.[2]. The aggregated p-values $Q^{(C)}$ are defined with $\gamma \in (0,1)$,

$$Q^{(C)}(\gamma) = \min\left\{1, q_\gamma\left(\left\{\frac{p_{\text{adj}}^{(C,b)}}{\gamma}; b = 1, ..., B\right\}\right)\right\}$$

$Q^{(C)}(\gamma)$ relies on an arbitrarily chosen γ. To avoid this value Meinshausen et al.[2] developed a procedure which results in:

$$P^{(C)} = \min\left\{1, (1 - \log_{\gamma_{\min}}) \inf_{\gamma_{\min},1} Q^{(C)}(\gamma)\right\}$$

Finally, the p-values can be hierarchically adjusted

$$p_h^{(C)} = \max_{D \in \mathcal{T}: C \subseteq D} P^{(C)}$$

In this way, it is possible to compute p-values for high-dimensional and possibly multi-collinear covariates. More discussion of these theoretical properties can be found in Mandozzi and Bühlmann[1].

Implementation of the Quantitative Trait Cluster Association Test

Implementation of clustering algorithm

Perfect correlation clustering implementation should avoid calculating all pairwise similarities to find the identical covariates in order to reduce memory requirements. First, a data-size-dependent number of covariates is selected as medoids. Those medoid covariates are selected to have a non perfect correlation. All the remaining covariates are then assigned to the closest medoid. Only in these clusters identical covariates can occur and therefore the number of pairwise comparisons are drastically reduced. During this step all similarity estimates are calculated on the fly.

K-medoids clustering expects one covariate from each of the perfect correlation clusters as input. In practice the number of representative covariates can still be very high. Therefore, calculation of all pairwise similarities would still be computationally expensive. Therefore K-medoids clustering is implemented as Clustering Large Applications based upon RANdomized Search (CLARANS) algorithm[3]. CLARANS is a modification of the Partitioning Around Medoids (PAM) algorithm[4]. Where the PAM algorithm is estimating all similarities between covariates and the respective medoids, CLARANS is searching a random subset of the covariates. This is independently repeated for several times and the result which minimises the average similarity the most is reported. This produces results close to those of the PAM algorithm[3]. The algorithm has two advantages: (i) the number of similarity comparisons is dramatically reduced; and (ii) parallelization is straightforward.

Hierarchical clustering is performed in parallel by complete linkage agglomerative hierarchical clustering[5]. For each of the parallel runs a cluster of covariates from the previous step is expected as input. For those covariates a similarity matrix is calculated. Thereafter, the standard implementation of hierarchical clustering implemented in `R` is used to perform the clustering.

Time complexity of the clustering is linear in regard to sample size. The time complexity in regard to the number of markers (p) depends on different steps. The CLARANS algorithm is used for a pre-clustering of p markers into k different clusters, which has its upper boundary at the PAM computing complexity of

$\mathcal{O}(k(p-k)^2)$ but in practice is much faster. The second step is hierarchical clustering of the members of each of these k clusters, which is carried out in $\mathcal{O}(p_k^3)$ (as compared to a computing time of $\mathcal{O}(p^3)$ of most hierarchical clustering algorithms). Hence, slightly increasing k can drastically reduce the total computing time, while only extreme values of k would affect the results, which makes the algorithm scalable to large data sets. For the Arabidopsis data k was only 22. Furthermore clustering is in respect to the marker data only, and can be re-used for the association of multiple phenotypes.

Implementation of Hierarchical Inference Testing

The HIT approach is computationally demanding. It is based on repeated sample-splitting with extremely high computational requirements at each step. The implementation in `qtcat` allows the user to choose the number of repeated sample-splittings; by default this is 50 which typically generates stable results. The ratio between the two groups of the sample-splitting can be defined by the user to be between 10% to 50% for the first half, where the rest is assigned to the second group.

Screening of covariates for an active set is performed with the first group of individuals of each sample-split. The model-selection is performed via a LASSO model, for which a highly efficient implementation is available in the `glmnet` package[6]. This implementation integrates ten-fold cross-validation for selecting the penalization parameter λ. The iterations are independent from each other, which allows for straightforward parallelization.

Significance testing at each node in the hierarchy. The number of nodes in a hierarchy structure is $P \times 2 - 1$ which implies that in case of large P it is challenging to test each node for significance. The current implementation allows choosing a maximum p-value, all nodes with a p-value higher than this maximum are assigned with a p-value of one, and thereby the algorithm avoids calculation of p-values for all nodes. This is possible as p-values are exclusively increasing along the hierarchy.

For this the F-test must be able to deal with highly correlated covariates. The `qtcat` package implements an F-test which, similarly to the standard implementation in `R`, is based on pivoted QR decomposition and hence is able to deal with perfectly correlated covariates. However, as the F-test implementation of `qtcat` is optimized for its specific purpose, it is faster than the standard implementation.

In this way, the HIT algorithm of the QTCAT approach is able to deal with large numbers of covariates. The resulting clusters of significantly associated covariates are in the quntitative genetic context named quantitative trait clusters (QTCs).

Time complexity of HIT is linear in regard to sample size. The time complexity in regard to the number of markers (p) is $\mathcal{O}(p \log p)$.

Supplementary References

1. Mandozzi, J. & Bühlmann, P. Hierarchical testing in the high-dimensional setting with correlated variables. *J. Am. Stat. Assoc.* 00003 (2015).

2. Meinshausen, N., Meier, L. & Bühlmann, P. p-values for high-dimensional regression. *J. Am. Stat. Assoc.* **104,** 1671–1681 (2009).

3. Ng, R. & Han, J. CLARANS: A method for clustering objects for spatial data mining. *IEEE T. Knowl. Data En.* **14,** 1003–1016 (2002).

4. Kaufman, L. & Rousseeuw, P. J. in *Statistical data analysis based on the L1 norm and related methods* (ed Dodge, Y.) 405–416 (North–Holland, Amsterdam, 1987).

5. Everitt, B. S., Landau, S., Leese, M. & Stahl, D. *Cluster Analysis* 5 edition. 346 pp. (Wiley, Chichester, West Sussex, U.K, 2011).

6. Friedman, J. H., Hastie, T. & Tibshirani, R. Regularization paths for generalized linear models via coordinate descent. *J. Stat. Softw.* **33,** 1–22 (2010).
